# Evaluation of Tannin-Delivery Approaches for Gut Microbiota Modulation: Comparison of Pectin-Based Microcapsules and Unencapsulated Extracts

**DOI:** 10.1021/acs.jafc.3c02949

**Published:** 2023-07-11

**Authors:** Silvia Molino, Alberto Lerma-Aguilera, Laura G. Gómez-Mascaraque, José Ángel Rufián-Henares, M. Pilar Francino

**Affiliations:** † Departamento de Nutrición y Bromatología, Centro de Investigación Biomédica, Instituto de Nutrición y Tecnología de los Alimentos, Universidad de Granada, Granada 18016, Spain; ‡ Silvateam Spa, R&D Unit, San Michele Monddoví 12080, Italy; § Area de Genòmica i Salut, Fundació per al Foment de la Investigació Sanitária i Biomèdica de la Comunitat Valenciana, (FISABIO-Salut Pública), València 46020, Spain; ∥ Food Chemistry and Technology Department, Teagasc Moorepark Food Research Centre, Fermoy, Co. Cork P61 C996, Ireland; ⊥ CIBER en Epidemiología y Salud Pública, Madrid 28029, Spain; # Instituto de Investigación Biosanitaria ibs.Granada, Granada 18012, Spain

**Keywords:** tannins, pectin, microbeads, gut microbiota, antioxidant, short-chain fatty acids

## Abstract

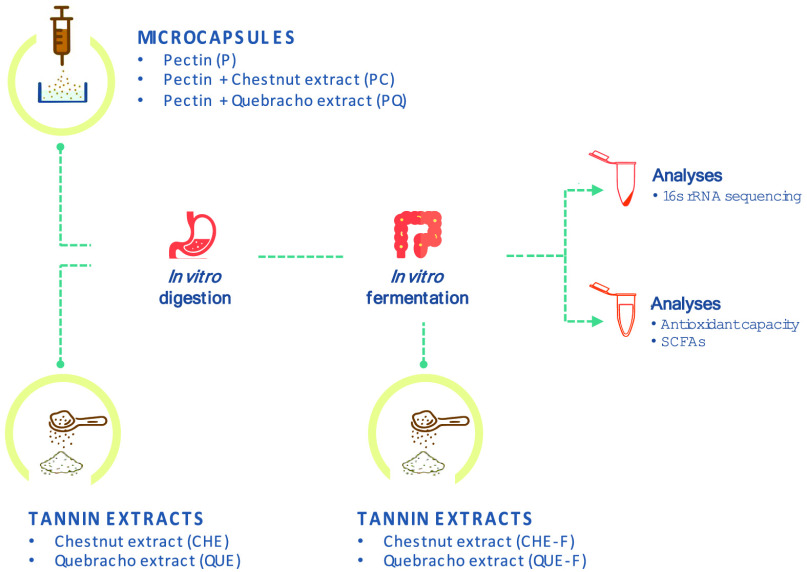

The aim of this study was to investigate the impact of tannins on gut microbiota composition and activity, and to evaluate the use of pectin-microencapsulation of tannins as a potential mode of tannin delivery. Thus, pectin-tannin microcapsules and unencapsulated tannin extracts were *in vitro* digested and fermented, and polyphenol content, antioxidant capacity, microbiota modulation, and short-chain fatty acid (SCFA) production were analyzed. Pectin microcapsules were not able to release their tannin content, keeping it trapped after the digestive process, and are therefore not recommended for tannin delivery. Unencapsulated tannin extracts were found to exert a positive effect on the human gut microbiota. The digestion step resulted to be a fundamental requirement in order to maximize tannin bioactive effects, especially with regard to condensed tannins, as the antioxidant capacity exerted and the SCFAs produced were greater when tannins were submitted to digestion prior to fermentation. Moreover, tannins interacted differently with the intestinal microbiota depending on whether they underwent prior digestion or not. Polyphenol content and antioxidant capacity correlated with SCFA production and with the abundance of several bacterial taxa.

## Introduction

The increasing interest of consumers in new healthy foods has given a big push to the development of innovative functional products.^1^ In this sense, there is a constant search for solutions for including natural bioactive ingredients into food products to boost them and make them attractive not only for their nutrient profile. Tannins are appealing bioactive ingredients for designing functional foods as they have been described to help in preventing or delaying the evolution of several diseases.^2^ Furthermore, it has been demonstrated that the incorporation of tannins into different types of food matrices induces healthy changes in the gut microbiota.^3^ These bioactive compounds, belonging to the big family of polyphenols, can be classified according to their sensitivity to hydrolytic break with acids. Hydrolyzable tannins, mainly composed of gallic acid with a sugar core, are then separated from condensed tannins, consisting of a repetition of building blocks of flavan-3-ol or flavan-3,4-diol units.^4^

The biggest challenge related to the direct incorporation of tannins into food is their unpleasant bitter and astringent taste.^5,6^ Furthermore, the great ability of these bioactive ingredients to bind proteins means that the addition of excessive quantities can lead to detrimental changes in food structure. One of the best strategies to introduce tannins into food is represented by microencapsulation that could mask the off-flavors and avoid contacts with food. Hydrogel microcapsules have been widely used for food applications, and they are formulated in food-grade conditions, giving stiff gelled capsules.^7^ Pectin, derived as a byproduct from apple or citrus fruits, is largely employed in the food industry as a stabilizer and a thickening and gelling agent. This biodegradable product may also be applied as a fat replacer and as dietary prebiotic fiber.^8^

Basic features required from capsules are the capacity to resist food processing and to minimize the release of bioactive compounds into the food matrix. On the other hand, capsules should liberate their encapsulated ingredients through digestion, allowing them to be bioaccessible and exert their beneficial effects.

In this work, free tannins and pectin-microencapsulated tannins were subjected to *in vitro* digestion and fermentation, in order to determine the best form of administering natural tannin extracts as a supplement to achieve beneficial effects. Two different types of tannin extract were microencapsulated with pectin, through external gelation. We evaluated the performance of the microcapsules over *in vitro* gastrointestinal digestion-fermentation regarding the release of antioxidant capacity and modulation of the gut microbiota and the production of SCFAs, compared to unencapsulated tannin extracts. Given that unencapsulated tannins are exposed to digestion *in vivo*, we also investigated the effect of gastrointestinal digestion on the free tannin extracts. To this aim, the same assessments were conducted on the extracts subjected to both *in vitro* digestion and fermentation or just to *in vitro* fermentation.

## Materials and Methods

### Study Design

This study has two parallel aims related to the evaluation of tannin delivery approaches for microbiota modulation: (1) to evaluate the adequacy of pectin-based microcapsules and (2) to assess the impact of digestion on free tannin extracts. To accomplish aim 1, we produced and evaluated pectin microcapsules enriched with quebracho (QUE) and chestnut (CHE) extracts, as well as pectin microcapsules devoid of extracts to be used as controls. These microcapsules will be referred to as PQ, PC, and P, respectively. The release of tannins from the microcapsules after digestion and fermentation was evaluated, by measuring total polyphenol content and antioxidant activity in the digestion and fermentation liquid phases, and the effects of the microcapsules on gut microbiota composition and activity were analyzed by 16S rRNA gene sequencing and measurement of short-chain fatty acid (SCFA) production. To achieve aim 2, the polyphenol content, antioxidant activity, and effects on the microbiota were compared between digested and undigested free tannin extracts, using the same methods as for aim 1.

**Figure 1:**
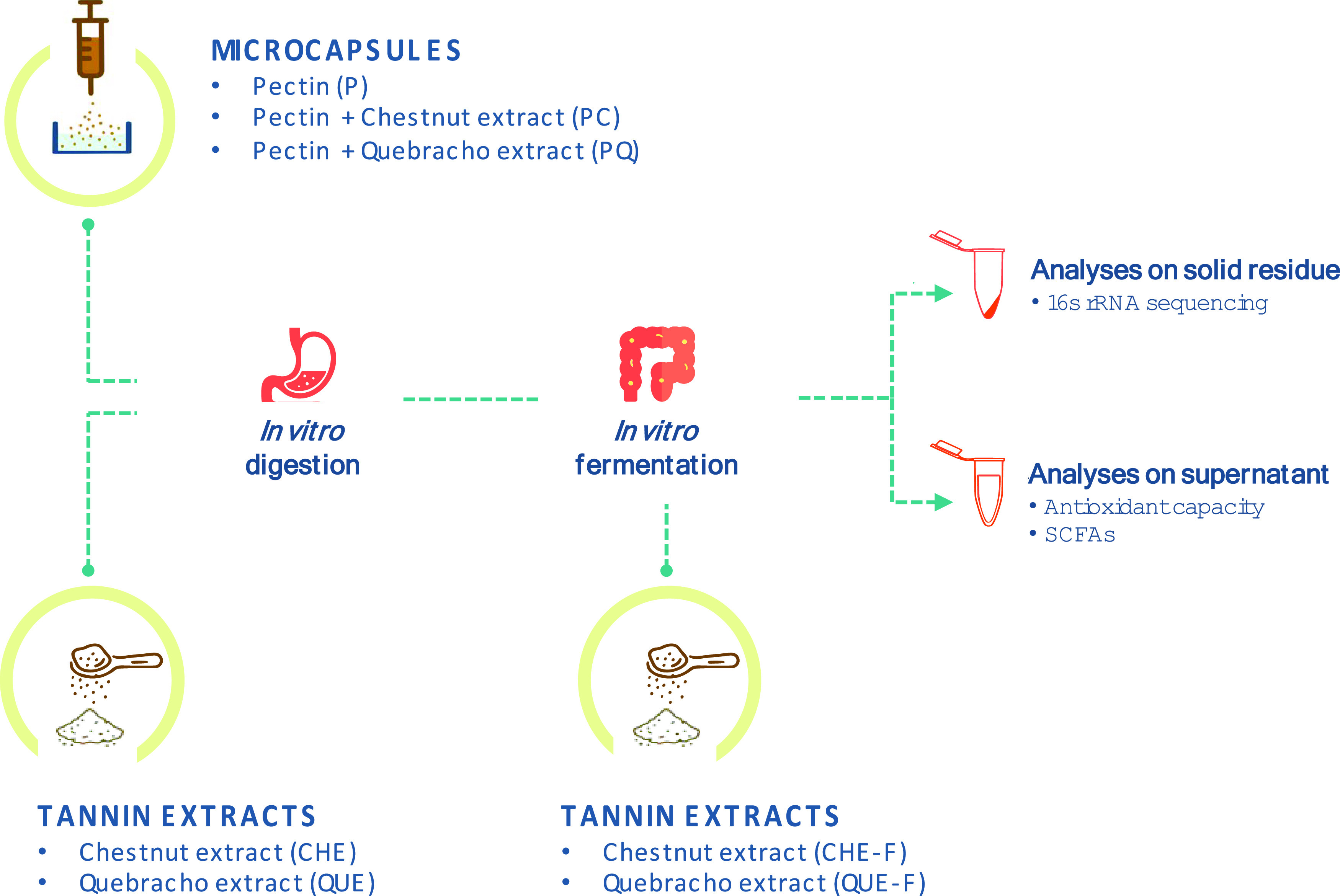
Scheme of the study design.

The scheme of the study design is resumed in Figure 1.

### Materials and Chemicals

Pectin AGLUPECTIN LA-20P was supplied from JRS Silvateam Food Ingredients S.r.l. (Italy). Silvateam Spa kindly provided the tannin extracts from quebracho (Welltan NU/Q) and chestnut (Welltan NU/C), as powder. Welltan NU/Q is a phytocomplex characterized by the presence of condensed tannins, deriving from profisetinidin, with a degree of polymerization up to 6.25.^9^ Welltan NU/C consists of a hydrolyzable ellagitannin mix, of which 30% is constituted by castalagin and vescalagin.^10^ To make the reading more fluent, the commercial products Welltan NU/Q and NU/C are referred to as QUE and CHE, respectively.

All chemical reagents used for *in vitro* digestion, fermentation, antioxidant assays, SCFA analysis, as well as DNA extraction were of analytical grade unless otherwise stated. Such reagents were from Sigma-Aldrich (Germany) and α Aesar (United Kingdom).

### Preparation of the Feed Formulations

A 2% (w/v) pectin aqueous solution was prepared by magnetic stirring at room temperature, and increasing concentrations of QUE and CHE with respect to the pectin mass (0 and 20% w/v) were added and mixed until a homogeneous dispersion was obtained. The concentration of tannins to be added to the feed formulation was established based on the results of preliminary studies.^11^

### Preparation of the Hydrogel Capsules

All of the pectin solutions were filtered through 0.8 μm pore syringe filters (Sartorius, Germany). Microcapsules were produced according to Gómez-Mascaraque et al.^12^ with slight modifications. Briefly, microbeads were produced with an InotechEncapsulator IER-50 (Inotech Biosystems Intl., Inc., Switzerland), by extrusion of the feed solutions through a 100 μm nozzle at a flow rate of 2.5 mL/min into the gelling bath (140 mm diameter) containing 250 mL of 0.1 M CaCl_2_ solution. The gelling bath was located at a distance of 16 cm from the nozzle and maintained under constant agitation. The formation of pectin droplets and their breakup was obtained with a nozzle vibration frequency of 1240 Hz and an applied voltage of 1.3 kV. The collection time was set at 4 min for each batch, and collection was followed by a curation within the gelling solution of 90 min. After that, microcapsules were filtered and thoroughly washed with deionized water. The produced microcapsules were stored at −80 °C and freeze-dried with a FreeZone benchtop freeze drier (Labconco), until further analysis.

### *In Vitro* Digestion and Fermentation

In order to study the effect of the microencapsulated tannins, the quantity of microcapsules containing 30 mg of tannin extracts (PC and PQ), the same quantity of pectin-only capsules (P), and 30 mg of free tannin extracts (CHE and QUE) were weighed and submitted to digestion and fermentation. In addition, a digestion blank containing no microcapsules or tannin extracts was also prepared. The *in vitro* digestion was performed following the protocol described by Pérez-Burillo et al.,^13^ based on the INFOGEST consensus^14^ and slightly modified. Briefly, the microbeads or the free tannins were added to Falcon tubes, and three steps were followed, resembling human oral, gastric, and intestinal digestion. The oral phase was mimicked with α-amylase for 2 min, at pH 7. For the gastric phase, pepsin was added with a following incubation of 2 h, at pH 2–3. The intestinal phase was performed with bile salts and pancreatin for 2 h, at pH 7. All of the incubations were performed under agitation and at a controlled temperature of 37 °C. From the digestions, a liquid-soluble fraction and a solid fraction were separated, after centrifugation of the mixture at 5000 rpm for 10 min at 4 °C. 10% of the supernatant was added to the solid residue to mimic the fraction that is not readily absorbed after digestion, while the remaining part was stored at −80 °C until further analysis.

500 mg of the digested wet-solid residues derived from the digestion process, or the corresponding amount of undigested tannin extracts (CHE-F and QUE-F), was subjected to *in vitro* fermentation, performed at 37 °C for 20 h, as described by Pérez-Burillo et al.^15^ The digestion blank as well as a fermentation blank containing only the fecal fermentation solution (composed of peptone, cysteine, and resazurin) were also submitted to the fermentation process.

The fermentations were performed with fecal samples from three healthy donors (two females and one male; mean body mass index = 21.2; mean age = 31; no antibiotics taken for 3 months prior to the assay) that were pooled together to reduce interindividual variability (University of Granada Ethics Committee approval no 1080/CEIH/2020). After centrifugation (10 min, at 500 rpm), the fecal pool supernatant was recovered to be used as inoculum (I) in the fermentations, which were performed in triplicate.

After *in vitro* digestion and fermentation, three fractions were obtained: a liquid fraction from digestion, a liquid fraction from fermentation, and a solid fermented fraction. The original inoculum (I) as well as the fermented digestion blank and the fermentation-only blank (B and B-F, respectively) were also recovered to be taken into account as controls in the following analyses.

### Tannin Release and Antioxidant Capacity Methods

The content of tannins in the free extracts and that released from the microbeads was estimated using the Folin–Ciocalteu (quantification of phenolic content) assay.^16^ Their antioxidant activity was assessed through the TEAC_FRAP_ and TEAC_ABTS_ assays.^16^ The determinations were conducted on the liquid fractions from both the digestion and the fermentation processes. In the case of the free extracts, determinations were performed for fermentations of both digested (CHE and QUE) and undigested (CHE-F and QUE-F) extracts. Results were expressed as mmol of gallic acid equivalent/ml of digested or fermented liquid fraction, for the Folin–Ciocalteu assay. For TEAC_FRAP_ and TEAC_ABTS_ assays, results were expressed as mmol of Trolox equivalent/ml of digested or fermented fraction.

### DNA Extraction

The bacterial suspensions obtained from the solid part deriving from *in vitro* fermentation were lysed with lysozyme at a final concentration of 0.1 mg/mL. Then, the extraction of genomic DNA was performed with the MagNA Pure LC JE379 platform (Roche) and DNA Isolation Kit III (Bacteria, Fungi) (REF 03264785001), following the manufacturer’s instructions. Agarose gel electrophoresis (0.8% w/v agarose in Tris-acetate-EDTA buffer) was used to determine DNA integrity, while the sample DNA was quantified with a Qubit 3·0 Fluorometer (Invitrogen). Finally, the DNA samples were stored at −20 °C until further processing.

### High-Throughput Amplicon Sequencing

12 ng of DNA was used as a template for the amplification of the V3-V4 hypervariable region of the 16S rRNA gene. Following the protocol of Klindworth et al.^17^ the forward primer (5′-TCGT CGGC AGCG TCAG ATGT GTAT AAGA GACA GCCT ACGG GNGG CWGCA-G3′) and reverse primer (5′-GTCT CGTG GGCT CGGA GATG TGTA TAAG AGAC AGGA CTAC HVGG GTAT CTAA TCC3′) were used for PCR amplification. The library construction was performed as described by the Illumina protocol for the 16S rRNA gene Metagenomic Sequencing Library Preparation (Cod 15044223 RevA). Primers were fitted with adapter sequences added to the gene-specific sequences to make them compatible with the Illumina Nextera XT Index kit. Then, amplicon sequencing was carried out with an Illumina MiSeq sequencer, according to the manufacturer’s instructions in a 2 × 300 cycles paired-end run (MiSeq Reagent kit v3). The data were deposited in the European Nucleotide Archive (ENA) at EMBL-EBI under accession number PRJEB48464.

### Bioinformatic Analyses

The DADA2 (v1.8.0) package from R (v3.6.0) was used to perform the sequence processing, assembly, Amplicon Sequence Variant (ASV) generation and annotation.^18^ The filter and trimming parameters used were the following: maxN=0, maxEE=c(2,5), truncQ=0, trimLeft=c(17,21), truncLen=c(270,220), and rm.phix=TRUE. A minimum overlap of 15 nucleotides and a maximum mismatch of 1 were required for the merging process of the forward and reverse reads. The reads were then aligned using Bowtie2 (v2,3,5,1) against the human genome (GRCh38.p13) and matches were subsequently discarded. The ASVs were generated by clustering sequences with 100% similarity. ASVs with less than 10 counts in total were discarded and the ASV count table was normalized by total-sum scaling (TSS). Taxonomic annotation was assigned by comparison to the SILVA 138 reference database using DADA2 v. 1.12.^19^ Annotation was assigned at species level for 100% similarity matches and for those matches that had a similarity of 97% or higher if there was a difference of at least 2% with the next highest match. Other sequences were annotated at the deepest possible taxonomic level.

### SCFA Analyses

The production of SCFAs was assessed on the liquid fractions deriving from fermentation. 1 mL was centrifuged to remove solid particles, filtered through a 0.22 μm nylon filter, and finally transferred to a vial for UHPLC (ultrahigh-performance liquid chromatography) analysis. The analysis of SCFAs was carried out on a 1290 Infinity II UHPLC (Agilent). The mobile phase was methanesulfonic acid 0.1 M pH 2.8/acetonitrile 99:1 v/v delivered at a 0.2 mL/min flow rate; the column used was an Accalim OA C18 reverse phase (Thermo Scientific) (150 × 2.1 mm, 3 μm), with a total run time of 22 min. Detection was carried out at 210 nm with a UV–vis PDA detector.

Three SCFAs were identified and quantified: acetic, propionic, and butyric acid. The respective standard solutions were quantified with concentrations ranging from 10,000 to 125 ppm. The results were expressed as mmol of SCFA per ml of fermented soluble fraction.

### Microbial Diversity and Statistical Analyses

The Shannon diversity index, Chao1 estimator, and ACE were obtained with Vegan (v2.5-2).^20^ To assess the effect of tannins on the bacterial composition, we analyzed the Bray–Curtis dissimilarity index between samples using Vegan (v2.5-2) and used this index for principal coordinate analysis (PCoA) generated with in-house R scripts. Wilcoxon signed-rank tests with adjustment for multiple comparisons were employed to evaluate differences in richness and diversity among samples. Analysis of the composition of microbiomes (ANCOM) was used to identify differentially abundant taxa among samples and significance was determined using the Benjamini–Hochberg procedure for false discovery rate control, as described by Kaul et al.^21^

Results of polyphenol content, antioxidant activity, and SCFA production were expressed as mean values of triplicates (*n* = 3) ± standard deviation (sd). One-way ANOVA with Bonferroni post-test correction was performed with the SPSS software (version 23, SPSS, Chicago, IL) to determine significant differences among mean values on all of the measured parameters.

Correlations between gut microbiota taxonomic groups and SCFAs, polyphenol content, and antioxidant activity were assessed using the network function in the mixOmics package (v6,10,9) from R, employing pair-wise similarity matrices that incorporate latent components obtained by sparse partial least squares (sPLS) regression. The values in the similarity matrix can be seen as a robust approximation of the Pearson correlation.^22^ Spearman correlations between gut microbiota taxa and SCFAs were also obtained.

## Results and Discussion

Microencapsulation of CHE and QUE in pectin microbeads by extrusion was successfully achieved. The microencapsulation efficiency values ranged between 29 and 35 for CHE and between 33 and 38% for QUE, depending on the different assays used (data not shown).

The bioactivity of free and microencapsulated tannins was measured as the antioxidant capacity exerted after *in vitro* digestion and fermentation, or fermentation only in the case of free extracts, and as the capacity for modifying the gut microbiota in terms of taxonomic composition and SCFA production.

### Tannin Release/Antioxidant Capacity

One of the required characteristics of the microcapsule matrix is that it dissolves at a certain point after ingestion, in order to release its content of bioactive compounds. So, quantifying the amount of tannins released during digestion and fermentation can be indicative of how well the capsules are liberating their content during the human digestive process.

Since there is no single method to quantify a phytocomplex such as those used in the study (QUE and CHE), their release through *in vitro* gastrointestinal digestion was tested using three different spectrophotometric techniques, i.e., Folin–Ciocalteu, TEAC_FRAP_, and TEAC_ABTS_ assays. By combining the results obtained from the three different methods, it is possible to obtain an overview that evaluates different aspects: total polyphenol content, reducing capacity, and scavenging capacity, respectively. Both liquids from digestion and fermentation were tested.

**Figure 2:**
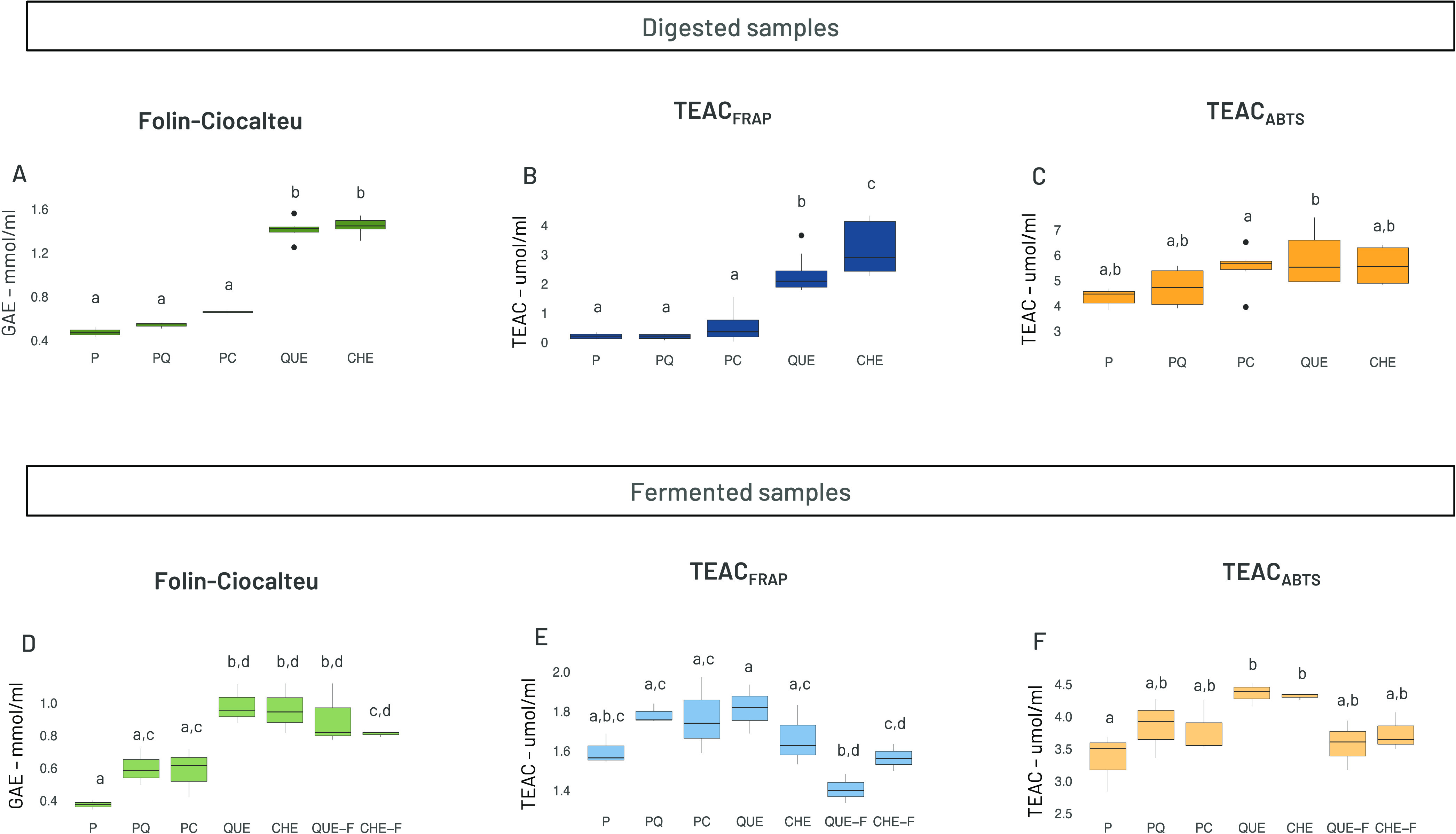
Total polyphenol content (by Folin–Ciocalteu) and antioxidant capacity (by TEAC_FRAP_ and TEAC_ABTS_) from digested (A–C) and fermented (D–F) fractions derived from pectin capsules and tannin extracts. (A, D) Folin–Ciocalteu, (B, E) TEAC_FRAP_, and (C, F) TEAC_ABTS_. P: pectin microbeads; PQ: pectin microbeads with quebracho; PC: pectin microbeads with chestnut; QUE: quebracho extract; CHE: chestnut extract; QUE-F: quebracho extract just fermented; CHE-F: chestnut extract just fermented. Median ± standard deviation values are reported. Different letters indicate significant differences (*p* < 0.05) among samples calculated by ANOVA with Bonferroni post hoc test.

As regards the liquid derived from the *in vitro* digestion, the lowest values for polyphenol content and antioxidant capacity were returned by the pectin microcapsules (P) devoid of tannins, as expected. Tannin-containing pectin microcapsules (PC and PQ) showed a significant increase in respect to P only in terms of total polyphenol content as detected by the Folin–Ciocalteu assay (Figure 2A). The digestion liquid of the nonencapsulated extracts (CHE and QUE) showed a much higher total polyphenol content (Figure 2A) and antioxidant capacity against ferric ions (TEAC_FRAP_) (Figure 2B) than that of the pectin microcapsules with or without tannins (P, PC, and PQ), but a similar antioxidant capacity against ABTS^*+^ radicals (TEAC_ABTS_) (Figure 2C). P already showed a high activity against ABTS^*+^ radicals (4.337 ± 0.339 μmol/mL) so that the minimal release of tannins during digestion of PQ and PC resulted in a total activity against ABTS^*+^ radicals almost reaching that exerted by the digested pure extracts QUE (5.863 ± 1.096 μmol/mL) and CHE (5.589 ± 0.784 μmol/mL) (Figure 2C).

After the *in vitro* fermentation process, only the Folin–Ciocalteu method consistently highlighted differences between the microcapsules and the pure extracts. As in the case of digestion, pectin showed the lowest values of total polyphenol content (0.373 ± 0.036 μmol/mL). Quantification in the liquids derived from the fermentation of PQ (0.6 ± 0.12 μmol/mL) and PC (0.5833 ± 0.138 μmol/ml) showed a slight but nonsignificant release of polyphenols. However, these values were lower in a statistically significant manner than those of the unencapsulated extracts (Figure 2D). This trend was not observed for the fermented fractions when testing the antioxidant capacity against ferric ions (TEAC_FRAP_) (Figure 2E). The bacterial fermentative action on pectin may have resulted in the release of compounds that have relatively high activity against ferric ions, comparable to that of tannin extracts. A similar effect resulted from the TEAC_ABTS_ assay, although P had significantly lower values than QUE (*p* = 0.007) and CHE (*p* = 0.007) (Figure 2F). Given the relatively high antioxidant capacity values provided by P, a substantial release of tannins from PC and PQ should have resulted in total antioxidant values much above those obtained from the unencapsulated tannin extracts. However, PQ and PC fermented liquids showed an antioxidant activity (for both TEAC_ABTS_ and TEAC_FRAP_) similar to that of the unencapsulated extracts, indicating only a slight release of tannins (Figure 2E,F).

These data suggest that there is little degradation of the encapsulating matrix (pectin) during *in vitro* digestion, resulting in a very small release of their tannin content. Although there is a slight release of QUE or CHE from the microcapsules in the soluble fraction of digestion, the measured polyphenol values are much lower compared to pure digested extracts. Moreover, the results showed that even after the fermentation process, there is no significant release of tannins into the fermentation liquid.

The combination of pectin and tannins to generate microcapsules was chosen because of the high binding affinity of these molecules. Tannins have been previously described as potential powerful cross-linkers, improving pectin gelling properties.^23^ But in this case the bonds formed between pectin and tannins during the microencapsulation process appear to have been so strong that neither the digestion process nor the action of the microbiota could degrade them.

Aguirre et al. investigated different encapsulation systems for beet waste extracts and, similarly to the present study, the products were subjected to *in vitro* digestion and fermentation.^24^ In this case, the encapsulated extracts showed a much higher antioxidant capacity than both nonloaded capsules and nonencapsulated extracts. It should be noted that the extracts used by Aguirre and co-workers were of a different chemical nature and the combination with a distinct formulation of the microcapsule matrix allowed for a more massive release of content and thus a combined antioxidant action.

As regards QUE and CHE, no significant differences were found between the two extracts. Similarly, the same trend was reported by Molino et al. when the pure extracts were submitted to *in vitro* digestion and fermentation.^25^ With the present study, it further emerged that CHE-F (undigested fermented CHE) did not show significant differences with the digested and fermented CHE (Figure 2D,E,F). Thus, the digestion process likely does not affect the CHE extract in terms of polyphenol content or antioxidant capacity. Conversely, a significant difference (*p* < 0.001) was recorded between QUE and QUE-F (undigested fermented QUE) with regard to reductive capacity (TEAC_FRAP_) (Figure 2E), suggesting that direct fermentation of this extract may result in a partial loss of the antioxidant capacity that the extract could exert if it were subjected to prior digestion.

### Effect on Microbiota Composition

**Figure 3:**
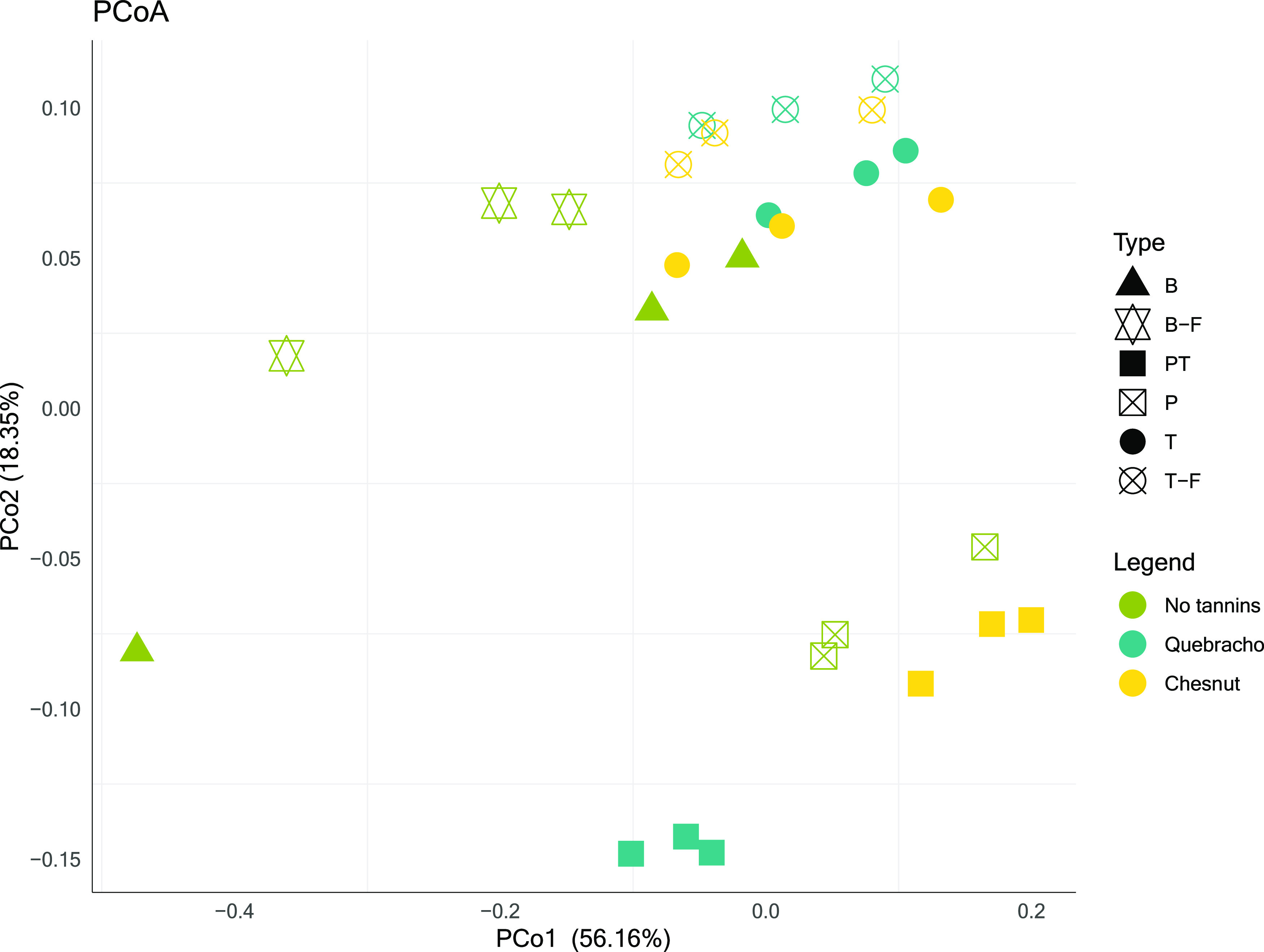
Principal coordinate analysis (PCoA) plot of total variation based on Bray–Curtis dissimilarity of microbial genus abundance among all profiled samples. The samples cluster by sample type. B (blank digested and fermented), B-F (blank just fermented), P (pectin capsules w/o tannins), PT (pectin capsules with tannins), T (tannin extracts digested and fermented), T-F (tannin extracts just fermented).

Figure 3 represents the distribution of β diversity among the microbiota communities in the different samples after fermentation, calculated as Bray–Curtis dissimilarity, where PCo1 and PCo2, respectively, contributed 56.16% and 18.35% of the total variation. The plot illustrates the overall differences in the relative abundance of taxa and shows a clustering of samples based on the type of material fermented, i.e., free tannin extracts or microcapsules. To illustrate this point in Figure 3, the two digested (CHE, QUE) and undigested (CHE-F, QUE-F) tannin extracts have been jointly labeled T and T-F, and the two tannin-containing microcapsules (PC and PQ) have been labeled PT. All of the fermentations of unencapsulated tannins (T, T-F) resulted in small overall differences in microbiota composition in relation to the blank fermentations (B, B-F). In contrast, all microcapsules (P, PT), including those not containing tannins, resulted in larger effects on gut microbiota composition, indicating a substantial role of pectin fermentation. The effect of CHE-containing microcapsules was similar to that of pectin-only microcapsules, whereas a more distinct microbiota was obtained after fermentation of the microcapsules containing QUE.

**Figure 4:**
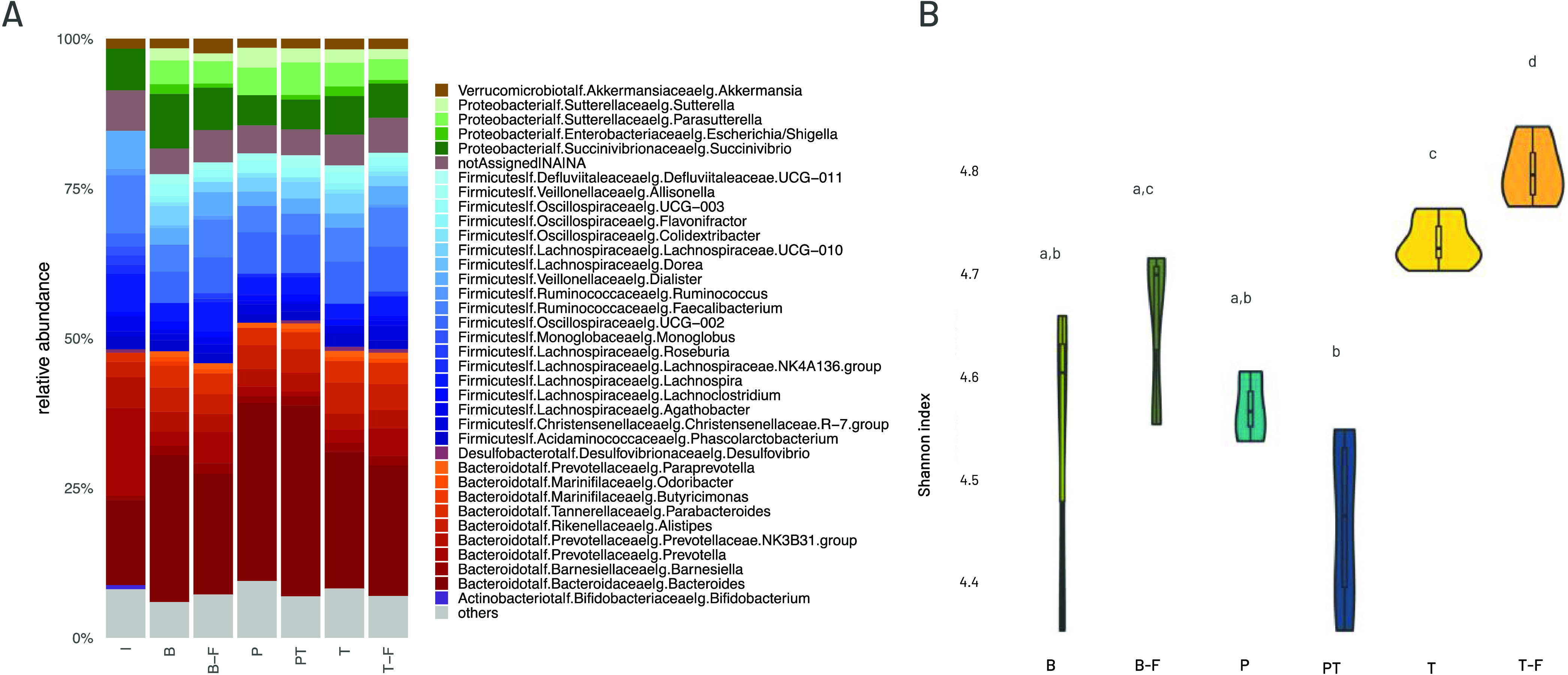
(A) Barplot of gut microbial community structure at genus level. Relative abundance obtained by total-sum scaling (TSS) from genus-level abundance table. “Others” include genera with relative abundance lower than 1% for all conditions. (B) Microbiota diversity measured as Shannon index. I (original inoculum), B (blank digested and fermented), B-F (blank just fermented), P (pectin capsules w/o tannins), PT (pectin capsules with tannins), T (tannin extracts digested and fermented), T-F (tannin extracts just fermented). Different letters indicate significant differences among samples (adjusted *p* < 0.05) by Wilcoxon test.

At the phylum level, the microbiota composition after *in vitro* fermentation was globally similar for all samples and dominated by Firmicutes and Bacteroidota, followed by Proteobacteria and Verrucomicrobia (Figure 4A and Table S1 in the Supporting Information). The presence of a relatively high abundance of Bacteroidota was already detected in the original inoculum (I) used to ferment the samples (Figure 4A). Thus, neither the *in vitro* fermentation process nor the fermented substrates were responsible for this high proportion of Bacteroidota.

Sample richness and diversity were estimated by different indexes, i.e., Chao1, ACE, and Shannon’s diversity index (Table S1 in the Supporting Information). When each tannin extract was analyzed individually, differences were small and not significant. This may be due to a low-test strength as the samples are only represented in triplicates. If the samples are grouped regardless of the type of tannin (T, T-F, PT), it can be observed that digested and fermented or fermented-only tannin extracts induce a significantly greater increase in diversity than when they are encapsulated (Figure 4B). This suggests that: (i) tannins are not totally released from the capsules during the digestion and fermentation process; (ii) although the amount of pectin is significantly larger than that of tannins, it is not able to elicit a change of equal magnitude.

ANCOM tests were applied to identify which bacterial taxa were significantly different among samples. Based on the previous findings, the data will be analyzed separately in order to study (i) the effect of microencapsulation of tannin extracts vs. the use of pure extracts and (ii) the effect of the digestion and fermentation process vs. the fermentation process alone on the tannin extracts.

### Effect of Microencapsulation

**Figure 5:**
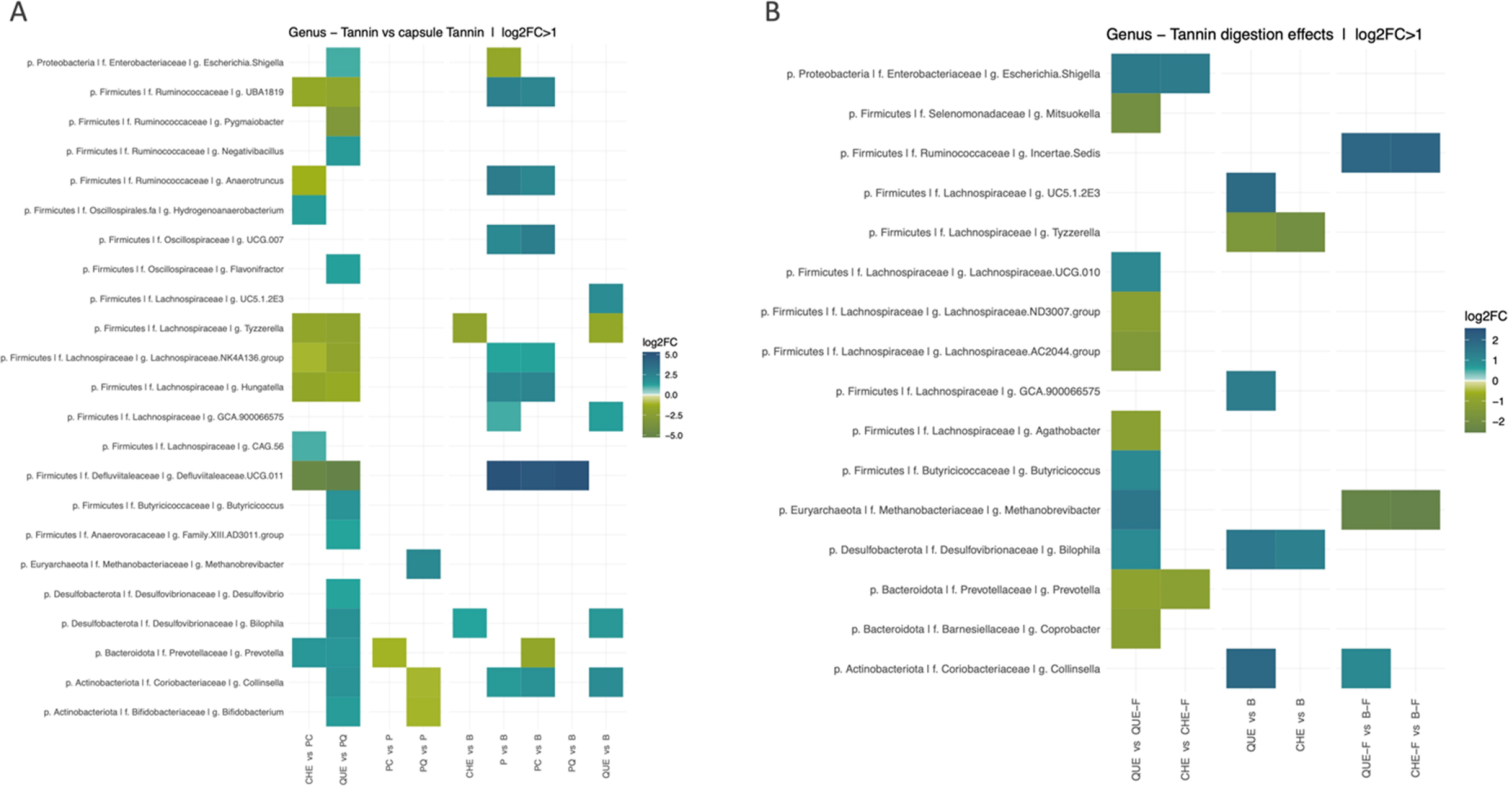
Effect on gut microbiota composition induced by tannins in relation to (A) microencapsulation and (B) digestion-fermentation. The heatmaps represent the fold-changes (log 2FC) in the relative abundance at genus level. All changes significant for adjusted *p* < 0.05 and filtered with log 2FC>1 at nominal level by ANCOM tests are shown.

As observed in Figure 5A, PC and P shared a very similar behavior compared to the blank that had undergone digestion and fermentation (B). This suggests that there was no significant contribution from the extract of hydrolyzable chestnut tannins and the interaction with the microbiota was attributable mostly to pectin. Among Firmicutes, several taxa were augmented after fermentation of both PC and P, i.e., Ruminococcaceae UBA1819, *Anaerotruncus*, Oscillospiraceae UCG 007, Lachnospiraceae NK4A136 group, and *Hungatella*. Bang and co-workers conducted *in vitro* fermentation of pectin to investigate possible changes in the gut microbiome and SCFA production. The hydrocolloid fermentation produced an increase of some taxa belonging to *Clostridium* cluster XIV, especially *Lachnospira*(26)

The fermentation of P and PC also led to an increase of *Collinsella*, belonging to Actinobacteriota. Regarding this genus, the information in the literature is controversial. Adamberg and co-workers correlated *Collinsella* to fiber-deficient diets,^27^ but the *in vitro* fermentation of Carlson et al. using a whole fiber diet (rich in pectin among others) promoted the growth of *Collinsella.*(28)

The only common finding between the three microbead-containing samples was a considerable increase in Defluviitaleaceae UCG-011 (Figure 5A). This is a newly discovered taxon, which has been recently associated with colitis induced by dextran sulfate sodium.^29^ In spite of the overall differences in microbiota composition reflected in Figure 3, we did not detect significant effects of PQ on specific taxon abundances in the microbiota, apart from that on Defluviitaleaceae UCG-011. This suggests that PQ exerted only small effects distributed among various taxa that, although not individually significant, were evidenced in the overall Bray–Curtis dissimilarity measures reflected in Figure 3. These results may relate to the fact that there is a strong interaction between pectin and quebracho extract molecules.^23^ Their association could possibly lead to the generation of complexes that reduce the interaction of both pectin and quebracho extract molecules with the microbiota due to steric hindrance.

The fact that tannins do not play an important role in modulating the microbiota when administered through microencapsulation with pectin is further highlighted by comparing the extract (QUE or CHE) with the corresponding microencapsulated sample (PQ or PC). It quickly becomes evident that there are several differences (Figure 5A). Compared to the microcapsules, the extracts result in substantially lower abundances of Defluviitaleaceae UCG-011 and of many of the earlier-mentioned Clostridiales (i.e., Ruminococcaceae UBA1819, *Anaerotruncus*, Lachnospiraceae NK4A136 group, and *Hungatella*), confirming that it is the capsules’ pectin rather than the tannins that induces the increase of these bacterial groups. The bacteria induced by the fermentation of unencapsulated tannins are discussed below in the context of the comparison of fermentations with and without a previous digestion step.

### Effect of Digestion

To investigate whether digestion has an impact on the compounds that will come into contact with the microbiota in the large intestine, QUE and CHE were subjected to both *in vitro* digestion and fermentation, and also to fermentation alone (QUE-F, CHE-F). Many microencapsulation systems are used to preserve a compound from degradation that can occur during the digestive process. This analysis therefore highlights whether it is possible to dispense with microencapsulation of tannins and still have them reach the intestine in order to produce an effect on the microbiota. Some studies report that tannins can pass through the digestive process almost intact. However, the two extracts under consideration show very distinct characteristics and enzymatic digestion may affect the chemical structure of these two extracts differently.

For chestnut extract, CHE and CHE-F induce different changes compared to their respective blanks (B and B-F), as can be seen in Figure 5B. CHE-F results in an increase of a Ruminococcaceae *incertae sedis* and a decrease of *Methanobrevibacter*, whereas the digested CHE induces an increase in *Bilophila* and a decrease in *Tyzzerella*. In addition, the direct comparison of CHE and CHE-F indicates that *Escherichia* is more abundant in the first and *Prevotella* is more abundant in the latter, indicating that previous digestion of the extract also results in a different effect on these genera. Several studies reported that both condensed and hydrolyzable tannins result in a decrease of *Prevotella*, at times correlated to inflammatory outcomes.^30−32^ Our results suggest that digestion increases this effect in the case of CHE.

On the other hand, QUE induced more changes than QUE-F compared with the respective blanks, suggesting that the chemical action of digestion may render the large characteristic structures of quebracho tannins more accessible to microbial fermentation. Digestion and fermentation of QUE led to an increase in Lachnospiraceae UC5.1.2E3, Lachnospiraceae GCA.900066575, *Bilophila*, and *Collinsella* and a decrease in *Tyzzerella*. Of these, only *Collinsella* also increased in the case of QUE-F, which showed in addition an increase of Ruminococcaceae *incertae sedis* and a decrease of *Methanobrevibacter*, also seen with CHE-F. The direct comparison of QUE and QUE-F highlights numerous further differences that reinforce the marked effect of digestion on quebracho extract. QUE resulted in a higher abundance of *Escherichia*, *Lachnoclostridium* UCG010, *Butyricicoccus*, *Methanobrevibacter*, and *Bilophila*, and a lower abundance of *Mitsuokella*, Lachnospiraceae groups ND3007 and AC2044, *Agathobacter*, *Prevotella*, and *Coprobacter*.

It is interesting to note that both the undigested CHE-F and QUE-F induced an increase in Ruminococcaceae *incertae sedis* and a decrease in *Methanobrevibacter* compared to the fermented blank (B-F) that were not observed with digested extracts. *Methanobrevibacter* is a methanogenic Archaea for which both an excessive abundance and a total absence have been associated with several pathologies. Therefore, some authors suggest using its relative abundance as an indicator of a healthy intestinal tract.^33^ The capacity of undigested tannins to modulate the abundance of *Methanobrevibacter* is therefore of interest, and it is important to know that their digestion will remove such capacity.

The difference in the effect of digestion on the two extracts probably relates to their chemical composition. Indeed, hydrolyzable tannins such as those present in chestnut extracts are identified as such because they can be fractionated hydrolytically into their components.^34^ This pronounced susceptibility to breakdown may mean that, even if they arrive intact in the intestinal environment, metabolization can occur in much the same way as when the extract is also subjected to prior digestion. In contrast, digestion of the nonhydrolyzable quebracho tannins appears to change more significantly the capacity of the microbiota to metabolize these compounds.

### SCFA Production

Nondigestible fibers are recognized to be great substrates for the production of SCFA. However, other substances, tannins among them, have recently also been attributed to the ability to stimulate the fermentative activity of the intestinal microbiota.^35,36^ Testing SCFA production is not only important to verify the stimulation of microbiota activity but also because these compounds exert various health-promoting effects.^37,38^

**Figure 6:**
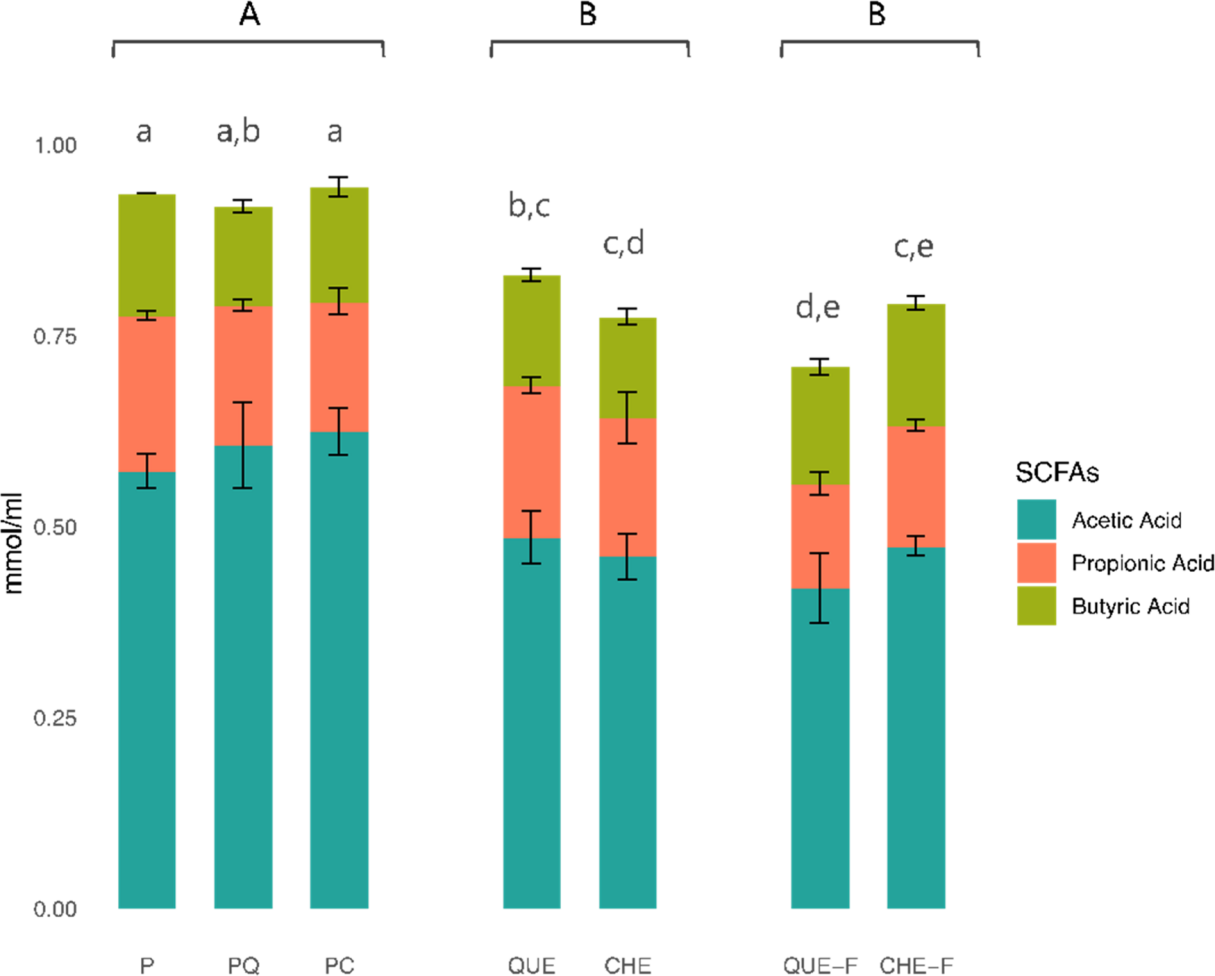
Release of SCFAs (mmol per ml of fermented liquid fraction). Mean ± standard deviation values are reported. Different uppercase letters indicate significant differences (*p* < 0.05) among group of samples (microcapsules P, PQ, PC; digested and fermented tannin extracts QUE, CHE; just fermented tannin extracts QUE-F, CHE-F), while lowercase letters indicate significant differences among all samples (*p* < 0.05), by ANOVA and Bonferroni post hoc test.

Figure 6 illustrates the sum of SCFAs (i.e., acetate, propionate, and butyrate) released after the fermentation of pectin-based microbeads with or without tannin extracts (P, PC, PQ), as well as after the fermentation of the extracts alone (CHE, QUE, CHE-F, QUE-F).

All of the tested samples showed high production of SCFAs, compared to the blank, confirming once again the prebiotic potential of tannins. When subdividing the samples into three groups (i.e., capsules, digested and fermented tannin extracts, and fermented tannin extracts), the capsules showed a higher release of SCFA (*p* < 0.001) compared to the extracts. When analyzing the different types of capsules, those containing tannins (PC and PQ) did not differ significantly from nonloaded capsules (P). This suggests that only the contribution of pectin is crucial in the production of SCFAs, further indicating that no appreciable amount of tannins is likely released from the capsule matrix.

Although the unencapsulated tannin extracts stimulated a lower production of total SCFAs in comparison to pectin capsules, it should be borne in mind that the amount subjected to *in vitro* digestion and fermentation (or fermentation only) has been calculated so as to equal their content within the capsules (*ca.* 4.5% w/w). Therefore, an amount of tannins 22-fold lower than that of pectin was able to stimulate a similar production of SCFAs. In support of the high prebiotic activity of the two extracts, when Molino et al. compared equal amounts of tannin extracts (both CHE and QUE) with inulin, the extracts presented much higher values of SCFA production.^25^

With regard to tannin extracts, no significant differences were found between CHE and CHE-F, whereas QUE-F presented a significantly lower (*p* = 0.025) SCFA release than QUE. Similar to 16S rRNA sequencing data, again it appears that chestnut hydrolyzable tannins can be easily metabolized directly by the gut microbiota in the absence of previous digestion. On the other hand, quebracho extract is characterized by a more complex chemical structure (condensed tannins) so that the preliminary step of enzymatic digestion seems to be essential to facilitate its microbial fermentation.

As for the production of individual SCFAs (Table 1), microcapsules resulted in a higher release of acetate compared to tannin extracts, independently of whether the latter were digested and fermented or just fermented. The fermented-only extracts showed much lower propionate production than the other two sample groups (capsules and digested-fermented extracts). In particular, QUE and QUE-F showed the largest difference, indicating that bypassing the enzymatic digestion process of the quebracho extract resulted in a significant decrease (*p* < 0.001) in the release of propionic acid. In contrast, QUE-F and CHE-F were the samples that resulted in the greatest release of butyric acid. However, the difference between QUE and QUE-F was not statistically significant, whereas the increase presented by CHE-F, compared to CHE, was considerably greater (*p* < 0.05).

**Table 1:** Short-Chain Fatty Acids (SCFAs) Produced after *In Vitro* Fermentation^a^

sample	acetic acid	propionic acid	butyric acid
BD	0.129 ± 0.020^a^	0.084 ± 0.024^a^	0.052 ± 0.004^a^
B-F	0.123 ± 0.013^a^	0.073 ± 0.011^a^	0.054 ± 0.008^a^
P	0.574 ± 0.021^b,c^	0.204 ± 0.004^b^	0.158 ± 0.003^b^
PC	0.624 ± 0.048^b^	0.169 ± 0.013^b,c^	0.149 ± 0.024^c^
PQ	0.606 ± 0.101^b^	0.183 ± 0.015^c,d^	0.130 ± 0.013^b,c^
QUE	0.484 ± 0.029^b,c,d^	0.198 ± 0.037^b,d^	0.145 ± 0.010^b,c^
CHE	0.460 ± 0.031^d^	0.181 ± 0.008^b,d,e^	0.133 ± 0.013^c^
QUE-F	0.418 ± 0.013^d^	0.137 ± 0.006^f^	0.154 ± 0.009^b,c^
CHE-F	0.476 ± 0.040^d^	0.159 ± 0.011^d,e,f^	0.161 ± 0.012^b^

a Different letters indicate statistically significant differences (*p* < 0.05) by ANOVA and Bonferroni post hoc test among samples, within each SCFA.

### Correlations among Antioxidant Capacity, Production of SCFA, and Taxon Abundances

**Figure 7:**
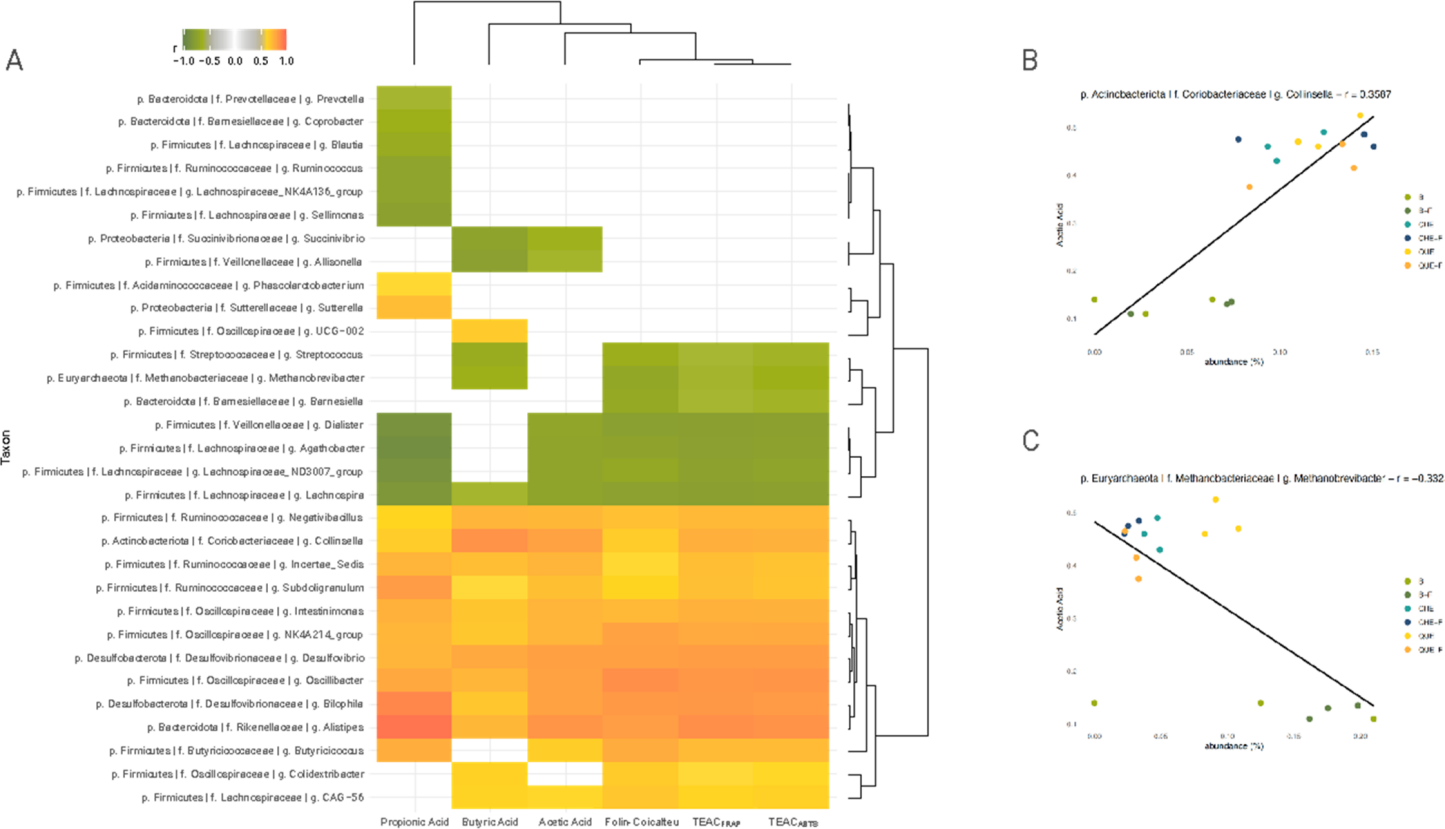
(A) Heatmap of the correlation coefficient (*r*) between microbial relative abundance and SCFA production, antioxidant capacity, and polyphenol abundance, calculated with the sPLS-based approach implemented in the mixOmics network function (correlation coefficient >0.6). Spearman correlation between (B) acetic acid and *Collinsella* and between (C) acetic acid and *Methanobrevibacter*.

Given that the pectin in the microcapsules overshadowed any potential effects of encapsulated tannins on the microbiota, we analyzed the correlations among tannin-related changes in antioxidant activity, production of SCFA, and taxon abundances using only the results obtained with nonencapsulated tannin extracts. First of all, it should be noted that the dendrogram in Figure 7A grouped the two techniques for measuring antioxidant activity (TEAC_ABTS_ and TEAC_FRAP_) with Folin–Ciocalteu. In fact, a close match among the three methods can be observed in the heatmap, showing that antioxidant activity and tannin content correlate with the abundance of the same bacterial taxa. Next to these, we detect the clustering of acetic acid, butyric acid, and lastly propionic acid production, which indicates that the production of acetic acid is more closely associated than that of the other SCFAs with polyphenol content and antioxidant activity. The same trend was found with Spearman correlations (Figure S1 in the Supporting Information).

Figure 7A shows that the presence of tannins increases the relative abundance of *Oscillibacter*, *Subdoligranulum*, Ruminococcaceae *incertae sedis*, *Negativibacillus*, *Collinsella*, *Bilophila*, *Alistipes*, *Intestinimonas*, Oscillospiraceae NK4A214 group, *Colidextribacter*, Lachnospiraceae CAG-56, *Butyricimonas*, and *Desulfovibrio.* Along with these, the release of SCFAs was also increased. Of interest, several of the mentioned taxa could be involved in tannin degradation. Indeed, *Collinsella* is well known for its potential for ring cleavage, dehydroxylation, and hydrogenation in polyphenols.^39^ Analogously, *Intestinimonas* is a relatively recently described genus, phylogenetically related to members of the genus *Flavonifractor*, which plays a key role in proanthocyanidin catabolism.^40,41^

Conversely, the relative abundance of *Dialister*, *Agathobacter*, Lachnospiraceae ND3007 group, *Lachnospira*, *Methanobrevibacter*, *Streptococcus*, and *Barnesiella* showed a negative correlation with the presence of tannins, and also with the production of all or some SCFAs. Tannins have been widely investigated for their potential for affecting the level of methane produced in ruminants, through the modulation of methanogenic genera, and *Methanobrevibacter* among them.^42^ The negative correlation with this taxon suggests that supplementation with tannins may help reduce bloating discomfort associated with methane production in humans.

The plots in Figure 7B,C further emphasize that the correlation between some taxa and acetate production is dependent on the presence of tannins. Taking *Collinsella* and *Methanobrevibacter* as examples of positive and negative correlation, respectively, it can be seen that only fermentations containing tannins, but not the fermentation blanks, contain high levels of acetate correlating with a high abundance of *Collinsella* (Figure 7B) and low abundance of *Methanobrevibacter* (Figure 7C). In contrast, in the case of some other taxa, their abundance correlated positively (i.e., *Sutterella*, *Phascolarctobacterium*, Oscillospiraceae UCG002) or negatively (i.e., Lachnospiraceae NK4A136 group, *Ruminococcus, Sellimonas, Coprobacter, Prevotella, Blautia, Allisonella*, *Succinivibrio*) with the production of SCFAs, but the variation of these taxa was not affected by the abundance of tannins (Figure 7A).

In conclusion, this study has shown that the complex structure formed between pectin and tannins during encapsulation via a gelation process results in an insufficient release of tannins after *in vitro* digestion and fermentation.

These results indicate that the binding pectin-tannins is so strong that, although microcapsules are efficiently generated, they cannot be used to deliver these bioactive compounds into the human body. Therefore, alternative encapsulation materials that do not interact as strongly with tannins should be tested in order to meet the basic requirement of releasing the transported molecules.

On the other hand, this study showed that tannins, particularly those extracted from quebracho, interact differently with the intestinal microbiota depending on whether they undergo prior digestion or not. The antioxidant activity exerted is greater when tannin extracts are submitted to both *in vitro* digestion and fermentation. Moreover, in the case of the quebracho extract, SCFA production by the microbiota is also elevated after digestion. Therefore, the ingestion of unencapsulated tannins can be recommended, as it produces beneficial effects on the gut microbiota that can be enhanced by the digestion step. Moreover, if microencapsulation is desired in order to mask the flavor of the tannins, it would be necessary to find a solution that releases tannins directly into the stomach to maximize their bioactive effect. However, some specific effects, such as the capacity to modulate the abundance of *Methanobrevibacter*, are better achieved when tannin extracts remain undigested so that the development of encapsulation processes that minimize digestion may also be warranted for this specific purpose. This underscores that the mode of tannin delivery will need to be adjusted to the aims pursued in each individual, highlighting the importance of personalized approaches in the future of human nutrition.

## Supporting Information Available

Indexes of α diversity and relative abundance of phyla (Table S1) and Spearman correlations between microbial relative abundance at genus level and SCFA production, antioxidant capacity (TEAC_FRAP_, TEAC_ABTS_), and polyphenol abundance (Folin–Ciocalteu) (Figure S1) (PDF)

## Abbreviations

QUE: quebracho extract
CHE: chestnut extract
QUE-F: quebracho extract just fermented
CHE: chestnut extract just fermented
P: pectin microbeads w/o tannins
PQ: pectin microbeads with quebracho
PC: pectin microbeads with chestnut
I: original inoculum
B: blank digested and fermented
B-F: blank just fermented
PT: pectin capsules with tannins
T: tannin extracts
T-F: tannin extracts just fermented
