# Supplementary material for: Evaluation of Tannin-Delivery Approaches for Gut Microbiota Modulation: Comparison of Pectin-Based Microcapsules and Unencapsulated Extracts
Source: J Agric Food Chem. 2023 Jul 11;71(38):13988–99. doi: 10.1021/acs.jafc.3c02949 (PMC10540208; doi:10.1021/acs.jafc.3c02949)
Supplement: Supplementary file 1 — jf3c02949_si_001.pdf [file jf3c02949_si_001.pdf]

Supplementary Material

Evaluation of tannin-delivery approaches for gut microbiota modulation: comparison of pectin-based microcapsules and unencapsulated extracts

Silvia Molino[a,b], Alberto Lerma-Aguilera[c], Laura G. Gómez-Mascaraque[d], José Ángel Rufián-Henares[a,e*], M. Pilar Francino[c,f]

[a] Departamento de Nutrición y Bromatología, Instituto de Nutrición y Tecnología de los Alimentos, Centro de Investigación Biomédica, Universidad de Granada, Granada, 18016, Spain.

[b] Silvateam Spa, R&D Unit, San Michele Mondoví, 12080, Italy

[c]Area de Genòmica i Salut, Fundació per al Foment de la Investigació Sanitária i Biomèdica de la Comunitat Valenciana (FISABIO-Salut Pública), València, 46020, Spain

[d]Food Chemistry and Technology Department, Teagasc Moorepark Food Research Centre, Fermoy, Co. Cork, P61 C996, Ireland.

[e]Instituto de Investigación Biosanitaria ibs.Granada, Granada, 18012, Spain.

[f]CIBER en Epidemiología y Salud Pública, Madrid, 28029, Spain.

*** Correspondence:**

José Ángel Rufián Henares
jarufian@ugr.es

1. Supplementary Table

Table S1. Indexes of α diversity and relative abundance of phyla. I (original inoculum), B (blank digested and fermented), B-F (blank just fermented), P (pectin capsules w/o tannins), PQ (pectin capsules with quebracho), PC (pectin capsules with chestnut), QUE (quebracho extract digested and fermented), CHE (chestnut extract digested and fermented), QUE-F (quebracho extract just fermented), CHE -F (chestnut extract just fermented.

	Diversity indexes					Phyla composition					
**Sample**	Shannon	Chao1	SE.Chao1	ACE	SE.ACE	Actinobacteria	Bacteroidota	Firmicutes	Proteobacteria	Verrucomicrobia	other
I	4.578	293.0	0.499	293.1	5.310	0.826	40.24	48.07	8.017	1.67	0.230
B	4.549	265.8	1.478	265.5	6.096	0.139	42.46	38.02	16.776	1.615	0.151
B-F	4.665	266.0	0.097	266.1	5.981	0.335	40.11	43.19	12.94	2.479	0.172
P	4.396	283.3	0.222	283.4	6.612	0.416	44.60	38.92	13.61	1.55	0.178
PQ	4.543	359.9	2.531	358.6	6.915	0.204	46.90	36.24	14.01	1.84	0.156
PC	4.579	347.8	0.942	347.7	7.024	0.389	45.90	37.89	13.23	1.41	0.231
QHE	4.756	349.8	0.998	349.6	6.957	0.531	41.00	40.81	14.26	1.918	0.292
CHE	4.721	346.7	1.645	346.3	7.201	0.502	40.55	41.41	14.52	1.703	0.260
QUE-F	4.829	370.5	1.736	370.0	7.214	0.609	42.77	42.62	11.32	1.716	0.189
CHE-F	4.784	343.2	1.043	343.1	6.735	0.627	41.77	42.68	11.982	1.899	0.205

2. Supplementary Figures

Figure S1

Spearman Correlations between microbial relative abundance at genus level and SCFA production, antioxidant capacity and polyphenol abundance.

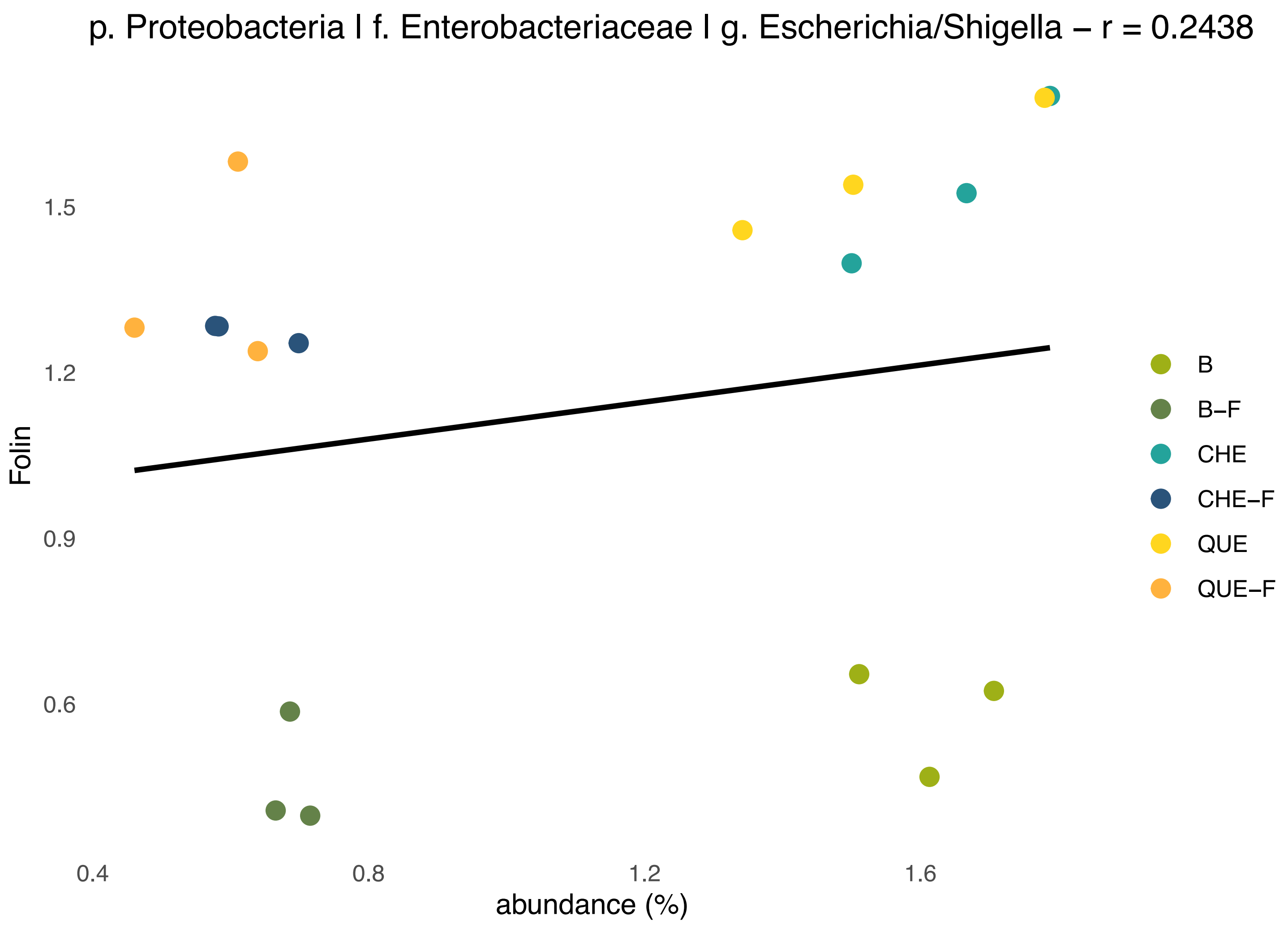
p. Proteobacteria l f. Enterobacteriaceae l g. Escherichia/Shigella – r = 0.2438
Folin
1.5
1.2
0.9
0.6
0.4
0.8
1.2
1.6
abundance (%)
B
B–F
CHE
CHE–F
QUE
QUE–F

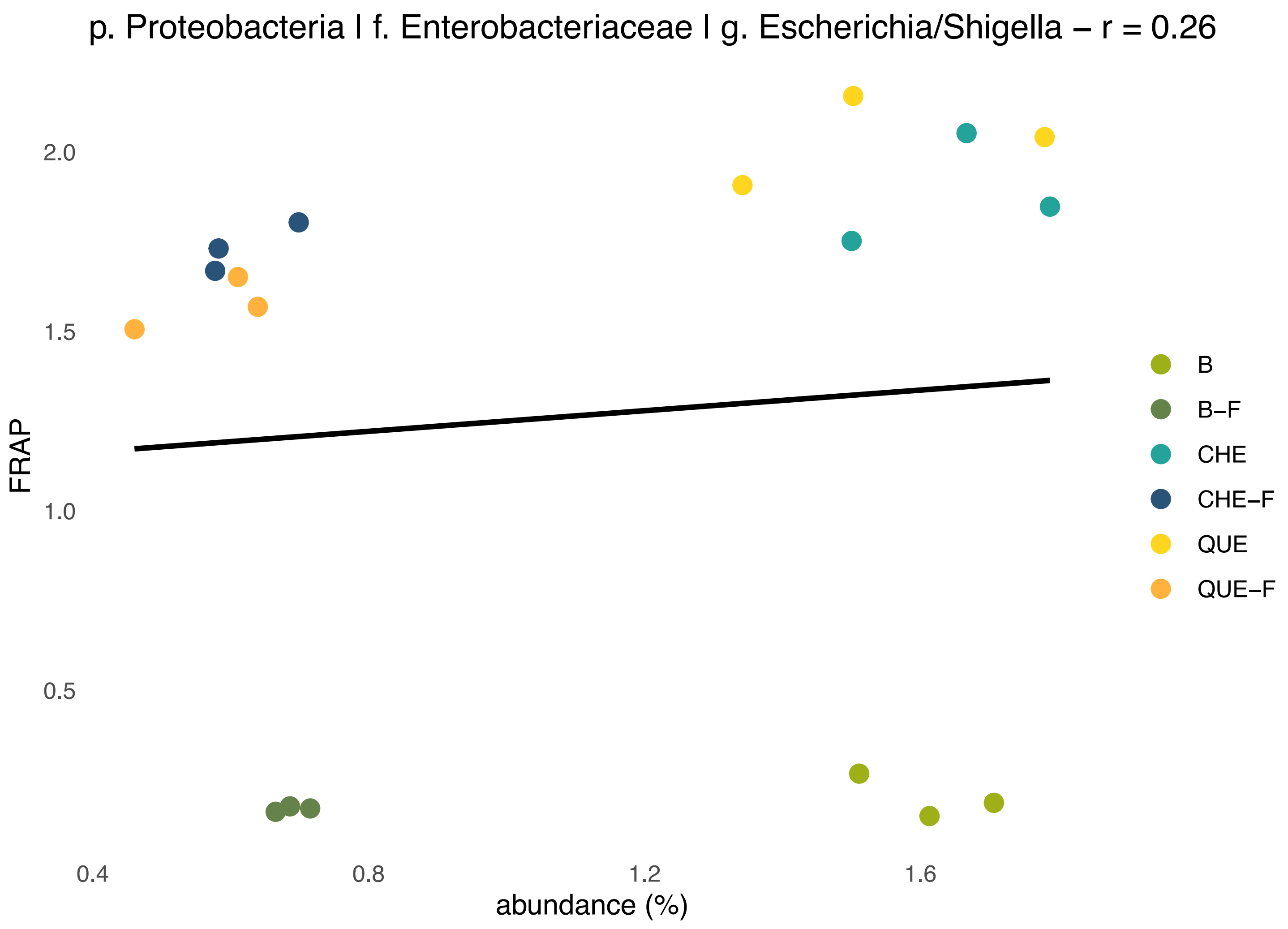

p. Proteobacteria I f. Enterobacteriaceae I g. Escherichia/Shigella – r = 0.26
2.0
1.5
1.0
0.5
FRAP
0.4
0.8
1.2
1.6
abundance (%)
B
B–F
CHE
CHE–F
QUE
QUE–F

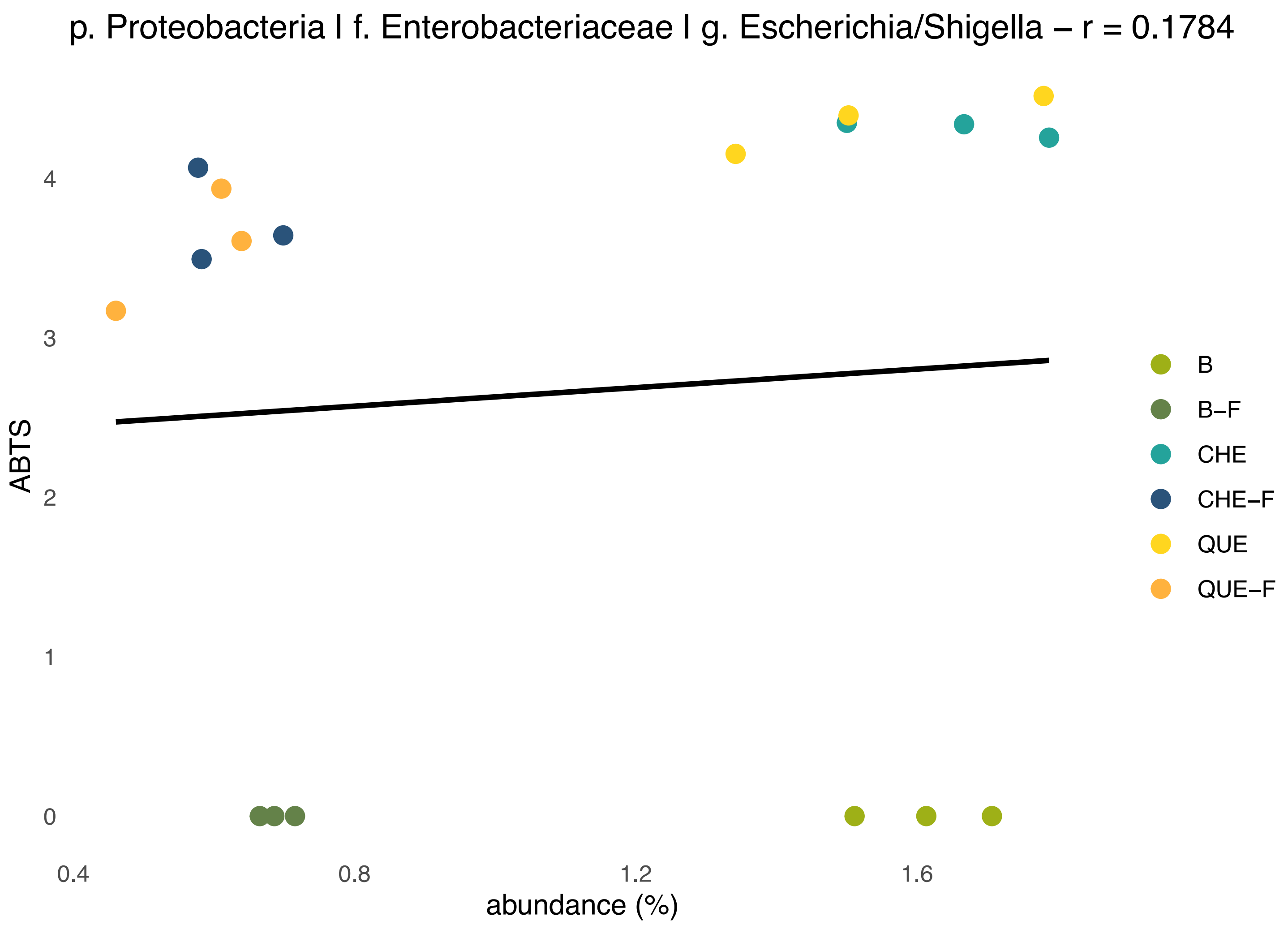
p. Proteobacteria I f. Enterobacteriaceae I g. Escherichia/Shigella – r = 0.1784
ABTS
4
3
2
1
0
0.4
0.8
1.2
1.6
abundance (%)
B
B–F
CHE
CHE–F
QUE
QUE–F

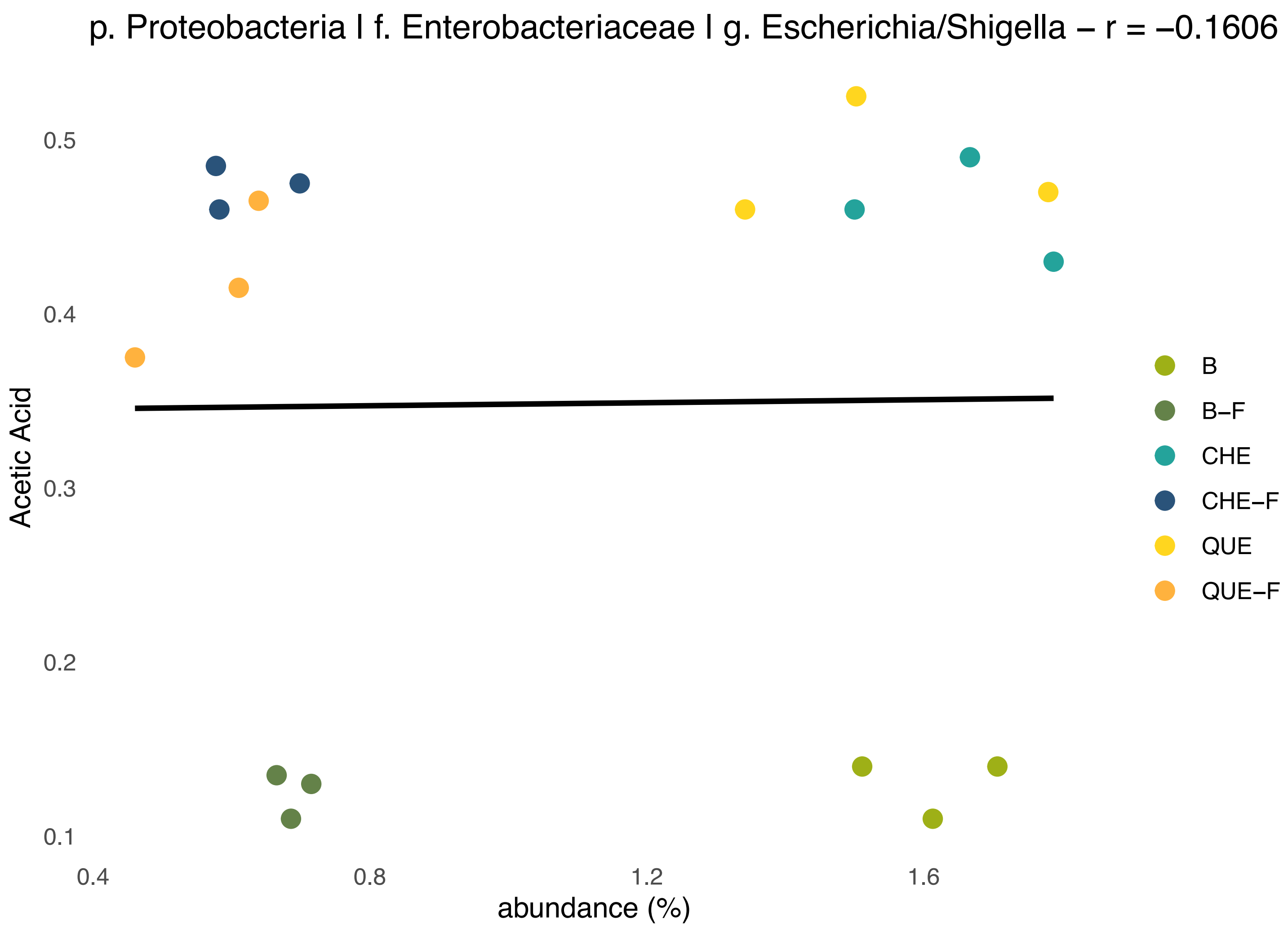

p. Proteobacteria | f. Enterobacteriaceae | g. Escherichia/Shigella – r = –0.1606
Acetic Acid
0.5
0.4
0.3
0.2
0.1
0.4
0.8
1.2
1.6
abundance (%)
B
B–F
CHE
CHE–F
QUE
QUE–F

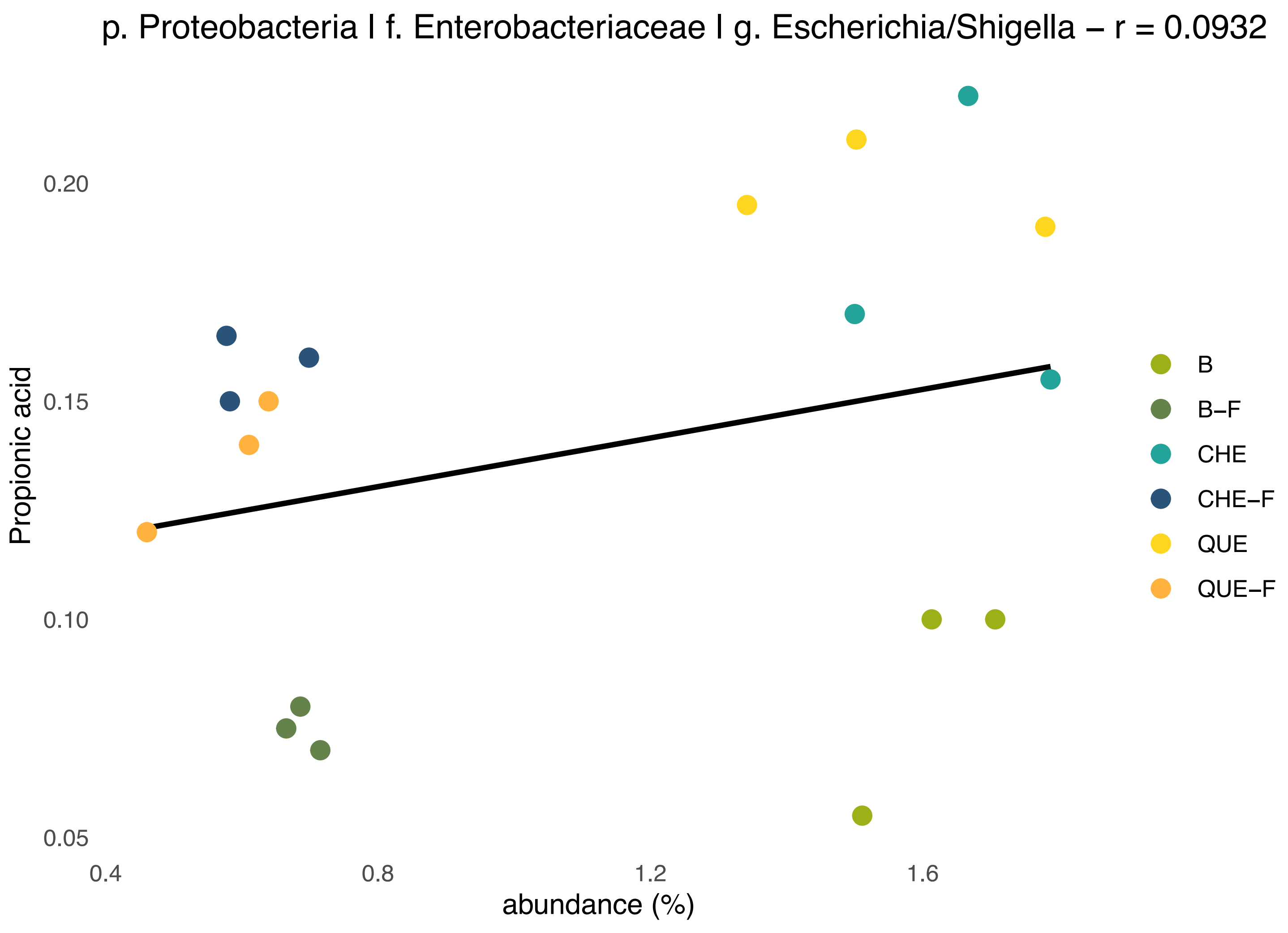
p. Proteobacteria I f. Enterobacteriaceae I g. Escherichia/Shigella – r = 0.0932
Propionic acid
0.20
0.15
0.10
0.05
0.4
0.8
1.2
1.6
abundance (%)
B
B–F
CHE
CHE–F
QUE
QUE–F

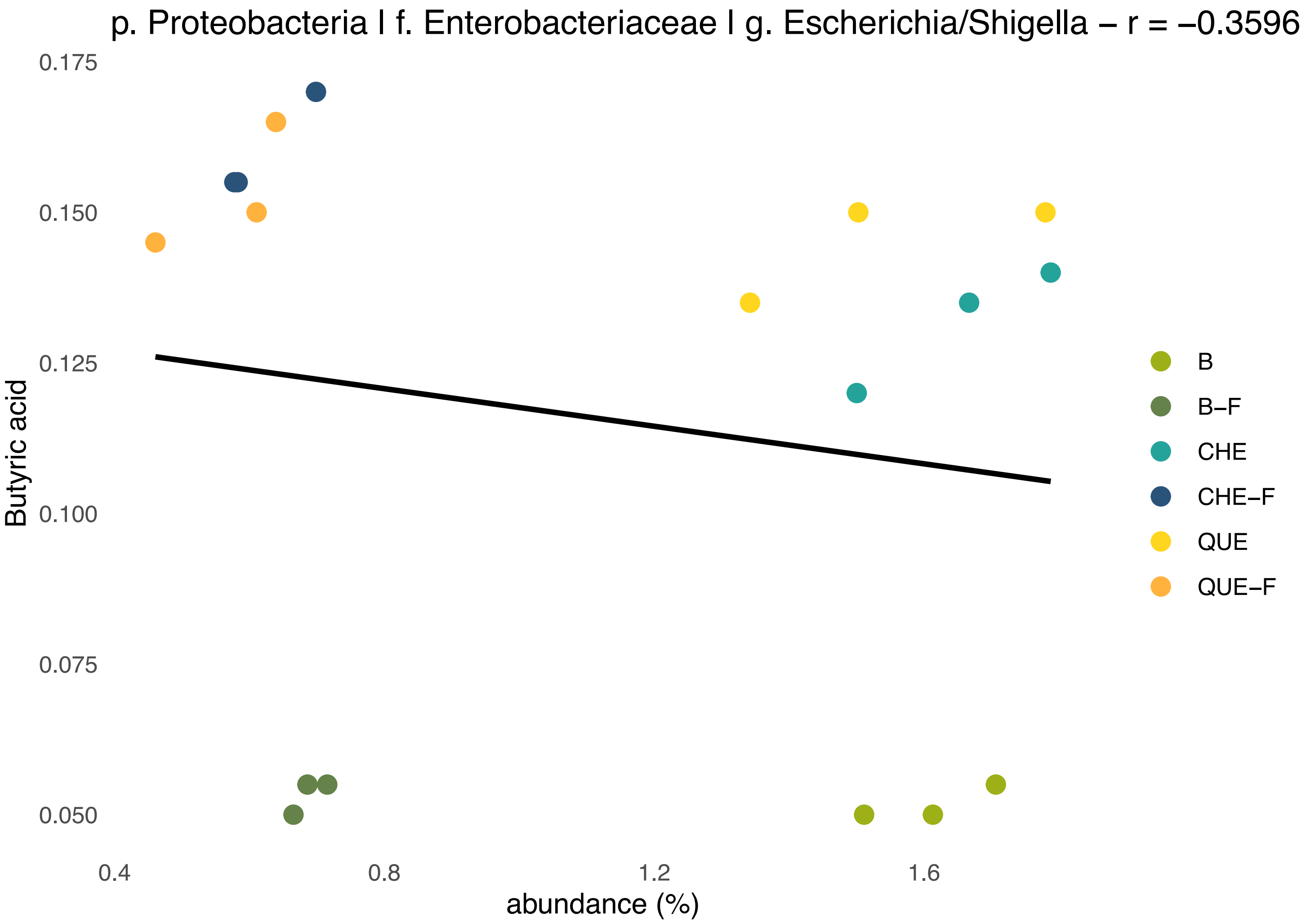
p. Proteobacteria | f. Enterobacteriaceae | g. Escherichia/Shigella – r = −0.3596
Butyric acid
0.175
0.150
0.125
0.100
0.075
0.050
0.4
0.8
1.2
1.6
abundance (%)
B
B−F
CHE
CHE−F
QUE
QUE−F

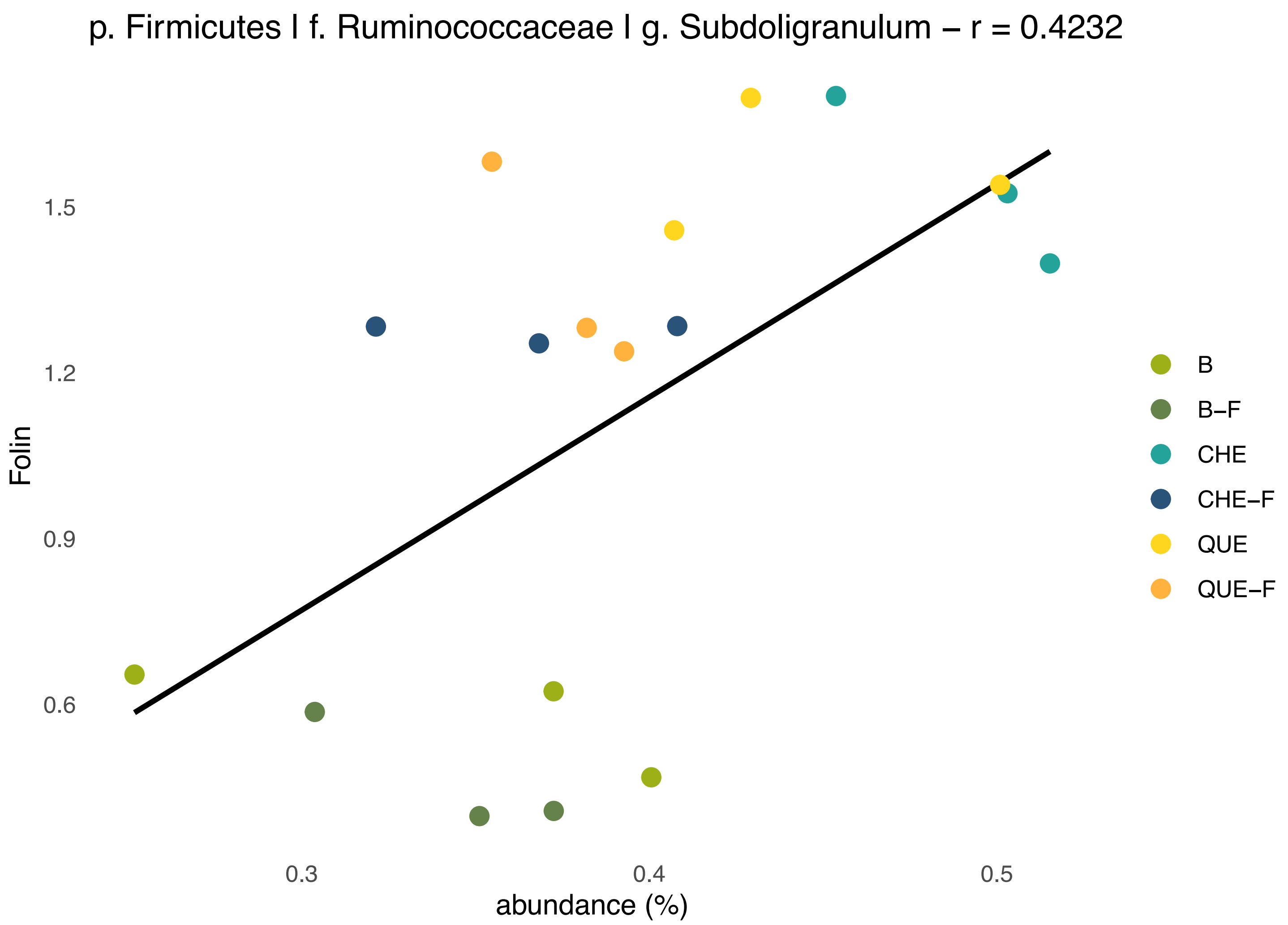
p. Firmicutes l f. Ruminococcaceae l g. Subdoligranulum – r = 0.4232
Folin
1.5
1.2
0.9
0.6
0.3
0.4
0.5
abundance (%)
B
B–F
CHE
CHE–F
QUE
QUE–F

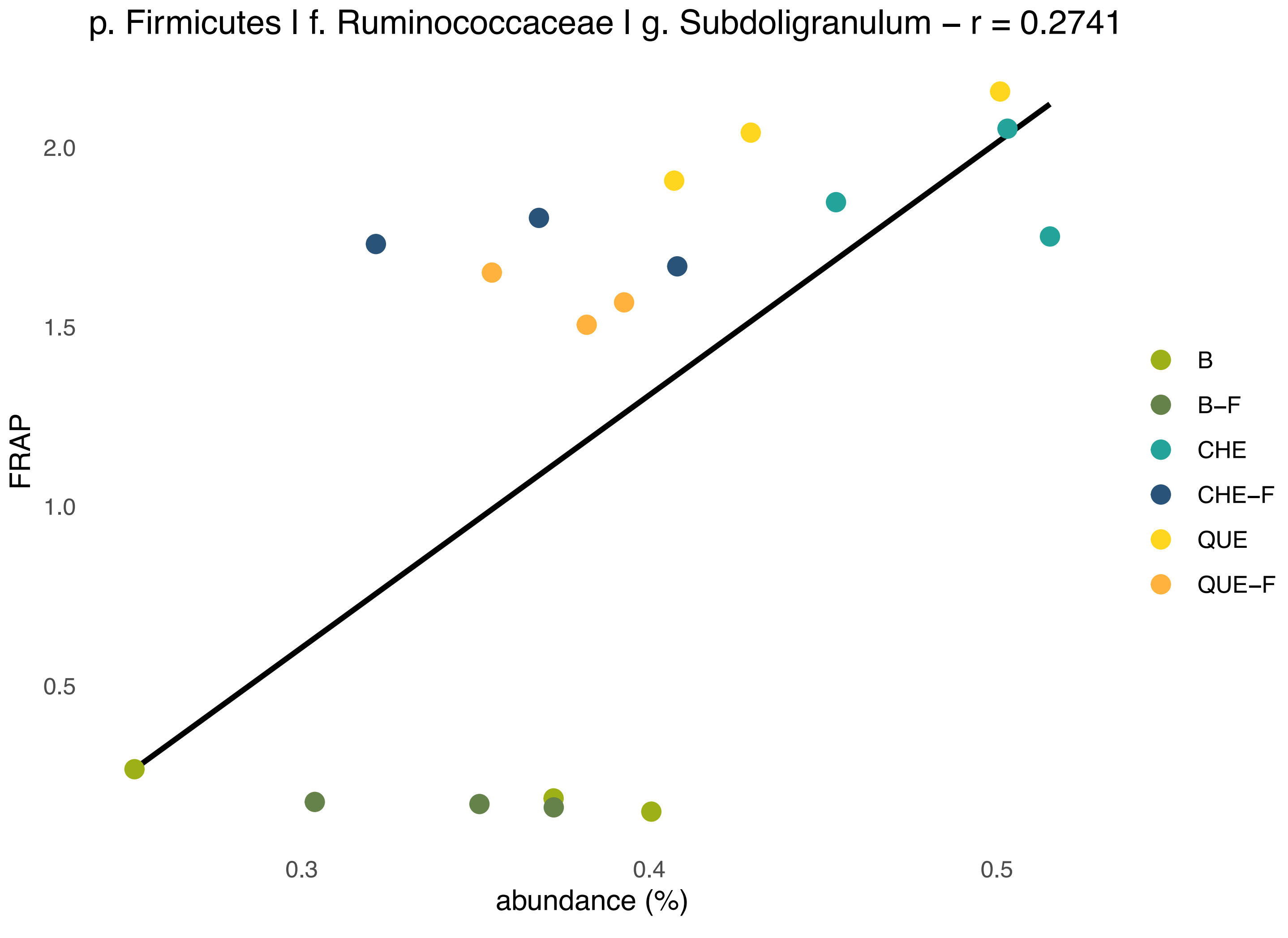
p. Firmicutes I f. Ruminococcaceae I g. Subdoligranulum – r = 0.2741
FRAP
2.0
1.5
1.0
0.5
0.3
0.4
0.5
abundance (%)
B
B–F
CHE
CHE–F
QUE
QUE–F

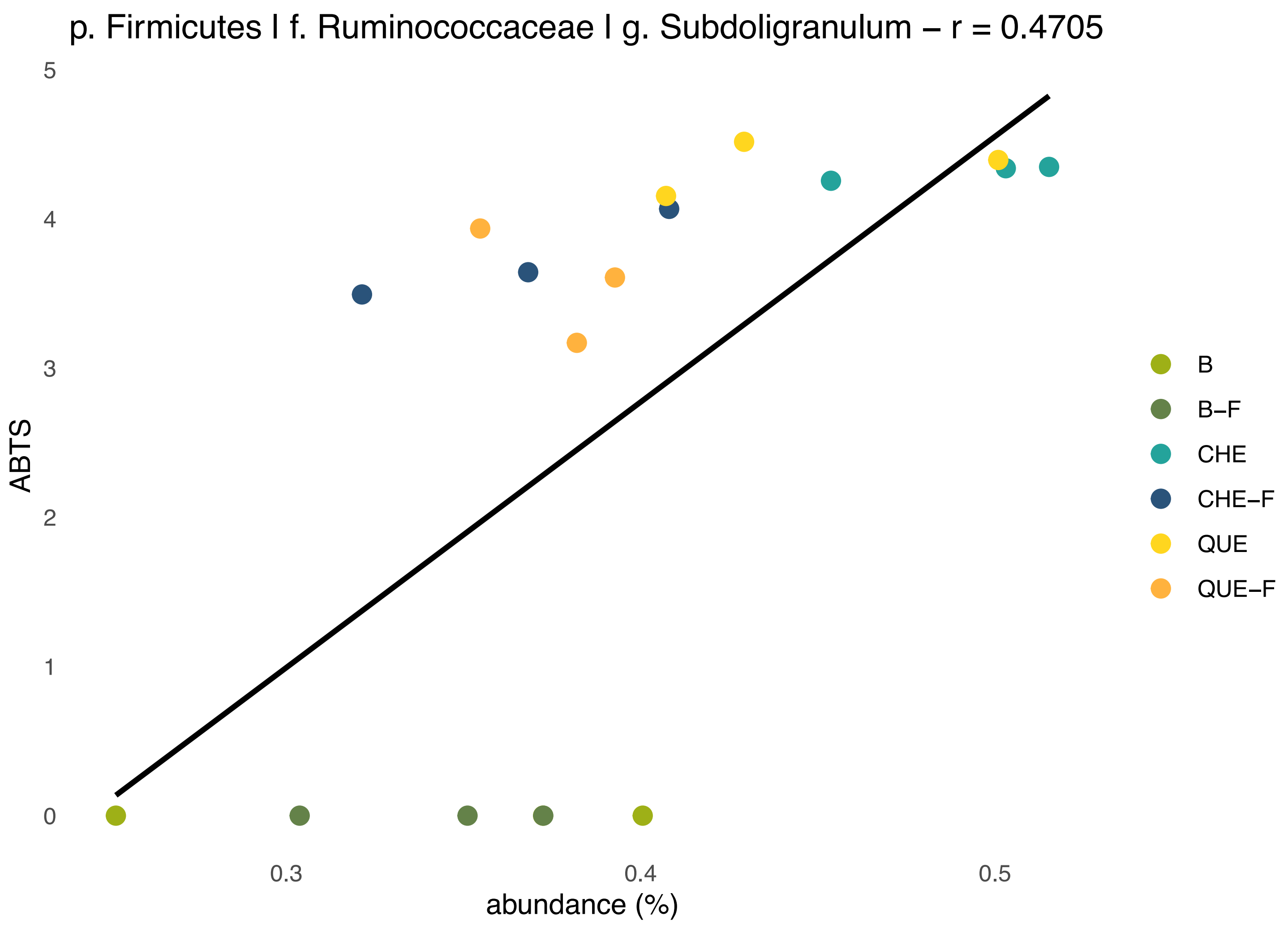
p. Firmicutes I f. Ruminococcaceae I g. Subdoligranulum – r = 0.4705
ABTS
abundance (%)
0
1
2
3
4
5
0.3
0.4
0.5
B
B–F
CHE
CHE–F
QUE
QUE–F

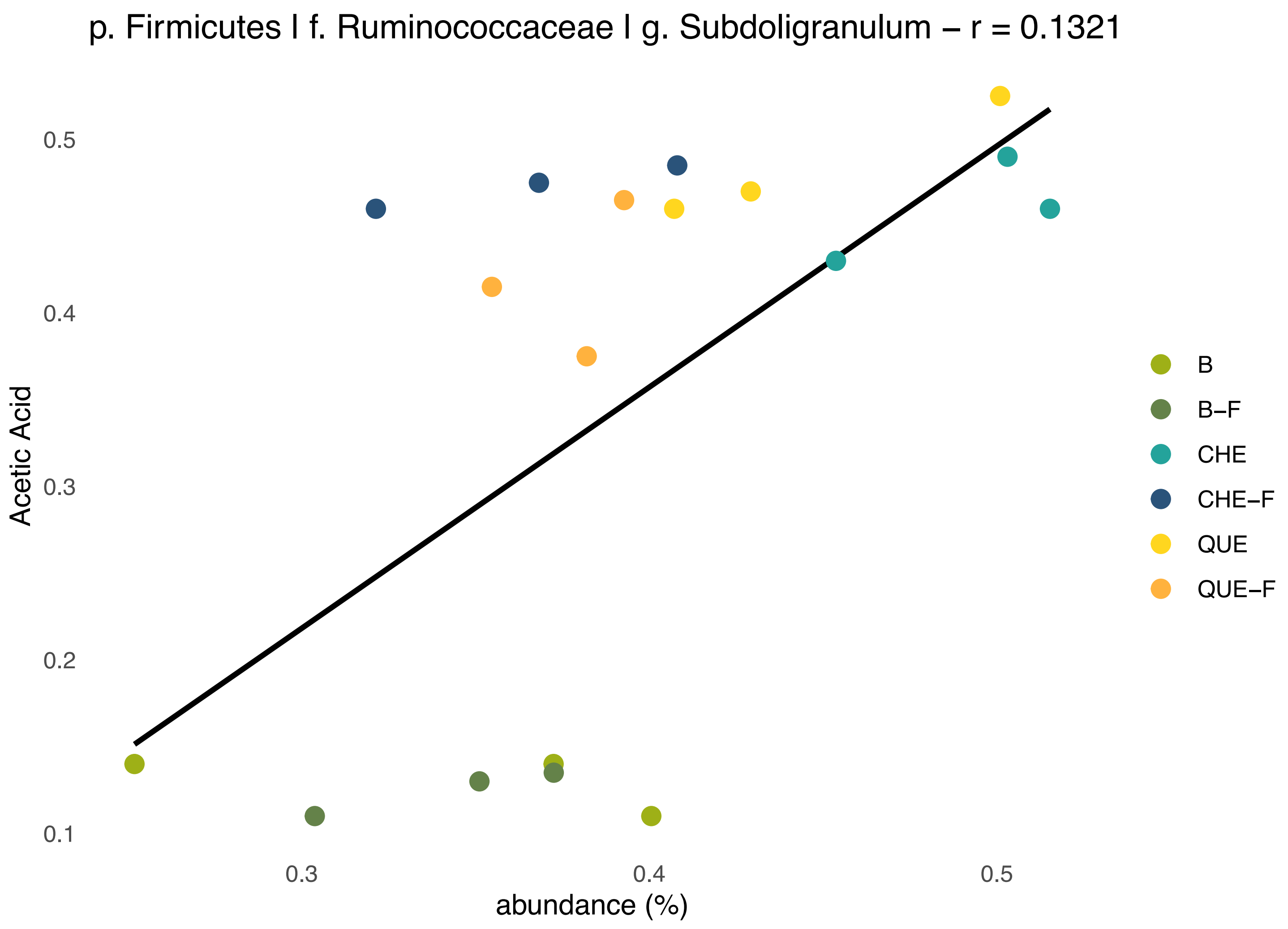
p. Firmicutes I f. Ruminococcaceae I g. Subdoligranulum – r = 0.1321
Acetic Acid
0.5
0.4
0.3
0.2
0.1
0.3
0.4
0.5
abundance (%)
B
B–F
CHE
CHE–F
QUE
QUE–F

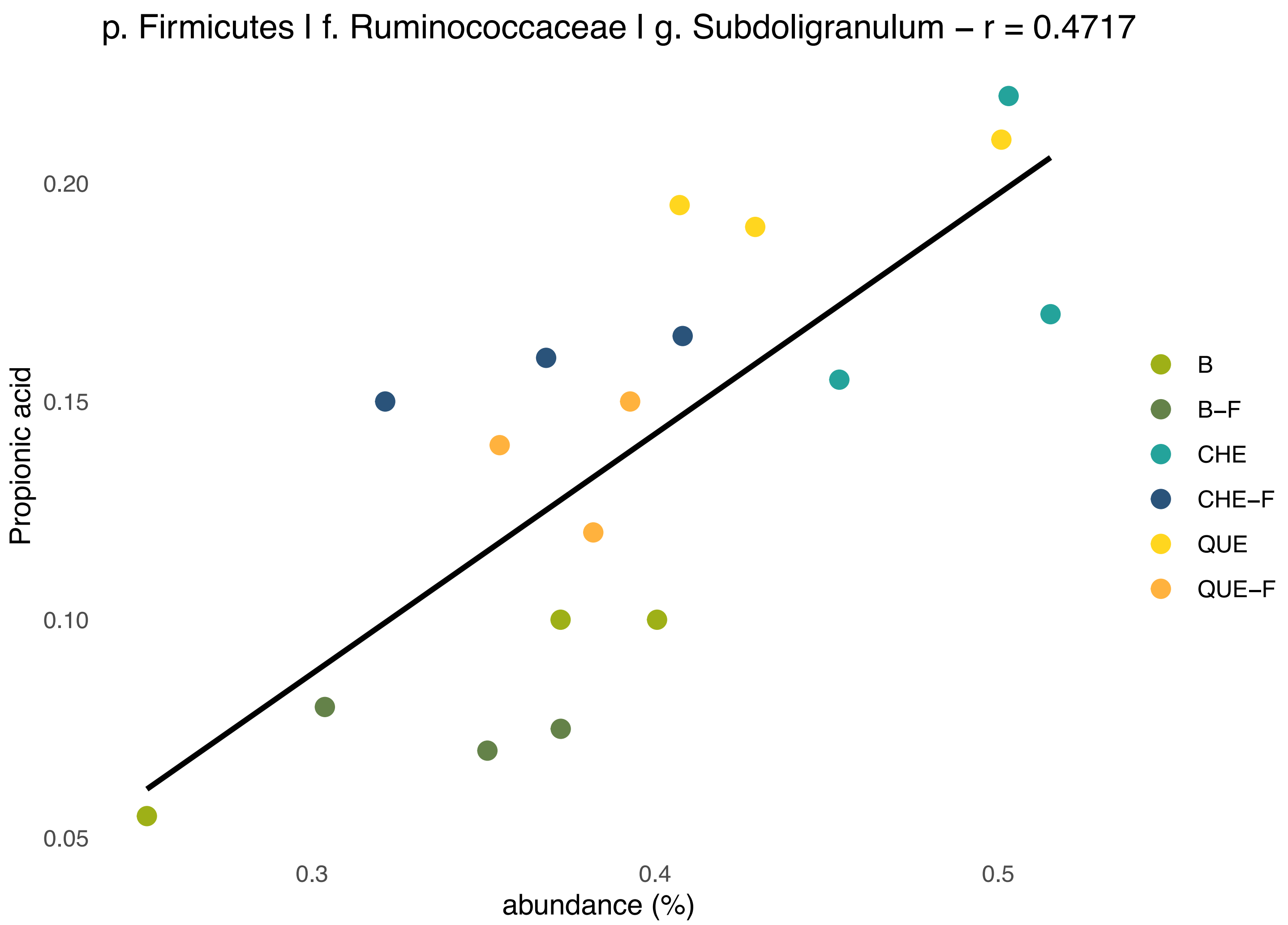
p. Firmicutes l f. Ruminococcaceae l g. Subdoligranulum − r = 0.4717
Propionic acid
0.20
0.15
0.10
0.05
0.3
0.4
0.5
abundance (%)
B
B−F
CHE
CHE−F
QUE
QUE−F

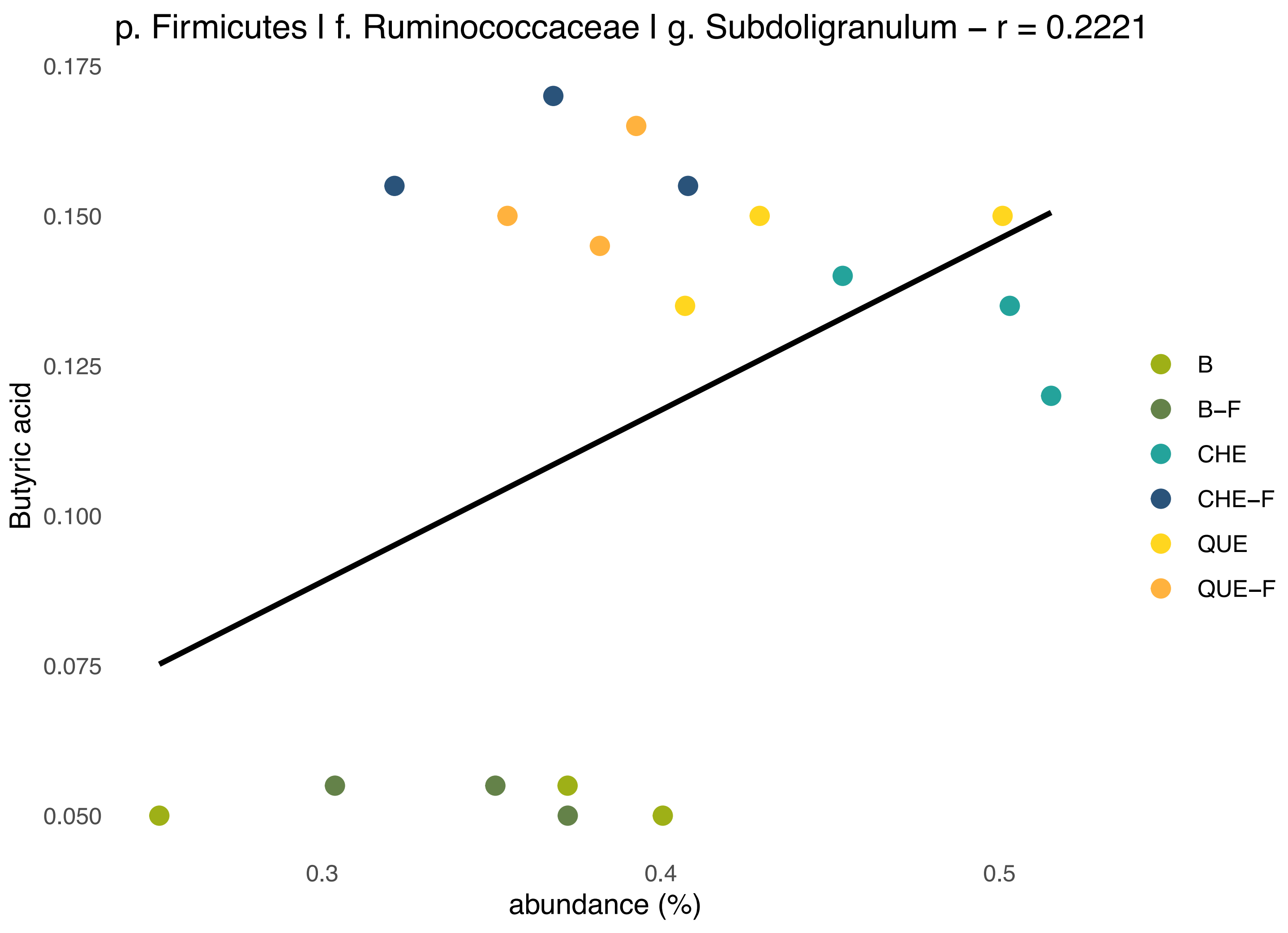

p. Firmicutes I f. Ruminococcaceae I g. Subdoligranulum – r = 0.2221
Butyric acid
0.175
0.150
0.125
0.100
0.075
0.050
0.3
0.4
0.5
abundance (%)
B
B–F
CHE
CHE–F
QUE
QUE–F

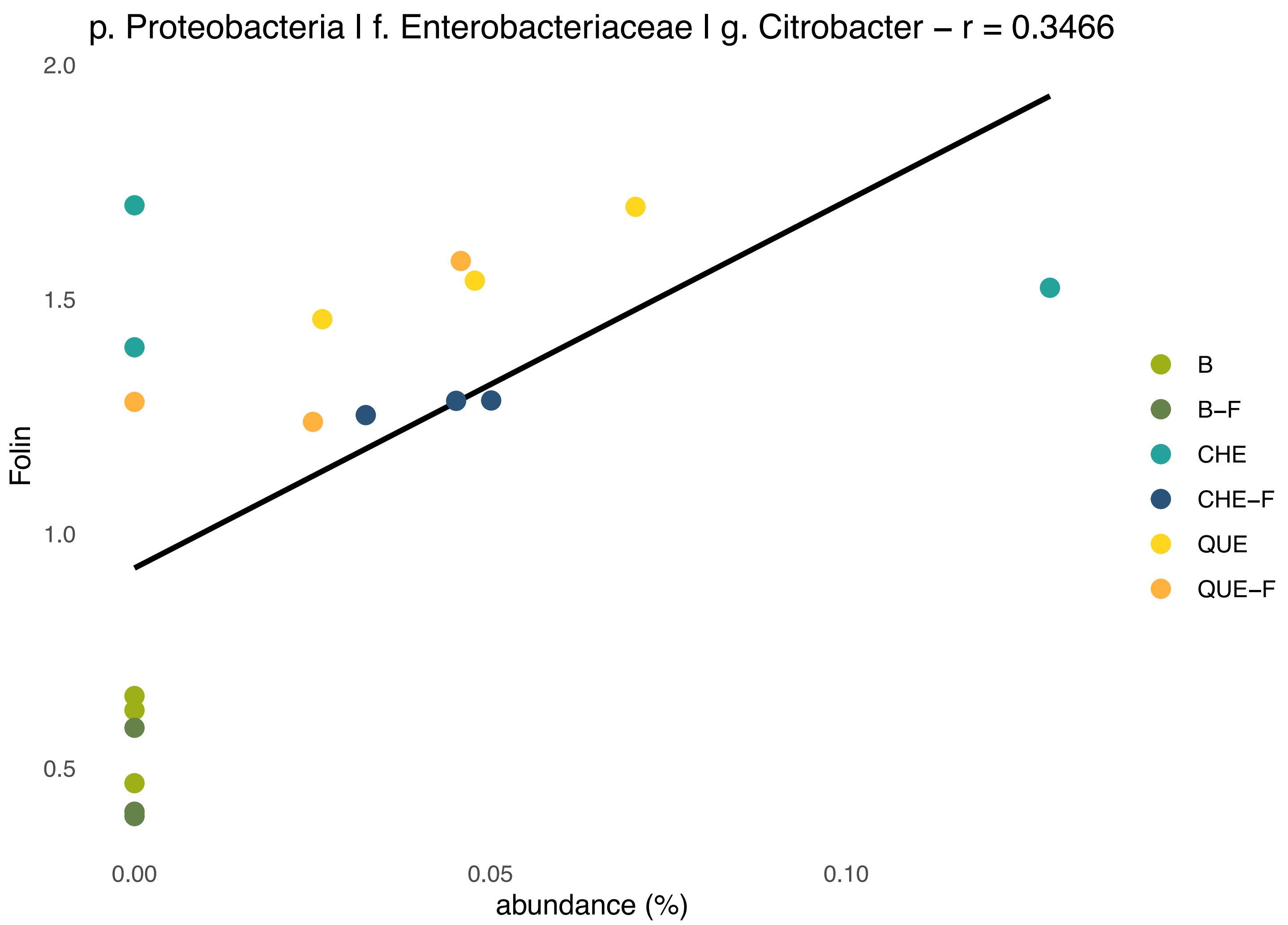

p. Proteobacteria | f. Enterobacteriaceae | g. Citrobacter – r = 0.3466
2.0
1.5
1.0
0.5
Folin
0.00
0.05
0.10
abundance (%)
B
B–F
CHE
CHE–F
QUE
QUE–F

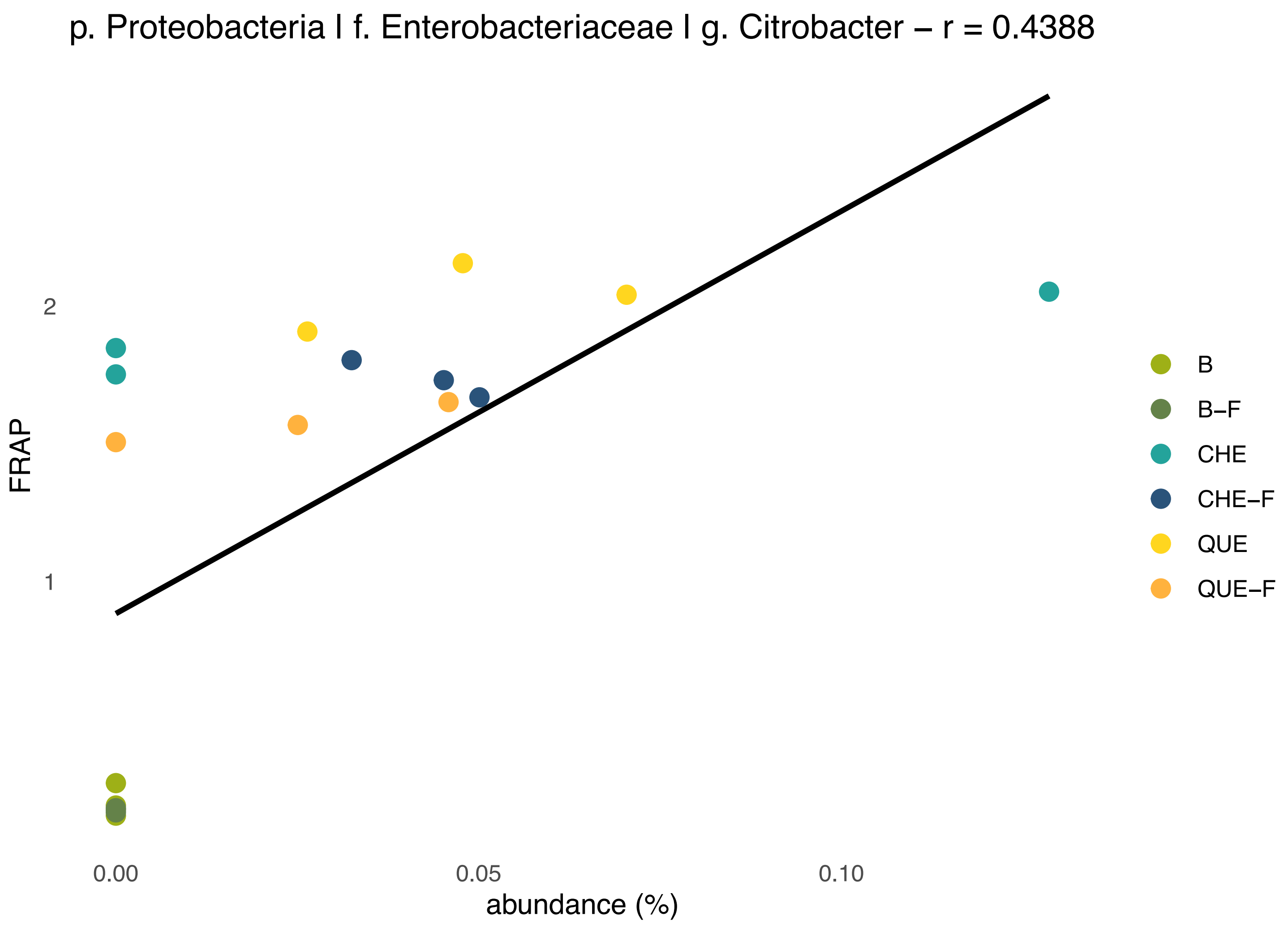

p. Proteobacteria | f. Enterobacteriaceae | g. Citrobacter – r = 0.4388
FRAP
2
1
0.00
0.05
0.10
abundance (%)
B
B–F
CHE
CHE–F
QUE
QUE–F

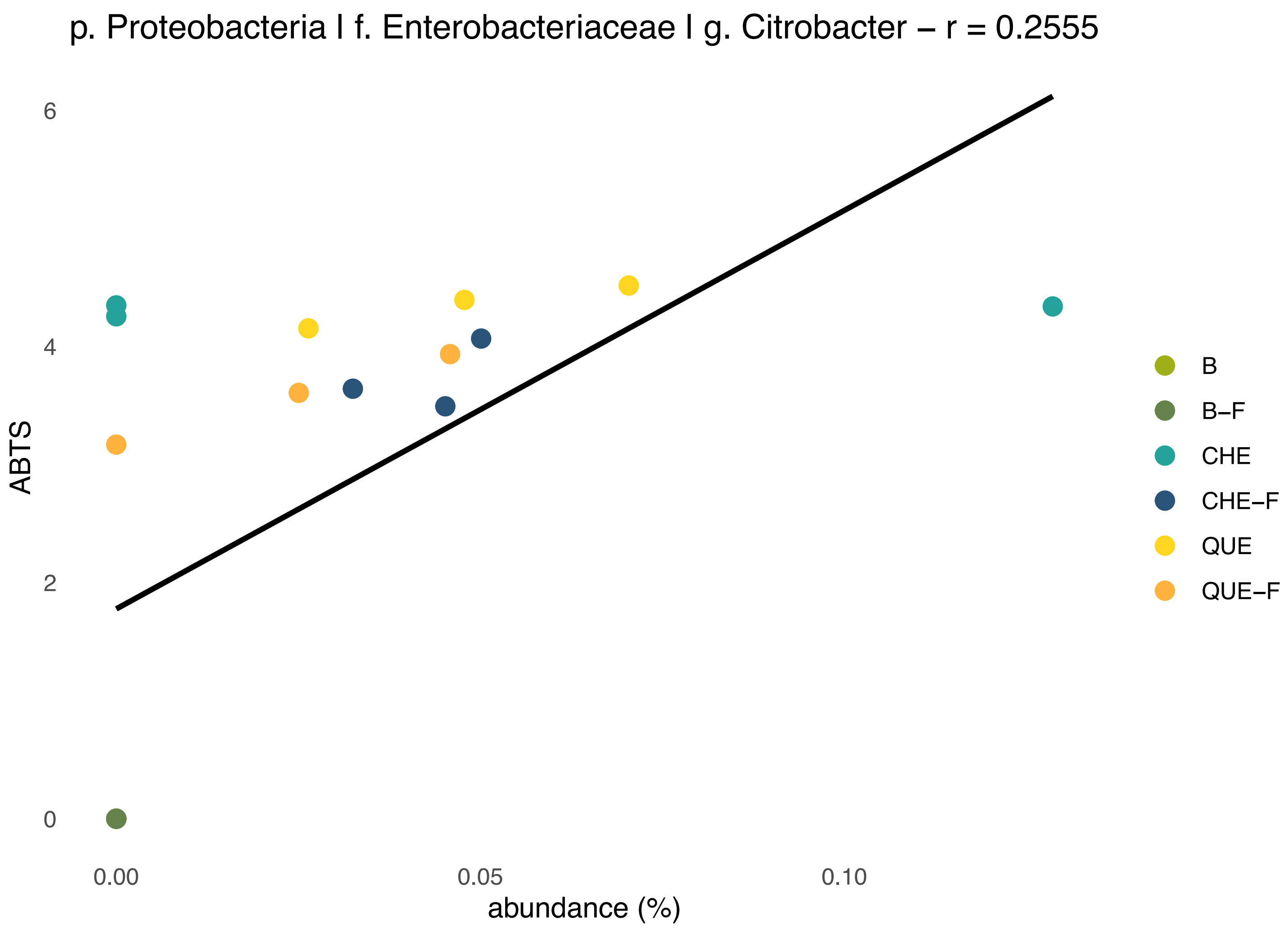
p. Proteobacteria | f. Enterobacteriaceae | g. Citrobacter – r = 0.2555
ABTS
6
4
2
0
0.00
0.05
0.10
abundance (%)
B
B–F
CHE
CHE–F
QUE
QUE–F

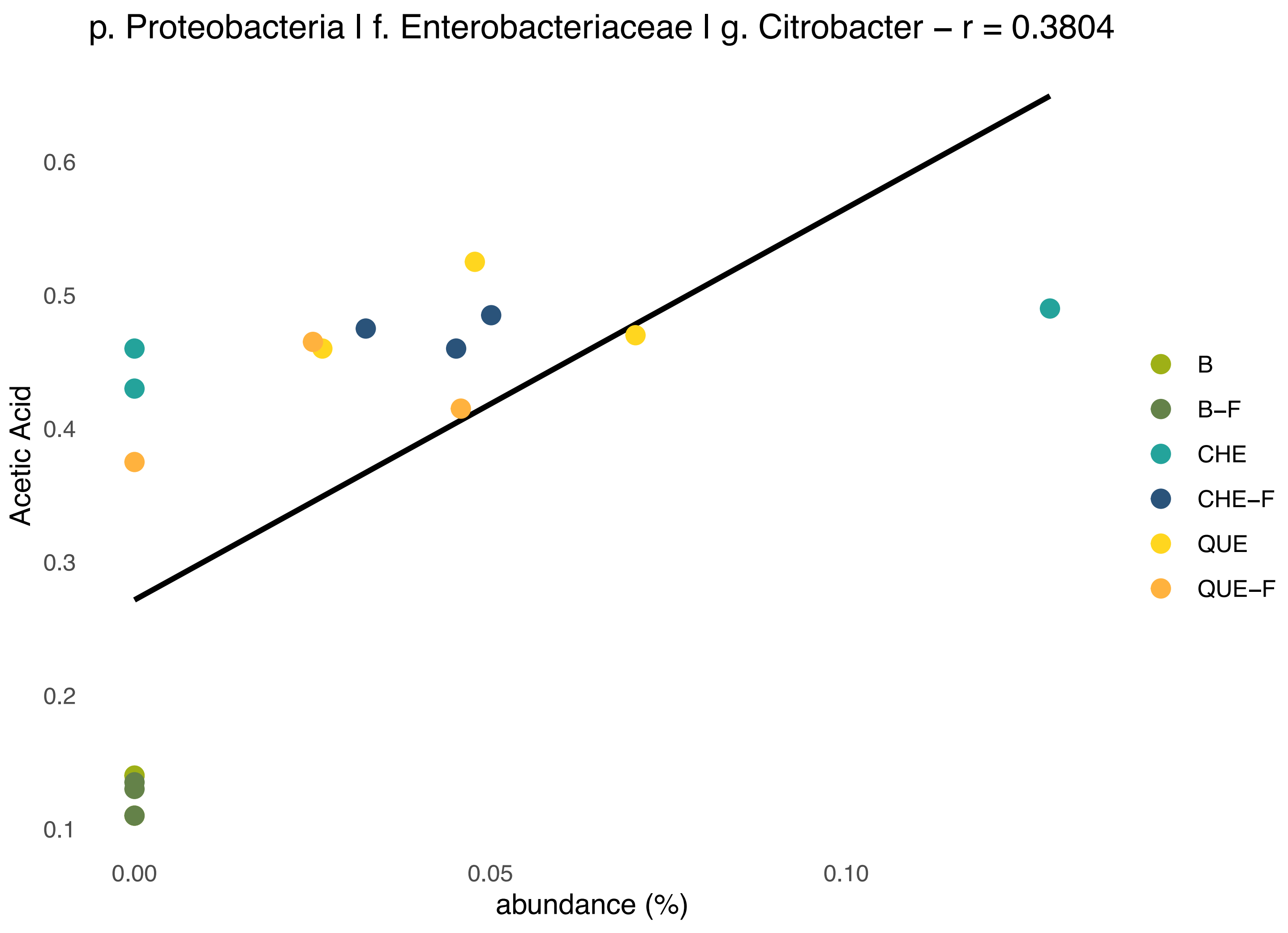
p. Proteobacteria I f. Enterobacteriaceae I g. Citrobacter – r = 0.3804
Acetic Acid
0.6
0.5
0.4
0.3
0.2
0.1
0.00
0.05
0.10
abundance (%)
B
B–F
CHE
CHE–F
QUE
QUE–F

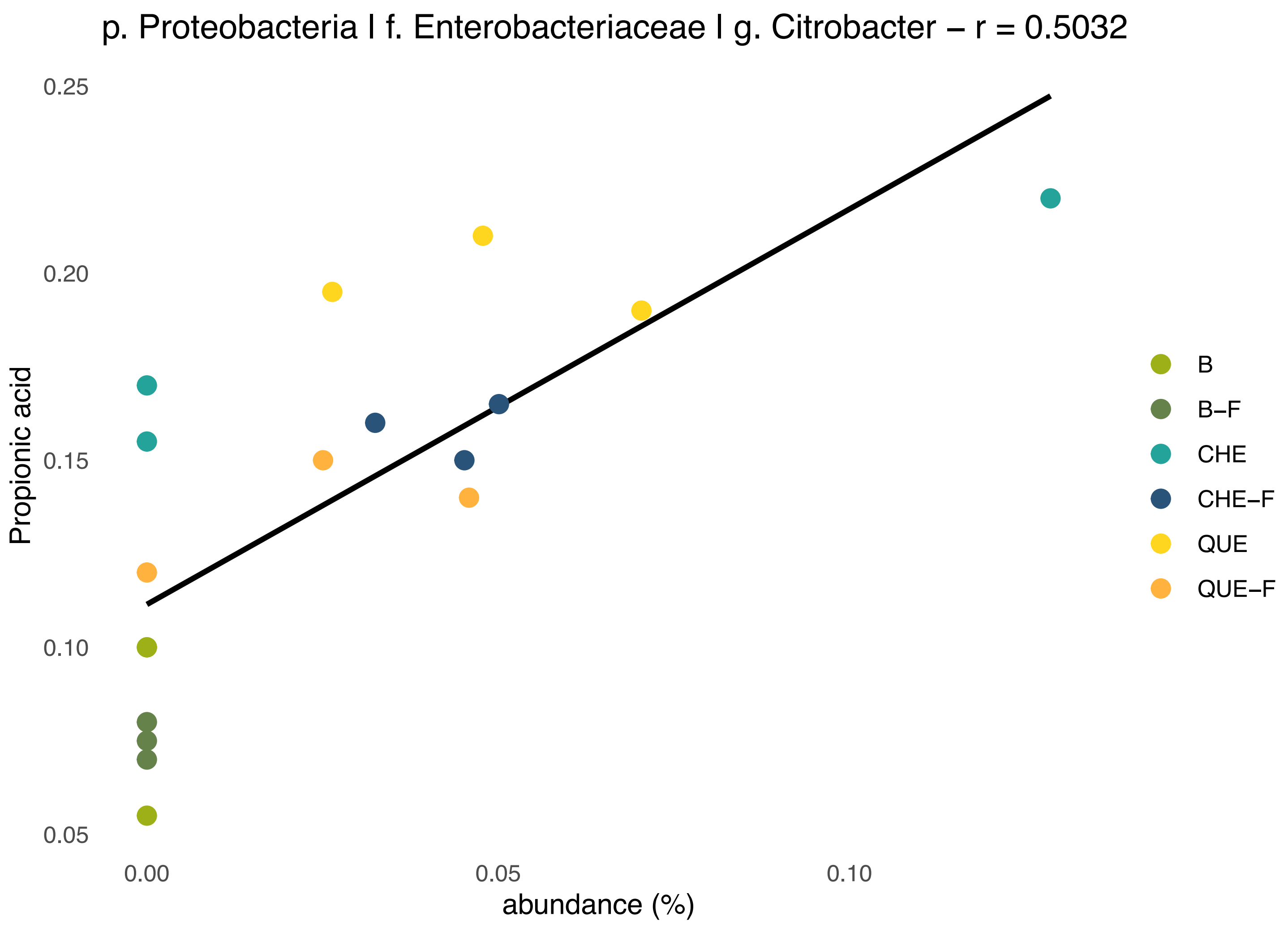

p. Proteobacteria I f. Enterobacteriaceae I g. Citrobacter – r = 0.5032
0.25
0.20
0.15
0.10
0.05
Propionic acid
0.00
0.05
0.10
abundance (%)
B
B–F
CHE
CHE–F
QUE
QUE–F

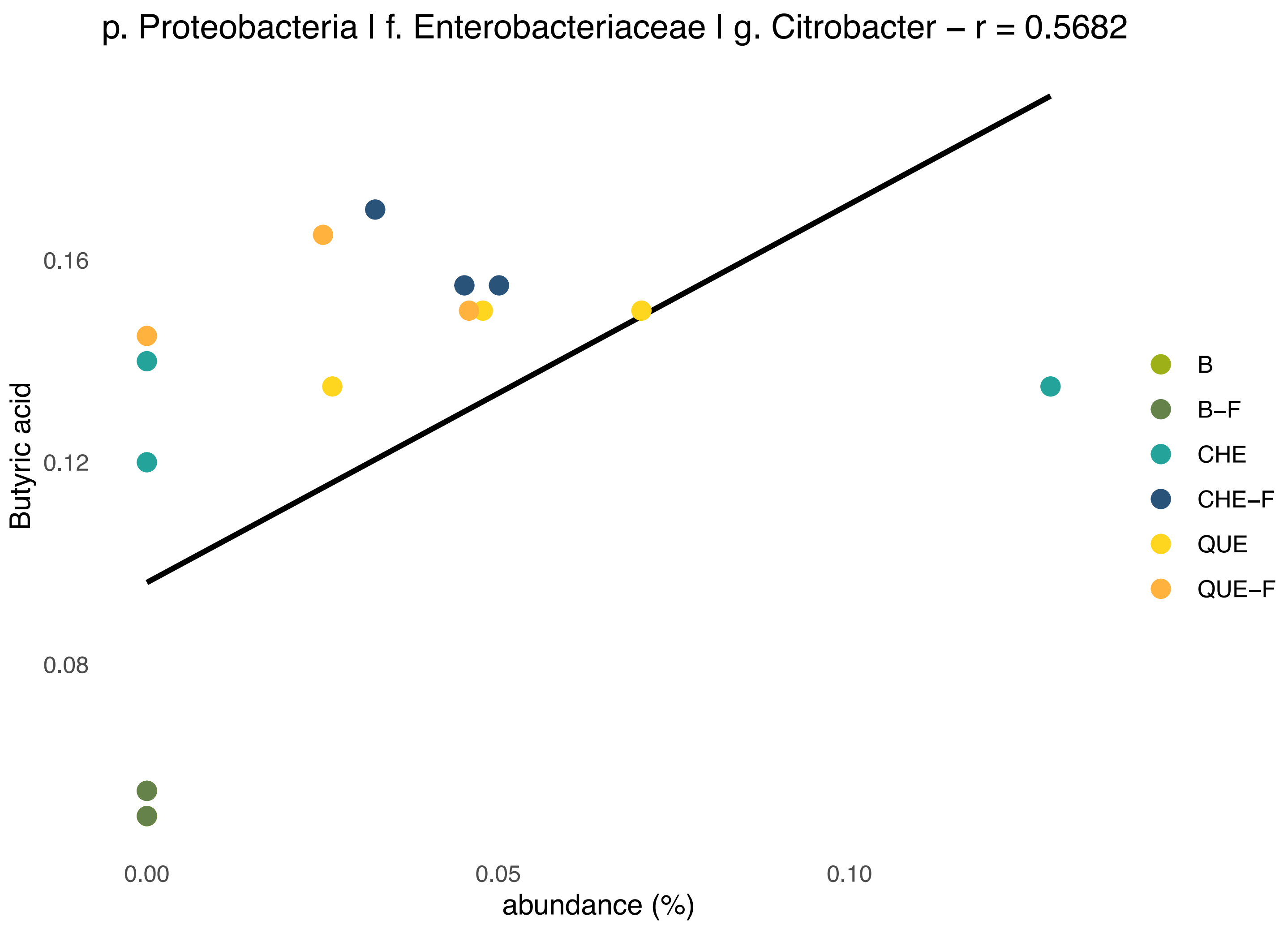

p. Proteobacteria I f. Enterobacteriaceae I g. Citrobacter – r = 0.5682
Butyric acid
0.16
0.12
0.08
0.00
0.05
0.10
abundance (%)
B
B–F
CHE
CHE–F
QUE
QUE–F

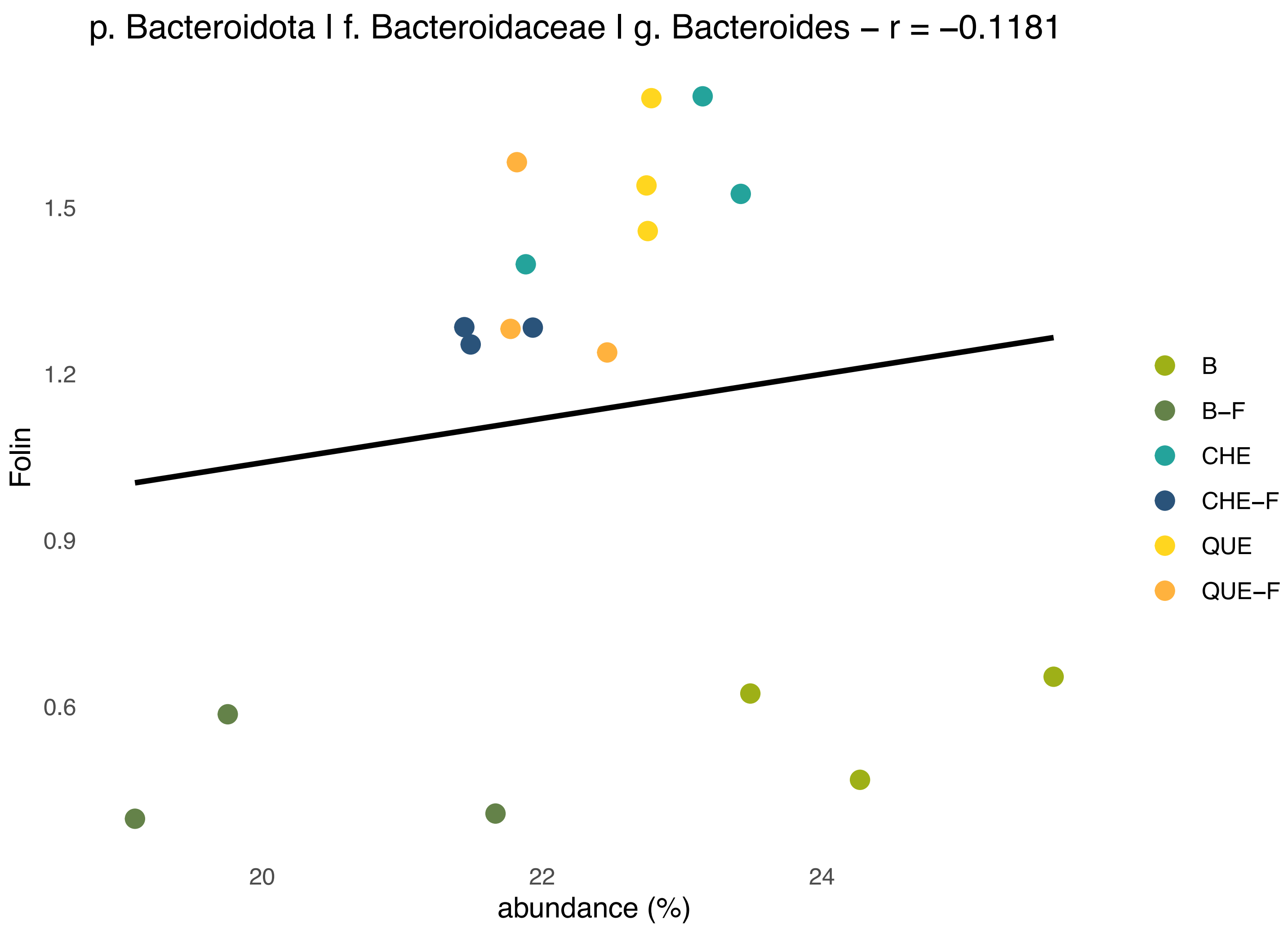

p. Bacteroidota l f. Bacteroidaceae l g. Bacteroides – r = –0.1181
Folin
1.5
1.2
0.9
0.6
20
22
24
abundance (%)
B
B–F
CHE
CHE–F
QUE
QUE–F

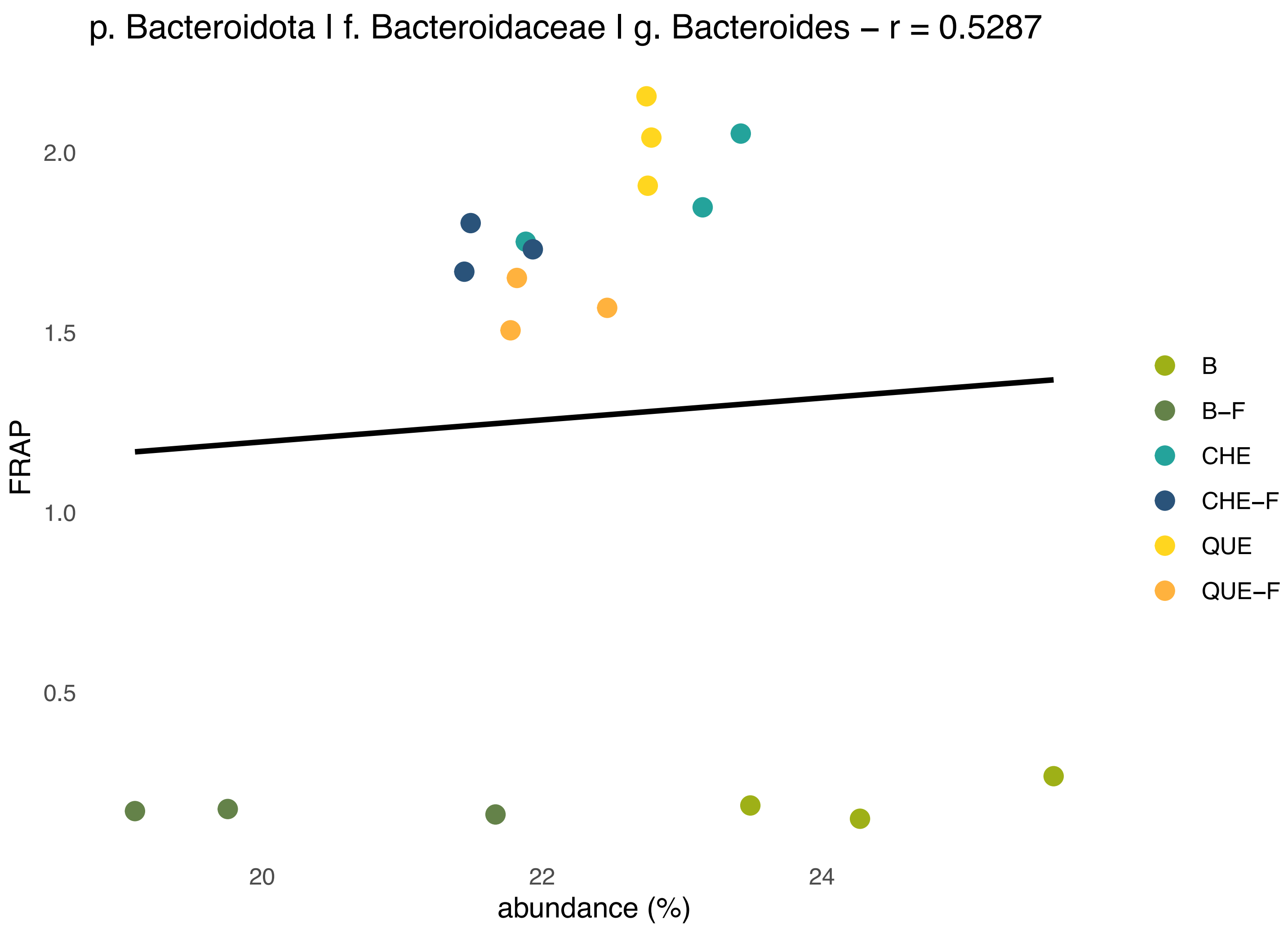

p. Bacteroidota I f. Bacteroidaceae I g. Bacteroides – r = 0.5287
FRAP
2.0
1.5
1.0
0.5
20
22
24
abundance (%)
B
B–F
CHE
CHE–F
QUE
QUE–F

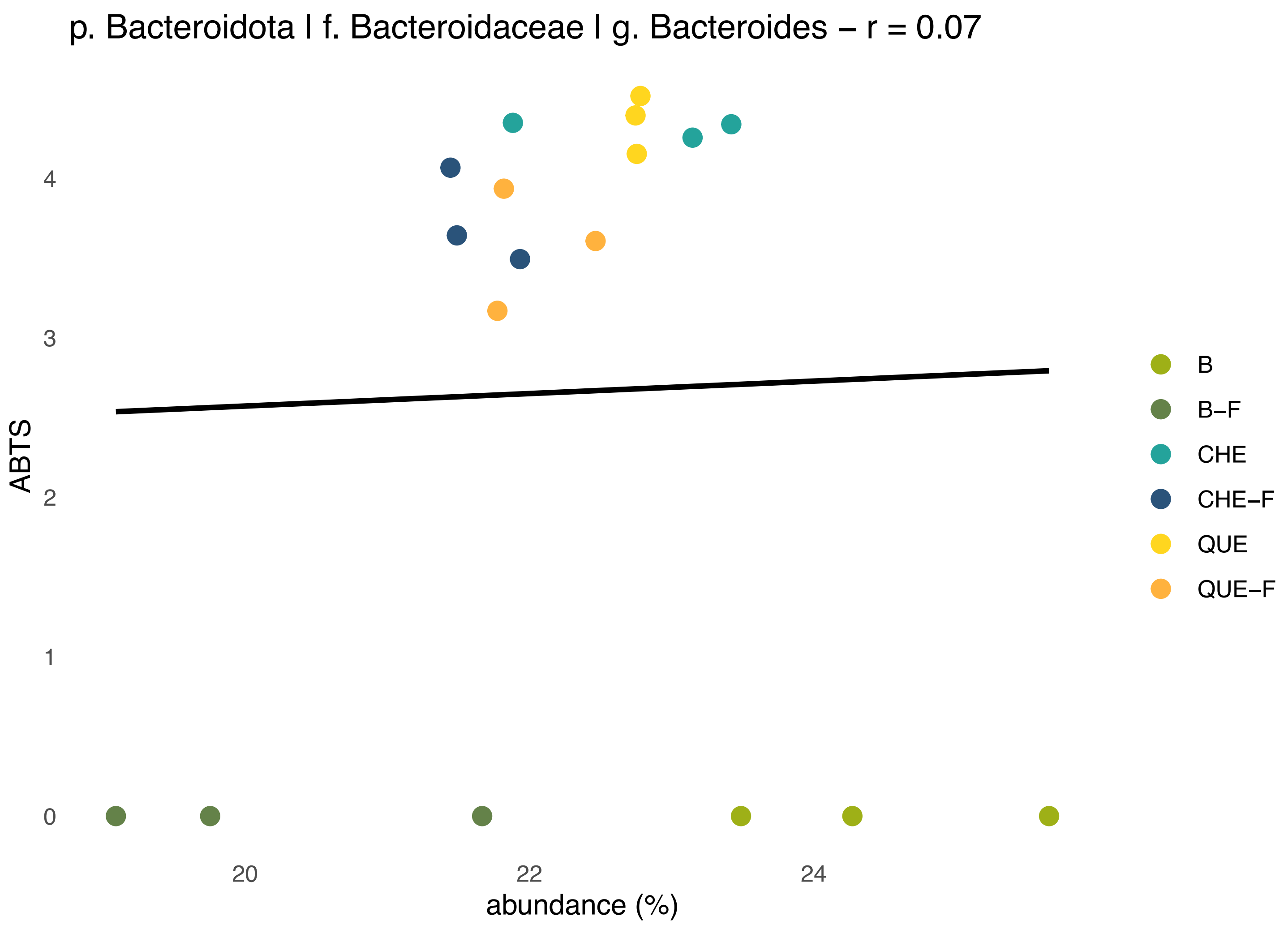
p. Bacteroidota | f. Bacteroidaceae | g. Bacteroides – r = 0.07
ABTS
4
3
2
1
0
20
22
24
abundance (%)
B
B–F
CHE
CHE–F
QUE
QUE–F

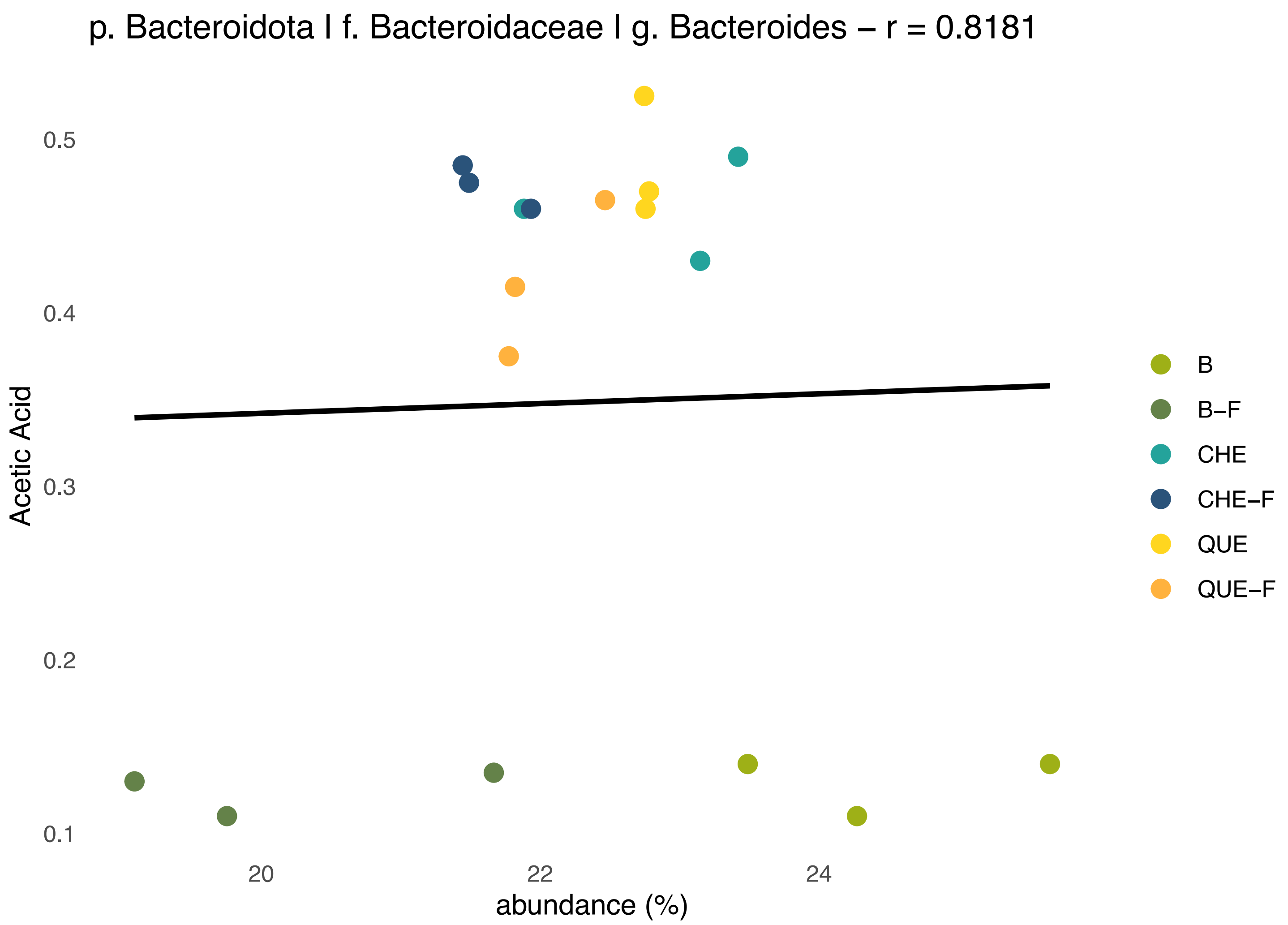
p. Bacteroidota I f. Bacteroidaceae I g. Bacteroides – r = 0.8181
Acetic Acid
0.5
0.4
0.3
0.2
0.1
20
22
24
abundance (%)
B
B–F
CHE
CHE–F
QUE
QUE–F

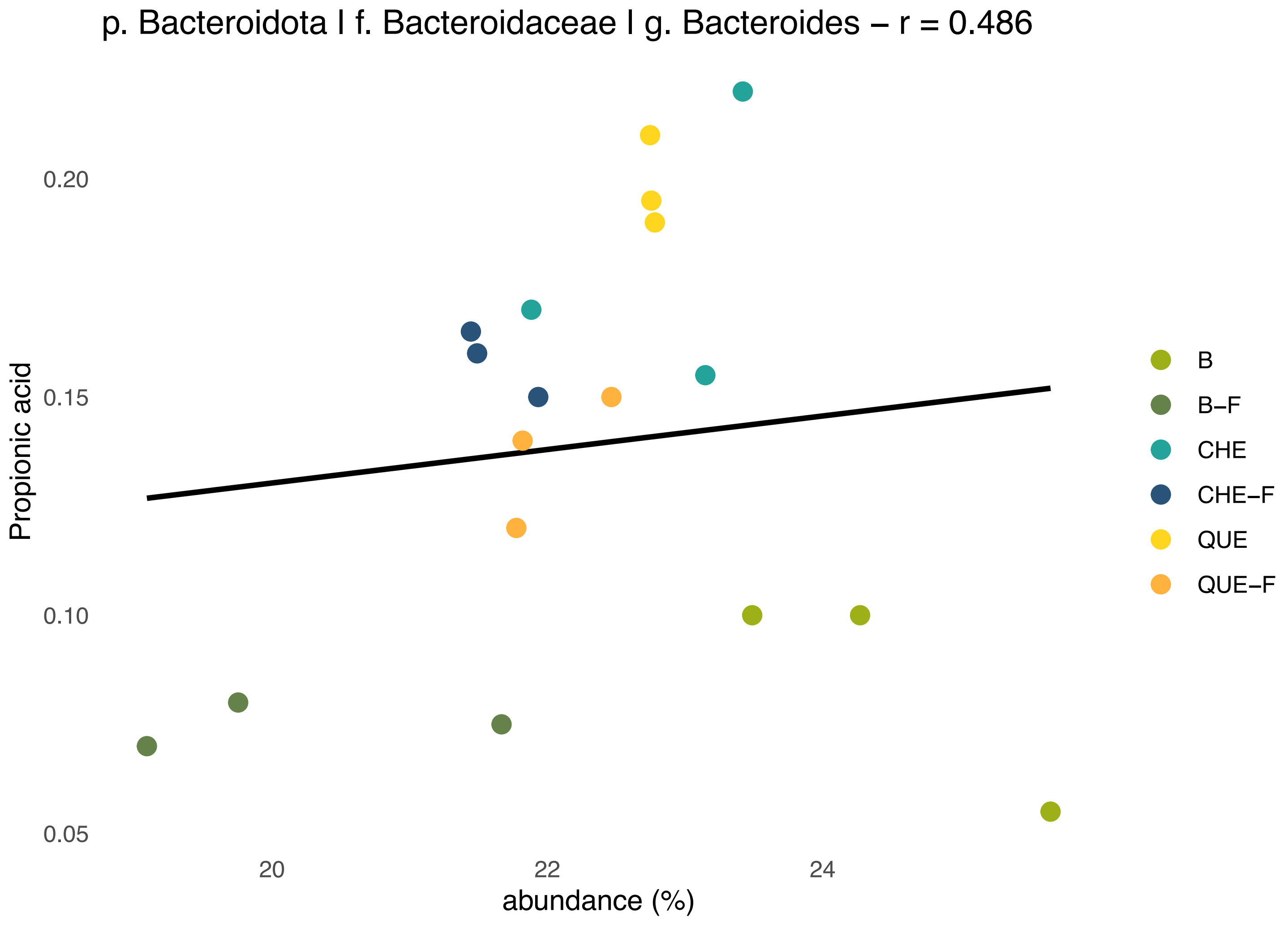

p. Bacteroidota | f. Bacteroidaceae | g. Bacteroides – r = 0.486
Propionic acid
0.20
0.15
0.10
0.05
20
22
24
abundance (%)
B
B–F
CHE
CHE–F
QUE
QUE–F

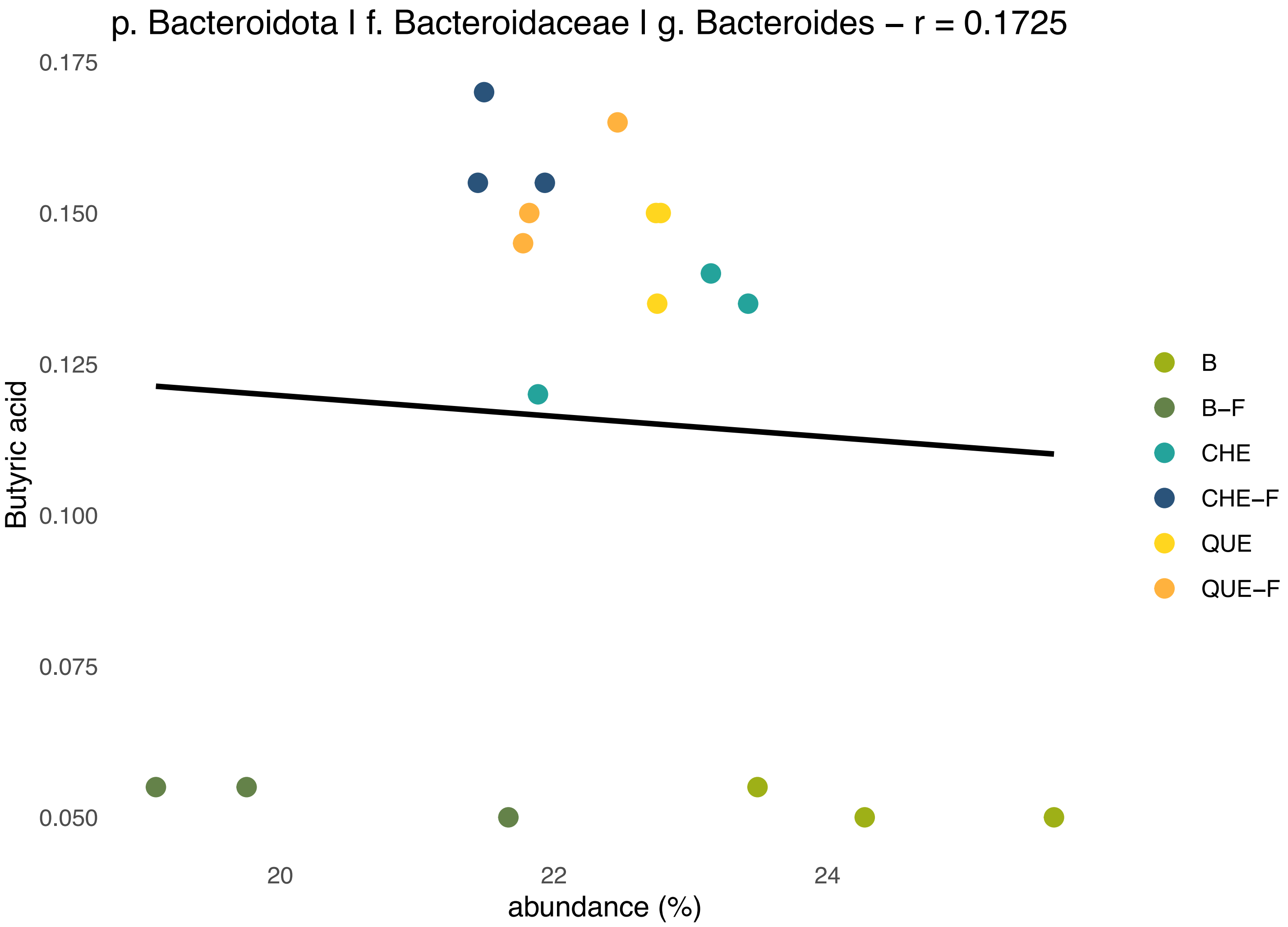

p. Bacteroidota I f. Bacteroidaceae I g. Bacteroides – r = 0.1725
0.175
0.150
0.125
0.100
0.075
0.050
Butyric acid
20
22
24
abundance (%)
B
B–F
CHE
CHE–F
QUE
QUE–F

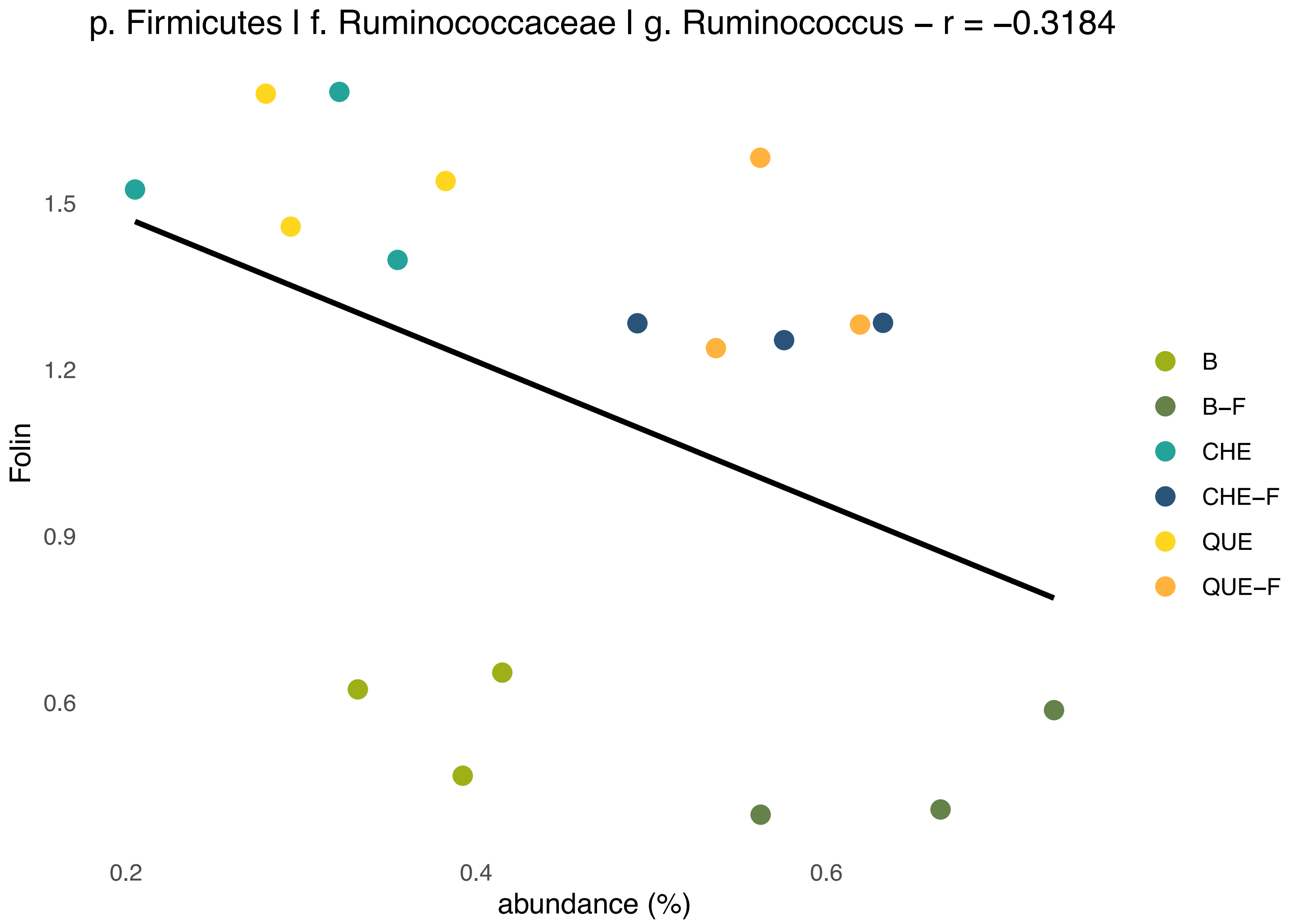
p. Firmicutes I f. Ruminococcaceae I g. Ruminococcus – r = –0.3184
1.5
1.2
0.9
0.6
Folin
0.2
0.4
0.6
abundance (%)
B
B–F
CHE
CHE–F
QUE
QUE–F

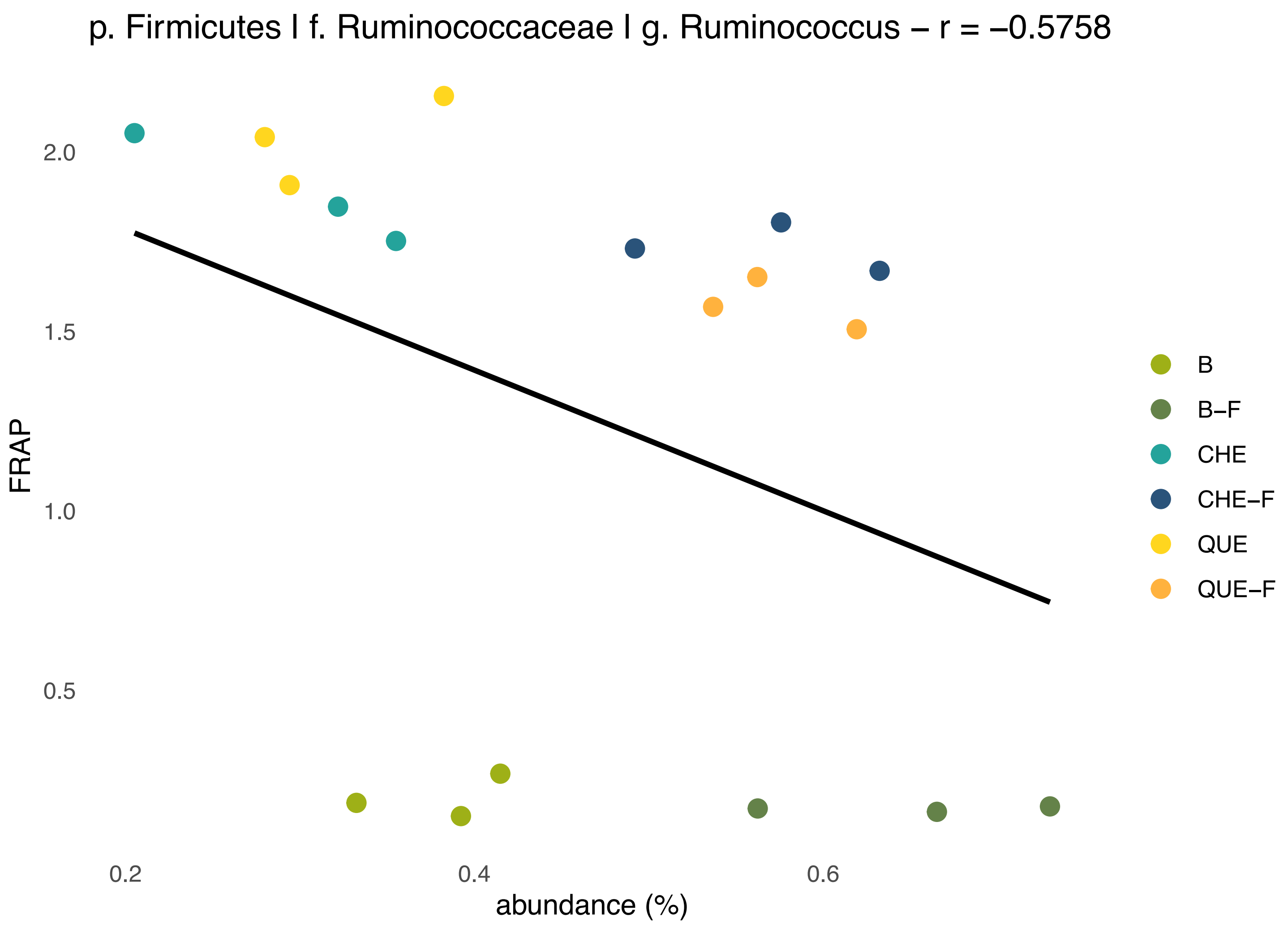

p. Firmicutes I f. Ruminococcaceae I g. Ruminococcus – r = –0.5758
FRAP
2.0
1.5
1.0
0.5
0.2
0.4
0.6
abundance (%)
B
B–F
CHE
CHE–F
QUE
QUE–F

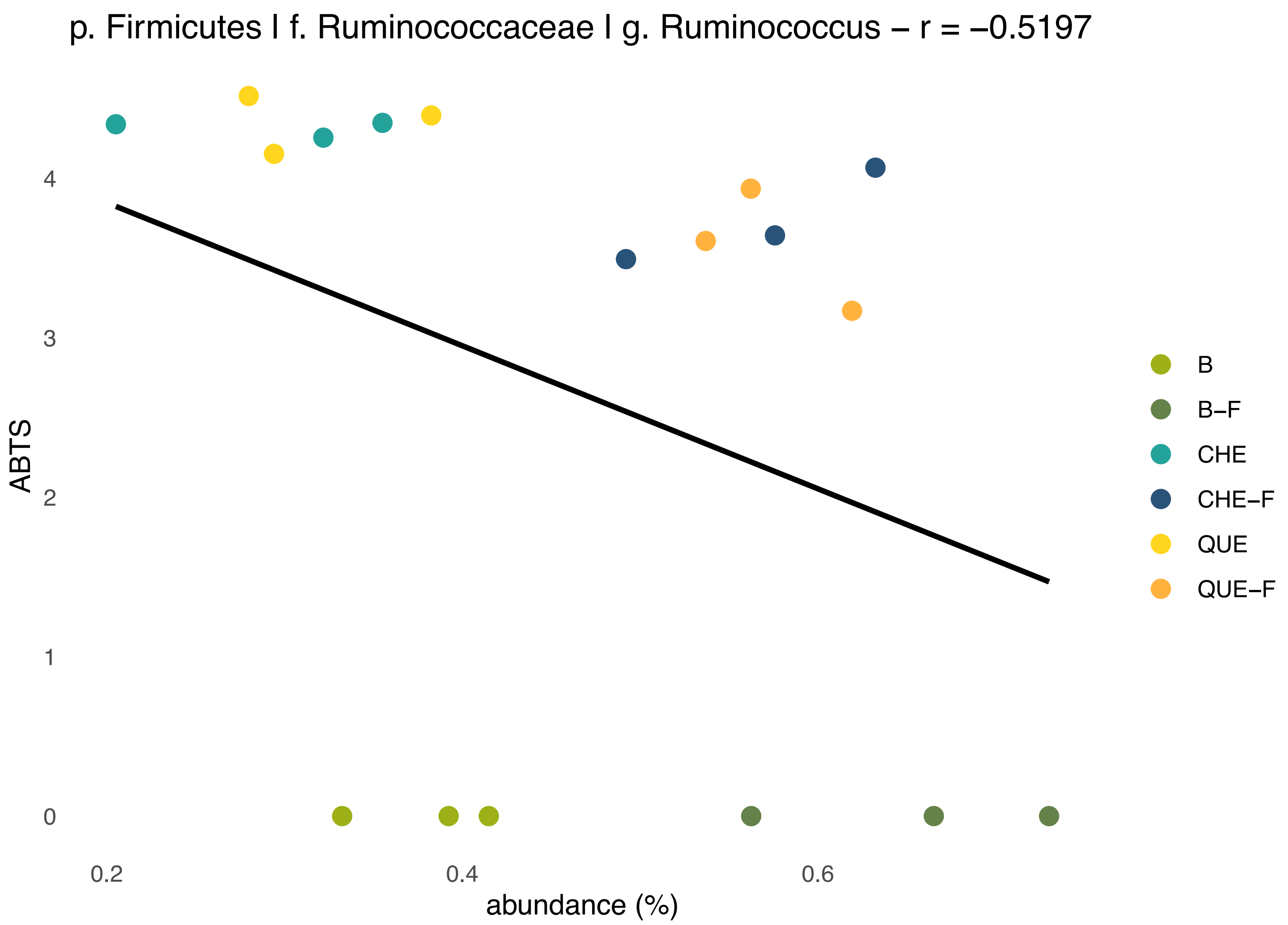
p. Firmicutes I f. Ruminococcaceae I g. Ruminococcus – r = −0.5197
ABTS
0
1
2
3
4
0.2
0.4
0.6
abundance (%)
B
B−F
CHE
CHE−F
QUE
QUE−F

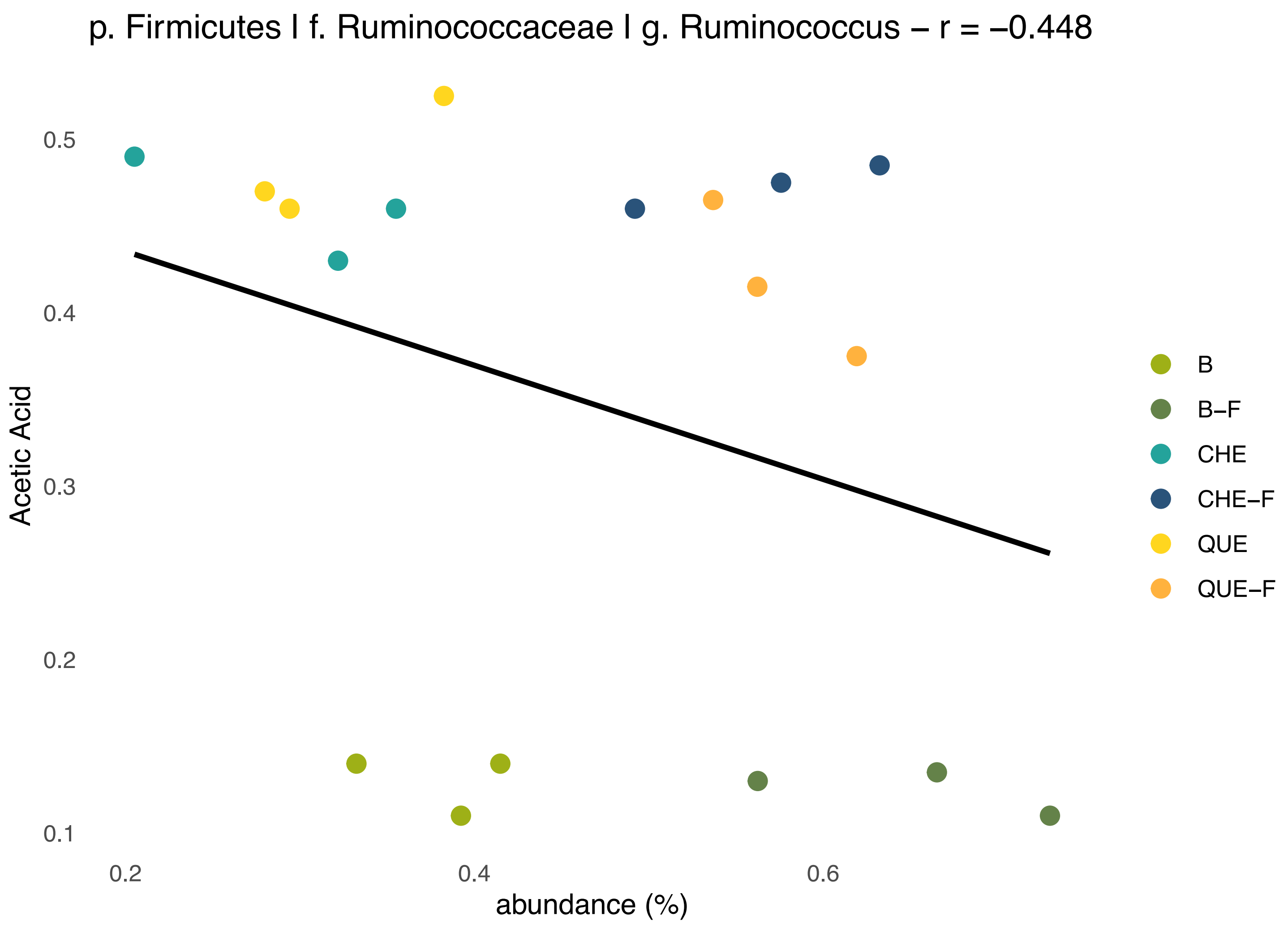
p. Firmicutes l f. Ruminococcaceae l g. Ruminococcus – r = –0.448
Acetic Acid
0.5
0.4
0.3
0.2
0.1
0.2
0.4
0.6
abundance (%)
B
B–F
CHE
CHE–F
QUE
QUE–F

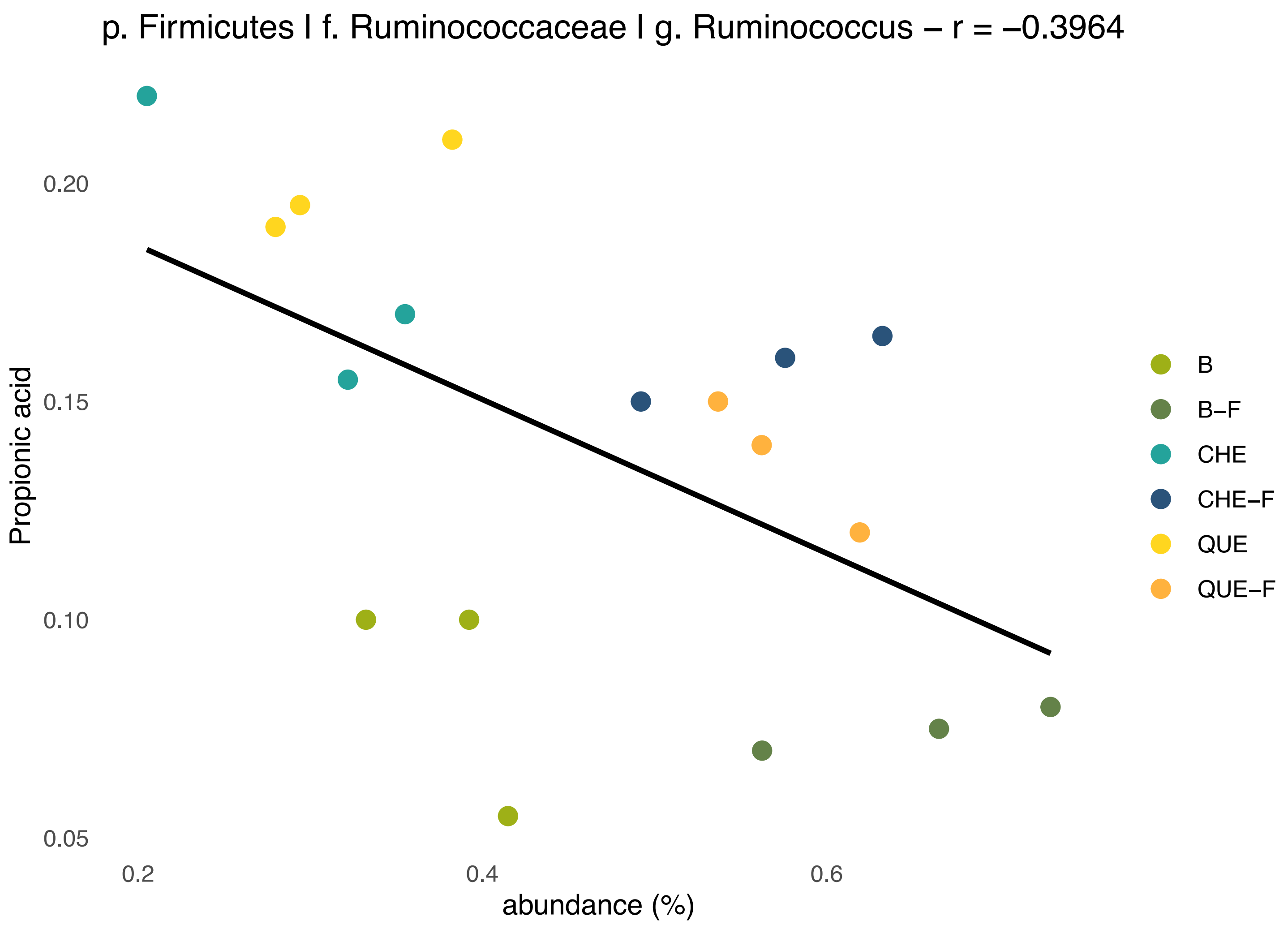
p. Firmicutes l f. Ruminococcaceae l g. Ruminococcus – r = –0.3964
Propionic acid
0.20
0.15
0.10
0.05
0.2
0.4
0.6
abundance (%)
B
B–F
CHE
CHE–F
QUE
QUE–F

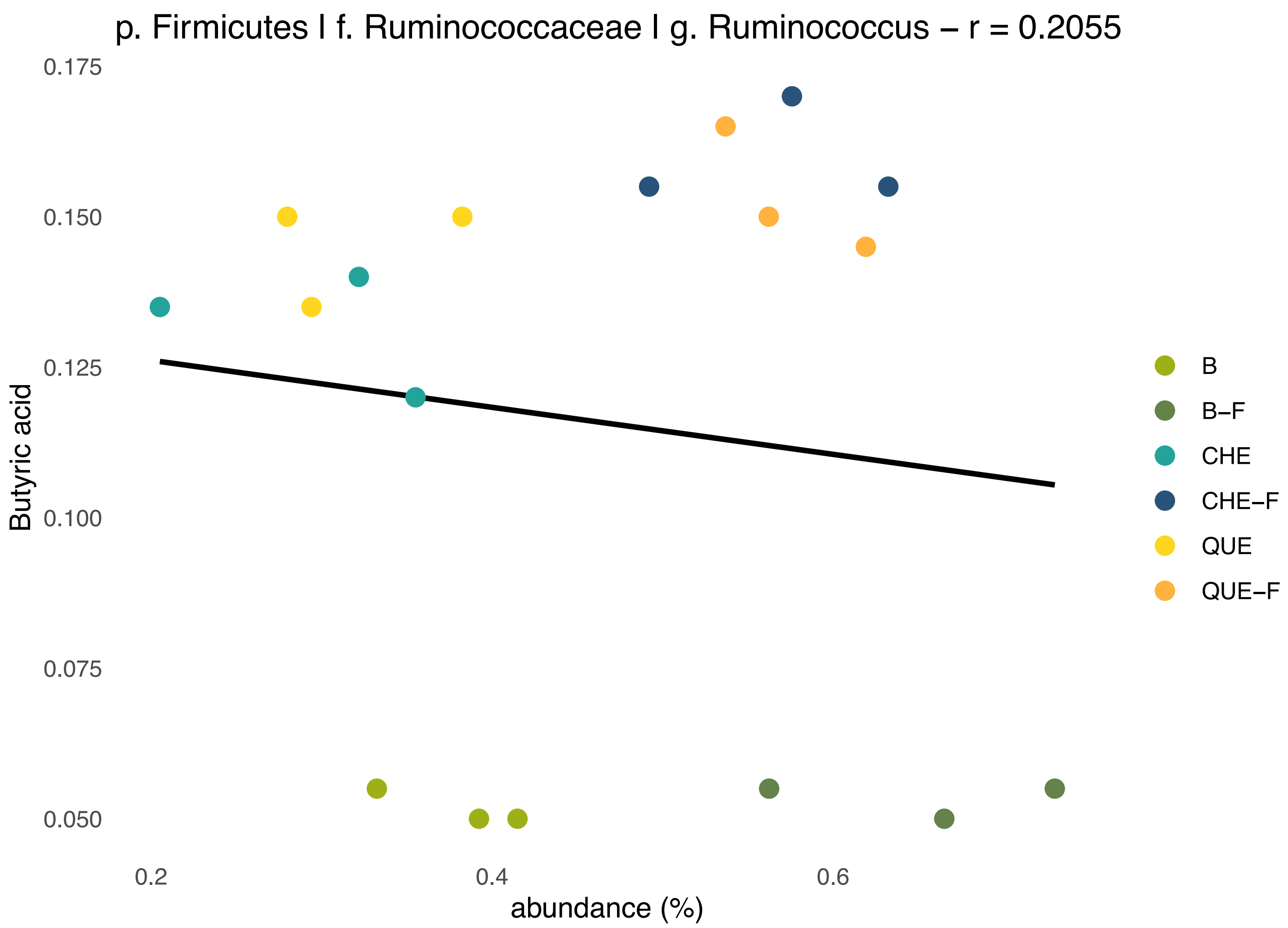
p. Firmicutes I f. Ruminococcaceae I g. Ruminococcus – r = 0.2055
0.175
0.150
0.125
0.100
0.075
0.050
Butyric acid
0.2
0.4
0.6
abundance (%)
B
B–F
CHE
CHE–F
QUE
QUE–F

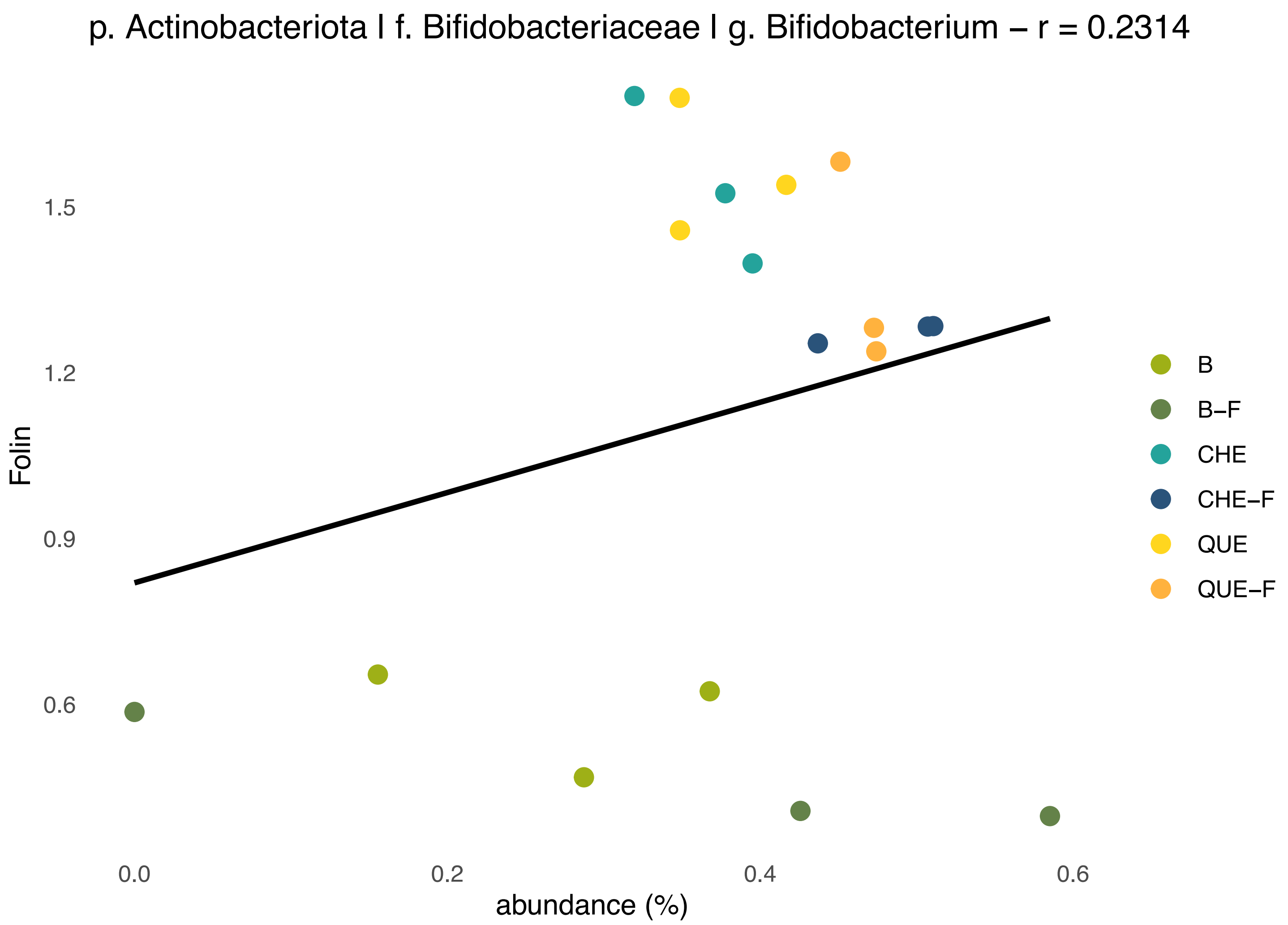
p. Actinobacteriota l f. Bifidobacteriaceae l g. Bifidobacterium – r = 0.2314
Folin
1.5
1.2
0.9
0.6
0.0
0.2
0.4
0.6
abundance (%)
B
B–F
CHE
CHE–F
QUE
QUE–F

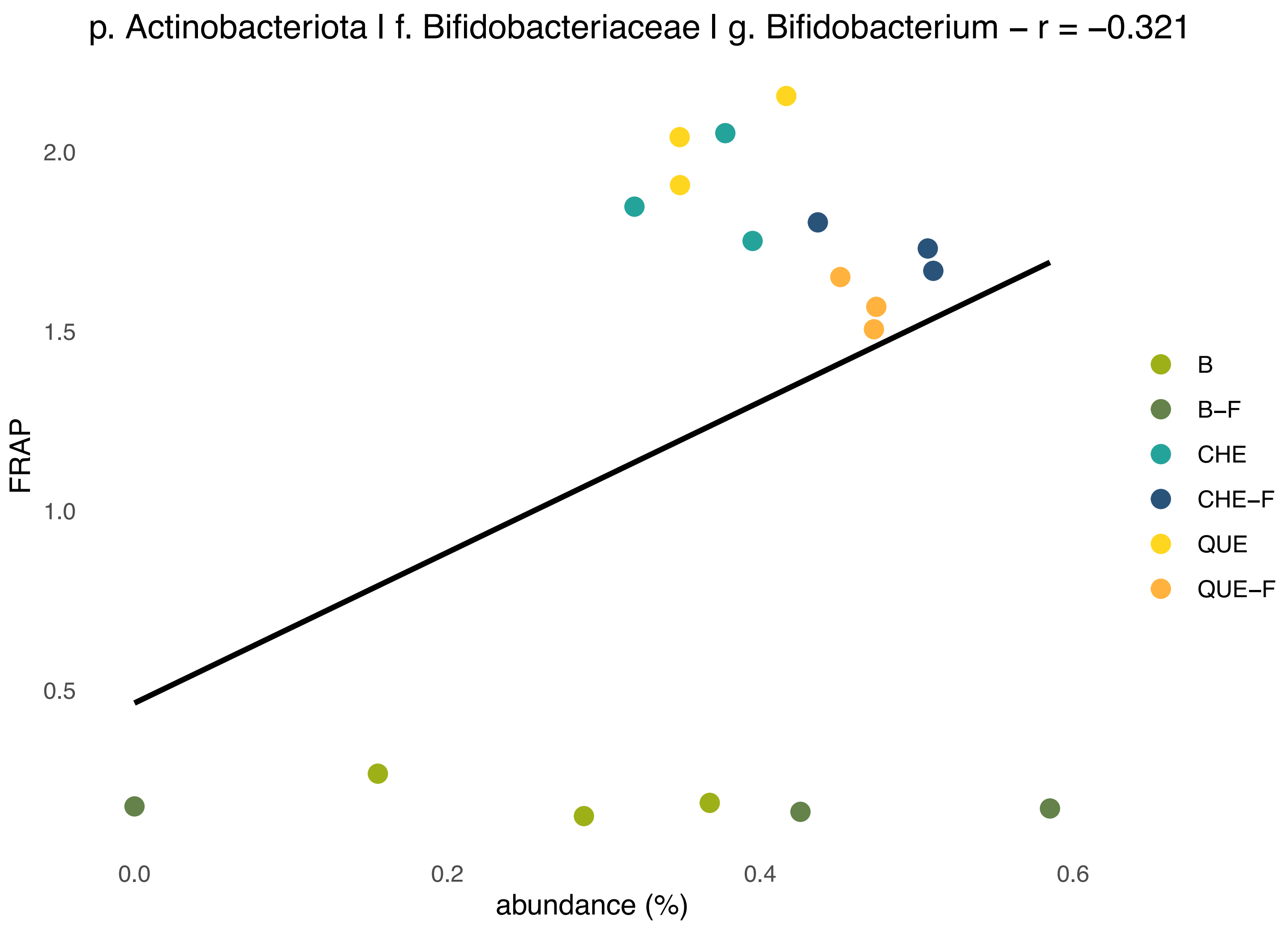
p. Actinobacteriota l f. Bifidobacteriaceae l g. Bifidobacterium − r = −0.321
FRAP
2.0
1.5
1.0
0.5
0.0
0.2
0.4
0.6
abundance (%)
B
B−F
CHE
CHE−F
QUE
QUE−F

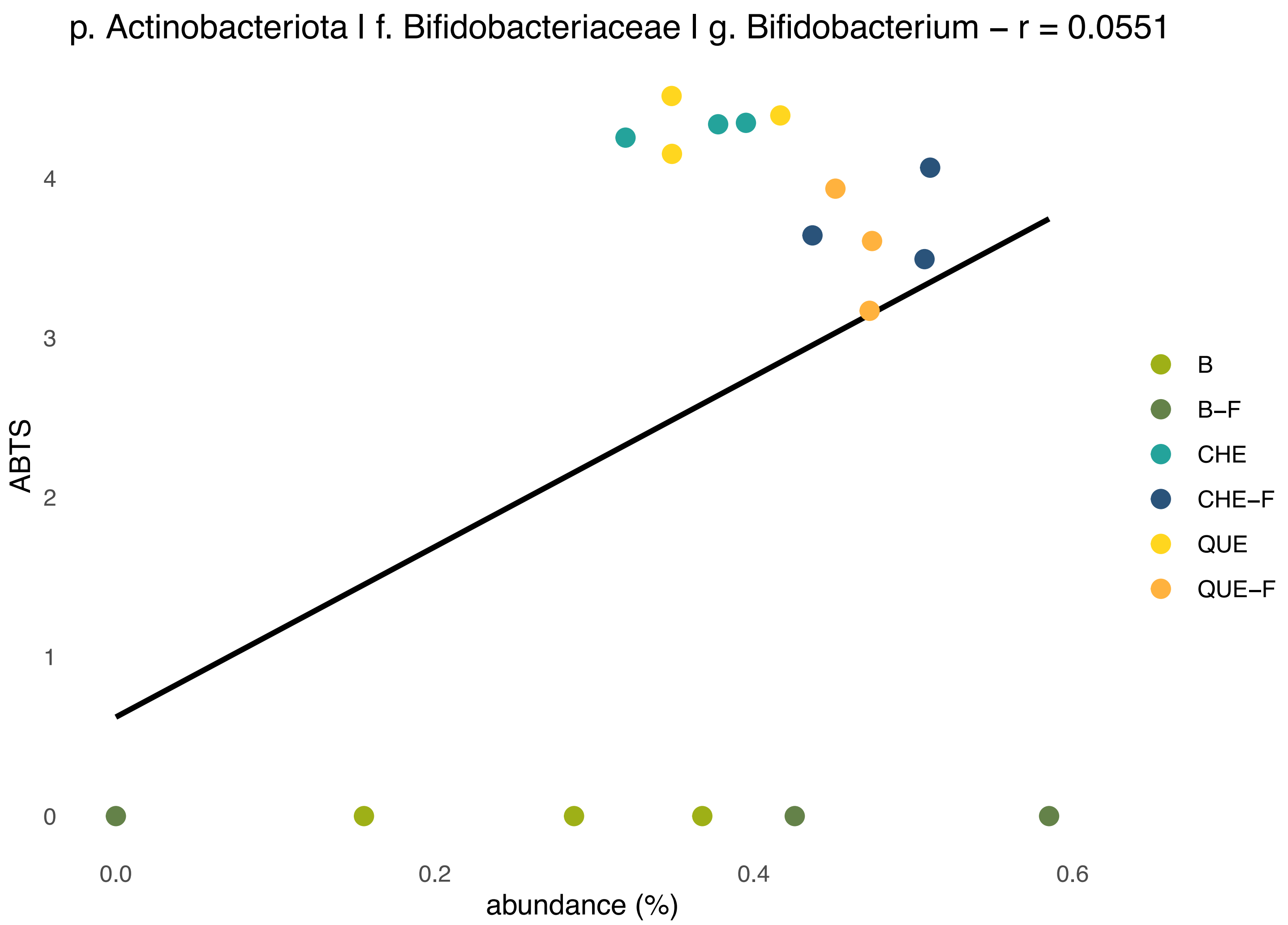
p. Actinobacteriota I f. Bifidobacteriaceae I g. Bifidobacterium – r = 0.0551
ABTS
4
3
2
1
0
0.0
0.2
0.4
0.6
abundance (%)
B
B–F
CHE
CHE–F
QUE
QUE–F

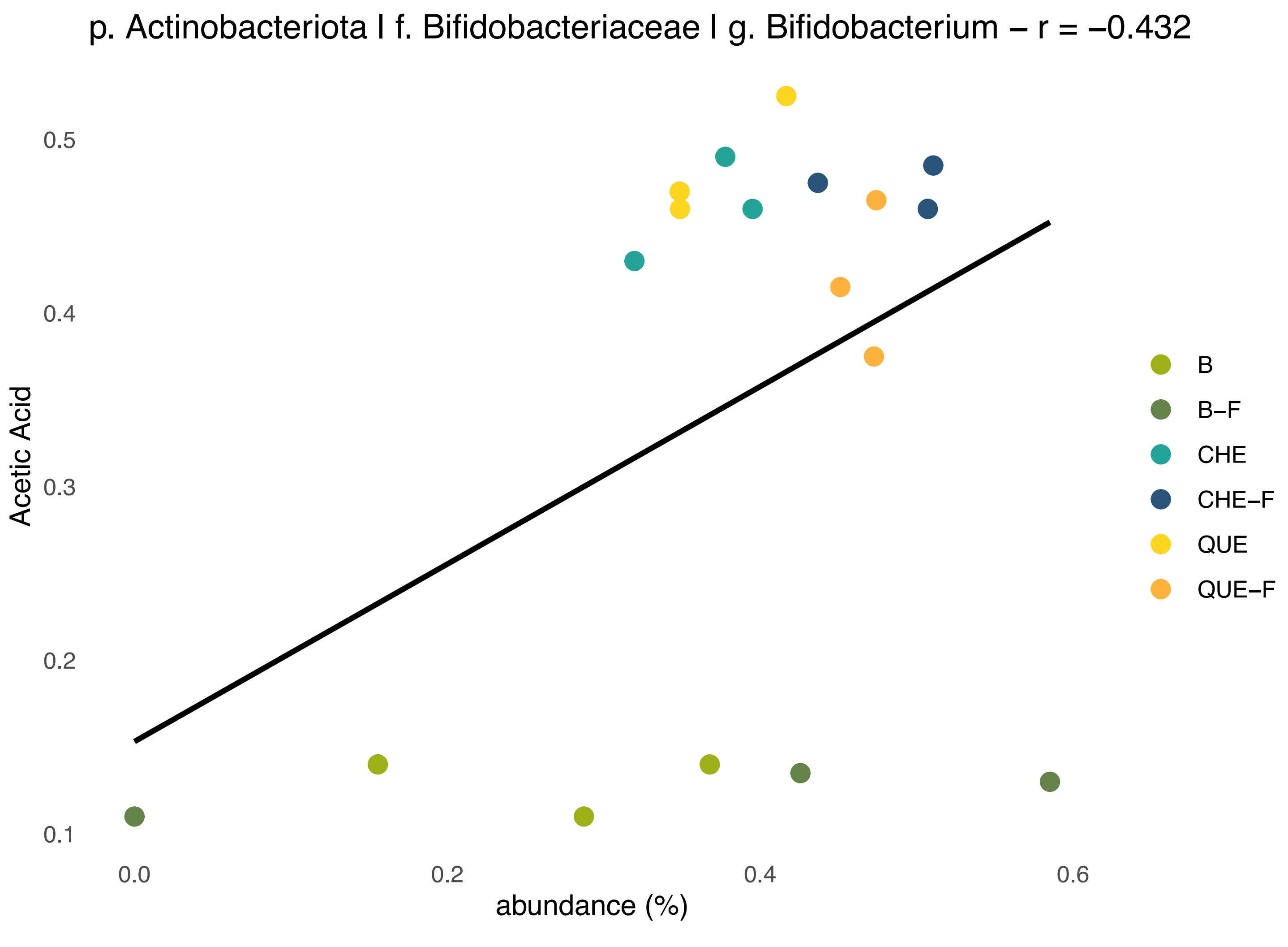

p. Actinobacteriota l f. Bifidobacteriaceae l g. Bifidobacterium – r = –0.432
Acetic Acid
0.5
0.4
0.3
0.2
0.1
abundance (%)
0.0
0.2
0.4
0.6
B
B–F
CHE
CHE–F
QUE
QUE–F

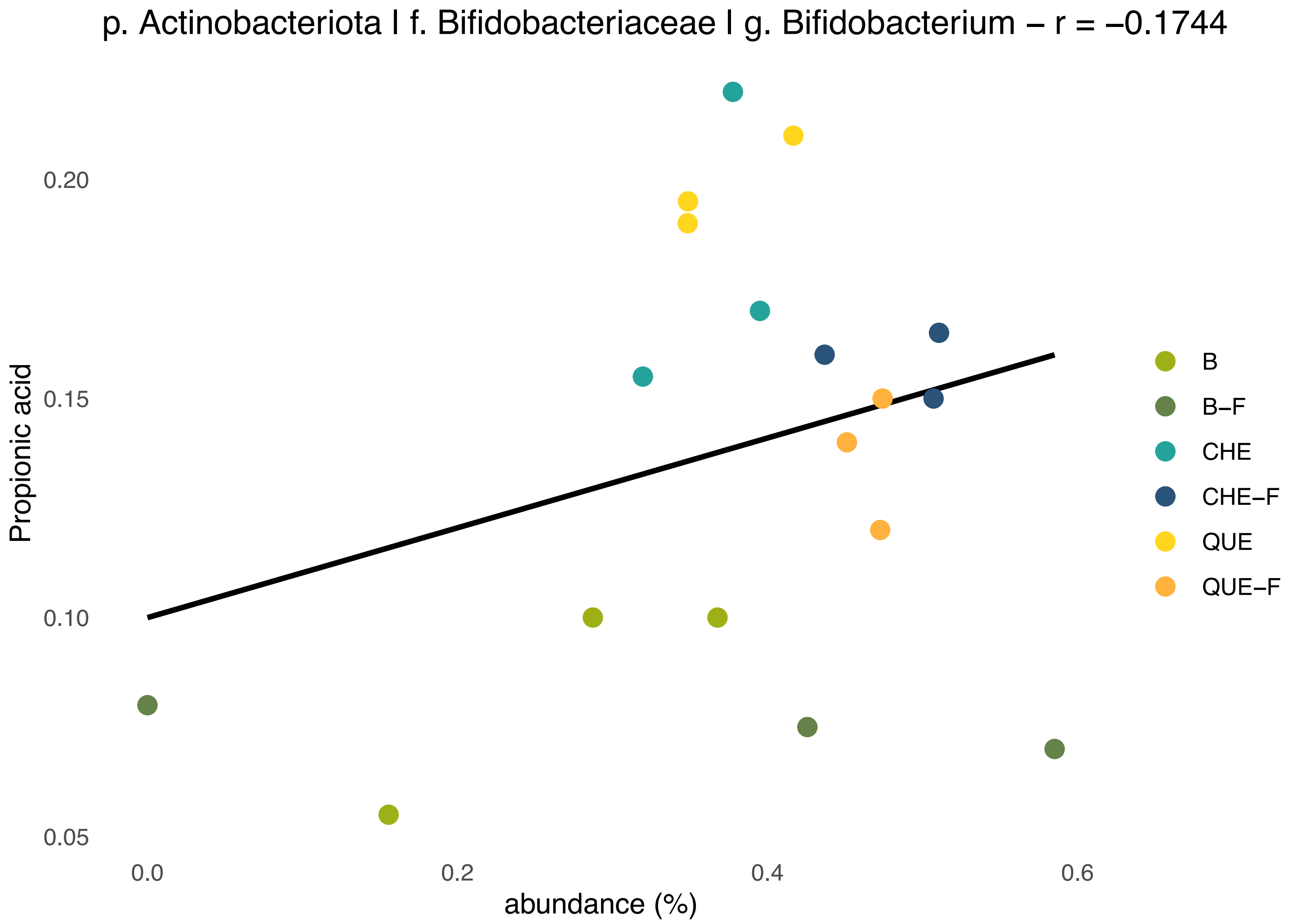

p. Actinobacteriota I f. Bifidobacteriaceae I g. Bifidobacterium – r = –0.1744
Propionic acid
0.20
0.15
0.10
0.05
0.0
0.2
0.4
0.6
abundance (%)
B
B–F
CHE
CHE–F
QUE
QUE–F

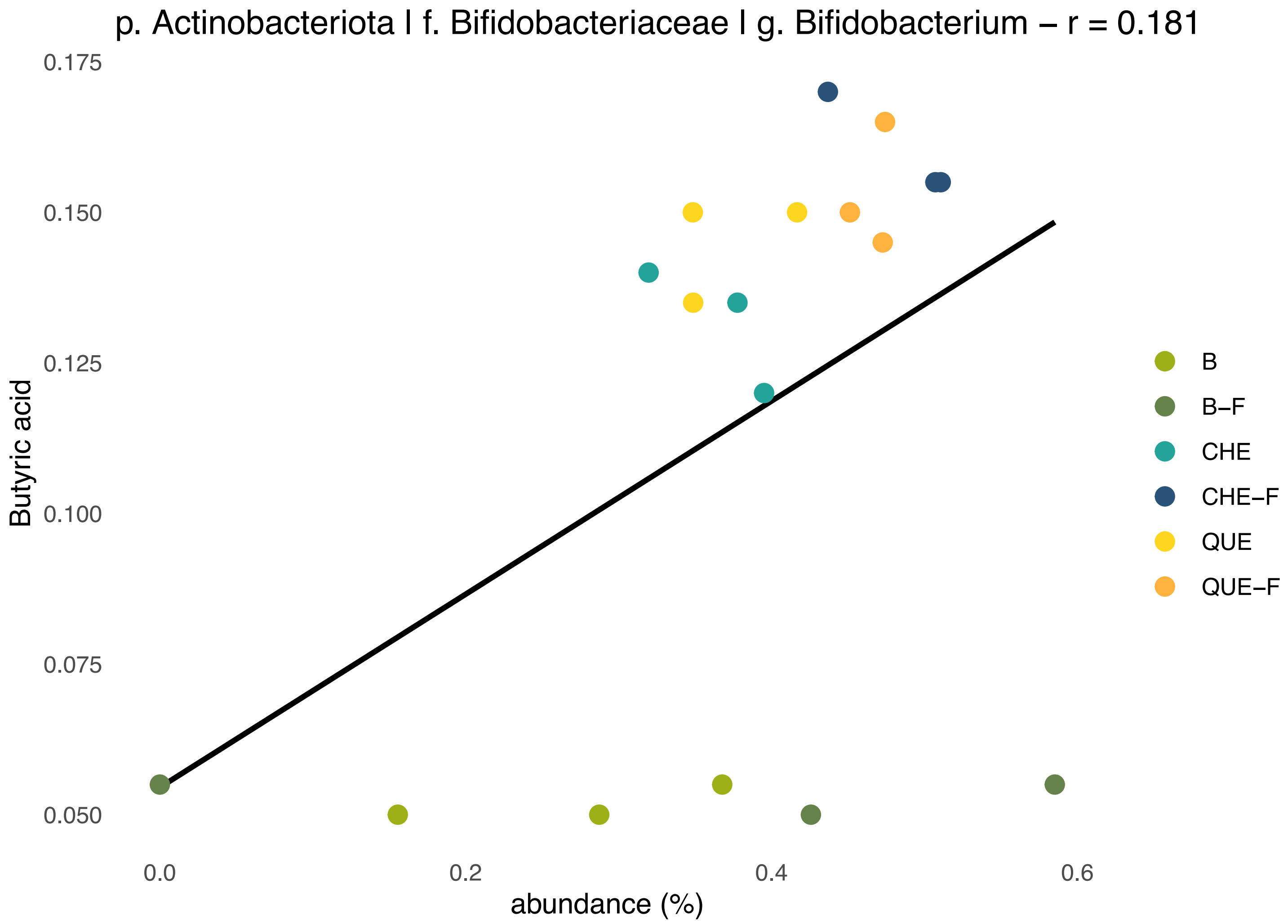

p. Actinobacteriota l f. Bifidobacteriaceae l g. Bifidobacterium – r = 0.181
0.175
0.150
0.125
0.100
0.075
0.050
Butyric acid
0.0
0.2
0.4
0.6
abundance (%)
B
B–F
CHE
CHE–F
QUE
QUE–F

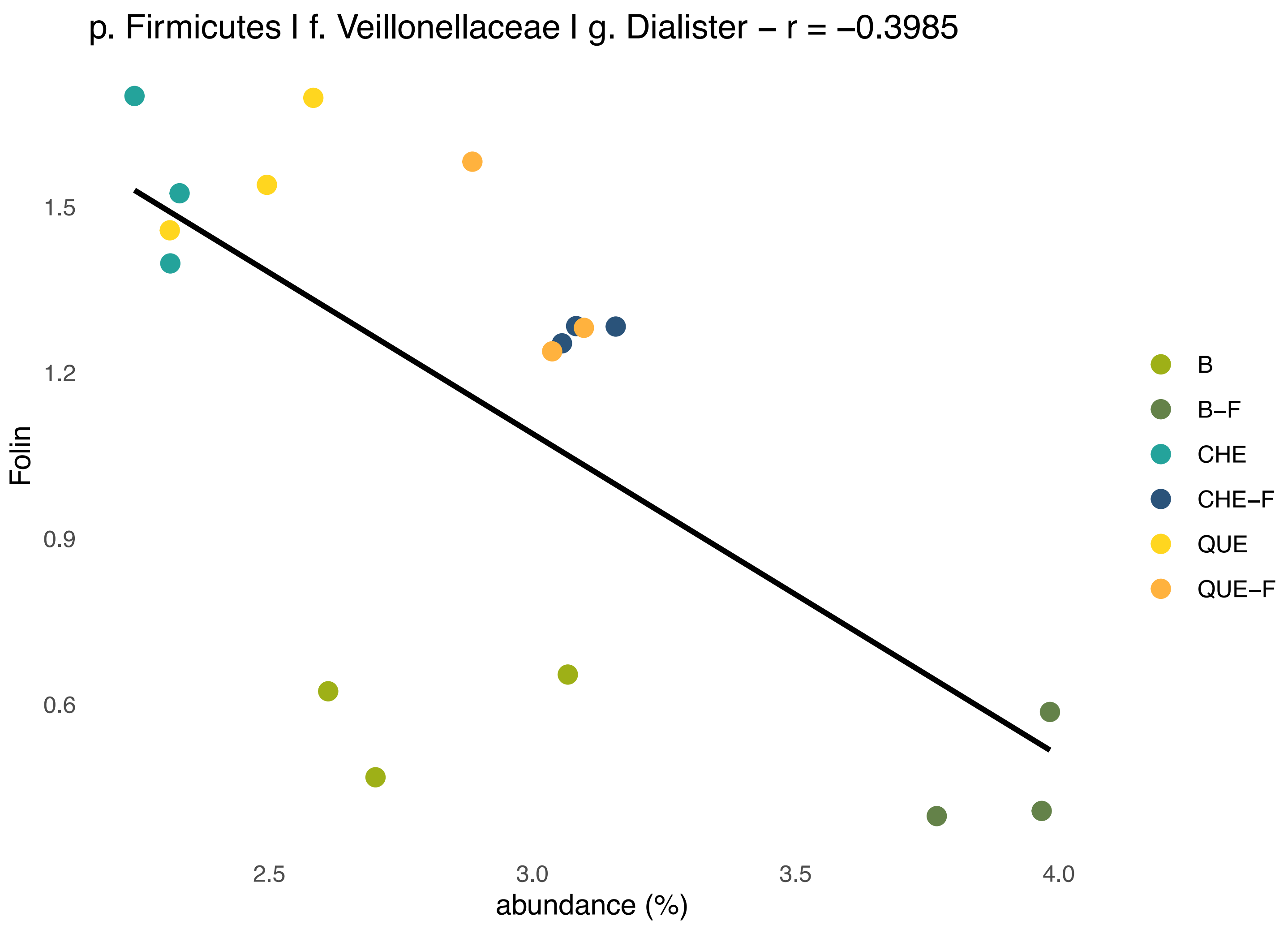
p. Firmicutes I f. Veillonellaceae I g. Dialister – r = –0.3985
1.5
1.2
0.9
0.6
Folin
2.5
3.0
3.5
4.0
abundance (%)
B
B–F
CHE
CHE–F
QUE
QUE–F

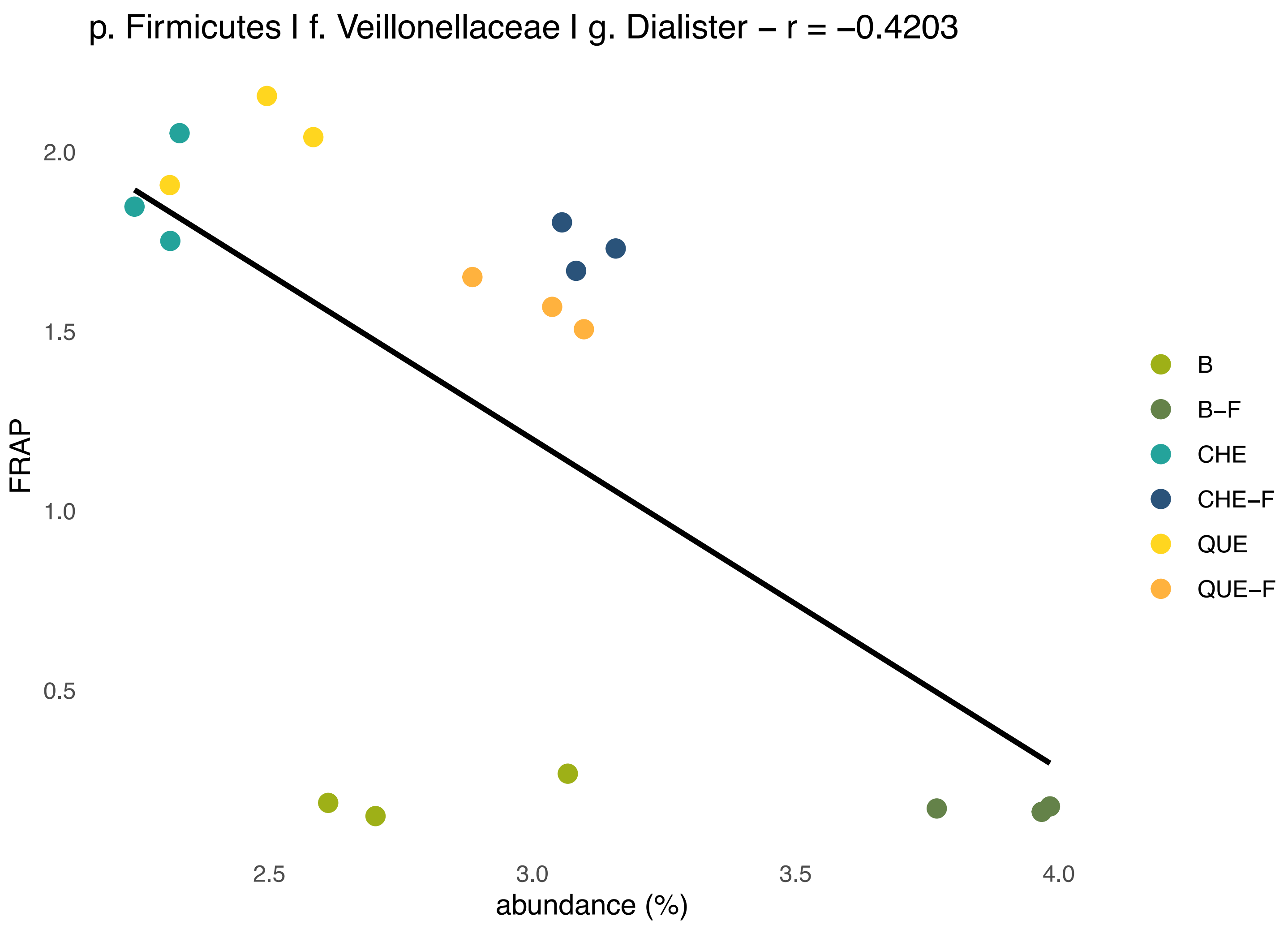

p. Firmicutes l f. Veillonellaceae l g. Dialister – r = –0.4203
FRAP
2.0
1.5
1.0
0.5
2.5
3.0
3.5
4.0
abundance (%)
B
B–F
CHE
CHE–F
QUE
QUE–F

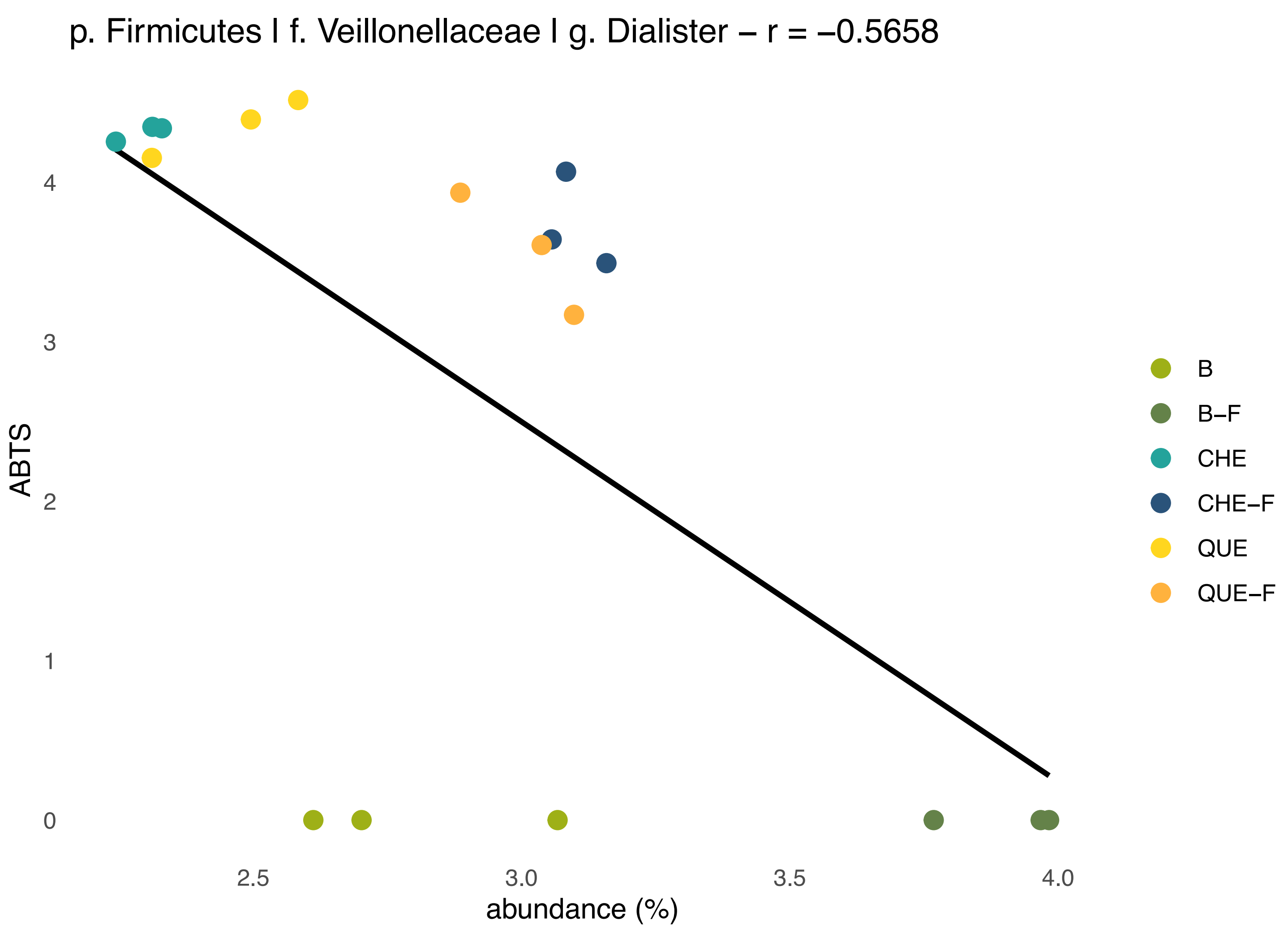

p. Firmicutes I f. Veillonellaceae I g. Dialister – r = –0.5658
ABTS
0
1
2
3
4
2.5
3.0
3.5
4.0
abundance (%)
B
B–F
CHE
CHE–F
QUE
QUE–F

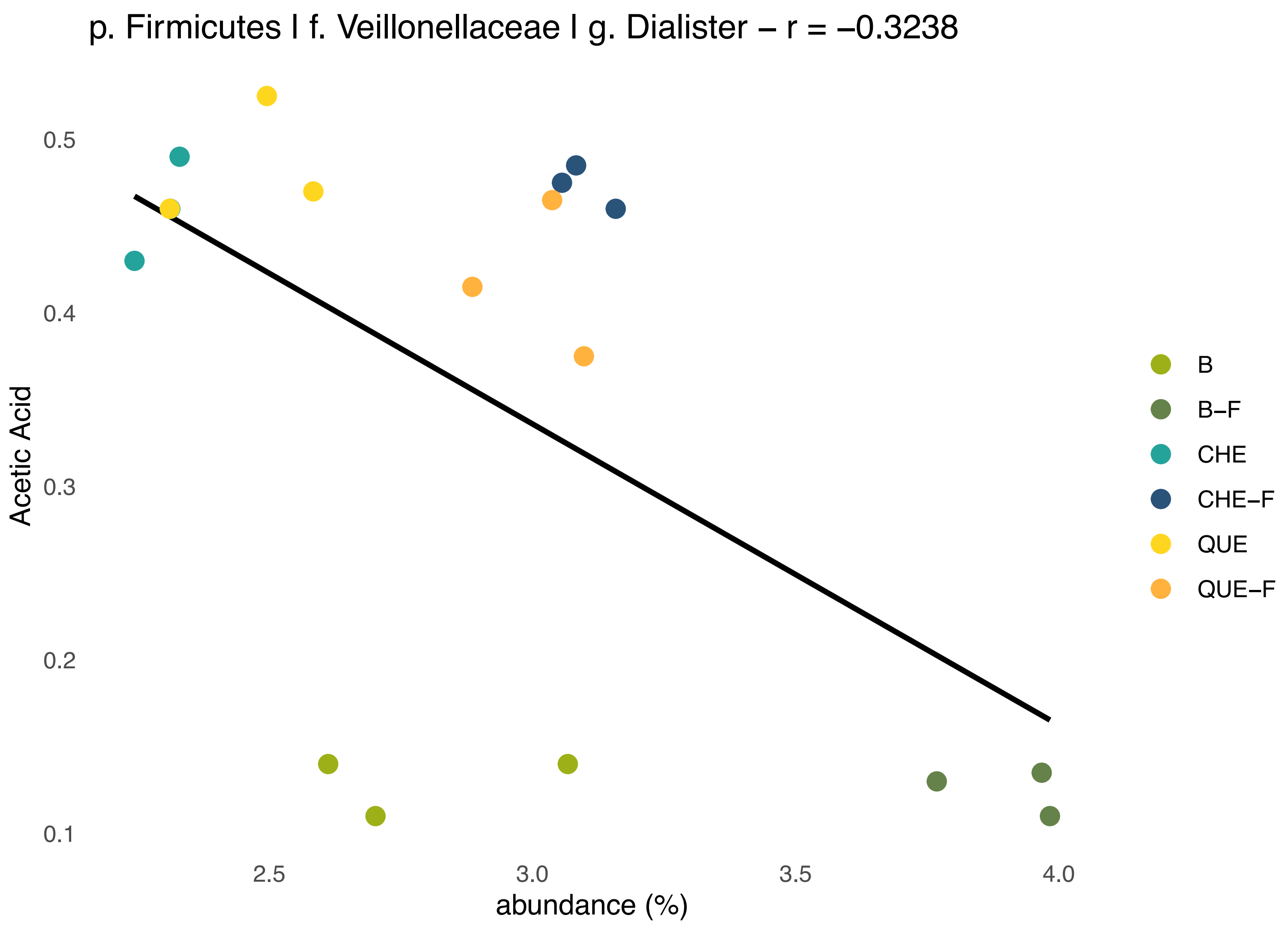

p. Firmicutes | f. Veillonellaceae | g. Dialister – r = –0.3238
Acetic Acid
0.5
0.4
0.3
0.2
0.1
2.5
3.0
3.5
4.0
abundance (%)
B
B–F
CHE
CHE–F
QUE
QUE–F

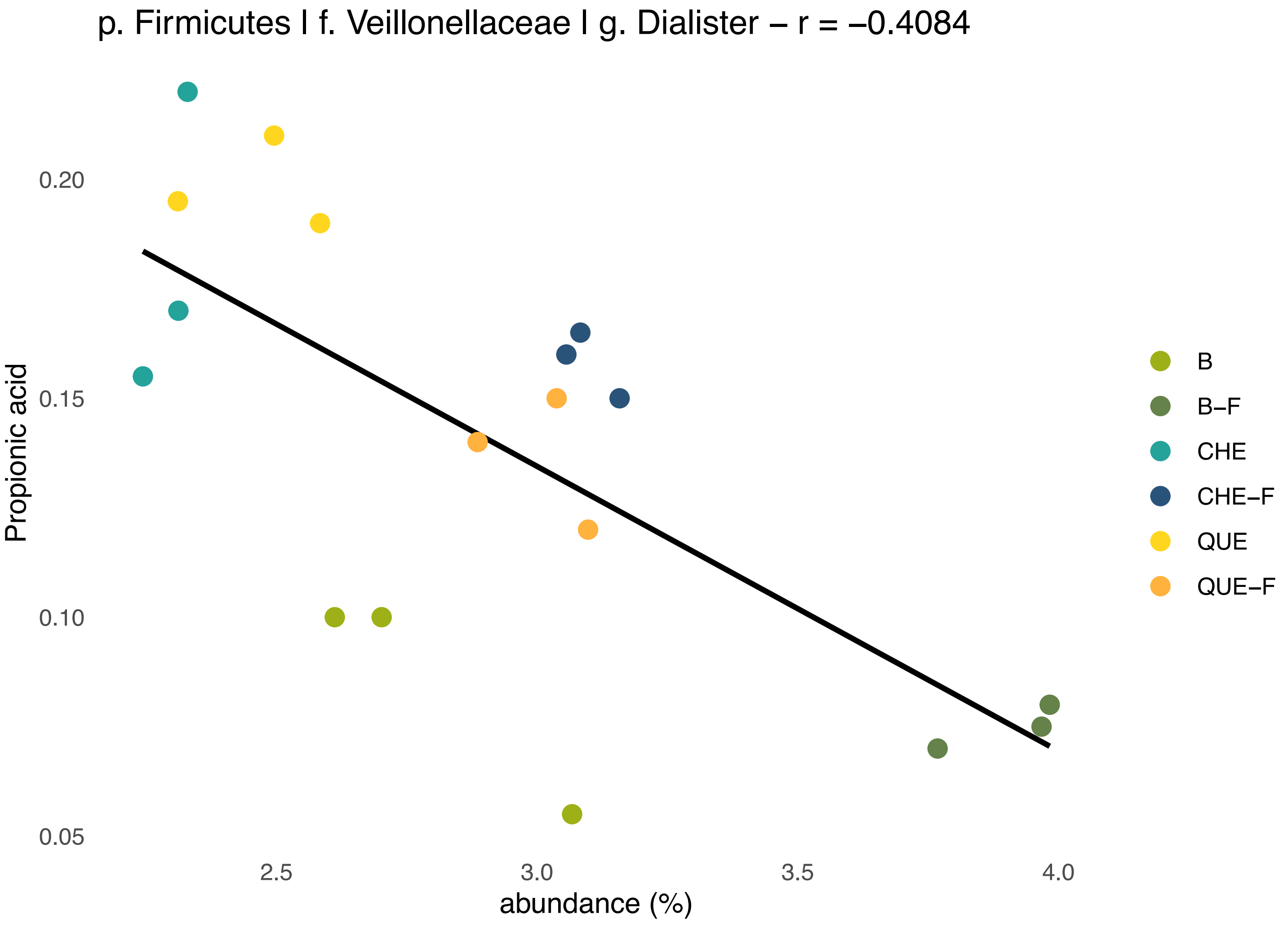
p. Firmicutes I f. Veillonellaceae I g. Dialister – r = –0.4084
Propionic acid
0.20
0.15
0.10
0.05
2.5
3.0
3.5
4.0
abundance (%)
B
B–F
CHE
CHE–F
QUE
QUE–F

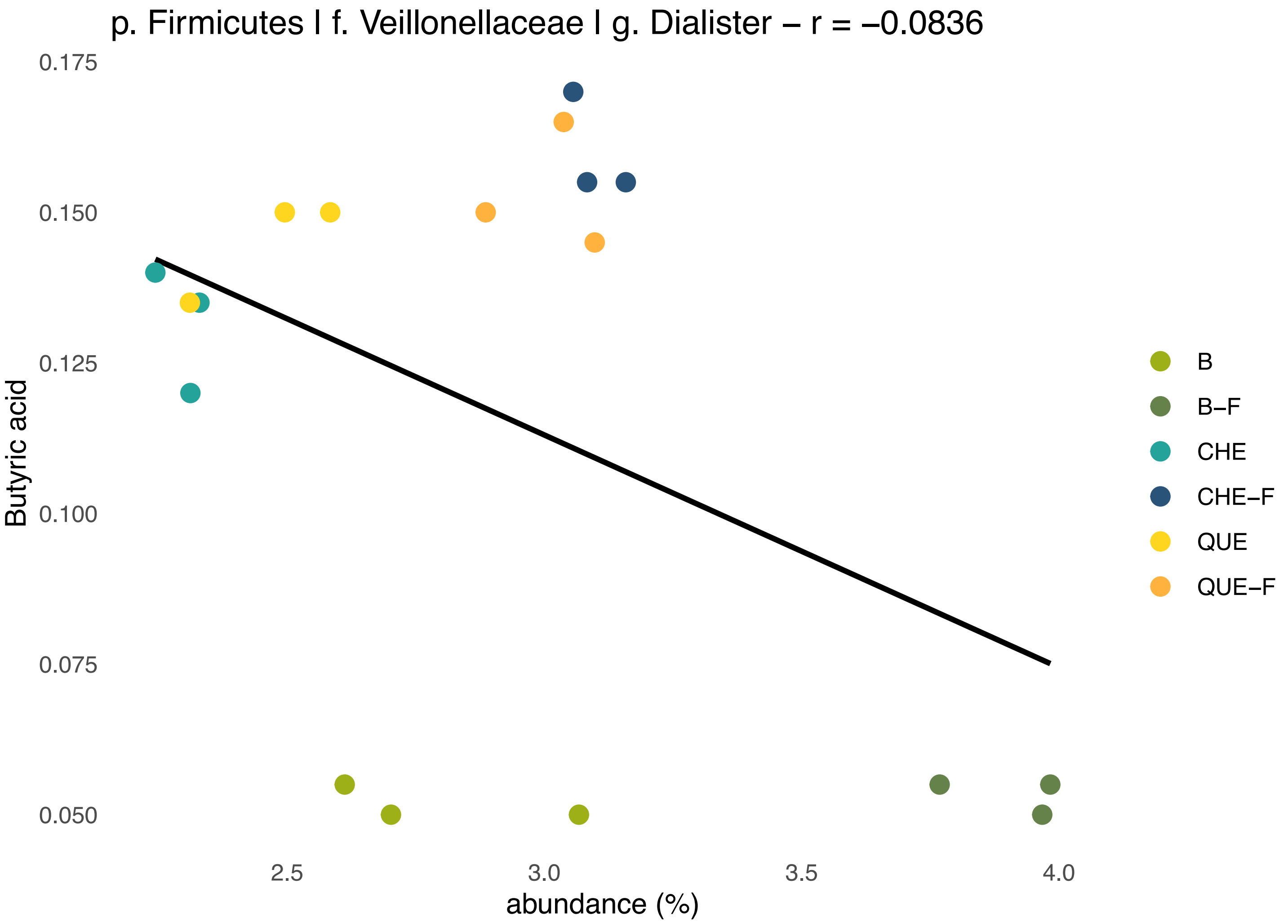

p. Firmicutes I f. Veillonellaceae I g. Dialister – r = –0.0836
0.175
0.150
0.125
0.100
0.075
0.050
Butyric acid
2.5
3.0
3.5
4.0
abundance (%)
B
B–F
CHE
CHE–F
QUE
QUE–F

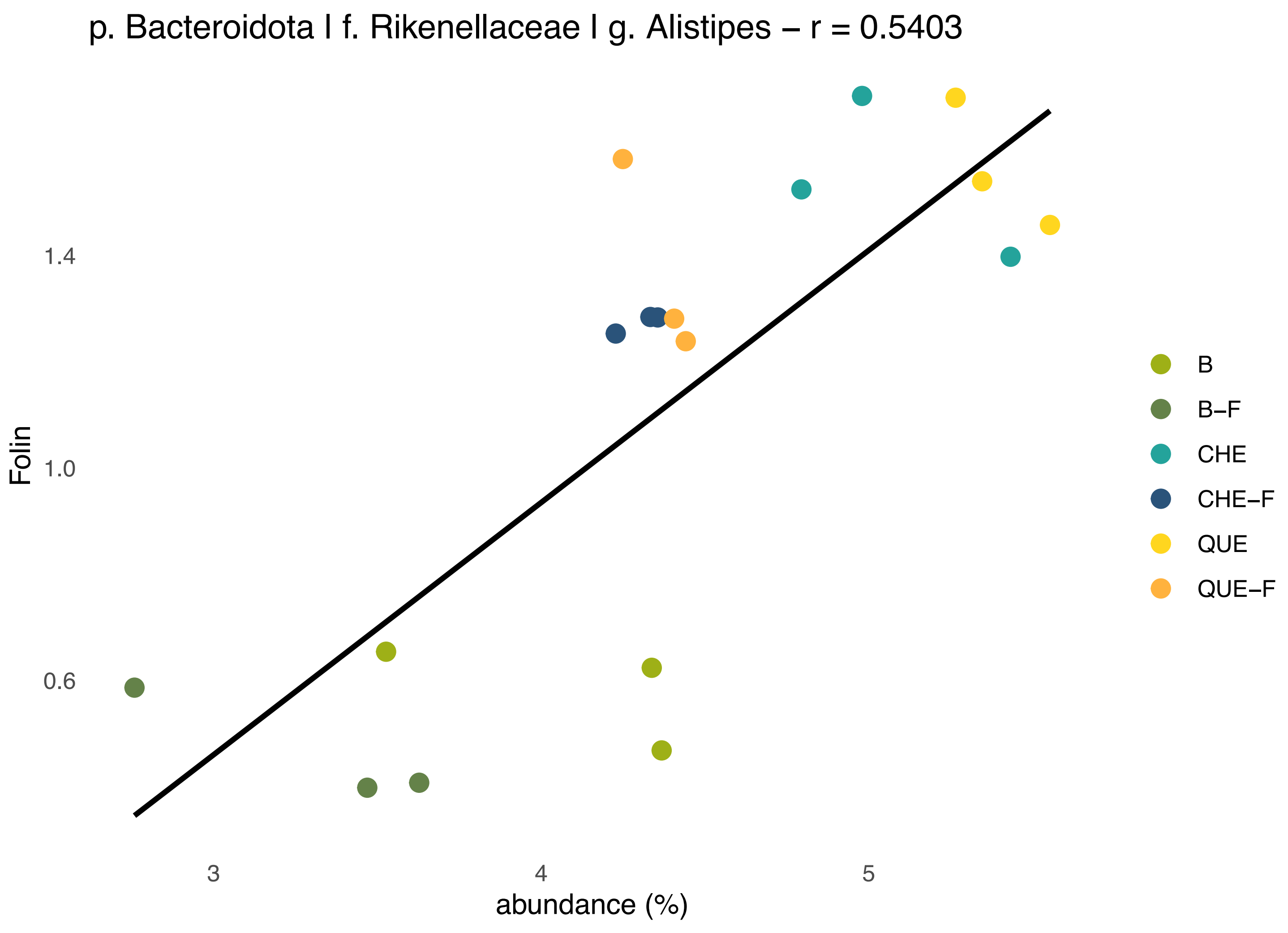
p. Bacteroidota | f. Rikenellaceae | g. Alistipes – r = 0.5403
Folin
1.4
1.0
0.6
3
4
5
abundance (%)
B
B–F
CHE
CHE–F
QUE
QUE–F

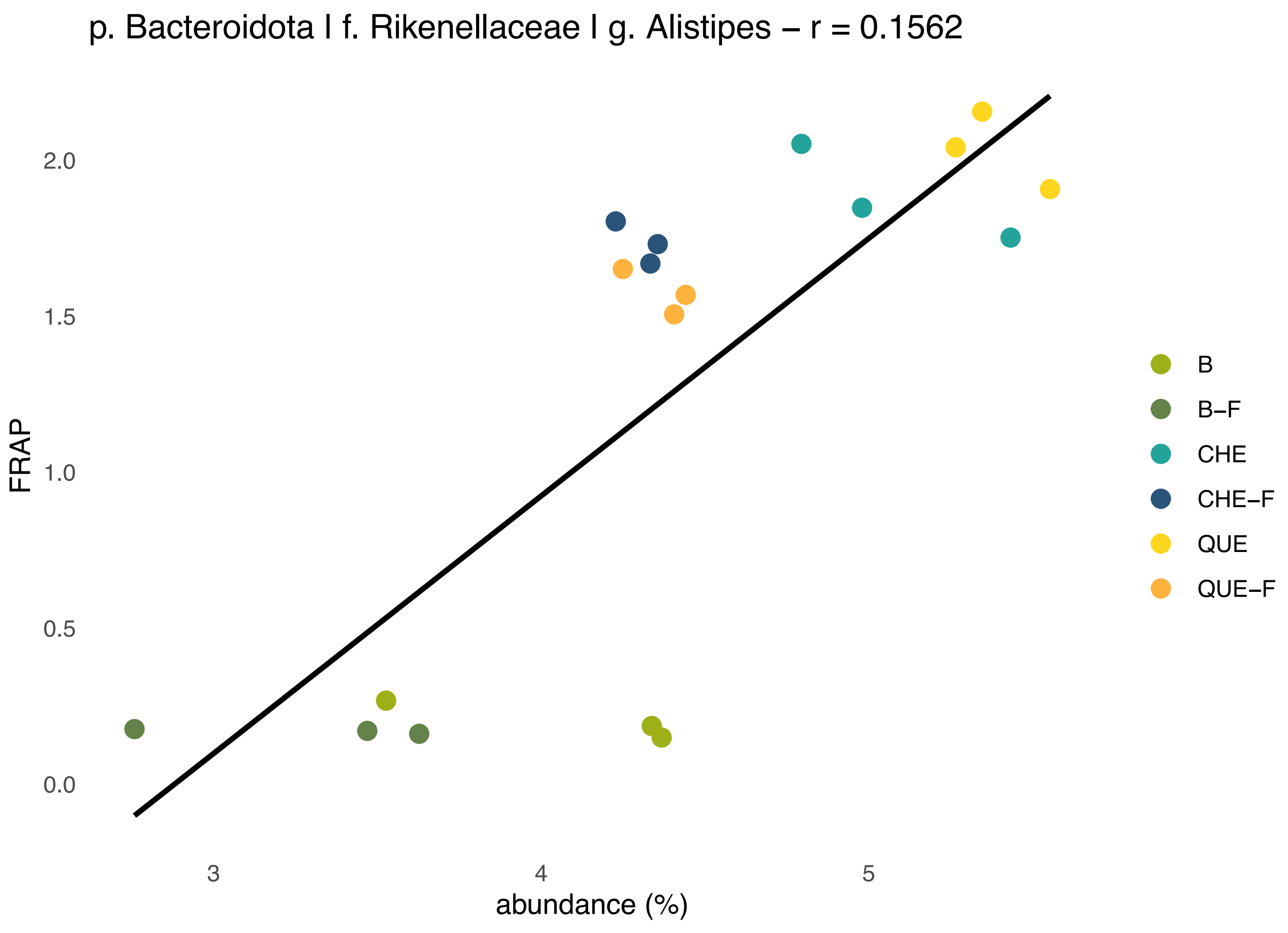
p. Bacteroidota I f. Rikenellaceae I g. Alistipes – r = 0.1562
FRAP
2.0
1.5
1.0
0.5
0.0
3
4
5
abundance (%)
B
B–F
CHE
CHE–F
QUE
QUE–F

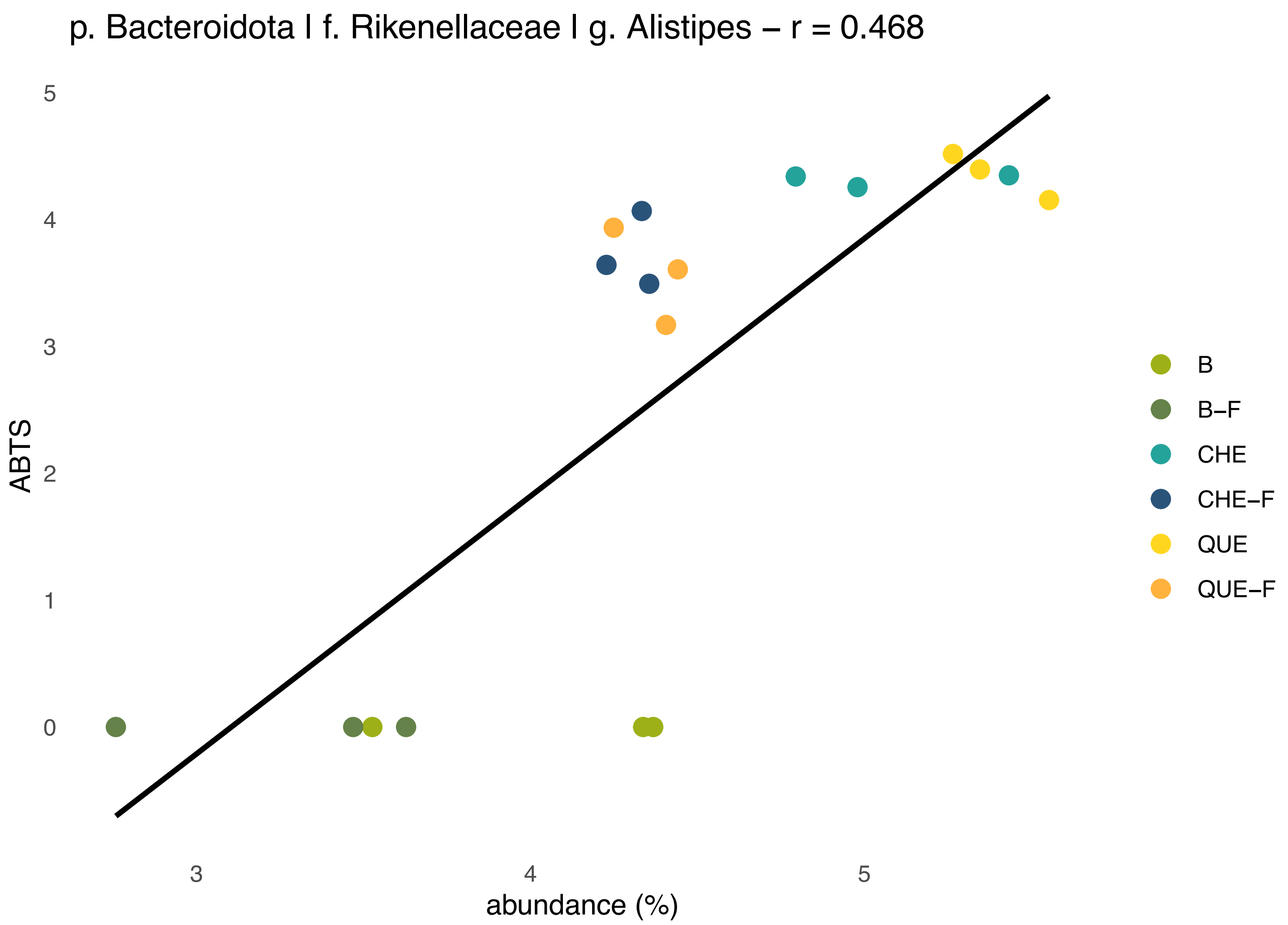
p. Bacteroidota I f. Rikenellaceae I g. Alistipes – r = 0.468
ABTS
5
4
3
2
1
0
3
4
5
abundance (%)
B
B–F
CHE
CHE–F
QUE
QUE–F

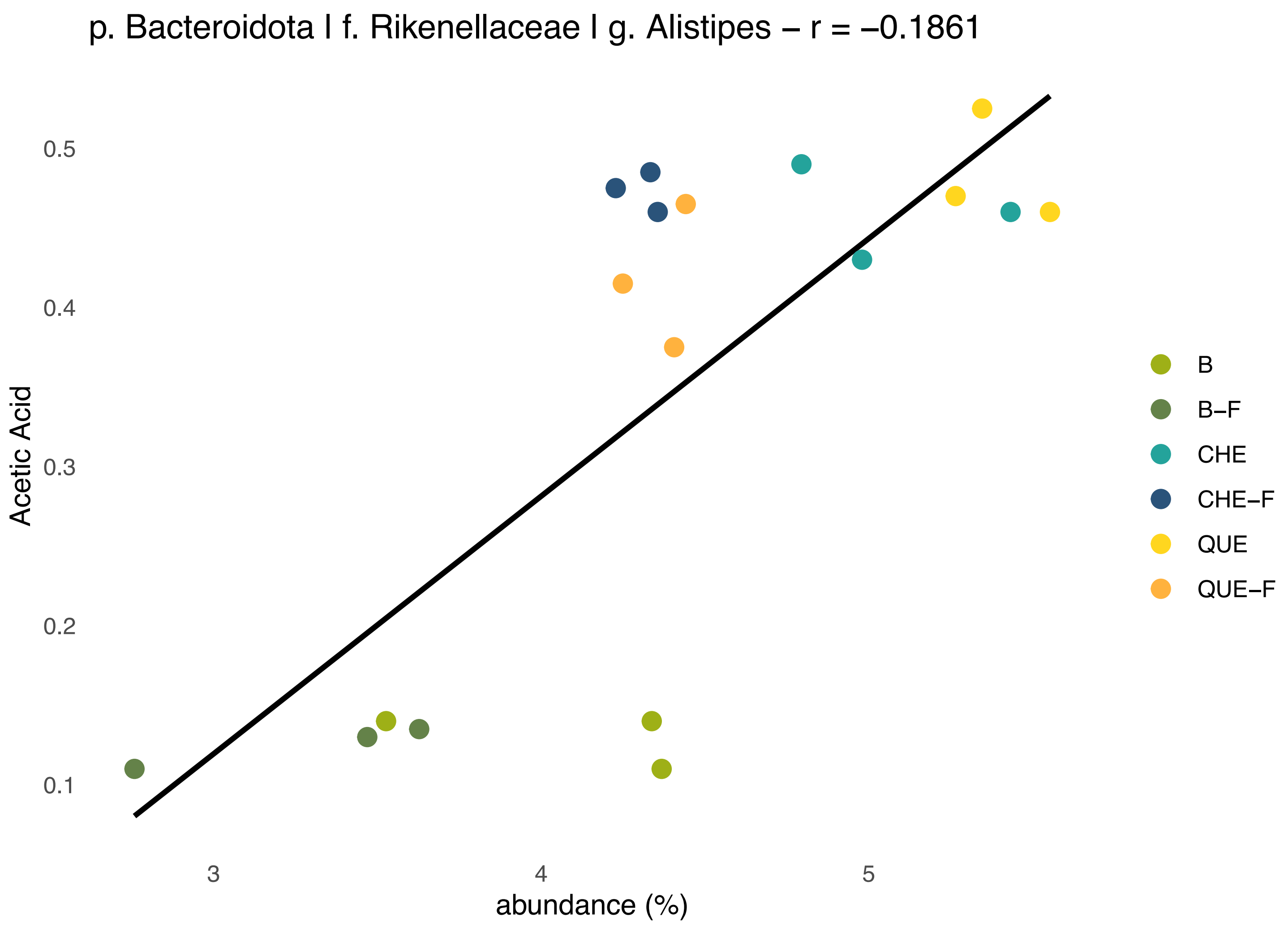

p. Bacteroidota | f. Rikenellaceae | g. Alistipes – r = –0.1861
Acetic Acid
0.5
0.4
0.3
0.2
0.1
3
4
5
abundance (%)
B
B–F
CHE
CHE–F
QUE
QUE–F

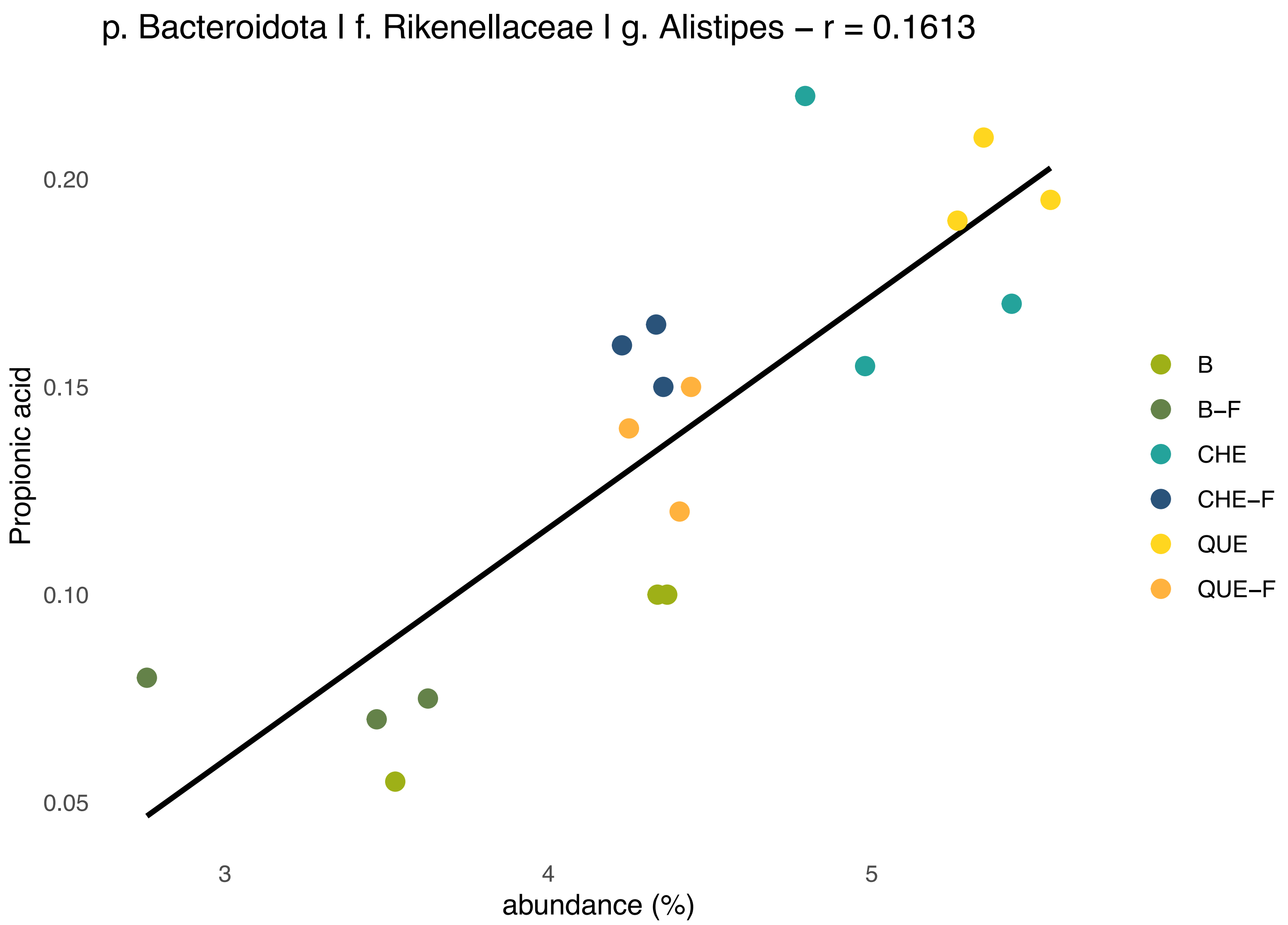

p. Bacteroidota | f. Rikenellaceae | g. Alistipes – r = 0.1613
Propionic acid
0.20
0.15
0.10
0.05
3
4
5
abundance (%)
B
B–F
CHE
CHE–F
QUE
QUE–F

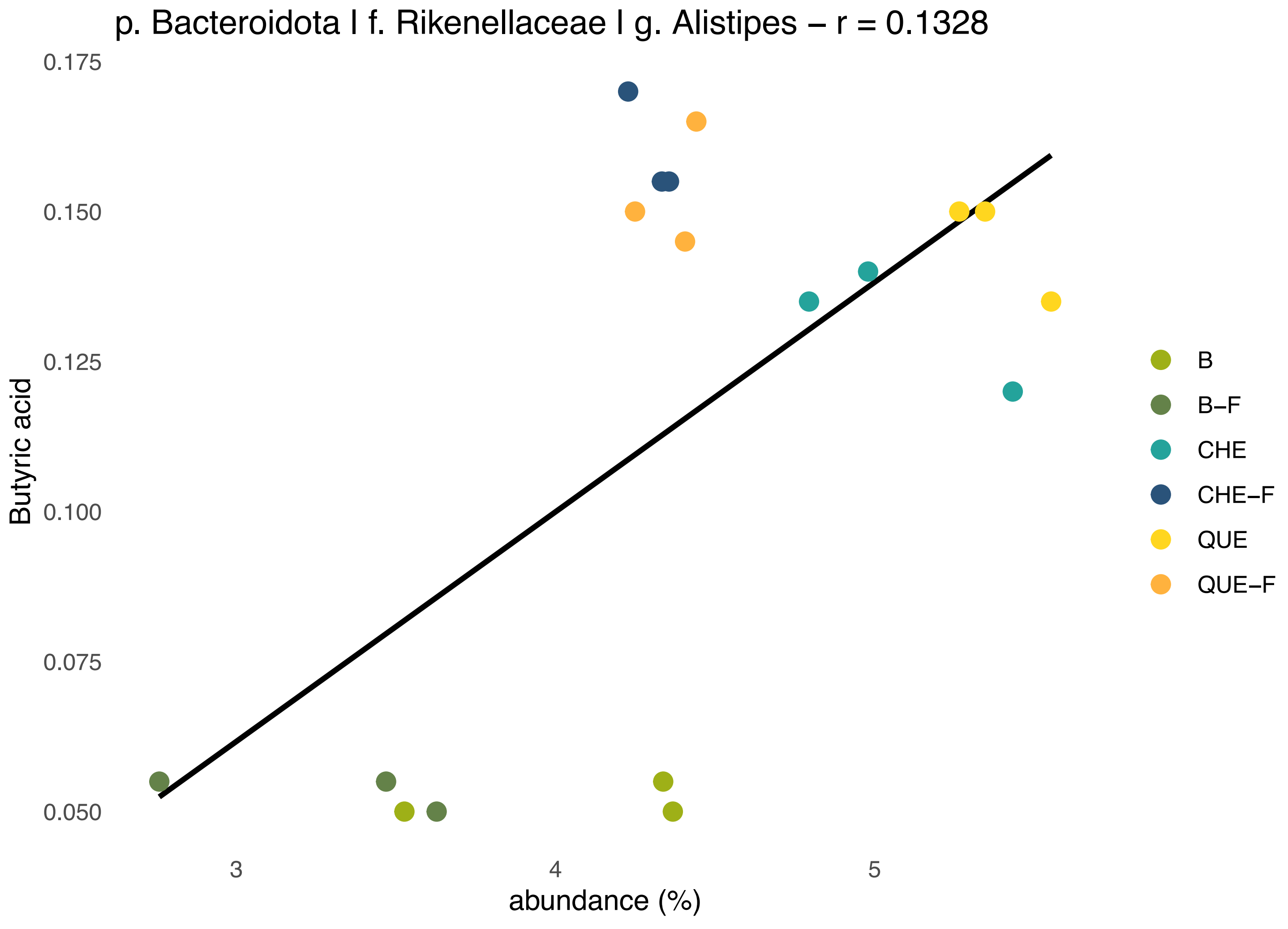

p. Bacteroidota | f. Rikenellaceae | g. Alistipes – r = 0.1328
0.175
0.150
0.125
0.100
0.075
0.050
Butyric acid
3
4
5
abundance (%)
B
B–F
CHE
CHE–F
QUE
QUE–F

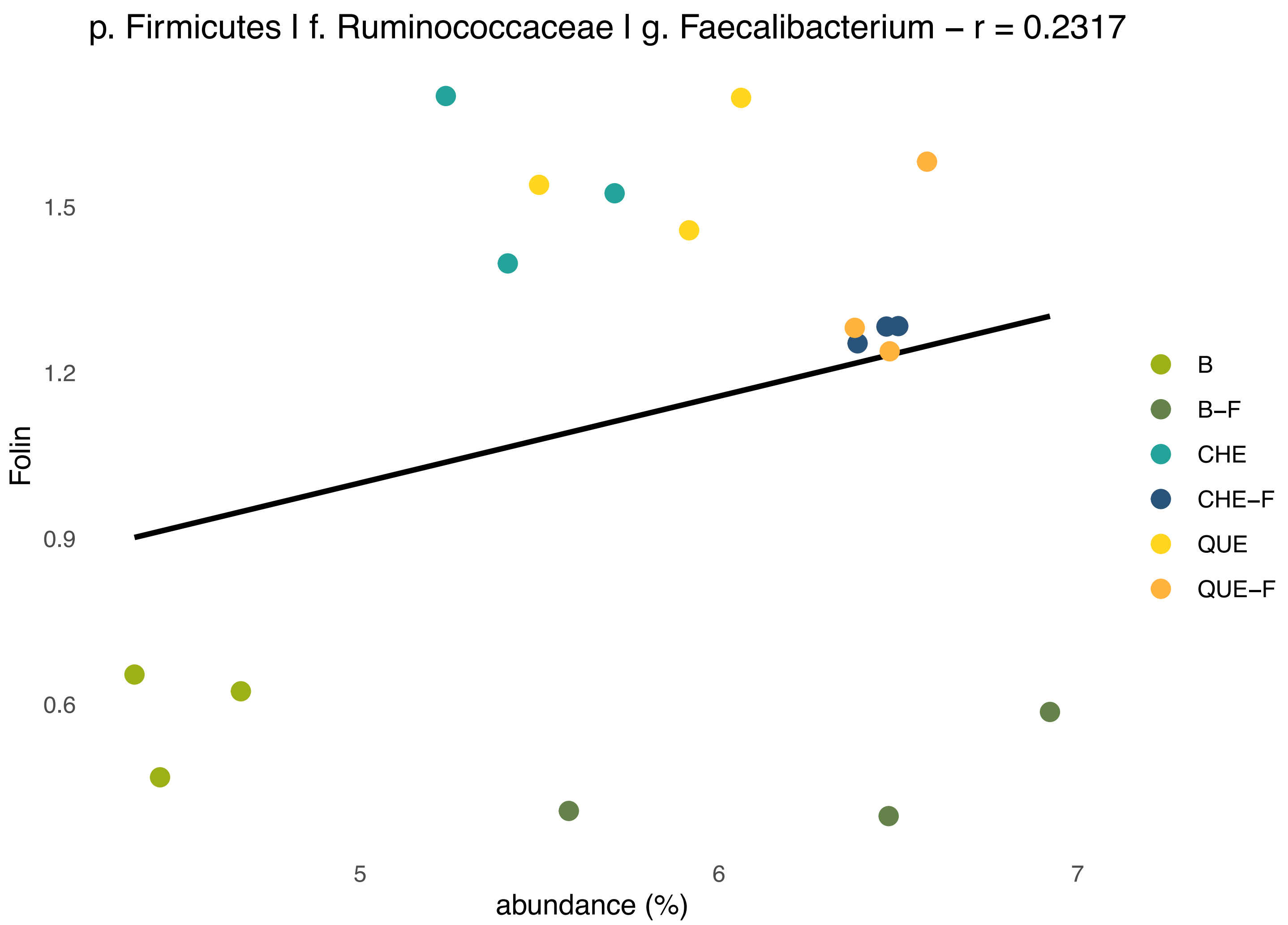
p. Firmicutes I f. Ruminococcaceae I g. Faecalibacterium – r = 0.2317
Folin
1.5
1.2
0.9
0.6
5
6
7
abundance (%)
B
B–F
CHE
CHE–F
QUE
QUE–F

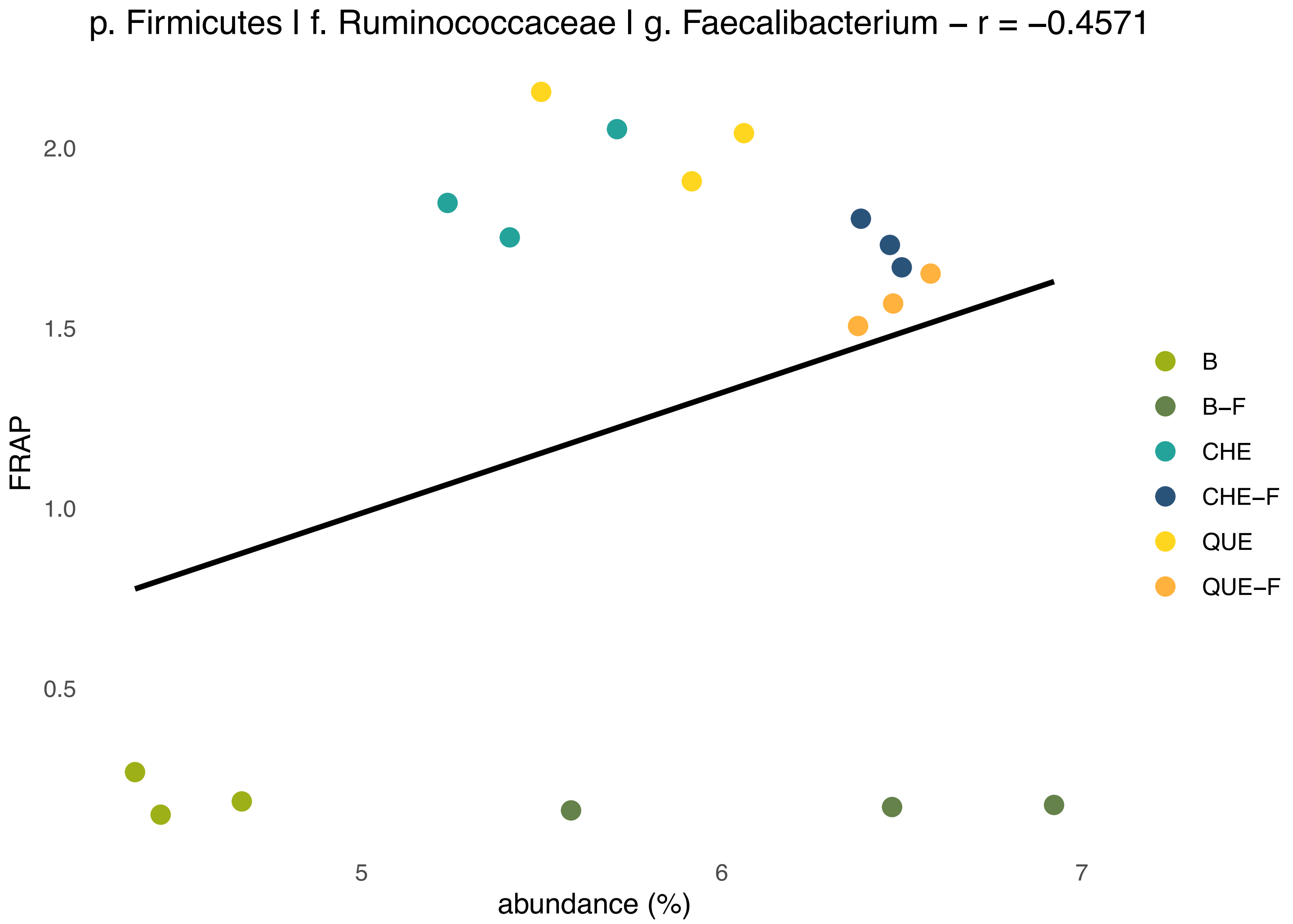
p. Firmicutes I f. Ruminococcaceae I g. Faecalibacterium – r = –0.4571
FRAP
2.0
1.5
1.0
0.5
5
6
7
abundance (%)
B
B–F
CHE
CHE–F
QUE
QUE–F

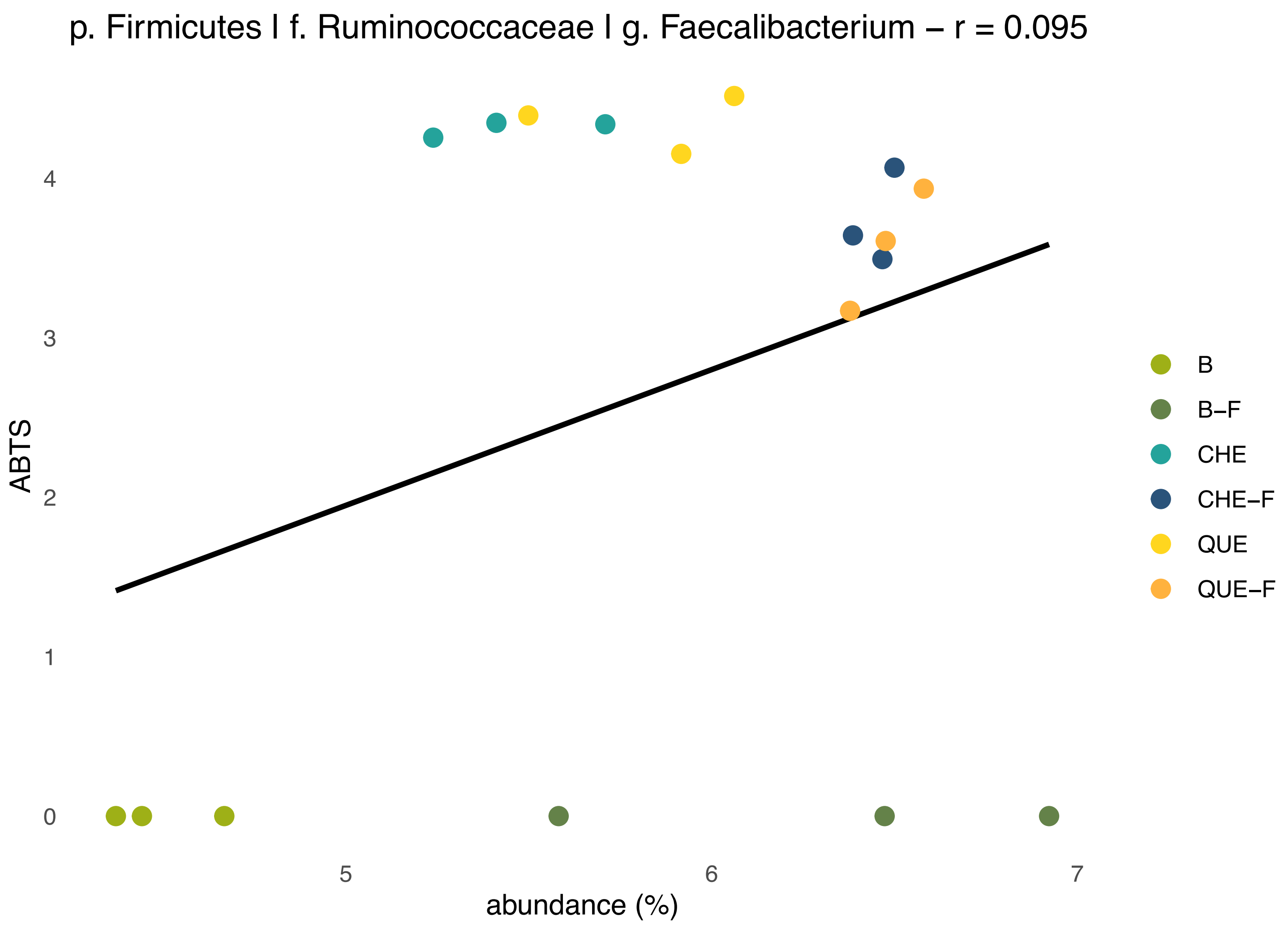

p. Firmicutes l f. Ruminococcaceae l g. Faecalibacterium – r = 0.095
ABTS
0
1
2
3
4
5
6
7
abundance (%)
B
B–F
CHE
CHE–F
QUE
QUE–F

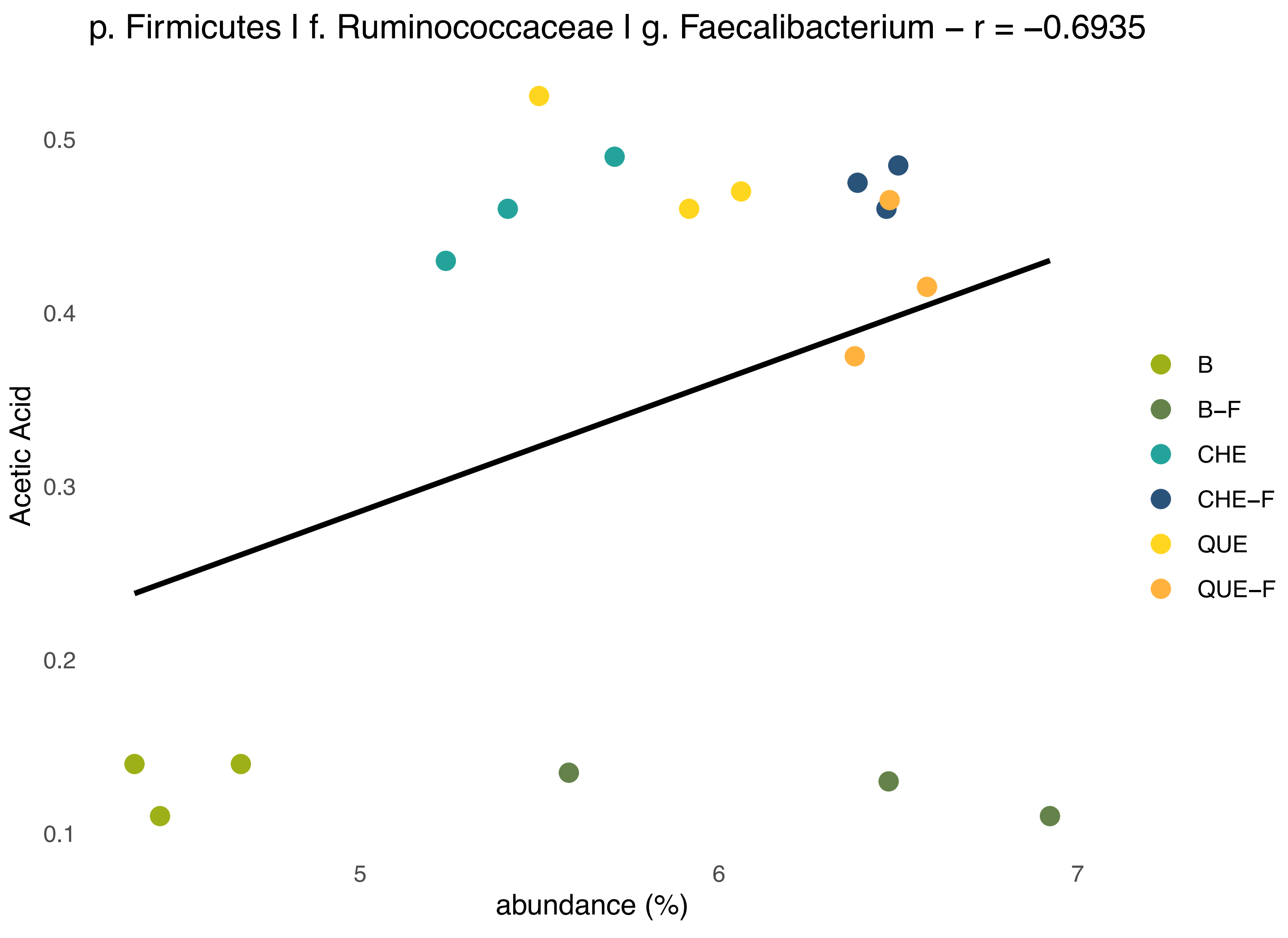

p. Firmicutes I f. Ruminococcaceae I g. Faecalibacterium – r = –0.6935
Acetic Acid
0.5
0.4
0.3
0.2
0.1
5
6
7
abundance (%)
B
B–F
CHE
CHE–F
QUE
QUE–F

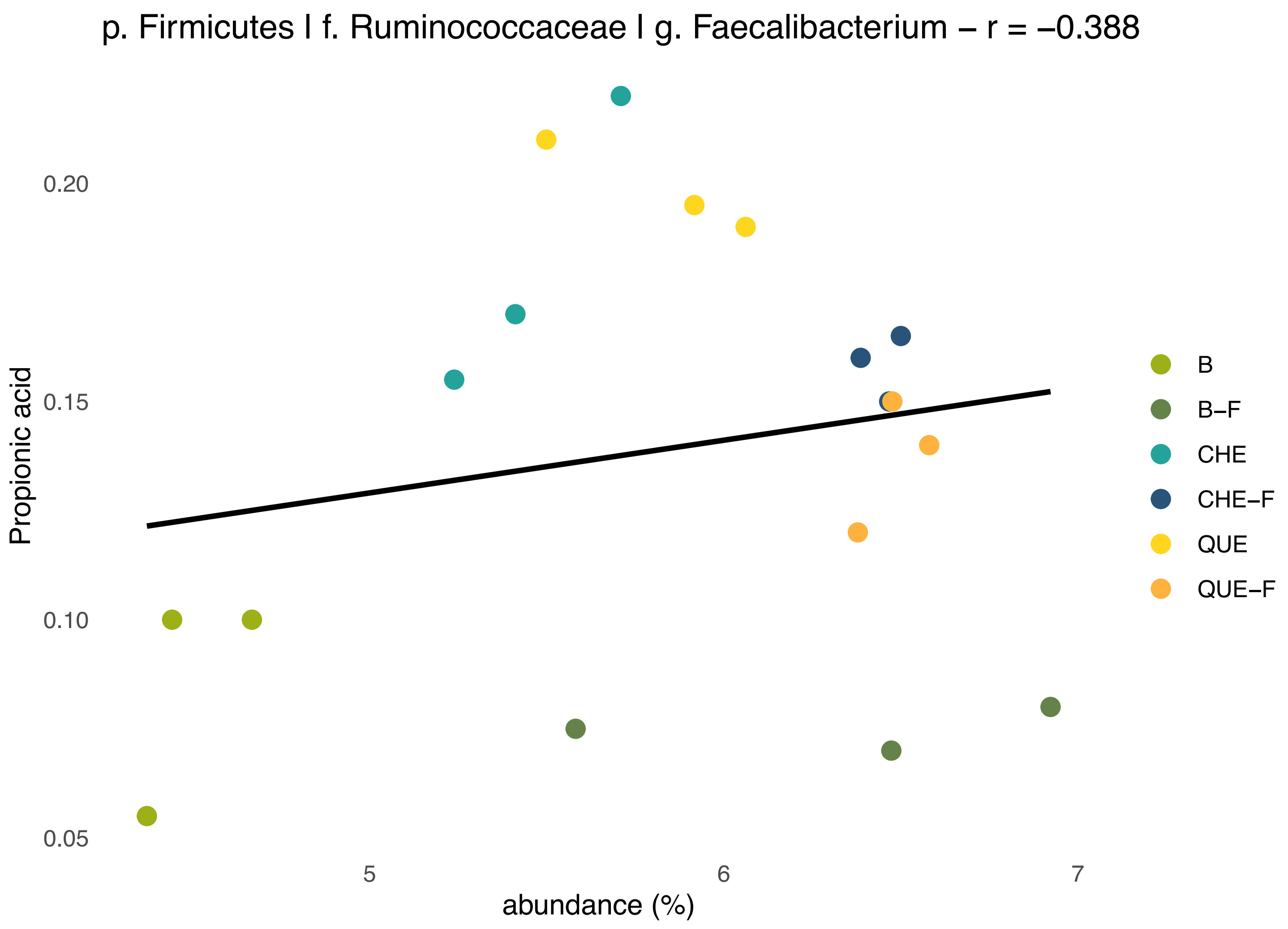
p. Firmicutes I f. Ruminococcaceae I g. Faecalibacterium – r = –0.388
Propionic acid
0.20
0.15
0.10
0.05
5
6
7
abundance (%)
B
B–F
CHE
CHE–F
QUE
QUE–F

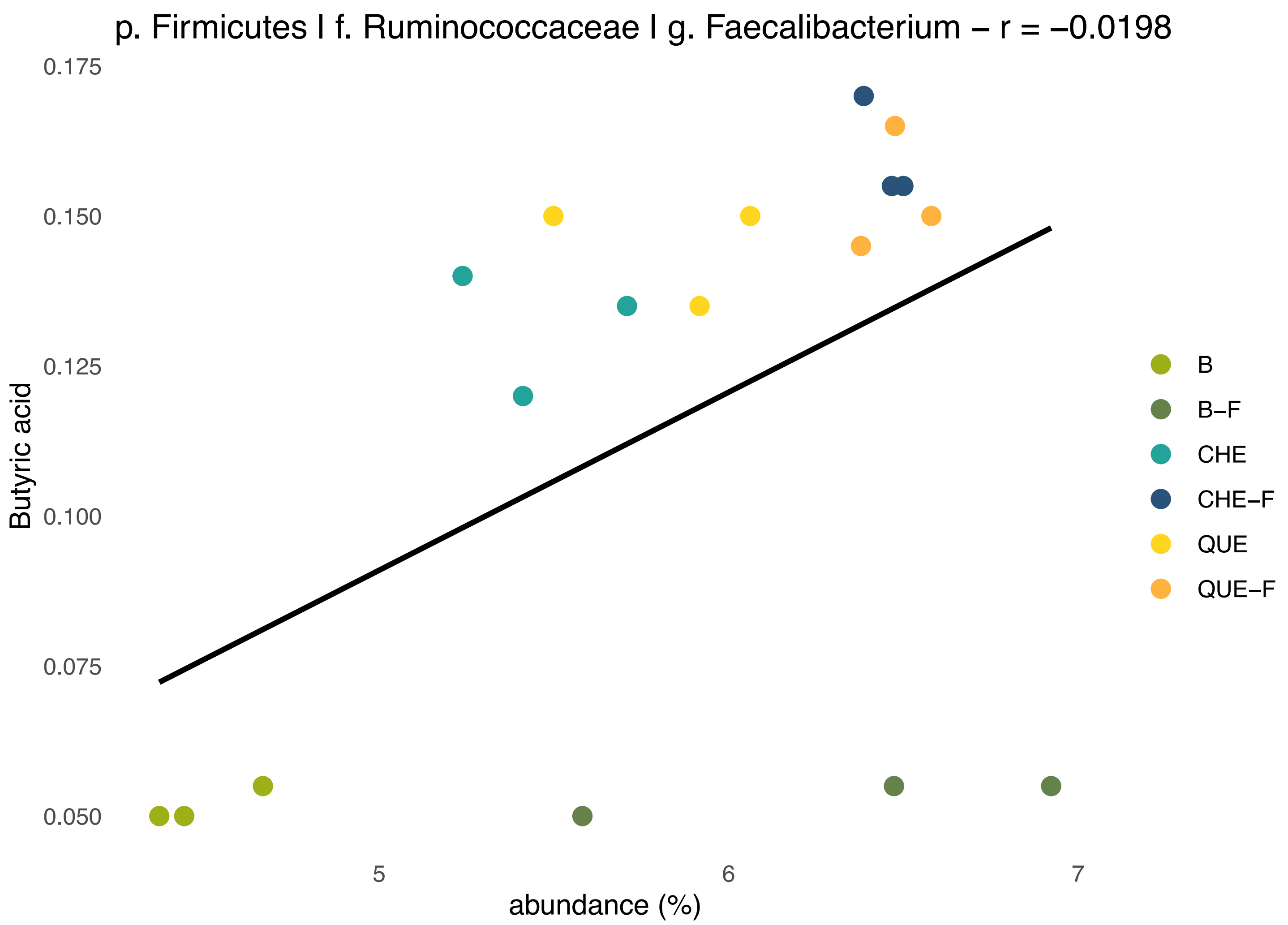
p. Firmicutes I f. Ruminococcaceae I g. Faecalibacterium – r = –0.0198
Butyric acid
0.175
0.150
0.125
0.100
0.075
0.050
5
6
7
abundance (%)
B
B–F
CHE
CHE–F
QUE
QUE–F

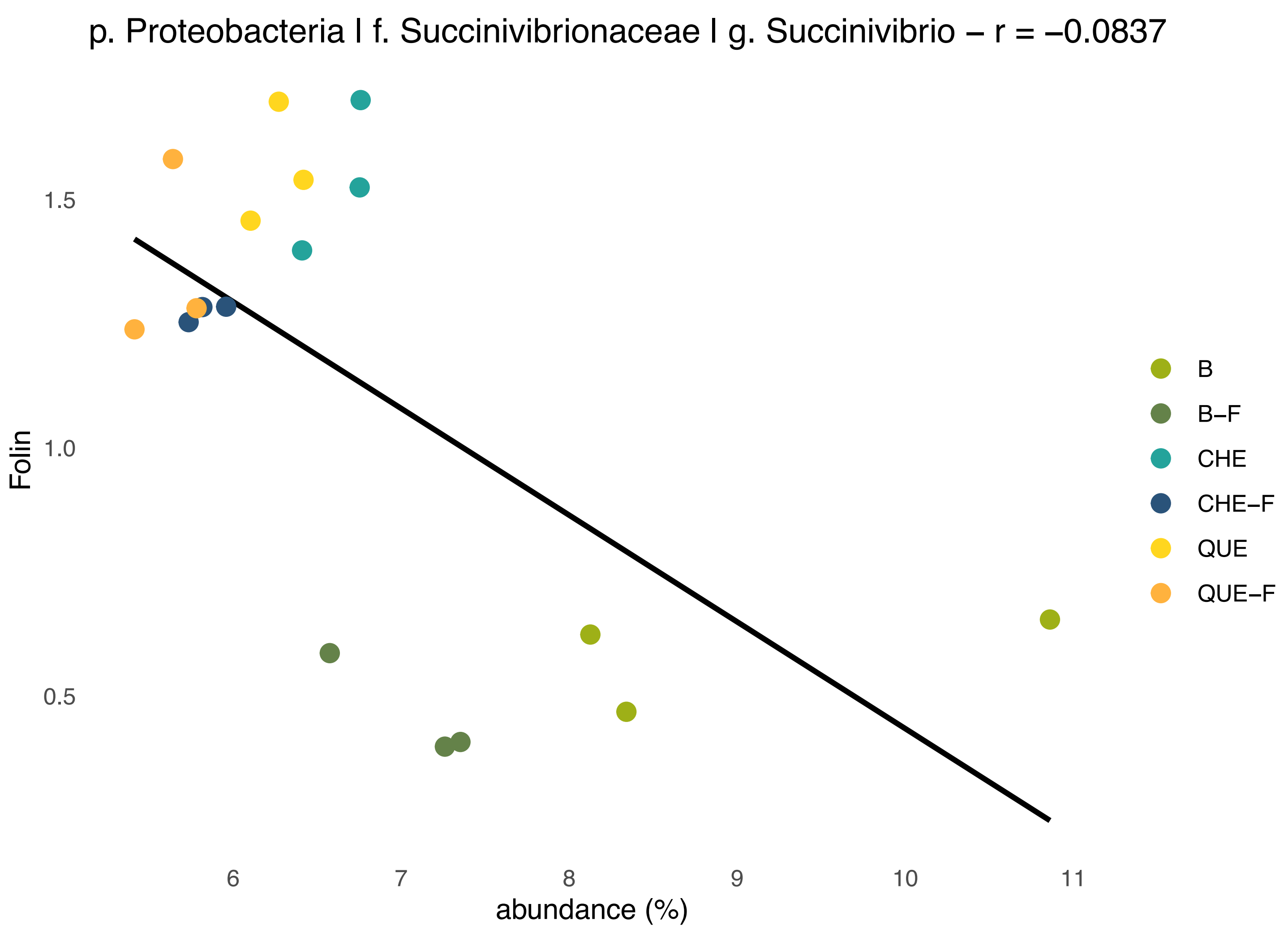

p. Proteobacteria I f. Succinivibrionaceae I g. Succinivibrio – r = −0.0837
Folin
1.5
1.0
0.5
6
7
8
9
10
11
abundance (%)
B
B−F
CHE
CHE−F
QUE
QUE−F

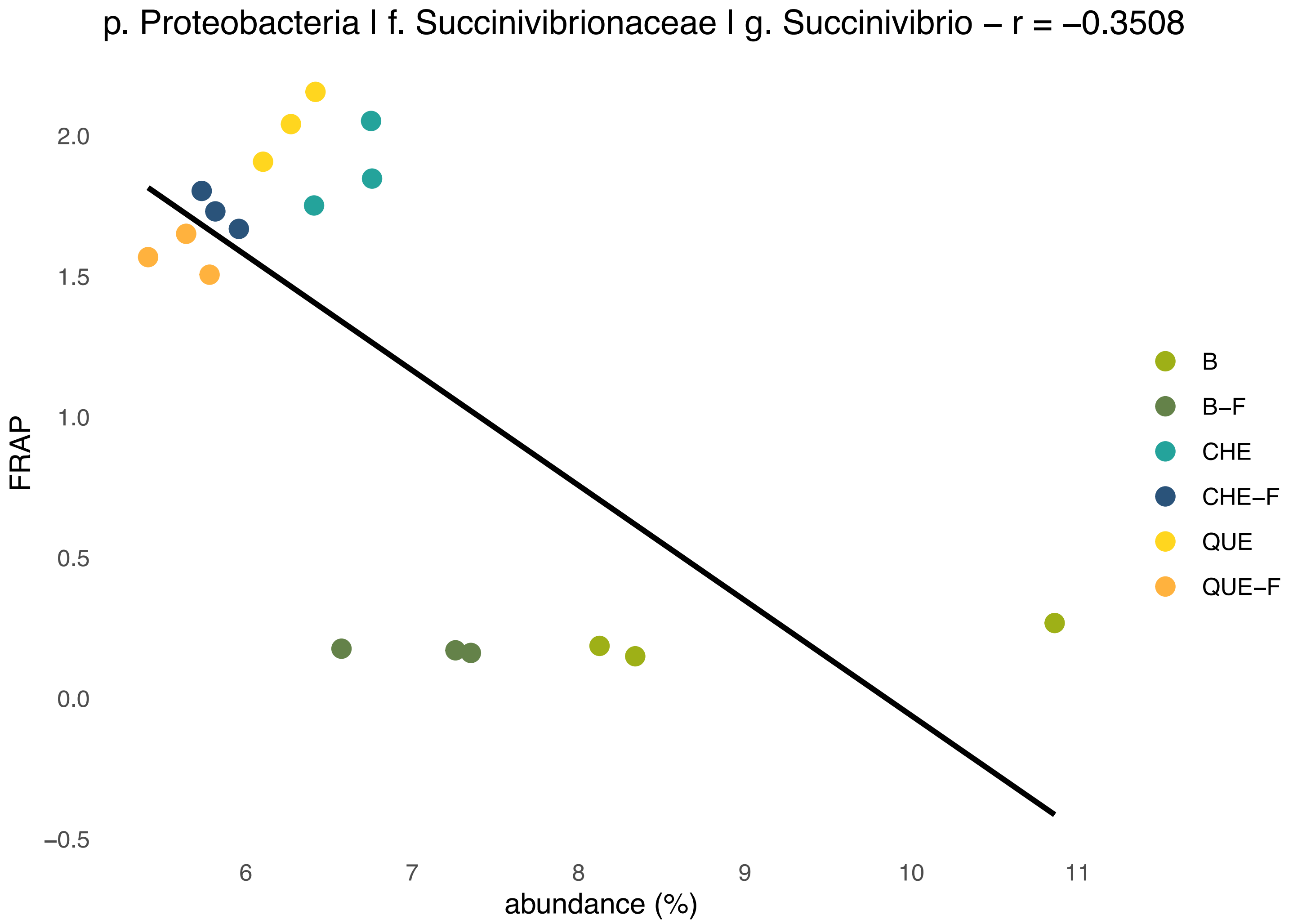
p. Proteobacteria I f. Succinivibrionaceae I g. Succinivibrio – r = –0.3508
FRAP
2.0
1.5
1.0
0.5
0.0
–0.5
6
7
8
9
10
11
abundance (%)
B
B–F
CHE
CHE–F
QUE
QUE–F

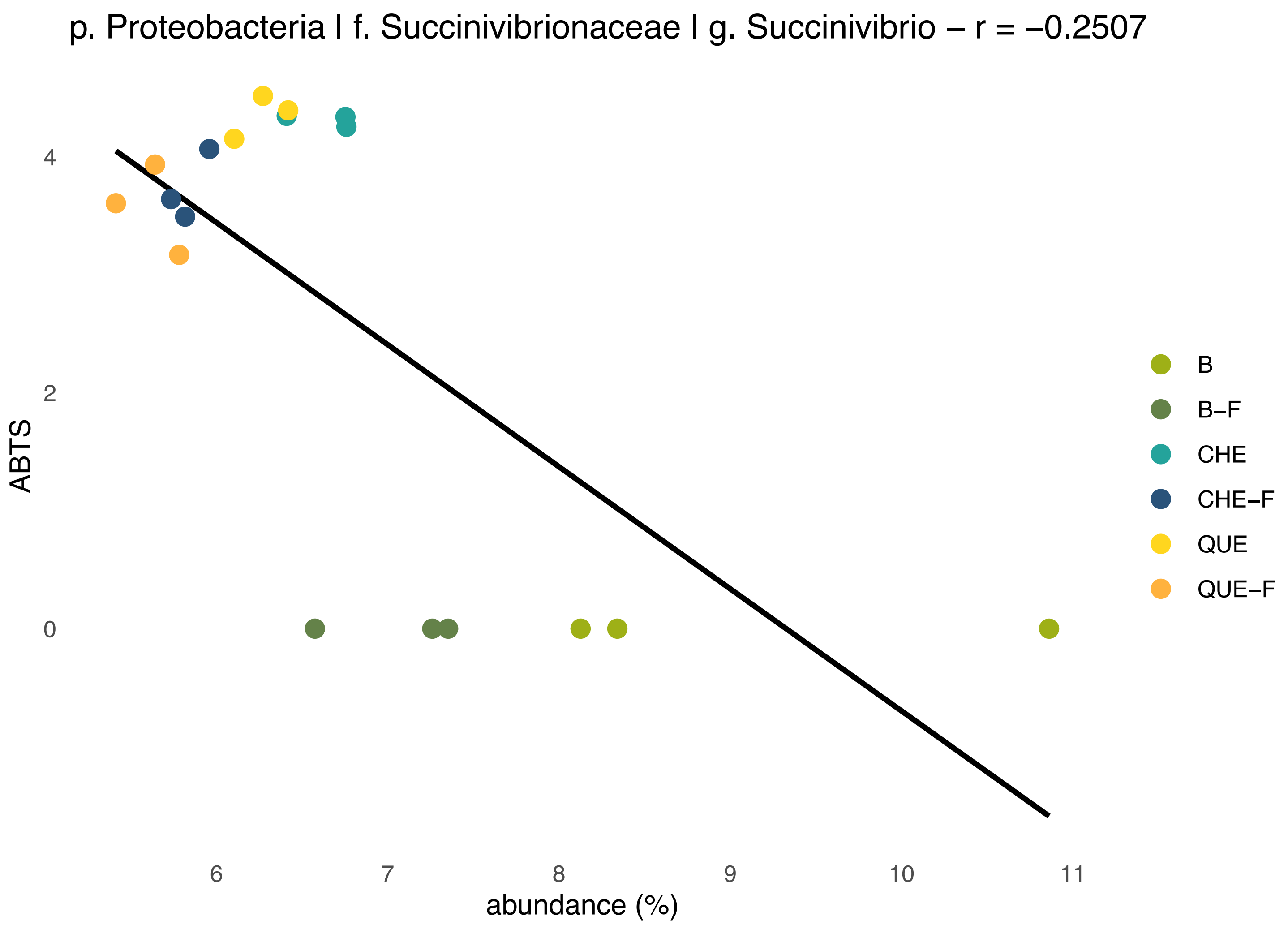
p. Proteobacteria I f. Succinivibrionaceae I g. Succinivibrio – r = –0.2507
ABTS
4
2
0
6
7
8
9
10
11
abundance (%)
B
B–F
CHE
CHE–F
QUE
QUE–F

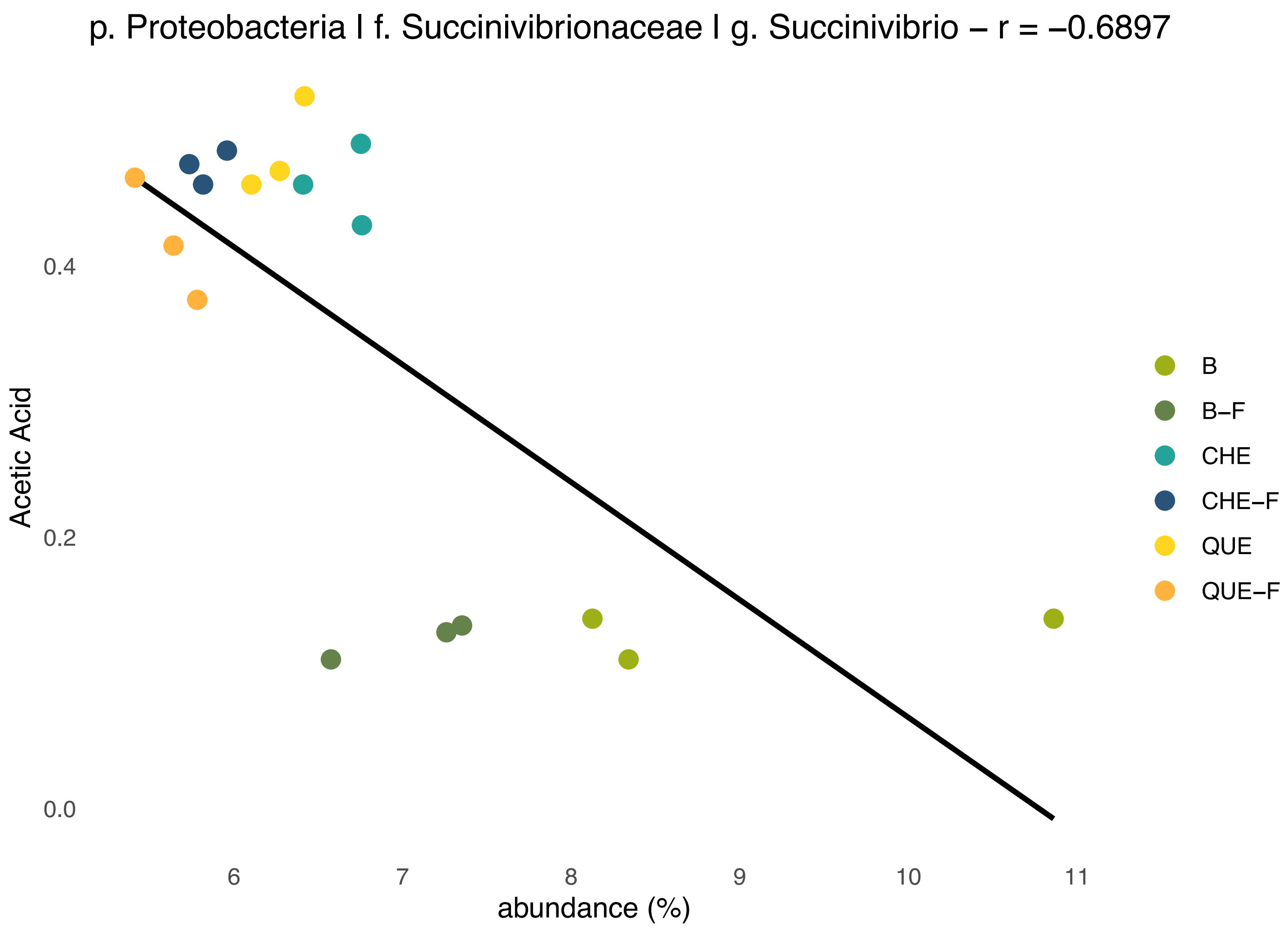

p. Proteobacteria l f. Succinivibrionaceae l g. Succinivibrio – r = –0.6897
Acetic Acid
0.0
0.2
0.4
6
7
8
9
10
11
abundance (%)
B
B–F
CHE
CHE–F
QUE
QUE–F

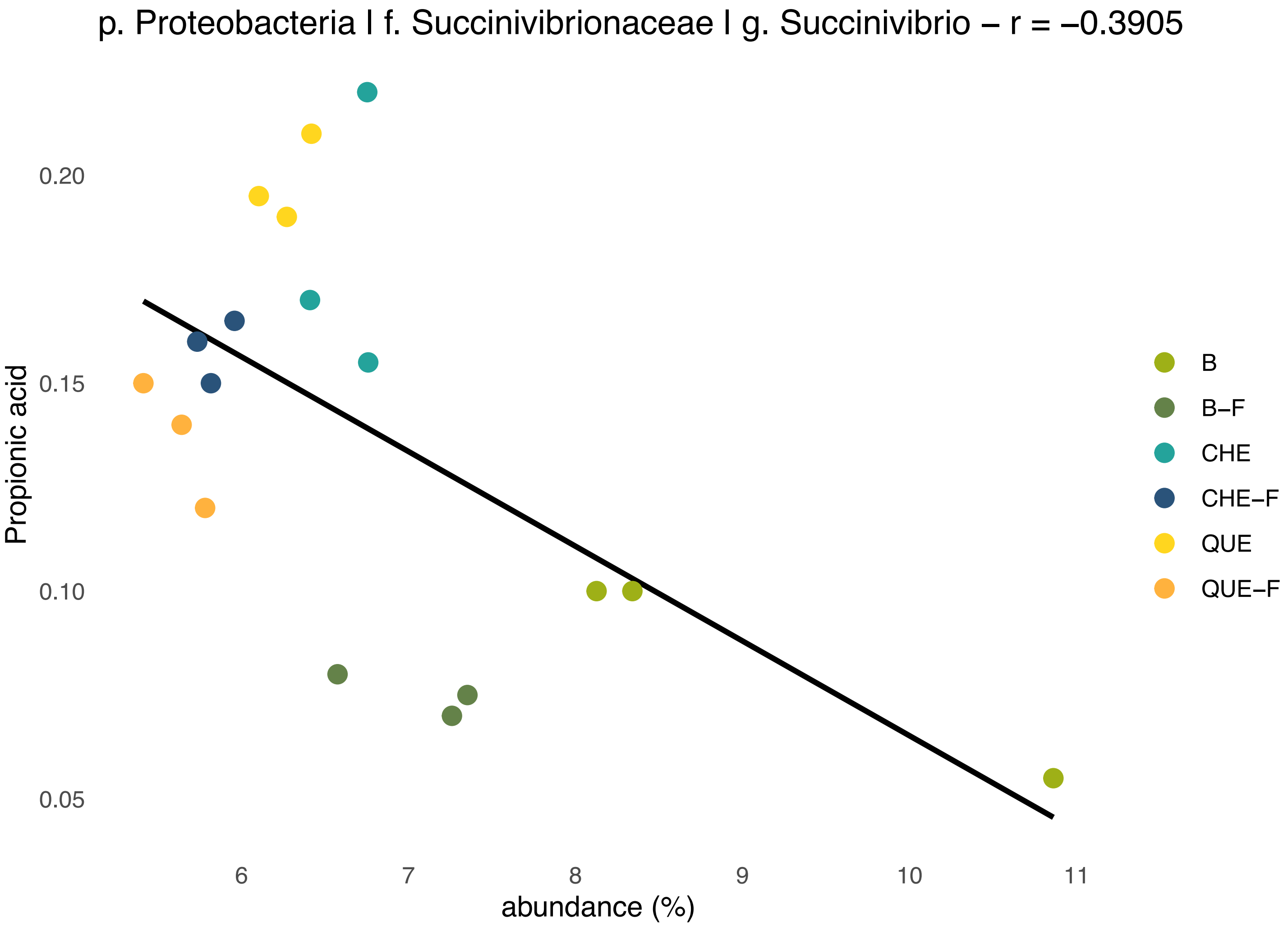

p. Proteobacteria I f. Succinivibrionaceae I g. Succinivibrio – r = –0.3905
Propionic acid
0.20
0.15
0.10
0.05
6
7
8
9
10
11
abundance (%)
B
B–F
CHE
CHE–F
QUE
QUE–F

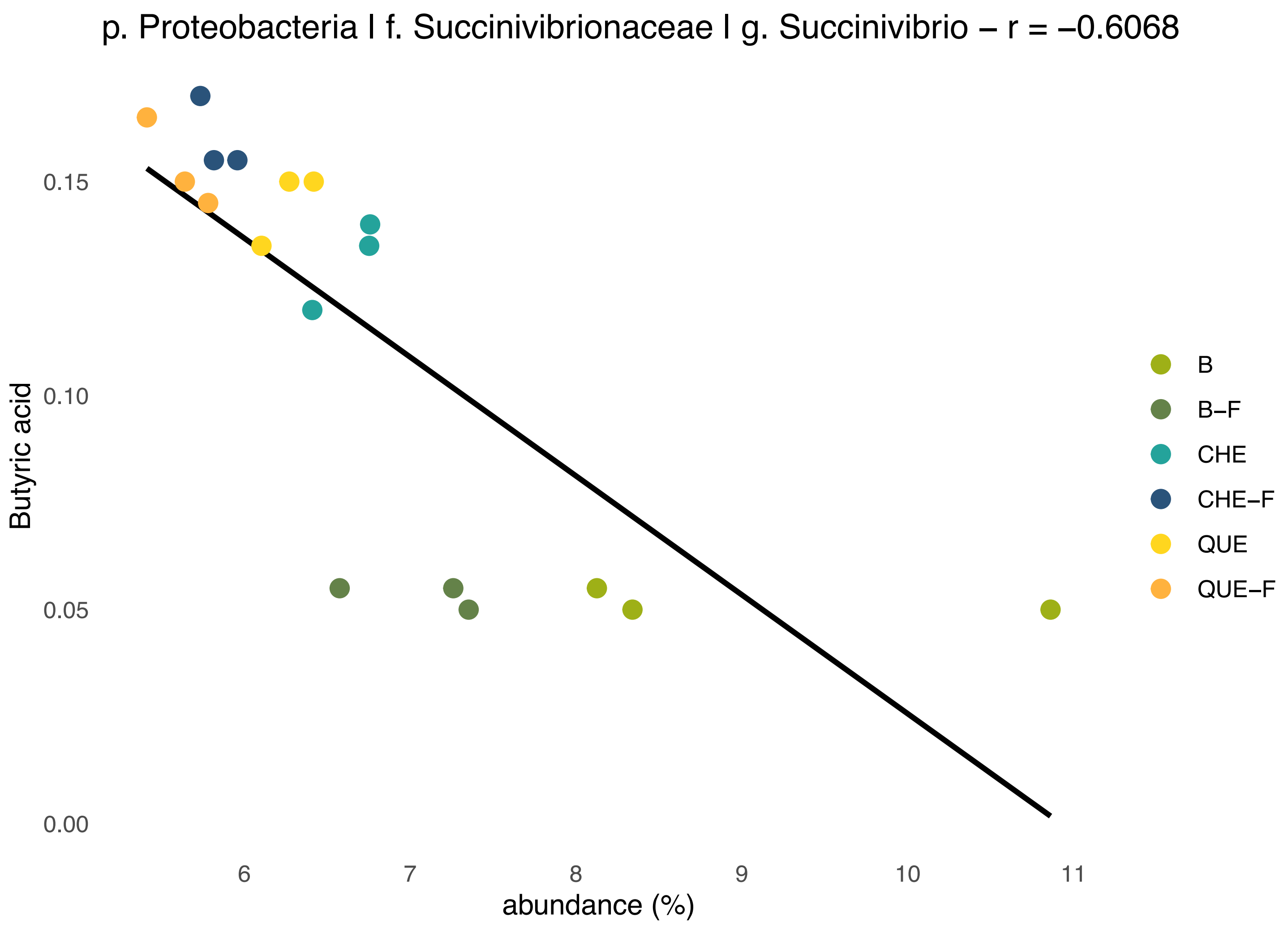
p. Proteobacteria I f. Succinivibrionaceae I g. Succinivibrio – r = –0.6068
Butyric acid
0.00
0.05
0.10
0.15
6
7
8
9
10
11
abundance (%)
B
B–F
CHE
CHE–F
QUE
QUE–F

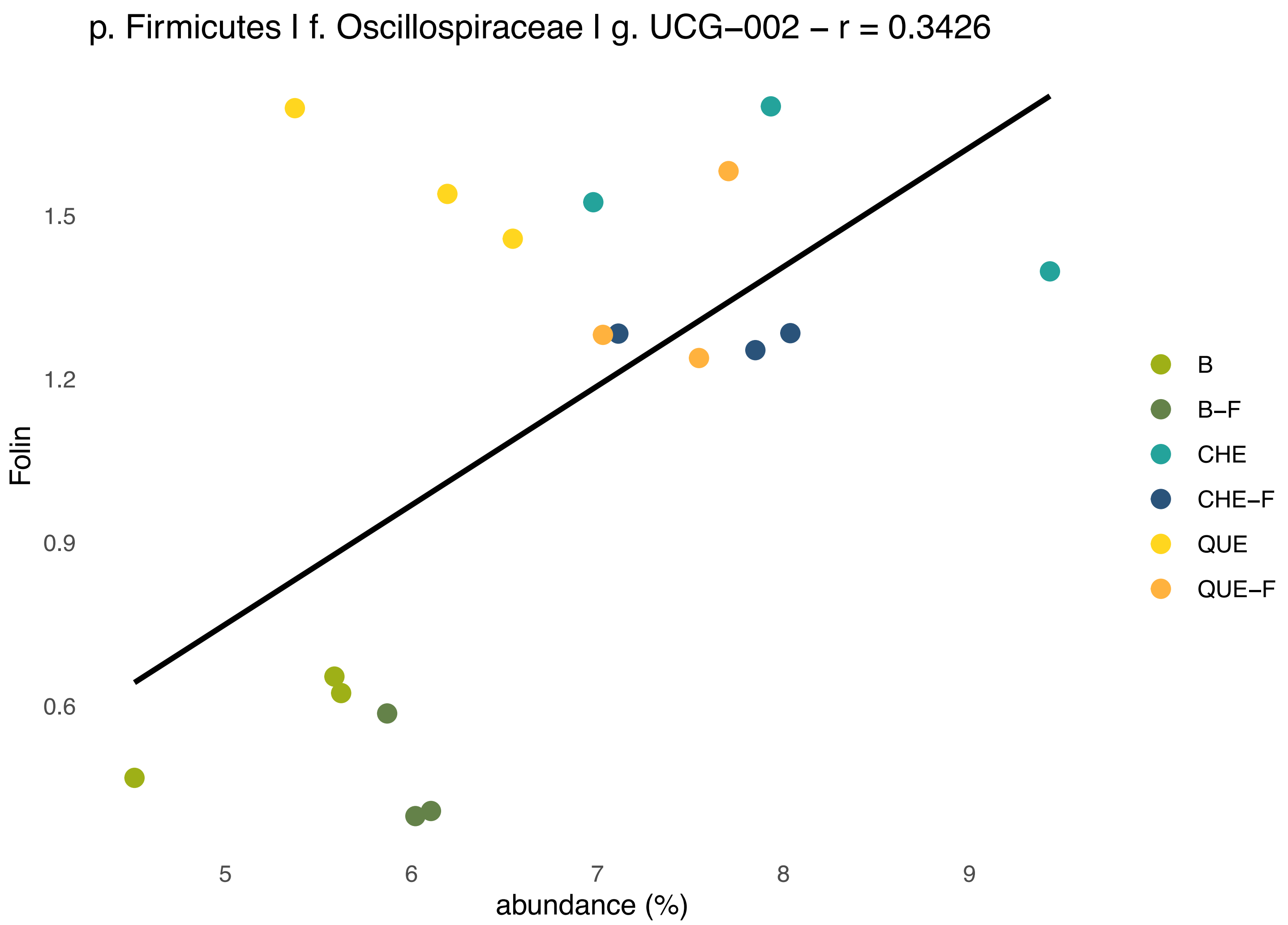
p. Firmicutes I f. Oscillospiraceae I g. UCG−002 − r = 0.3426
Folin
1.5
1.2
0.9
0.6
5
6
7
8
9
abundance (%)
B
B−F
CHE
CHE−F
QUE
QUE−F

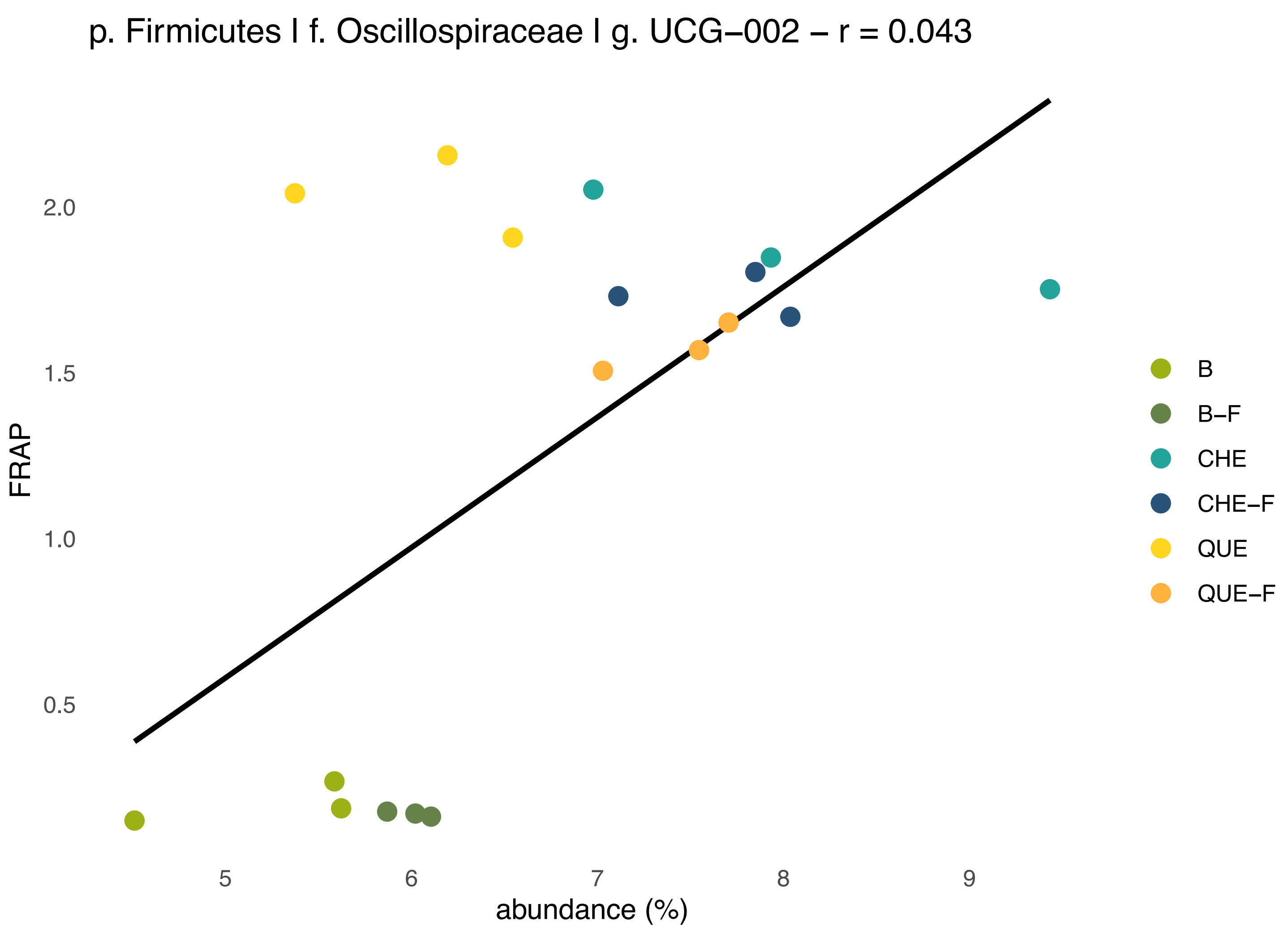

p. Firmicutes I f. Oscillospiraceae I g. UCG−002 − r = 0.043
FRAP
2.0
1.5
1.0
0.5
5
6
7
8
9
abundance (%)
B
B−F
CHE
CHE−F
QUE
QUE−F

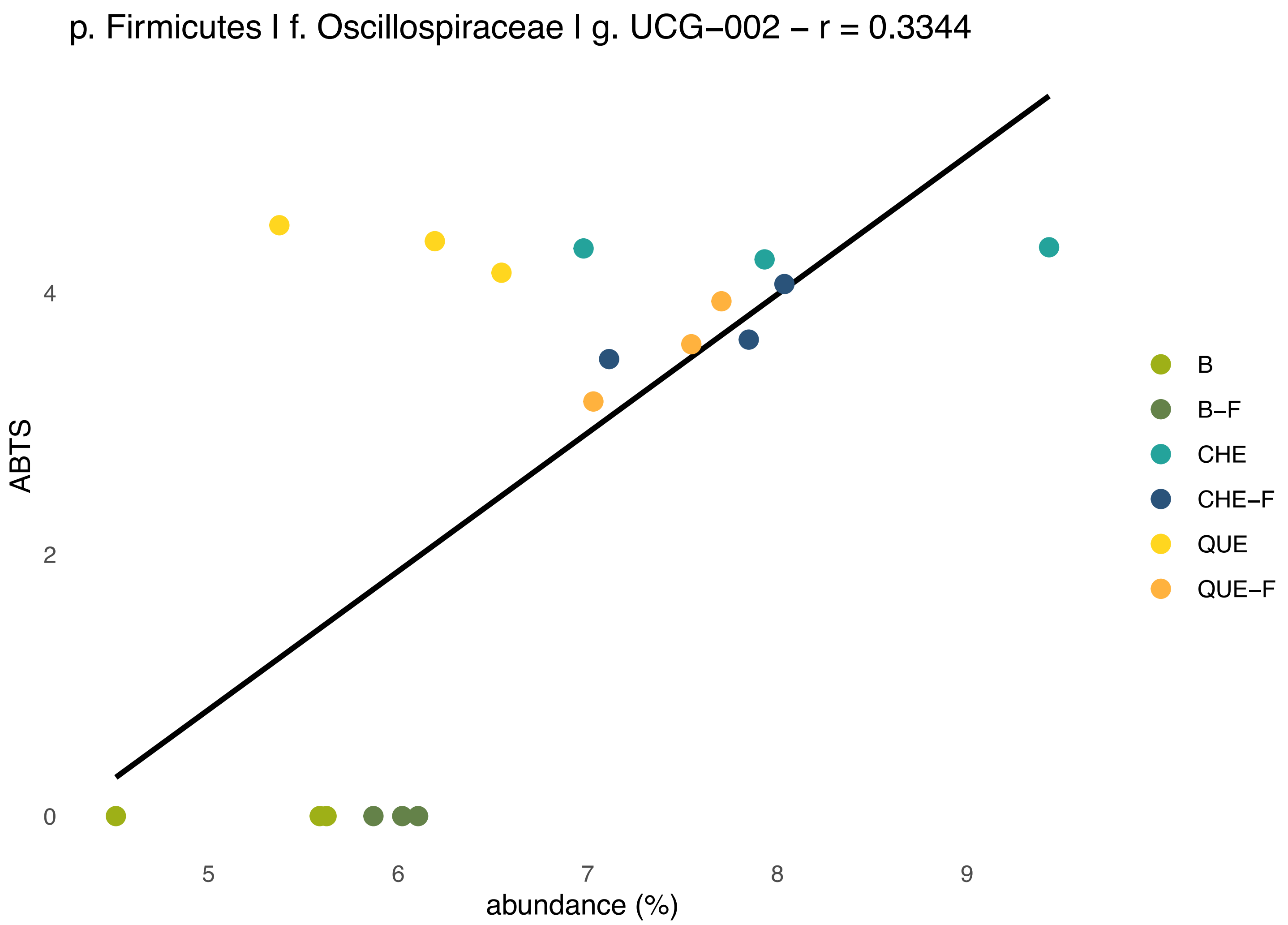

p. Firmicutes I f. Oscillospiraceae I g. UCG−002 − r = 0.3344
ABTS
4
2
0
5
6
7
8
9
abundance (%)
B
B−F
CHE
CHE−F
QUE
QUE−F

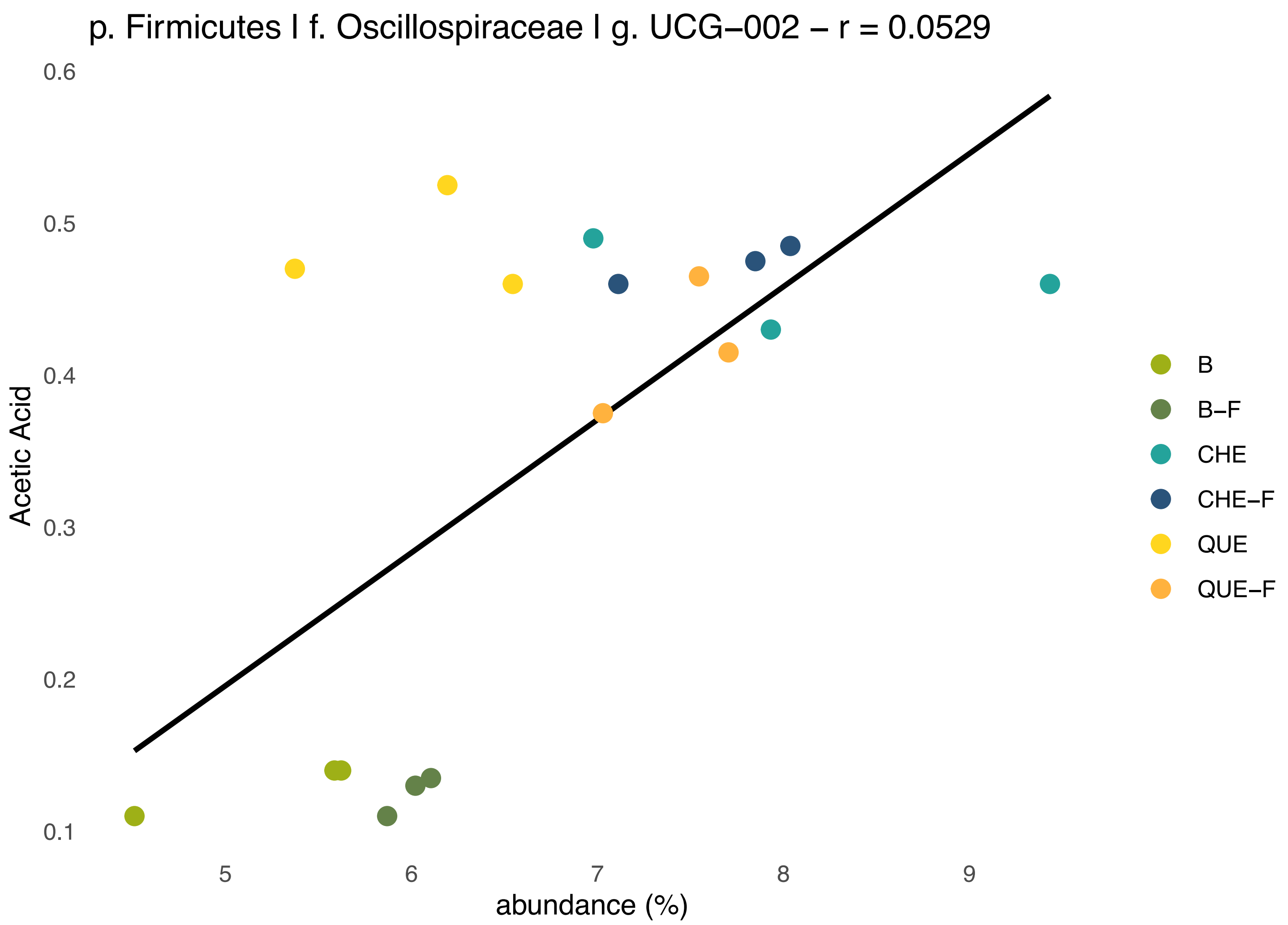

p. Firmicutes I f. Oscillospiraceae I g. UCG−002 − r = 0.0529
Acetic Acid
0.6
0.5
0.4
0.3
0.2
0.1
5
6
7
8
9
abundance (%)
B
B−F
CHE
CHE−F
QUE
QUE−F

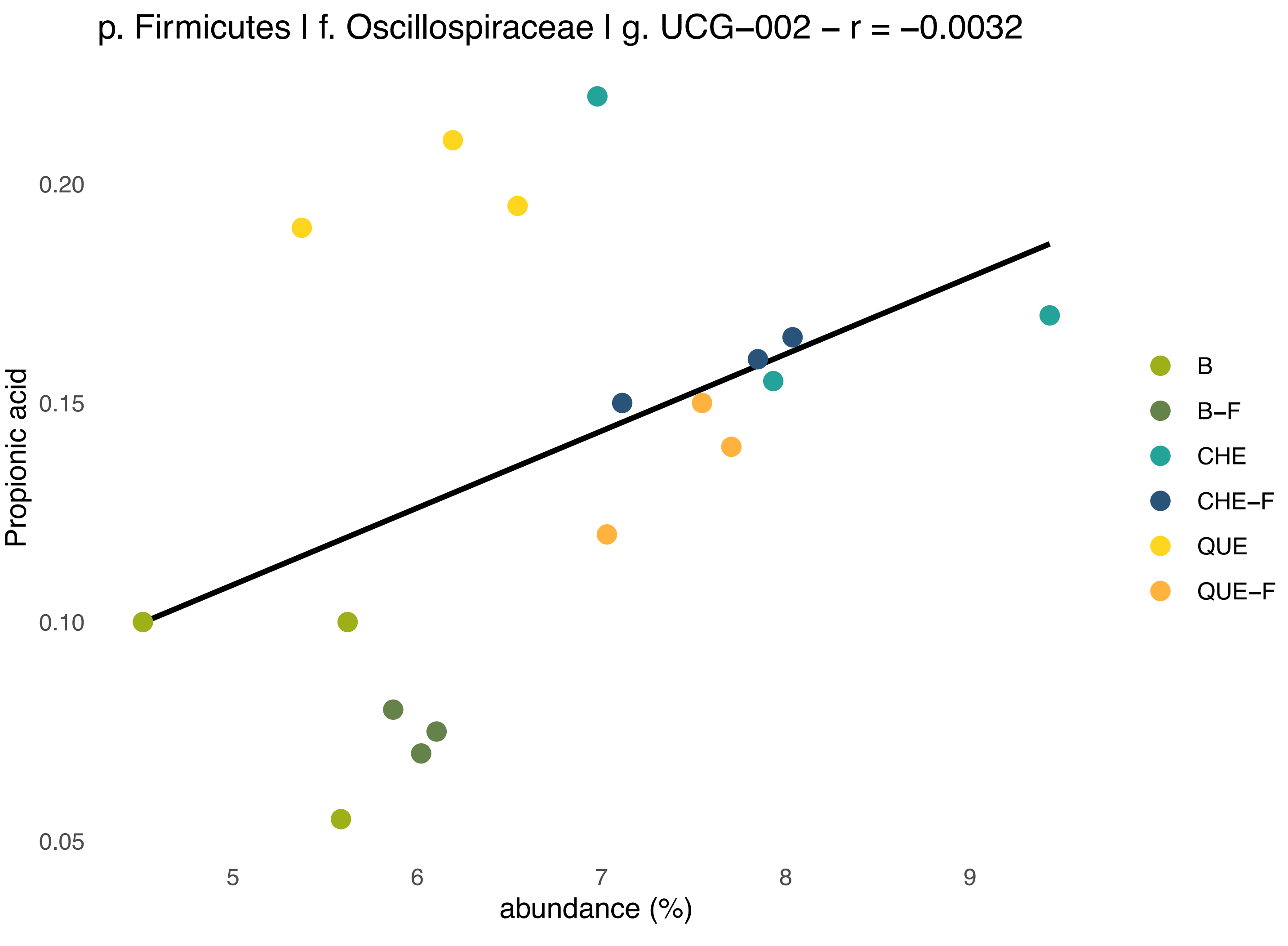
p. Firmicutes I f. Oscillospiraceae I g. UCG−002 − r = −0.0032
Propionic acid
0.20
0.15
0.10
0.05
5
6
7
8
9
abundance (%)
B
B−F
CHE
CHE−F
QUE
QUE−F

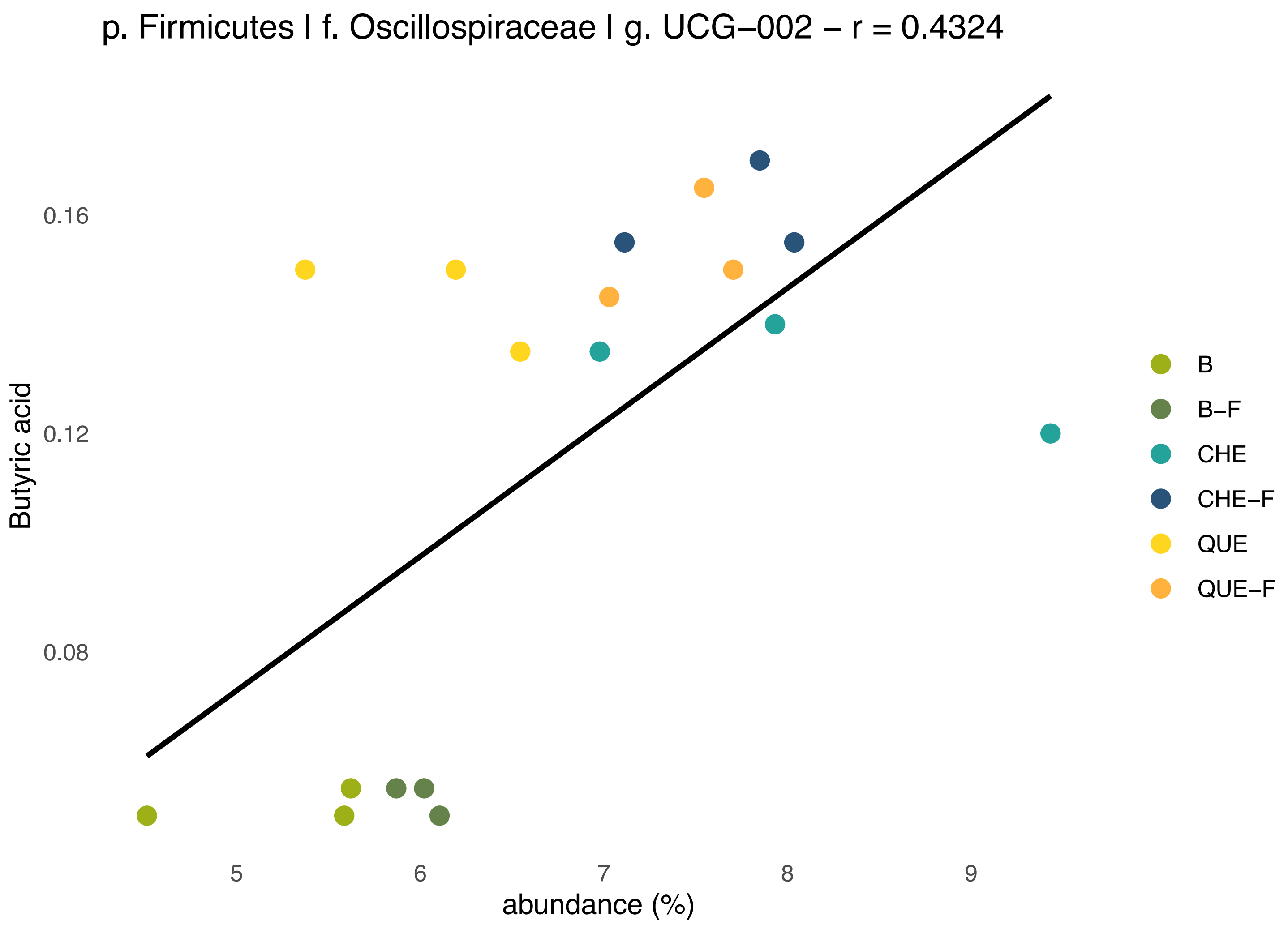

p. Firmicutes l f. Oscillospiraceae l g. UCG−002 − r = 0.4324
Butyric acid
0.16
0.12
0.08
5
6
7
8
9
abundance (%)
B
B−F
CHE
CHE−F
QUE
QUE−F

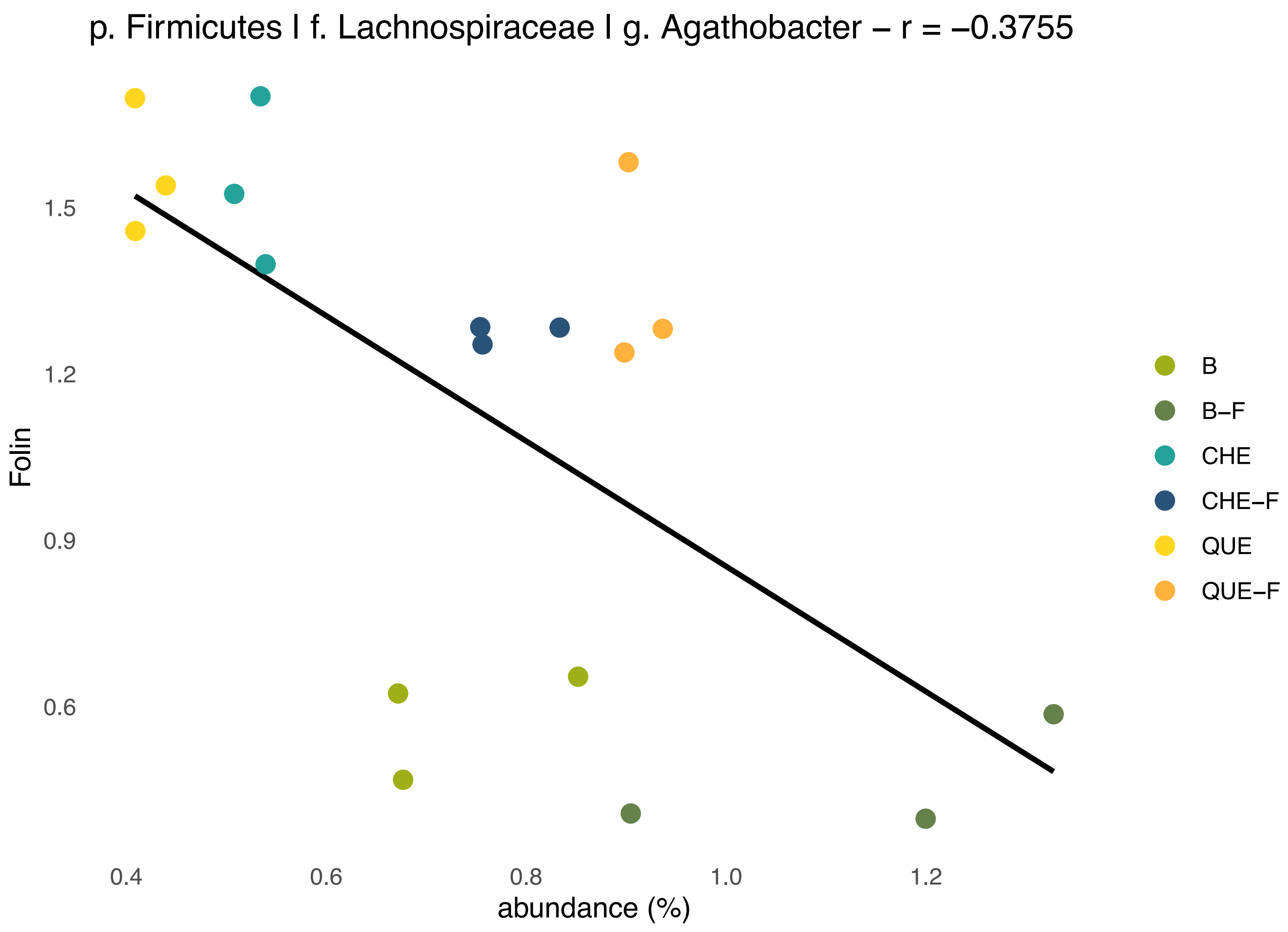
p. Firmicutes | f. Lachnospiraceae | g. Agathobacter – r = –0.3755
Folin
1.5
1.2
0.9
0.6
0.4
0.6
0.8
1.0
1.2
abundance (%)
B
B–F
CHE
CHE–F
QUE
QUE–F

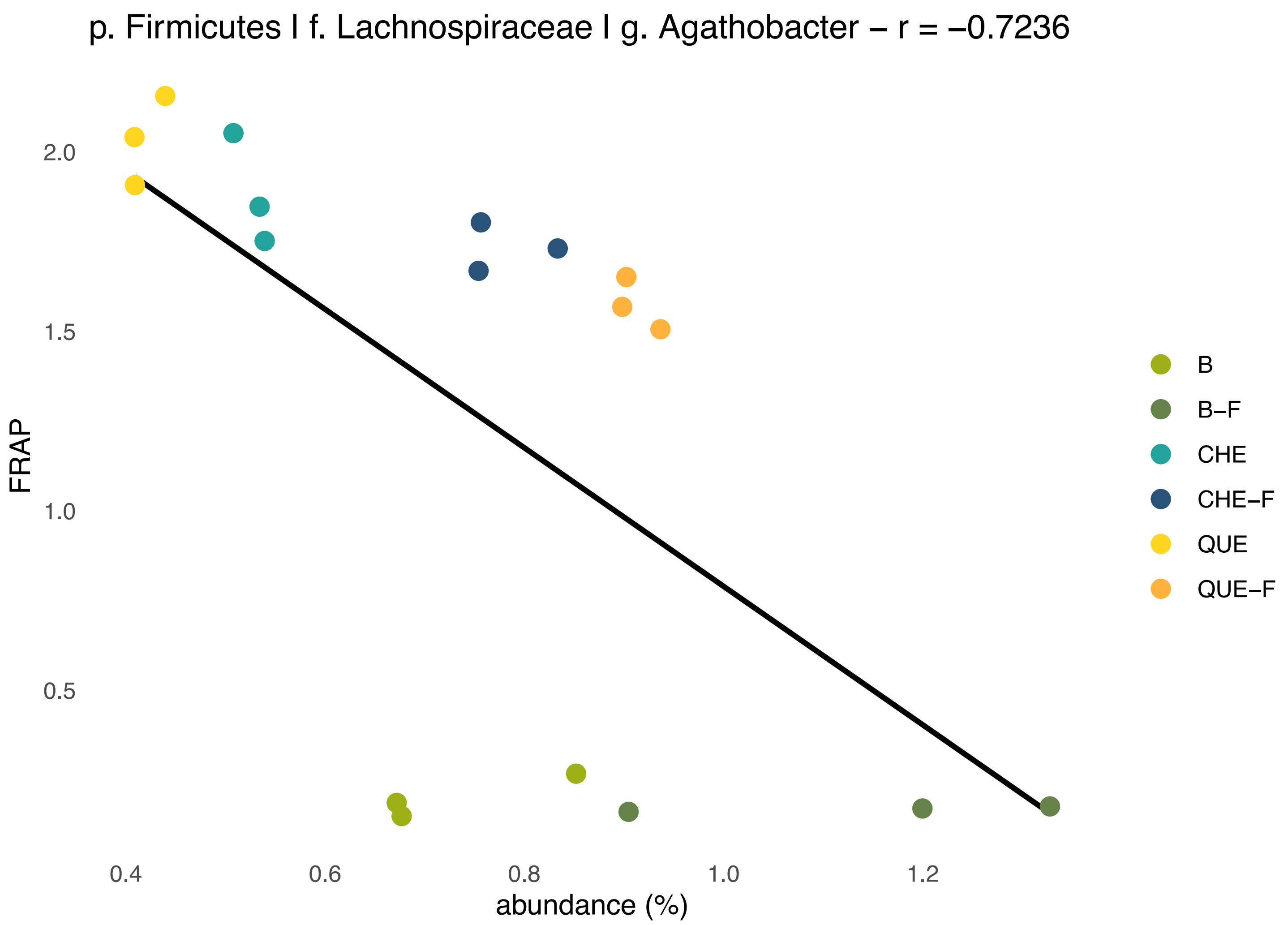
p. Firmicutes I f. Lachnospiraceae I g. Agathobacter – r = –0.7236
FRAP
2.0
1.5
1.0
0.5
0.4
0.6
0.8
1.0
1.2
abundance (%)
B
B–F
CHE
CHE–F
QUE
QUE–F

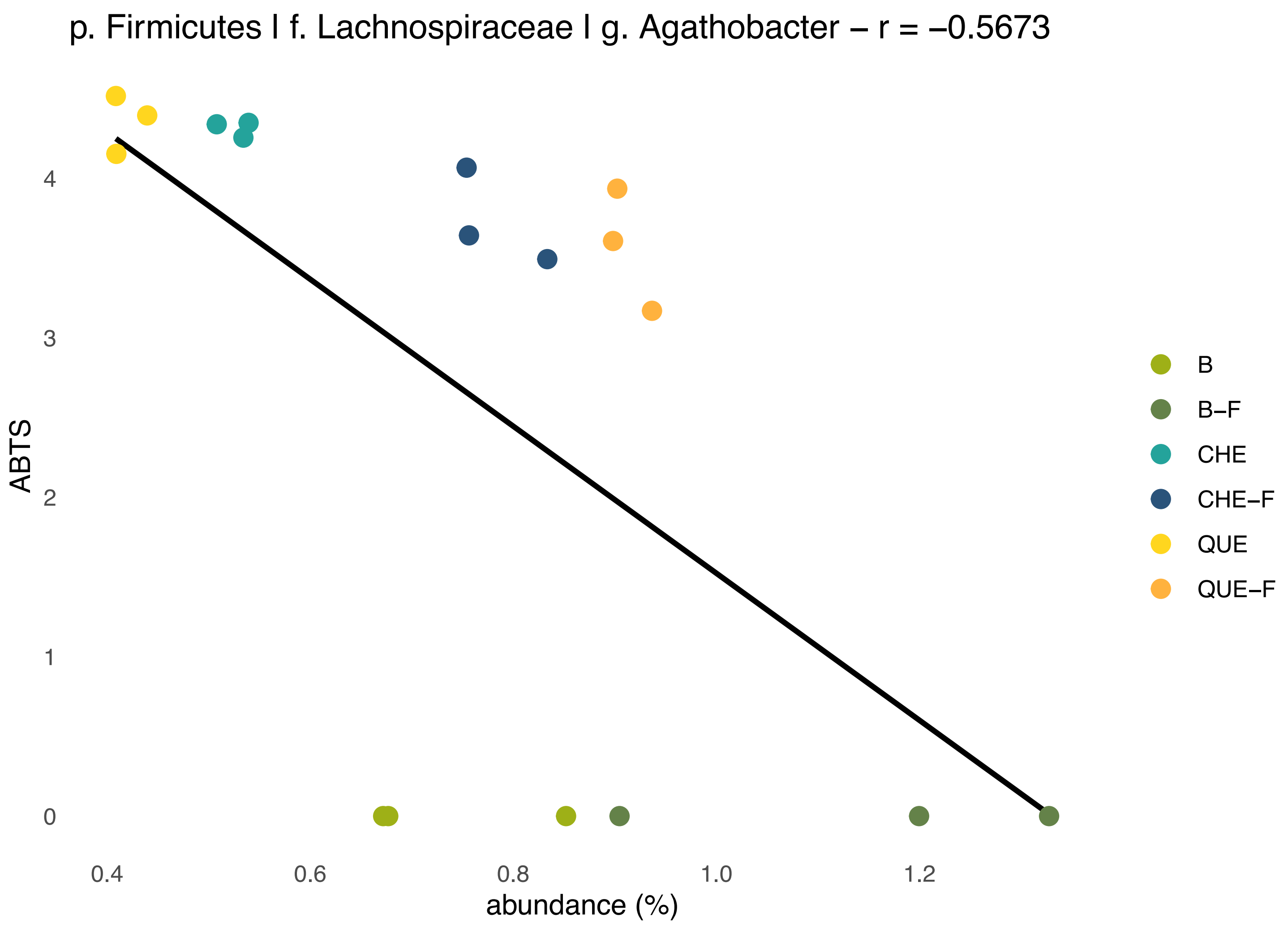

p. Firmicutes I f. Lachnospiraceae I g. Agathobacter – r = –0.5673
ABTS
4
3
2
1
0
0.4
0.6
0.8
1.0
1.2
abundance (%)
B
B–F
CHE
CHE–F
QUE
QUE–F

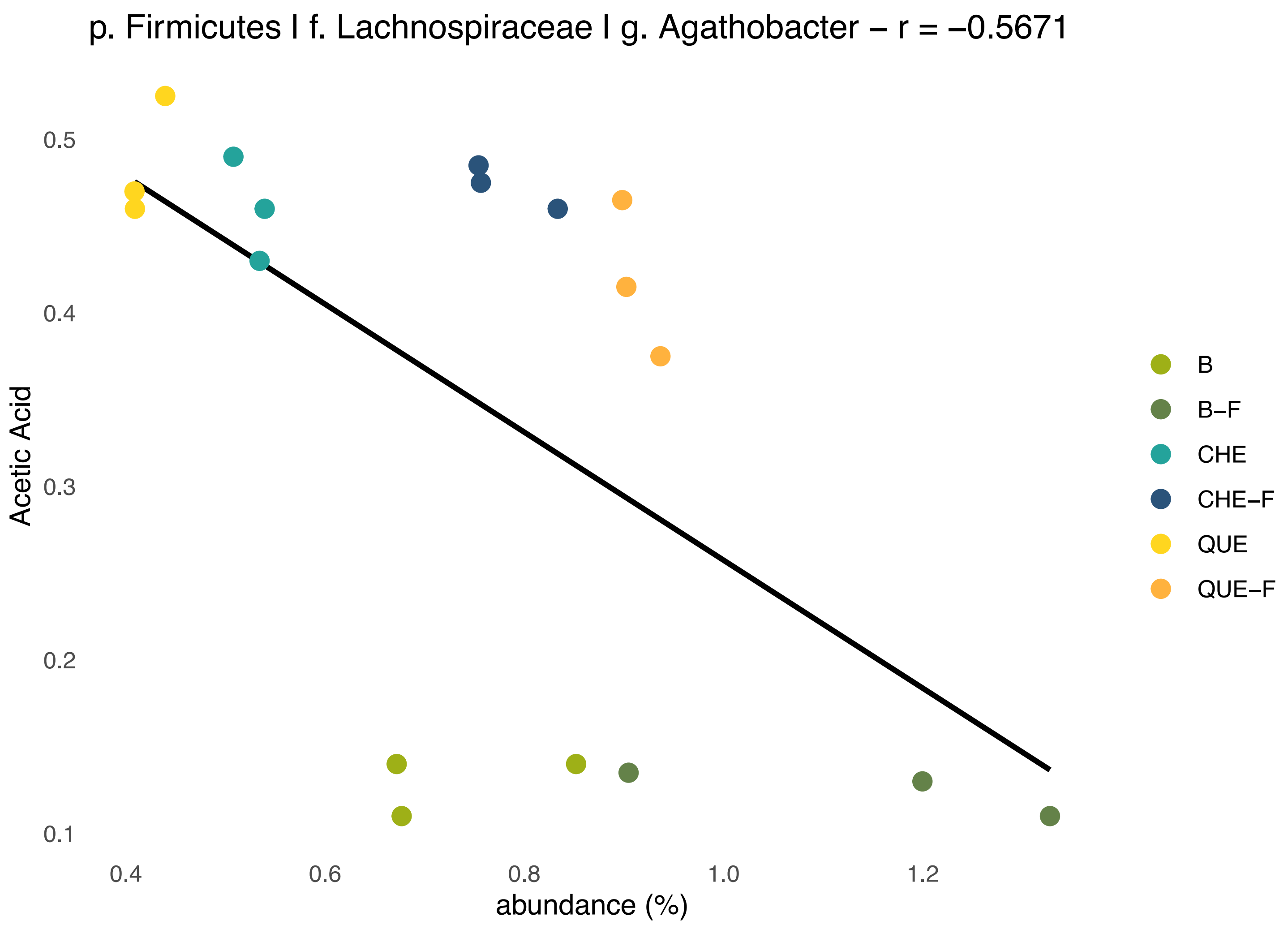

p. Firmicutes | f. Lachnospiraceae | g. Agathobacter – r = –0.5671
Acetic Acid
0.5
0.4
0.3
0.2
0.1
0.4
0.6
0.8
1.0
1.2
abundance (%)
B
B–F
CHE
CHE–F
QUE
QUE–F

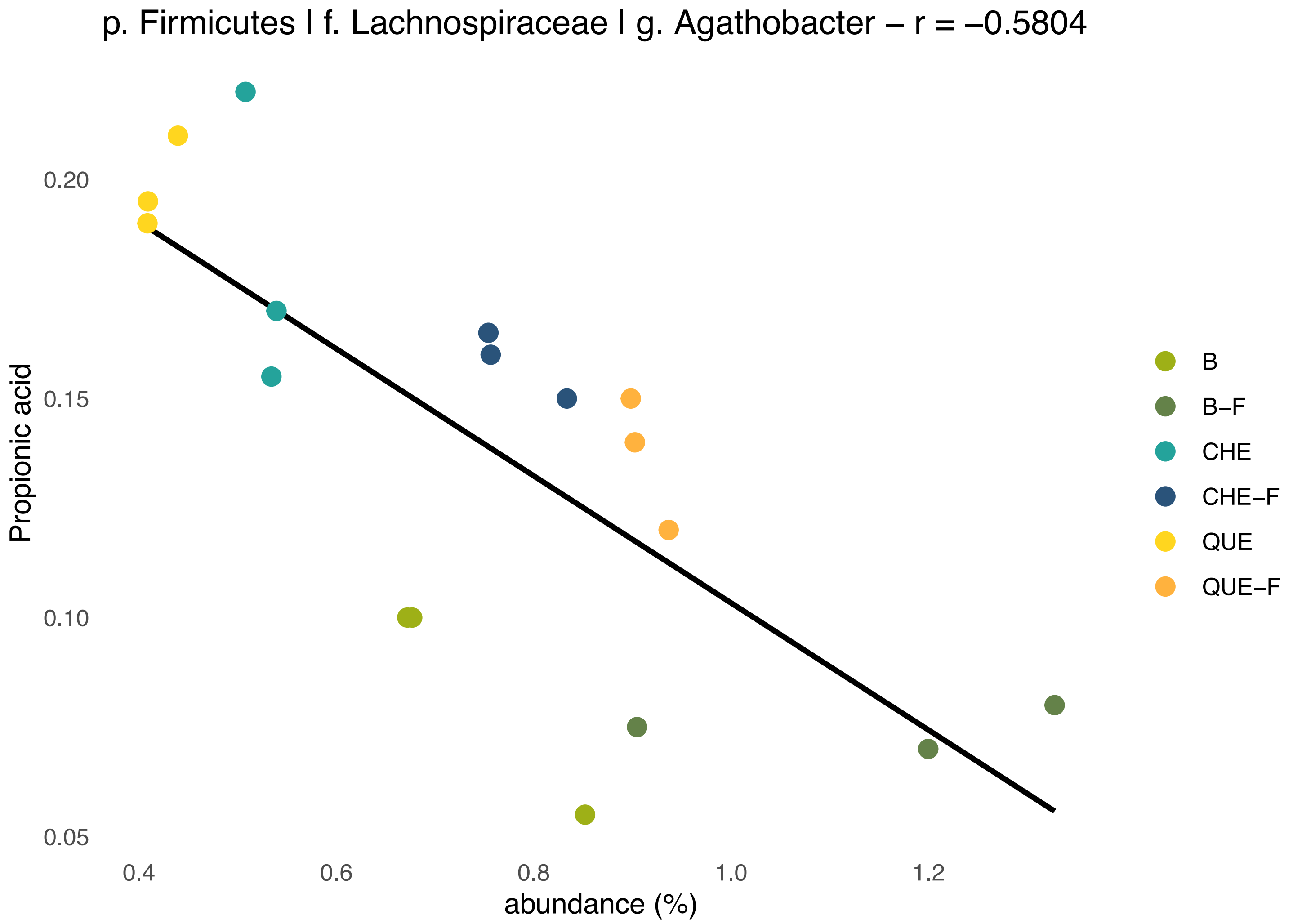
p. Firmicutes I f. Lachnospiraceae I g. Agathobacter – r = –0.5804
Propionic acid
0.20
0.15
0.10
0.05
0.4
0.6
0.8
1.0
1.2
abundance (%)
B
B–F
CHE
CHE–F
QUE
QUE–F

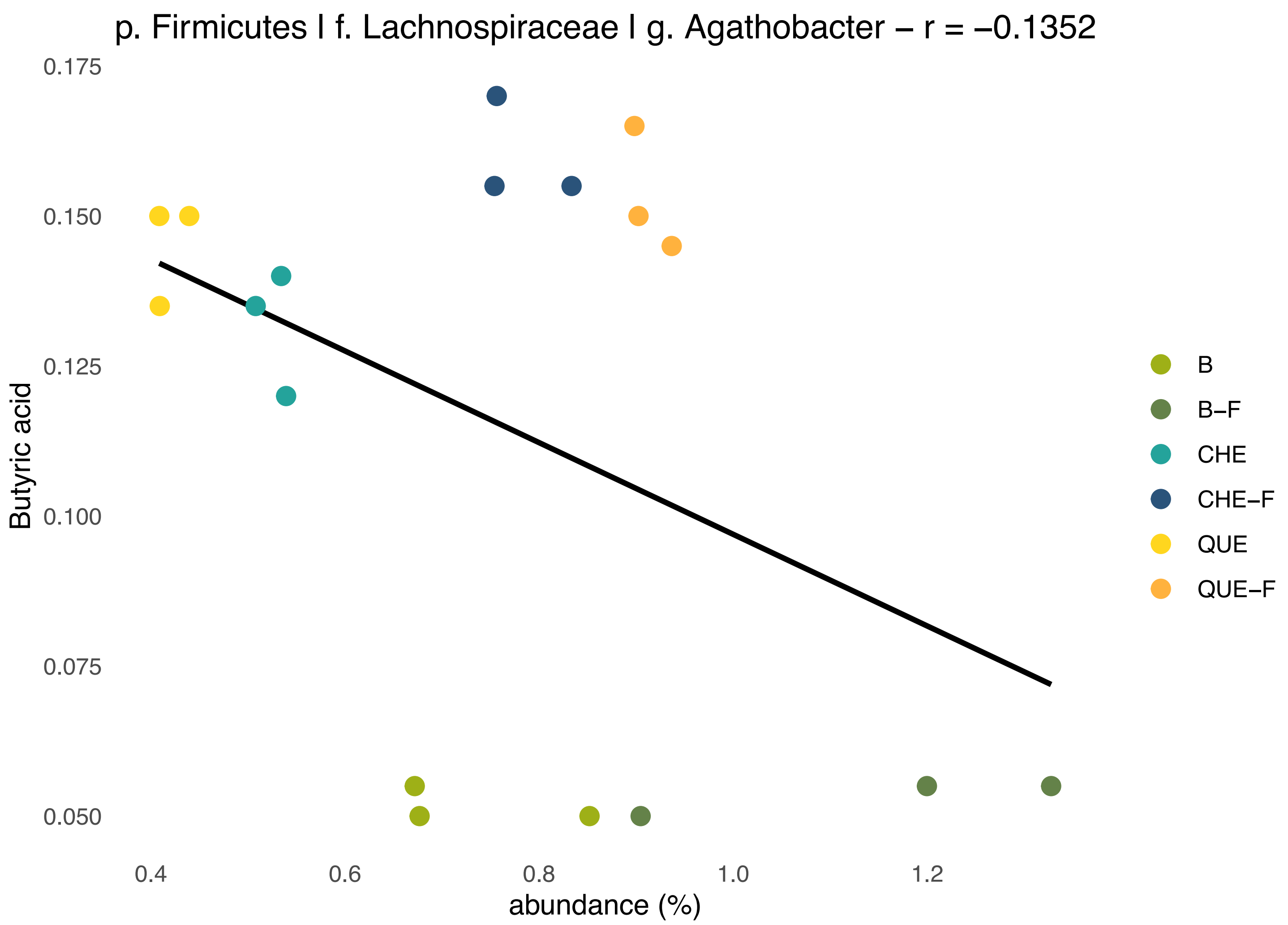

p. Firmicutes l f. Lachnospiraceae l g. Agathobacter – r = –0.1352
0.175
0.150
0.125
0.100
0.075
0.050
Butyric acid
0.4
0.6
0.8
1.0
1.2
abundance (%)
B
B–F
CHE
CHE–F
QUE
QUE–F

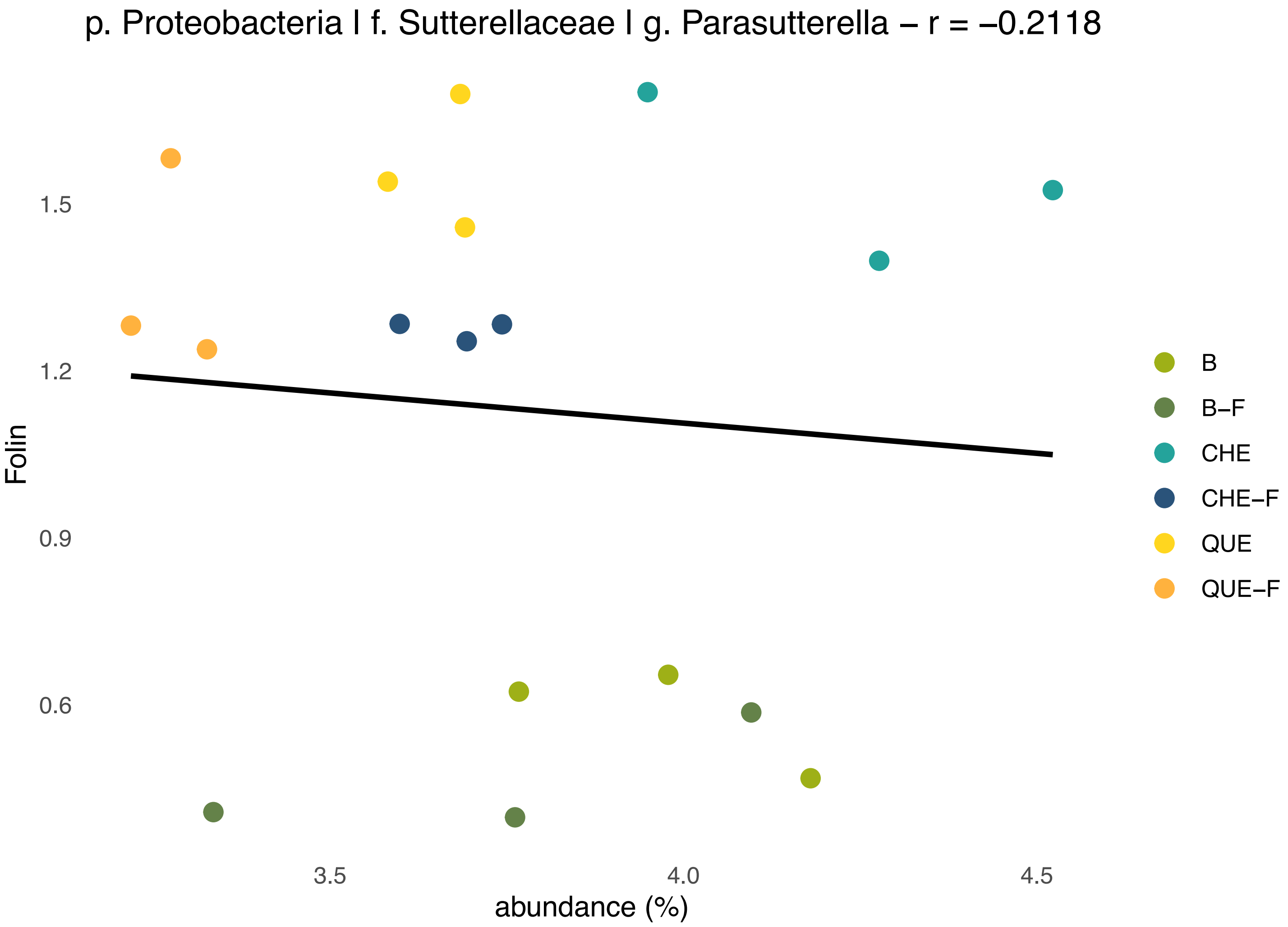
p. Proteobacteria I f. Sutterellaceae I g. Parasutterella – r = −0.2118
1.5
1.2
0.9
0.6
Folin
3.5
4.0
4.5
abundance (%)
B
B−F
CHE
CHE−F
QUE
QUE−F

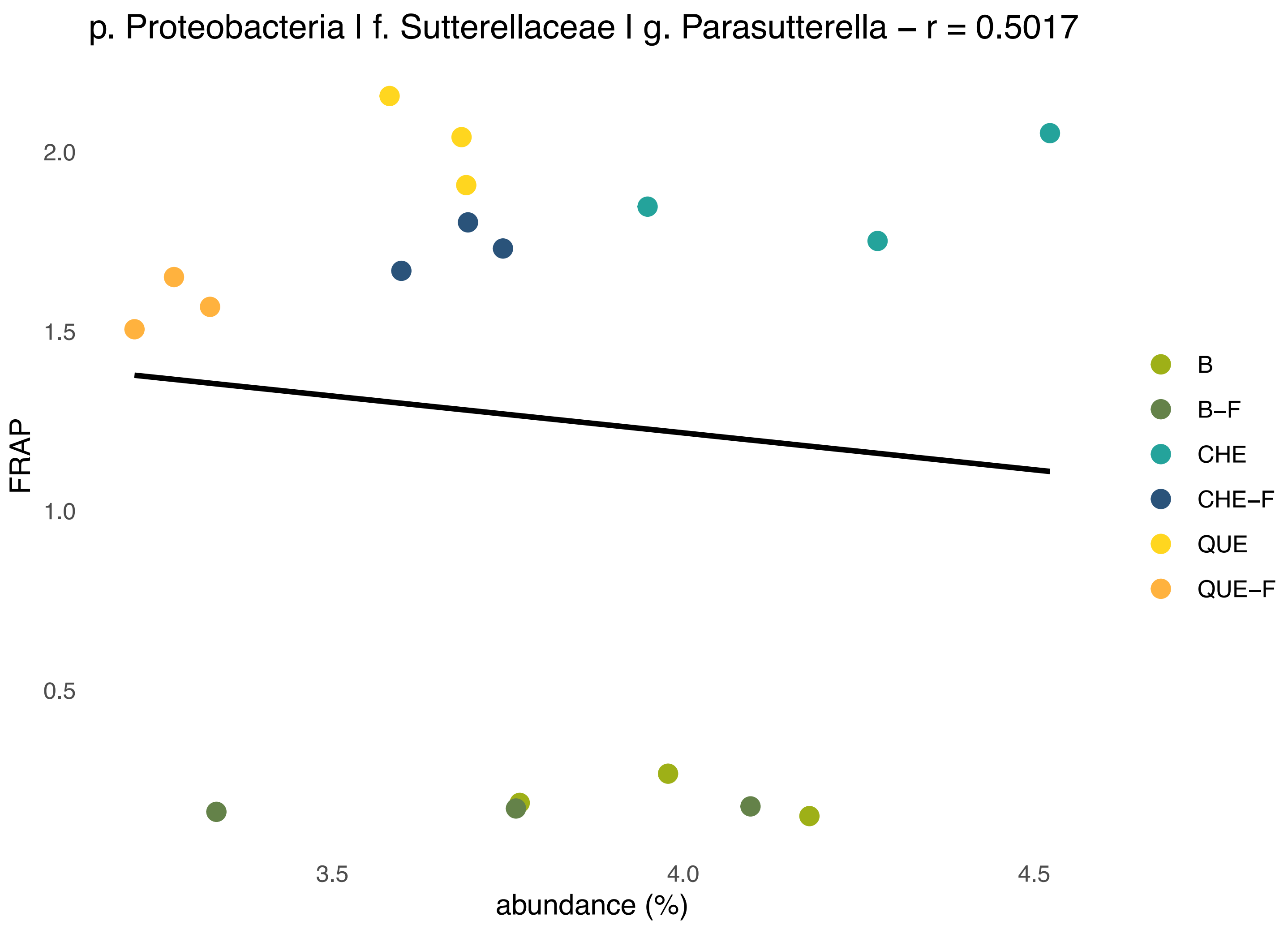

p. Proteobacteria I f. Sutterellaceae I g. Parasutterella – r = 0.5017
FRAP
2.0
1.5
1.0
0.5
3.5
4.0
4.5
abundance (%)
B
B–F
CHE
CHE–F
QUE
QUE–F

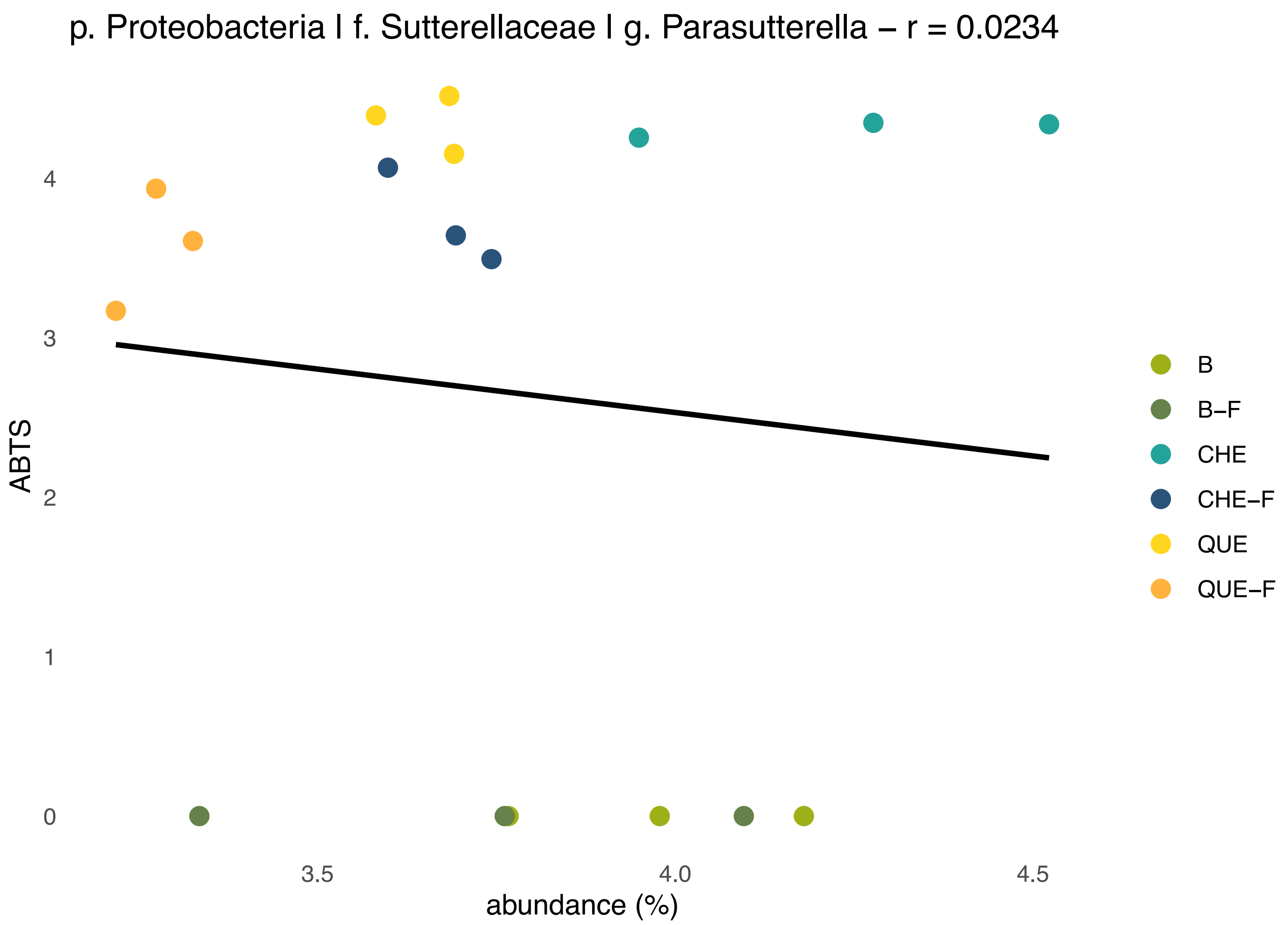

p. Proteobacteria l f. Sutterellaceae l g. Parasutterella – r = 0.0234
ABTS
4
3
2
1
0
3.5
4.0
4.5
abundance (%)
B
B–F
CHE
CHE–F
QUE
QUE–F

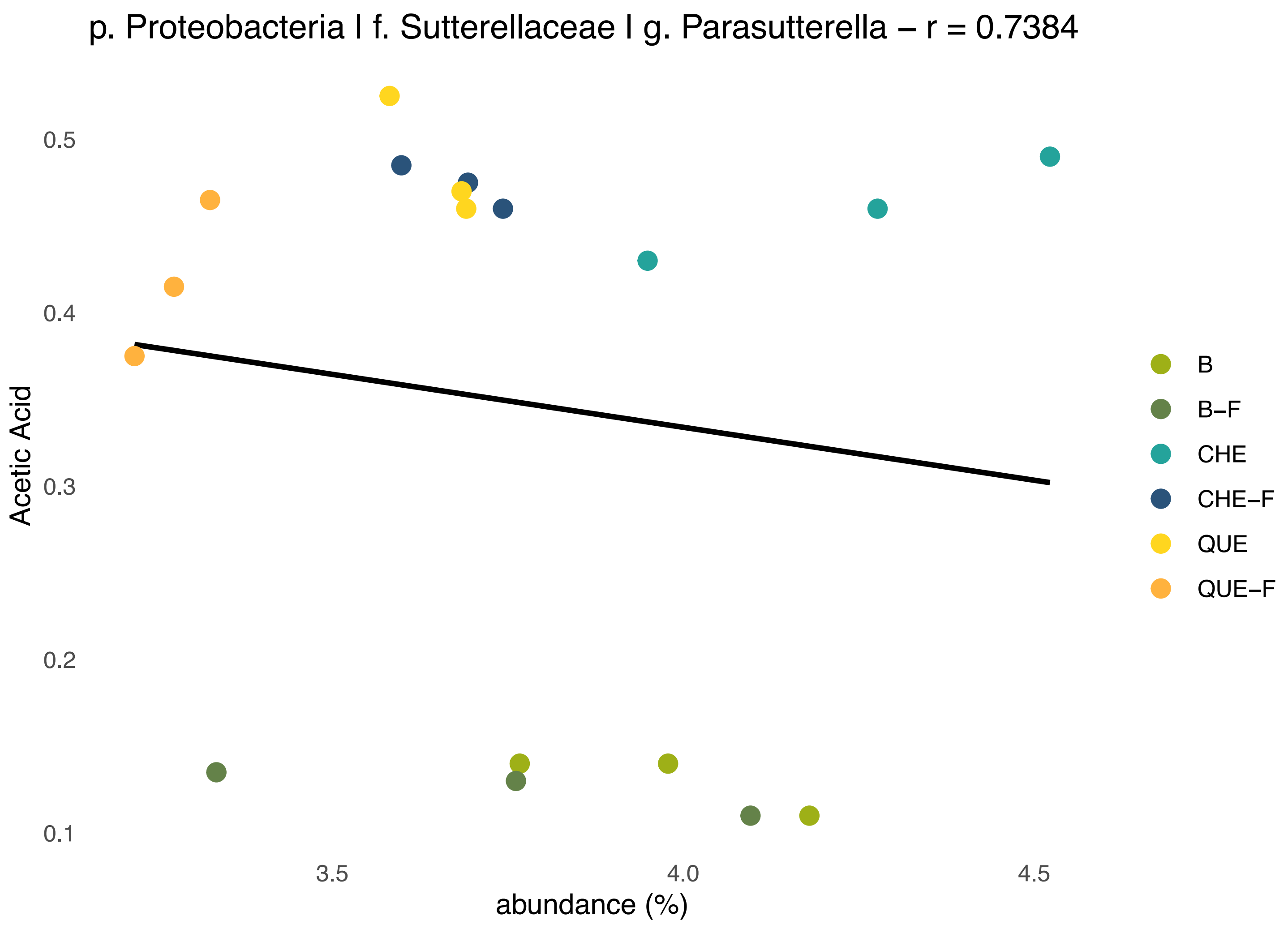

p. Proteobacteria I f. Sutterellaceae I g. Parasutterella – r = 0.7384
Acetic Acid
0.5
0.4
0.3
0.2
0.1
3.5
4.0
4.5
abundance (%)
B
B–F
CHE
CHE–F
QUE
QUE–F

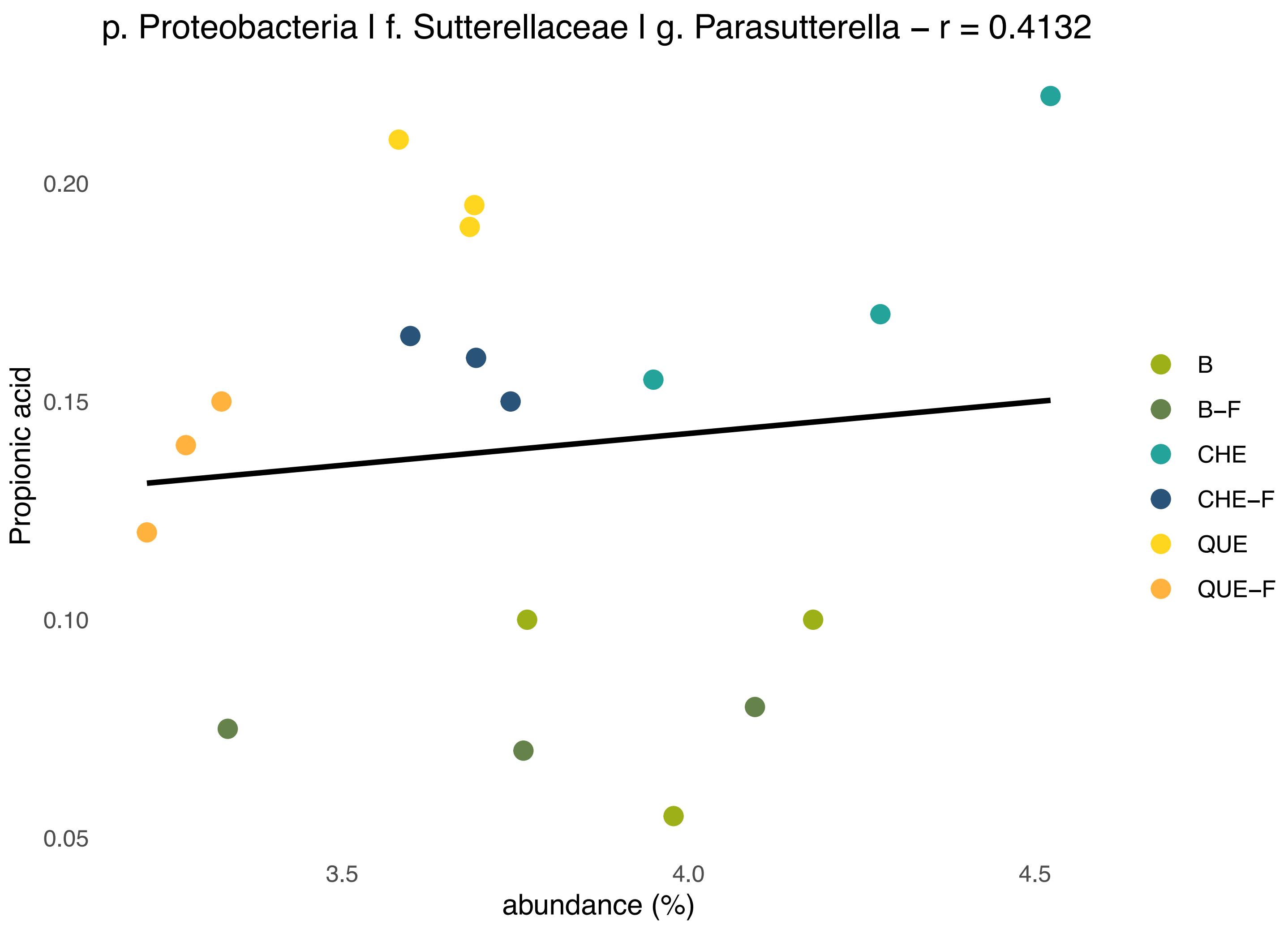

p. Proteobacteria I f. Sutterellaceae I g. Parasutterella – r = 0.4132
Propionic acid
0.20
0.15
0.10
0.05
3.5
4.0
4.5
abundance (%)
B
B–F
CHE
CHE–F
QUE
QUE–F

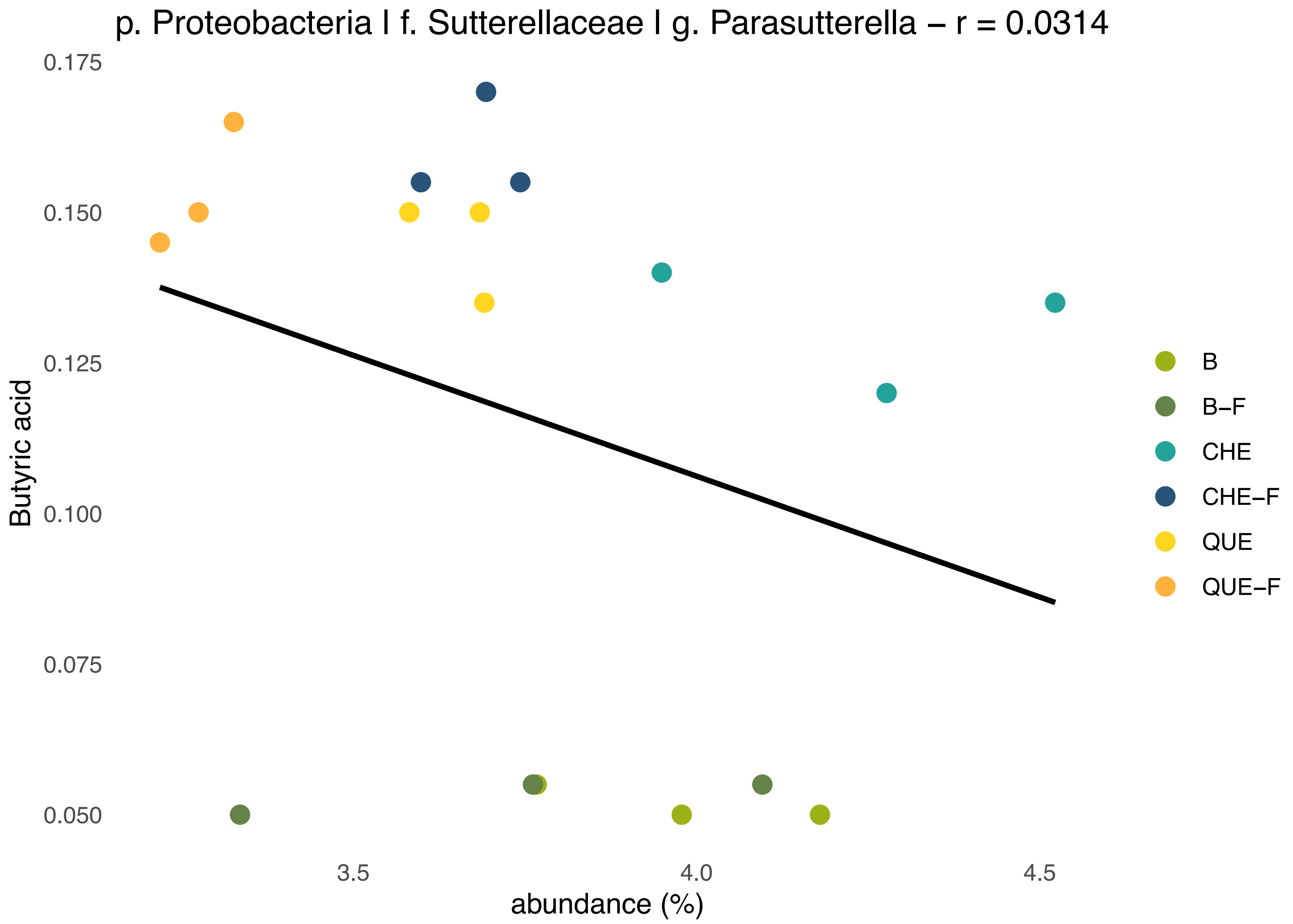
p. Proteobacteria l f. Sutterellaceae l g. Parasutterella – r = 0.0314
0.175
0.150
0.125
0.100
0.075
0.050
Butyric acid
3.5
4.0
4.5
abundance (%)
B
B–F
CHE
CHE–F
QUE
QUE–F

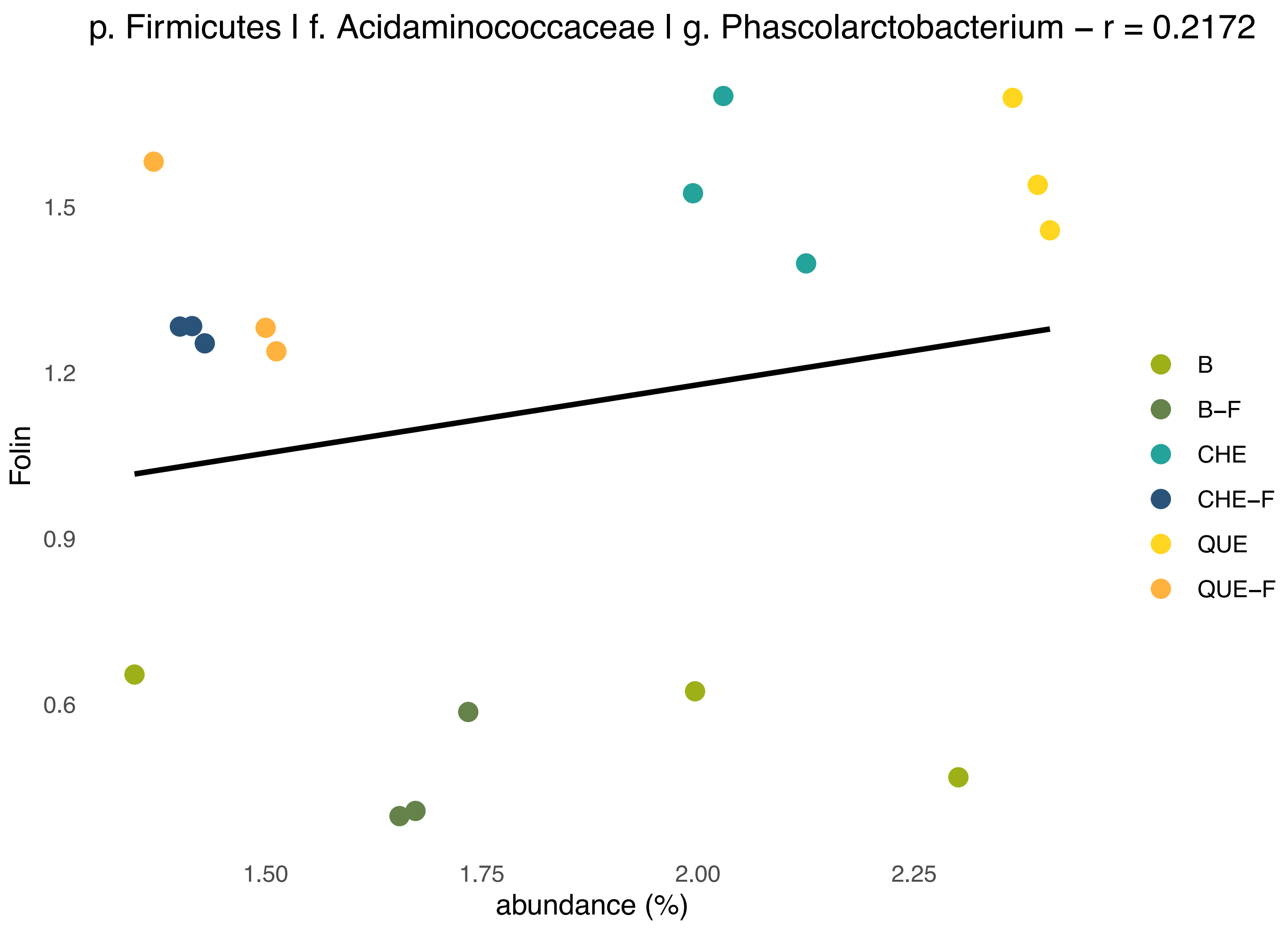

p. Firmicutes I f. Acidaminococcaceae I g. Phascolarctobacterium – r = 0.2172
Folin
1.5
1.2
0.9
0.6
1.50
1.75
2.00
2.25
abundance (%)
B
B–F
CHE
CHE–F
QUE
QUE–F

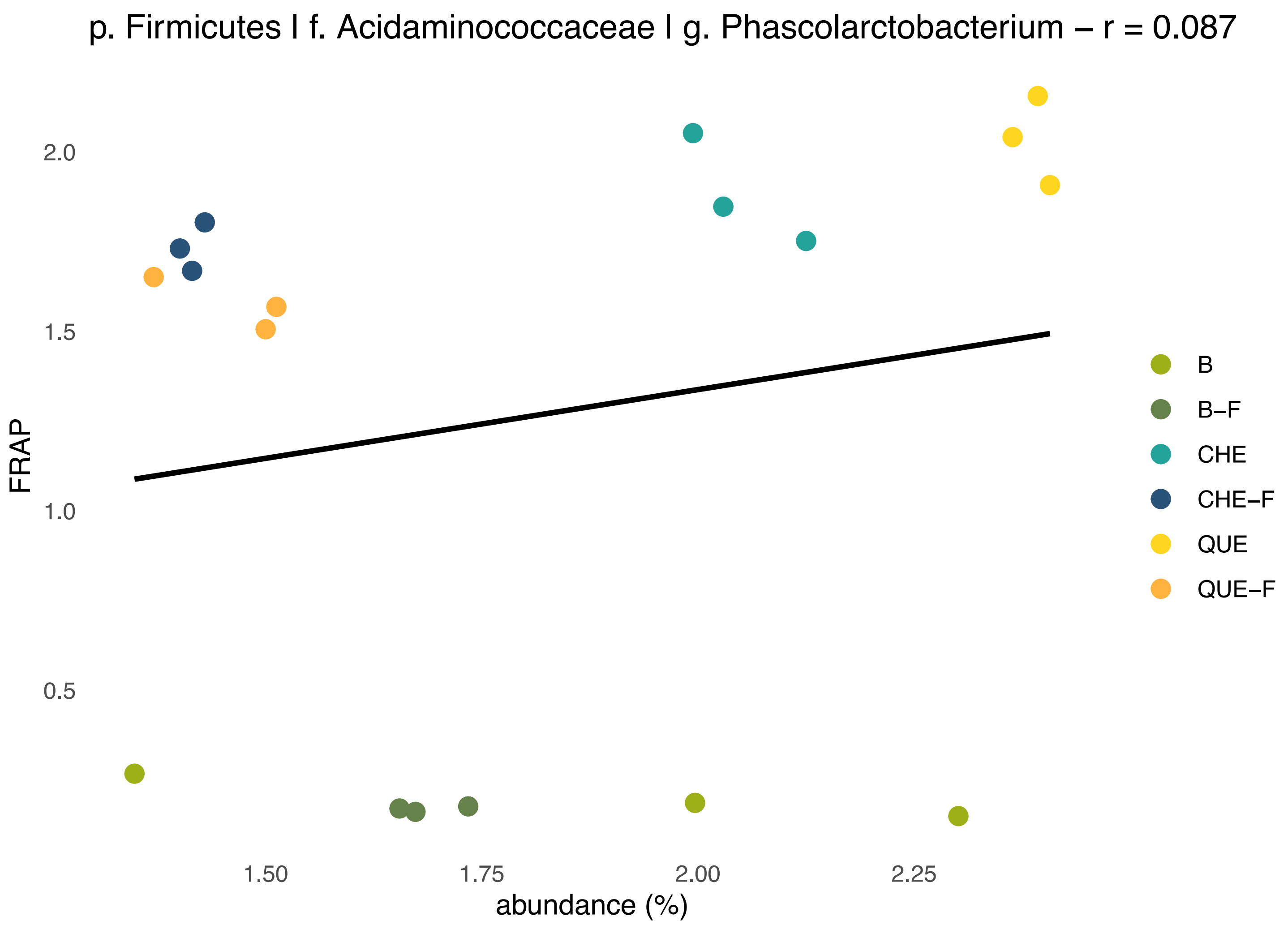
p. Firmicutes I f. Acidaminococcaceae I g. Phascolarctobacterium – r = 0.087
FRAP
2.0
1.5
1.0
0.5
1.50
1.75
2.00
2.25
abundance (%)
B
B–F
CHE
CHE–F
QUE
QUE–F

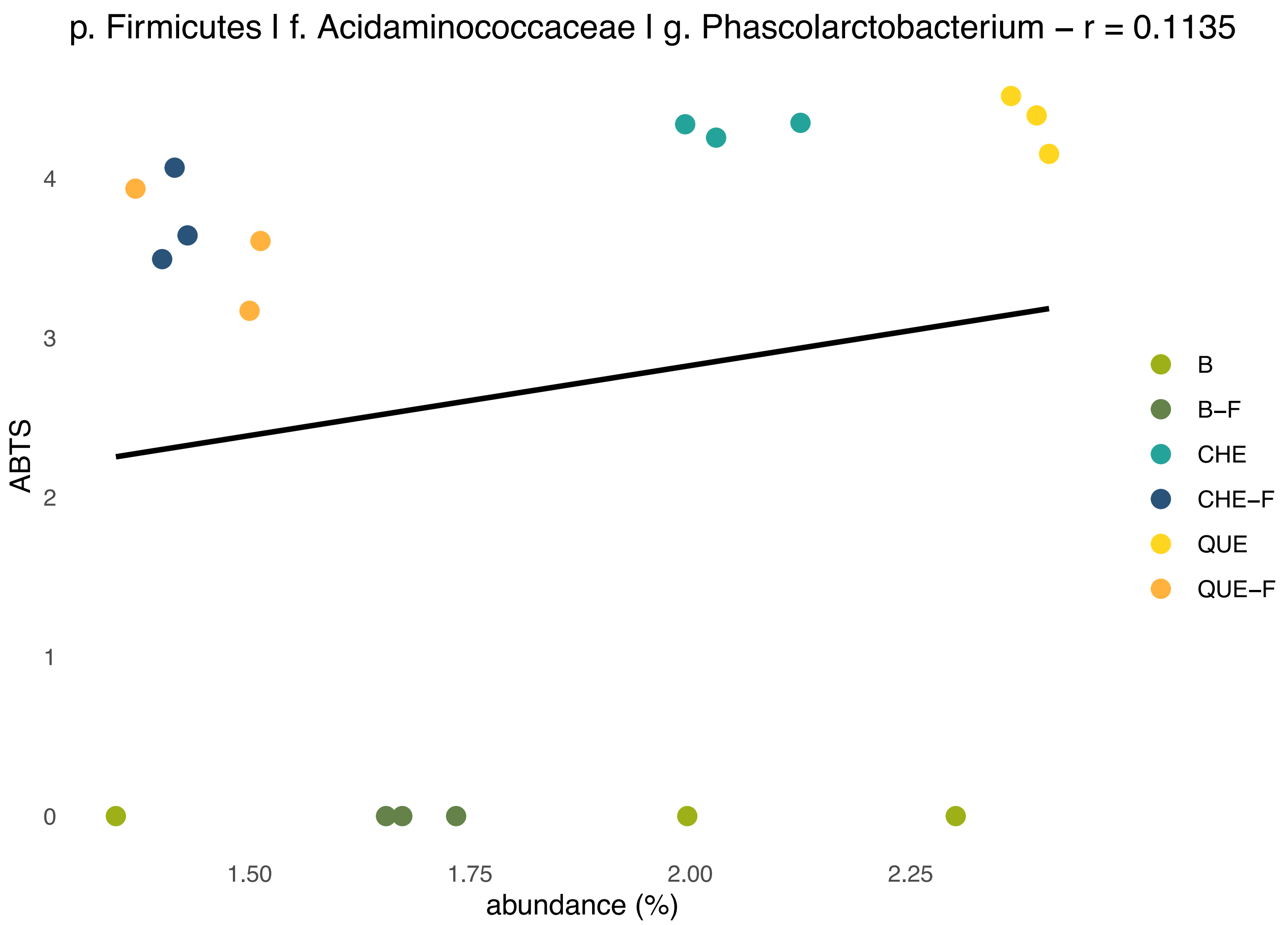

p. Firmicutes l f. Acidaminococcaceae l g. Phascolarctobacterium – r = 0.1135
ABTS
4
3
2
1
0
1.50
1.75
2.00
2.25
abundance (%)
B
B–F
CHE
CHE–F
QUE
QUE–F

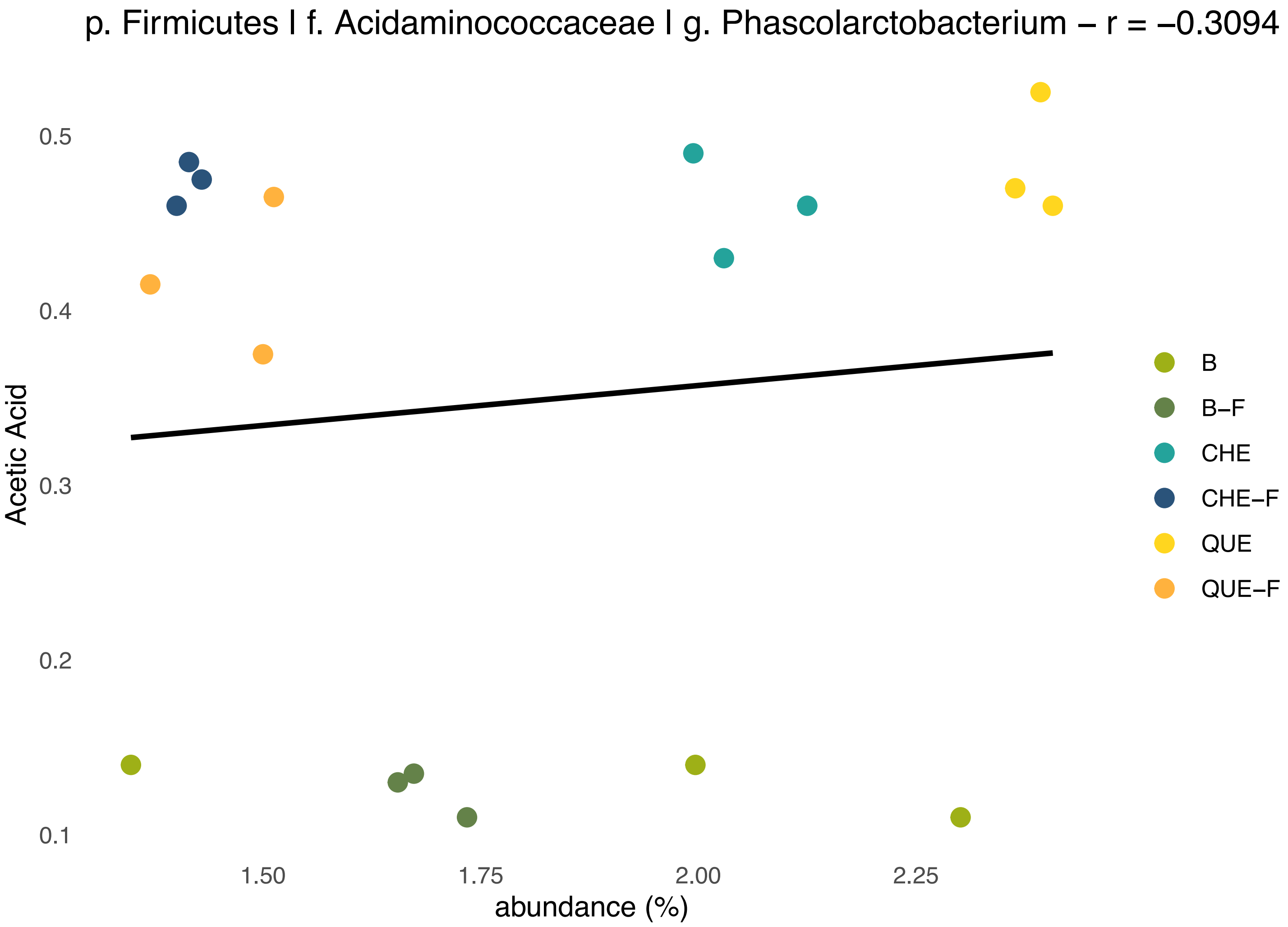
p. Firmicutes I f. Acidaminococcaceae I g. Phascolarctobacterium – r = –0.3094
Acetic Acid
0.5
0.4
0.3
0.2
0.1
1.50
1.75
2.00
2.25
abundance (%)
B
B–F
CHE
CHE–F
QUE
QUE–F

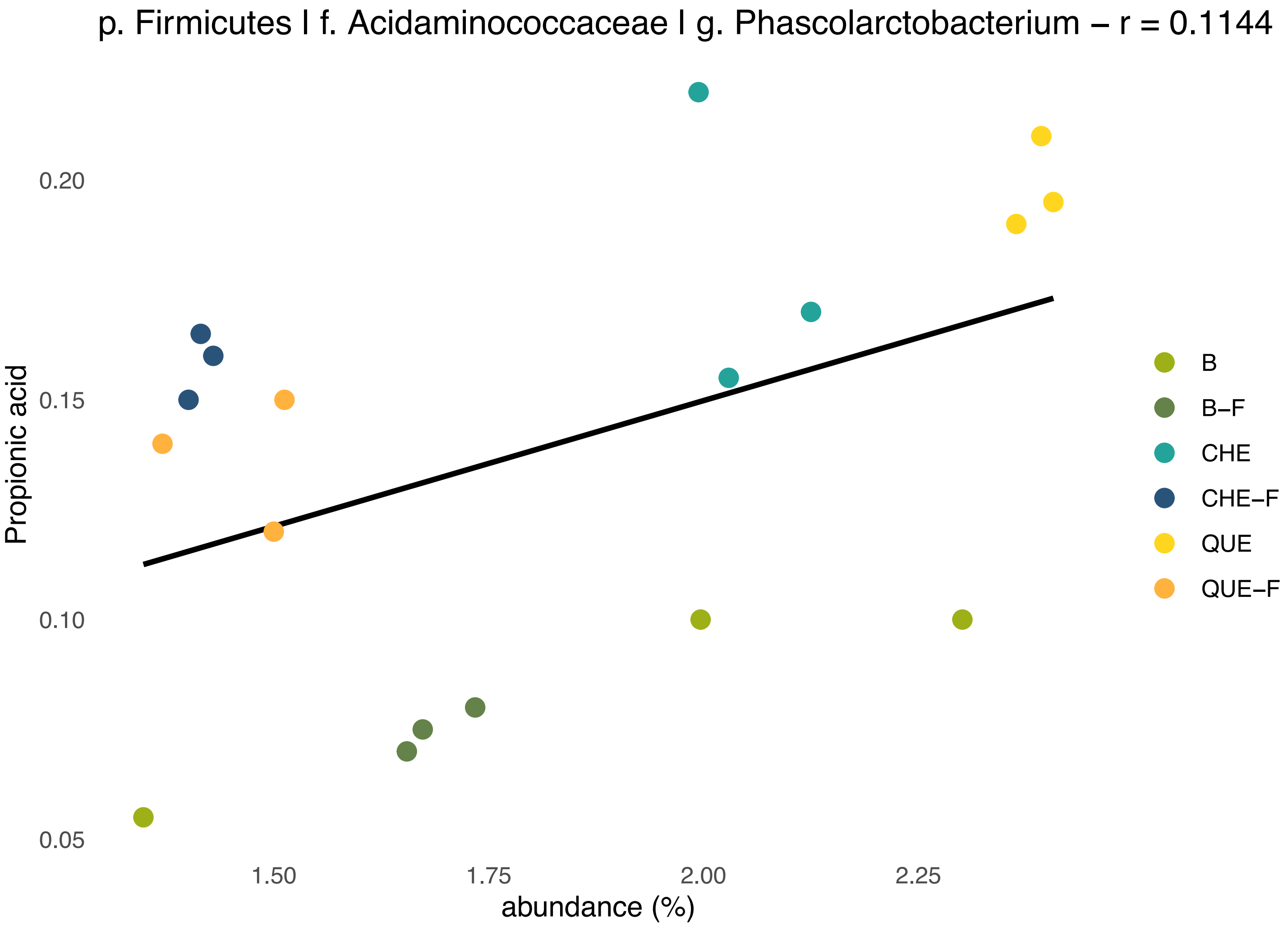

p. Firmicutes I f. Acidaminococcaceae I g. Phascolarctobacterium – r = 0.1144
Propionic acid
0.20
0.15
0.10
0.05
1.50
1.75
2.00
2.25
abundance (%)
B
B–F
CHE
CHE–F
QUE
QUE–F

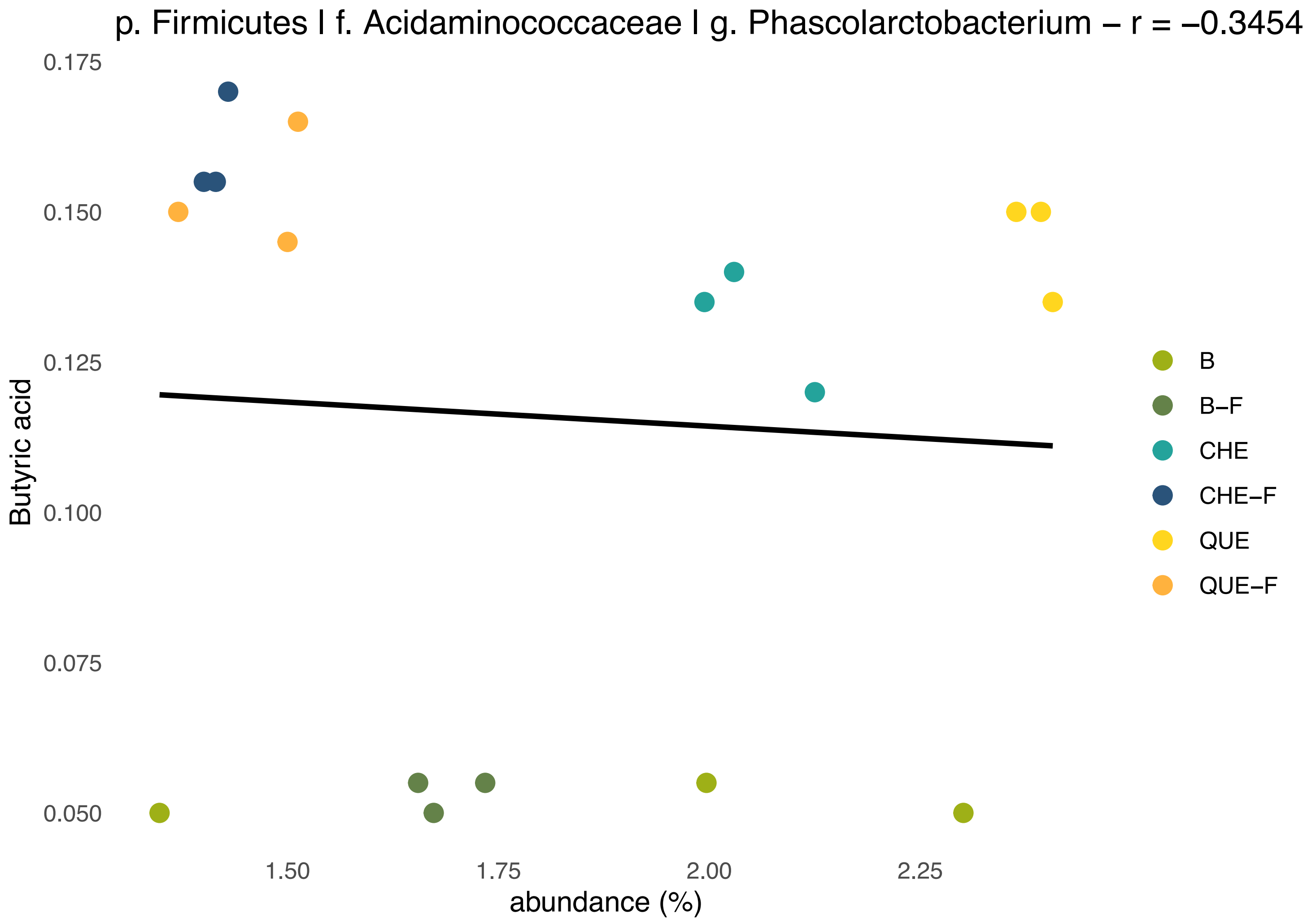
p. Firmicutes I f. Acidaminococcaceae I g. Phascolarctobacterium – r = –0.3454
Butyric acid
0.175
0.150
0.125
0.100
0.075
0.050
abundance (%)
1.50
1.75
2.00
2.25
B
B–F
CHE
CHE–F
QUE
QUE–F

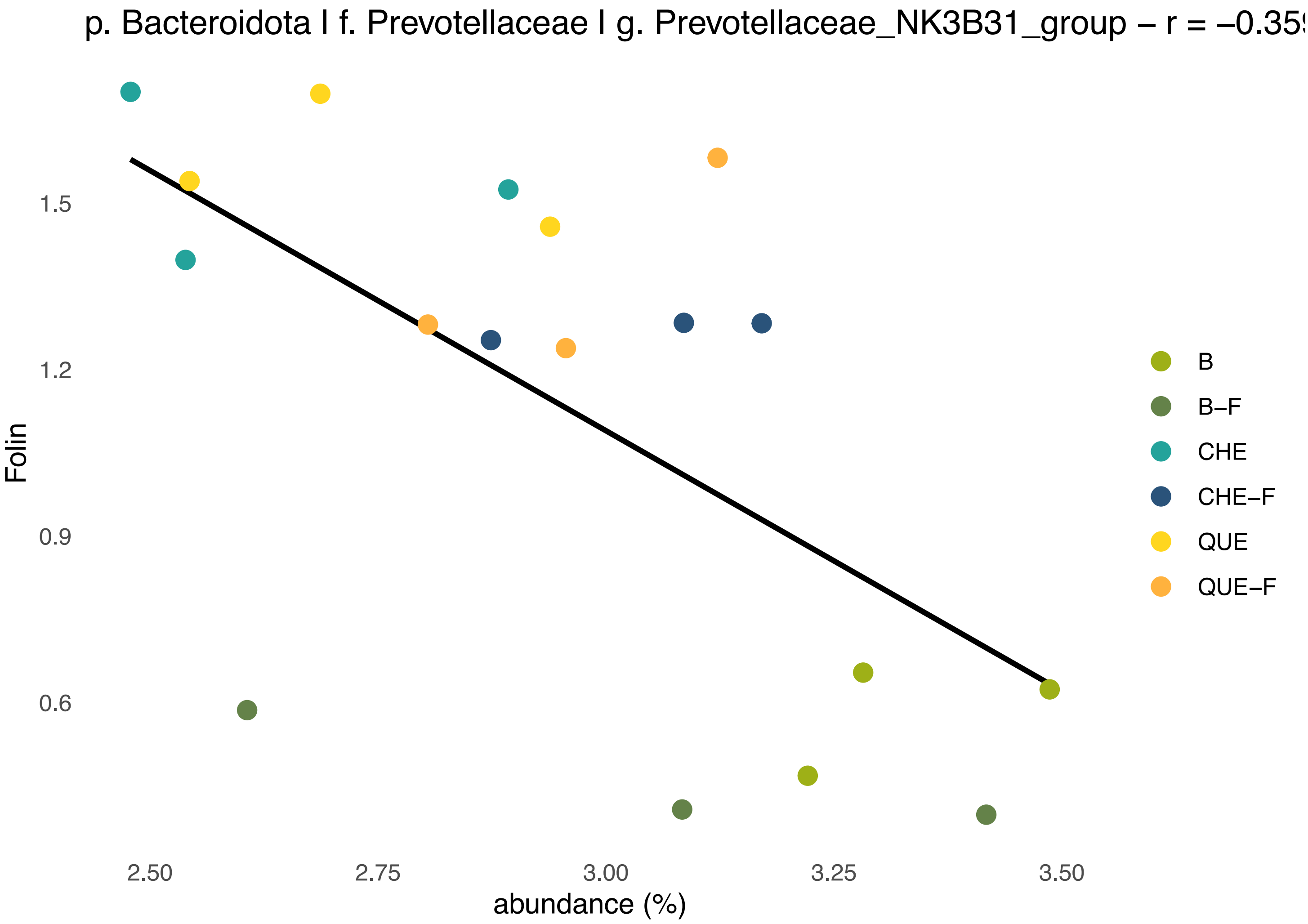

p. Bacteroidota I f. Prevotellaceae I g. Prevotellaceae_NK3B31_group – r = –0.35
Folin
1.5
1.2
0.9
0.6
2.50
2.75
3.00
3.25
3.50
abundance (%)
B
B–F
CHE
CHE–F
QUE
QUE–F

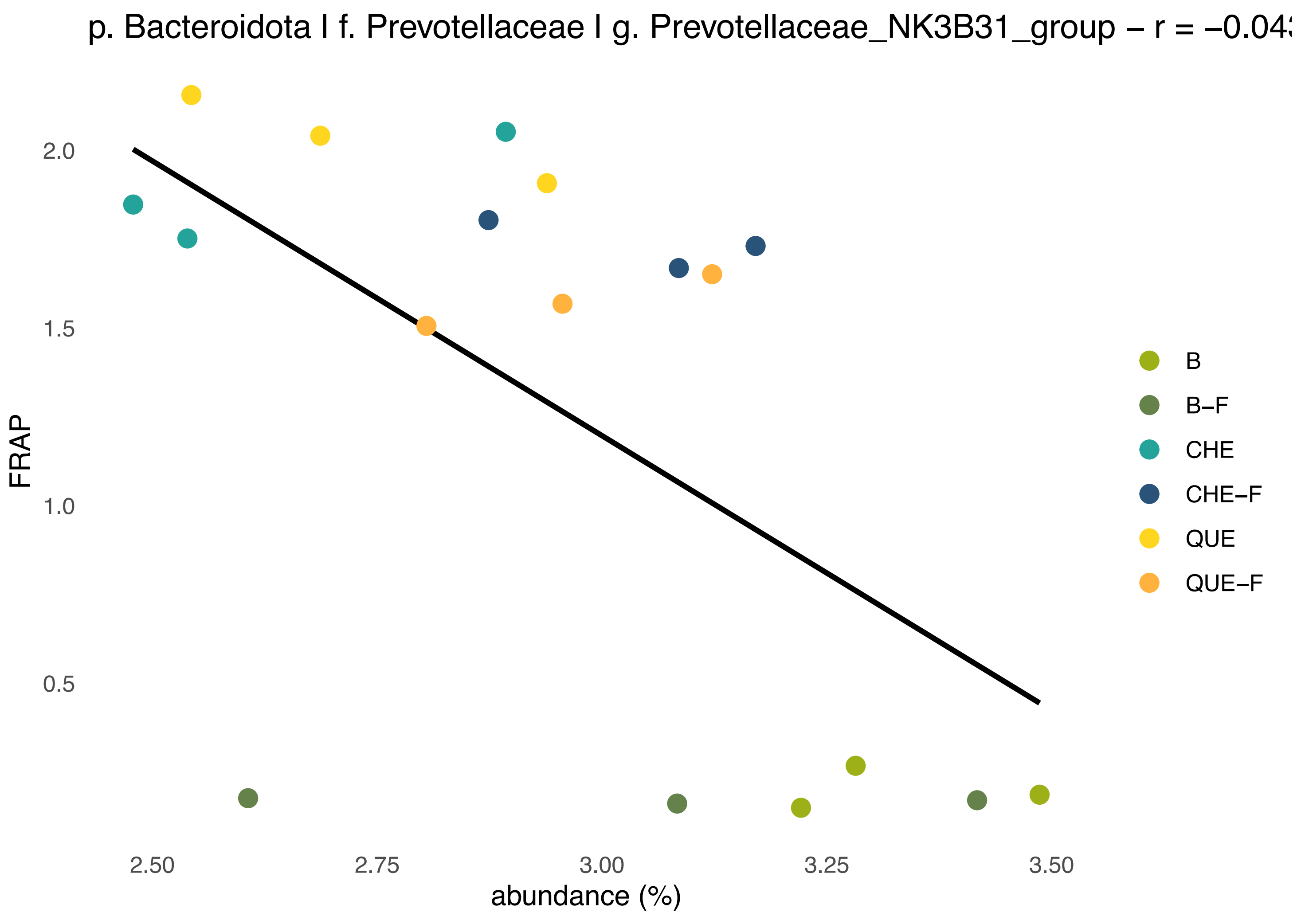

p. Bacteroidota I f. Prevotellaceae I g. Prevotellaceae_NK3B31_group – r = –0.04
FRAP
2.0
1.5
1.0
0.5
2.50
2.75
3.00
3.25
3.50
abundance (%)
B
B–F
CHE
CHE–F
QUE
QUE–F

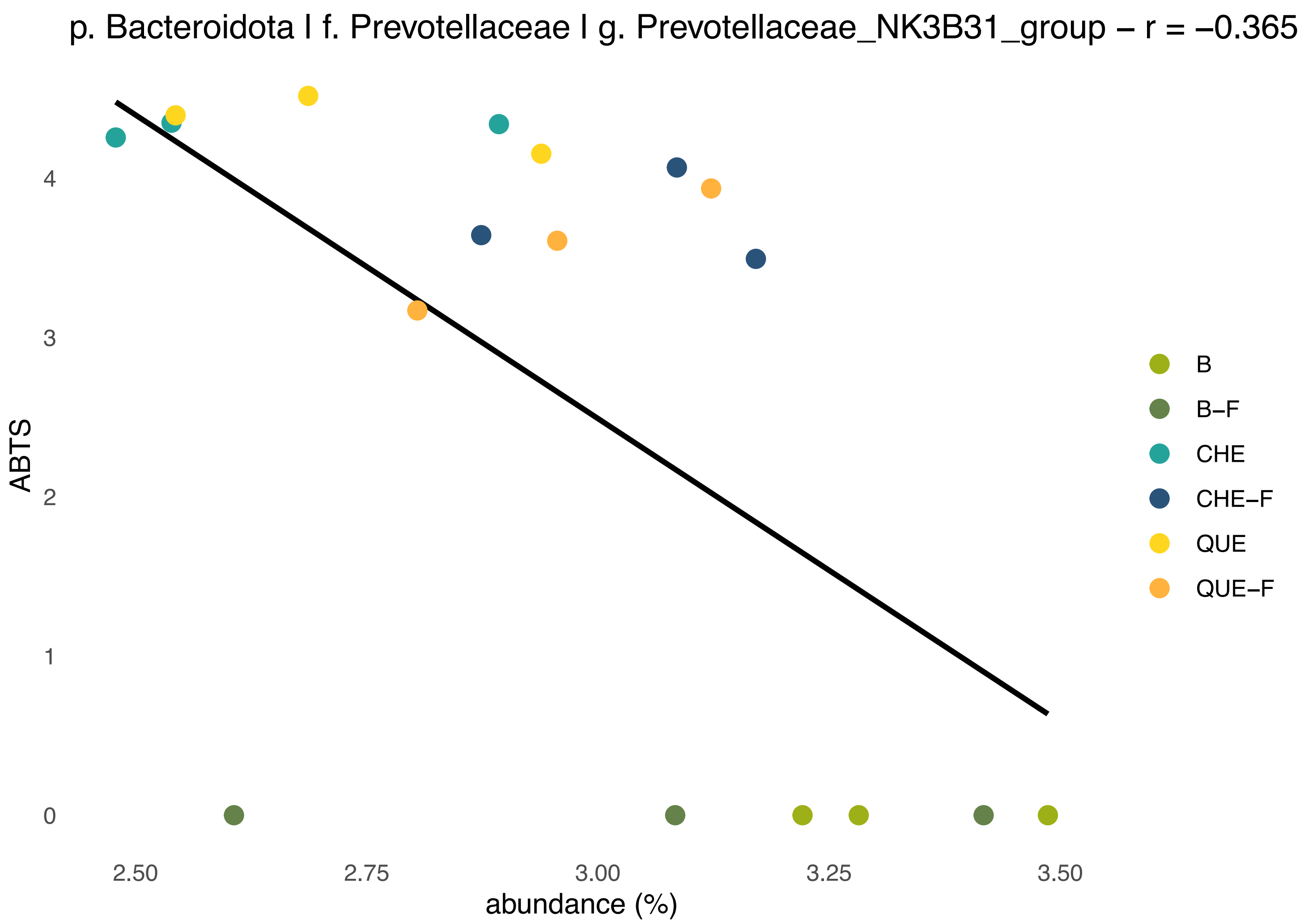

p. Bacteroidota I f. Prevotellaceae I g. Prevotellaceae_NK3B31_group – r = –0.365
ABTS
0
1
2
3
4
2.50
2.75
3.00
3.25
3.50
abundance (%)
B
B–F
CHE
CHE–F
QUE
QUE–F

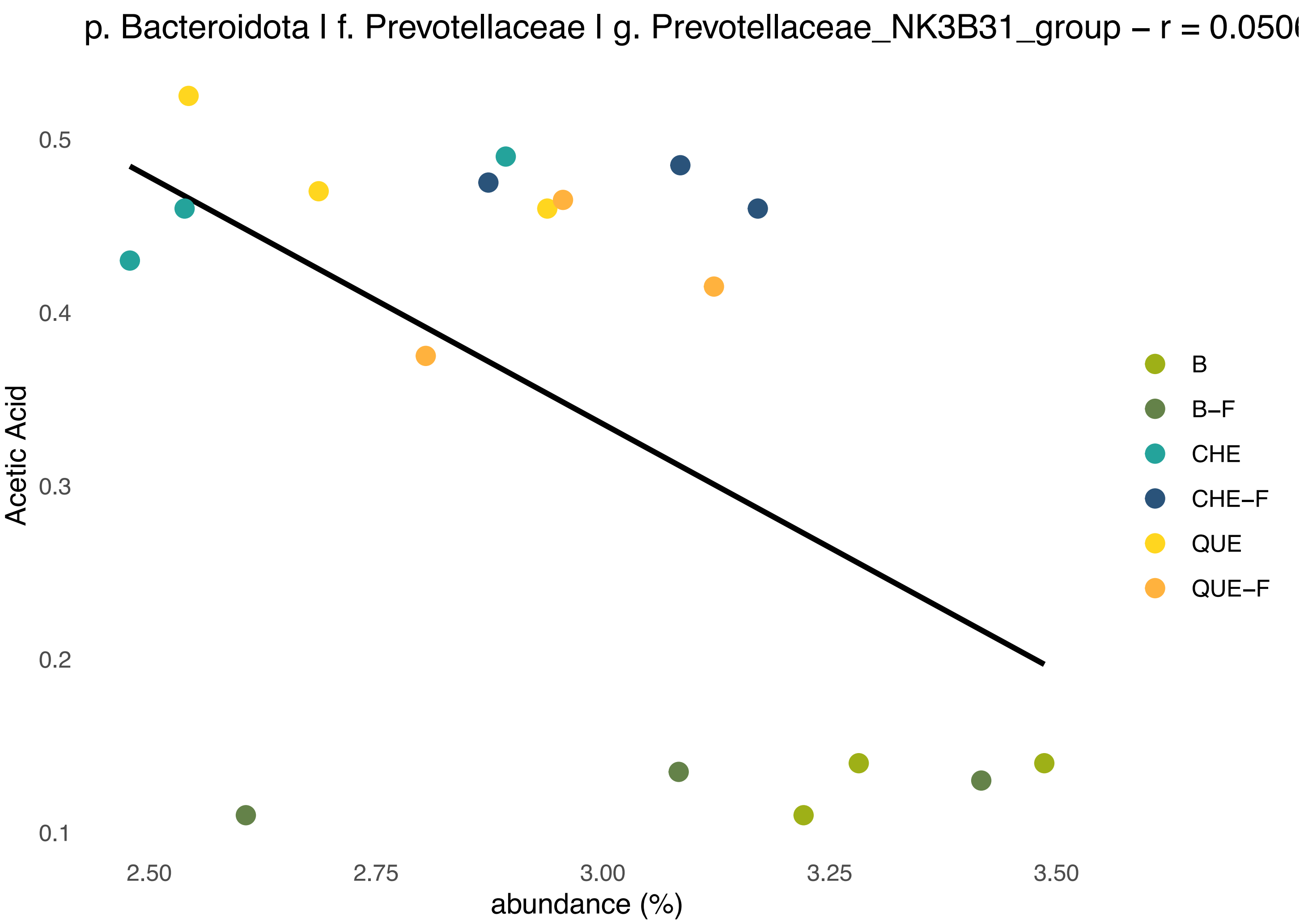
p. Bacteroidota | f. Prevotellaceae | g. Prevotellaceae_NK3B31_group – r = 0.050
Acetic Acid
0.5
0.4
0.3
0.2
0.1
2.50
2.75
3.00
3.25
3.50
abundance (%)
B
B–F
CHE
CHE–F
QUE
QUE–F

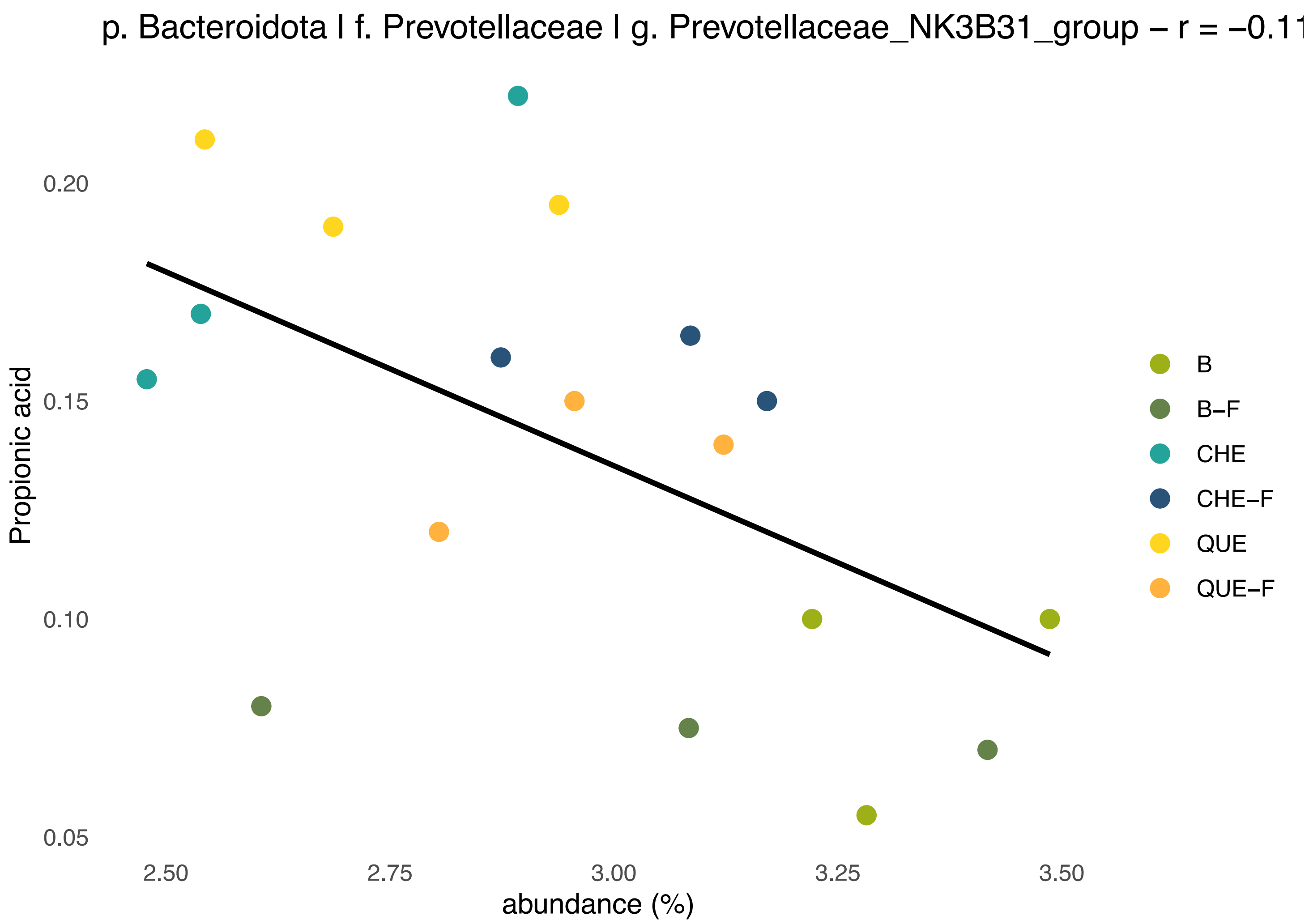
p. Bacteroidota I f. Prevotellaceae I g. Prevotellaceae_NK3B31_group – r = –0.11
Propionic acid
0.20
0.15
0.10
0.05
2.50
2.75
3.00
3.25
3.50
abundance (%)
B
B–F
CHE
CHE–F
QUE
QUE–F

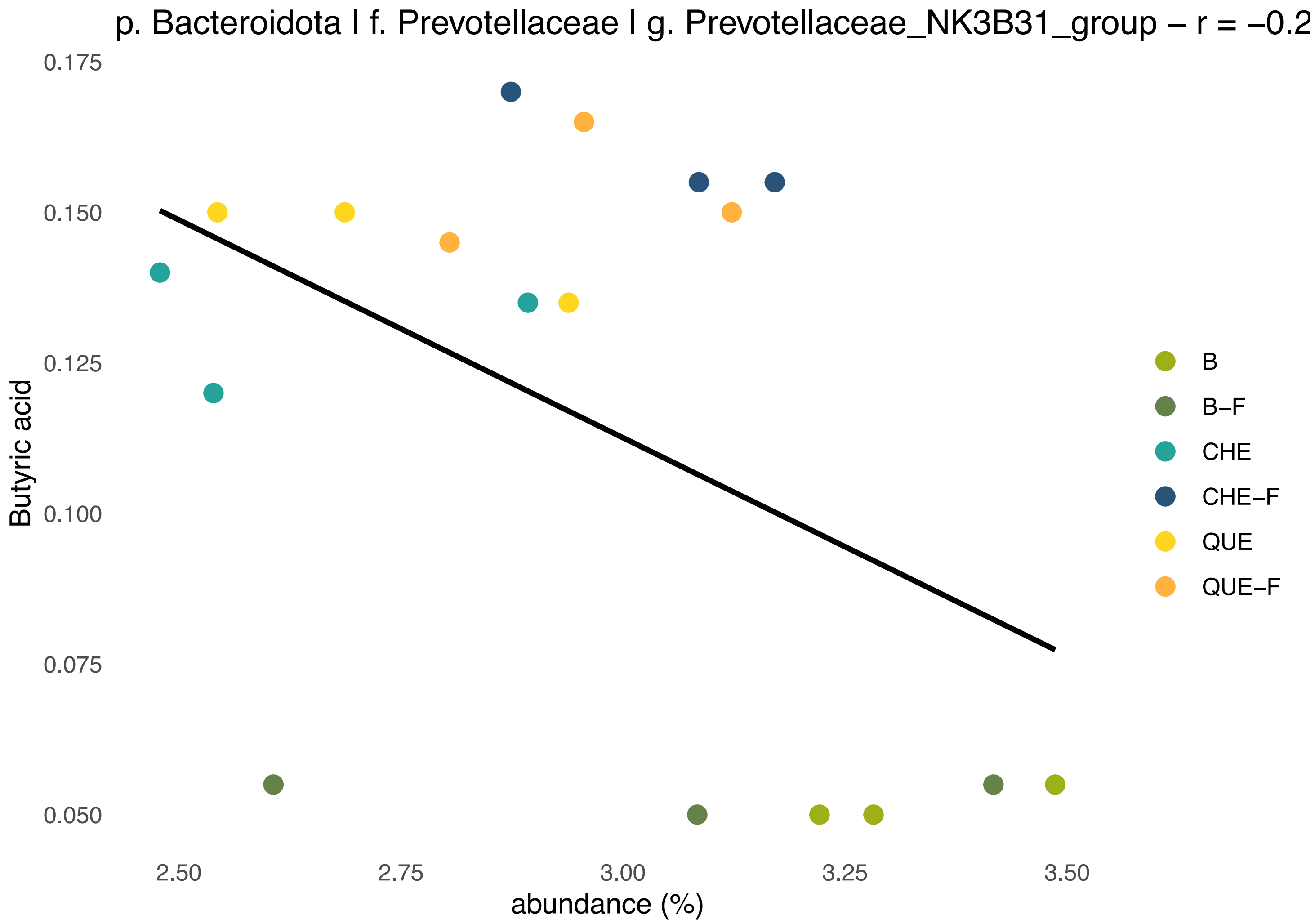

p. Bacteroidota | f. Prevotellaceae | g. Prevotellaceae_NK3B31_group – r = –0.2
Butyric acid
0.175
0.150
0.125
0.100
0.075
0.050
2.50
2.75
3.00
3.25
3.50
abundance (%)
B
B–F
CHE
CHE–F
QUE
QUE–F

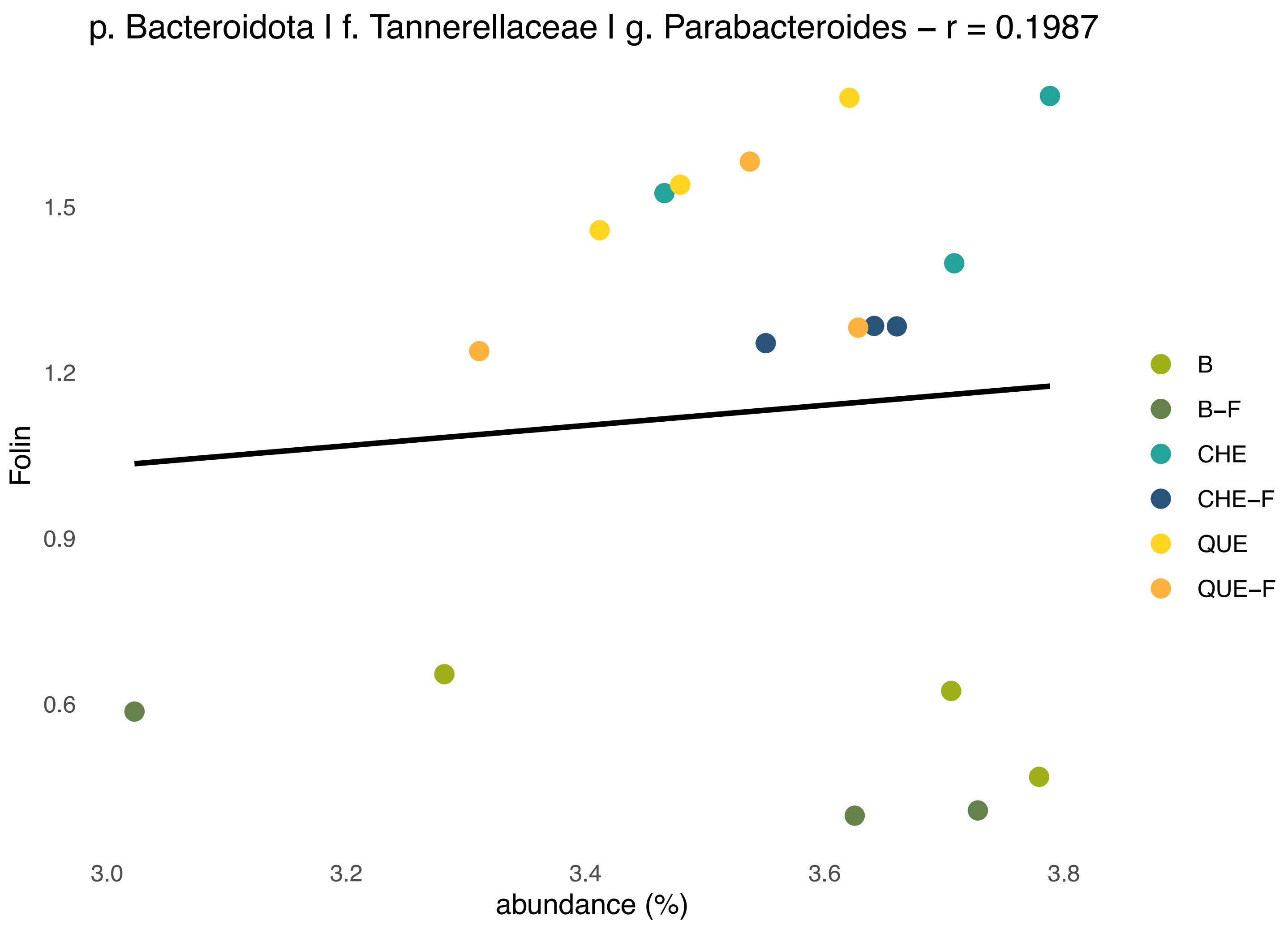
p. Bacteroidota I f. Tannerellaceae I g. Parabacteroides – r = 0.1987
1.5
1.2
0.9
0.6
Folin
3.0
3.2
3.4
3.6
3.8
abundance (%)
B
B–F
CHE
CHE–F
QUE
QUE–F

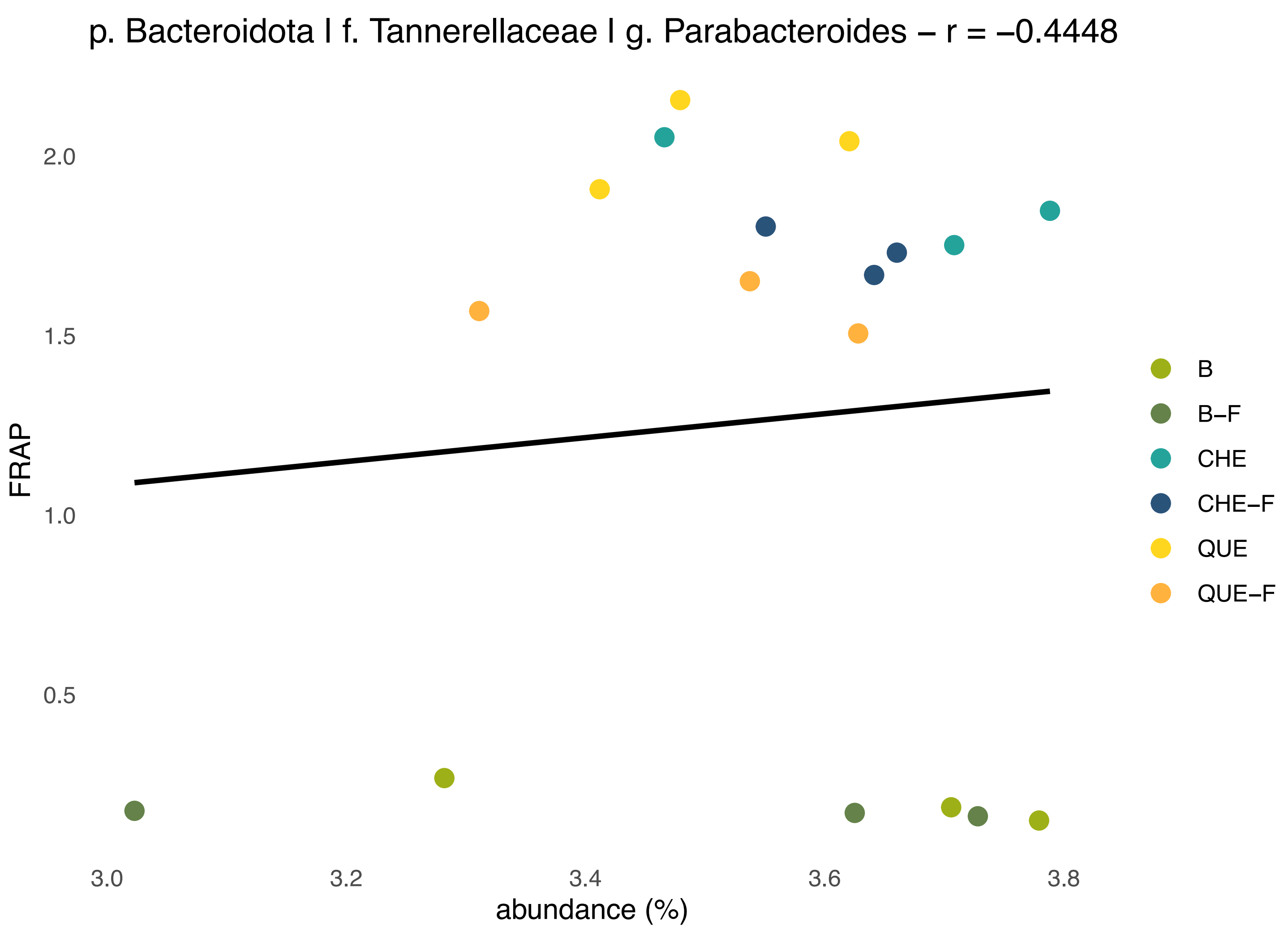

p. Bacteroidota | f. Tannerellaceae | g. Parabacteroides – r = –0.4448
FRAP
2.0
1.5
1.0
0.5
3.0
3.2
3.4
3.6
3.8
abundance (%)
B
B–F
CHE
CHE–F
QUE
QUE–F

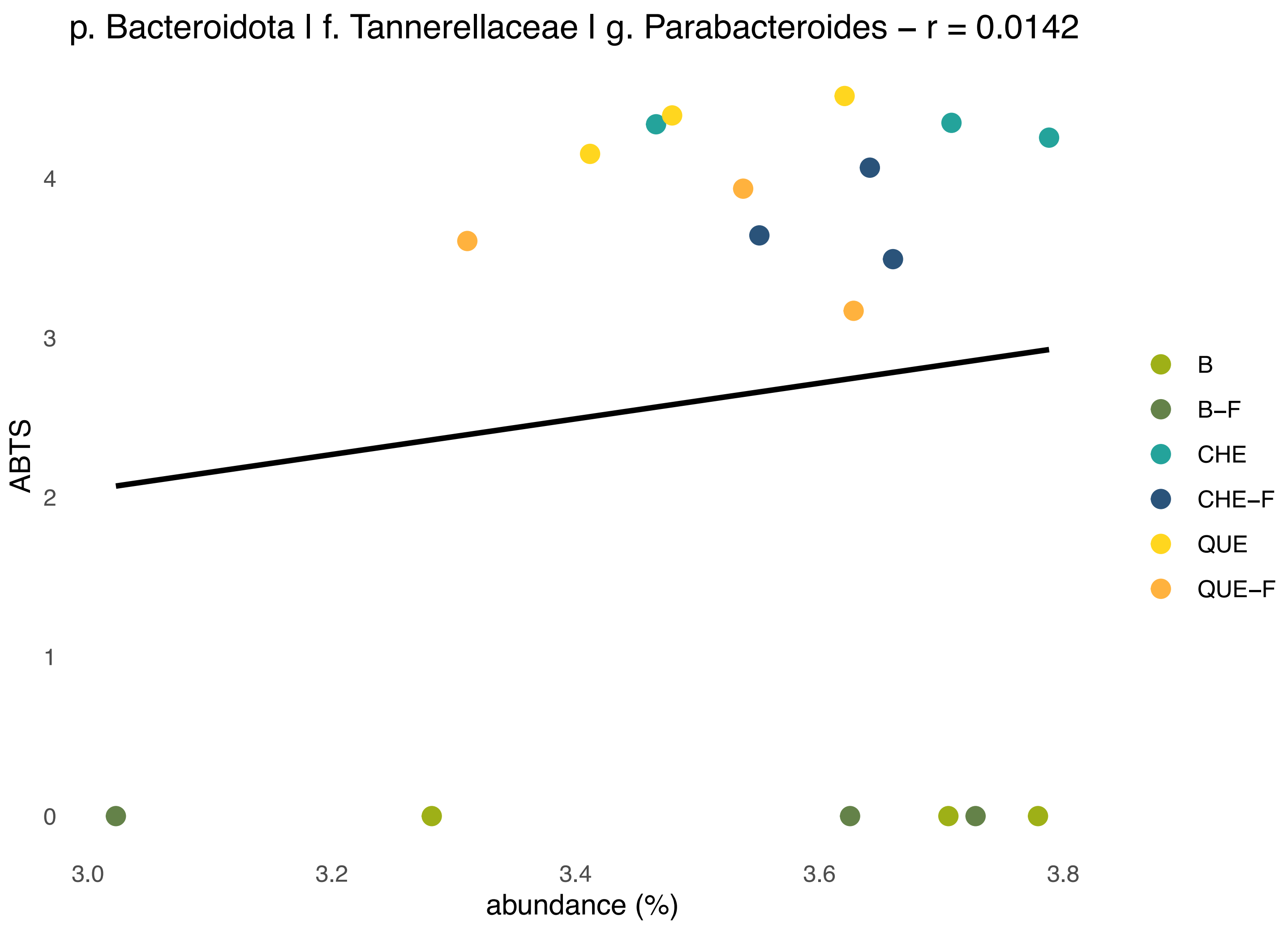
p. Bacteroidota | f. Tannerellaceae | g. Parabacteroides – r = 0.0142
ABTS
0
1
2
3
4
3.0
3.2
3.4
3.6
3.8
abundance (%)
B
B–F
CHE
CHE–F
QUE
QUE–F

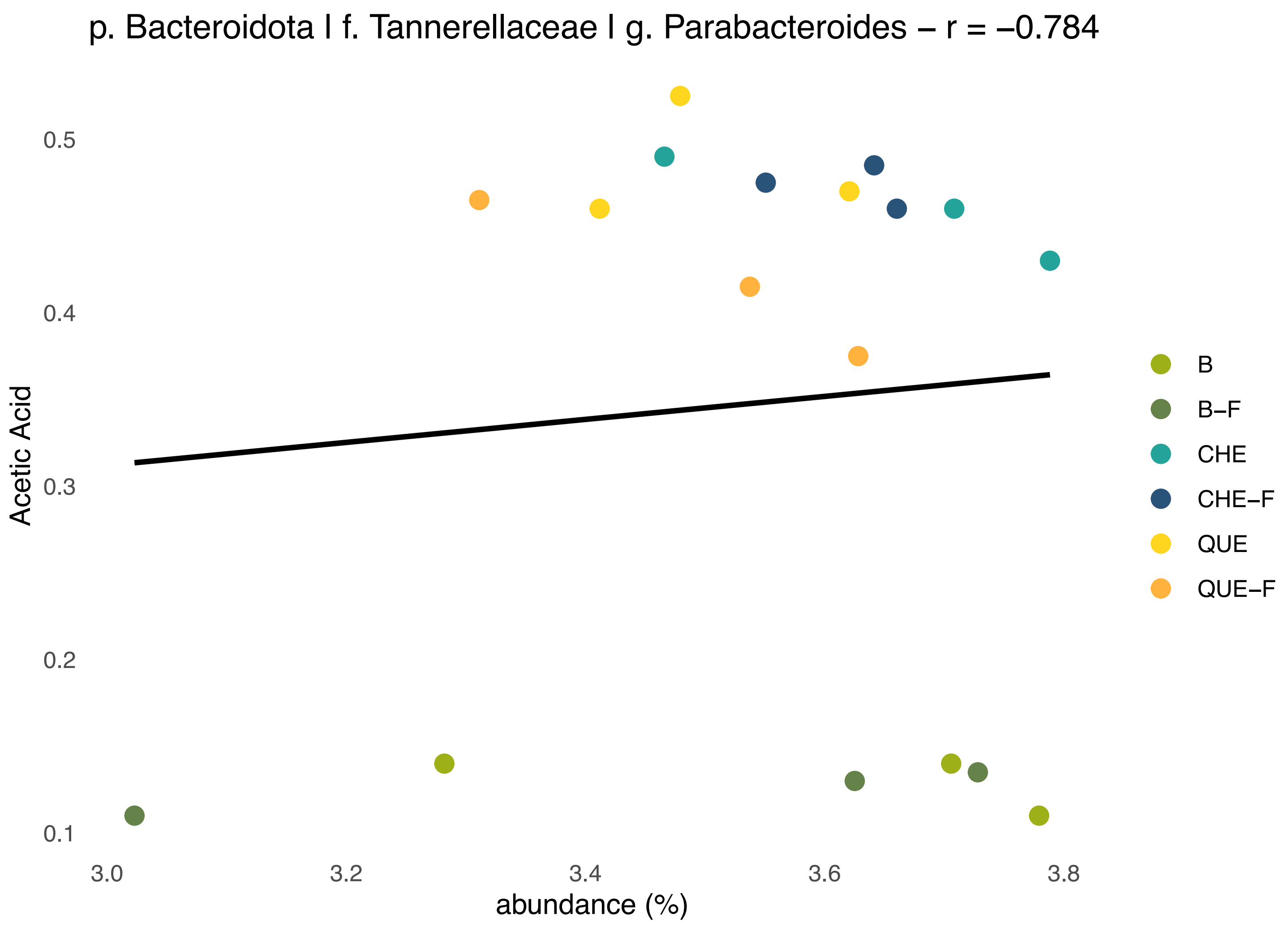

p. Bacteroidota l f. Tannerellaceae l g. Parabacteroides – r = –0.784
Acetic Acid
0.5
0.4
0.3
0.2
0.1
3.0
3.2
3.4
3.6
3.8
abundance (%)
B
B–F
CHE
CHE–F
QUE
QUE–F

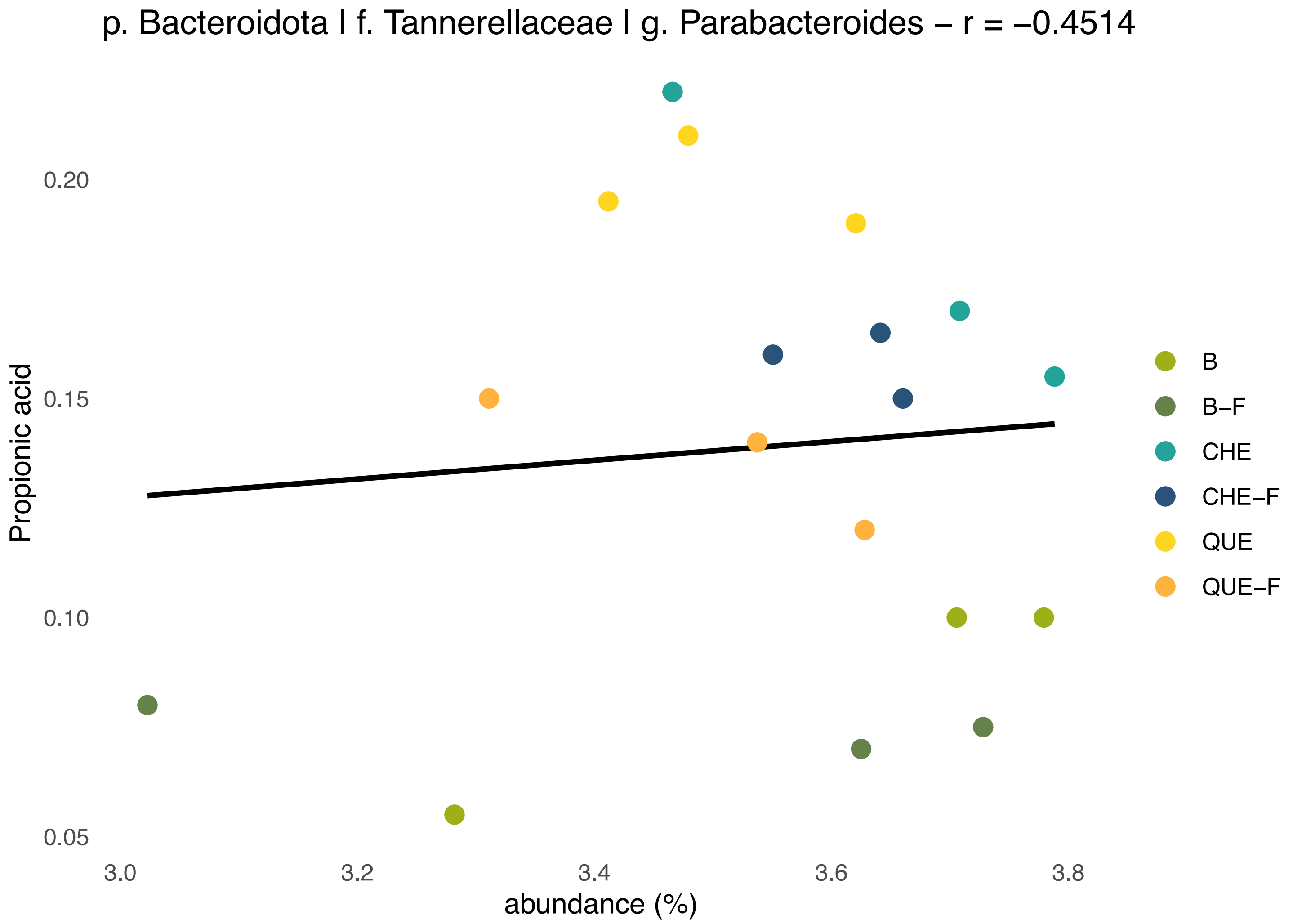
p. Bacteroidota I f. Tannerellaceae I g. Parabacteroides – r = –0.4514
Propionic acid
0.20
0.15
0.10
0.05
3.0
3.2
3.4
3.6
3.8
abundance (%)
B
B–F
CHE
CHE–F
QUE
QUE–F

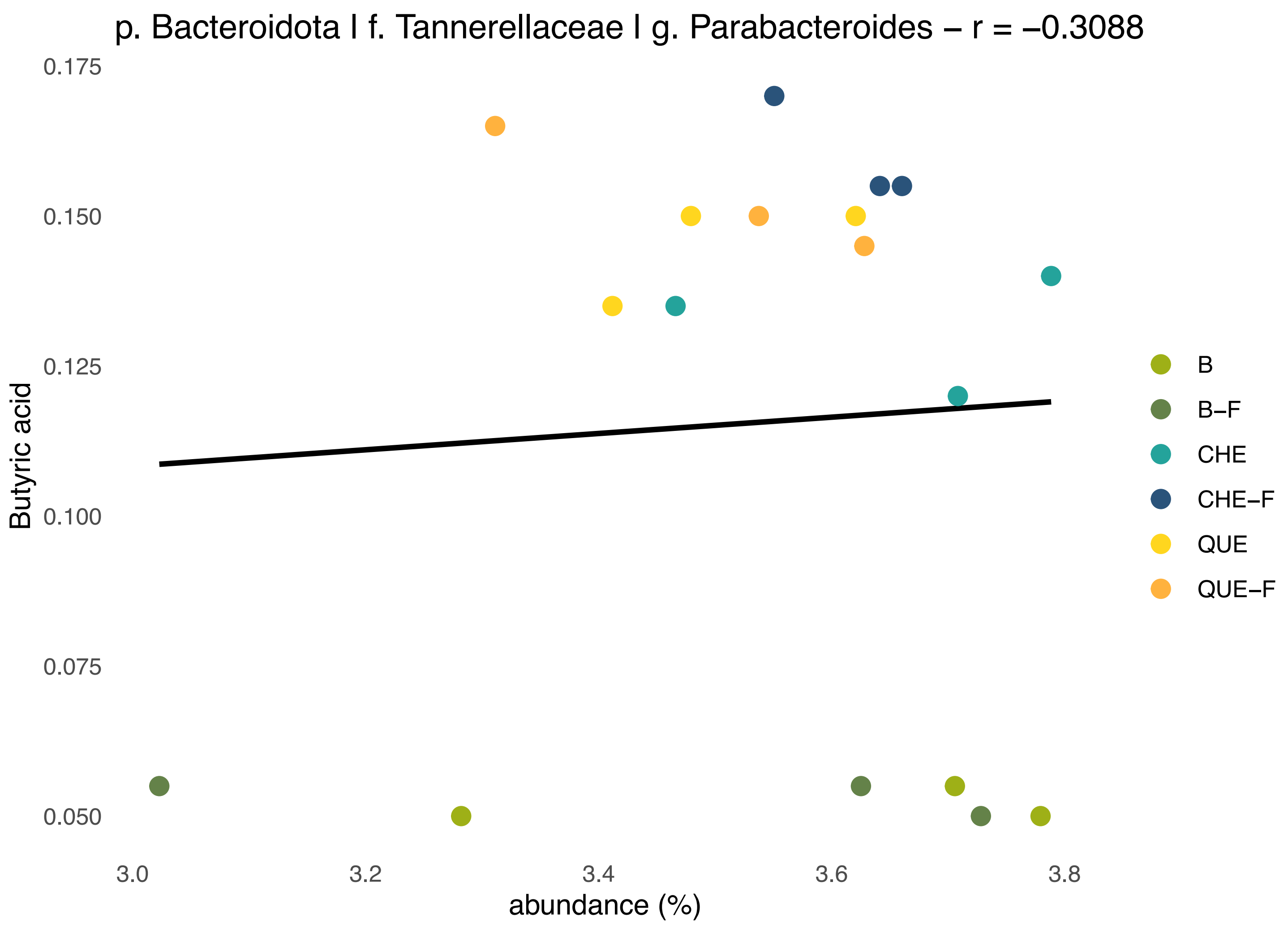
p. Bacteroidota | f. Tannerellaceae | g. Parabacteroides – r = −0.3088
Butyric acid
0.175
0.150
0.125
0.100
0.075
0.050
3.0
3.2
3.4
3.6
3.8
abundance (%)
B
B−F
CHE
CHE−F
QUE
QUE−F

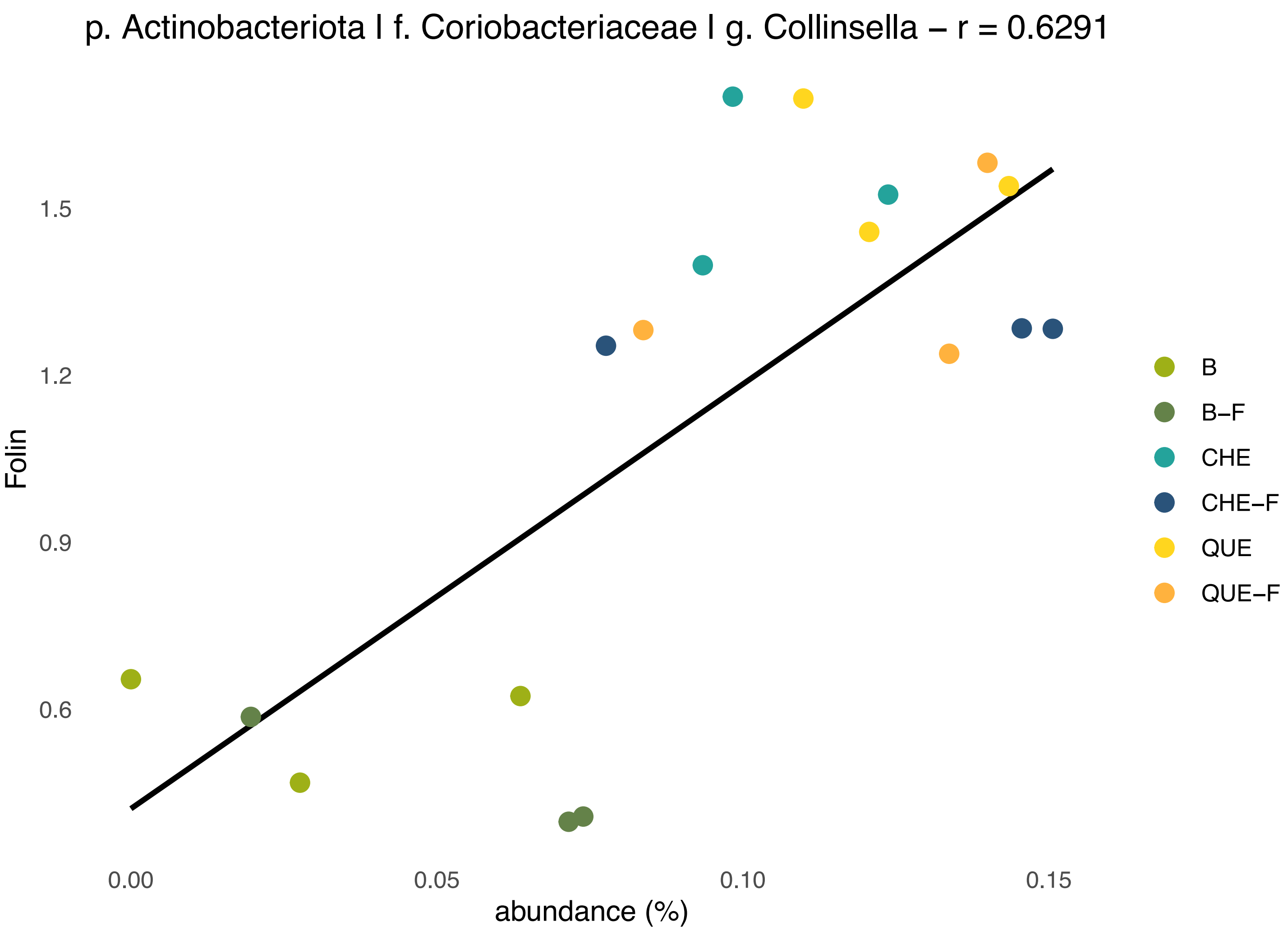
p. Actinobacteriota I f. Coriobacteriaceae I g. Collinsella – r = 0.6291
1.5
1.2
0.9
0.6
Folin
0.00
0.05
0.10
0.15
abundance (%)
B
B–F
CHE
CHE–F
QUE
QUE–F

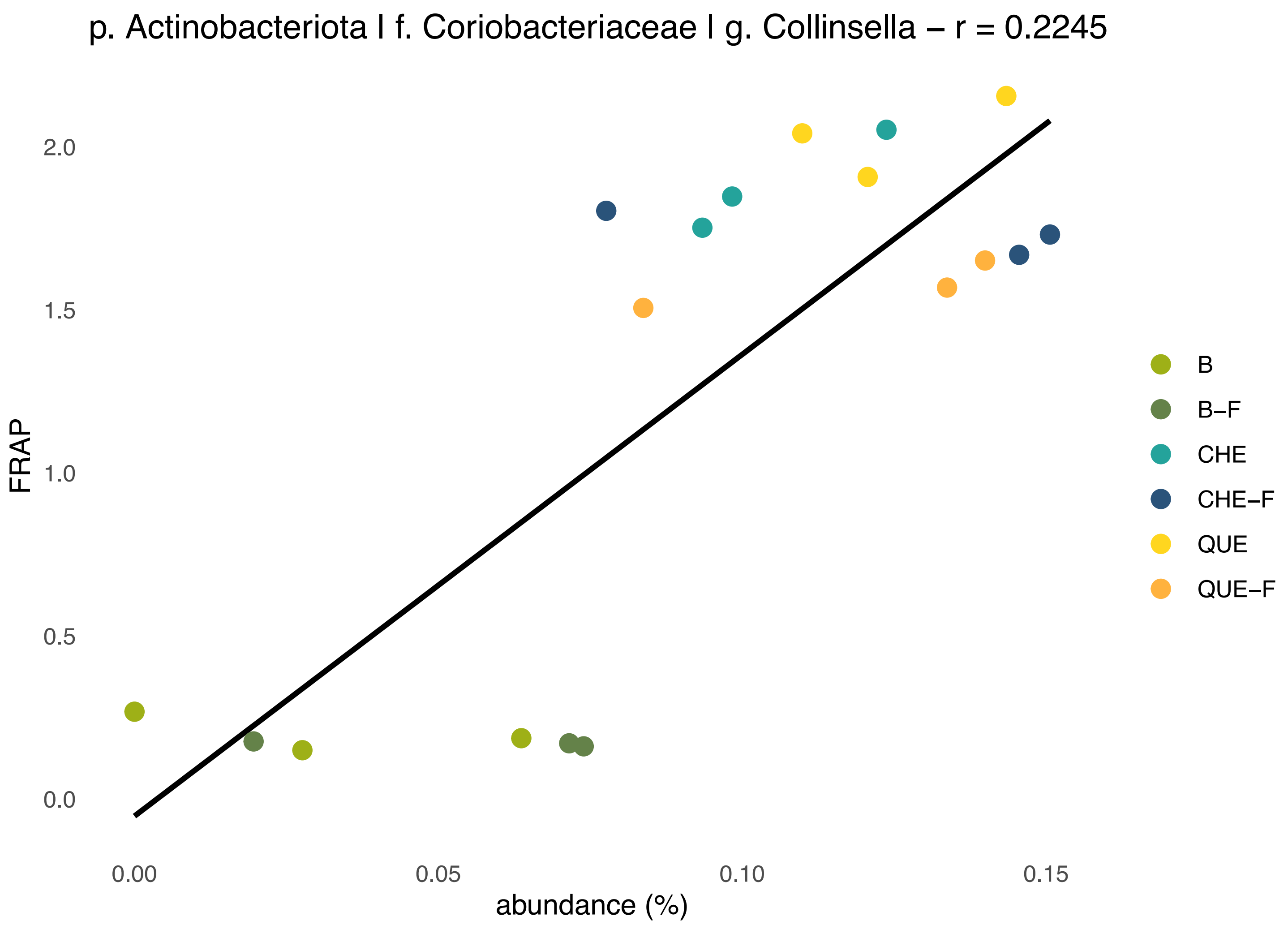

p. Actinobacteriota I f. Coriobacteriaceae I g. Collinsella – r = 0.2245
FRAP
2.0
1.5
1.0
0.5
0.0
0.00
0.05
0.10
0.15
abundance (%)
B
B–F
CHE
CHE–F
QUE
QUE–F

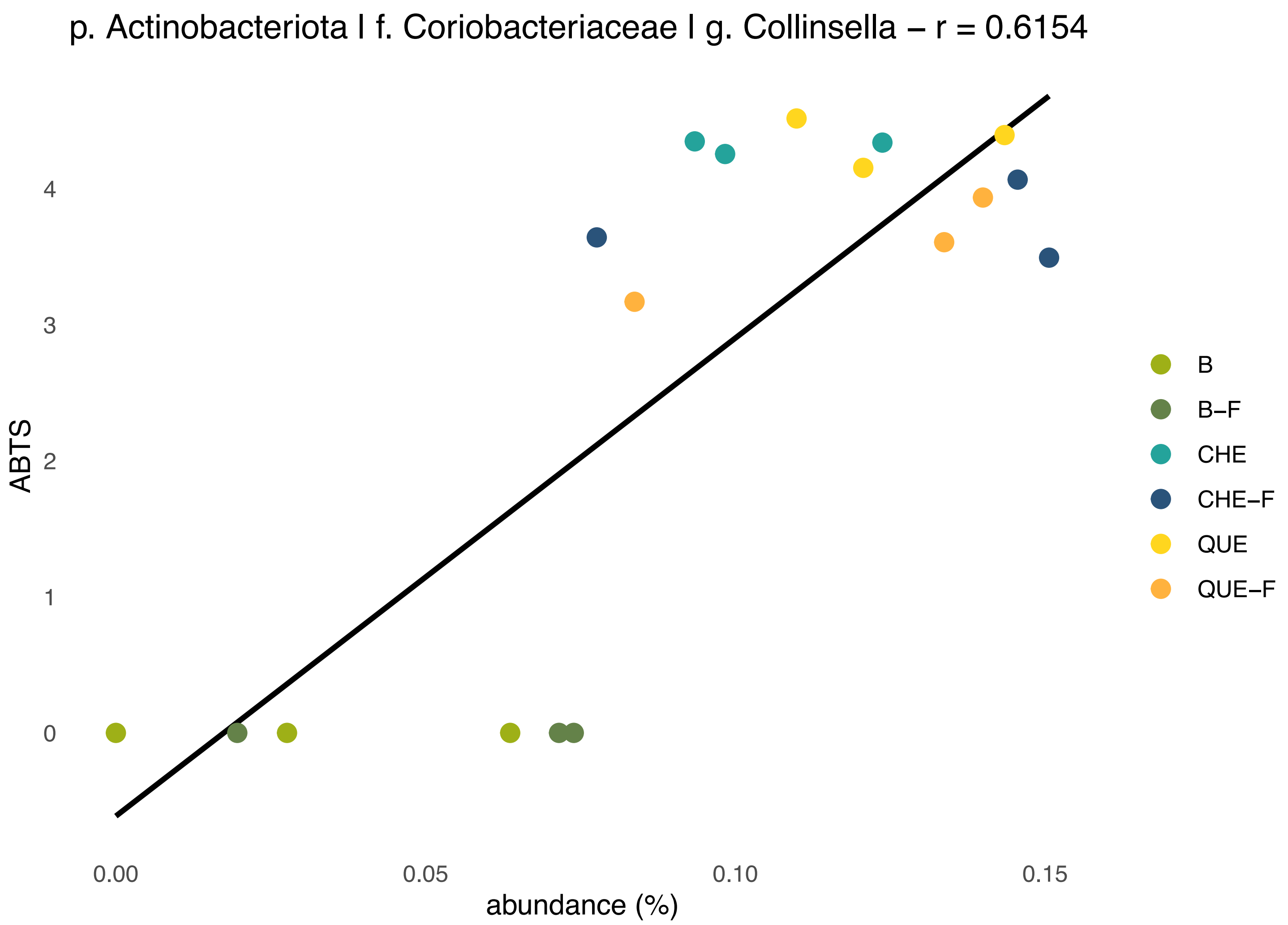
p. Actinobacteriota I f. Coriobacteriaceae I g. Collinsella – r = 0.6154
ABTS
4
3
2
1
0
0.00
0.05
0.10
0.15
abundance (%)
B
B–F
CHE
CHE–F
QUE
QUE–F

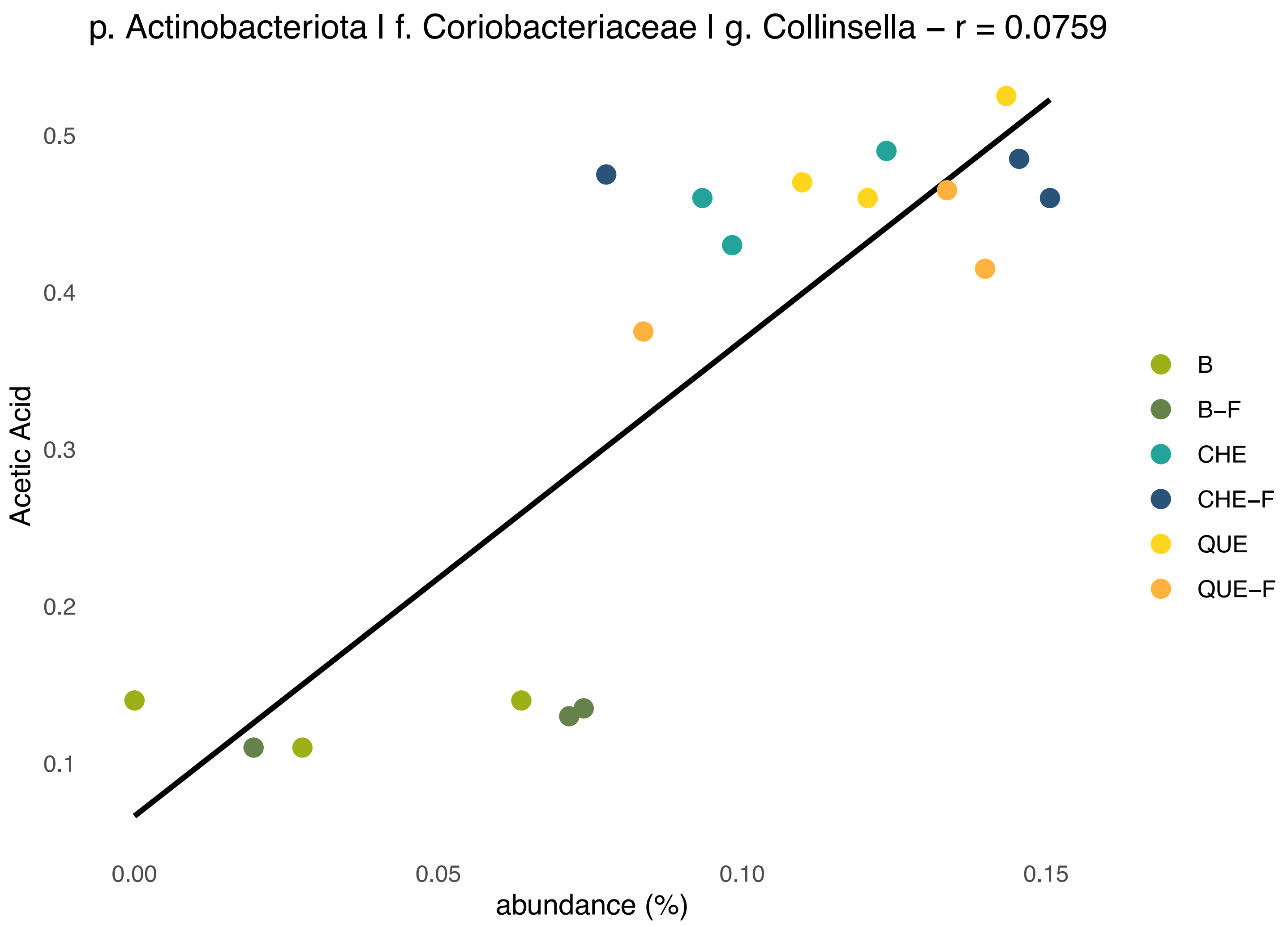

p. Actinobacteriota I f. Coriobacteriaceae I g. Collinsella – r = 0.0759
Acetic Acid
0.5
0.4
0.3
0.2
0.1
0.00
0.05
0.10
0.15
abundance (%)
B
B–F
CHE
CHE–F
QUE
QUE–F

p. Actinobacteriota I f. Coriobacteriaceae I g. Collinsella – r = 0.2172

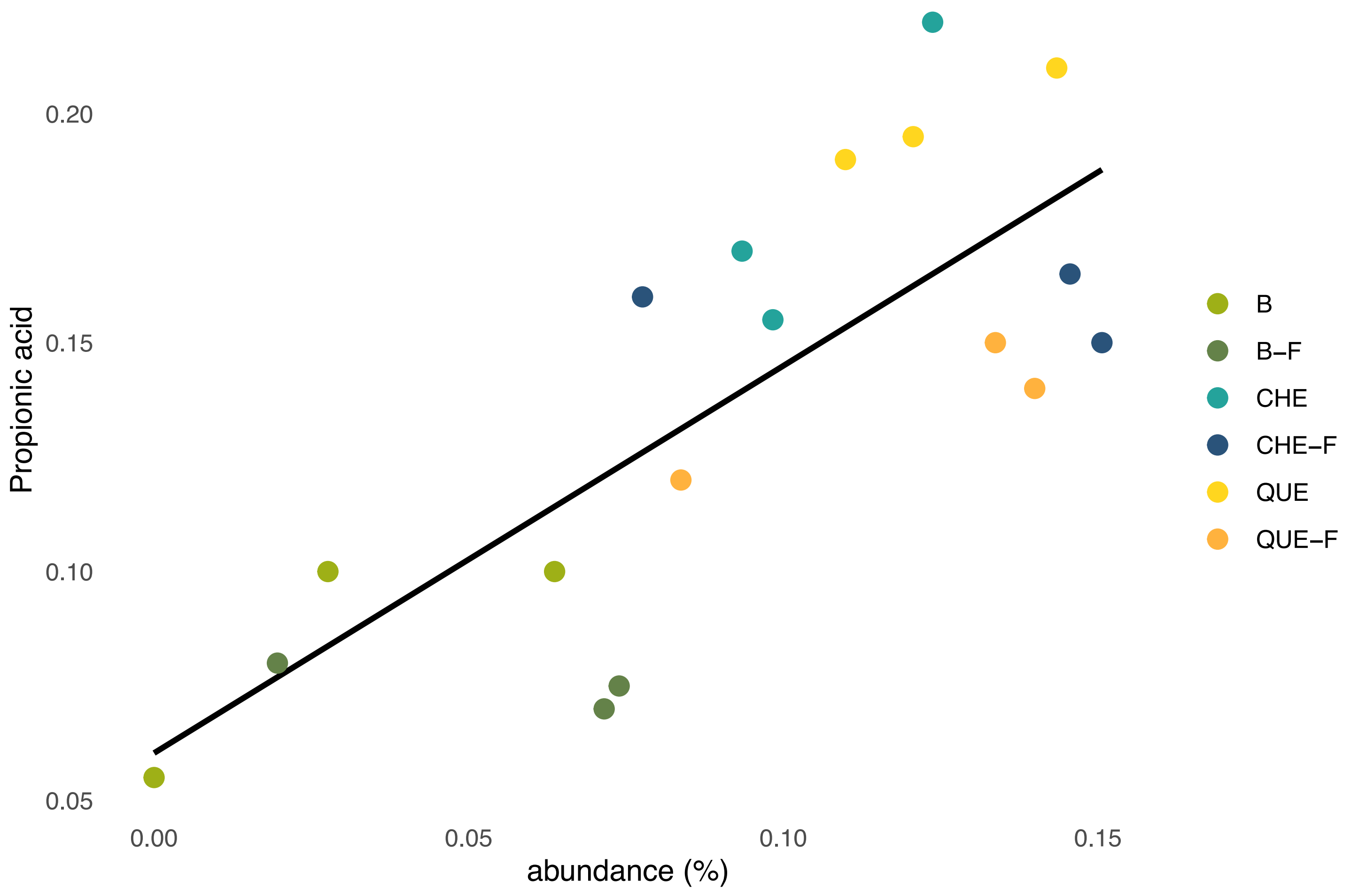

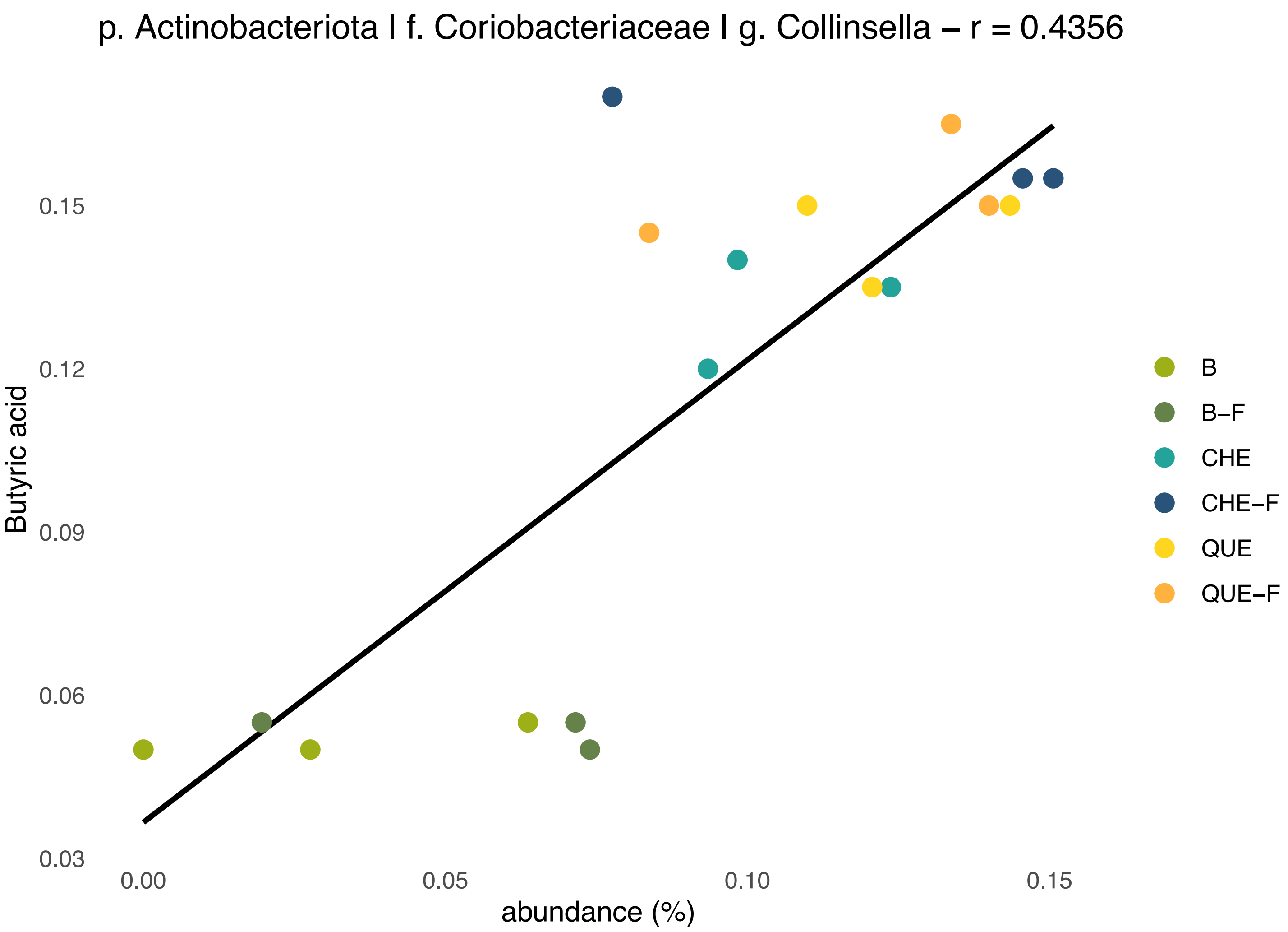
p. Actinobacteriota I f. Coriobacteriaceae I g. Collinsella – r = 0.4356
Butyric acid
0.03
0.06
0.09
0.12
0.15
0.00
0.05
0.10
0.15
abundance (%)
B
B–F
CHE
CHE–F
QUE
QUE–F

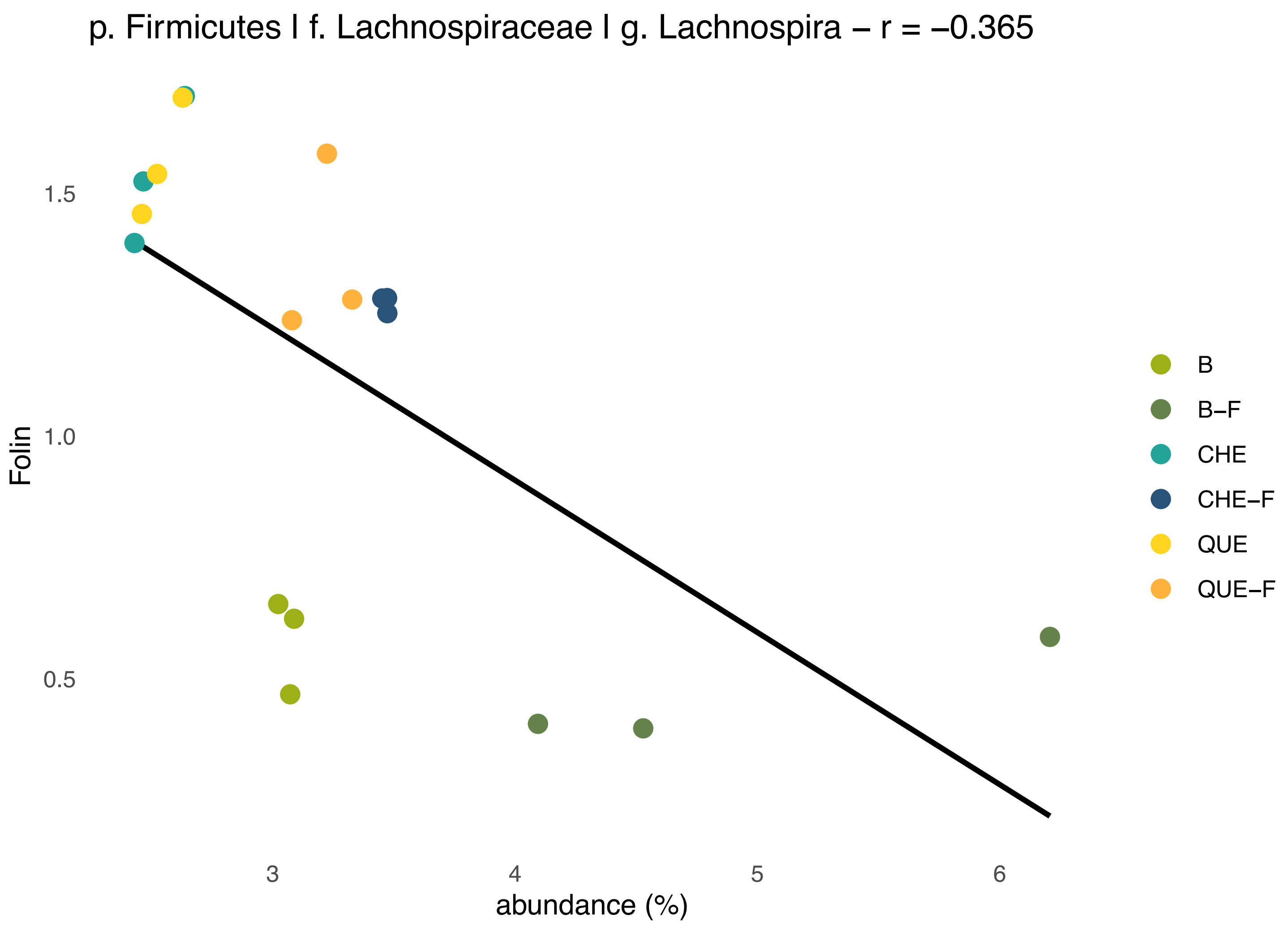

p. Firmicutes l f. Lachnospiraceae l g. Lachnospira – r = –0.365
Folin
1.5
1.0
0.5
3
4
5
6
abundance (%)
B
B–F
CHE
CHE–F
QUE
QUE–F

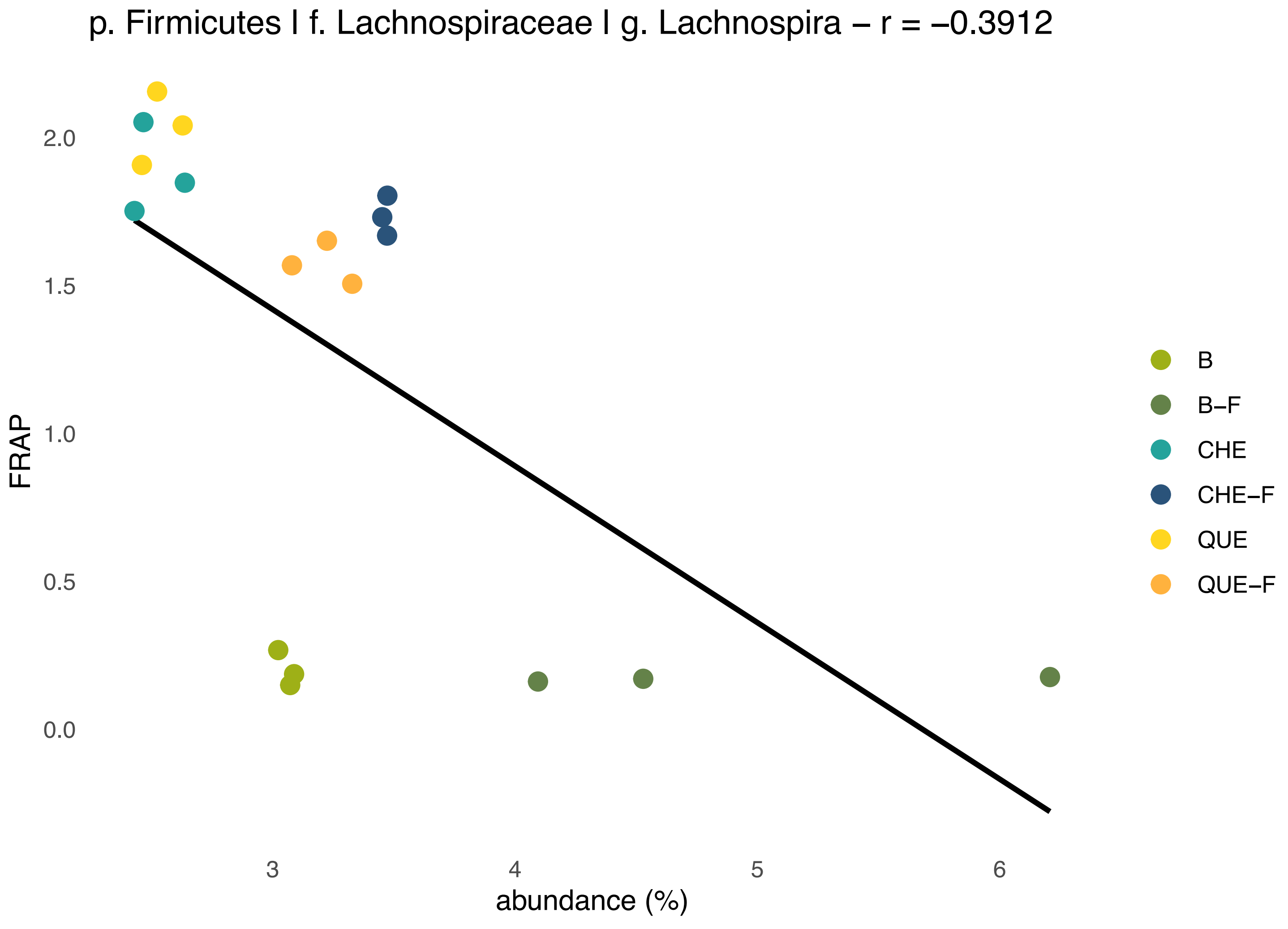
p. Firmicutes I f. Lachnospiraceae I g. Lachnospira – r = −0.3912
FRAP
2.0
1.5
1.0
0.5
0.0
3
4
5
6
abundance (%)
B
B−F
CHE
CHE−F
QUE
QUE−F

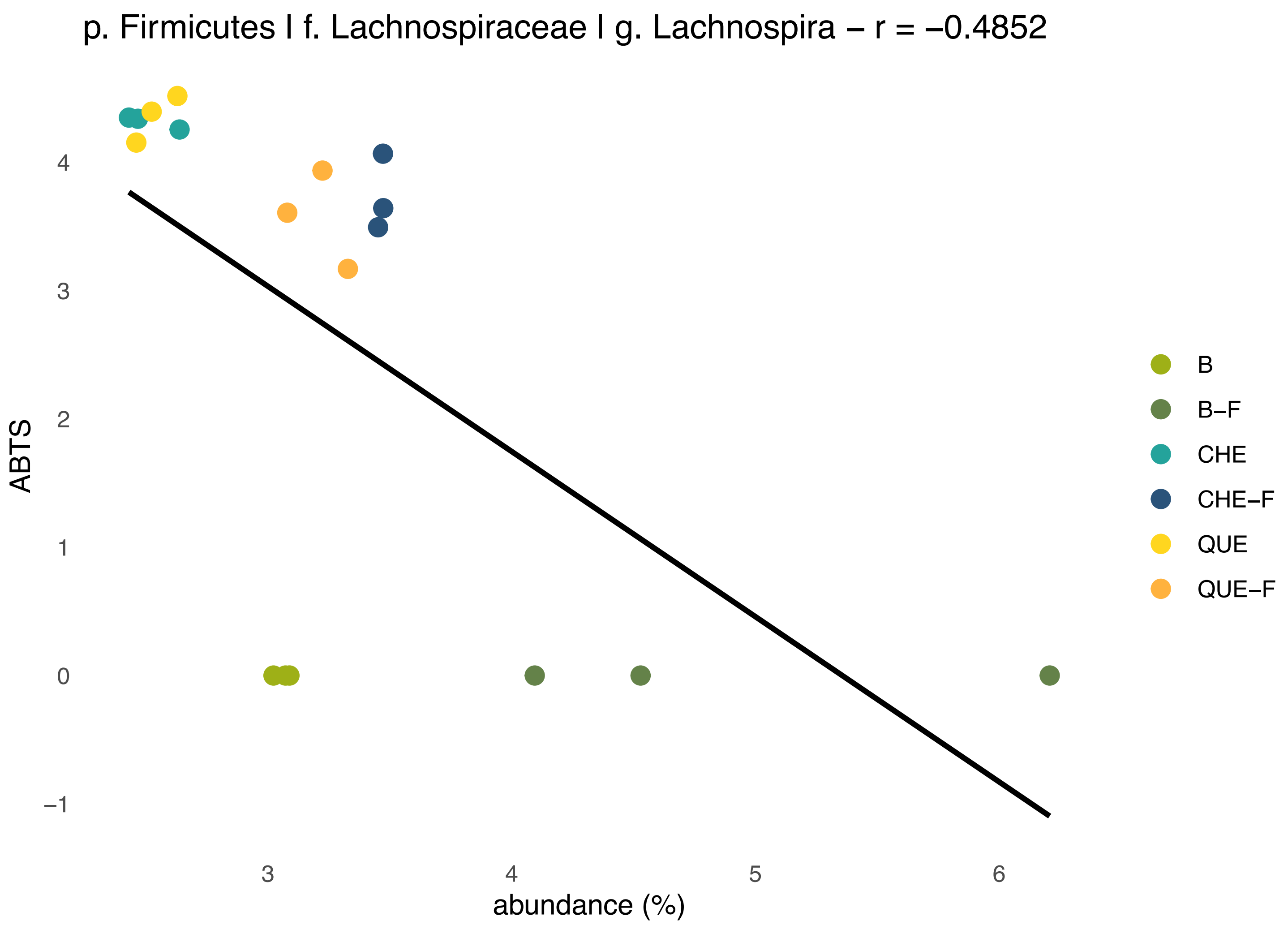

p. Firmicutes I f. Lachnospiraceae I g. Lachnospira – r = –0.4852
ABTS
4
3
2
1
0
–1
3
4
5
6
abundance (%)
B
B–F
CHE
CHE–F
QUE
QUE–F

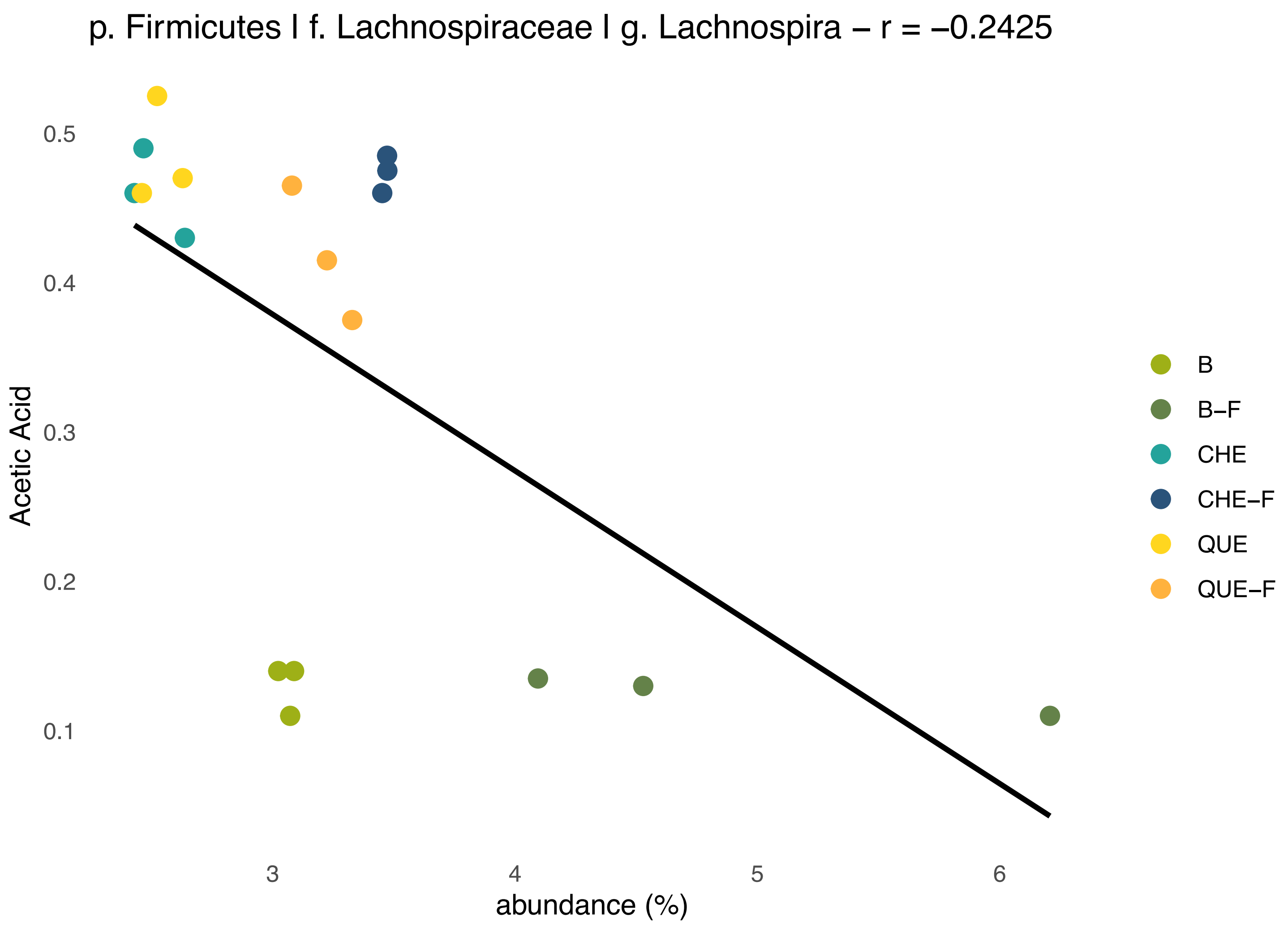

p. Firmicutes I f. Lachnospiraceae I g. Lachnospira – r = –0.2425
Acetic Acid
0.5
0.4
0.3
0.2
0.1
3
4
5
6
abundance (%)
B
B–F
CHE
CHE–F
QUE
QUE–F

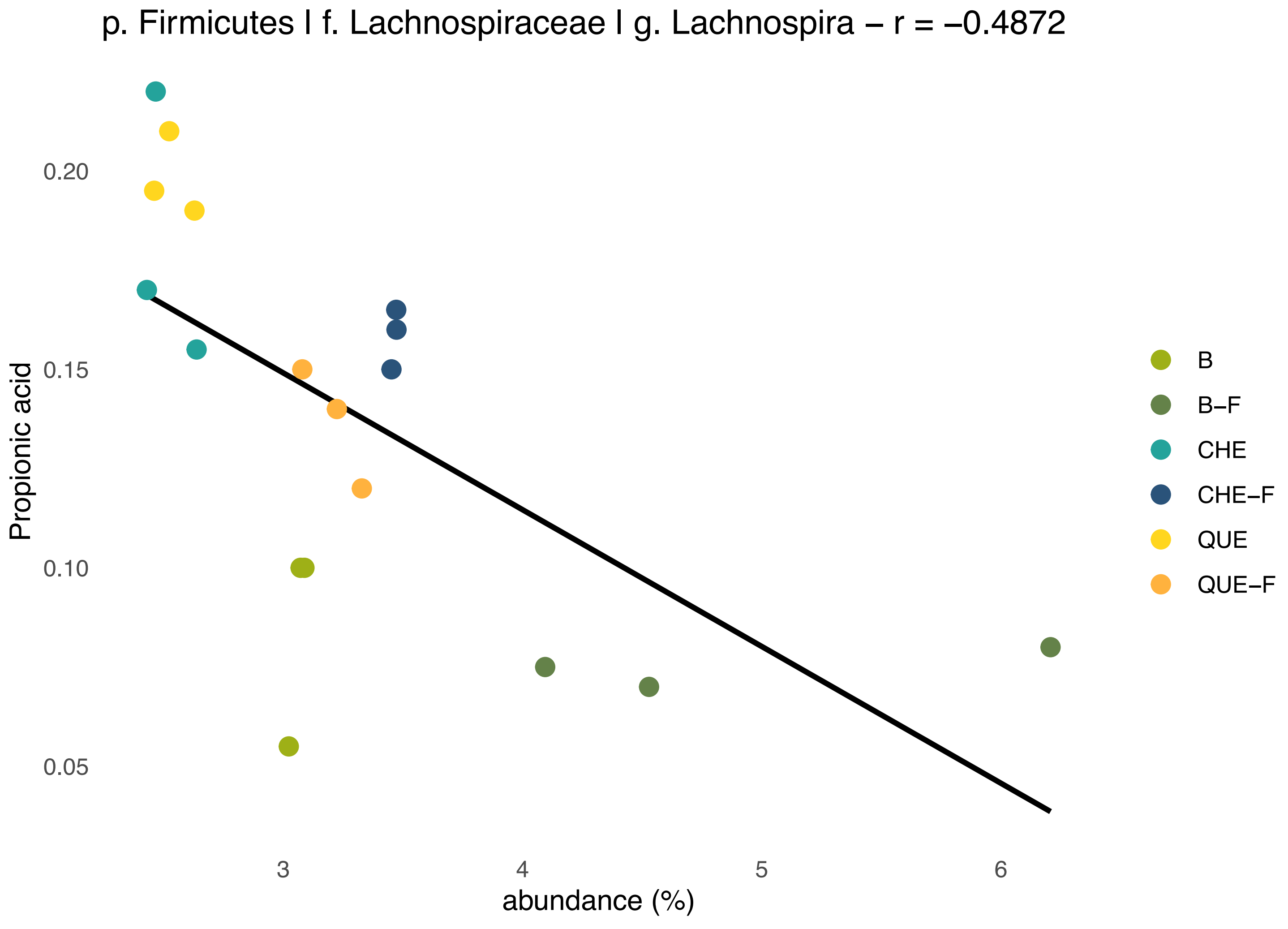

p. Firmicutes I f. Lachnospiraceae I g. Lachnospira – r = −0.4872
Propionic acid
0.20
0.15
0.10
0.05
3
4
5
6
abundance (%)
B
B−F
CHE
CHE−F
QUE
QUE−F

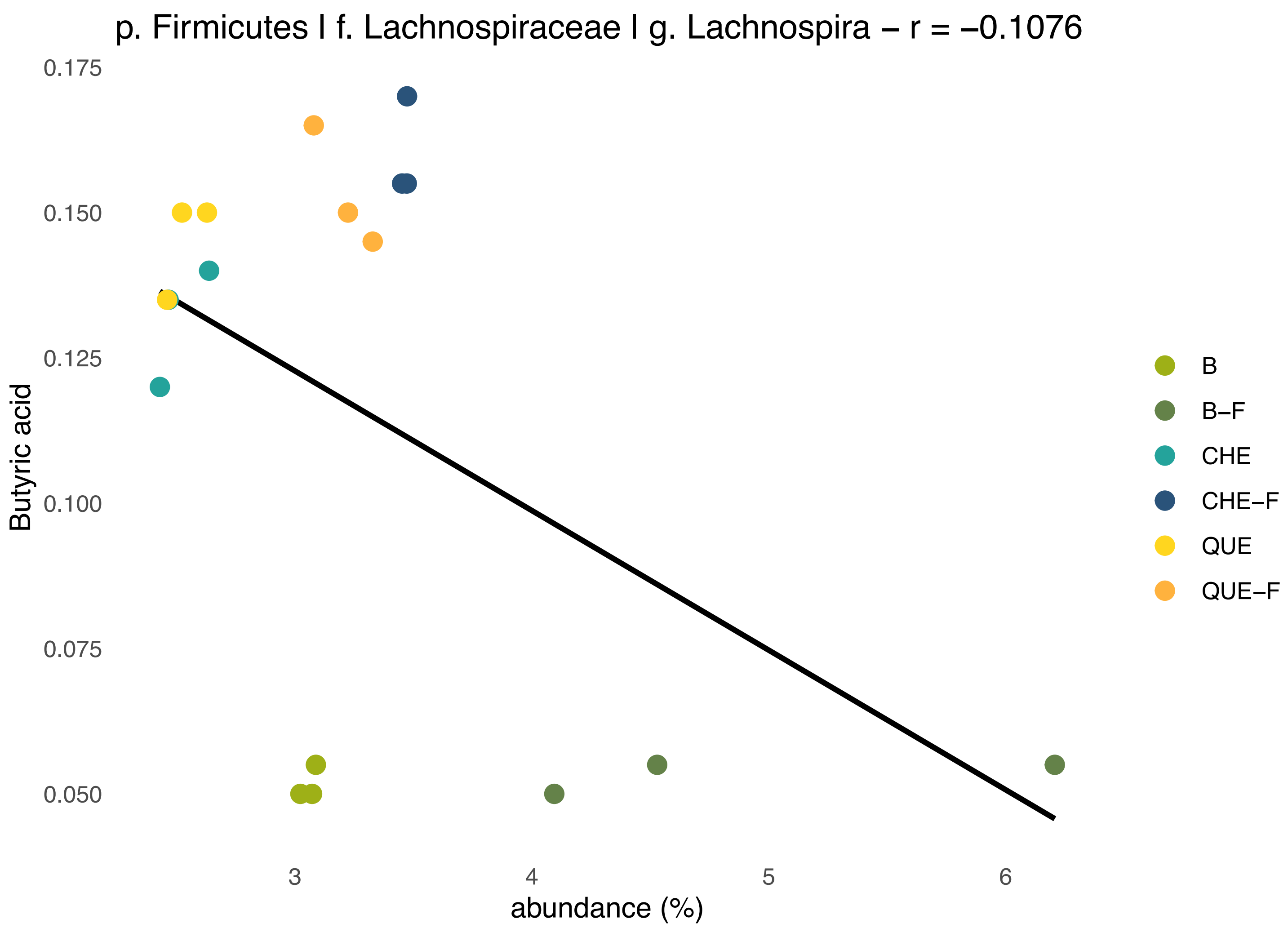

p. Firmicutes I f. Lachnospiraceae I g. Lachnospira – r = –0.1076
0.175
0.150
0.125
0.100
0.075
0.050
Butyric acid
3
4
5
6
abundance (%)
B
B–F
CHE
CHE–F
QUE
QUE–F

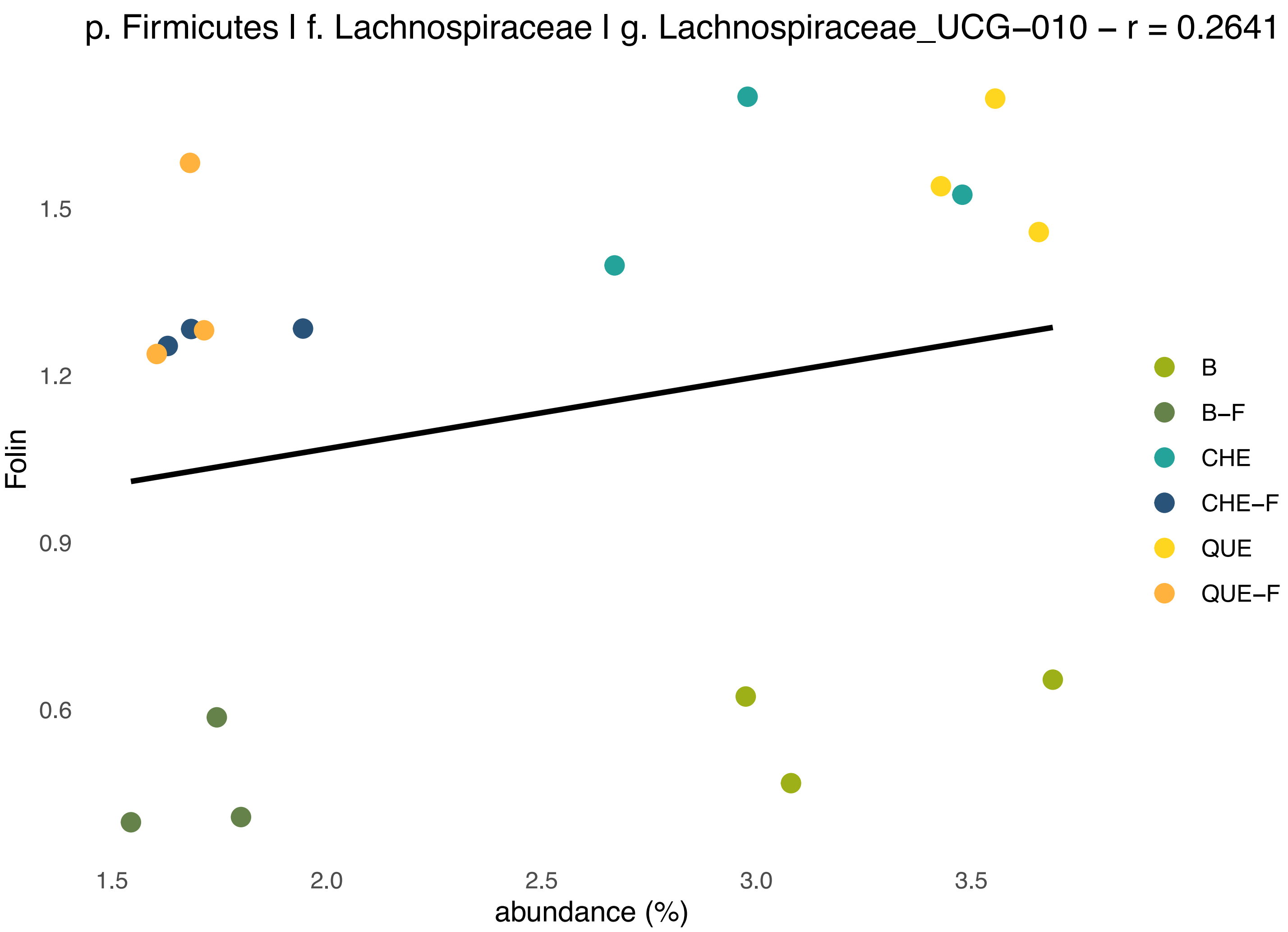

p. Firmicutes I f. Lachnospiraceae I g. Lachnospiraceae_UCG−010 − r = 0.2641
Folin
1.5
1.2
0.9
0.6
1.5
2.0
2.5
3.0
3.5
abundance (%)
B
B−F
CHE
CHE−F
QUE
QUE−F

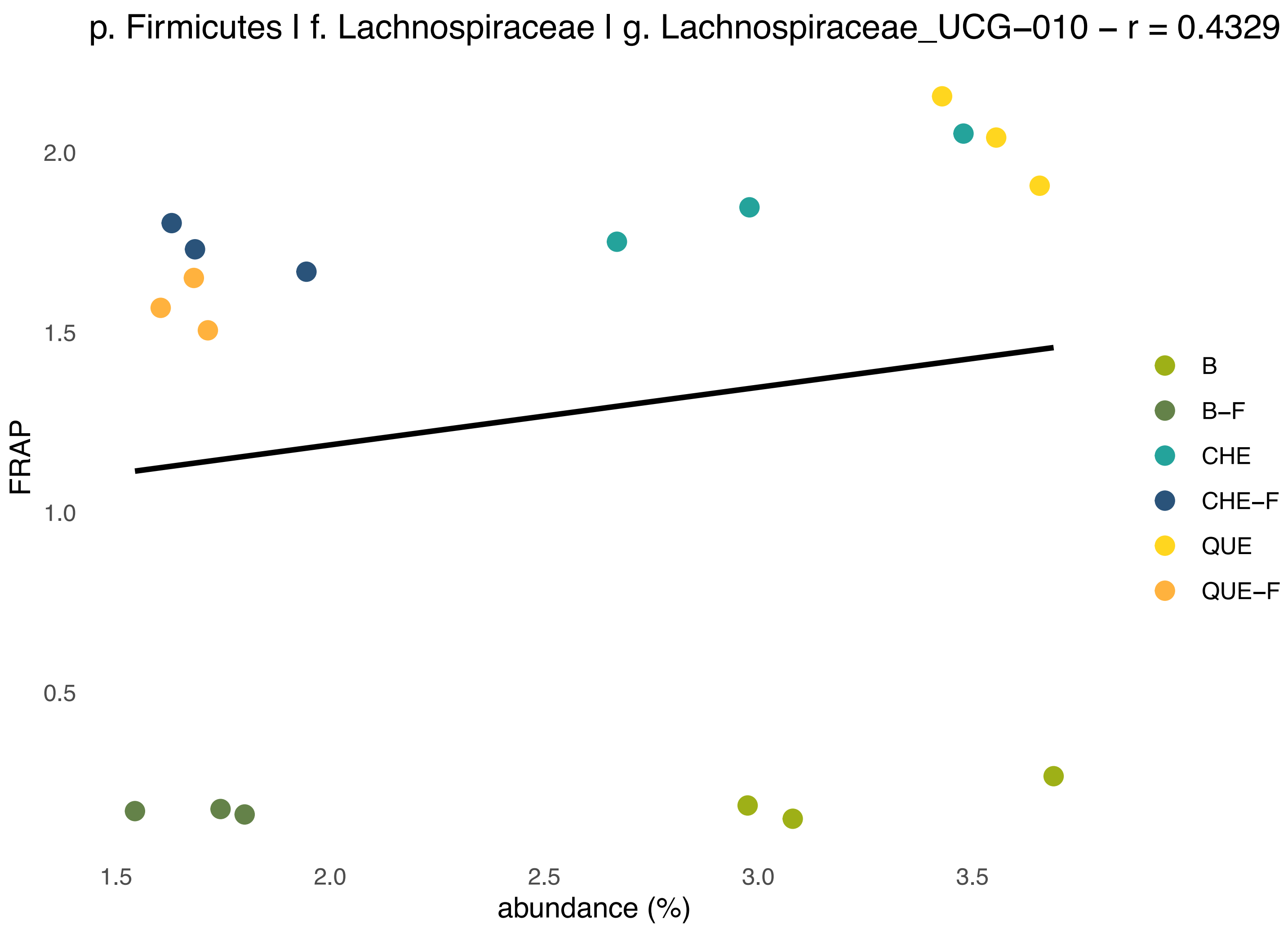

p. Firmicutes I f. Lachnospiraceae I g. Lachnospiraceae_UCG−010 − r = 0.4329
FRAP
2.0
1.5
1.0
0.5
1.5
2.0
2.5
3.0
3.5
abundance (%)
B
B−F
CHE
CHE−F
QUE
QUE−F

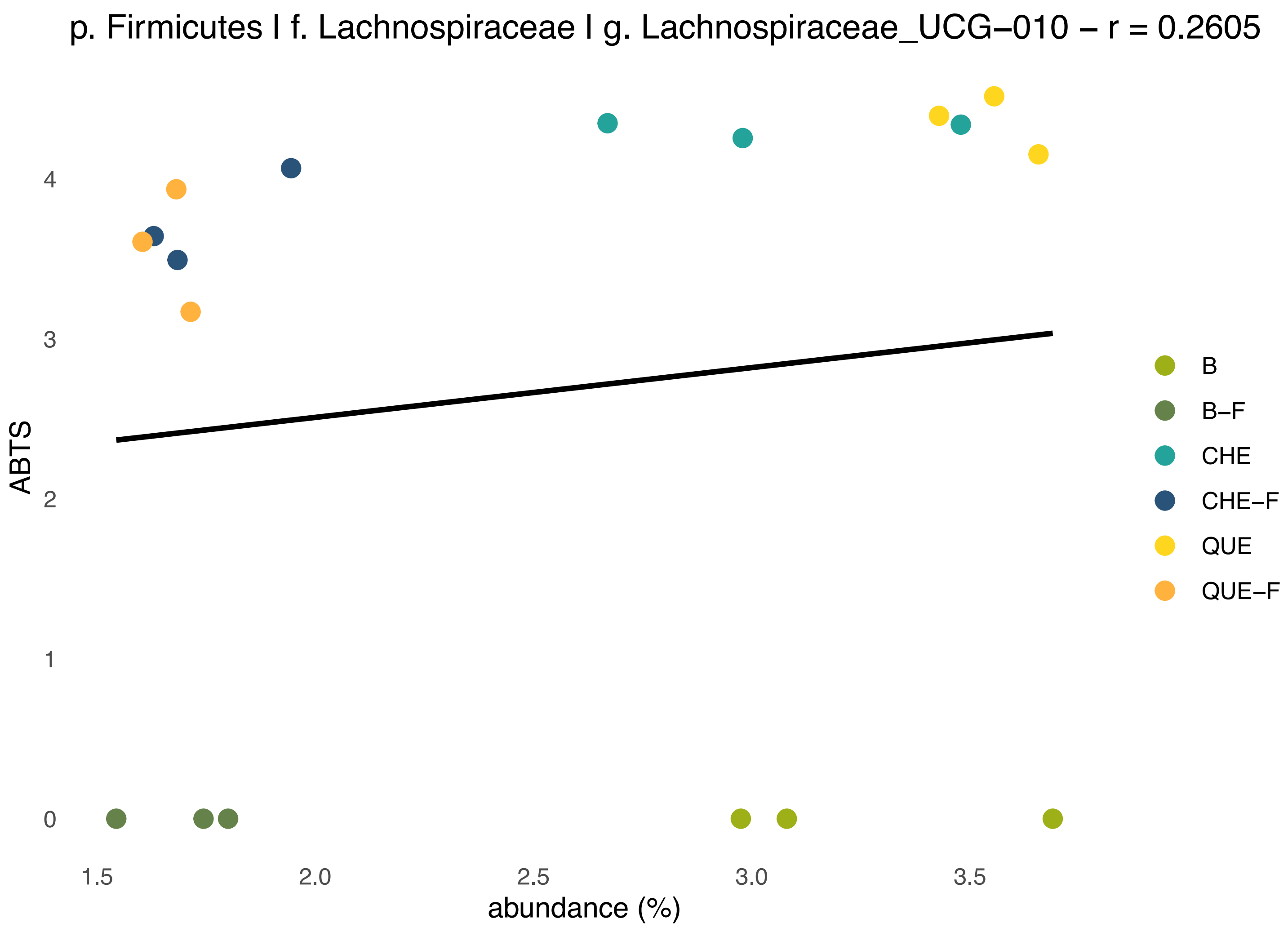

p. Firmicutes I f. Lachnospiraceae I g. Lachnospiraceae_UCG−010 − r = 0.2605
ABTS
4
3
2
1
0
1.5
2.0
2.5
3.0
3.5
abundance (%)
B
B−F
CHE
CHE−F
QUE
QUE−F

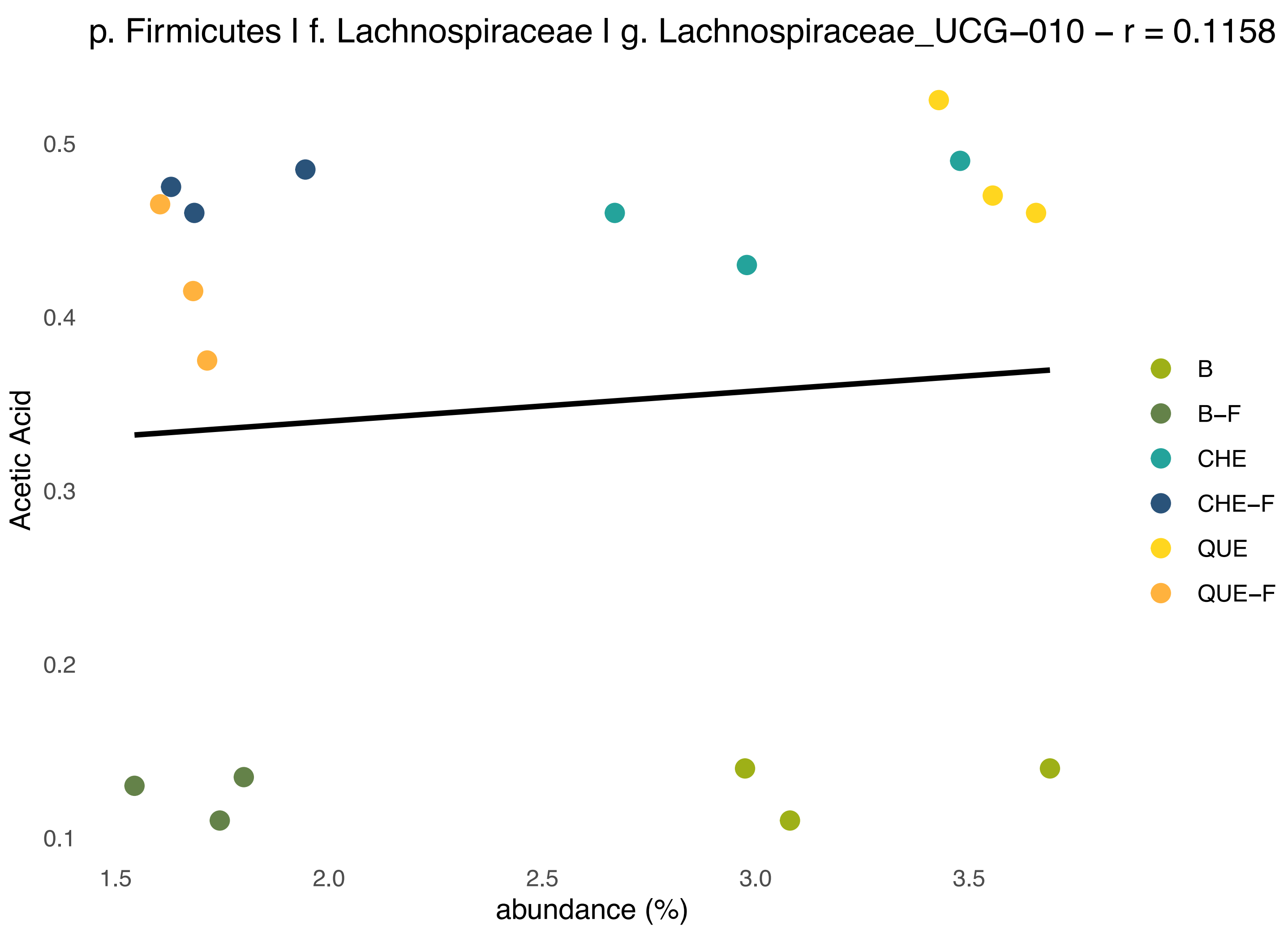
p. Firmicutes l f. Lachnospiraceae l g. Lachnospiraceae_UCG−010 − r = 0.1158
Acetic Acid
0.5
0.4
0.3
0.2
0.1
1.5
2.0
2.5
3.0
3.5
abundance (%)
B
B−F
CHE
CHE−F
QUE
QUE−F

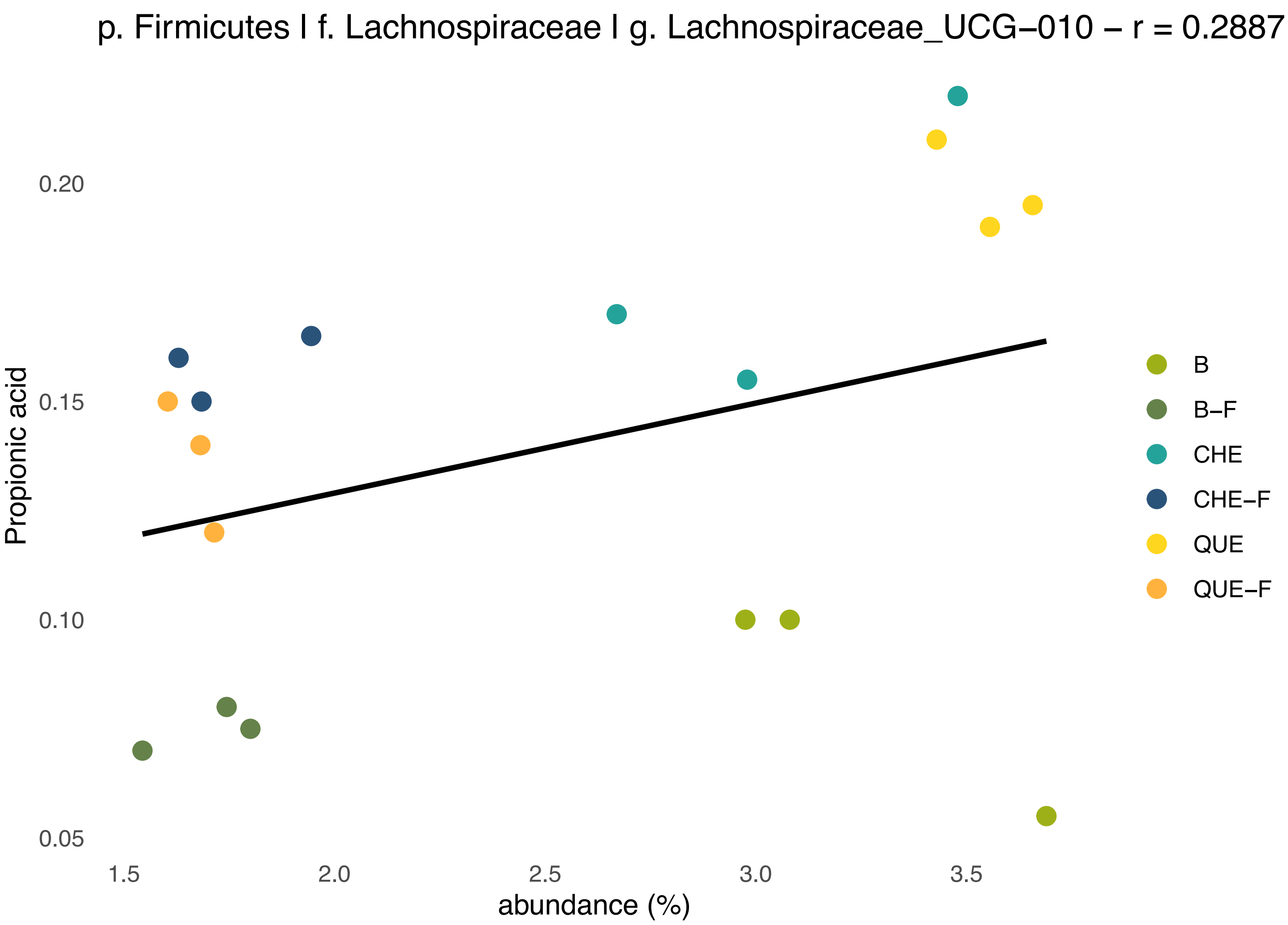

p. Firmicutes I f. Lachnospiraceae I g. Lachnospiraceae_UCG−010 − r = 0.2887
Propionic acid
0.20
0.15
0.10
0.05
1.5
2.0
2.5
3.0
3.5
abundance (%)
B
B−F
CHE
CHE−F
QUE
QUE−F

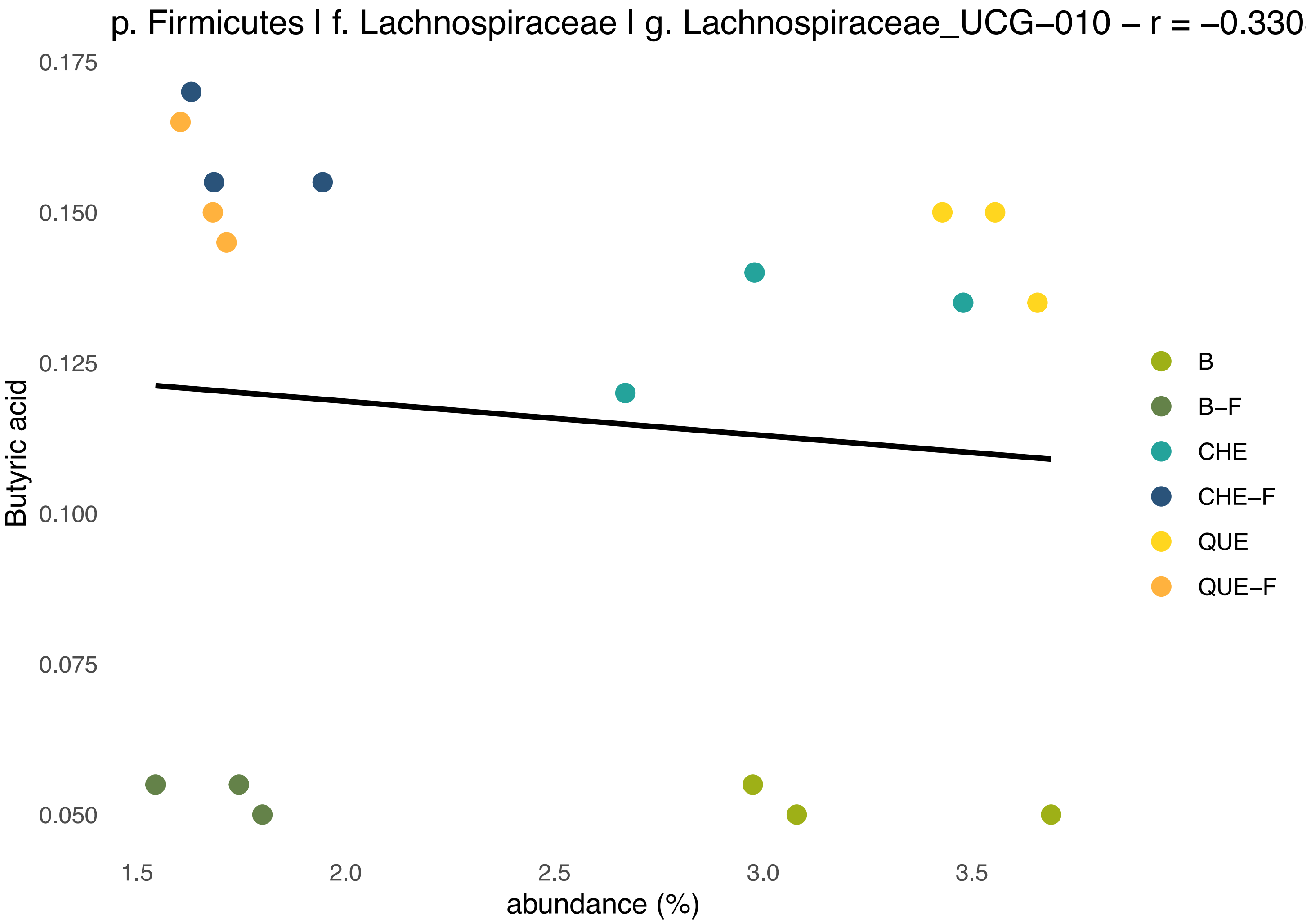
p. Firmicutes I f. Lachnospiraceae I g. Lachnospiraceae_UCG−010 − r = −0.330
Butyric acid
0.175
0.150
0.125
0.100
0.075
0.050
1.5
2.0
2.5
3.0
3.5
abundance (%)
B
B−F
CHE
CHE−F
QUE
QUE−F

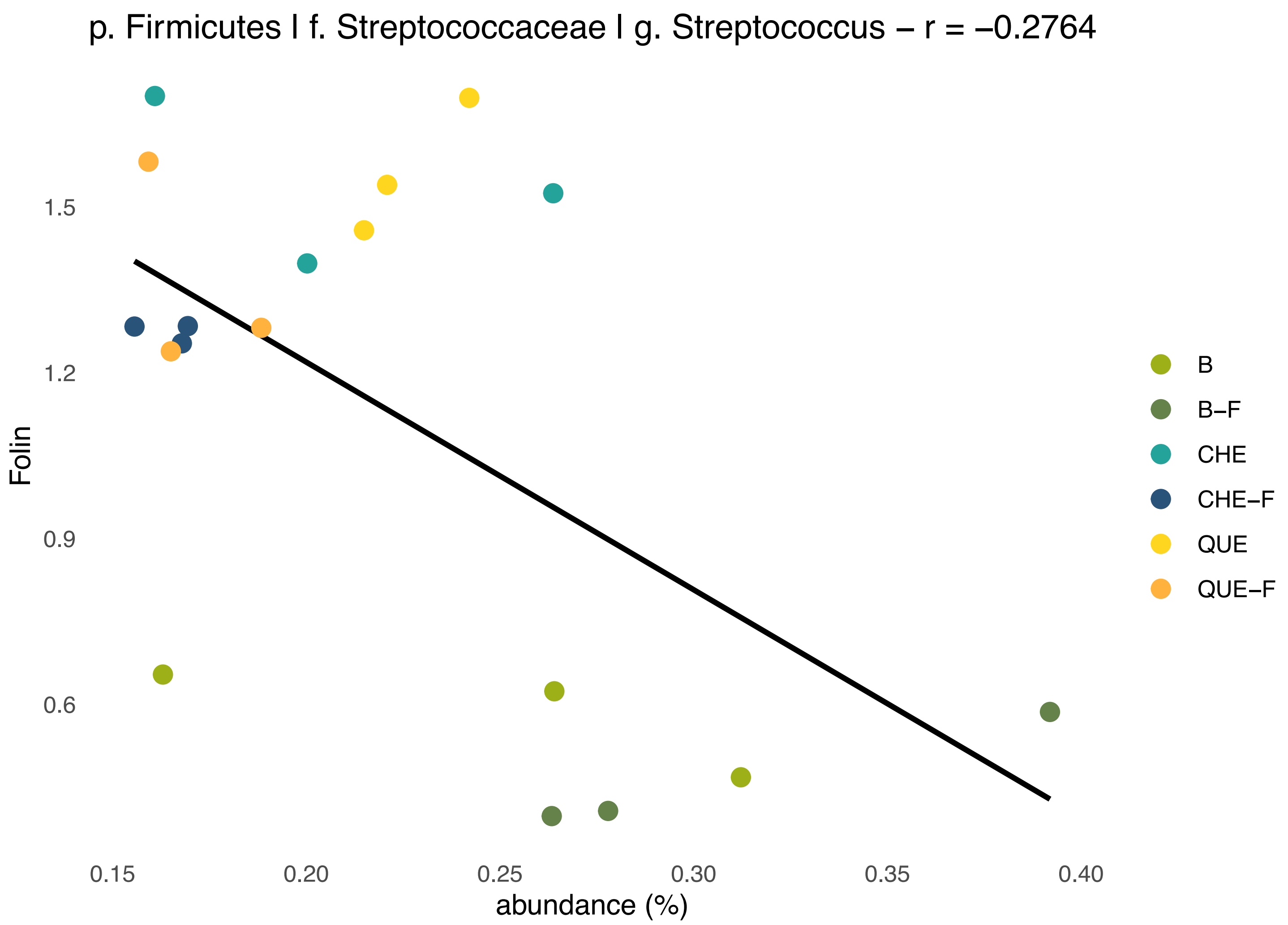
p. Firmicutes I f. Streptococcaceae I g. Streptococcus – r = –0.2764
Folin
1.5
1.2
0.9
0.6
0.15
0.20
0.25
0.30
0.35
0.40
abundance (%)
B
B–F
CHE
CHE–F
QUE
QUE–F

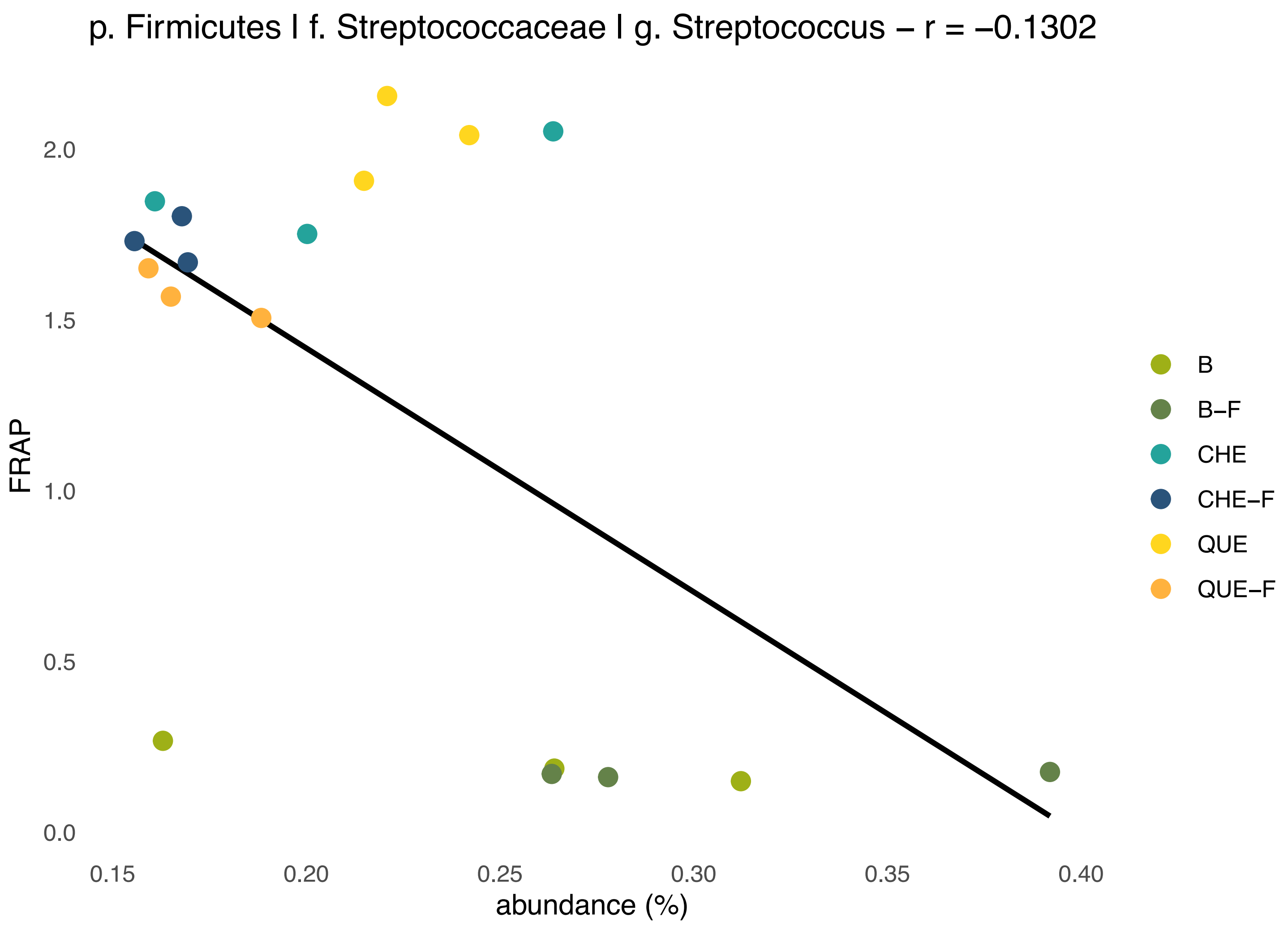
p. Firmicutes I f. Streptococcaceae I g. Streptococcus – r = –0.1302
FRAP
2.0
1.5
1.0
0.5
0.0
0.15
0.20
0.25
0.30
0.35
0.40
abundance (%)
B
B–F
CHE
CHE–F
QUE
QUE–F

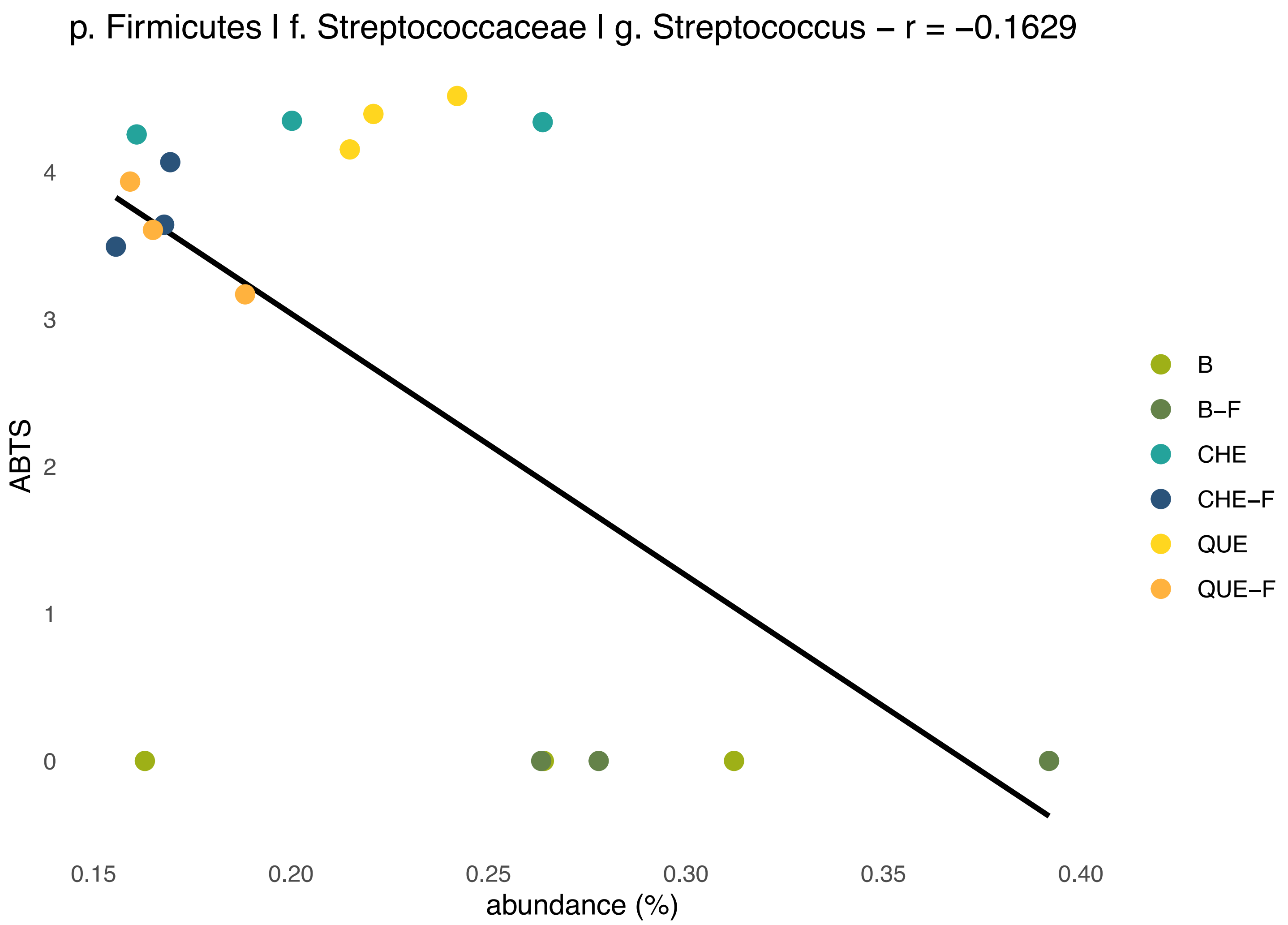

p. Firmicutes I f. Streptococcaceae I g. Streptococcus – r = –0.1629
ABTS
0
1
2
3
4
0.15
0.20
0.25
0.30
0.35
0.40
abundance (%)
B
B–F
CHE
CHE–F
QUE
QUE–F

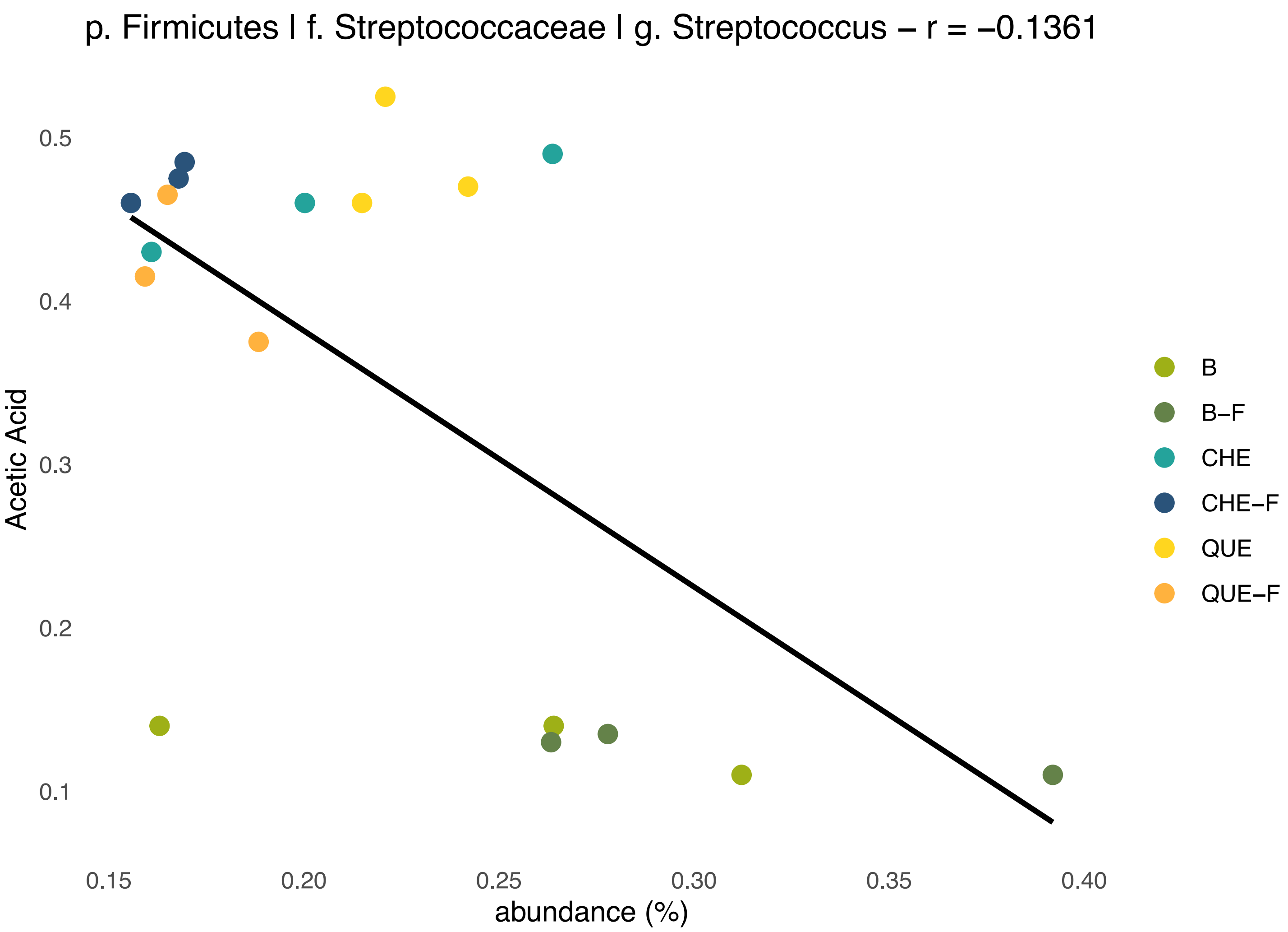
p. Firmicutes I f. Streptococcaceae I g. Streptococcus – r = –0.1361
Acetic Acid
0.5
0.4
0.3
0.2
0.1
0.15
0.20
0.25
0.30
0.35
0.40
abundance (%)
B
B–F
CHE
CHE–F
QUE
QUE–F

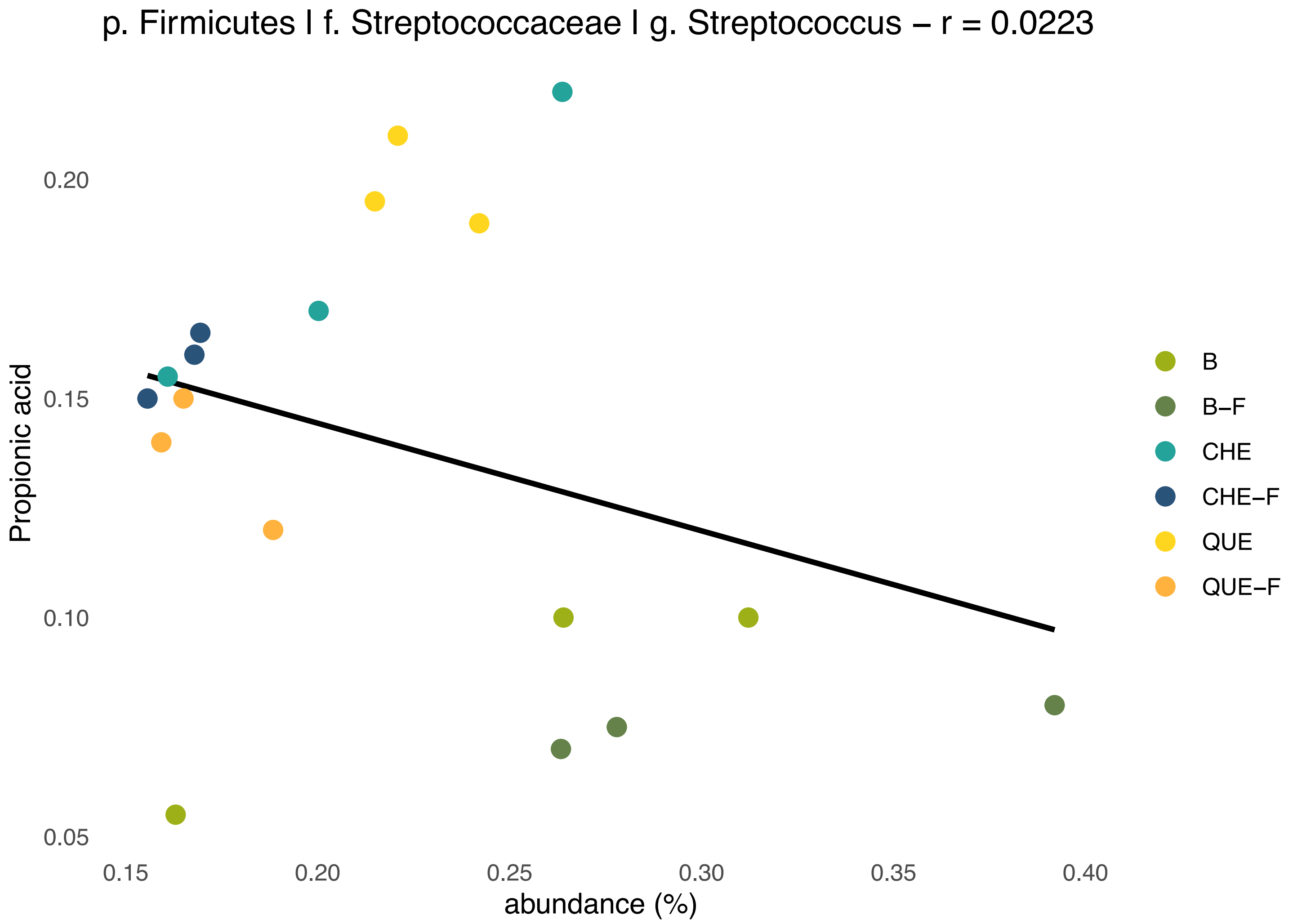
p. Firmicutes I f. Streptococcaceae I g. Streptococcus – r = 0.0223
Propionic acid
0.20
0.15
0.10
0.05
0.15
0.20
0.25
0.30
0.35
0.40
abundance (%)
B
B–F
CHE
CHE–F
QUE
QUE–F

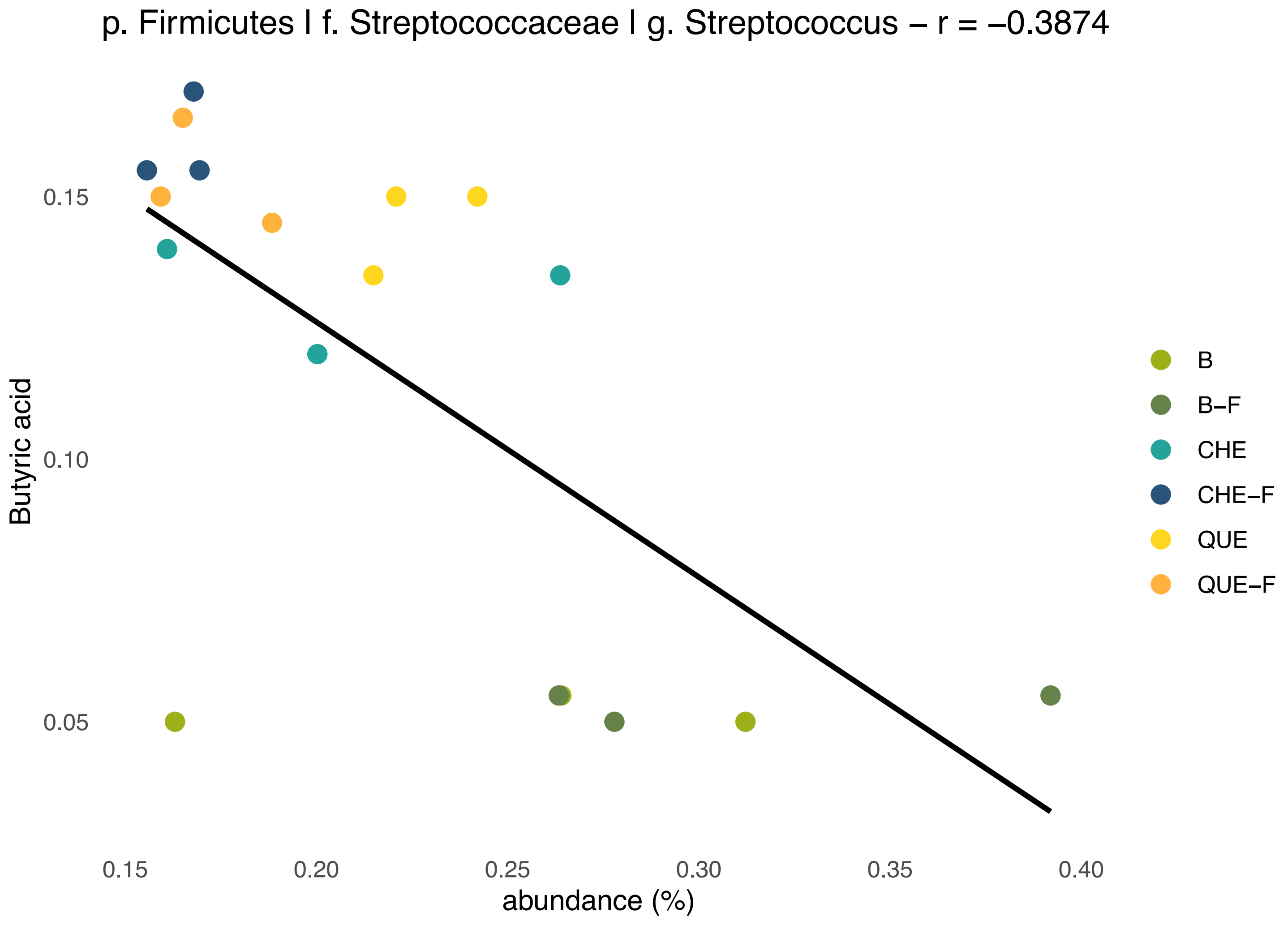
p. Firmicutes I f. Streptococcaceae I g. Streptococcus – r = –0.3874
Butyric acid
0.15
0.10
0.05
0.15
0.20
0.25
0.30
0.35
0.40
abundance (%)
B
B–F
CHE
CHE–F
QUE
QUE–F

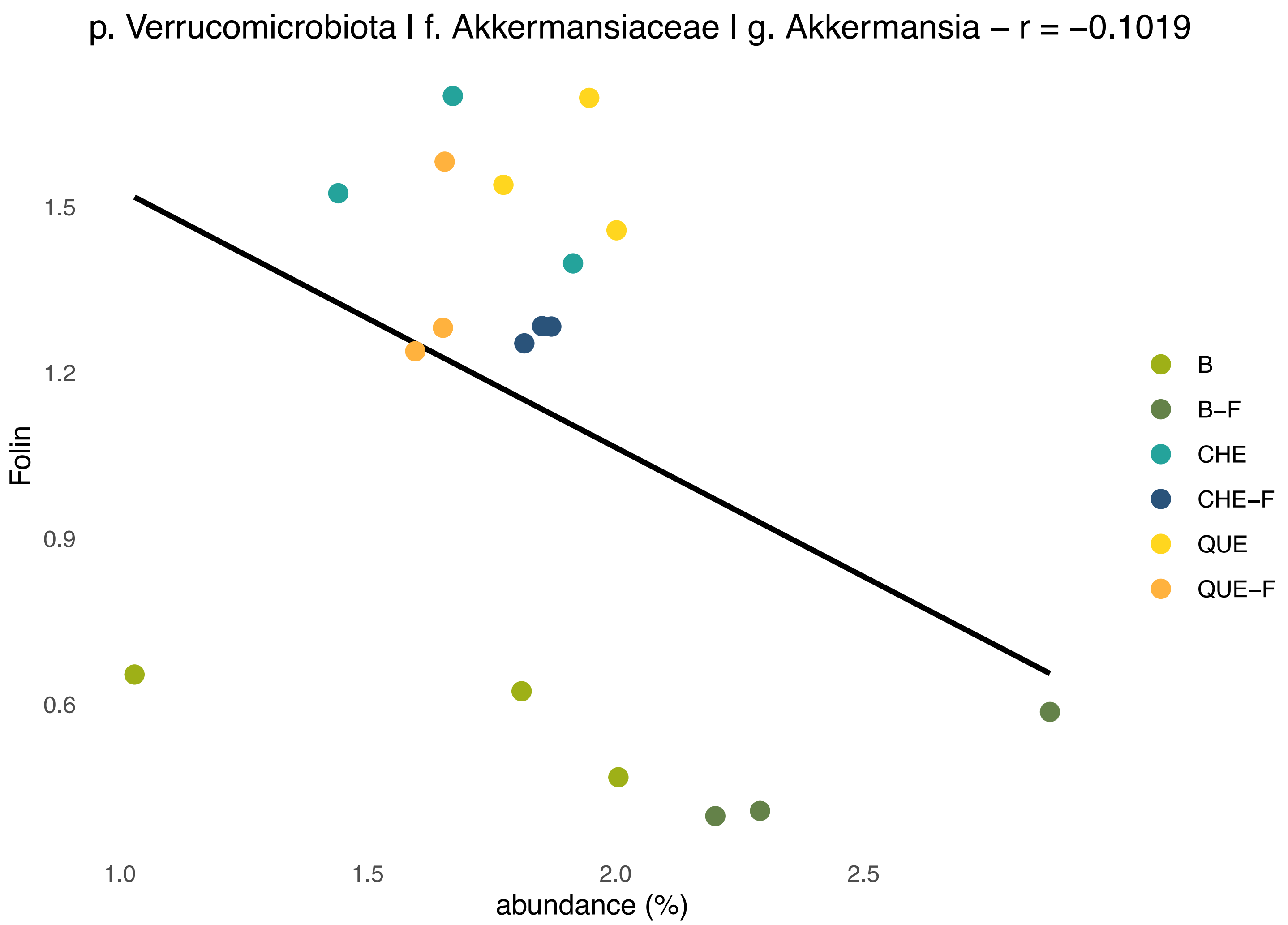

p. Verrucomicrobiota | f. Akkermansiaceae | g. Akkermansia – r = –0.1019
Folin
1.5
1.2
0.9
0.6
1.0
1.5
2.0
2.5
abundance (%)
B
B–F
CHE
CHE–F
QUE
QUE–F

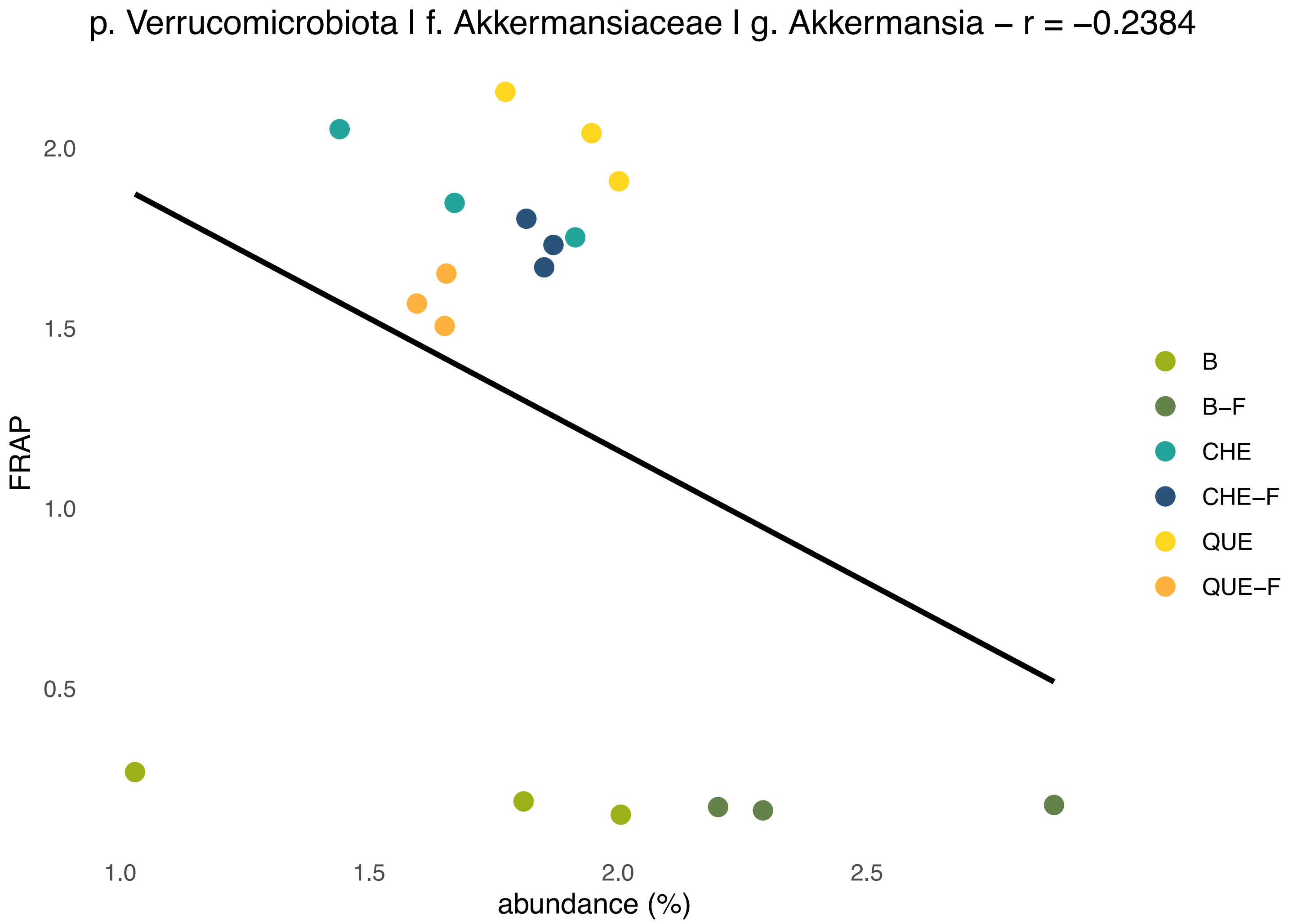

p. Verrucomicrobiota | f. Akkermansiaceae | g. Akkermansia – r = −0.2384
FRAP
2.0
1.5
1.0
0.5
1.0
1.5
2.0
2.5
abundance (%)
B
B−F
CHE
CHE−F
QUE
QUE−F

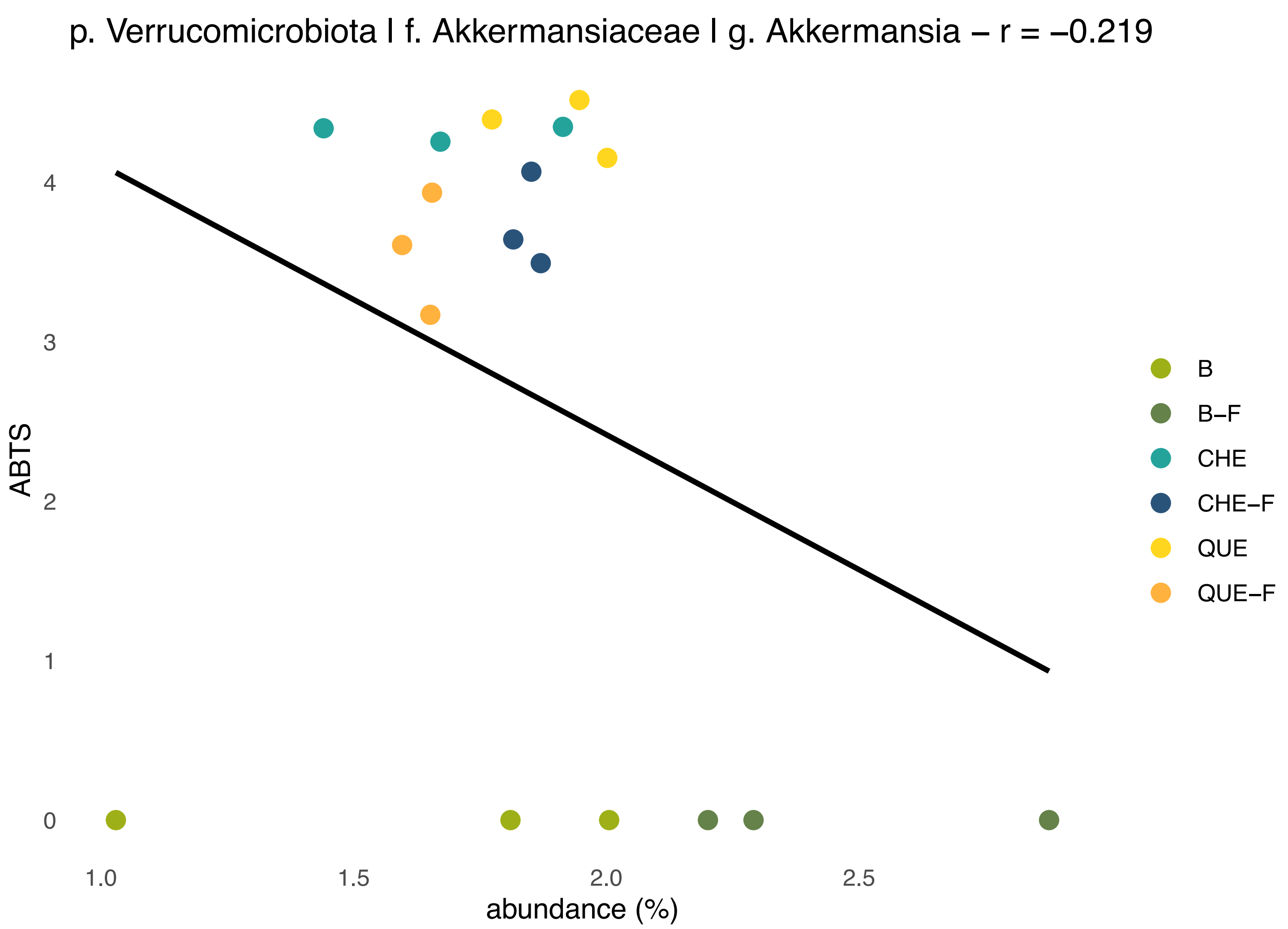

p. Verrucomicrobiota I f. Akkermansiaceae I g. Akkermansia – r = –0.219
ABTS
0
1
2
3
4
1.0
1.5
2.0
2.5
abundance (%)
B
B–F
CHE
CHE–F
QUE
QUE–F

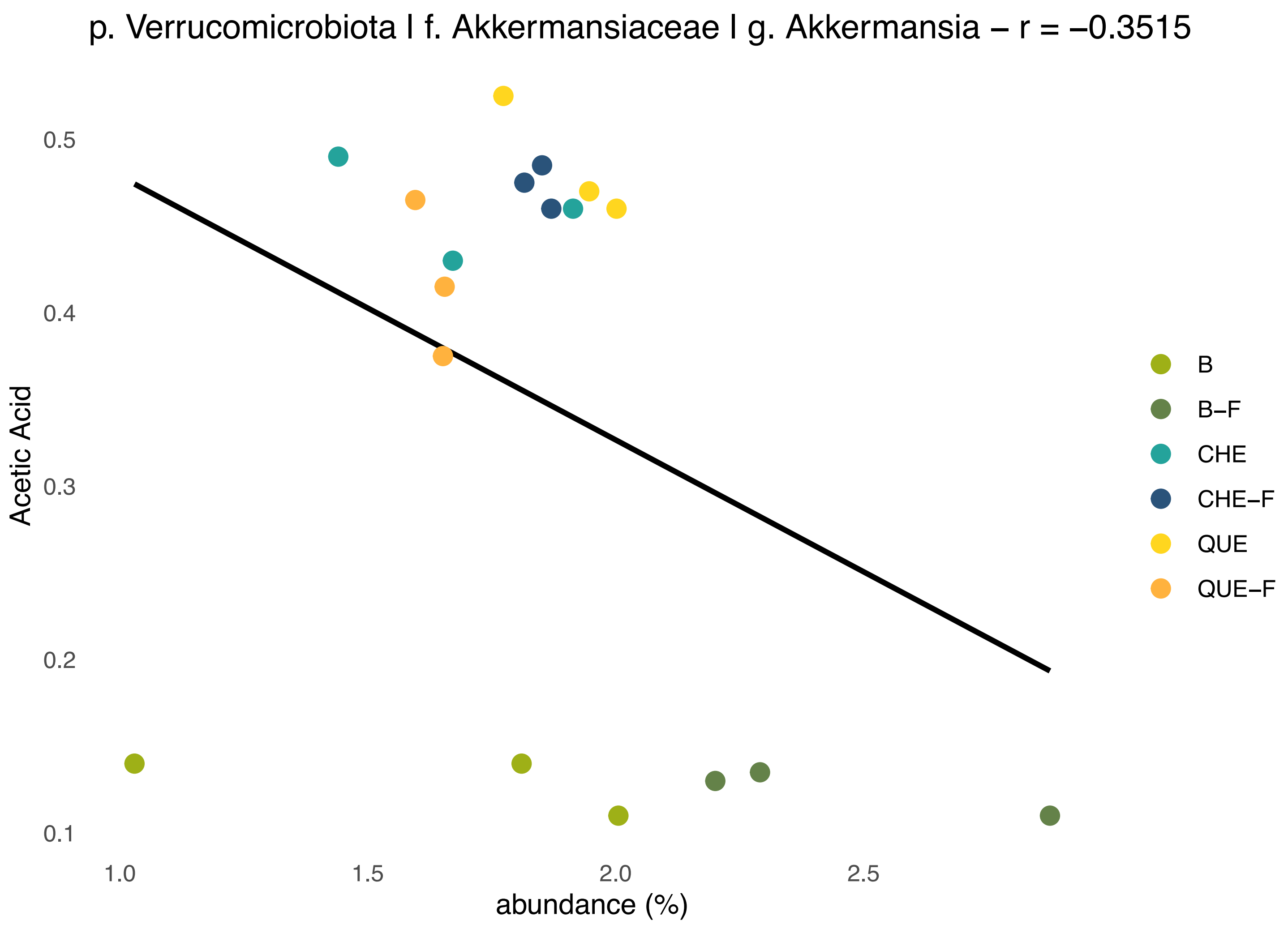
p. Verrucomicrobiota I f. Akkermansiaceae I g. Akkermansia – r = –0.3515
Acetic Acid
0.1
0.2
0.3
0.4
0.5
abundance (%)
1.0
1.5
2.0
2.5
B
B–F
CHE
CHE–F
QUE
QUE–F

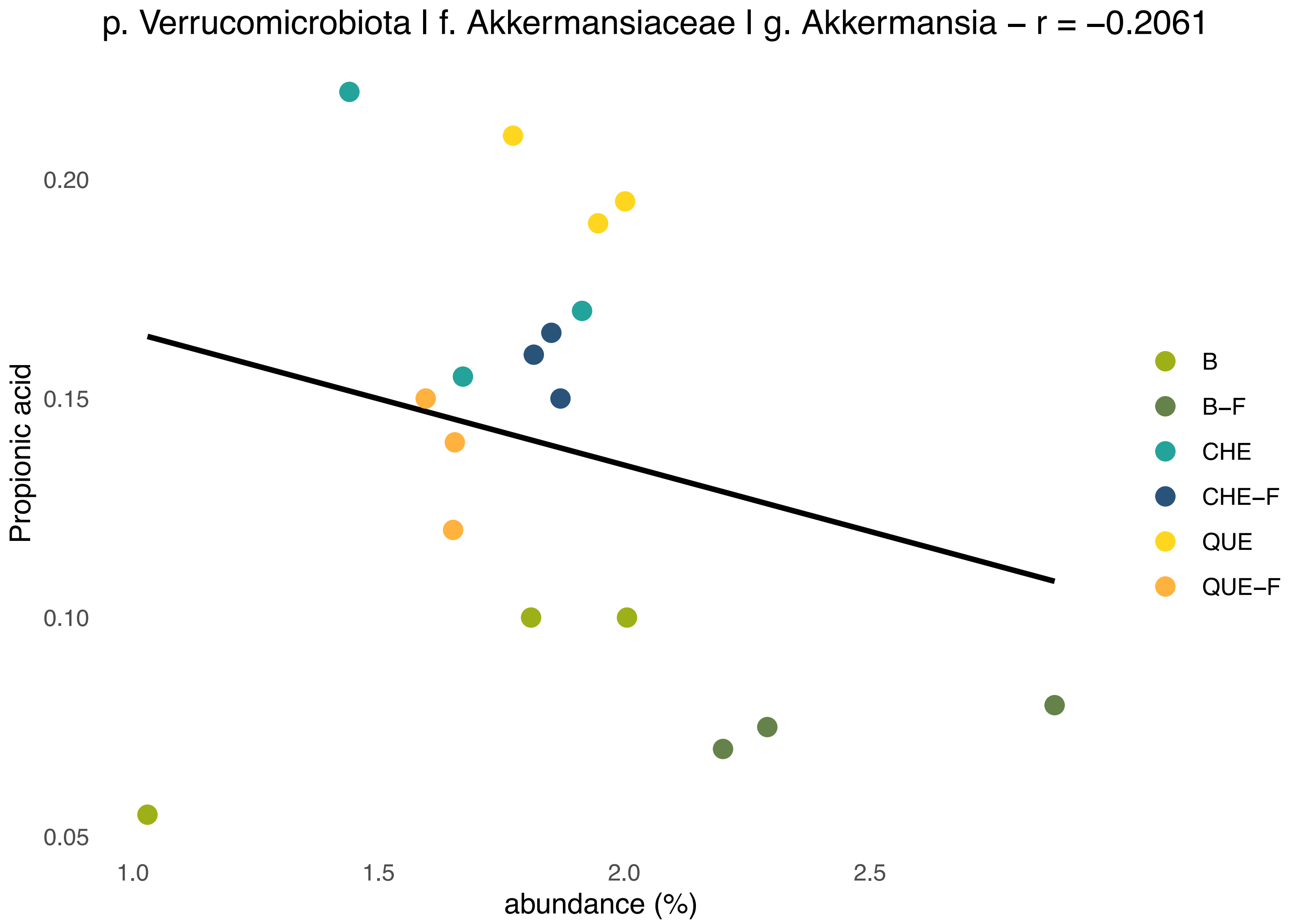

p. Verrucomicrobiota | f. Akkermansiaceae | g. Akkermansia – r = –0.2061
Propionic acid
0.20
0.15
0.10
0.05
1.0
1.5
2.0
2.5
abundance (%)
B
B–F
CHE
CHE–F
QUE
QUE–F

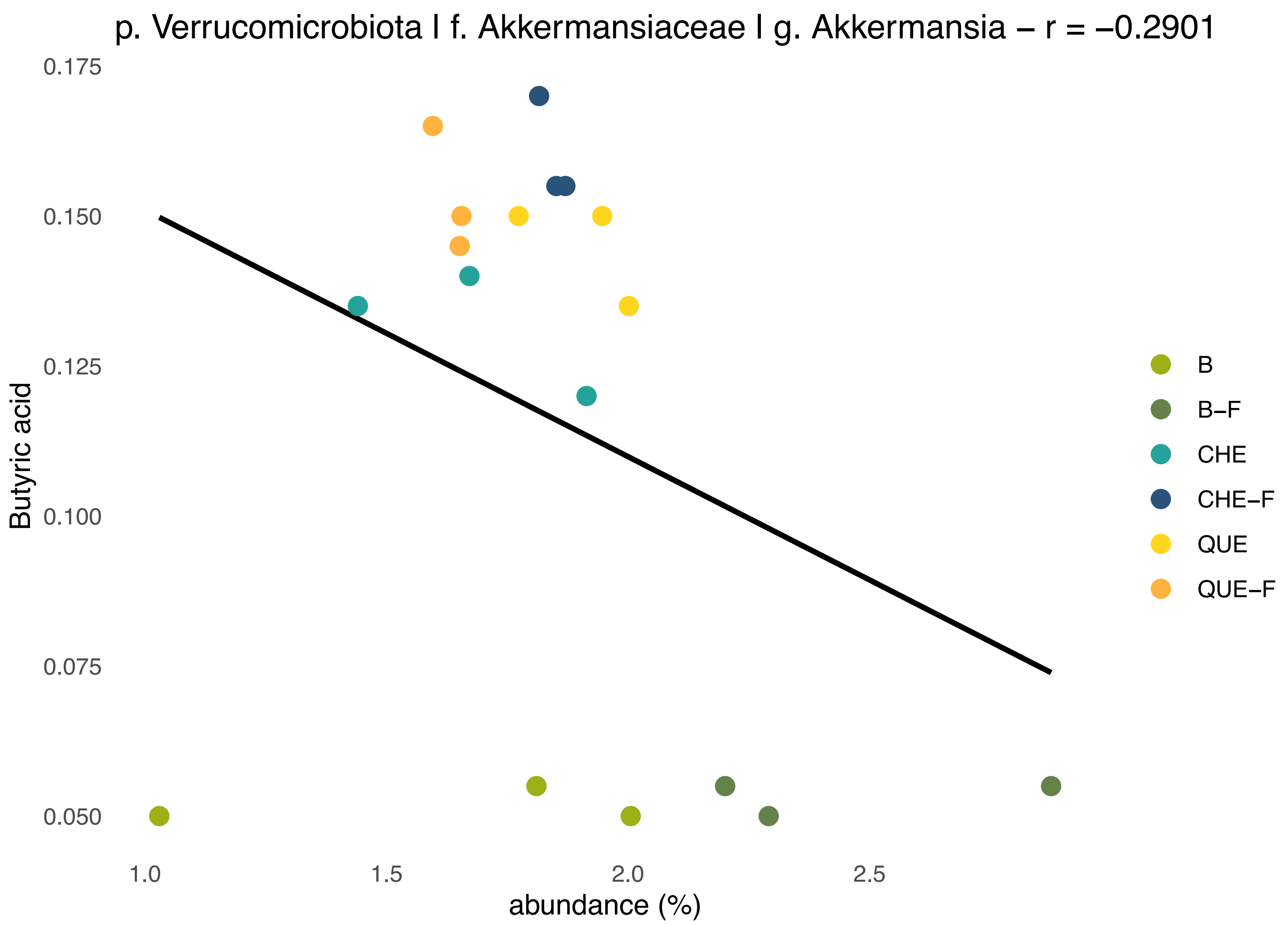
p. Verrucomicrobiota I f. Akkermansiaceae I g. Akkermansia – r = –0.2901
0.175
0.150
0.125
0.100
0.075
0.050
Butyric acid
1.0
1.5
2.0
2.5
abundance (%)
B
B–F
CHE
CHE–F
QUE
QUE–F

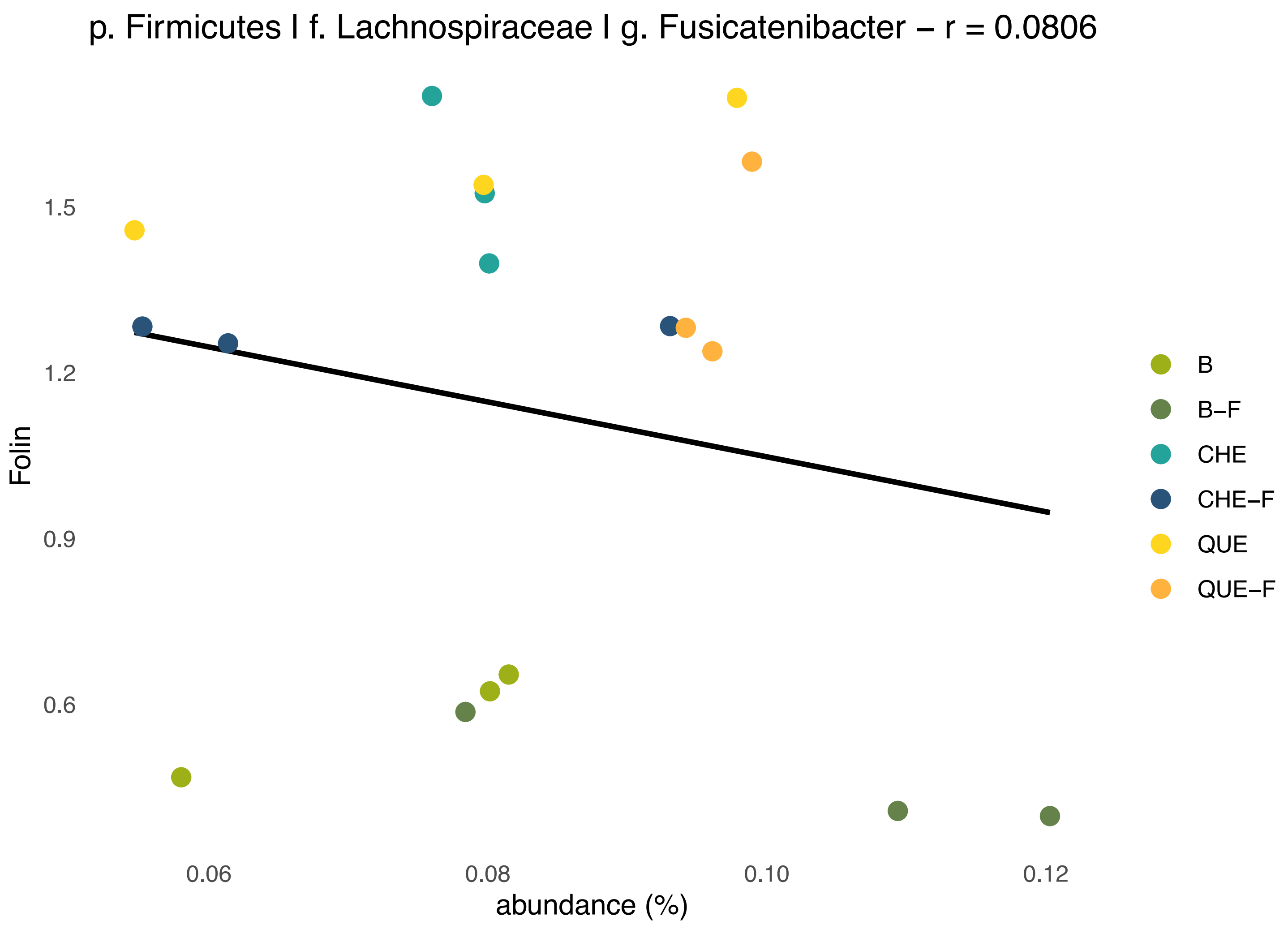

p. Firmicutes l f. Lachnospiraceae l g. Fusicatenibacter – r = 0.0806
1.5
1.2
0.9
0.6
Folin
0.06
0.08
0.10
0.12
abundance (%)
B
B–F
CHE
CHE–F
QUE
QUE–F

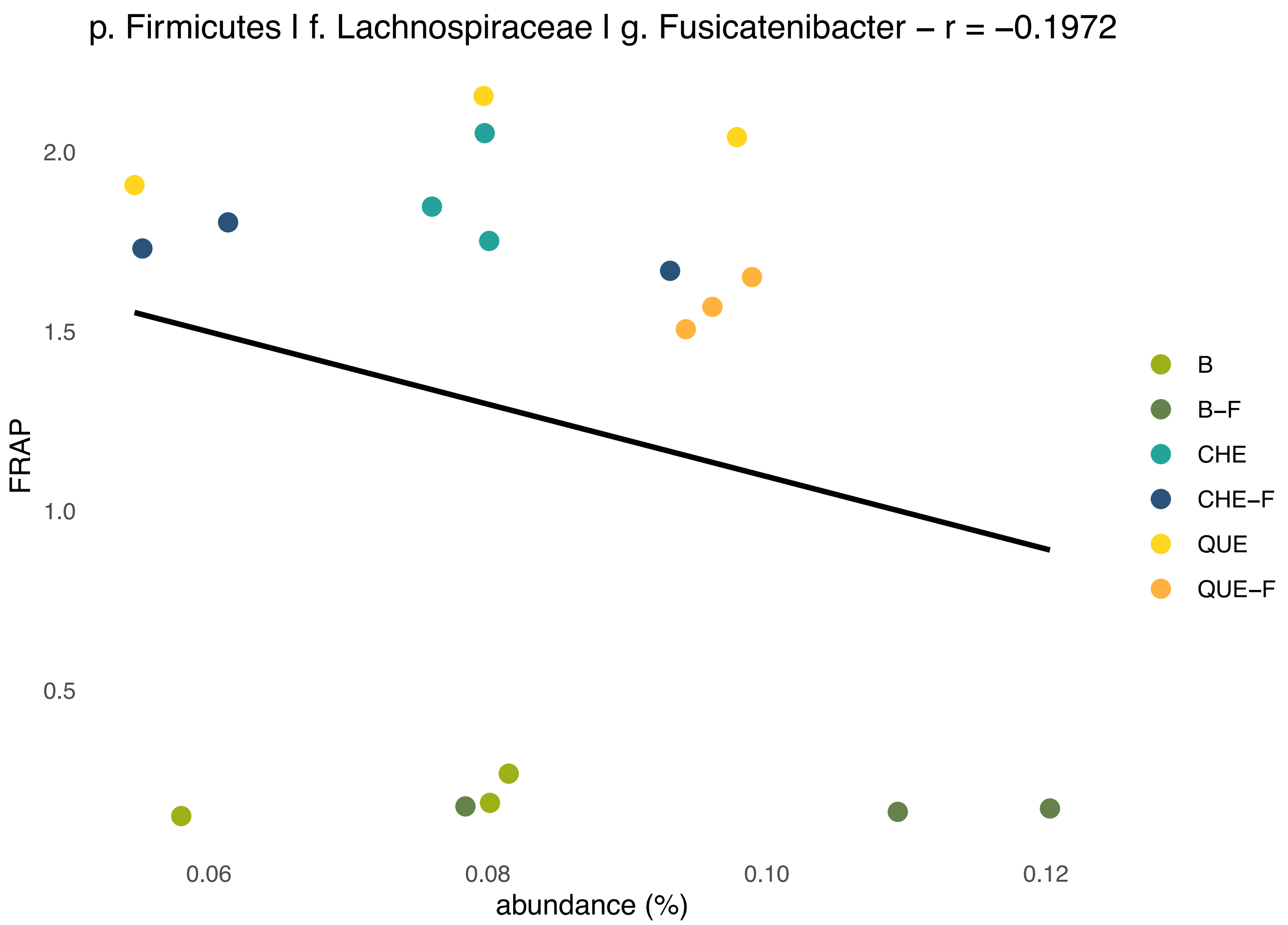
p. Firmicutes I f. Lachnospiraceae I g. Fusicatenibacter – r = −0.1972
FRAP
2.0
1.5
1.0
0.5
0.06
0.08
0.10
0.12
abundance (%)
B
B−F
CHE
CHE−F
QUE
QUE−F

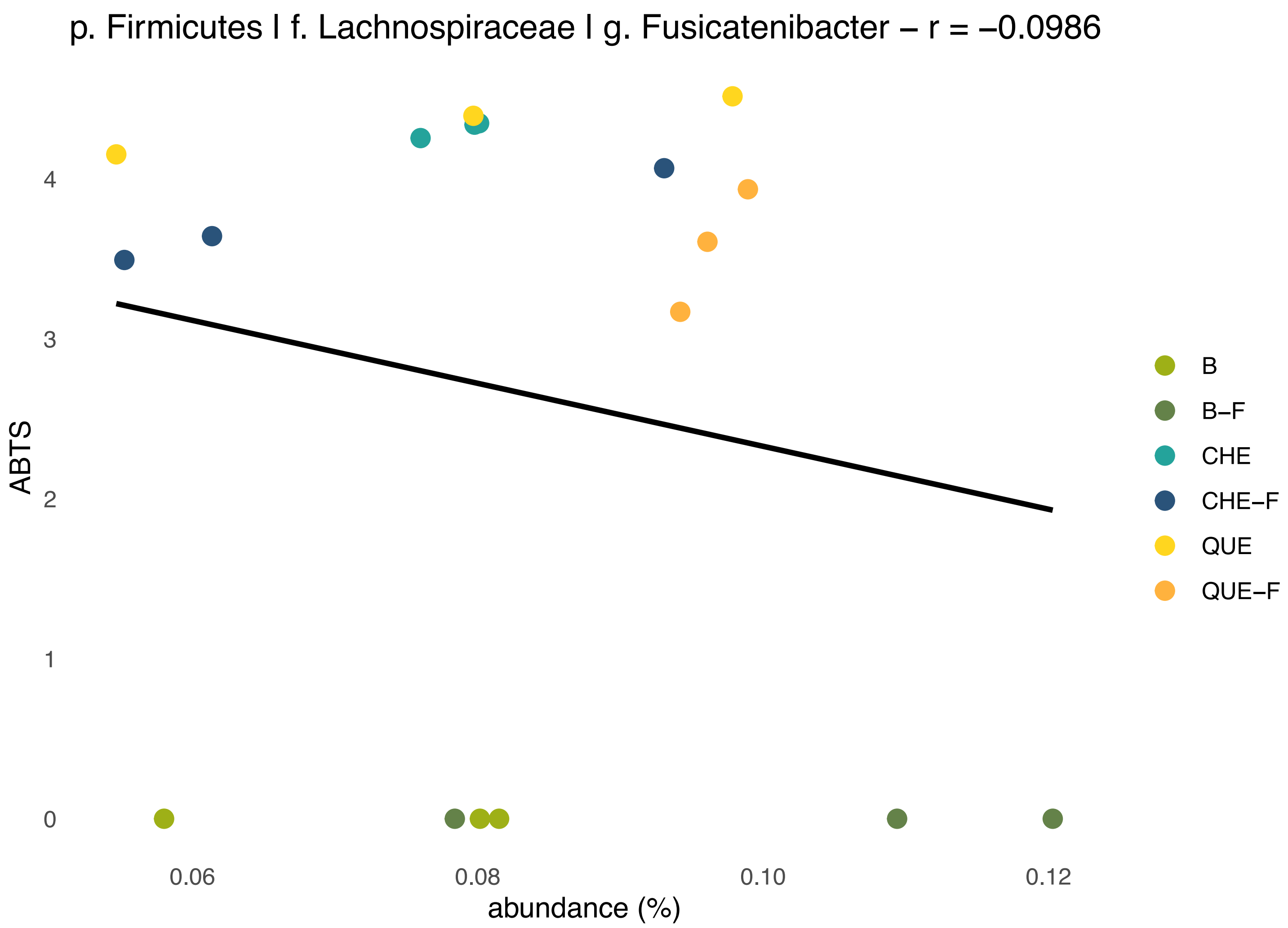

p. Firmicutes I f. Lachnospiraceae I g. Fusicatenibacter – r = –0.0986
ABTS
0
1
2
3
4
0.06
0.08
0.10
0.12
abundance (%)
B
B–F
CHE
CHE–F
QUE
QUE–F

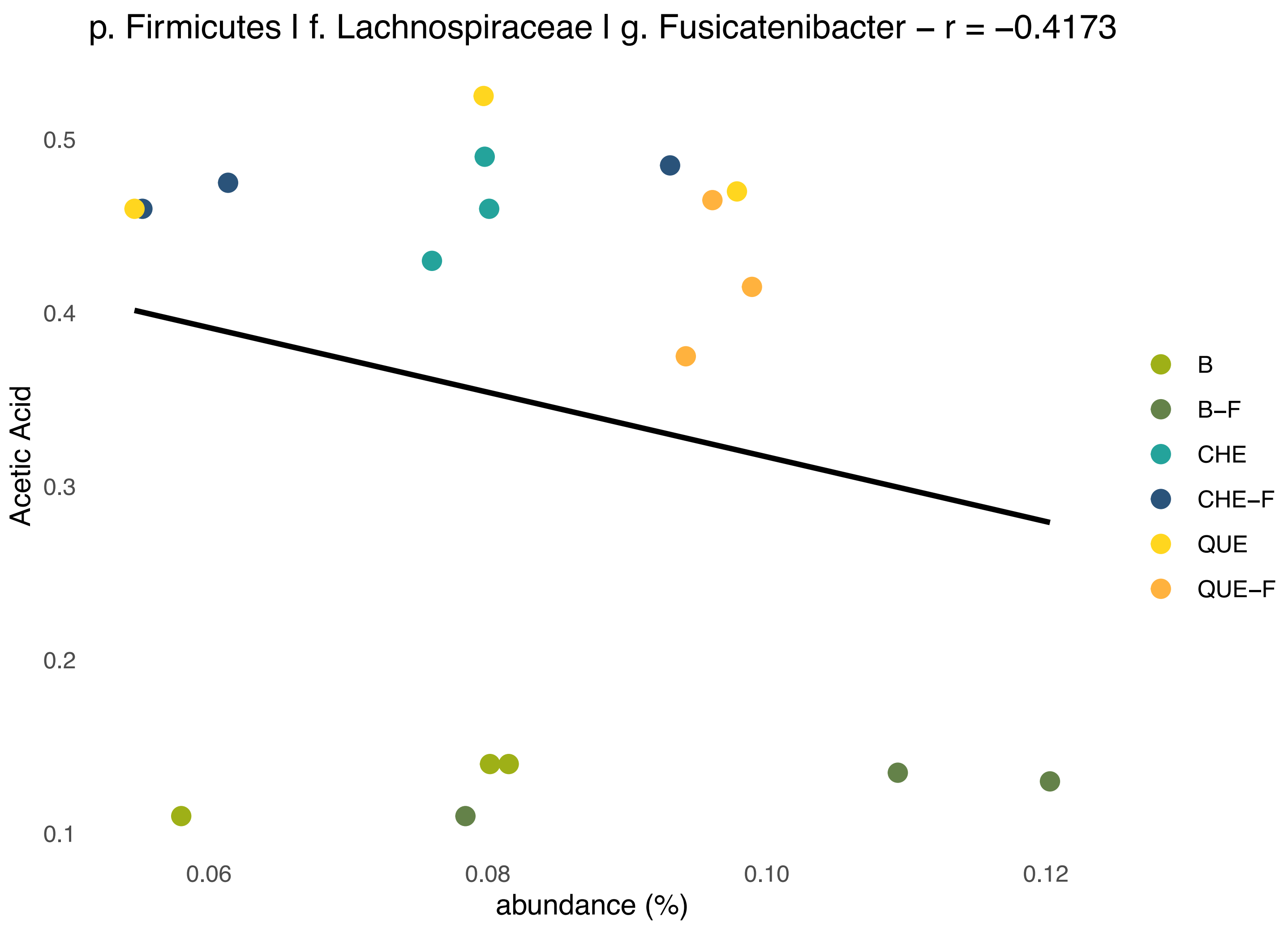
p. Firmicutes | f. Lachnospiraceae | g. Fusicatenibacter – r = –0.4173
Acetic Acid
0.5
0.4
0.3
0.2
0.1
0.06
0.08
0.10
0.12
abundance (%)
B
B–F
CHE
CHE–F
QUE
QUE–F

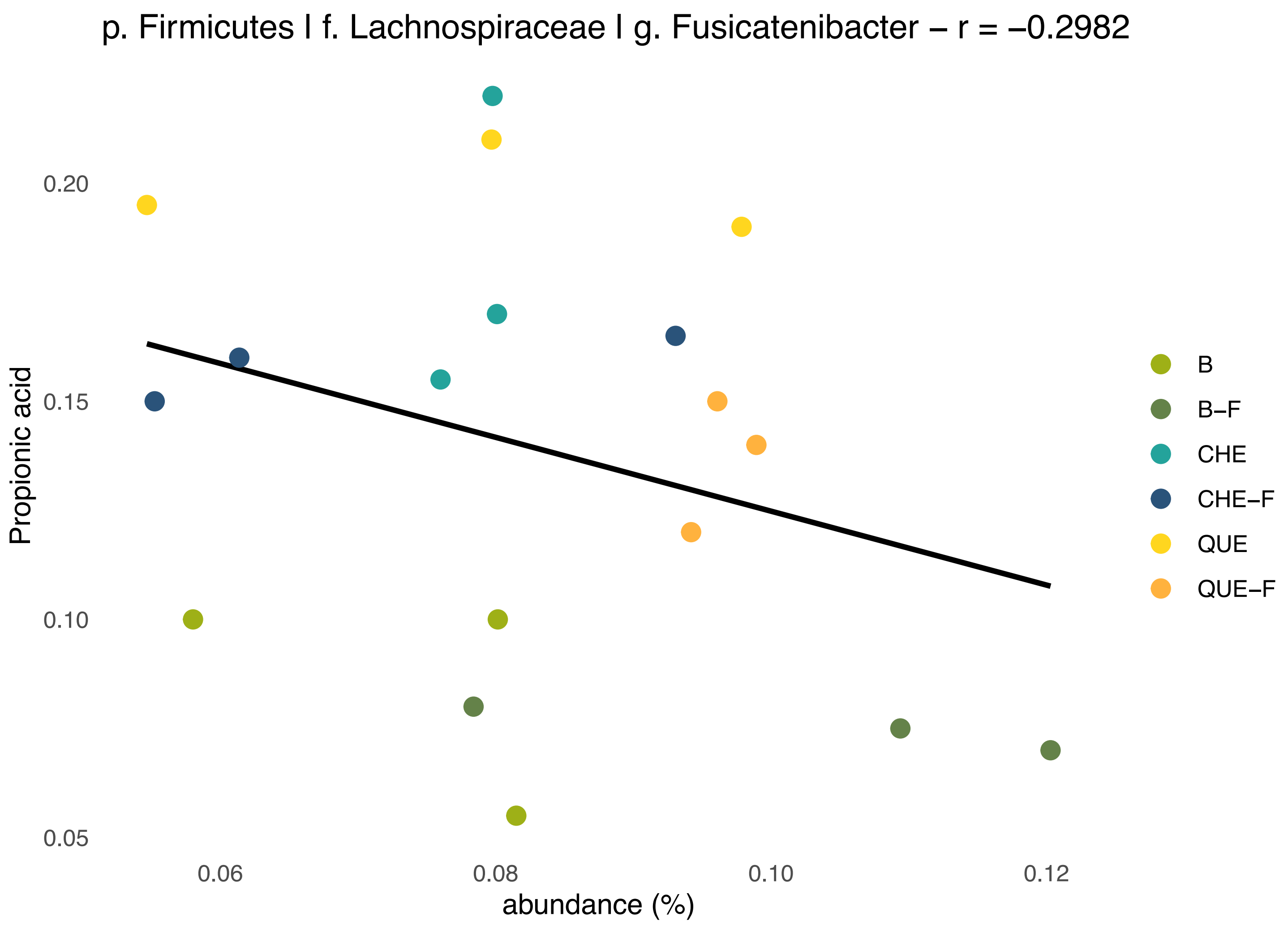
p. Firmicutes I f. Lachnospiraceae I g. Fusicatenibacter – r = –0.2982
Propionic acid
0.20
0.15
0.10
0.05
0.06
0.08
0.10
0.12
abundance (%)
B
B–F
CHE
CHE–F
QUE
QUE–F

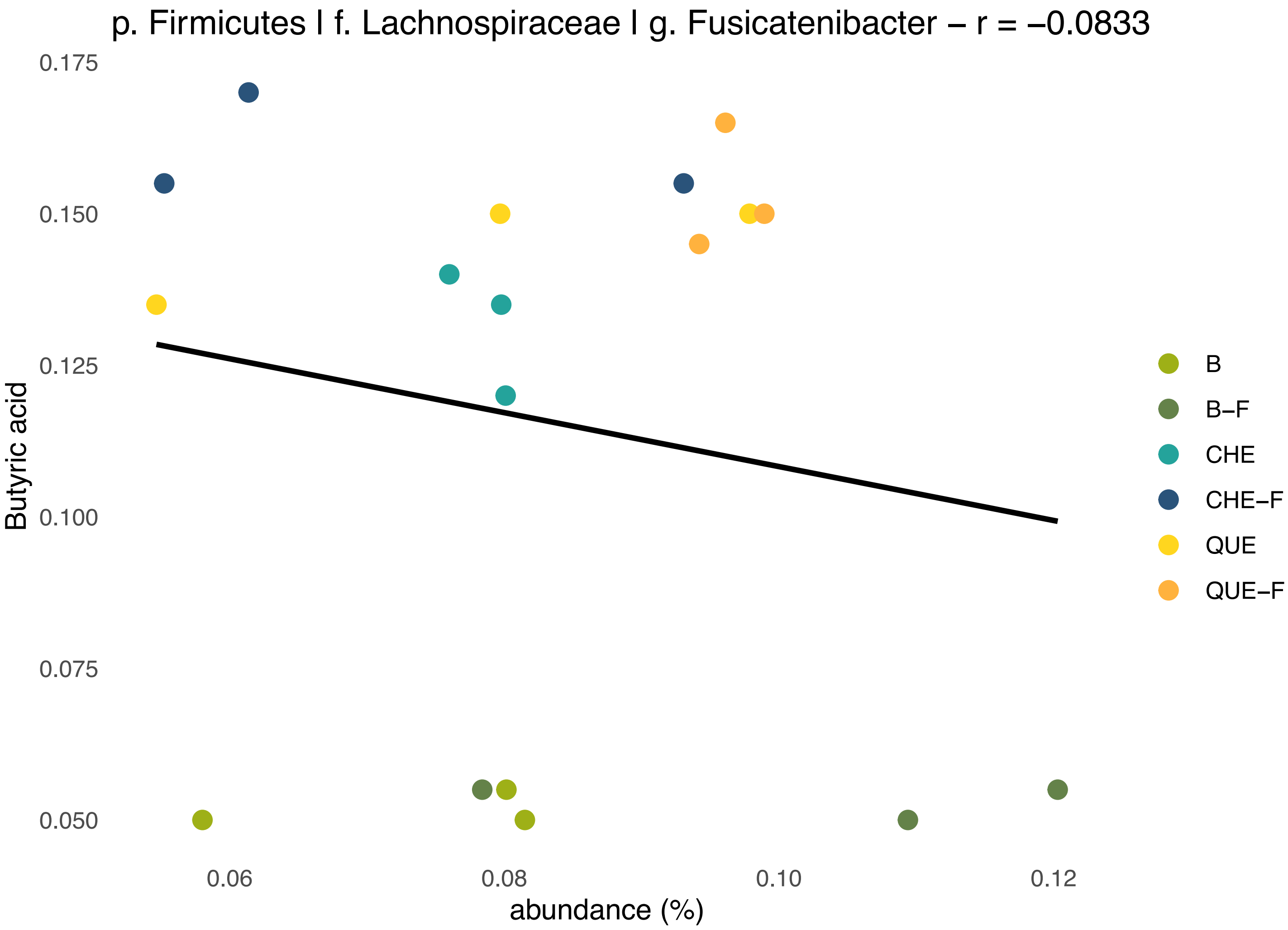
p. Firmicutes l f. Lachnospiraceae l g. Fusicatenibacter – r = –0.0833
0.175
0.150
0.125
0.100
0.075
0.050
Butyric acid
0.06
0.08
0.10
0.12
abundance (%)
B
B–F
CHE
CHE–F
QUE
QUE–F

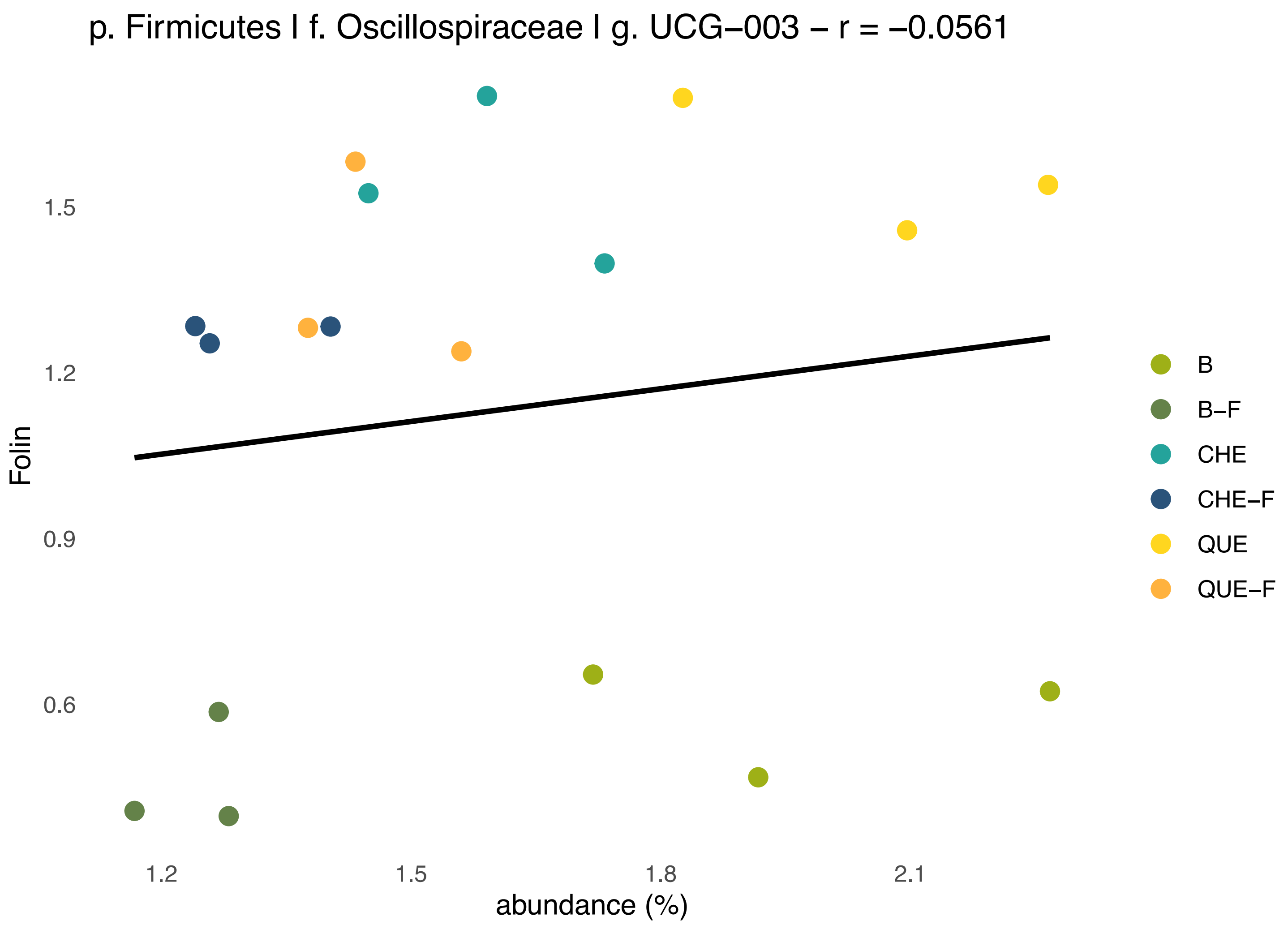

p. Firmicutes I f. Oscillospiraceae I g. UCG−003 − r = −0.0561
Folin
1.5
1.2
0.9
0.6
1.2
1.5
1.8
2.1
abundance (%)
B
B−F
CHE
CHE−F
QUE
QUE−F

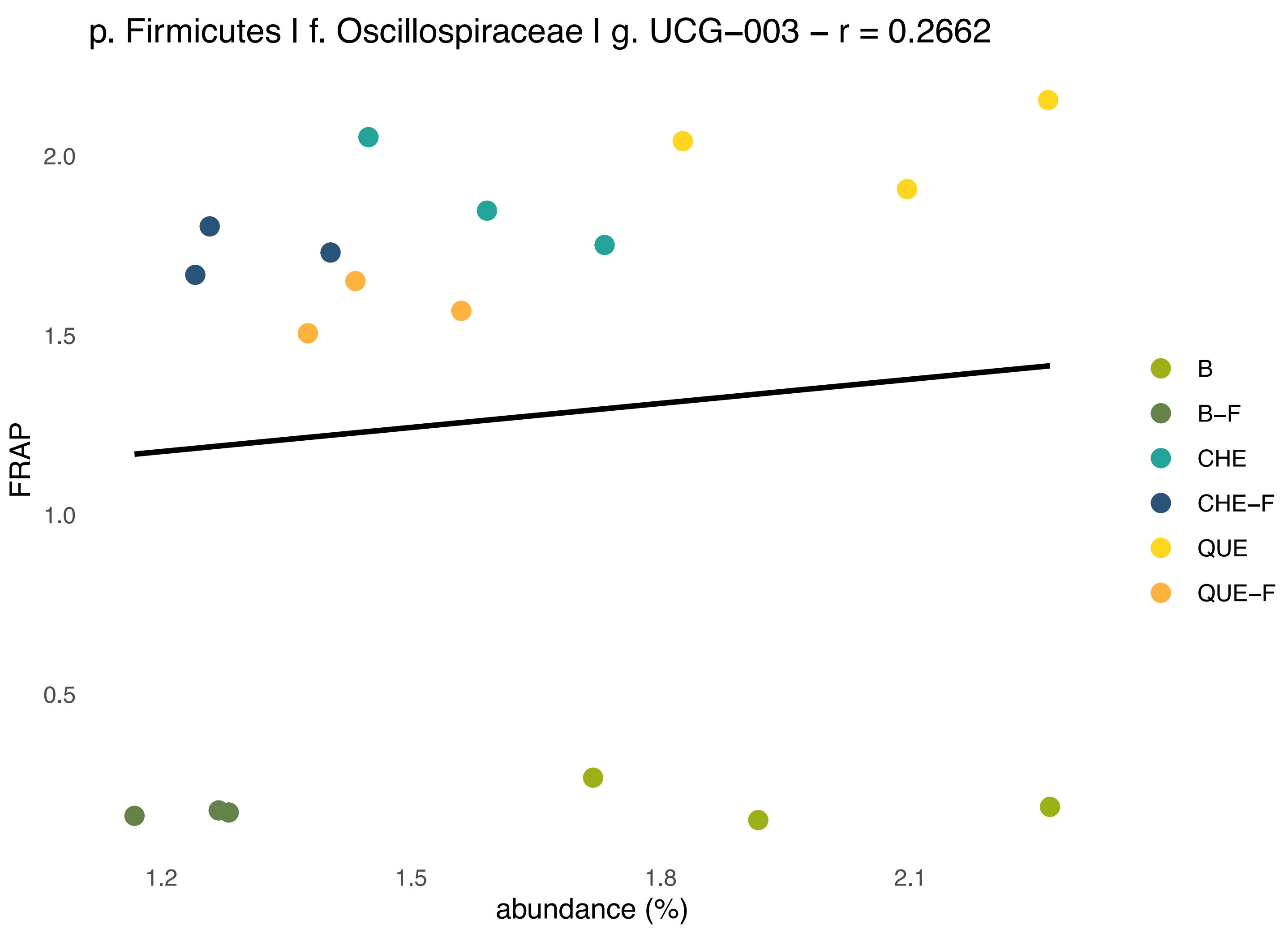

p. Firmicutes | f. Oscillospiraceae | g. UCG−003 − r = 0.2662
2.0
1.5
1.0
0.5
FRAP
1.2
1.5
1.8
2.1
abundance (%)
B
B−F
CHE
CHE−F
QUE
QUE−F

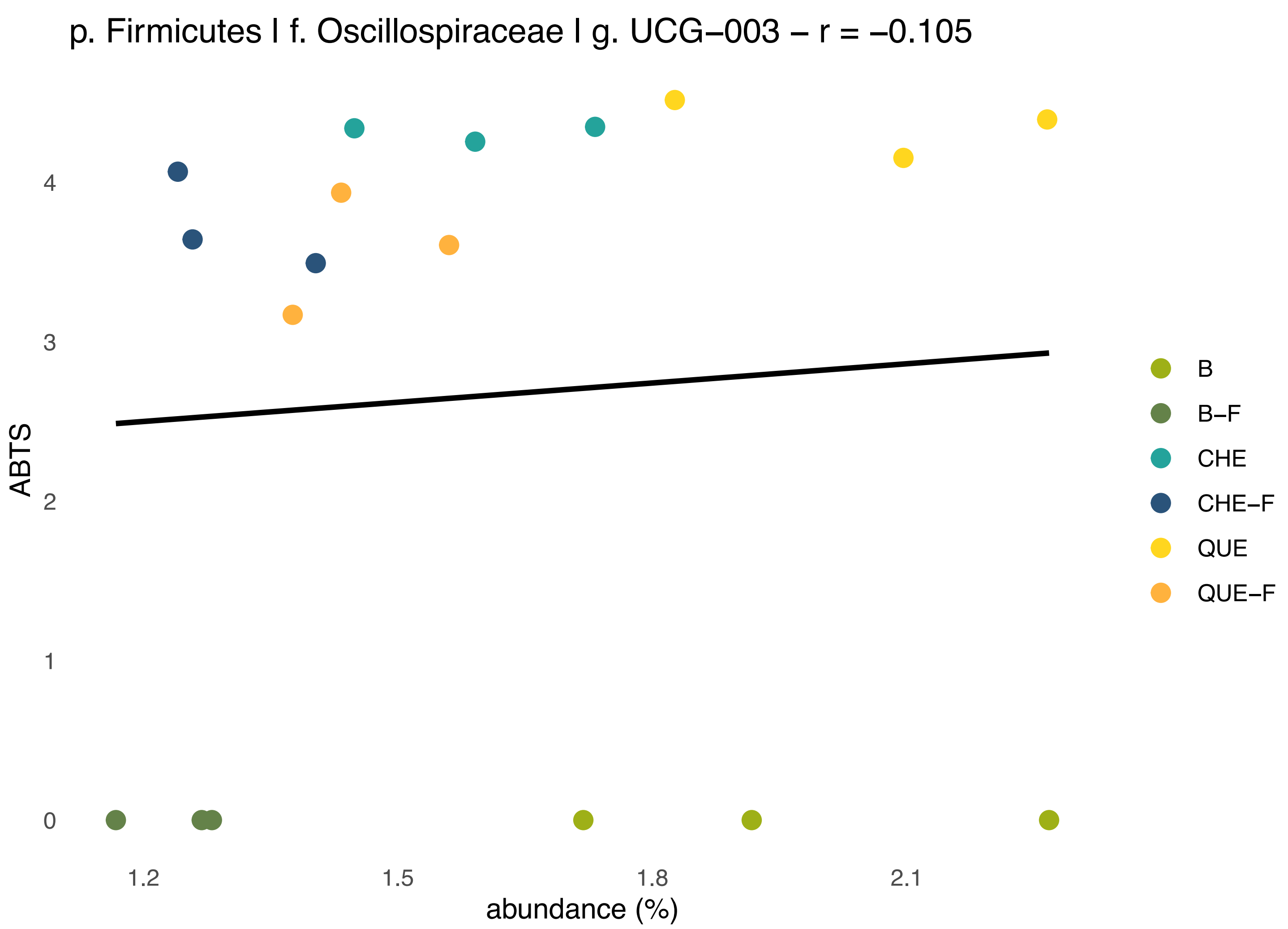

p. Firmicutes I f. Oscillospiraceae I g. UCG−003 − r = −0.105
ABTS
4
3
2
1
0
1.2
1.5
1.8
2.1
abundance (%)
B
B−F
CHE
CHE−F
QUE
QUE−F

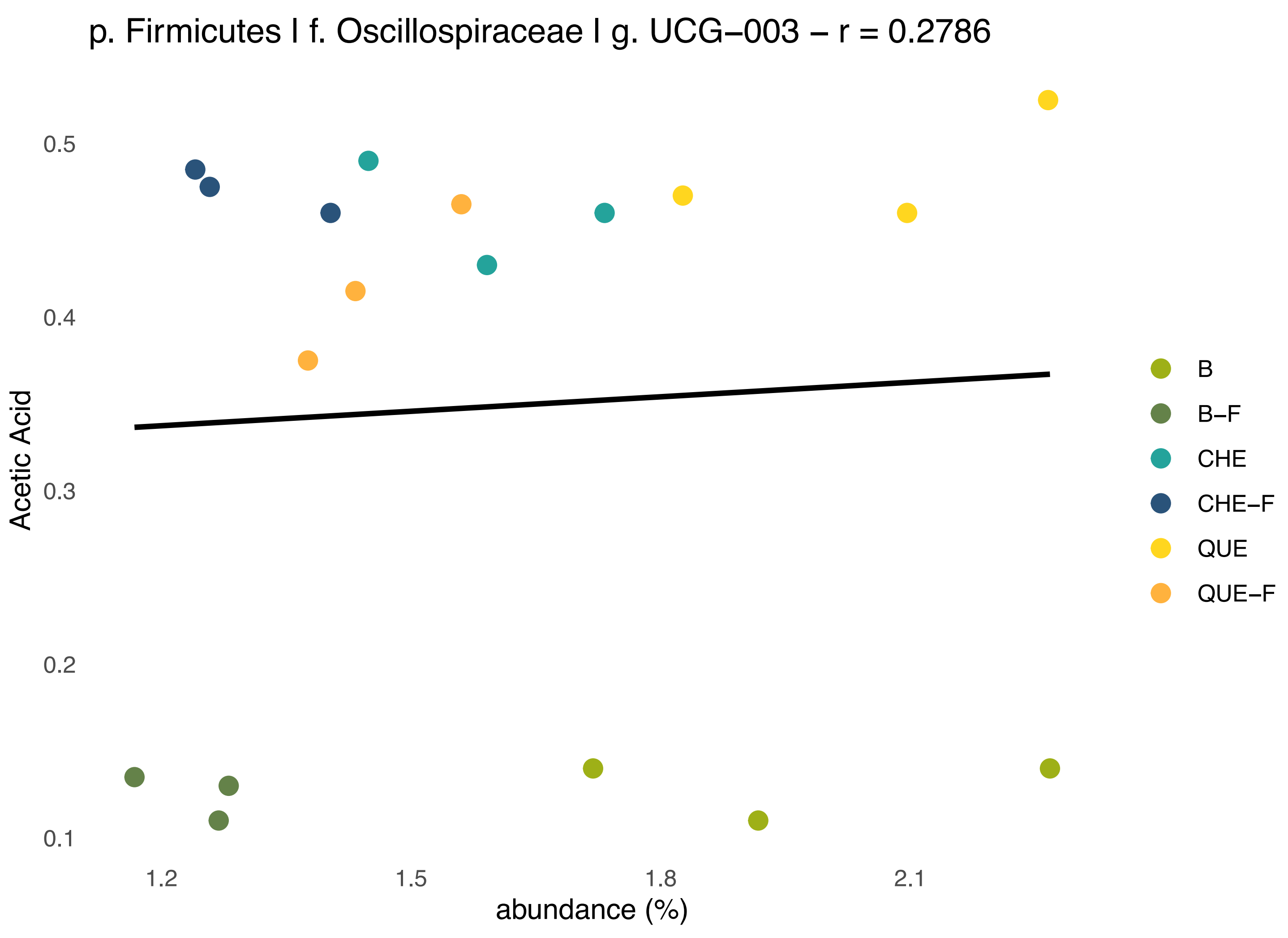
p. Firmicutes I f. Oscillospiraceae I g. UCG−003 − r = 0.2786
Acetic Acid
0.5
0.4
0.3
0.2
0.1
1.2
1.5
1.8
2.1
abundance (%)
B
B−F
CHE
CHE−F
QUE
QUE−F

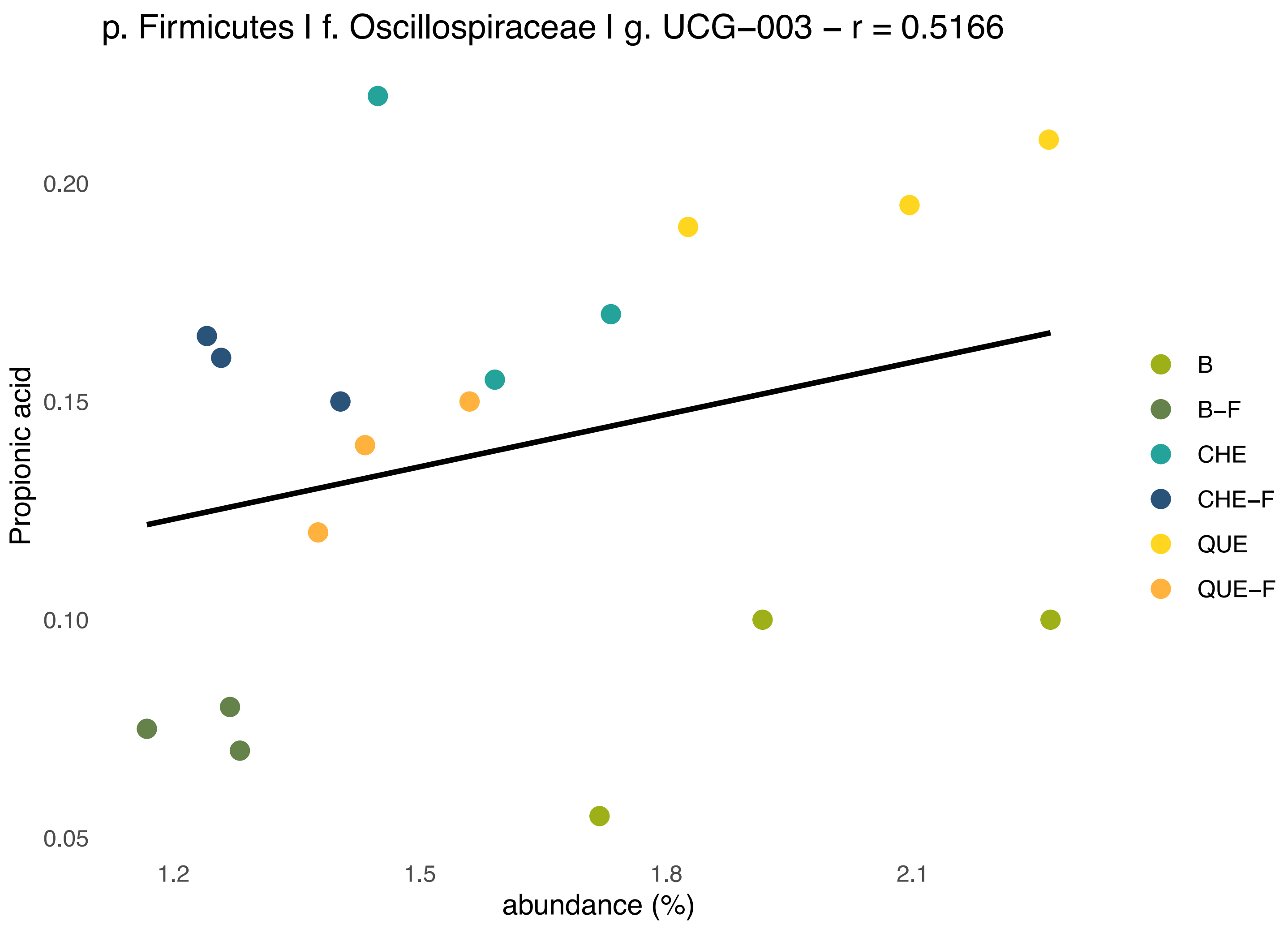
p. Firmicutes I f. Oscillospiraceae I g. UCG−003 − r = 0.5166
Propionic acid
0.20
0.15
0.10
0.05
1.2
1.5
1.8
2.1
abundance (%)
B
B−F
CHE
CHE−F
QUE
QUE−F

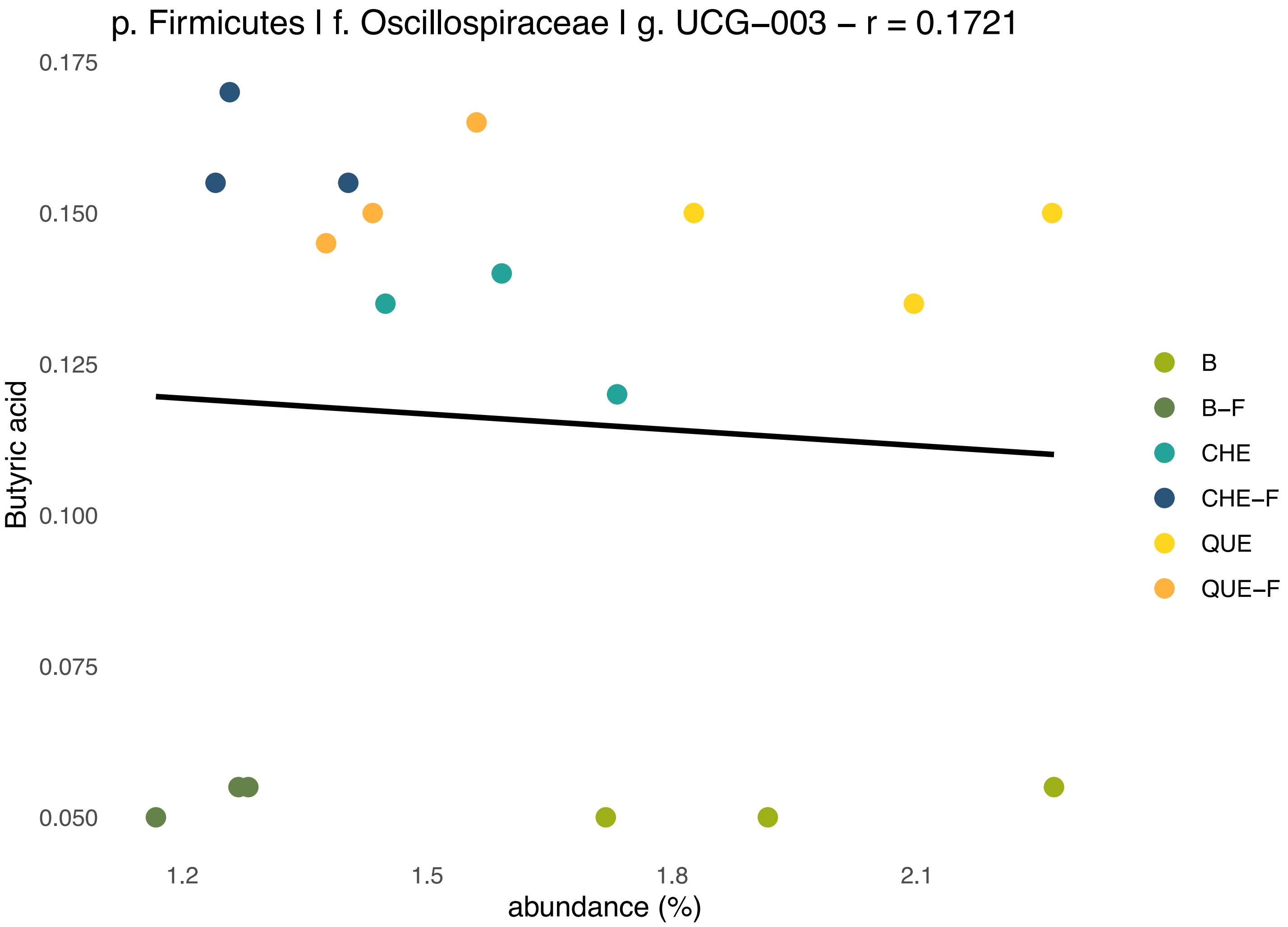
p. Firmicutes I f. Oscillospiraceae I g. UCG−003 − r = 0.1721
0.175
0.150
0.125
0.100
0.075
0.050
Butyric acid
1.2
1.5
1.8
2.1
abundance (%)
B
B−F
CHE
CHE−F
QUE
QUE−F

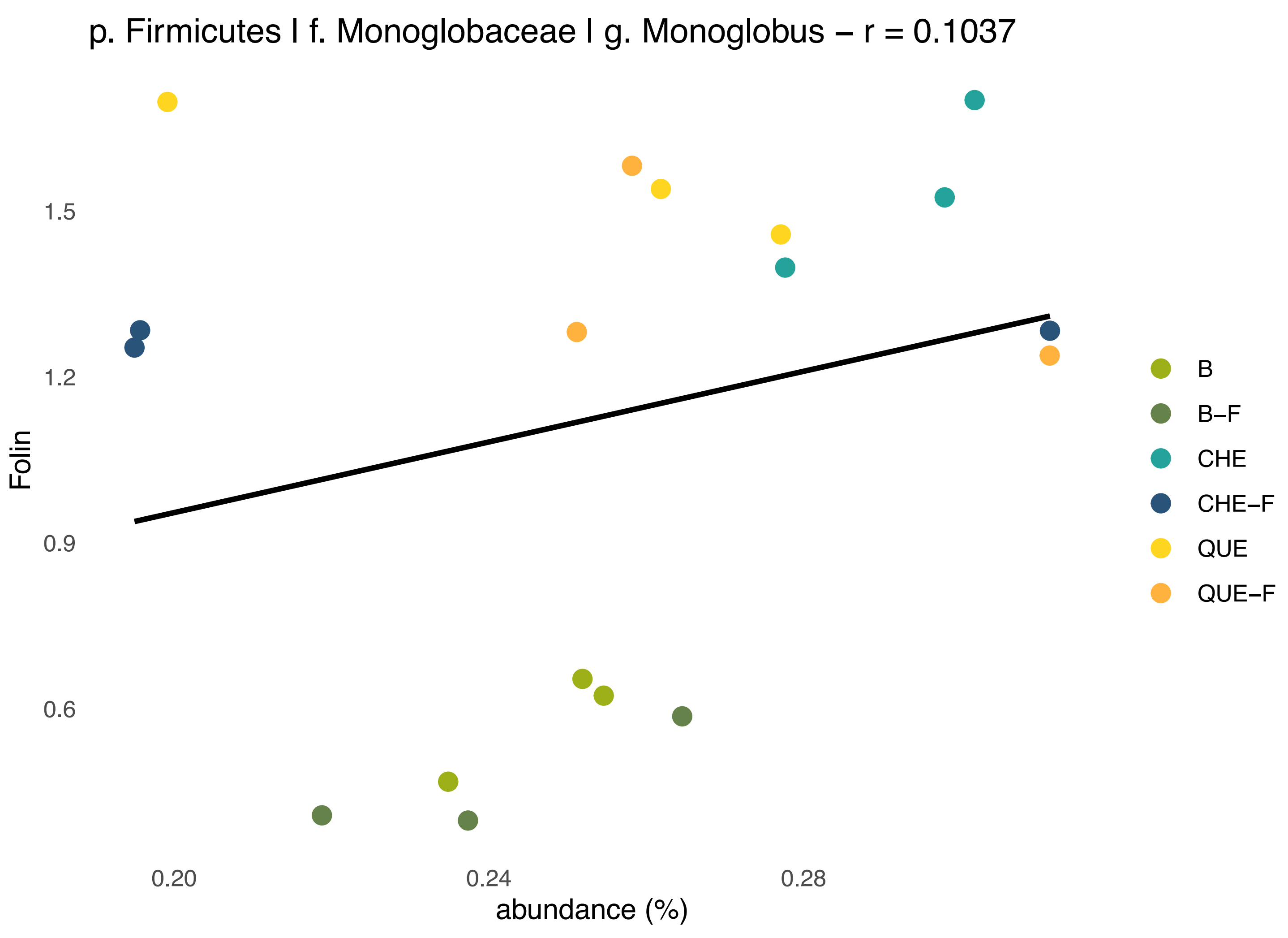
p. Firmicutes I f. Monoglobaceae I g. Monoglobus – r = 0.1037
Folin
1.5
1.2
0.9
0.6
0.20
0.24
0.28
abundance (%)
B
B–F
CHE
CHE–F
QUE
QUE–F

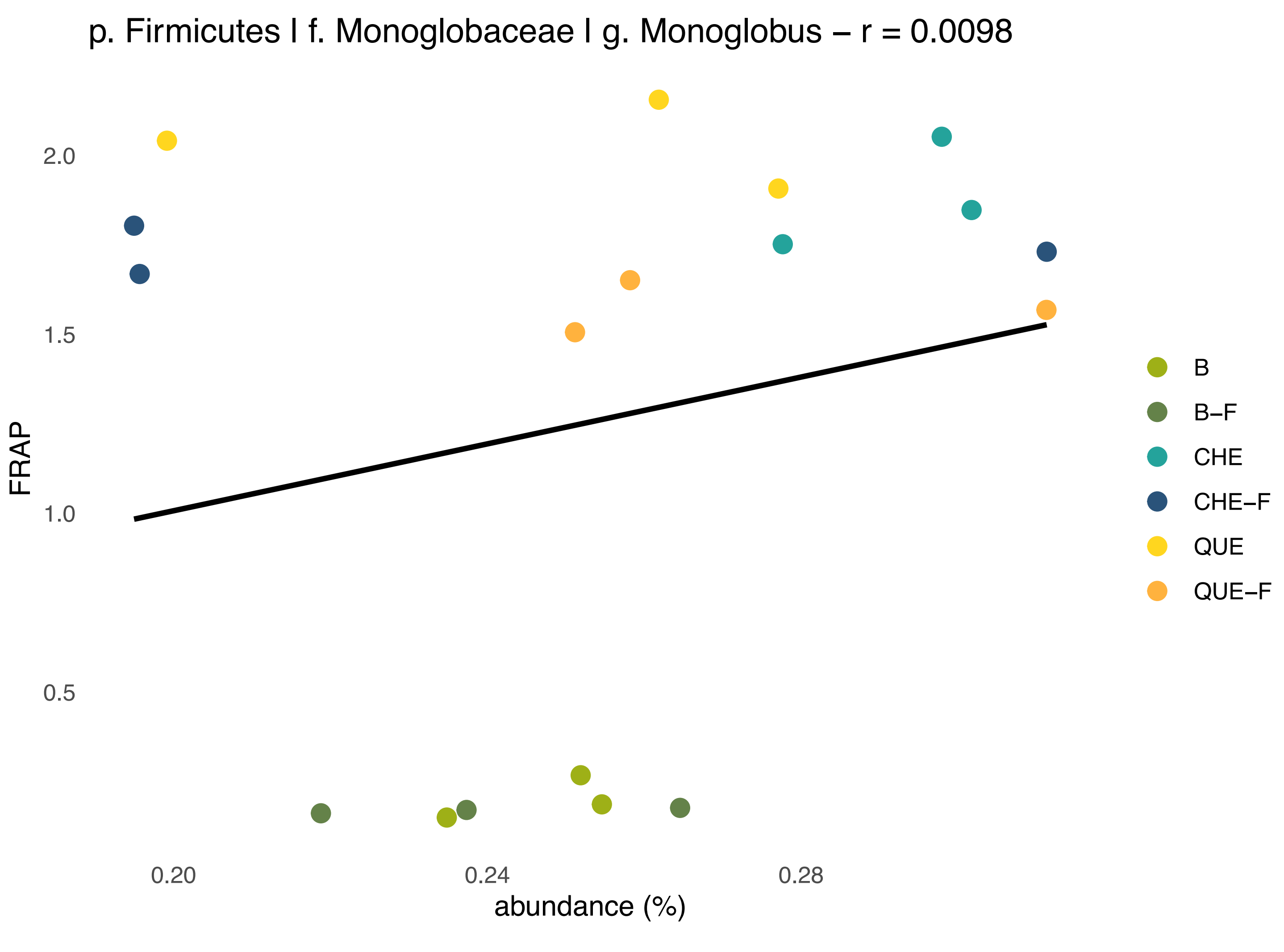
p. Firmicutes I f. Monoglobaceae I g. Monoglobus − r = 0.0098
FRAP
2.0
1.5
1.0
0.5
0.20
0.24
0.28
abundance (%)
B
B−F
CHE
CHE−F
QUE
QUE−F

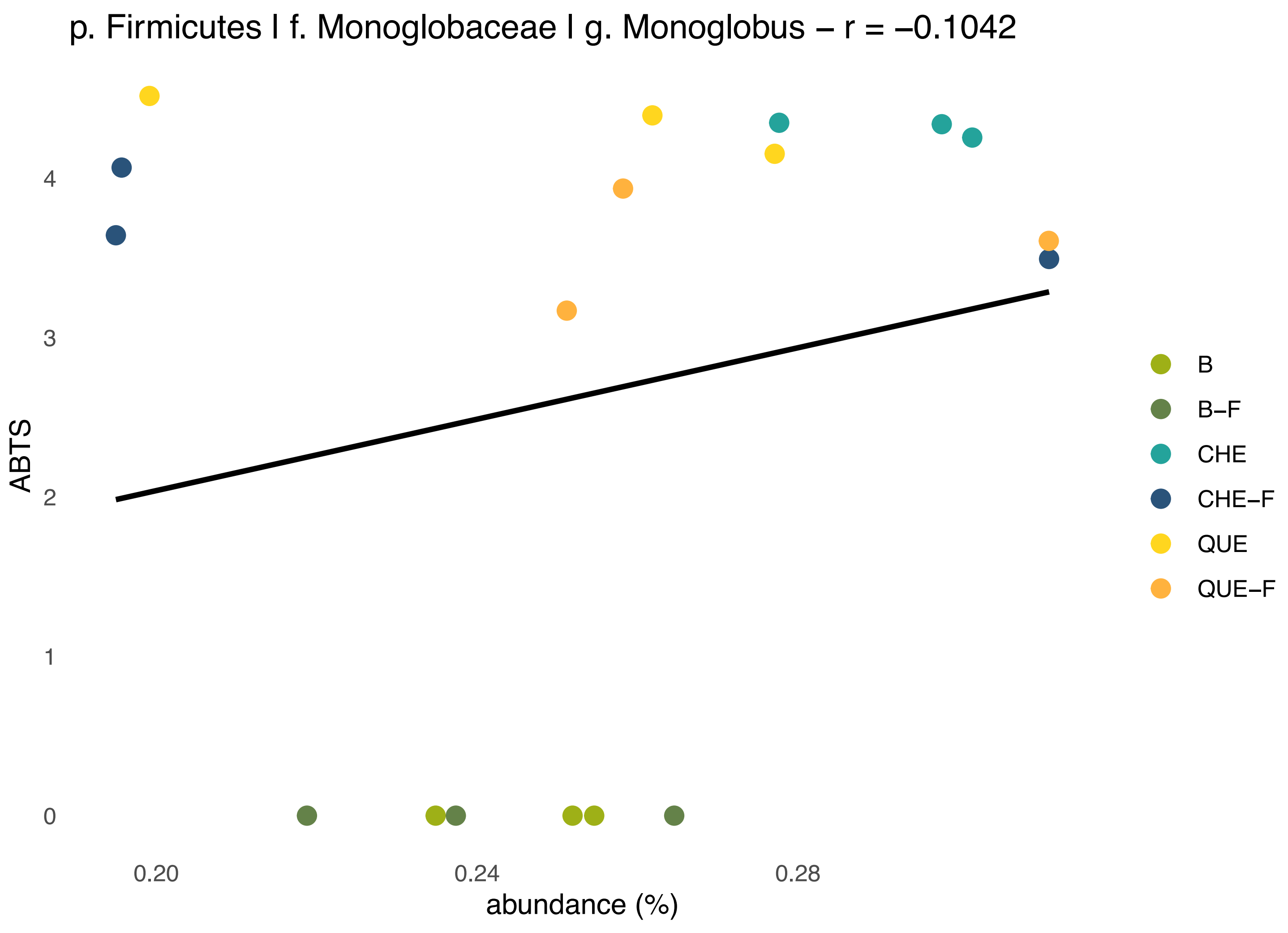

p. Firmicutes I f. Monoglobaceae I g. Monoglobus – r = –0.1042
ABTS
0
1
2
3
4
0.20
0.24
0.28
abundance (%)
B
B–F
CHE
CHE–F
QUE
QUE–F

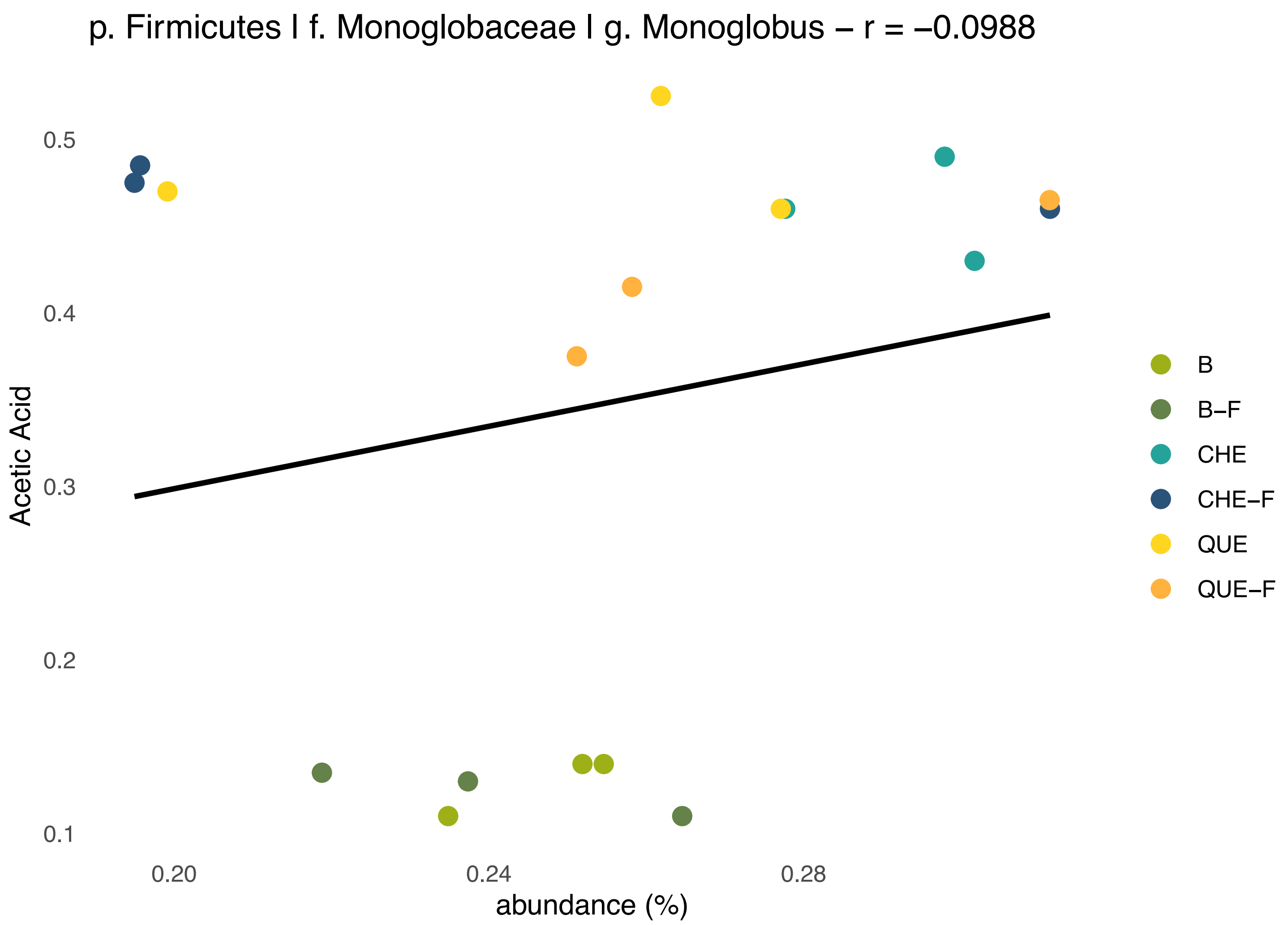
p. Firmicutes I f. Monoglobaceae I g. Monoglobus – r = –0.0988
0.5
0.4
0.3
0.2
0.1
Acetic Acid
0.20
0.24
0.28
abundance (%)
B
B–F
CHE
CHE–F
QUE
QUE–F

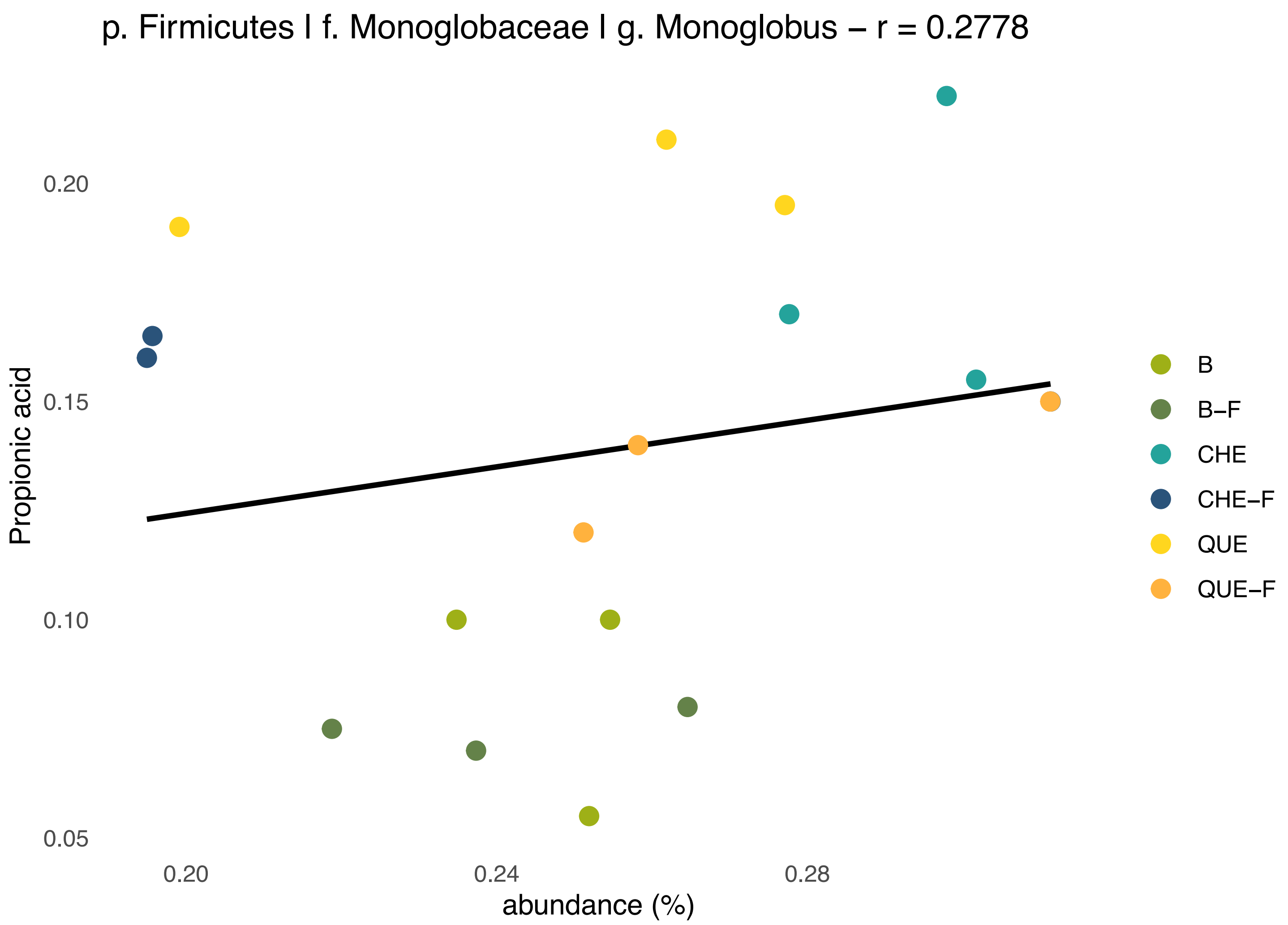
p. Firmicutes l f. Monoglobaceae l g. Monoglobus − r = 0.2778
Propionic acid
0.20
0.15
0.10
0.05
0.20
0.24
0.28
abundance (%)
B
B−F
CHE
CHE−F
QUE
QUE−F

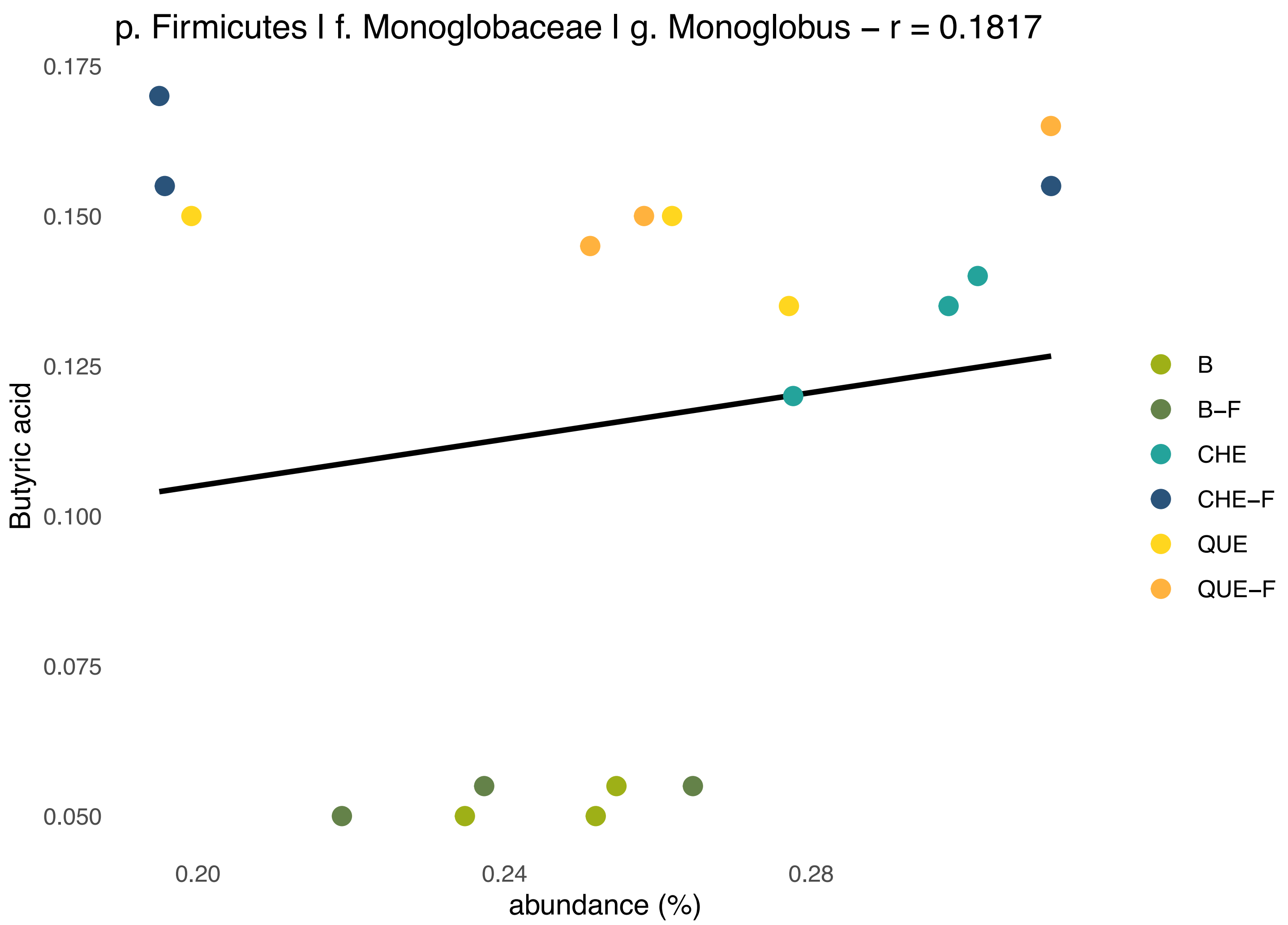
p. Firmicutes I f. Monoglobaceae I g. Monoglobus – r = 0.1817
Butyric acid
0.175
0.150
0.125
0.100
0.075
0.050
0.20
0.24
0.28
abundance (%)
B
B–F
CHE
CHE–F
QUE
QUE–F

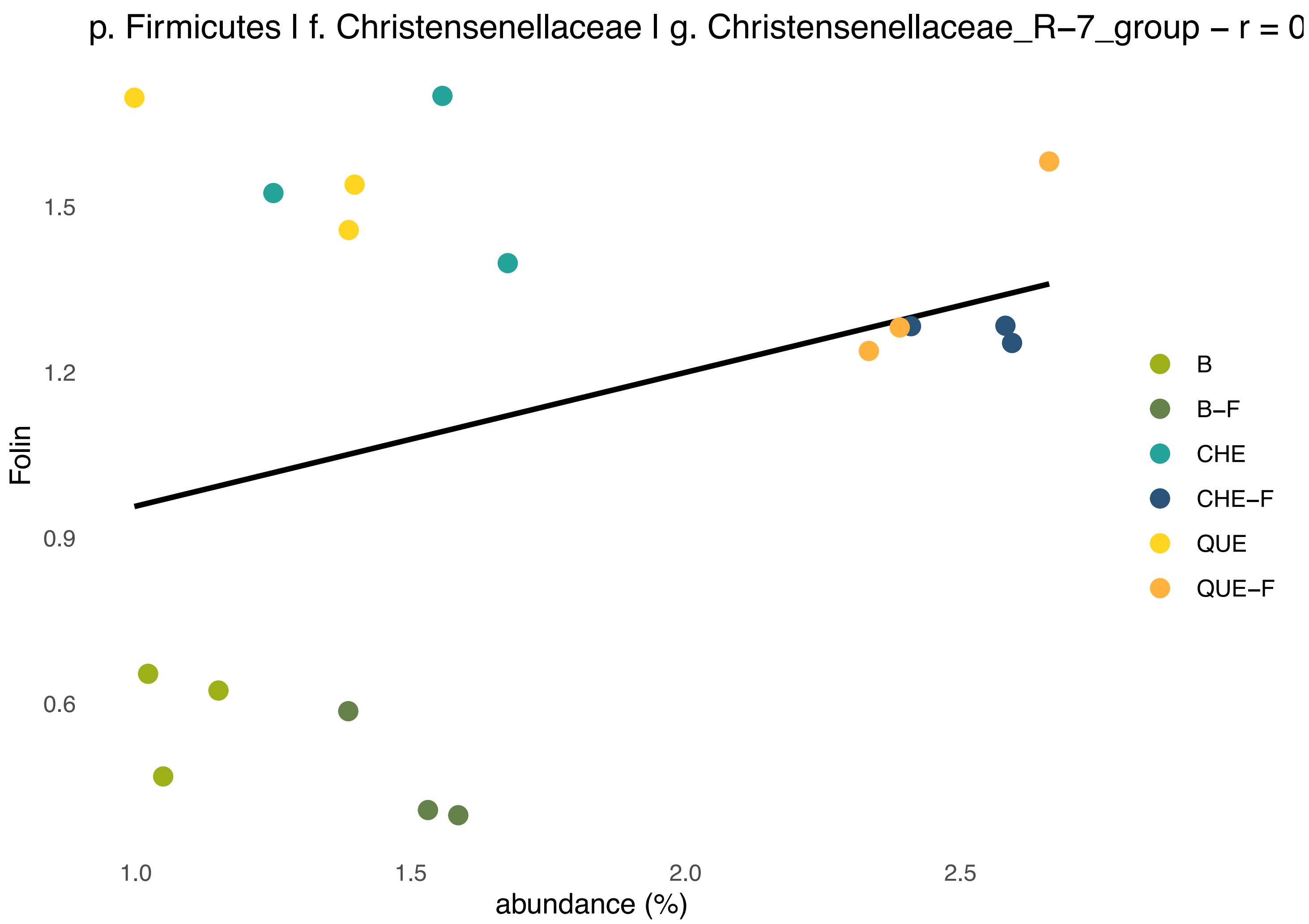

p. Firmicutes I f. Christensenellaceae I g. Christensenellaceae_R−7_group − r = 0
Folin
1.5
1.2
0.9
0.6
1.0
1.5
2.0
2.5
abundance (%)
B
B−F
CHE
CHE−F
QUE
QUE−F

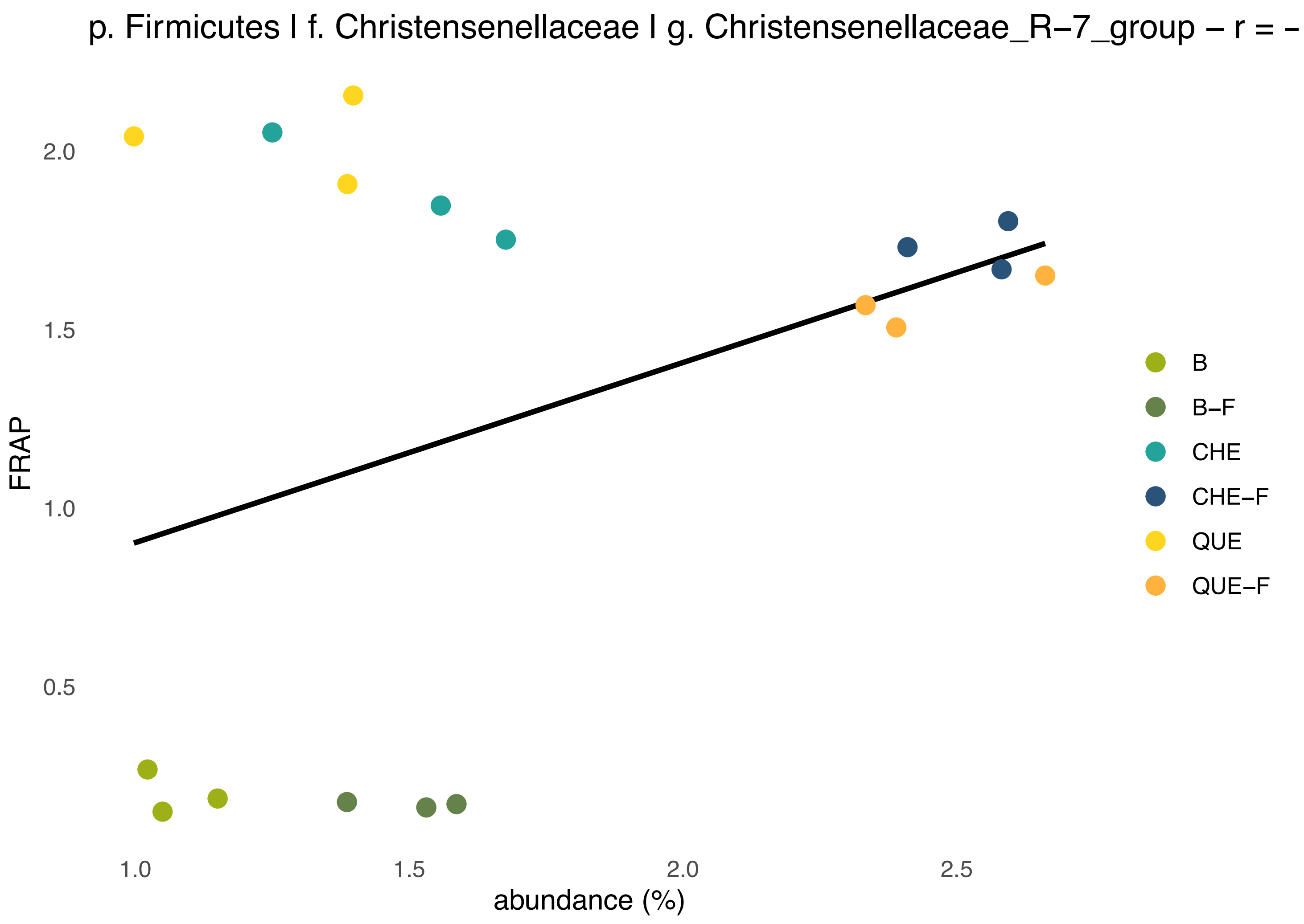
p. Firmicutes I f. Christensenellaceae I g. Christensenellaceae_R−7_group − r = −
FRAP
2.0
1.5
1.0
0.5
1.0
1.5
2.0
2.5
abundance (%)
B
B−F
CHE
CHE−F
QUE
QUE−F

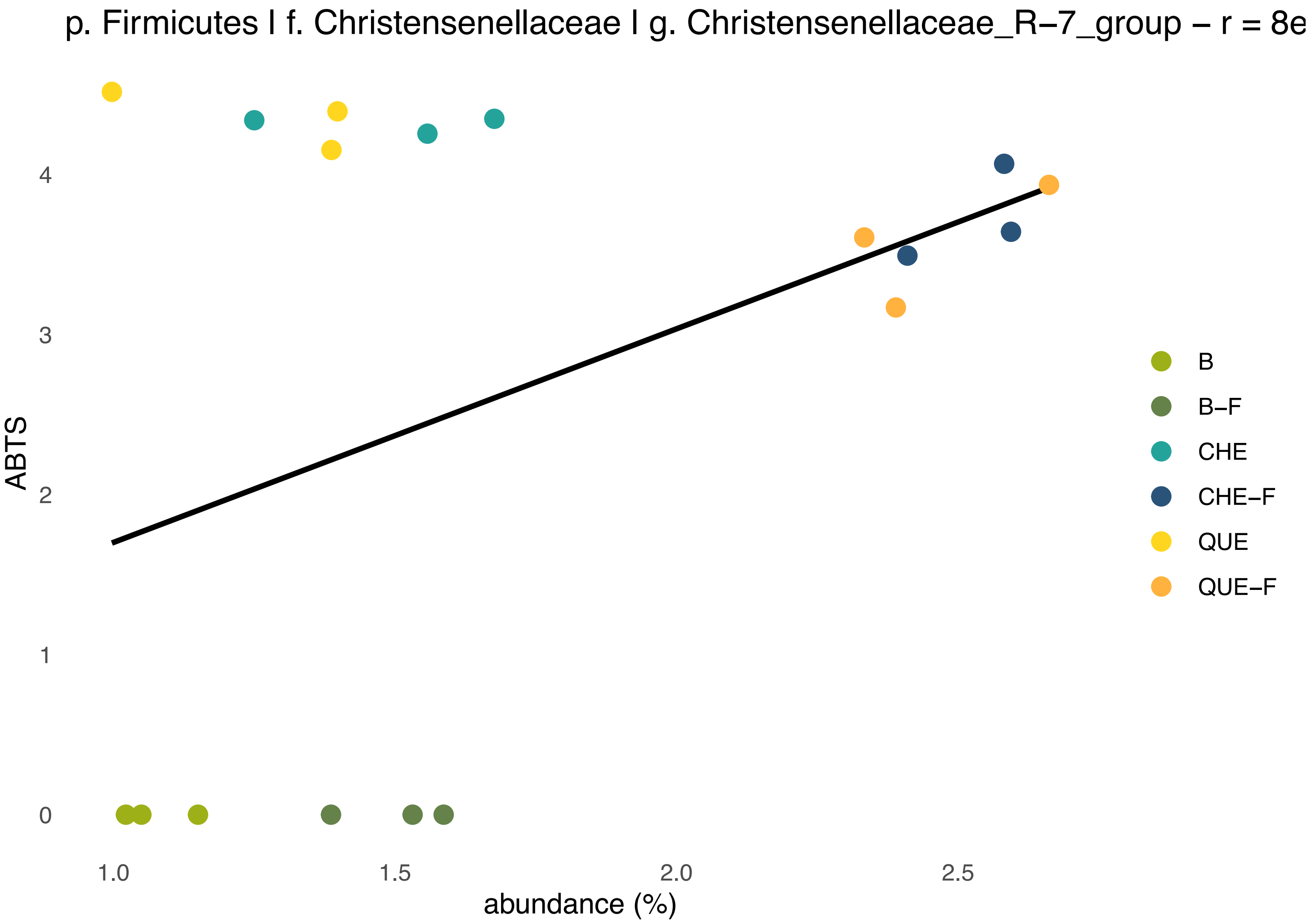

p. Firmicutes I f. Christensenellaceae I g. Christensenellaceae_R−7_group − r = 8e
ABTS
0
1
2
3
4
1.0
1.5
2.0
2.5
abundance (%)
B
B−F
CHE
CHE−F
QUE
QUE−F

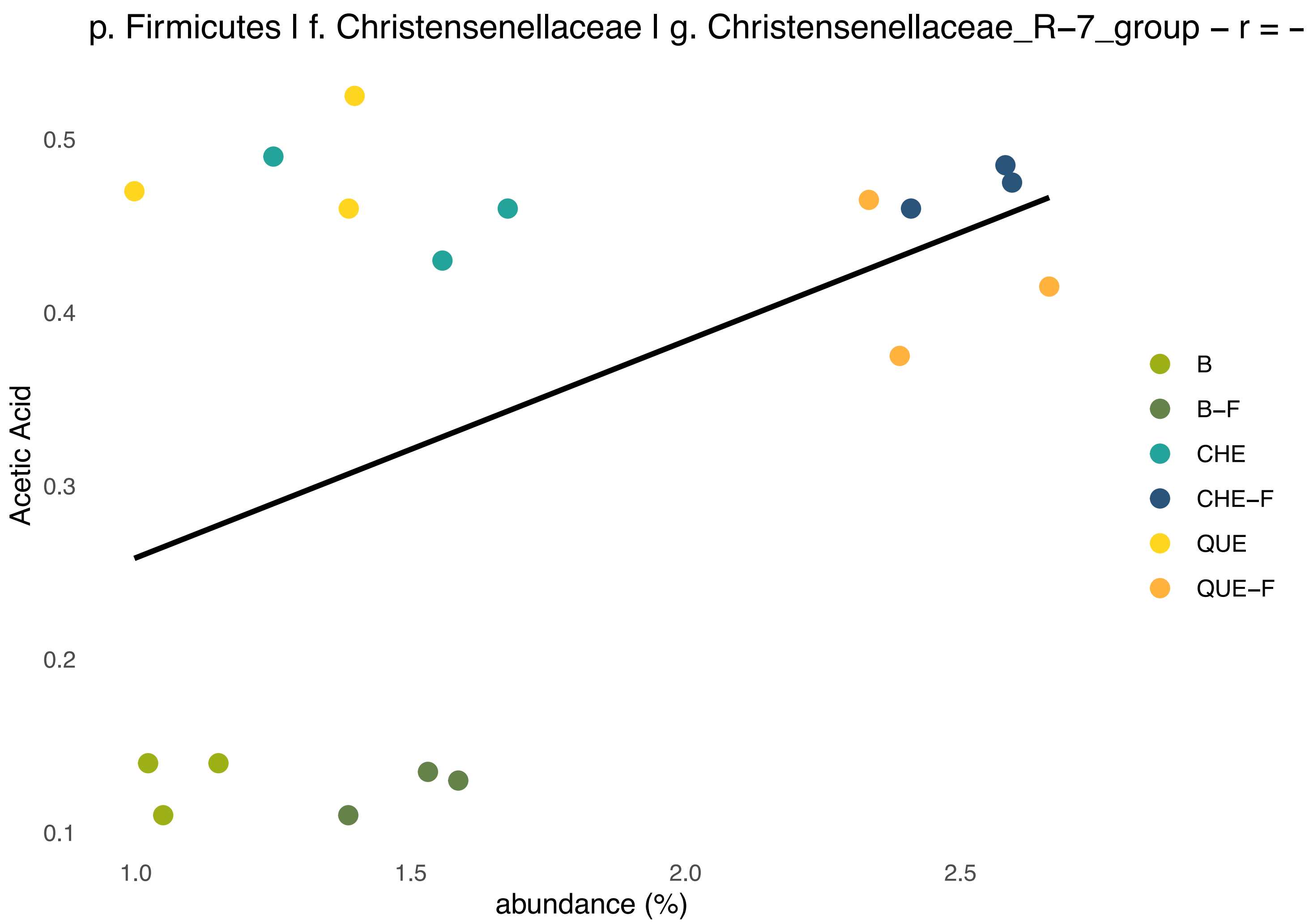

p. Firmicutes I f. Christensenellaceae I g. Christensenellaceae_R−7_group − r = −
0.5
0.4
0.3
0.2
0.1
Acetic Acid
1.0
1.5
2.0
2.5
abundance (%)
B
B−F
CHE
CHE−F
QUE
QUE−F

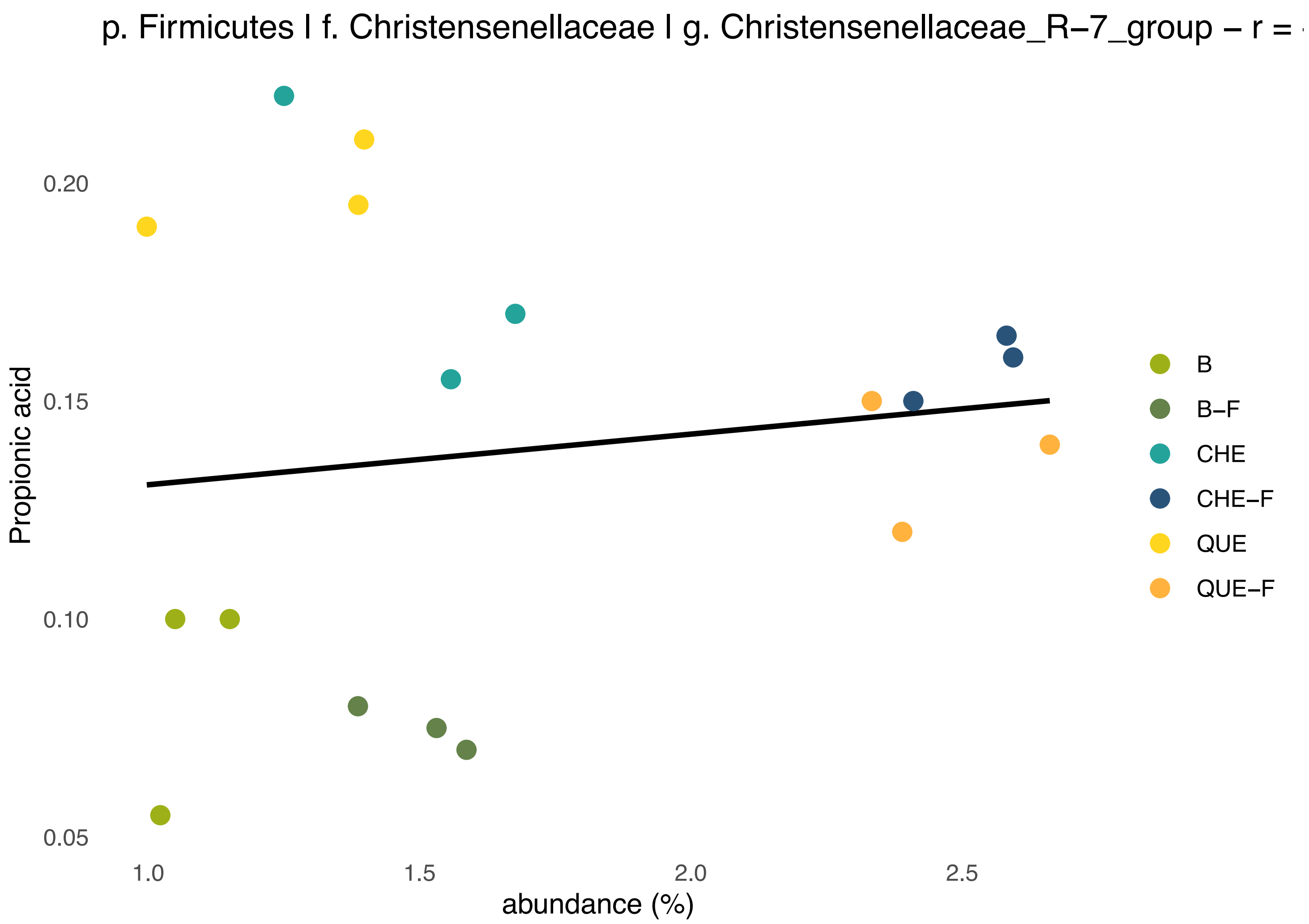
p. Firmicutes I f. Christensenellaceae I g. Christensenellaceae_R−7_group − r =
Propionic acid
0.20
0.15
0.10
0.05
1.0
1.5
2.0
2.5
abundance (%)
B
B−F
CHE
CHE−F
QUE
QUE−F

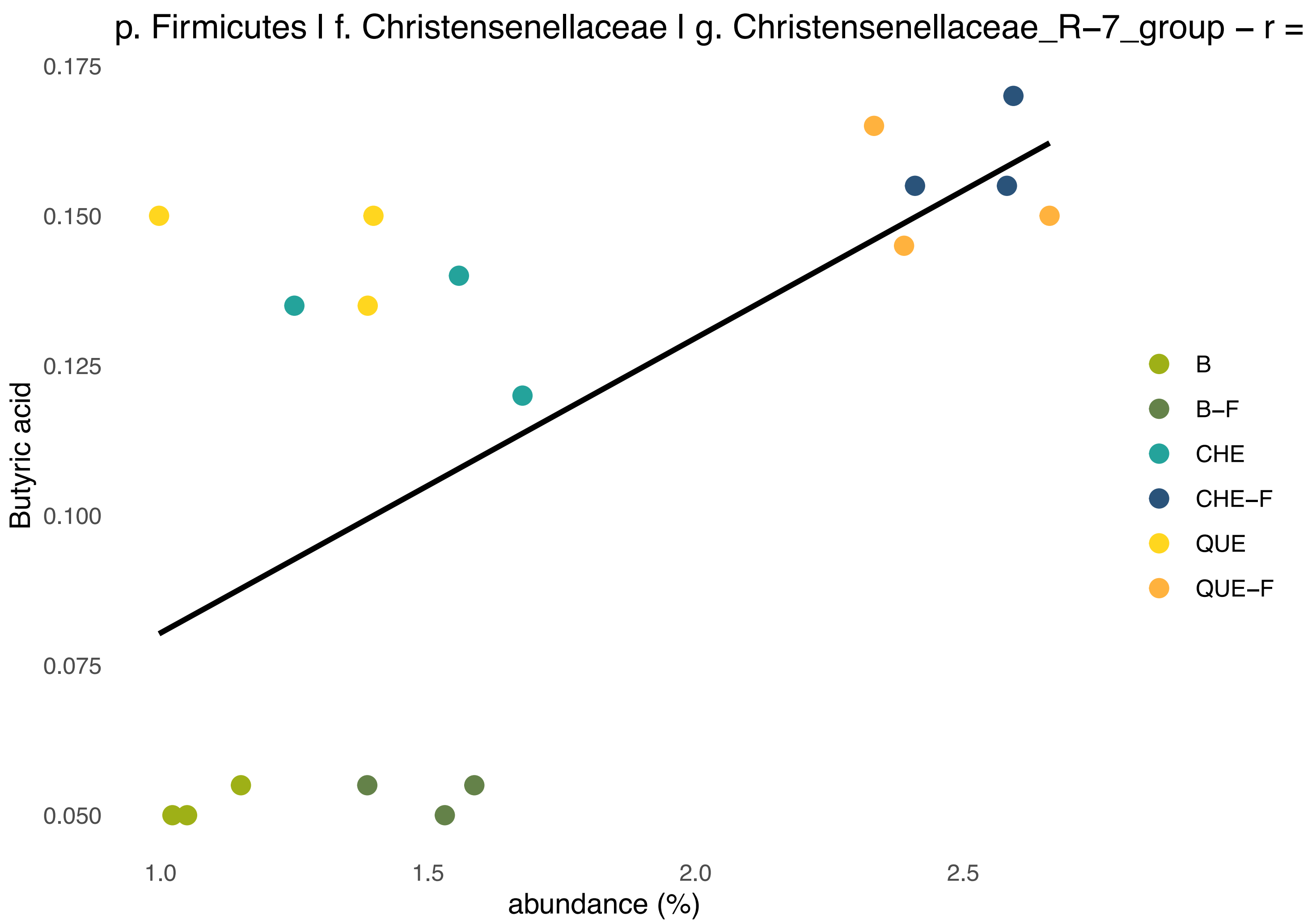

p. Firmicutes I f. Christensenellaceae I g. Christensenellaceae_R−7_group − r =
Butyric acid
0.175
0.150
0.125
0.100
0.075
0.050
1.0
1.5
2.0
2.5
abundance (%)
B
B−F
CHE
CHE−F
QUE
QUE−F

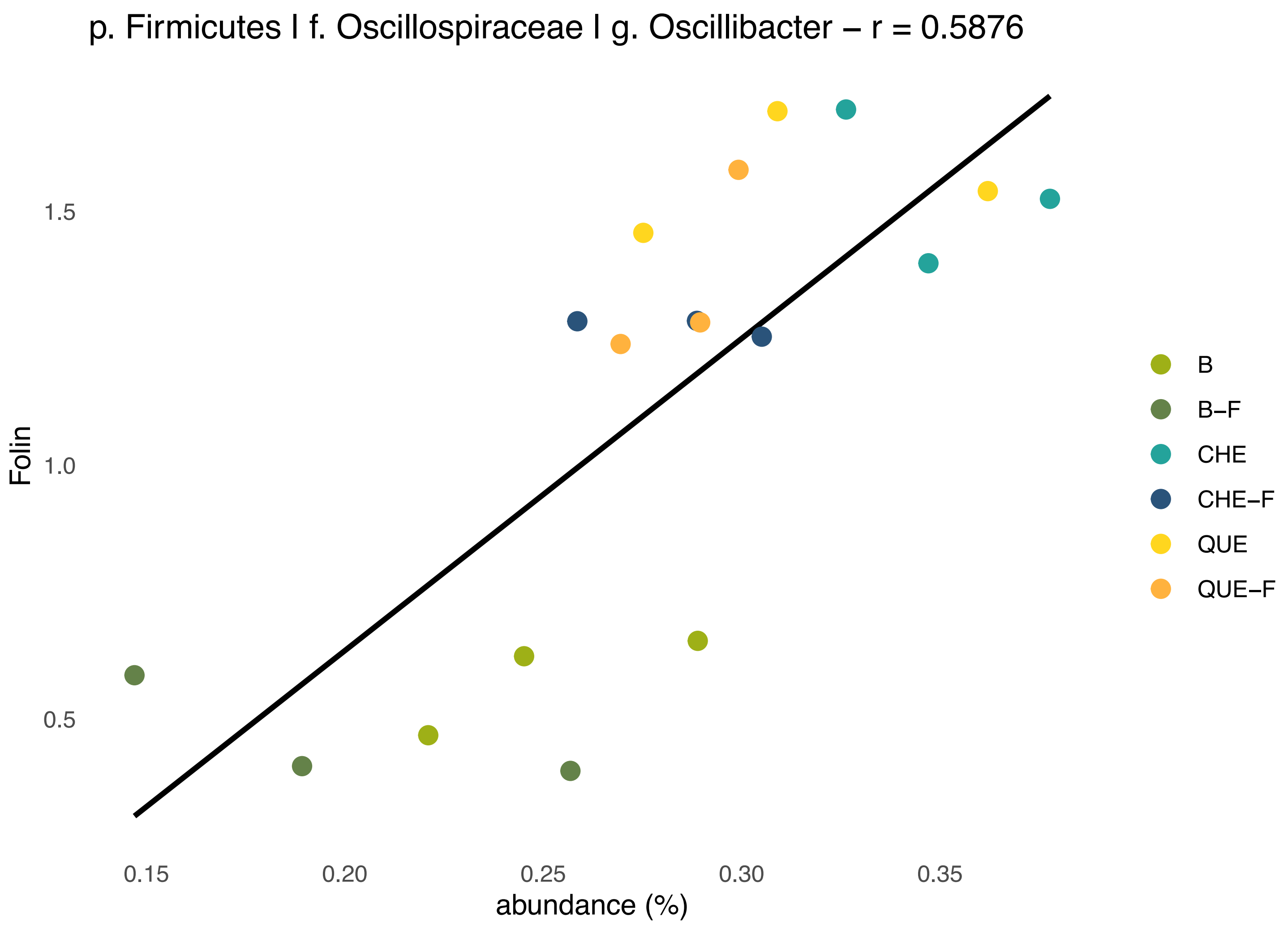

p. Firmicutes I f. Oscillospiraceae I g. Oscillibacter – r = 0.5876
Folin
1.5
1.0
0.5
0.15
0.20
0.25
0.30
0.35
abundance (%)
B
B–F
CHE
CHE–F
QUE
QUE–F

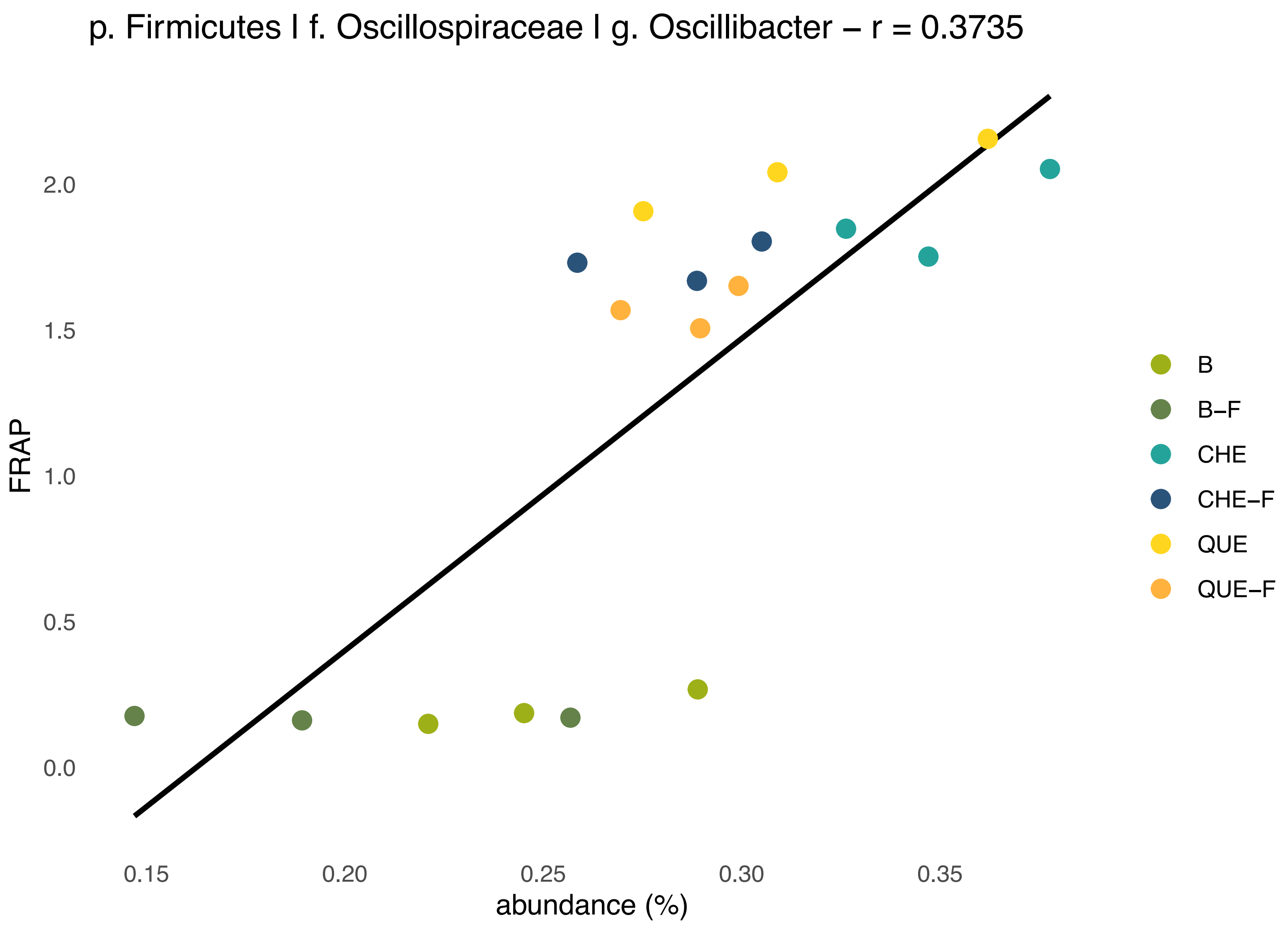

p. Firmicutes I f. Oscillospiraceae I g. Oscillibacter – r = 0.3735
FRAP
2.0
1.5
1.0
0.5
0.0
0.15
0.20
0.25
0.30
0.35
abundance (%)
B
B–F
CHE
CHE–F
QUE
QUE–F

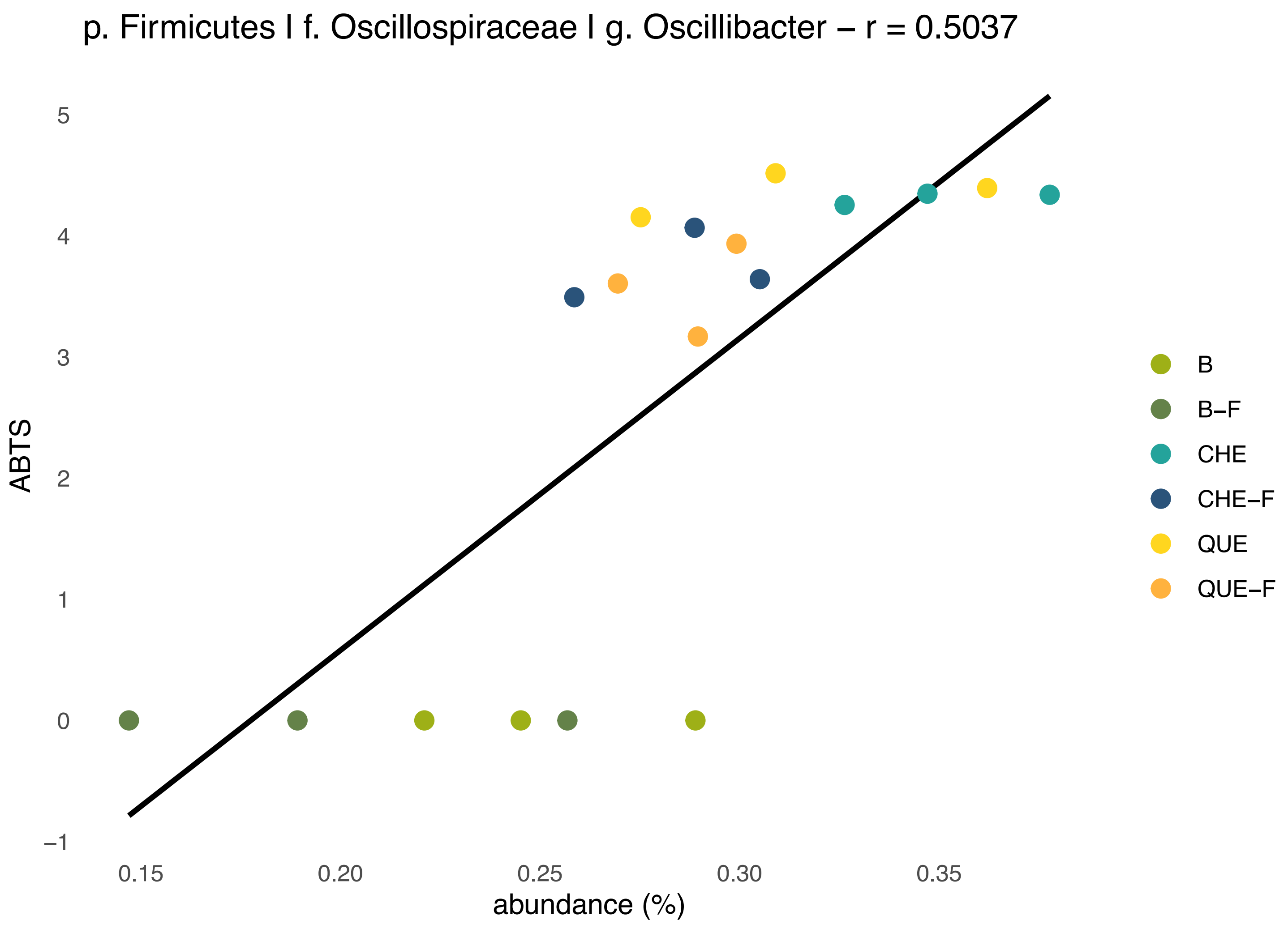
p. Firmicutes I f. Oscillospiraceae I g. Oscillibacter – r = 0.5037
ABTS
5
4
3
2
1
0
−1
0.15
0.20
0.25
0.30
0.35
abundance (%)
B
B–F
CHE
CHE–F
QUE
QUE–F

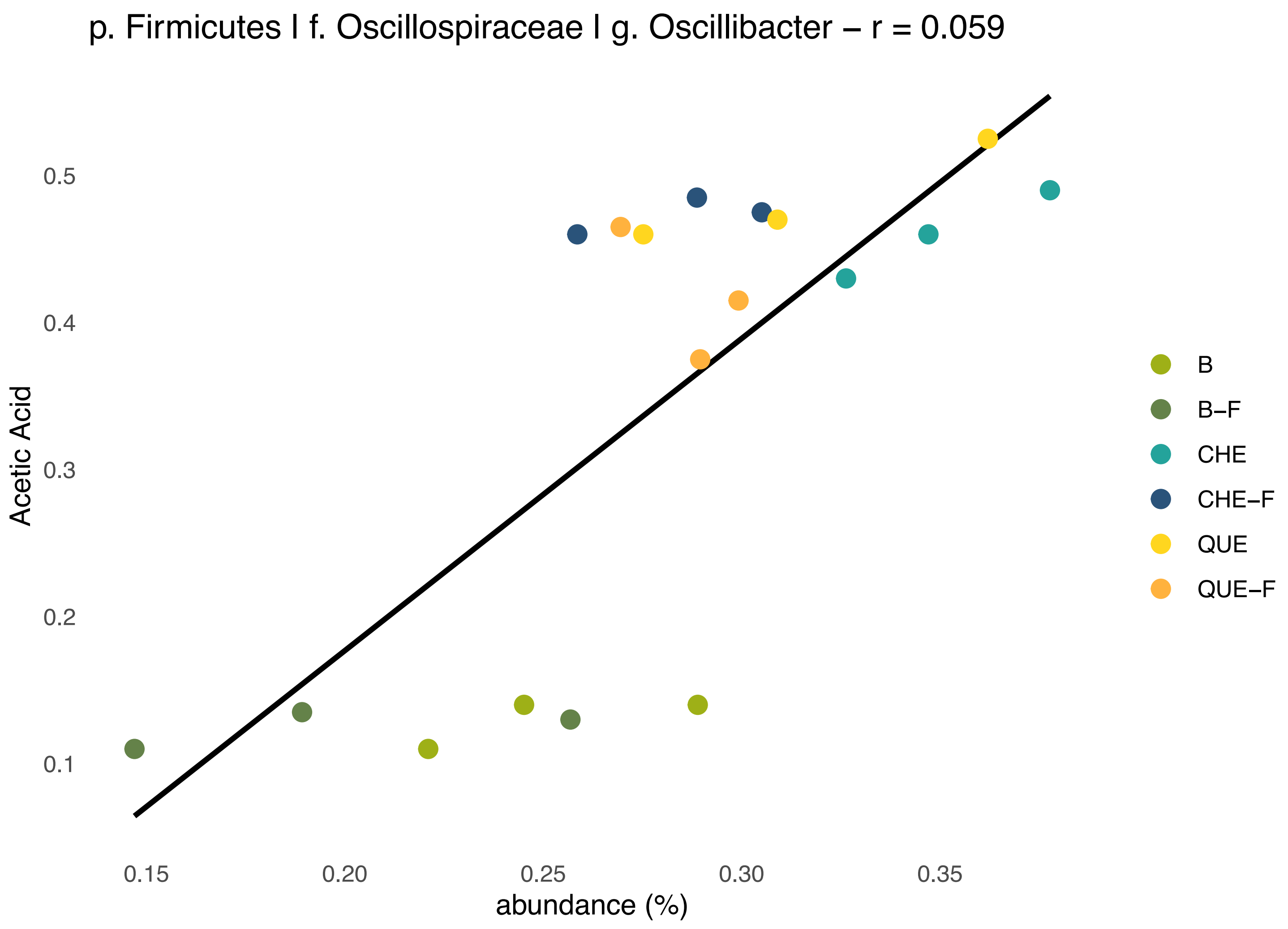
p. Firmicutes l f. Oscillospiraceae l g. Oscillibacter – r = 0.059
Acetic Acid
0.1
0.2
0.3
0.4
0.5
0.15
0.20
0.25
0.30
0.35
abundance (%)
B
B−F
CHE
CHE−F
QUE
QUE−F

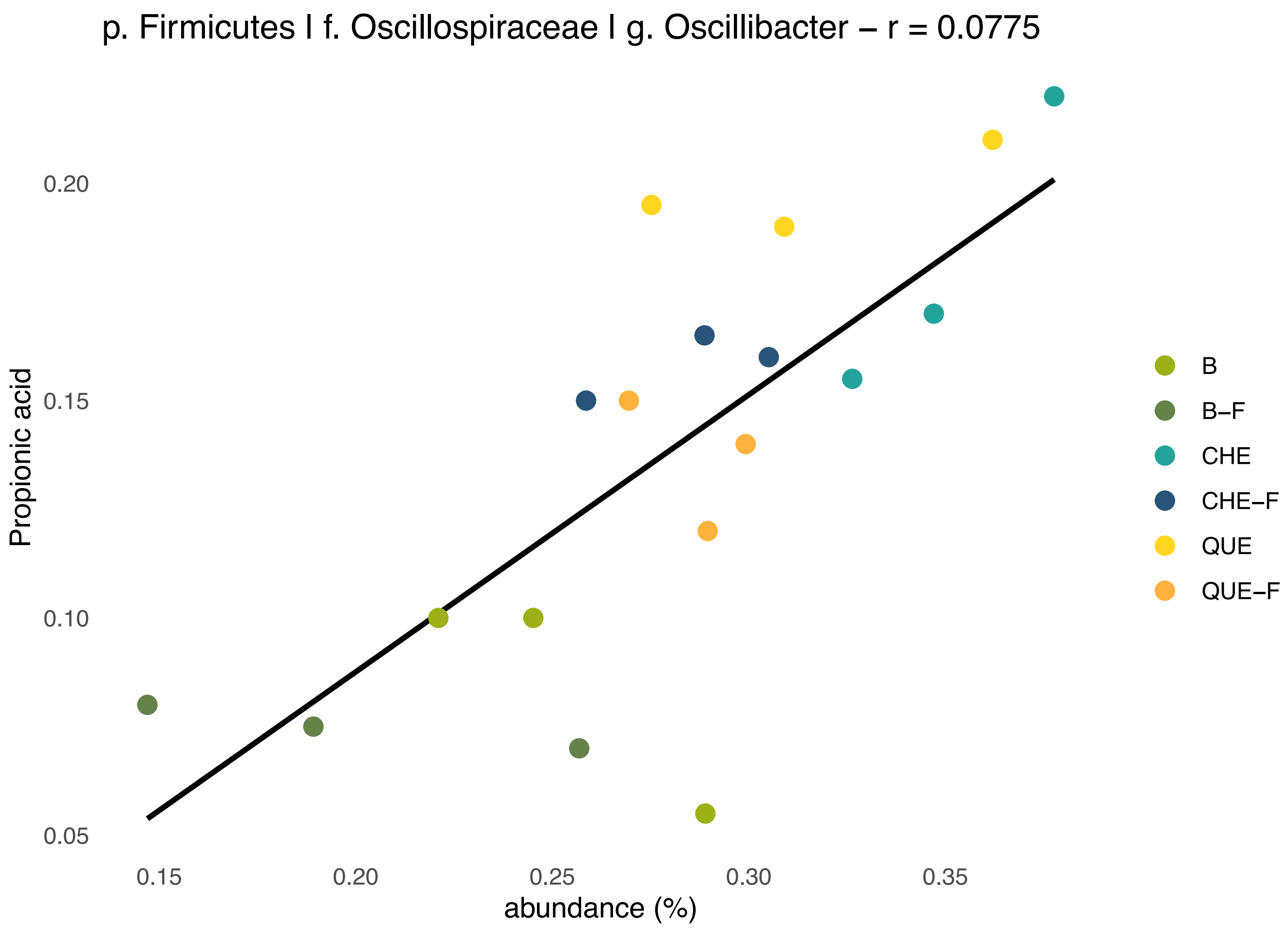

p. Firmicutes I f. Oscillospiraceae I g. Oscillibacter – r = 0.0775
Propionic acid
0.20
0.15
0.10
0.05
0.15
0.20
0.25
0.30
0.35
abundance (%)
B
B–F
CHE
CHE–F
QUE
QUE–F

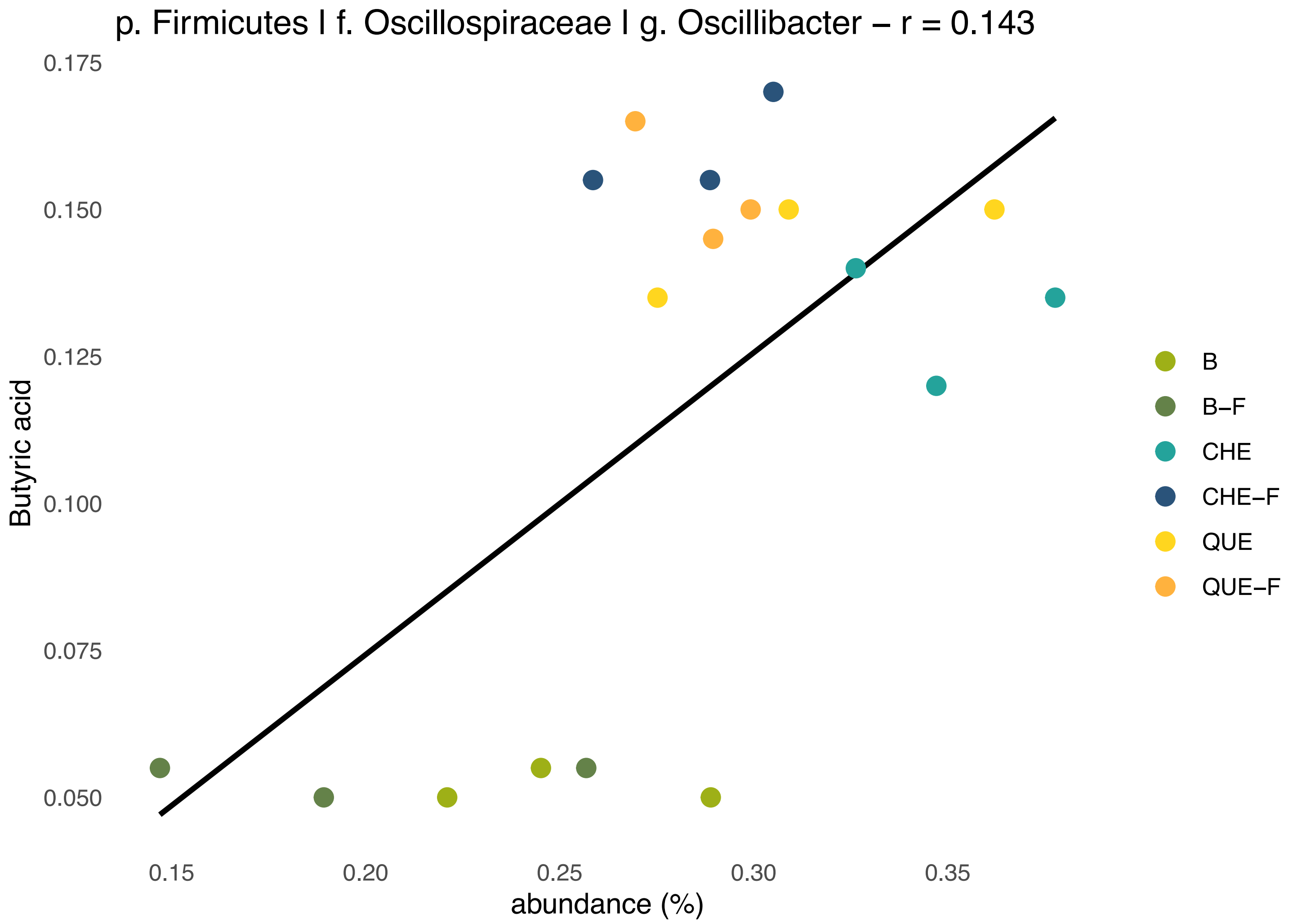
p. Firmicutes I f. Oscillospiraceae I g. Oscillibacter – r = 0.143
0.175
0.150
0.125
0.100
0.075
0.050
Butyric acid
0.15
0.20
0.25
0.30
0.35
abundance (%)
B
B–F
CHE
CHE–F
QUE
QUE–F

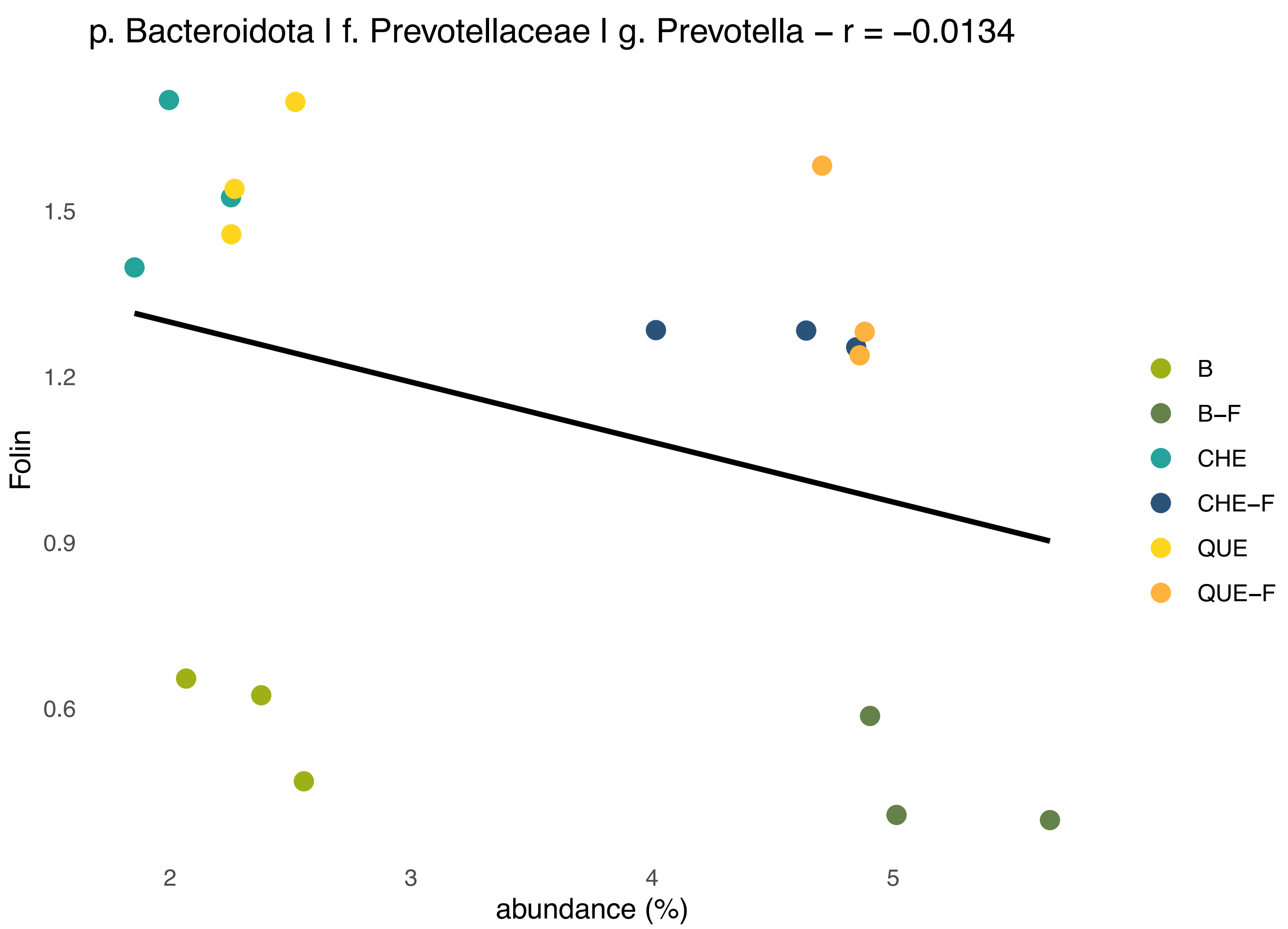
p. Bacteroidota | f. Prevotellaceae | g. Prevotella – r = –0.0134
Folin
1.5
1.2
0.9
0.6
2
3
4
5
abundance (%)
B
B–F
CHE
CHE–F
QUE
QUE–F

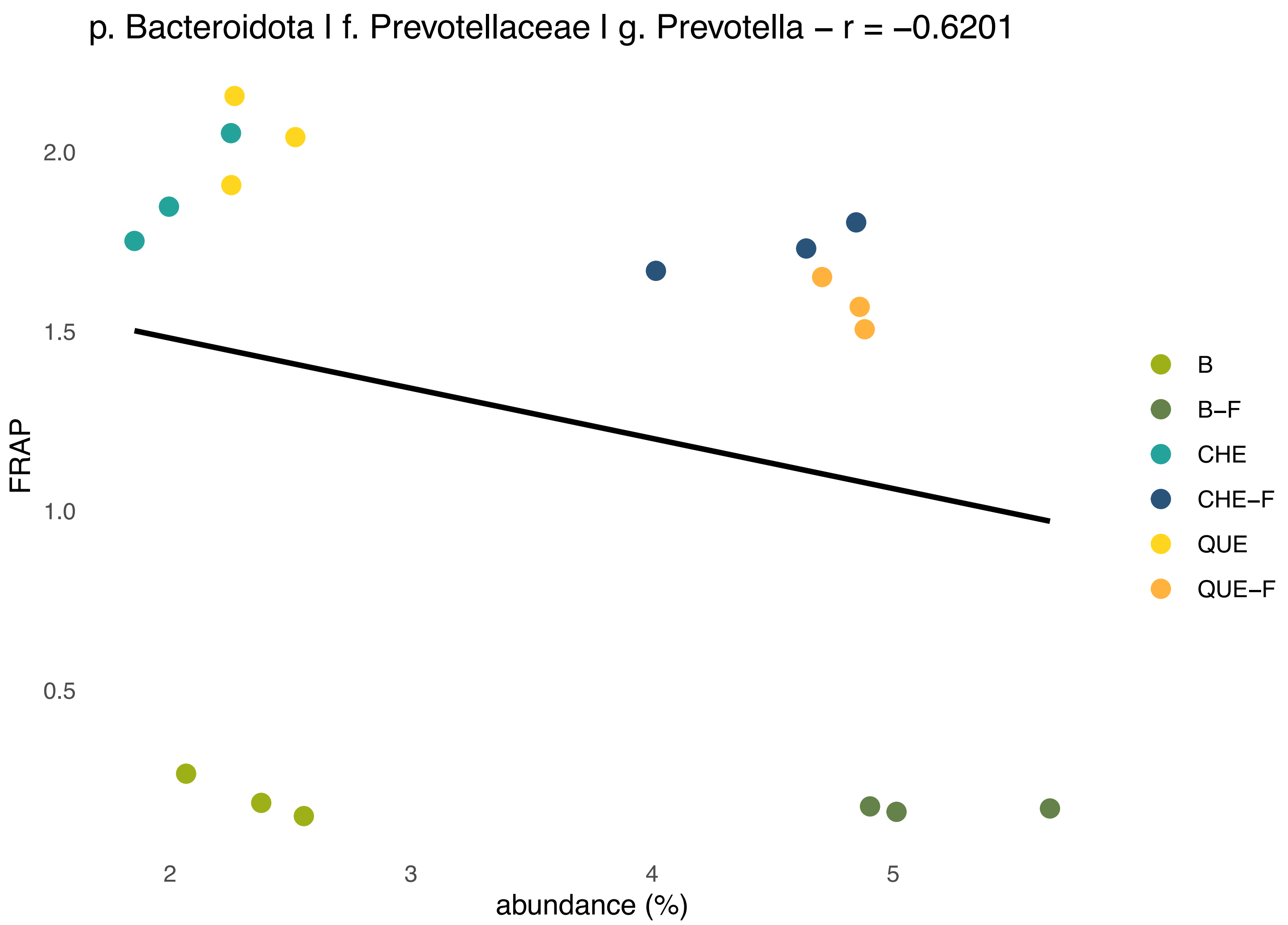
p. Bacteroidota | f. Prevotellaceae | g. Prevotella – r = –0.6201
FRAP
2.0
1.5
1.0
0.5
2
3
4
5
abundance (%)
B
B–F
CHE
CHE–F
QUE
QUE–F

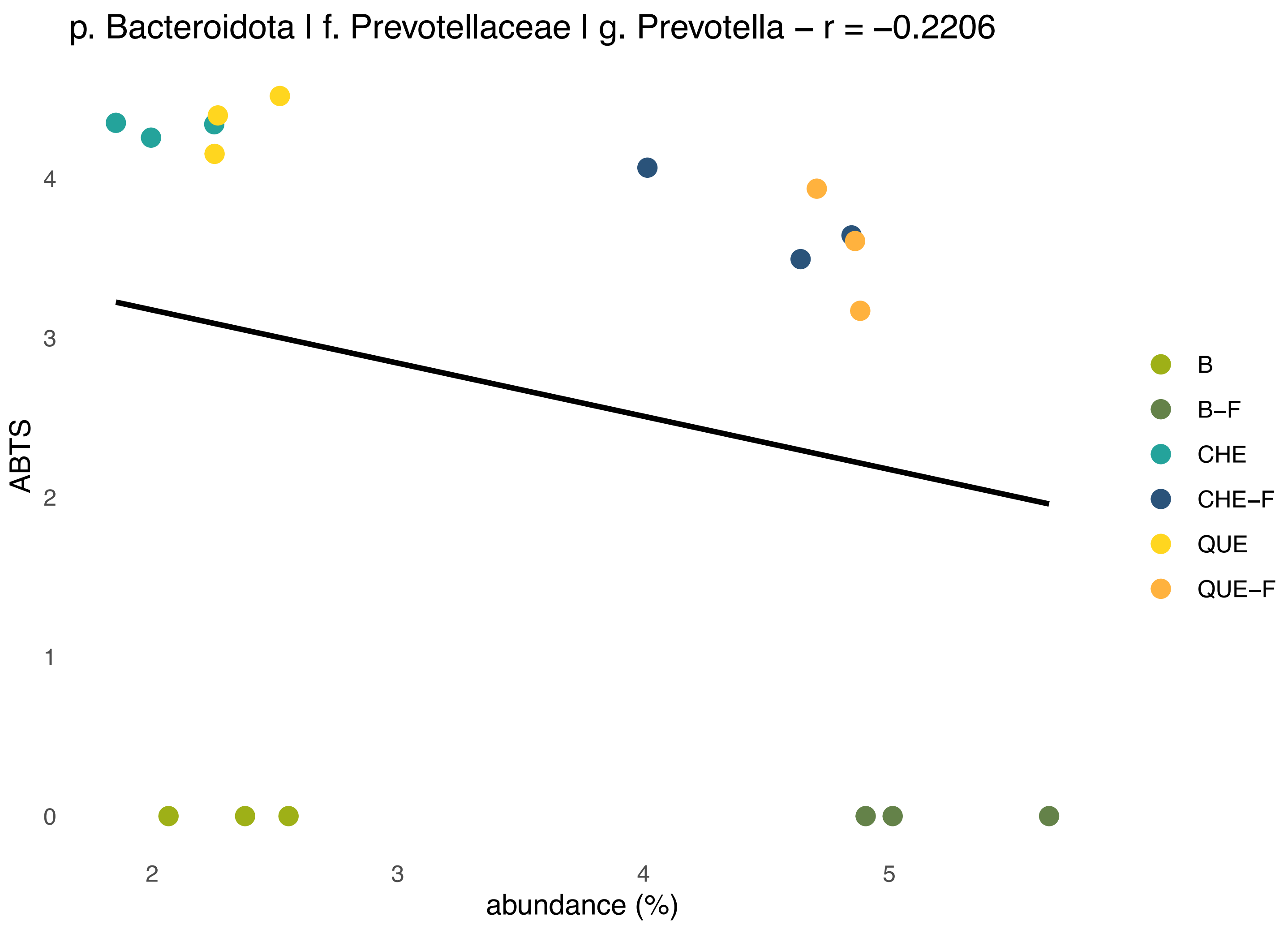

p. Bacteroidota I f. Prevotellaceae I g. Prevotella – r = –0.2206
ABTS
0
1
2
3
4
2
3
4
5
abundance (%)
B
B–F
CHE
CHE–F
QUE
QUE–F

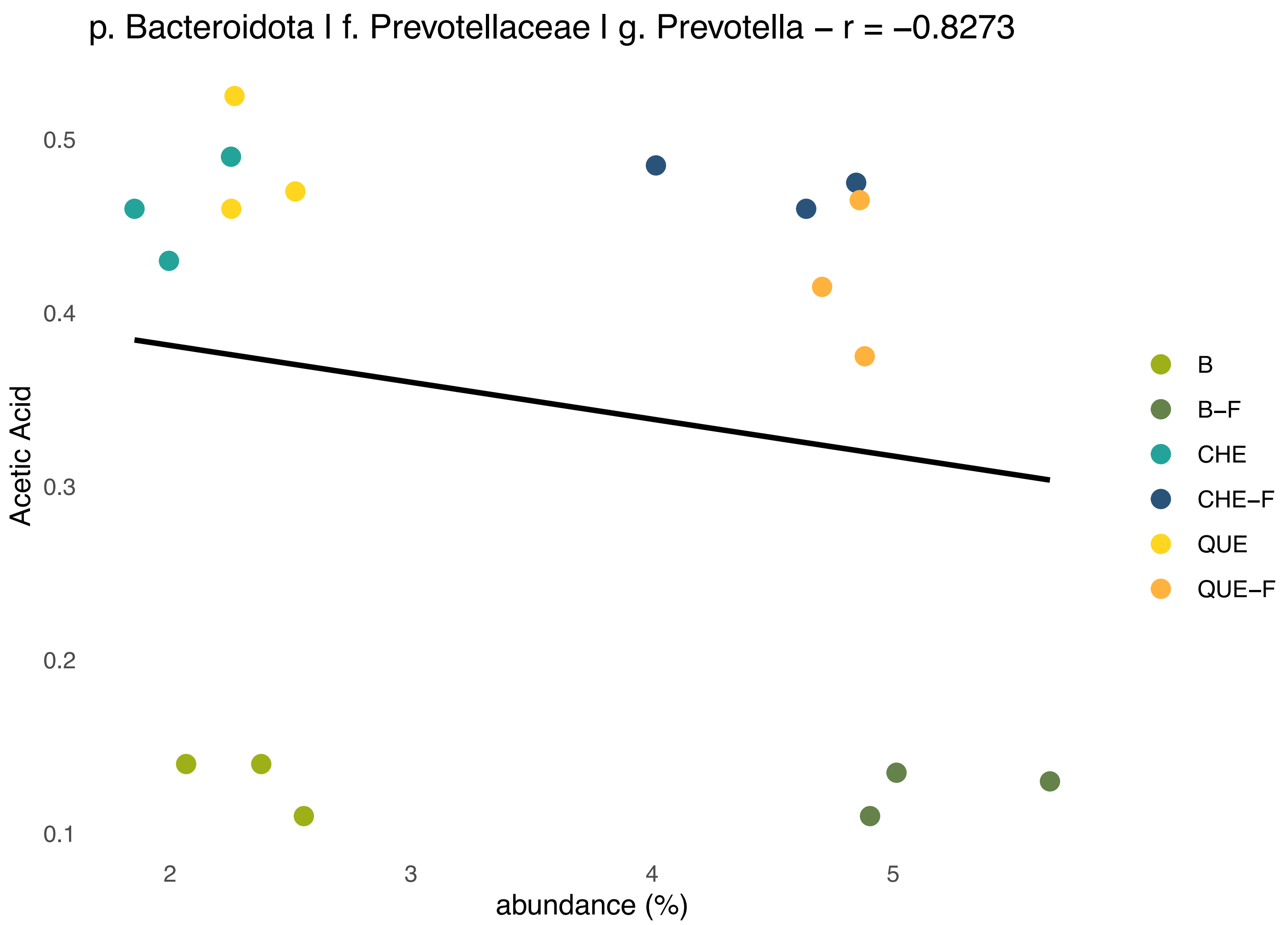
p. Bacteroidota I f. Prevotellaceae I g. Prevotella – r = –0.8273
Acetic Acid
0.5
0.4
0.3
0.2
0.1
2
3
4
5
abundance (%)
B
B–F
CHE
CHE–F
QUE
QUE–F

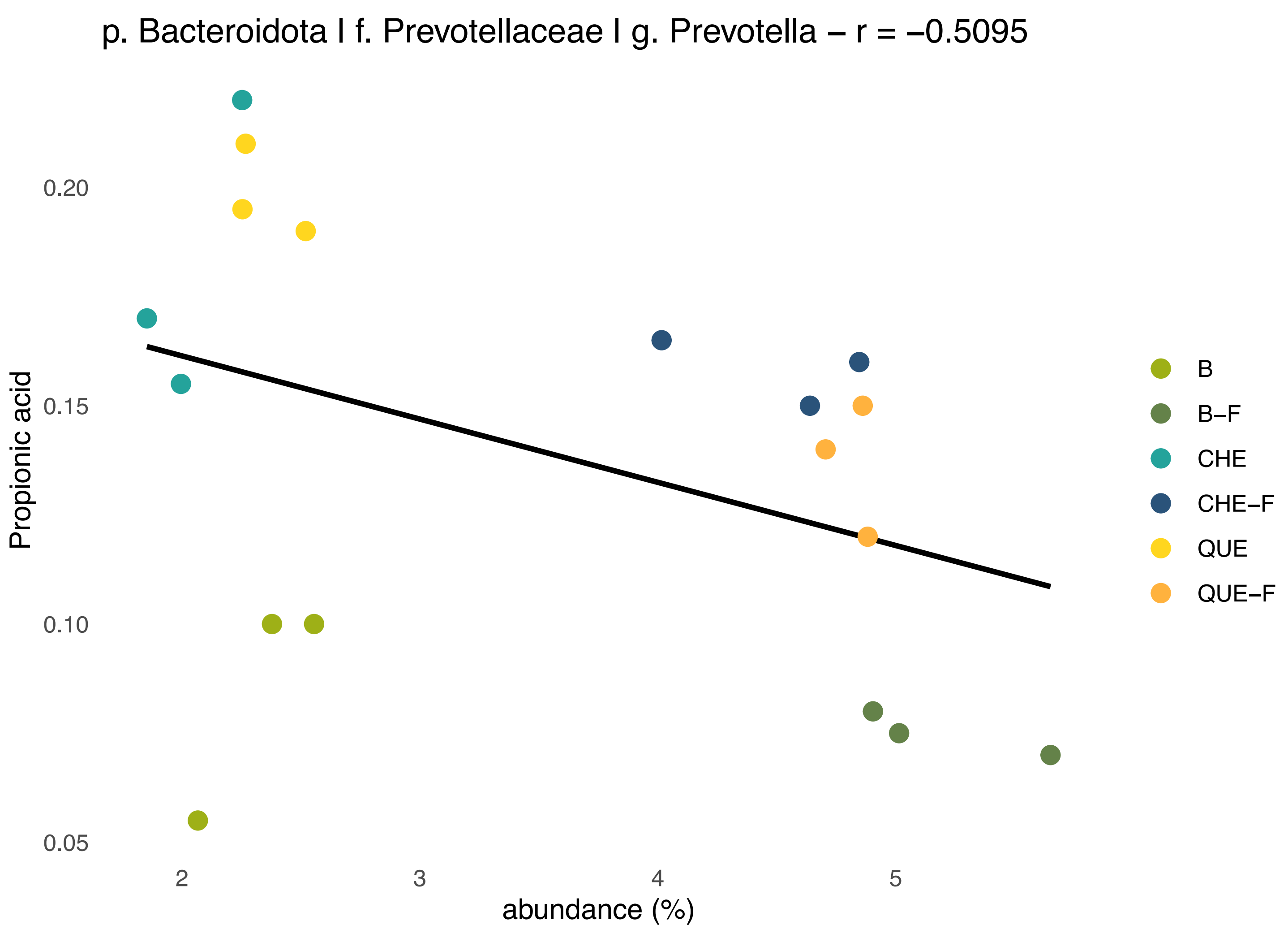
p. Bacteroidota | f. Prevotellaceae | g. Prevotella – r = −0.5095
Propionic acid
0.20
0.15
0.10
0.05
2
3
4
5
abundance (%)
B
B−F
CHE
CHE−F
QUE
QUE−F

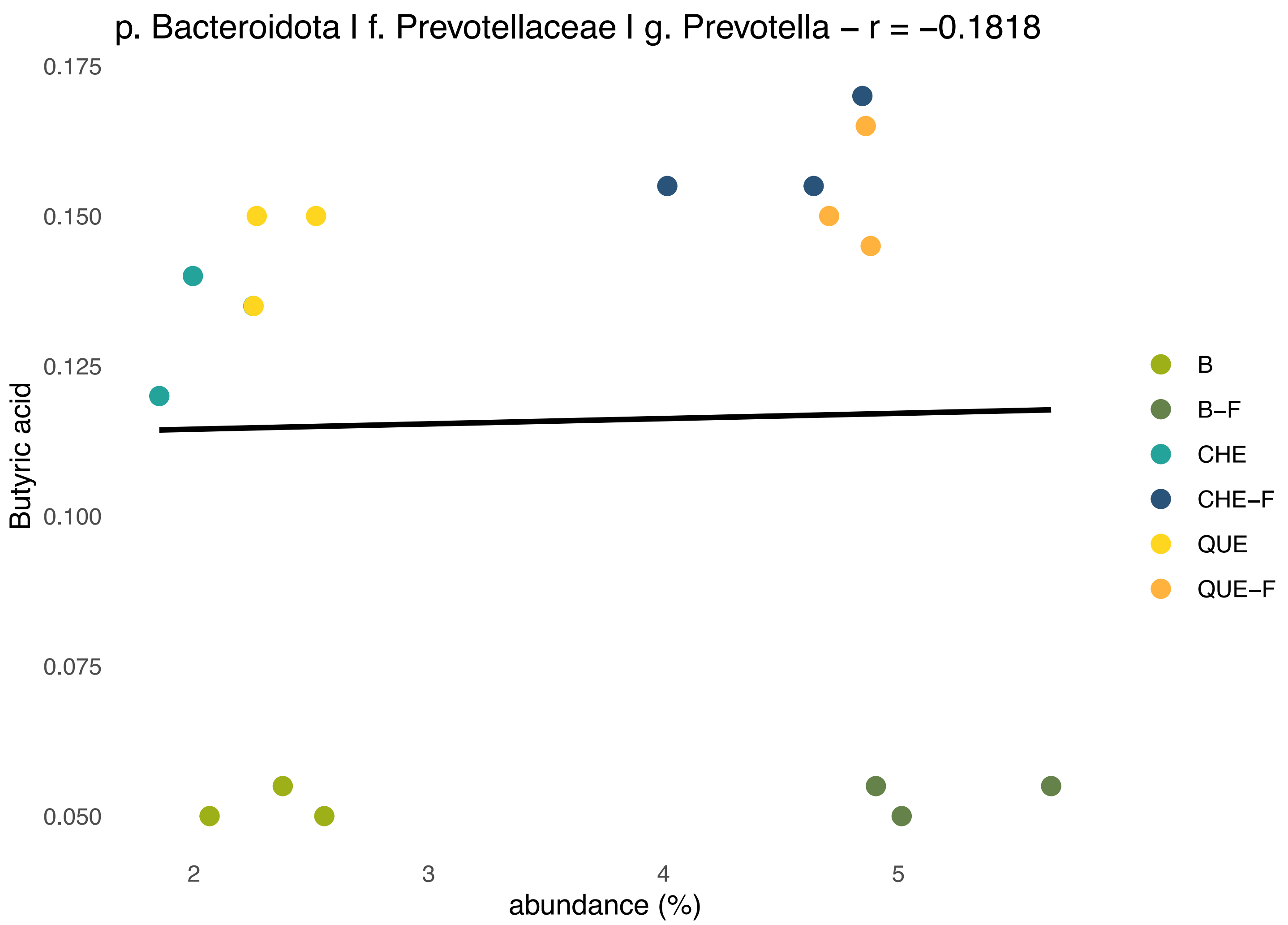

p. Bacteroidota | f. Prevotellaceae | g. Prevotella – r = –0.1818
Butyric acid
0.175
0.150
0.125
0.100
0.075
0.050
2
3
4
5
abundance (%)
B
B–F
CHE
CHE–F
QUE
QUE–F

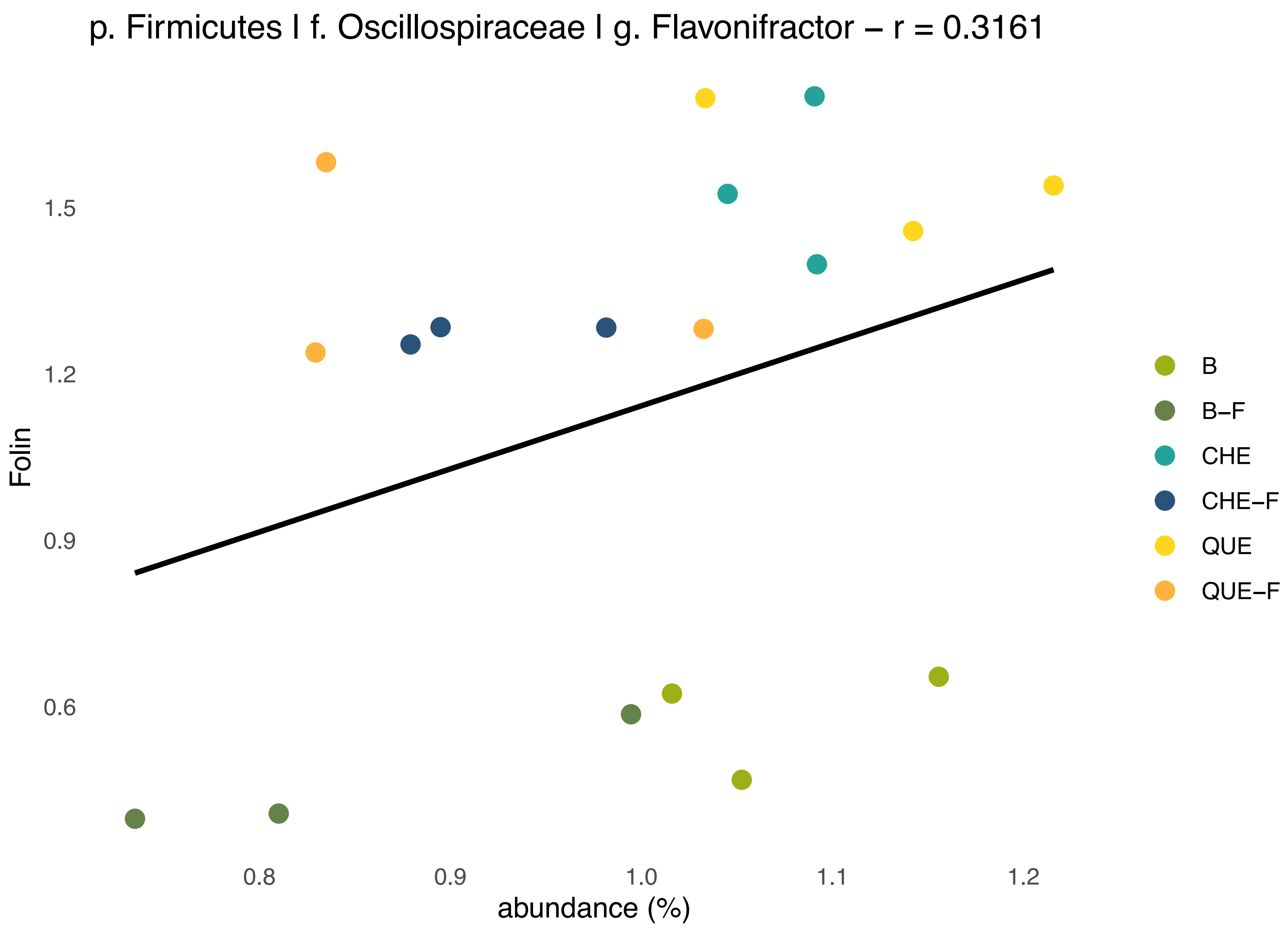
p. Firmicutes I f. Oscillospiraceae I g. Flavonifractor – r = 0.3161
Folin
1.5
1.2
0.9
0.6
0.8
0.9
1.0
1.1
1.2
abundance (%)
B
B–F
CHE
CHE–F
QUE
QUE–F

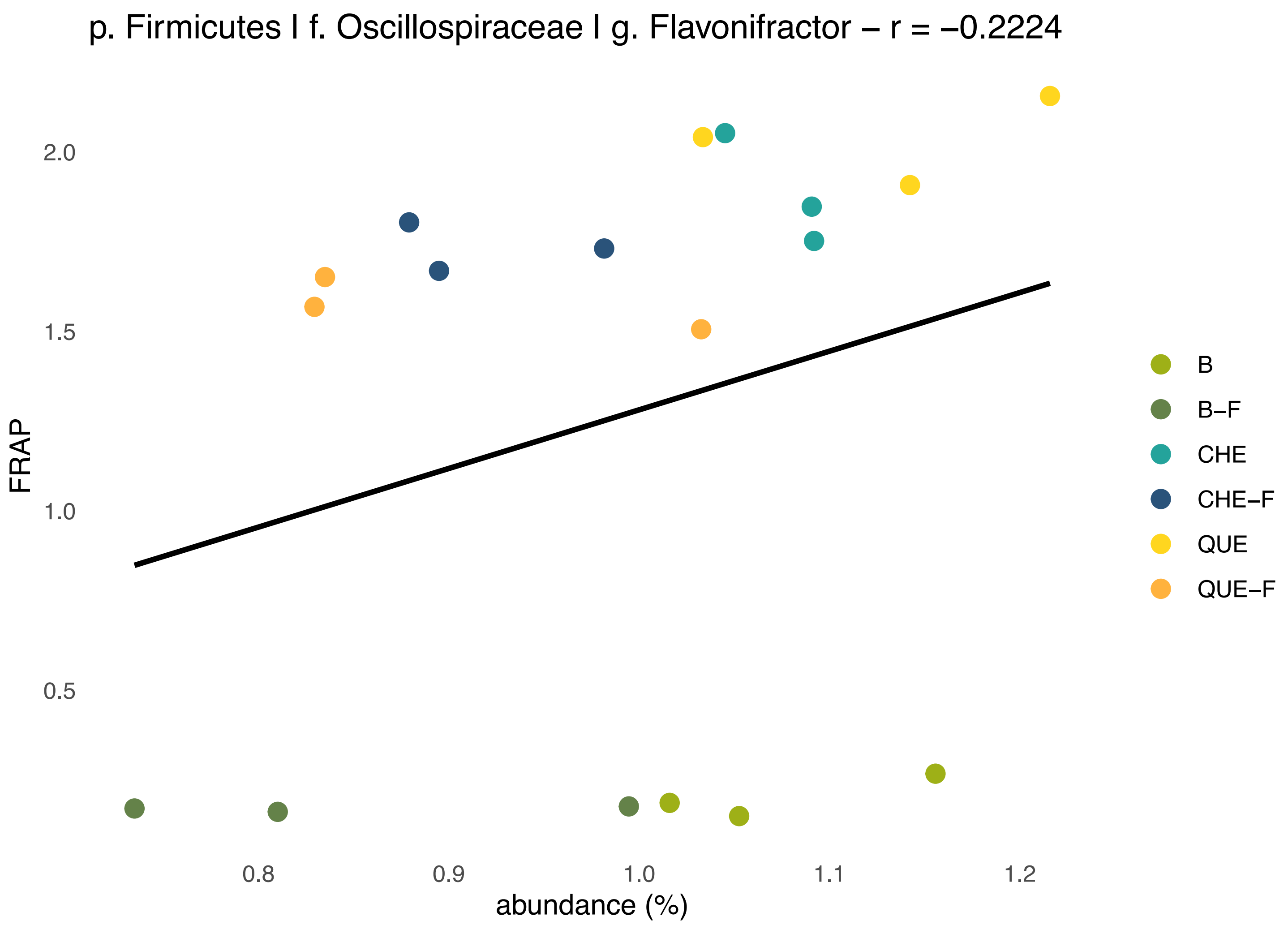
p. Firmicutes I f. Oscillospiraceae I g. Flavonifractor – r = –0.2224
FRAP
2.0
1.5
1.0
0.5
0.8
0.9
1.0
1.1
1.2
abundance (%)
B
B–F
CHE
CHE–F
QUE
QUE–F

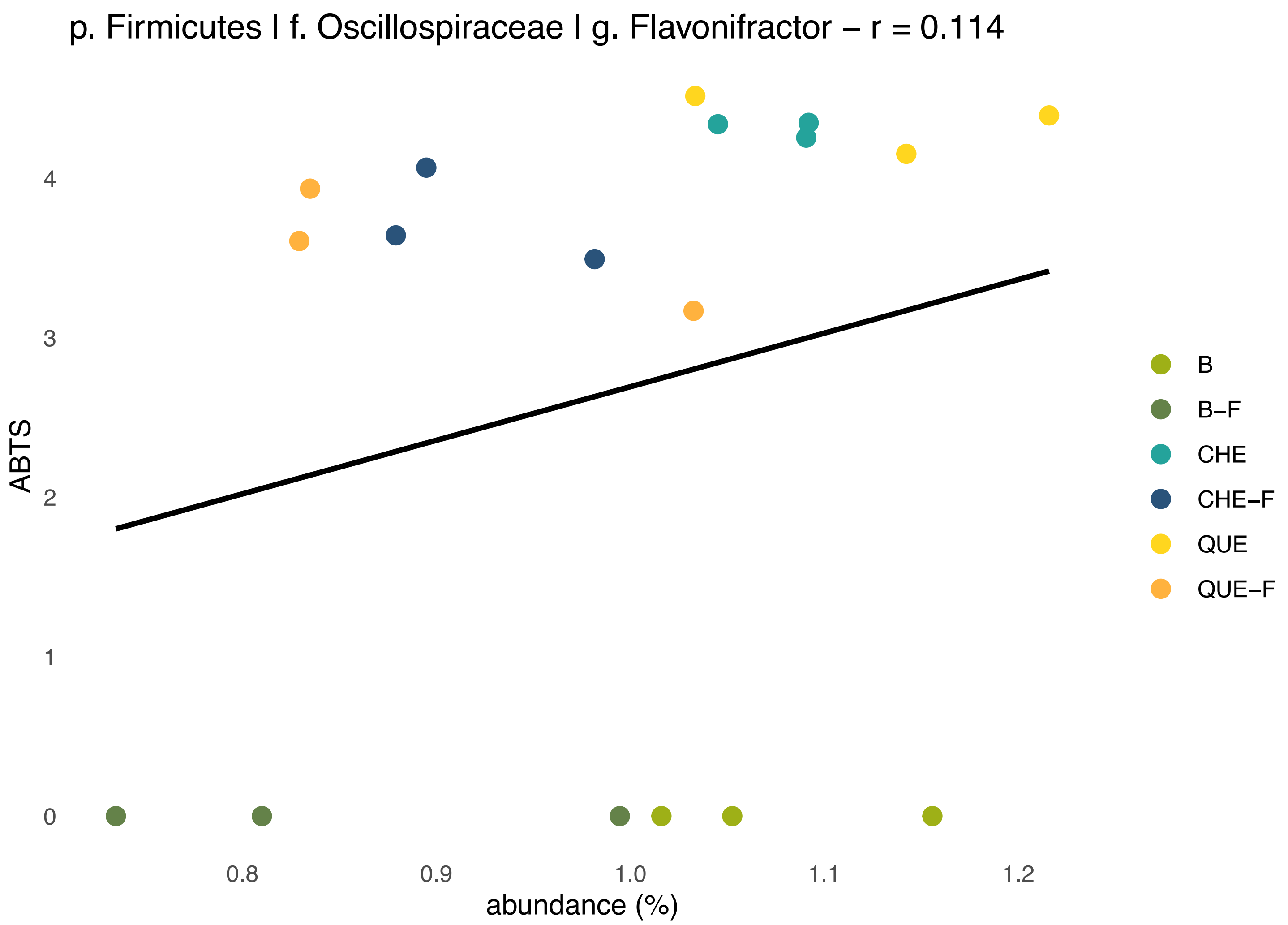

p. Firmicutes I f. Oscillospiraceae I g. Flavonifractor – r = 0.114
ABTS
0
1
2
3
4
0.8
0.9
1.0
1.1
1.2
abundance (%)
B
B–F
CHE
CHE–F
QUE
QUE–F

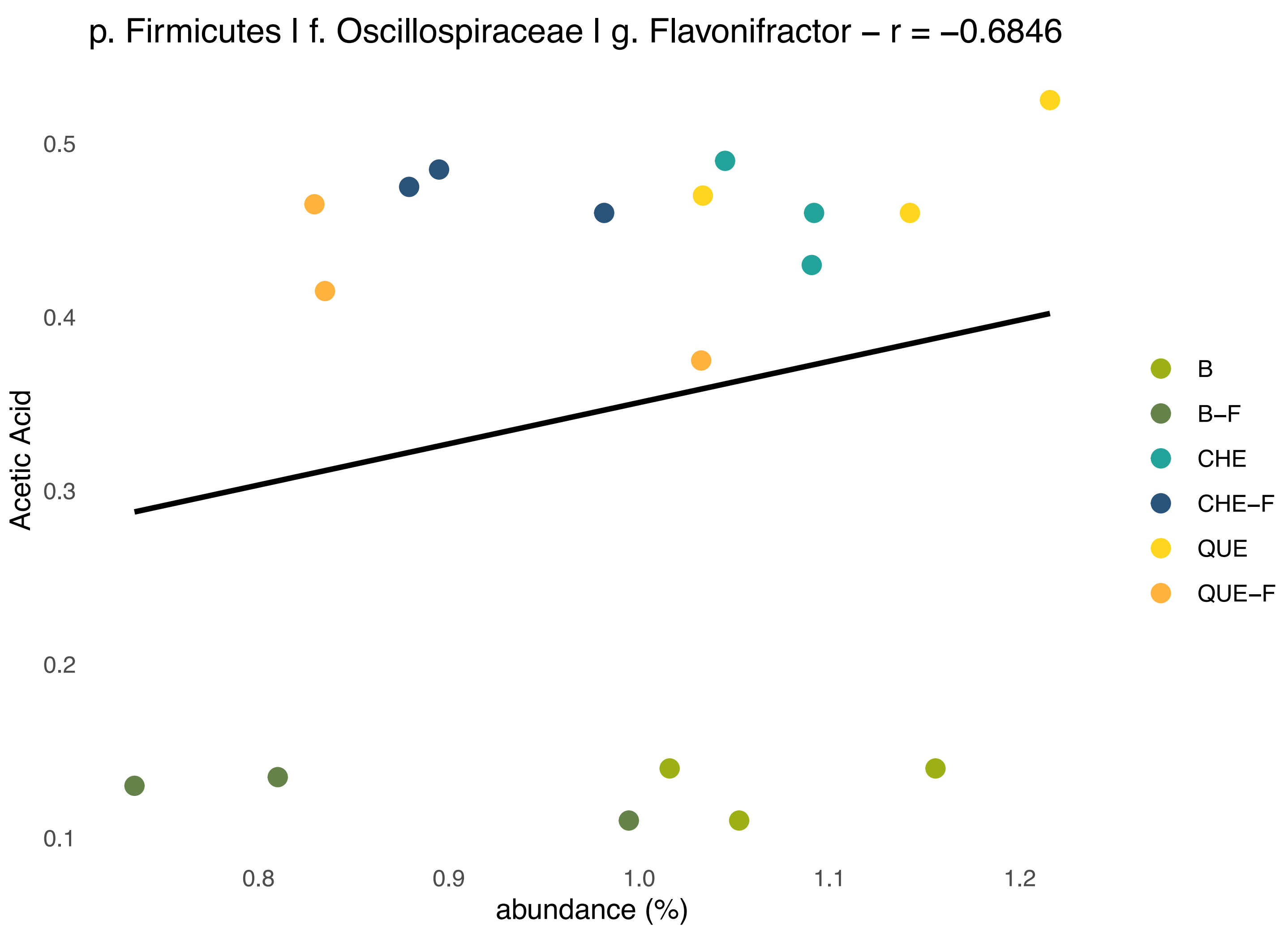
p. Firmicutes I f. Oscillospiraceae I g. Flavonifractor – r = –0.6846
Acetic Acid
0.5
0.4
0.3
0.2
0.1
0.8
0.9
1.0
1.1
1.2
abundance (%)
B
B–F
CHE
CHE–F
QUE
QUE–F

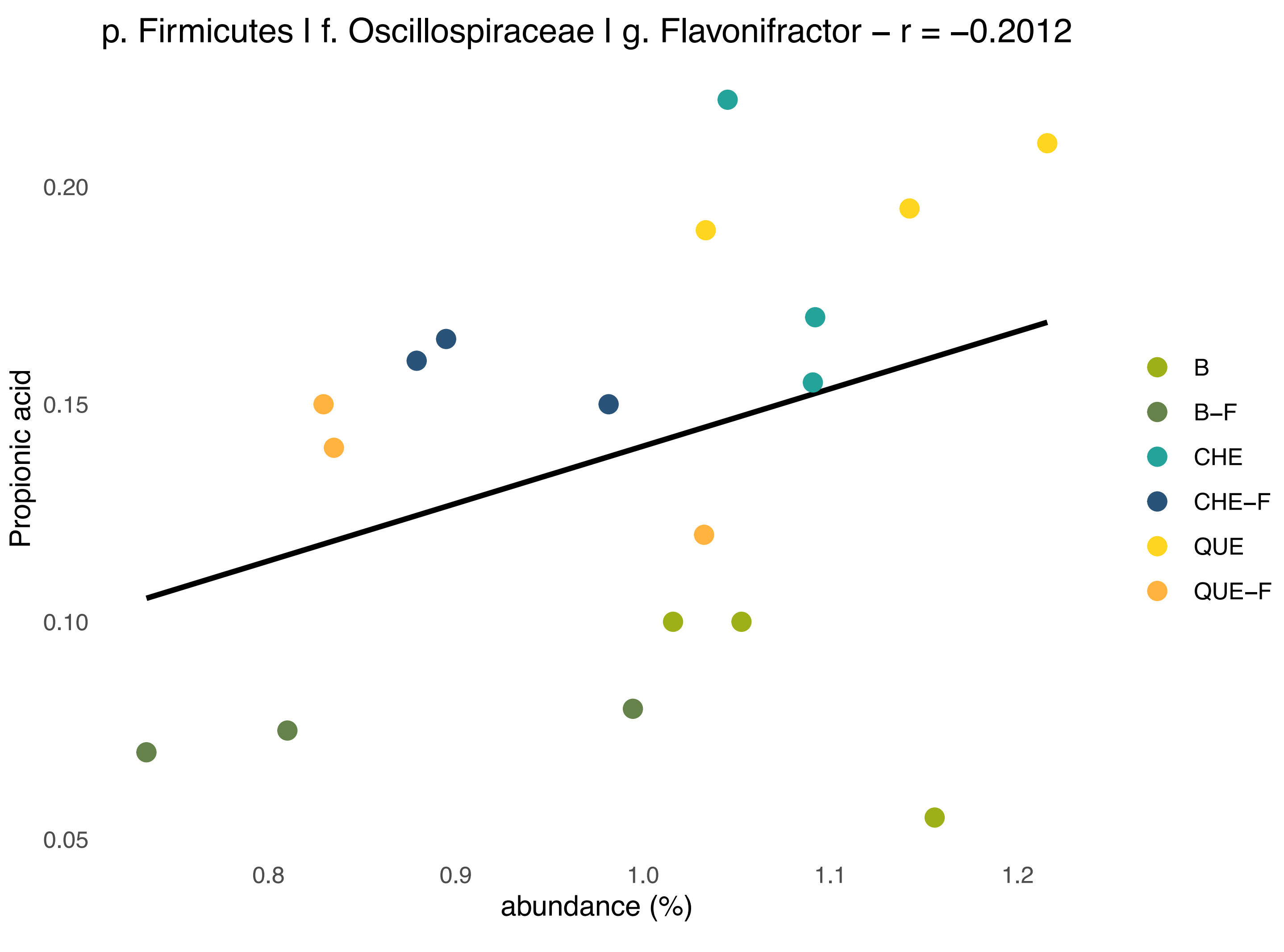
p. Firmicutes I f. Oscillospiraceae I g. Flavonifractor – r = −0.2012
Propionic acid
0.20
0.15
0.10
0.05
0.8
0.9
1.0
1.1
1.2
abundance (%)
B
B−F
CHE
CHE−F
QUE
QUE−F

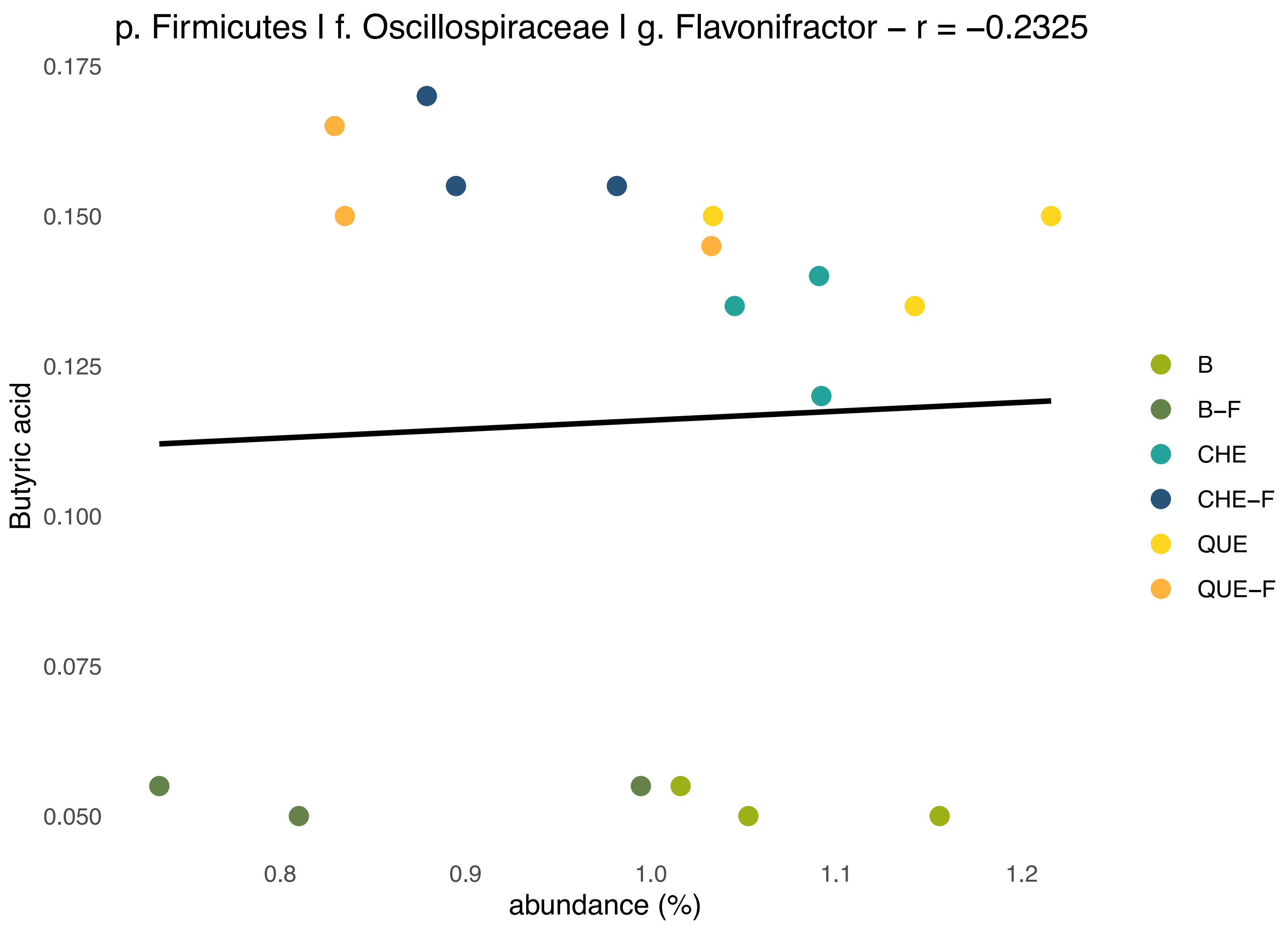
p. Firmicutes l f. Oscillospiraceae l g. Flavonifractor – r = −0.2325
0.175
0.150
0.125
0.100
0.075
0.050
Butyric acid
0.8
0.9
1.0
1.1
1.2
abundance (%)
B
B−F
CHE
CHE−F
QUE
QUE−F

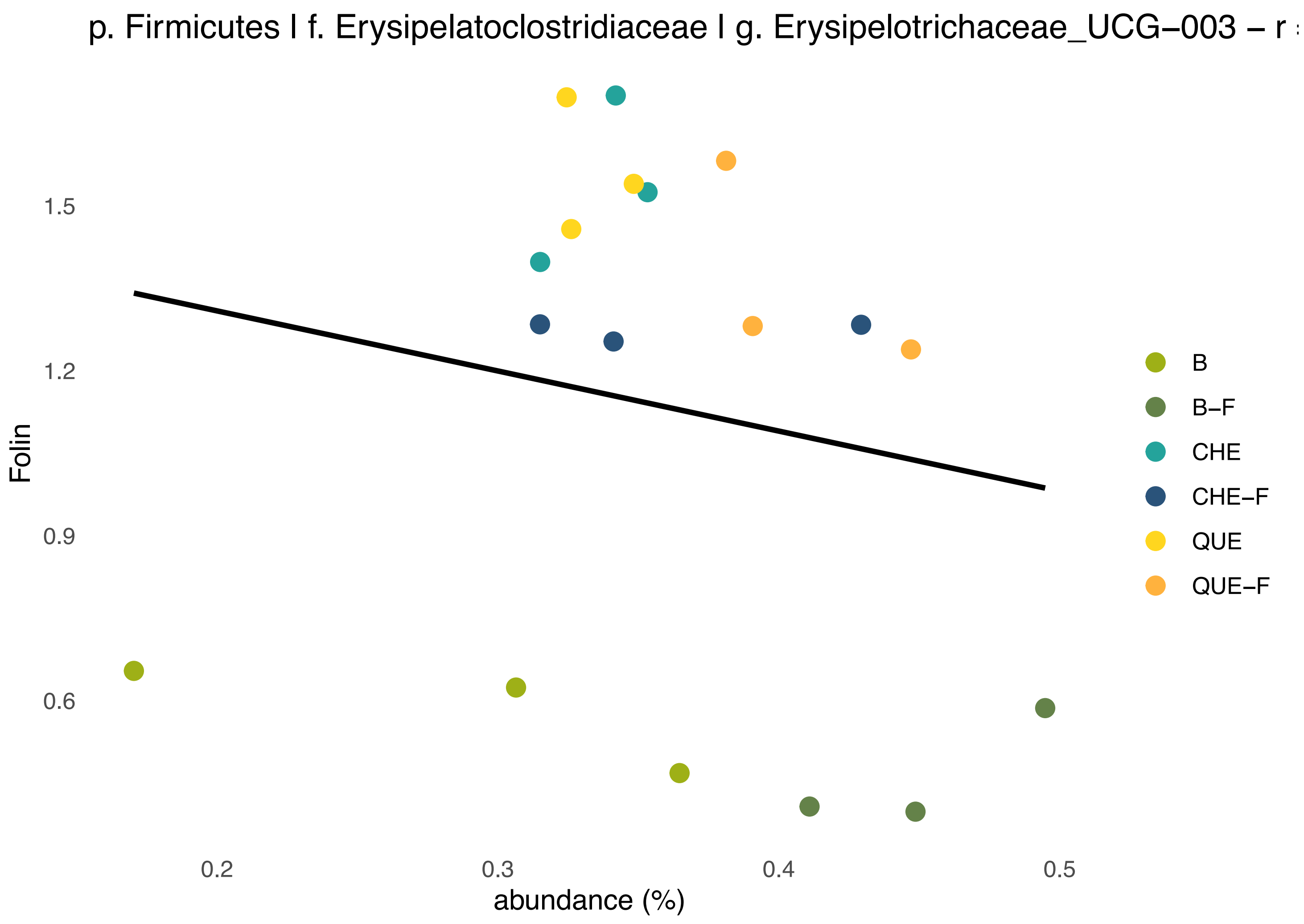
p. Firmicutes I f. Erysipelatoclostridiaceae I g. Erysipelotrichaceae_UCG−003 − r
1.5
1.2
0.9
0.6
Folin
0.2
0.3
0.4
0.5
abundance (%)
B
B−F
CHE
CHE−F
QUE
QUE−F

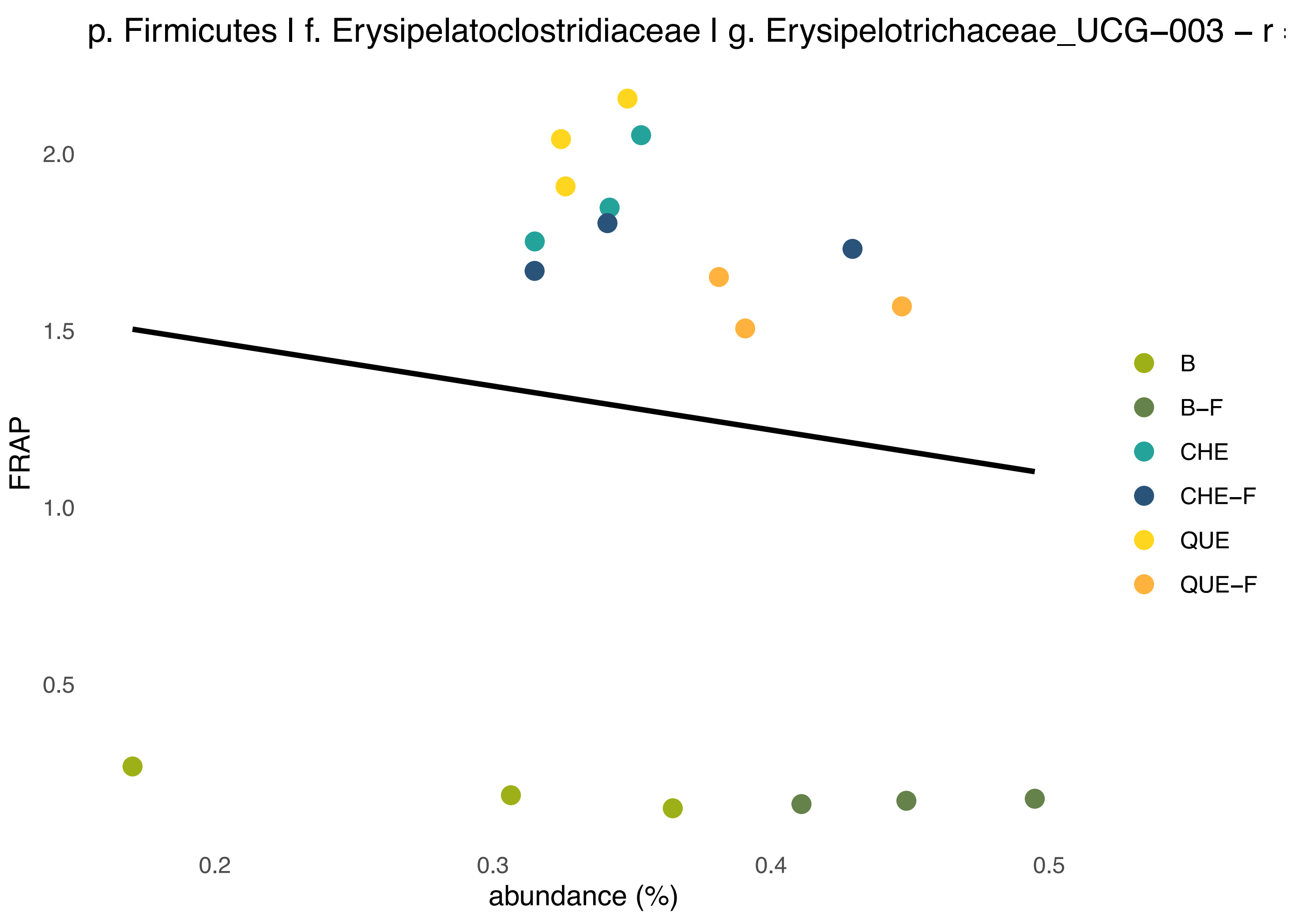
p. Firmicutes I f. Erysipelatoclostridiaceae I g. Erysipelotrichaceae_UCG−003 − r
FRAP
2.0
1.5
1.0
0.5
0.2
0.3
0.4
0.5
abundance (%)
B
B−F
CHE
CHE−F
QUE
QUE−F

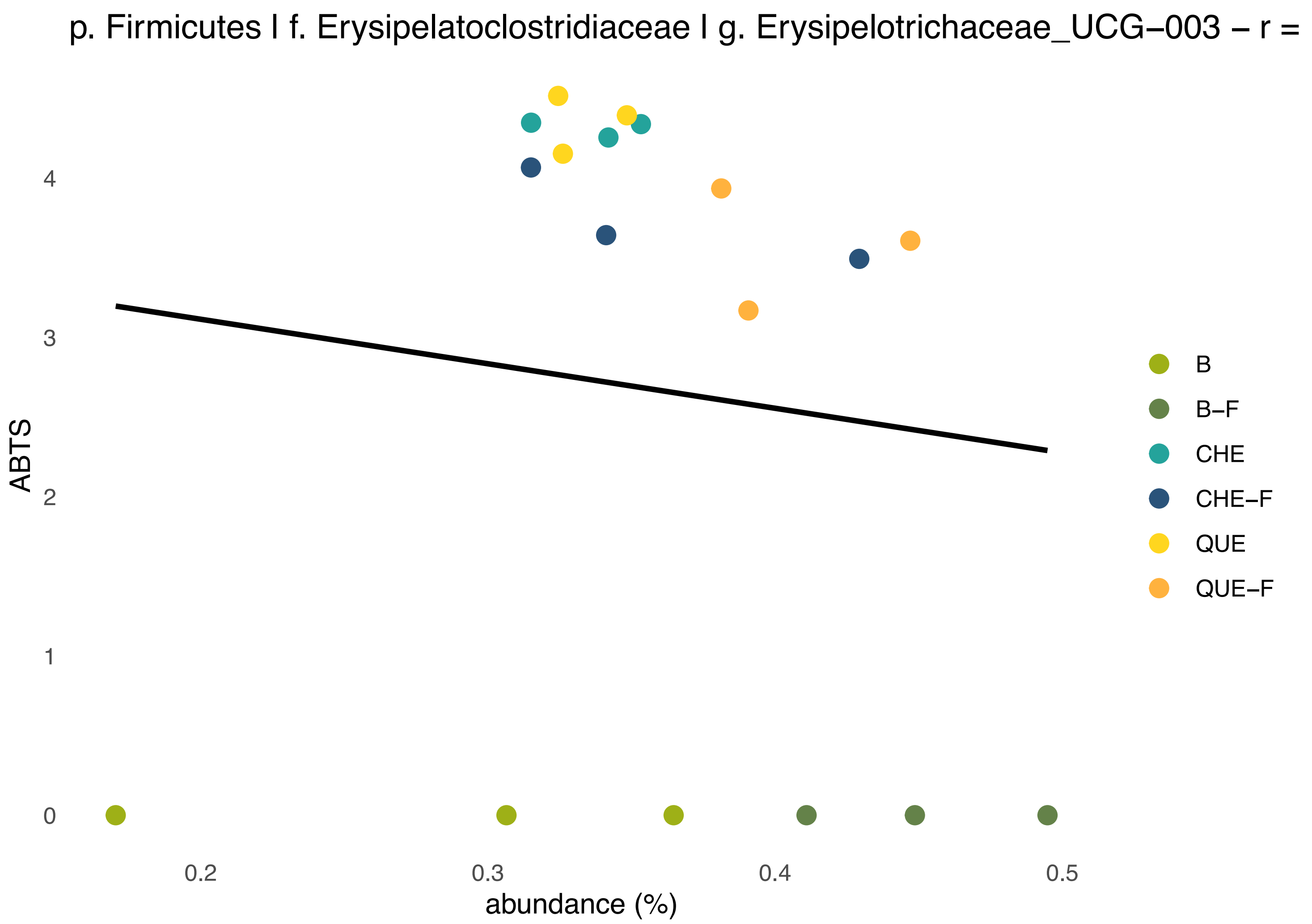

p. Firmicutes I f. Erysipelatoclostridiaceae I g. Erysipelotrichaceae_UCG−003 − r =
ABTS
4
3
2
1
0
0.2
0.3
0.4
0.5
abundance (%)
B
B−F
CHE
CHE−F
QUE
QUE−F

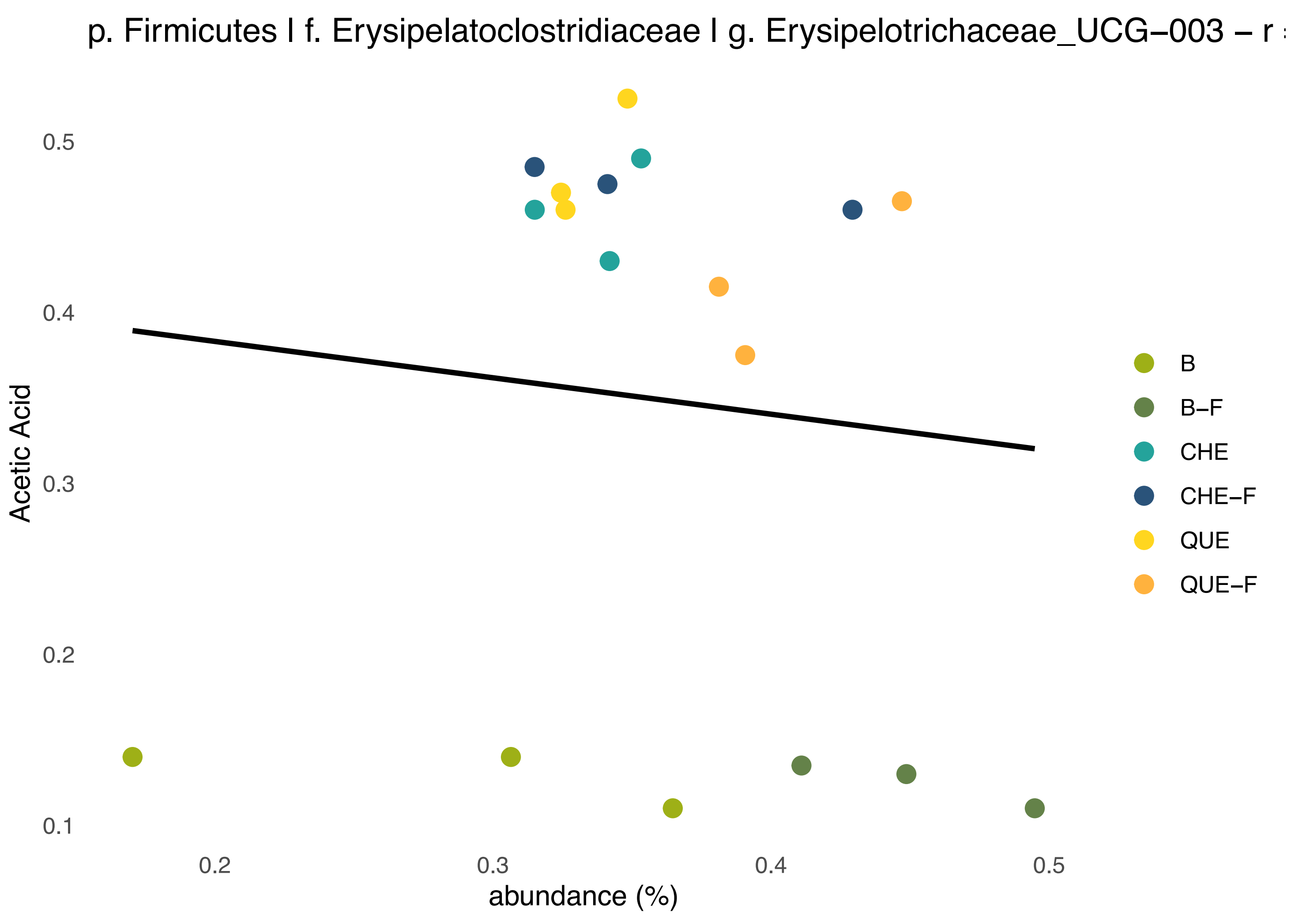

p. Firmicutes I f. Erysipelatoclostridiaceae I g. Erysipelotrichaceae_UCG−003 − r
Acetic Acid
0.5
0.4
0.3
0.2
0.1
0.2
0.3
0.4
0.5
abundance (%)
B
B−F
CHE
CHE−F
QUE
QUE−F

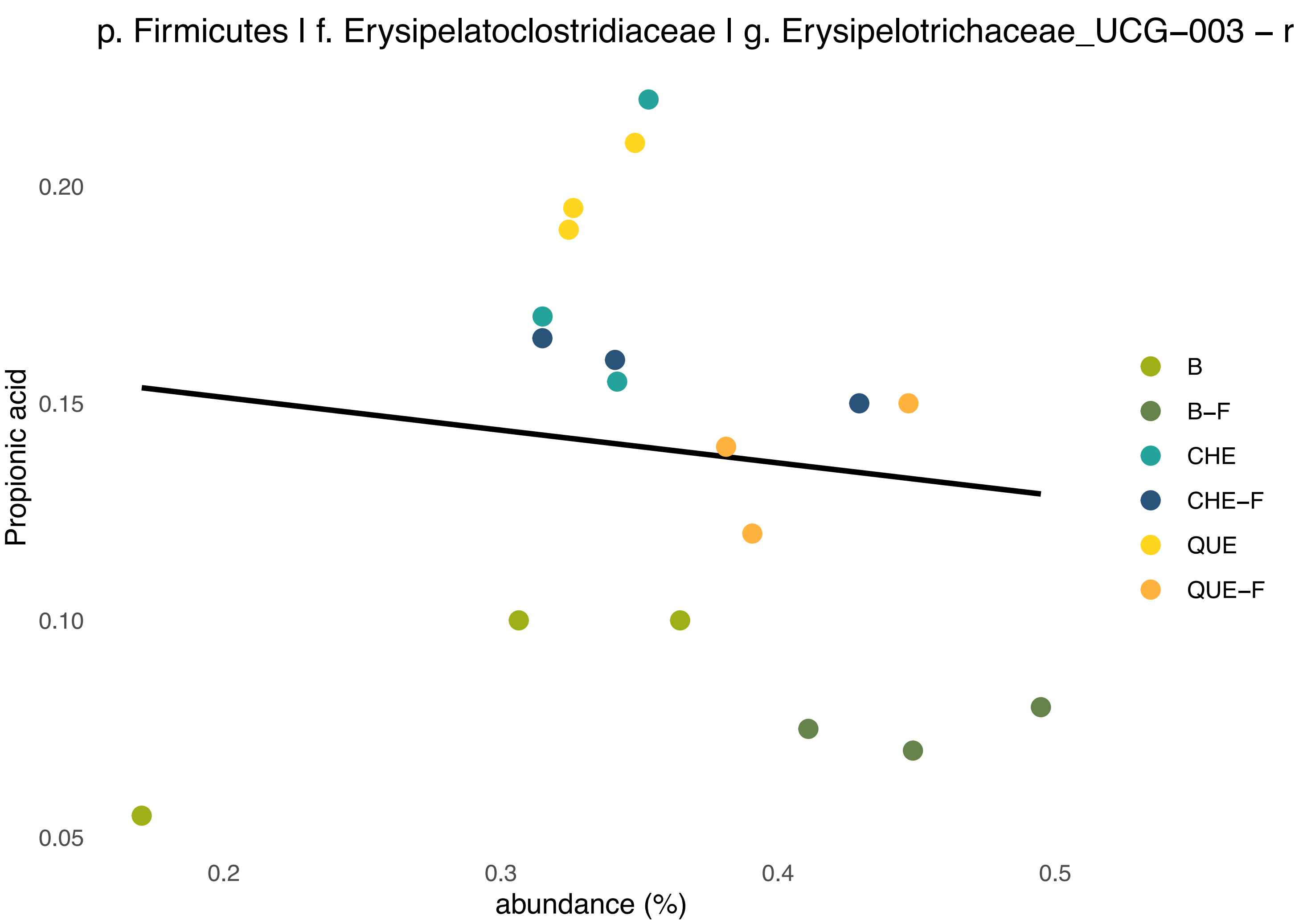
p. Firmicutes l f. Erysipelatoclostridiaceae l g. Erysipelotrichaceae_UCG−003 – r
0.20
0.15
0.10
0.05
Propionic acid
0.2
0.3
0.4
0.5
abundance (%)
B
B−F
CHE
CHE−F
QUE
QUE−F

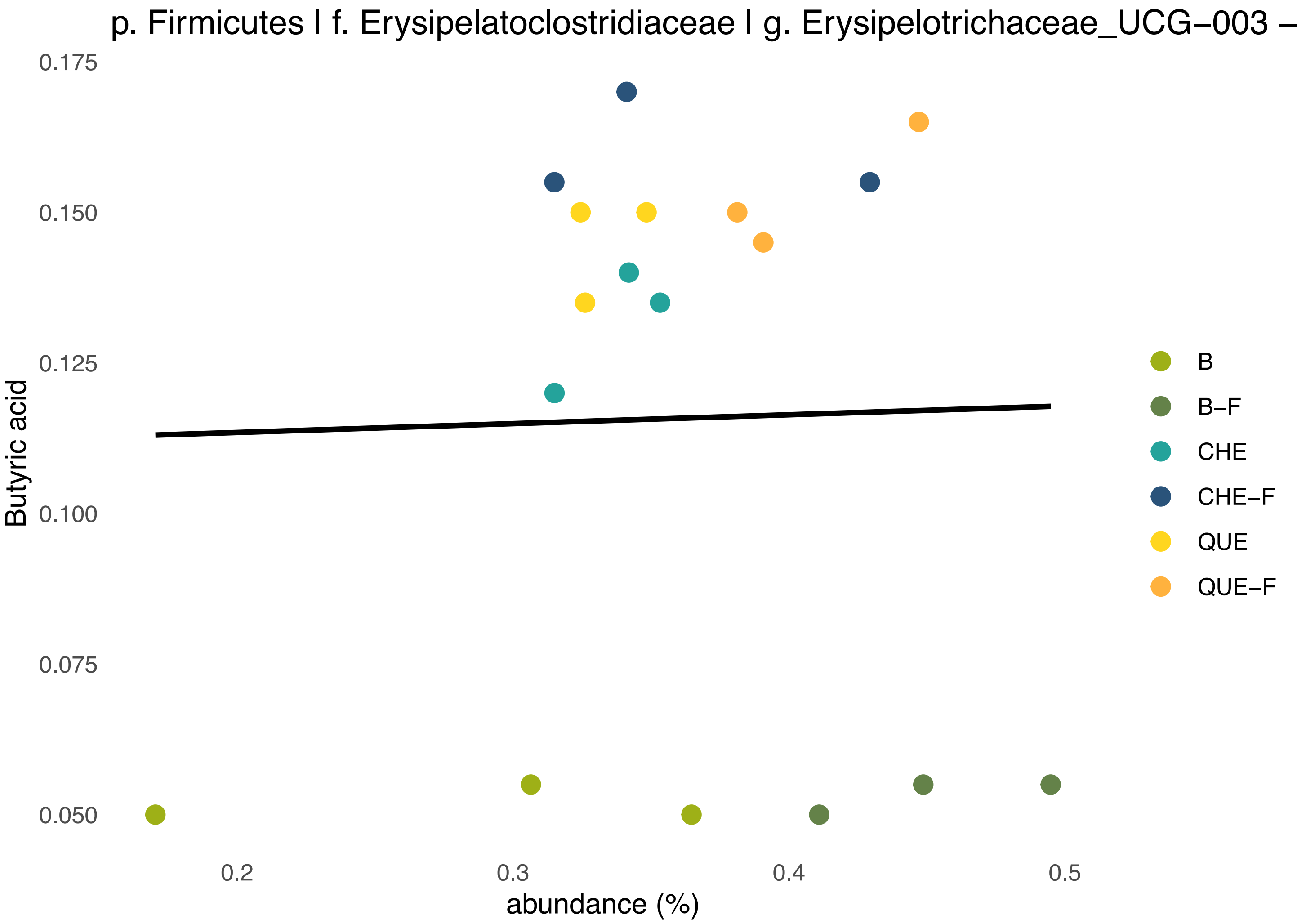
p. Firmicutes I f. Erysipelatoclostridiaceae I g. Erysipelotrichaceae_UCG−003 –
Butyric acid
0.175
0.150
0.125
0.100
0.075
0.050
0.2
0.3
0.4
0.5
abundance (%)
B
B−F
CHE
CHE−F
QUE
QUE−F

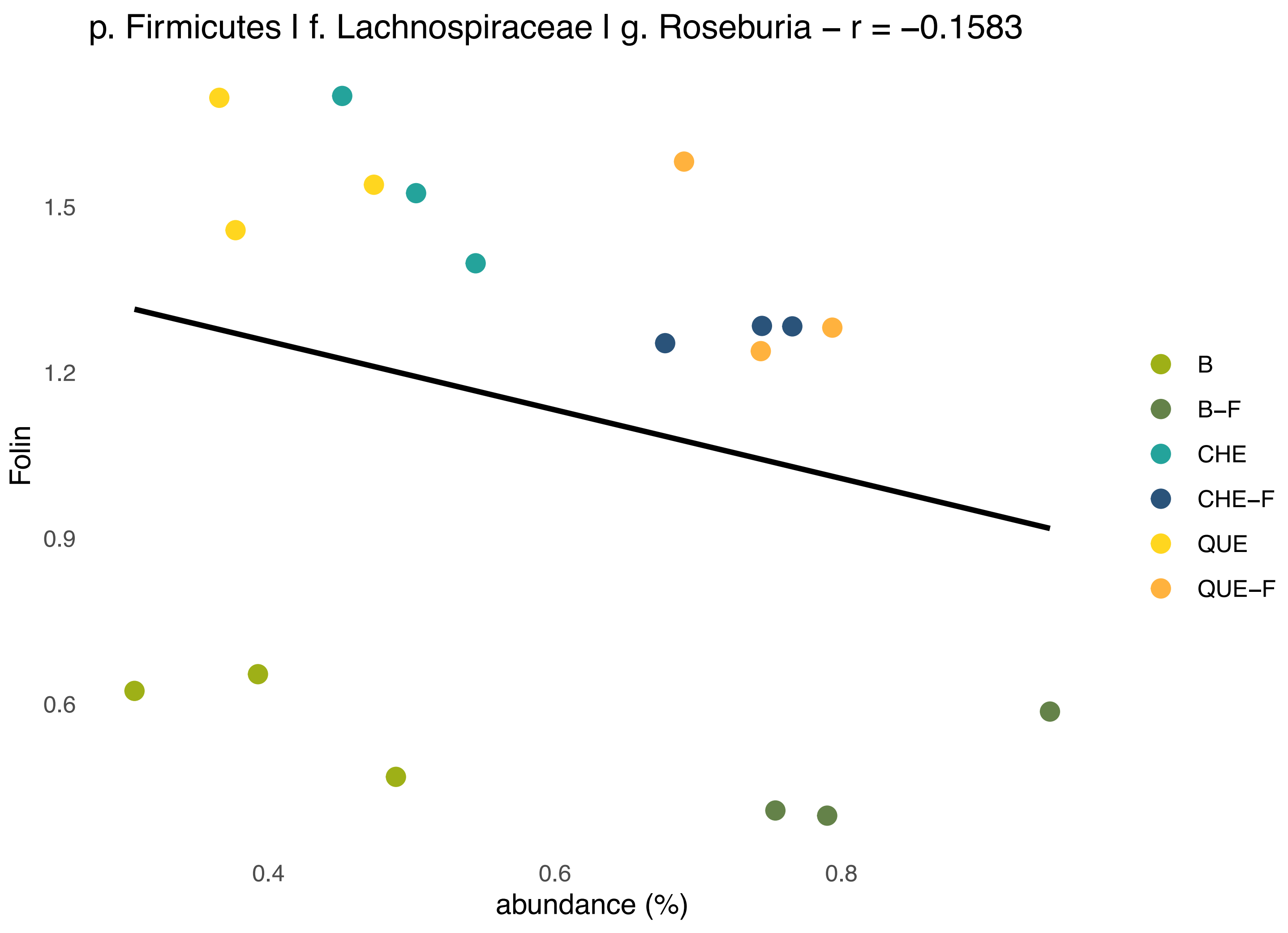
p. Firmicutes l f. Lachnospiraceae l g. Roseburia – r = –0.1583
1.5
1.2
0.9
0.6
Folin
0.4
0.6
0.8
abundance (%)
B
B–F
CHE
CHE–F
QUE
QUE–F

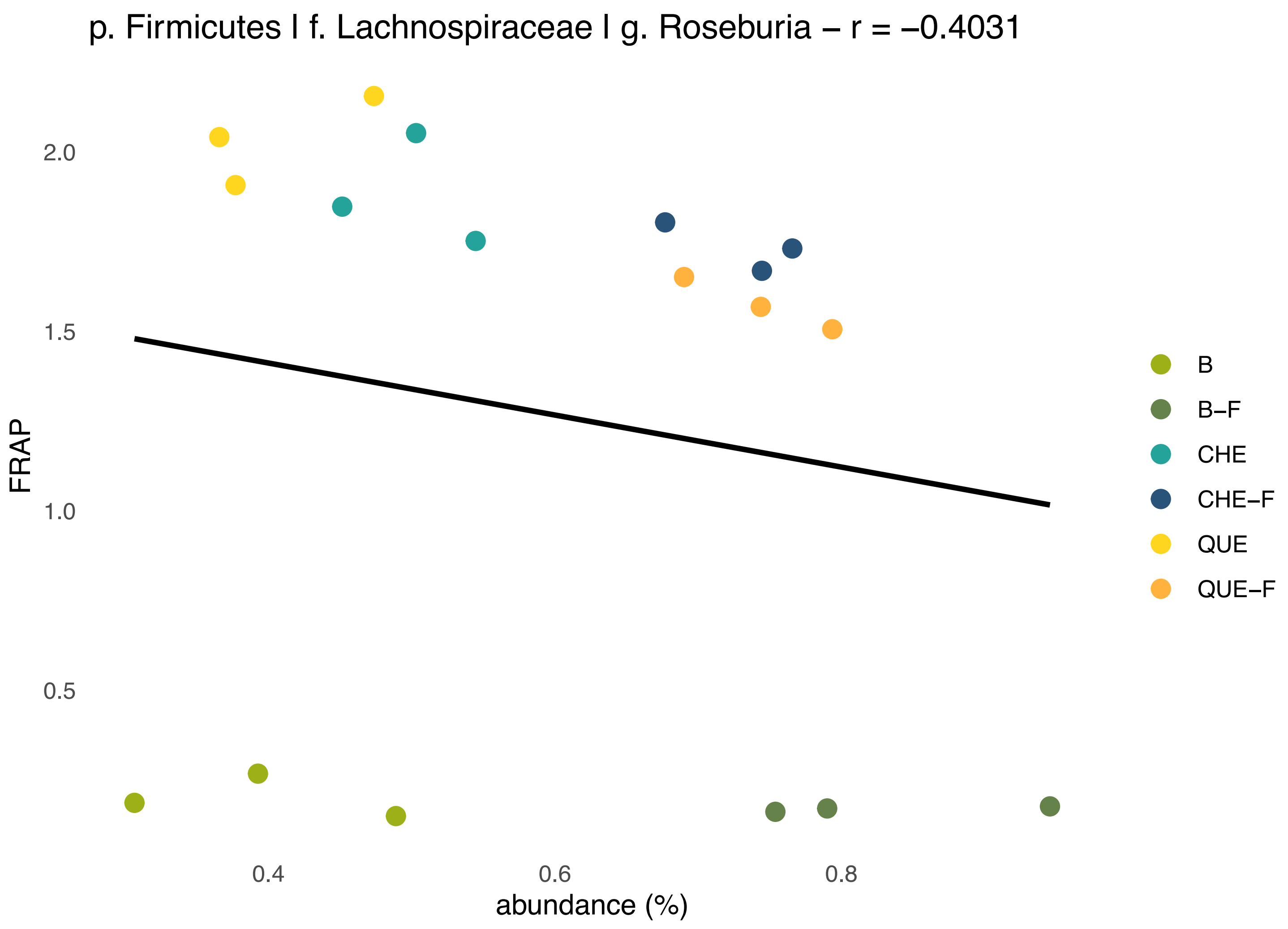
p. Firmicutes I f. Lachnospiraceae I g. Roseburia − r = −0.4031
FRAP
2.0
1.5
1.0
0.5
0.4
0.6
0.8
abundance (%)
B
B−F
CHE
CHE−F
QUE
QUE−F

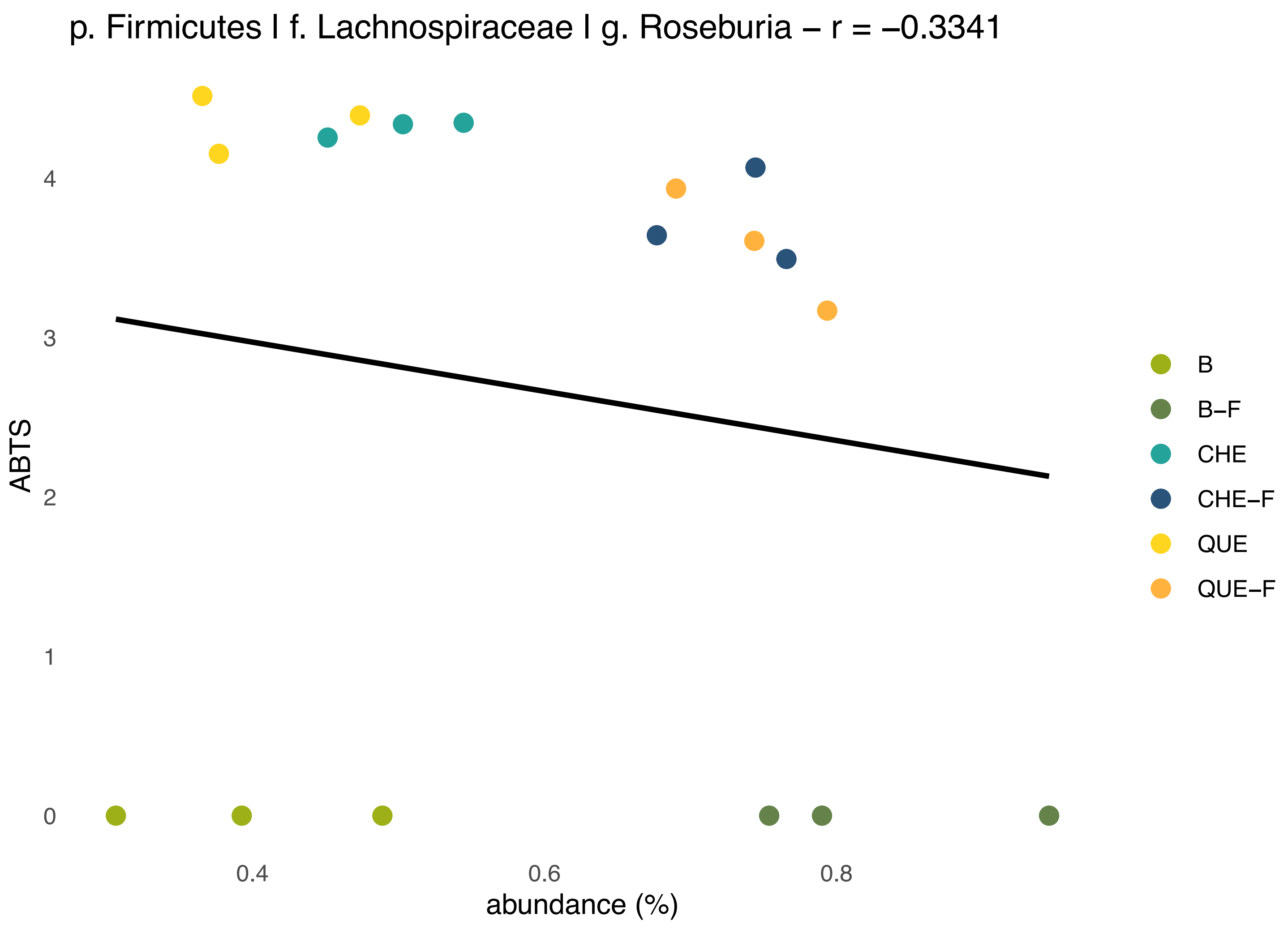
p. Firmicutes | f. Lachnospiraceae | g. Roseburia – r = −0.3341
ABTS
0
1
2
3
4
0.4
0.6
0.8
abundance (%)
B
B−F
CHE
CHE−F
QUE
QUE−F

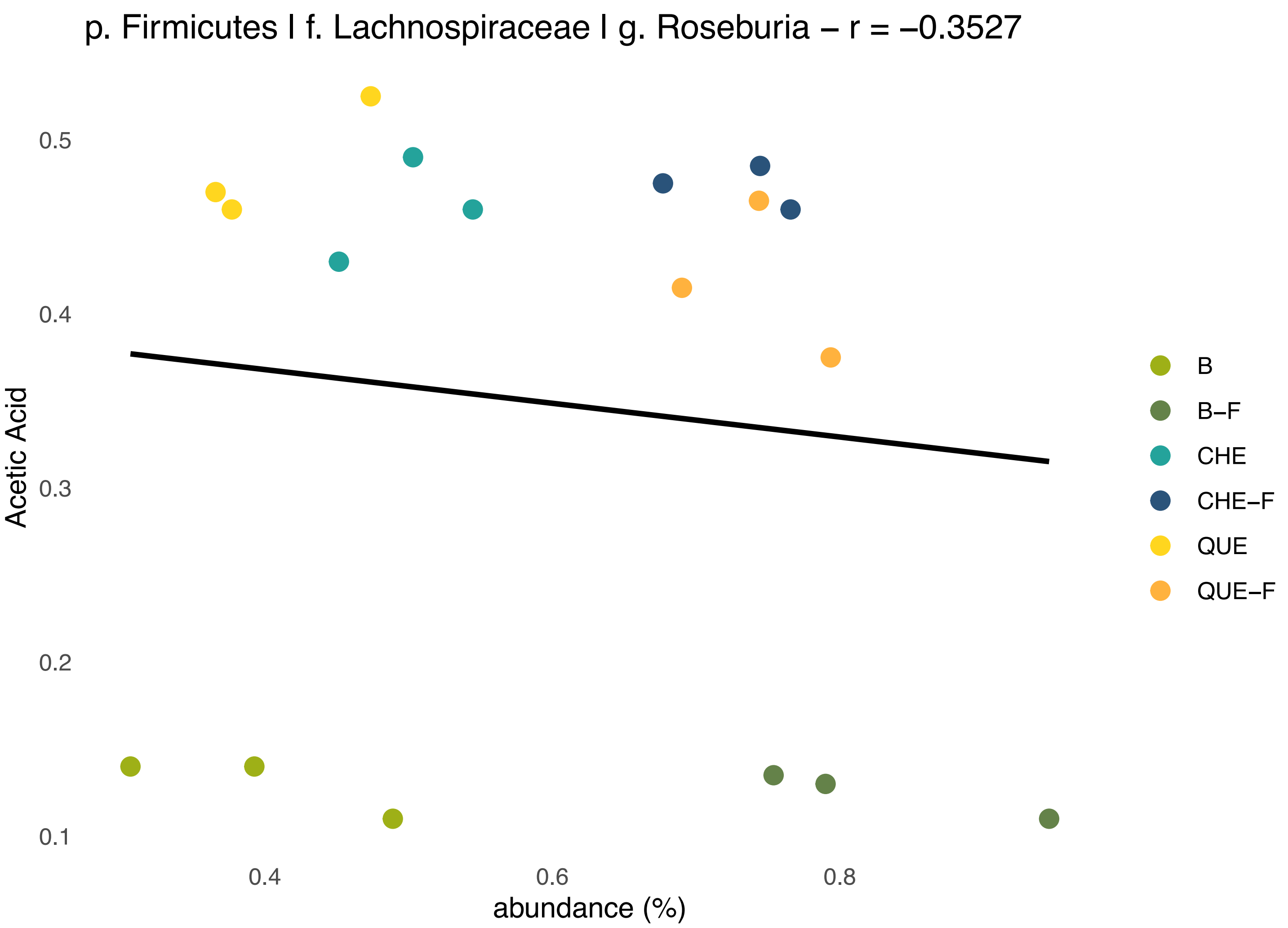

p. Firmicutes | f. Lachnospiraceae | g. Roseburia – r = –0.3527
Acetic Acid
0.5
0.4
0.3
0.2
0.1
0.4
0.6
0.8
abundance (%)
B
B–F
CHE
CHE–F
QUE
QUE–F

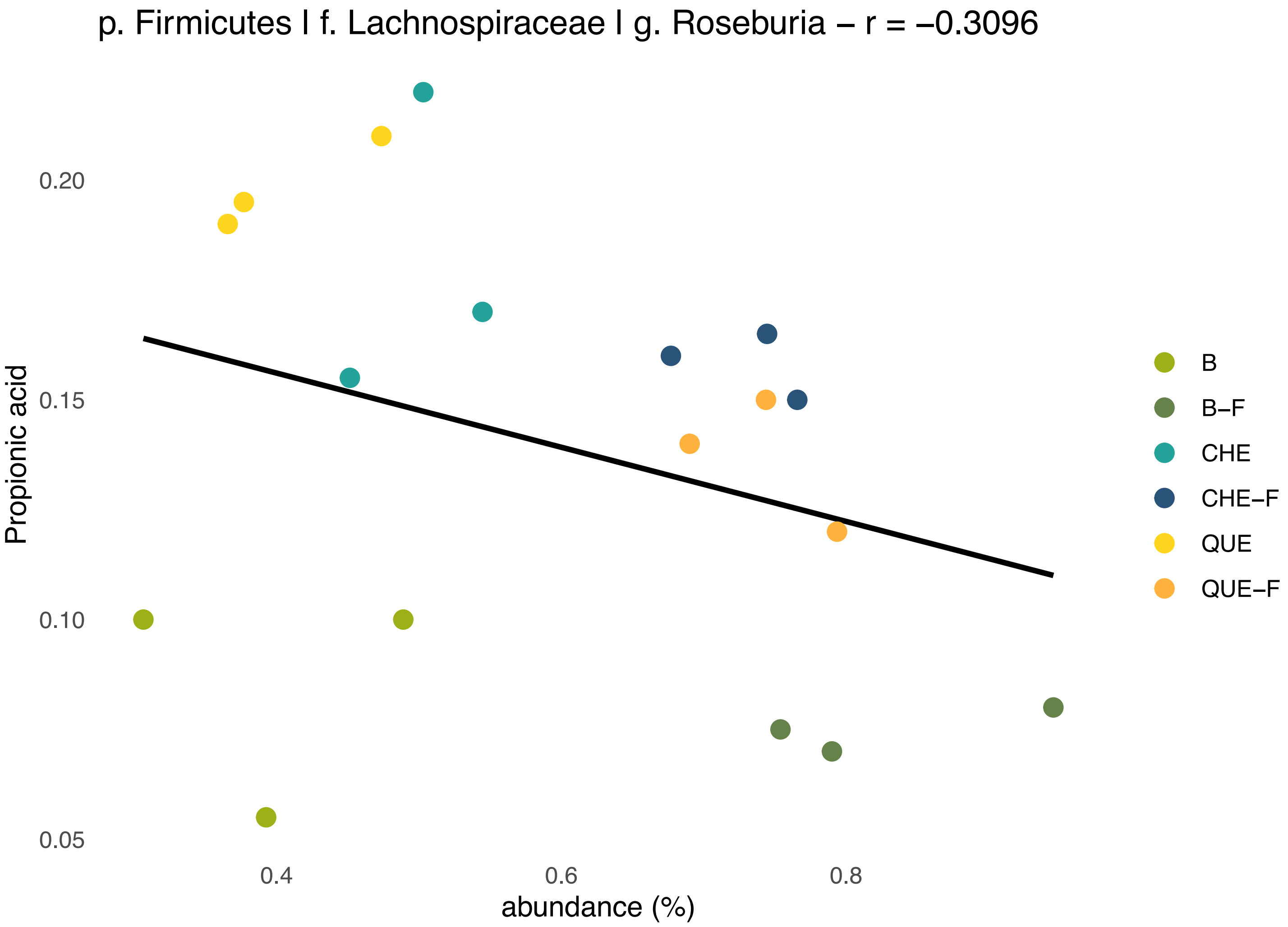
p. Firmicutes I f. Lachnospiraceae I g. Roseburia – r = –0.3096
Propionic acid
0.20
0.15
0.10
0.05
0.4
0.6
0.8
abundance (%)
B
B–F
CHE
CHE–F
QUE
QUE–F

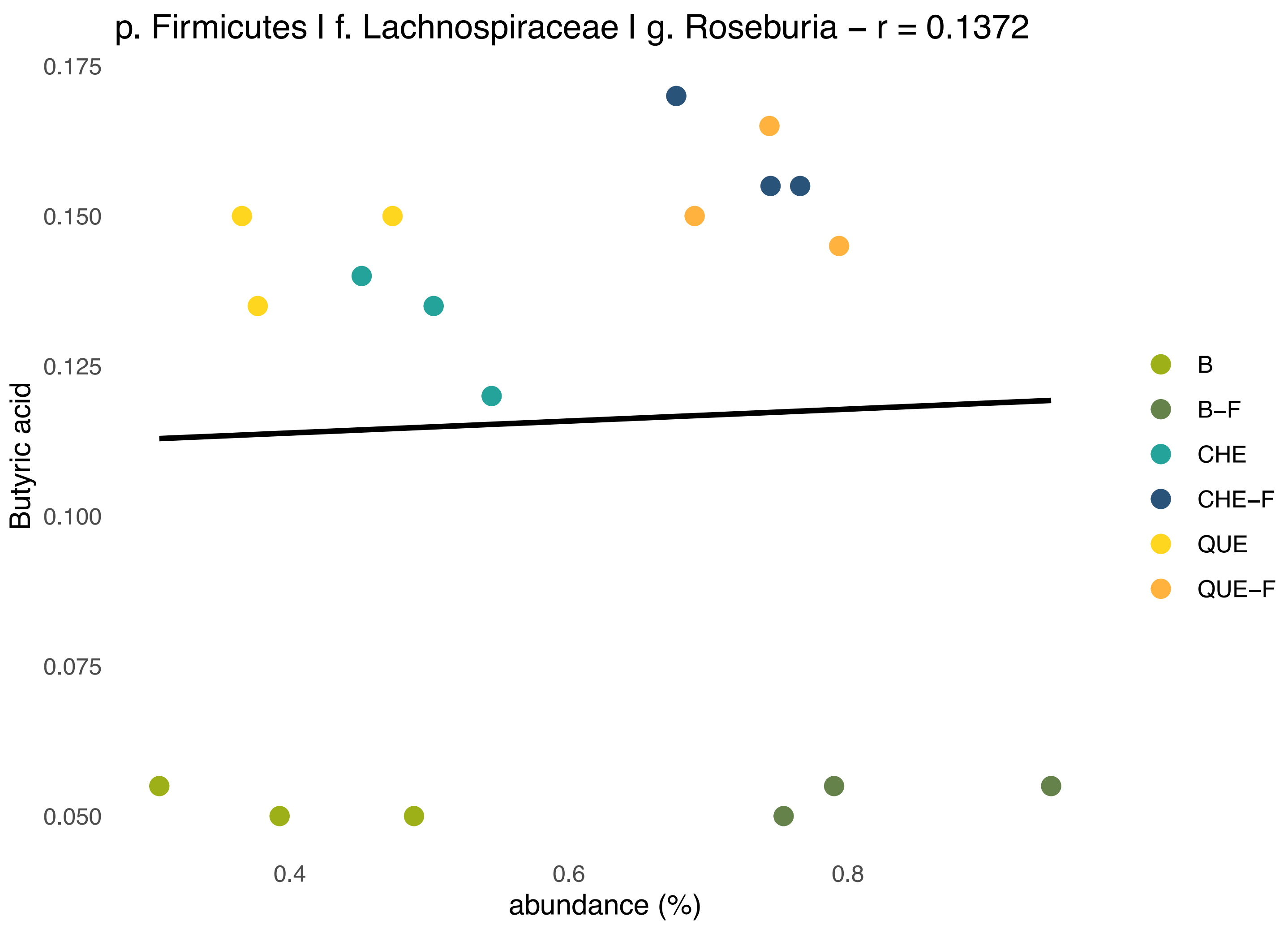

p. Firmicutes I f. Lachnospiraceae I g. Roseburia – r = 0.1372
0.175
0.150
0.125
0.100
0.075
0.050
Butyric acid
0.4
0.6
0.8
abundance (%)
B
B–F
CHE
CHE–F
QUE
QUE–F

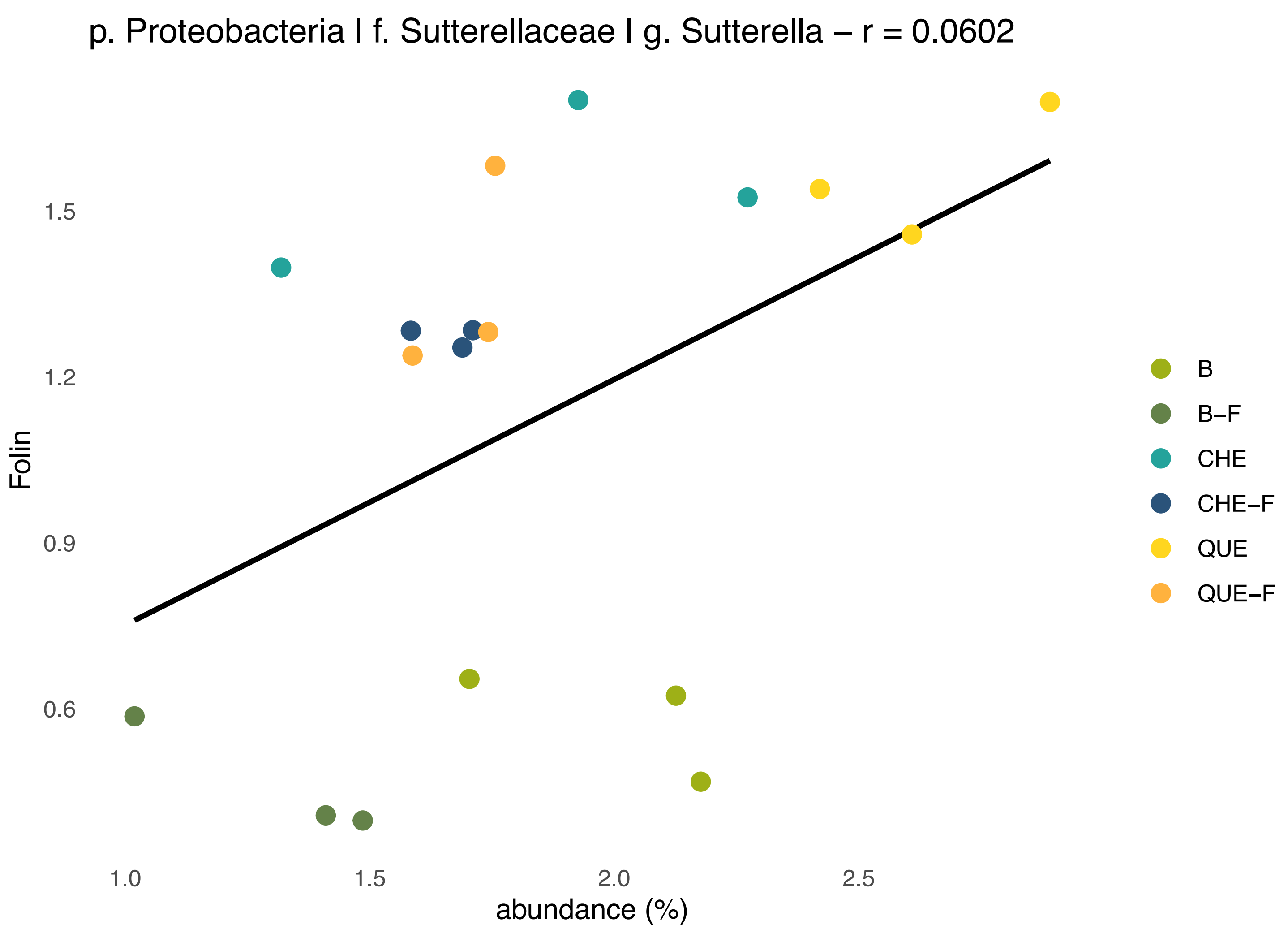
p. Proteobacteria I f. Sutterellaceae I g. Sutterella – r = 0.0602
Folin
1.5
1.2
0.9
0.6
1.0
1.5
2.0
2.5
abundance (%)
B
B–F
CHE
CHE–F
QUE
QUE–F

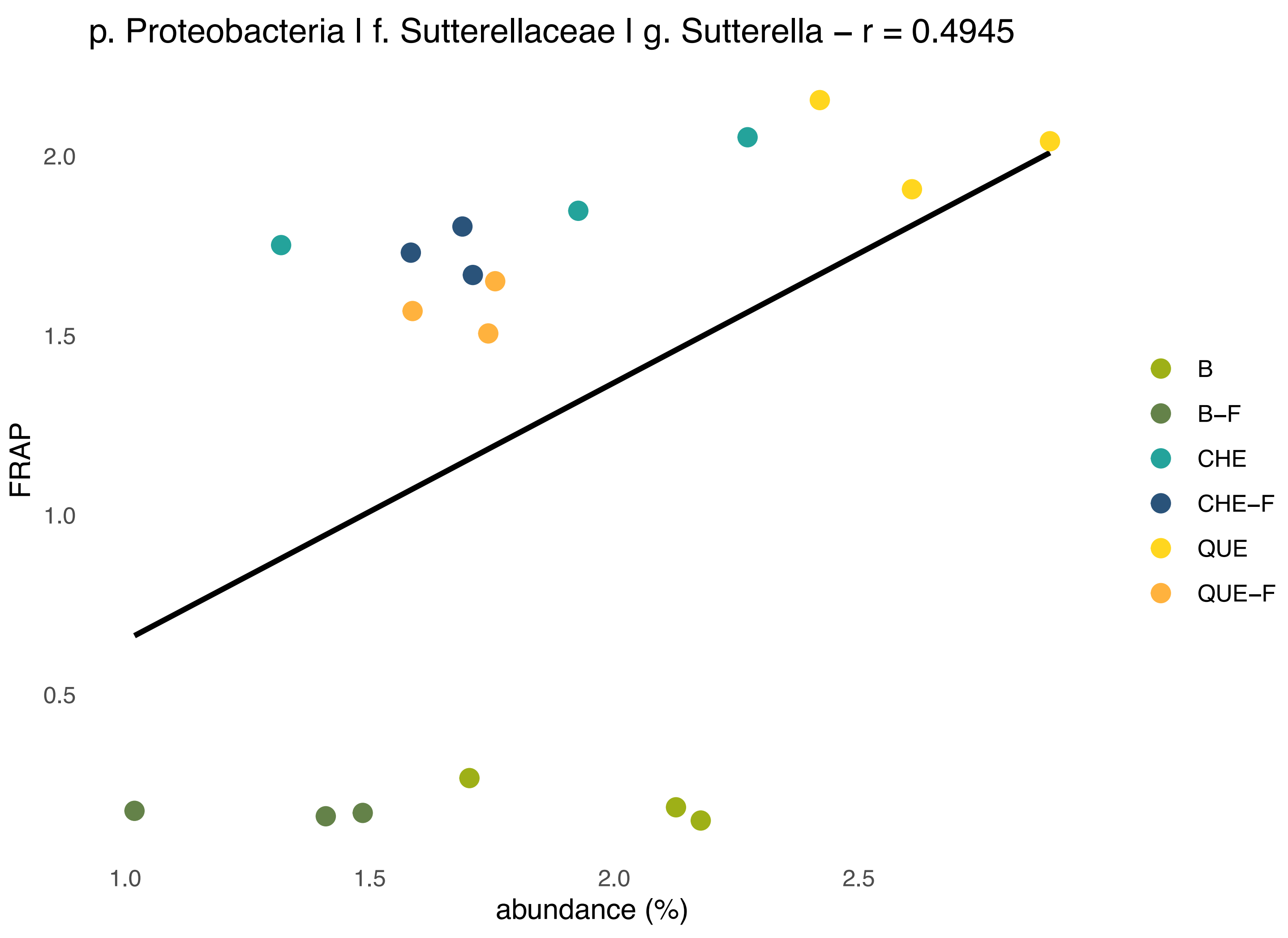
p. Proteobacteria I f. Sutterellaceae I g. Sutterella – r = 0.4945
FRAP
2.0
1.5
1.0
0.5
1.0
1.5
2.0
2.5
abundance (%)
B
B–F
CHE
CHE–F
QUE
QUE–F

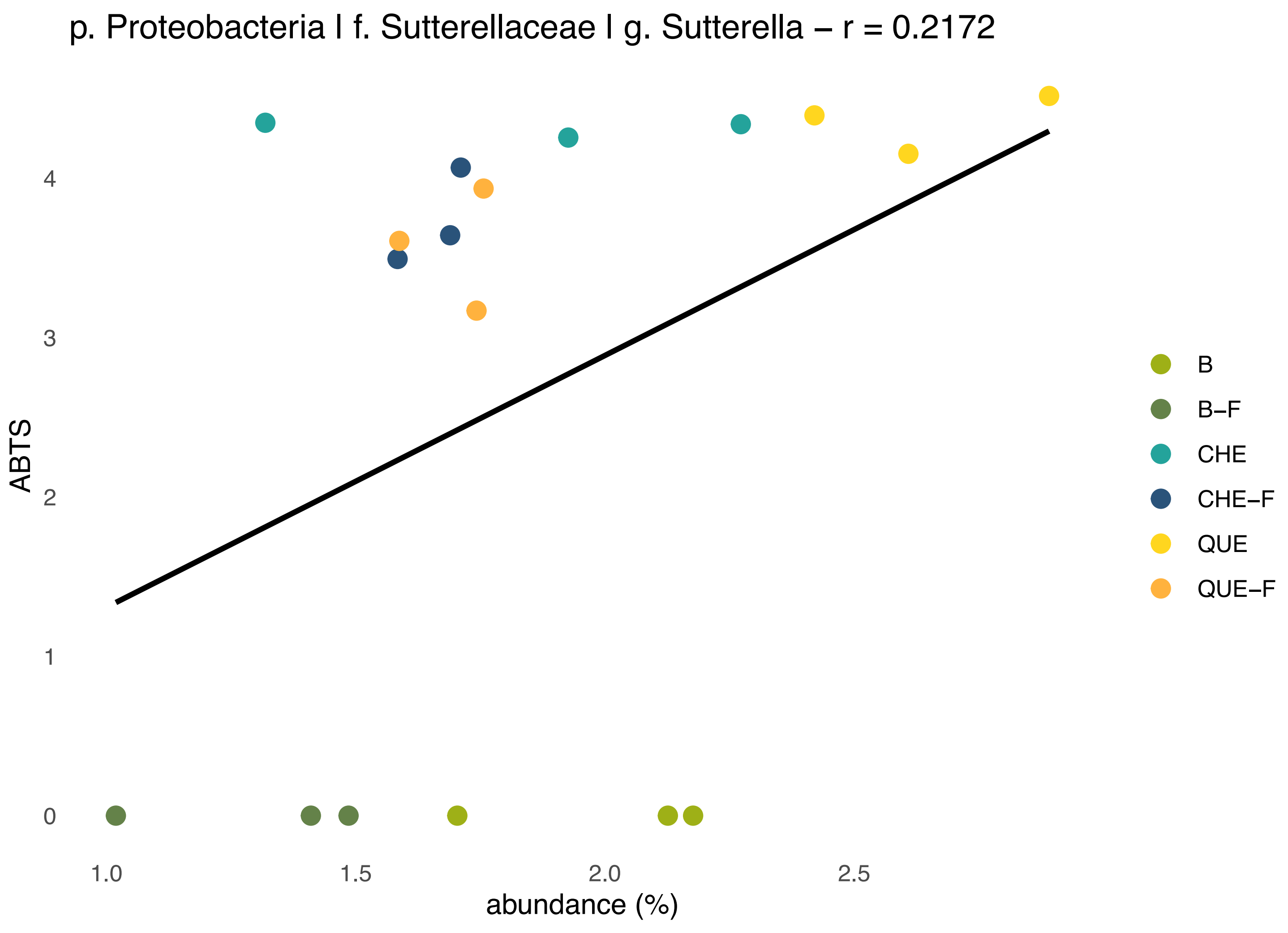
p. Proteobacteria I f. Sutterellaceae I g. Sutterella – r = 0.2172
ABTS
0
1
2
3
4
1.0
1.5
2.0
2.5
abundance (%)
B
B–F
CHE
CHE–F
QUE
QUE–F

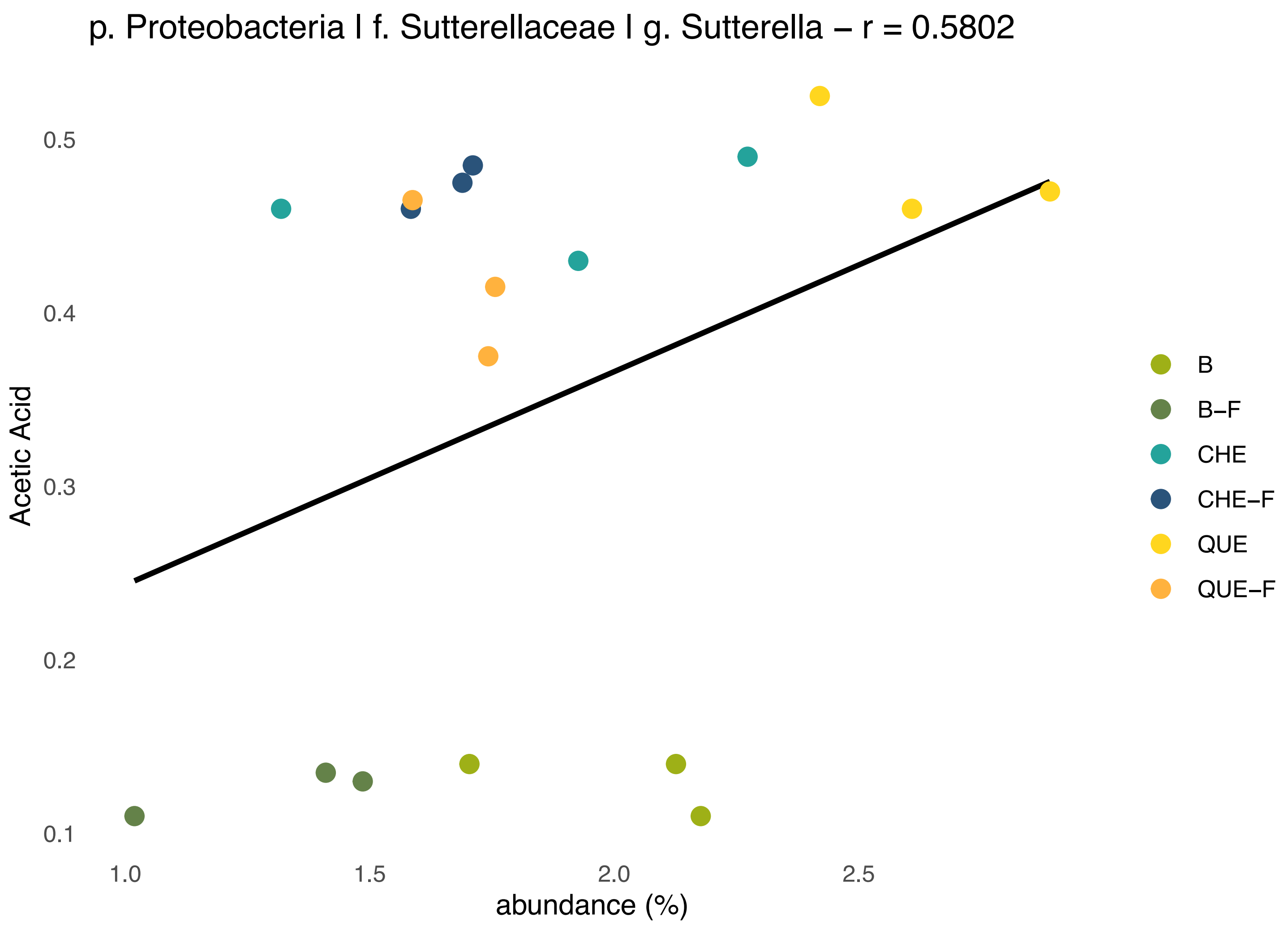
p. Proteobacteria I f. Sutterellaceae I g. Sutterella − r = 0.5802
Acetic Acid
0.5
0.4
0.3
0.2
0.1
1.0
1.5
2.0
2.5
abundance (%)
B
B−F
CHE
CHE−F
QUE
QUE−F

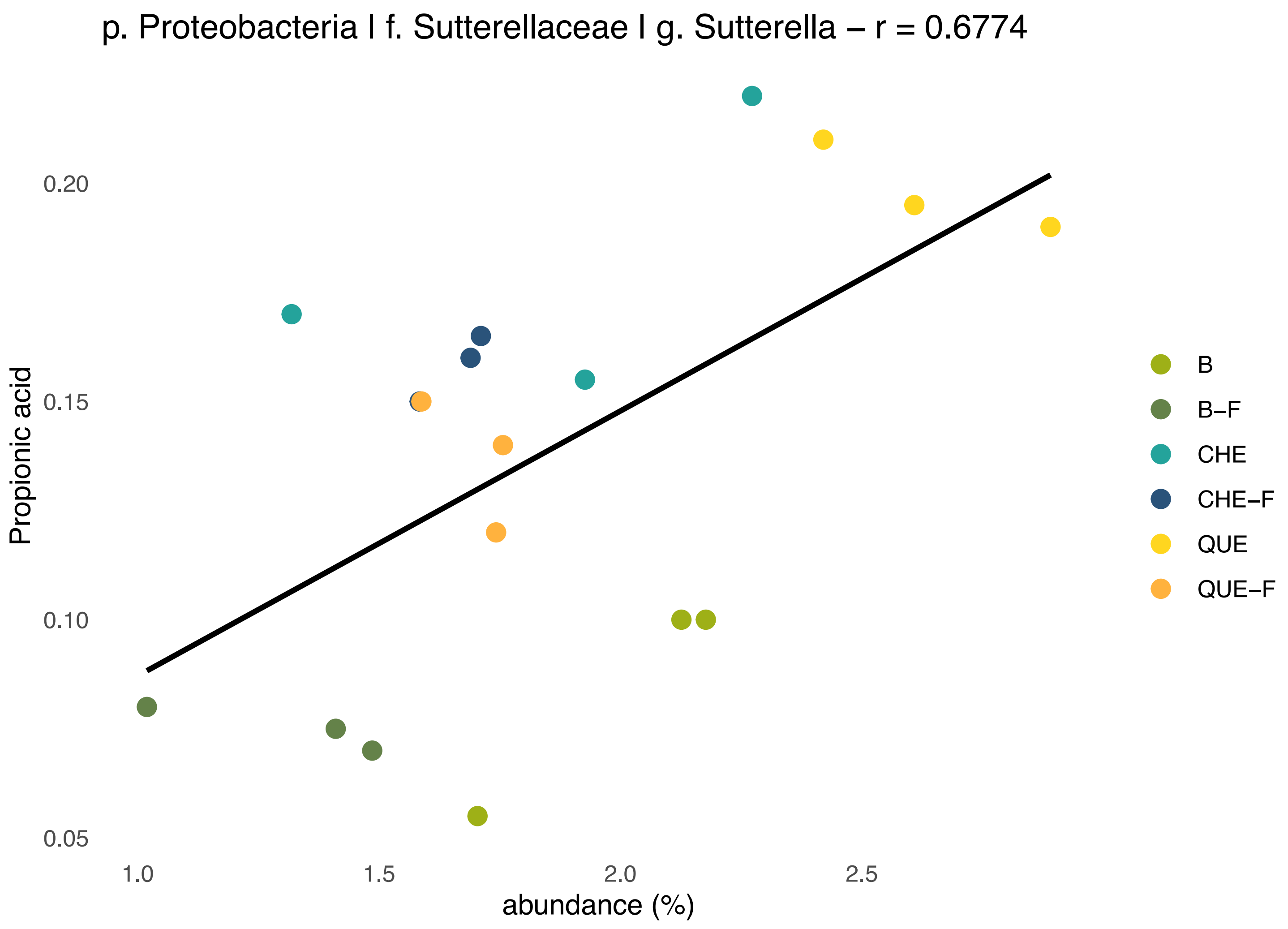

p. Proteobacteria I f. Sutterellaceae I g. Sutterella – r = 0.6774
Propionic acid
0.20
0.15
0.10
0.05
1.0
1.5
2.0
2.5
abundance (%)
B
B–F
CHE
CHE–F
QUE
QUE–F

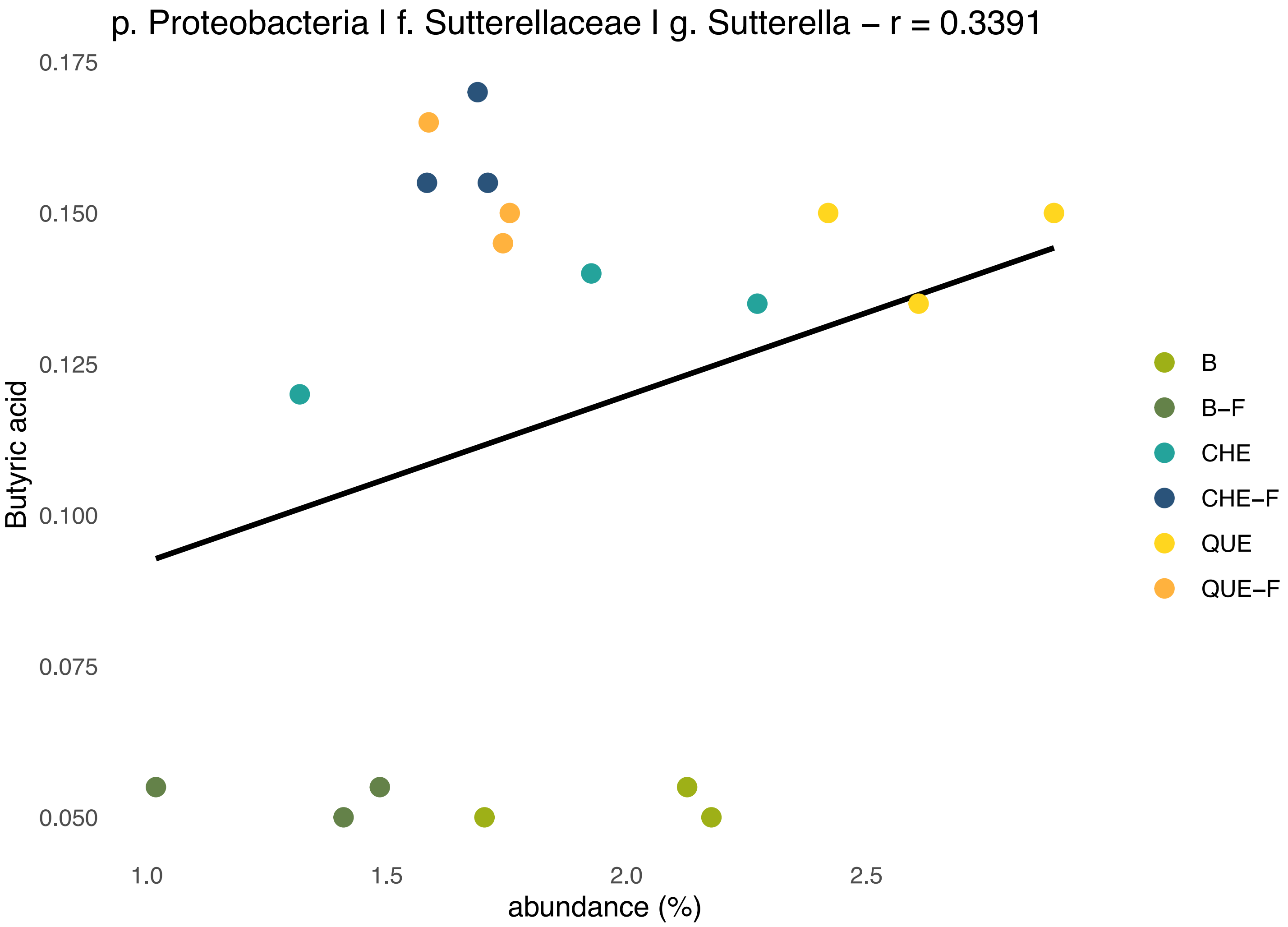
p. Proteobacteria I f. Sutterellaceae I g. Sutterella – r = 0.3391
0.175
0.150
0.125
0.100
0.075
0.050
Butyric acid
1.0
1.5
2.0
2.5
abundance (%)
B
B–F
CHE
CHE–F
QUE
QUE–F

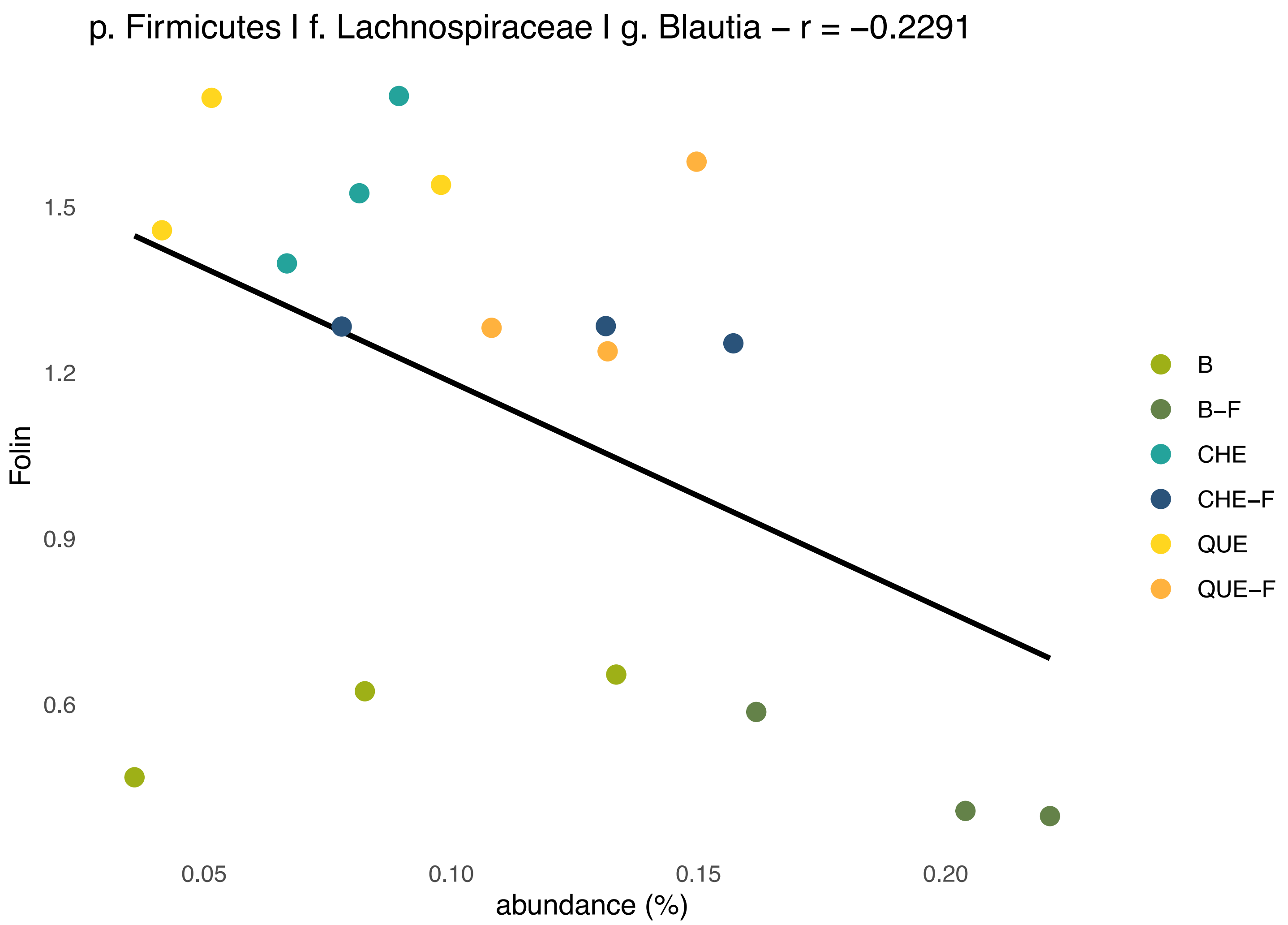

p. Firmicutes I f. Lachnospiraceae I g. Blautia – r = −0.2291
Folin
1.5
1.2
0.9
0.6
0.05
0.10
0.15
0.20
abundance (%)
B
B−F
CHE
CHE−F
QUE
QUE−F

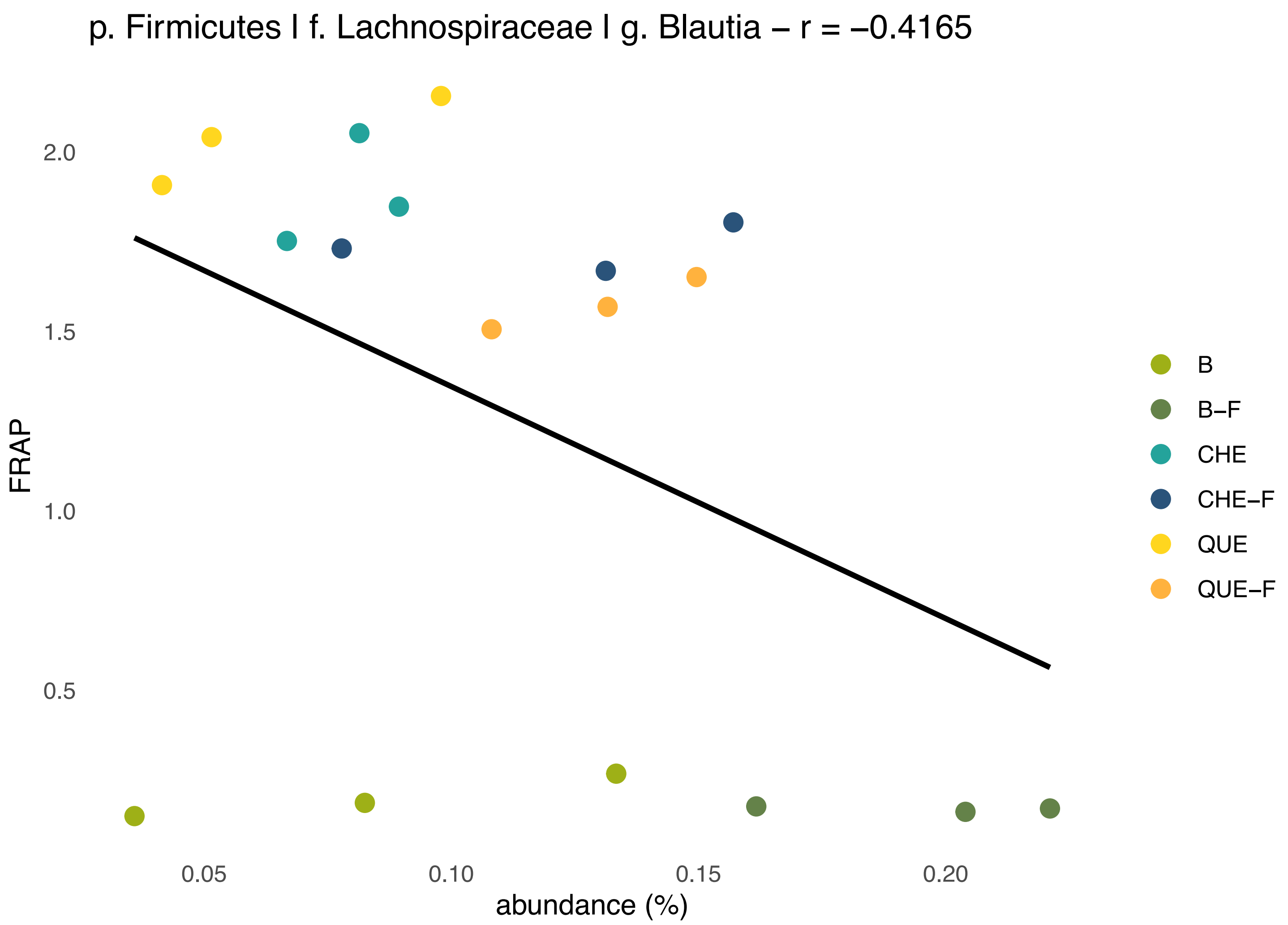

p. Firmicutes I f. Lachnospiraceae I g. Blautia – r = –0.4165
FRAP
2.0
1.5
1.0
0.5
0.05
0.10
0.15
0.20
abundance (%)
B
B–F
CHE
CHE–F
QUE
QUE–F

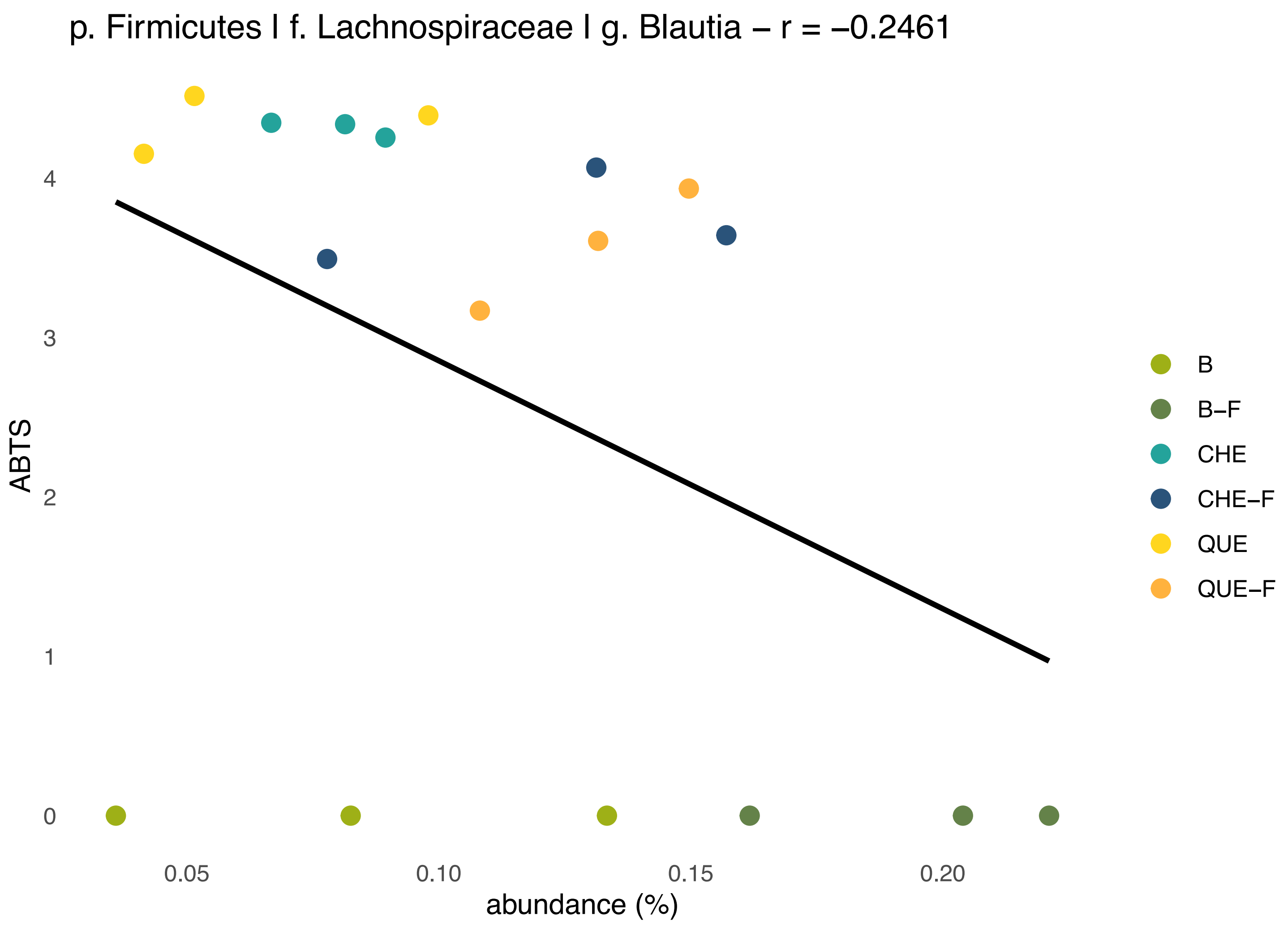

p. Firmicutes | f. Lachnospiraceae | g. Blautia – r = –0.2461
ABTS
0
1
2
3
4
0.05
0.10
0.15
0.20
abundance (%)
B
B–F
CHE
CHE–F
QUE
QUE–F

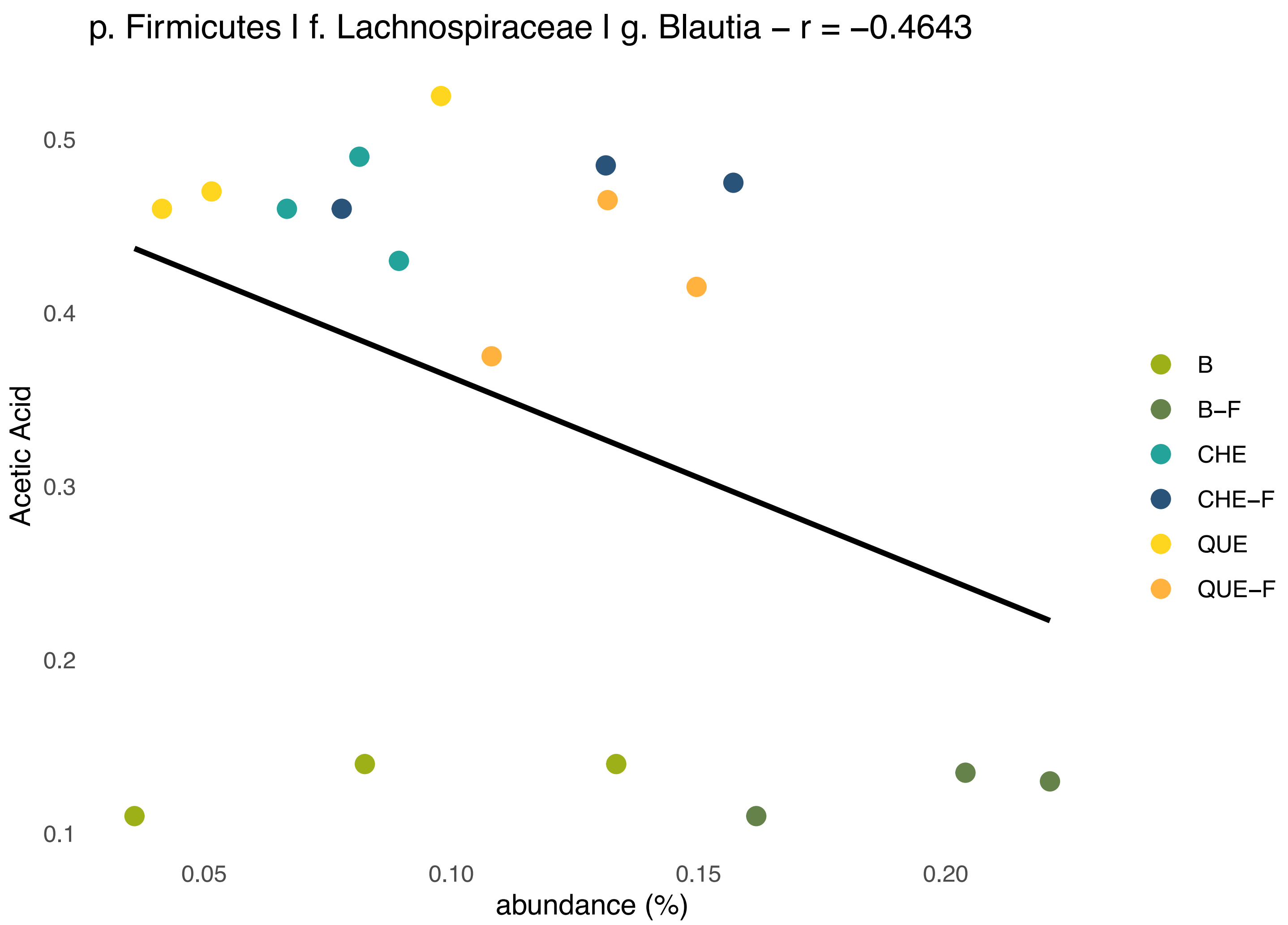

p. Firmicutes I f. Lachnospiraceae I g. Blautia – r = –0.4643
0.5
0.4
0.3
0.2
0.1
Acetic Acid
0.05
0.10
0.15
0.20
abundance (%)
B
B–F
CHE
CHE–F
QUE
QUE–F

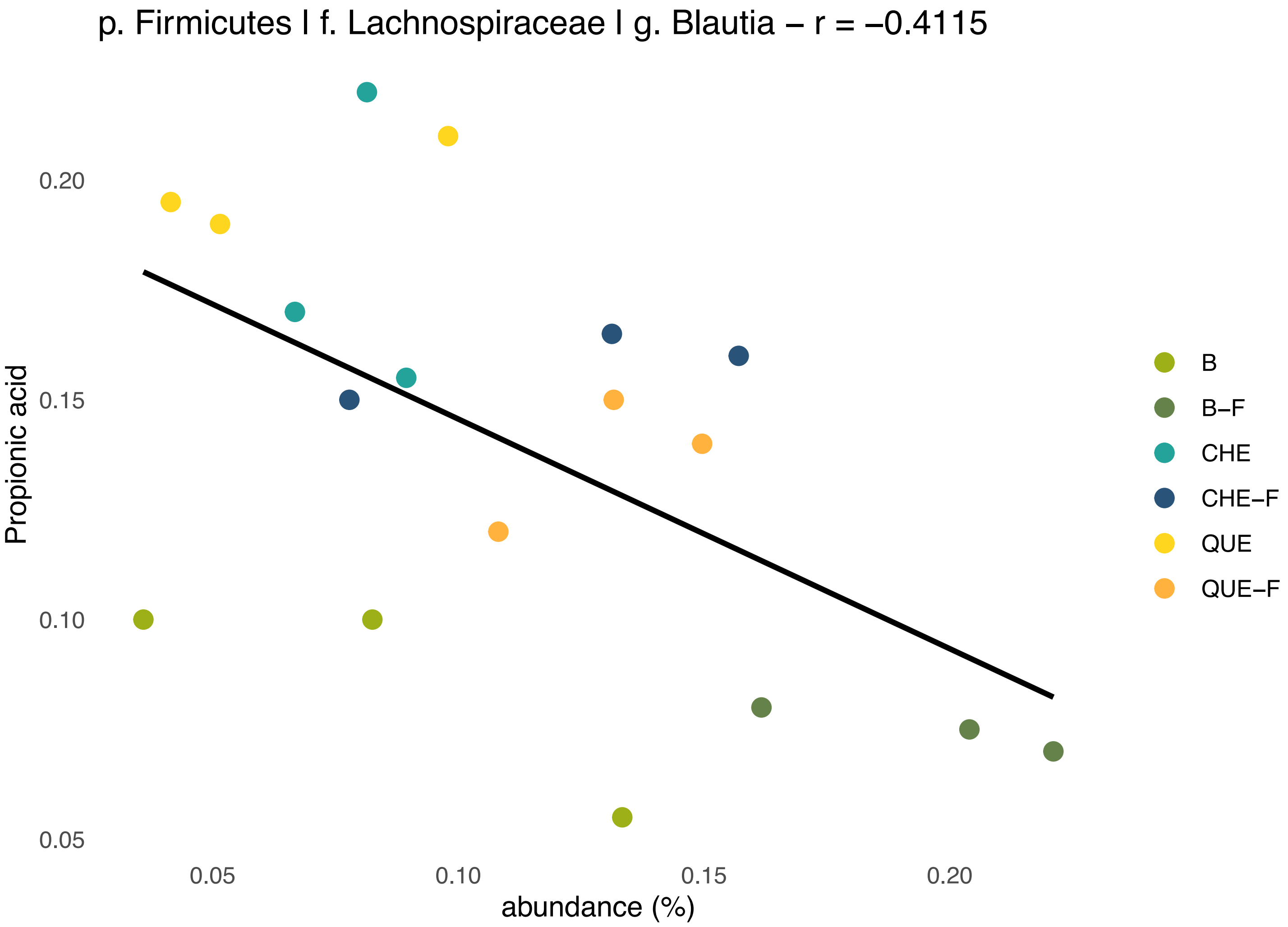

p. Firmicutes I f. Lachnospiraceae I g. Blautia – r = –0.4115
Propionic acid
0.20
0.15
0.10
0.05
0.05
0.10
0.15
0.20
abundance (%)
B
B–F
CHE
CHE–F
QUE
QUE–F

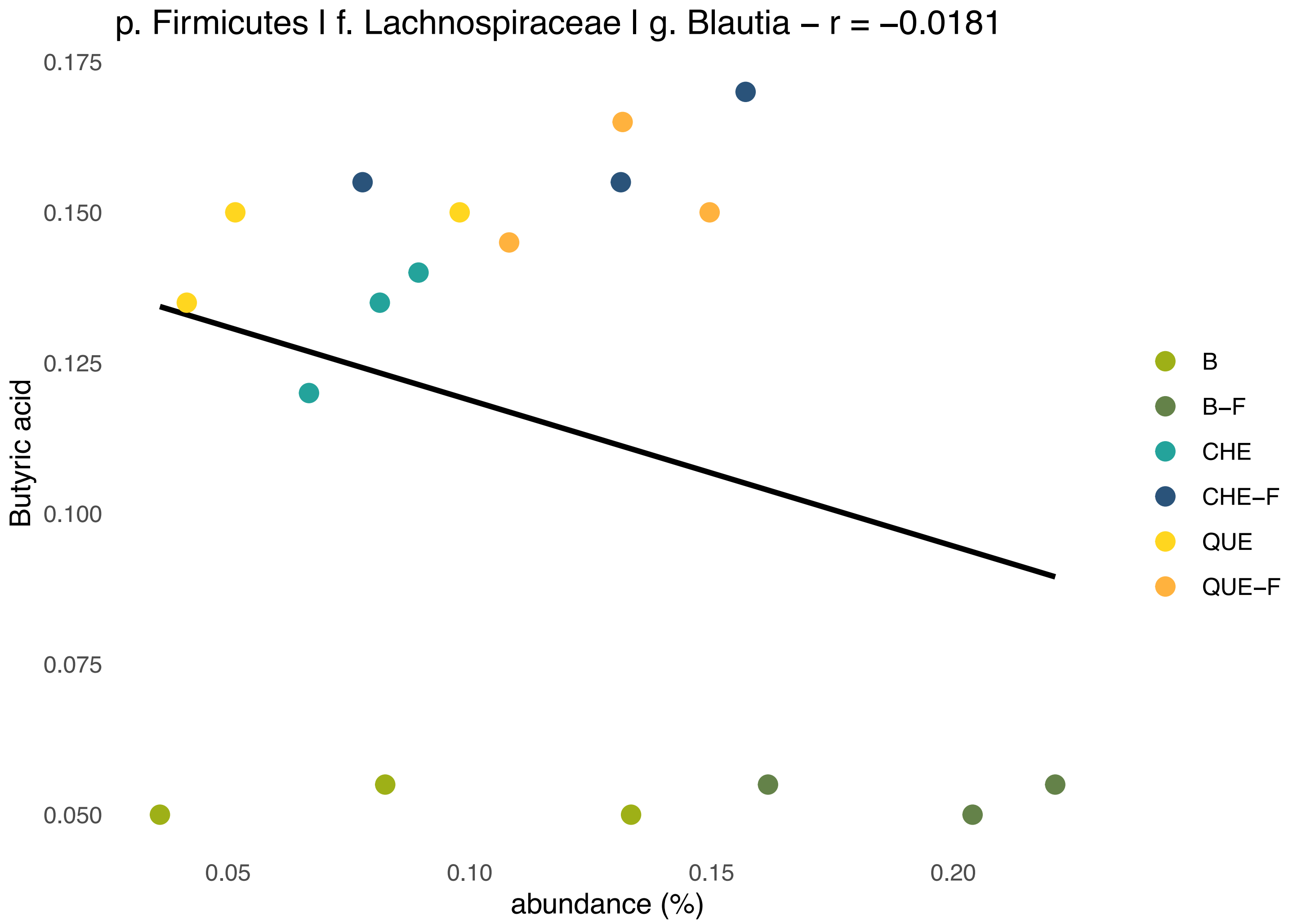

p. Firmicutes | f. Lachnospiraceae | g. Blautia – r = –0.0181
Butyric acid
0.175
0.150
0.125
0.100
0.075
0.050
0.05
0.10
0.15
0.20
abundance (%)
B
B–F
CHE
CHE–F
QUE
QUE–F

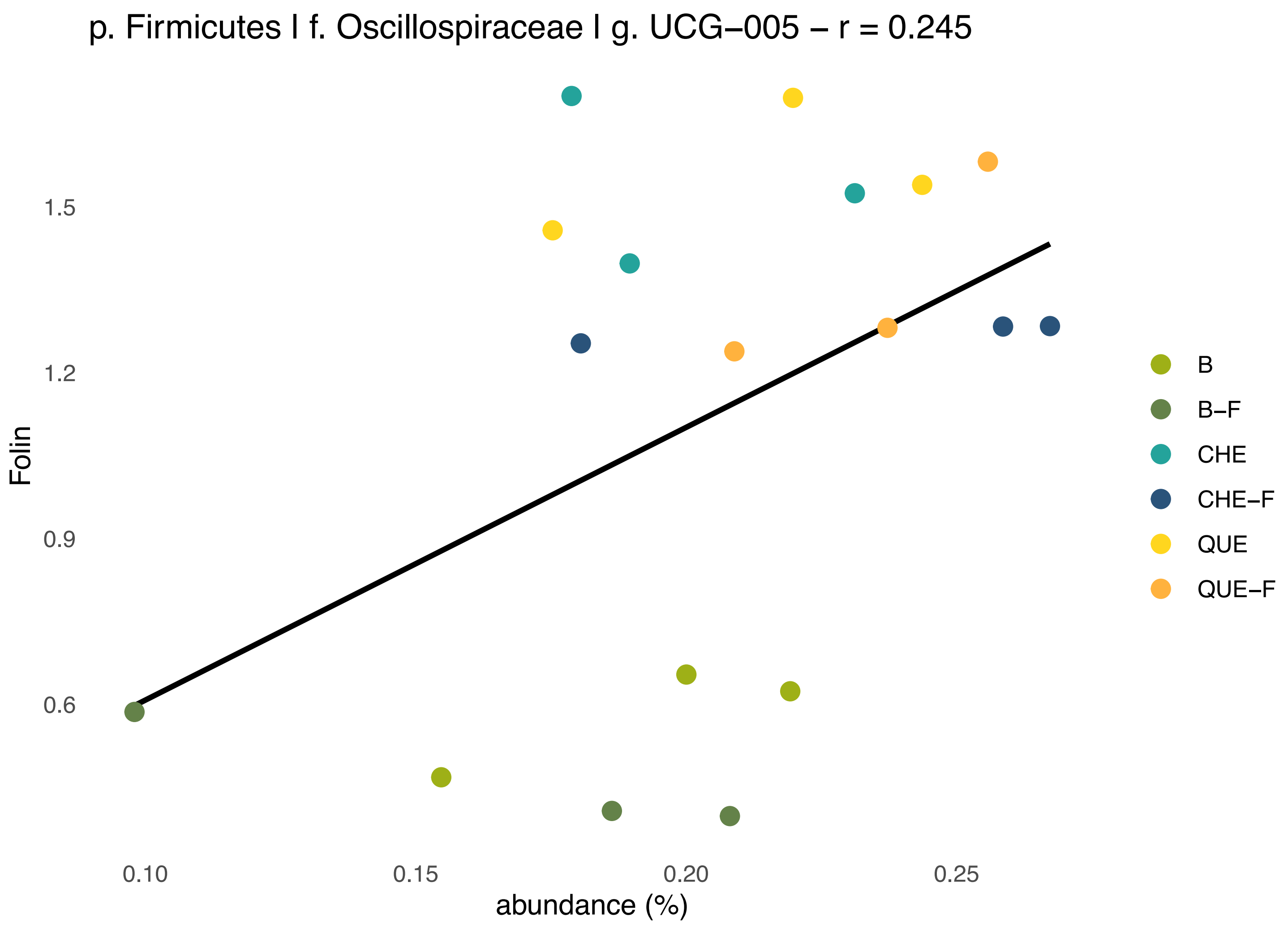

p. Firmicutes | f. Oscillospiraceae | g. UCG−005 − r = 0.245
Folin
1.5
1.2
0.9
0.6
0.10
0.15
0.20
0.25
abundance (%)
B
B−F
CHE
CHE−F
QUE
QUE−F

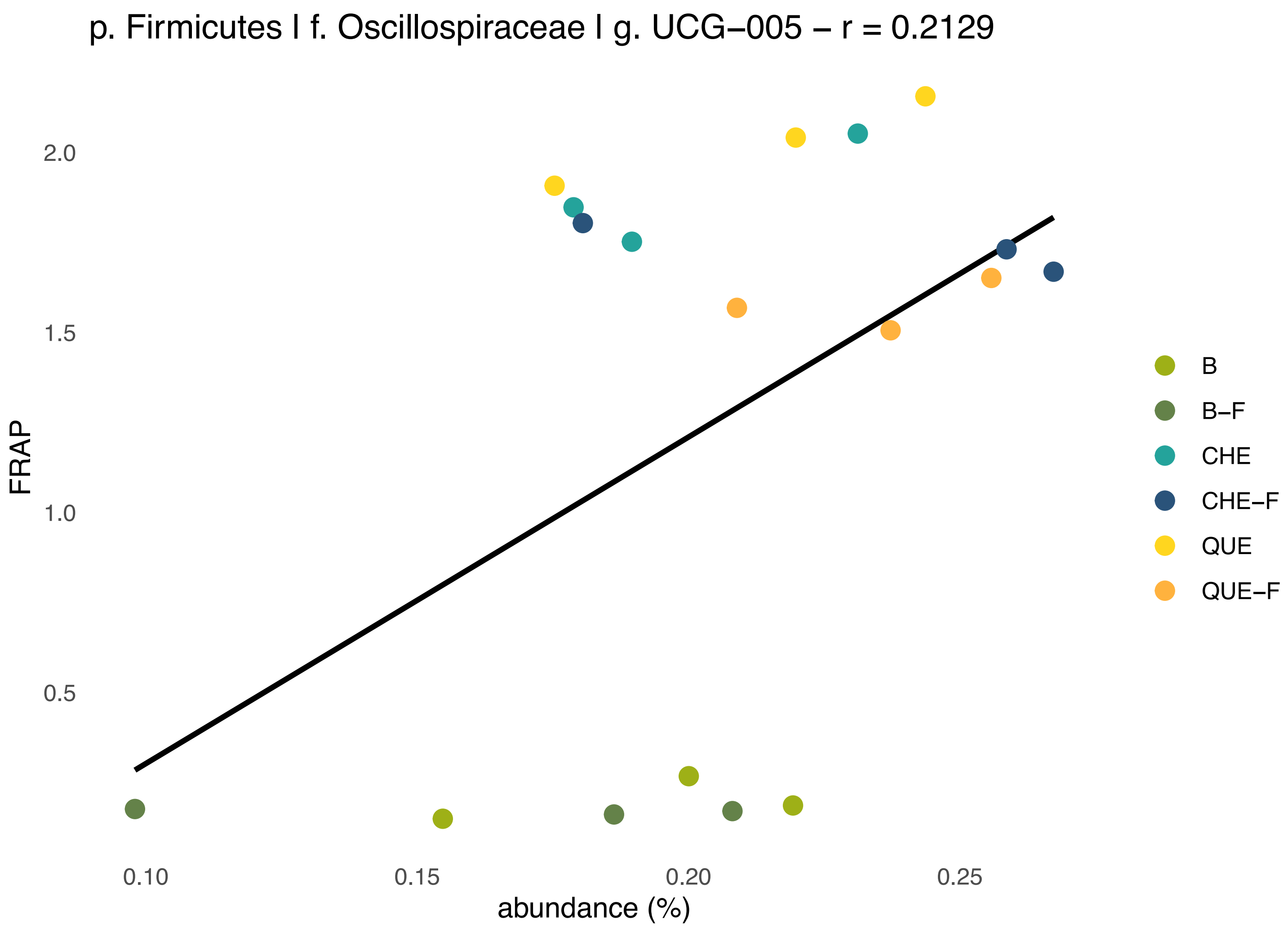

p. Firmicutes I f. Oscillospiraceae I g. UCG−005 − r = 0.2129
FRAP
2.0
1.5
1.0
0.5
0.10
0.15
0.20
0.25
abundance (%)
B
B−F
CHE
CHE−F
QUE
QUE−F

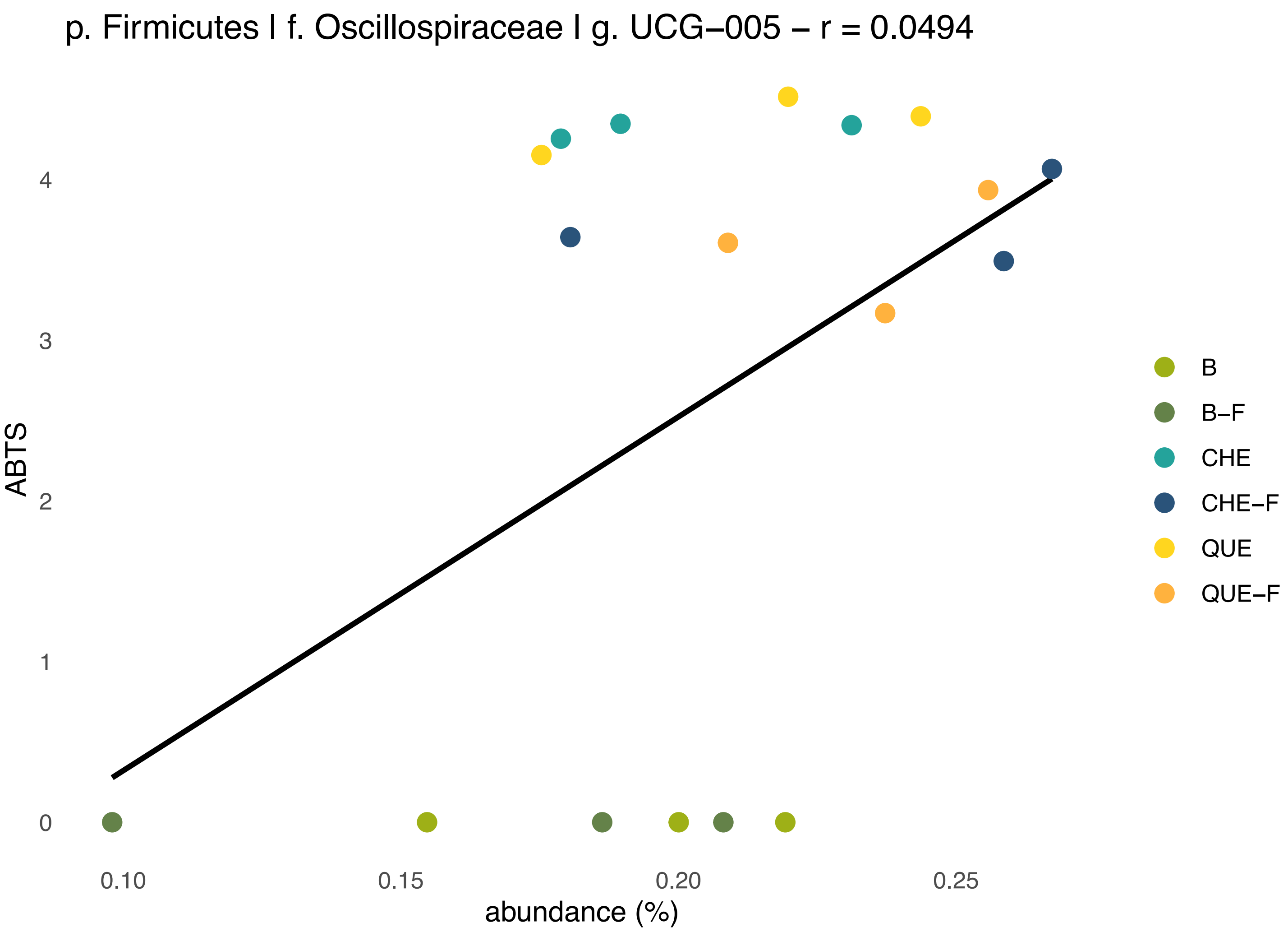

p. Firmicutes I f. Oscillospiraceae I g. UCG−005 − r = 0.0494
ABTS
4
3
2
1
0
0.10
0.15
0.20
0.25
abundance (%)
B
B−F
CHE
CHE−F
QUE
QUE−F

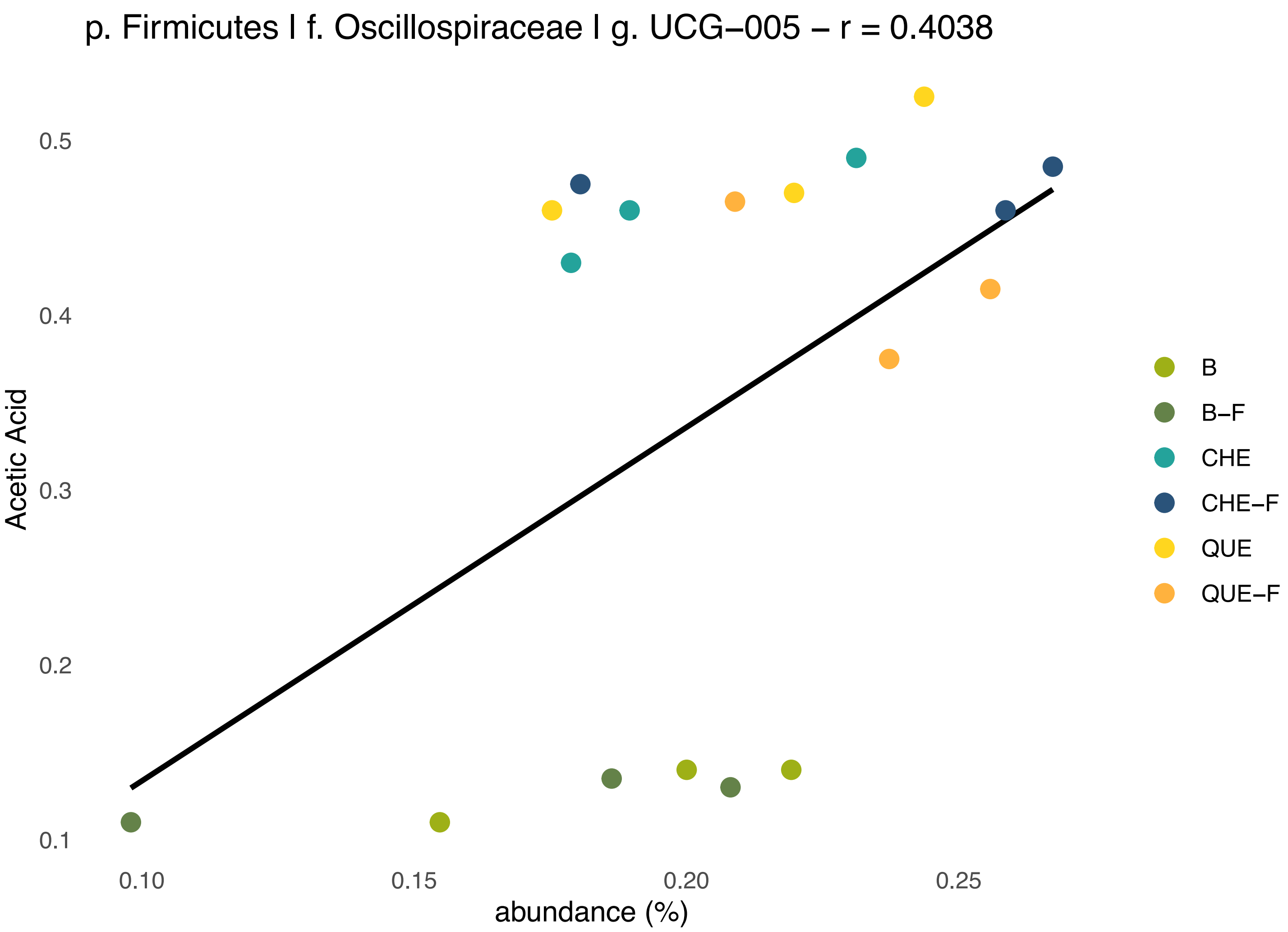
p. Firmicutes I f. Oscillospiraceae I g. UCG−005 − r = 0.4038
0.5
0.4
0.3
0.2
0.1
Acetic Acid
0.10
0.15
0.20
0.25
abundance (%)
B
B−F
CHE
CHE−F
QUE
QUE−F

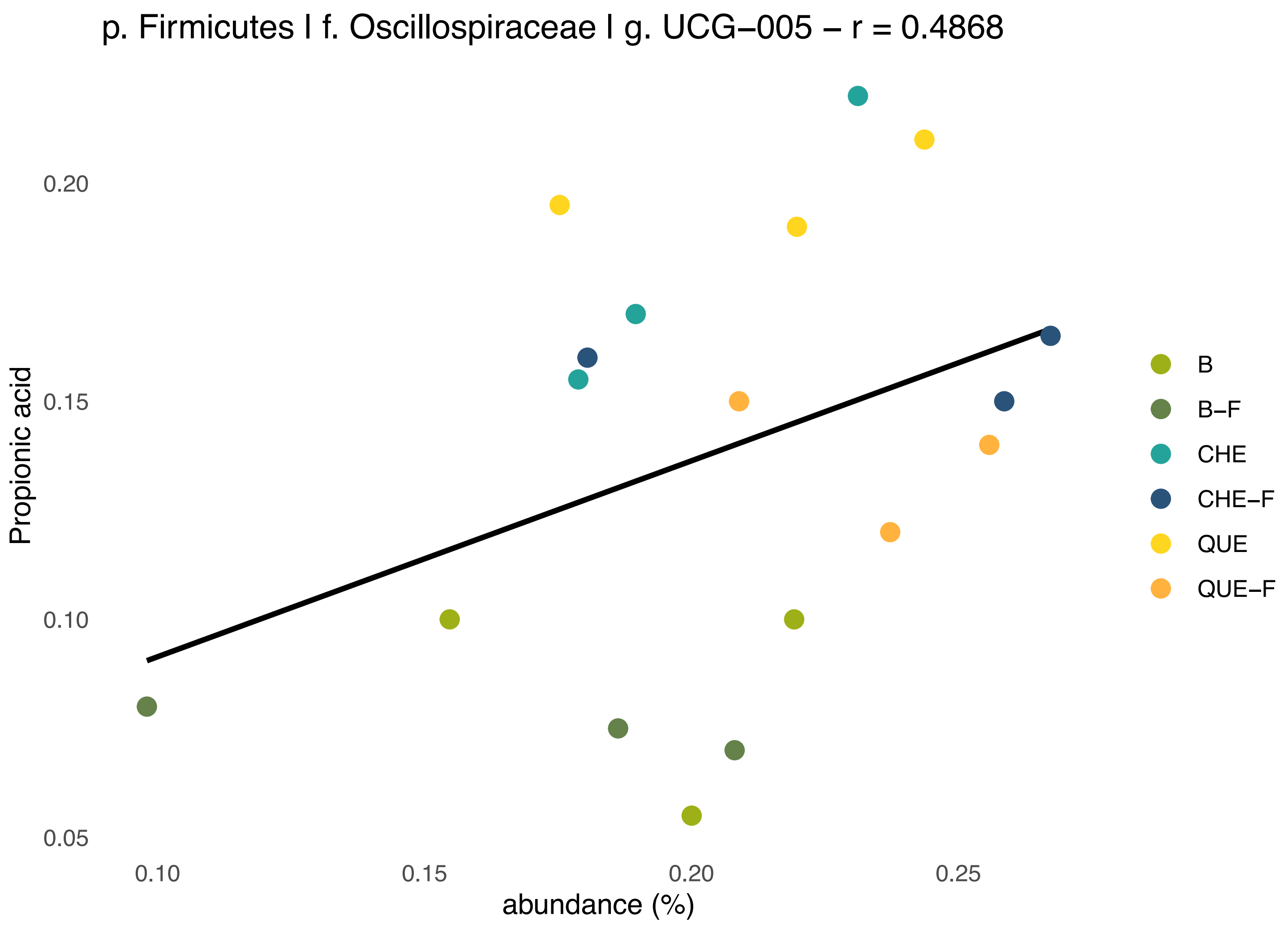

p. Firmicutes I f. Oscillospiraceae I g. UCG−005 − r = 0.4868
Propionic acid
0.20
0.15
0.10
0.05
0.10
0.15
0.20
0.25
abundance (%)
B
B−F
CHE
CHE−F
QUE
QUE−F

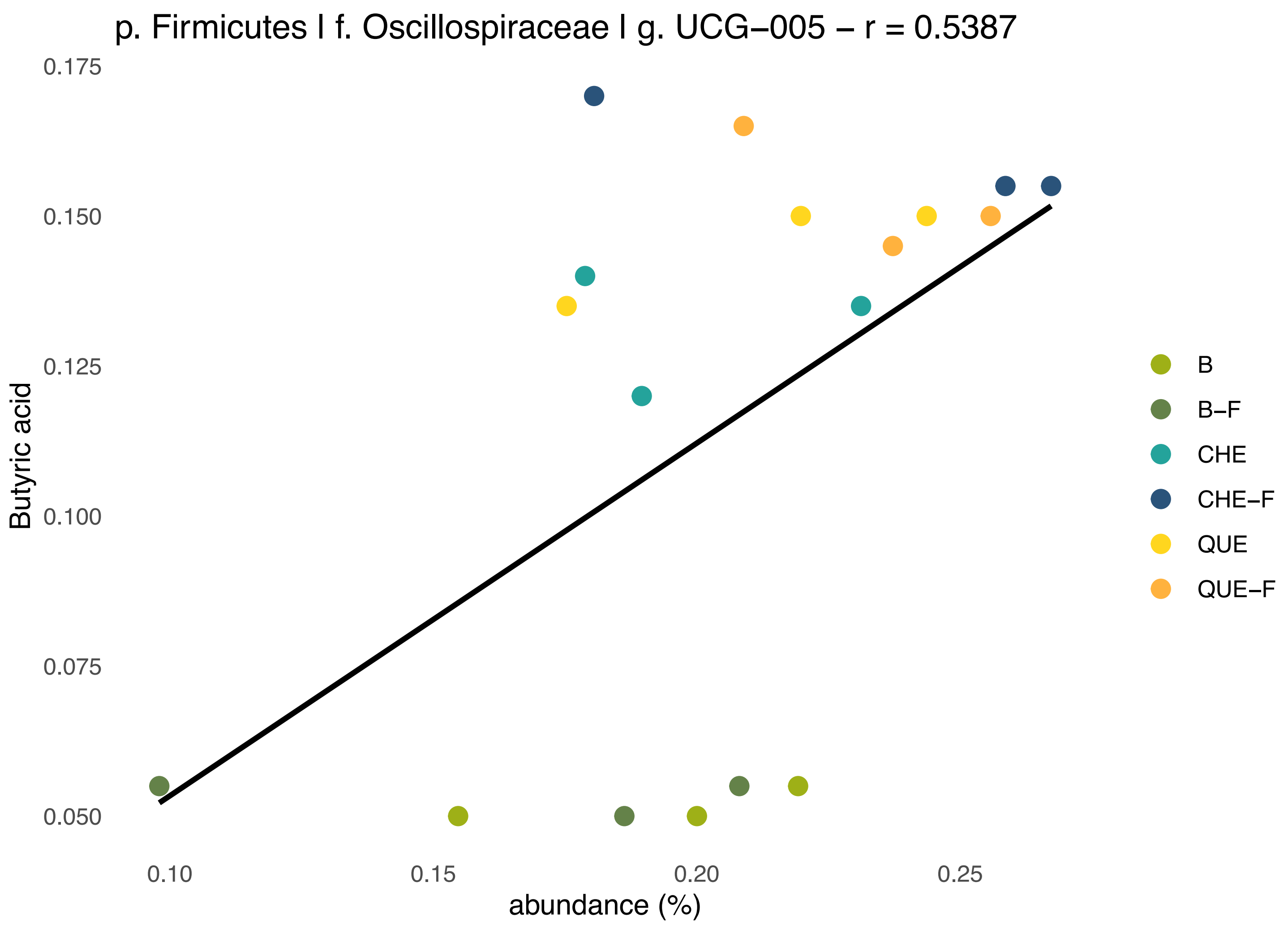
p. Firmicutes I f. Oscillospiraceae I g. UCG−005 − r = 0.5387
0.175
0.150
0.125
0.100
0.075
0.050
Butyric acid
0.10
0.15
0.20
0.25
abundance (%)
B
B−F
CHE
CHE−F
QUE
QUE−F

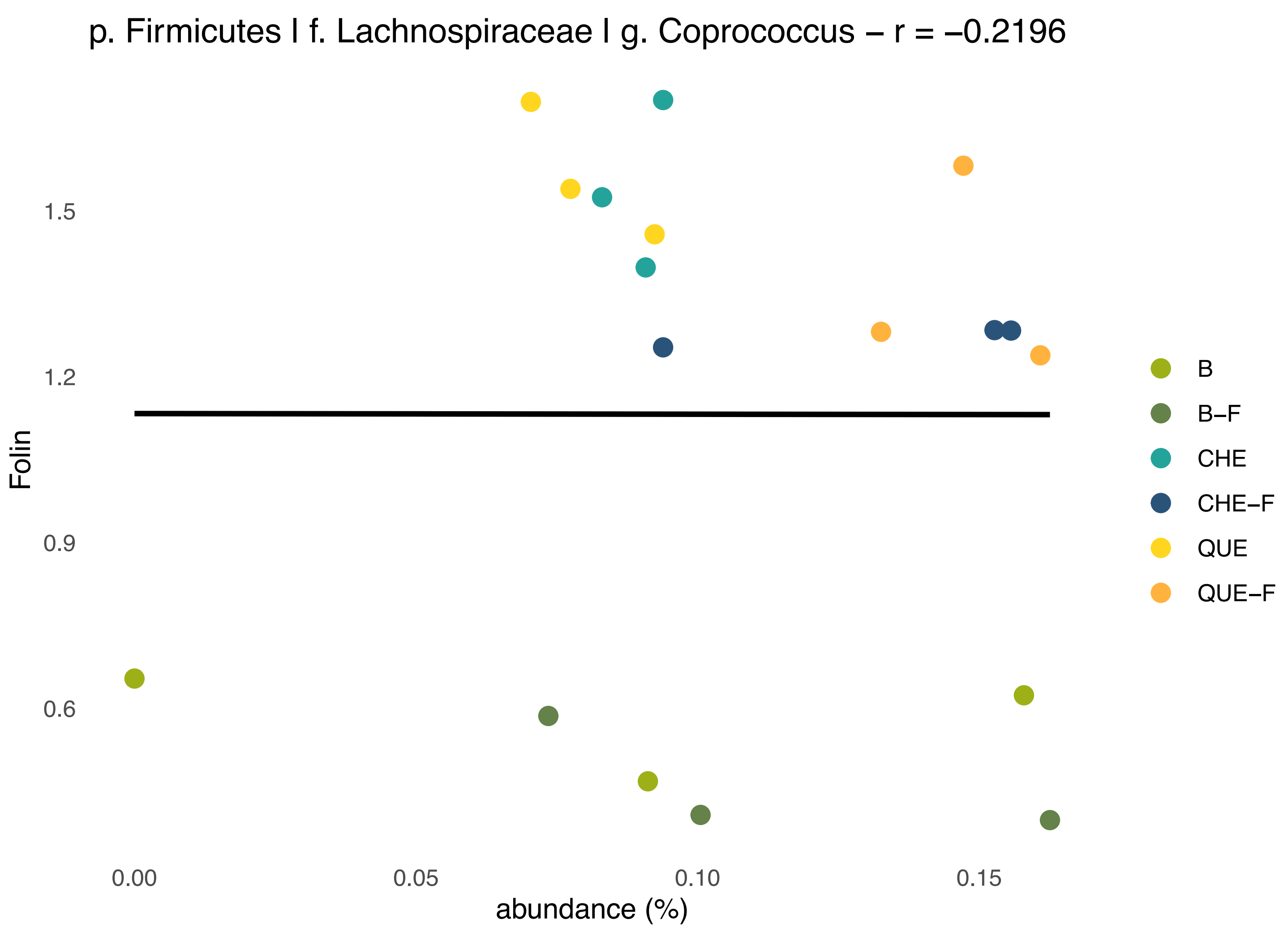

p. Firmicutes | f. Lachnospiraceae | g. Coprococcus – r = –0.2196
Folin
1.5
1.2
0.9
0.6
0.00
0.05
0.10
0.15
abundance (%)
B
B–F
CHE
CHE–F
QUE
QUE–F

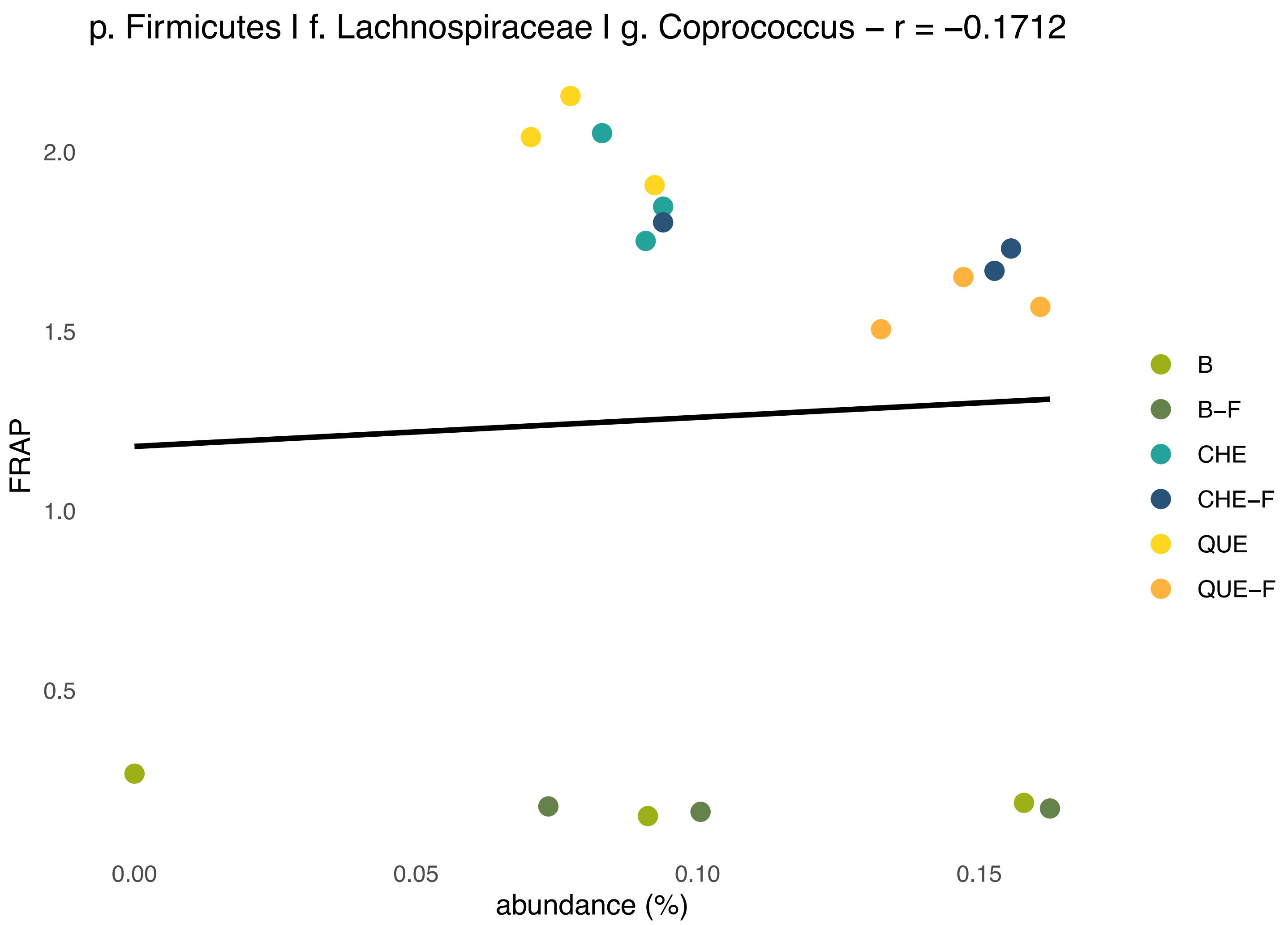

p. Firmicutes | f. Lachnospiraceae | g. Coprococcus – r = –0.1712
FRAP
2.0
1.5
1.0
0.5
0.00
0.05
0.10
0.15
abundance (%)
B
B–F
CHE
CHE–F
QUE
QUE–F

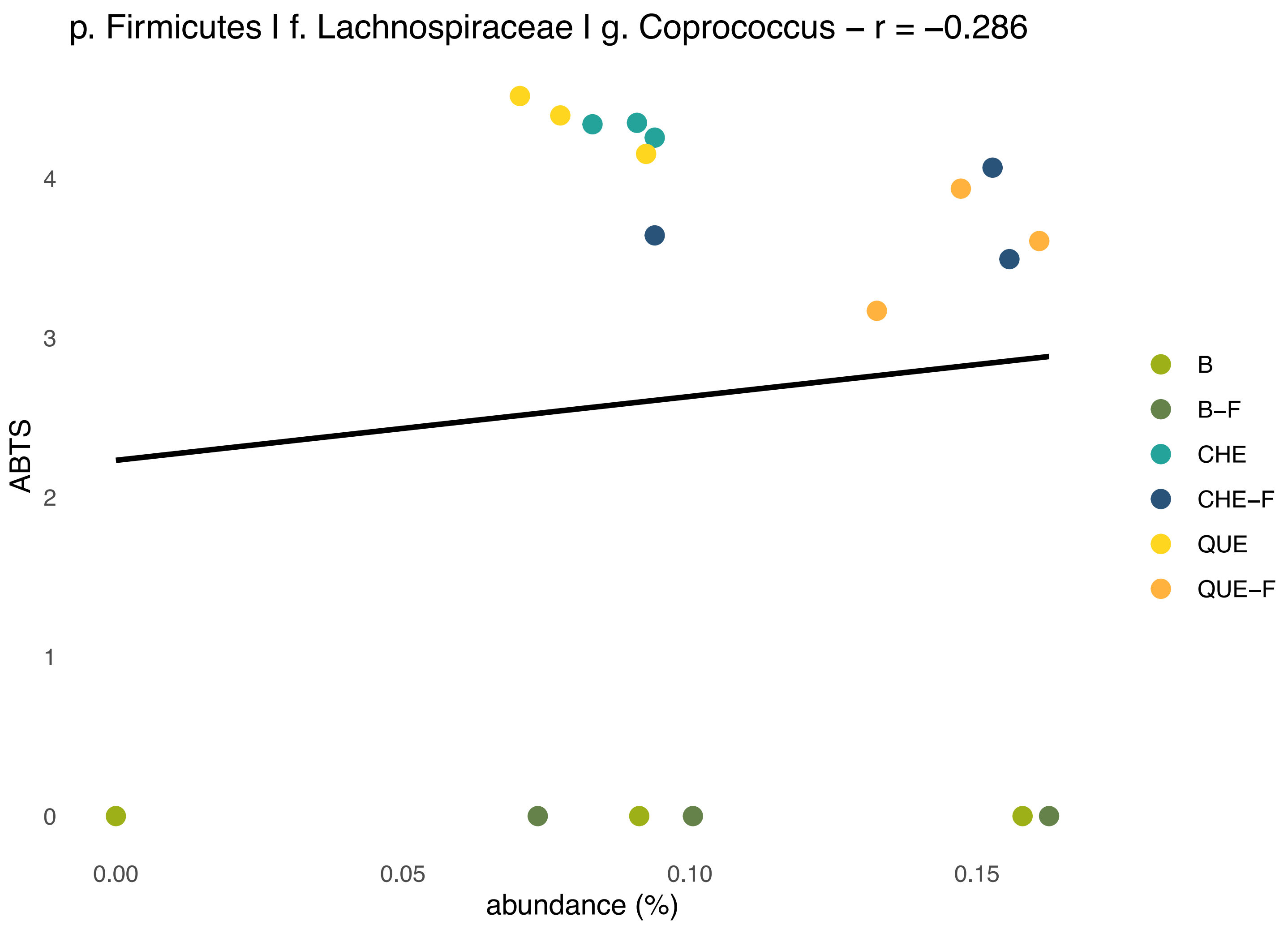

p. Firmicutes I f. Lachnospiraceae I g. Coprococcus – r = –0.286
ABTS
4
3
2
1
0
0.00
0.05
0.10
0.15
abundance (%)
B
B–F
CHE
CHE–F
QUE
QUE–F

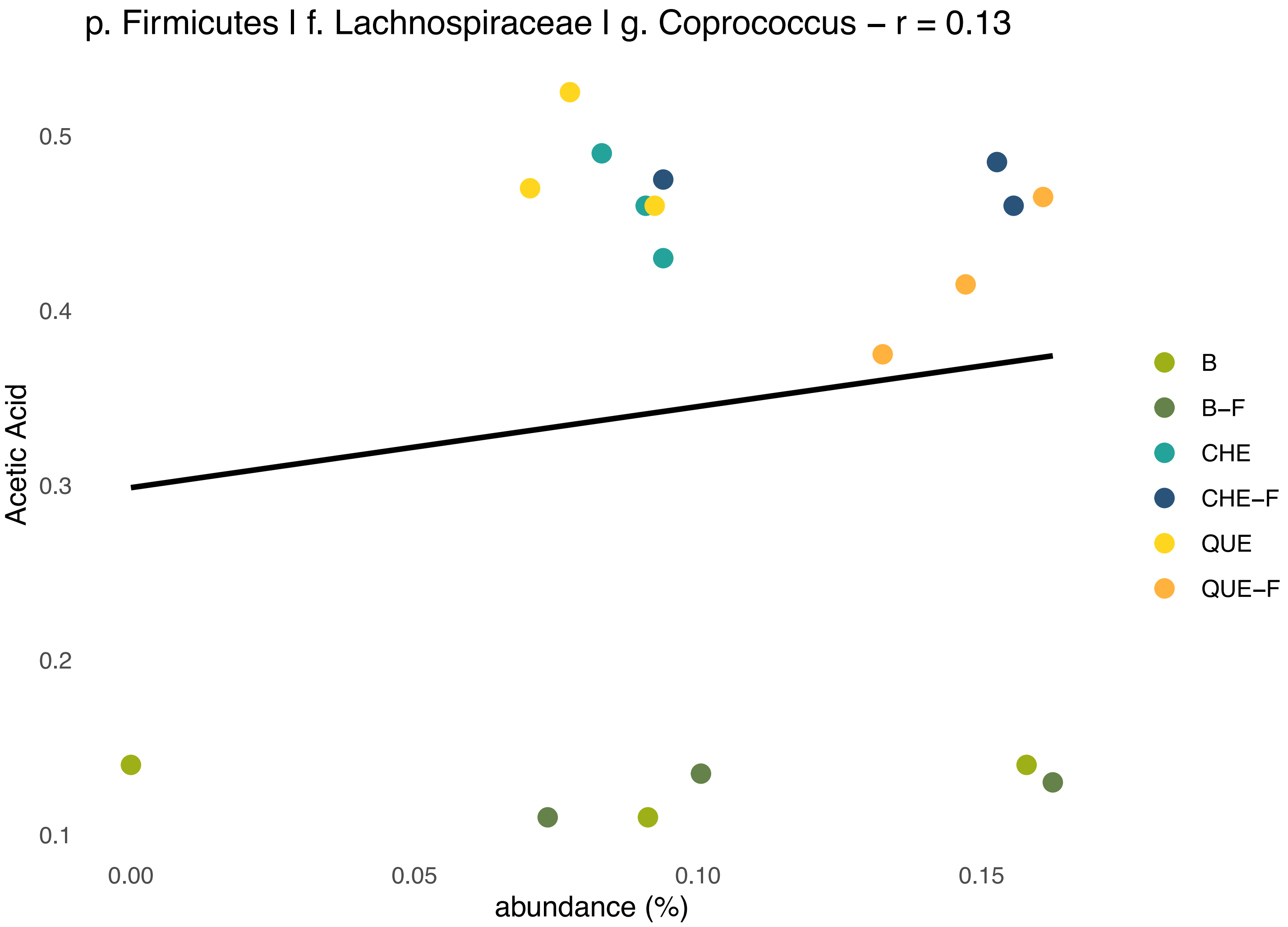

p. Firmicutes I f. Lachnospiraceae I g. Coprococcus – r = 0.13
Acetic Acid
0.5
0.4
0.3
0.2
0.1
0.00
0.05
0.10
0.15
abundance (%)
B
B−F
CHE
CHE−F
QUE
QUE−F

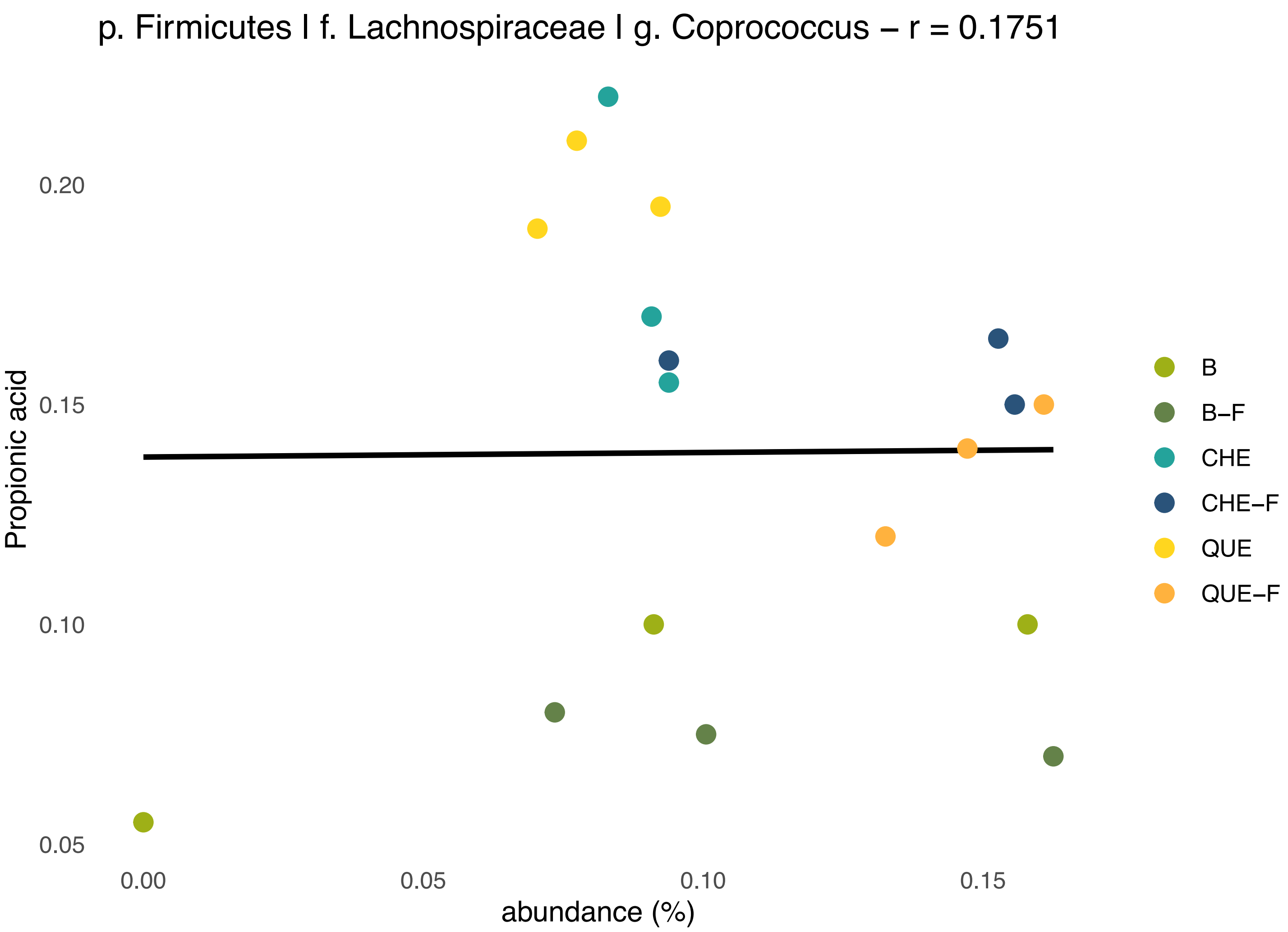
p. Firmicutes I f. Lachnospiraceae I g. Coprococcus – r = 0.1751
Propionic acid
0.20
0.15
0.10
0.05
0.00
0.05
0.10
0.15
abundance (%)
B
B–F
CHE
CHE–F
QUE
QUE–F

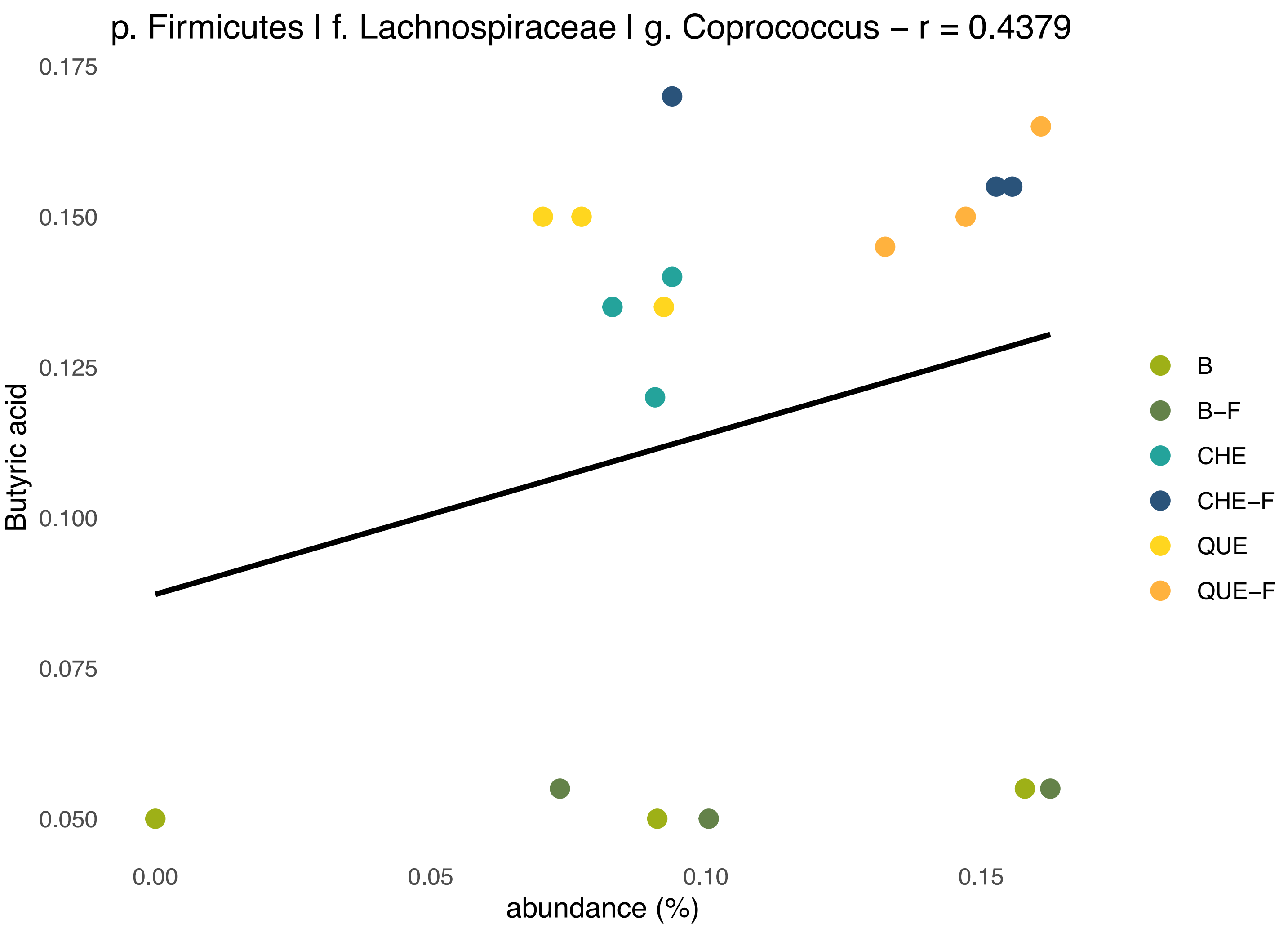
p. Firmicutes | f. Lachnospiraceae | g. Coprococcus – r = 0.4379
Butyric acid
0.175
0.150
0.125
0.100
0.075
0.050
0.00
0.05
0.10
0.15
abundance (%)
B
B–F
CHE
CHE–F
QUE
QUE–F

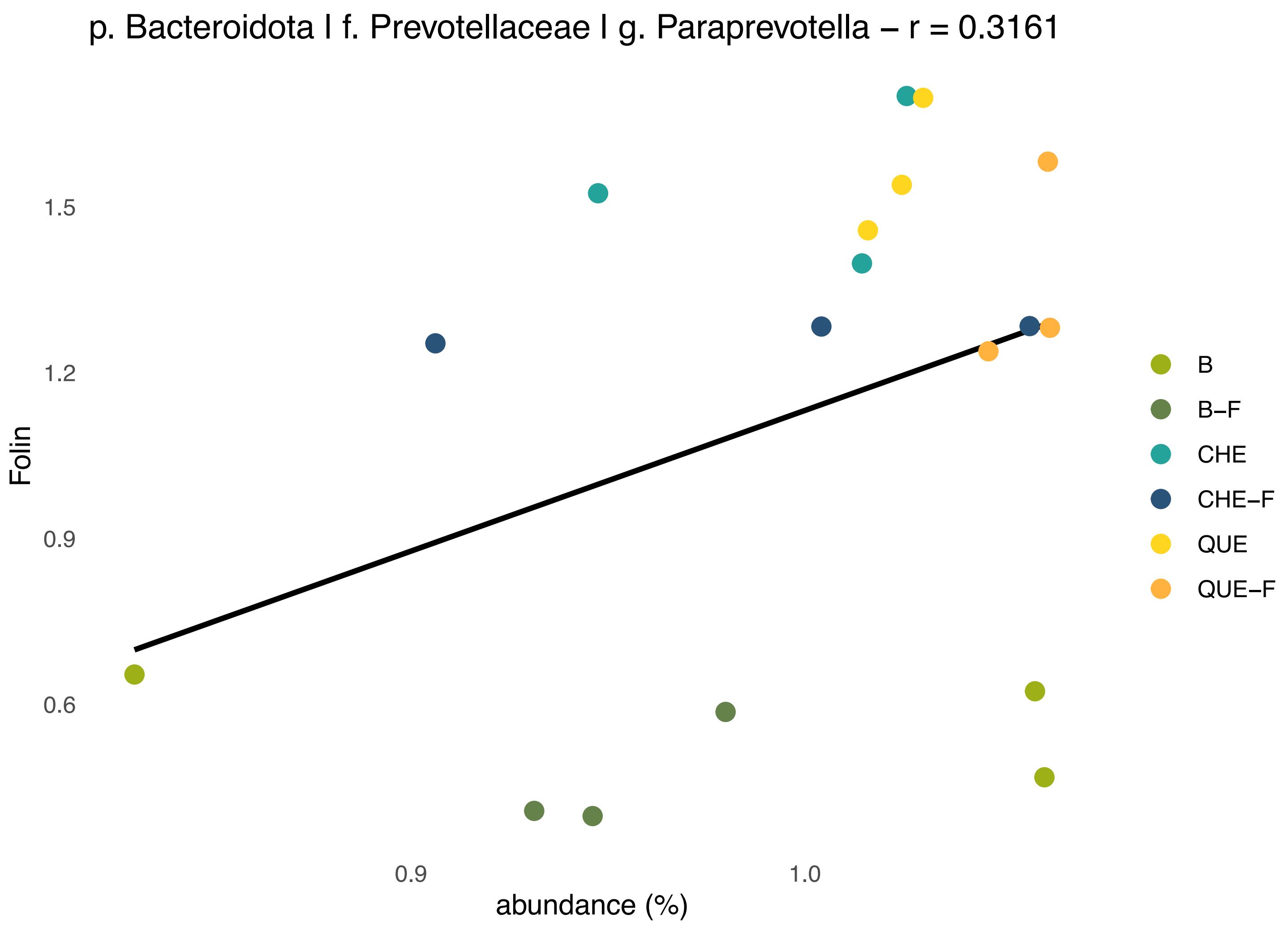
p. Bacteroidota I f. Prevotellaceae I g. Paraprevotella – r = 0.3161
Folin
1.5
1.2
0.9
0.6
0.9
1.0
abundance (%)
B
B–F
CHE
CHE–F
QUE
QUE–F

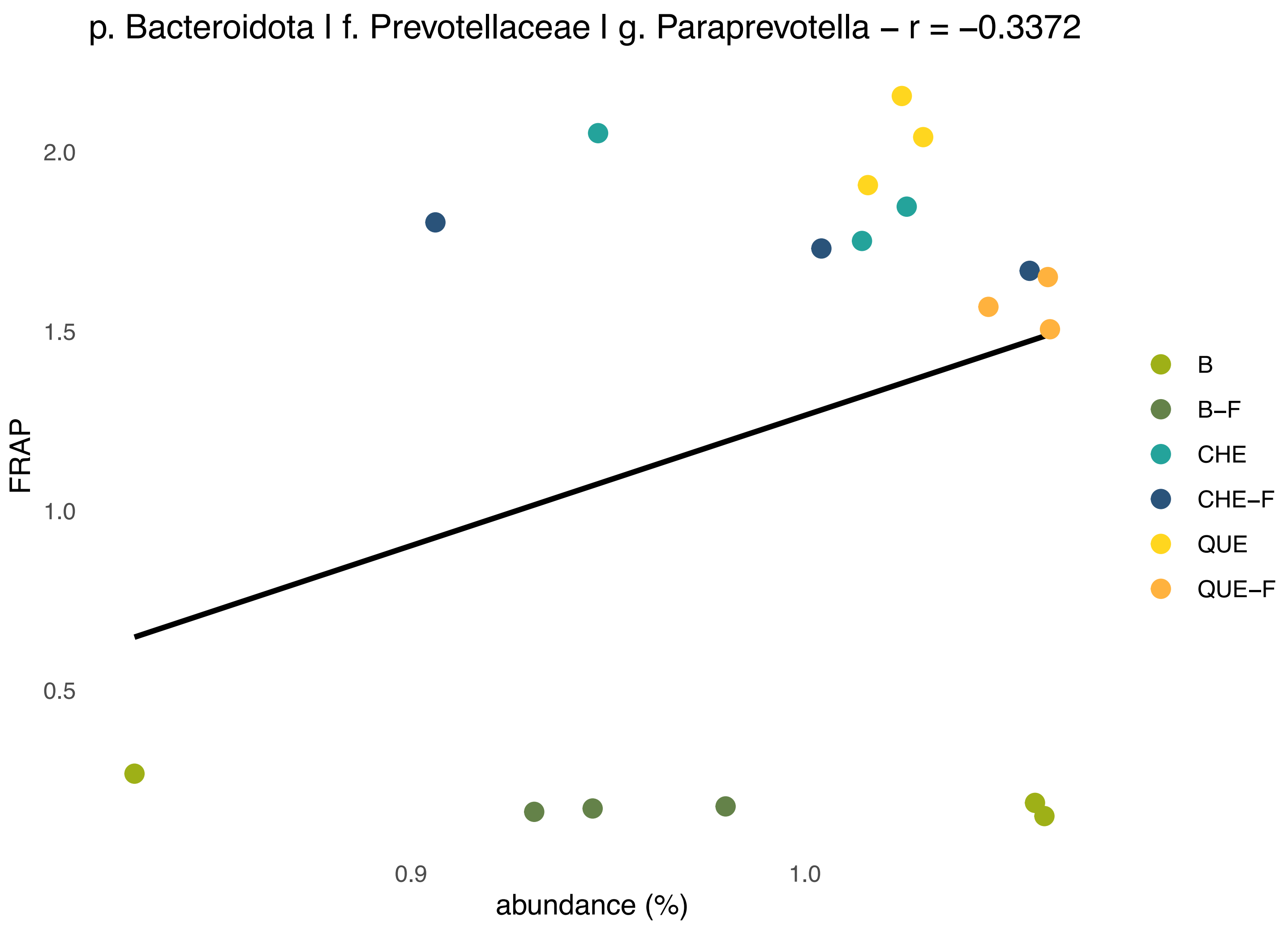
p. Bacteroidota | f. Prevotellaceae | g. Paraprevotella – r = –0.3372
FRAP
2.0
1.5
1.0
0.5
0.9
1.0
abundance (%)
B
B–F
CHE
CHE–F
QUE
QUE–F

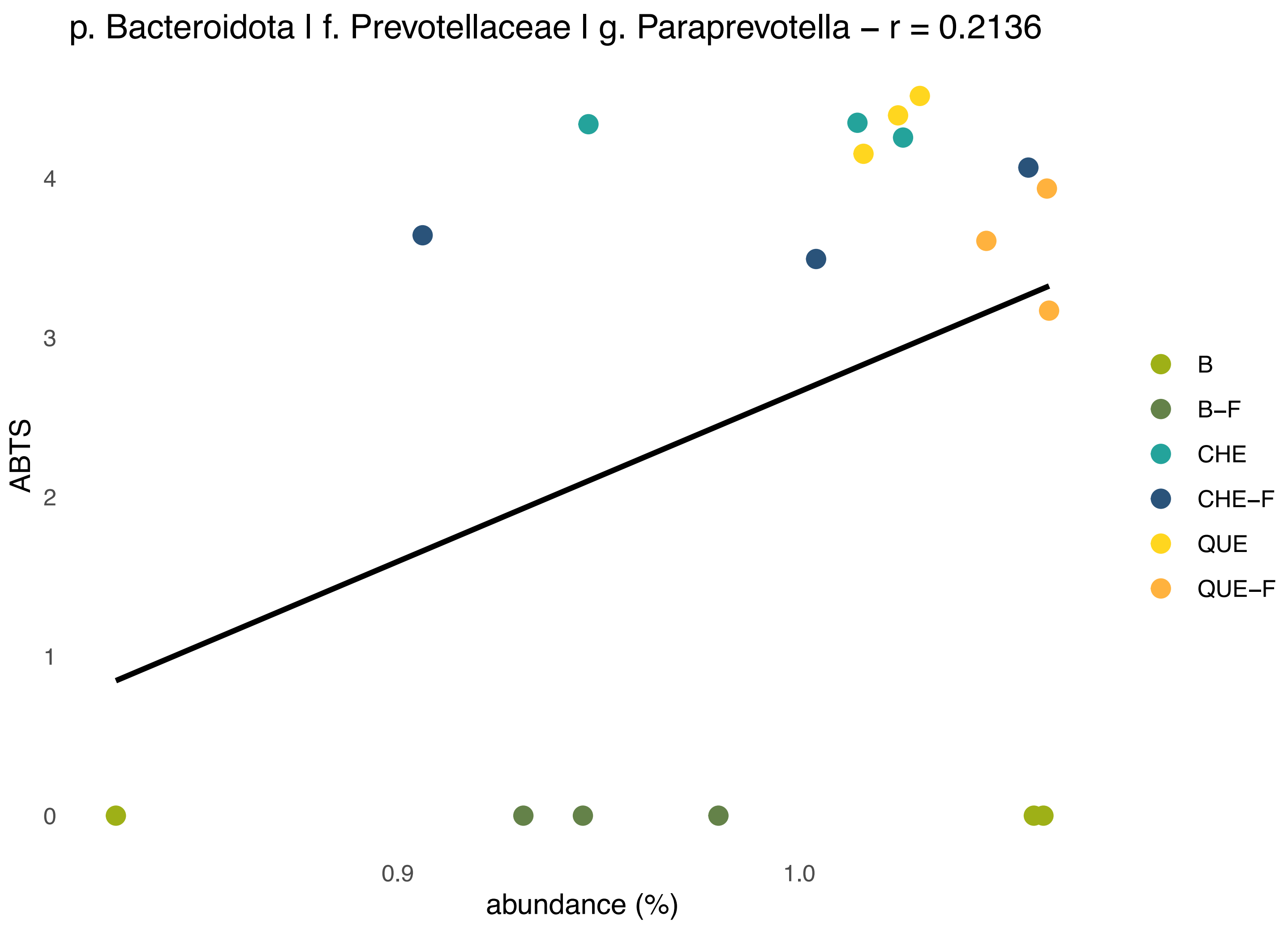
p. Bacteroidota I f. Prevotellaceae I g. Paraprevotella – r = 0.2136
ABTS
0
1
2
3
4
0.9
1.0
abundance (%)
B
B–F
CHE
CHE–F
QUE
QUE–F

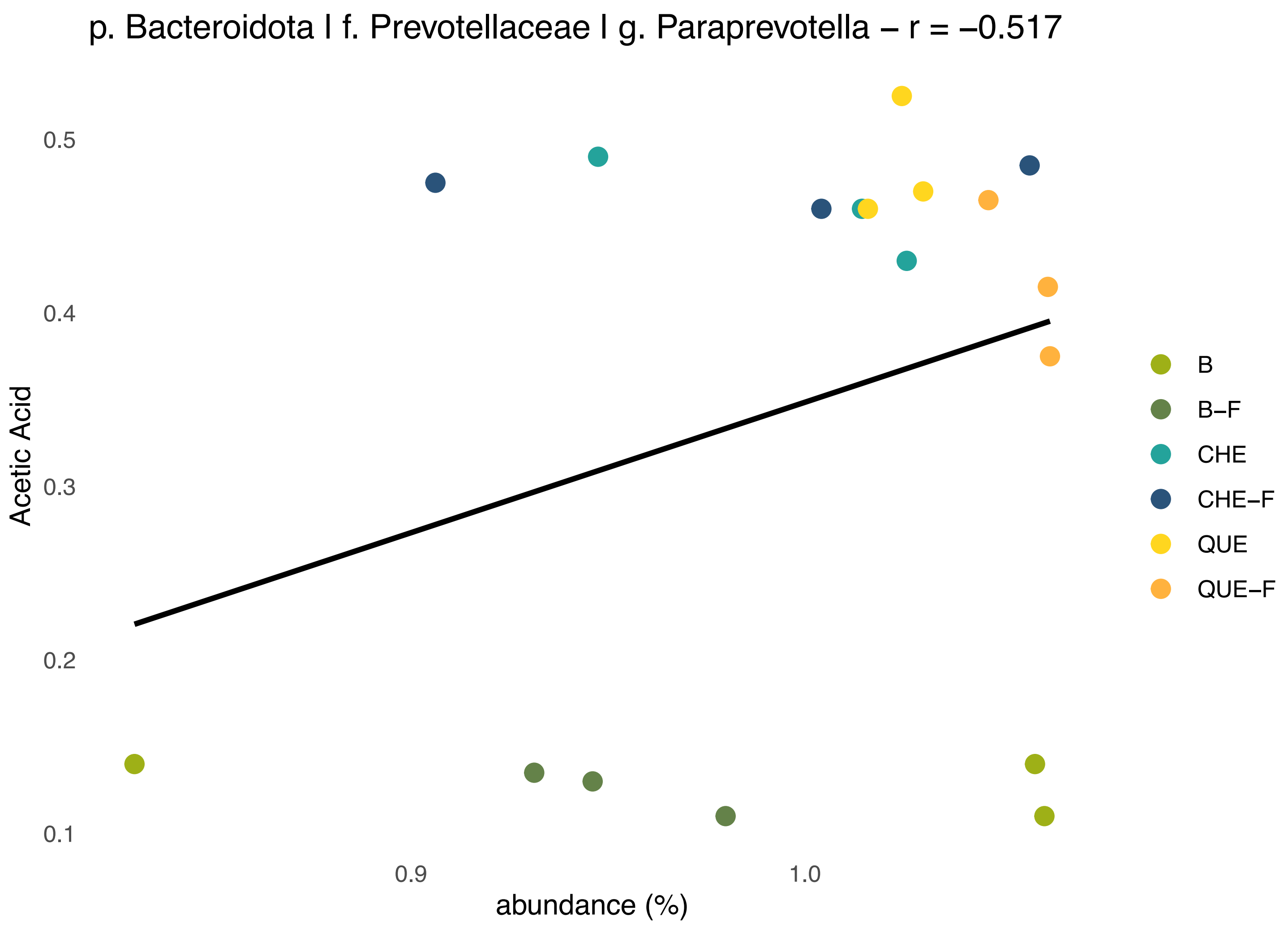

p. Bacteroidota I f. Prevotellaceae I g. Paraprevotella – r = –0.517
Acetic Acid
0.5
0.4
0.3
0.2
0.1
0.9
1.0
abundance (%)
B
B–F
CHE
CHE–F
QUE
QUE–F

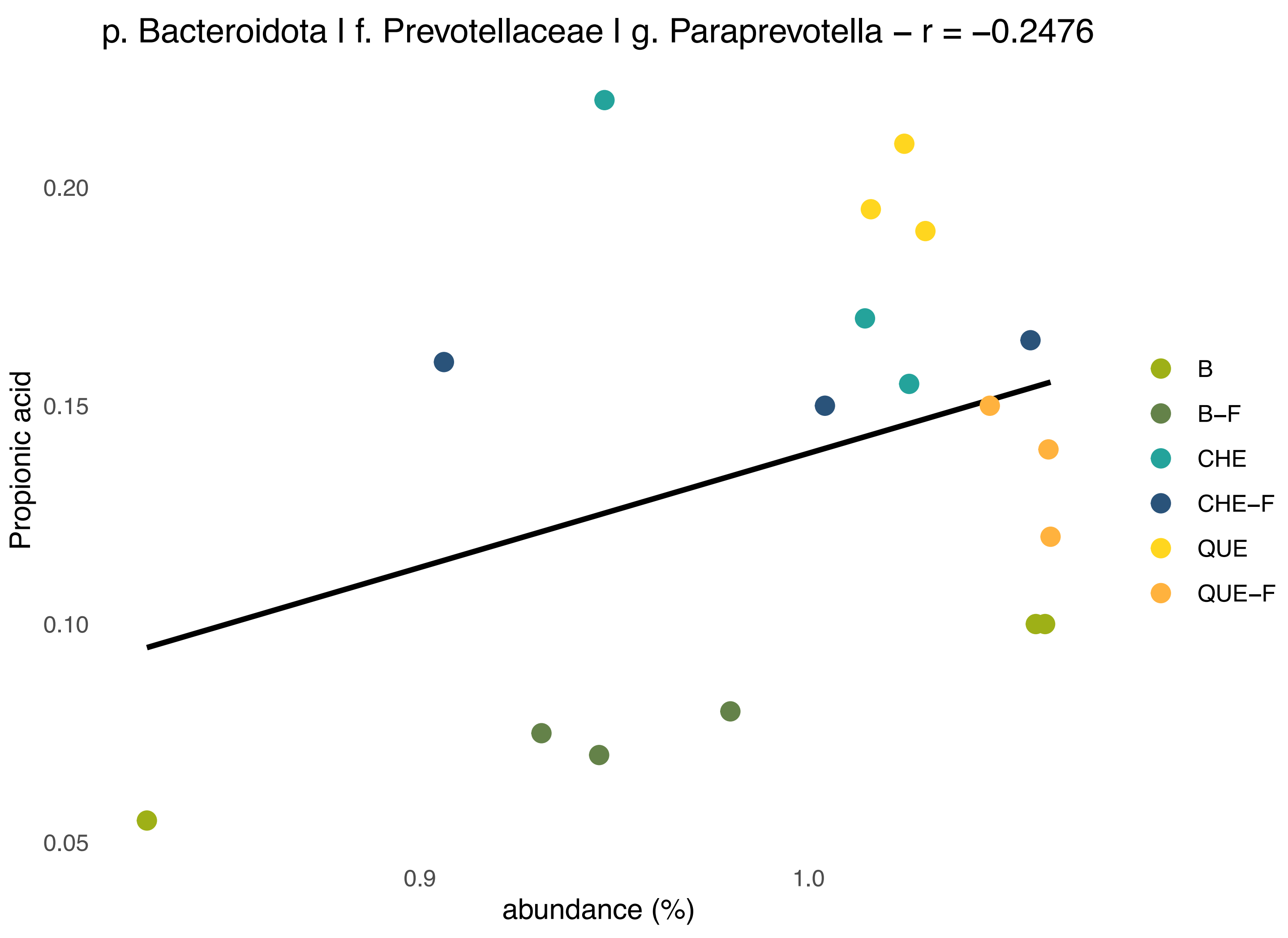

p. Bacteroidota | f. Prevotellaceae | g. Paraprevotella – r = −0.2476
Propionic acid
0.20
0.15
0.10
0.05
0.9
1.0
abundance (%)
B
B−F
CHE
CHE−F
QUE
QUE−F

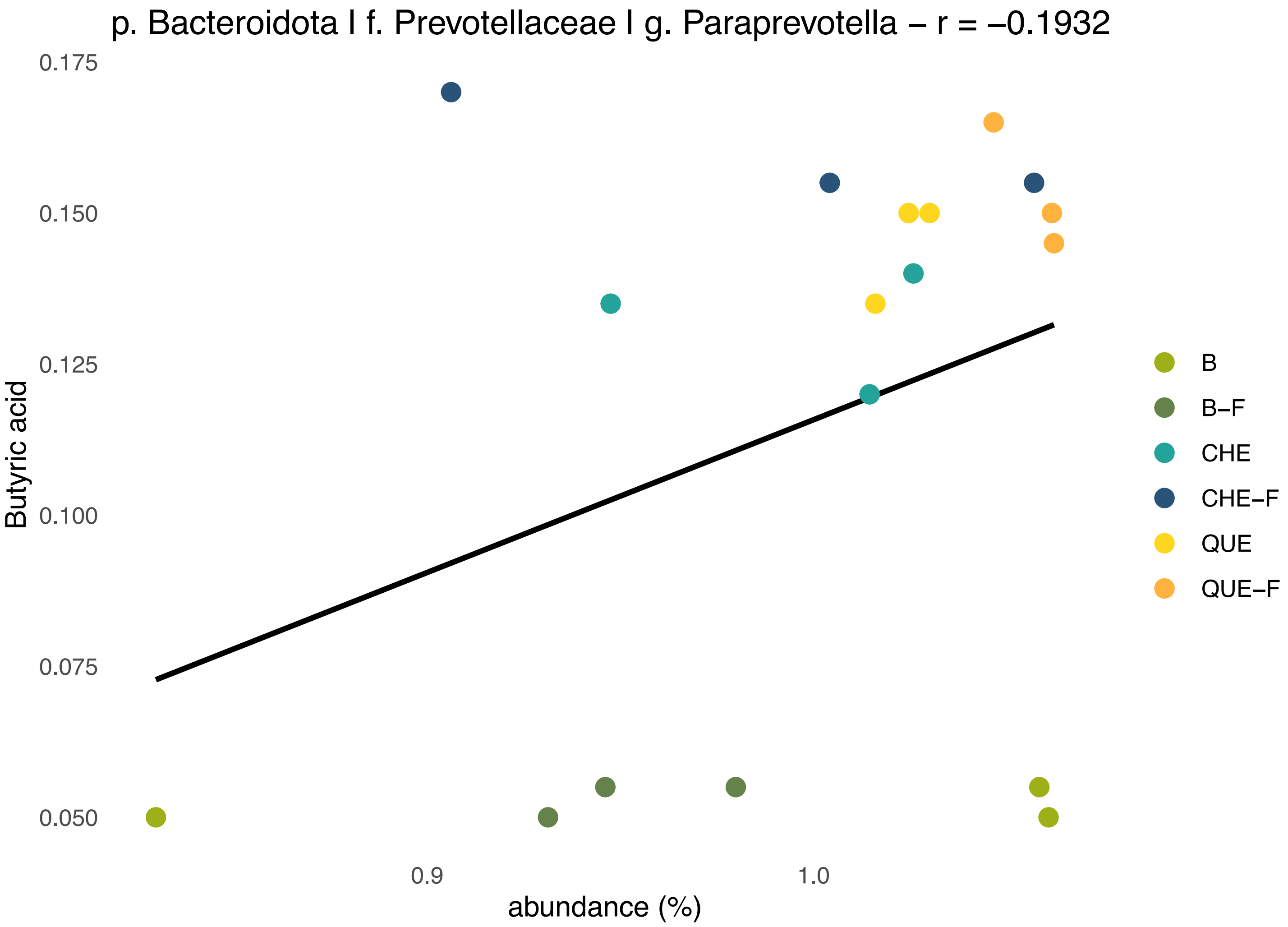
p. Bacteroidota | f. Prevotellaceae | g. Paraprevotella – r = −0.1932
Butyric acid
0.175
0.150
0.125
0.100
0.075
0.050
0.9
1.0
abundance (%)
B
B−F
CHE
CHE−F
QUE
QUE−F

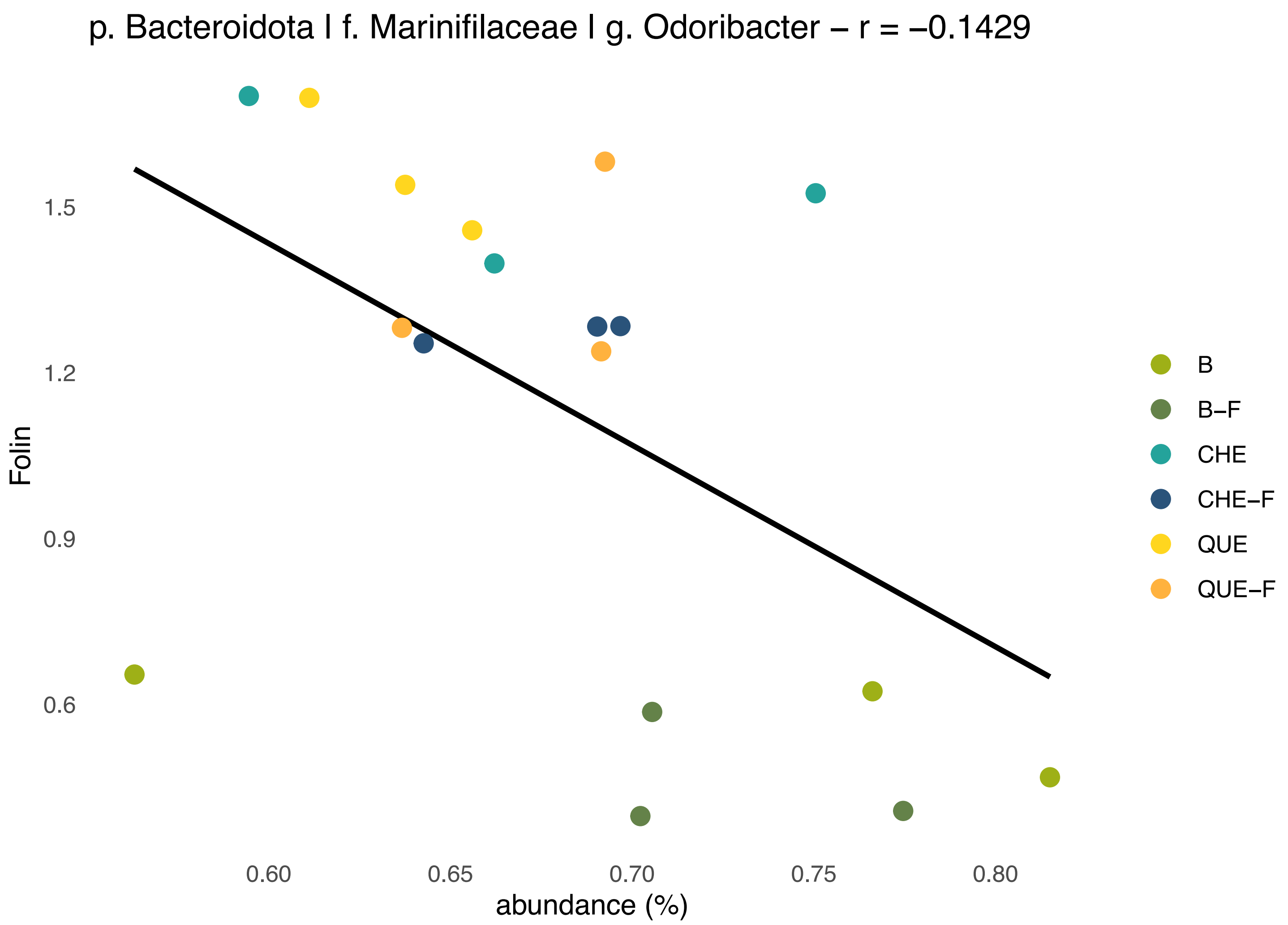
p. Bacteroidota I f. Marinifilaceae I g. Odoribacter – r = –0.1429
Folin
1.5
1.2
0.9
0.6
0.60
0.65
0.70
0.75
0.80
abundance (%)
B
B–F
CHE
CHE–F
QUE
QUE–F

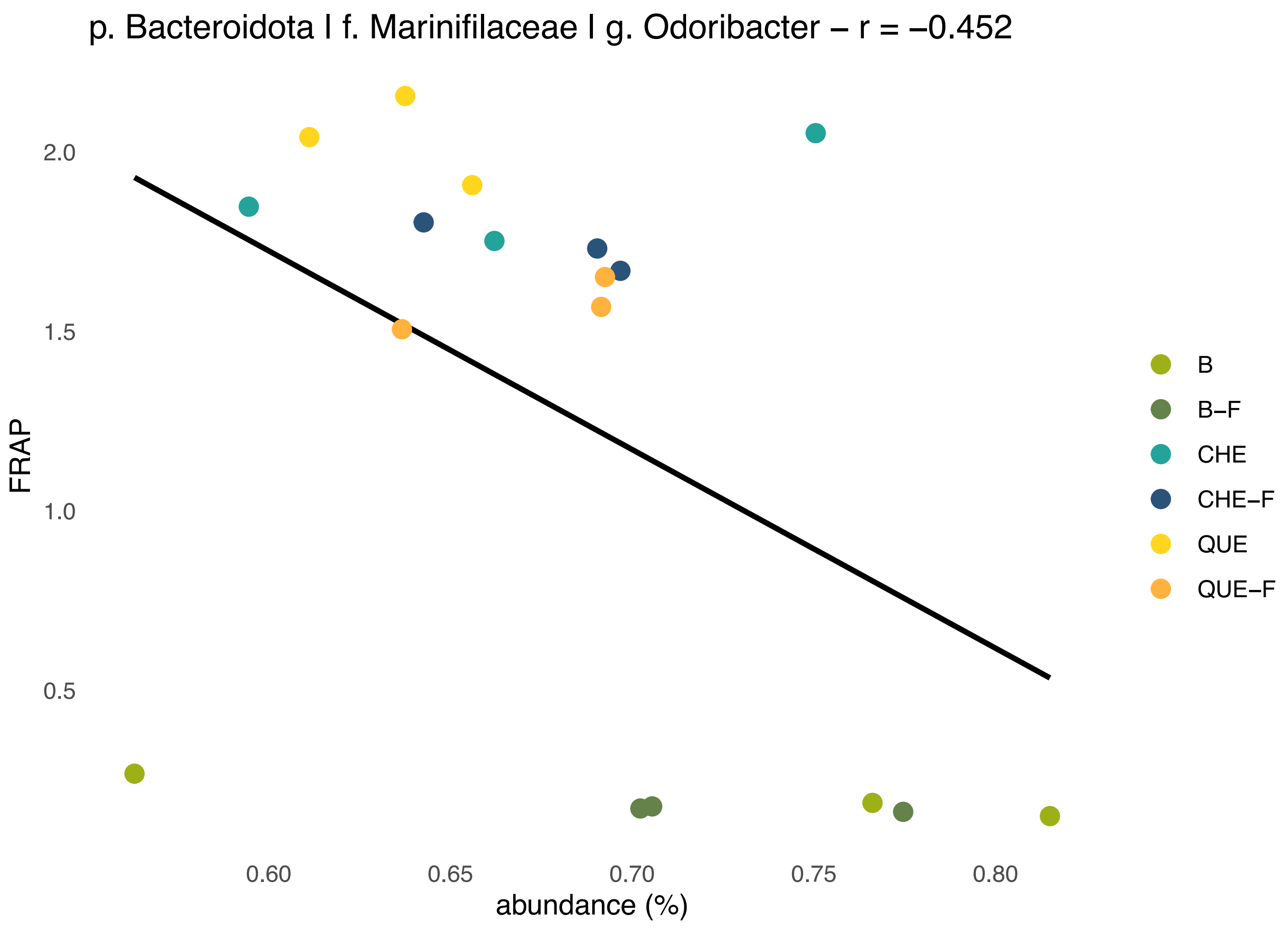

p. Bacteroidota I f. Marinifilaceae I g. Odoribacter – r = –0.452
FRAP
2.0
1.5
1.0
0.5
0.60
0.65
0.70
0.75
0.80
abundance (%)
B
B–F
CHE
CHE–F
QUE
QUE–F

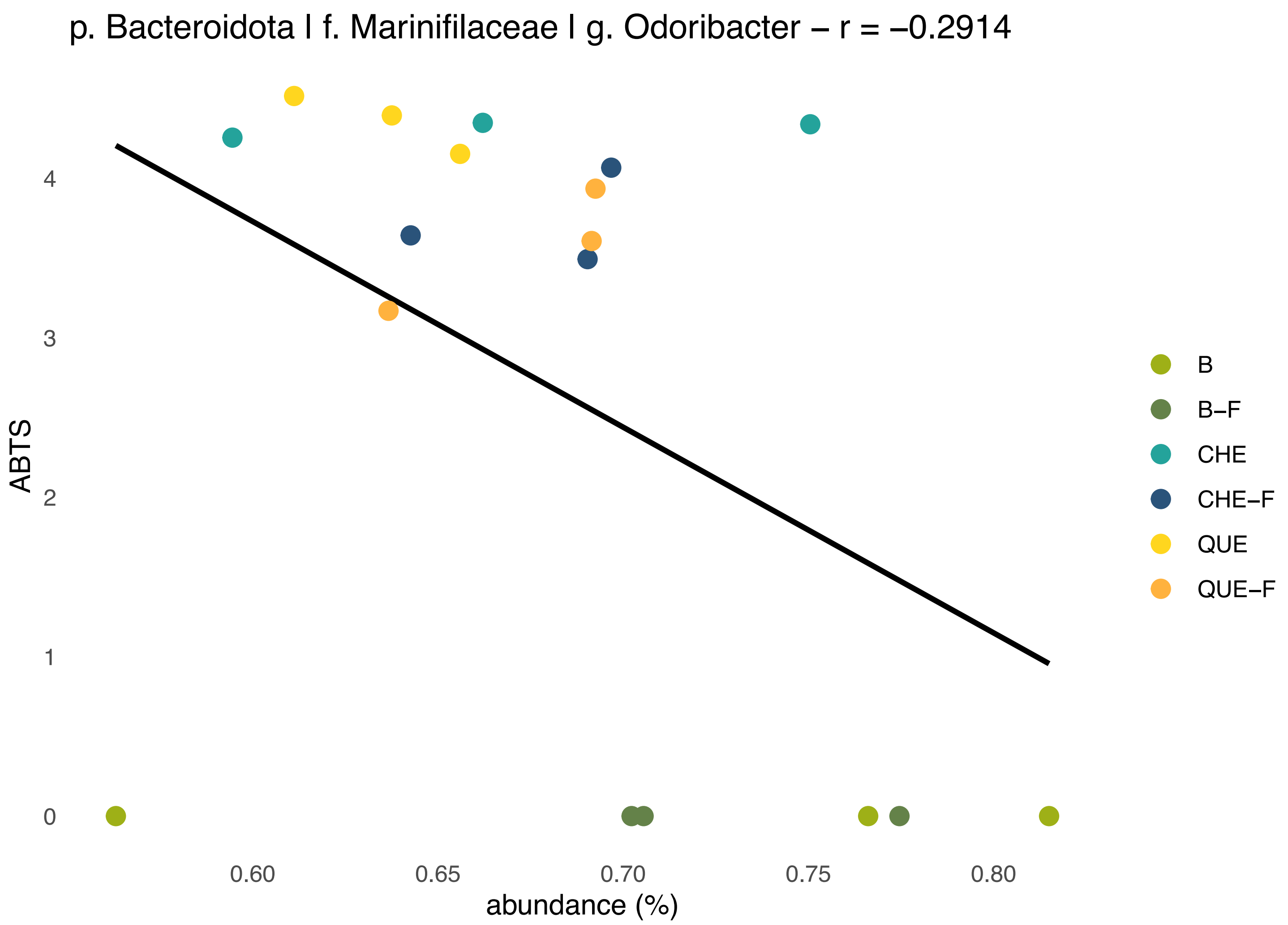

p. Bacteroidota I f. Marinifilaceae I g. Odoribacter – r = –0.2914
ABTS
0
1
2
3
4
0.60
0.65
0.70
0.75
0.80
abundance (%)
B
B–F
CHE
CHE–F
QUE
QUE–F

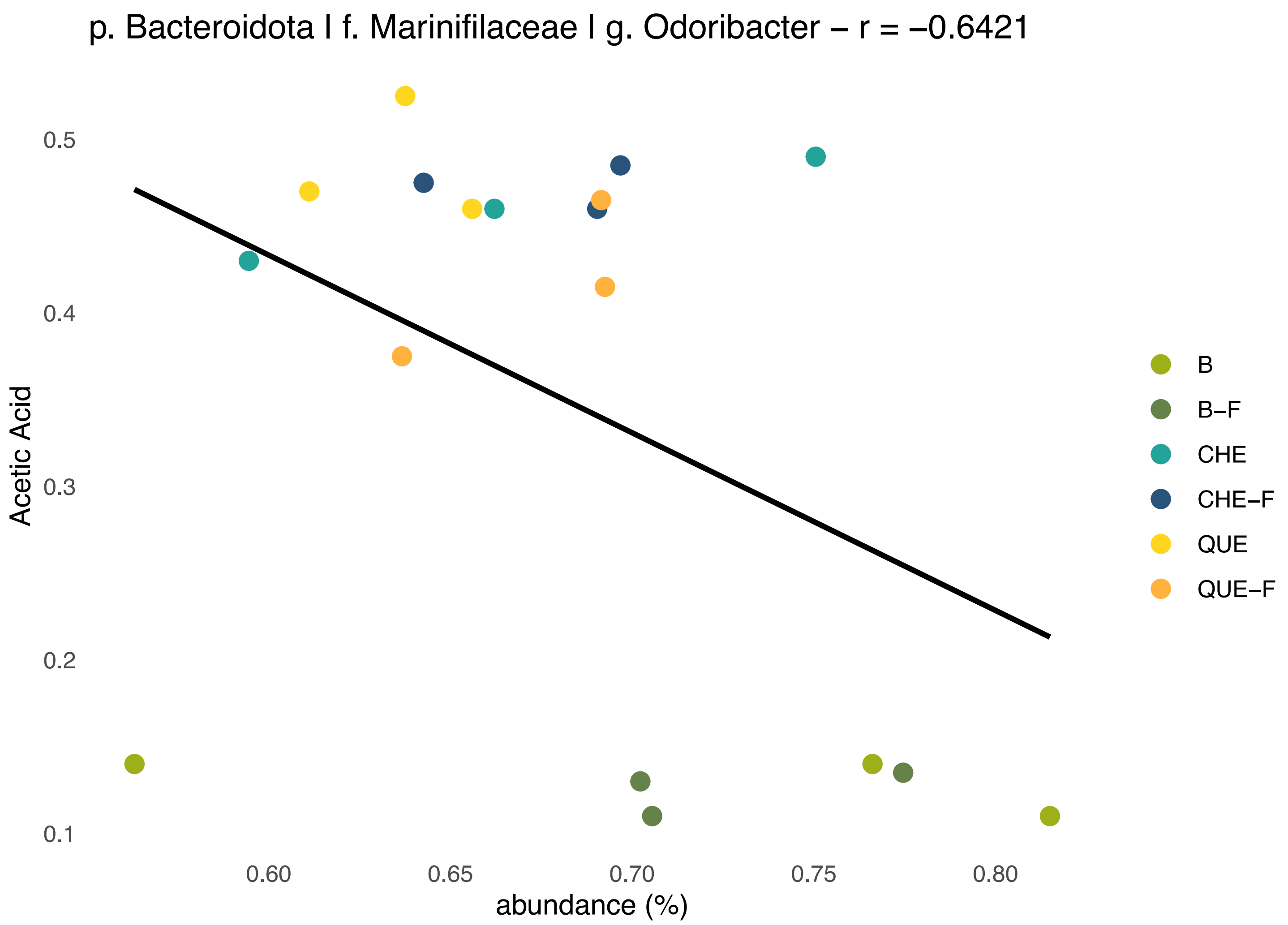
p. Bacteroidota I f. Marinifilaceae I g. Odoribacter – r = –0.6421
Acetic Acid
0.5
0.4
0.3
0.2
0.1
0.60
0.65
0.70
0.75
0.80
abundance (%)
B
B–F
CHE
CHE–F
QUE
QUE–F

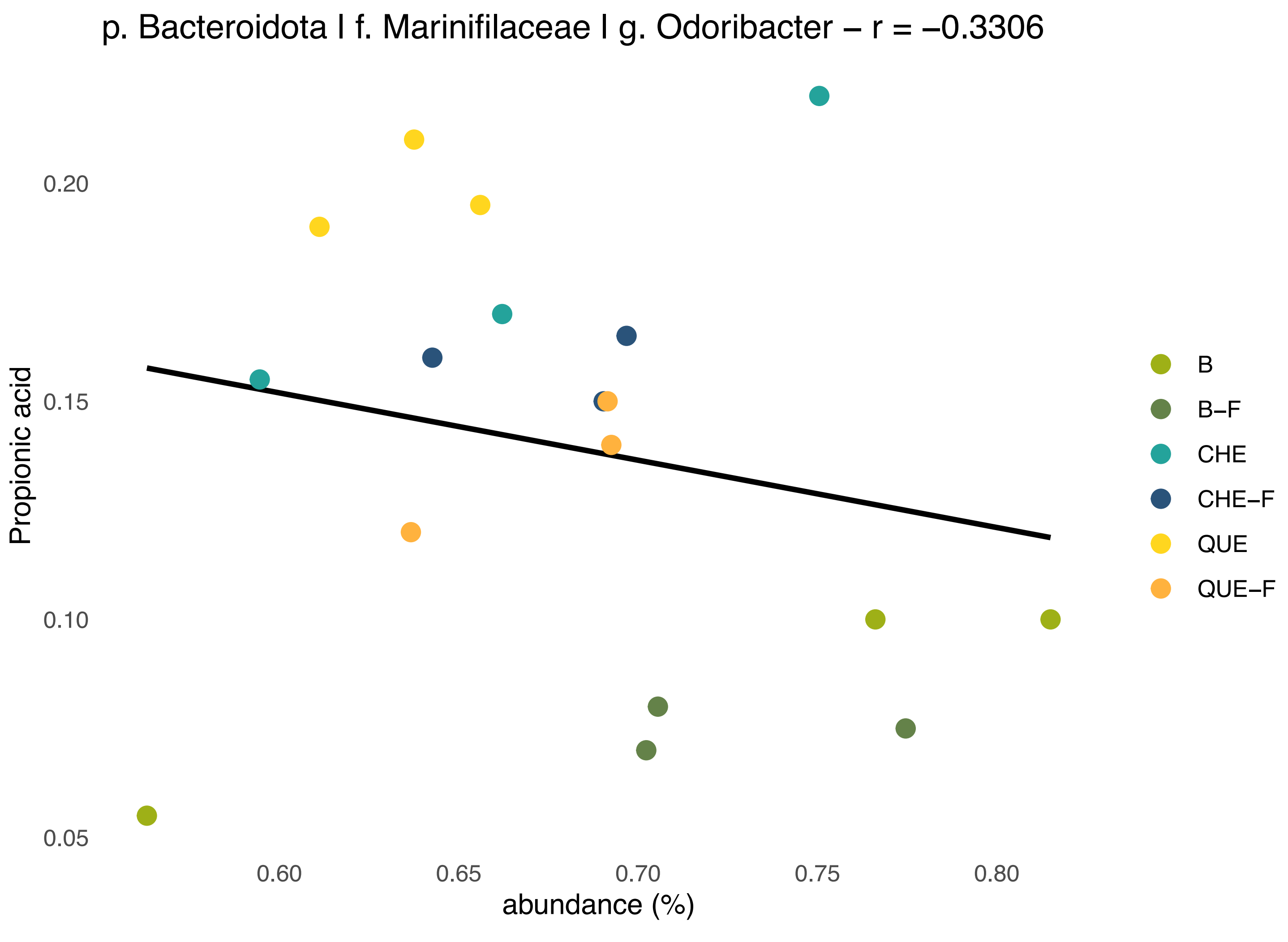
p. Bacteroidota | f. Marinifilaceae | g. Odoribacter – r = –0.3306
Propionic acid
0.20
0.15
0.10
0.05
0.60
0.65
0.70
0.75
0.80
abundance (%)
B
B–F
CHE
CHE–F
QUE
QUE–F

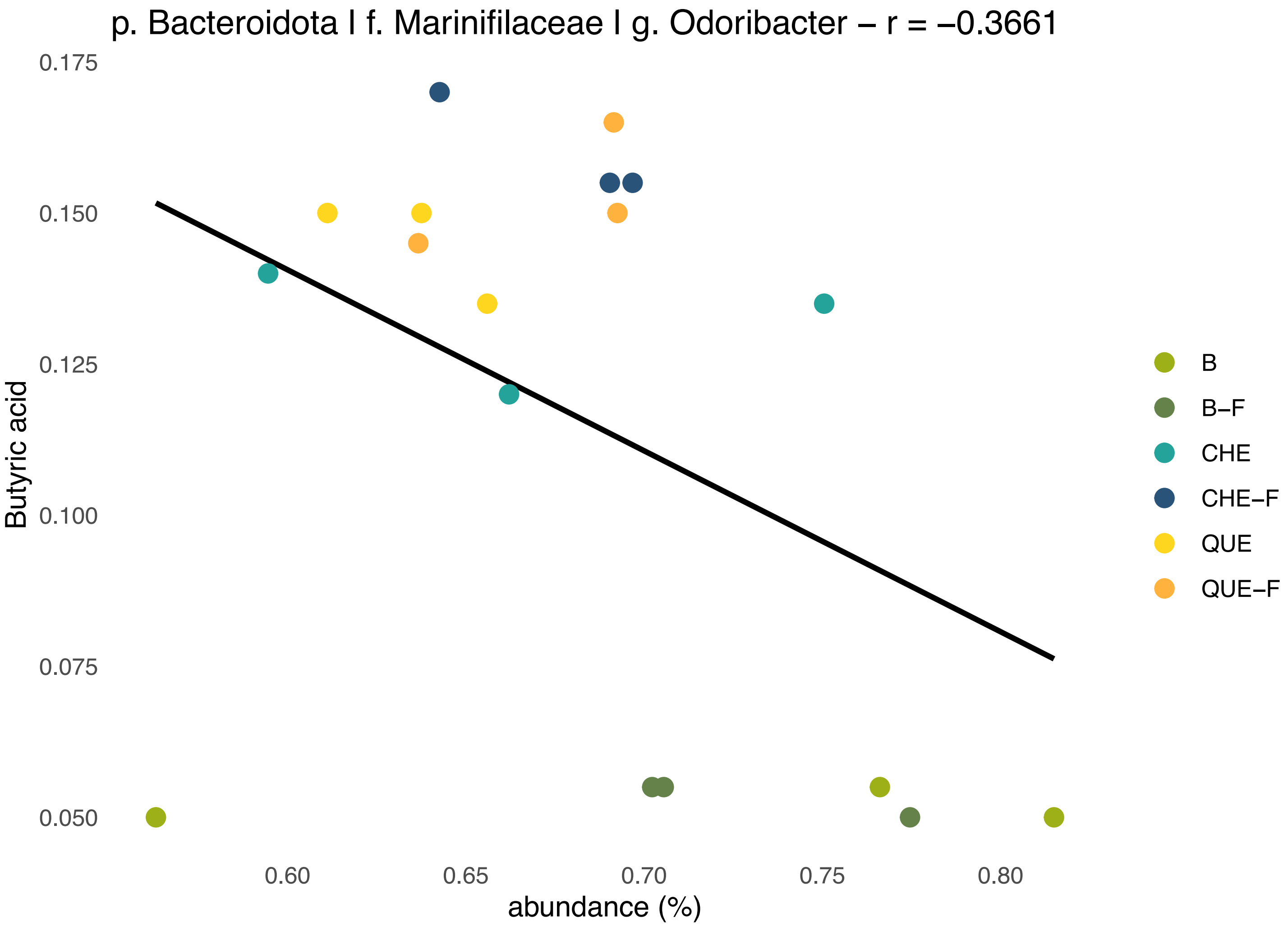
p. Bacteroidota I f. Marinifilaceae I g. Odoribacter – r = –0.3661
Butyric acid
0.175
0.150
0.125
0.100
0.075
0.050
0.60
0.65
0.70
0.75
0.80
abundance (%)
B
B–F
CHE
CHE–F
QUE
QUE–F

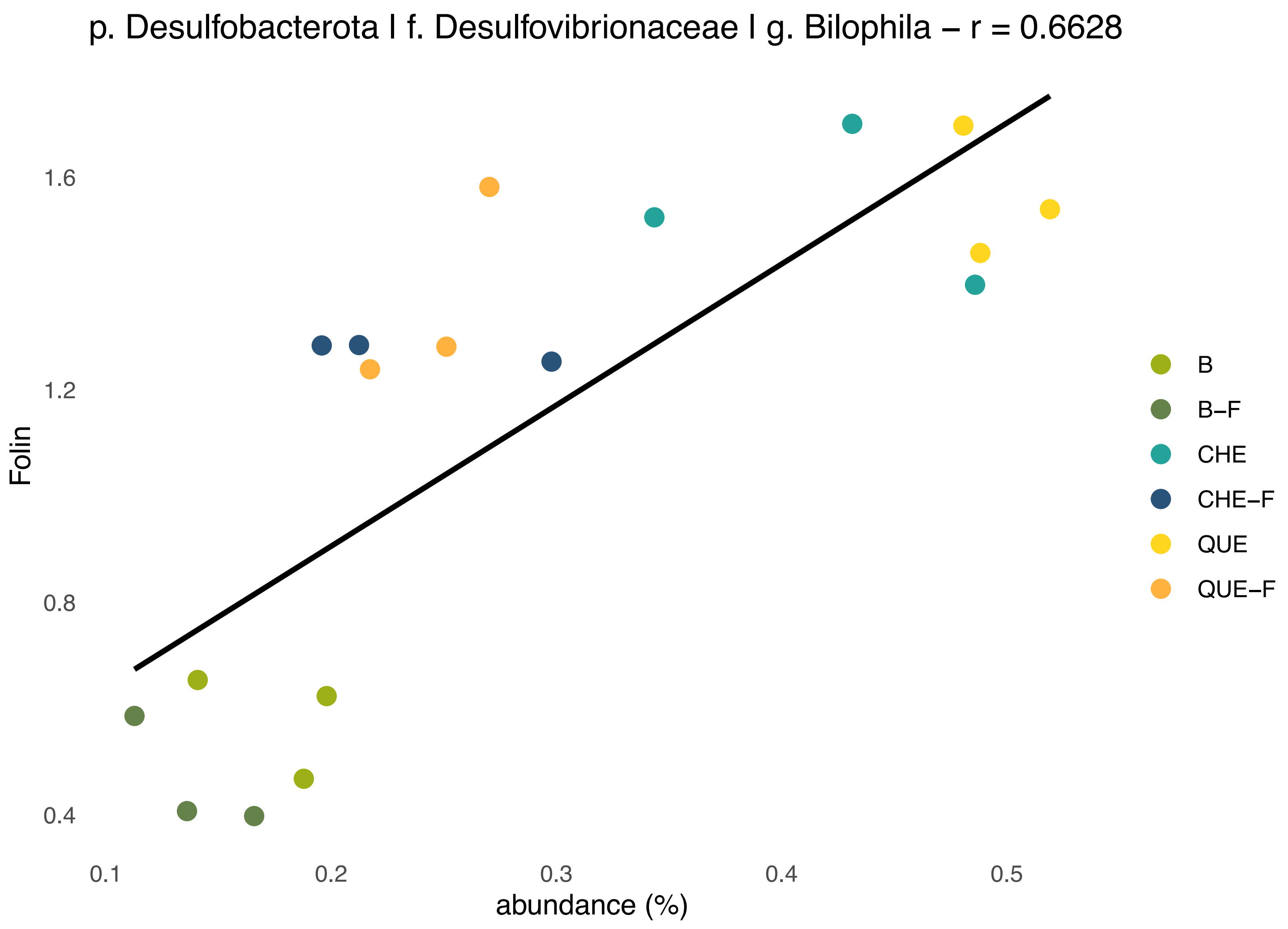
p. Desulfobacterota I f. Desulfovibrionaceae I g. Bilophila – r = 0.6628
Folin
1.6
1.2
0.8
0.4
0.1
0.2
0.3
0.4
0.5
abundance (%)
B
B–F
CHE
CHE–F
QUE
QUE–F

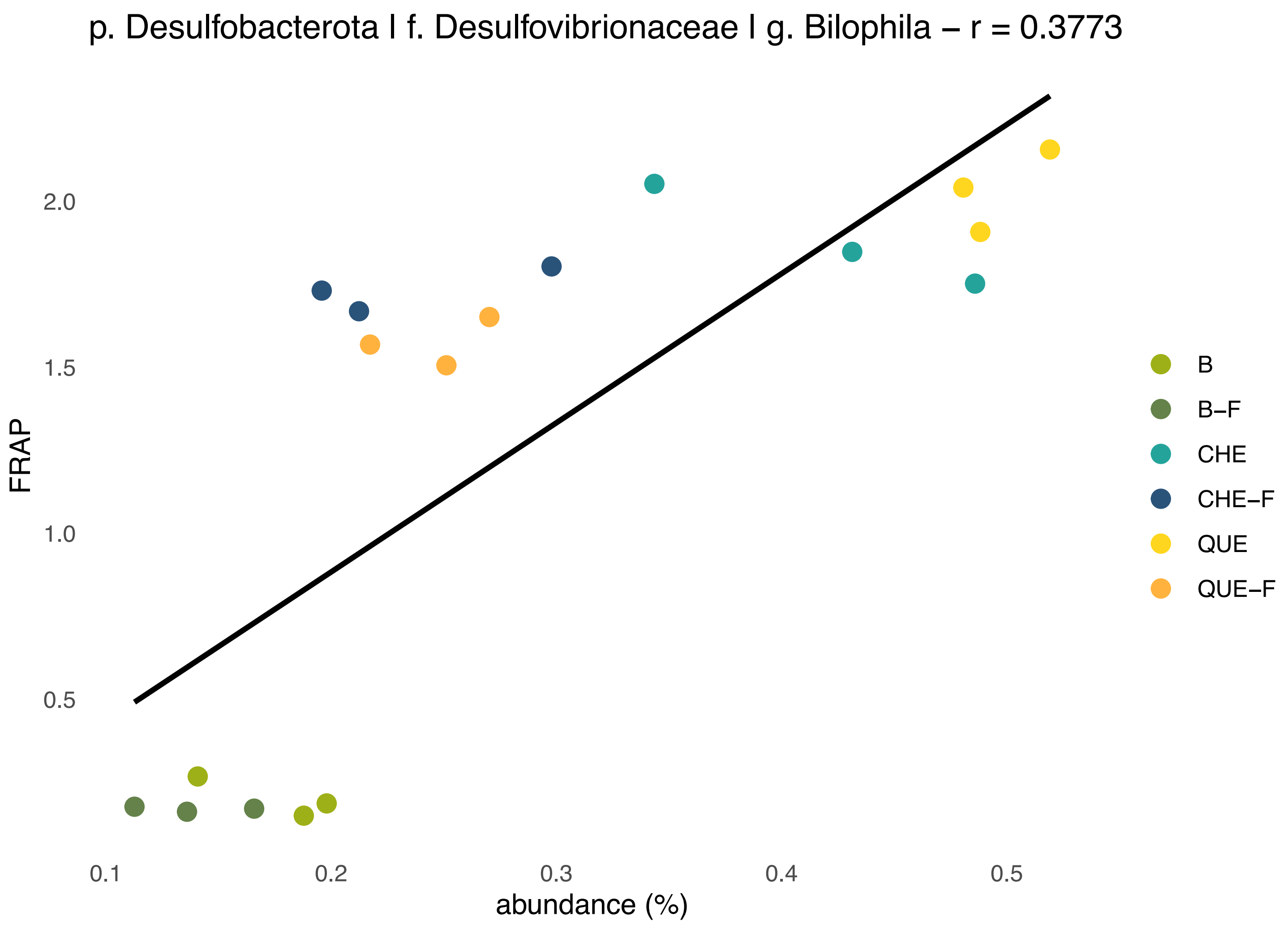
p. Desulfobacterota l f. Desulfovibrionaceae l g. Bilophila – r = 0.3773
FRAP
2.0
1.5
1.0
0.5
0.1
0.2
0.3
0.4
0.5
abundance (%)
B
B−F
CHE
CHE−F
QUE
QUE−F

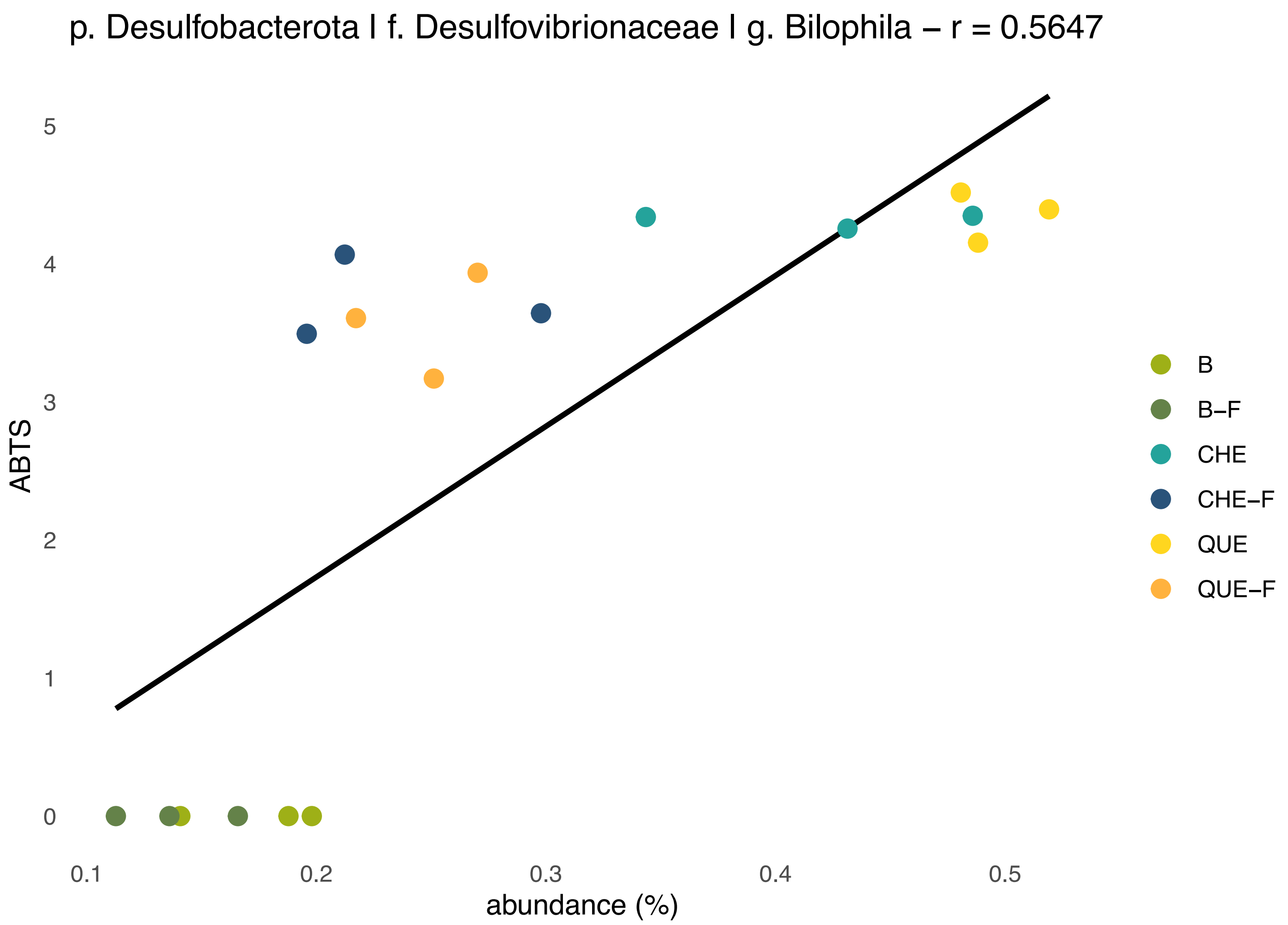
p. Desulfobacterota I f. Desulfovibrionaceae I g. Bilophila – r = 0.5647
ABTS
5
4
3
2
1
0
0.1
0.2
0.3
0.4
0.5
abundance (%)
B
B–F
CHE
CHE–F
QUE
QUE–F

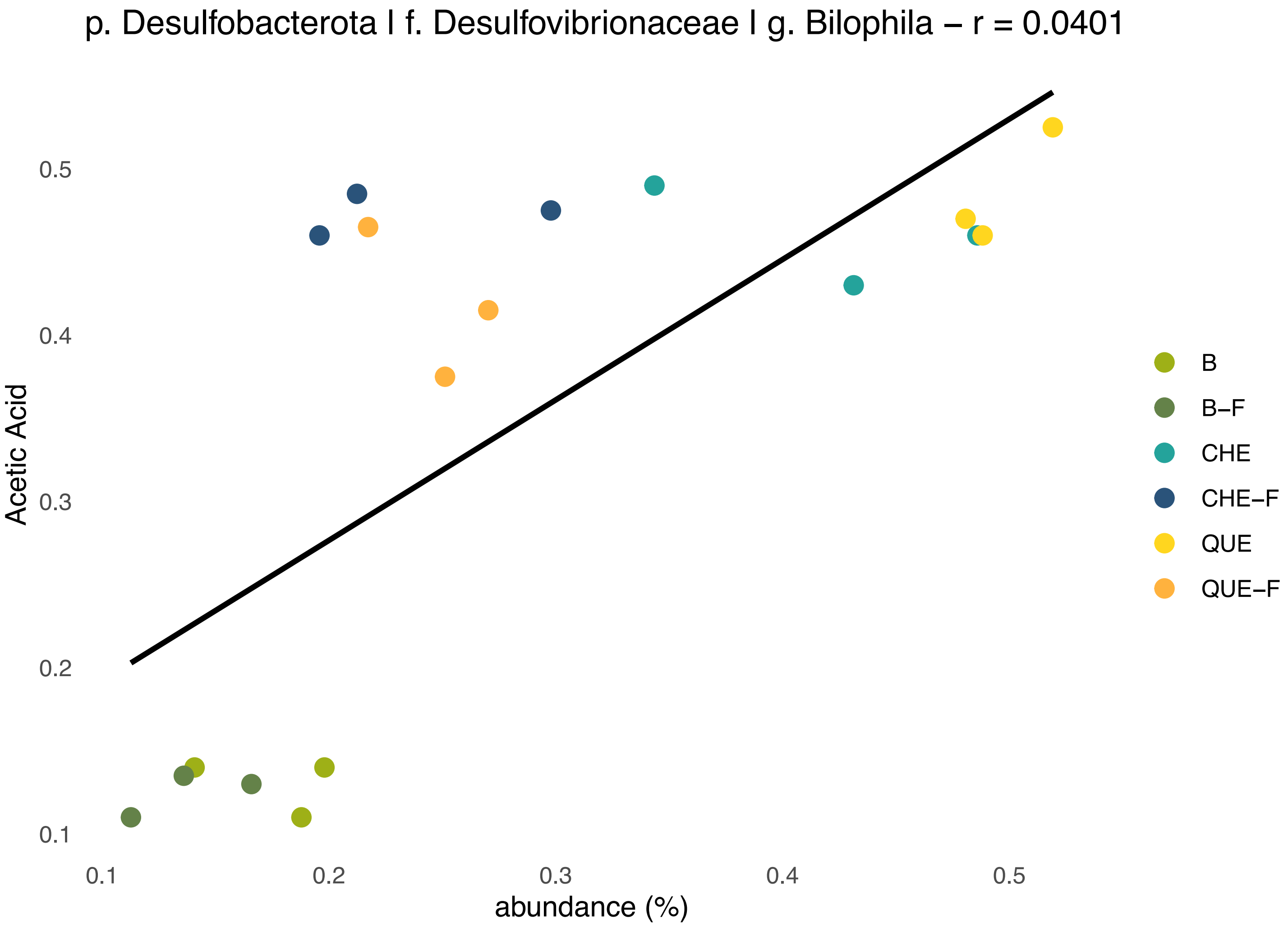
p. Desulfobacterota l f. Desulfovibrionaceae l g. Bilophila – r = 0.0401
Acetic Acid
0.5
0.4
0.3
0.2
0.1
0.1
0.2
0.3
0.4
0.5
abundance (%)
B
B–F
CHE
CHE–F
QUE
QUE–F

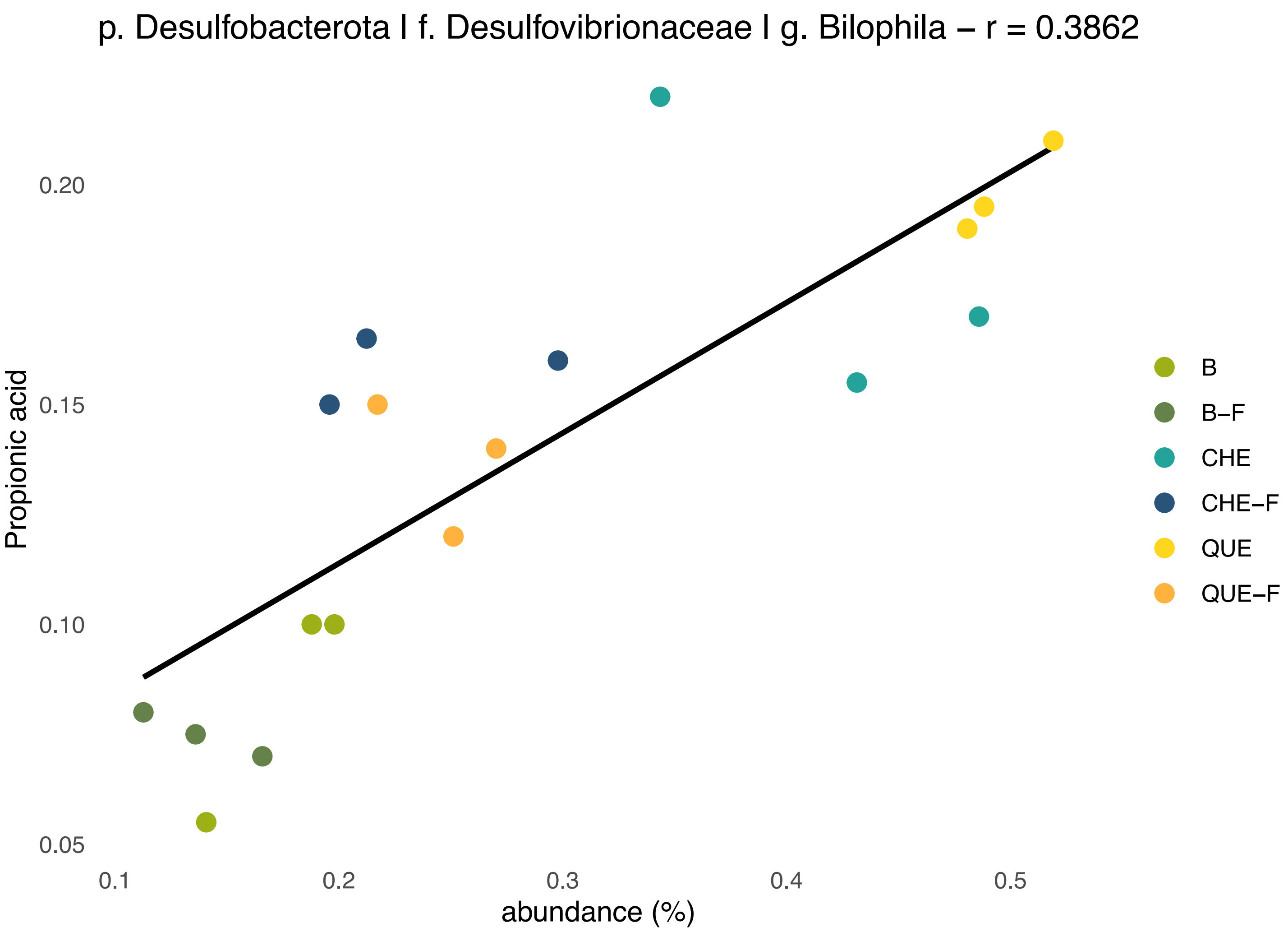
p. Desulfobacterota I f. Desulfovibrionaceae I g. Bilophila − r = 0.3862
Propionic acid
0.20
0.15
0.10
0.05
0.1
0.2
0.3
0.4
0.5
abundance (%)
B
B−F
CHE
CHE−F
QUE
QUE−F

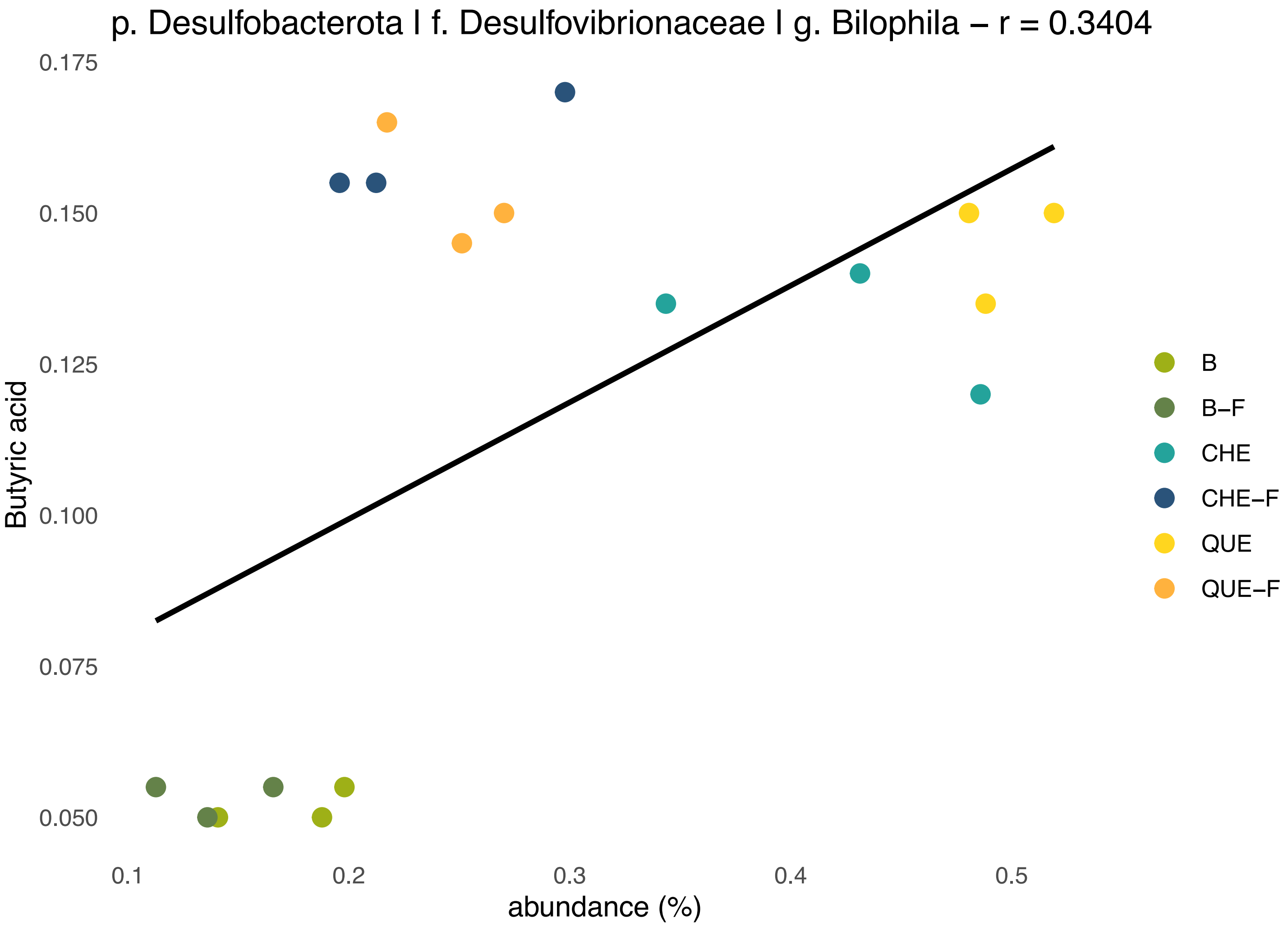
p. Desulfobacterota I f. Desulfovibrionaceae I g. Bilophila – r = 0.3404
0.175
0.150
0.125
0.100
0.075
0.050
Butyric acid
0.1
0.2
0.3
0.4
0.5
abundance (%)
B
B–F
CHE
CHE–F
QUE
QUE–F

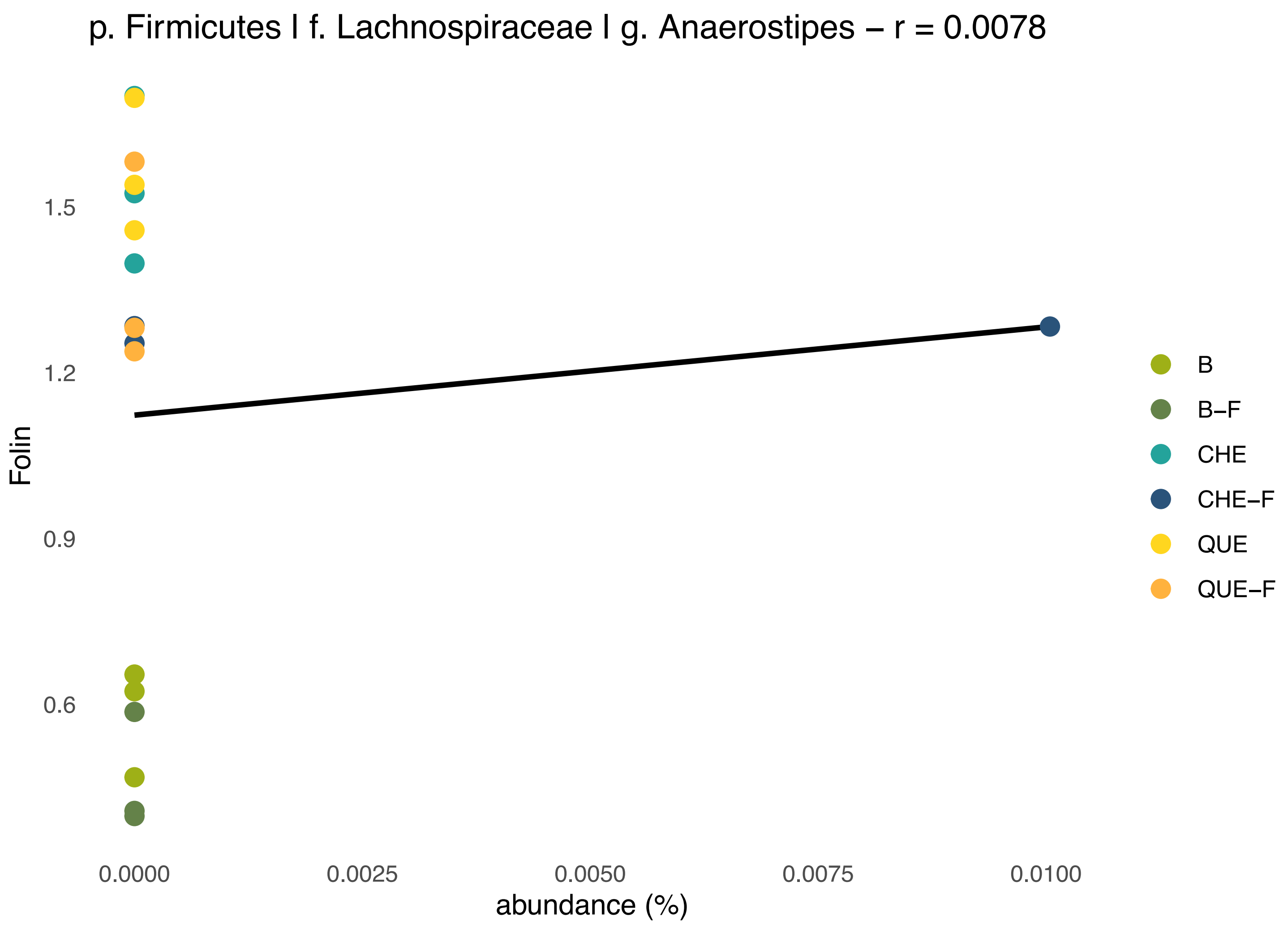

p. Firmicutes I f. Lachnospiraceae I g. Anaerostipes – r = 0.0078
Folin
1.5
1.2
0.9
0.6
0.0000
0.0025
0.0050
0.0075
0.0100
abundance (%)
B
B–F
CHE
CHE–F
QUE
QUE–F

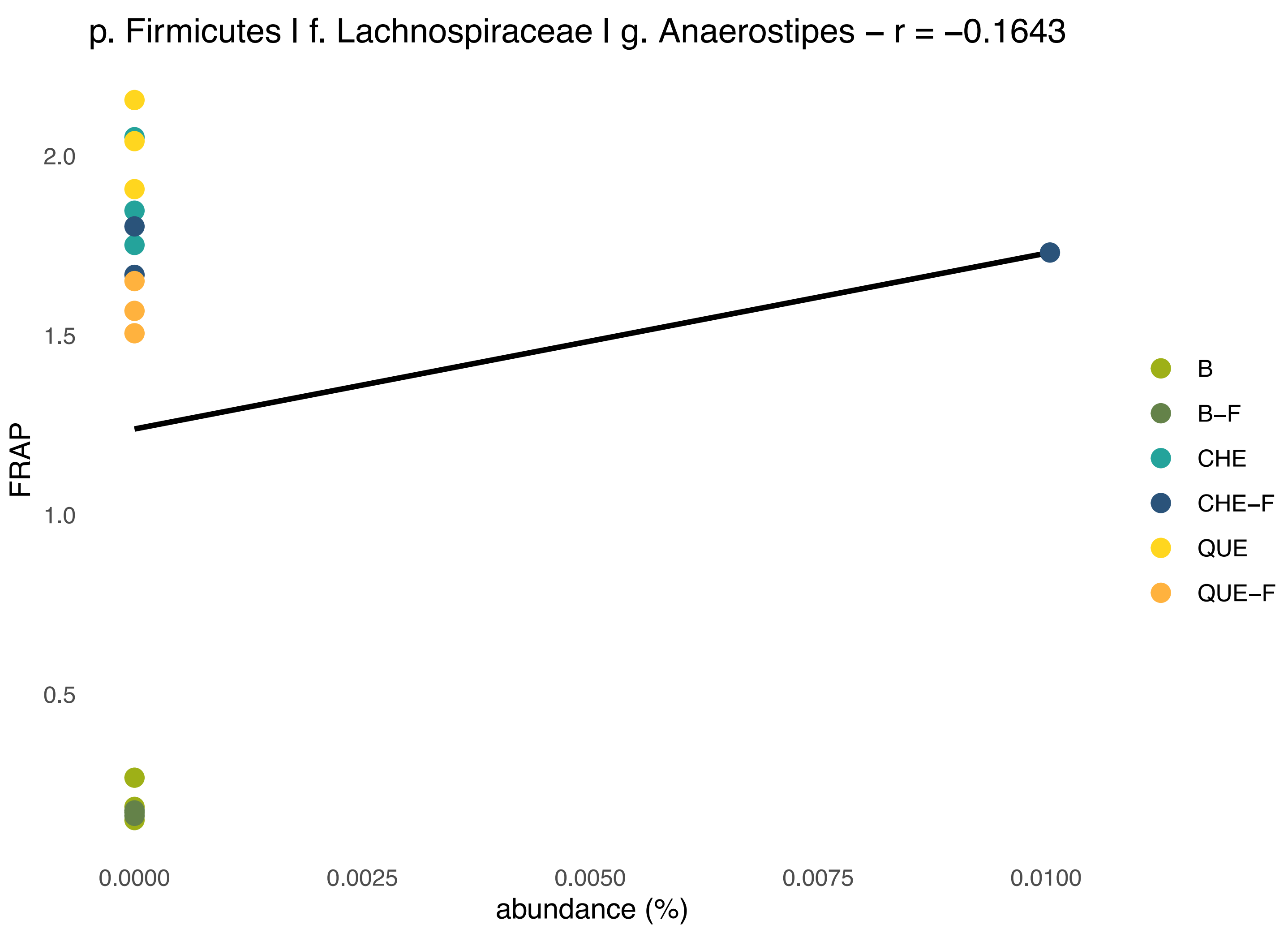

p. Firmicutes I f. Lachnospiraceae I g. Anaerostipes – r = –0.1643
FRAP
2.0
1.5
1.0
0.5
0.0000
0.0025
0.0050
0.0075
0.0100
abundance (%)
B
B–F
CHE
CHE–F
QUE
QUE–F

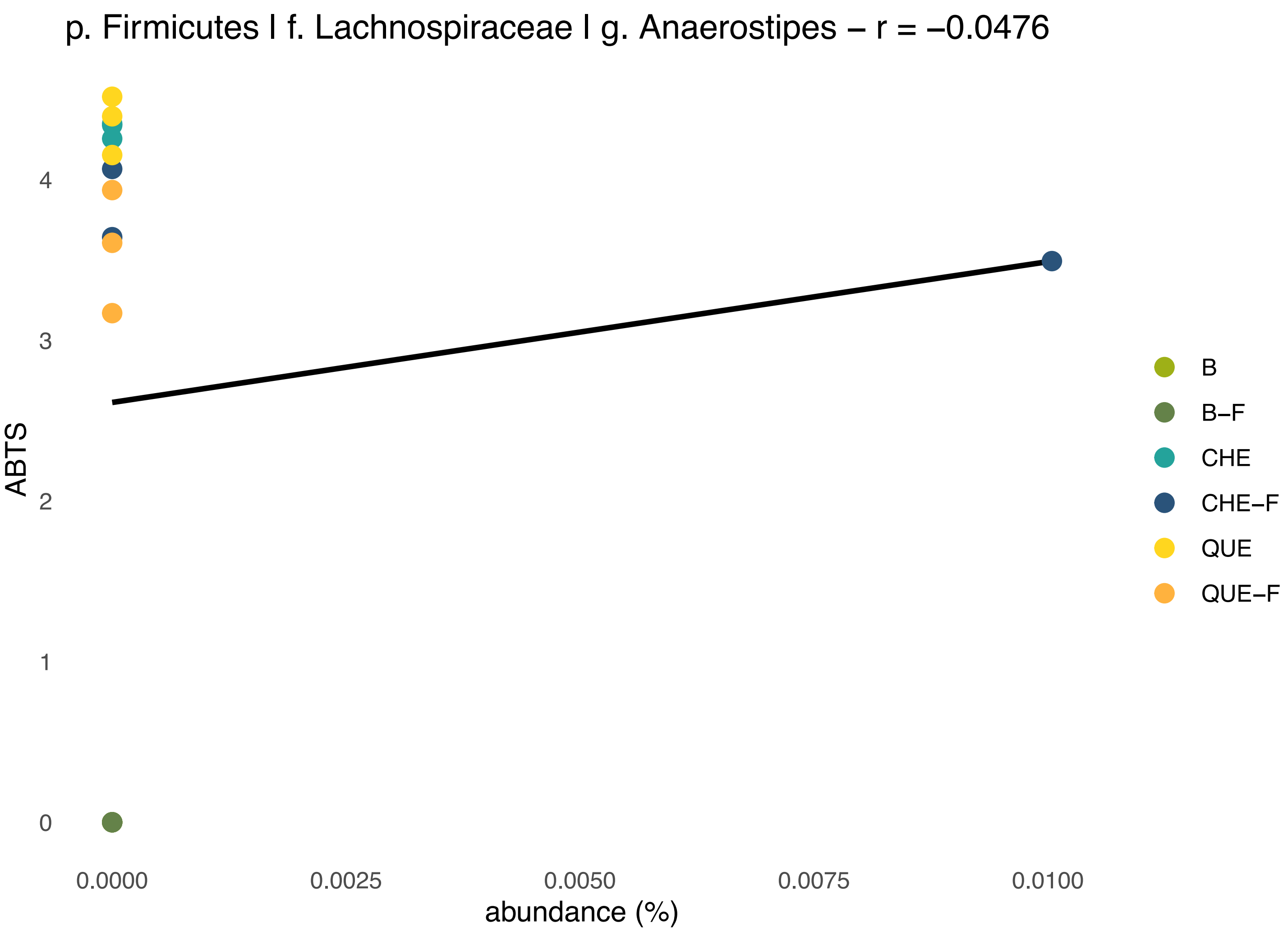

p. Firmicutes I f. Lachnospiraceae I g. Anaerostipes – r = –0.0476
ABTS
0
1
2
3
4
abundance (%)
0.0000
0.0025
0.0050
0.0075
0.0100
B
B–F
CHE
CHE–F
QUE
QUE–F

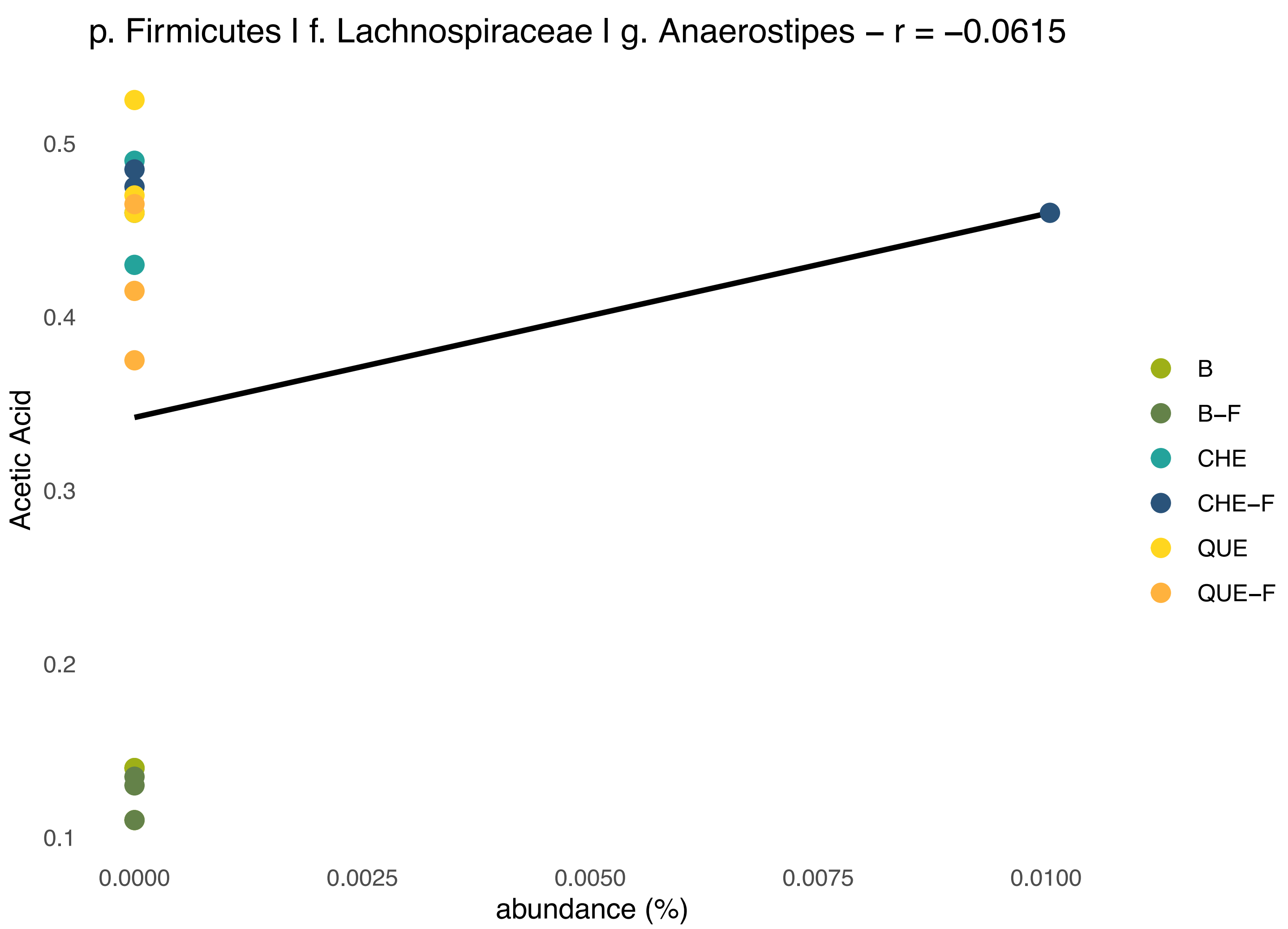
p. Firmicutes I f. Lachnospiraceae I g. Anaerostipes – r = –0.0615
Acetic Acid
0.5
0.4
0.3
0.2
0.1
0.0000
0.0025
0.0050
0.0075
0.0100
abundance (%)
B
B–F
CHE
CHE–F
QUE
QUE–F

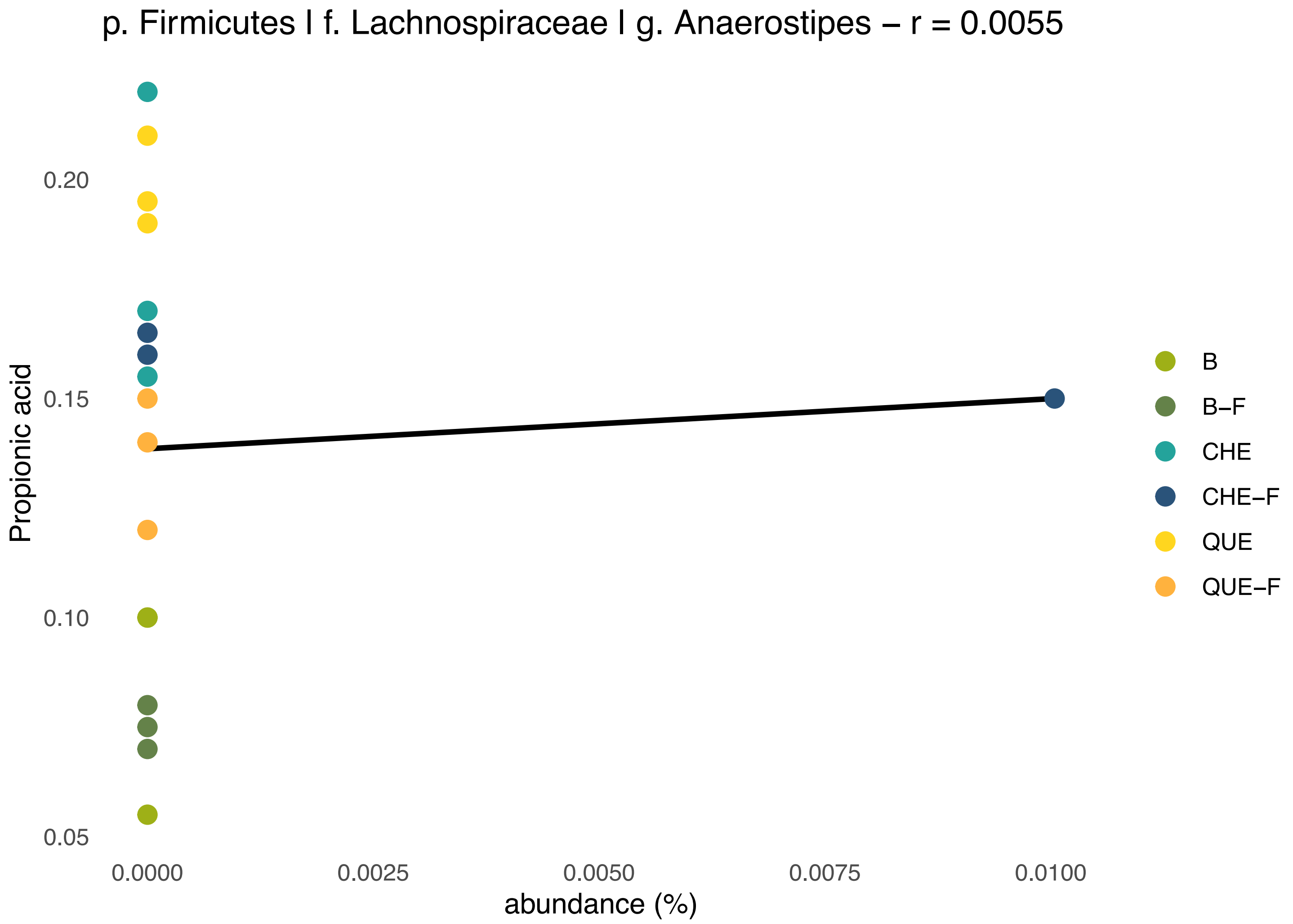

p. Firmicutes l f. Lachnospiraceae l g. Anaerostipes – r = 0.0055
Propionic acid
0.20
0.15
0.10
0.05
0.0000
0.0025
0.0050
0.0075
0.0100
abundance (%)
B
B–F
CHE
CHE–F
QUE
QUE–F

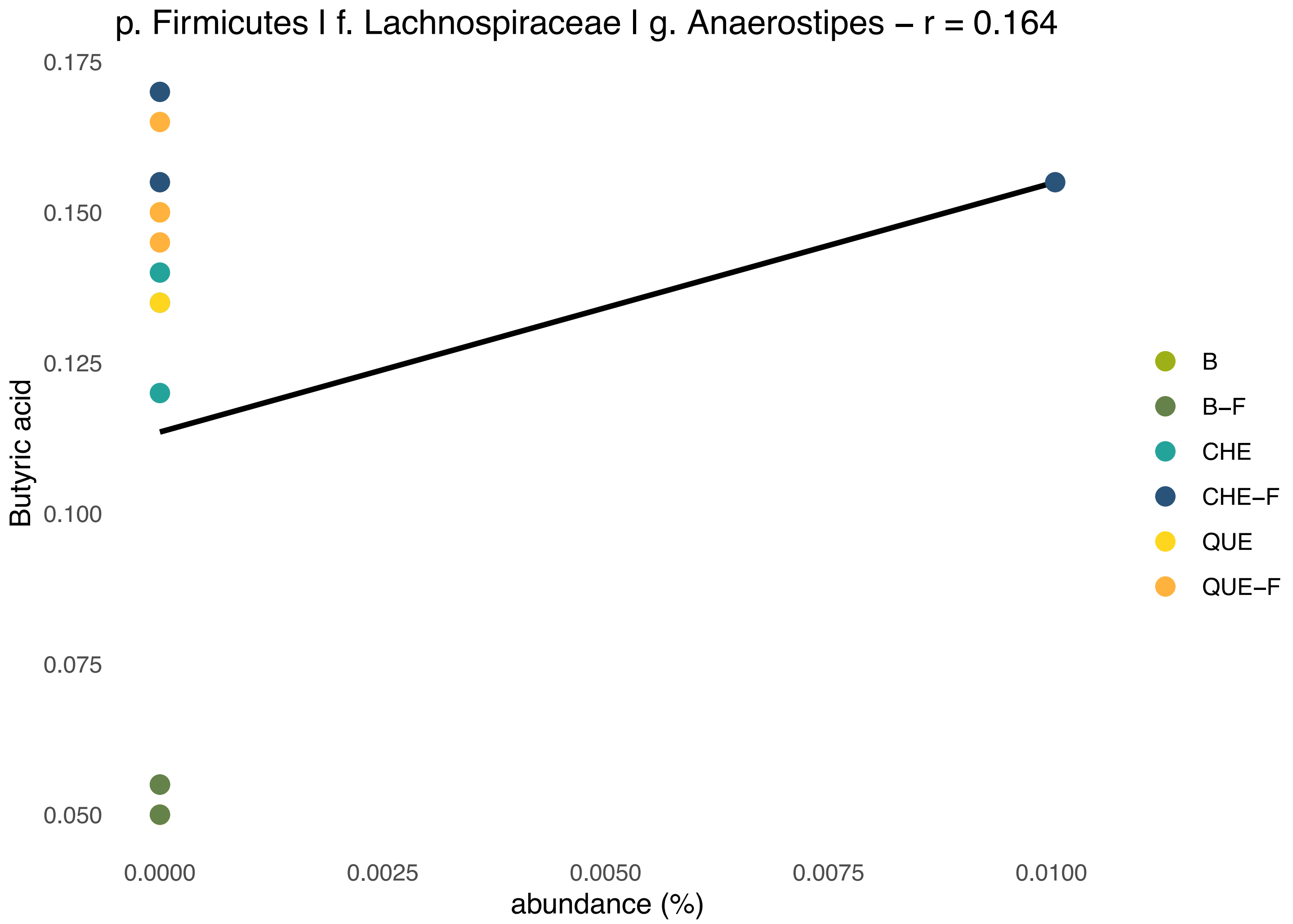
p. Firmicutes I f. Lachnospiraceae I g. Anaerostipes – r = 0.164
Butyric acid
0.175
0.150
0.125
0.100
0.075
0.050
0.0000
0.0025
0.0050
0.0075
0.0100
abundance (%)
B
B–F
CHE
CHE–F
QUE
QUE–F

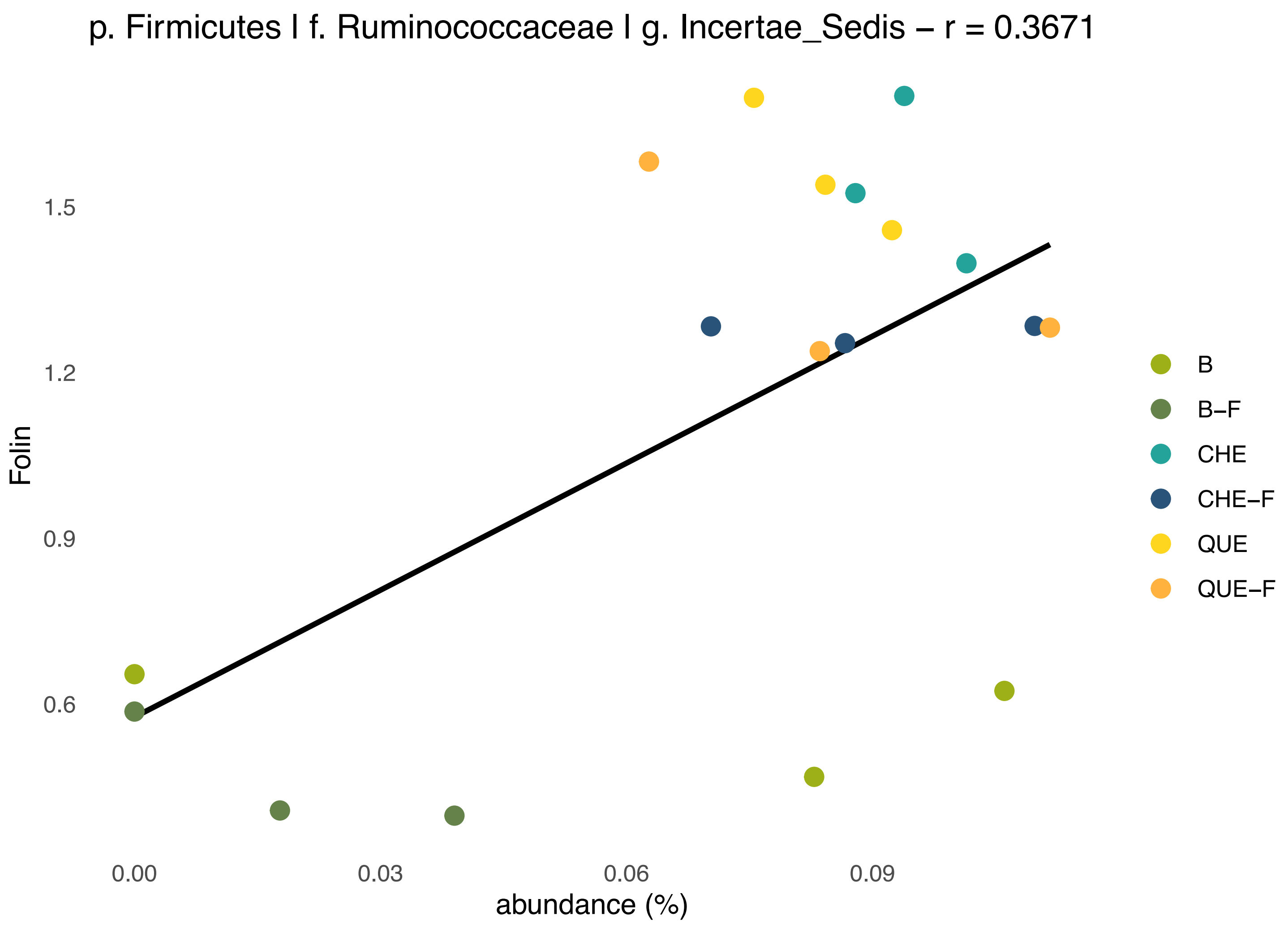

p. Firmicutes | f. Ruminococcaceae | g. Incertae_Sedis – r = 0.3671
Folin
1.5
1.2
0.9
0.6
0.00
0.03
0.06
0.09
abundance (%)
B
B–F
CHE
CHE–F
QUE
QUE–F

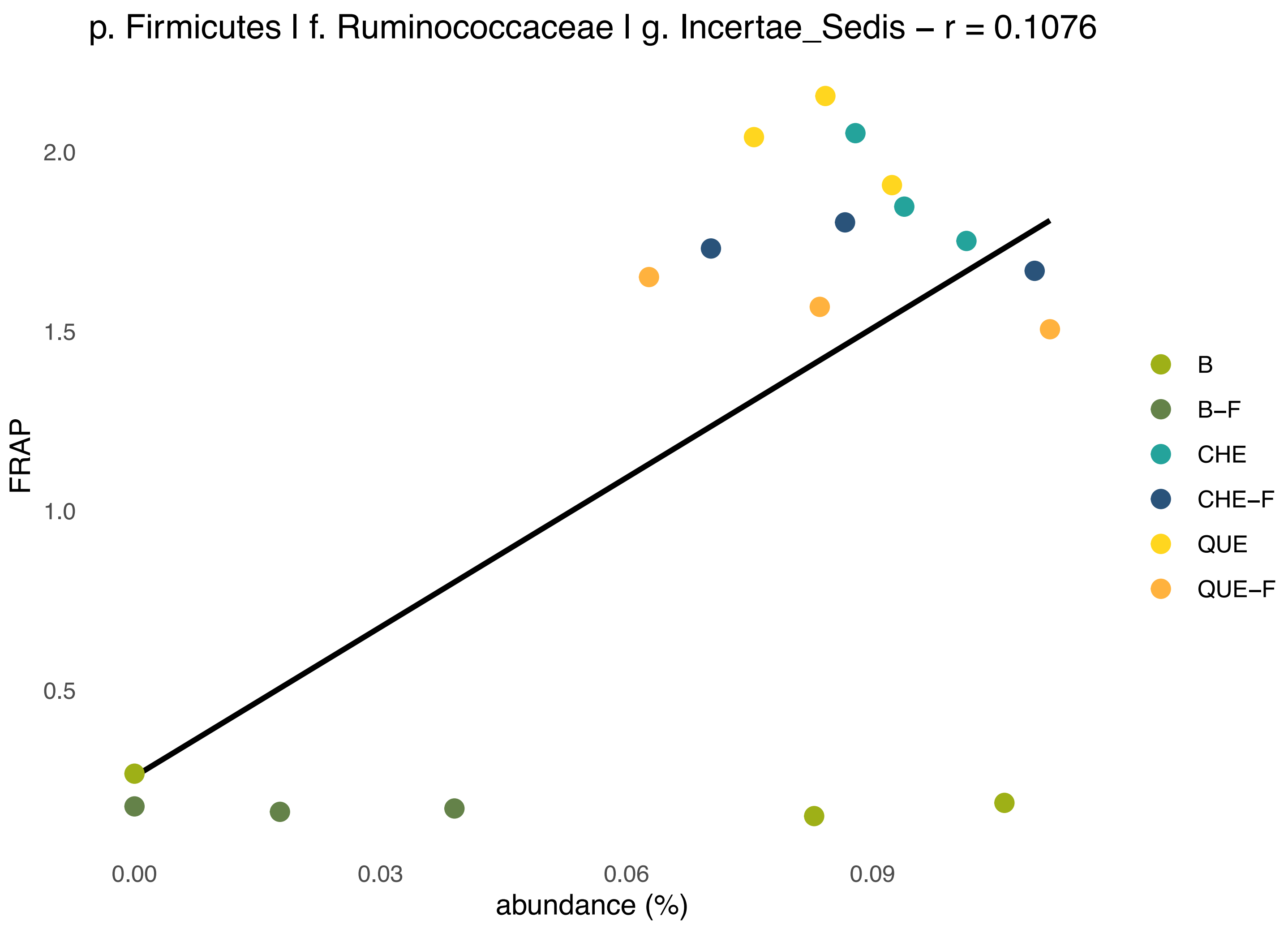
p. Firmicutes I f. Ruminococcaceae I g. Incertae_Sedis – r = 0.1076
FRAP
2.0
1.5
1.0
0.5
0.00
0.03
0.06
0.09
abundance (%)
B
B–F
CHE
CHE–F
QUE
QUE–F

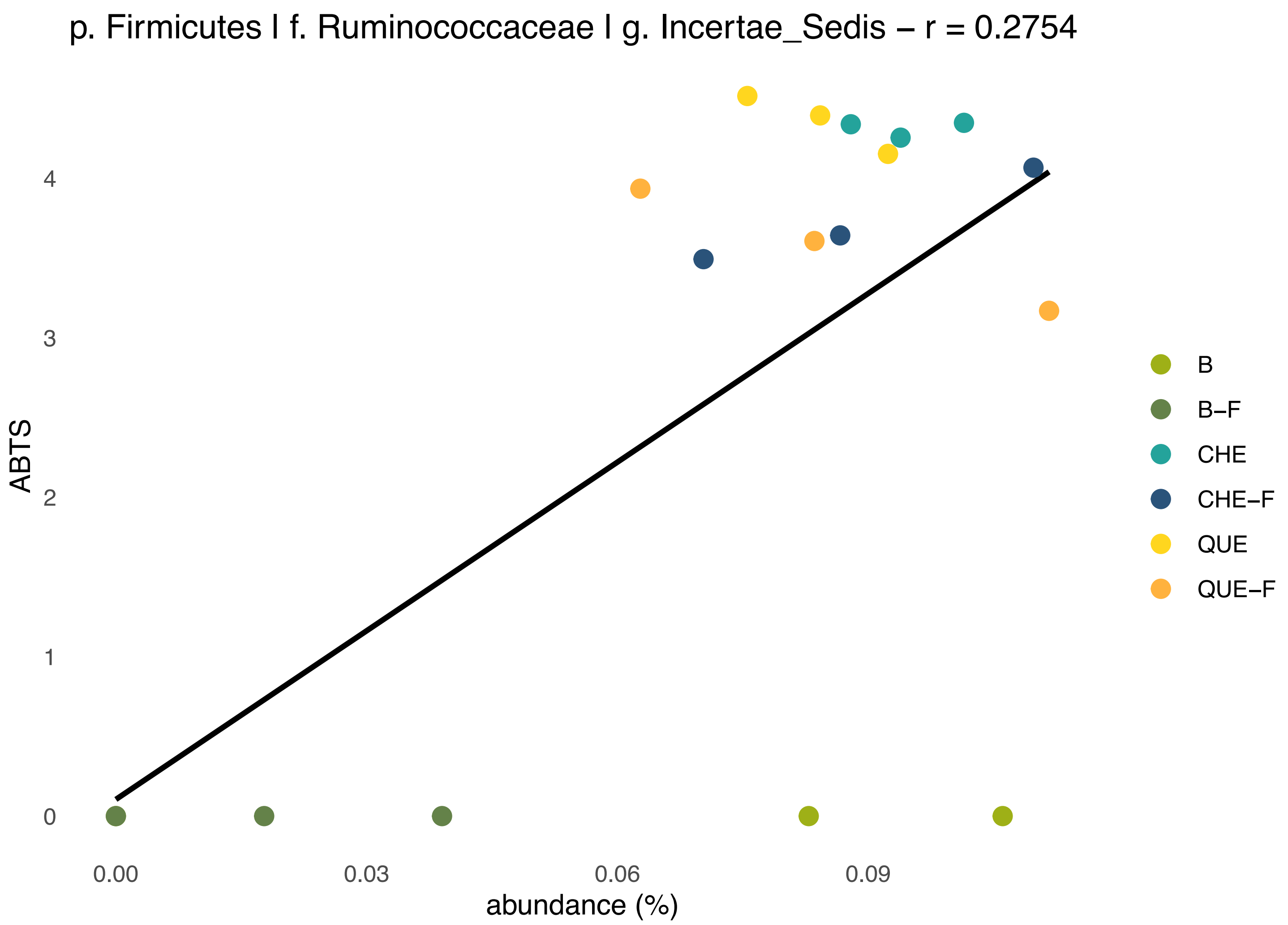

p. Firmicutes I f. Ruminococcaceae I g. Incertae_Sedis – r = 0.2754
ABTS
4
3
2
1
0
0.00
0.03
0.06
0.09
abundance (%)
B
B–F
CHE
CHE–F
QUE
QUE–F

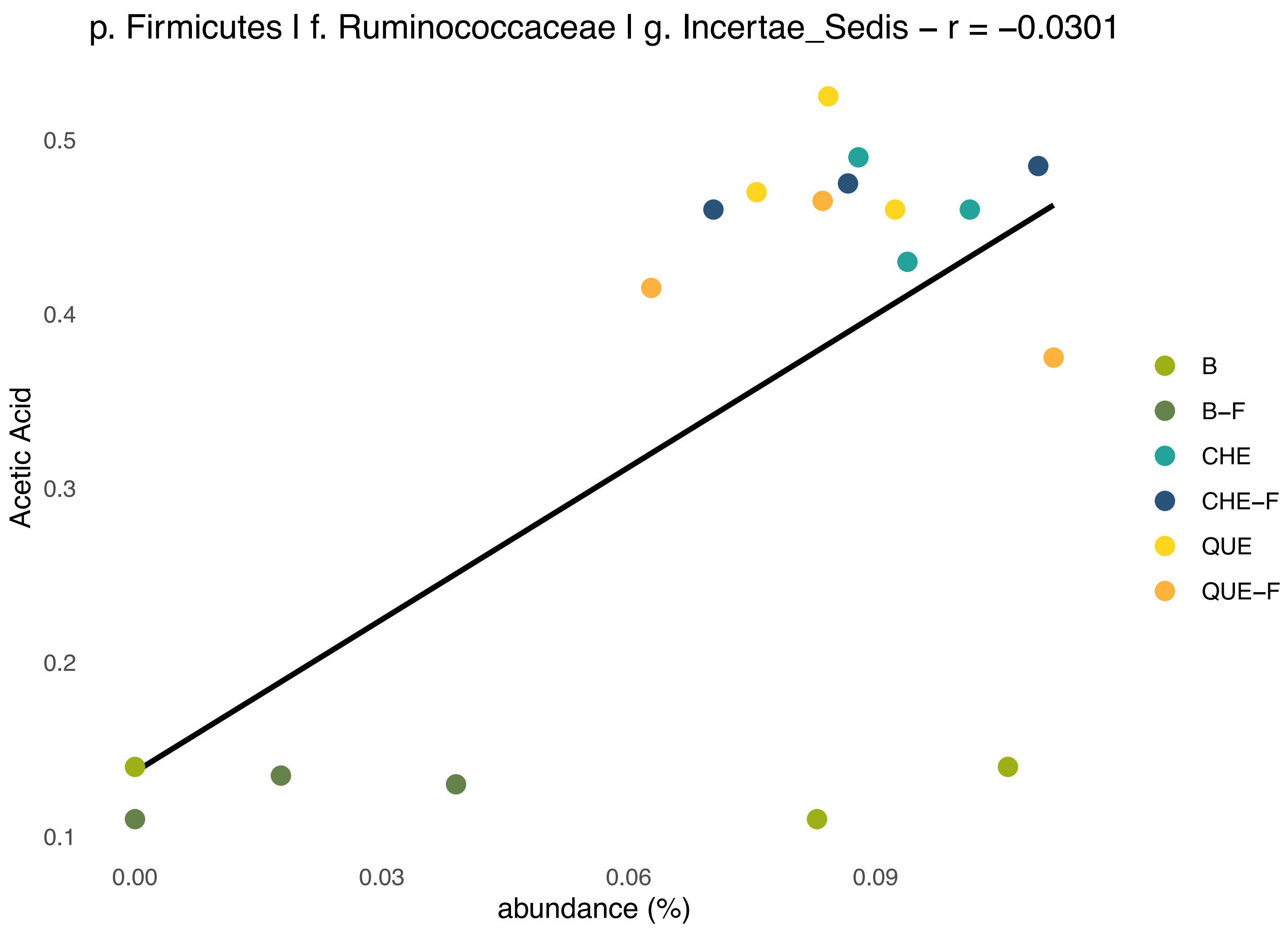

p. Firmicutes I f. Ruminococcaceae I g. Incertae_Sedis – r = –0.0301
Acetic Acid
0.5
0.4
0.3
0.2
0.1
0.00
0.03
0.06
0.09
abundance (%)
B
B–F
CHE
CHE–F
QUE
QUE–F

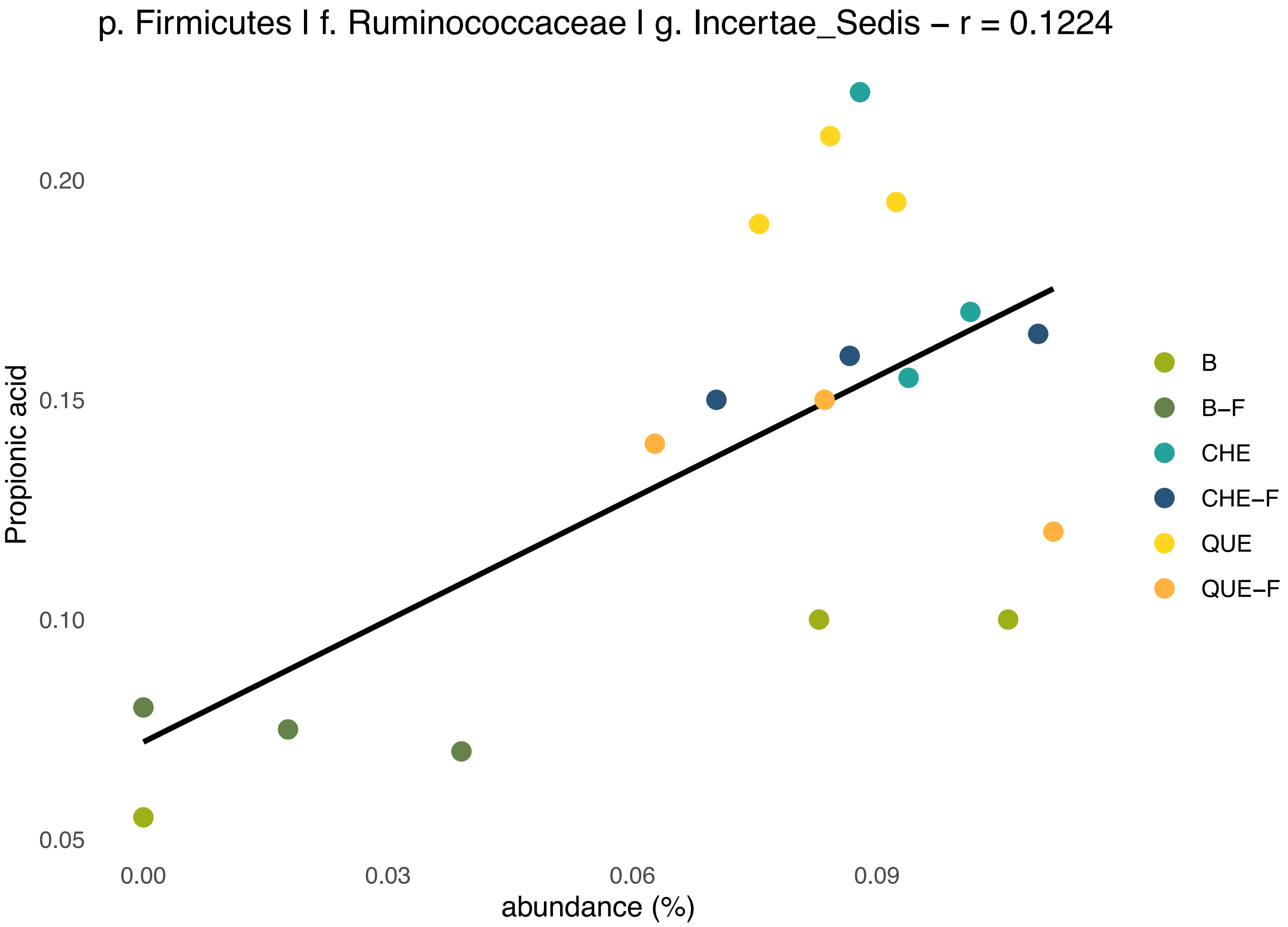

p. Firmicutes l f. Ruminococcaceae l g. Incertae_Sedis – r = 0.1224
Propionic acid
0.20
0.15
0.10
0.05
0.00
0.03
0.06
0.09
abundance (%)
B
B–F
CHE
CHE–F
QUE
QUE–F

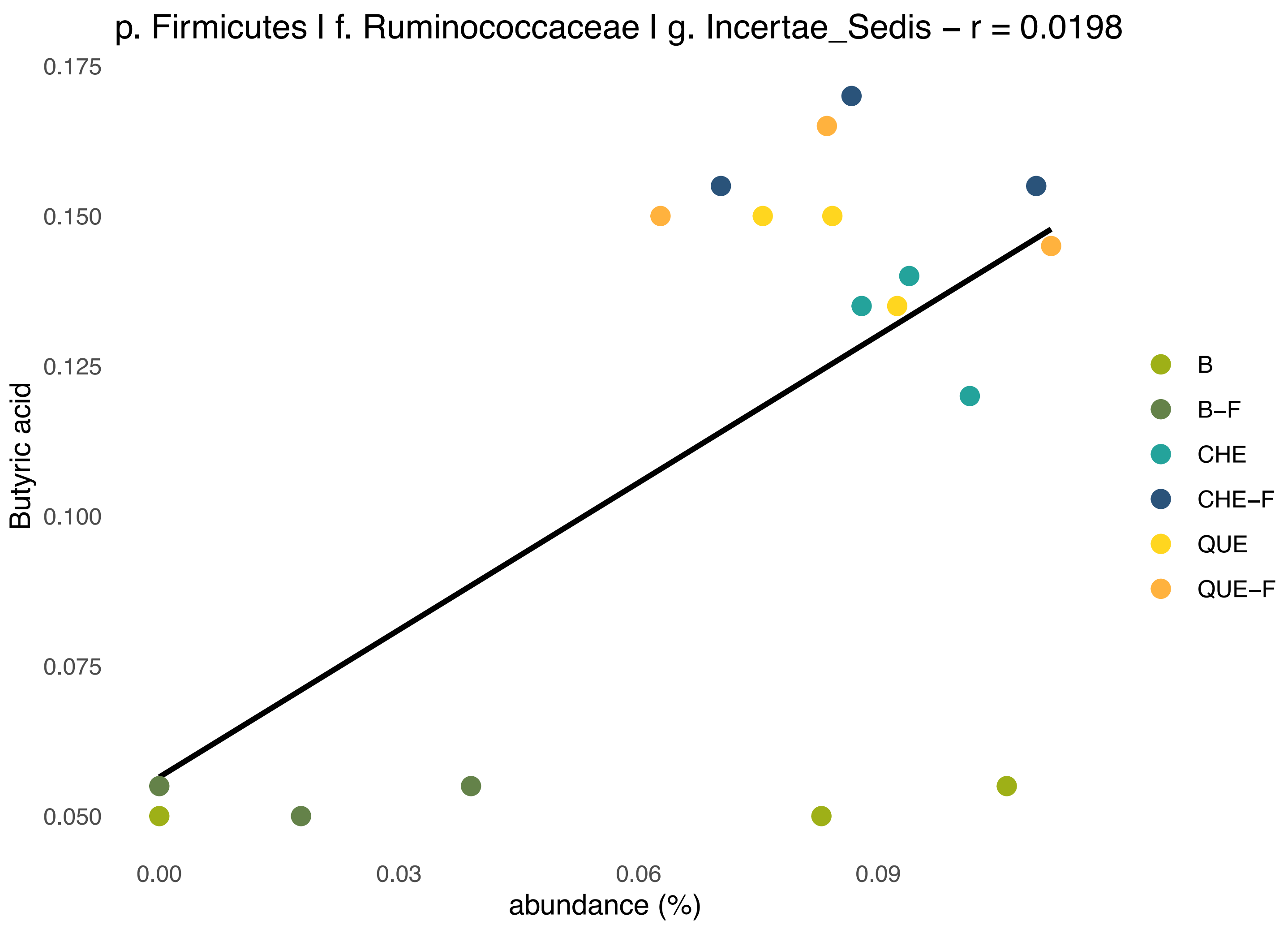
p. Firmicutes l f. Ruminococcaceae l g. Incertae_Sedis – r = 0.0198
Butyric acid
0.175
0.150
0.125
0.100
0.075
0.050
0.00
0.03
0.06
0.09
abundance (%)
B
B–F
CHE
CHE–F
QUE
QUE–F

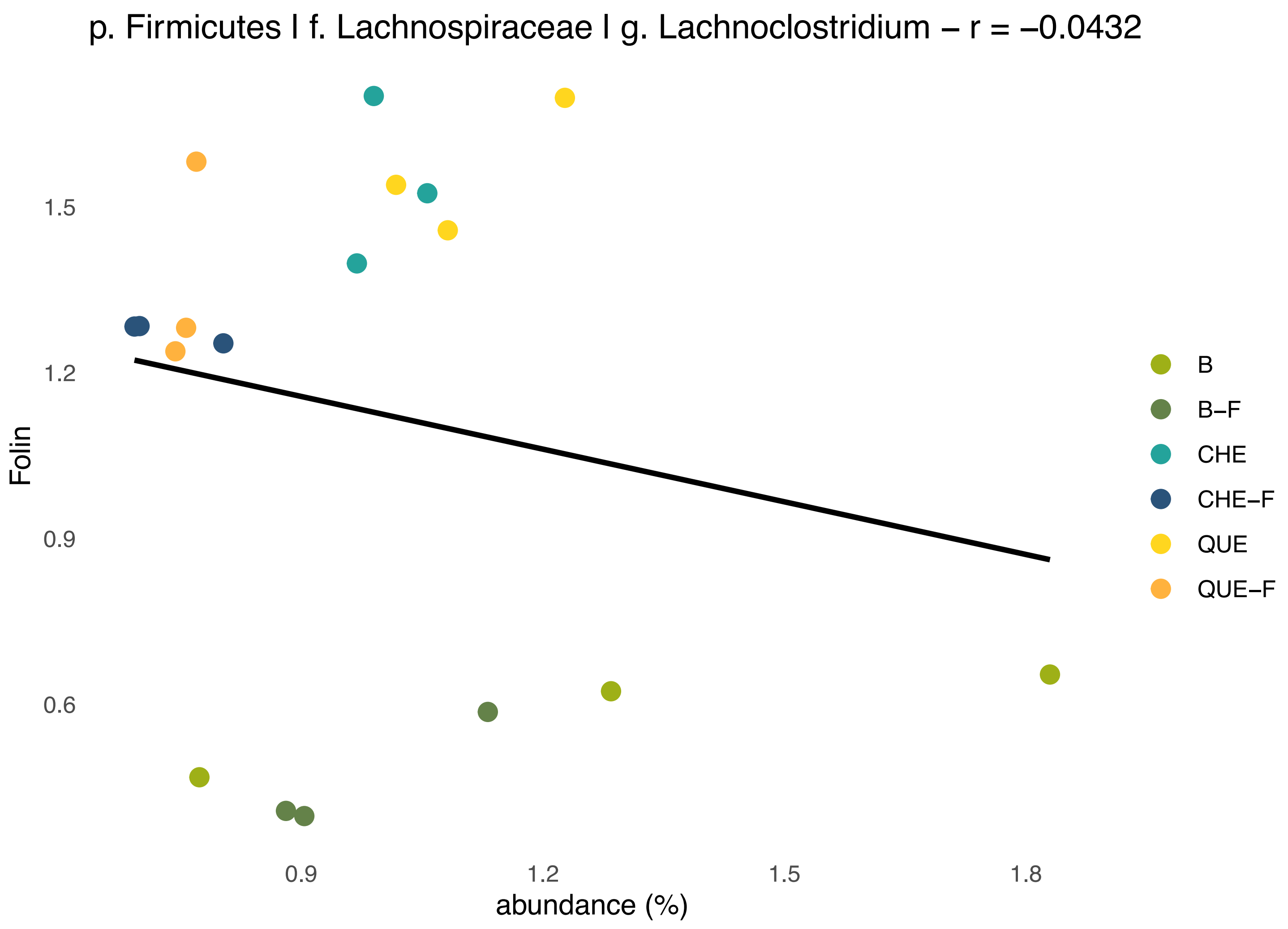
p. Firmicutes I f. Lachnospiraceae I g. Lachnoclostridium – r = –0.0432
1.5
1.2
0.9
0.6
Folin
0.9
1.2
1.5
1.8
abundance (%)
B
B–F
CHE
CHE–F
QUE
QUE–F

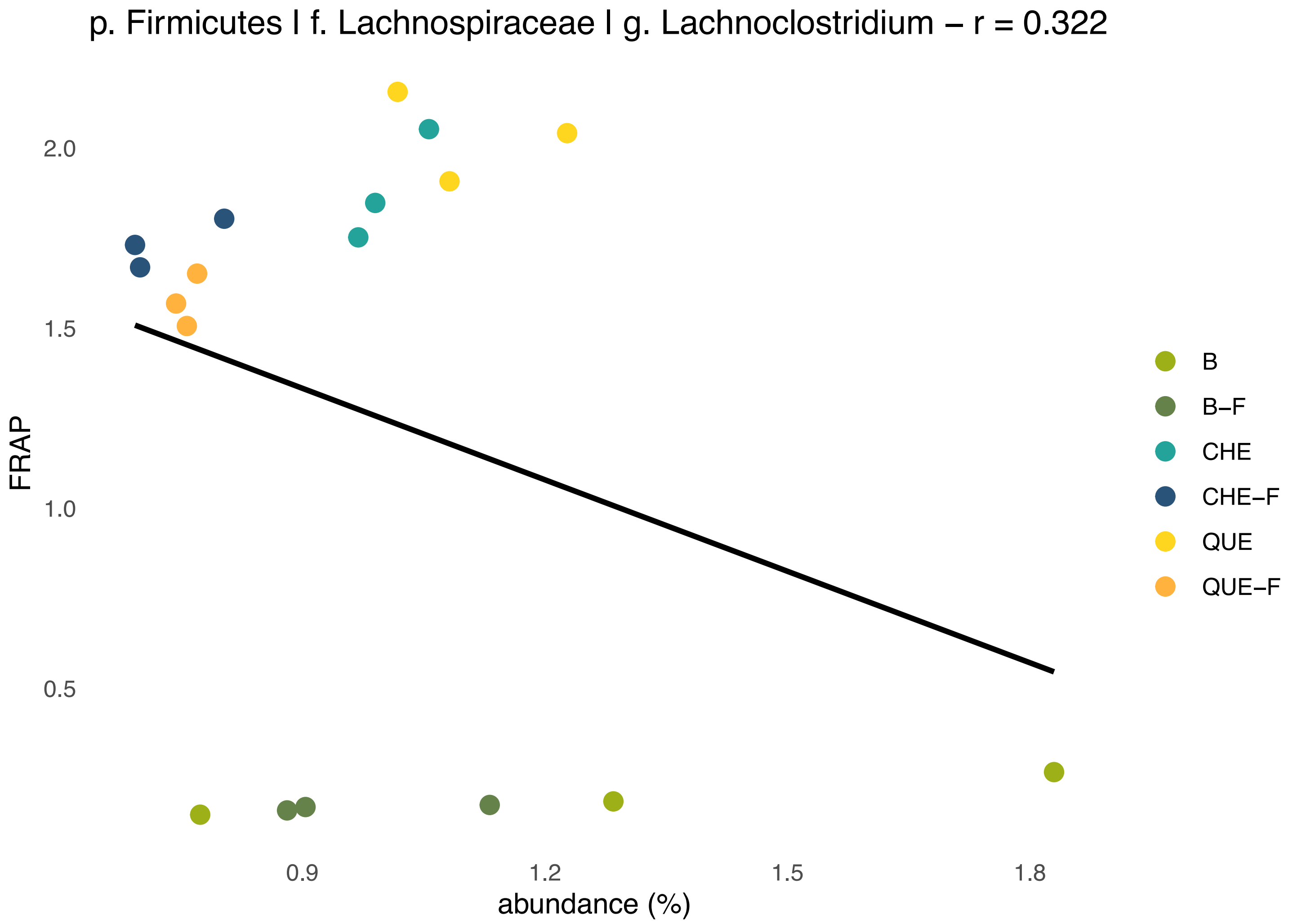
p. Firmicutes I f. Lachnospiraceae I g. Lachnoclostridium – r = 0.322
FRAP
2.0
1.5
1.0
0.5
0.9
1.2
1.5
1.8
abundance (%)
B
B–F
CHE
CHE–F
QUE
QUE–F

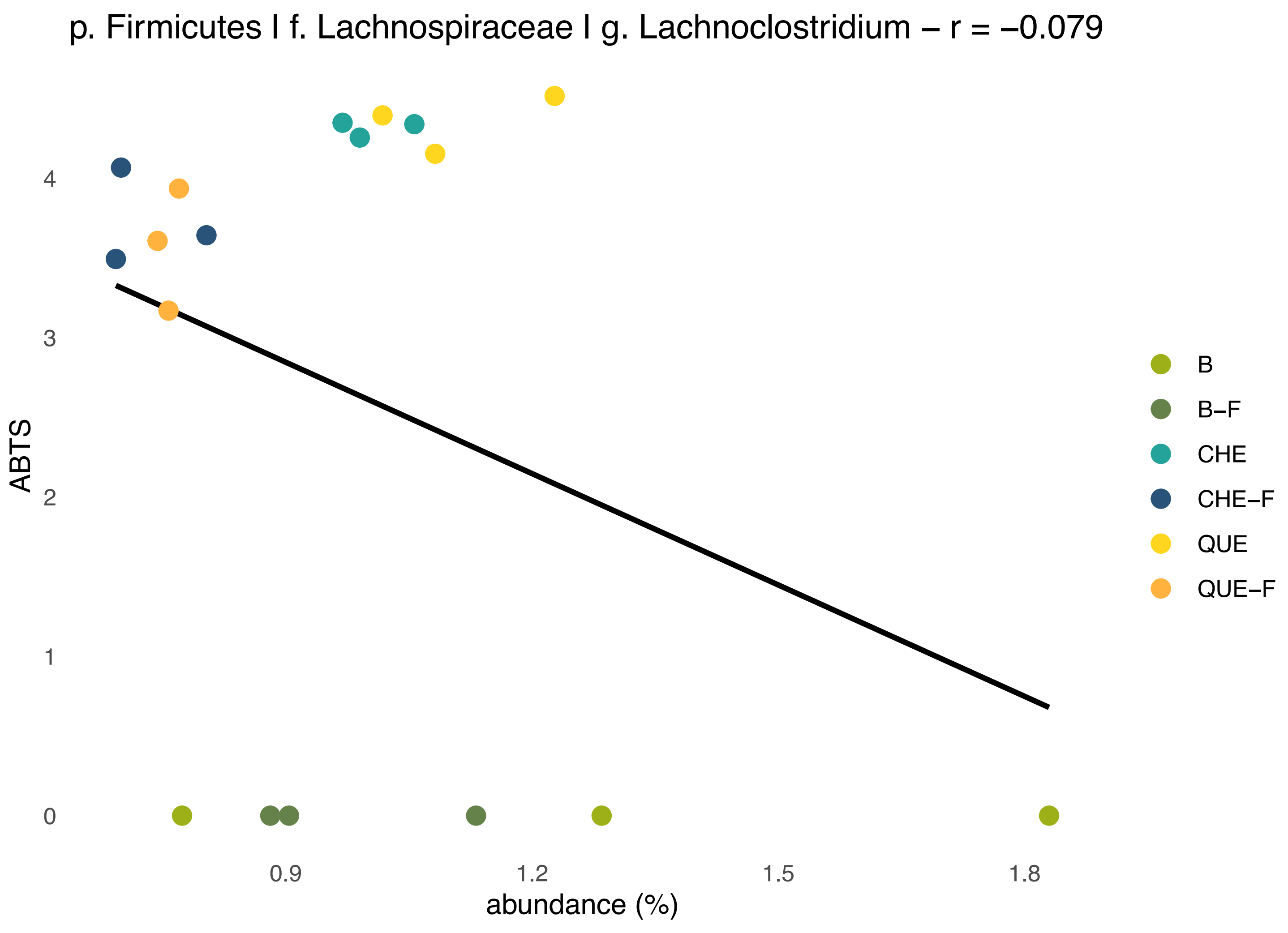
p. Firmicutes | f. Lachnospiraceae | g. Lachnoclostridium – r = –0.079
ABTS
4
3
2
1
0
0.9
1.2
1.5
1.8
abundance (%)
B
B–F
CHE
CHE–F
QUE
QUE–F

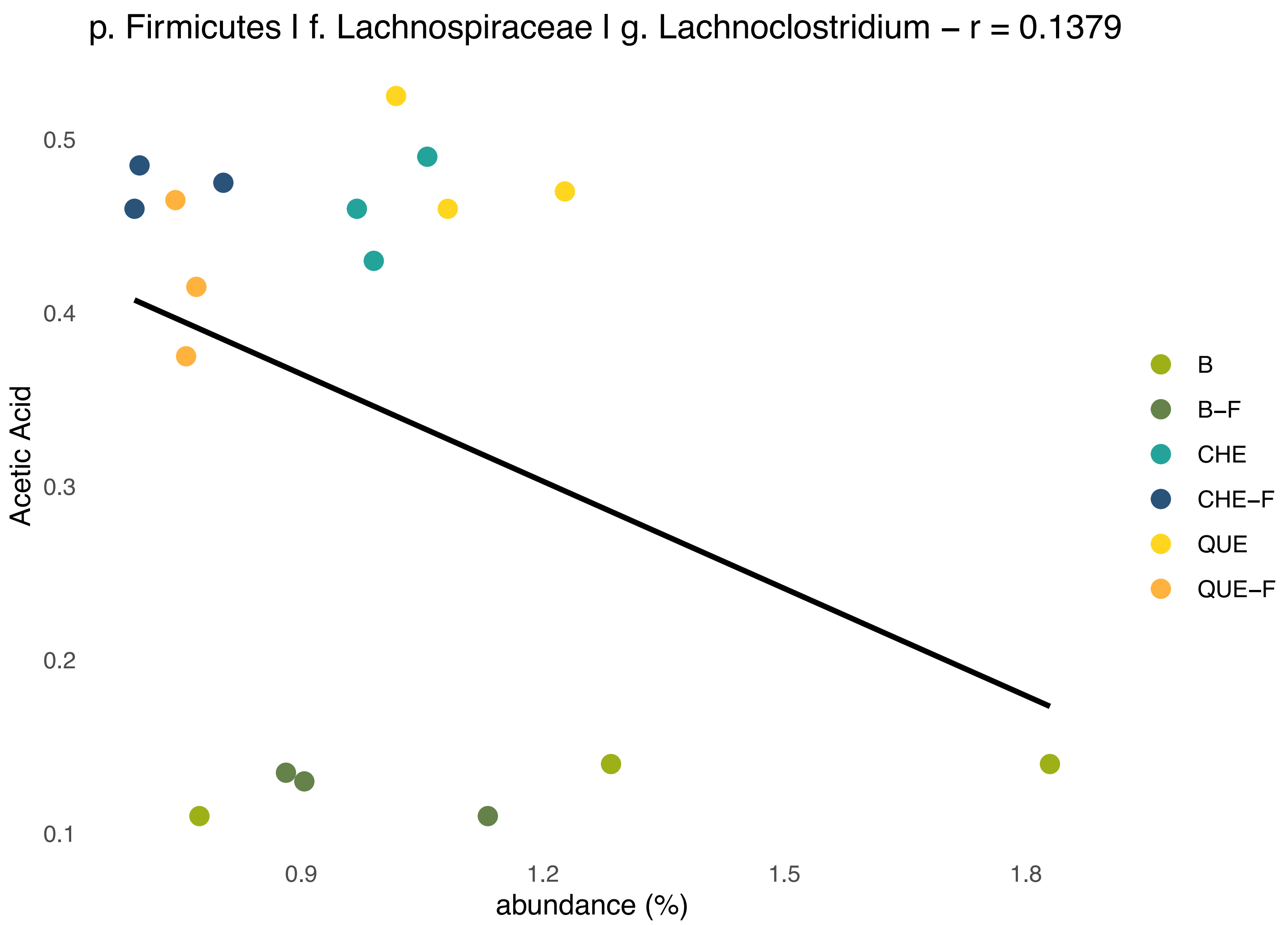
p. Firmicutes I f. Lachnospiraceae I g. Lachnoclostridium – r = 0.1379
Acetic Acid
0.5
0.4
0.3
0.2
0.1
0.9
1.2
1.5
1.8
abundance (%)
B
B–F
CHE
CHE–F
QUE
QUE–F

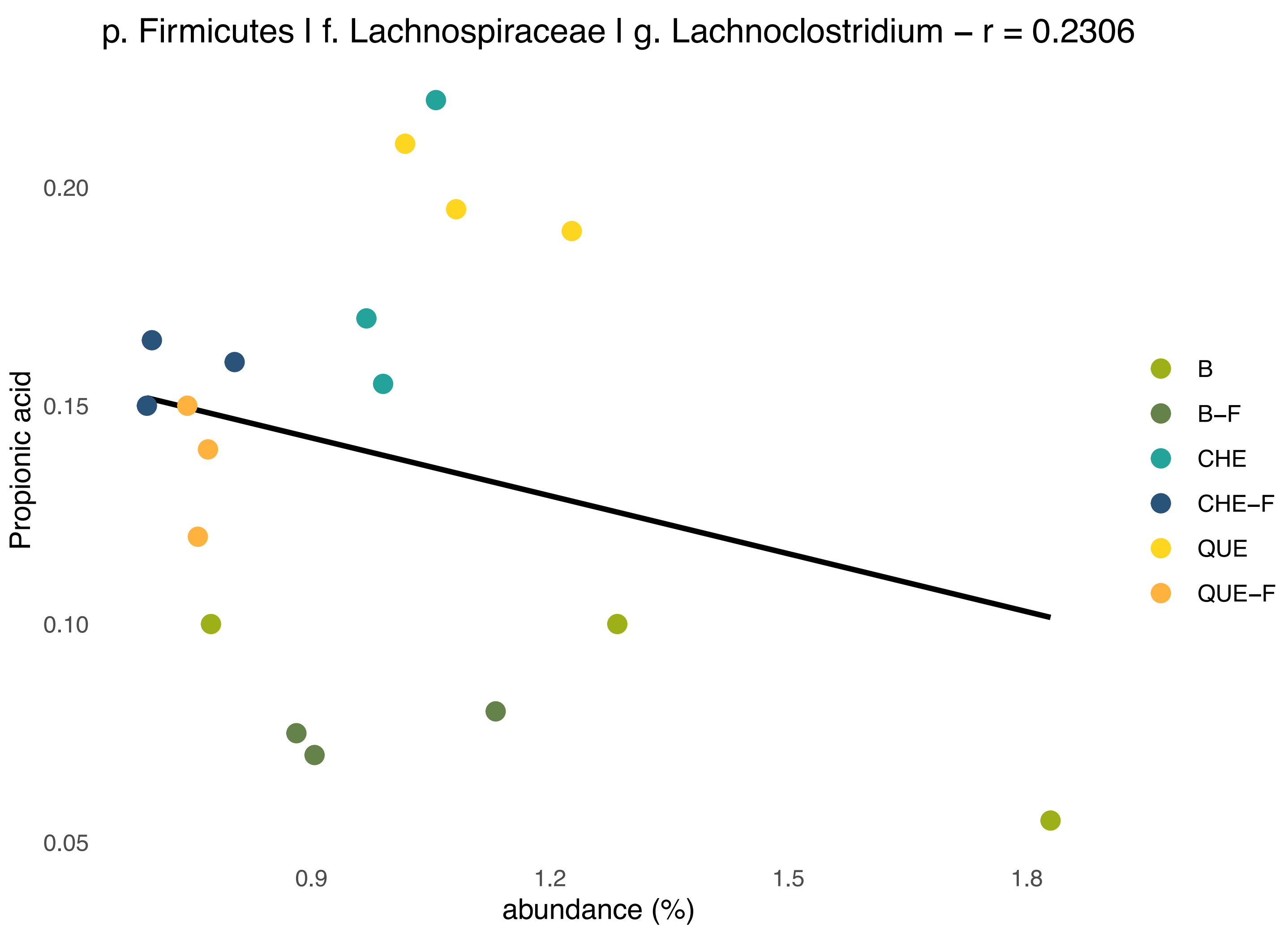

p. Firmicutes I f. Lachnospiraceae I g. Lachnoclostridium – r = 0.2306
Propionic acid
0.20
0.15
0.10
0.05
0.9
1.2
1.5
1.8
abundance (%)
B
B–F
CHE
CHE–F
QUE
QUE–F

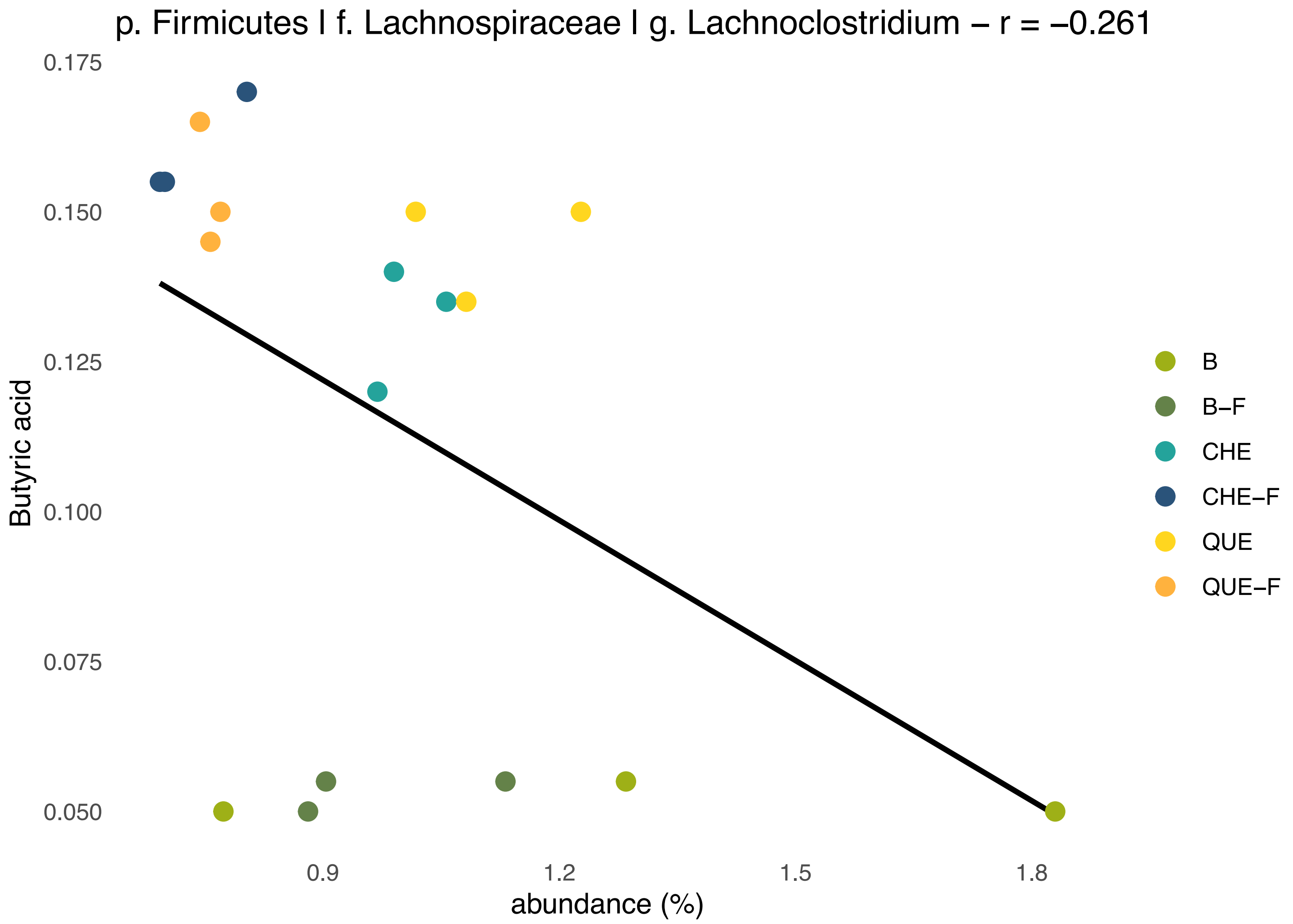

p. Firmicutes I f. Lachnospiraceae I g. Lachnoclostridium – r = –0.261
0.175
0.150
0.125
0.100
0.075
0.050
Butyric acid
0.9
1.2
1.5
1.8
abundance (%)
B
B–F
CHE
CHE–F
QUE
QUE–F

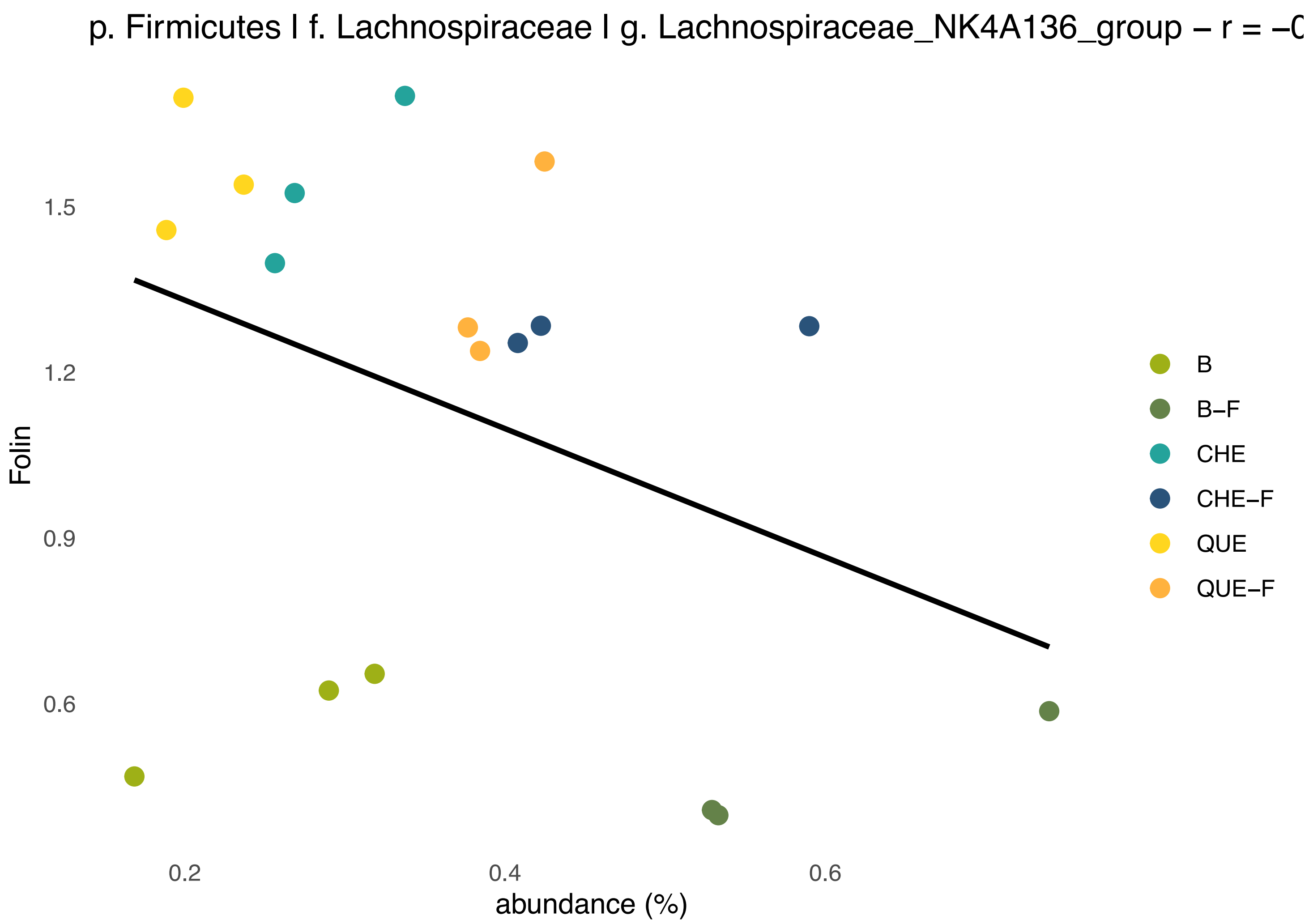

p. Firmicutes l f. Lachnospiraceae l g. Lachnospiraceae_NK4A136_group – r = –0
Folin
1.5
1.2
0.9
0.6
0.2
0.4
0.6
abundance (%)
B
B–F
CHE
CHE–F
QUE
QUE–F

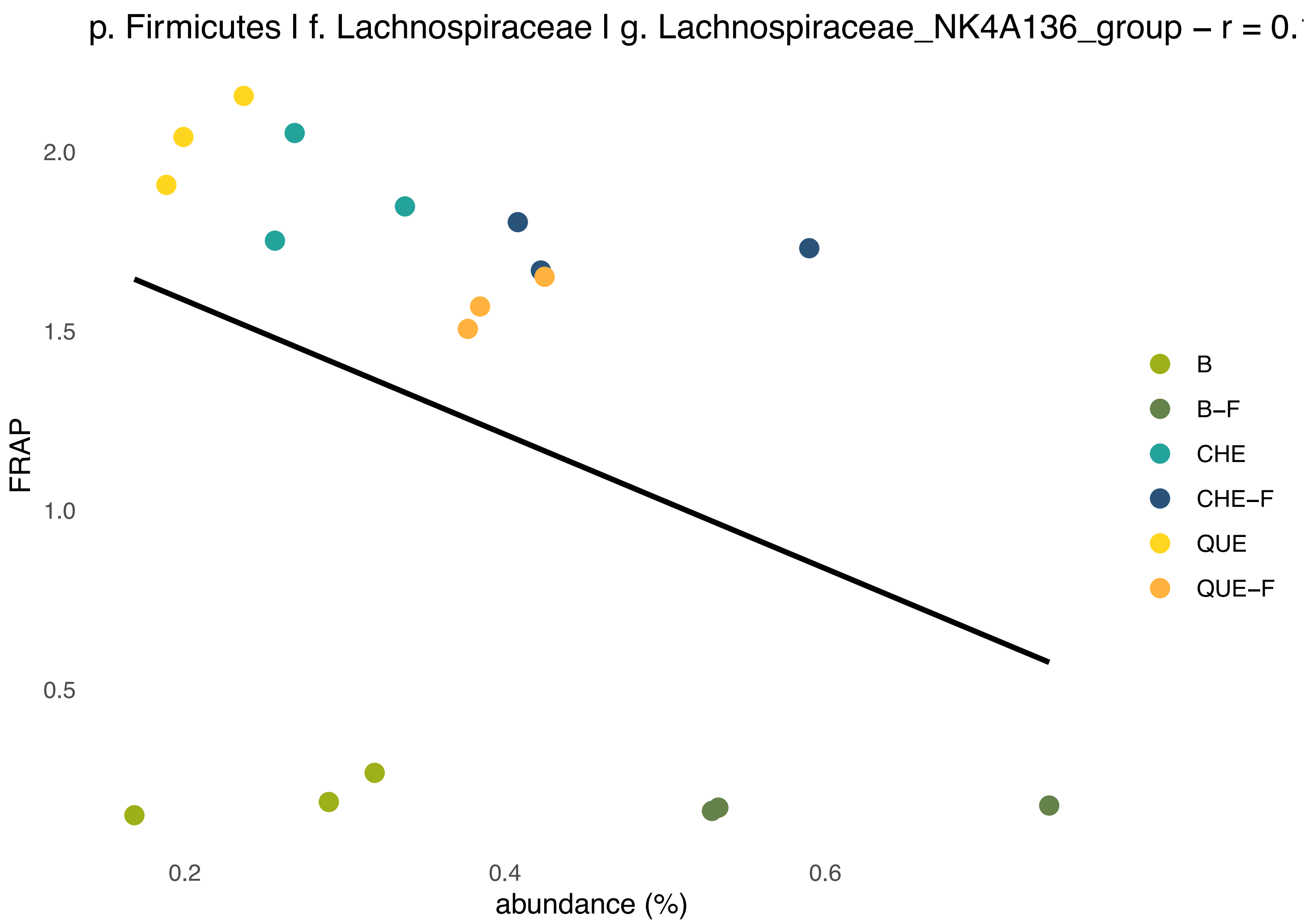
p. Firmicutes I f. Lachnospiraceae I g. Lachnospiraceae_NK4A136_group – r = 0.
2.0
1.5
1.0
0.5
FRAP
0.2
0.4
0.6
abundance (%)
B
B–F
CHE
CHE–F
QUE
QUE–F

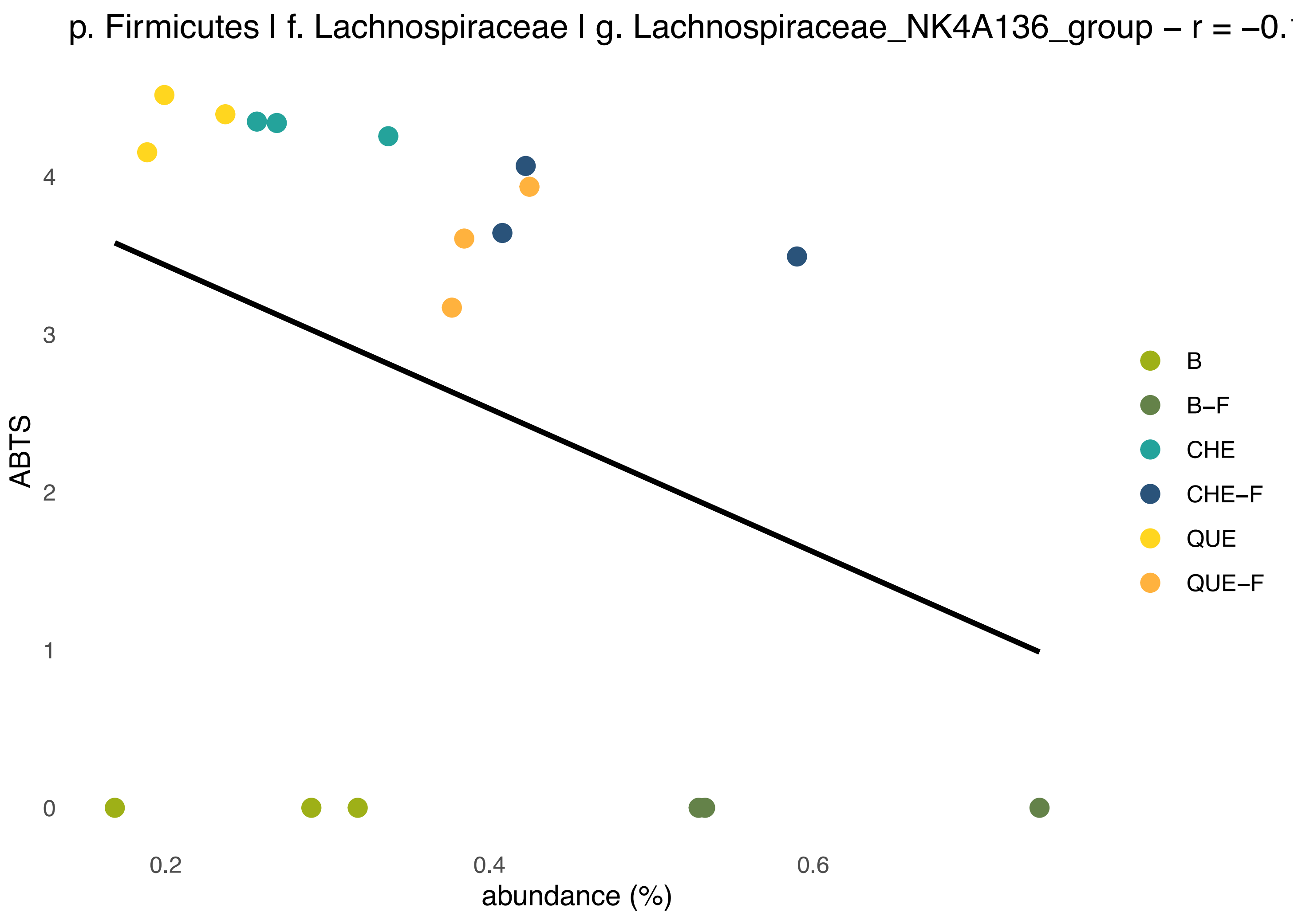

p. Firmicutes l f. Lachnospiraceae l g. Lachnospiraceae_NK4A136_group – r = –0.
ABTS
0
1
2
3
4
0.2
0.4
0.6
abundance (%)
B
B–F
CHE
CHE–F
QUE
QUE–F

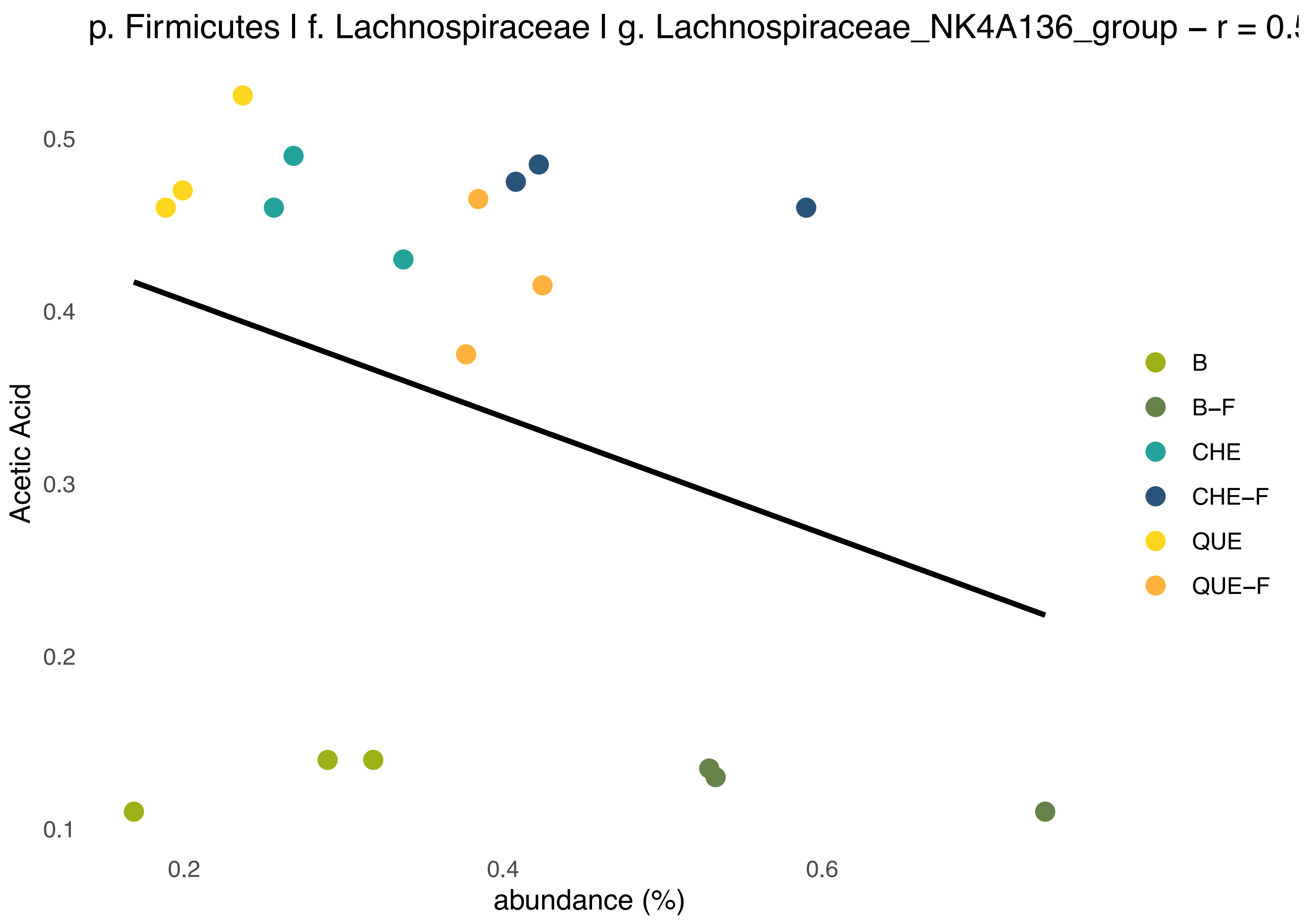

p. Firmicutes I f. Lachnospiraceae I g. Lachnospiraceae_NK4A136_group – r = 0.
Acetic Acid
0.5
0.4
0.3
0.2
0.1
0.2
0.4
0.6
abundance (%)
B
B–F
CHE
CHE–F
QUE
QUE–F

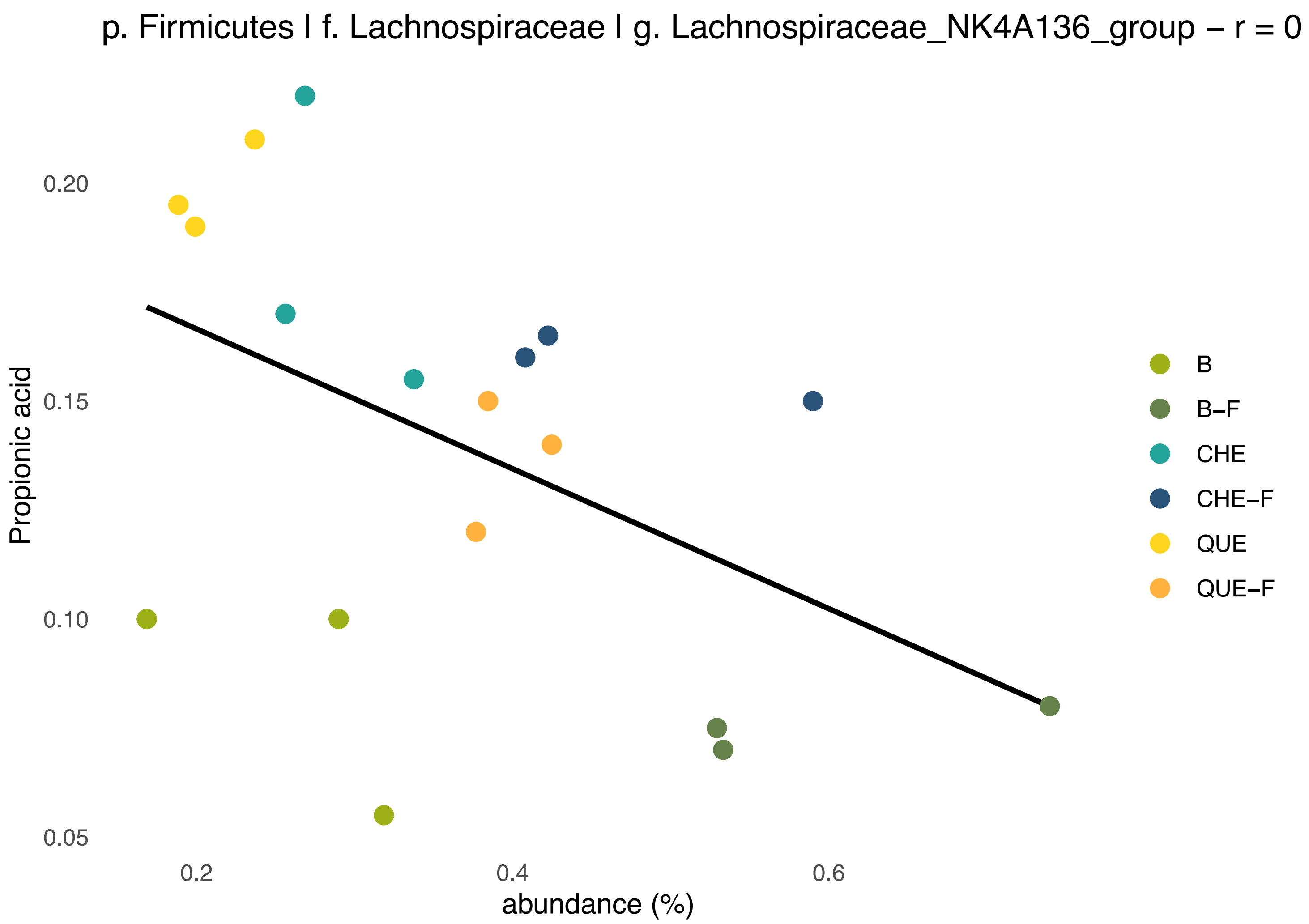
p. Firmicutes l f. Lachnospiraceae l g. Lachnospiraceae_NK4A136_group − r = 0
Propionic acid
0.20
0.15
0.10
0.05
0.2
0.4
0.6
abundance (%)
B
B−F
CHE
CHE−F
QUE
QUE−F

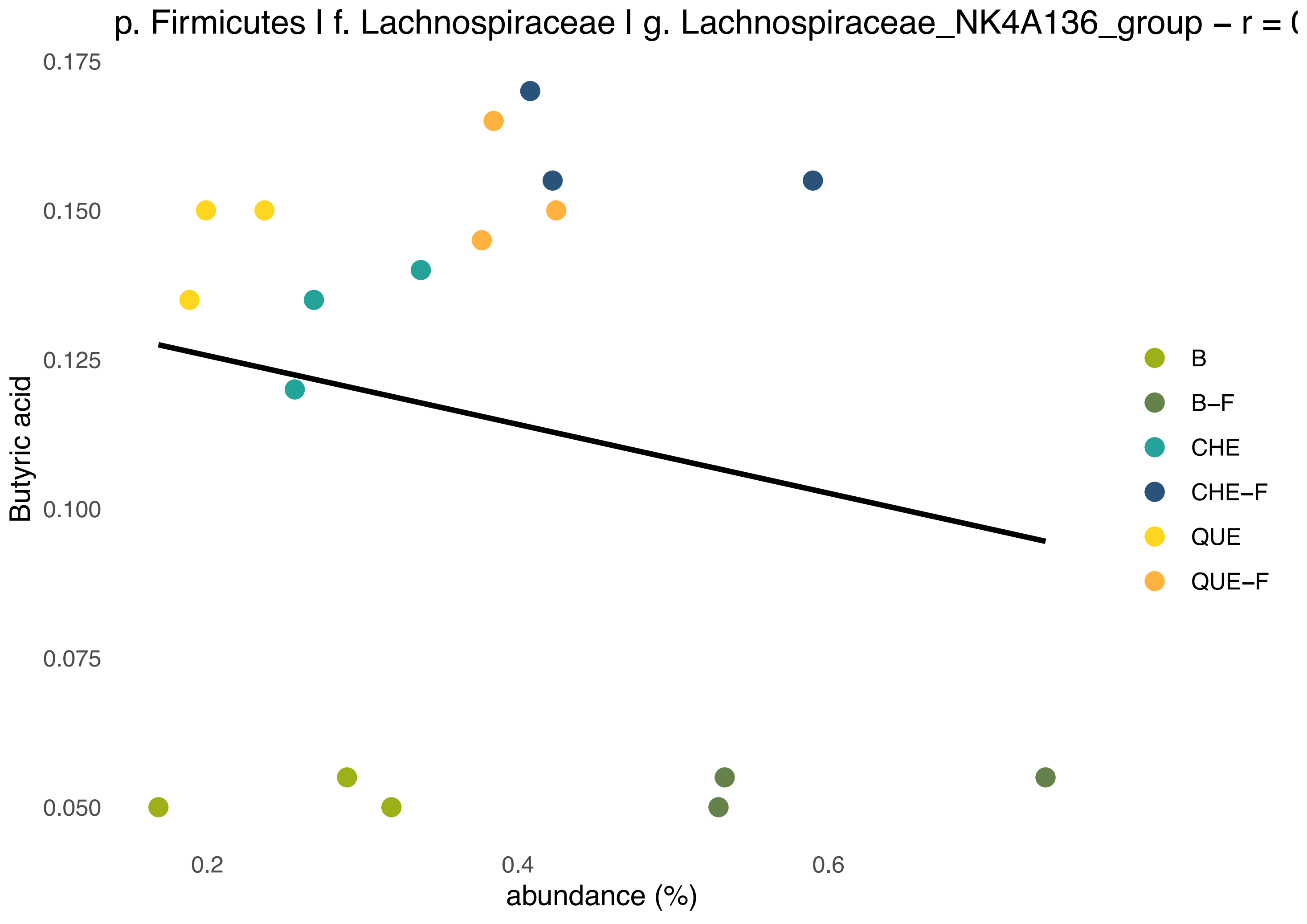

p. Firmicutes l f. Lachnospiraceae l g. Lachnospiraceae_NK4A136_group – r = (
Butyric acid
0.175
0.150
0.125
0.100
0.075
0.050
0.2
0.4
0.6
abundance (%)
B
B–F
CHE
CHE–F
QUE
QUE–F

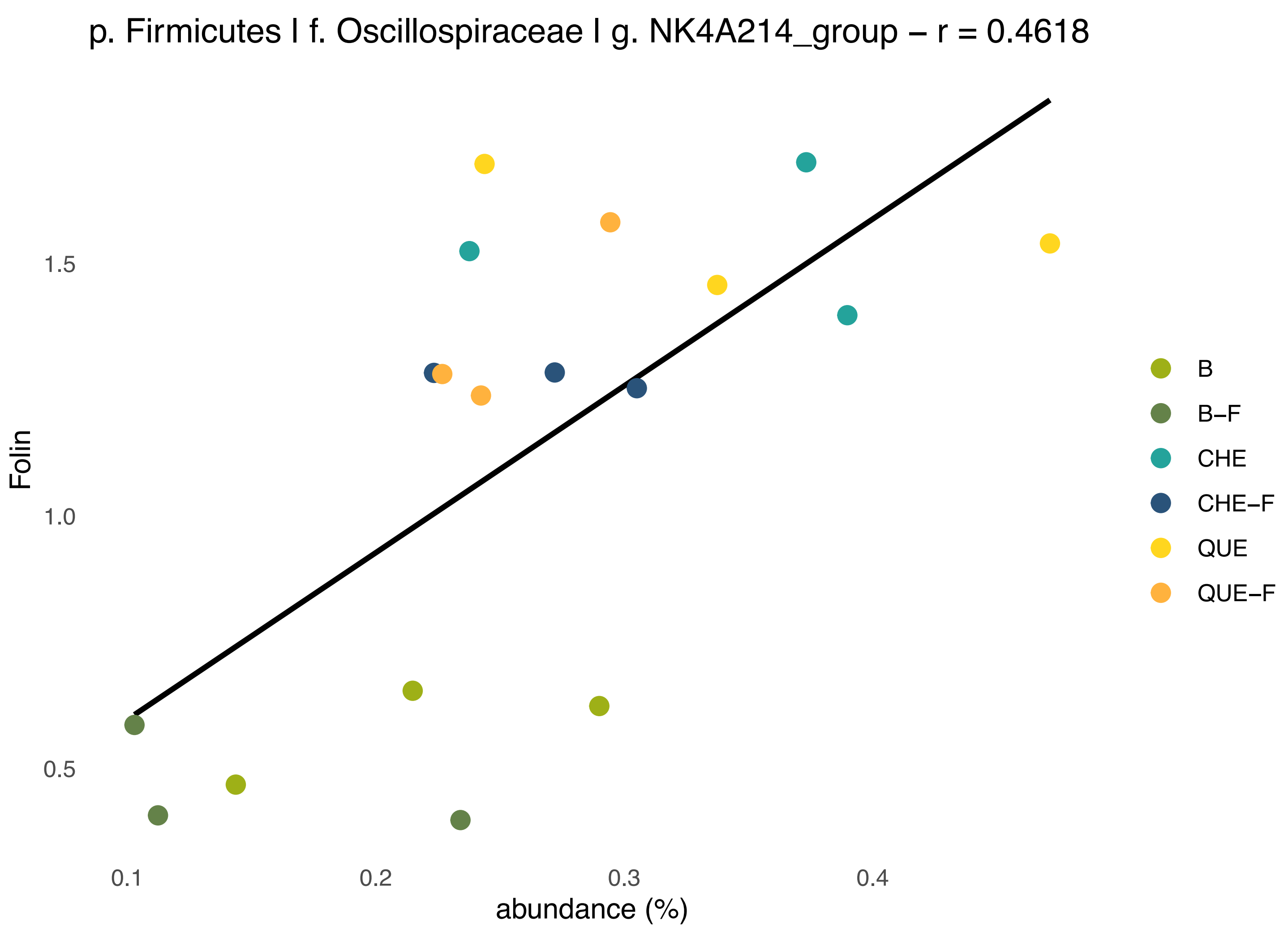

p. Firmicutes I f. Oscillospiraceae I g. NK4A214_group – r = 0.4618
Folin
1.5
1.0
0.5
0.1
0.2
0.3
0.4
abundance (%)
B
B–F
CHE
CHE–F
QUE
QUE–F

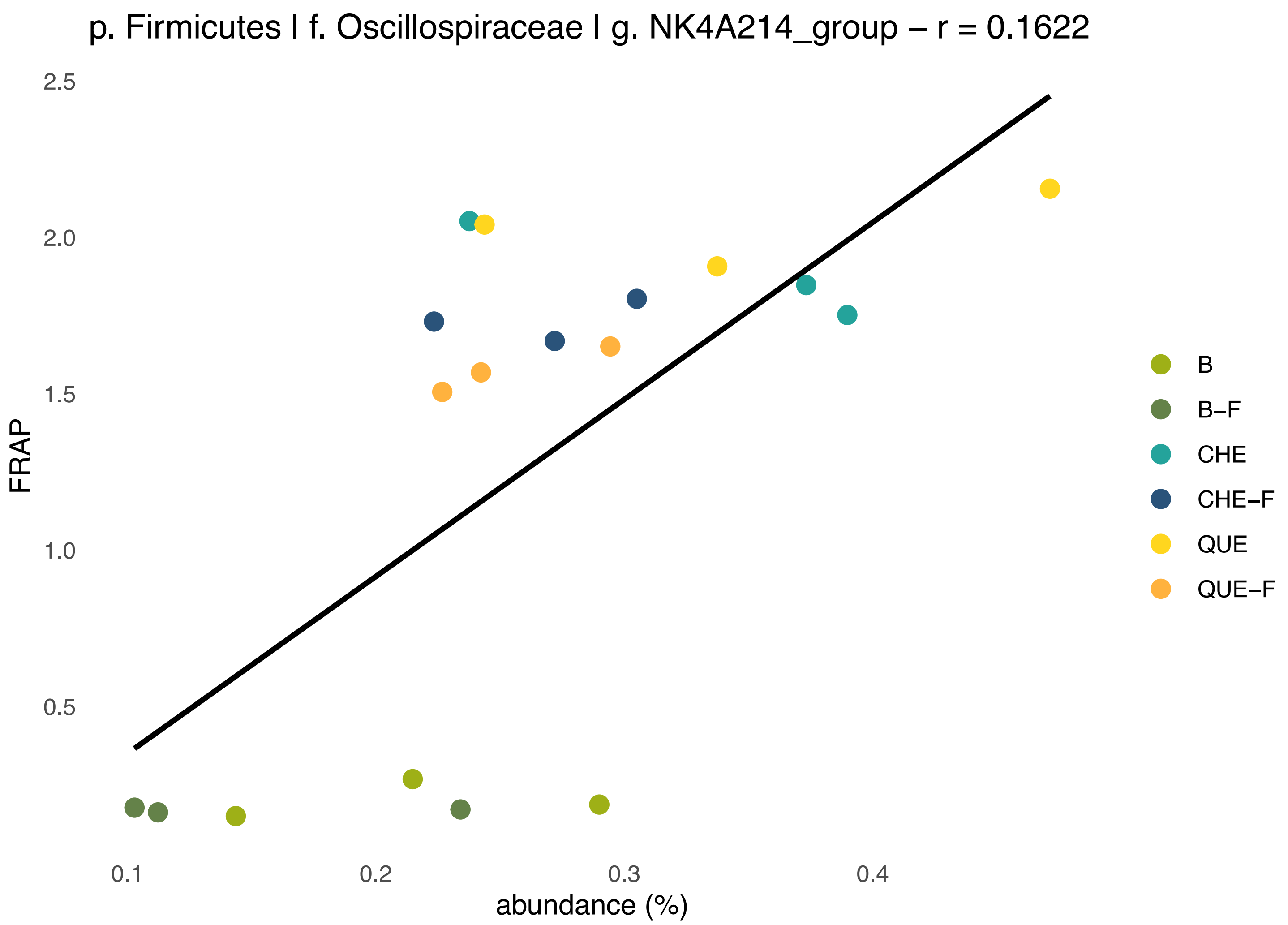

p. Firmicutes l f. Oscillospiraceae l g. NK4A214_group – r = 0.1622
FRAP
2.5
2.0
1.5
1.0
0.5
0.1
0.2
0.3
0.4
abundance (%)
B
B–F
CHE
CHE–F
QUE
QUE–F

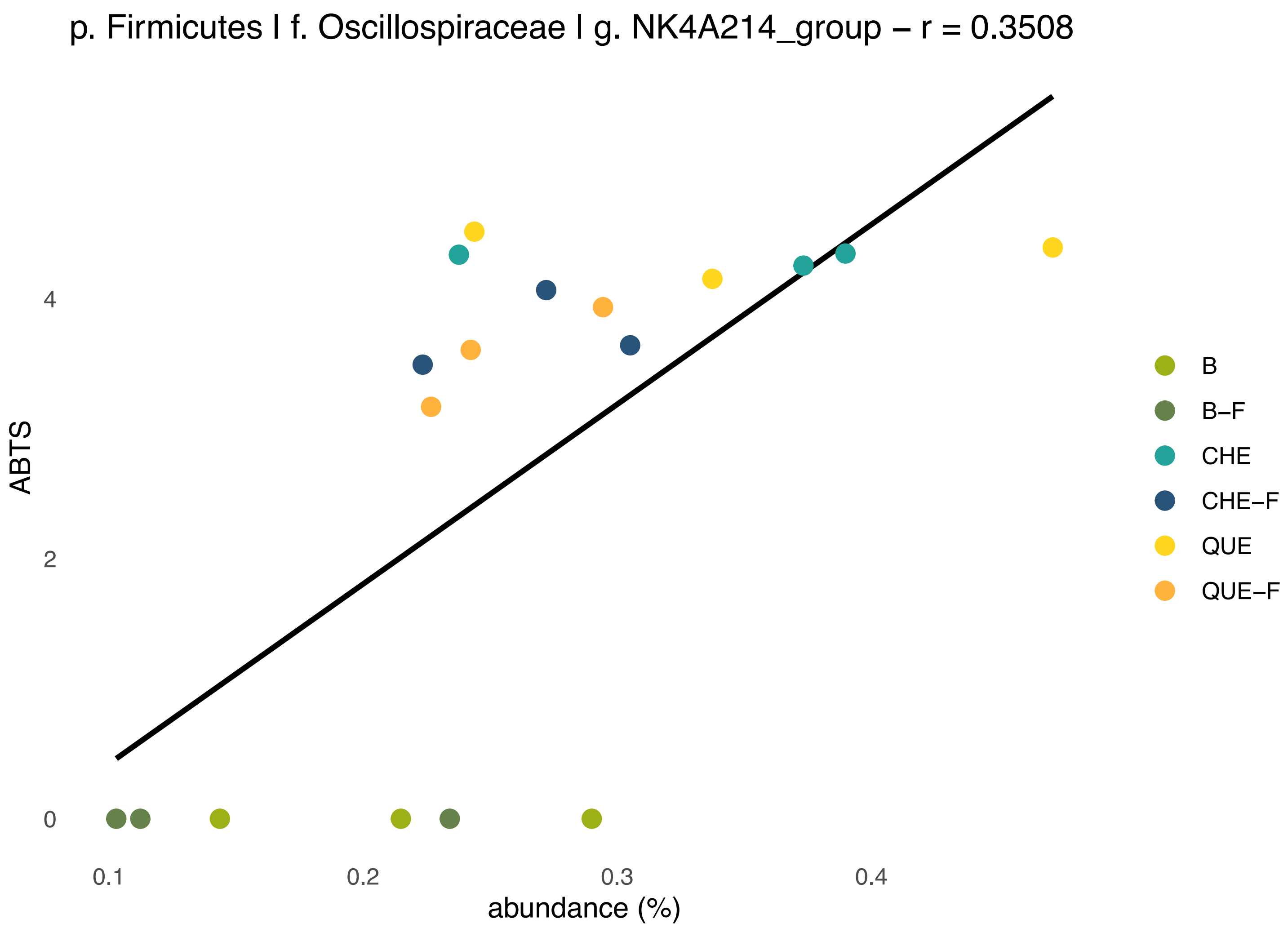

p. Firmicutes I f. Oscillospiraceae I g. NK4A214_group – r = 0.3508
ABTS
4
2
0
0.1
0.2
0.3
0.4
abundance (%)
B
B–F
CHE
CHE–F
QUE
QUE–F

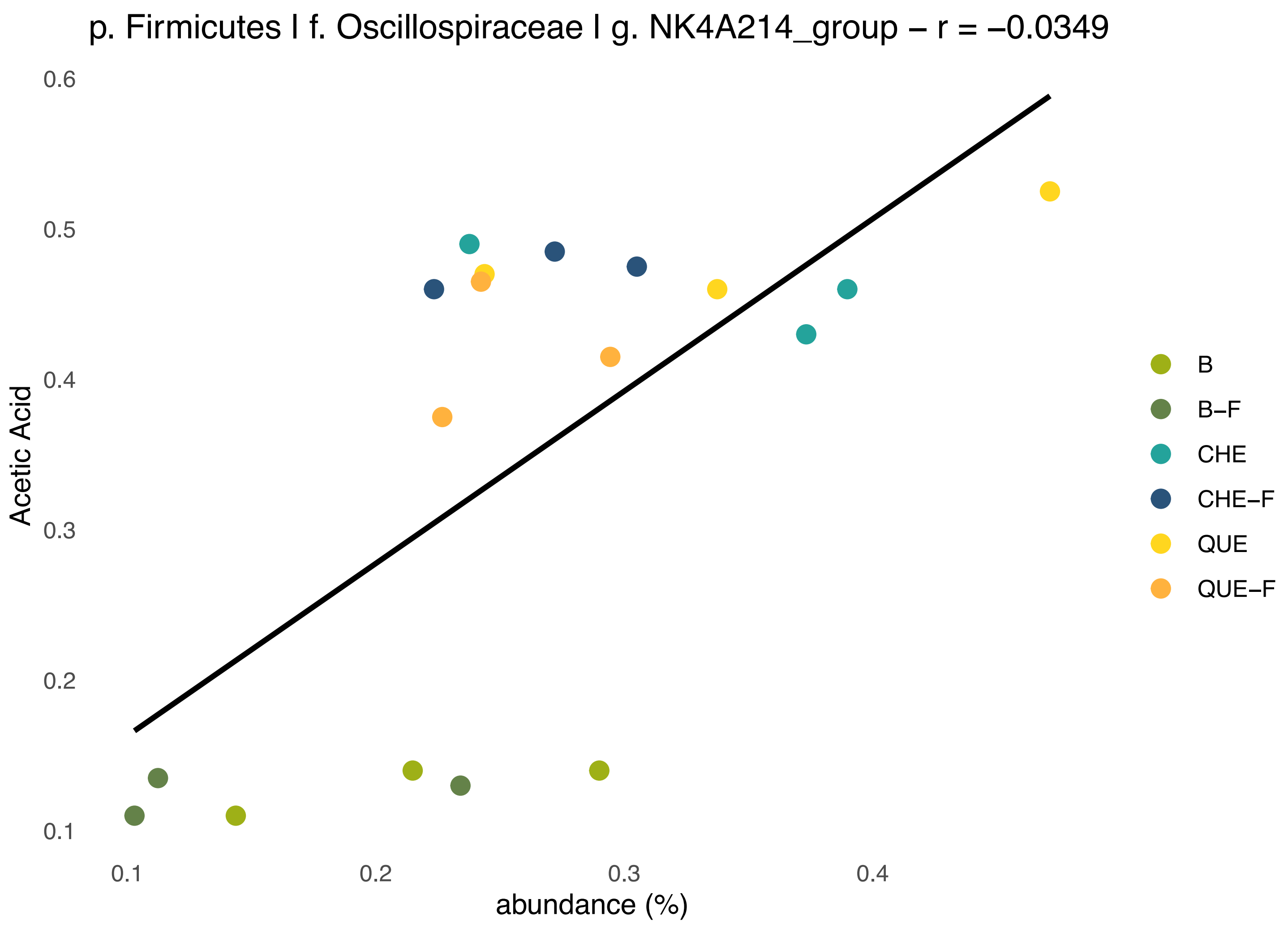

p. Firmicutes l f. Oscillospiraceae l g. NK4A214_group − r = −0.0349
Acetic Acid
abundance (%)
0.1
0.2
0.3
0.4
0.5
0.6
B
B−F
CHE
CHE−F
QUE
QUE−F

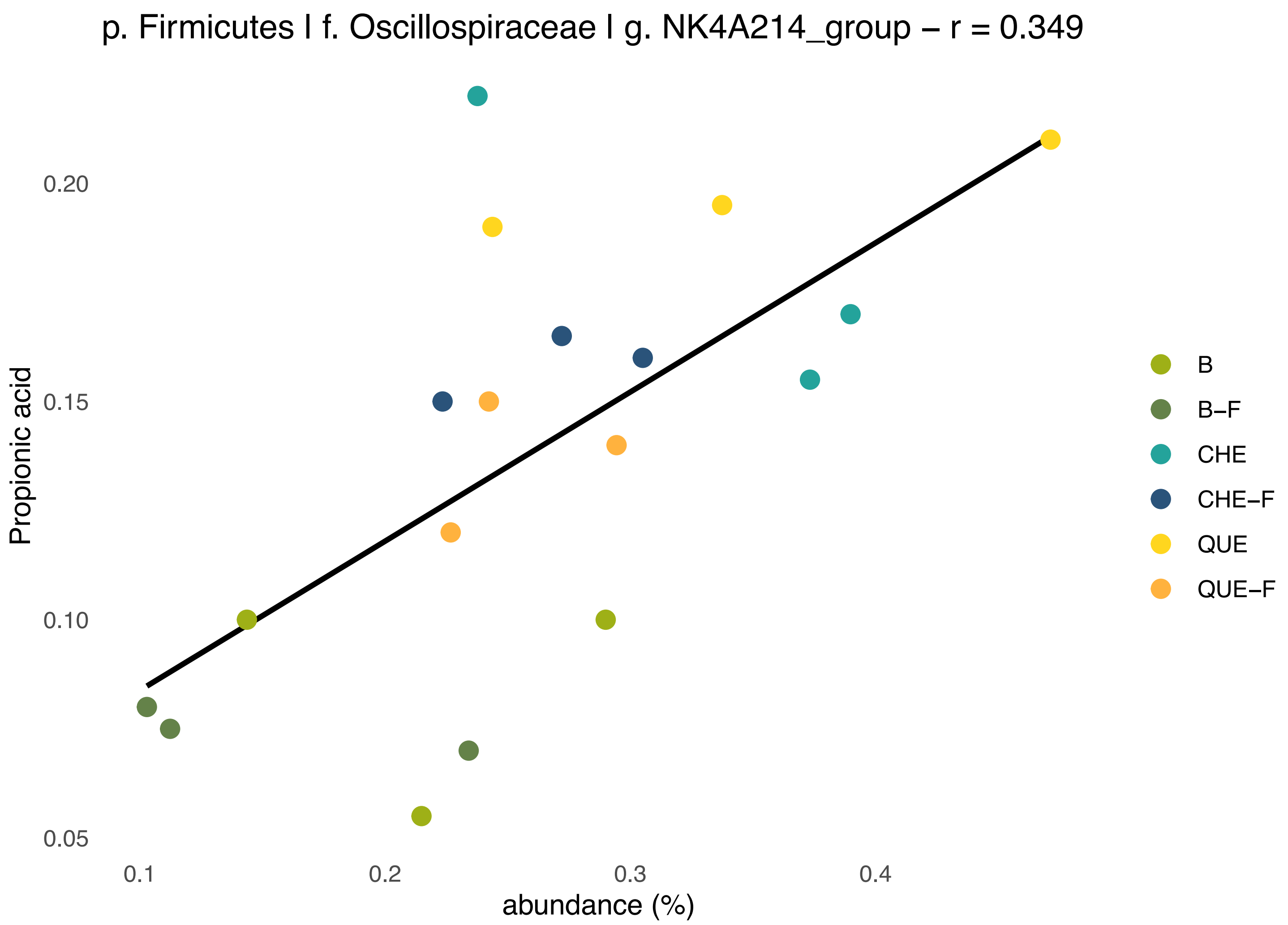
p. Firmicutes I f. Oscillospiraceae I g. NK4A214_group − r = 0.349
Propionic acid
0.20
0.15
0.10
0.05
0.1
0.2
0.3
0.4
abundance (%)
B
B−F
CHE
CHE−F
QUE
QUE−F

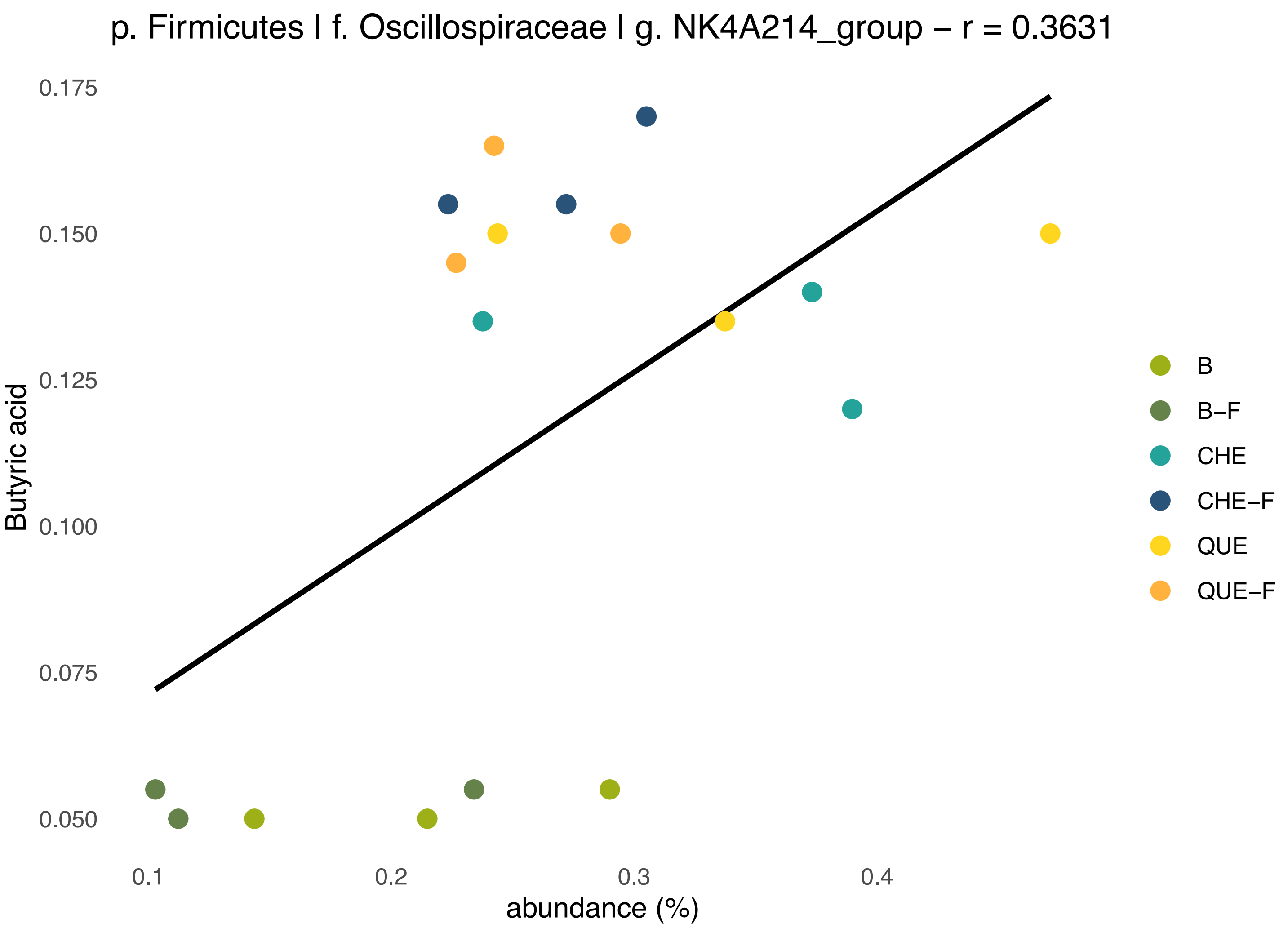
p. Firmicutes I f. Oscillospiraceae I g. NK4A214_group – r = 0.3631
0.175
0.150
0.125
0.100
0.075
0.050
Butyric acid
0.1
0.2
0.3
0.4
abundance (%)
B
B–F
CHE
CHE–F
QUE
QUE–F

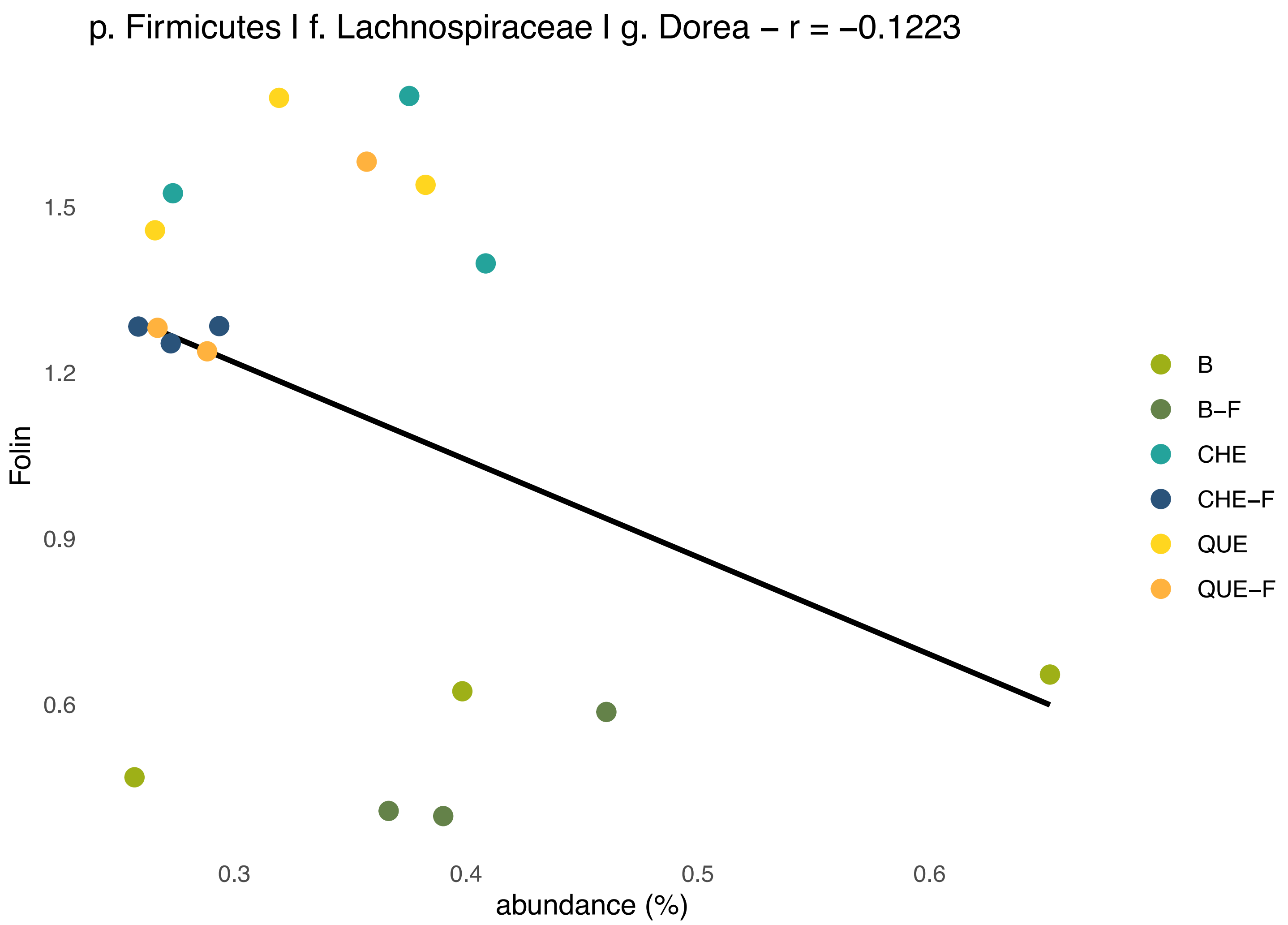
p. Firmicutes I f. Lachnospiraceae I g. Dorea – r = −0.1223
Folin
1.5
1.2
0.9
0.6
0.3
0.4
0.5
0.6
abundance (%)
B
B−F
CHE
CHE−F
QUE
QUE−F

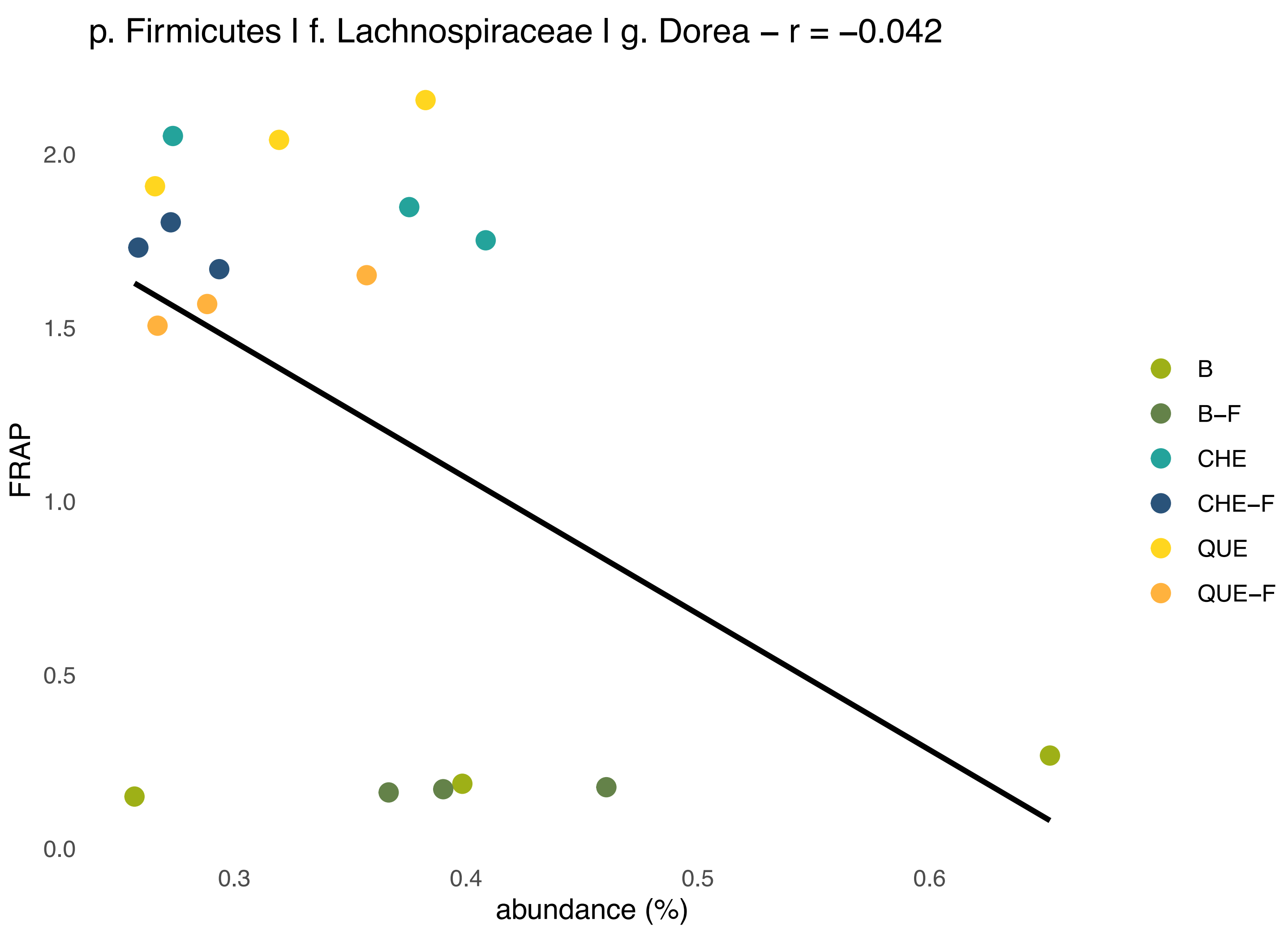

p. Firmicutes I f. Lachnospiraceae I g. Dorea – r = –0.042
FRAP
2.0
1.5
1.0
0.5
0.0
0.3
0.4
0.5
0.6
abundance (%)
B
B–F
CHE
CHE–F
QUE
QUE–F

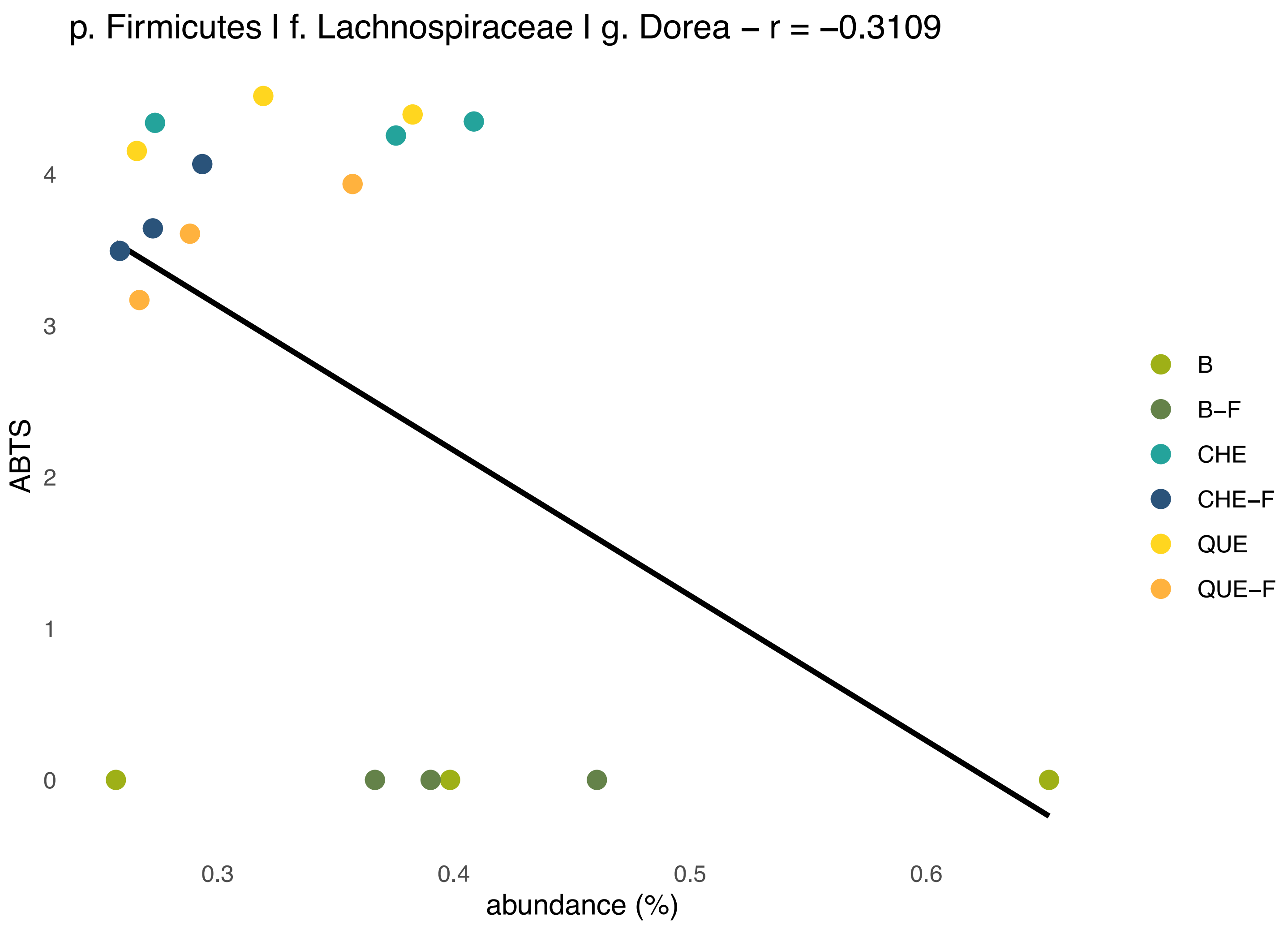
p. Firmicutes I f. Lachnospiraceae I g. Dorea – r = –0.3109
ABTS
0
1
2
3
4
0.3
0.4
0.5
0.6
abundance (%)
B
B–F
CHE
CHE–F
QUE
QUE–F

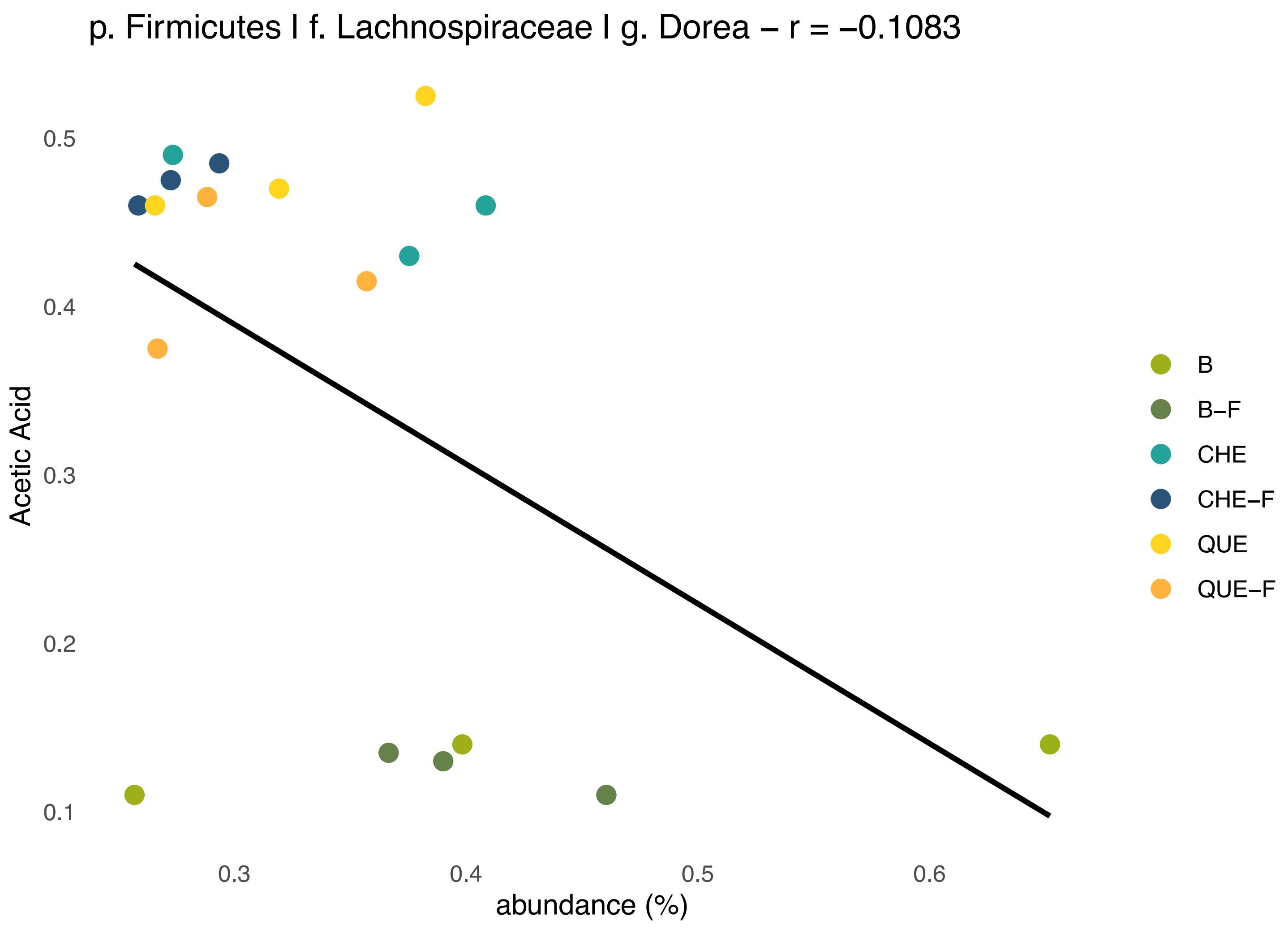

p. Firmicutes I f. Lachnospiraceae I g. Dorea – r = –0.1083
Acetic Acid
0.5
0.4
0.3
0.2
0.1
0.3
0.4
0.5
0.6
abundance (%)
B
B–F
CHE
CHE–F
QUE
QUE–F

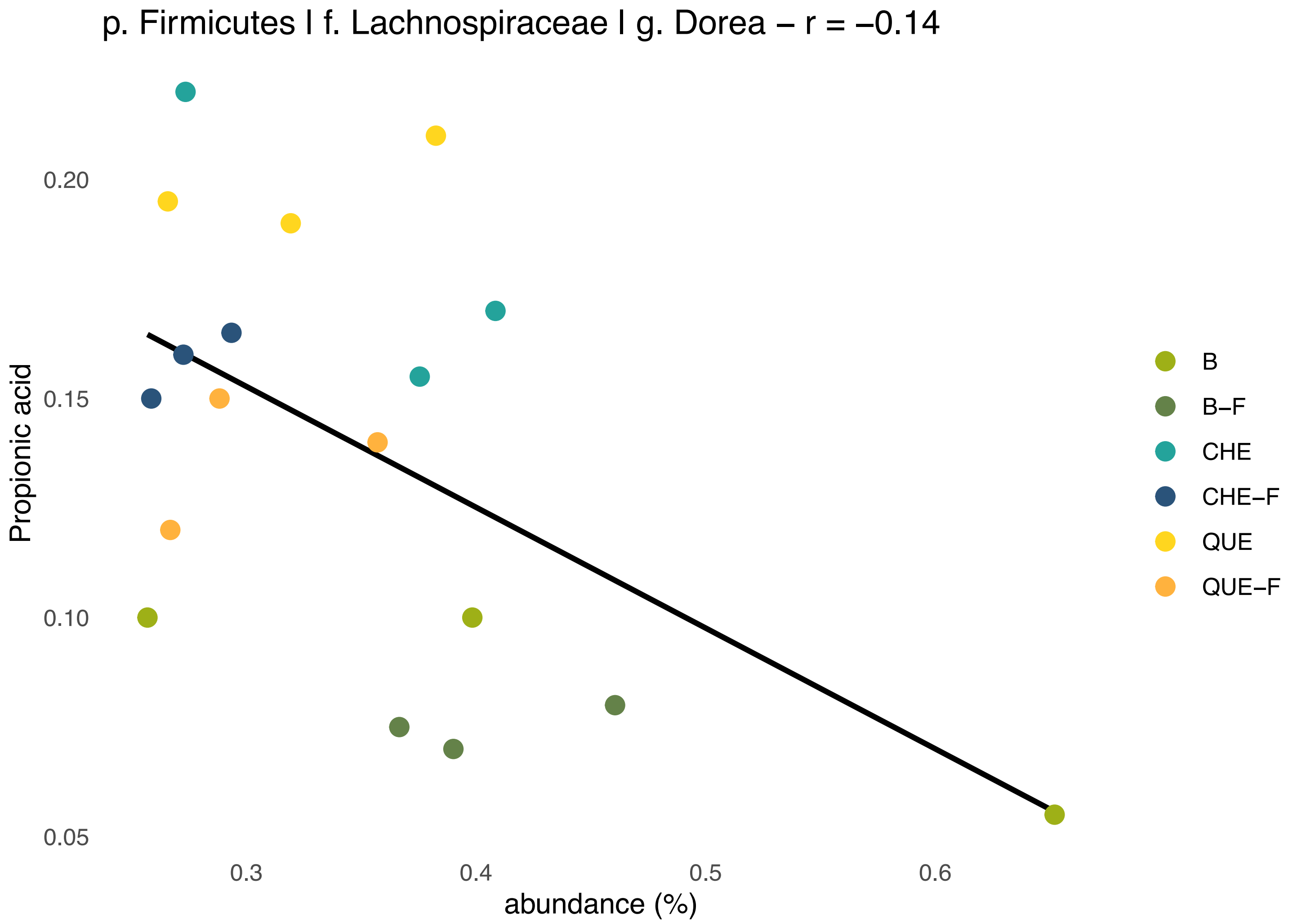

p. Firmicutes | f. Lachnospiraceae | g. Dorea – r = –0.14
Propionic acid
0.20
0.15
0.10
0.05
0.3
0.4
0.5
0.6
abundance (%)
B
B–F
CHE
CHE–F
QUE
QUE–F

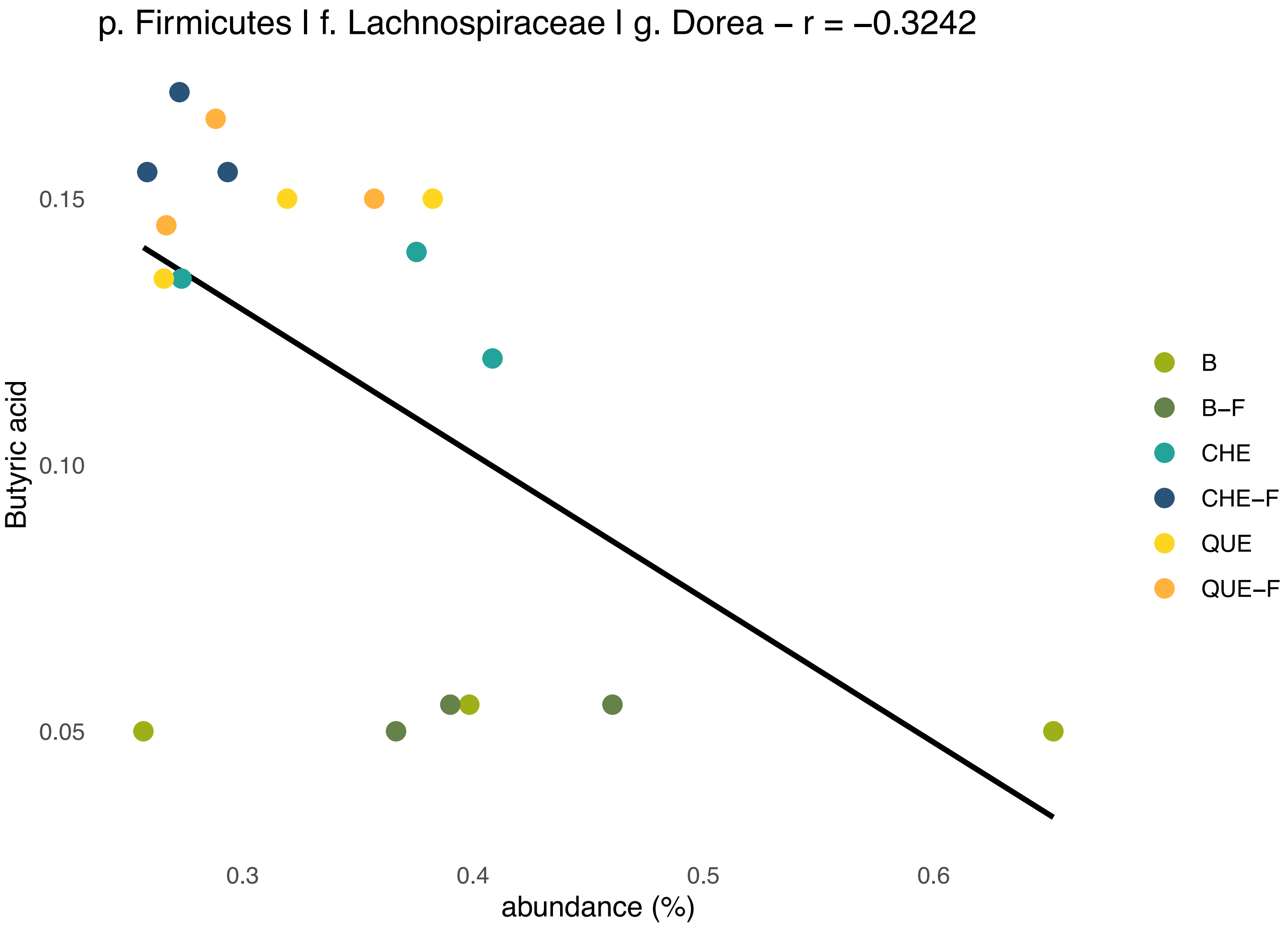

p. Firmicutes l f. Lachnospiraceae l g. Dorea – r = –0.3242
Butyric acid
0.15
0.10
0.05
0.3
0.4
0.5
0.6
abundance (%)
B
B–F
CHE
CHE–F
QUE
QUE–F

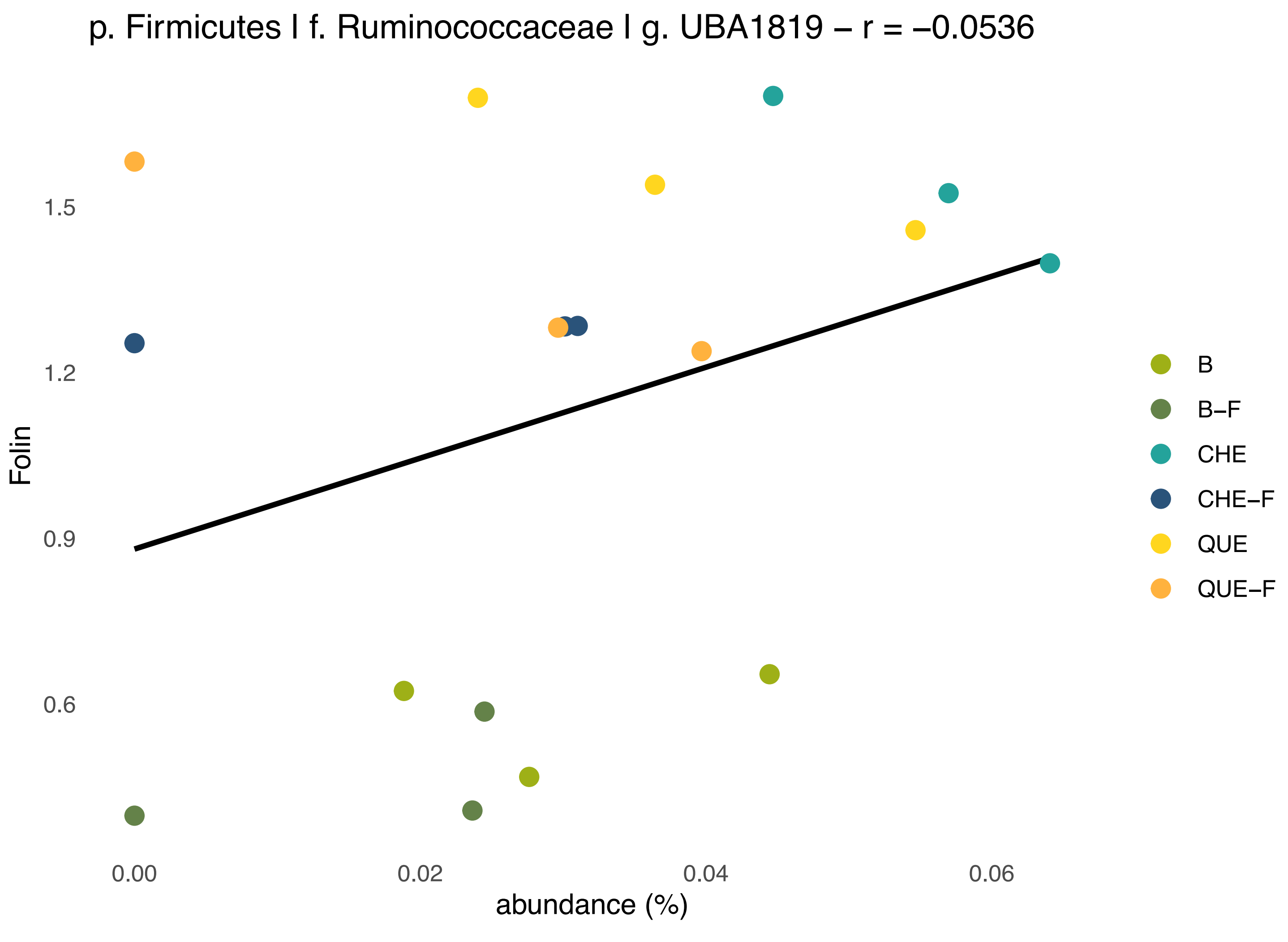

p. Firmicutes I f. Ruminococcaceae I g. UBA1819 – r = –0.0536
1.5
1.2
0.9
0.6
Folin
0.00
0.02
0.04
0.06
abundance (%)
B
B–F
CHE
CHE–F
QUE
QUE–F

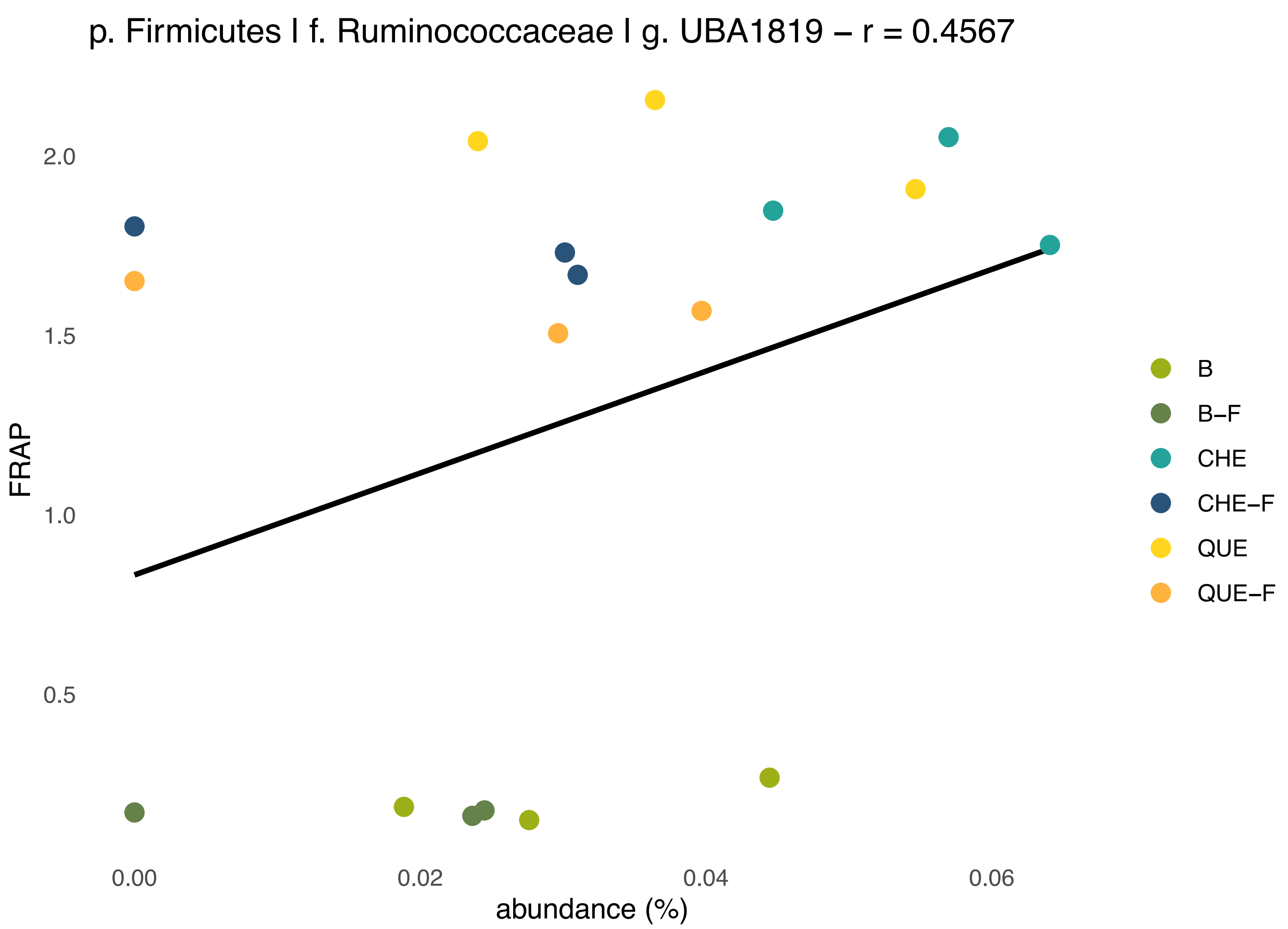
p. Firmicutes I f. Ruminococcaceae I g. UBA1819 – r = 0.4567
FRAP
2.0
1.5
1.0
0.5
0.00
0.02
0.04
0.06
abundance (%)
B
B–F
CHE
CHE–F
QUE
QUE–F

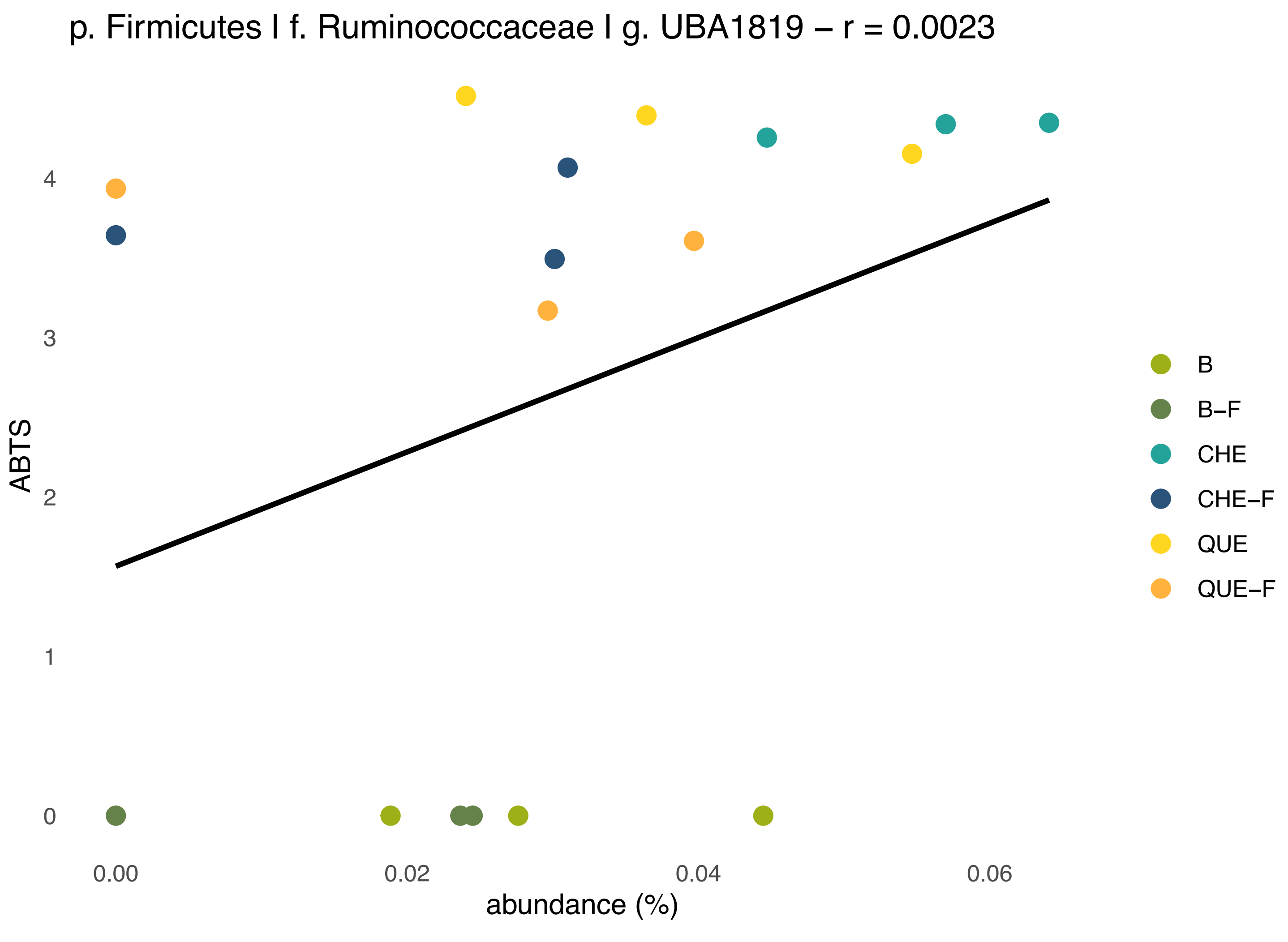
p. Firmicutes l f. Ruminococcaceae l g. UBA1819 – r = 0.0023
ABTS
4
3
2
1
0
0.00
0.02
0.04
0.06
abundance (%)
B
B–F
CHE
CHE–F
QUE
QUE–F

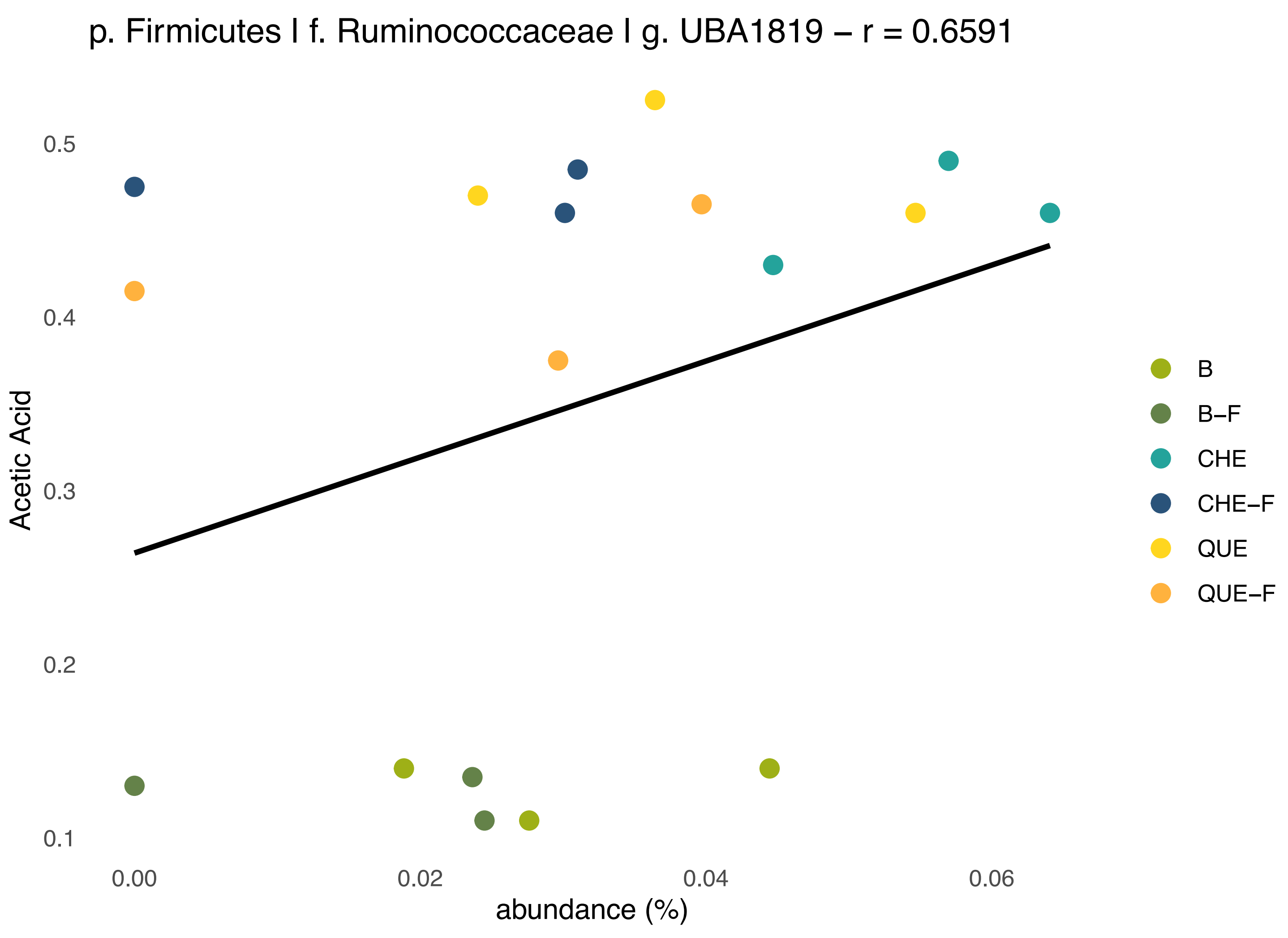
p. Firmicutes I f. Ruminococcaceae I g. UBA1819 – r = 0.6591
Acetic Acid
0.5
0.4
0.3
0.2
0.1
0.00
0.02
0.04
0.06
abundance (%)
B
B–F
CHE
CHE–F
QUE
QUE–F

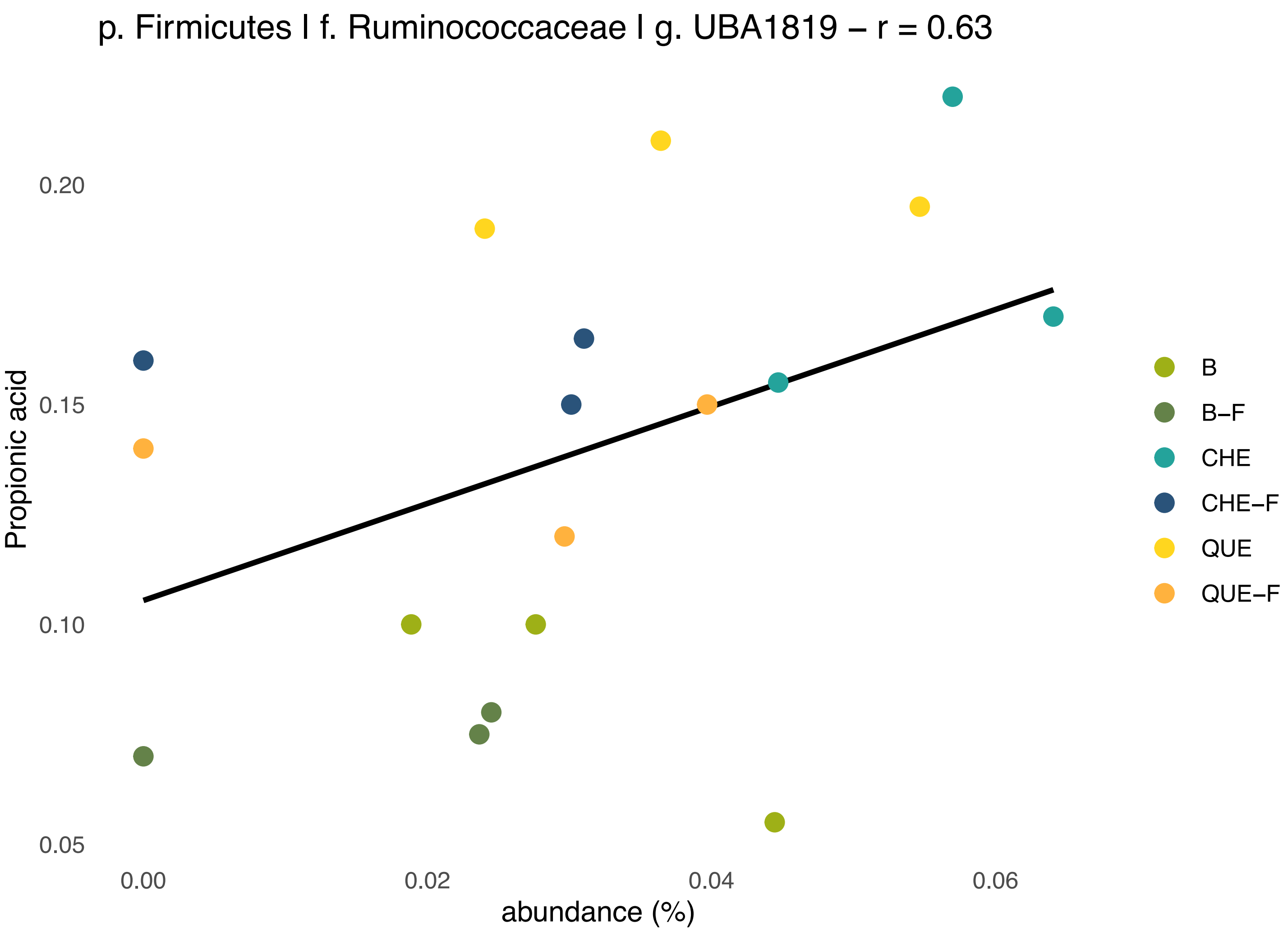

p. Firmicutes I f. Ruminococcaceae I g. UBA1819 – r = 0.63
Propionic acid
0.20
0.15
0.10
0.05
0.00
0.02
0.04
0.06
abundance (%)
B
B–F
CHE
CHE–F
QUE
QUE–F

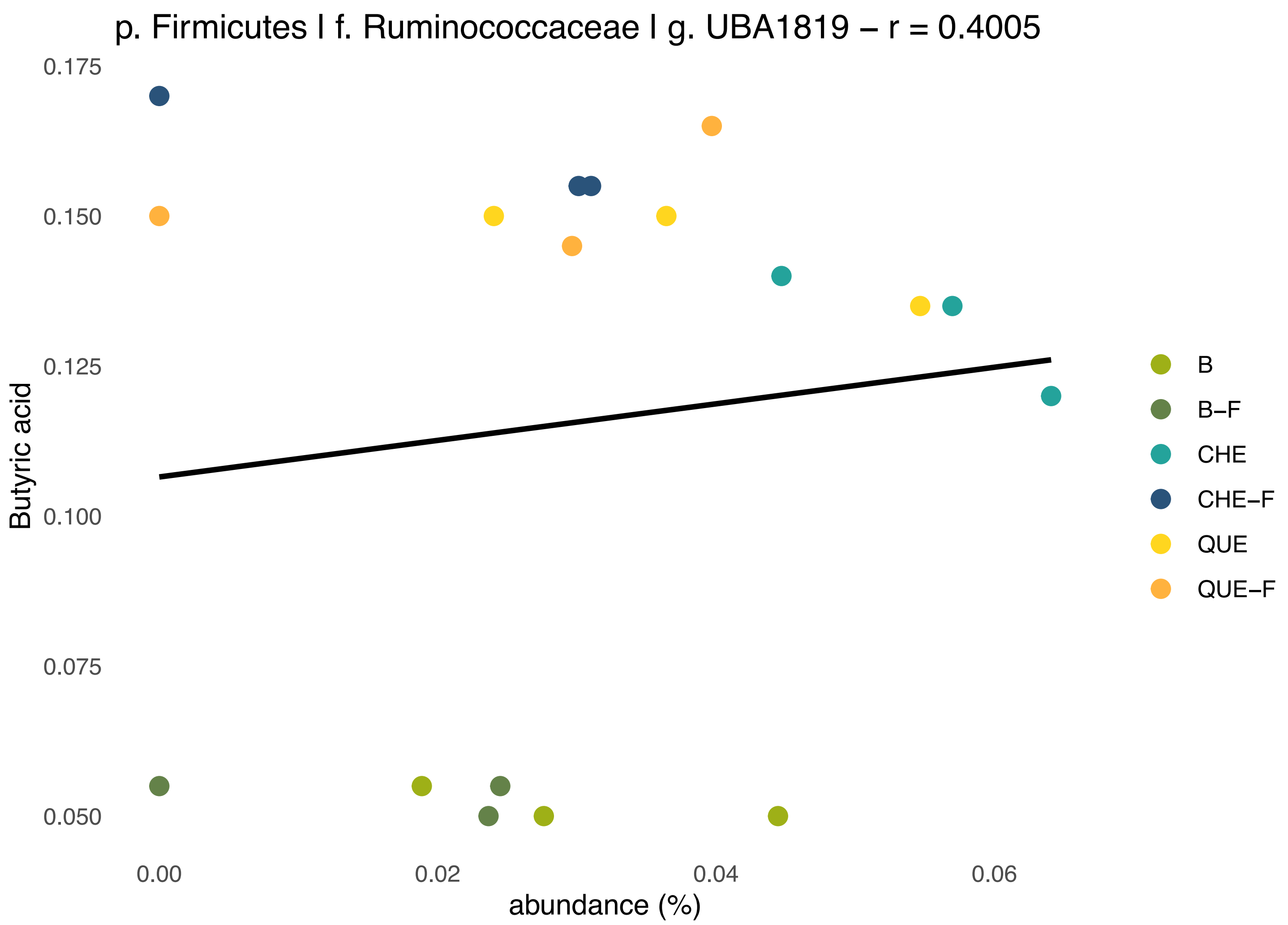

p. Firmicutes I f. Ruminococcaceae I g. UBA1819 – r = 0.4005
Butyric acid
0.175
0.150
0.125
0.100
0.075
0.050
0.00
0.02
0.04
0.06
abundance (%)
B
B–F
CHE
CHE–F
QUE
QUE–F

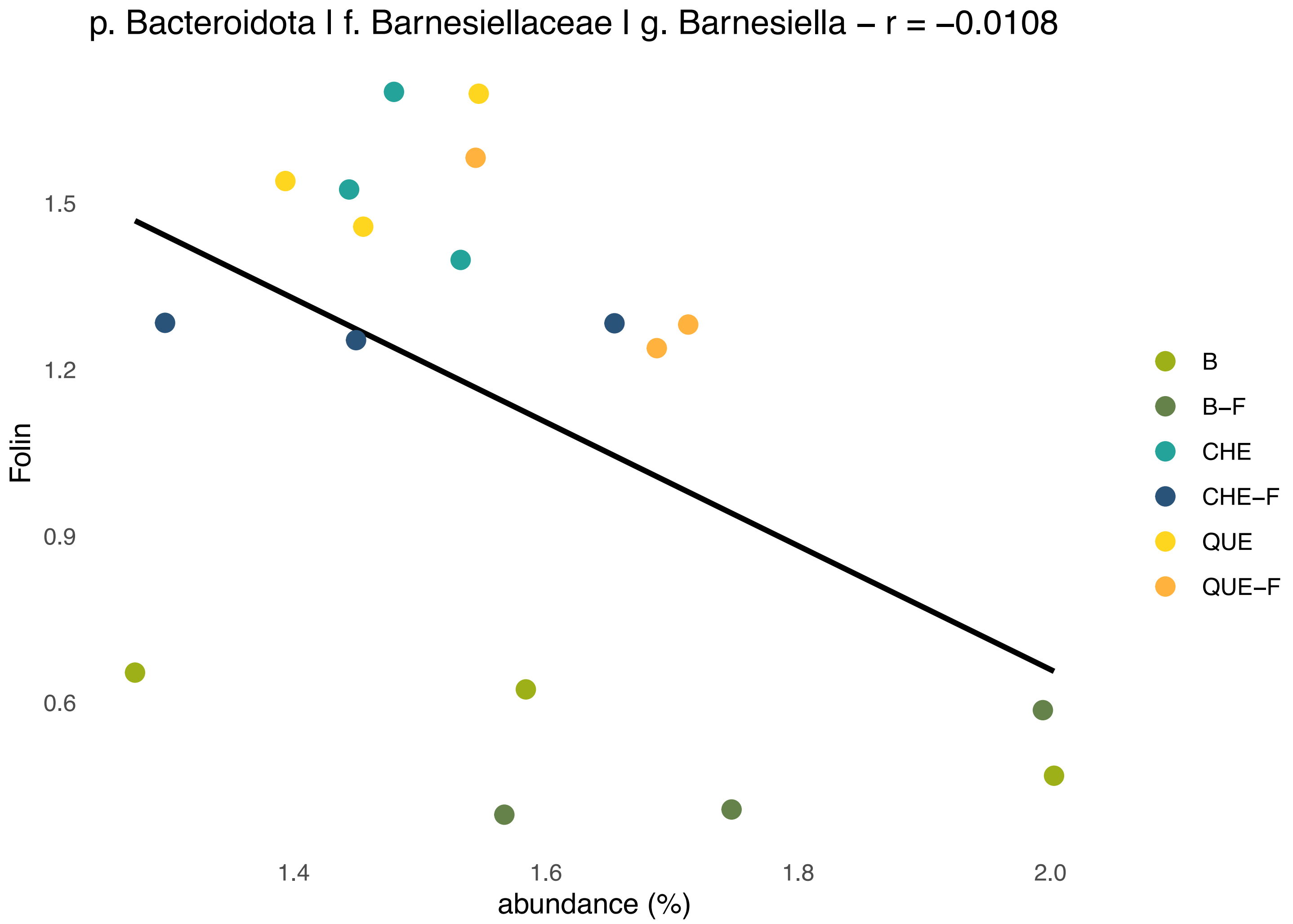

p. Bacteroidota I f. Barnesiellaceae I g. Barnesiella – r = −0.0108
Folin
1.5
1.2
0.9
0.6
1.4
1.6
1.8
2.0
abundance (%)
B
B−F
CHE
CHE−F
QUE
QUE−F

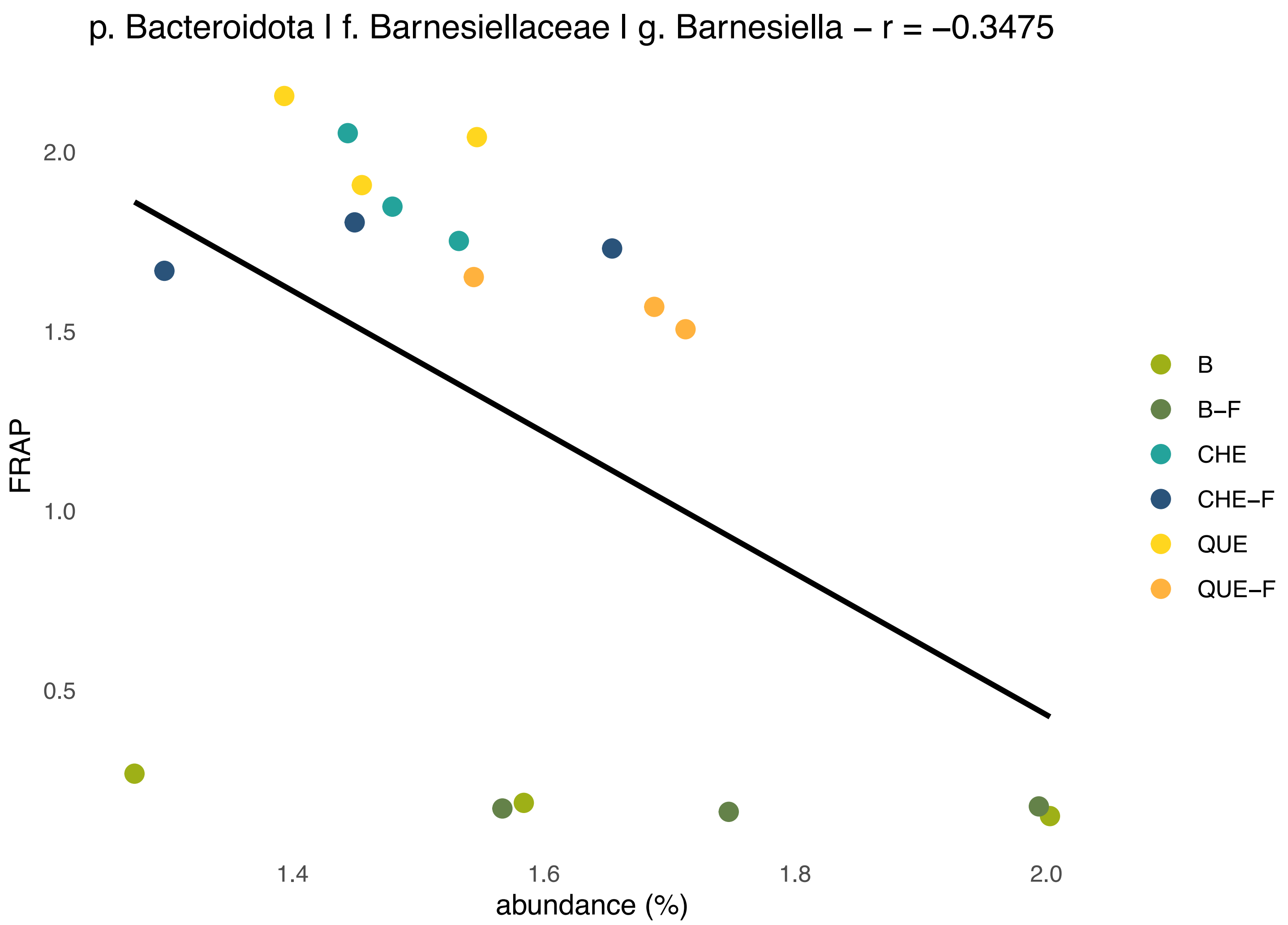

p. Bacteroidota | f. Barnesiellaceae | g. Barnesiella – r = –0.3475
FRAP
2.0
1.5
1.0
0.5
1.4
1.6
1.8
2.0
abundance (%)
B
B–F
CHE
CHE–F
QUE
QUE–F

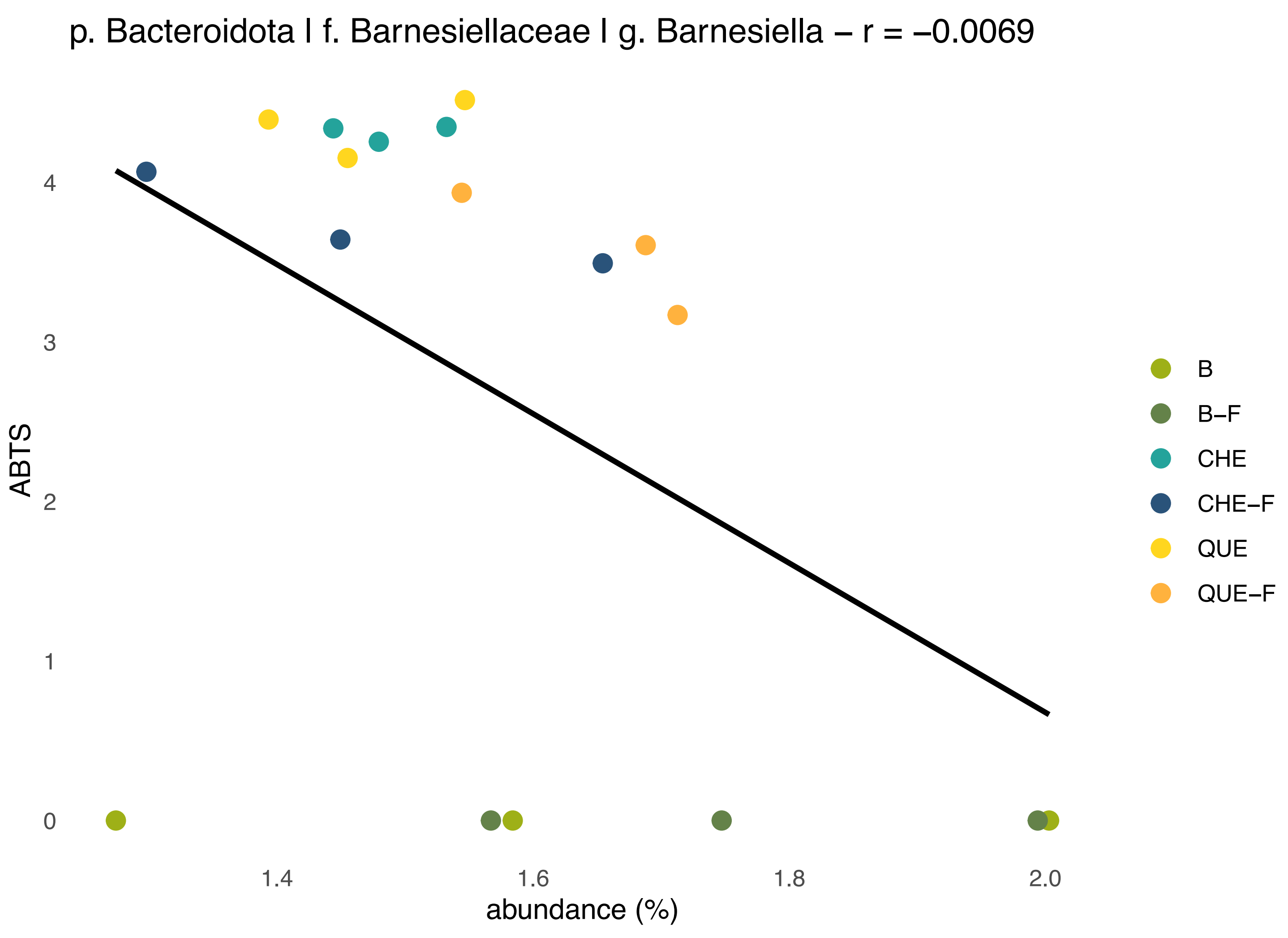

p. Bacteroidota | f. Barnesiellaceae | g. Barnesiella – r = –0.0069
ABTS
0
1
2
3
4
1.4
1.6
1.8
2.0
abundance (%)
B
B–F
CHE
CHE–F
QUE
QUE–F

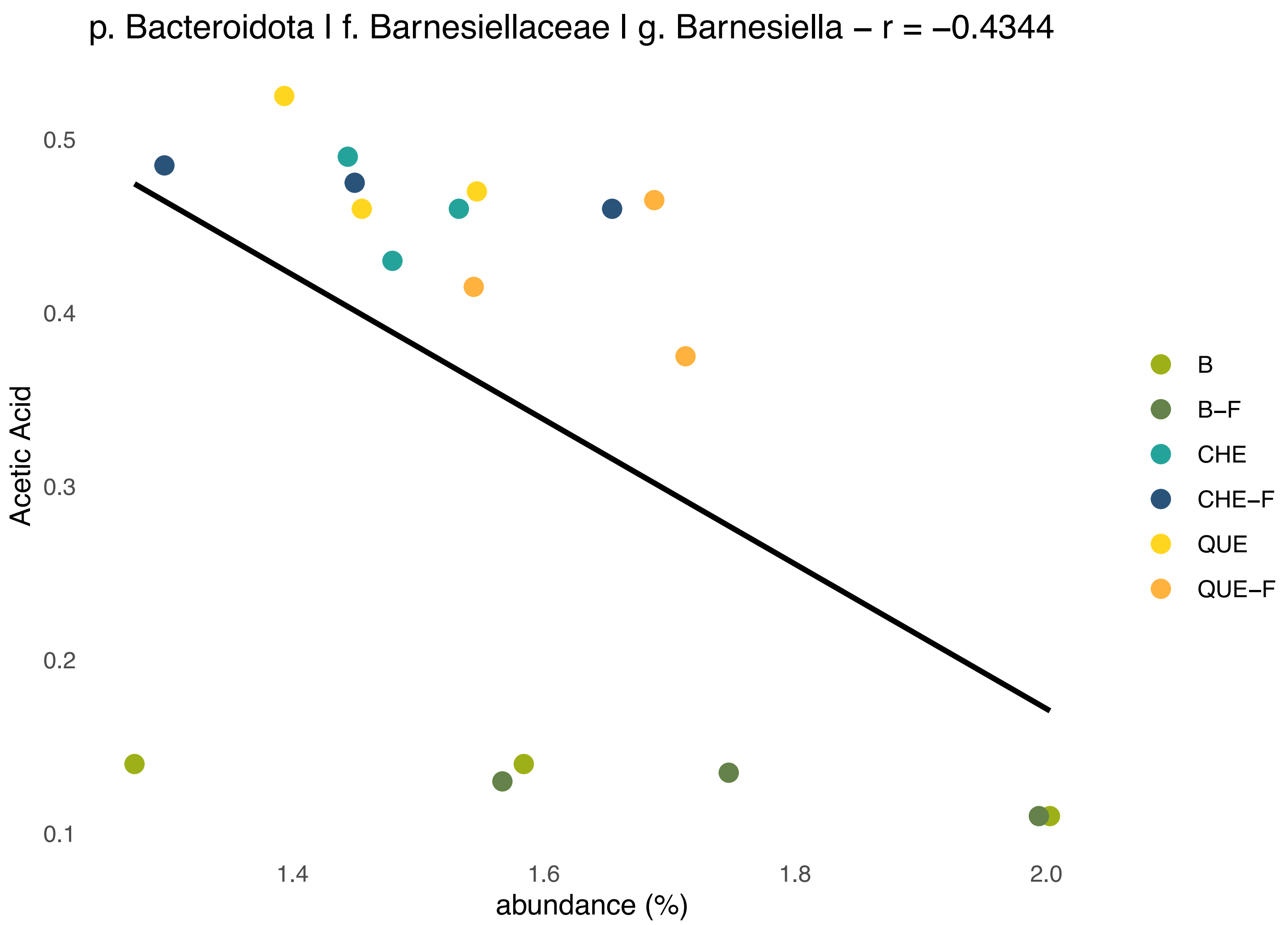

p. Bacteroidota I f. Barnesiellaceae I g. Barnesiella – r = −0.4344
Acetic Acid
0.5
0.4
0.3
0.2
0.1
1.4
1.6
1.8
2.0
abundance (%)
B
B−F
CHE
CHE−F
QUE
QUE−F

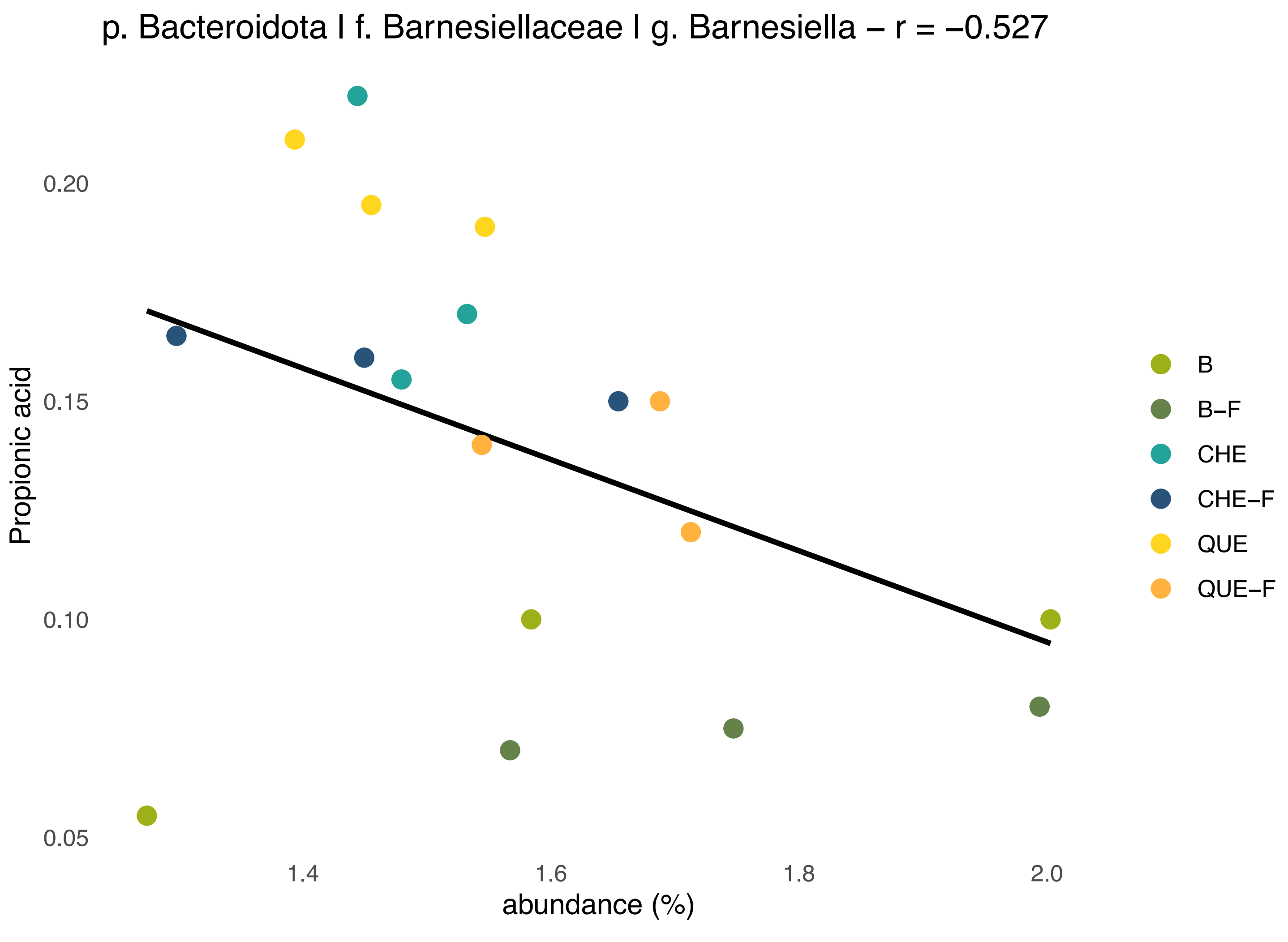
p. Bacteroidota l f. Barnesiellaceae l g. Barnesiella – r = −0.527
Propionic acid
0.20
0.15
0.10
0.05
1.4
1.6
1.8
2.0
abundance (%)
B
B−F
CHE
CHE−F
QUE
QUE−F

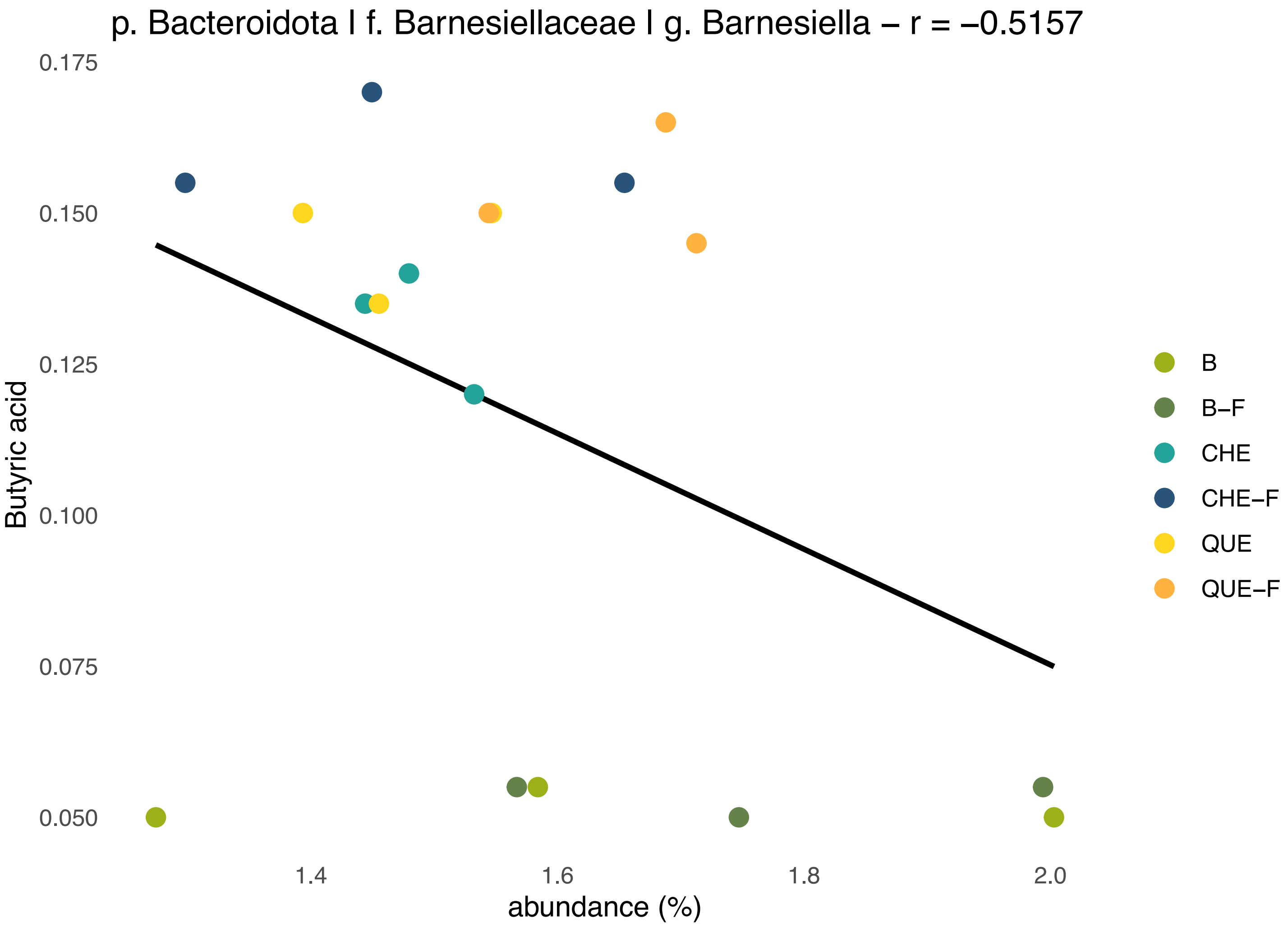
p. Bacteroidota | f. Barnesiellaceae | g. Barnesiella – r = –0.5157
0.175
0.150
0.125
0.100
0.075
0.050
Butyric acid
1.4
1.6
1.8
2.0
abundance (%)
B
B–F
CHE
CHE–F
QUE
QUE–F

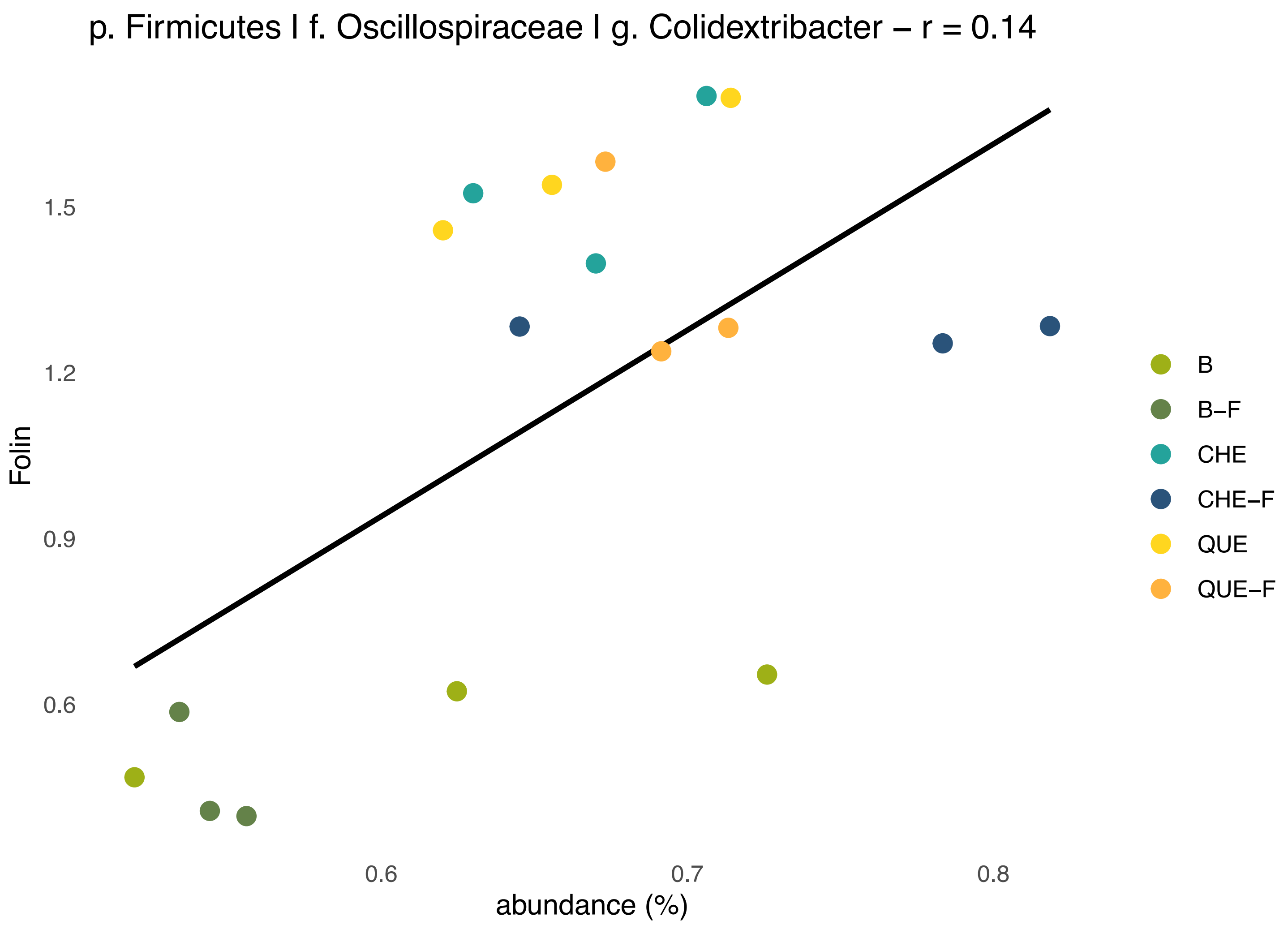
p. Firmicutes I f. Oscillospiraceae I g. Colidextribacter – r = 0.14
Folin
1.5
1.2
0.9
0.6
0.6
0.7
0.8
abundance (%)
B
B–F
CHE
CHE–F
QUE
QUE–F

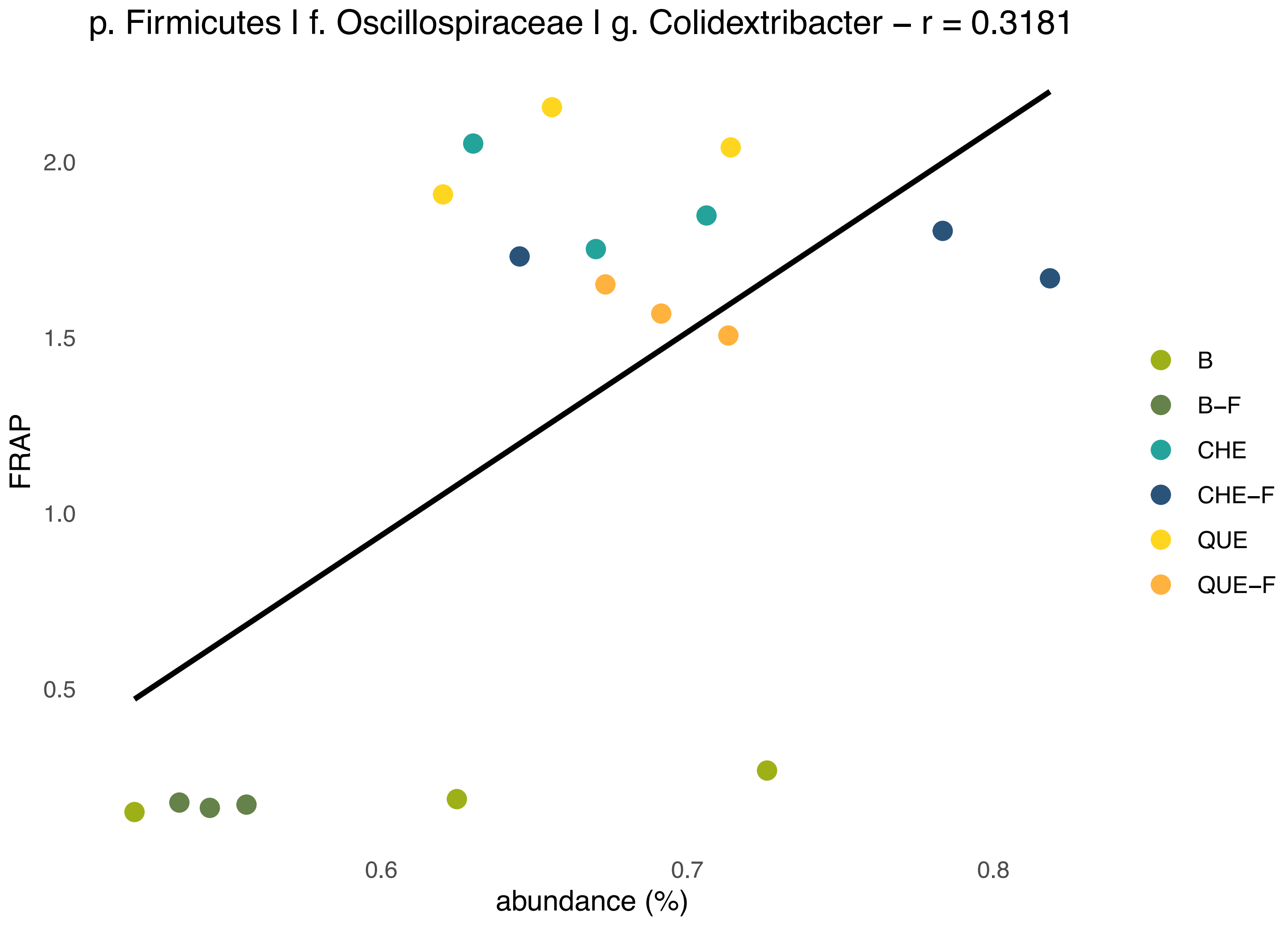
p. Firmicutes I f. Oscillospiraceae I g. Colidextribacter – r = 0.3181
FRAP
2.0
1.5
1.0
0.5
0.6
0.7
0.8
abundance (%)
B
B–F
CHE
CHE–F
QUE
QUE–F

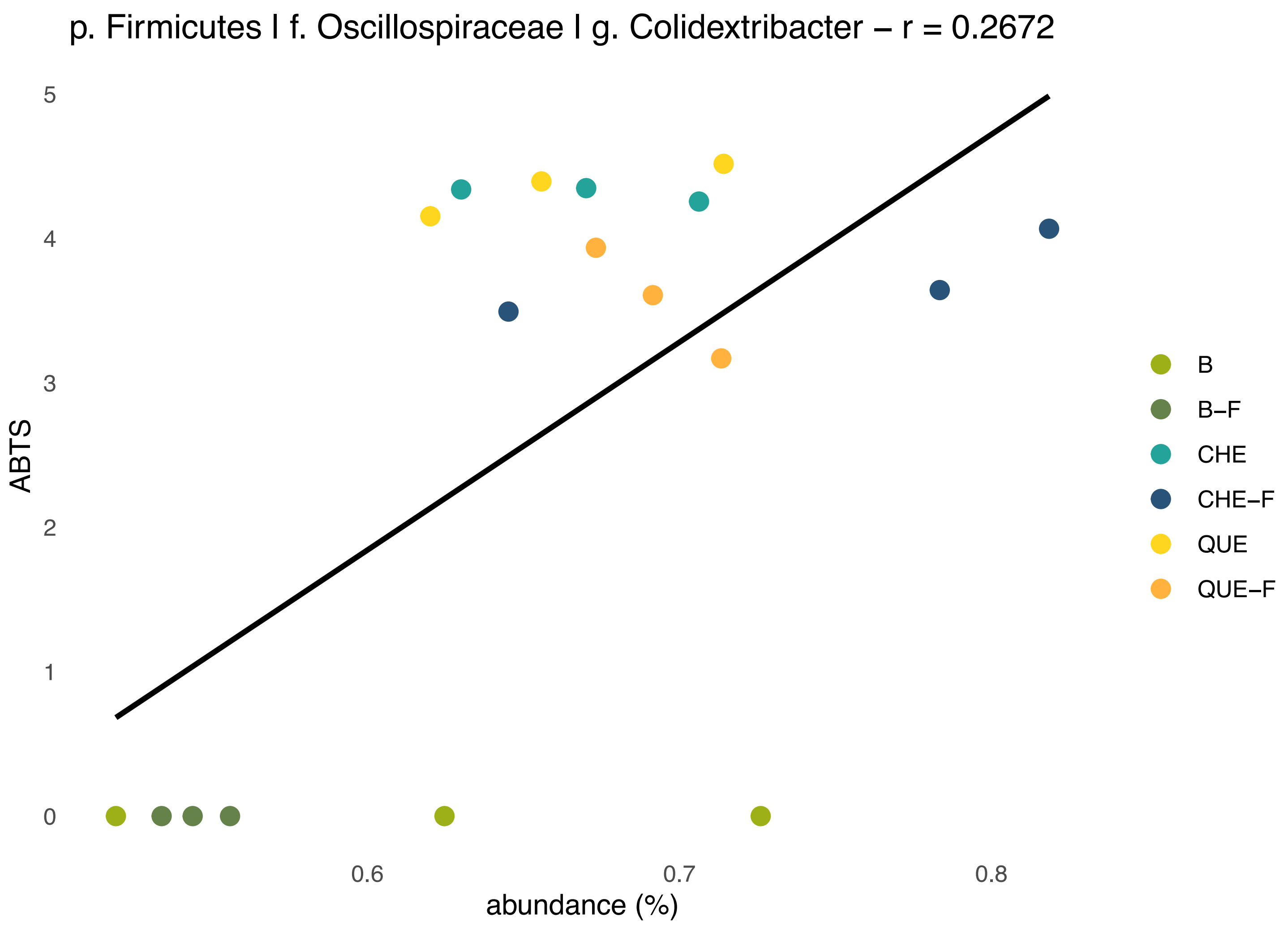

p. Firmicutes I f. Oscillospiraceae I g. Colidextribacter – r = 0.2672
ABTS
5
4
3
2
1
0
0.6
0.7
0.8
abundance (%)
B
B–F
CHE
CHE–F
QUE
QUE–F

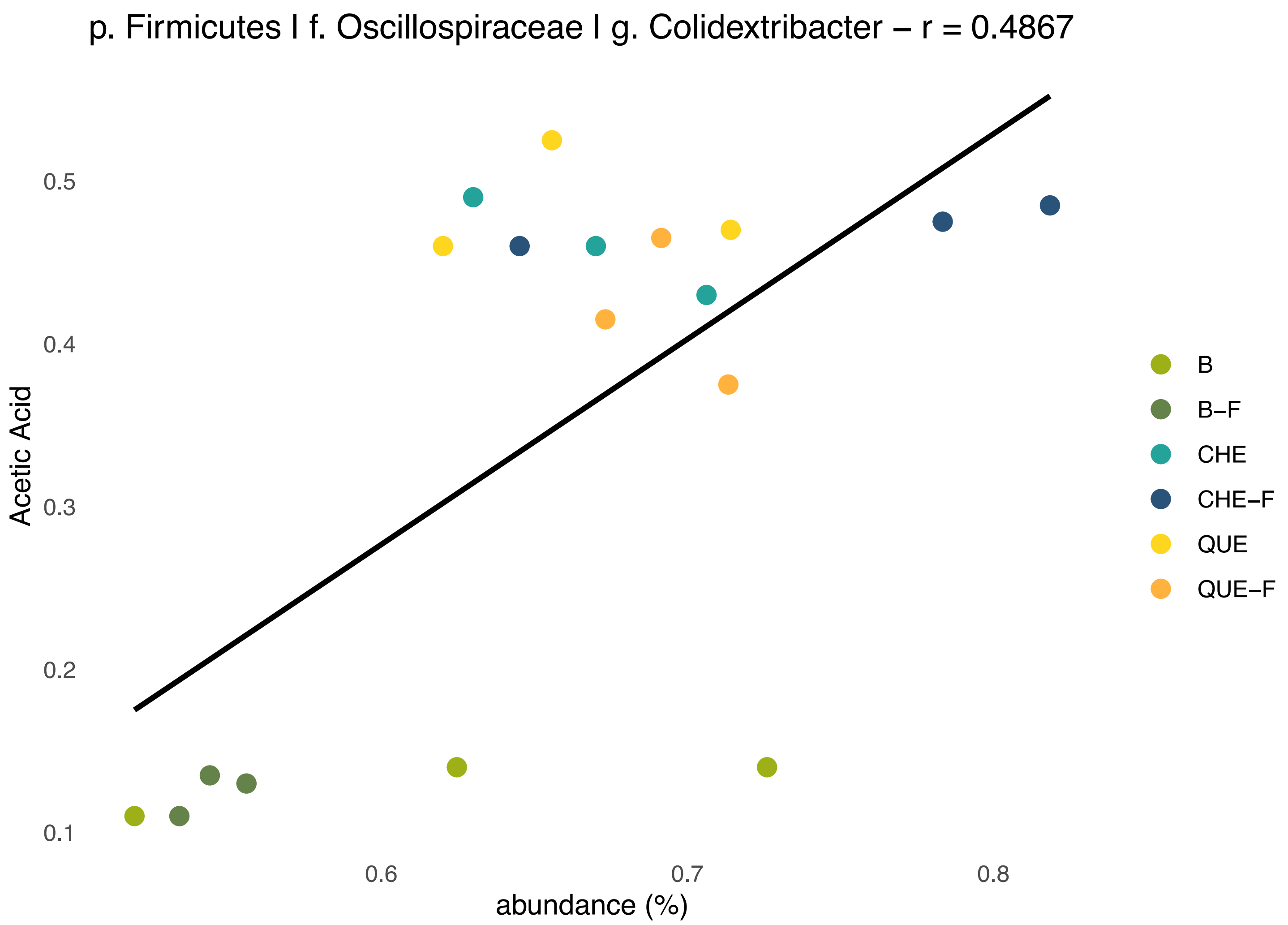
p. Firmicutes I f. Oscillospiraceae I g. Colidextribacter – r = 0.4867
Acetic Acid
0.1
0.2
0.3
0.4
0.5
abundance (%)
0.6
0.7
0.8
B
B–F
CHE
CHE–F
QUE
QUE–F

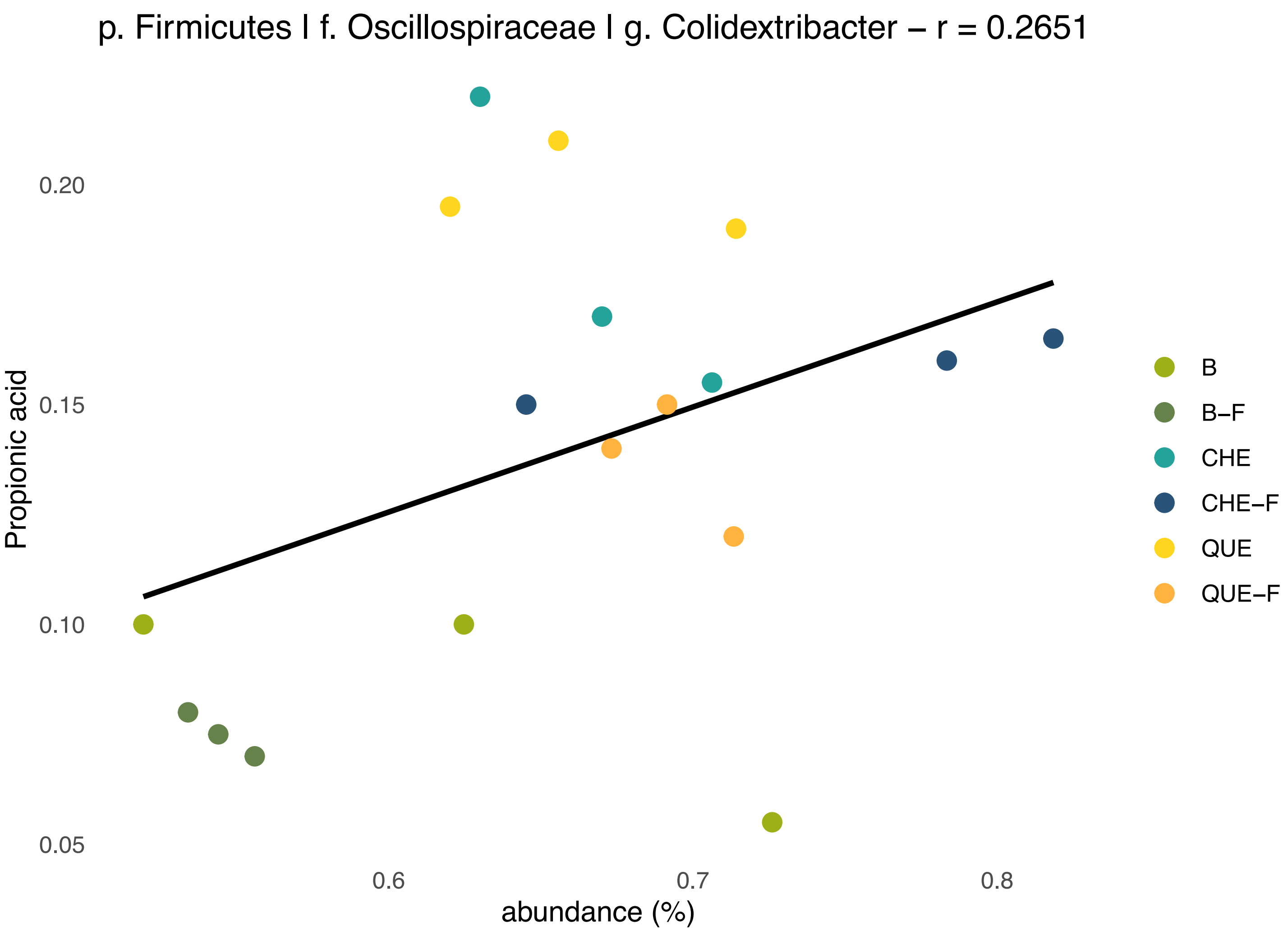

p. Firmicutes I f. Oscillospiraceae I g. Colidextribacter – r = 0.2651
Propionic acid
0.20
0.15
0.10
0.05
0.6
0.7
0.8
abundance (%)
B
B–F
CHE
CHE–F
QUE
QUE–F

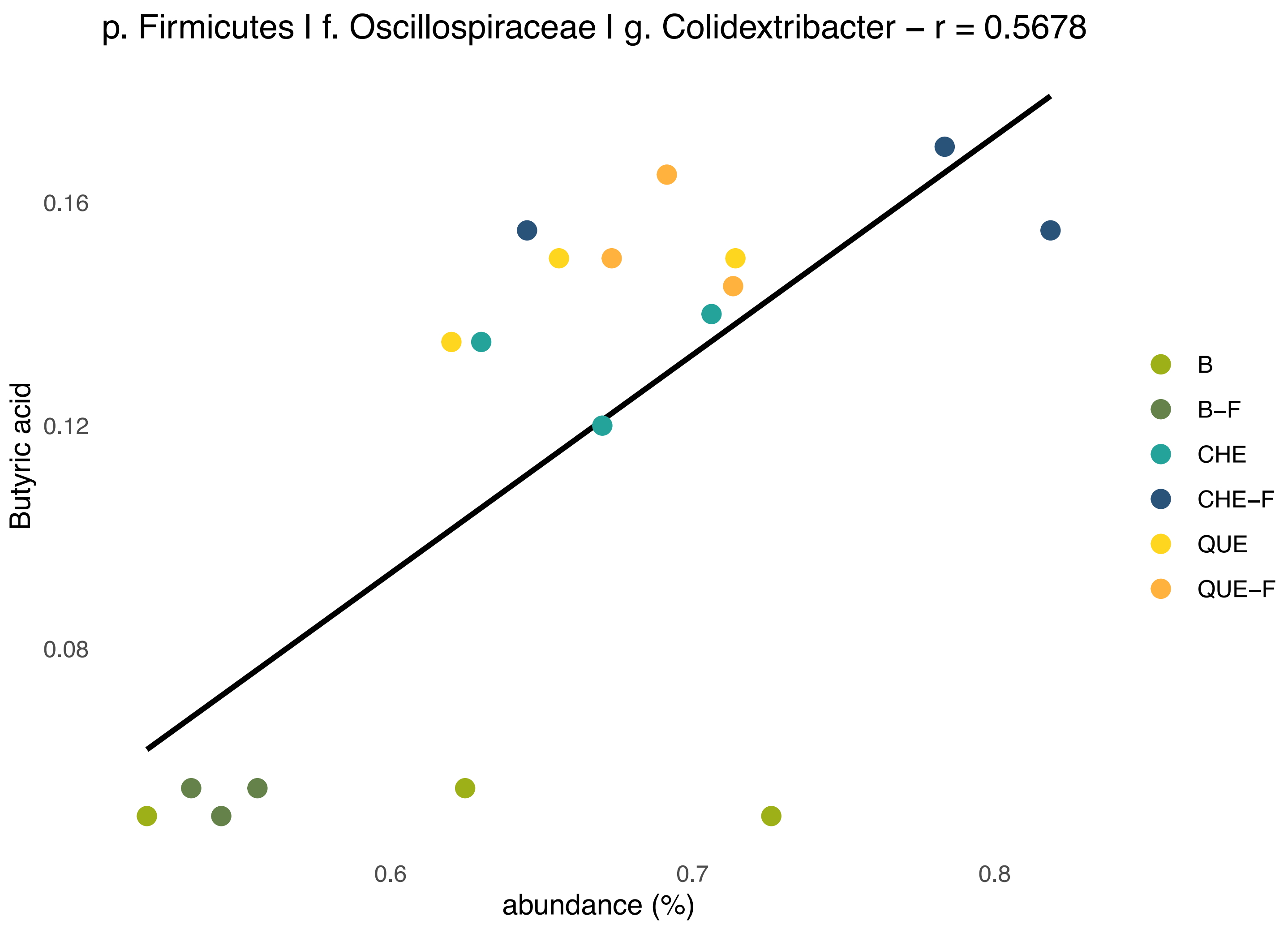
p. Firmicutes I f. Oscillospiraceae I g. Colidextribacter – r = 0.5678
Butyric acid
0.16
0.12
0.08
0.6
0.7
0.8
abundance (%)
B
B–F
CHE
CHE–F
QUE
QUE–F

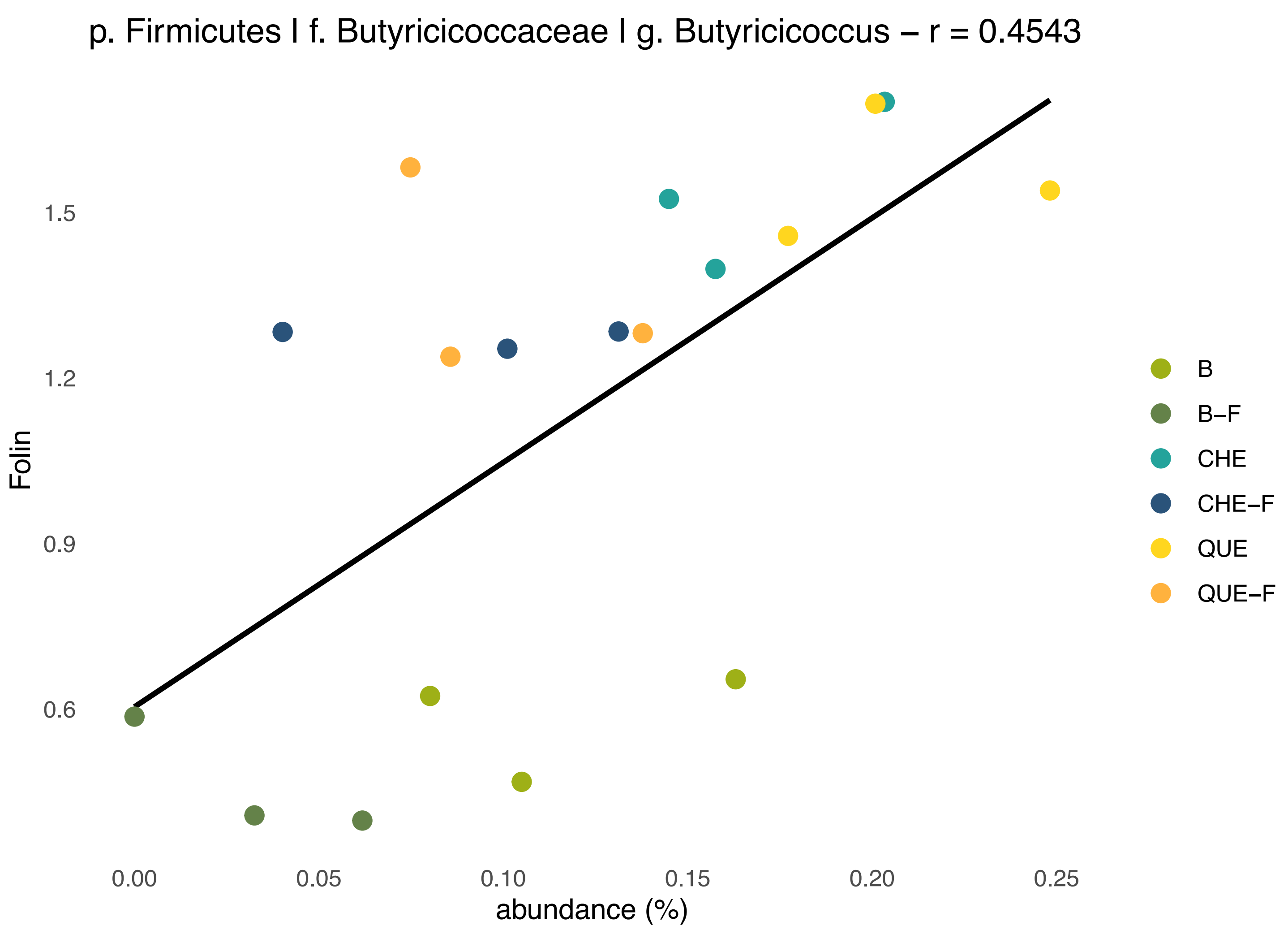

p. Firmicutes | f. Butyricicoccaceae | g. Butyricicoccus – r = 0.4543
Folin
1.5
1.2
0.9
0.6
0.00
0.05
0.10
0.15
0.20
0.25
abundance (%)
B
B–F
CHE
CHE–F
QUE
QUE–F

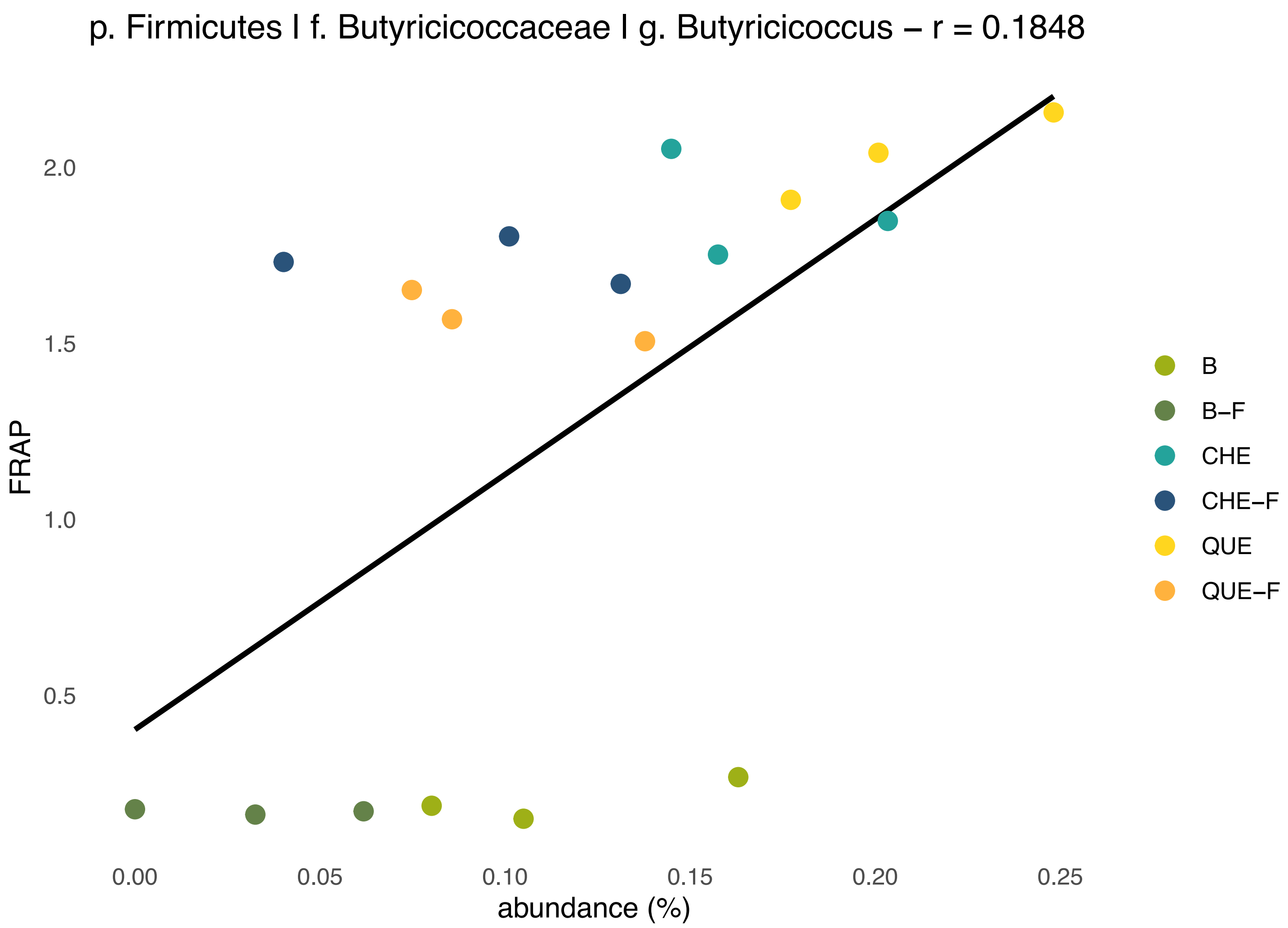

p. Firmicutes I f. Butyricicoccaceae I g. Butyricicoccus – r = 0.1848
FRAP
abundance (%)
0.5
1.0
1.5
2.0
0.00
0.05
0.10
0.15
0.20
0.25
B
B–F
CHE
CHE–F
QUE
QUE–F

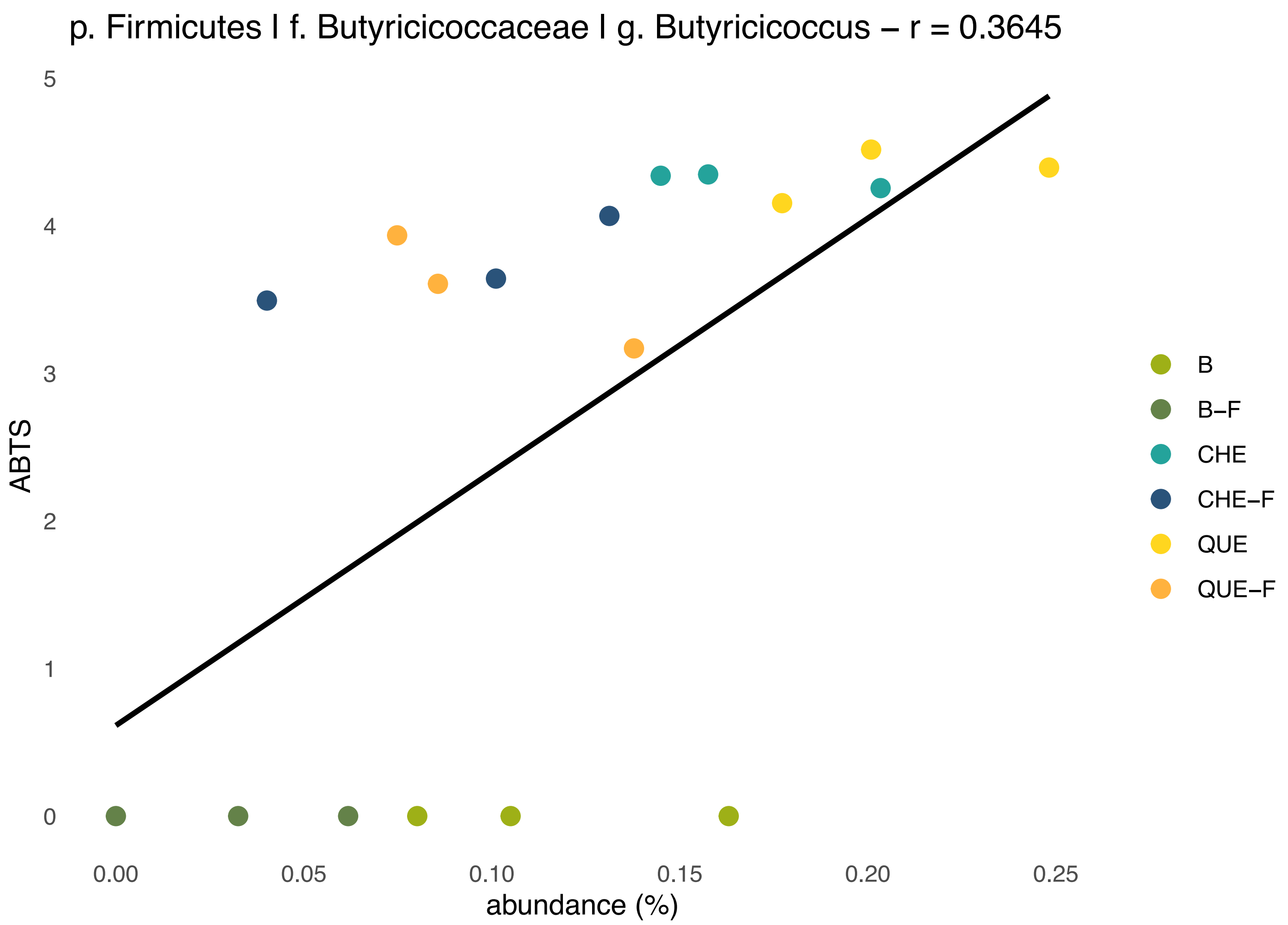
p. Firmicutes l f. Butyricicoccaceae l g. Butyricicoccus – r = 0.3645
ABTS
5
4
3
2
1
0
0.00
0.05
0.10
0.15
0.20
0.25
abundance (%)
B
B–F
CHE
CHE–F
QUE
QUE–F

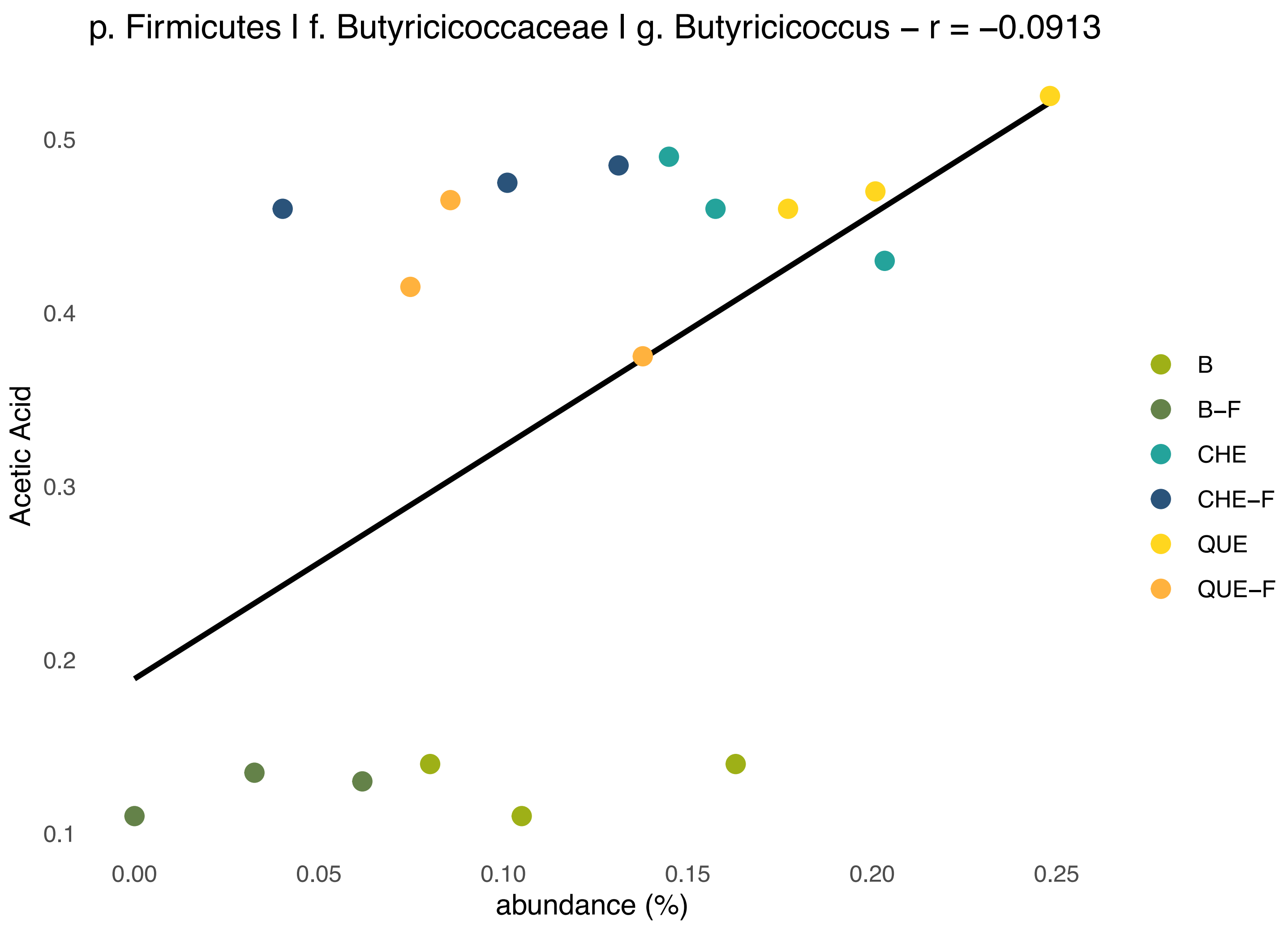

p. Firmicutes | f. Butyricicoccaceae | g. Butyricicoccus – r = −0.0913
Acetic Acid
0.1
0.2
0.3
0.4
0.5
0.00
0.05
0.10
0.15
0.20
0.25
abundance (%)
B
B−F
CHE
CHE−F
QUE
QUE−F

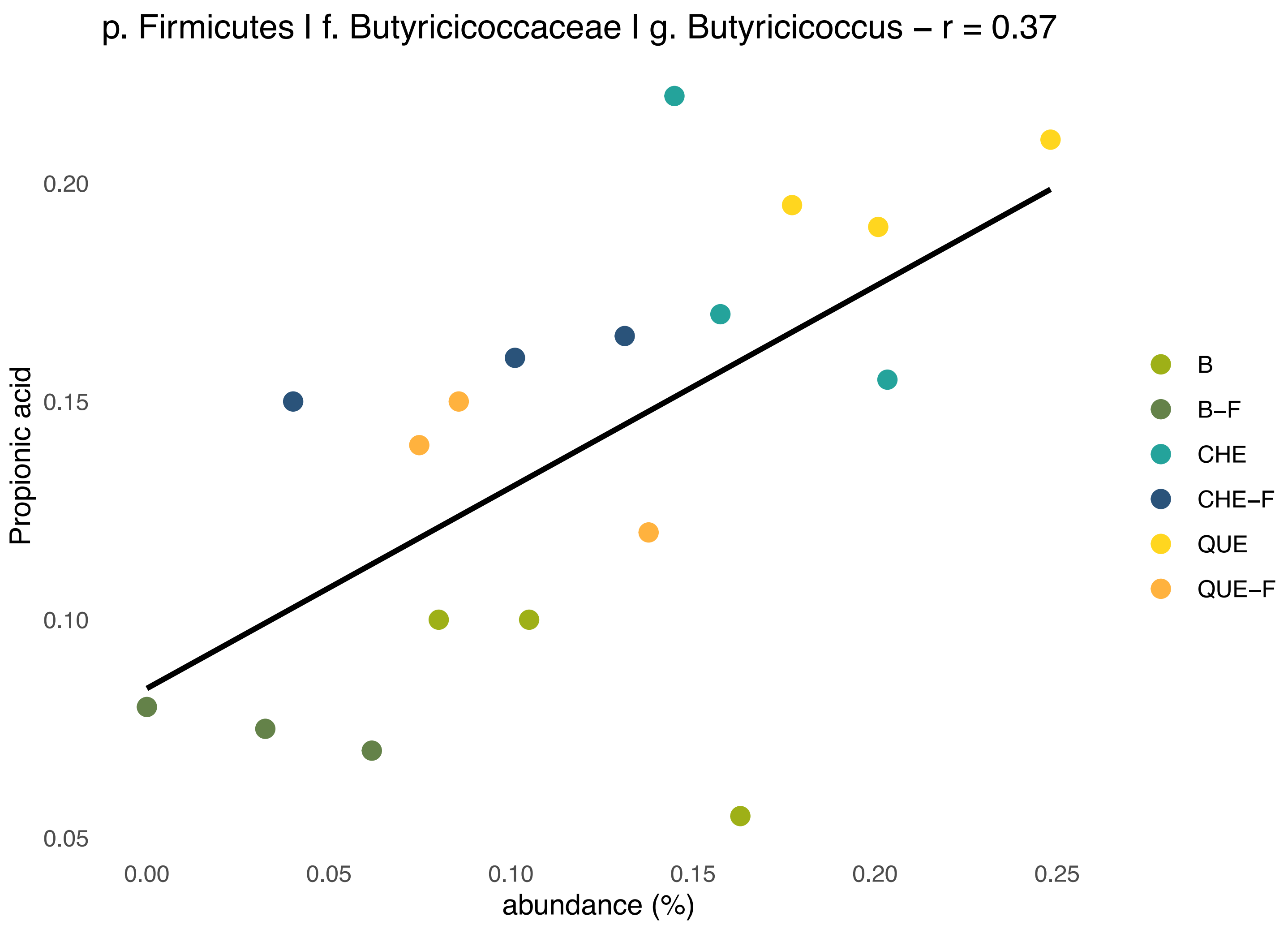

p. Firmicutes l f. Butyricicoccaceae l g. Butyricicoccus – r = 0.37
Propionic acid
0.20
0.15
0.10
0.05
0.00
0.05
0.10
0.15
0.20
0.25
abundance (%)
B
B–F
CHE
CHE–F
QUE
QUE–F

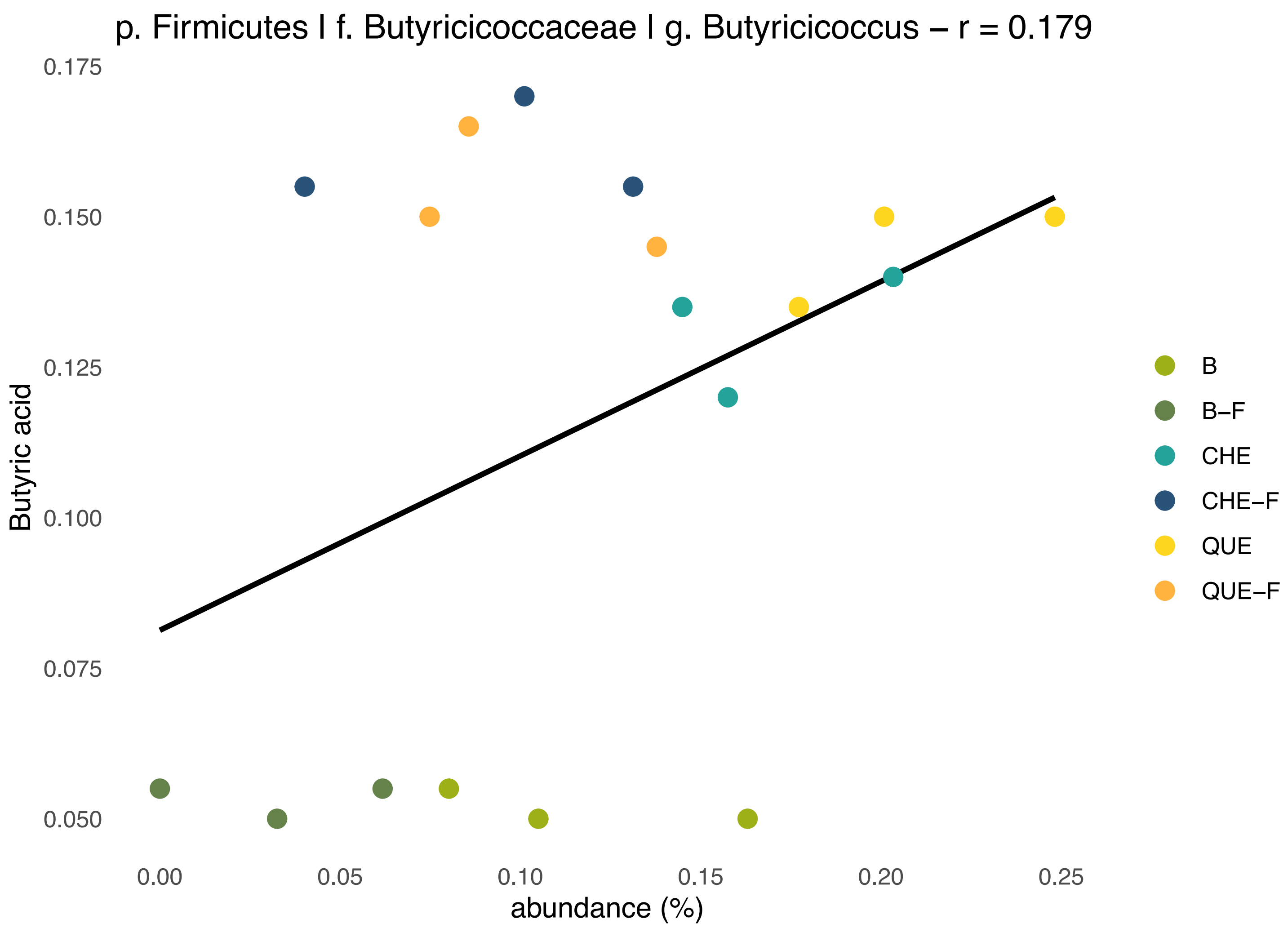
p. Firmicutes I f. Butyricicoccaceae I g. Butyricicoccus – r = 0.179
Butyric acid
0.175
0.150
0.125
0.100
0.075
0.050
0.00
0.05
0.10
0.15
0.20
0.25
abundance (%)
B
B–F
CHE
CHE–F
QUE
QUE–F

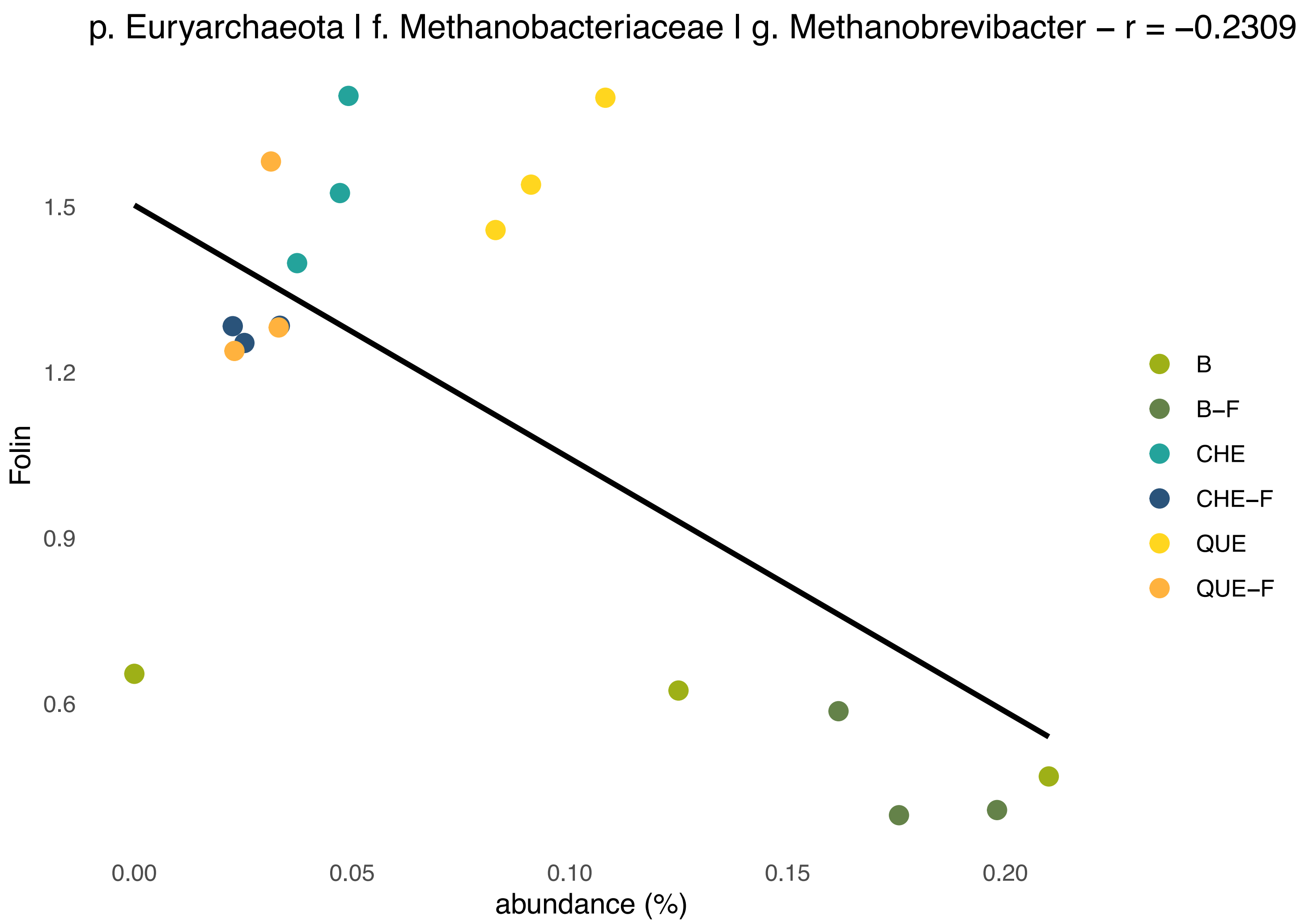
p. Euryarchaeota I f. Methanobacteriaceae I g. Methanobrevibacter – r = −0.2309
Folin
1.5
1.2
0.9
0.6
0.00
0.05
0.10
0.15
0.20
abundance (%)
B
B−F
CHE
CHE−F
QUE
QUE−F

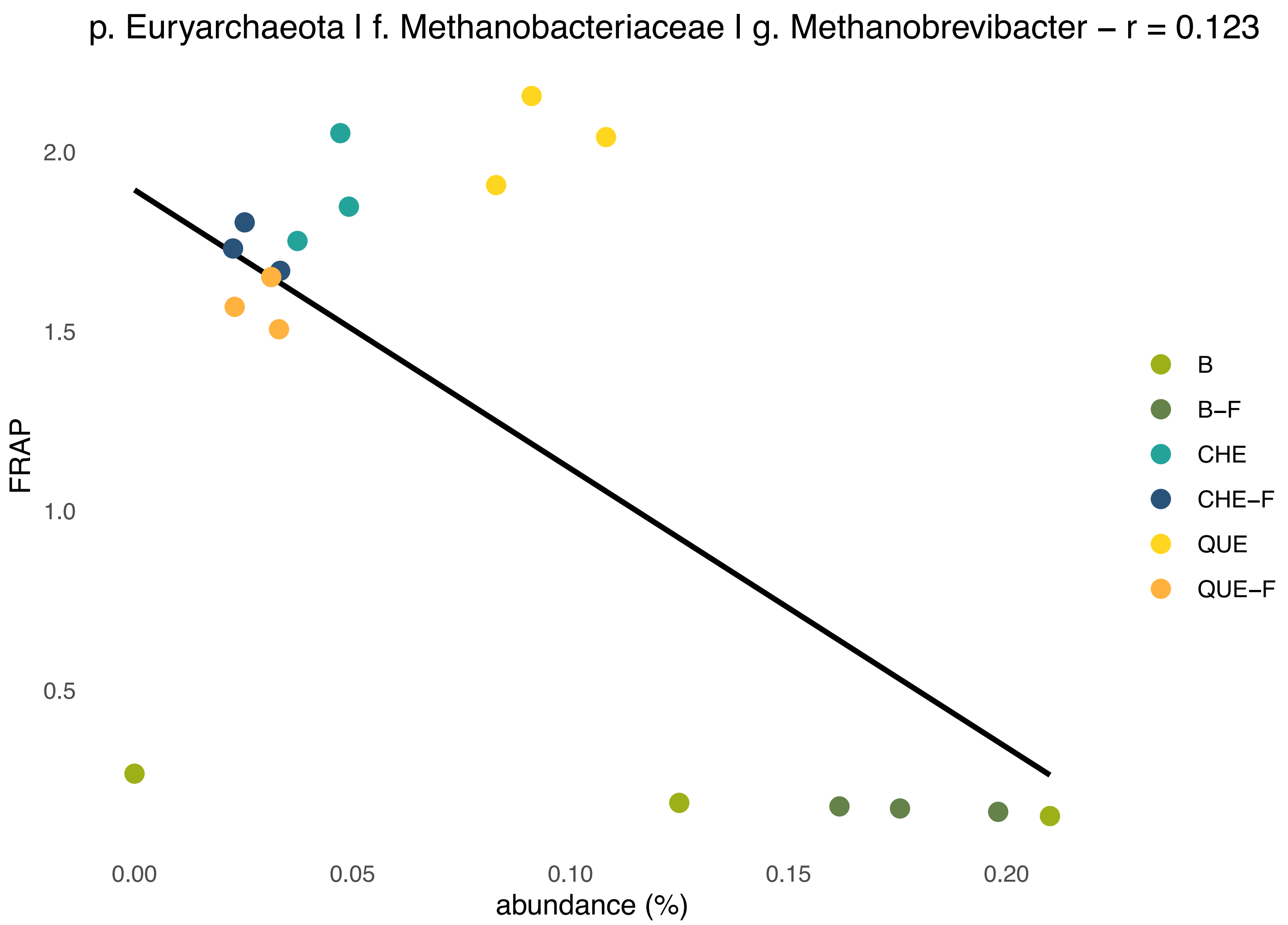

p. Euryarchaeota | f. Methanobacteriaceae | g. Methanobrevibacter – r = 0.123
FRAP
2.0
1.5
1.0
0.5
0.00
0.05
0.10
0.15
0.20
abundance (%)
B
B–F
CHE
CHE–F
QUE
QUE–F

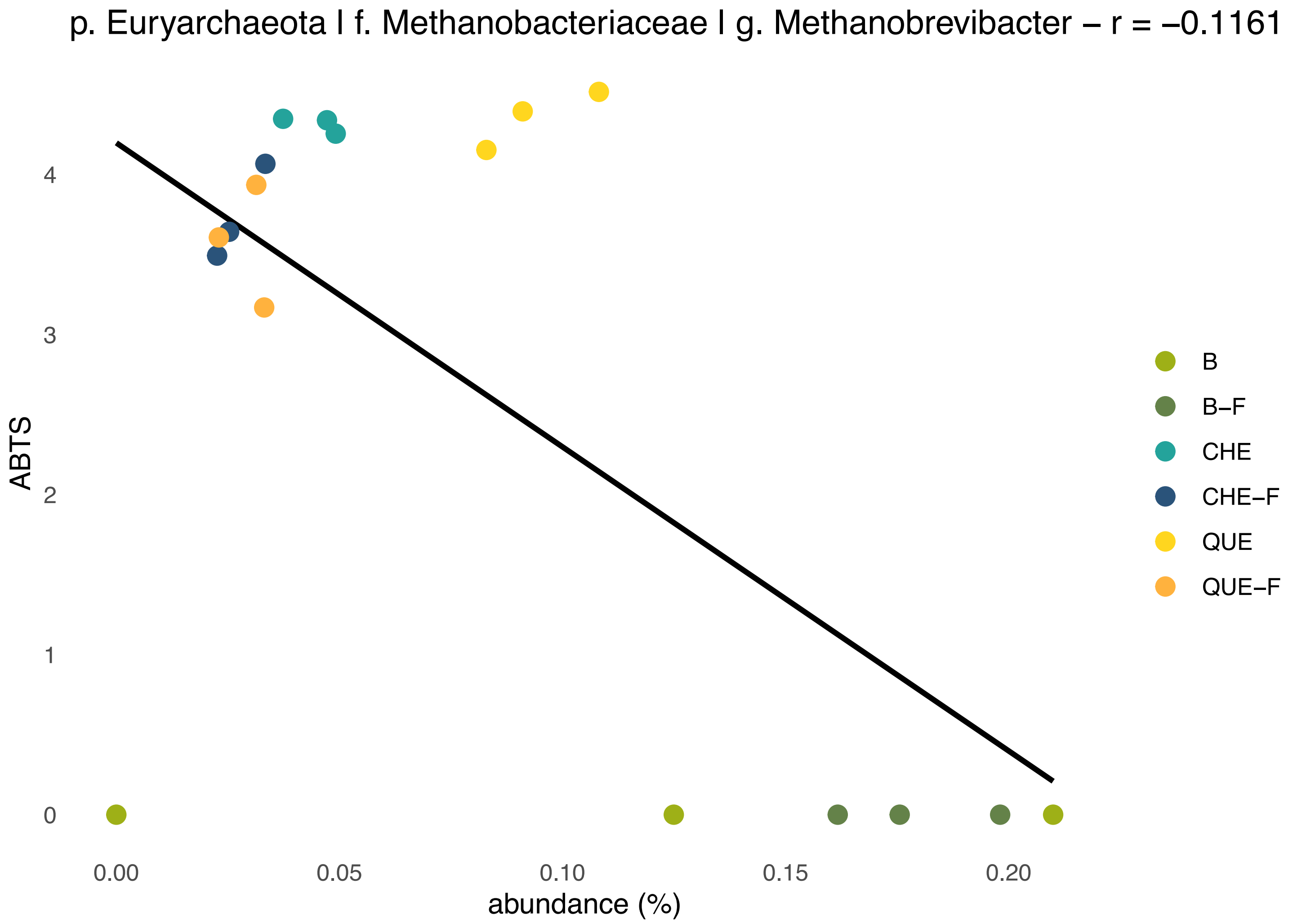

p. Euryarchaeota I f. Methanobacteriaceae I g. Methanobrevibacter – r = –0.1161
ABTS
4
3
2
1
0
0.00
0.05
0.10
0.15
0.20
abundance (%)
B
B–F
CHE
CHE–F
QUE
QUE–F

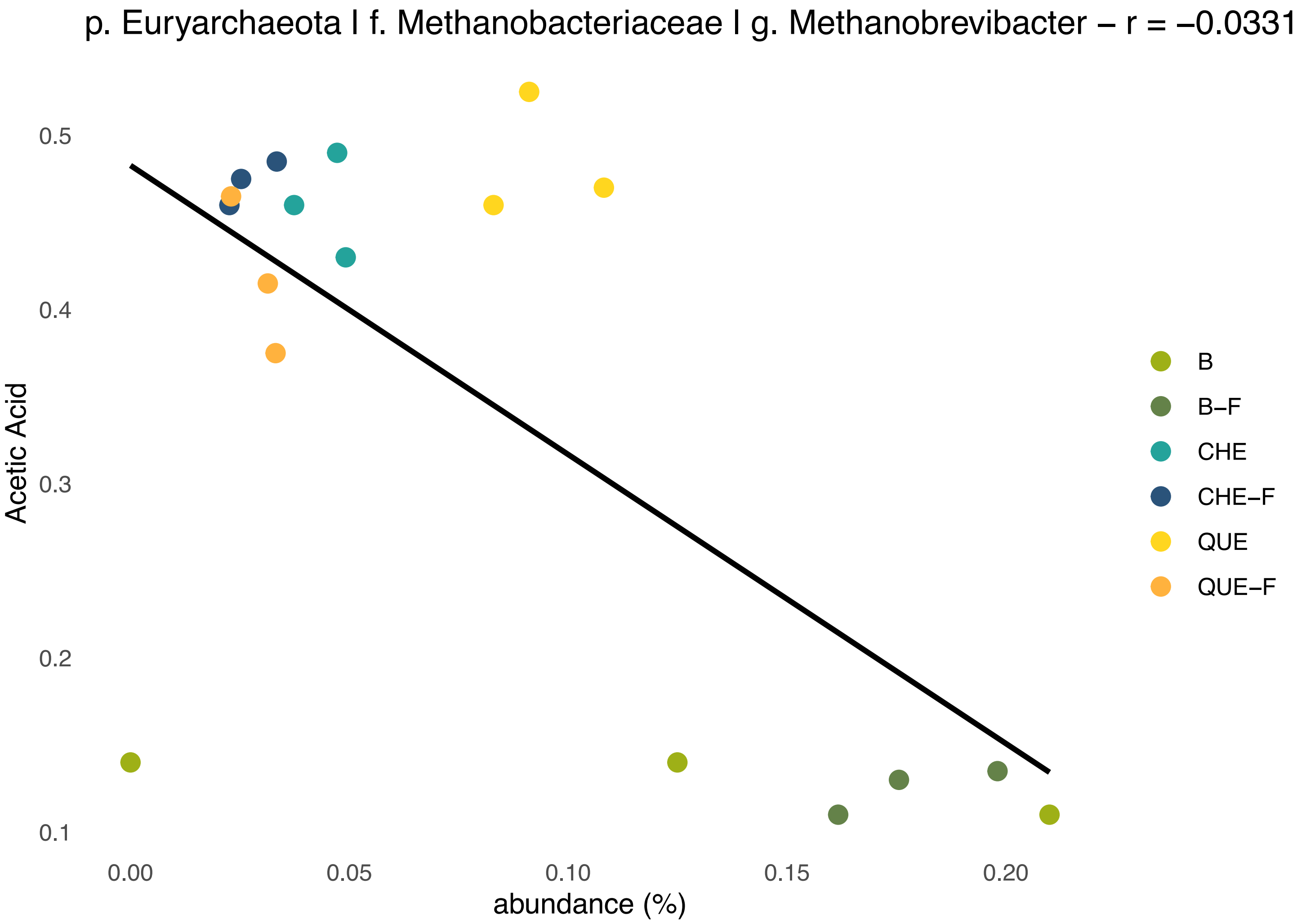
p. Euryarchaeota I f. Methanobacteriaceae I g. Methanobrevibacter – r = −0.0331
Acetic Acid
0.5
0.4
0.3
0.2
0.1
0.00
0.05
0.10
0.15
0.20
abundance (%)
B
B−F
CHE
CHE−F
QUE
QUE−F

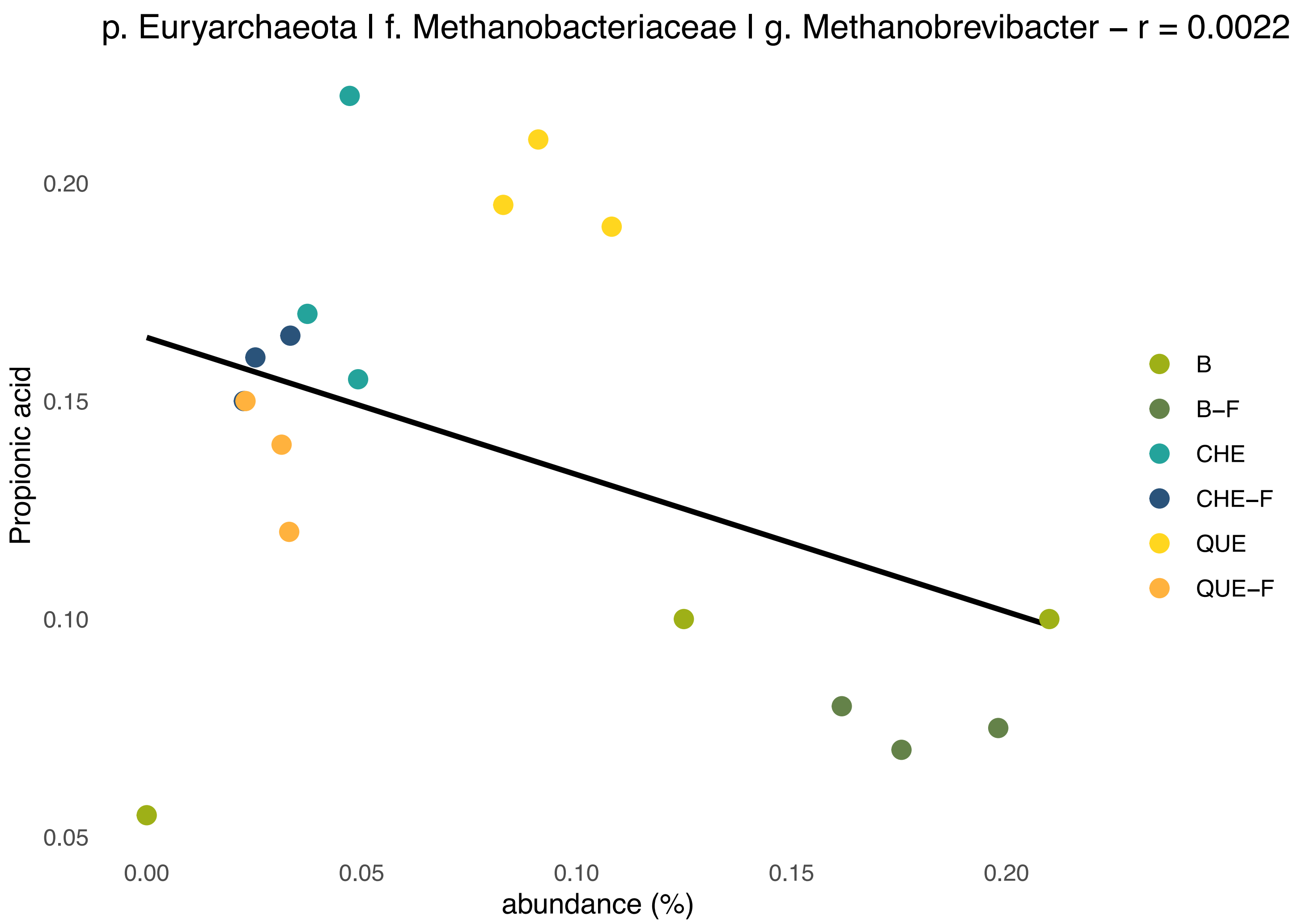

p. Euryarchaeota I f. Methanobacteriaceae I g. Methanobrevibacter – r = 0.0022
Propionic acid
0.20
0.15
0.10
0.05
0.00
0.05
0.10
0.15
0.20
abundance (%)
B
B–F
CHE
CHE–F
QUE
QUE–F

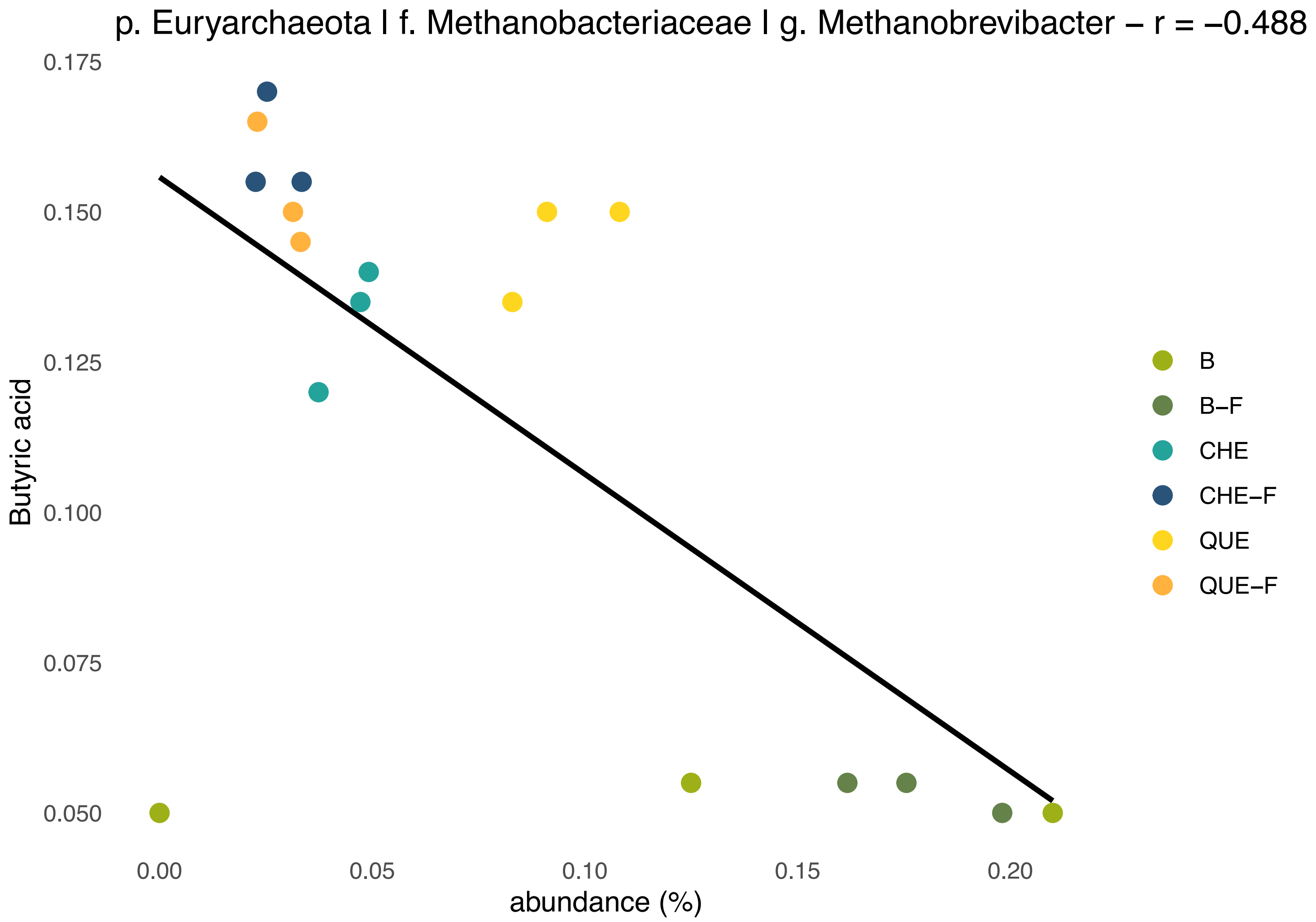

p. Euryarchaeota I f. Methanobacteriaceae I g. Methanobrevibacter – r = –0.488
0.175
0.150
0.125
0.100
0.075
0.050
Butyric acid
0.00
0.05
0.10
0.15
0.20
abundance (%)
B
B–F
CHE
CHE–F
QUE
QUE–F

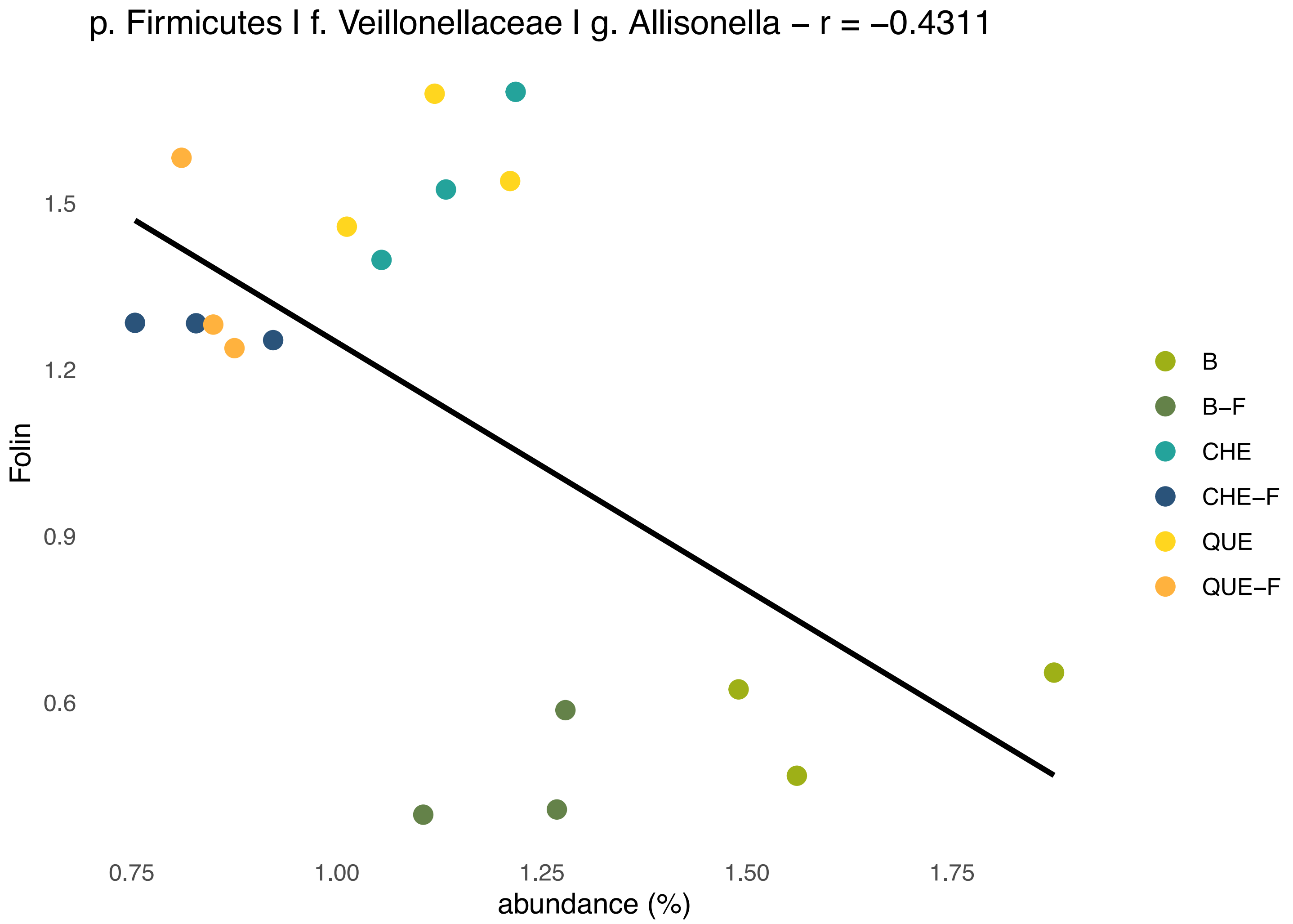
p. Firmicutes I f. Veillonellaceae I g. Allisonella – r = –0.4311
1.5
1.2
0.9
0.6
Folin
0.75
1.00
1.25
1.50
1.75
abundance (%)
B
B–F
CHE
CHE–F
QUE
QUE–F

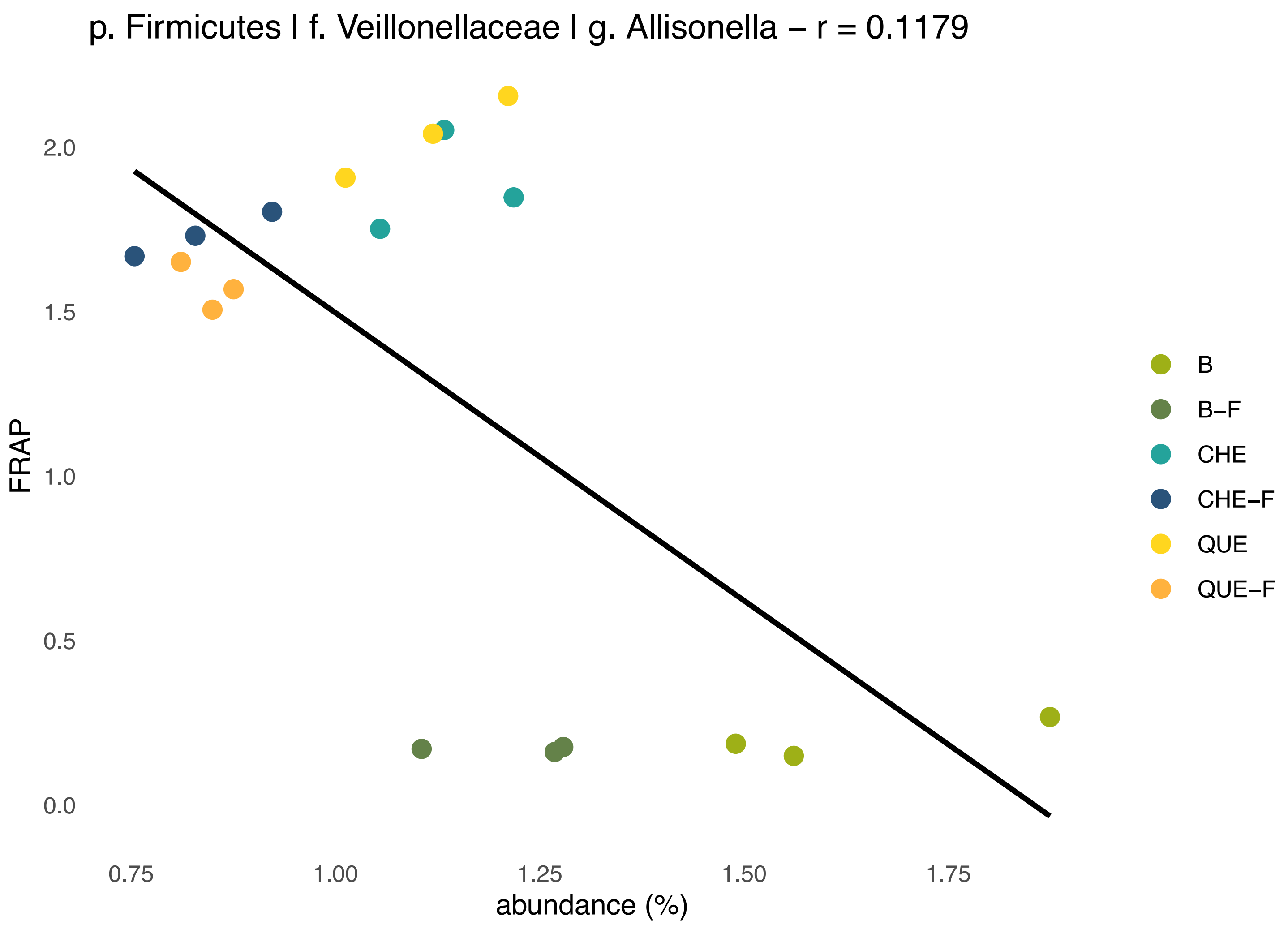
p. Firmicutes l f. Veillonellaceae l g. Allisonella – r = 0.1179
FRAP
2.0
1.5
1.0
0.5
0.0
0.75
1.00
1.25
1.50
1.75
abundance (%)
B
B–F
CHE
CHE–F
QUE
QUE–F

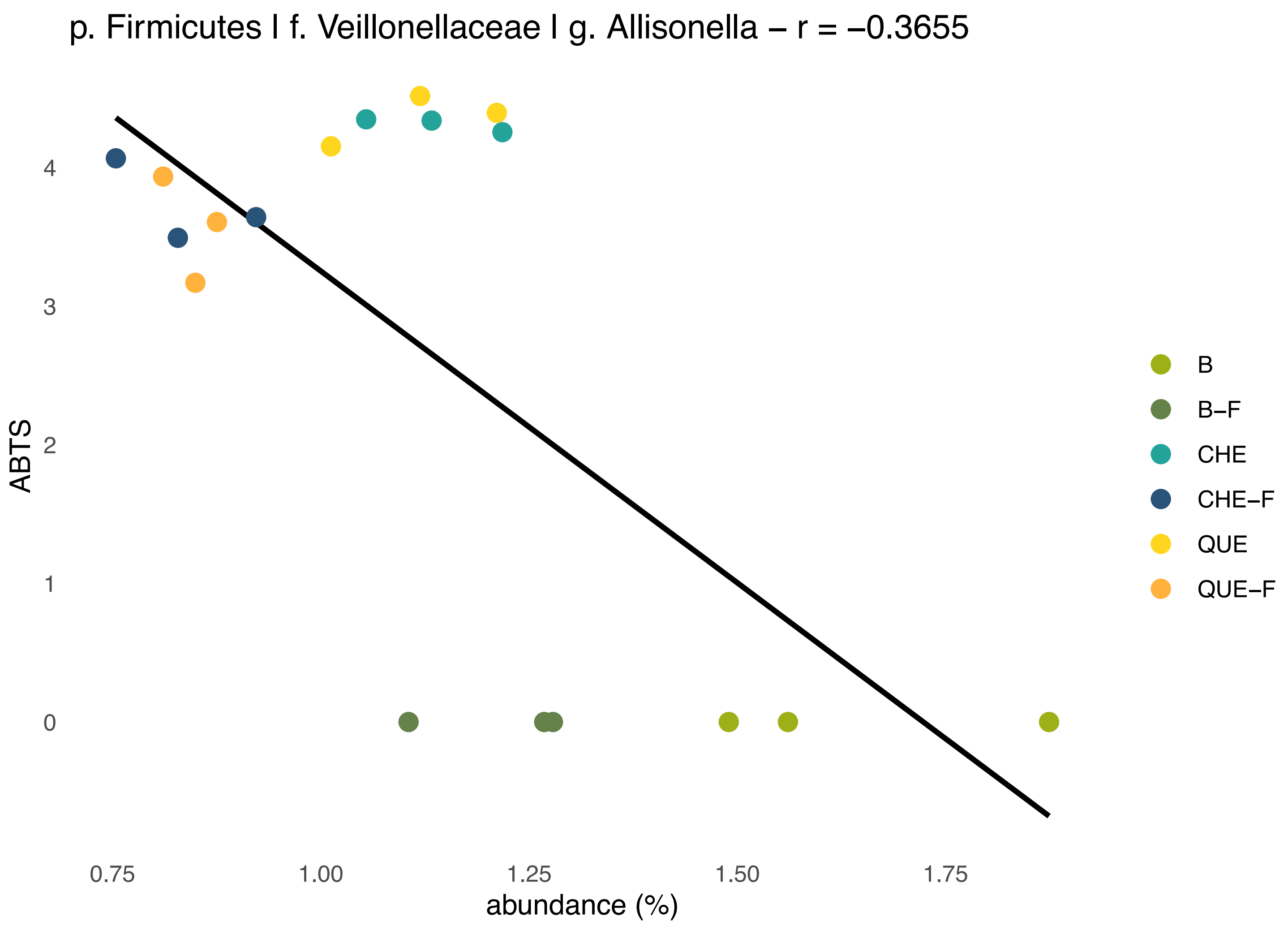

p. Firmicutes I f. Veillonellaceae I g. Allisonella – r = –0.3655
ABTS
4
3
2
1
0
0.75
1.00
1.25
1.50
1.75
abundance (%)
B
B–F
CHE
CHE–F
QUE
QUE–F

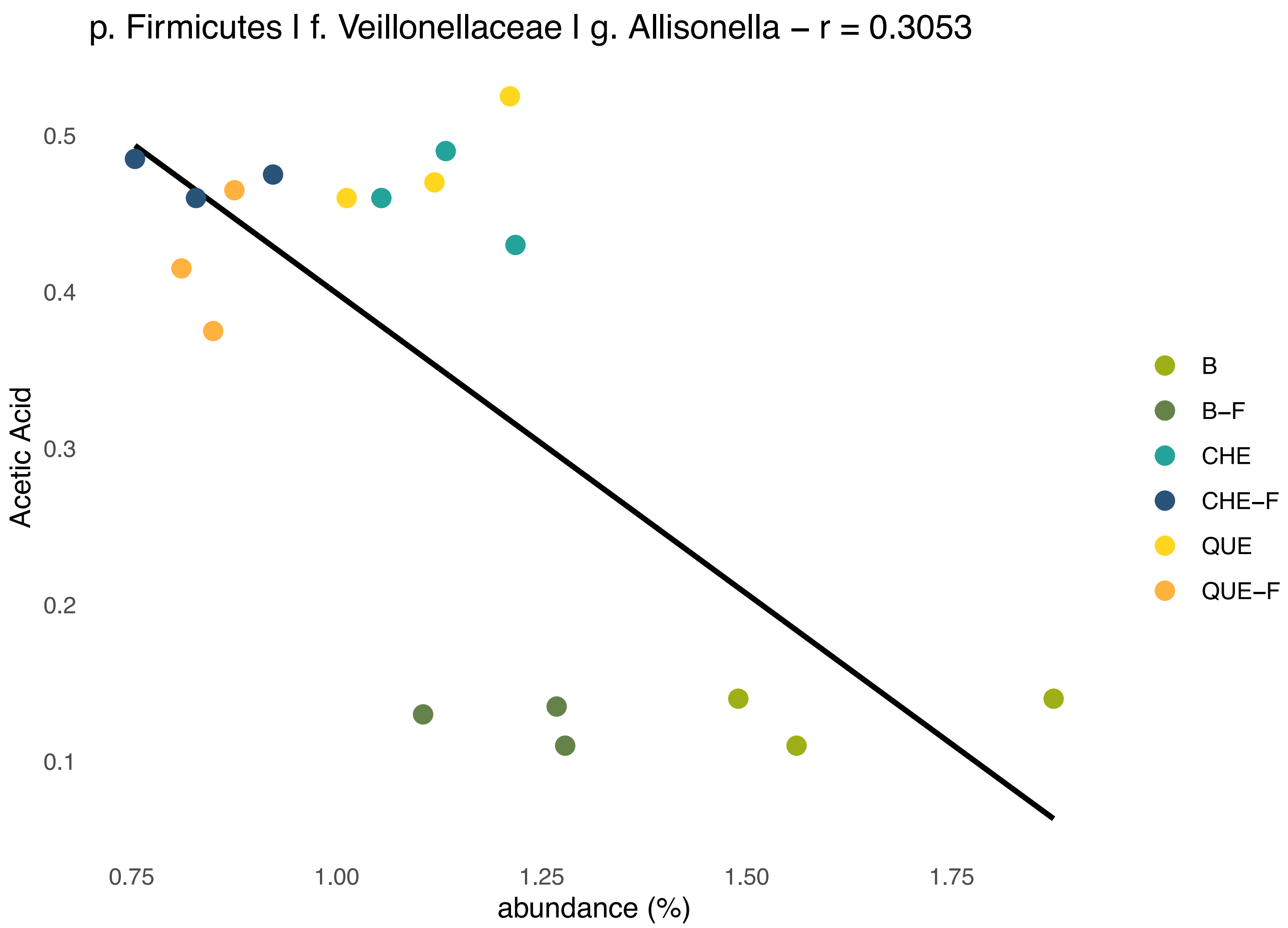

p. Firmicutes | f. Veillonellaceae | g. Allisonella − r = 0.3053
Acetic Acid
0.5
0.4
0.3
0.2
0.1
0.75
1.00
1.25
1.50
1.75
abundance (%)
B
B−F
CHE
CHE−F
QUE
QUE−F

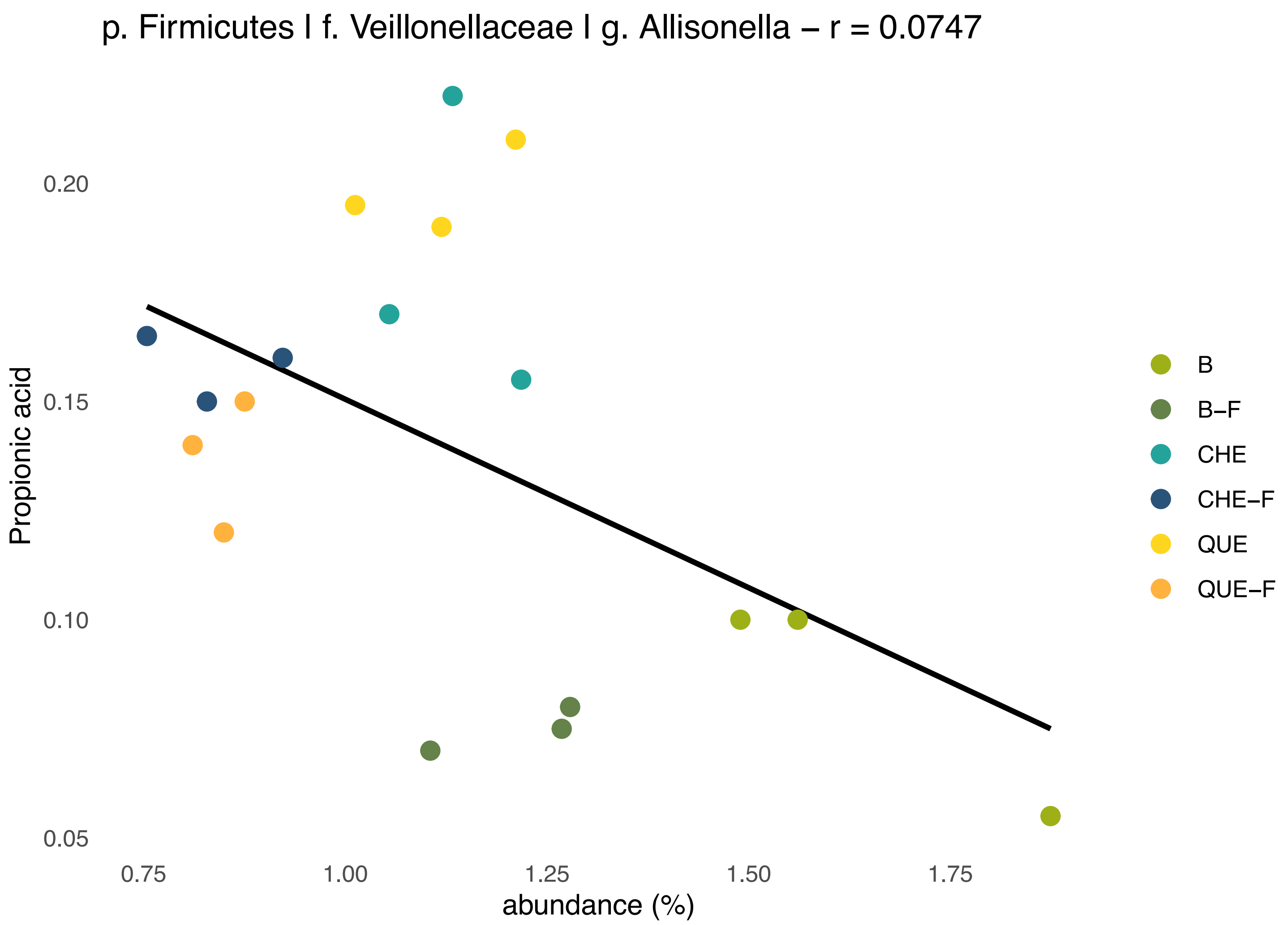

p. Firmicutes l f. Veillonellaceae l g. Allisonella – r = 0.0747
Propionic acid
0.20
0.15
0.10
0.05
0.75
1.00
1.25
1.50
1.75
abundance (%)
B
B–F
CHE
CHE–F
QUE
QUE–F

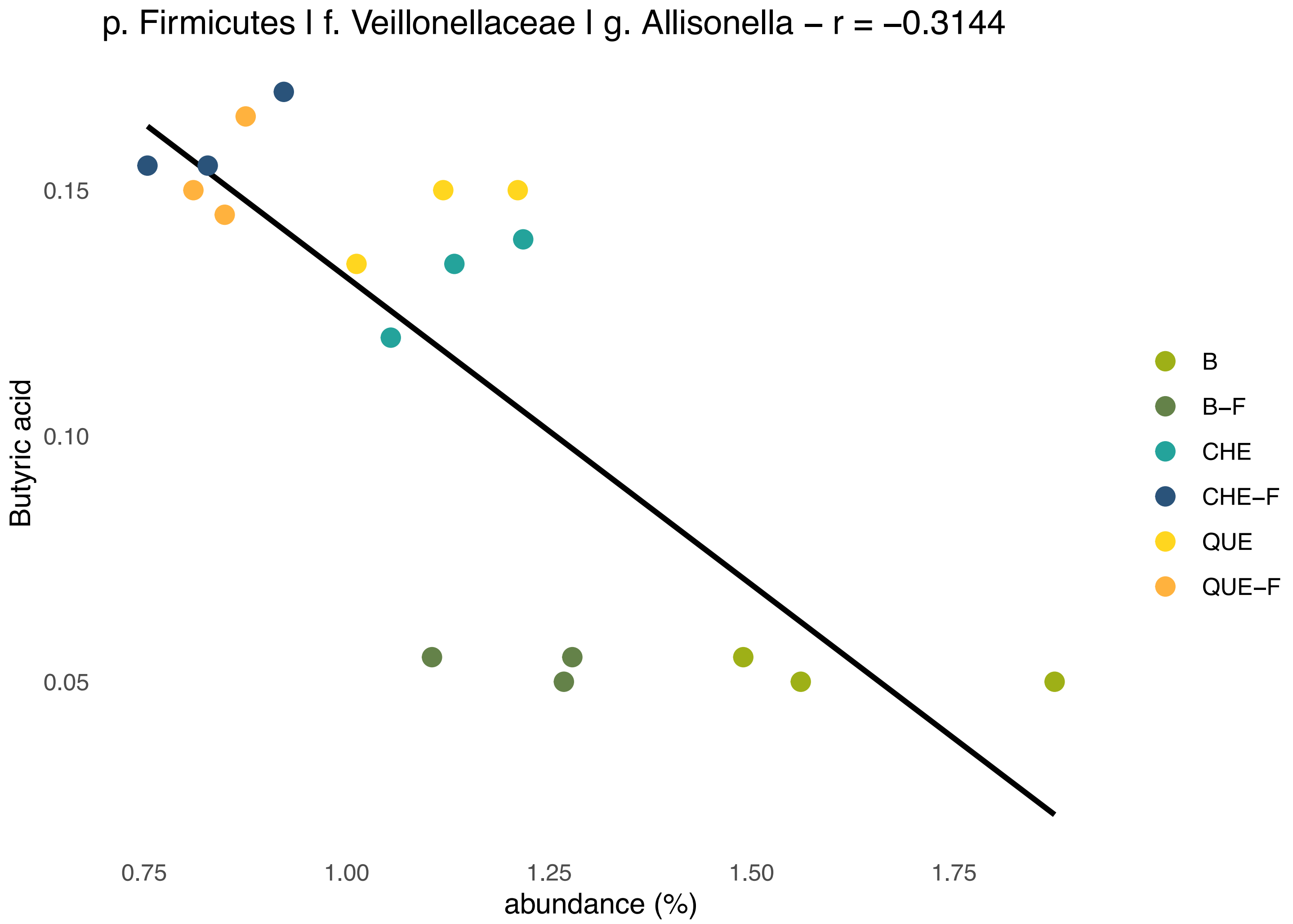
p. Firmicutes l f. Veillonellaceae l g. Allisonella – r = –0.3144
Butyric acid
0.15
0.10
0.05
0.75
1.00
1.25
1.50
1.75
abundance (%)
B
B–F
CHE
CHE–F
QUE
QUE–F

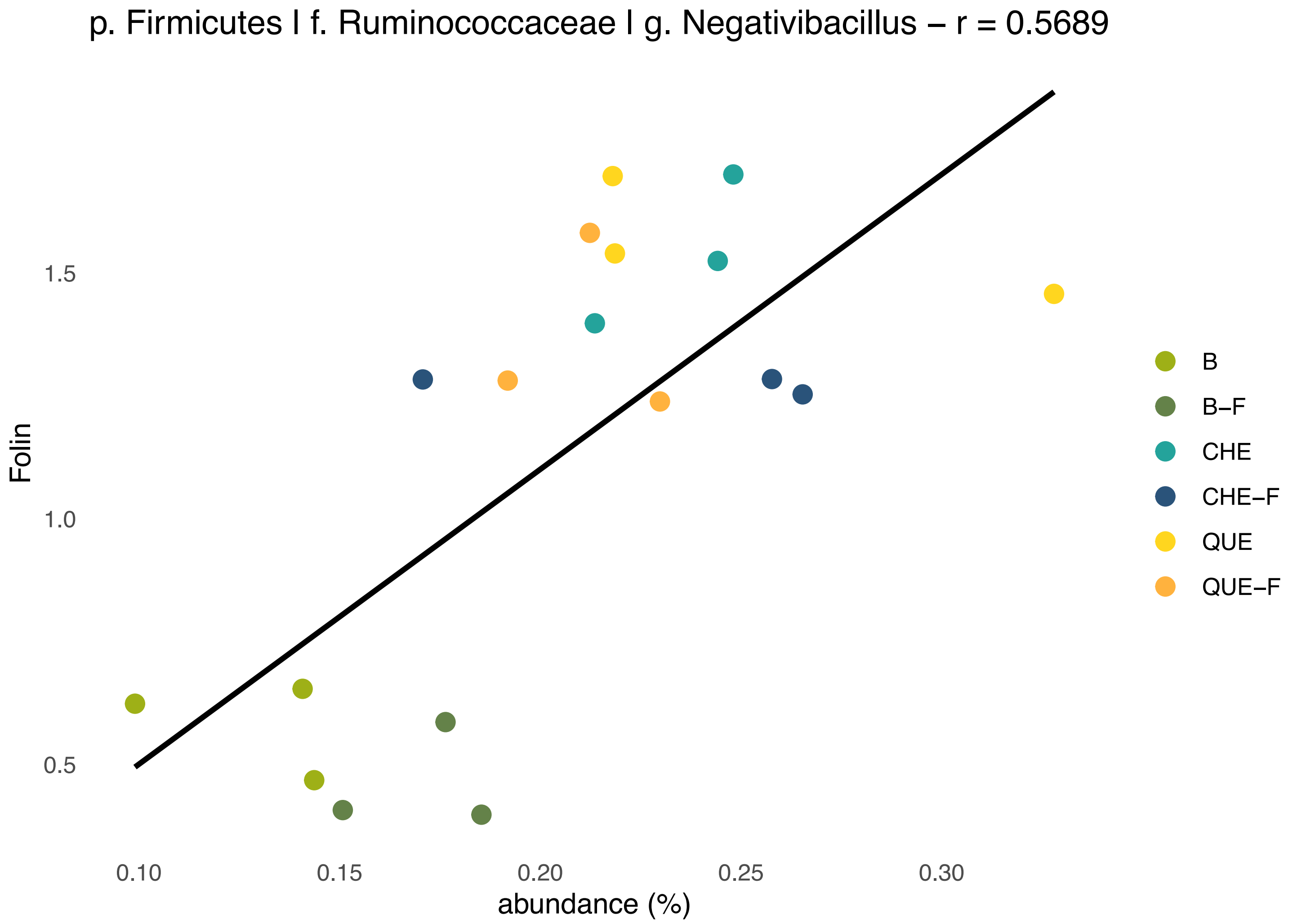

p. Firmicutes | f. Ruminococcaceae | g. Negativibacillus – r = 0.5689
Folin
1.5
1.0
0.5
0.10
0.15
0.20
0.25
0.30
abundance (%)
B
B–F
CHE
CHE–F
QUE
QUE–F

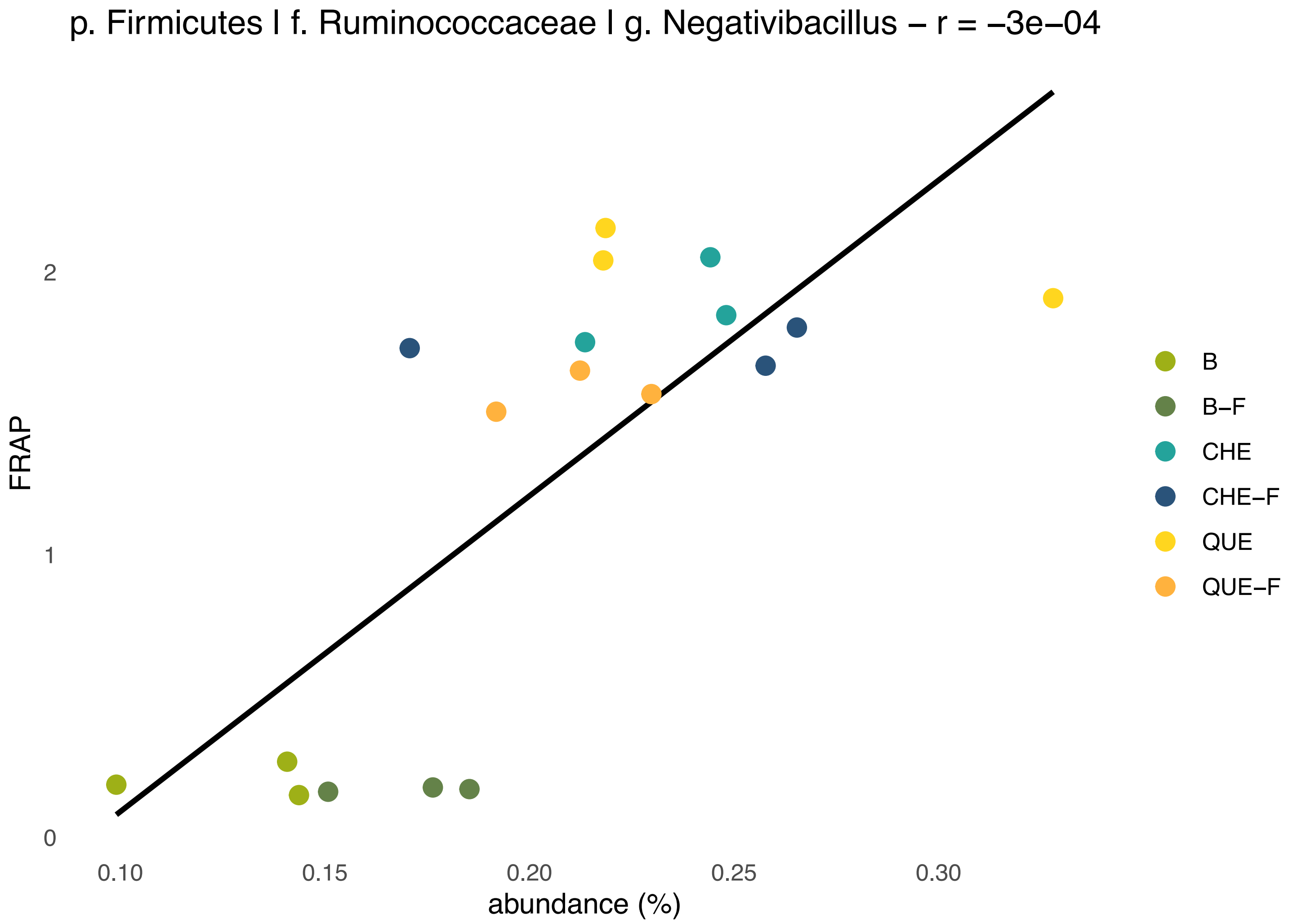
p. Firmicutes I f. Ruminococcaceae I g. Negativibacillus – r = –3e–04
FRAP
2
1
0
0.10
0.15
0.20
0.25
0.30
abundance (%)
B
B–F
CHE
CHE–F
QUE
QUE–F

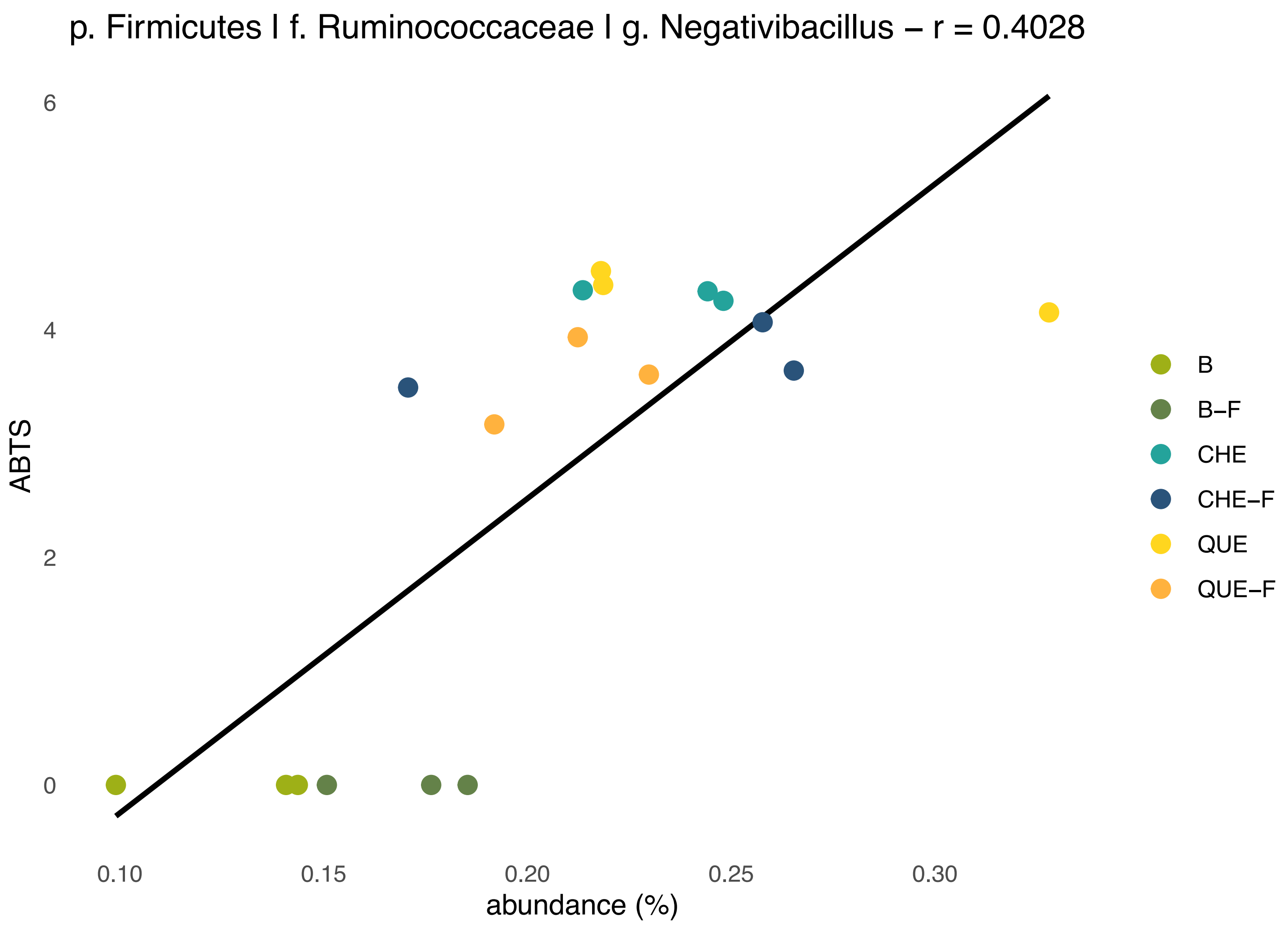
p. Firmicutes I f. Ruminococcaceae I g. Negativibacillus – r = 0.4028
ABTS
6
4
2
0
0.10
0.15
0.20
0.25
0.30
abundance (%)
B
B–F
CHE
CHE–F
QUE
QUE–F

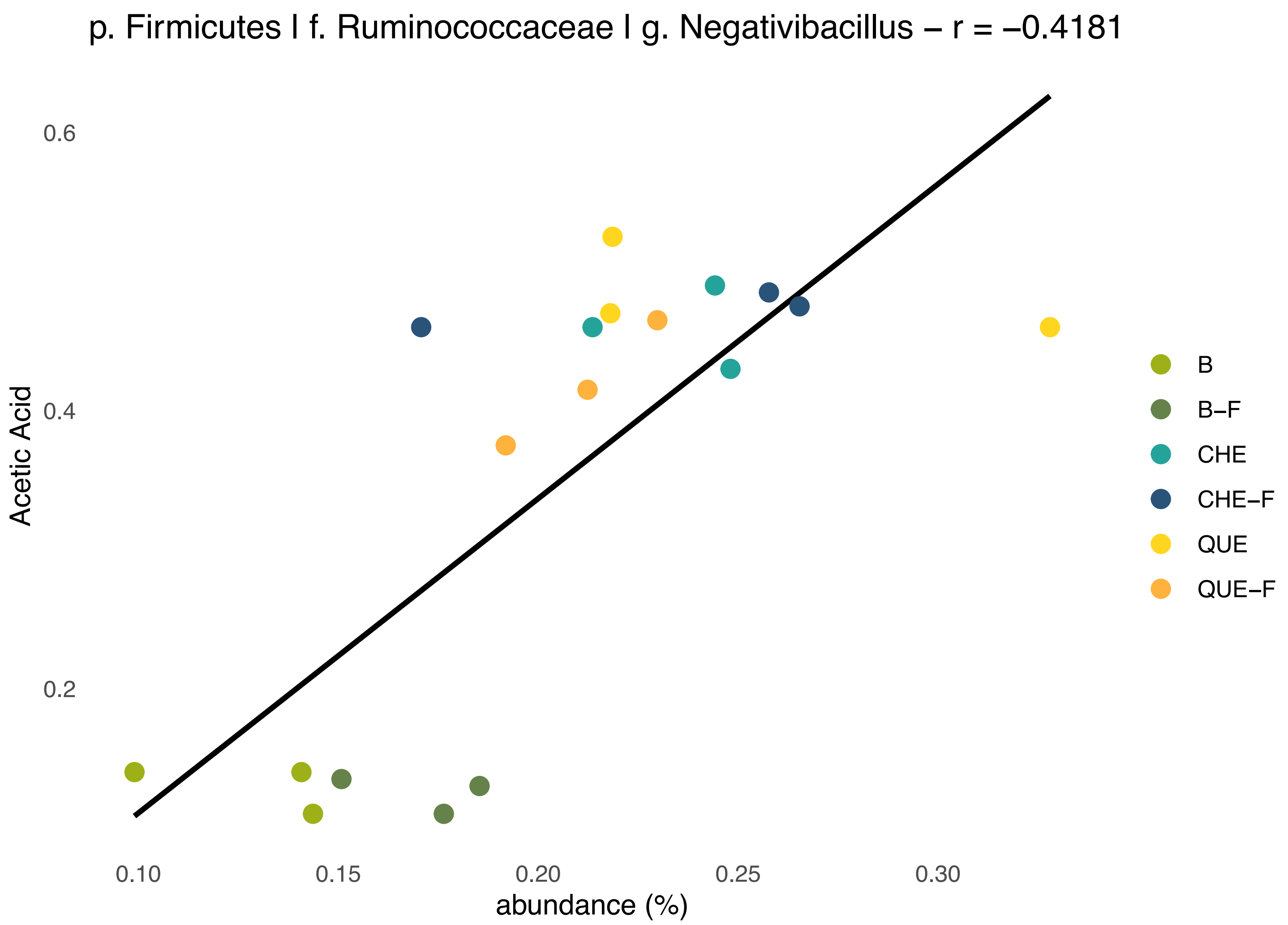
p. Firmicutes | f. Ruminococcaceae | g. Negativibacillus – r = −0.4181
Acetic Acid
0.6
0.4
0.2
0.10
0.15
0.20
0.25
0.30
abundance (%)
B
B−F
CHE
CHE−F
QUE
QUE−F

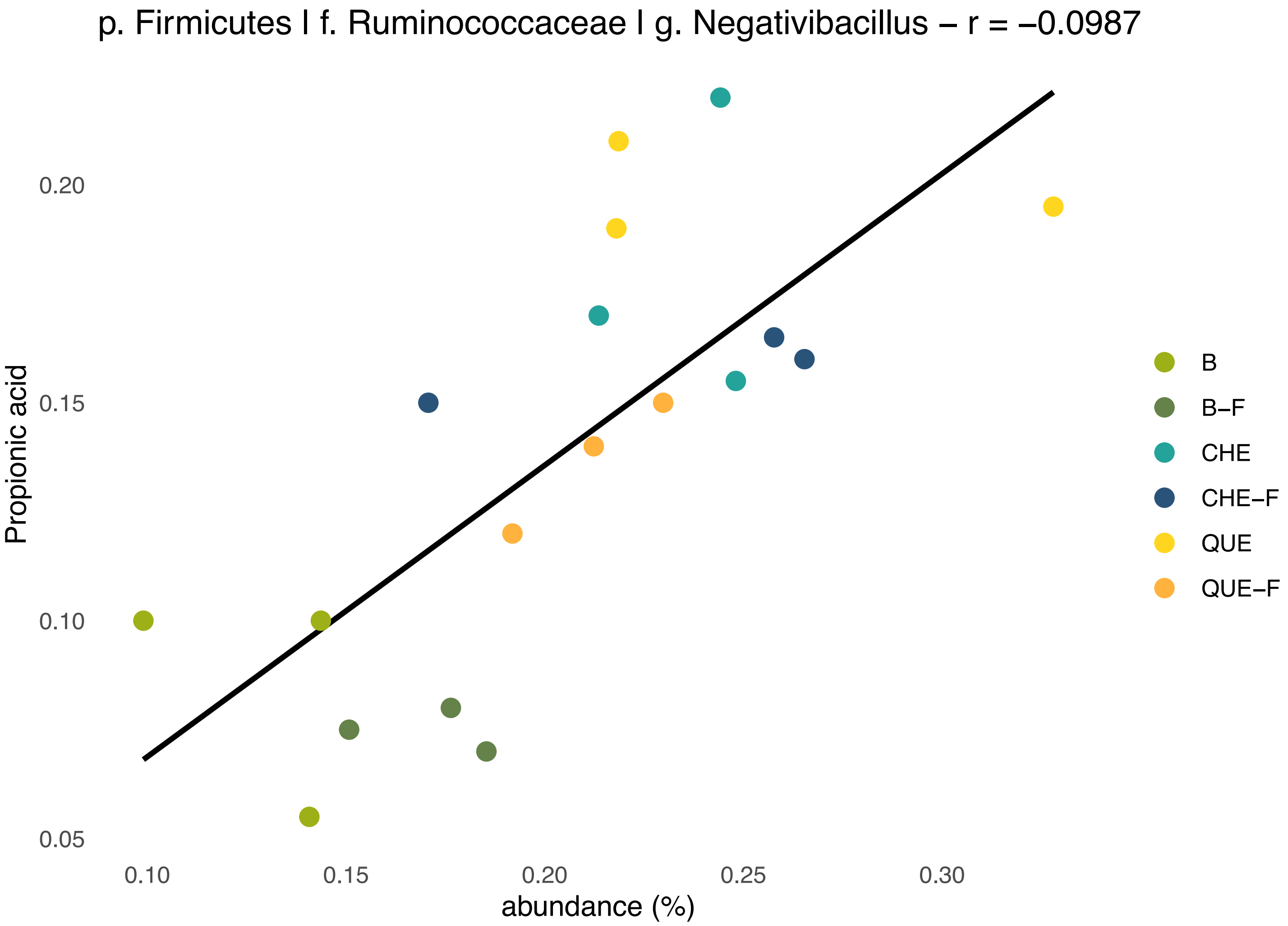

p. Firmicutes I f. Ruminococcaceae I g. Negativibacillus – r = –0.0987
Propionic acid
0.20
0.15
0.10
0.05
0.10
0.15
0.20
0.25
0.30
abundance (%)
B
B–F
CHE
CHE–F
QUE
QUE–F

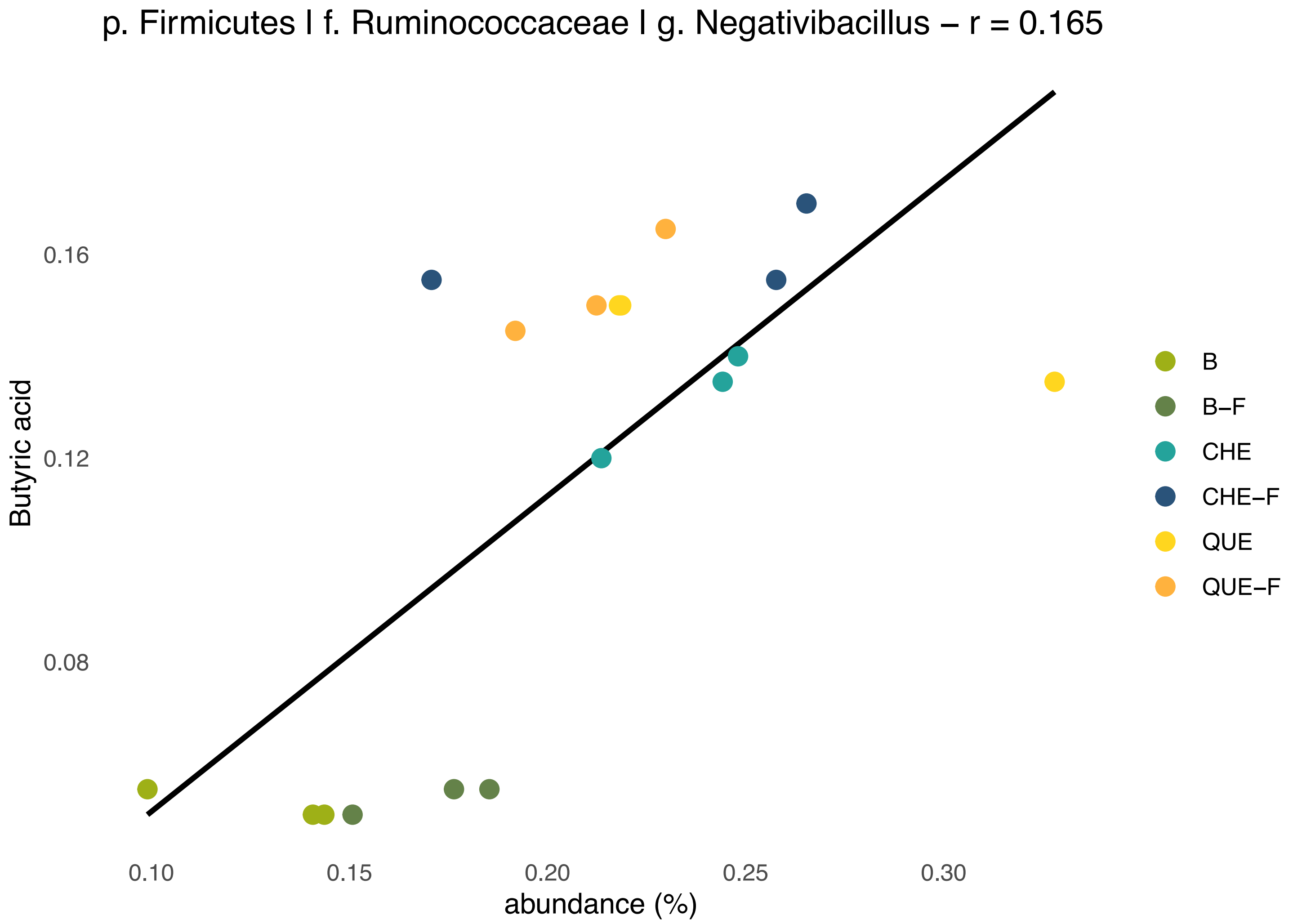

p. Firmicutes I f. Ruminococcaceae I g. Negativibacillus – r = 0.165
Butyric acid
0.16
0.12
0.08
0.10
0.15
0.20
0.25
0.30
abundance (%)
B
B–F
CHE
CHE–F
QUE
QUE–F

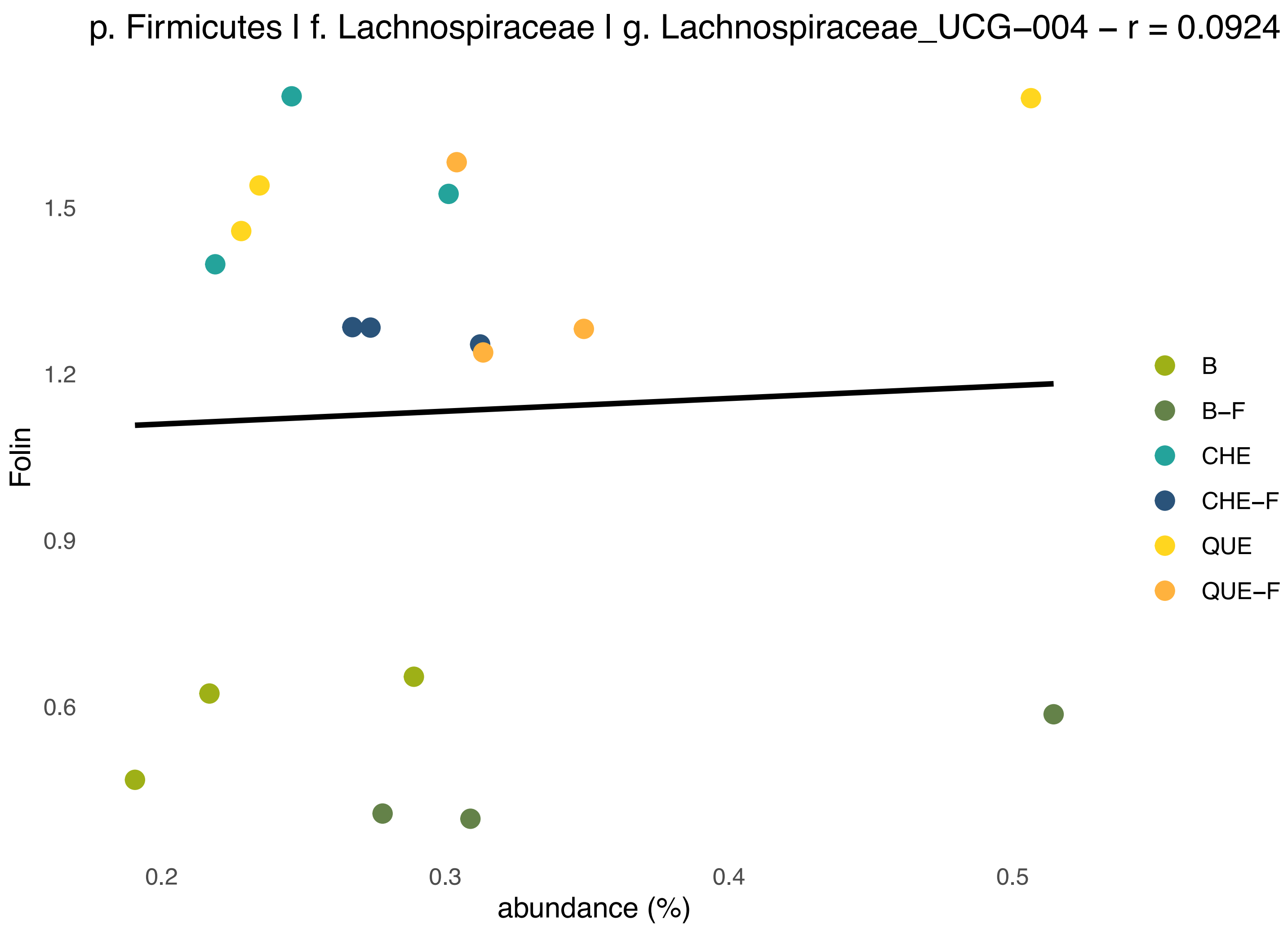

p. Firmicutes I f. Lachnospiraceae I g. Lachnospiraceae_UCG–004 – r = 0.0924
1.5
1.2
0.9
0.6
Folin
0.2
0.3
0.4
0.5
abundance (%)
B
B–F
CHE
CHE–F
QUE
QUE–F

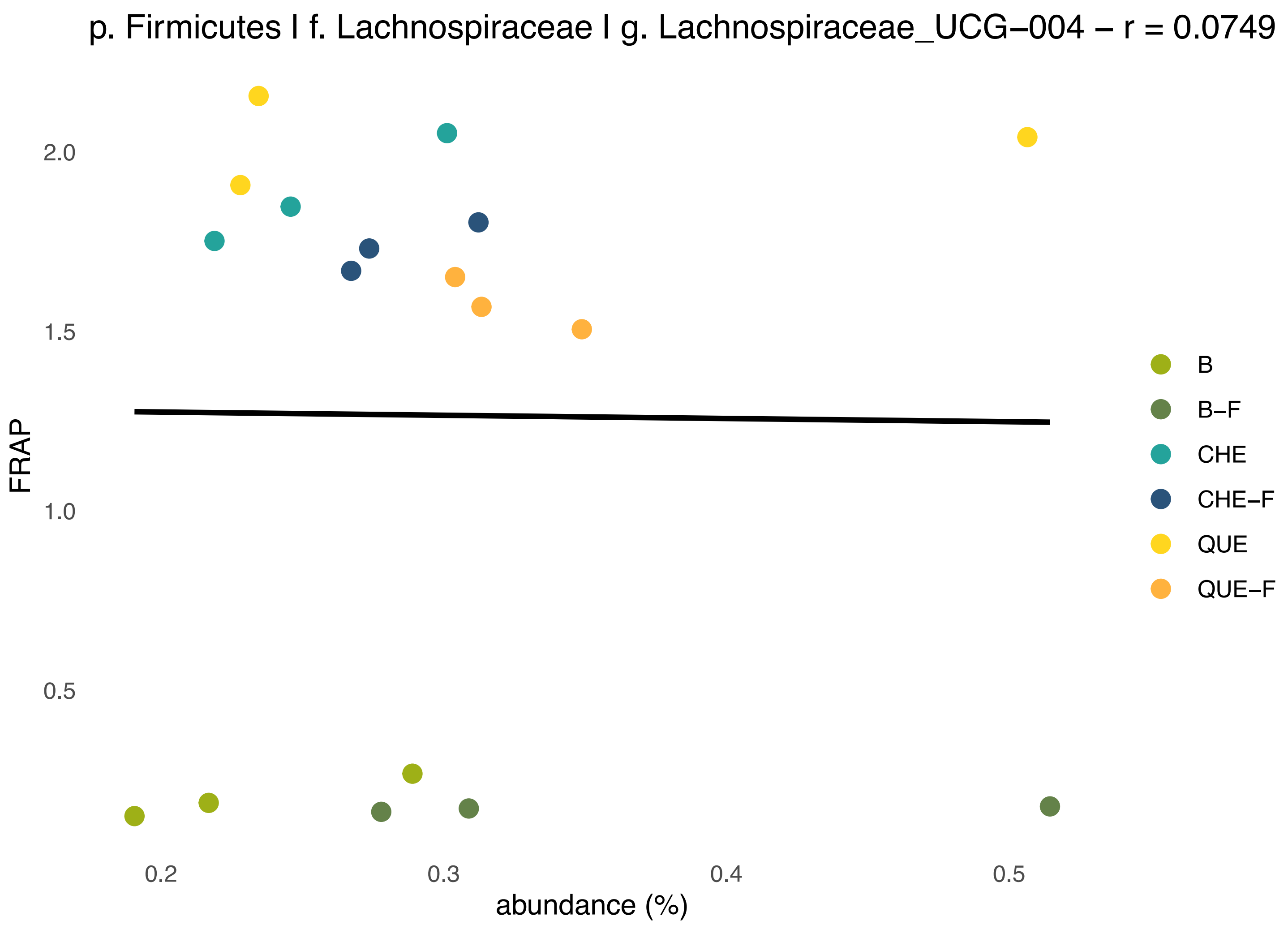
p. Firmicutes l f. Lachnospiraceae l g. Lachnospiraceae_UCG−004 − r = 0.0749
FRAP
2.0
1.5
1.0
0.5
0.2
0.3
0.4
0.5
abundance (%)
B
B−F
CHE
CHE−F
QUE
QUE−F

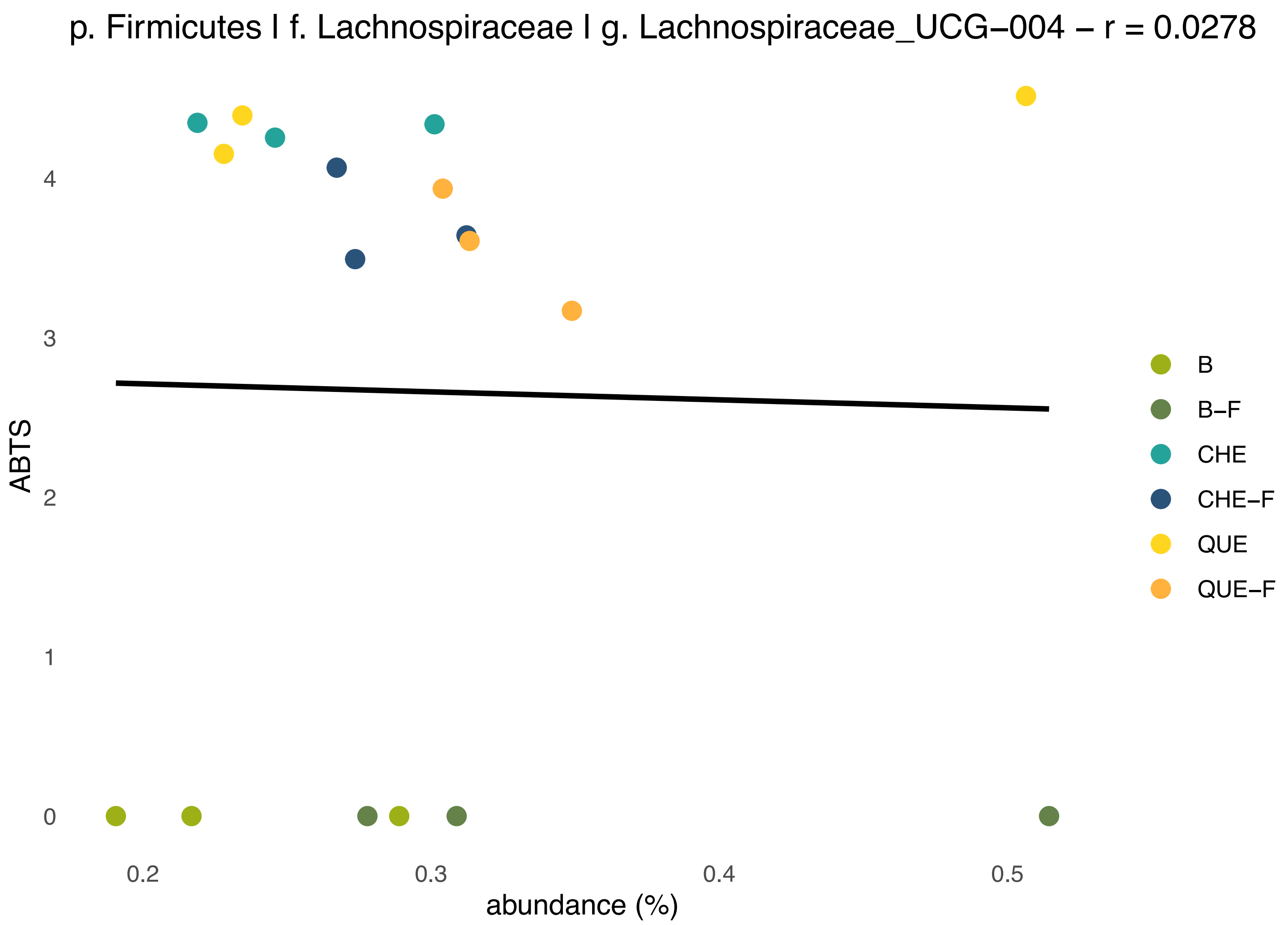
p. Firmicutes l f. Lachnospiraceae l g. Lachnospiraceae_UCG−004 − r = 0.0278
ABTS
0
1
2
3
4
0.2
0.3
0.4
0.5
abundance (%)
B
B−F
CHE
CHE−F
QUE
QUE−F

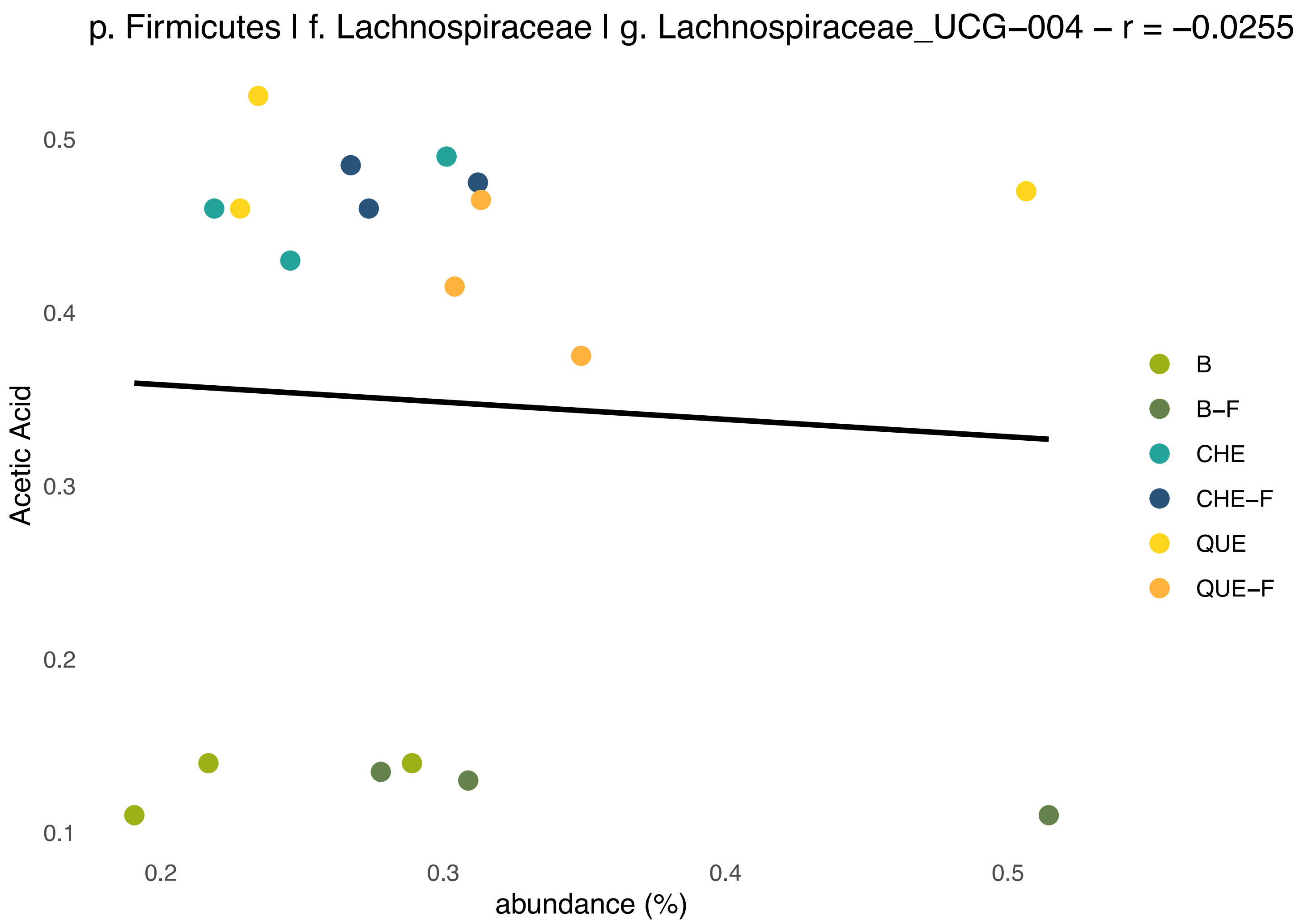

p. Firmicutes l f. Lachnospiraceae l g. Lachnospiraceae_UCG−004 − r = −0.0255
0.5
0.4
0.3
0.2
0.1
Acetic Acid
0.2
0.3
0.4
0.5
abundance (%)
B
B−F
CHE
CHE−F
QUE
QUE−F

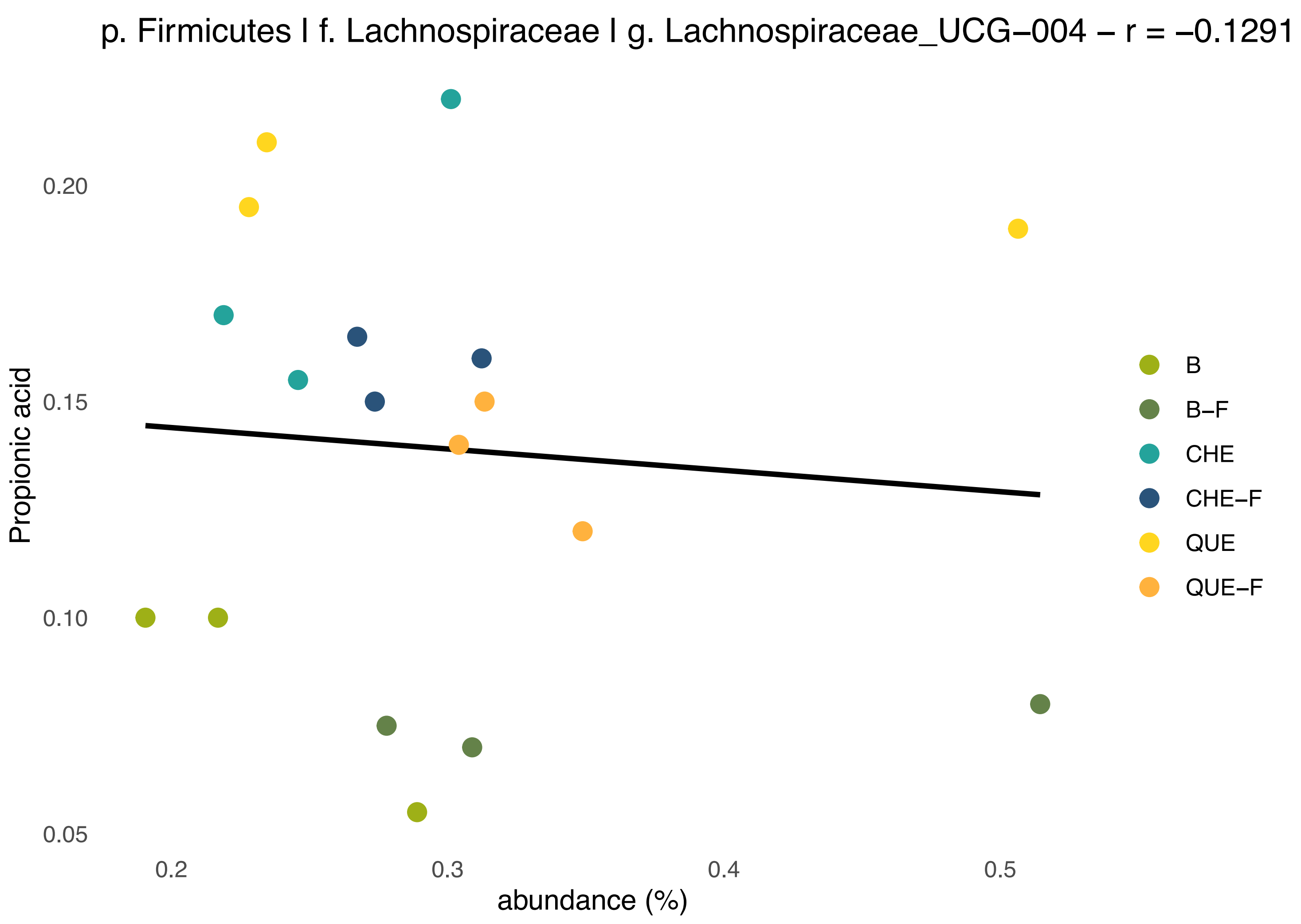

p. Firmicutes l f. Lachnospiraceae l g. Lachnospiraceae_UCG−004 − r = −0.1291
Propionic acid
0.20
0.15
0.10
0.05
0.2
0.3
0.4
0.5
abundance (%)
B
B−F
CHE
CHE−F
QUE
QUE−F

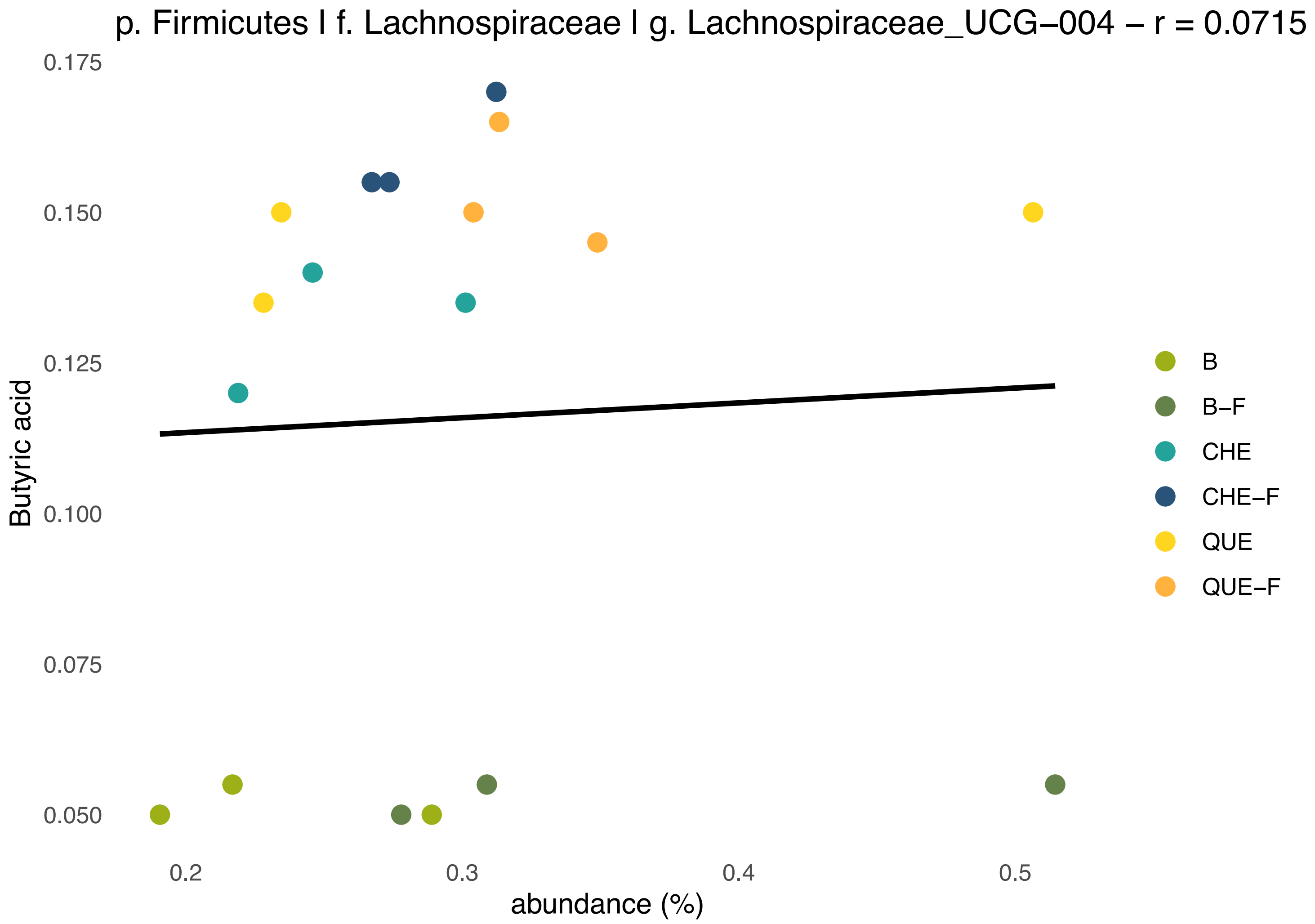
p. Firmicutes I f. Lachnospiraceae I g. Lachnospiraceae_UCG−004 − r = 0.0715
0.175
0.150
0.125
0.100
0.075
0.050
Butyric acid
0.2
0.3
0.4
0.5
abundance (%)
B
B−F
CHE
CHE−F
QUE
QUE−F

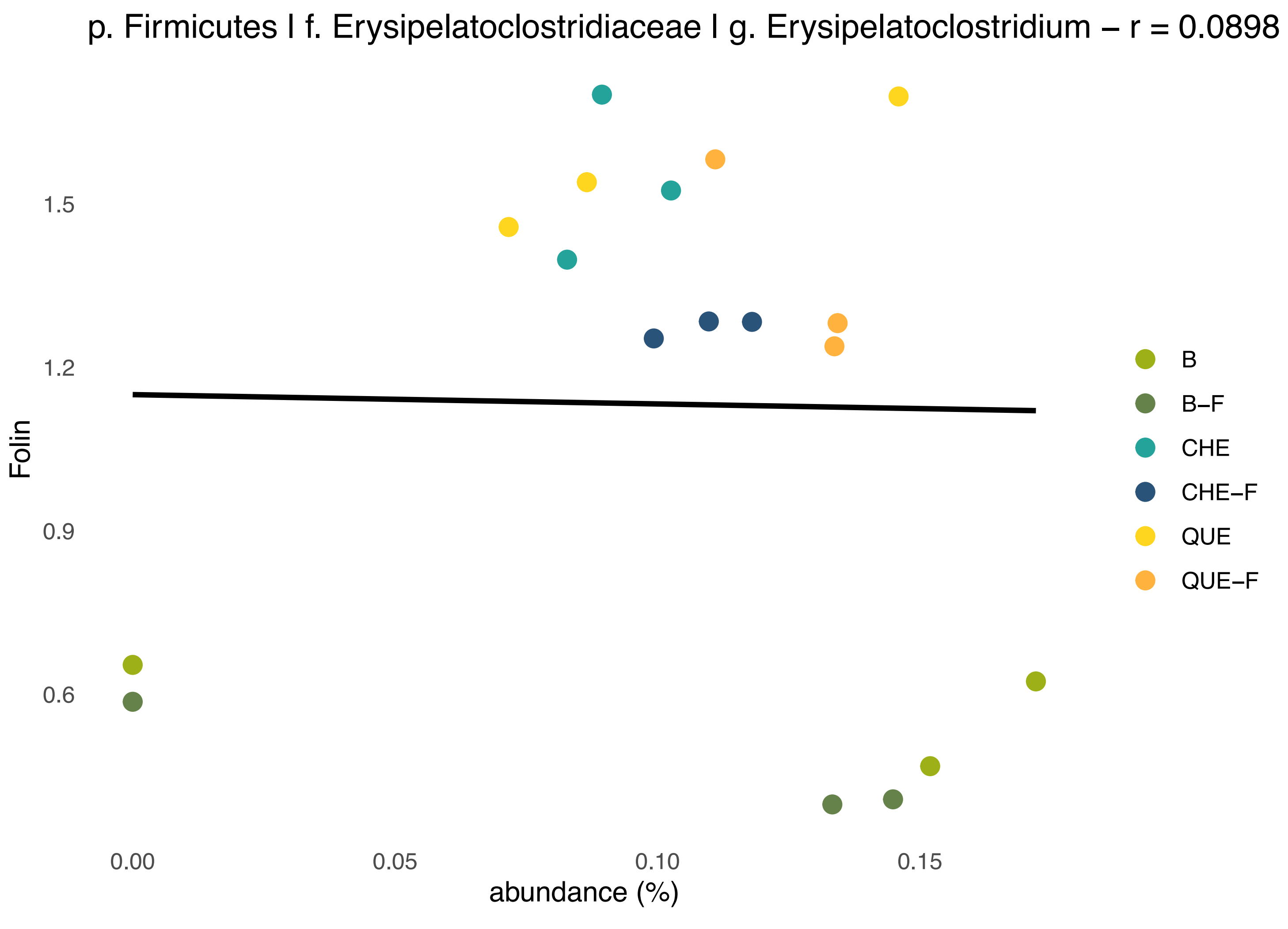

p. Firmicutes l f. Erysipelatoclostridiaceae l g. Erysipelatoclostridium – r = 0.0898
1.5
1.2
0.9
0.6
Folin
0.00
0.05
0.10
0.15
abundance (%)
B
B–F
CHE
CHE–F
QUE
QUE–F

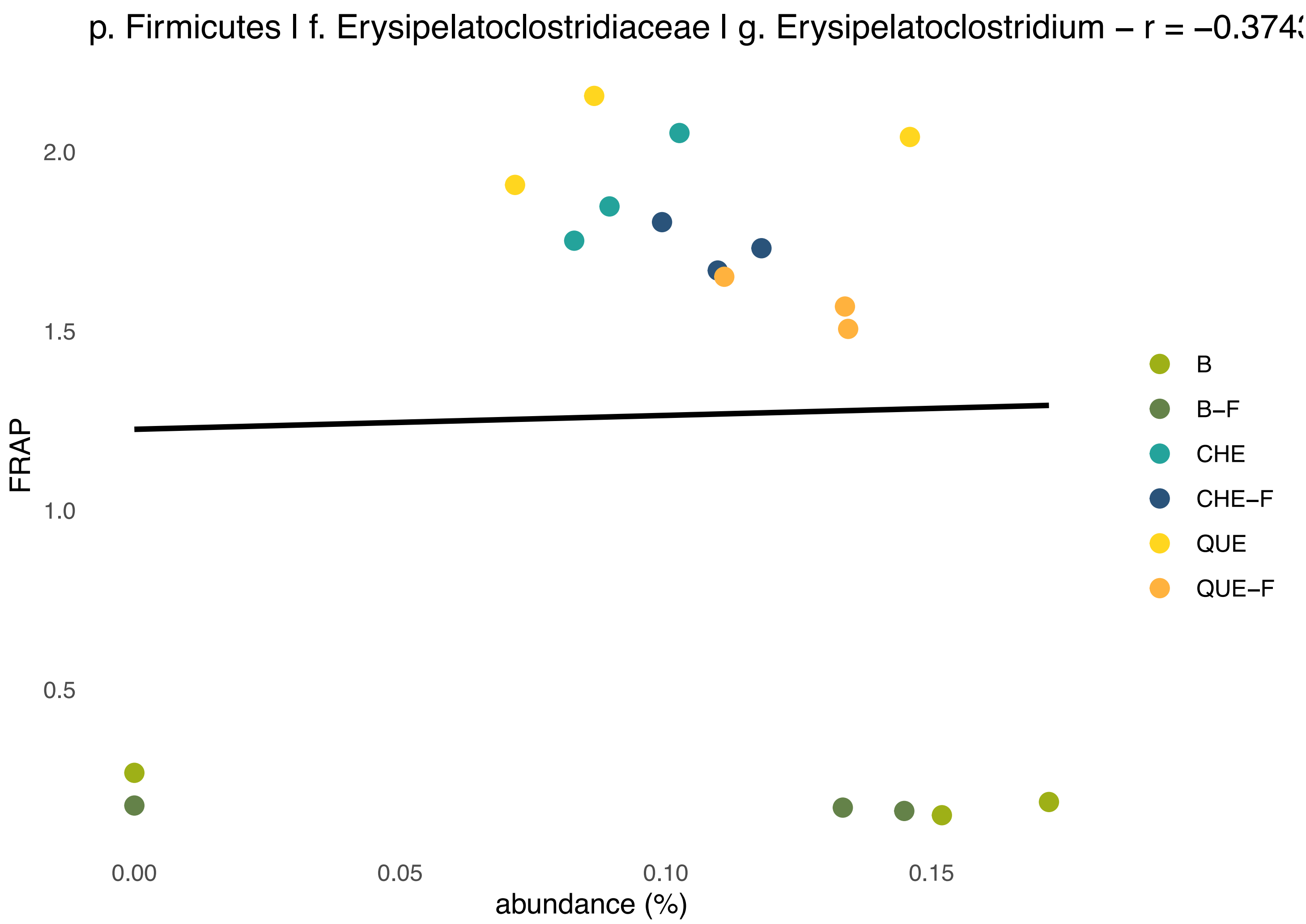
p. Firmicutes I f. Erysipelatoclostridiaceae I g. Erysipelatoclostridium – r = –0.374
FRAP
2.0
1.5
1.0
0.5
0.00
0.05
0.10
0.15
abundance (%)
B
B–F
CHE
CHE–F
QUE
QUE–F

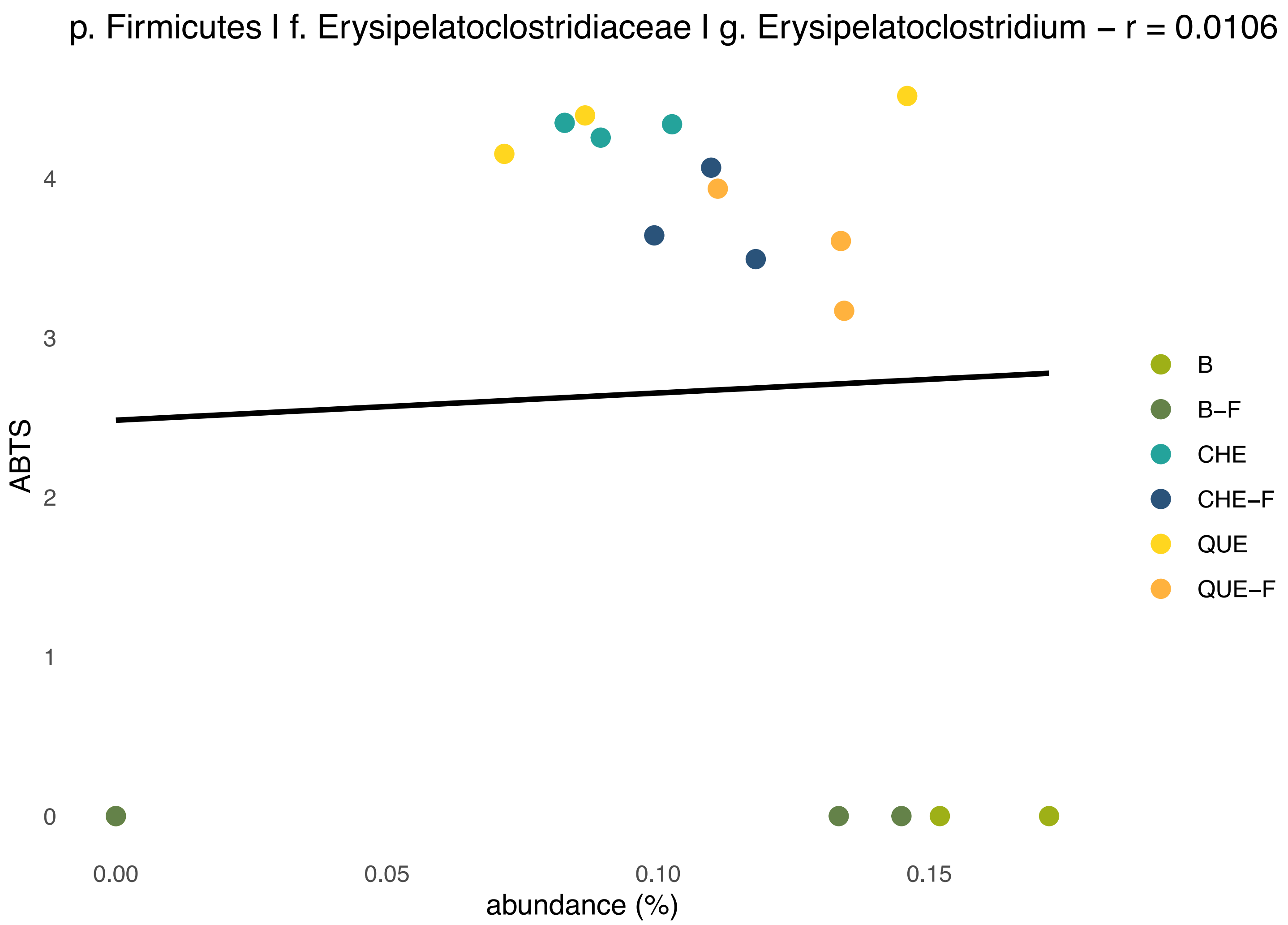

p. Firmicutes I f. Erysipelatoclostridiaceae I g. Erysipelatoclostridium – r = 0.0106
ABTS
4
3
2
1
0
0.00
0.05
0.10
0.15
abundance (%)
B
B–F
CHE
CHE–F
QUE
QUE–F

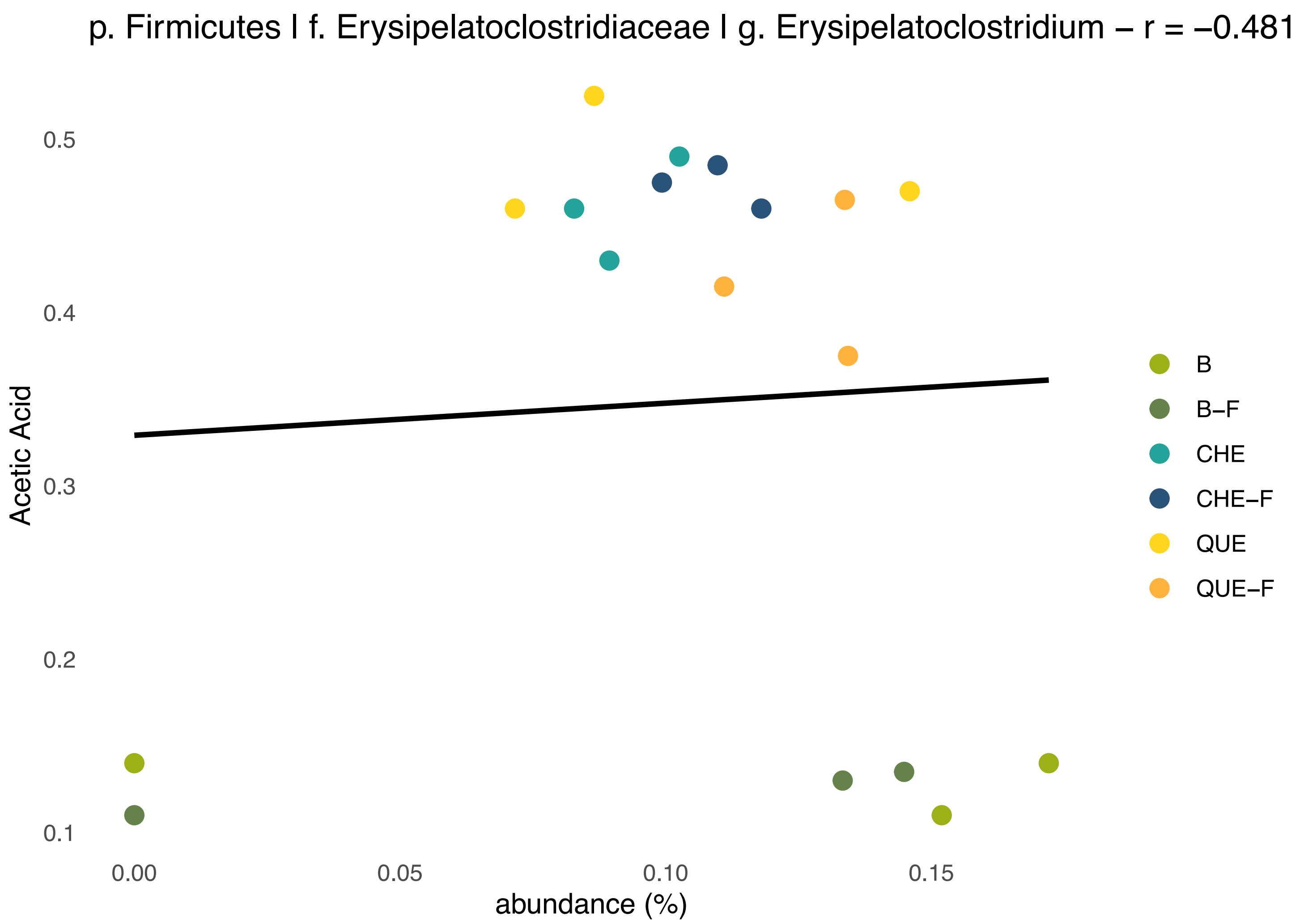

p. Firmicutes | f. Erysipelatoclostridiaceae | g. Erysipelatoclostridium – r = –0.481
Acetic Acid
0.5
0.4
0.3
0.2
0.1
0.00
0.05
0.10
0.15
abundance (%)
B
B–F
CHE
CHE–F
QUE
QUE–F

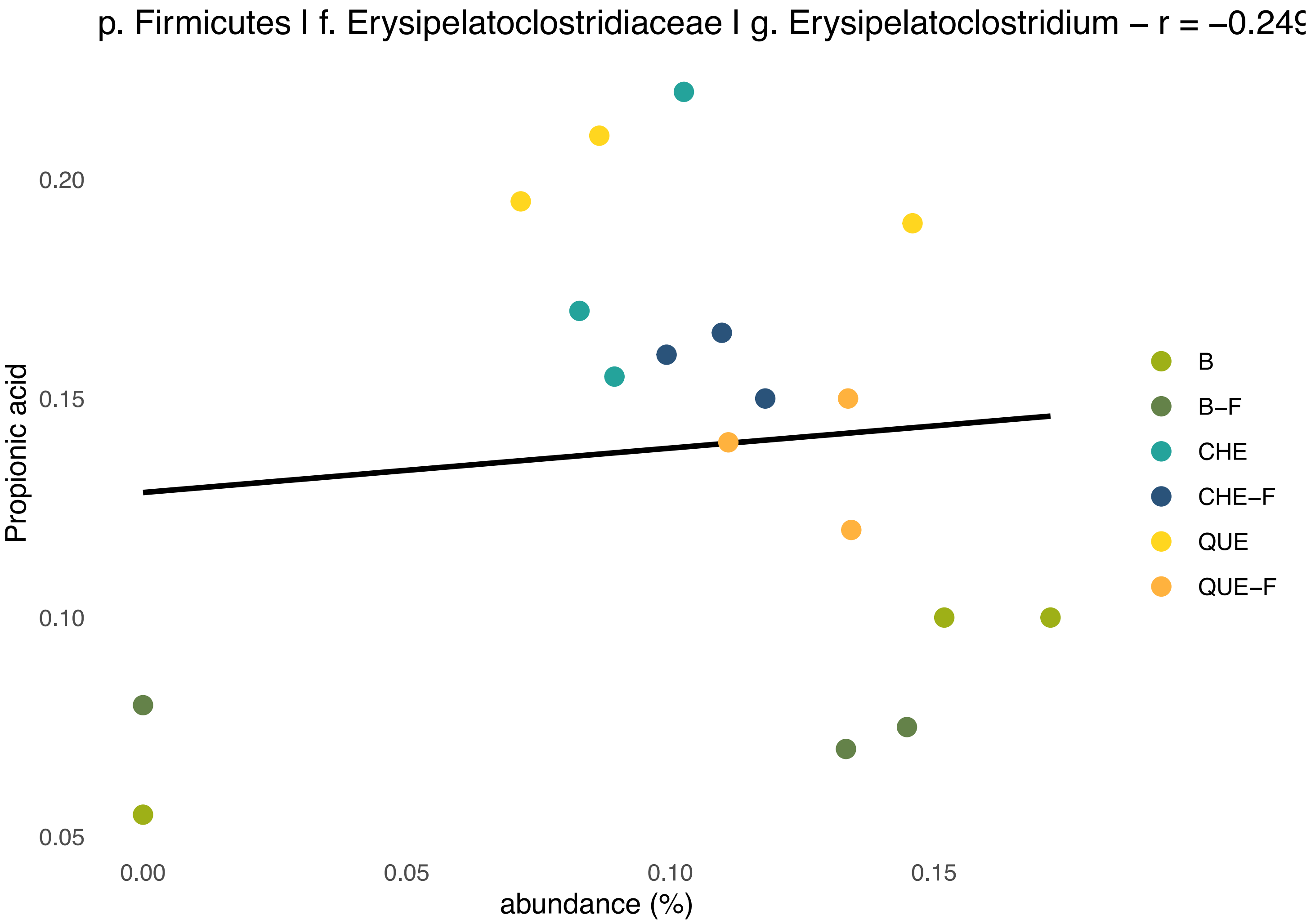

p. Firmicutes I f. Erysipelatoclostridiaceae I g. Erysipelatoclostridium – r = –0.249
Propionic acid
0.20
0.15
0.10
0.05
0.00
0.05
0.10
0.15
abundance (%)
B
B–F
CHE
CHE–F
QUE
QUE–F

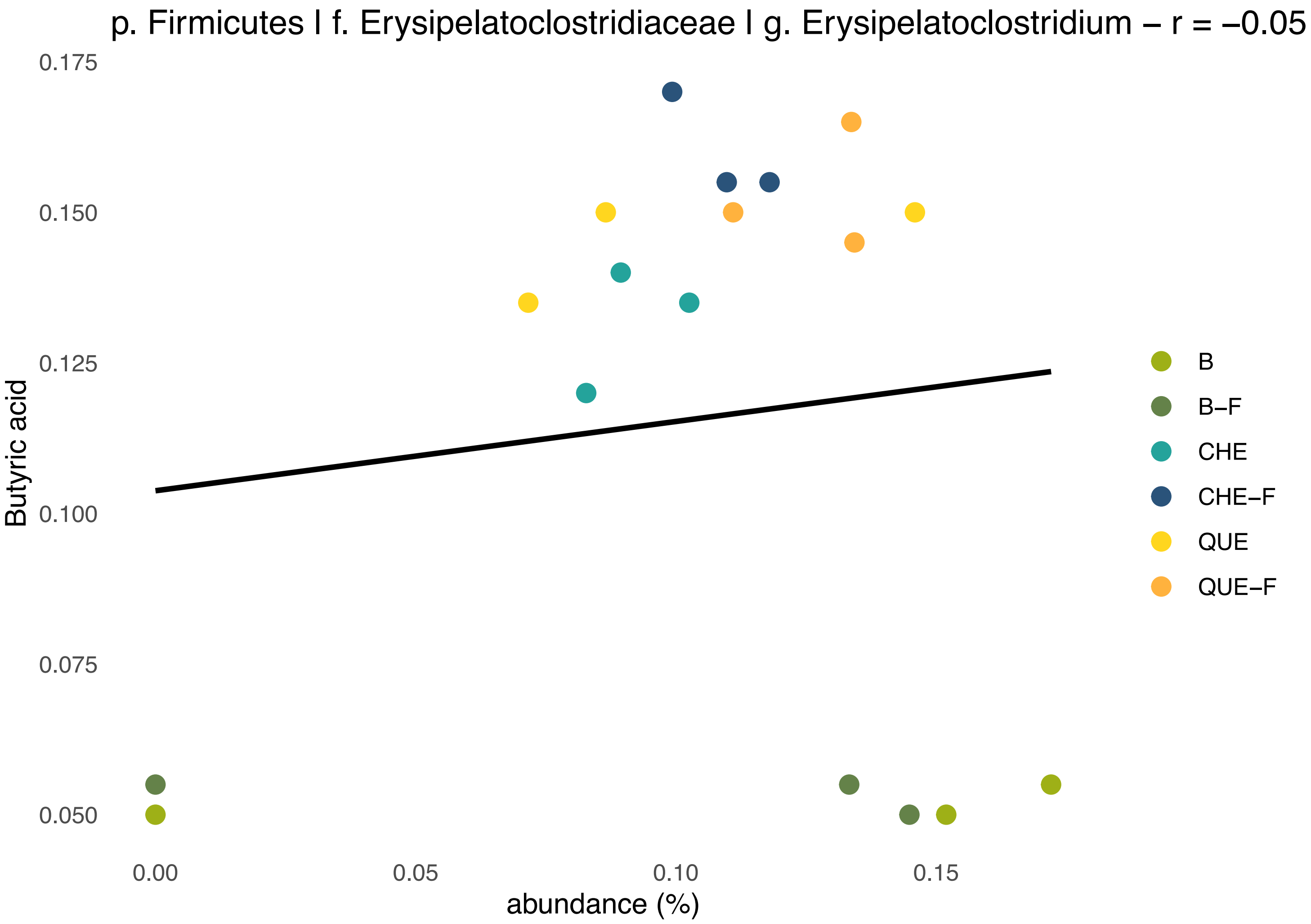
p. Firmicutes I f. Erysipelatoclostridiaceae I g. Erysipelatoclostridium – r = –0.05
0.175
0.150
0.125
0.100
0.075
0.050
Butyric acid
0.00
0.05
0.10
0.15
abundance (%)
B
B–F
CHE
CHE–F
QUE
QUE–F

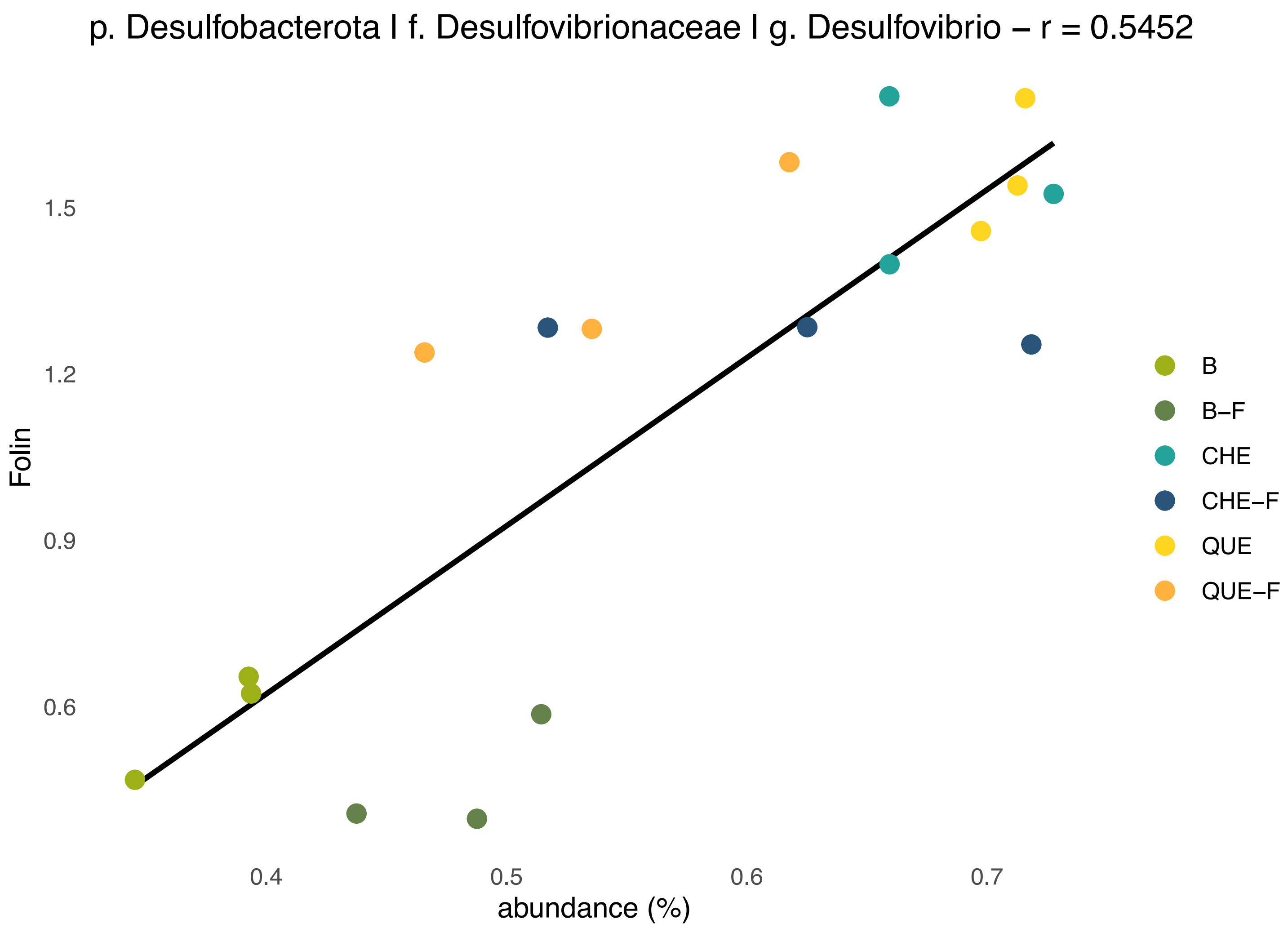
p. Desulfobacterota I f. Desulfovibrionaceae I g. Desulfovibrio − r = 0.5452
1.5
1.2
0.9
0.6
Folin
0.4
0.5
0.6
0.7
abundance (%)
B
B−F
CHE
CHE−F
QUE
QUE−F

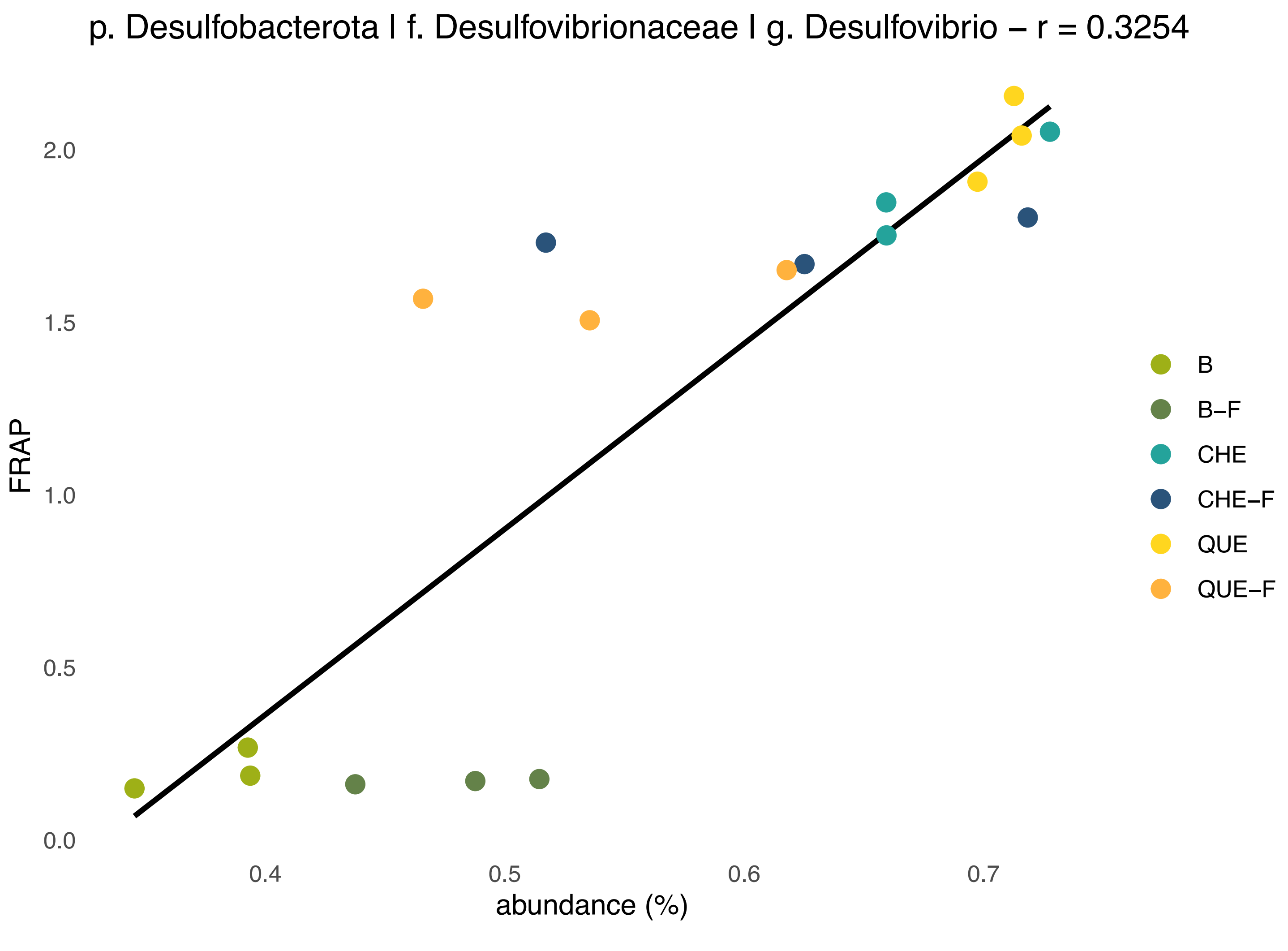
p. Desulfobacterota I f. Desulfovibrionaceae I g. Desulfovibrio − r = 0.3254
FRAP
2.0
1.5
1.0
0.5
0.0
0.4
0.5
0.6
0.7
abundance (%)
B
B−F
CHE
CHE−F
QUE
QUE−F

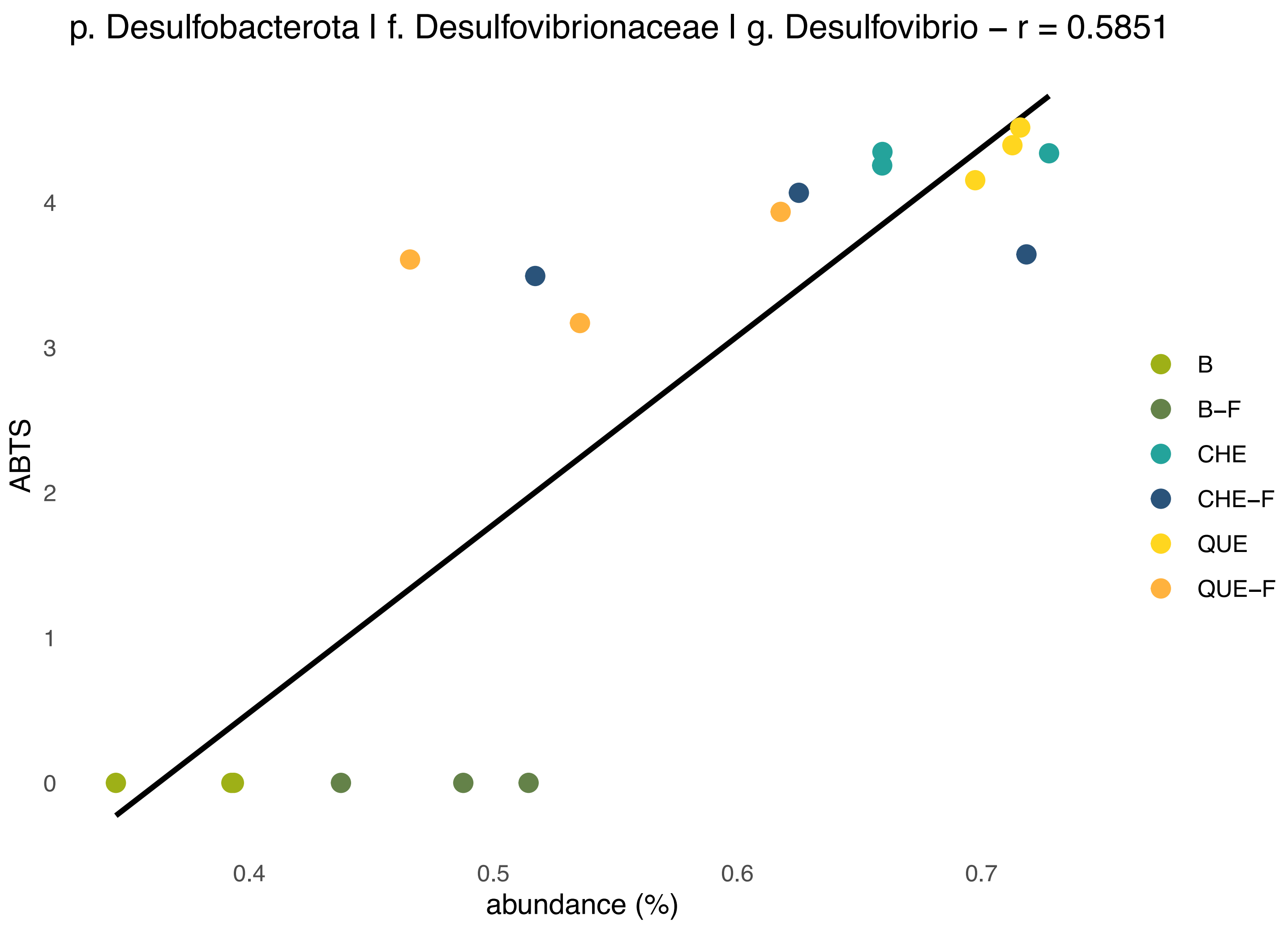
p. Desulfobacterota I f. Desulfovibrionaceae I g. Desulfovibrio – r = 0.5851
ABTS
0
1
2
3
4
0.4
0.5
0.6
0.7
abundance (%)
B
B–F
CHE
CHE–F
QUE
QUE–F

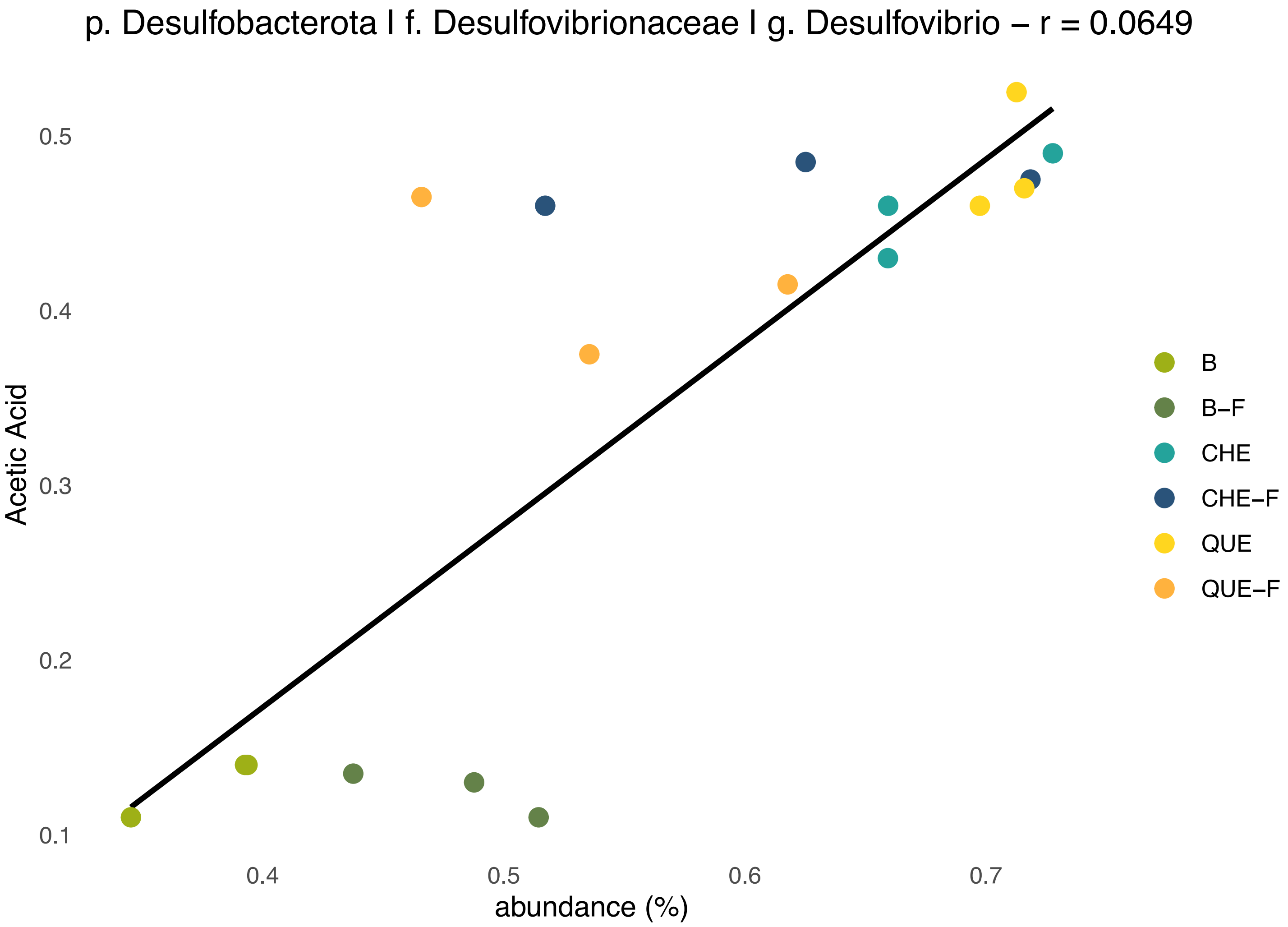
p. Desulfobacterota I f. Desulfovibrionaceae I g. Desulfovibrio − r = 0.0649
Acetic Acid
0.5
0.4
0.3
0.2
0.1
0.4
0.5
0.6
0.7
abundance (%)
B
B−F
CHE
CHE−F
QUE
QUE−F

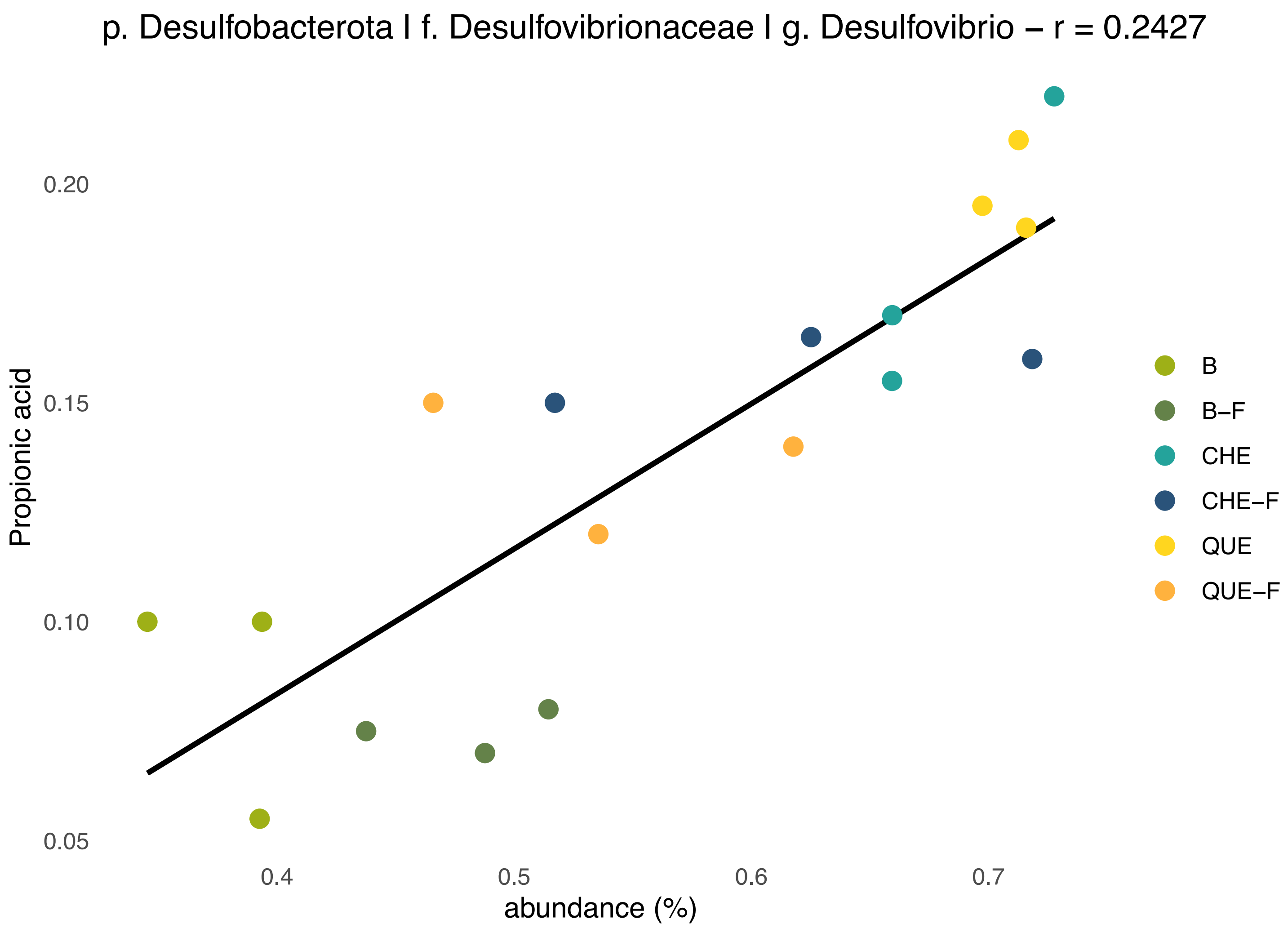
p. Desulfobacterota I f. Desulfovibrionaceae I g. Desulfovibrio − r = 0.2427
Propionic acid
0.20
0.15
0.10
0.05
0.4
0.5
0.6
0.7
abundance (%)
B
B−F
CHE
CHE−F
QUE
QUE−F

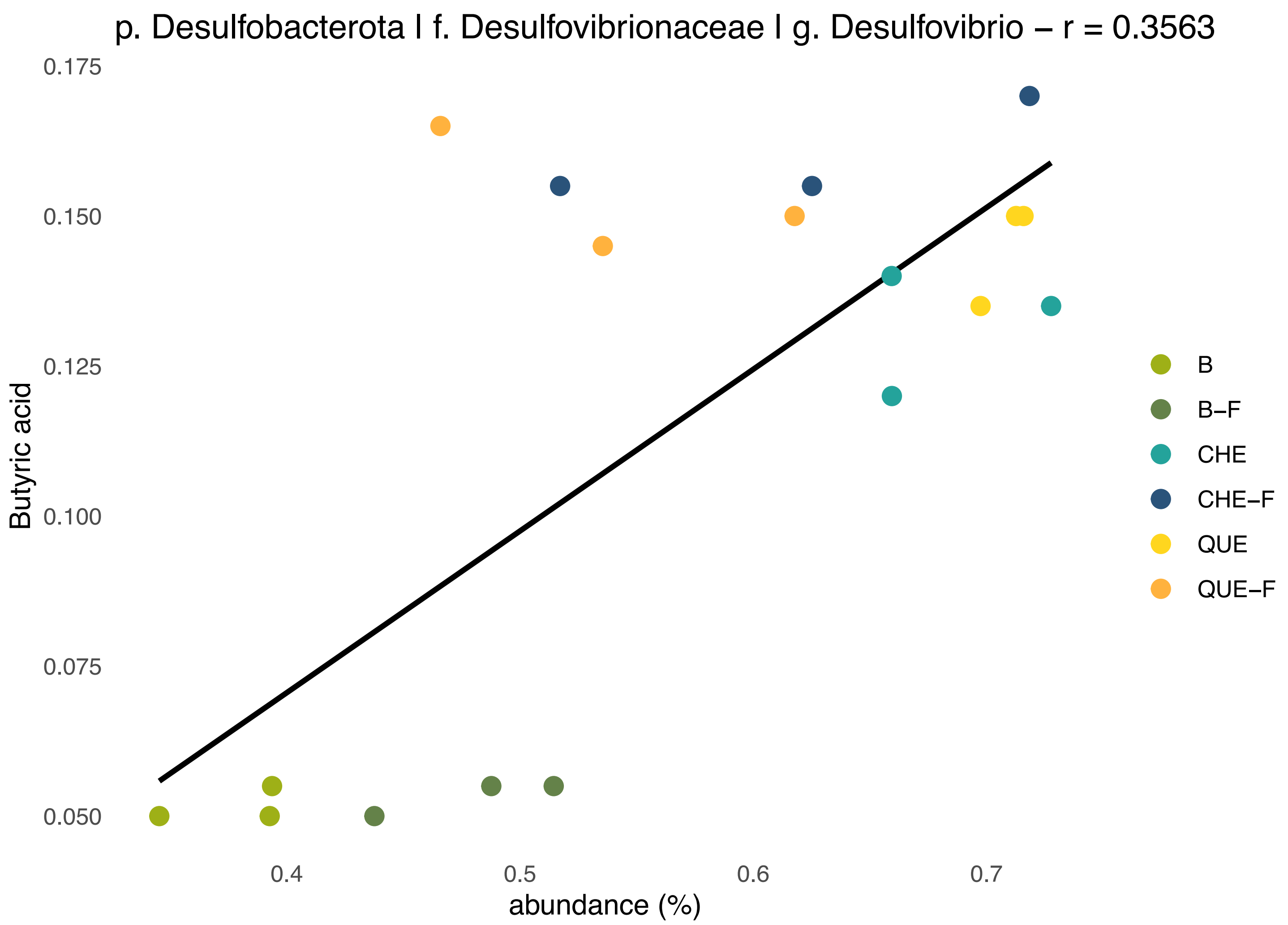

p. Desulfobacterota I f. Desulfovibrionaceae I g. Desulfovibrio – r = 0.3563
0.175
0.150
0.125
0.100
0.075
0.050
Butyric acid
0.4
0.5
0.6
0.7
abundance (%)
B
B–F
CHE
CHE–F
QUE
QUE–F

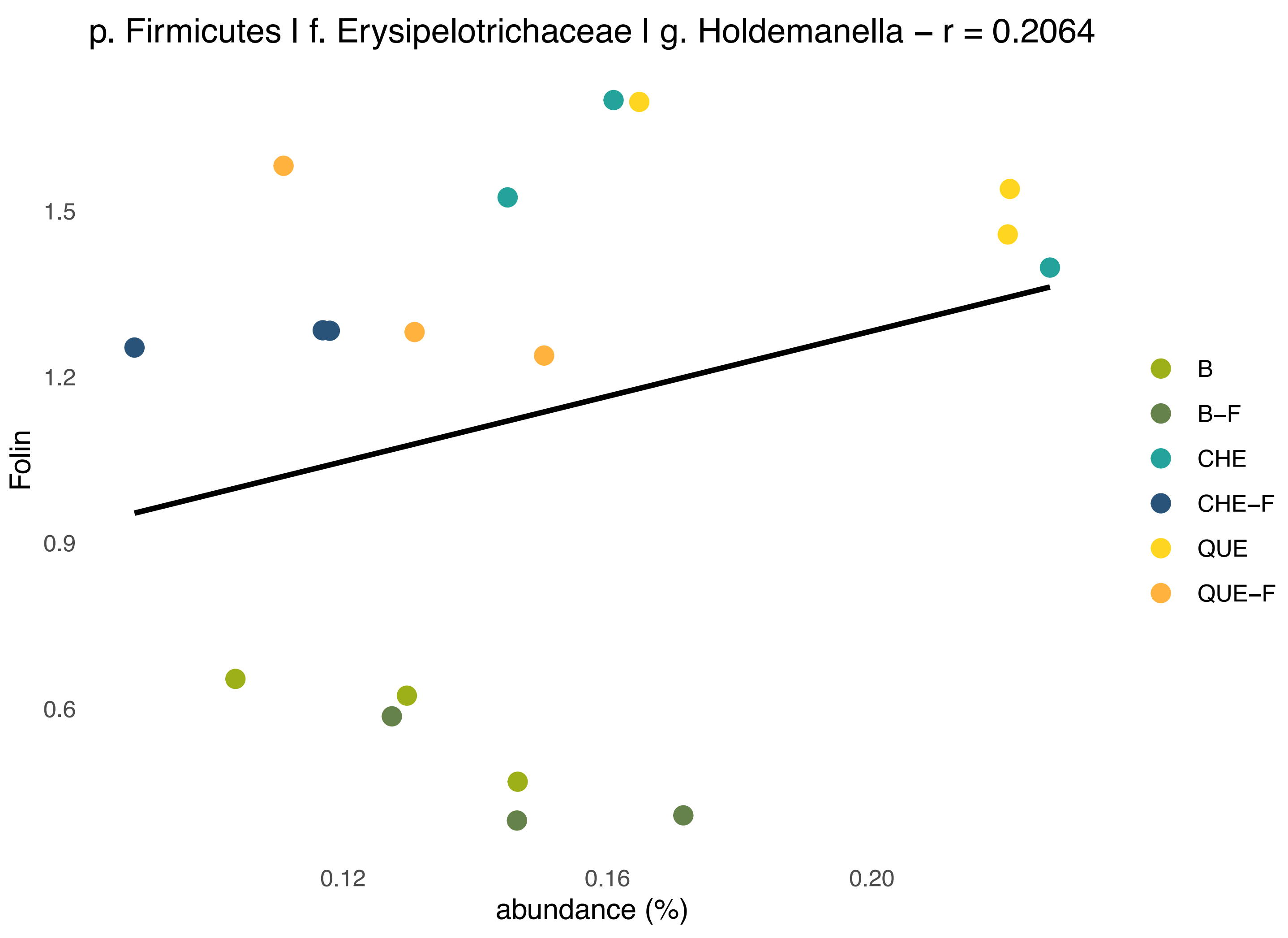

p. Firmicutes I f. Erysipelotrichaceae I g. Holdemanella – r = 0.2064
1.5
1.2
0.9
0.6
Folin
0.12
0.16
0.20
abundance (%)
B
B–F
CHE
CHE–F
QUE
QUE–F

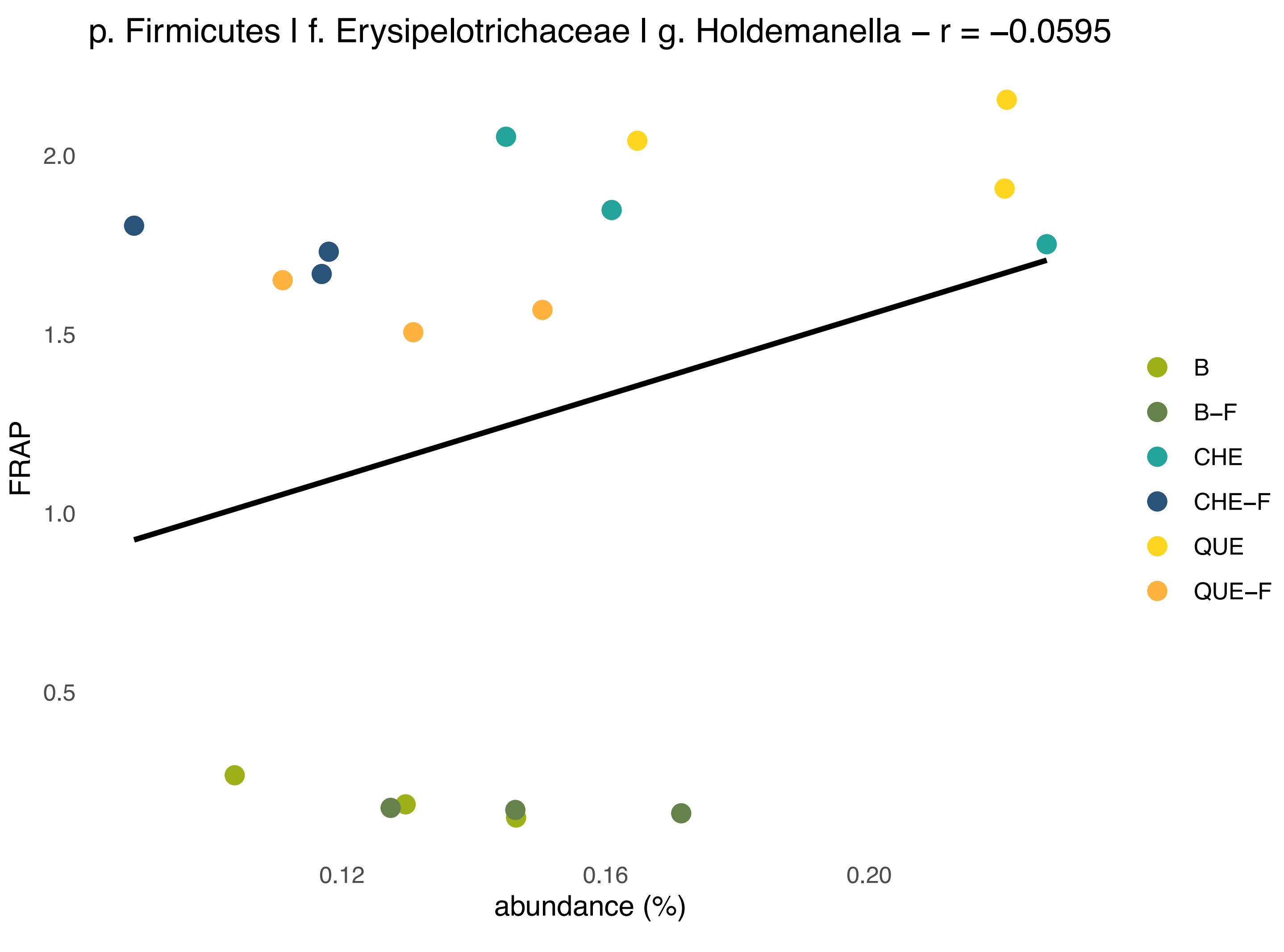
p. Firmicutes I f. Erysipelotrichaceae I g. Holdemanella – r = –0.0595
2.0
1.5
1.0
0.5
FRAP
0.12
0.16
0.20
abundance (%)
B
B–F
CHE
CHE–F
QUE
QUE–F

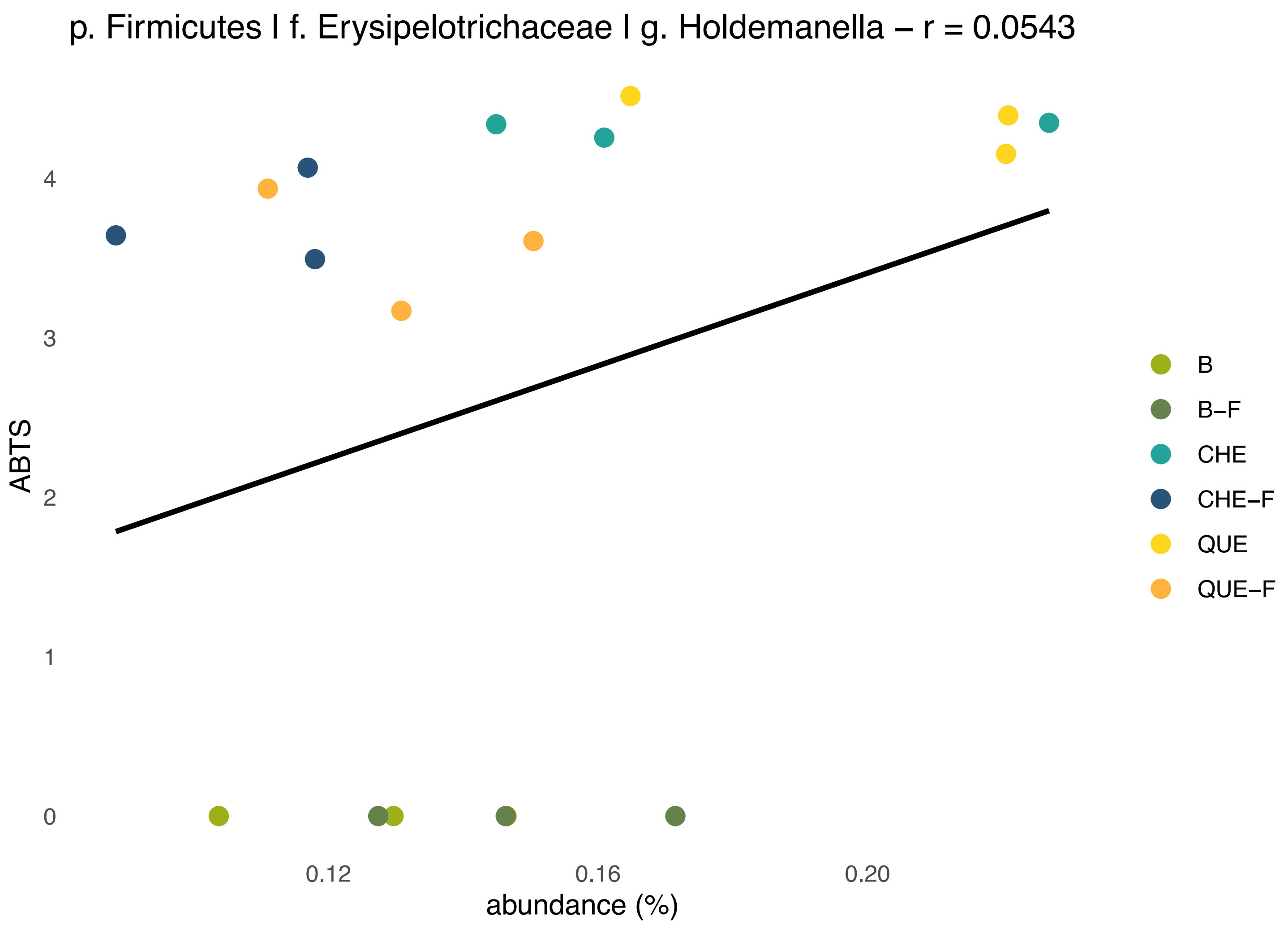
p. Firmicutes I f. Erysipelotrichaceae I g. Holdemanella – r = 0.0543
ABTS
0
1
2
3
4
0.12
0.16
0.20
abundance (%)
B
B–F
CHE
CHE–F
QUE
QUE–F

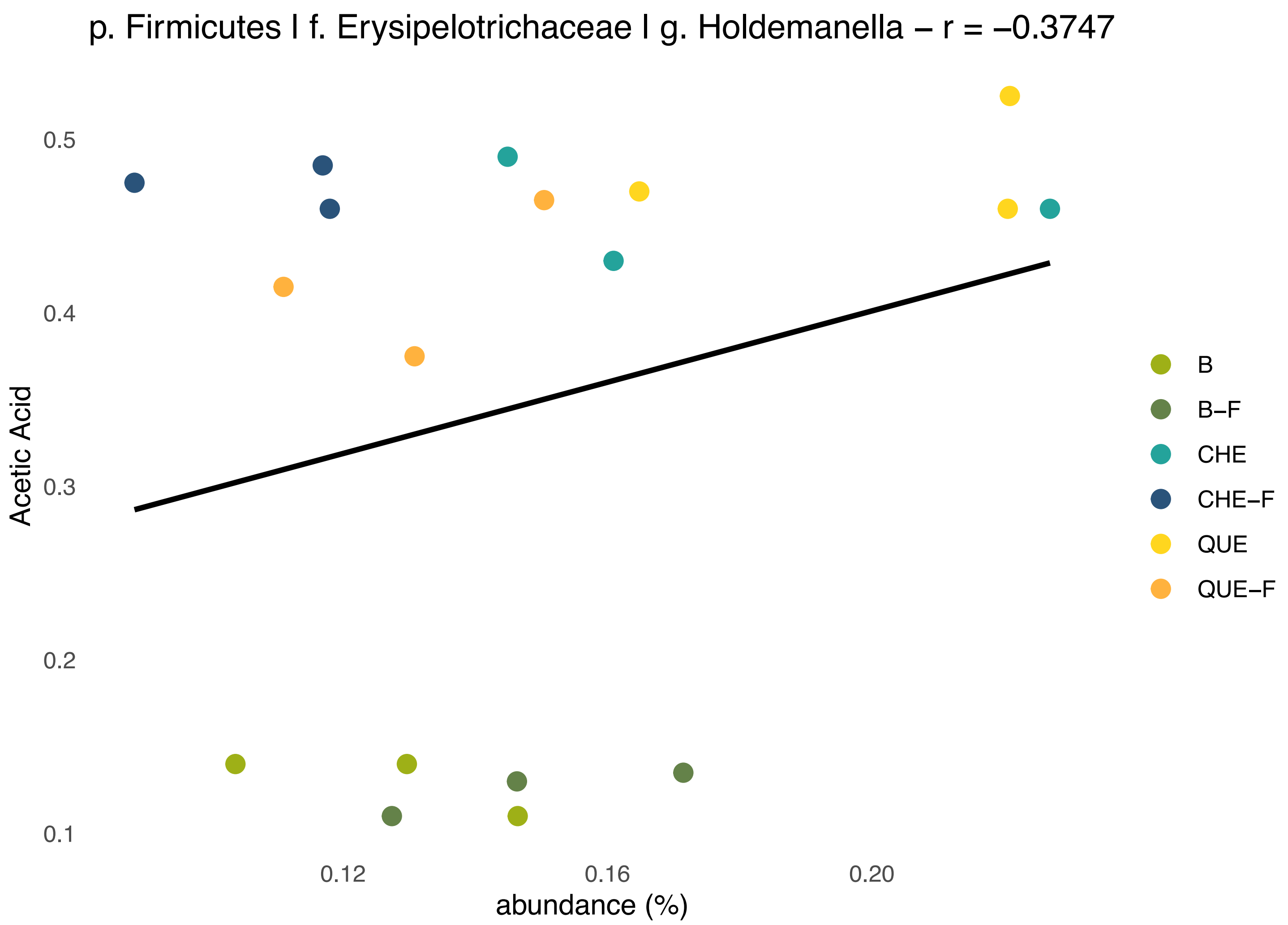

p. Firmicutes I f. Erysipelotrichaceae I g. Holdemanella – r = −0.3747
Acetic Acid
0.5
0.4
0.3
0.2
0.1
0.12
0.16
0.20
abundance (%)
B
B−F
CHE
CHE−F
QUE
QUE−F

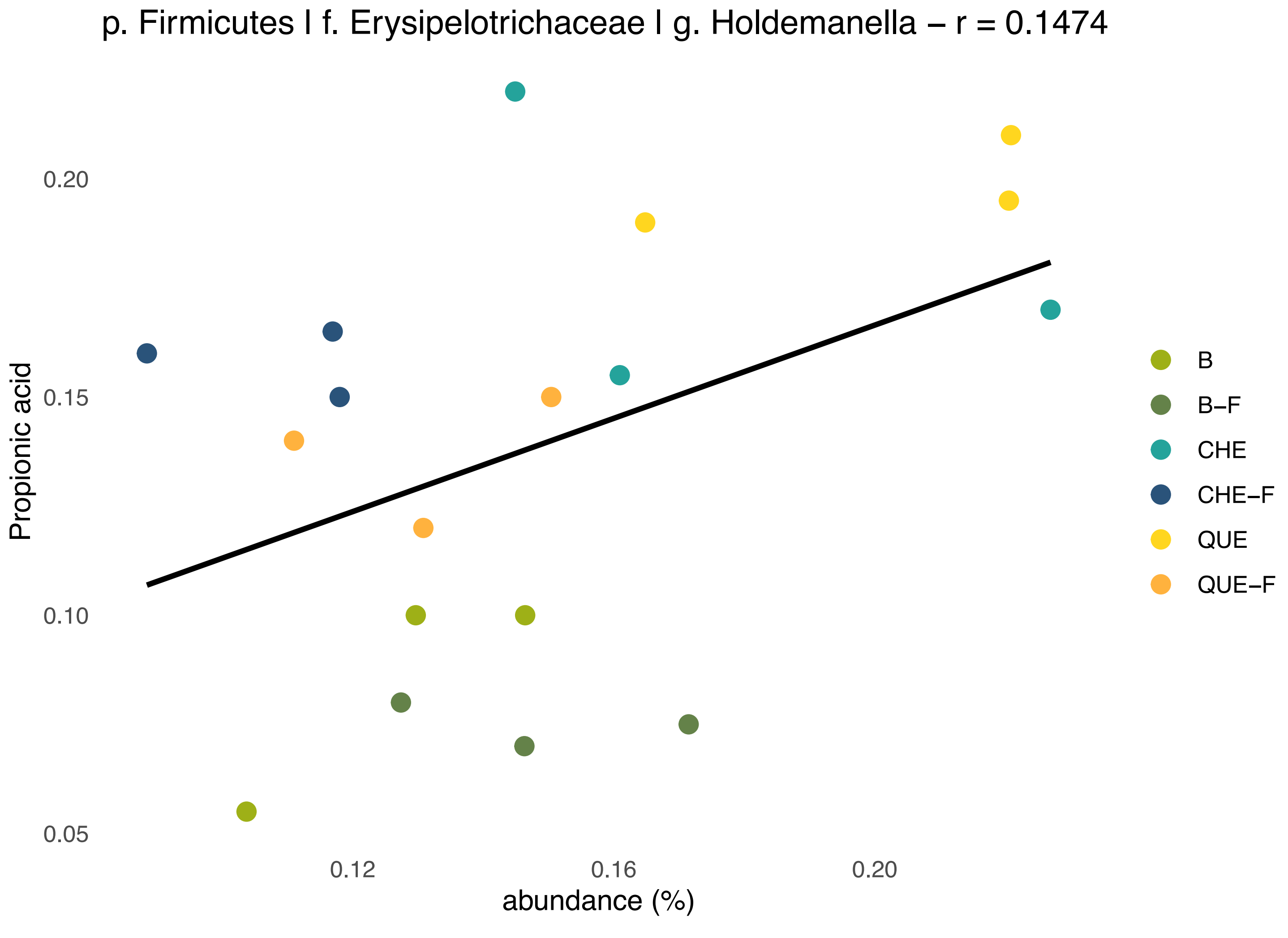

p. Firmicutes I f. Erysipelotrichaceae I g. Holdemanella – r = 0.1474
Propionic acid
0.20
0.15
0.10
0.05
0.12
0.16
0.20
abundance (%)
B
B–F
CHE
CHE–F
QUE
QUE–F

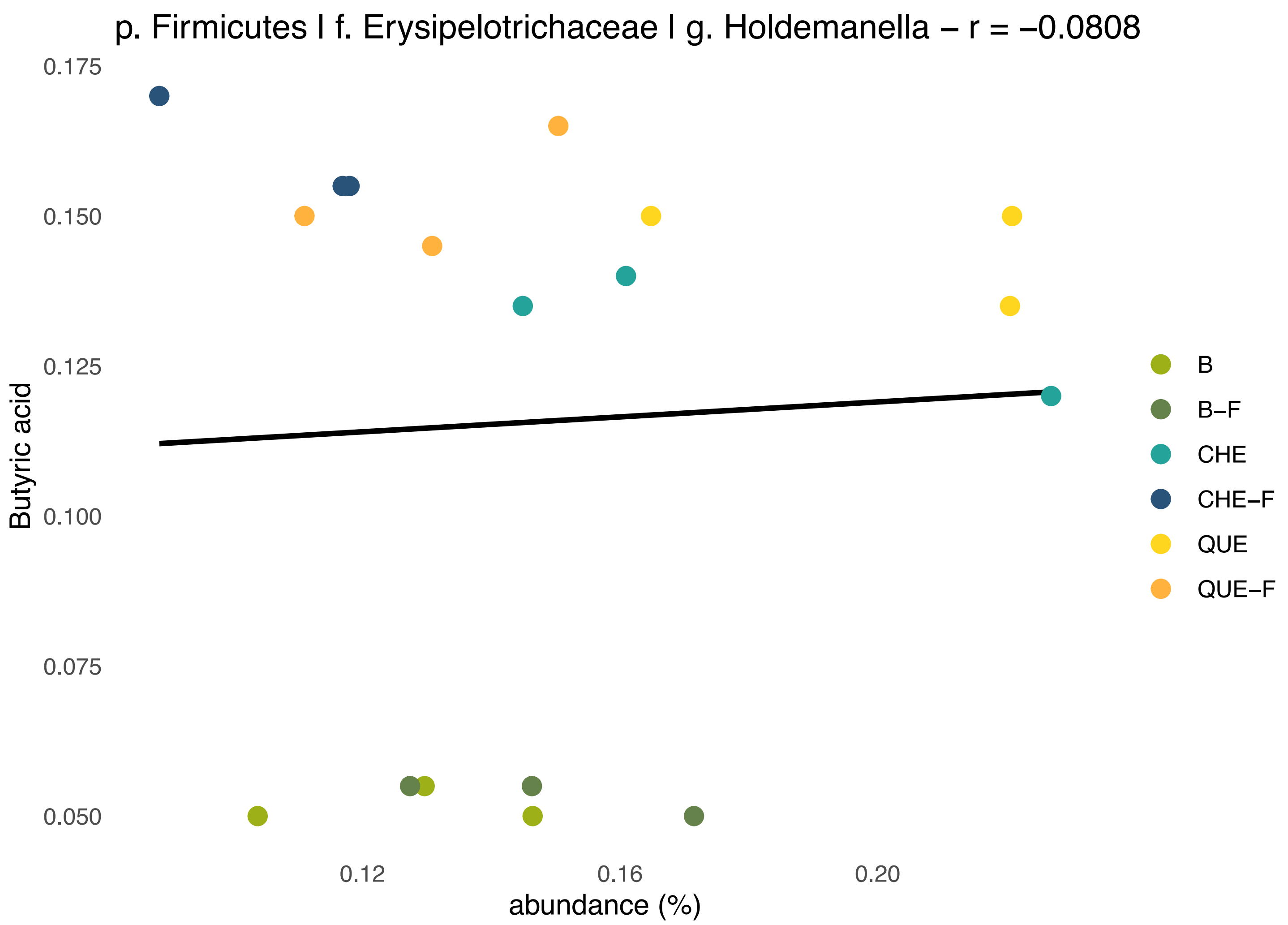

p. Firmicutes I f. Erysipelotrichaceae I g. Holdemanella – r = –0.0808
Butyric acid
0.175
0.150
0.125
0.100
0.075
0.050
0.12
0.16
0.20
abundance (%)
B
B–F
CHE
CHE–F
QUE
QUE–F

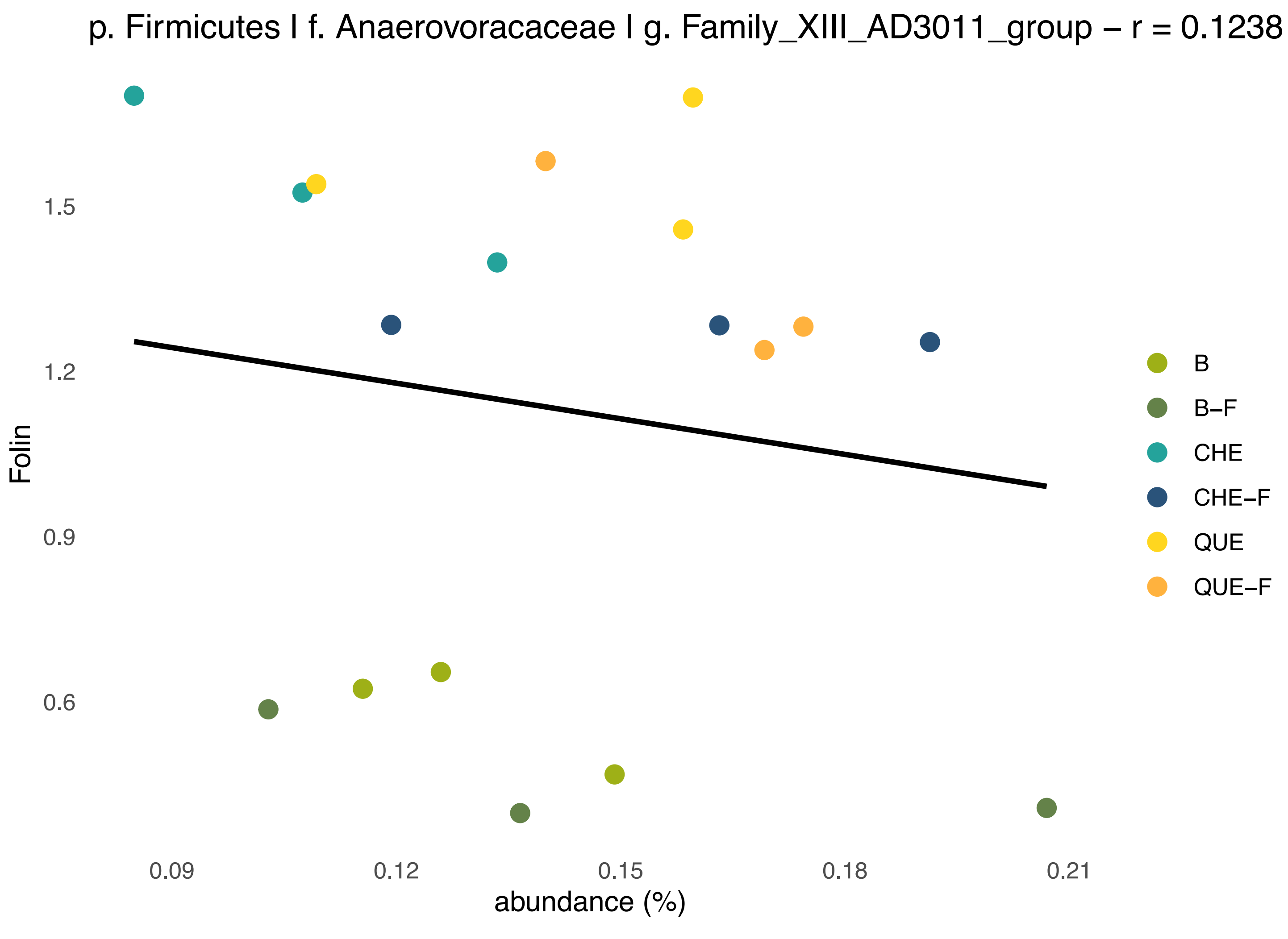

p. Firmicutes I f. Anaerovoracaceae I g. Family_XIII_AD3011_group – r = 0.1238
Folin
1.5
1.2
0.9
0.6
0.09
0.12
0.15
0.18
0.21
abundance (%)
B
B–F
CHE
CHE–F
QUE
QUE–F

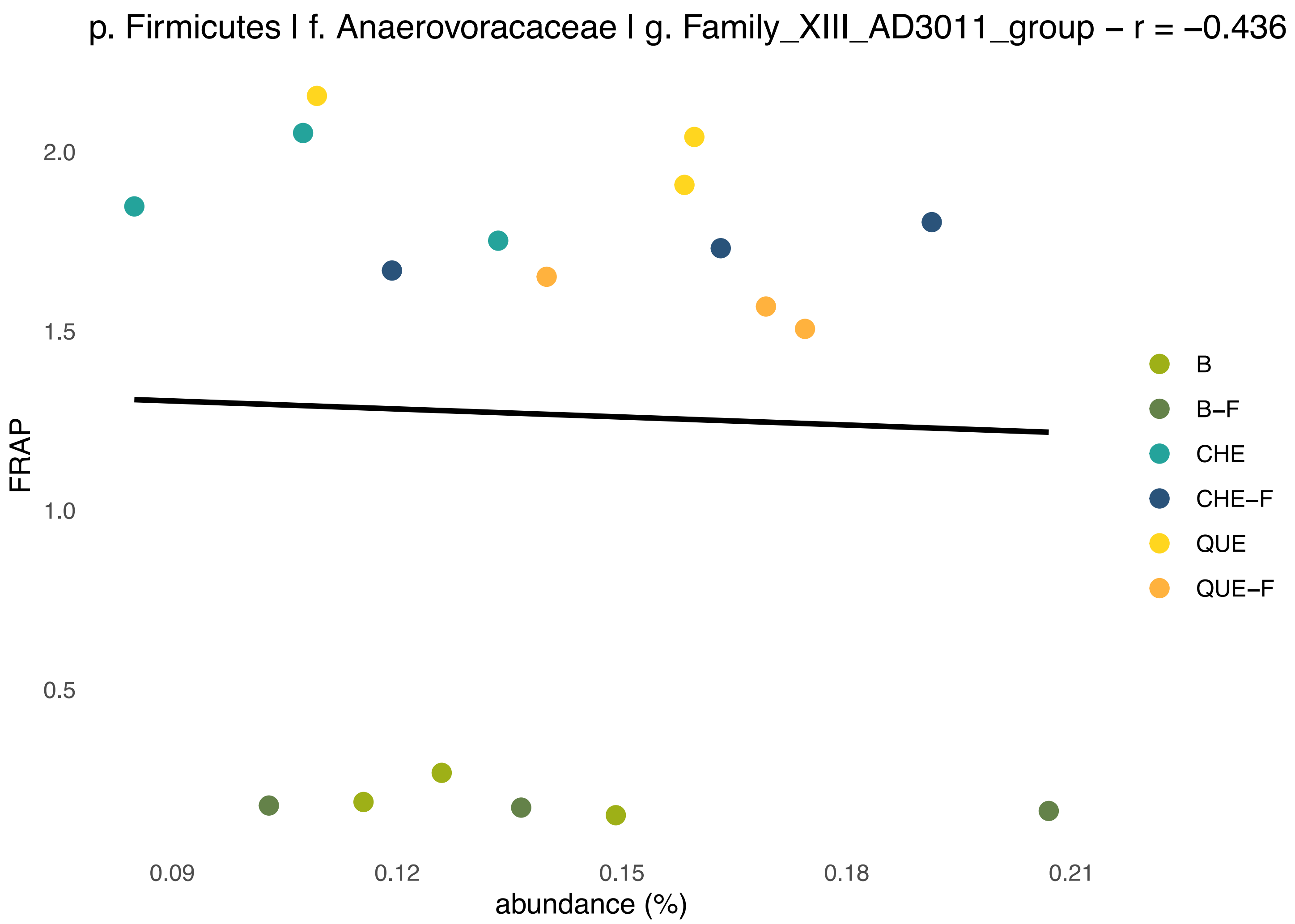

p. Firmicutes I f. Anaerovoracaceae I g. Family_XIII_AD3011_group – r = –0.436
FRAP
2.0
1.5
1.0
0.5
0.09
0.12
0.15
0.18
0.21
abundance (%)
B
B–F
CHE
CHE–F
QUE
QUE–F

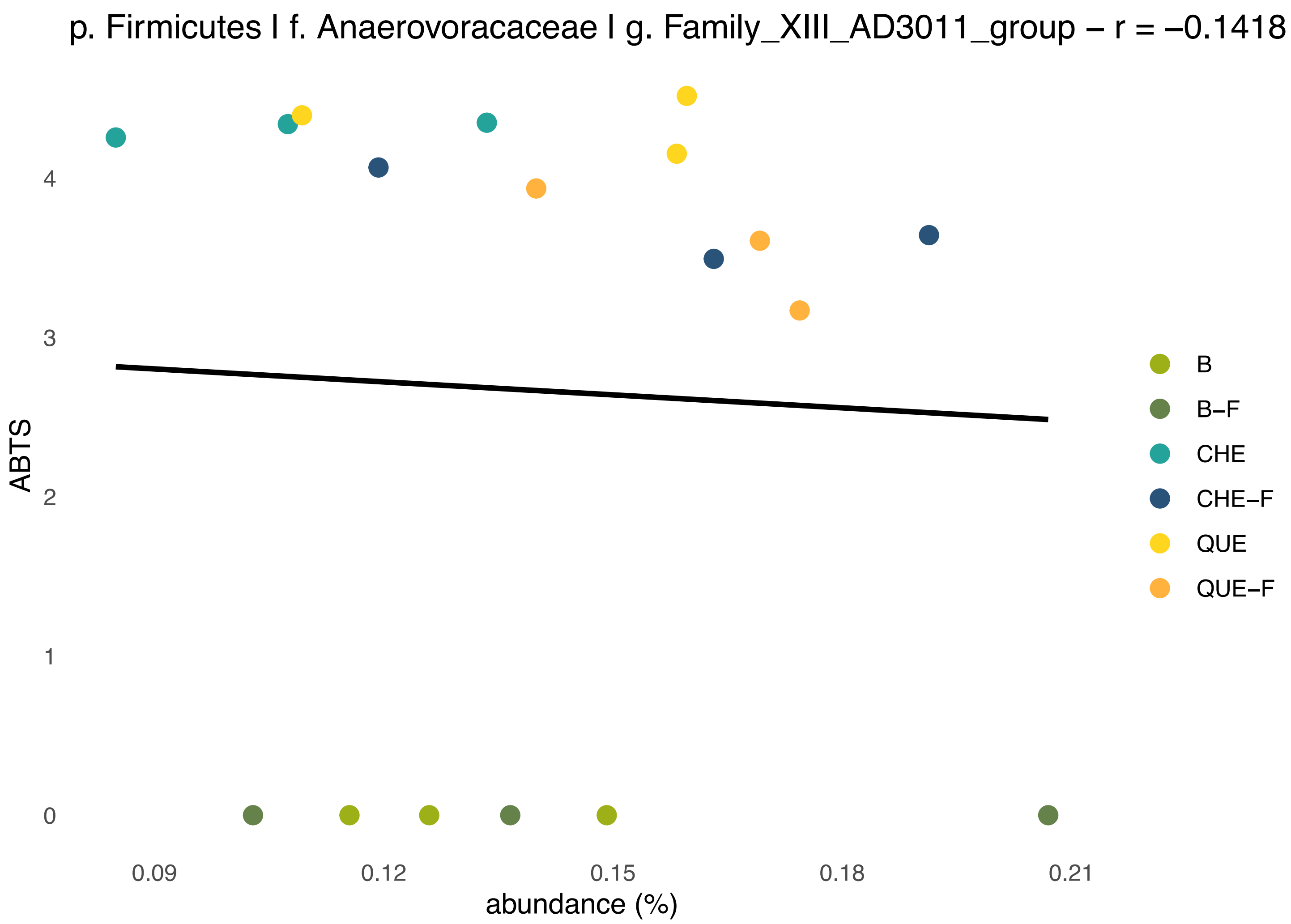
p. Firmicutes I f. Anaerovoracaceae I g. Family_XIII_AD3011_group – r = –0.1418
ABTS
0
1
2
3
4
abundance (%)
0.09
0.12
0.15
0.18
0.21
B
B–F
CHE
CHE–F
QUE
QUE–F

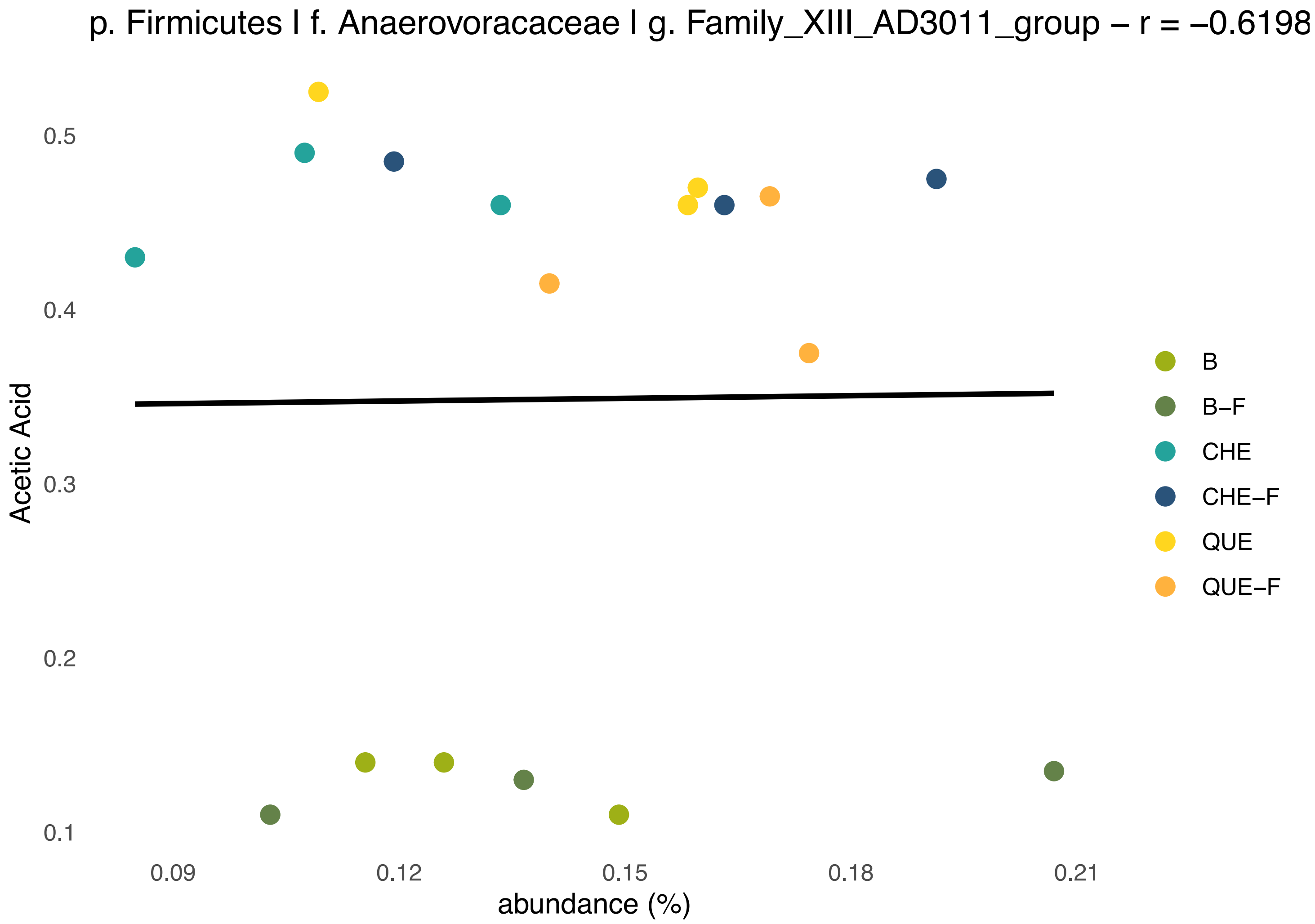
p. Firmicutes I f. Anaerovoracaceae I g. Family_XIII_AD3011_group – r = –0.6198
Acetic Acid
0.5
0.4
0.3
0.2
0.1
0.09
0.12
0.15
0.18
0.21
abundance (%)
B
B–F
CHE
CHE–F
QUE
QUE–F

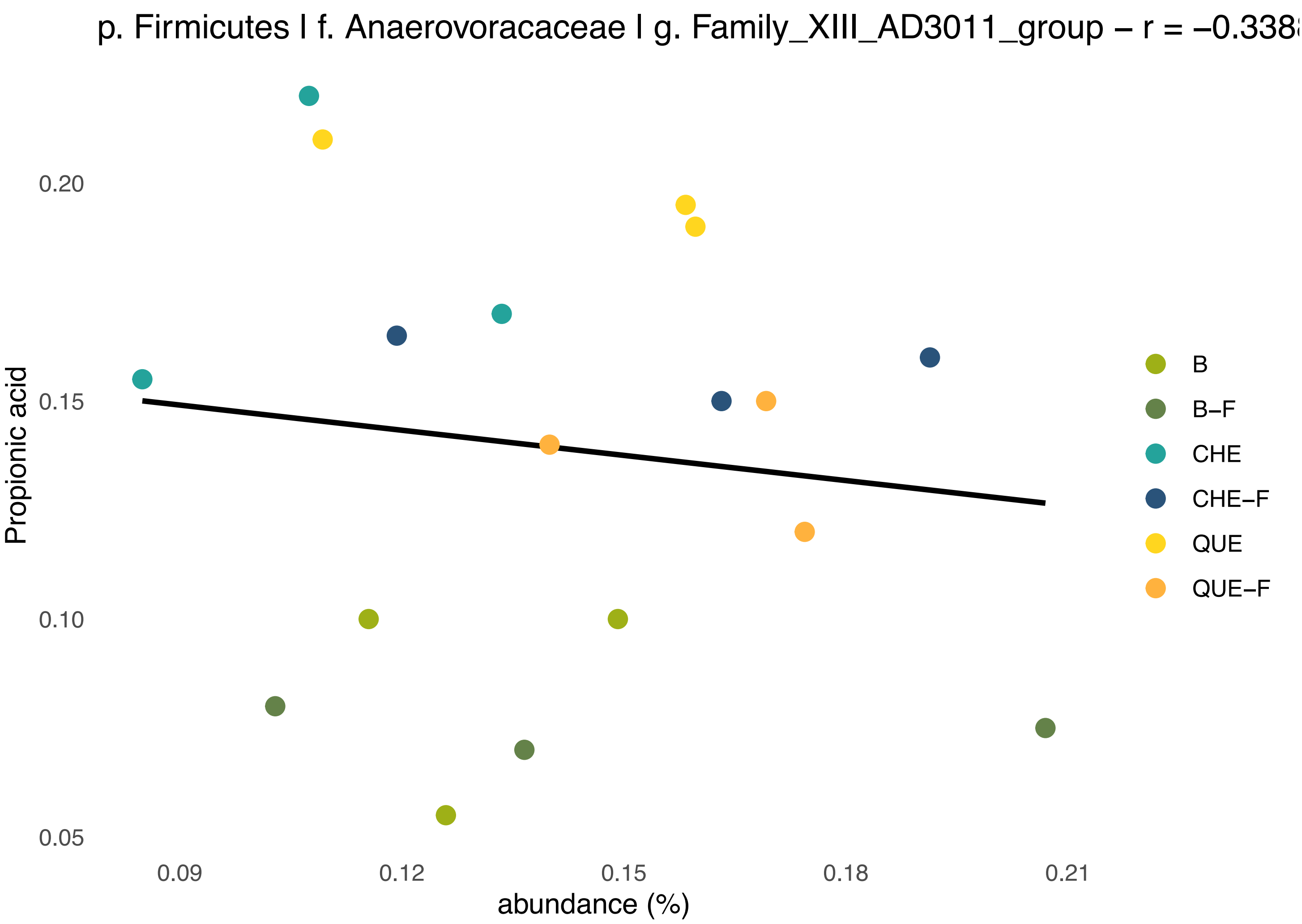
p. Firmicutes I f. Anaerovoracaceae I g. Family_XIII_AD3011_group – r = −0.338
Propionic acid
0.20
0.15
0.10
0.05
0.09
0.12
0.15
0.18
0.21
abundance (%)
B
B−F
CHE
CHE−F
QUE
QUE−F

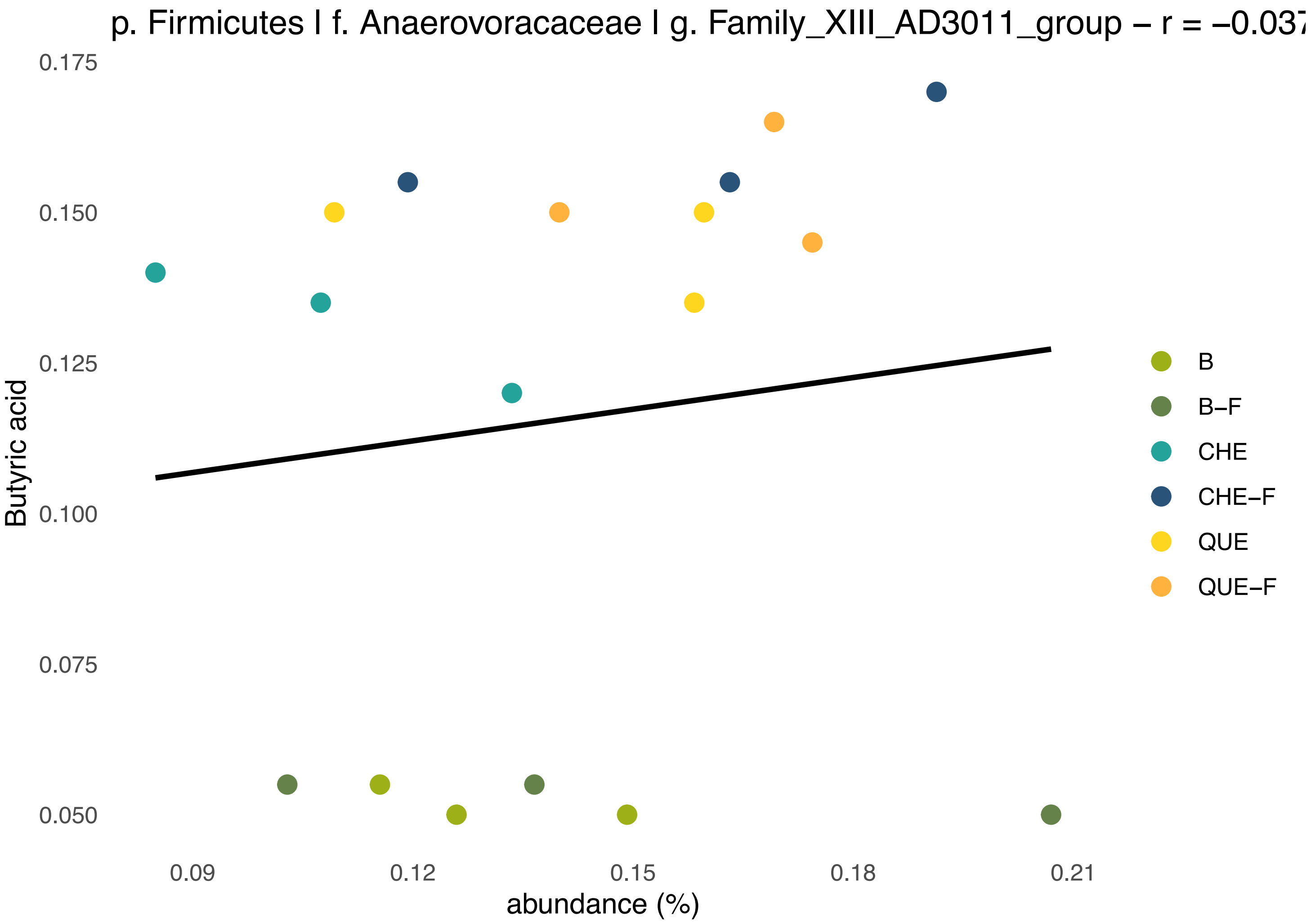

p. Firmicutes I f. Anaerovoracaceae I g. Family_XIII_AD3011_group – r = −0.037
0.175
0.150
0.125
0.100
0.075
0.050
Butyric acid
0.09
0.12
0.15
0.18
0.21
abundance (%)
B
B−F
CHE
CHE−F
QUE
QUE−F

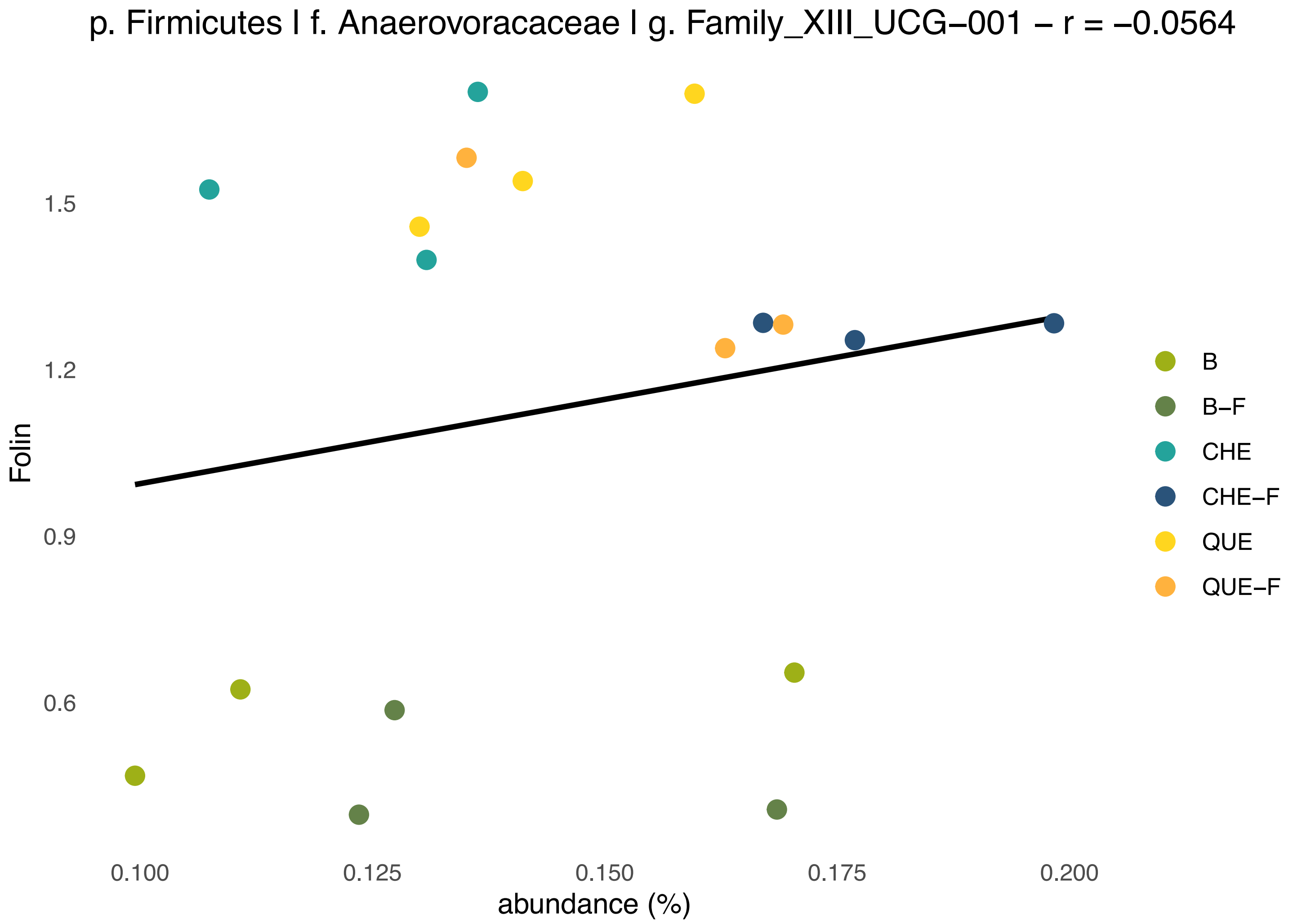

p. Firmicutes I f. Anaerovoracaceae I g. Family_XIII_UCG−001 − r = −0.0564
1.5
1.2
0.9
0.6
Folin
0.100
0.125
0.150
0.175
0.200
abundance (%)
B
B−F
CHE
CHE−F
QUE
QUE−F

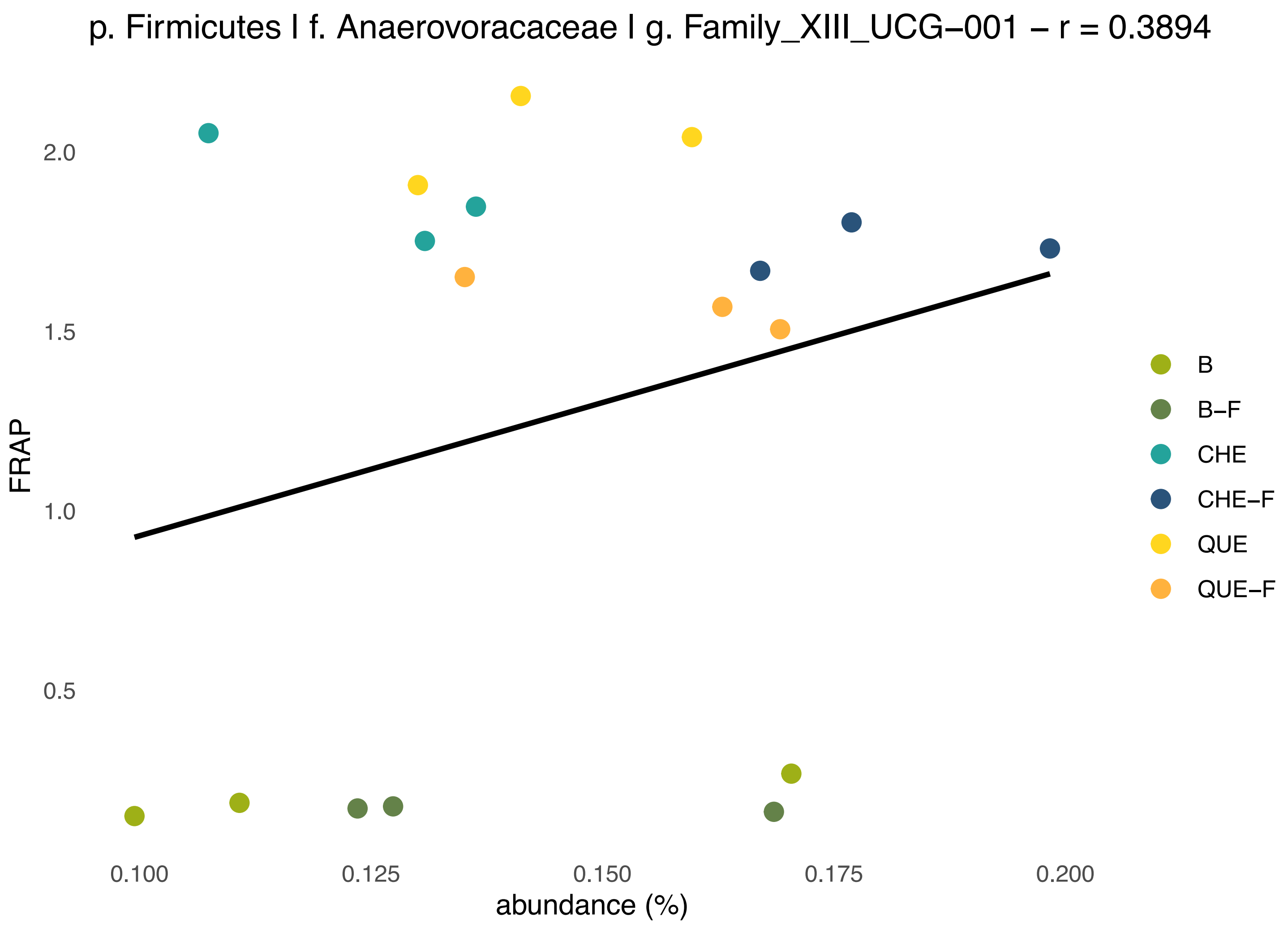

p. Firmicutes I f. Anaerovoracaceae I g. Family_XIII_UCG−001 − r = 0.3894
FRAP
2.0
1.5
1.0
0.5
0.100
0.125
0.150
0.175
0.200
abundance (%)
B
B−F
CHE
CHE−F
QUE
QUE−F

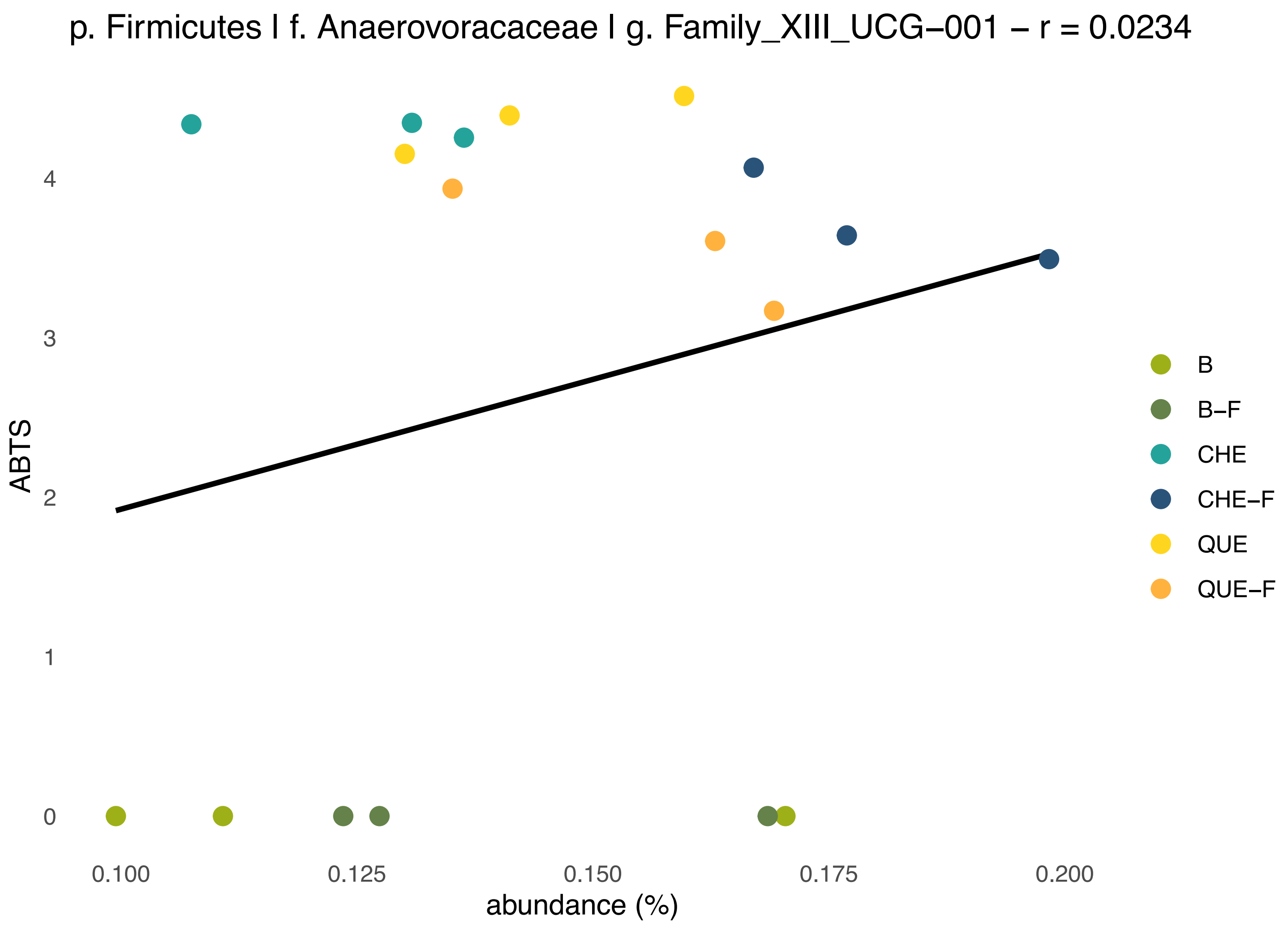

p. Firmicutes I f. Anaerovoracaceae I g. Family_XIII_UCG–001 – r = 0.0234
ABTS
0
1
2
3
4
0.100
0.125
0.150
0.175
0.200
abundance (%)
B
B–F
CHE
CHE–F
QUE
QUE–F

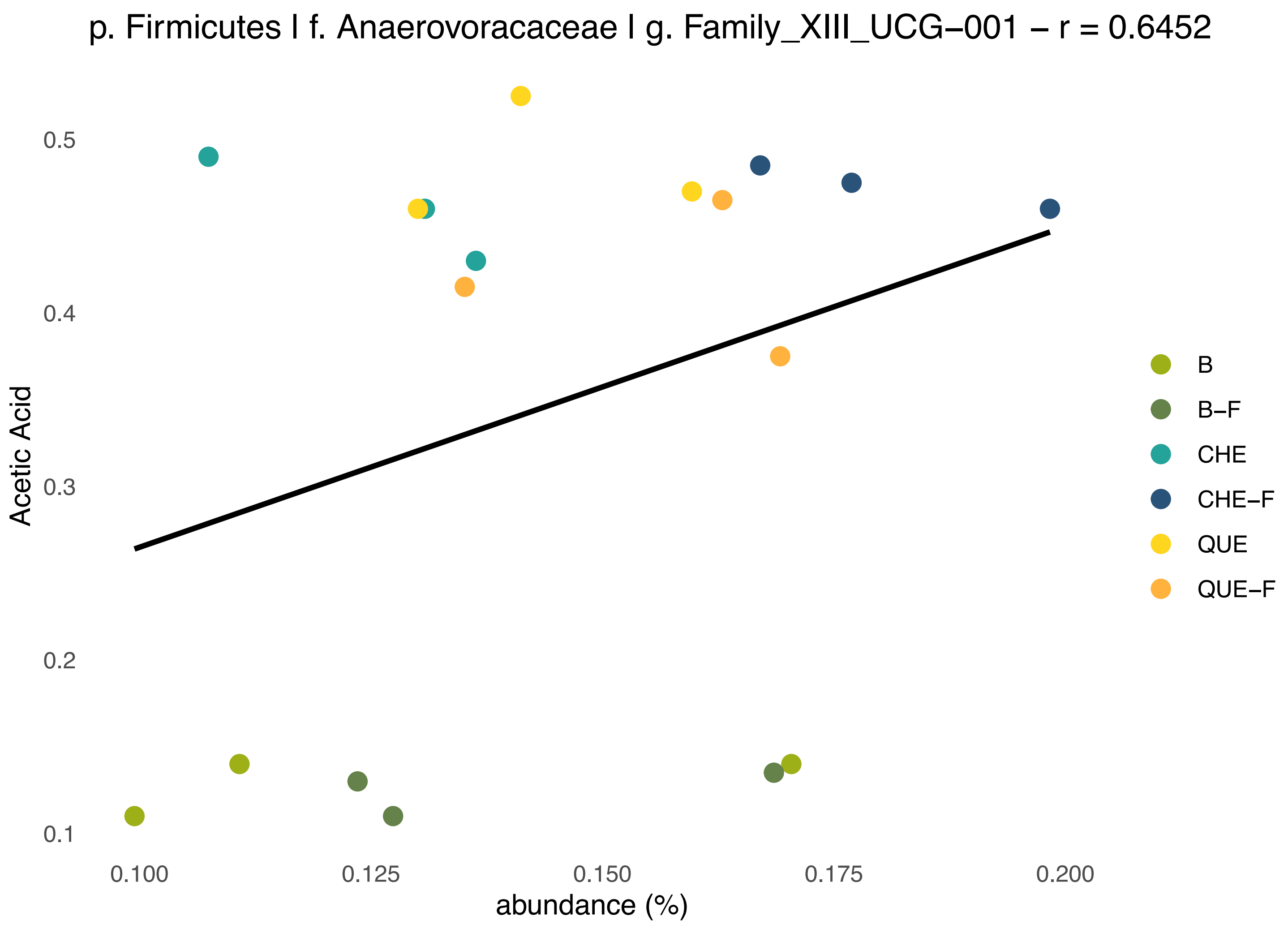
p. Firmicutes | f. Anaerovoracaceae | g. Family_XIII_UCG−001 − r = 0.6452
Acetic Acid
0.5
0.4
0.3
0.2
0.1
0.100
0.125
0.150
0.175
0.200
abundance (%)
B
B−F
CHE
CHE−F
QUE
QUE−F

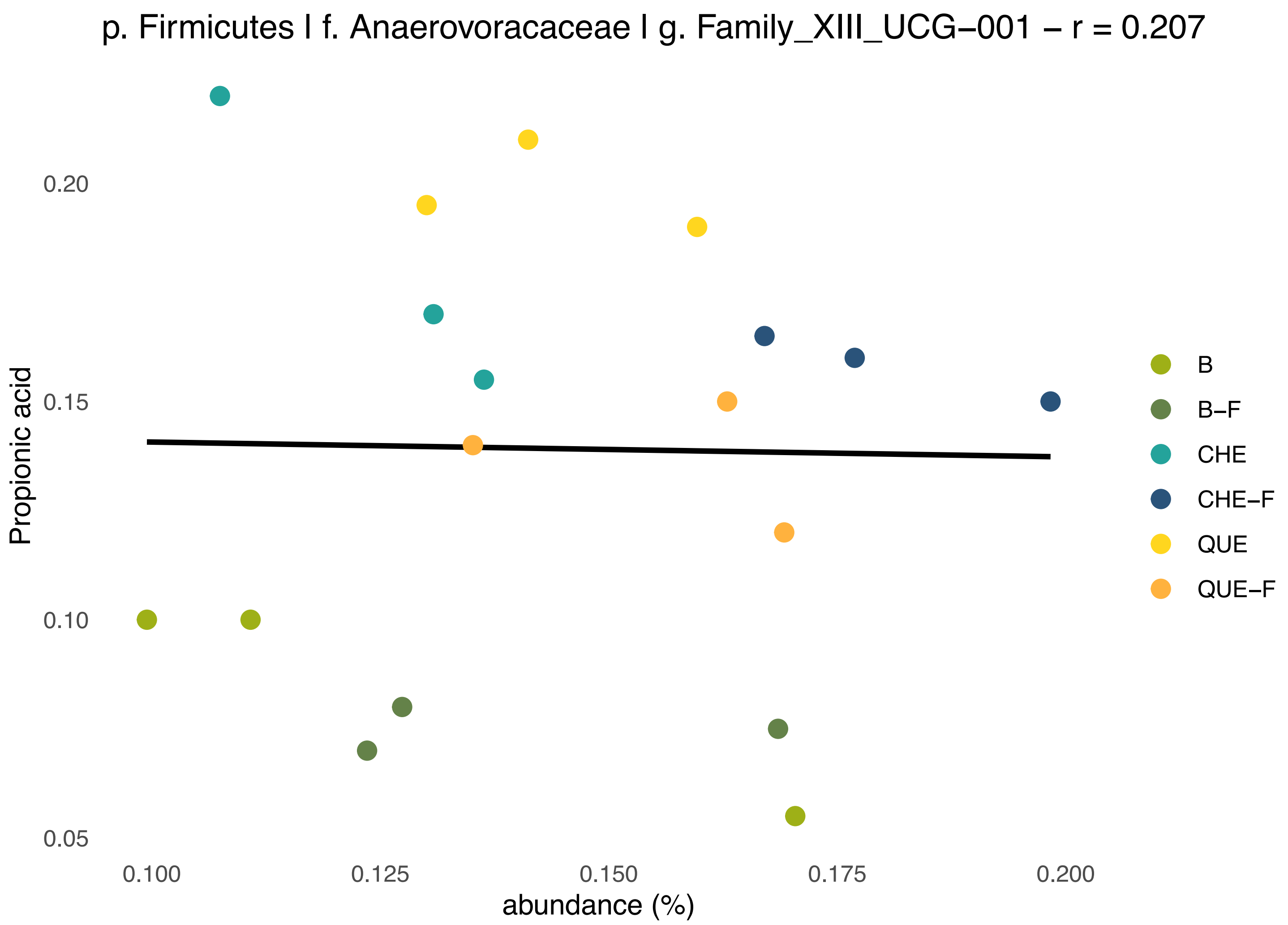

p. Firmicutes I f. Anaerovoracaceae I g. Family_XIII_UCG−001 − r = 0.207
Propionic acid
0.20
0.15
0.10
0.05
0.100
0.125
0.150
0.175
0.200
abundance (%)
B
B−F
CHE
CHE−F
QUE
QUE−F

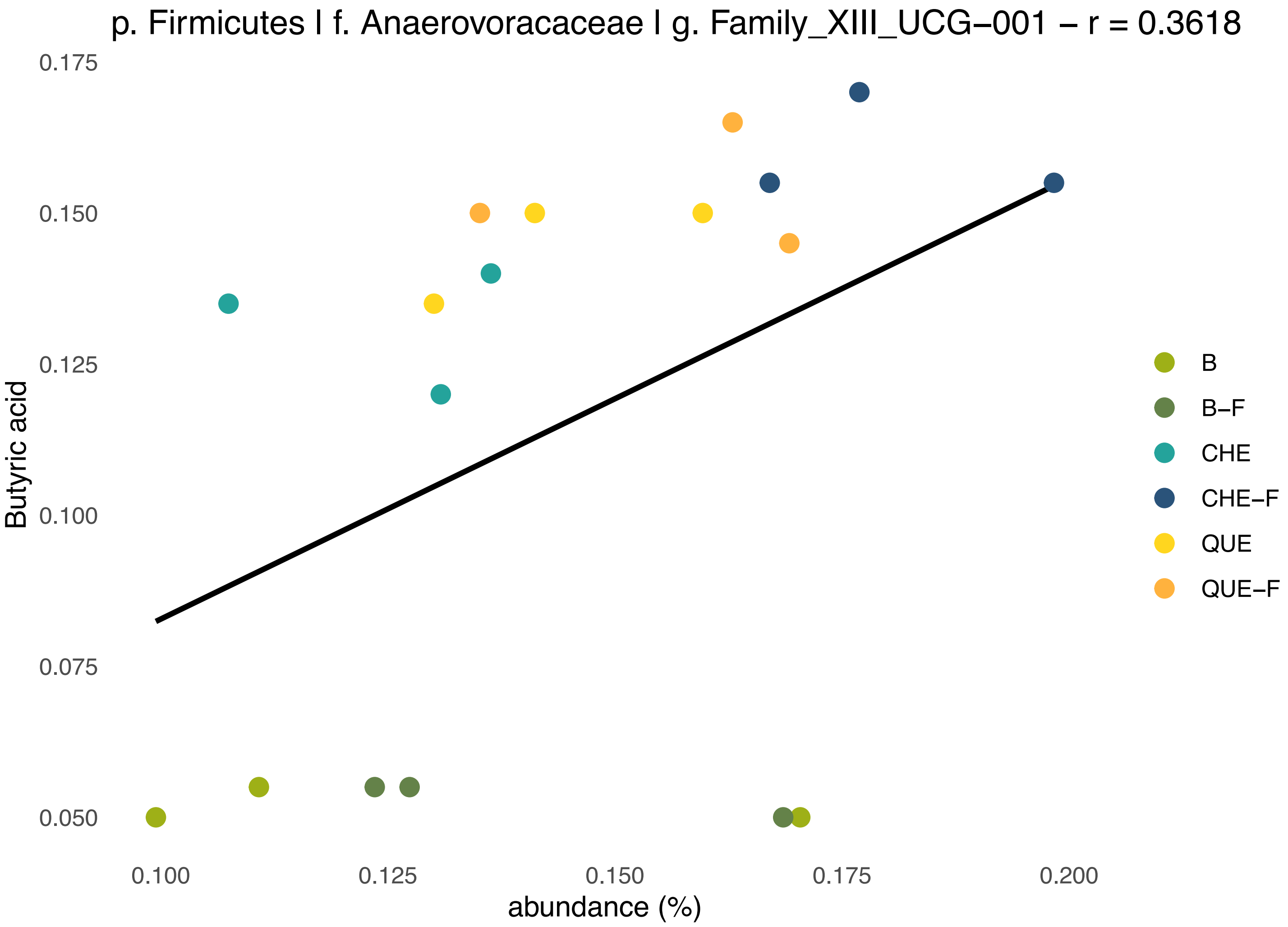
p. Firmicutes l f. Anaerovoracaceae l g. Family_XIII_UCG−001 − r = 0.3618
0.175
0.150
0.125
0.100
0.075
0.050
Butyric acid
0.100
0.125
0.150
0.175
0.200
abundance (%)
B
B−F
CHE
CHE−F
QUE
QUE−F

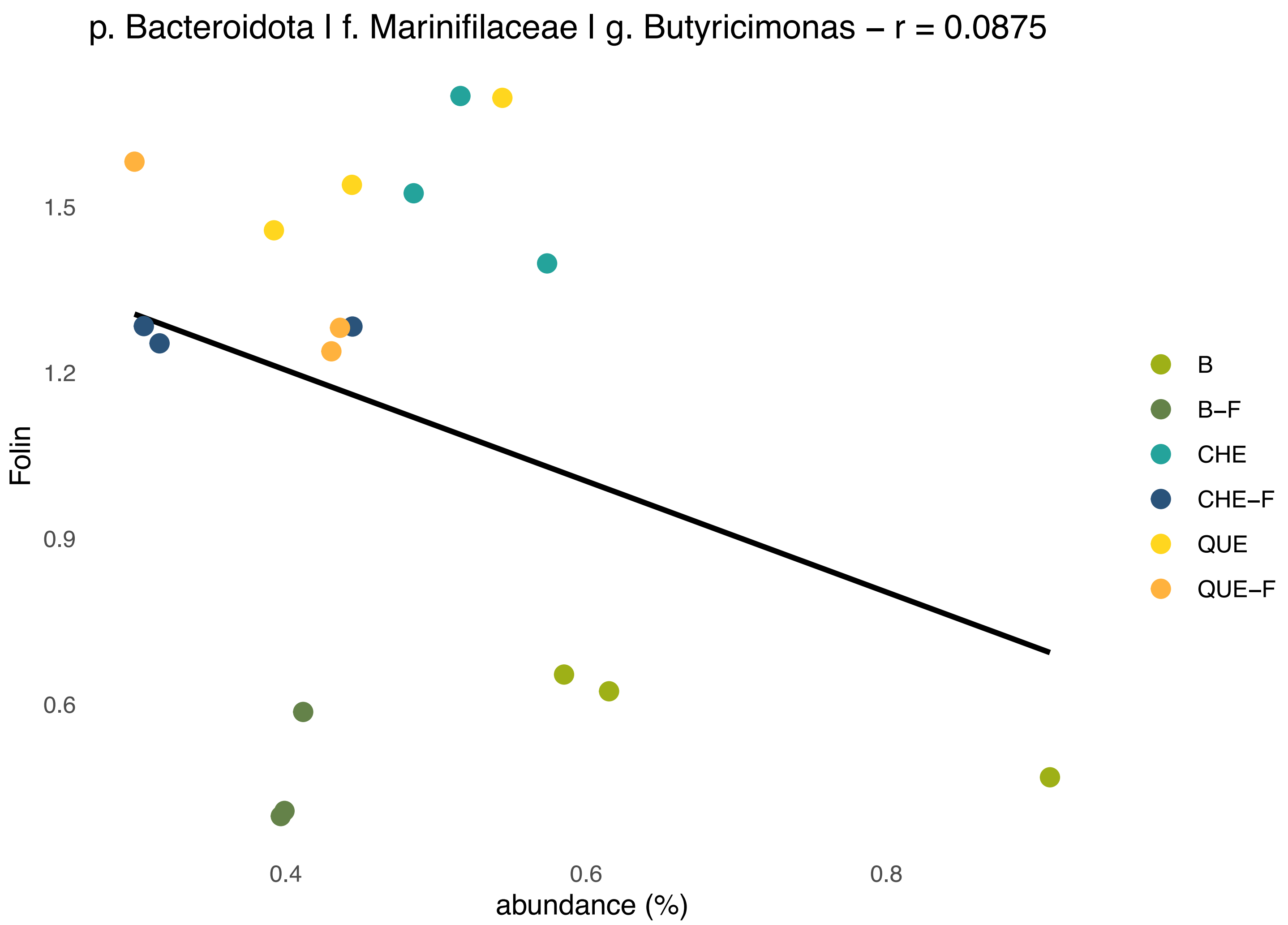
p. Bacteroidota I f. Marinifilaceae I g. Butyricimonas – r = 0.0875
Folin
1.5
1.2
0.9
0.6
0.4
0.6
0.8
abundance (%)
B
B–F
CHE
CHE–F
QUE
QUE–F

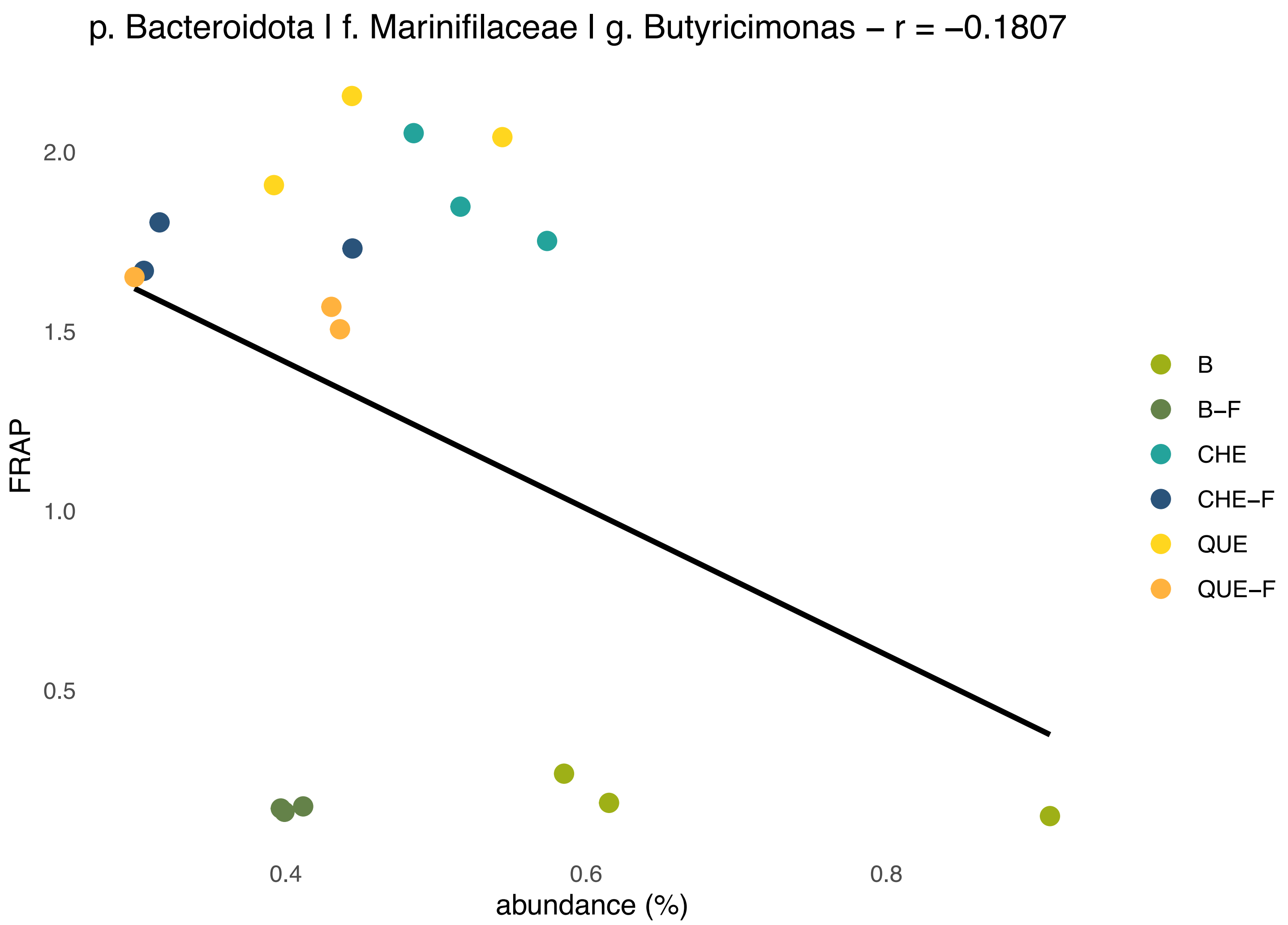
p. Bacteroidota | f. Marinifilaceae | g. Butyricimonas – r = −0.1807
FRAP
2.0
1.5
1.0
0.5
0.4
0.6
0.8
abundance (%)
B
B−F
CHE
CHE−F
QUE
QUE−F

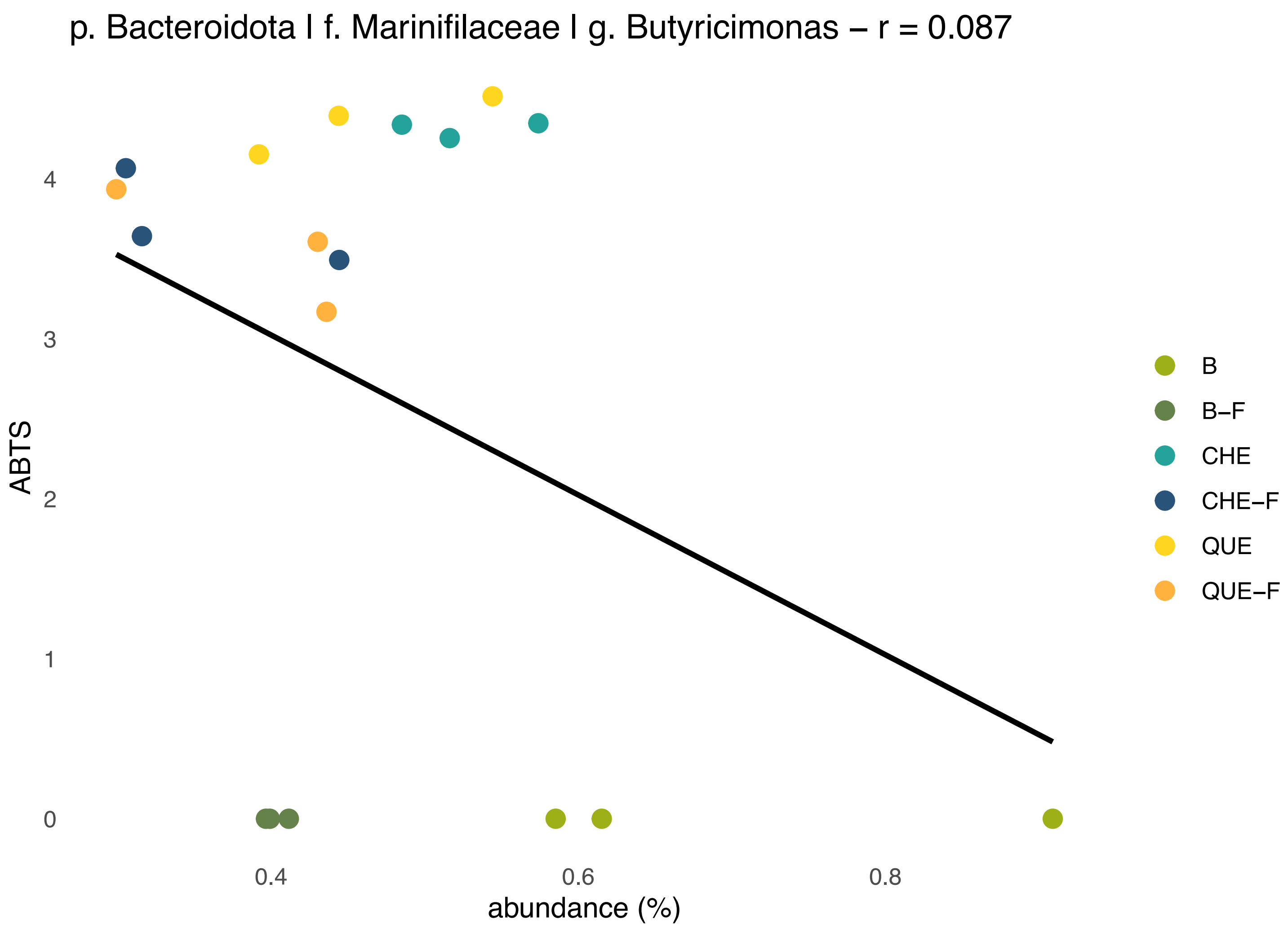

p. Bacteroidota I f. Marinifilaceae I g. Butyricimonas – r = 0.087
ABTS
4
3
2
1
0
0.4
0.6
0.8
abundance (%)
B
B–F
CHE
CHE–F
QUE
QUE–F

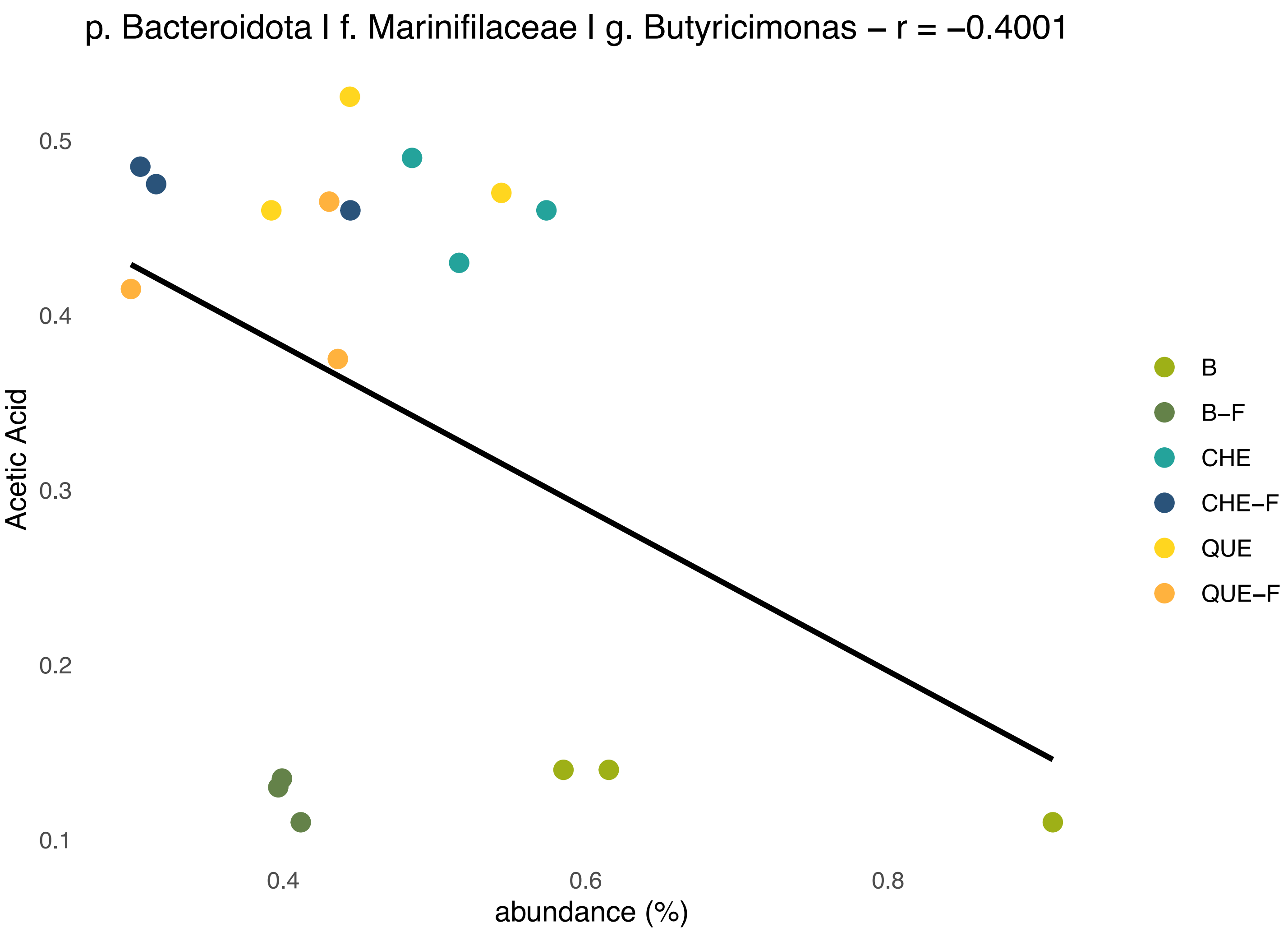
p. Bacteroidota | f. Marinifilaceae | g. Butyricimonas – r = −0.4001
Acetic Acid
0.5
0.4
0.3
0.2
0.1
0.4
0.6
0.8
abundance (%)
B
B−F
CHE
CHE−F
QUE
QUE−F

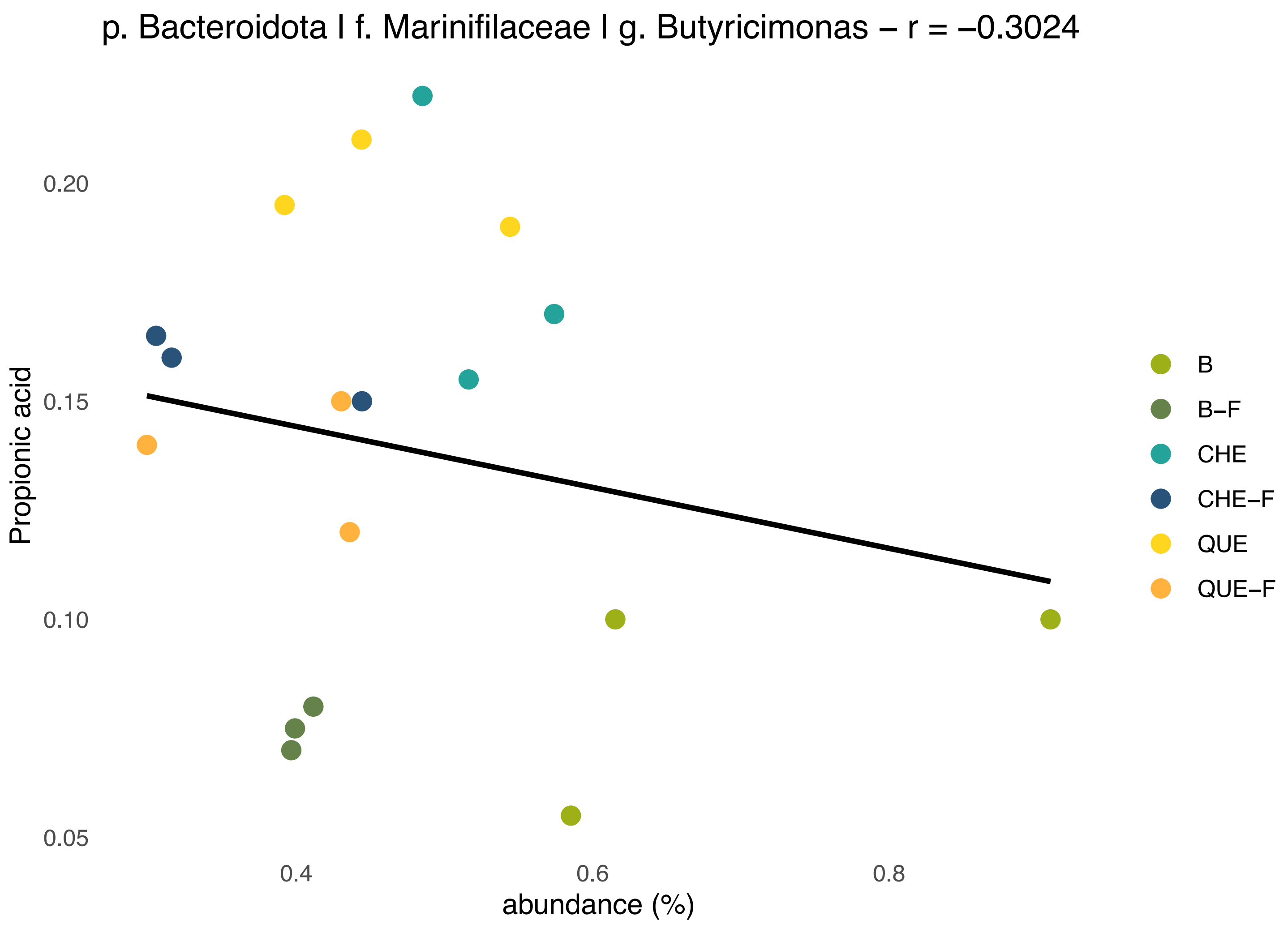
p. Bacteroidota I f. Marinifilaceae I g. Butyricimonas – r = −0.3024
0.20
0.15
0.10
0.05
Propionic acid
0.4
0.6
0.8
abundance (%)
B
B−F
CHE
CHE−F
QUE
QUE−F

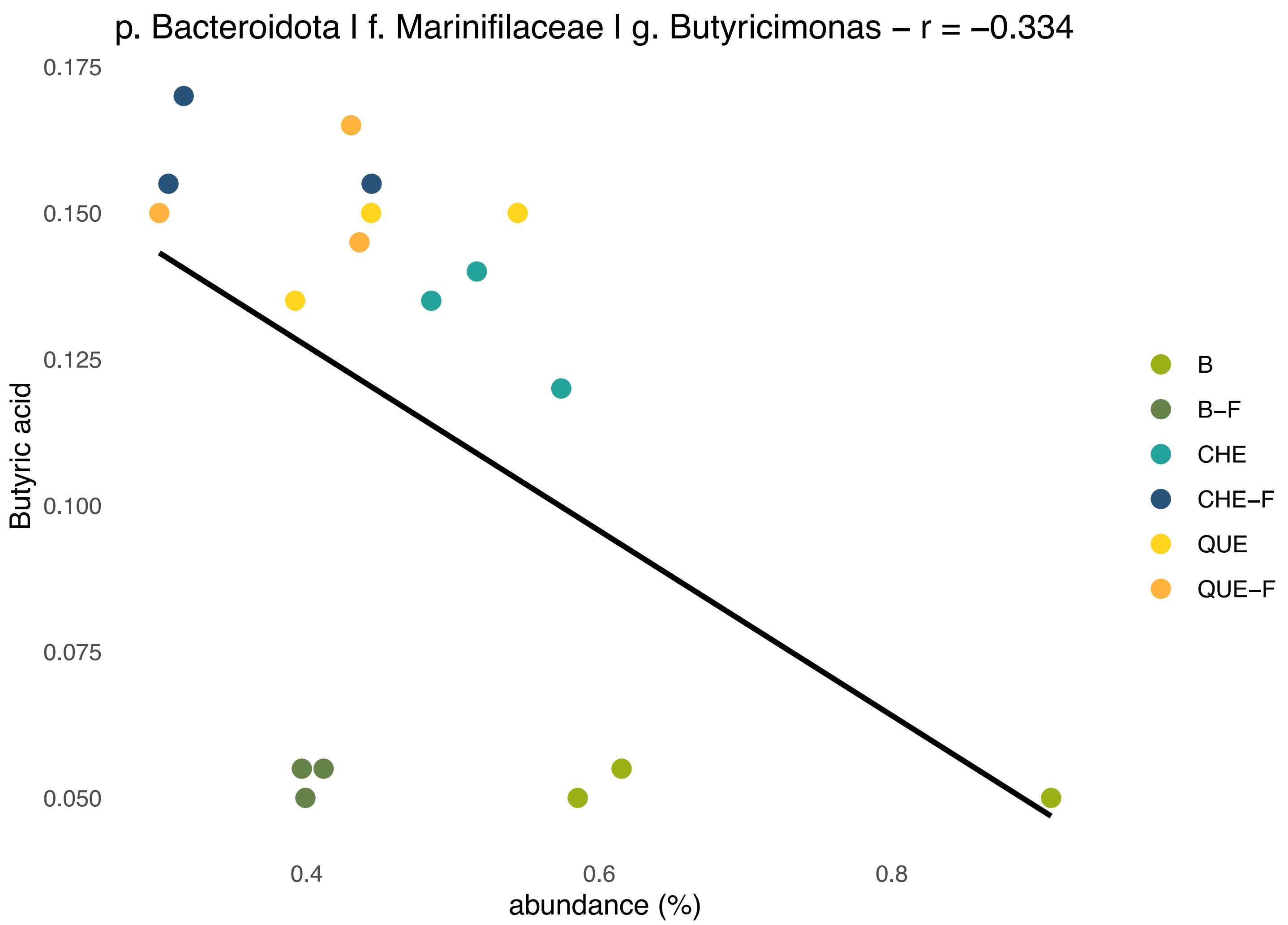
p. Bacteroidota I f. Marinifilaceae I g. Butyricimonas – r = –0.334
0.175
0.150
0.125
0.100
0.075
0.050
Butyric acid
0.4
0.6
0.8
abundance (%)
B
B–F
CHE
CHE–F
QUE
QUE–F

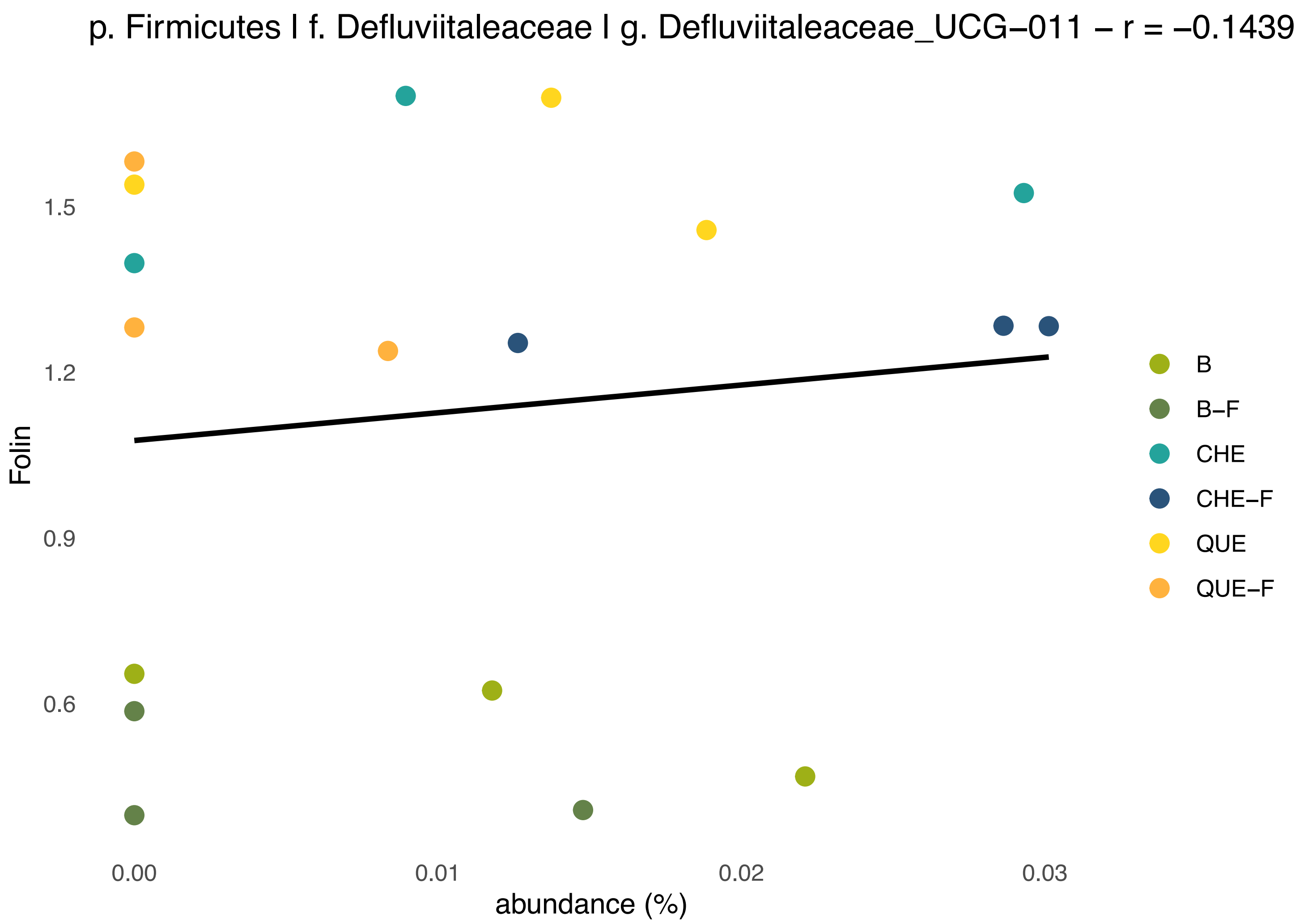

p. Firmicutes I f. Defluviitaleaceae I g. Defluviitaleaceae_UCG−011 − r = −0.1439
Folin
1.5
1.2
0.9
0.6
0.00
0.01
0.02
0.03
abundance (%)
B
B−F
CHE
CHE−F
QUE
QUE−F

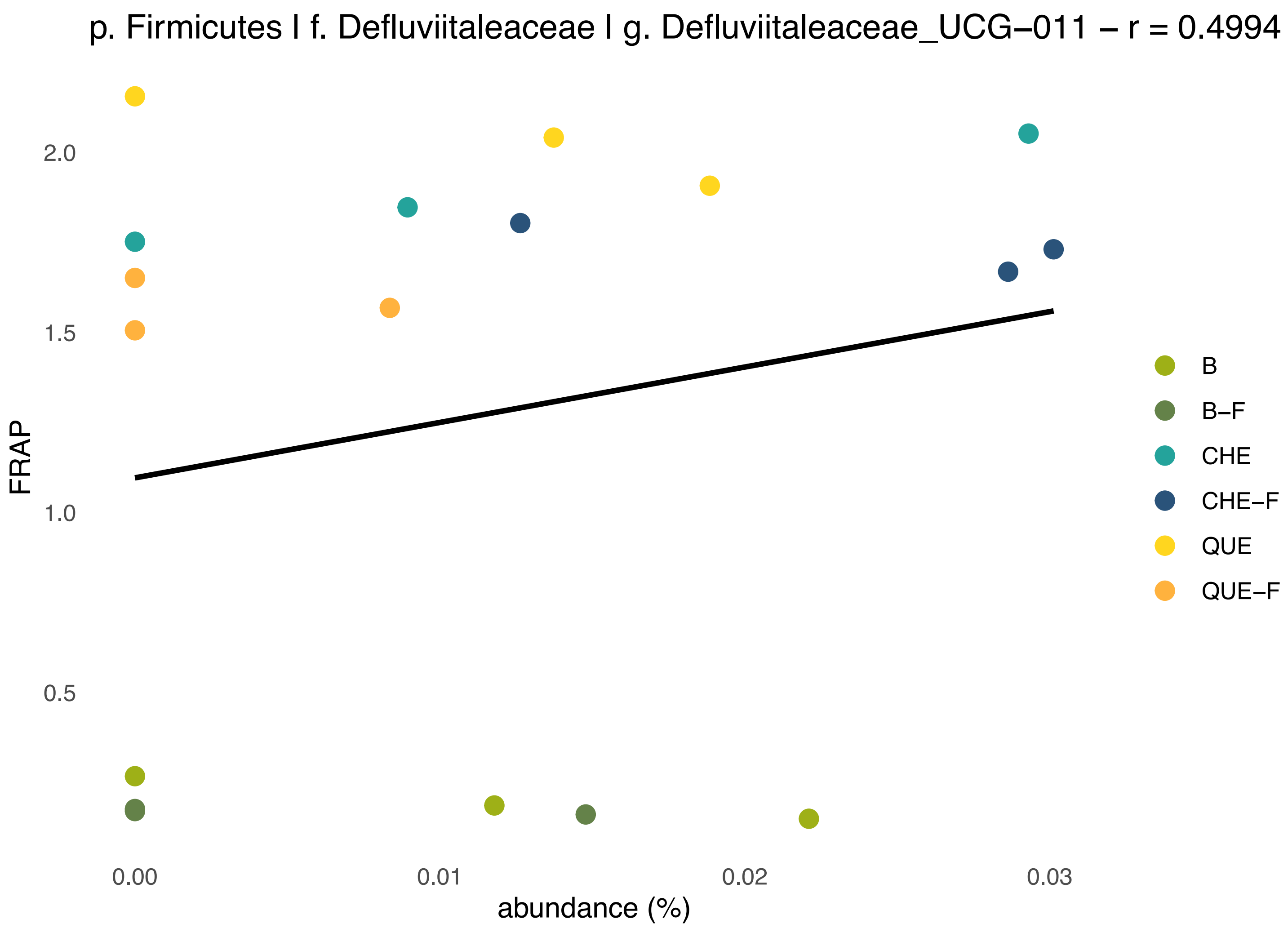
p. Firmicutes I f. Defluviitaleaceae I g. Defluviitaleaceae_UCG−011 − r = 0.4994
FRAP
2.0
1.5
1.0
0.5
0.00
0.01
0.02
0.03
abundance (%)
B
B−F
CHE
CHE−F
QUE
QUE−F

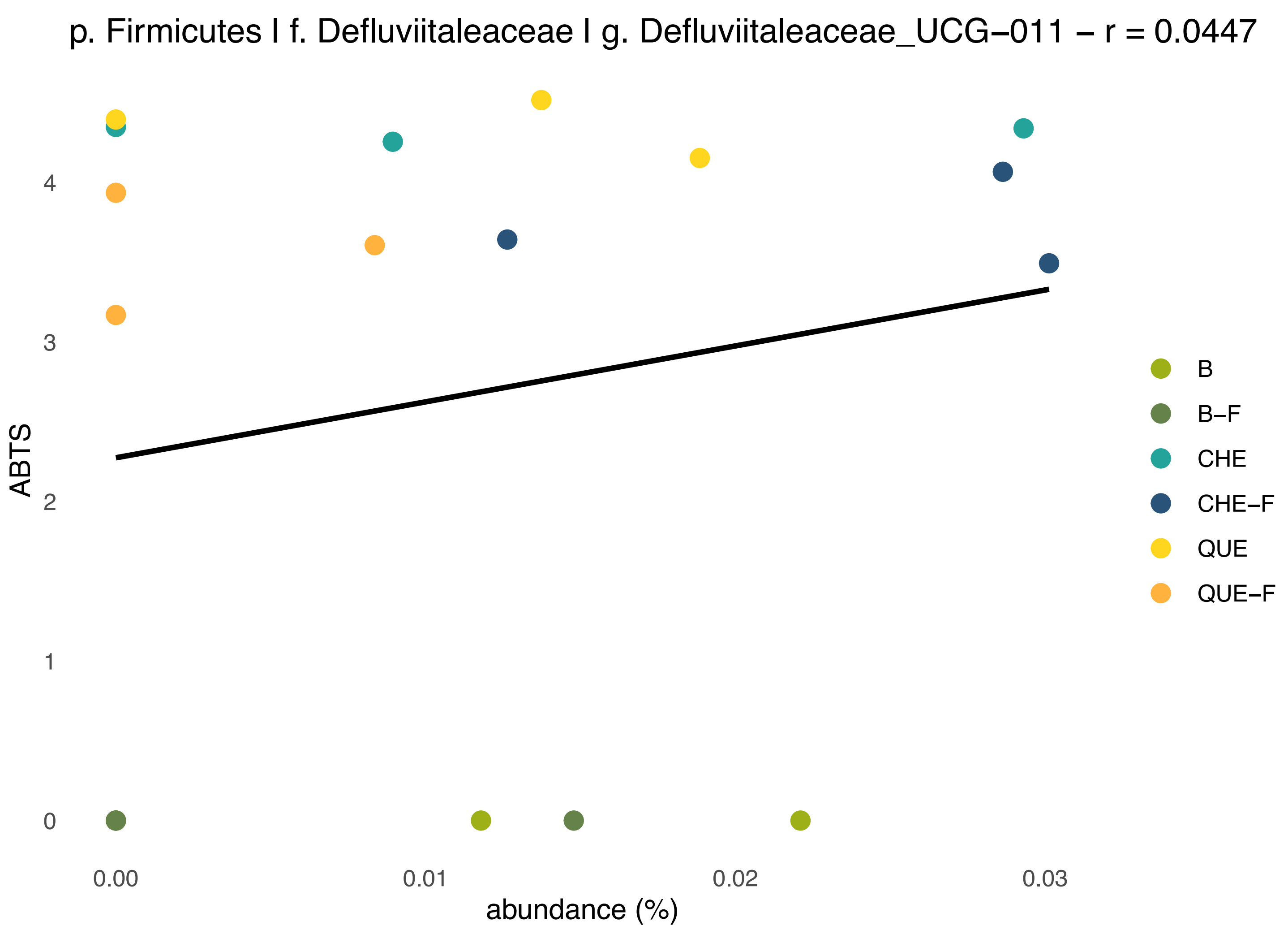
p. Firmicutes I f. Defluviitaleaceae I g. Defluviitaleaceae_UCG−011 − r = 0.0447
ABTS
0
1
2
3
4
0.00
0.01
0.02
0.03
abundance (%)
B
B−F
CHE
CHE−F
QUE
QUE−F

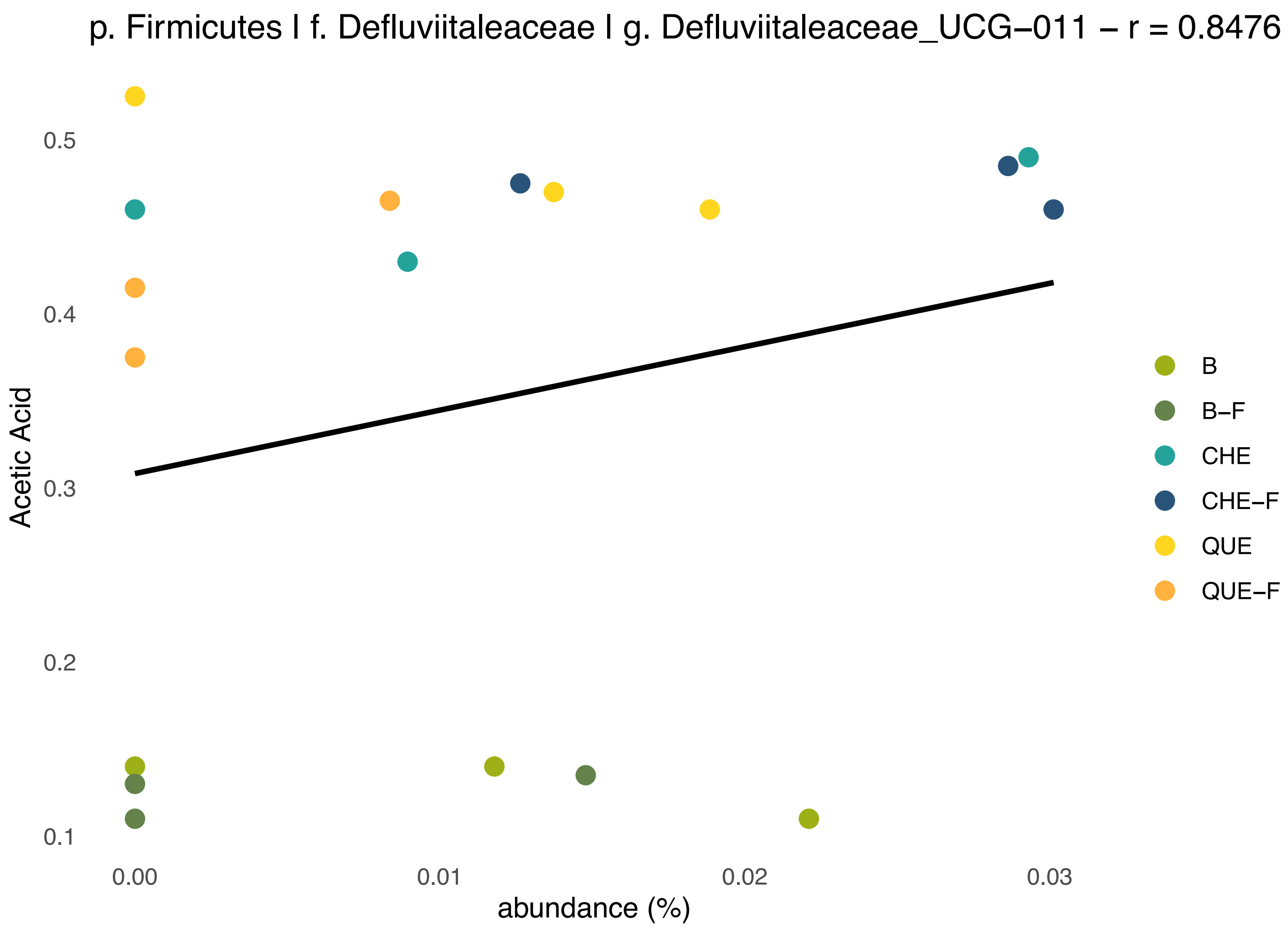

p. Firmicutes I f. Defluviitaleaceae I g. Defluviitaleaceae_UCG−011 − r = 0.8476
Acetic Acid
0.5
0.4
0.3
0.2
0.1
0.00
0.01
0.02
0.03
abundance (%)
B
B−F
CHE
CHE−F
QUE
QUE−F

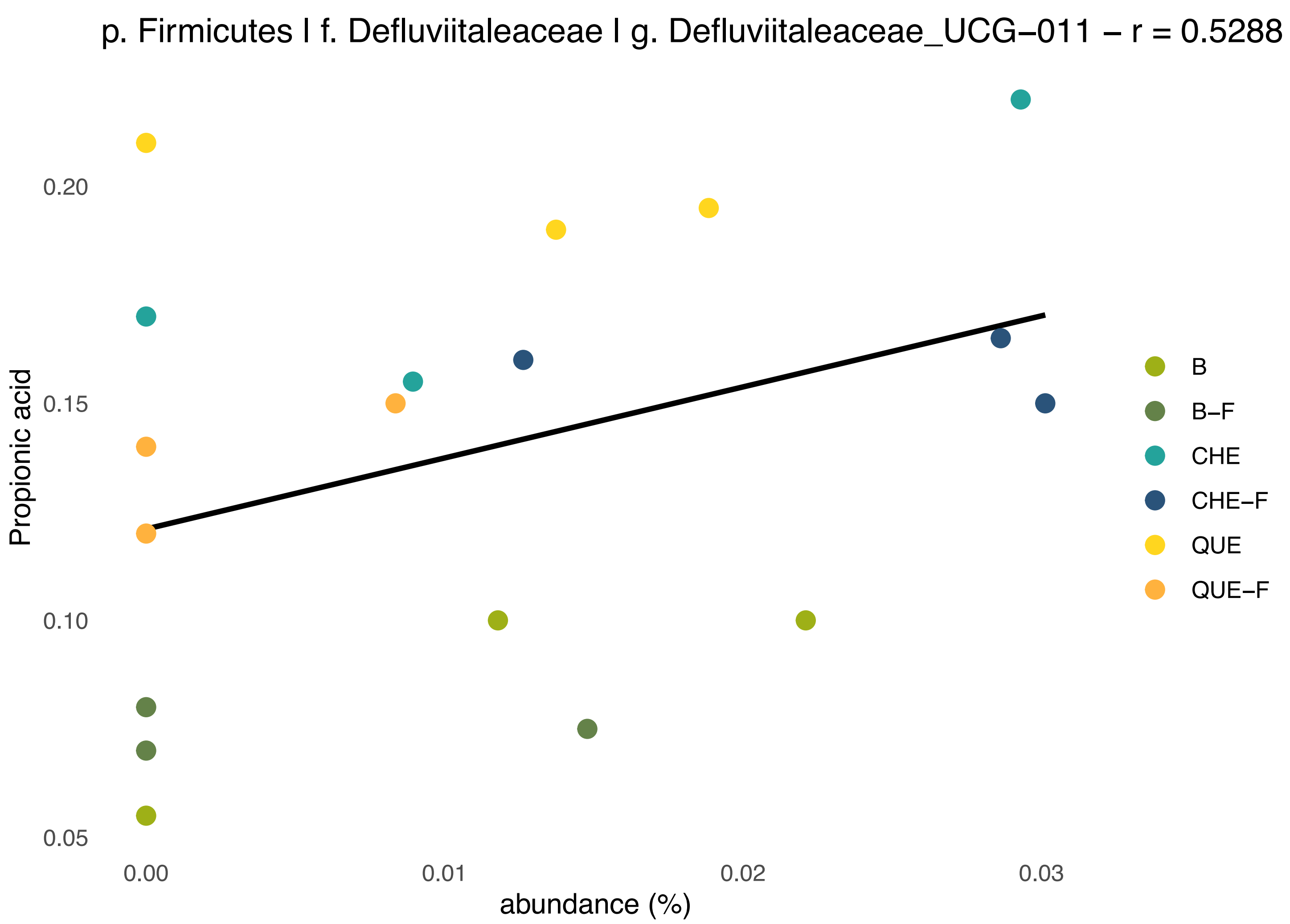
p. Firmicutes I f. Defluviitaleaceae I g. Defluviitaleaceae_UCG−011 − r = 0.5288
Propionic acid
0.20
0.15
0.10
0.05
0.00
0.01
0.02
0.03
abundance (%)
B
B−F
CHE
CHE−F
QUE
QUE−F

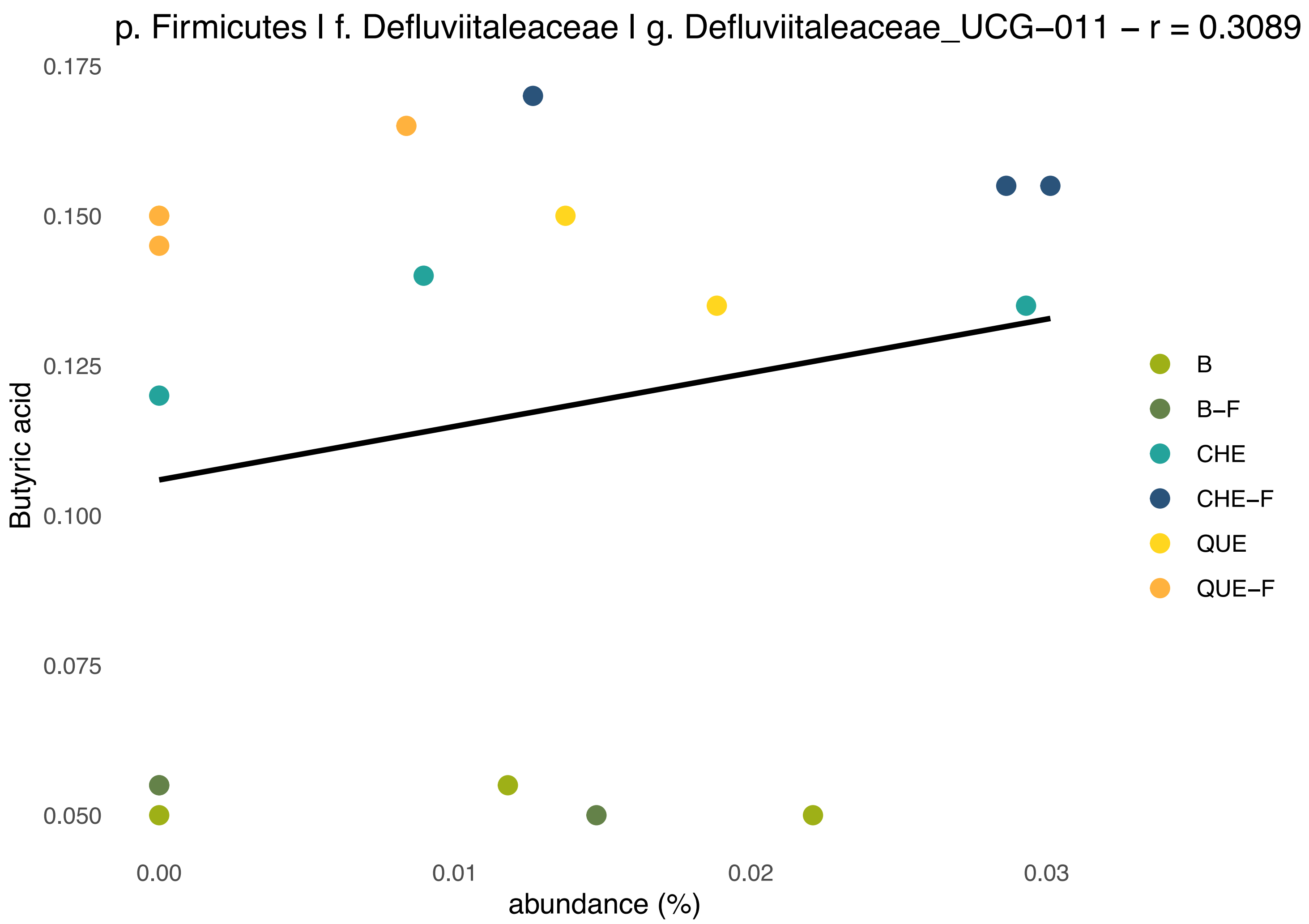
p. Firmicutes I f. Defluviitaleaceae I g. Defluviitaleaceae_UCG−011 − r = 0.3089
Butyric acid
0.175
0.150
0.125
0.100
0.075
0.050
0.00
0.01
0.02
0.03
abundance (%)
B
B−F
CHE
CHE−F
QUE
QUE−F

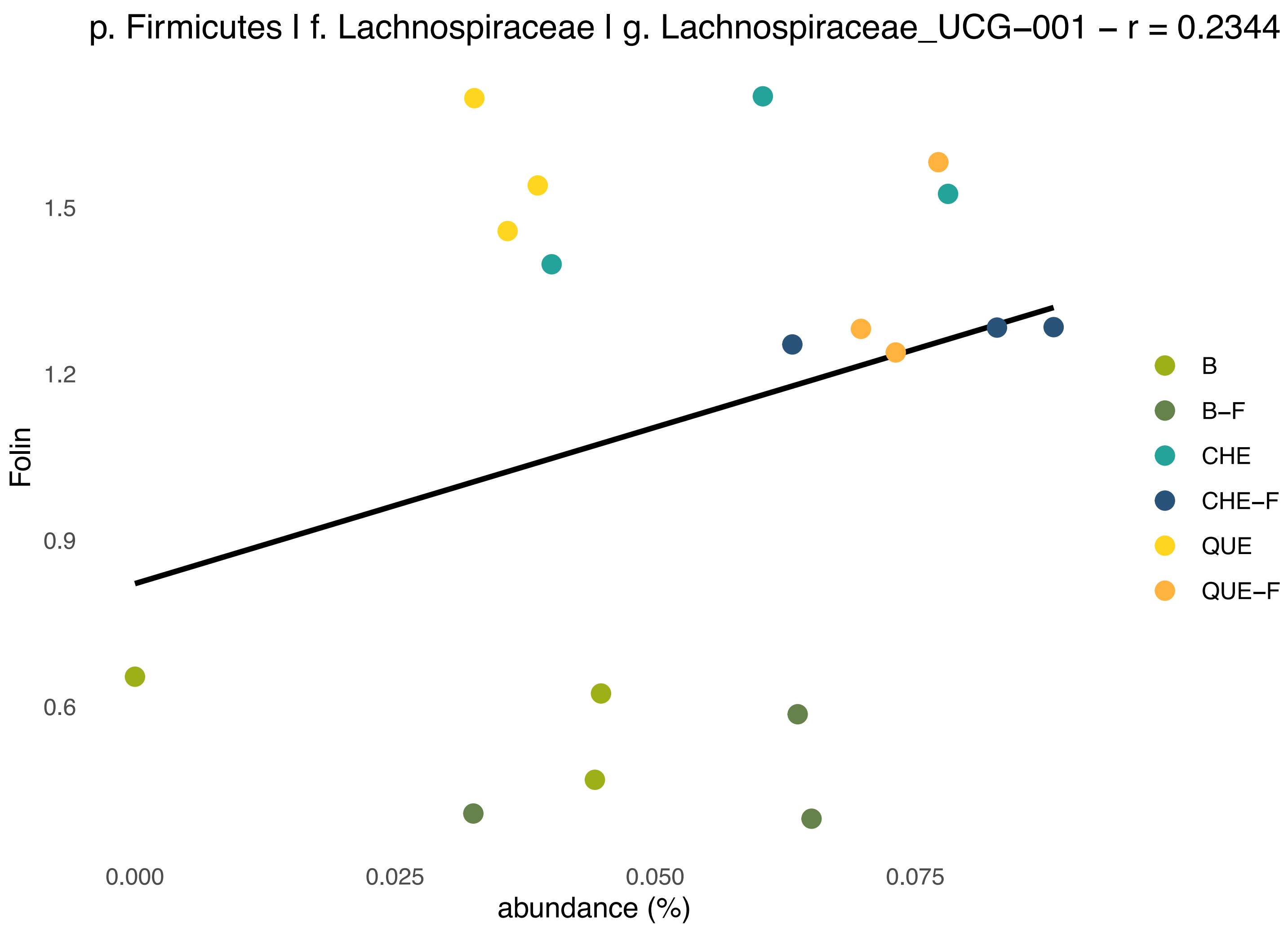

p. Firmicutes | f. Lachnospiraceae | g. Lachnospiraceae_UCG−001 − r = 0.2344
1.5
1.2
0.9
0.6
Folin
0.000
0.025
0.050
0.075
abundance (%)
B
B−F
CHE
CHE−F
QUE
QUE−F

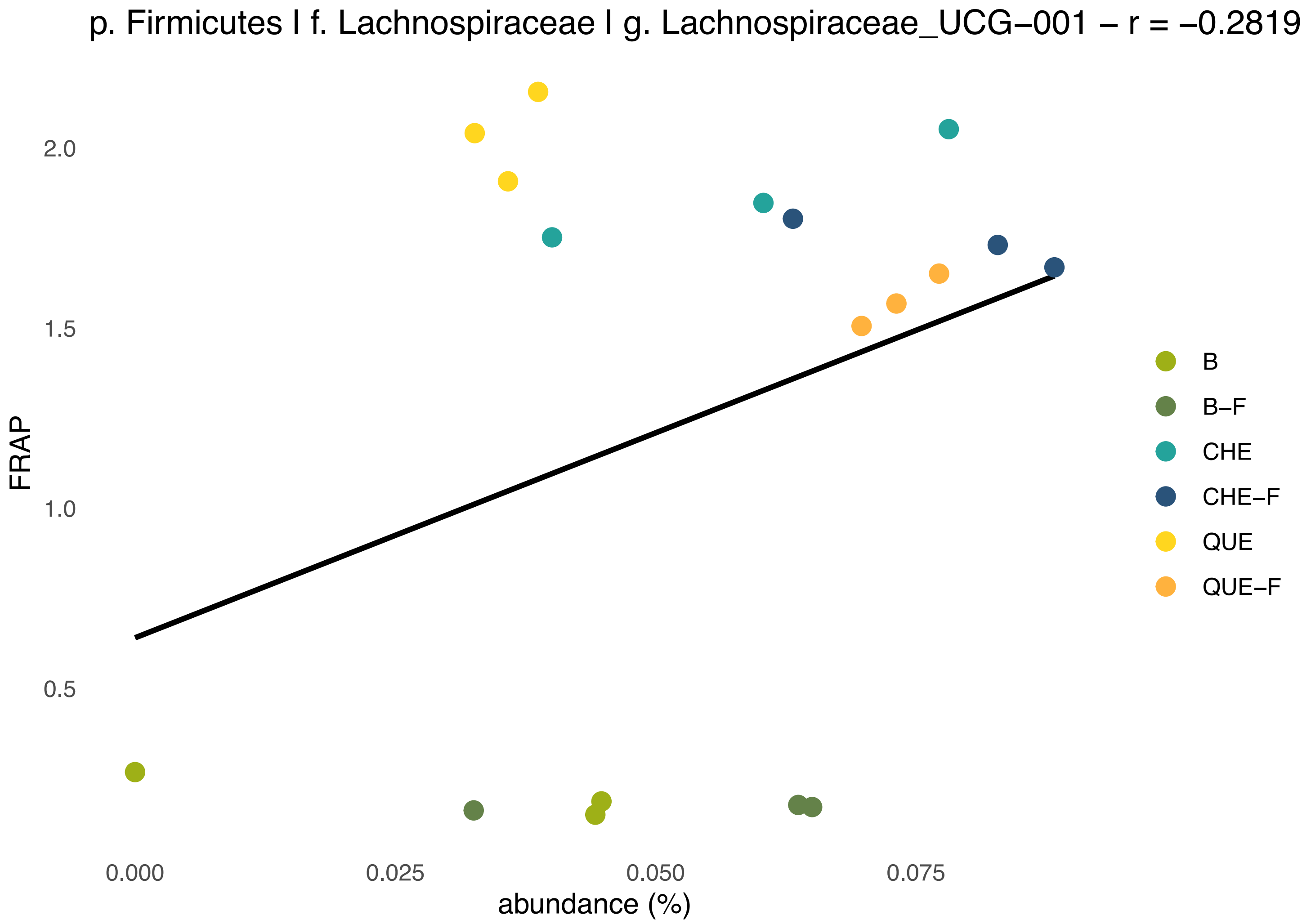
p. Firmicutes l f. Lachnospiraceae l g. Lachnospiraceae_UCG−001 − r = −0.2819
FRAP
2.0
1.5
1.0
0.5
0.000
0.025
0.050
0.075
abundance (%)
B
B−F
CHE
CHE−F
QUE
QUE−F

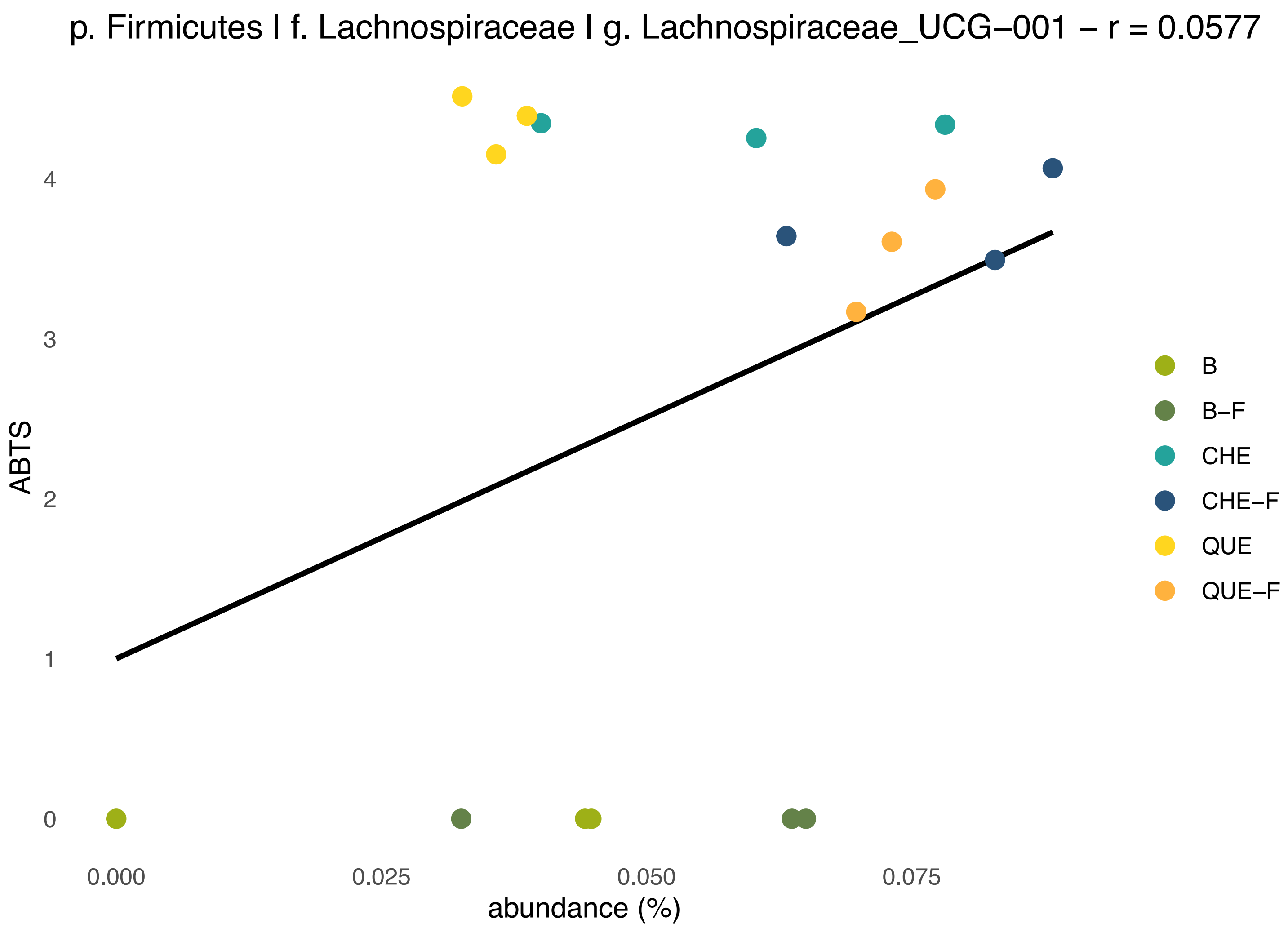
p. Firmicutes I f. Lachnospiraceae I g. Lachnospiraceae_UCG−001 − r = 0.0577
ABTS
4
3
2
1
0
0.000
0.025
0.050
0.075
abundance (%)
B
B−F
CHE
CHE−F
QUE
QUE−F

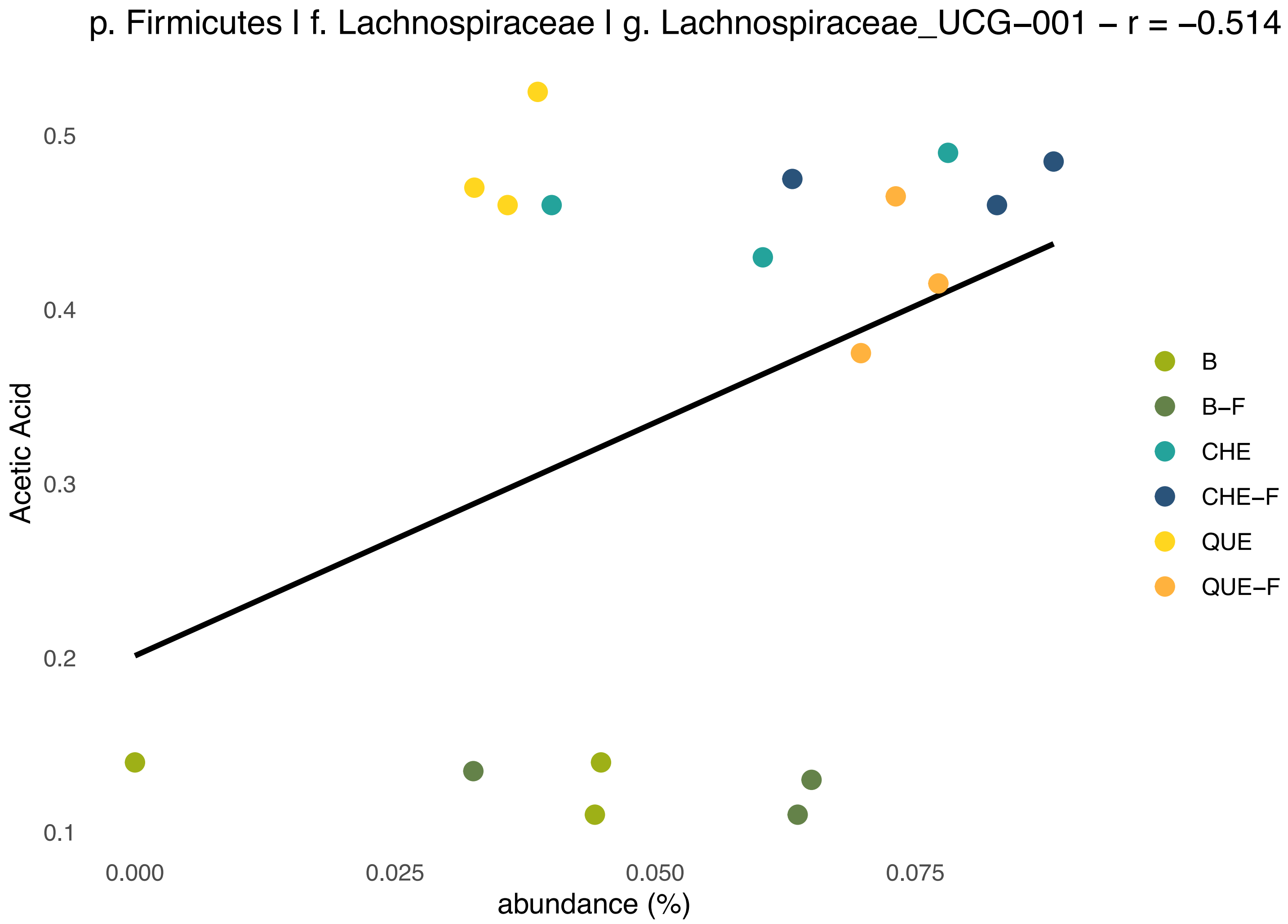
p. Firmicutes | f. Lachnospiraceae | g. Lachnospiraceae_UCG−001 − r = −0.514
Acetic Acid
0.5
0.4
0.3
0.2
0.1
0.000
0.025
0.050
0.075
abundance (%)
B
B−F
CHE
CHE−F
QUE
QUE−F

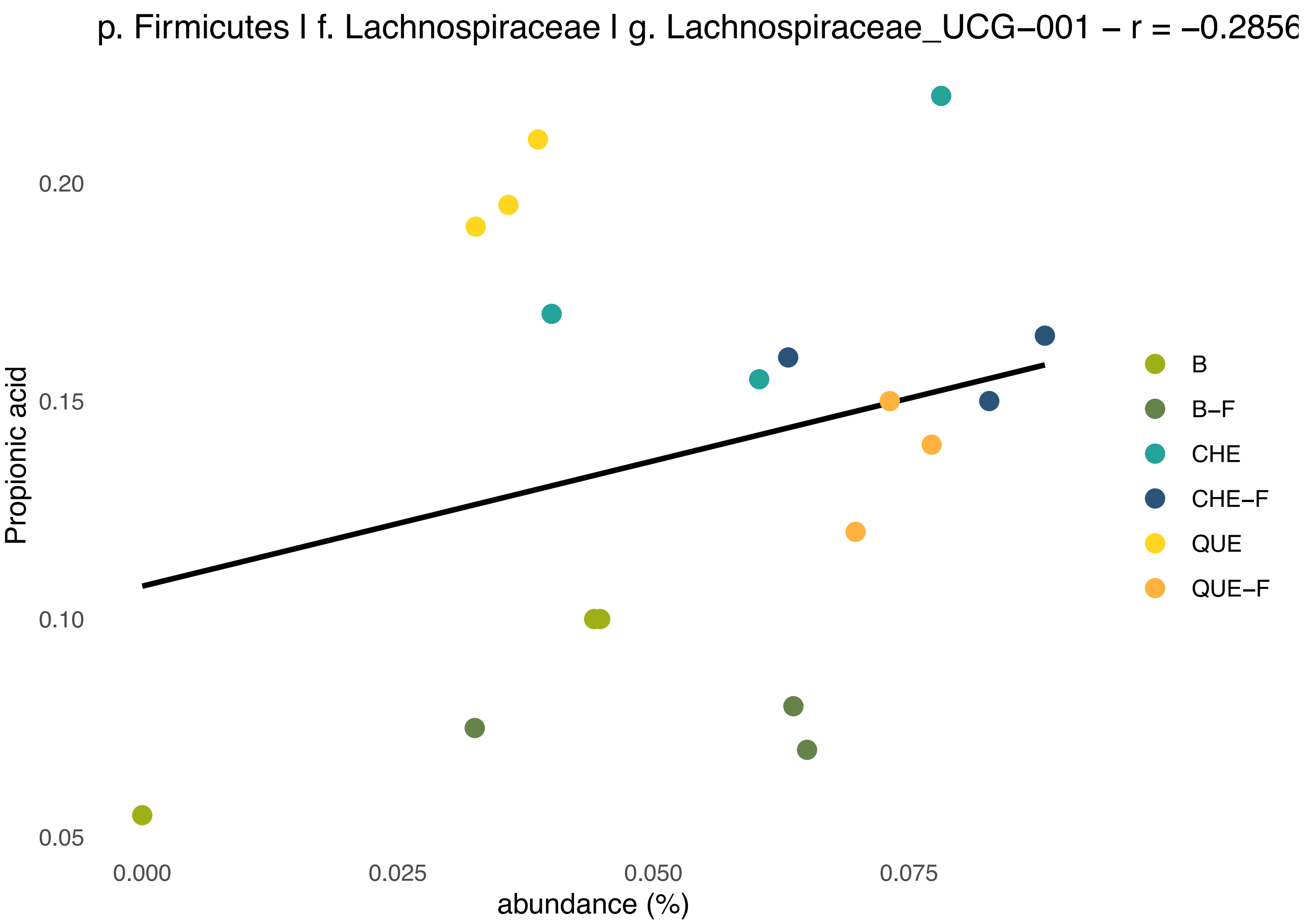

p. Firmicutes I f. Lachnospiraceae I g. Lachnospiraceae_UCG−001 − r = −0.2856
Propionic acid
0.20
0.15
0.10
0.05
0.000
0.025
0.050
0.075
abundance (%)
B
B−F
CHE
CHE−F
QUE
QUE−F

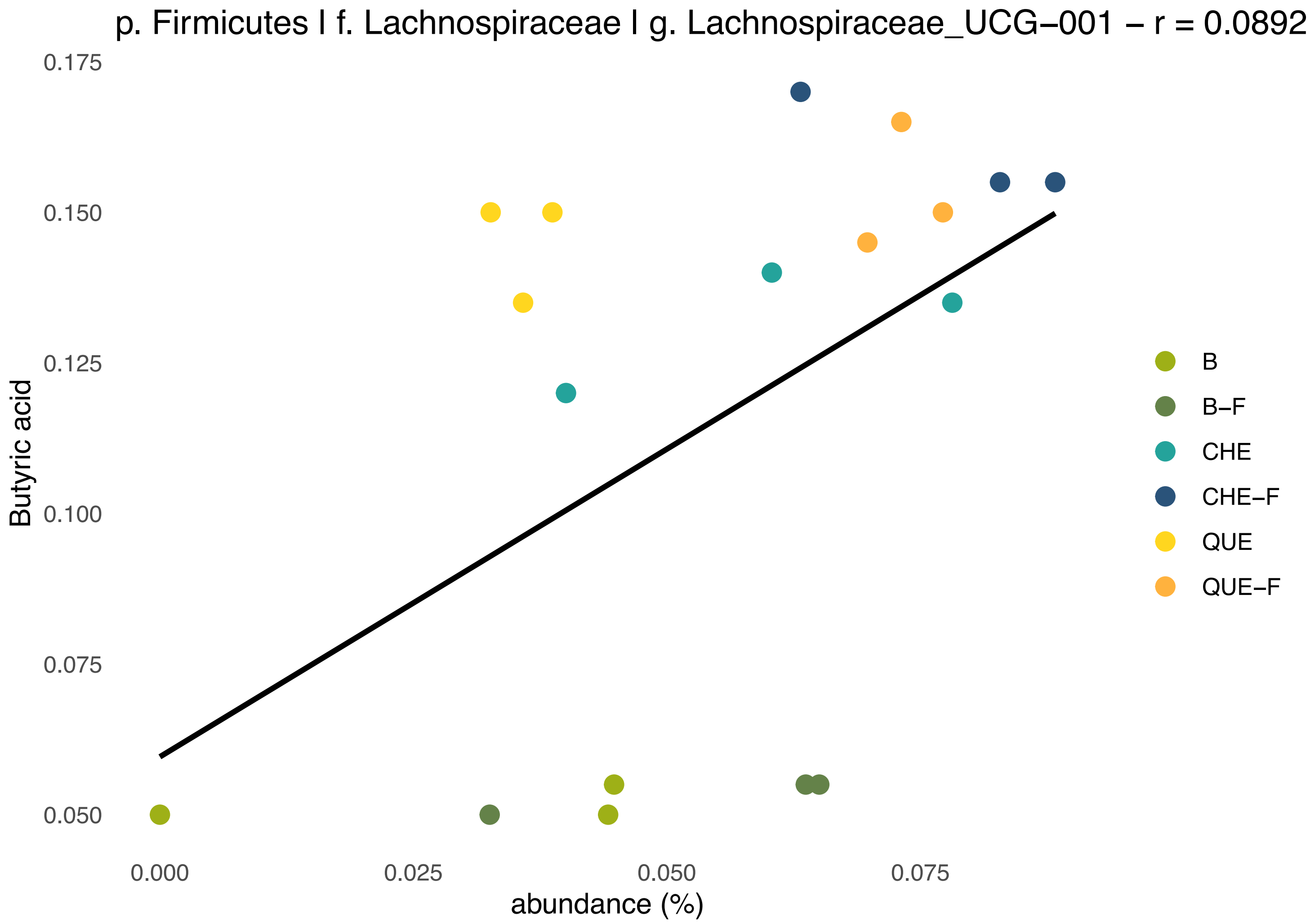
p. Firmicutes I f. Lachnospiraceae I g. Lachnospiraceae_UCG−001 − r = 0.0892
Butyric acid
0.175
0.150
0.125
0.100
0.075
0.050
0.000
0.025
0.050
0.075
abundance (%)
B
B−F
CHE
CHE−F
QUE
QUE−F

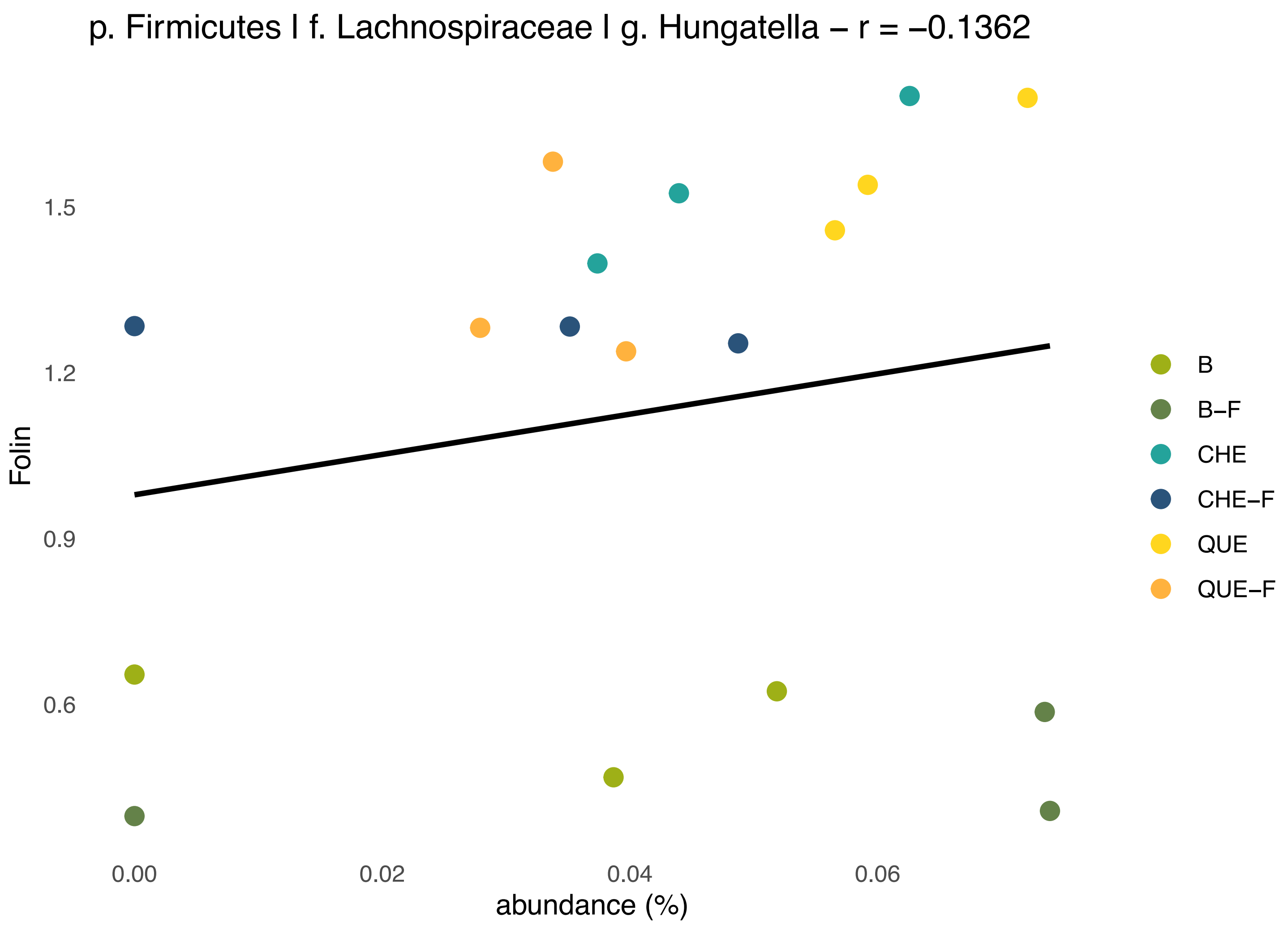
p. Firmicutes I f. Lachnospiraceae I g. Hungatella – r = –0.1362
1.5
1.2
0.9
0.6
Folin
0.00
0.02
0.04
0.06
abundance (%)
B
B–F
CHE
CHE–F
QUE
QUE–F

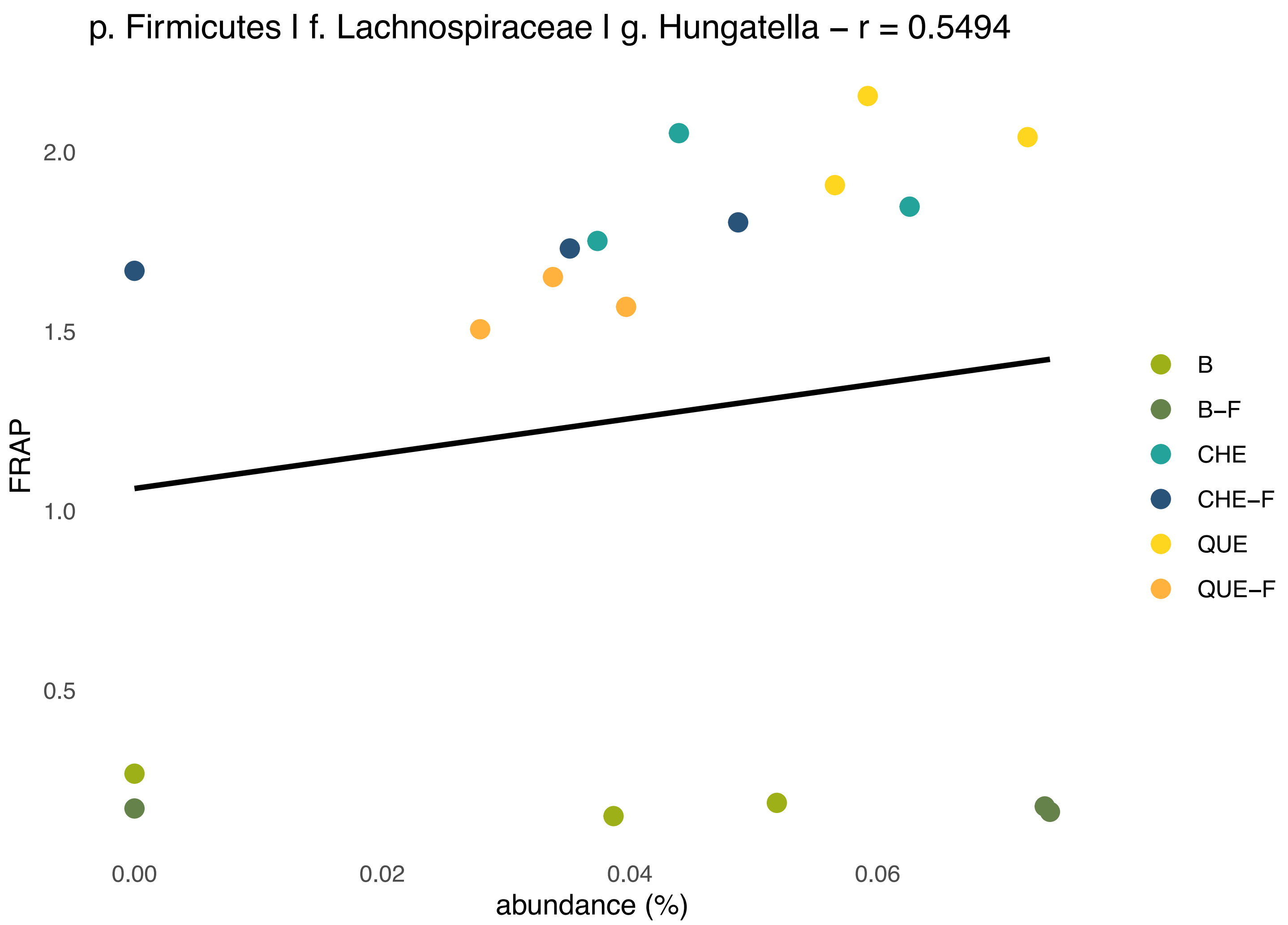
p. Firmicutes I f. Lachnospiraceae I g. Hungatella – r = 0.5494
FRAP
2.0
1.5
1.0
0.5
0.00
0.02
0.04
0.06
abundance (%)
B
B–F
CHE
CHE–F
QUE
QUE–F

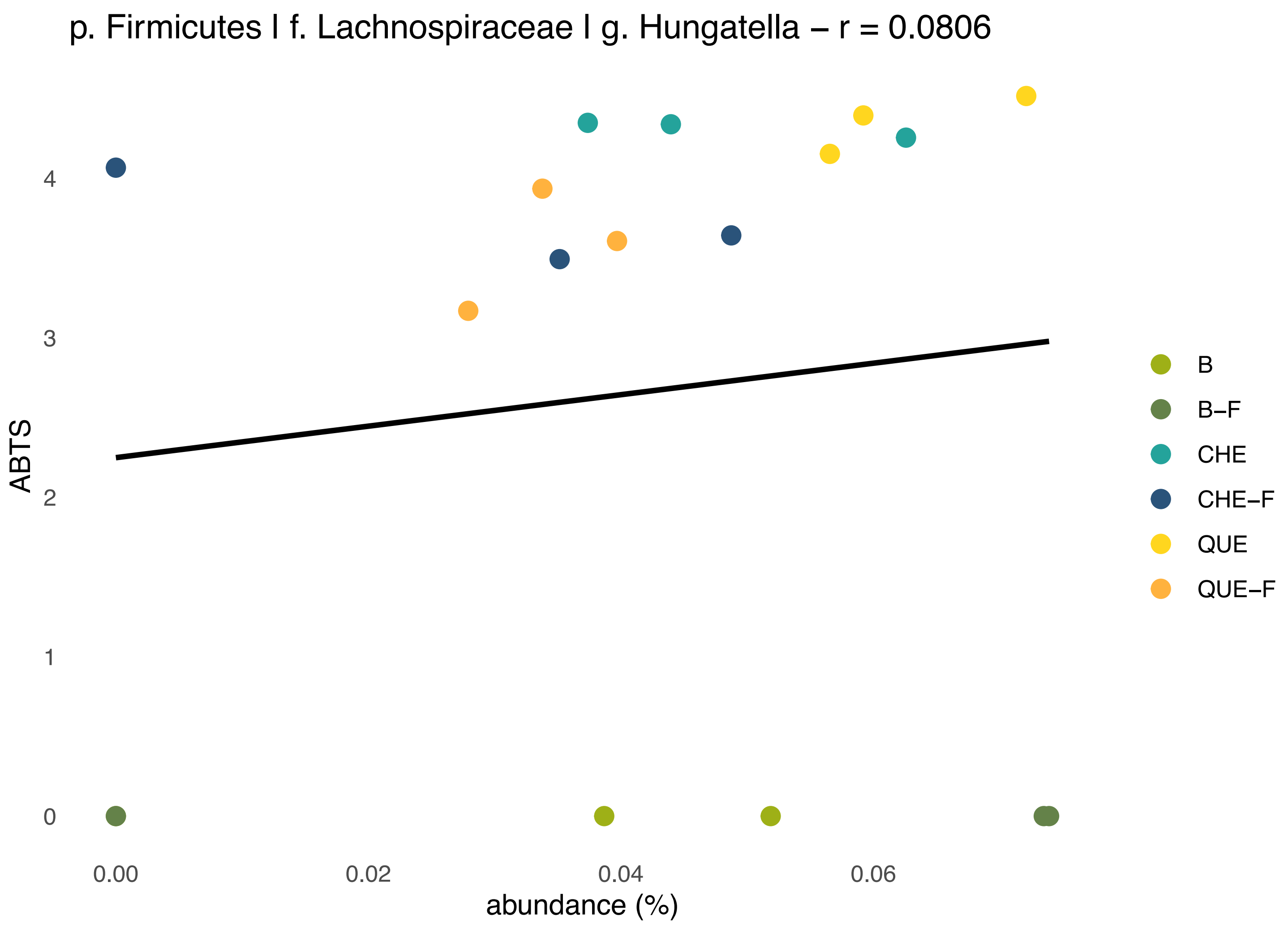

p. Firmicutes I f. Lachnospiraceae I g. Hungatella – r = 0.0806
ABTS
0
1
2
3
4
0.00
0.02
0.04
0.06
abundance (%)
B
B–F
CHE
CHE–F
QUE
QUE–F

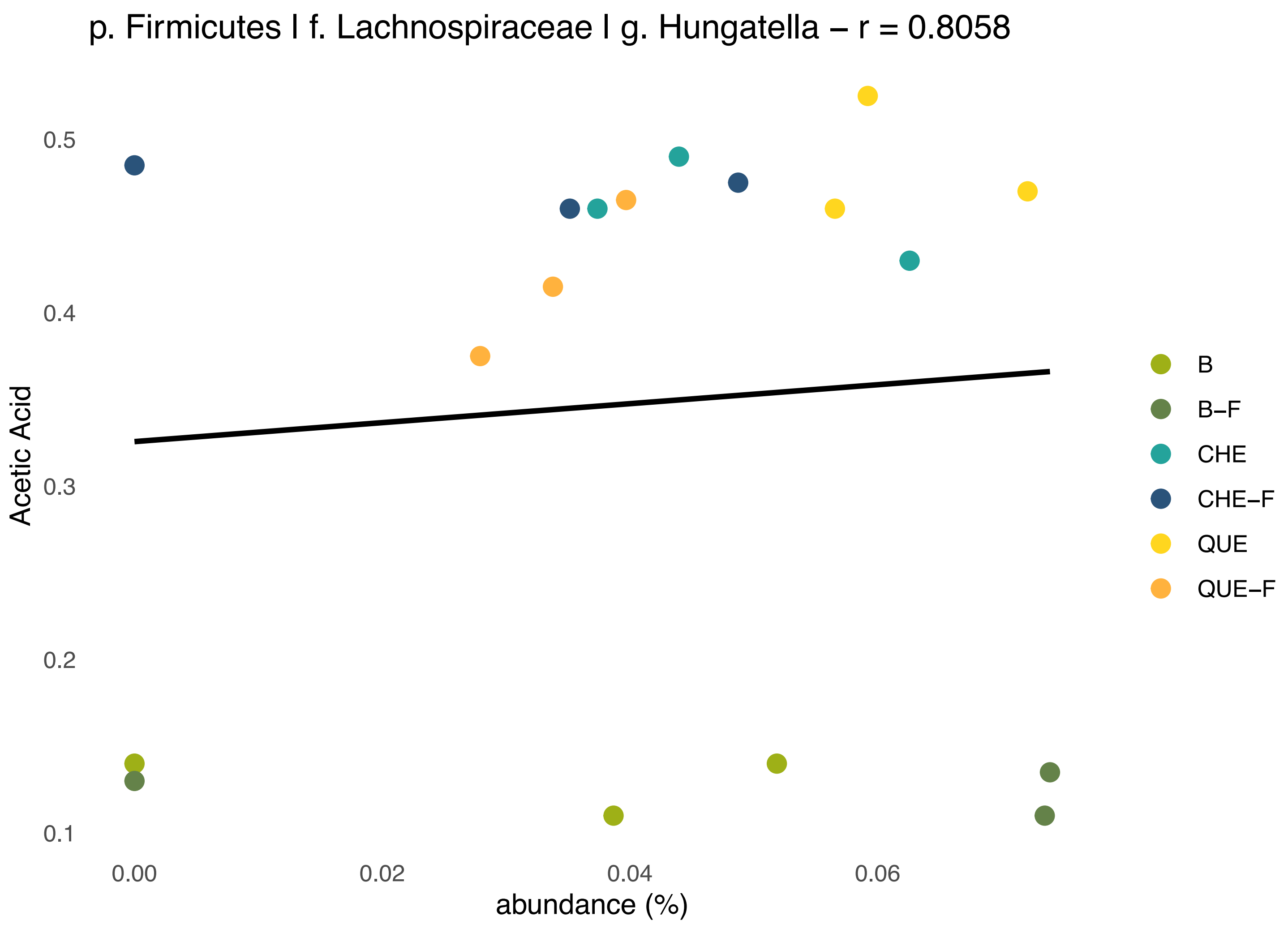

p. Firmicutes I f. Lachnospiraceae I g. Hungatella – r = 0.8058
Acetic Acid
0.5
0.4
0.3
0.2
0.1
0.00
0.02
0.04
0.06
abundance (%)
B
B–F
CHE
CHE–F
QUE
QUE–F

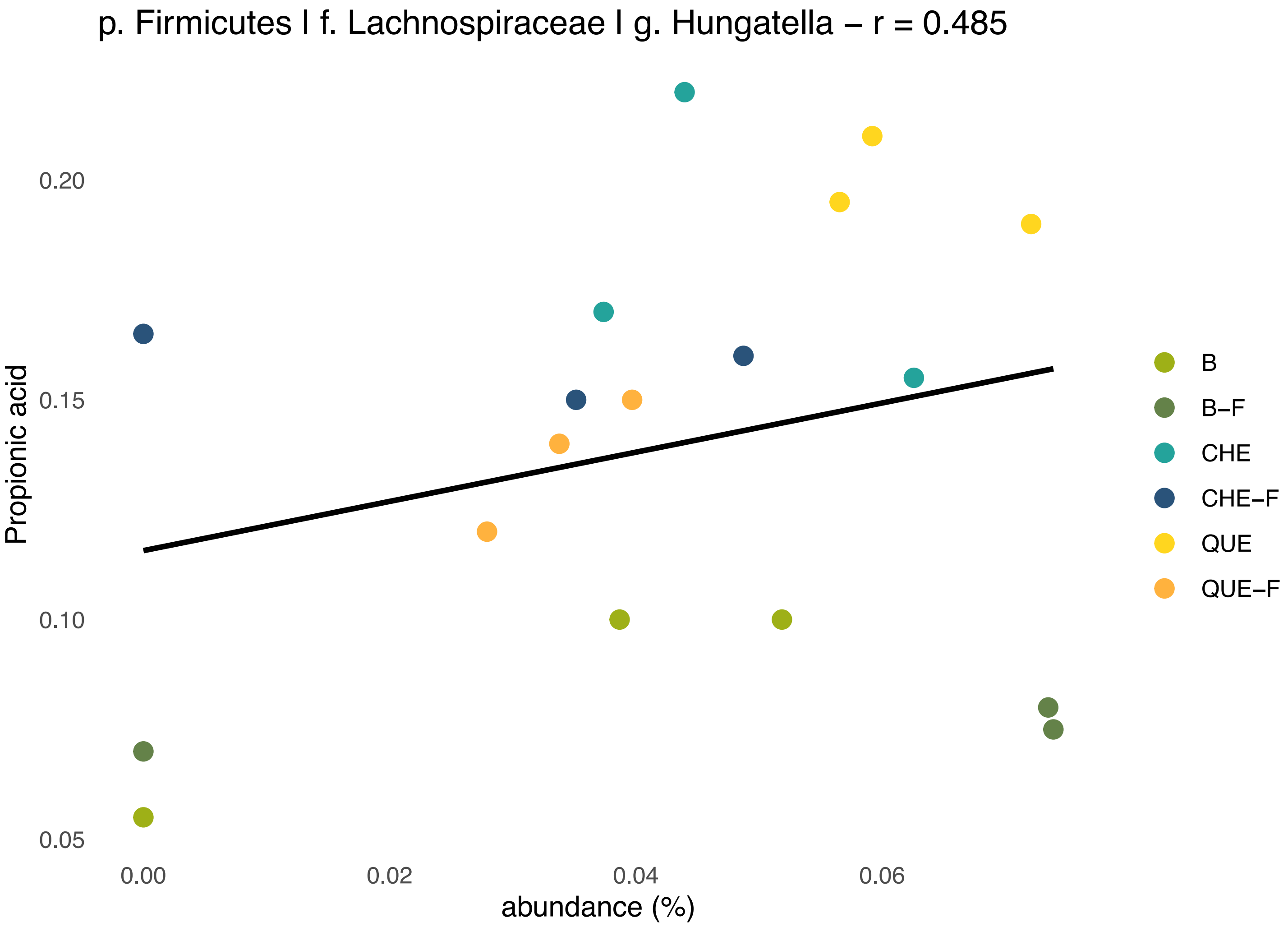
p. Firmicutes I f. Lachnospiraceae I g. Hungatella – r = 0.485
Propionic acid
0.20
0.15
0.10
0.05
0.00
0.02
0.04
0.06
abundance (%)
B
B–F
CHE
CHE–F
QUE
QUE–F

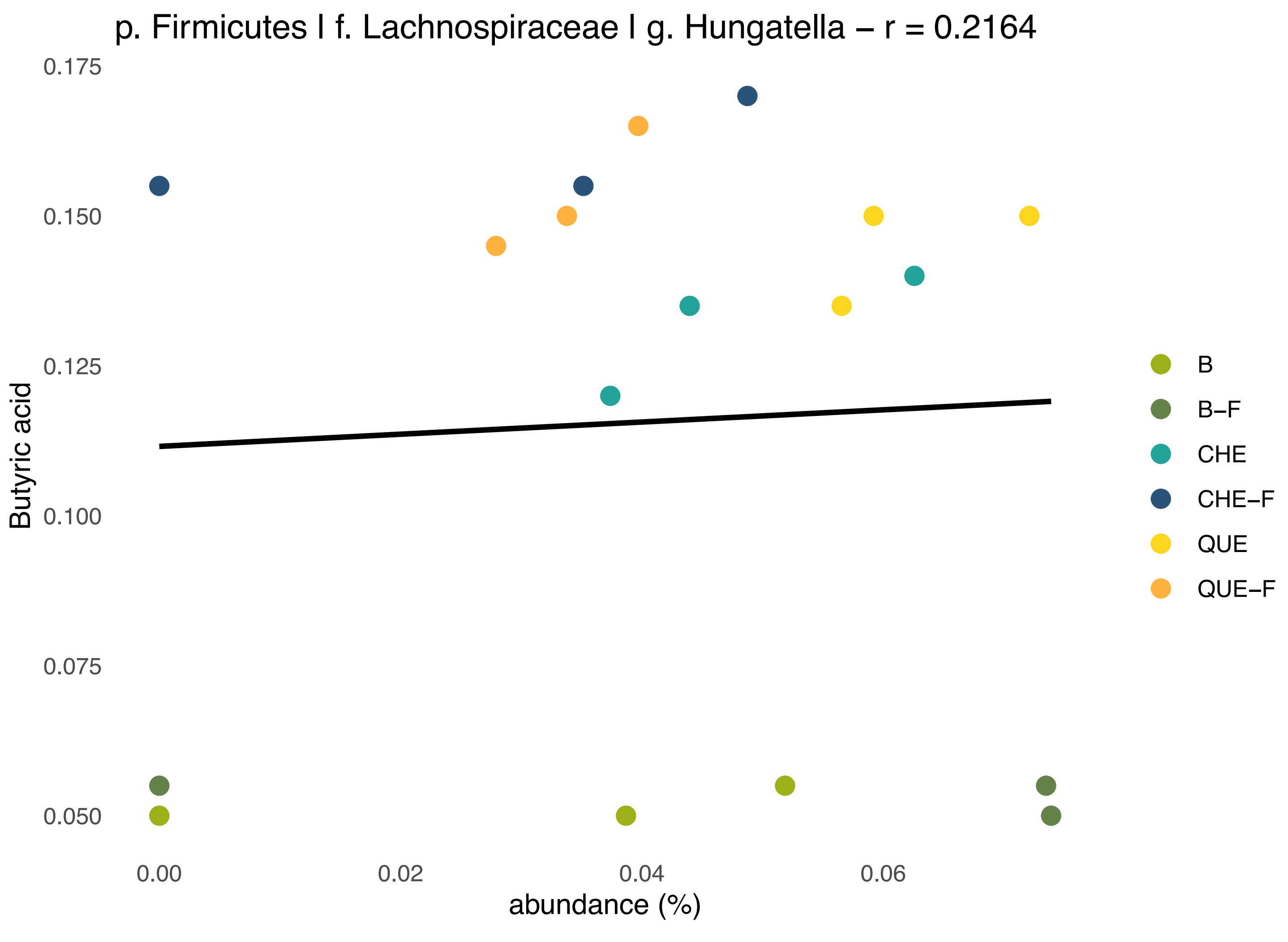
p. Firmicutes l f. Lachnospiraceae l g. Hungatella – r = 0.2164
Butyric acid
0.175
0.150
0.125
0.100
0.075
0.050
0.00
0.02
0.04
0.06
abundance (%)
B
B–F
CHE
CHE–F
QUE
QUE–F

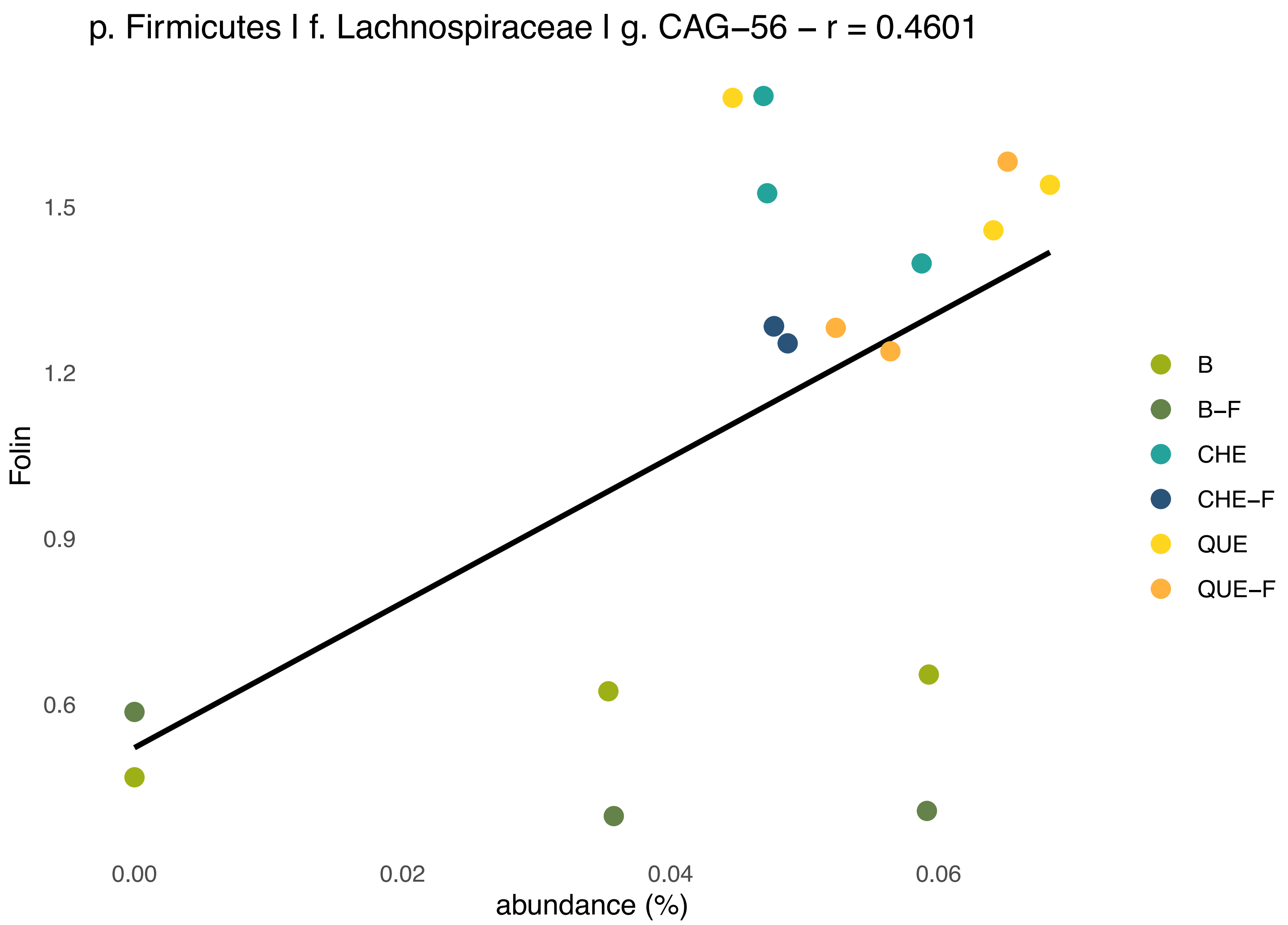
p. Firmicutes I f. Lachnospiraceae I g. CAG−56 − r = 0.4601
Folin
1.5
1.2
0.9
0.6
0.00
0.02
0.04
0.06
abundance (%)
B
B−F
CHE
CHE−F
QUE
QUE−F

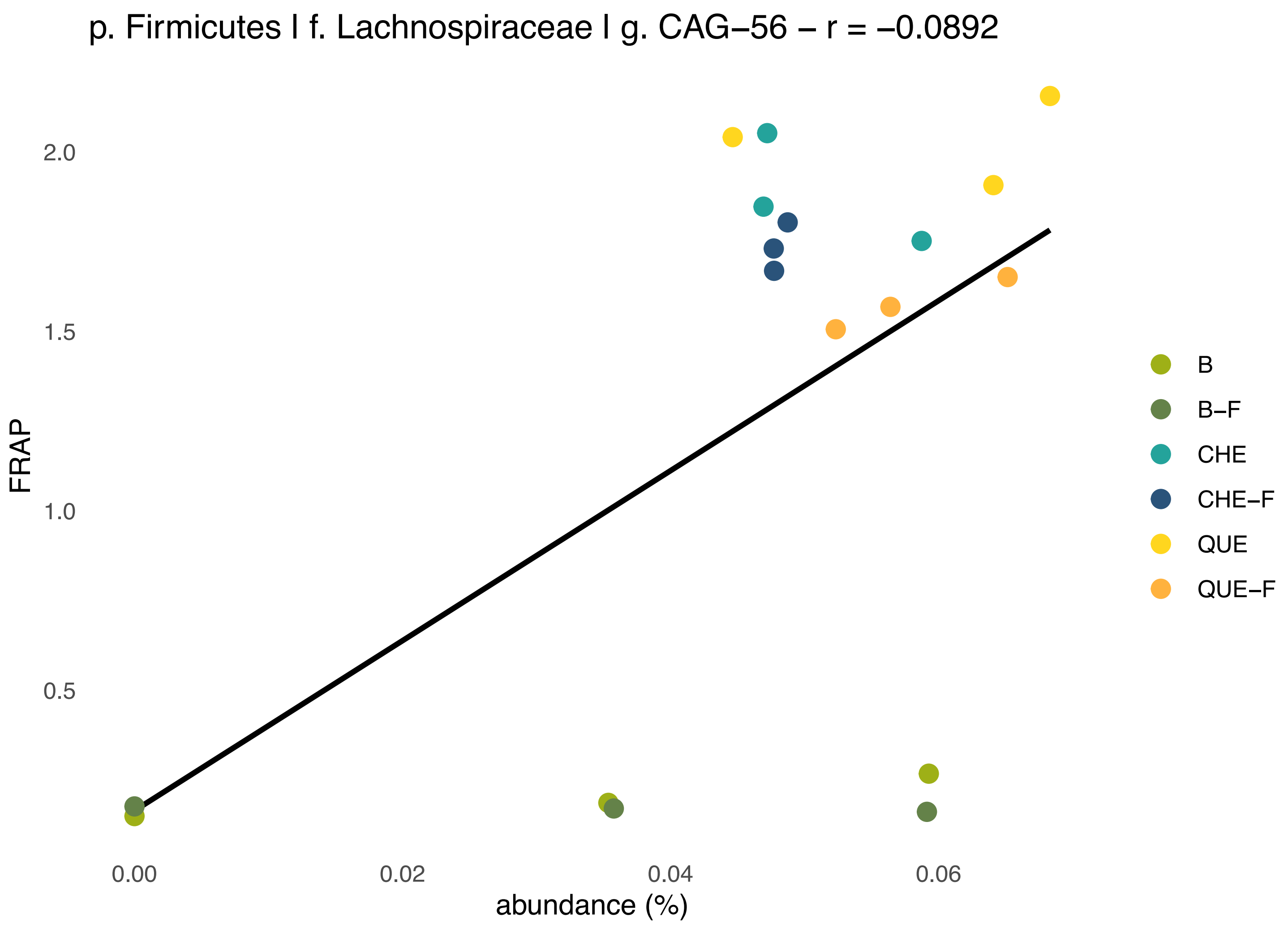

p. Firmicutes I f. Lachnospiraceae I g. CAG−56 − r = −0.0892
FRAP
2.0
1.5
1.0
0.5
0.00
0.02
0.04
0.06
abundance (%)
B
B−F
CHE
CHE−F
QUE
QUE−F

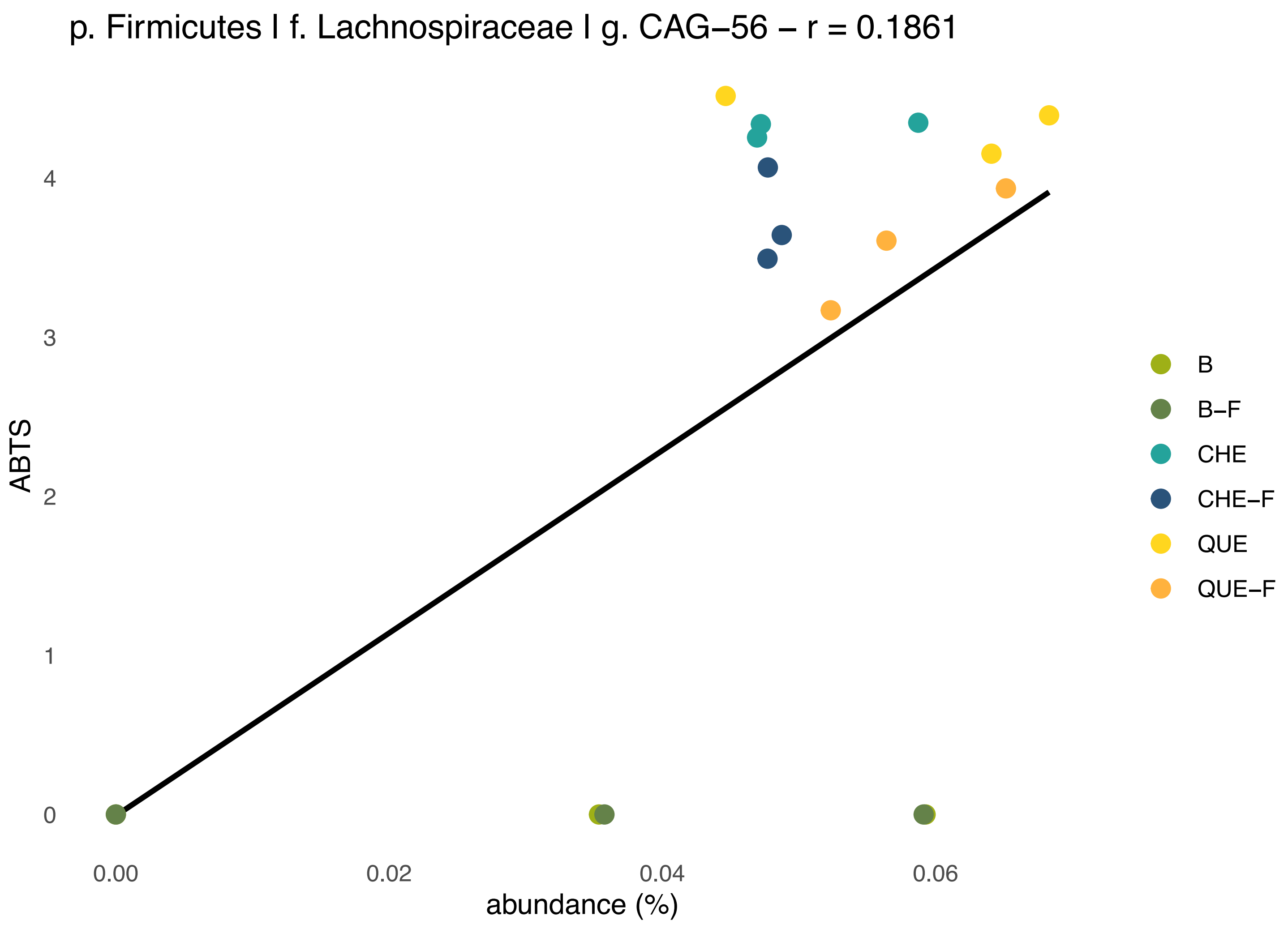

p. Firmicutes l f. Lachnospiraceae l g. CAG−56 − r = 0.1861
ABTS
4
3
2
1
0
0.00
0.02
0.04
0.06
abundance (%)
B
B−F
CHE
CHE−F
QUE
QUE−F

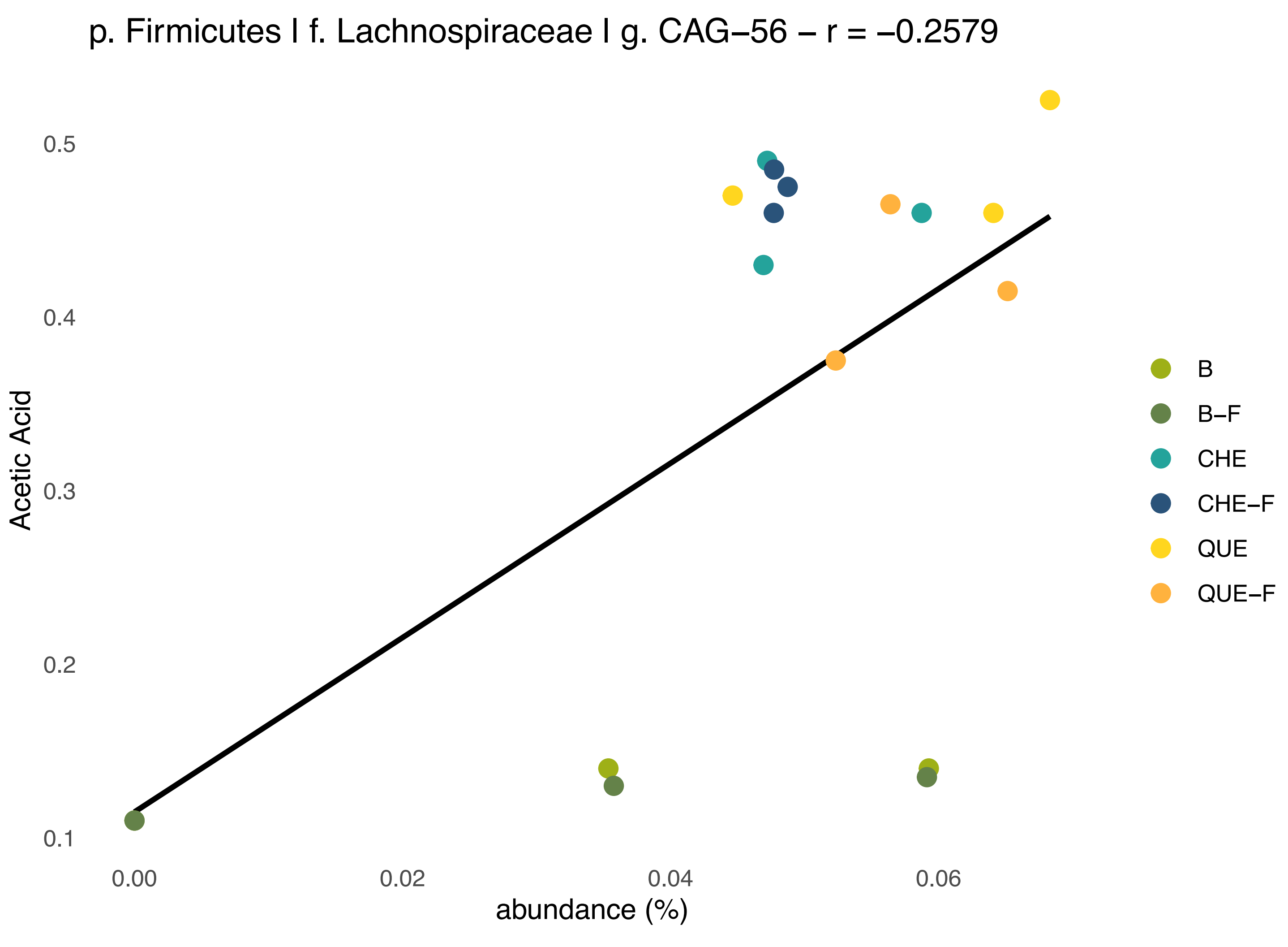
p. Firmicutes I f. Lachnospiraceae I g. CAG−56 − r = −0.2579
Acetic Acid
0.5
0.4
0.3
0.2
0.1
0.00
0.02
0.04
0.06
abundance (%)
B
B−F
CHE
CHE−F
QUE
QUE−F

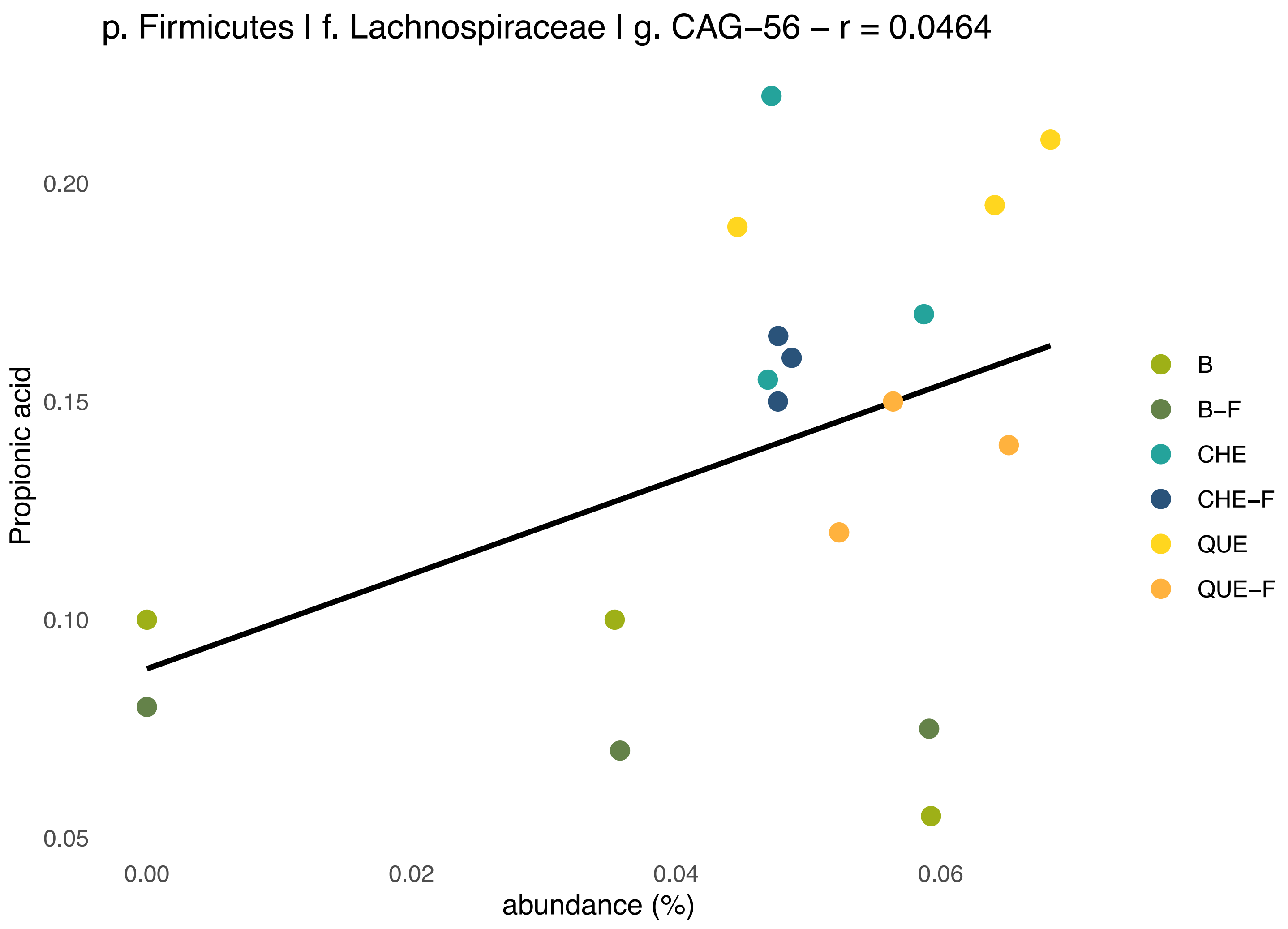

p. Firmicutes I f. Lachnospiraceae I g. CAG−56 − r = 0.0464
Propionic acid
0.20
0.15
0.10
0.05
0.00
0.02
0.04
0.06
abundance (%)
B
B−F
CHE
CHE−F
QUE
QUE−F

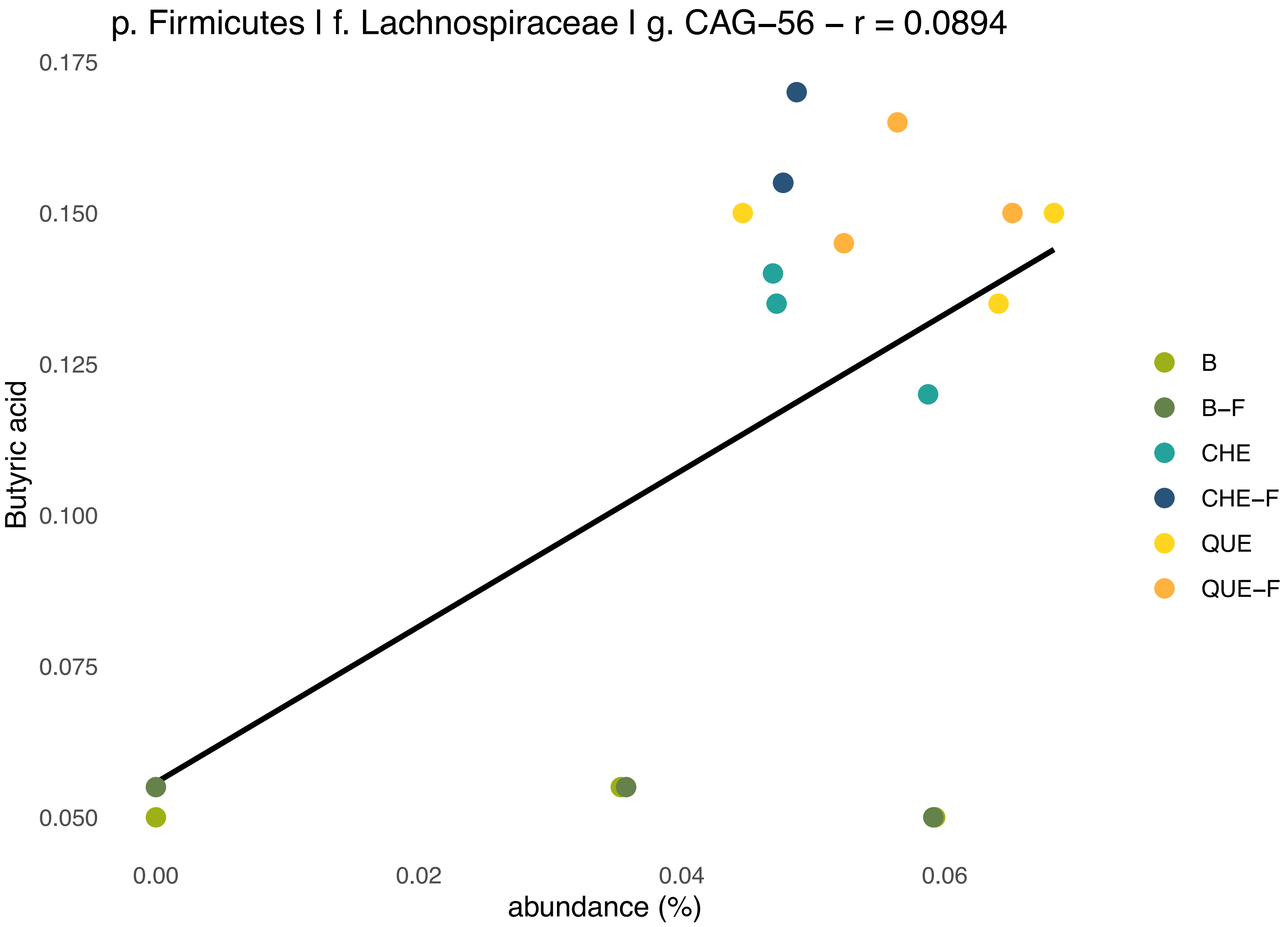

p. Firmicutes I f. Lachnospiraceae I g. CAG−56 − r = 0.0894
Butyric acid
0.175
0.150
0.125
0.100
0.075
0.050
0.00
0.02
0.04
0.06
abundance (%)
B
B−F
CHE
CHE−F
QUE
QUE−F

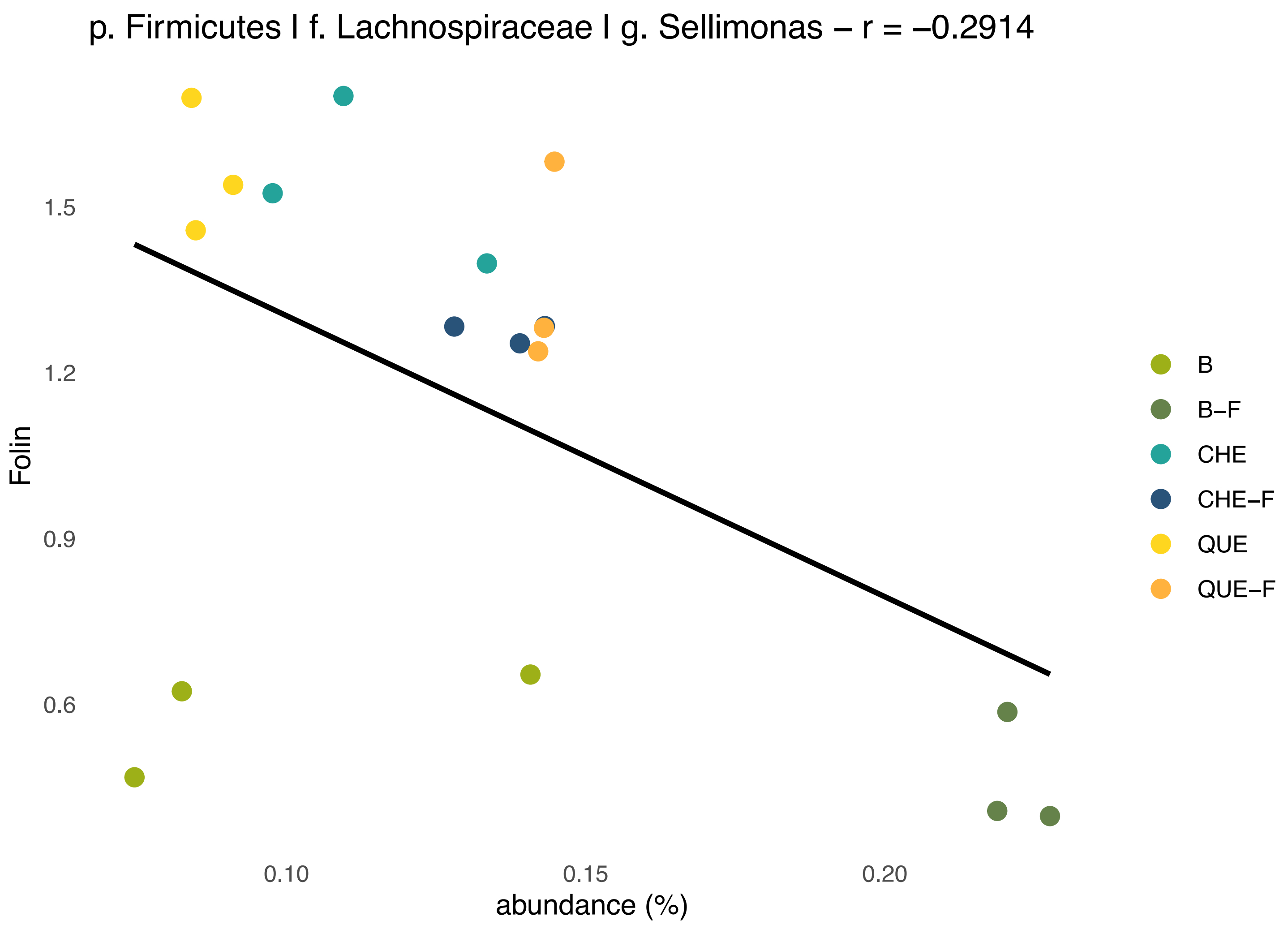

p. Firmicutes | f. Lachnospiraceae | g. Sellimonas – r = –0.2914
Folin
1.5
1.2
0.9
0.6
0.10
0.15
0.20
abundance (%)
B
B–F
CHE
CHE–F
QUE
QUE–F

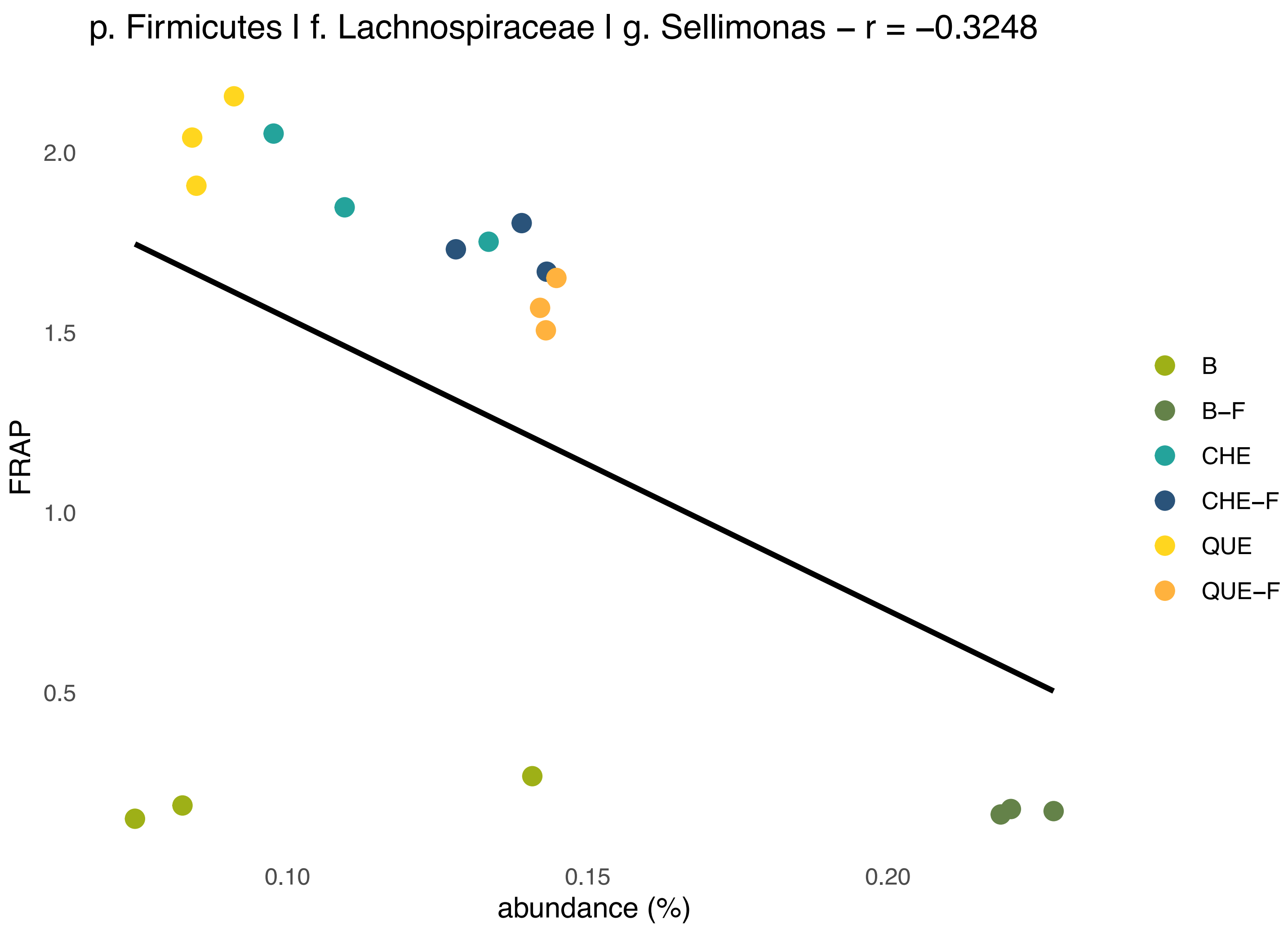

p. Firmicutes I f. Lachnospiraceae I g. Sellimonas – r = –0.3248
FRAP
2.0
1.5
1.0
0.5
0.10
0.15
0.20
abundance (%)
B
B–F
CHE
CHE–F
QUE
QUE–F

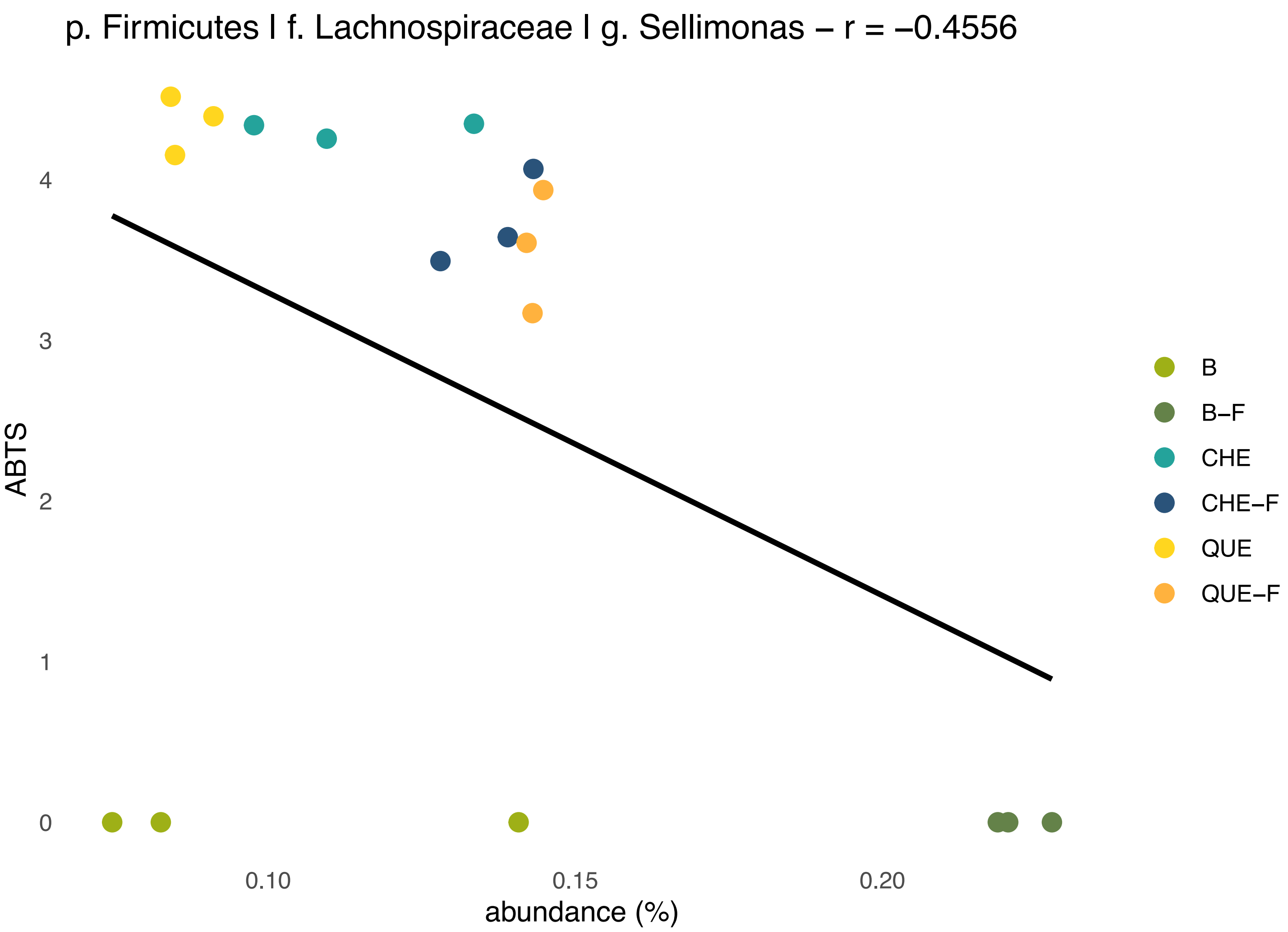

p. Firmicutes I f. Lachnospiraceae I g. Sellimonas – r = –0.4556
ABTS
4
3
2
1
0
0.10
0.15
0.20
abundance (%)
B
B–F
CHE
CHE–F
QUE
QUE–F

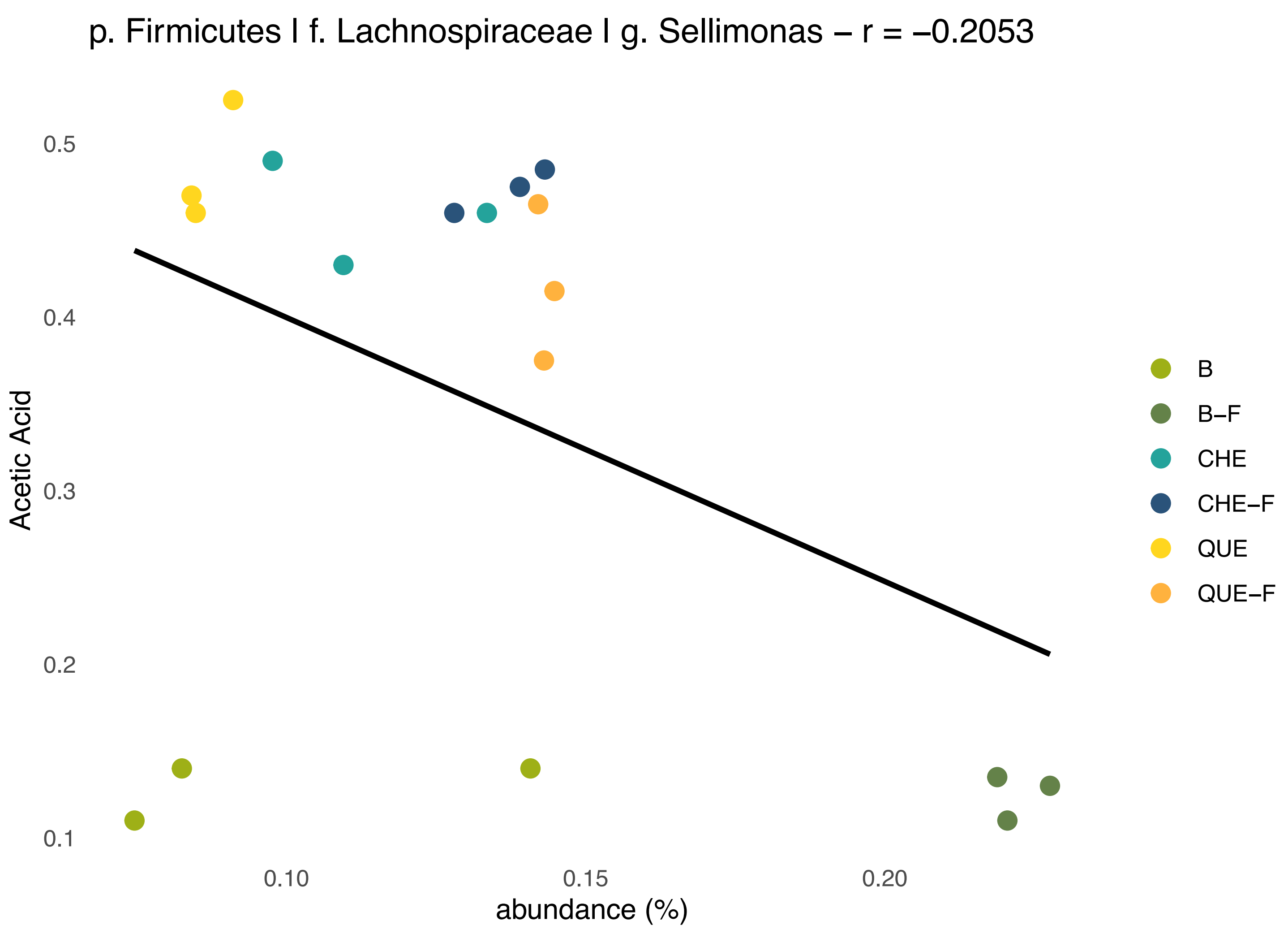

p. Firmicutes l f. Lachnospiraceae l g. Sellimonas – r = –0.2053
Acetic Acid
0.5
0.4
0.3
0.2
0.1
0.10
0.15
0.20
abundance (%)
B
B–F
CHE
CHE–F
QUE
QUE–F

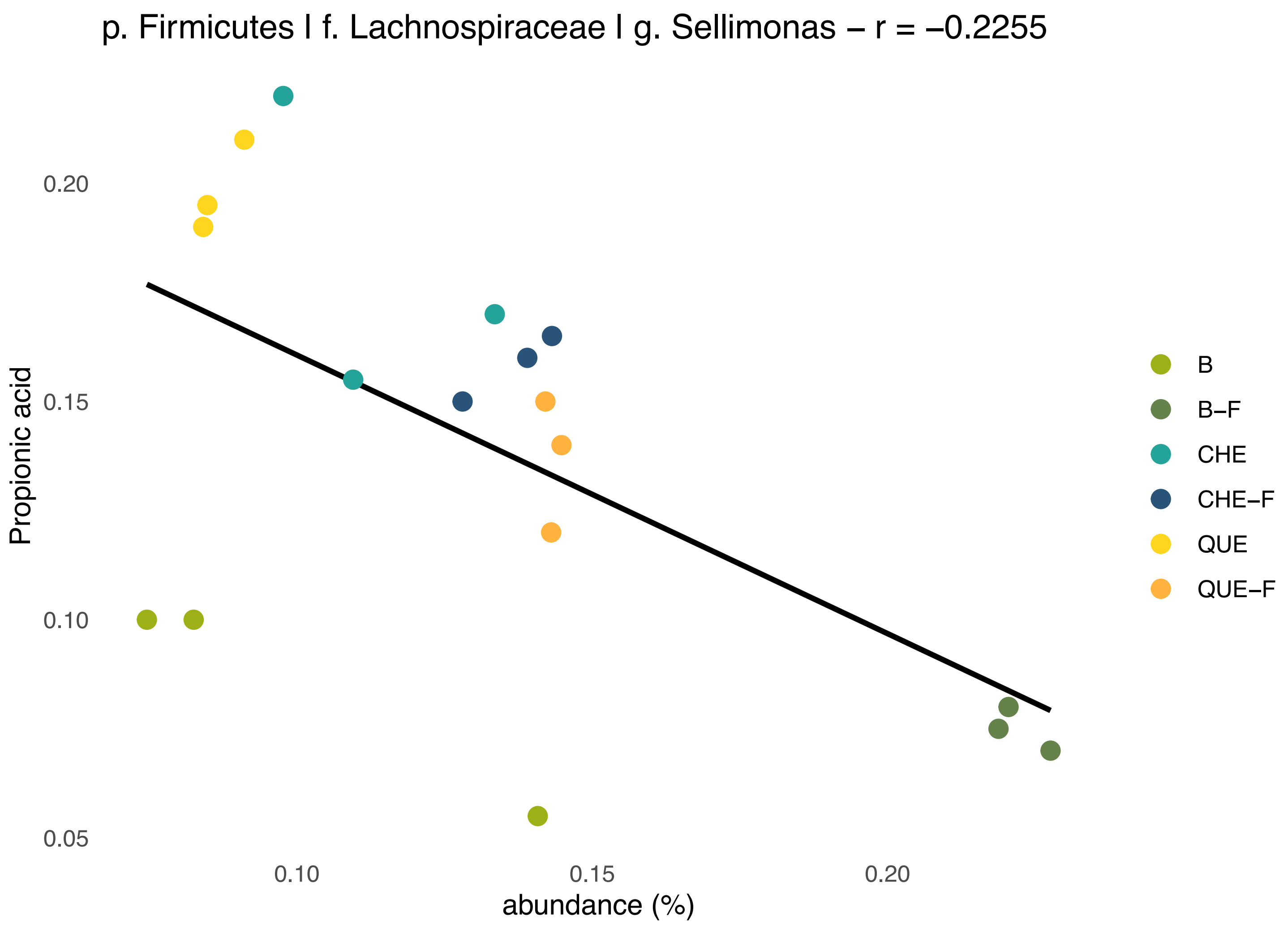

p. Firmicutes I f. Lachnospiraceae I g. Sellimonas – r = –0.2255
Propionic acid
0.20
0.15
0.10
0.05
0.10
0.15
0.20
abundance (%)
B
B–F
CHE
CHE–F
QUE
QUE–F

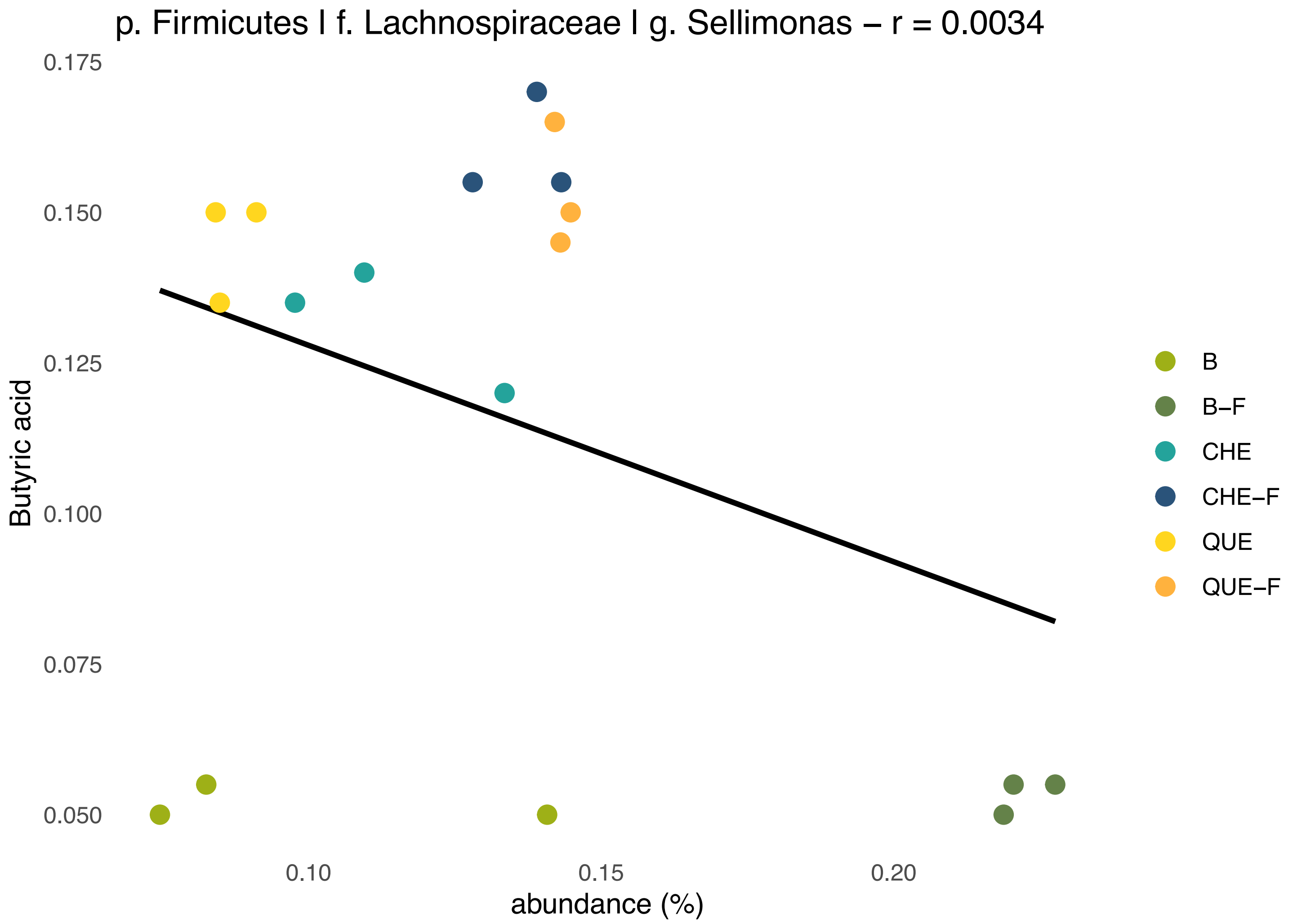

p. Firmicutes I f. Lachnospiraceae I g. Sellimonas – r = 0.0034
Butyric acid
0.175
0.150
0.125
0.100
0.075
0.050
0.10
0.15
0.20
abundance (%)
B
B–F
CHE
CHE–F
QUE
QUE–F

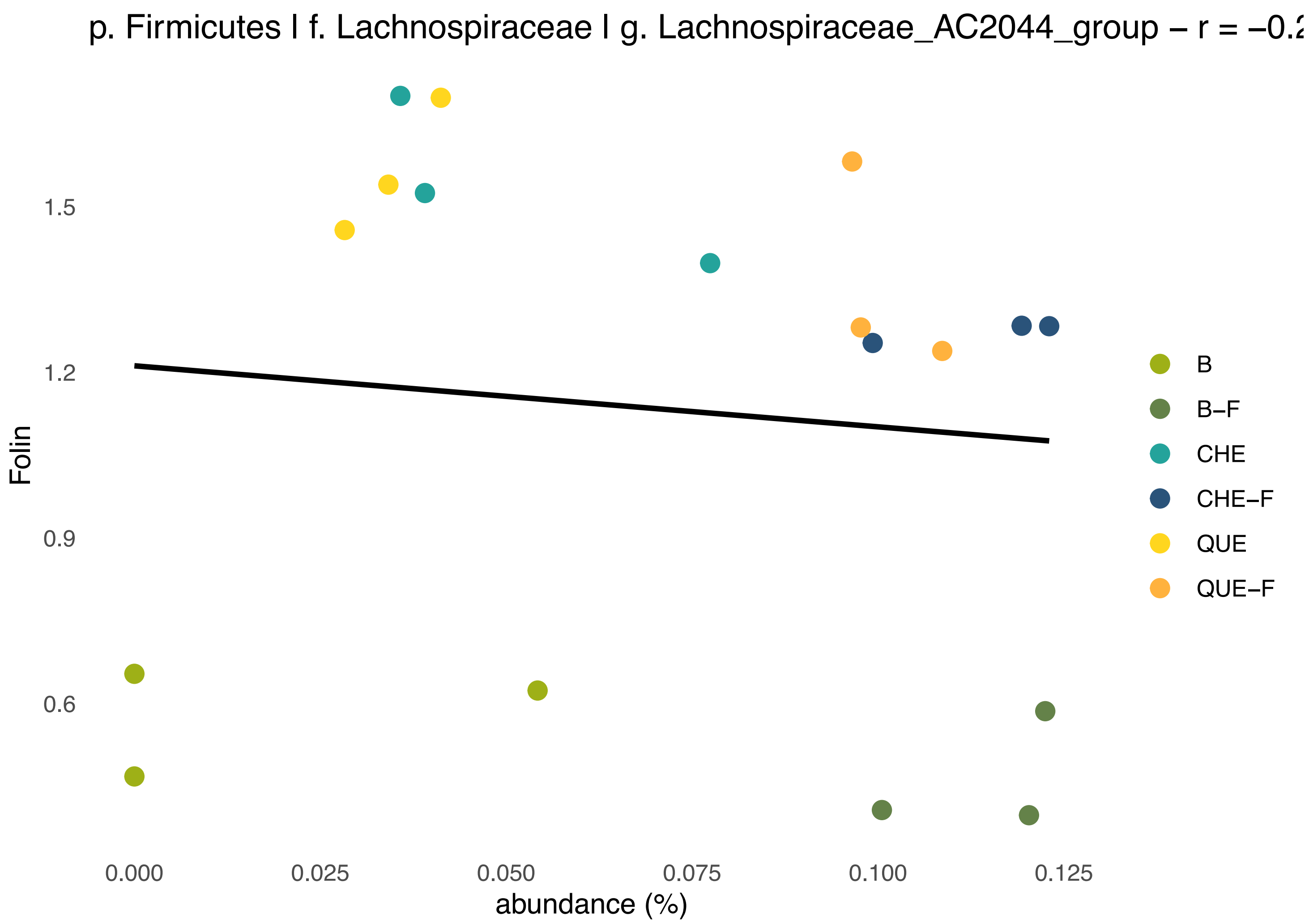

p. Firmicutes l f. Lachnospiraceae l g. Lachnospiraceae_AC2044_group – r = –0.2
Folin
1.5
1.2
0.9
0.6
0.000
0.025
0.050
0.075
0.100
0.125
abundance (%)
B
B–F
CHE
CHE–F
QUE
QUE–F

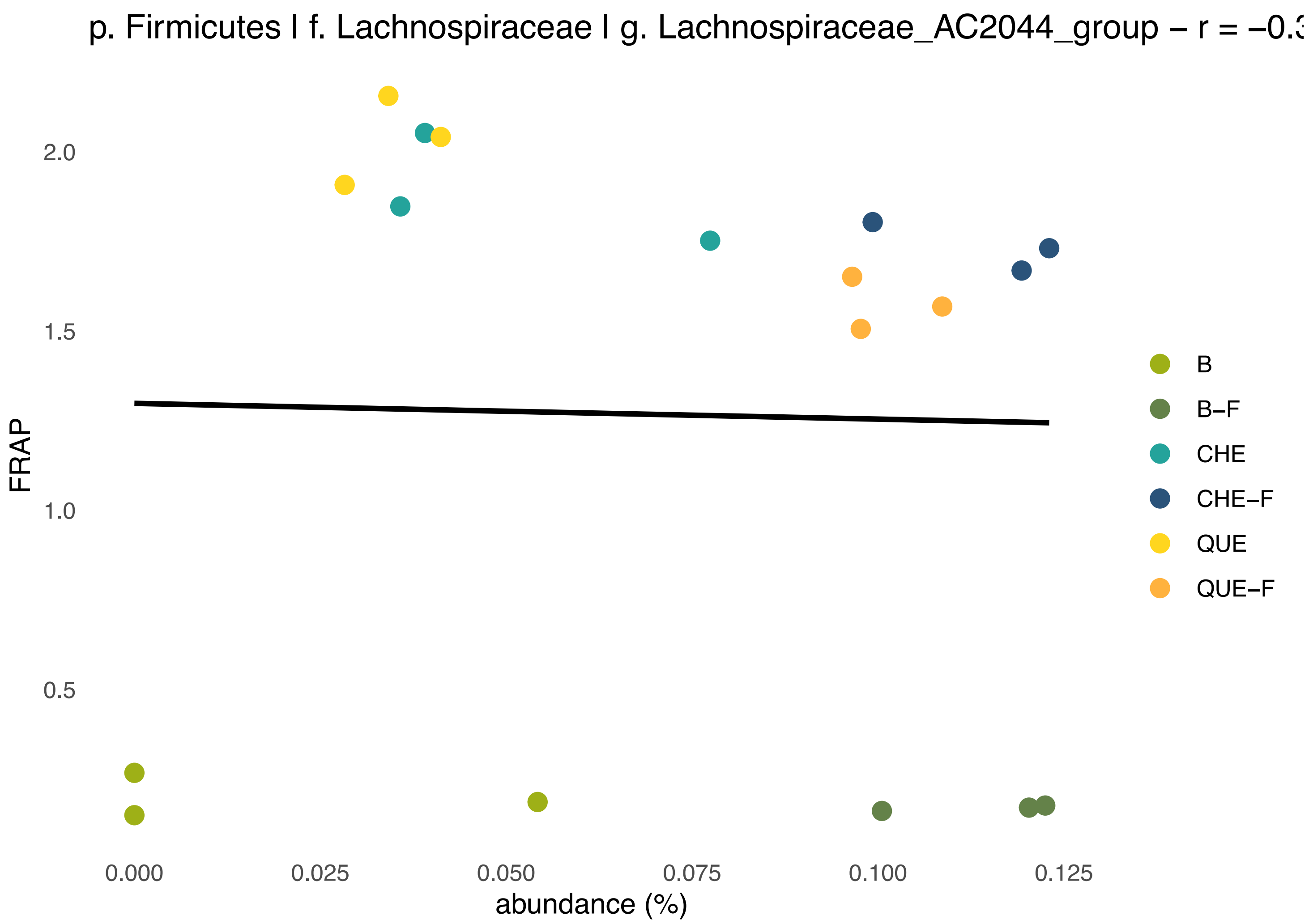
p. Firmicutes I f. Lachnospiraceae I g. Lachnospiraceae_AC2044_group – r = –0.3
FRAP
2.0
1.5
1.0
0.5
0.000
0.025
0.050
0.075
0.100
0.125
abundance (%)
B
B–F
CHE
CHE–F
QUE
QUE–F

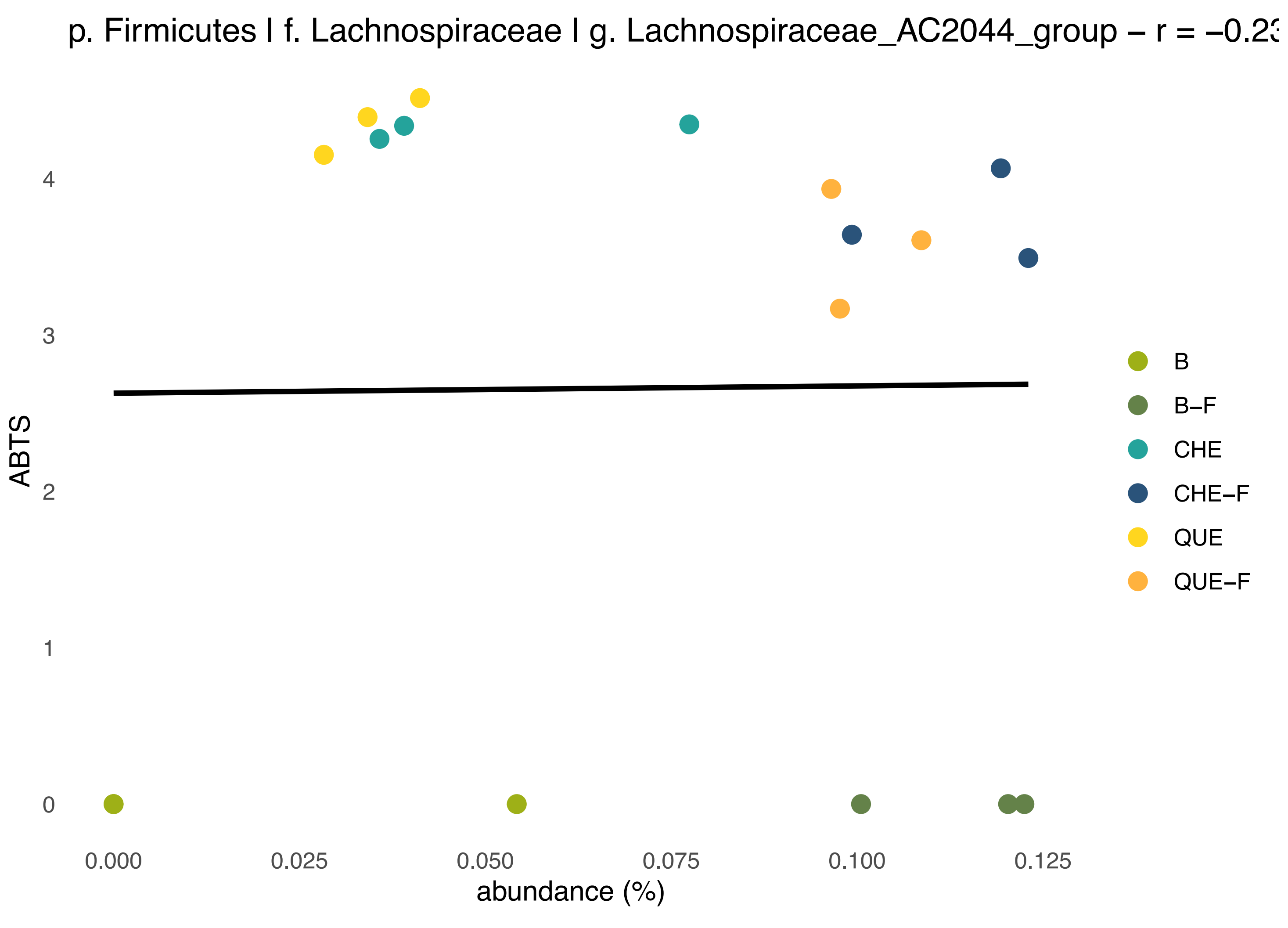

p. Firmicutes l f. Lachnospiraceae l g. Lachnospiraceae_AC2044_group – r = –0.23
ABTS
0
1
2
3
4
0.000
0.025
0.050
0.075
0.100
0.125
abundance (%)
B
B–F
CHE
CHE–F
QUE
QUE–F

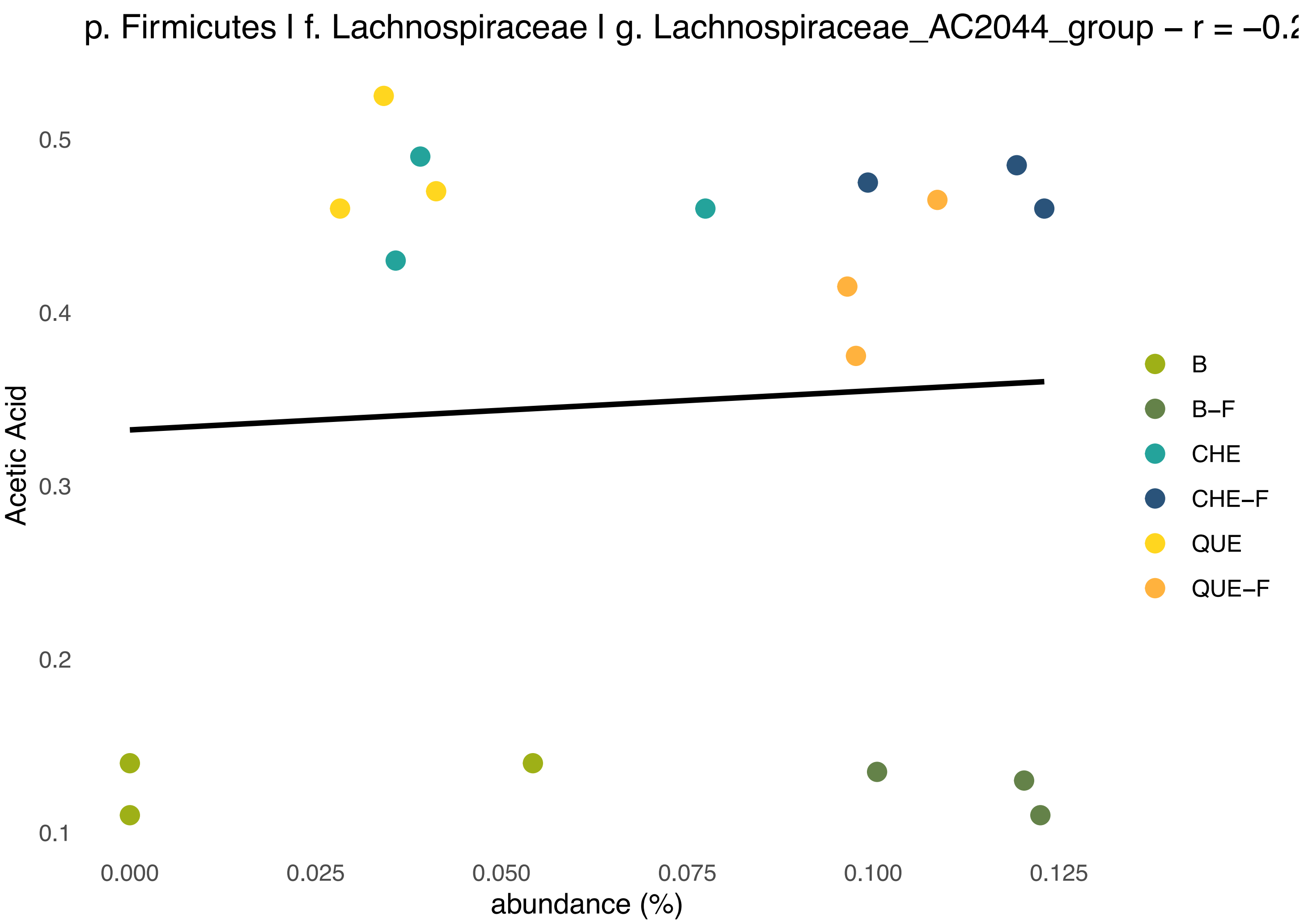
p. Firmicutes I f. Lachnospiraceae I g. Lachnospiraceae_AC2044_group – r = –0.2
Acetic Acid
0.5
0.4
0.3
0.2
0.1
0.000
0.025
0.050
0.075
0.100
0.125
abundance (%)
B
B–F
CHE
CHE–F
QUE
QUE–F

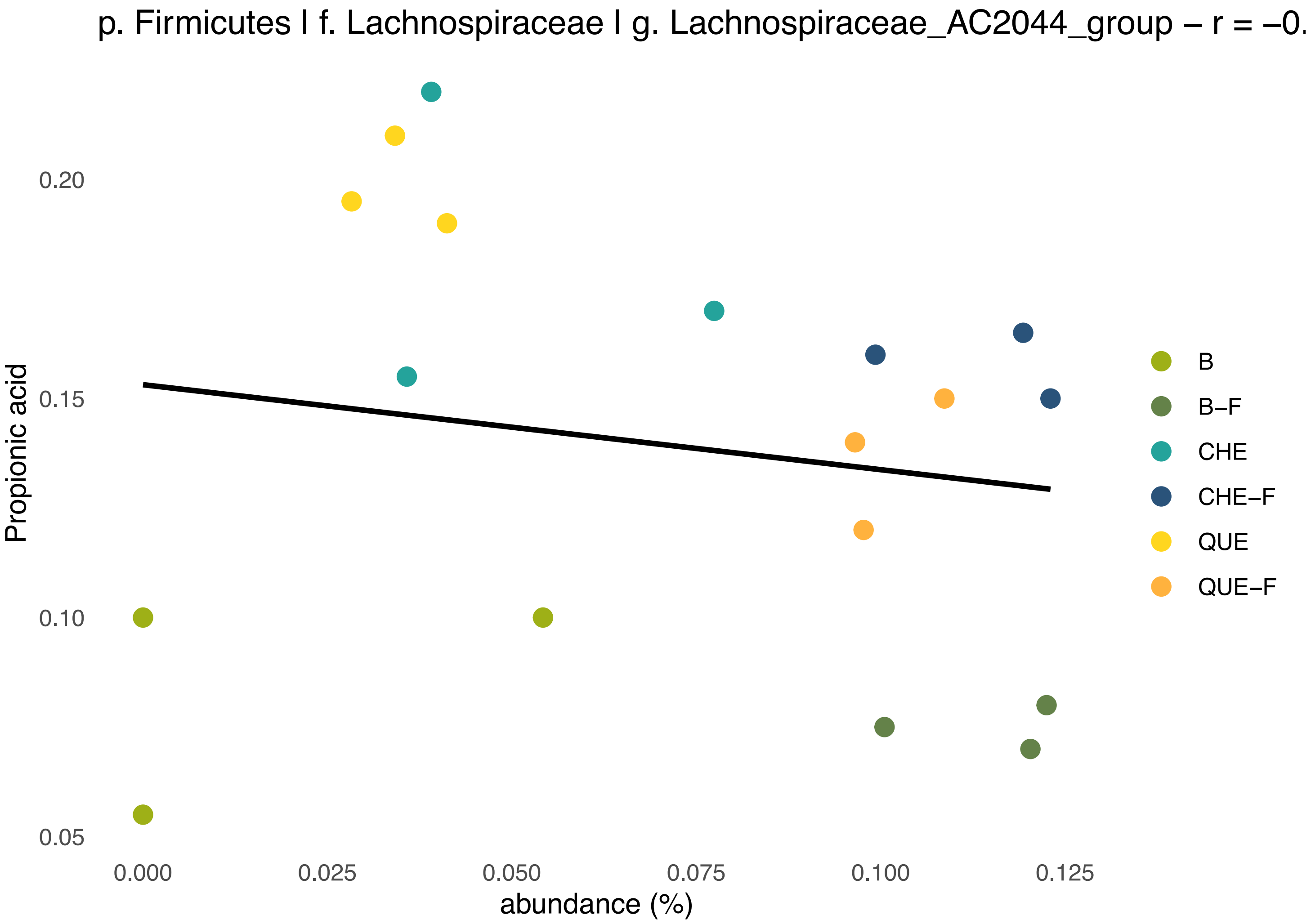
p. Firmicutes I f. Lachnospiraceae I g. Lachnospiraceae_AC2044_group – r = –0.
Propionic acid
0.20
0.15
0.10
0.05
0.000
0.025
0.050
0.075
0.100
0.125
abundance (%)
B
B–F
CHE
CHE–F
QUE
QUE–F

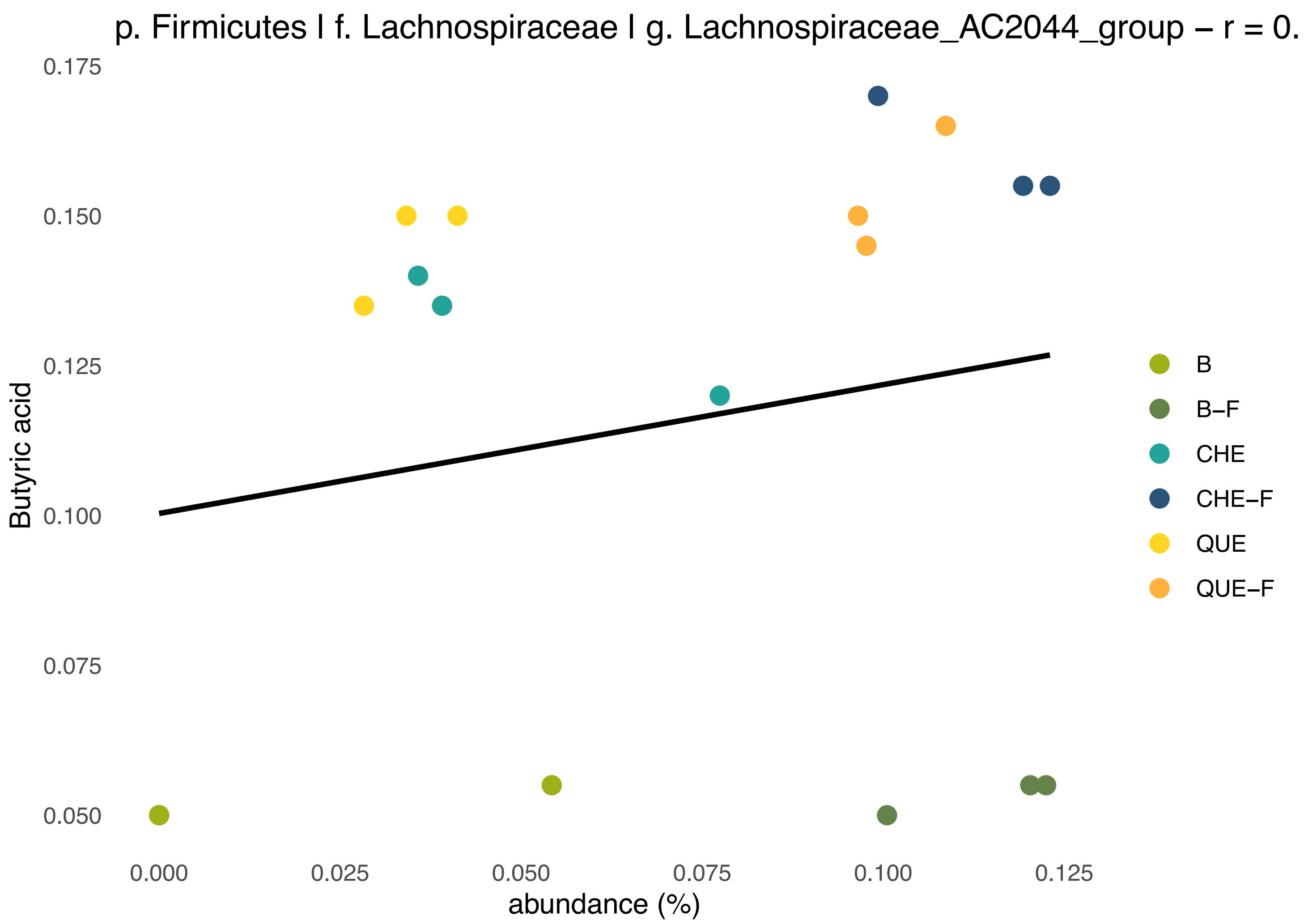
p. Firmicutes I f. Lachnospiraceae I g. Lachnospiraceae_AC2044_group – r = 0.
Butyric acid
0.175
0.150
0.125
0.100
0.075
0.050
0.000
0.025
0.050
0.075
0.100
0.125
abundance (%)
B
B–F
CHE
CHE–F
QUE
QUE–F

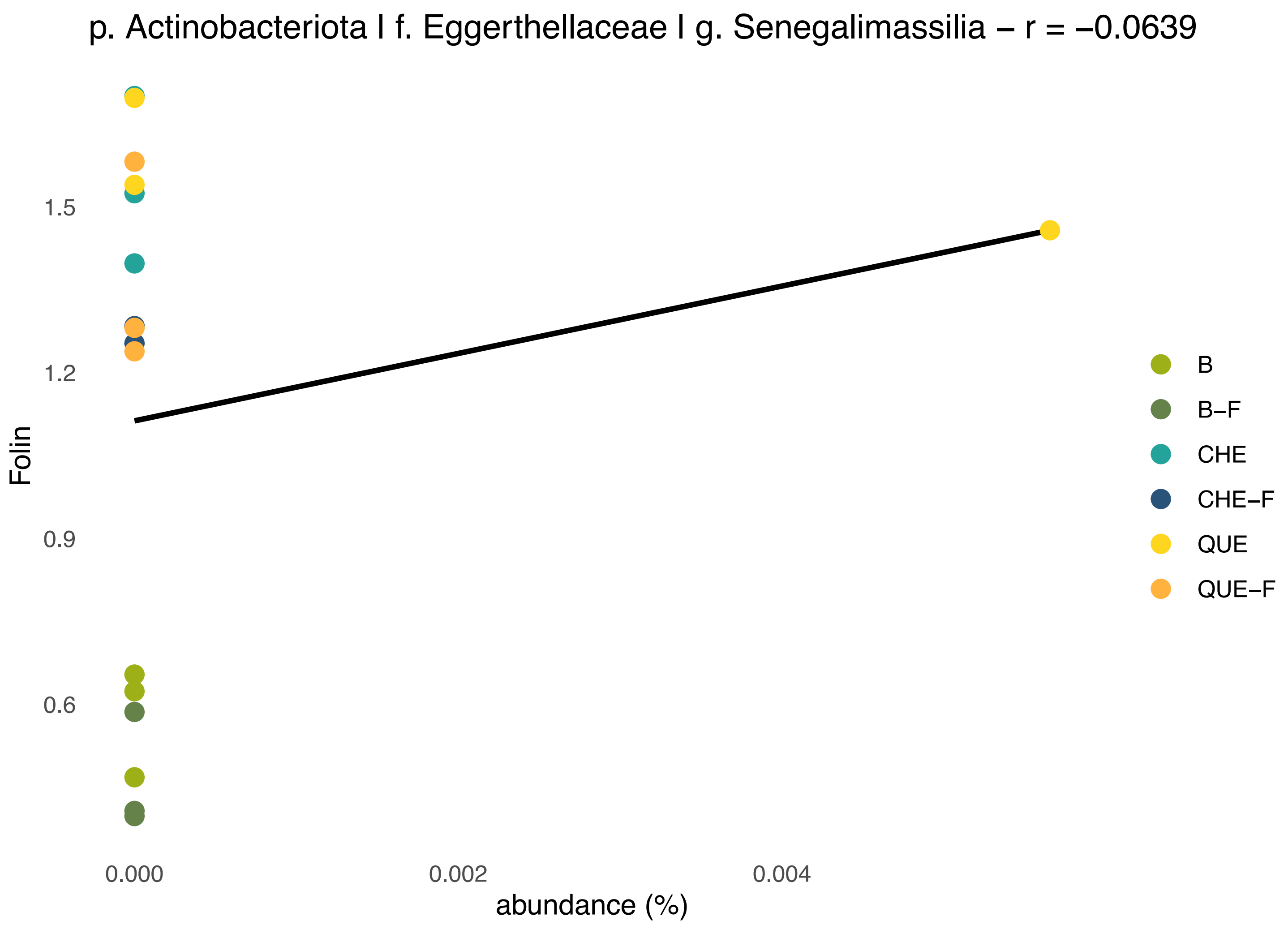

p. Actinobacteriota | f. Eggerthellaceae | g. Senegalimassilia – r = −0.0639
1.5
1.2
0.9
0.6
Folin
0.000
0.002
0.004
abundance (%)
B
B−F
CHE
CHE−F
QUE
QUE−F

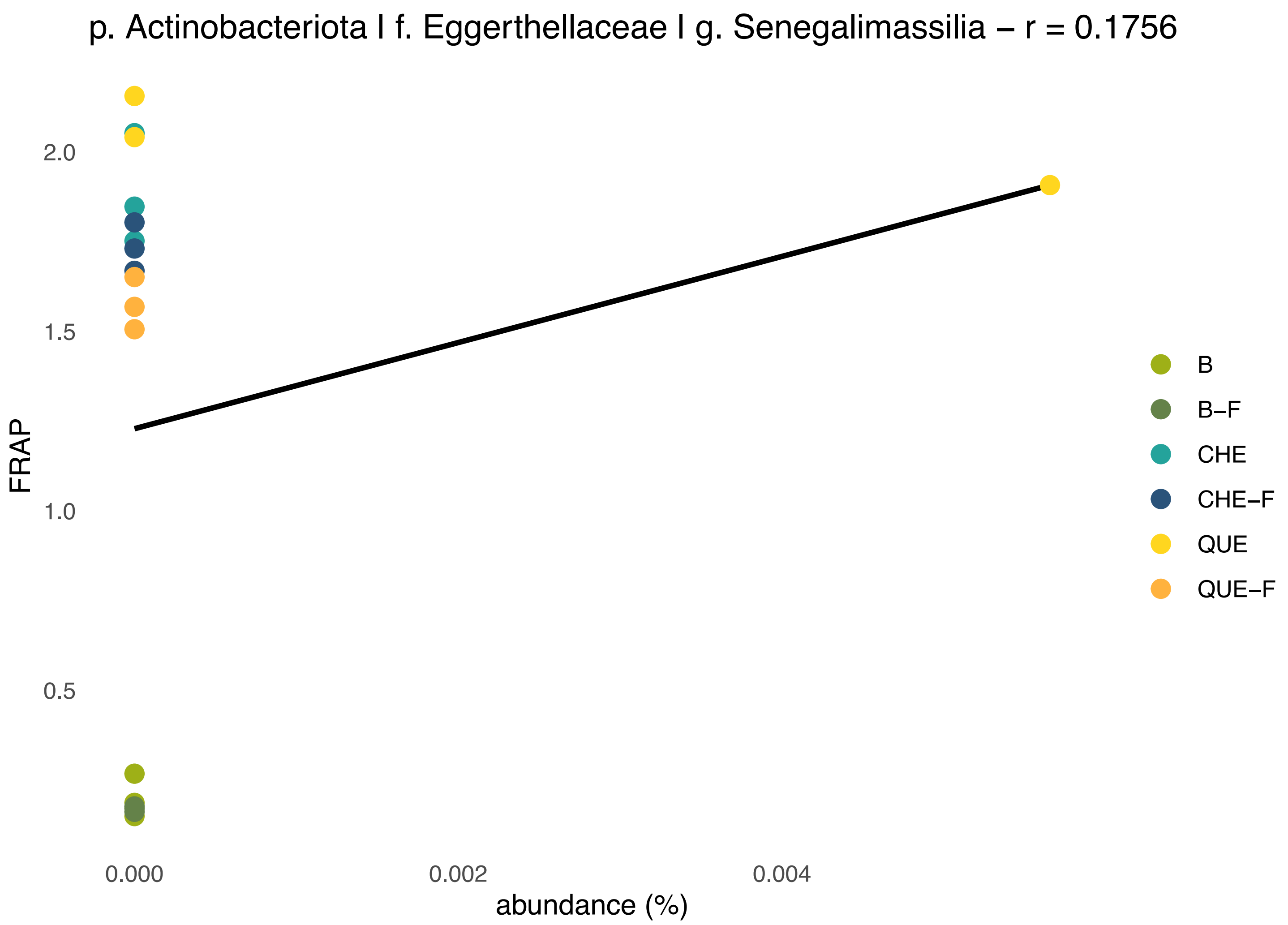
p. Actinobacteriota I f. Eggerthellaceae I g. Senegalimassilia – r = 0.1756
FRAP
2.0
1.5
1.0
0.5
0.000
0.002
0.004
abundance (%)
B
B–F
CHE
CHE–F
QUE
QUE–F

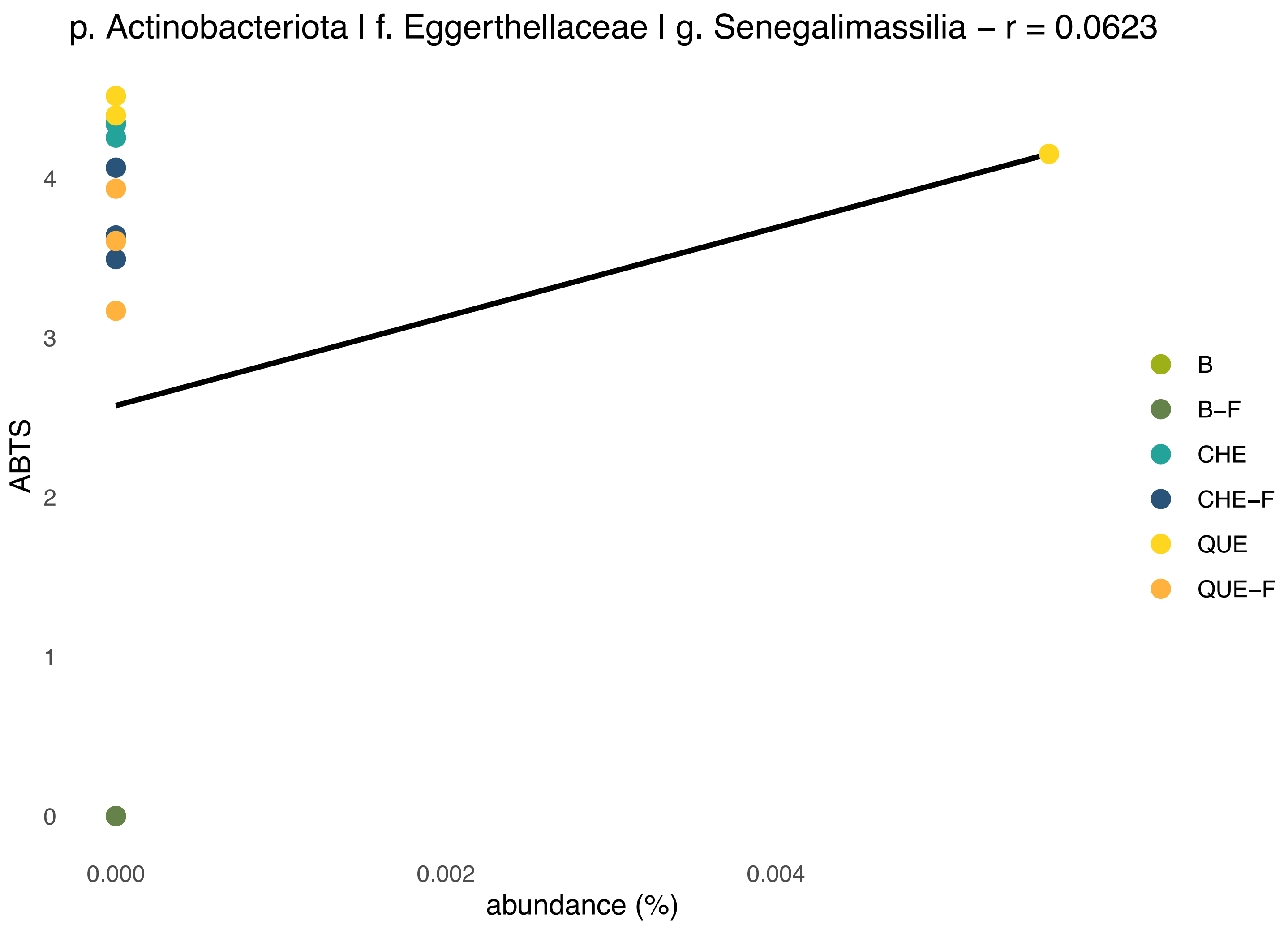

p. Actinobacteriota l f. Eggerthellaceae l g. Senegalimassilia – r = 0.0623
ABTS
0
1
2
3
4
0.000
0.002
0.004
abundance (%)
B
B–F
CHE
CHE–F
QUE
QUE–F

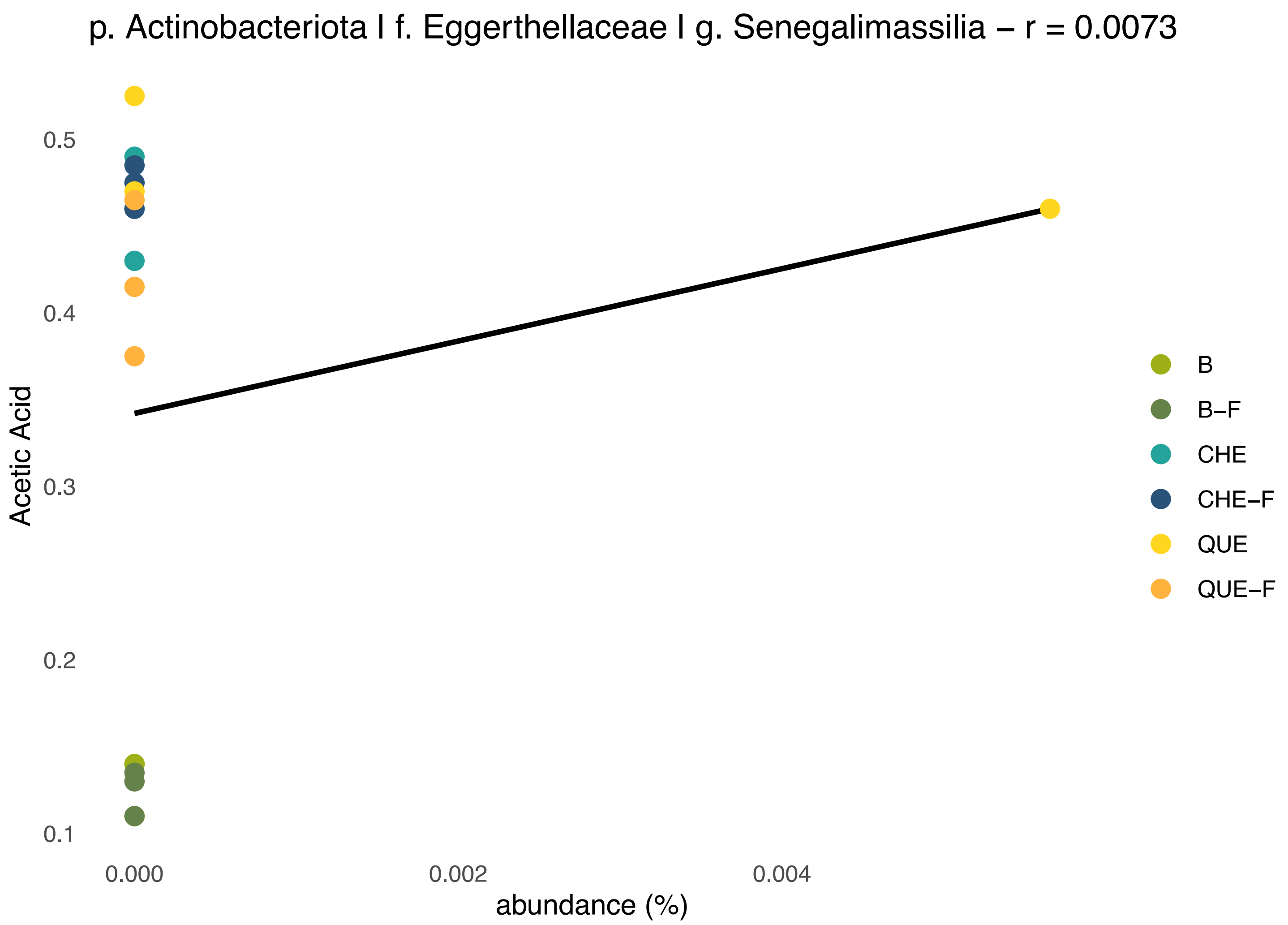

p. Actinobacteriota I f. Eggerthellaceae I g. Senegalimassilia – r = 0.0073
Acetic Acid
0.5
0.4
0.3
0.2
0.1
0.000
0.002
0.004
abundance (%)
B
B–F
CHE
CHE–F
QUE
QUE–F

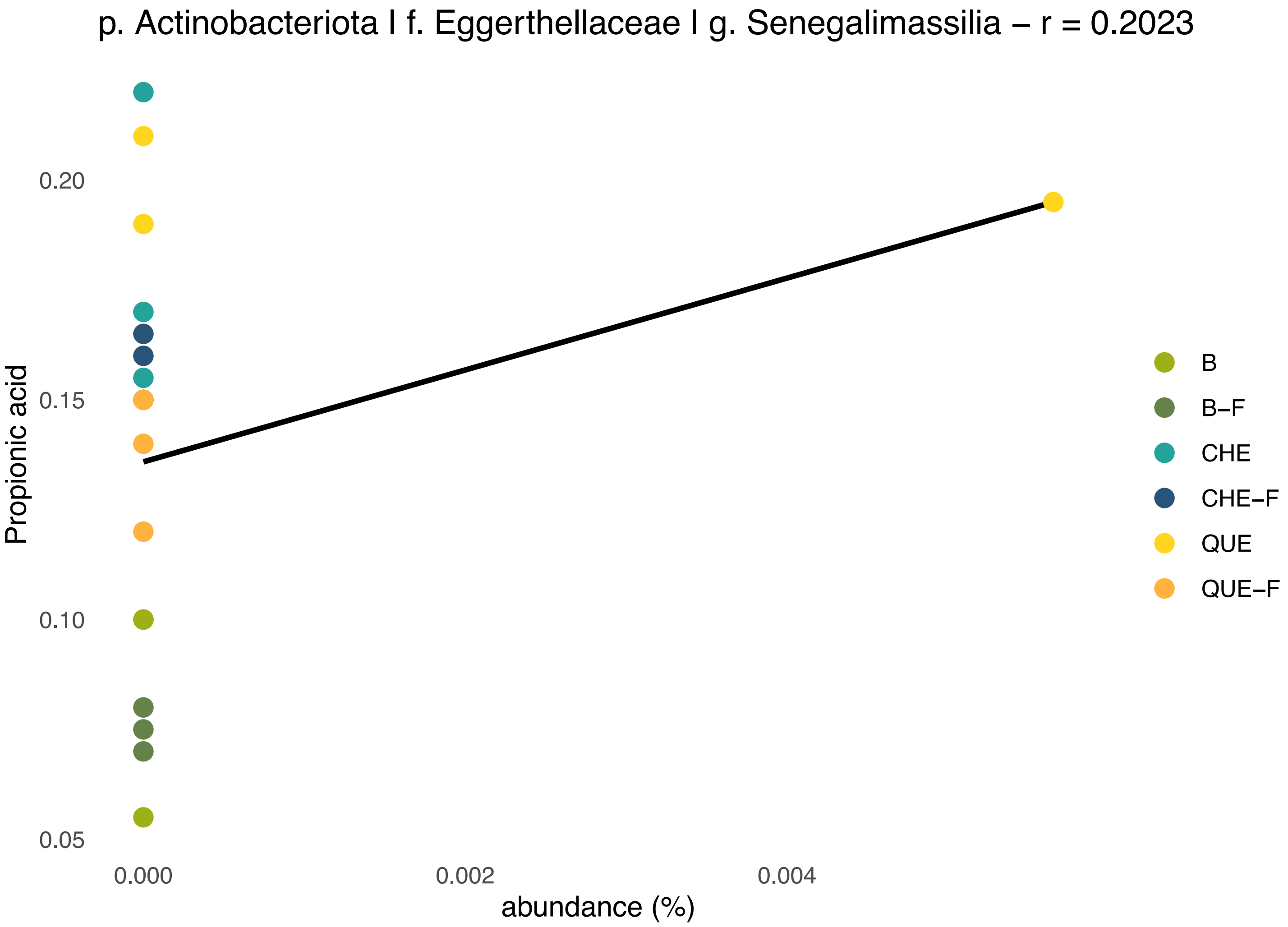

p. Actinobacteriota I f. Eggerthellaceae I g. Senegalimassilia – r = 0.2023
Propionic acid
0.20
0.15
0.10
0.05
0.000
0.002
0.004
abundance (%)
B
B–F
CHE
CHE–F
QUE
QUE–F

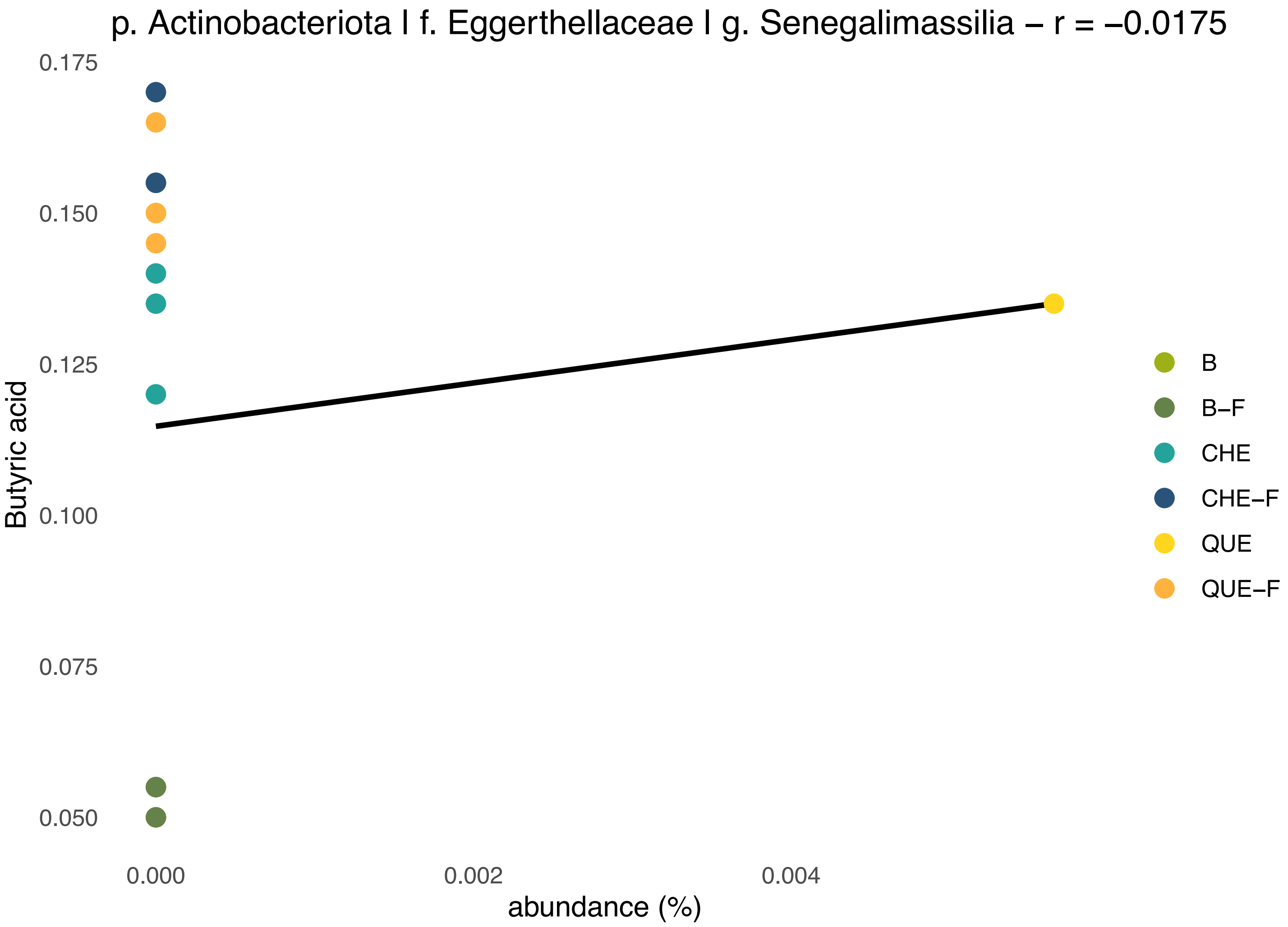

p. Actinobacteriota | f. Eggerthellaceae | g. Senegalimassilia – r = −0.0175
Butyric acid
0.175
0.150
0.125
0.100
0.075
0.050
0.000
0.002
0.004
abundance (%)
B
B−F
CHE
CHE−F
QUE
QUE−F

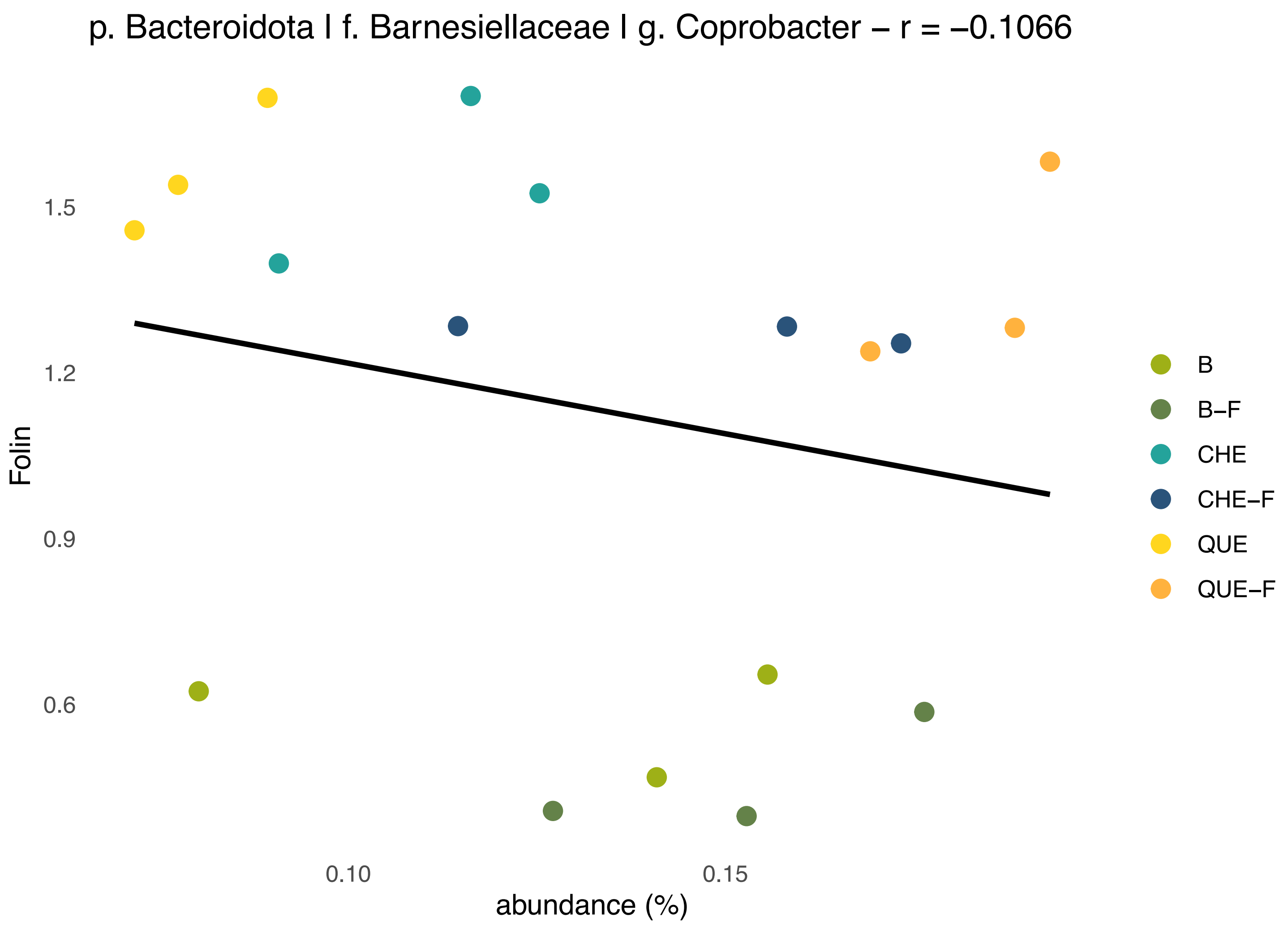
p. Bacteroidota | f. Barnesiellaceae | g. Coprobacter – r = –0.1066
1.5
1.2
0.9
0.6
Folin
0.10
0.15
abundance (%)
B
B–F
CHE
CHE–F
QUE
QUE–F

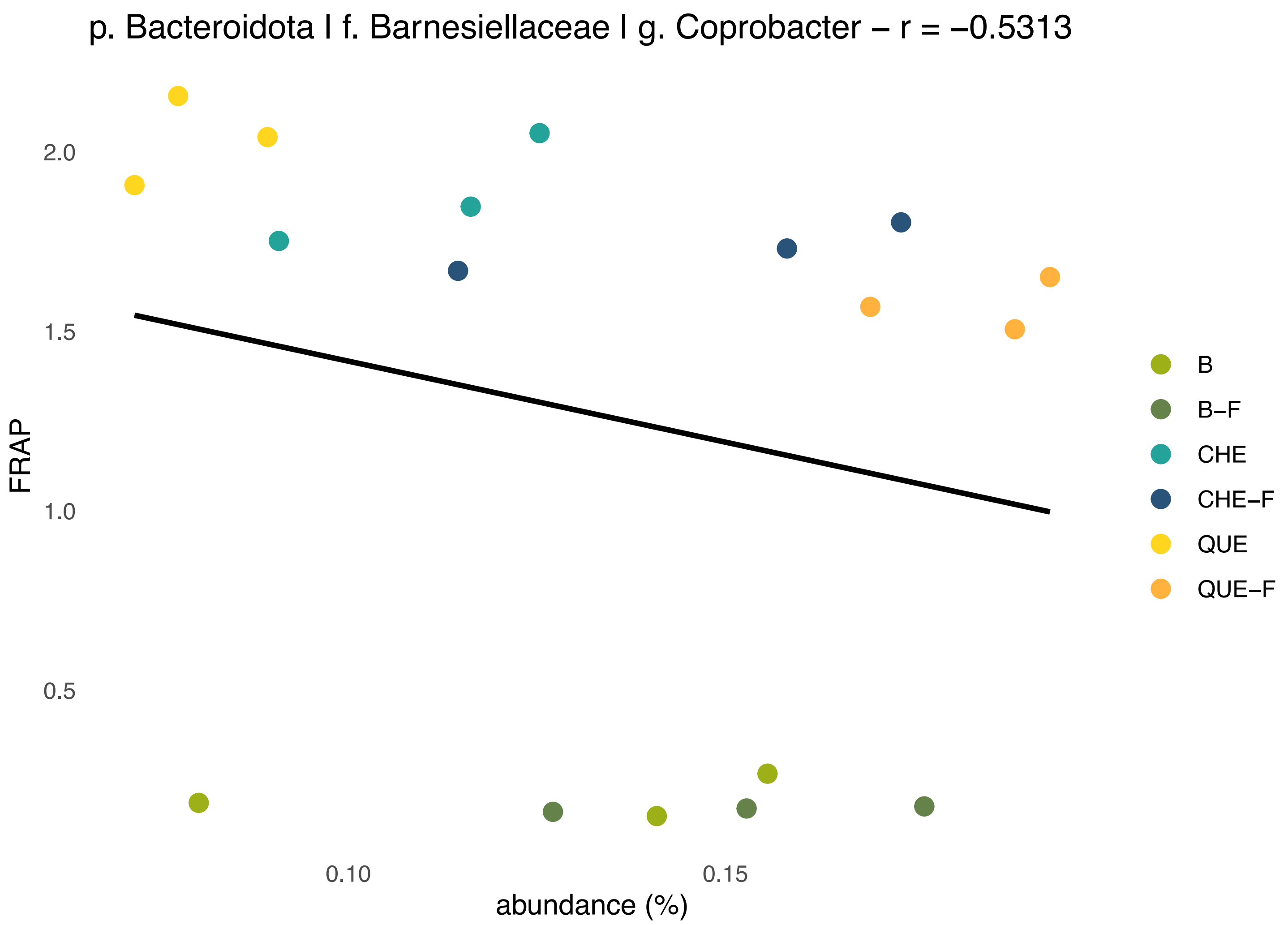

p. Bacteroidota l f. Barnesiellaceae l g. Coprobacter – r = –0.5313
2.0
1.5
1.0
0.5
FRAP
0.10
0.15
abundance (%)
B
B–F
CHE
CHE–F
QUE
QUE–F

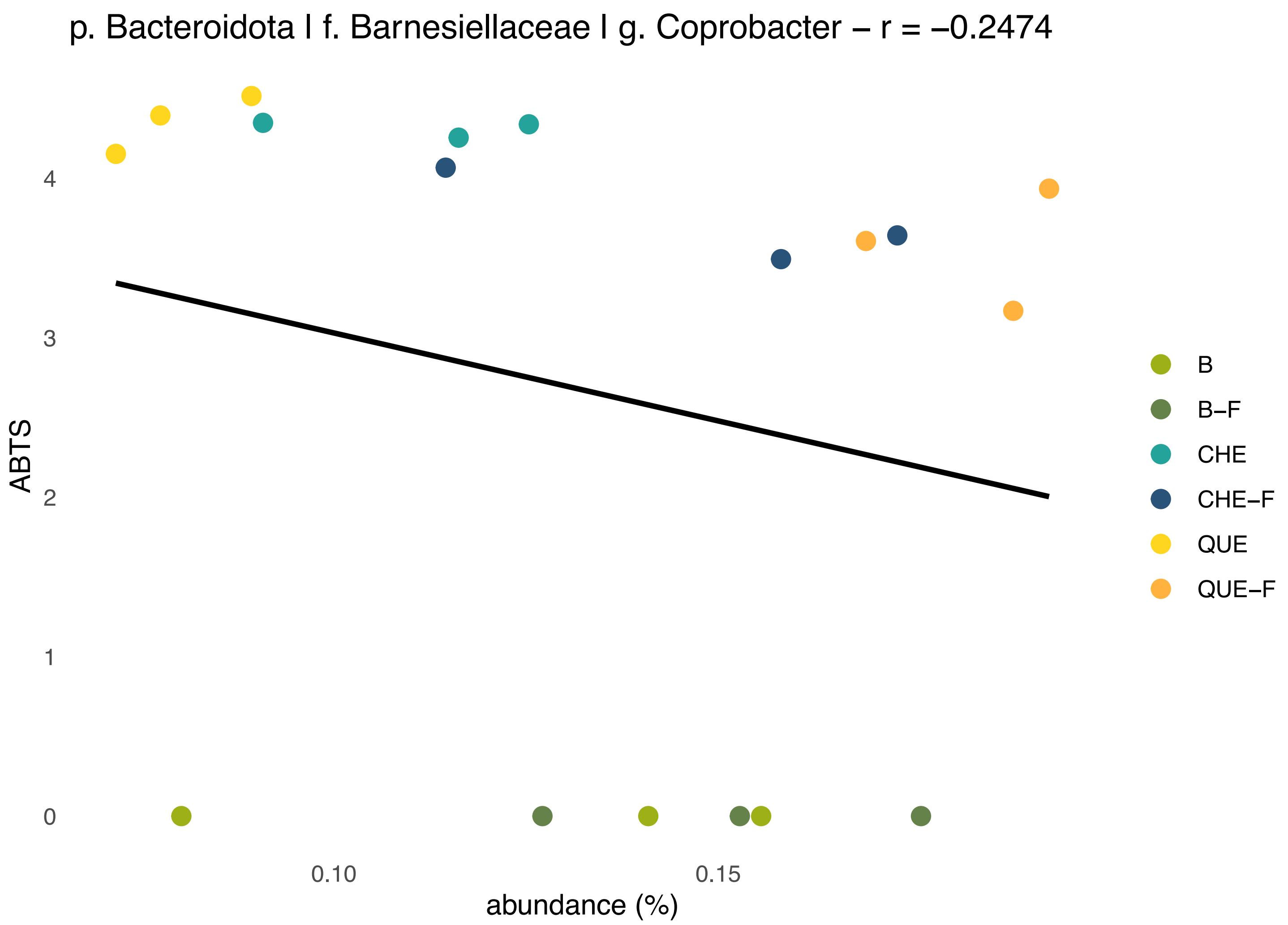
p. Bacteroidota I f. Barnesiellaceae I g. Coprobacter − r = −0.2474
ABTS
4
3
2
1
0
0.10
0.15
abundance (%)
B
B−F
CHE
CHE−F
QUE
QUE−F

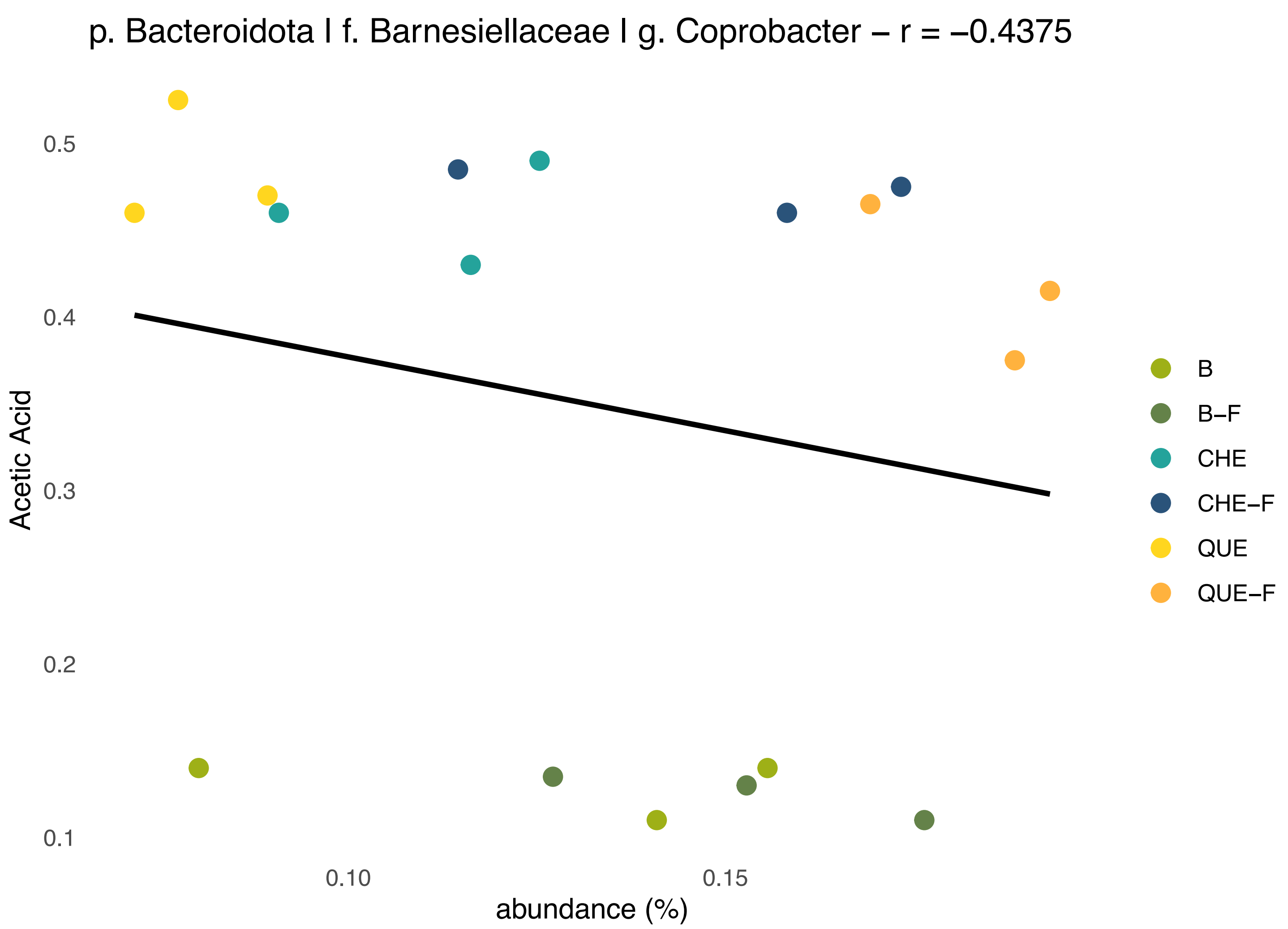
p. Bacteroidota l f. Barnesiellaceae l g. Coprobacter – r = –0.4375
Acetic Acid
0.5
0.4
0.3
0.2
0.1
abundance (%)
0.10
0.15
B
B–F
CHE
CHE–F
QUE
QUE–F

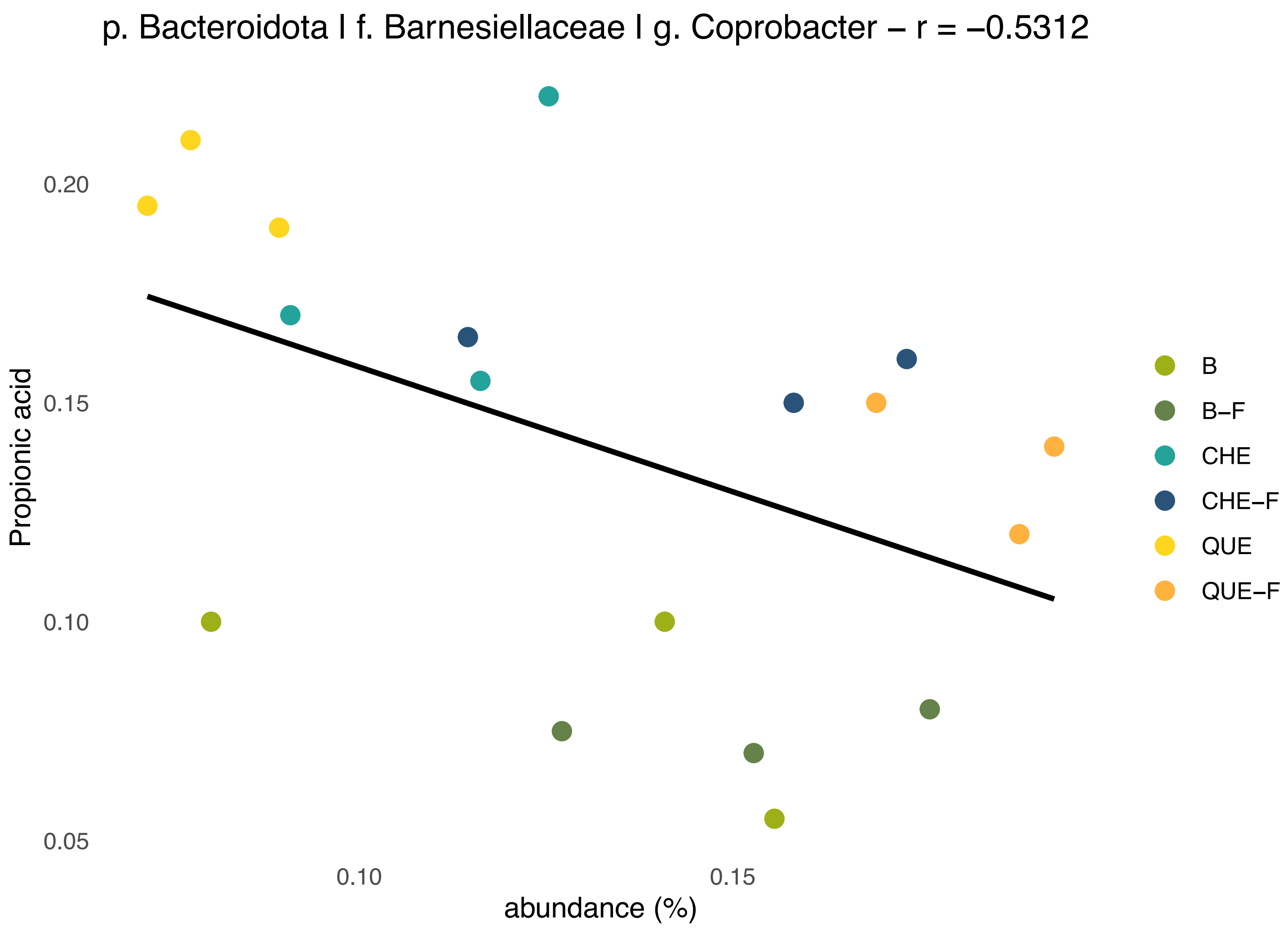
p. Bacteroidota I f. Barnesiellaceae I g. Coprobacter – r = –0.5312
Propionic acid
0.20
0.15
0.10
0.05
0.10
0.15
abundance (%)
B
B–F
CHE
CHE–F
QUE
QUE–F

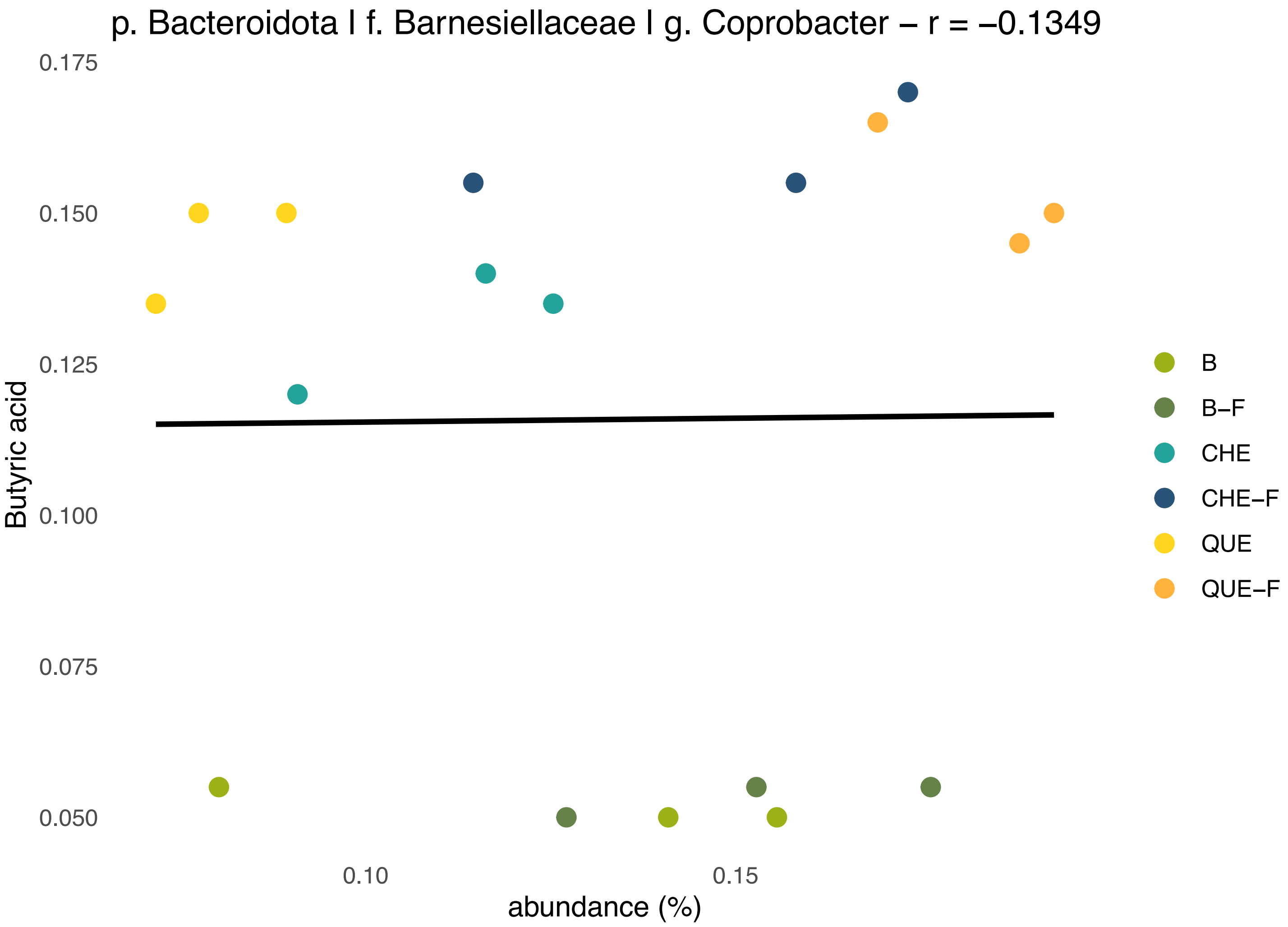
p. Bacteroidota I f. Barnesiellaceae I g. Coprobacter – r = –0.1349
0.175
0.150
0.125
0.100
0.075
0.050
Butyric acid
0.10
0.15
abundance (%)
B
B–F
CHE
CHE–F
QUE
QUE–F

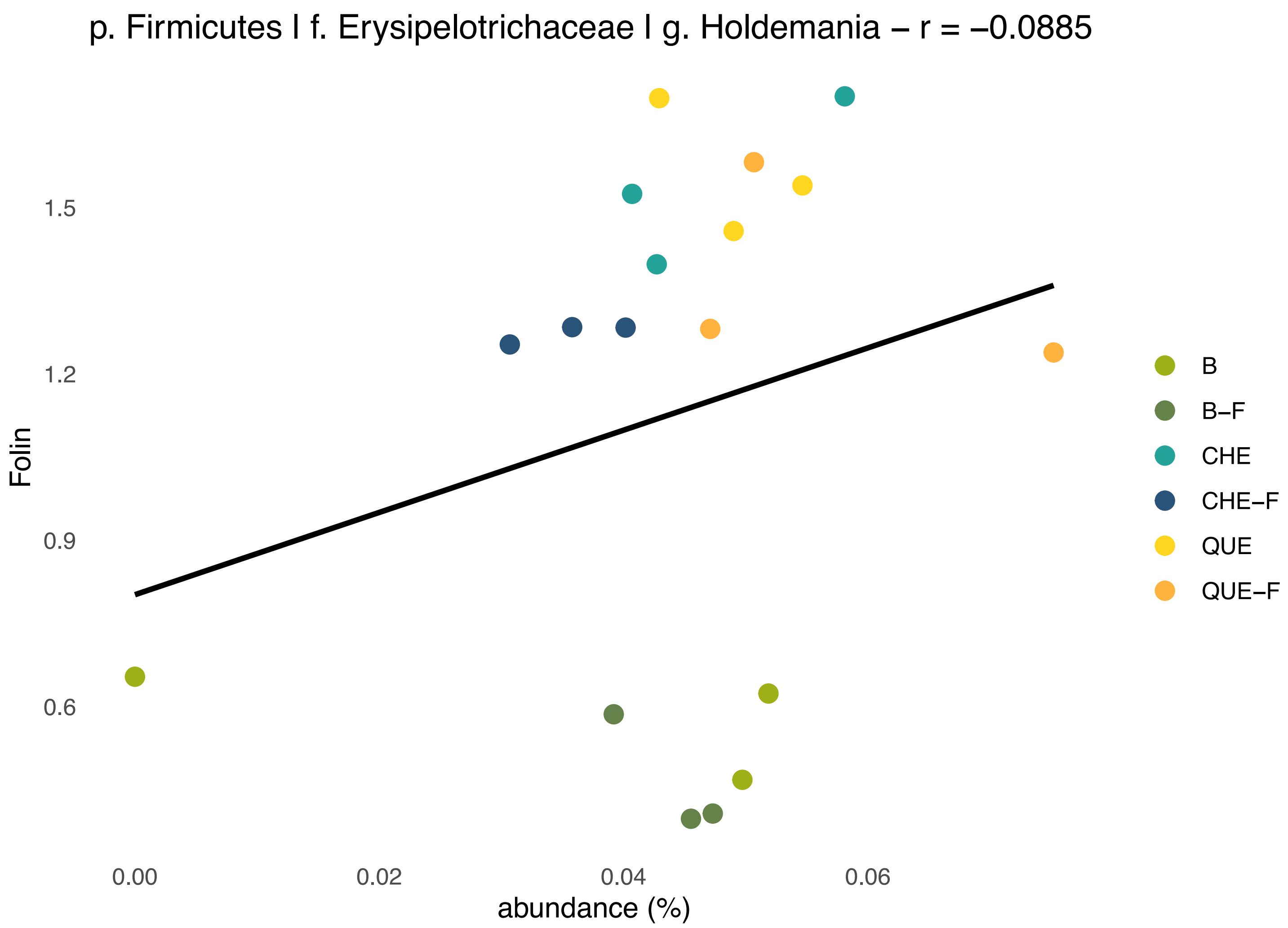

p. Firmicutes I f. Erysipelotrichaceae I g. Holdemania – r = –0.0885
Folin
1.5
1.2
0.9
0.6
0.00
0.02
0.04
0.06
abundance (%)
B
B–F
CHE
CHE–F
QUE
QUE–F

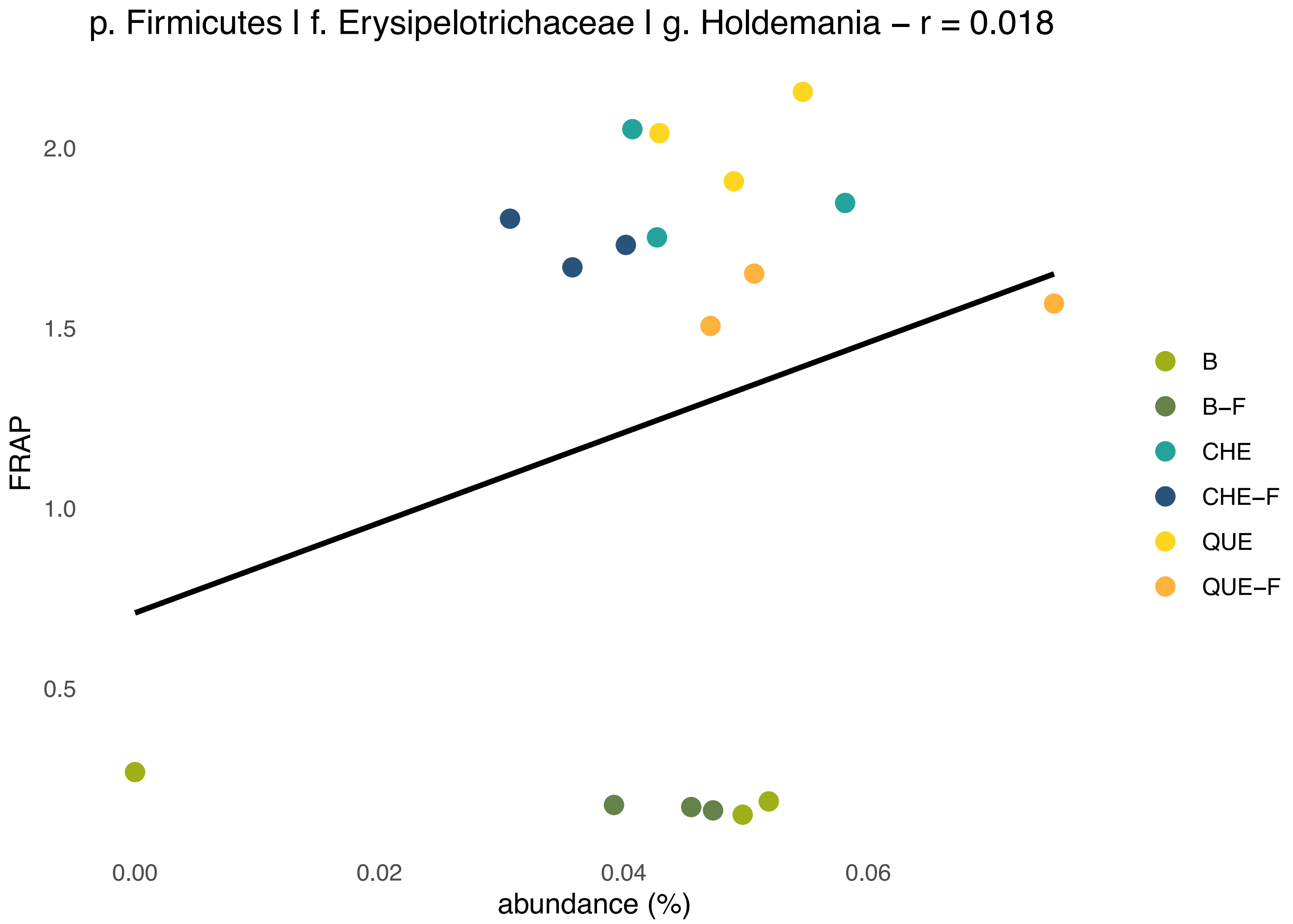

p. Firmicutes I f. Erysipelotrichaceae I g. Holdemania − r = 0.018
FRAP
2.0
1.5
1.0
0.5
0.00
0.02
0.04
0.06
abundance (%)
B
B−F
CHE
CHE−F
QUE
QUE−F

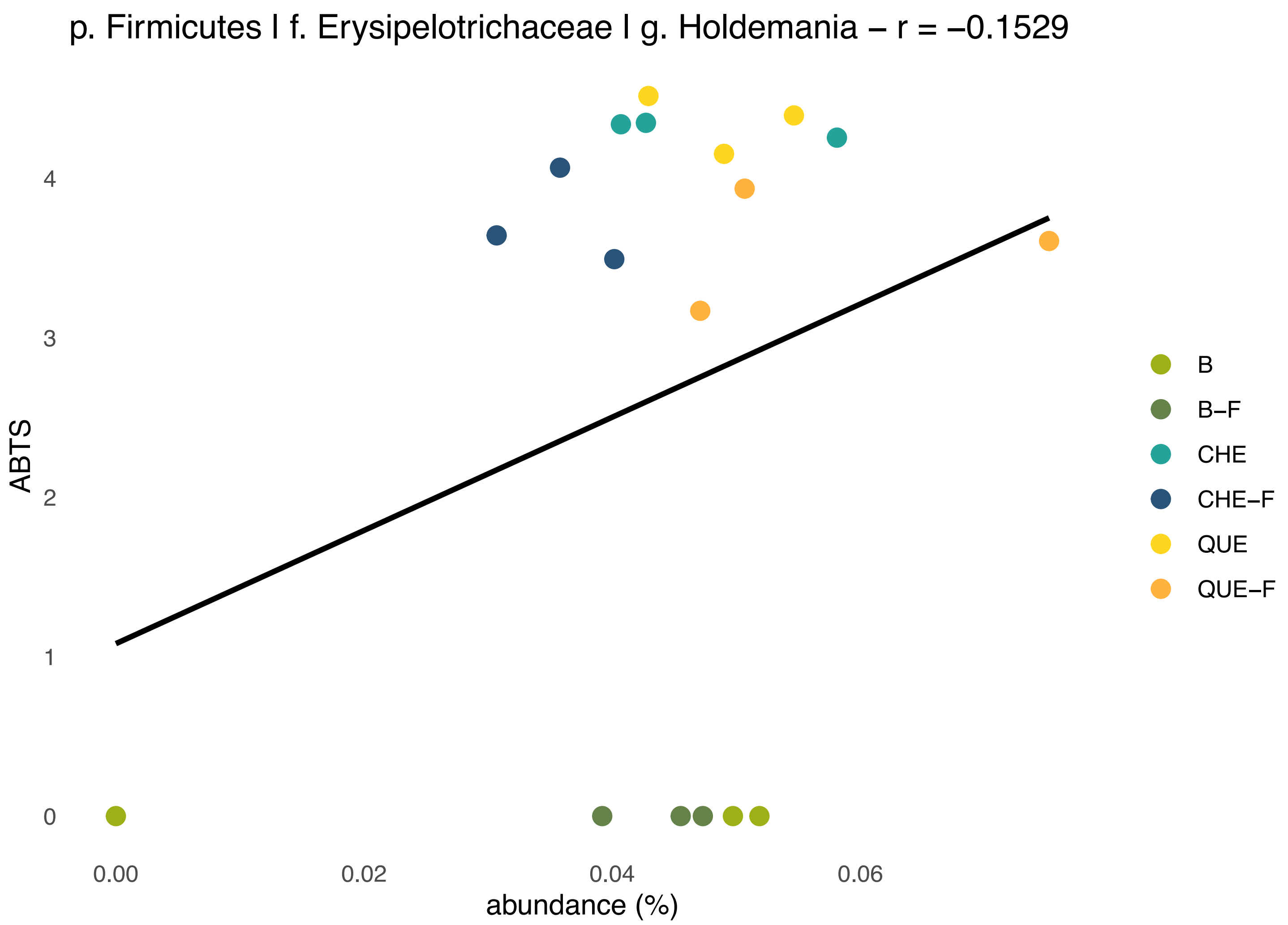

p. Firmicutes l f. Erysipelotrichaceae l g. Holdemania – r = –0.1529
ABTS
4
3
2
1
0
0.00
0.02
0.04
0.06
abundance (%)
B
B–F
CHE
CHE–F
QUE
QUE–F

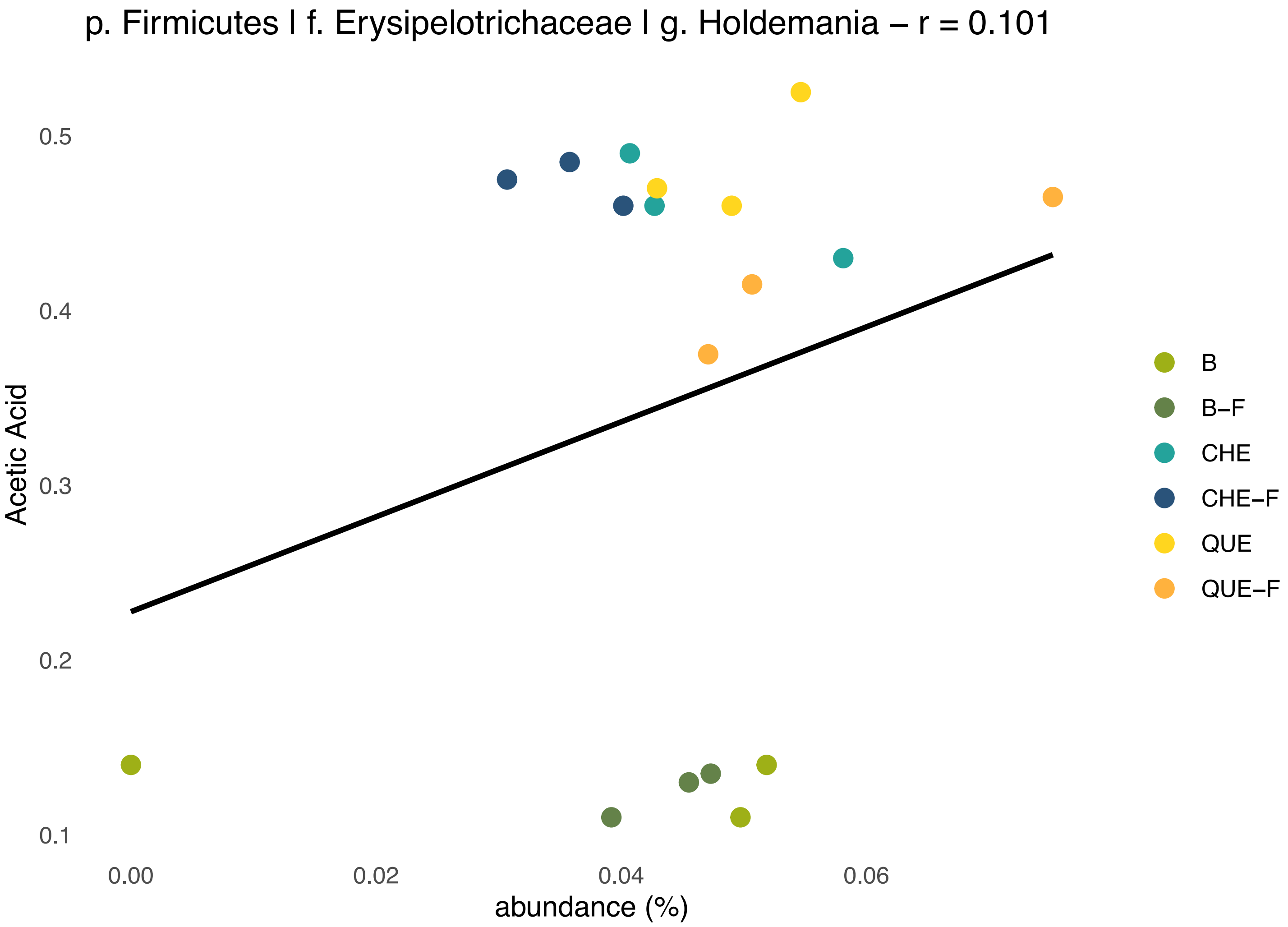
p. Firmicutes l f. Erysipelotrichaceae l g. Holdemania – r = 0.101
Acetic Acid
0.5
0.4
0.3
0.2
0.1
0.00
0.02
0.04
0.06
abundance (%)
B
B–F
CHE
CHE–F
QUE
QUE–F

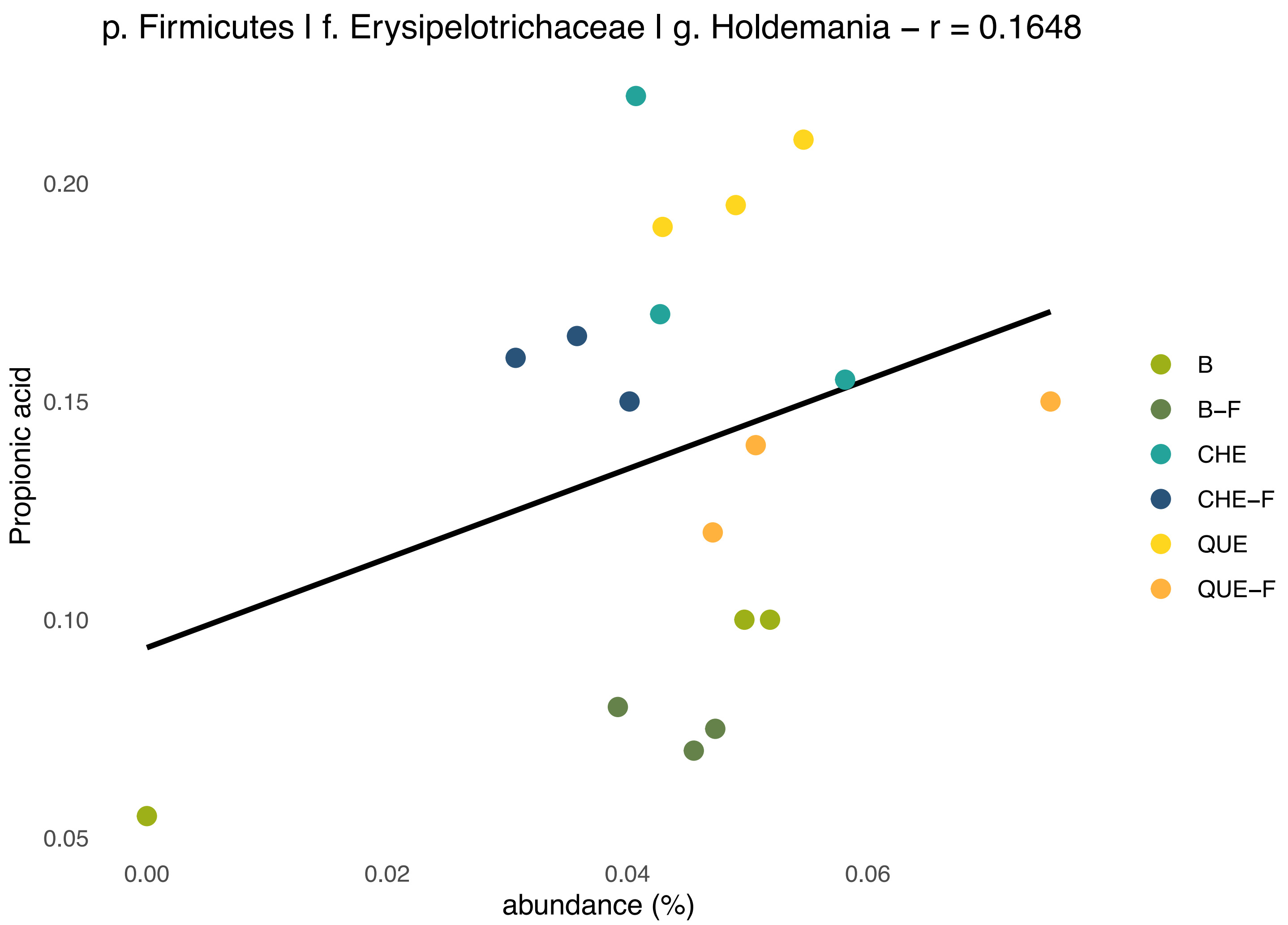

p. Firmicutes l f. Erysipelotrichaceae l g. Holdemania – r = 0.1648
Propionic acid
0.20
0.15
0.10
0.05
0.00
0.02
0.04
0.06
abundance (%)
B
B–F
CHE
CHE–F
QUE
QUE–F

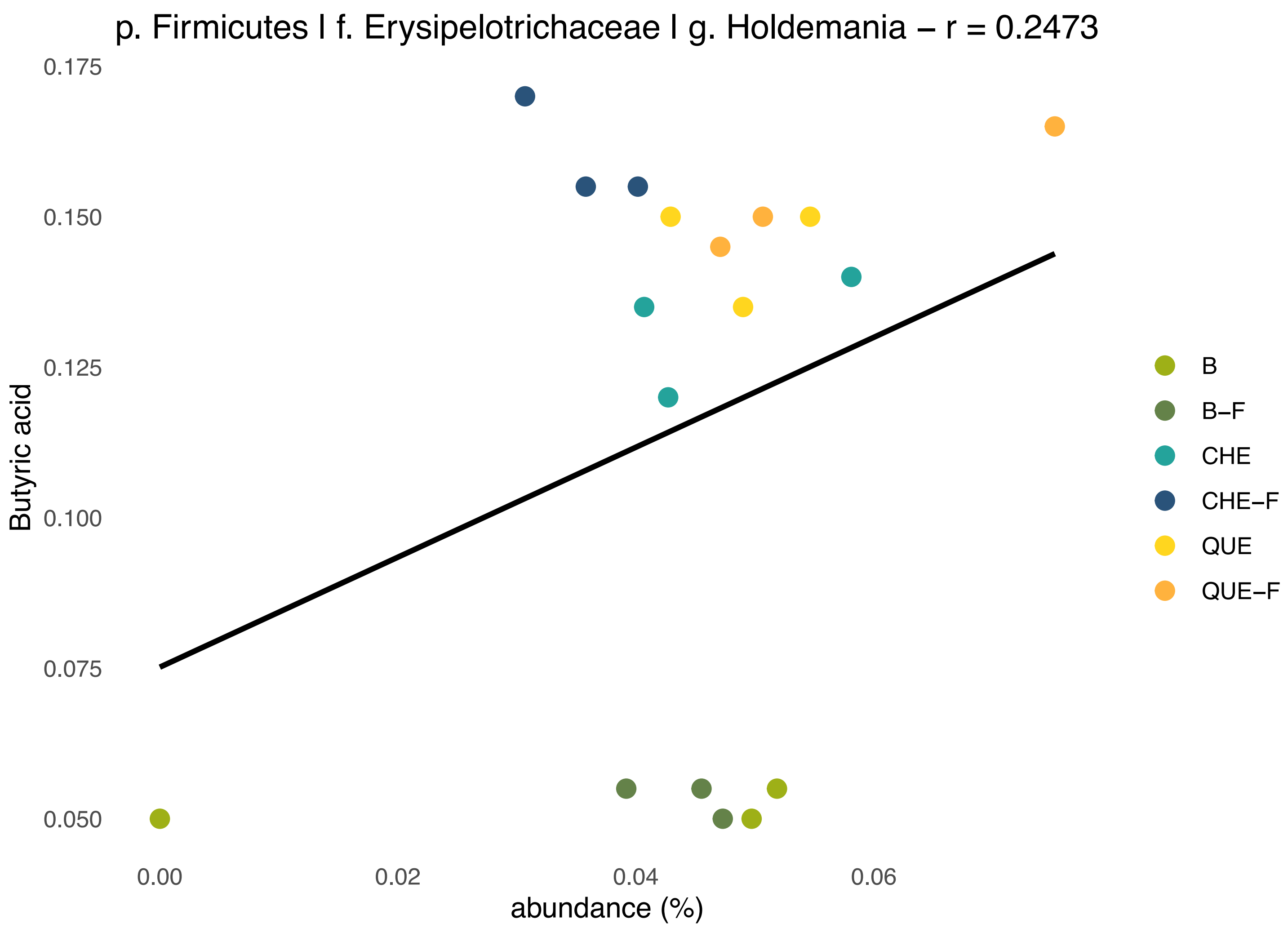

p. Firmicutes l f. Erysipelotrichaceae l g. Holdemania – r = 0.2473
Butyric acid
0.175
0.150
0.125
0.100
0.075
0.050
0.00
0.02
0.04
0.06
abundance (%)
B
B–F
CHE
CHE–F
QUE
QUE–F

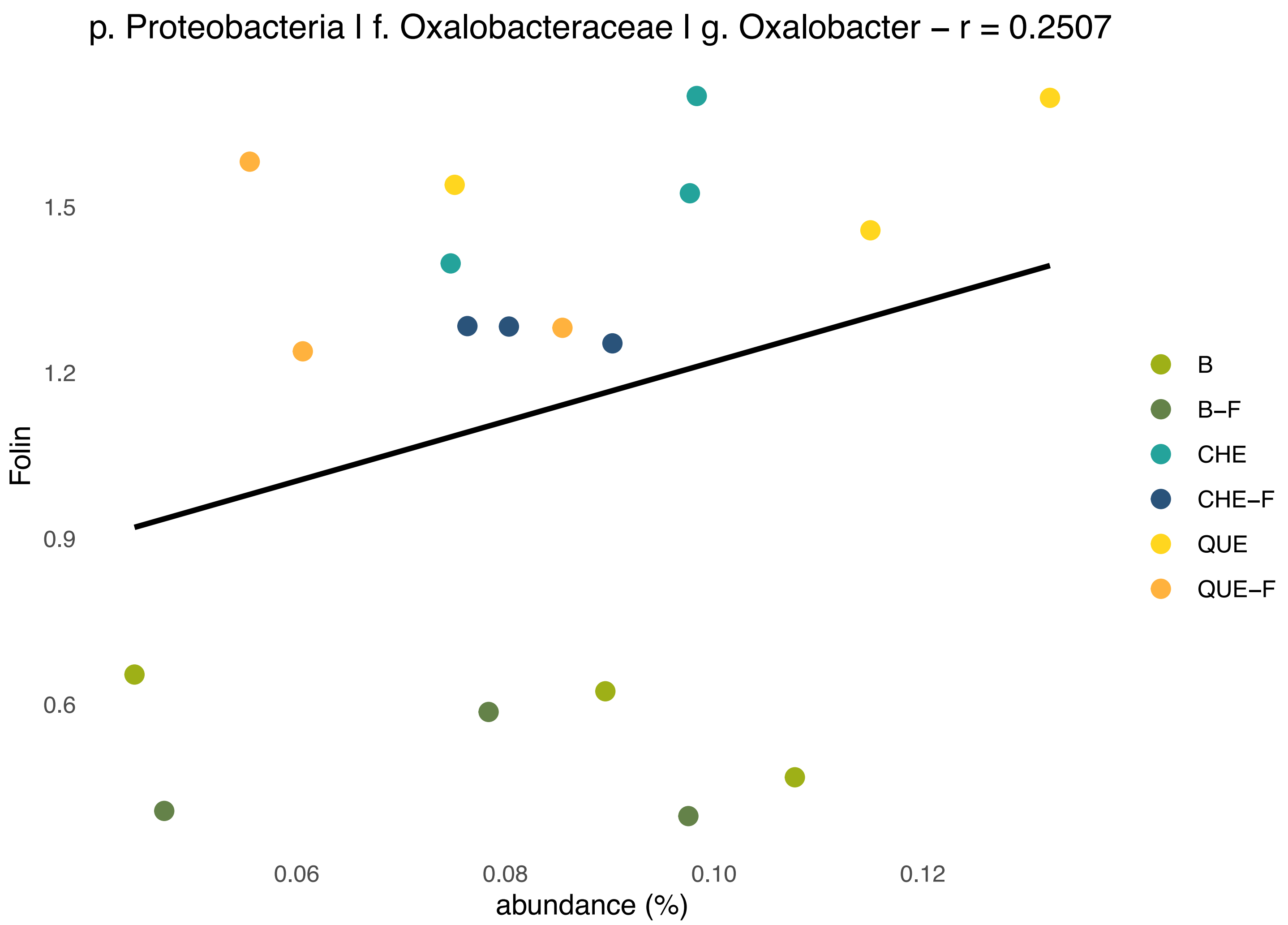

p. Proteobacteria l f. Oxalobacteraceae l g. Oxalobacter – r = 0.2507
1.5
1.2
0.9
0.6
Folin
0.06
0.08
0.10
0.12
abundance (%)
B
B–F
CHE
CHE–F
QUE
QUE–F

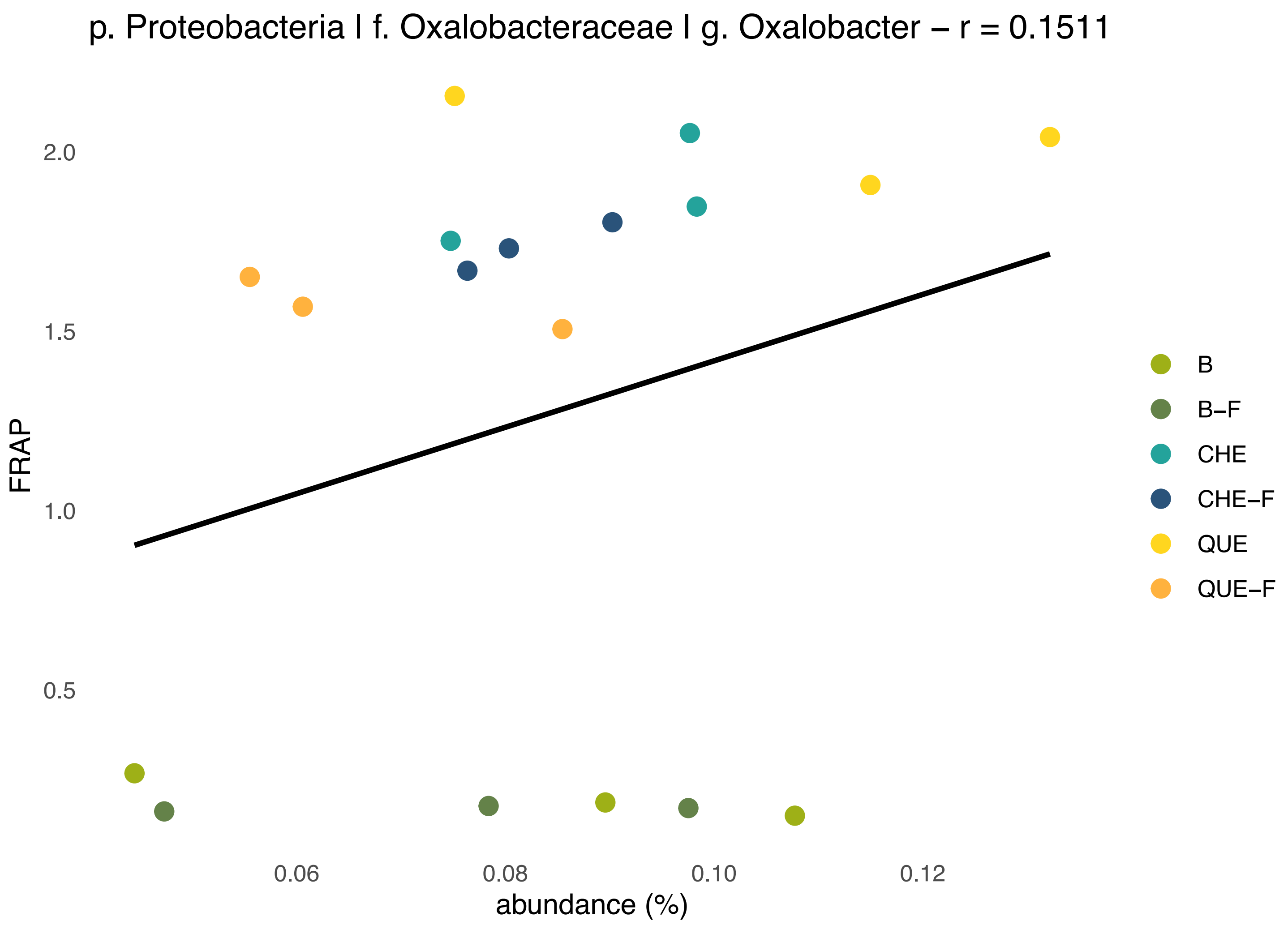
p. Proteobacteria I f. Oxalobacteraceae I g. Oxalobacter – r = 0.1511
FRAP
2.0
1.5
1.0
0.5
0.06
0.08
0.10
0.12
abundance (%)
B
B–F
CHE
CHE–F
QUE
QUE–F

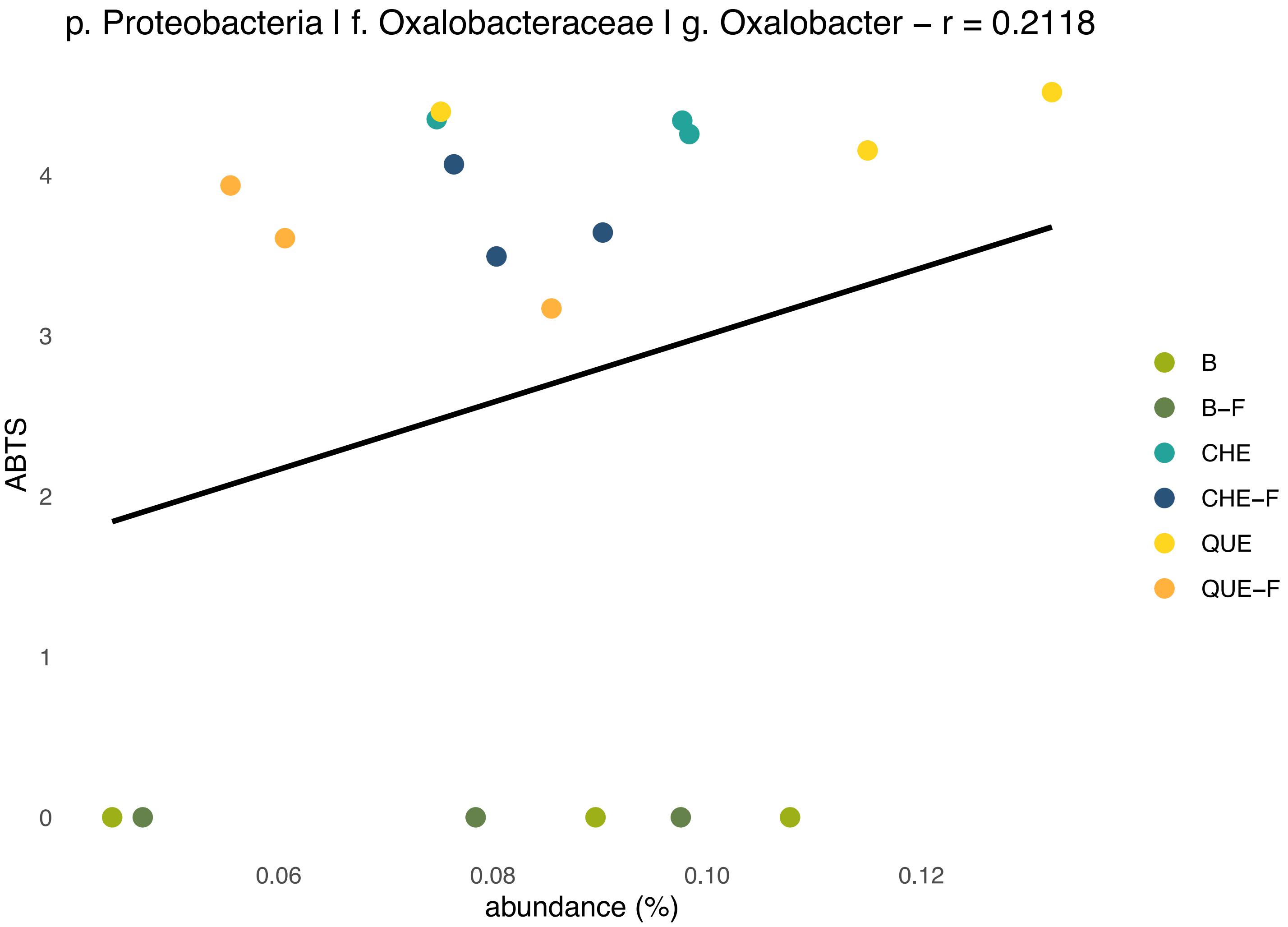

p. Proteobacteria I f. Oxalobacteraceae I g. Oxalobacter − r = 0.2118
ABTS
4
3
2
1
0
0.06
0.08
0.10
0.12
abundance (%)
B
B−F
CHE
CHE−F
QUE
QUE−F

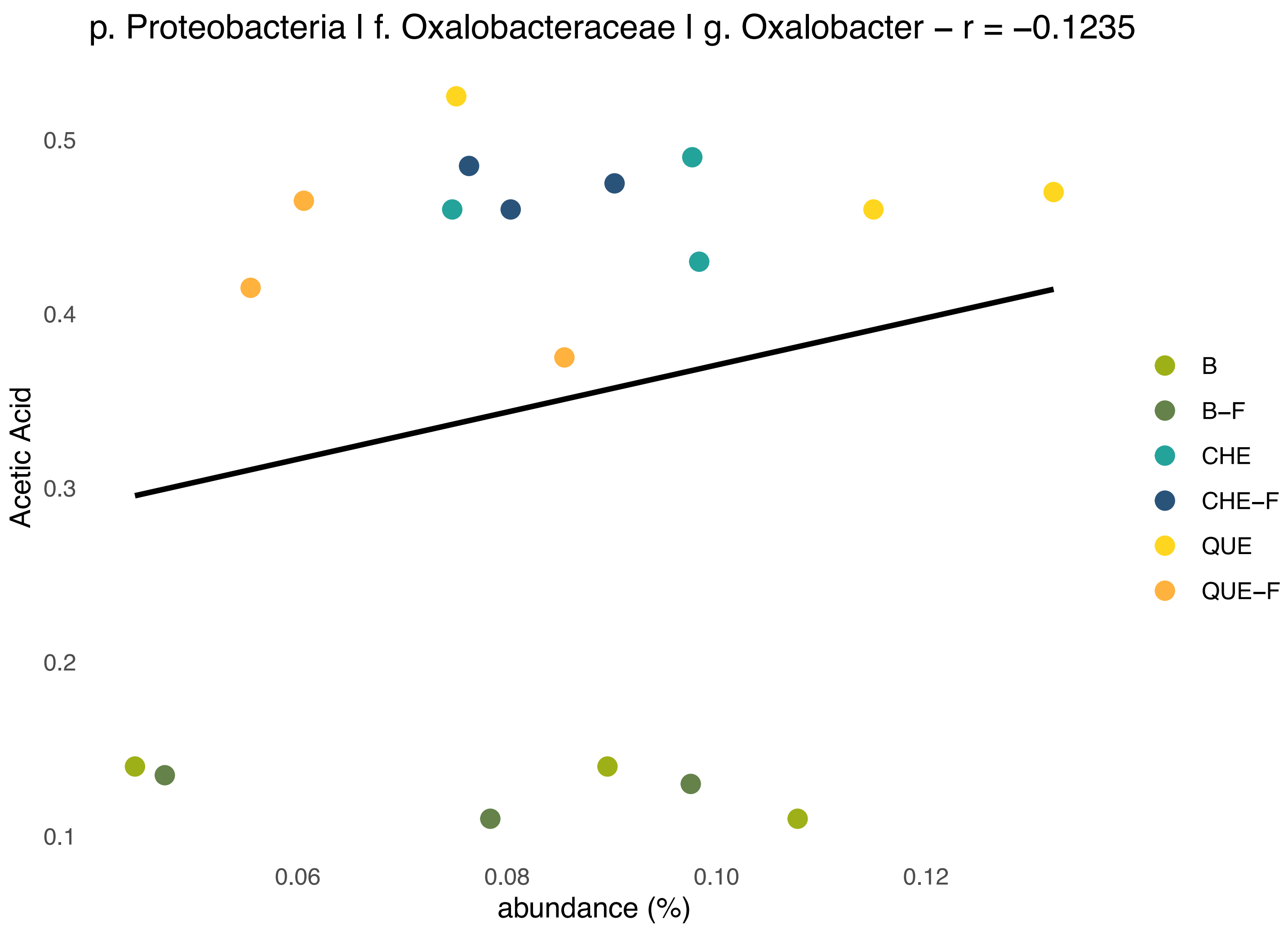
p. Proteobacteria | f. Oxalobacteraceae | g. Oxalobacter – r = –0.1235
Acetic Acid
0.5
0.4
0.3
0.2
0.1
0.06
0.08
0.10
0.12
abundance (%)
B
B–F
CHE
CHE–F
QUE
QUE–F

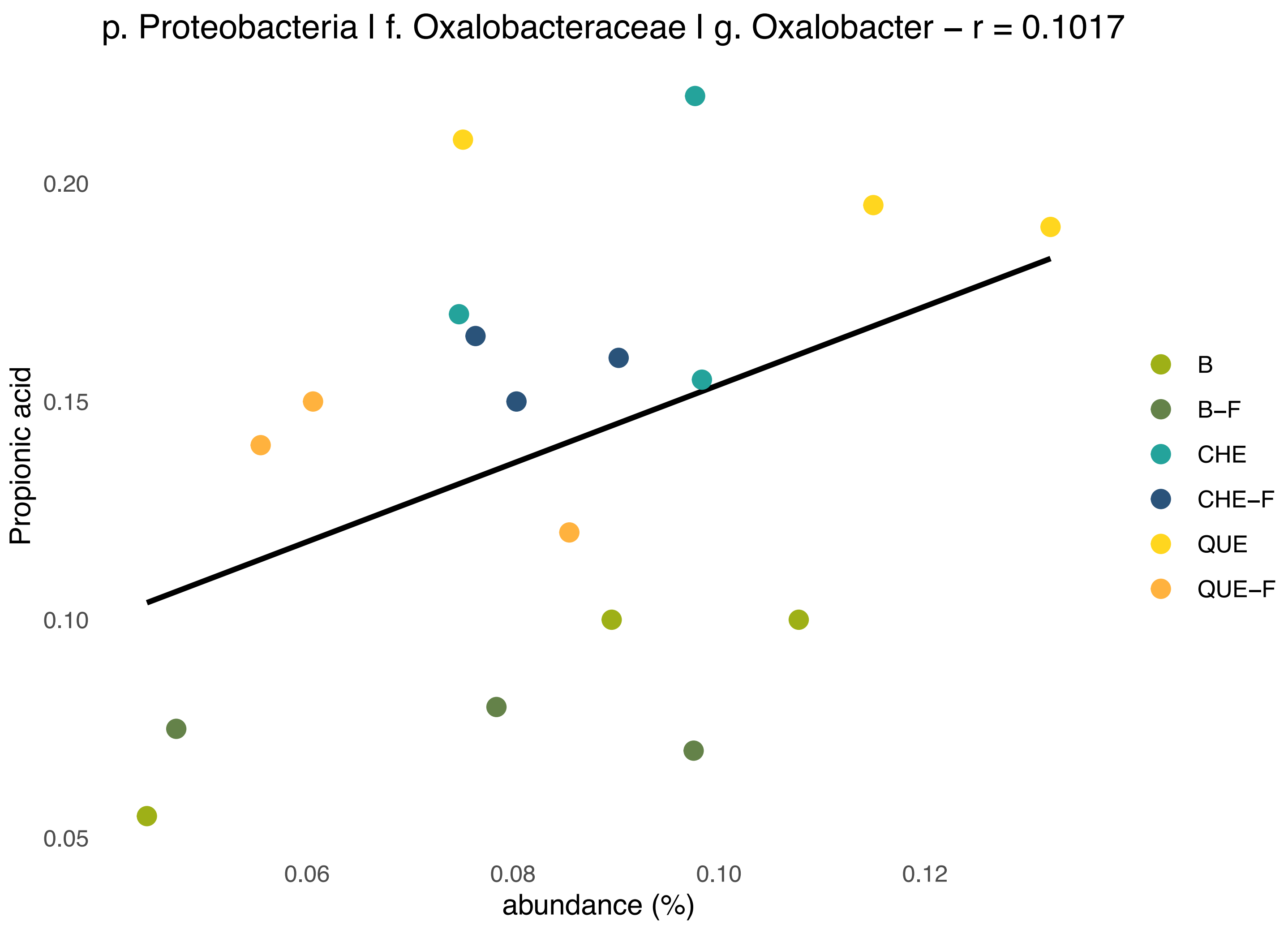

p. Proteobacteria I f. Oxalobacteraceae I g. Oxalobacter – r = 0.1017
Propionic acid
0.20
0.15
0.10
0.05
0.06
0.08
0.10
0.12
abundance (%)
B
B–F
CHE
CHE–F
QUE
QUE–F

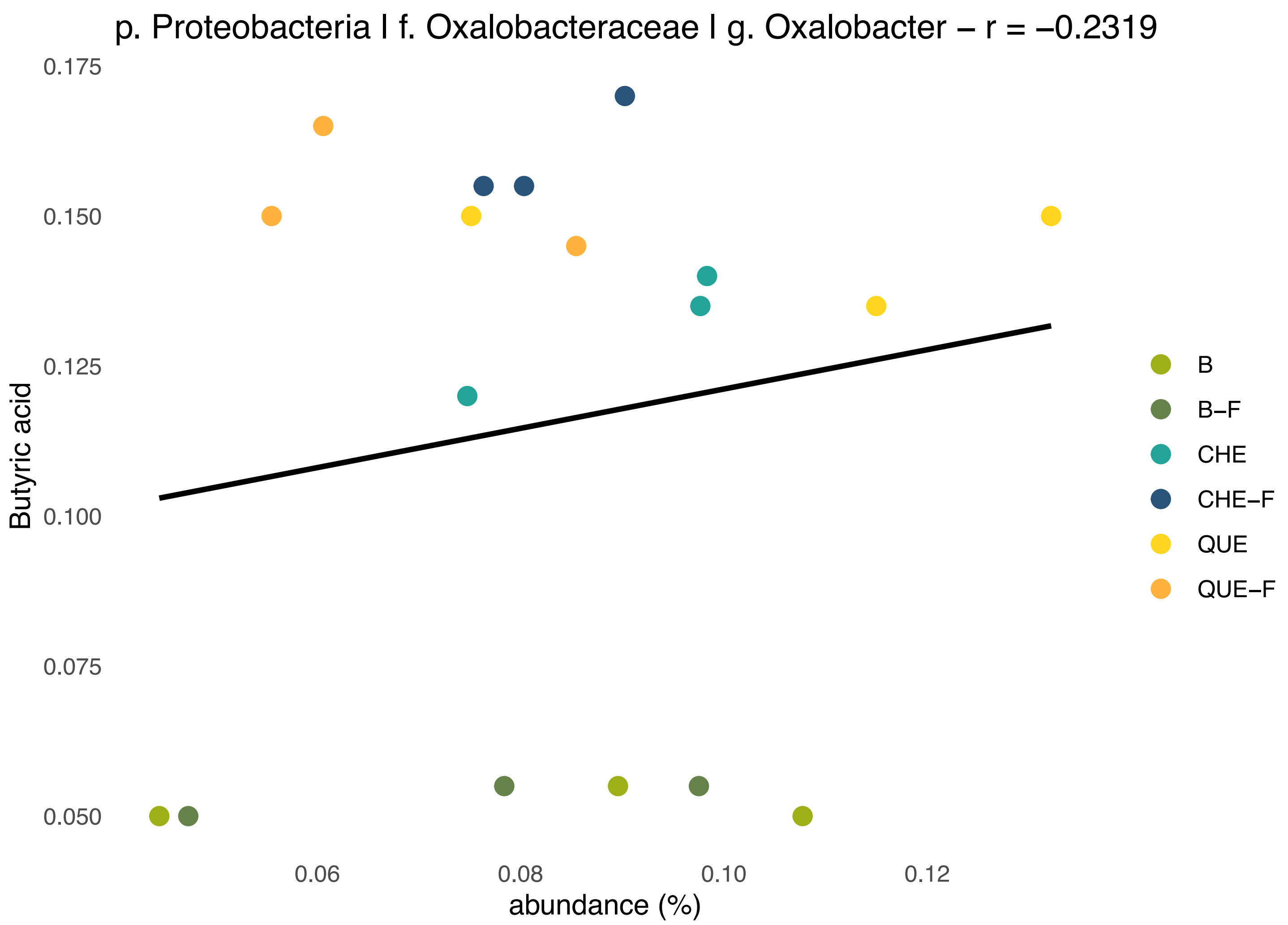

p. Proteobacteria I f. Oxalobacteraceae I g. Oxalobacter – r = –0.2319
Butyric acid
0.175
0.150
0.125
0.100
0.075
0.050
0.06
0.08
0.10
0.12
abundance (%)
B
B–F
CHE
CHE–F
QUE
QUE–F

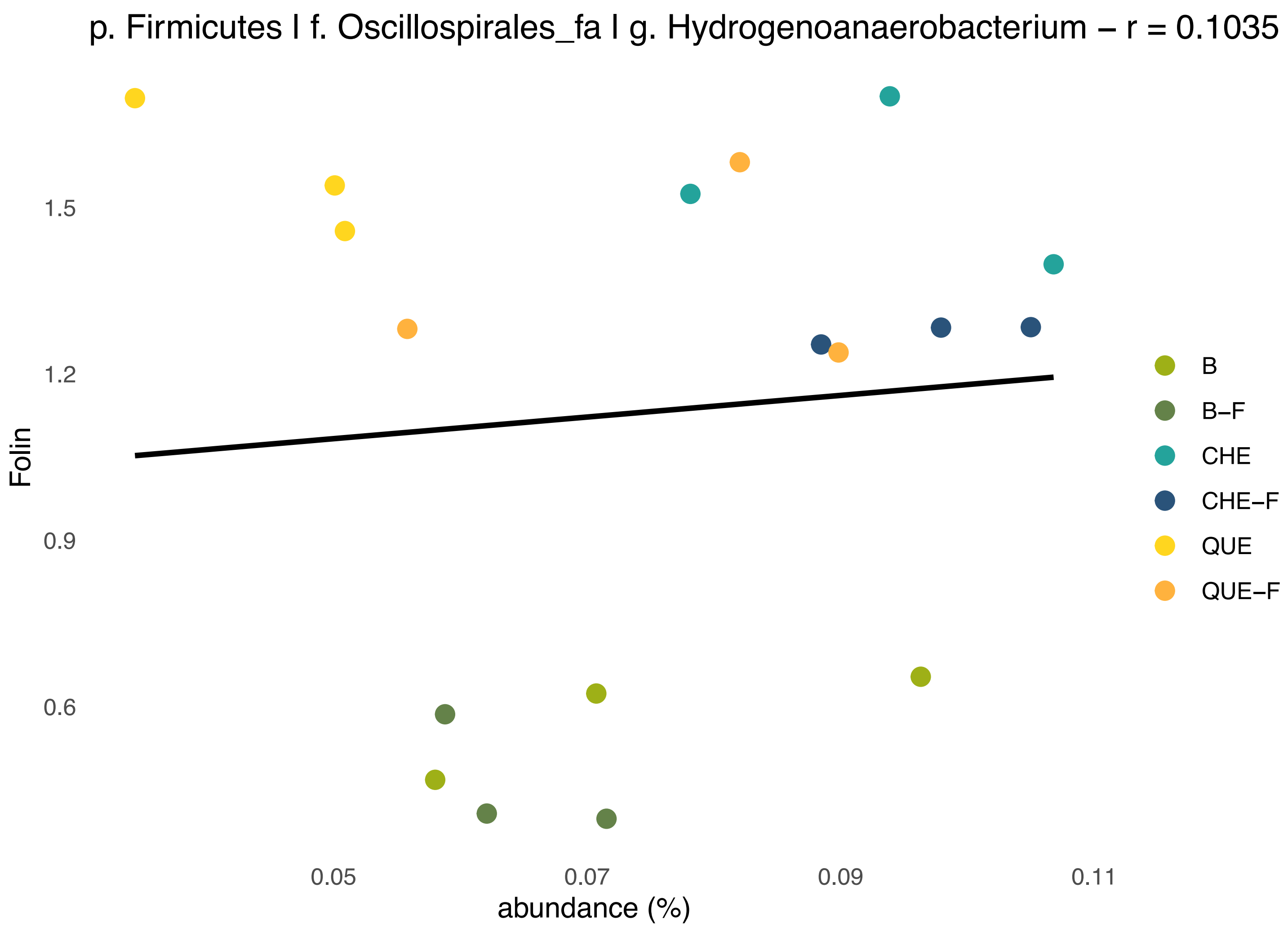

p. Firmicutes I f. Oscillospirales_fa I g. Hydrogenoanaerobacterium − r = 0.1035
Folin
1.5
1.2
0.9
0.6
0.05
0.07
0.09
0.11
abundance (%)
B
B−F
CHE
CHE−F
QUE
QUE−F

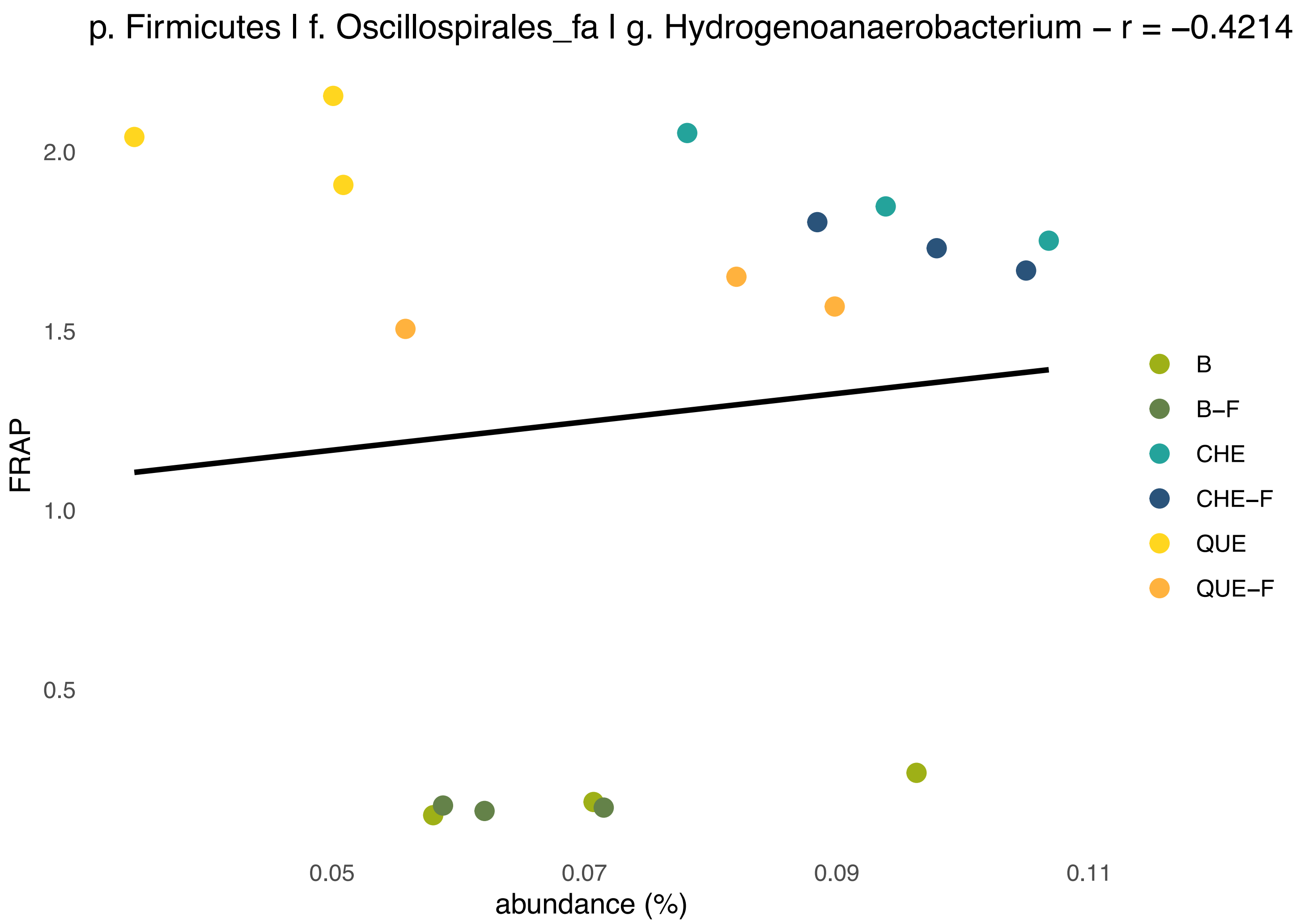
p. Firmicutes I f. Oscillospirales_fa I g. Hydrogenoanaerobacterium – r = –0.4214
FRAP
2.0
1.5
1.0
0.5
0.05
0.07
0.09
0.11
abundance (%)
B
B–F
CHE
CHE–F
QUE
QUE–F

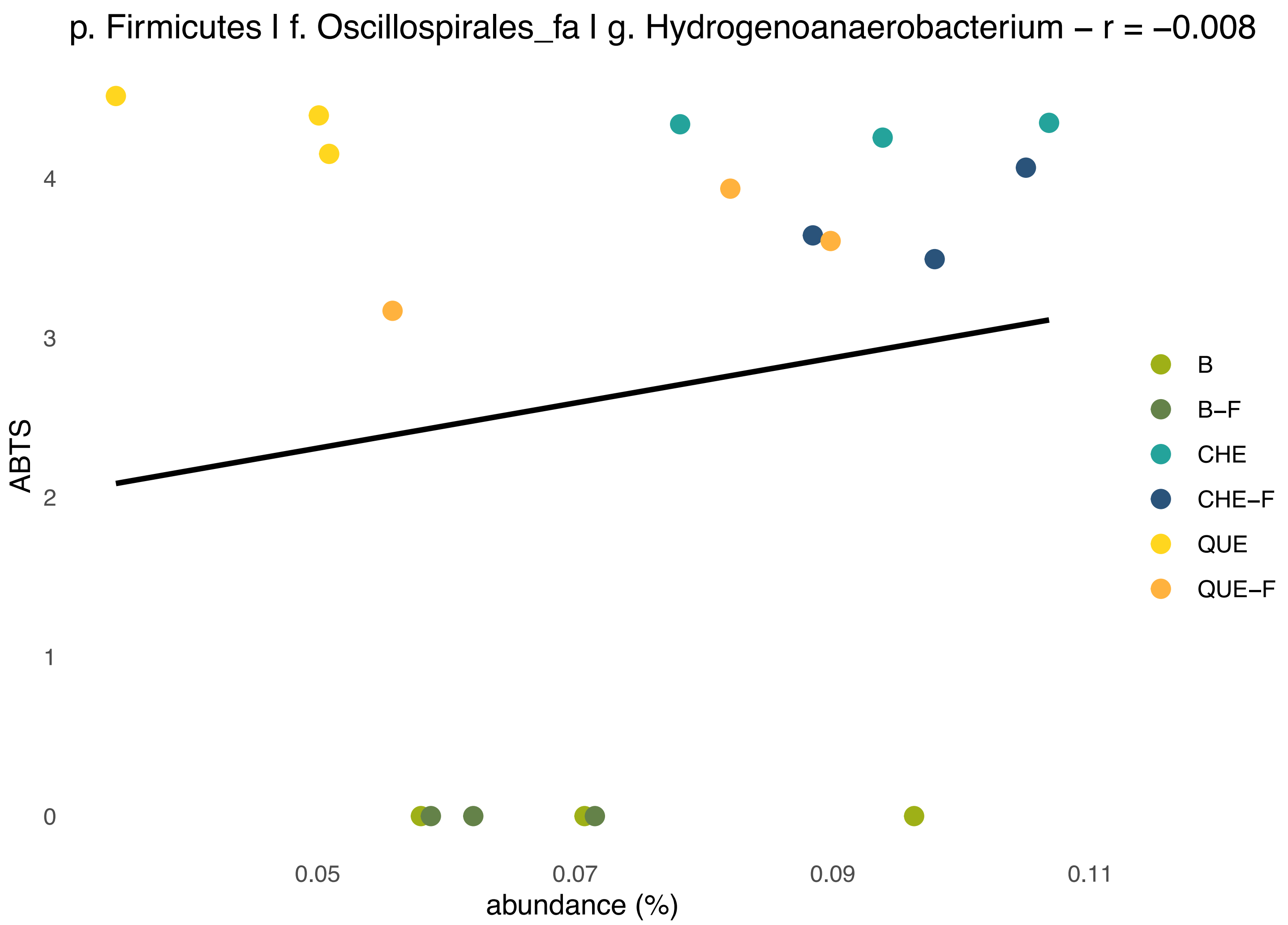
p. Firmicutes I f. Oscillospirales_fa I g. Hydrogenoanaerobacterium – r = −0.008
ABTS
0
1
2
3
4
0.05
0.07
0.09
0.11
abundance (%)
B
B−F
CHE
CHE−F
QUE
QUE−F

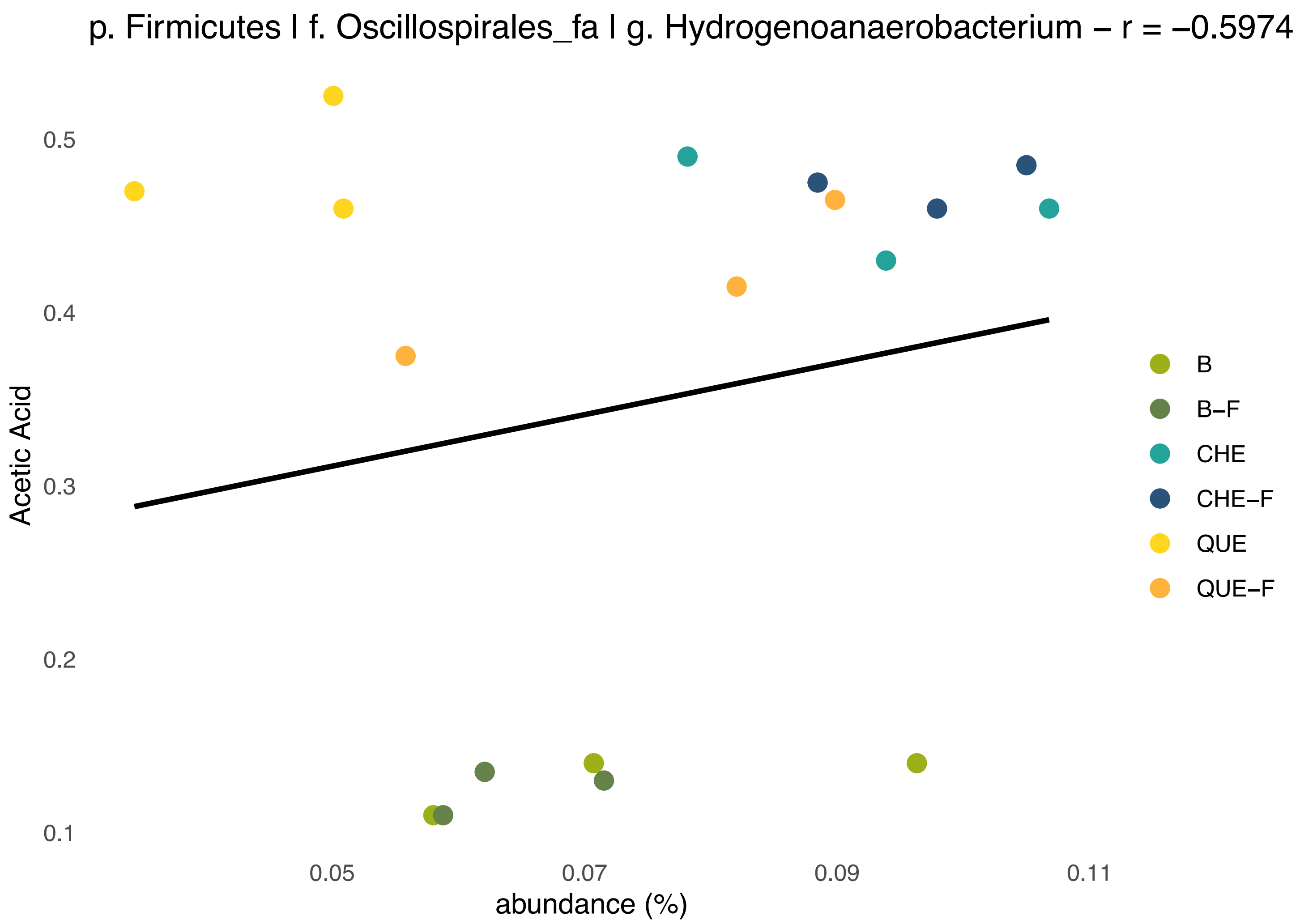

p. Firmicutes I f. Oscillospirales_fa I g. Hydrogenoanaerobacterium – r = –0.5974
Acetic Acid
0.5
0.4
0.3
0.2
0.1
0.05
0.07
0.09
0.11
abundance (%)
B
B–F
CHE
CHE–F
QUE
QUE–F

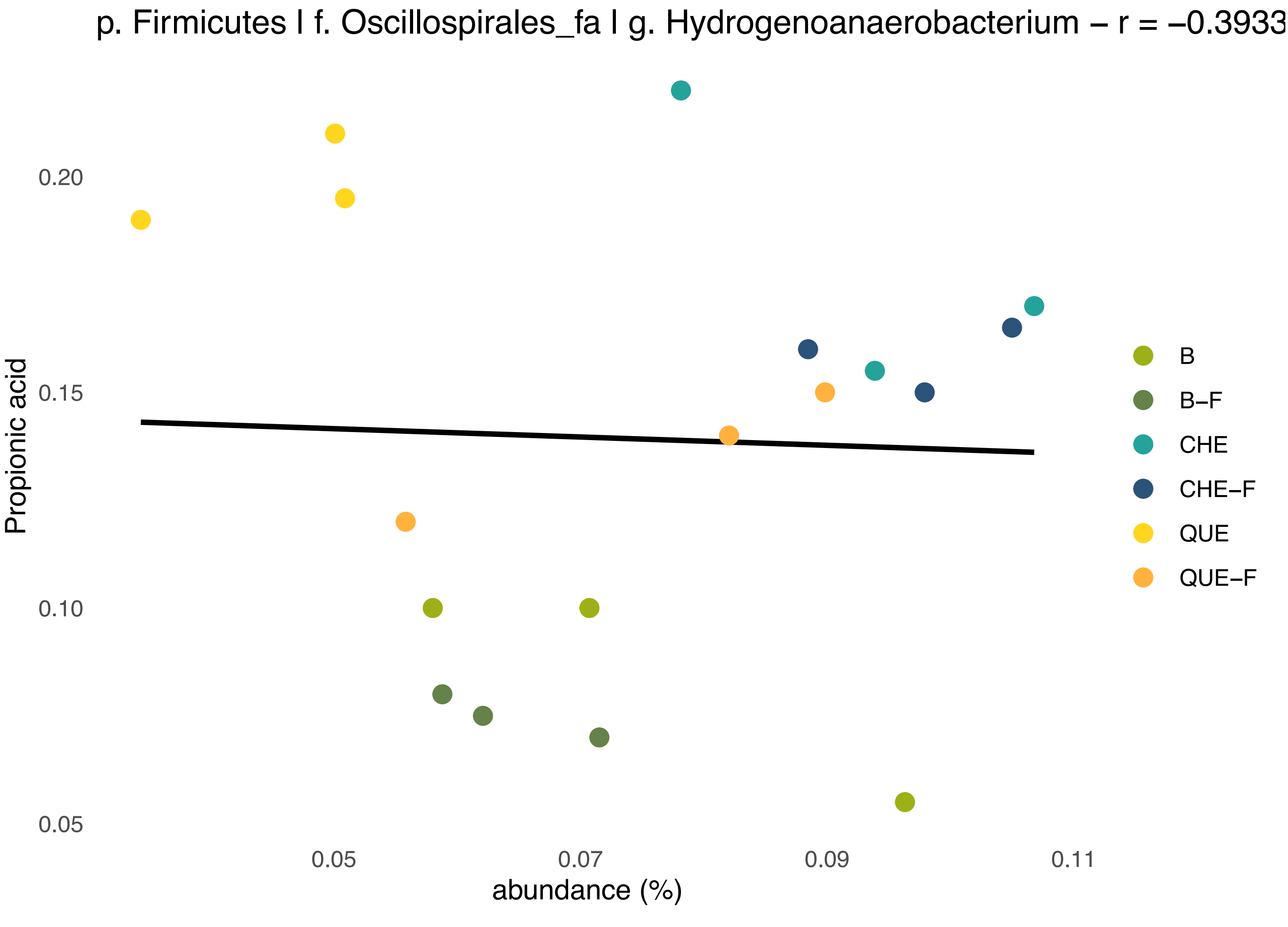
p. Firmicutes I f. Oscillospirales_fa I g. Hydrogenoanaerobacterium – r = –0.3933
Propionic acid
0.20
0.15
0.10
0.05
0.05
0.07
0.09
0.11
abundance (%)
B
B–F
CHE
CHE–F
QUE
QUE–F

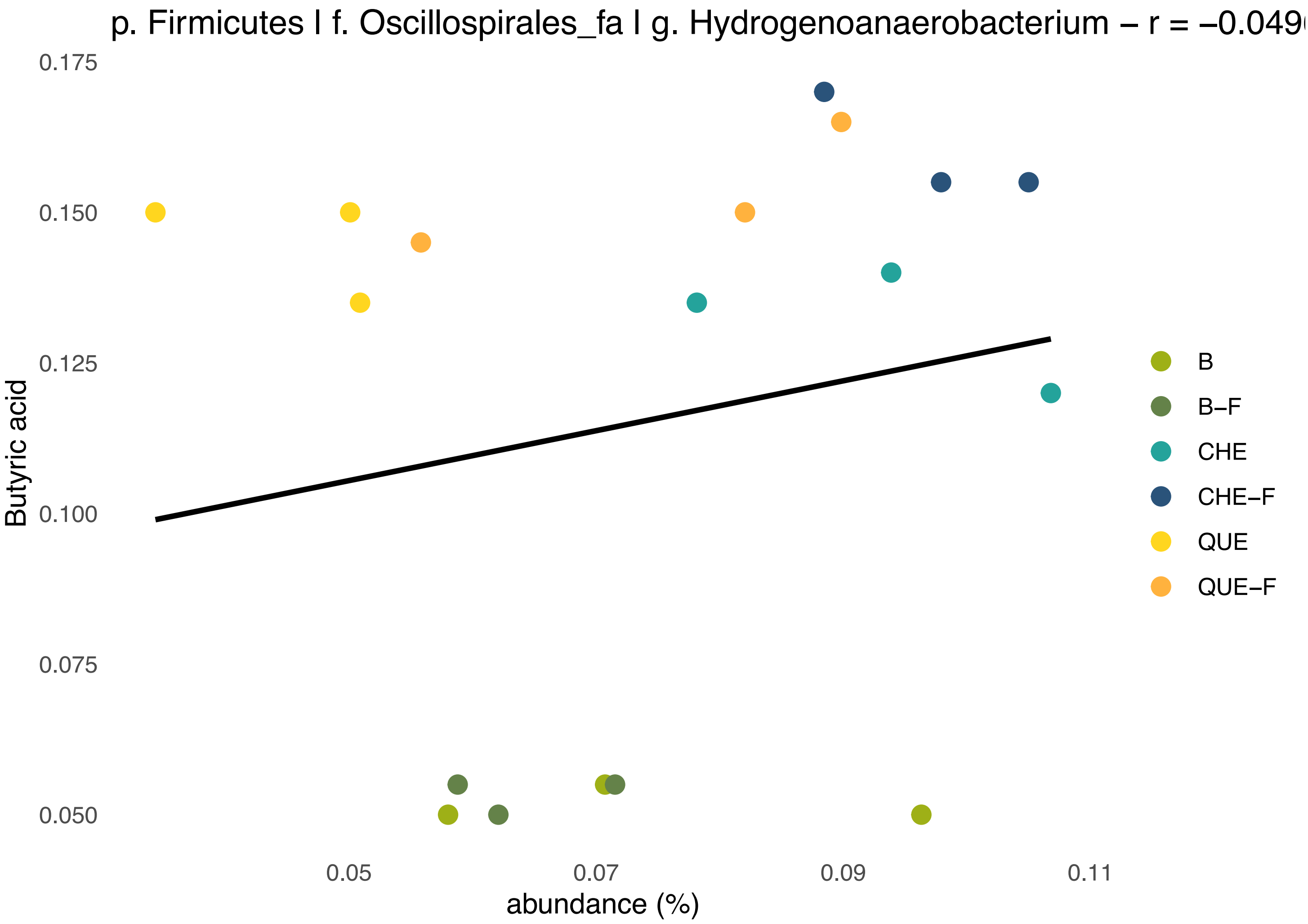
p. Firmicutes I f. Oscillospirales_fa I g. Hydrogenoanaerobacterium – r = –0.049
0.175
0.150
0.125
0.100
0.075
0.050
Butyric acid
0.05
0.07
0.09
0.11
abundance (%)
B
B–F
CHE
CHE–F
QUE
QUE–F

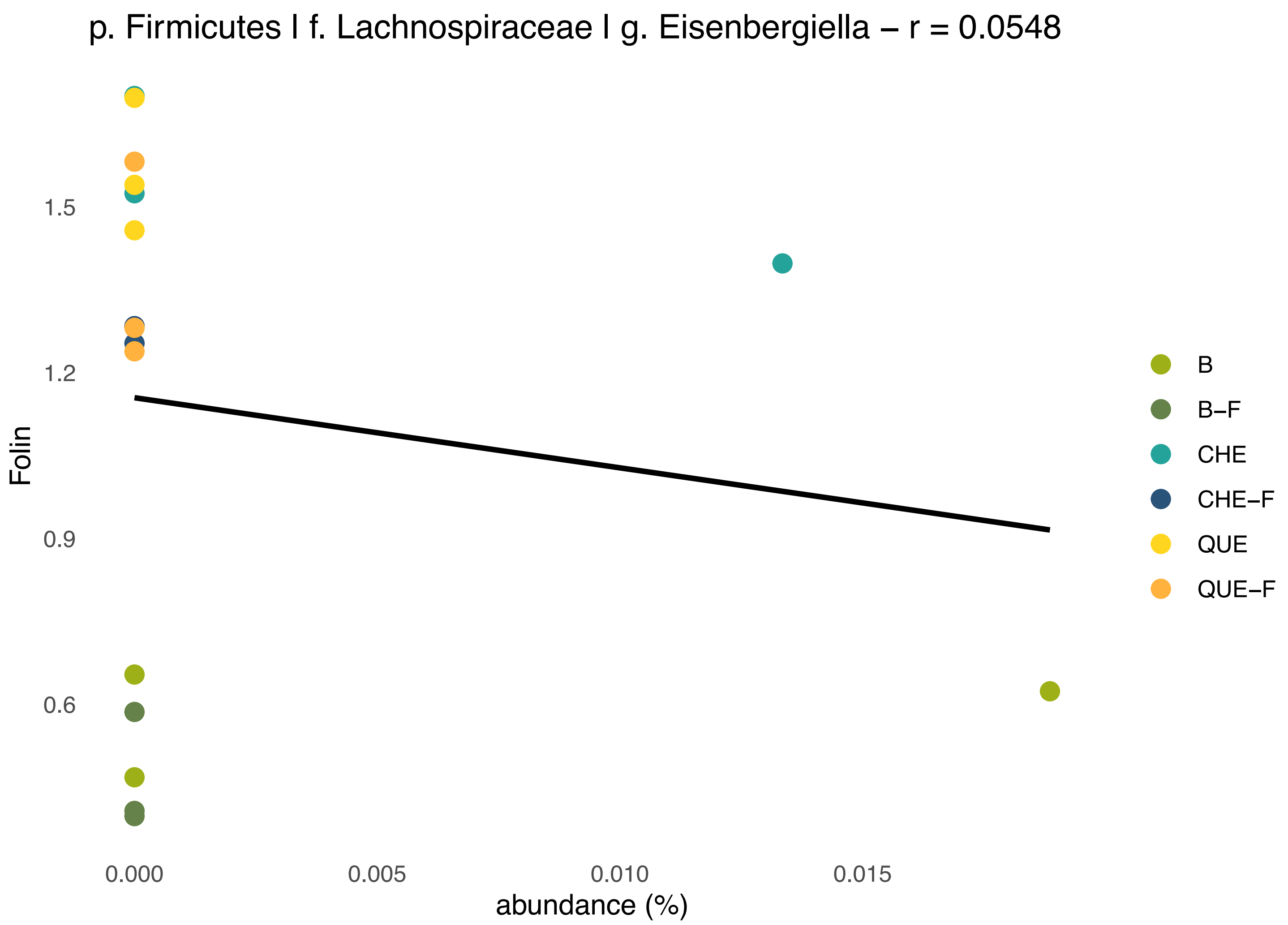
p. Firmicutes I f. Lachnospiraceae I g. Eisenbergiella – r = 0.0548
Folin
1.5
1.2
0.9
0.6
0.000
0.005
0.010
0.015
abundance (%)
B
B–F
CHE
CHE–F
QUE
QUE–F

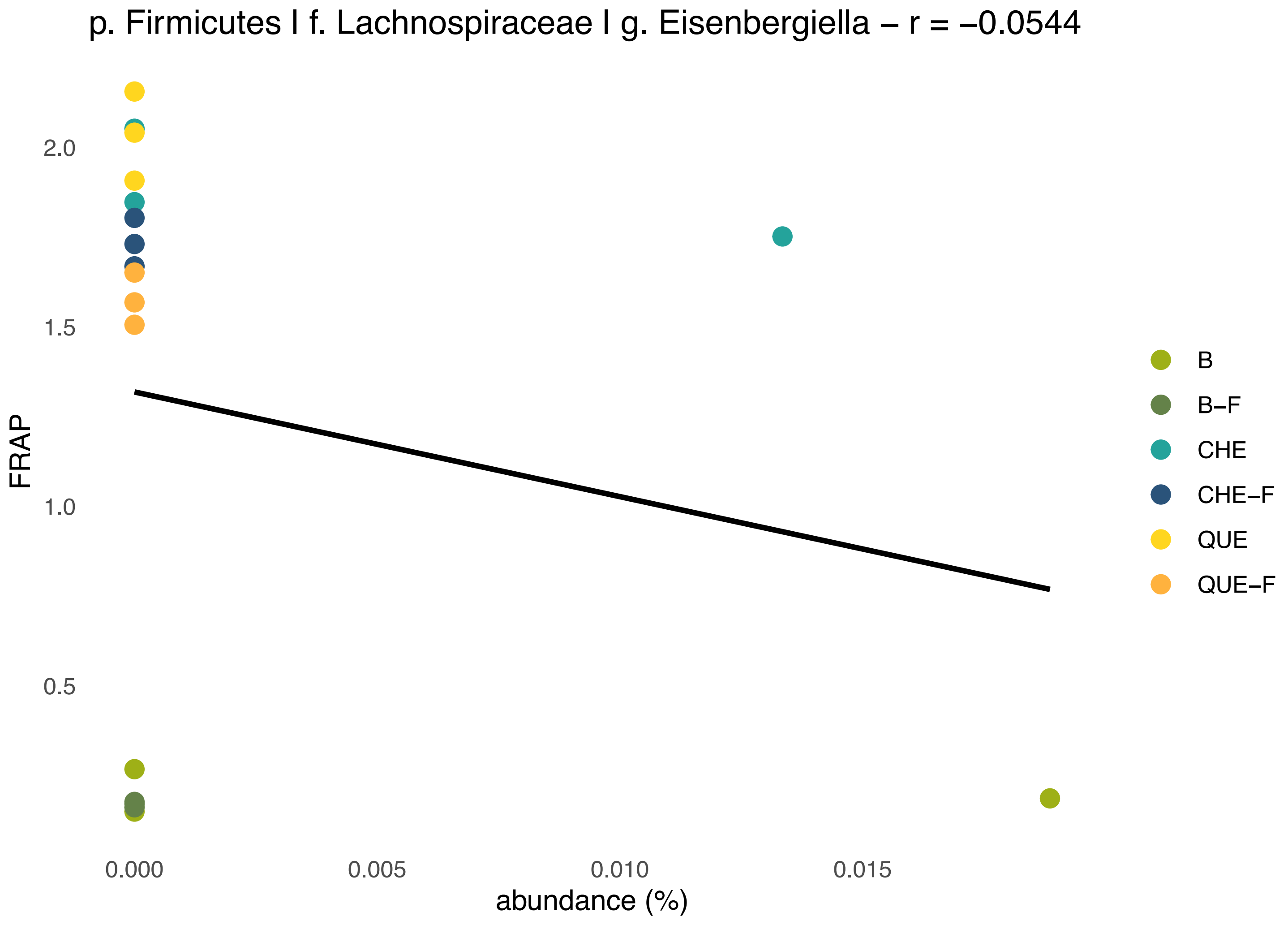

p. Firmicutes I f. Lachnospiraceae I g. Eisenbergiella – r = –0.0544
FRAP
2.0
1.5
1.0
0.5
0.000
0.005
0.010
0.015
abundance (%)
B
B–F
CHE
CHE–F
QUE
QUE–F

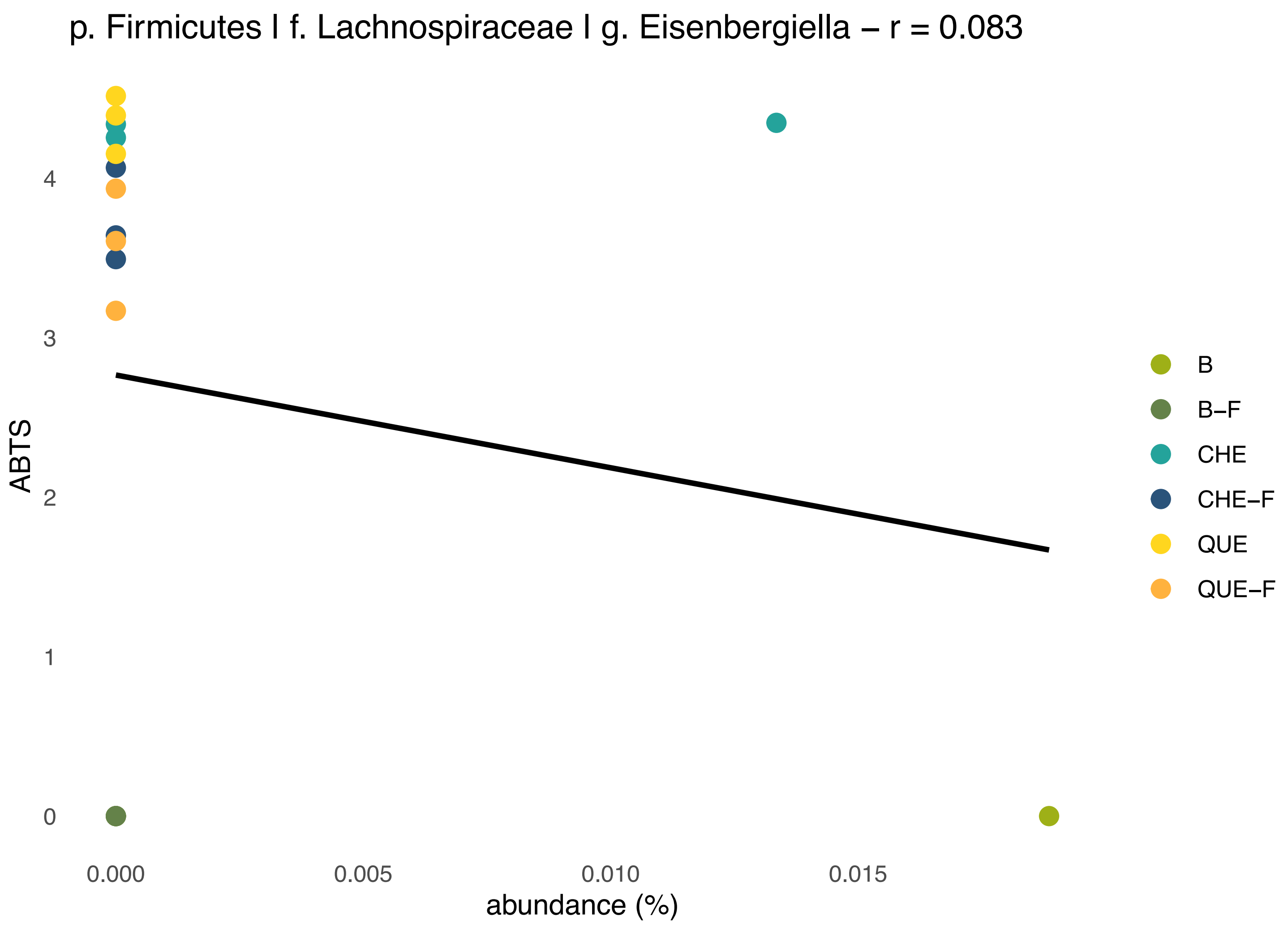
p. Firmicutes I f. Lachnospiraceae I g. Eisenbergiella – r = 0.083
ABTS
4
3
2
1
0
0.000
0.005
0.010
0.015
abundance (%)
B
B–F
CHE
CHE–F
QUE
QUE–F

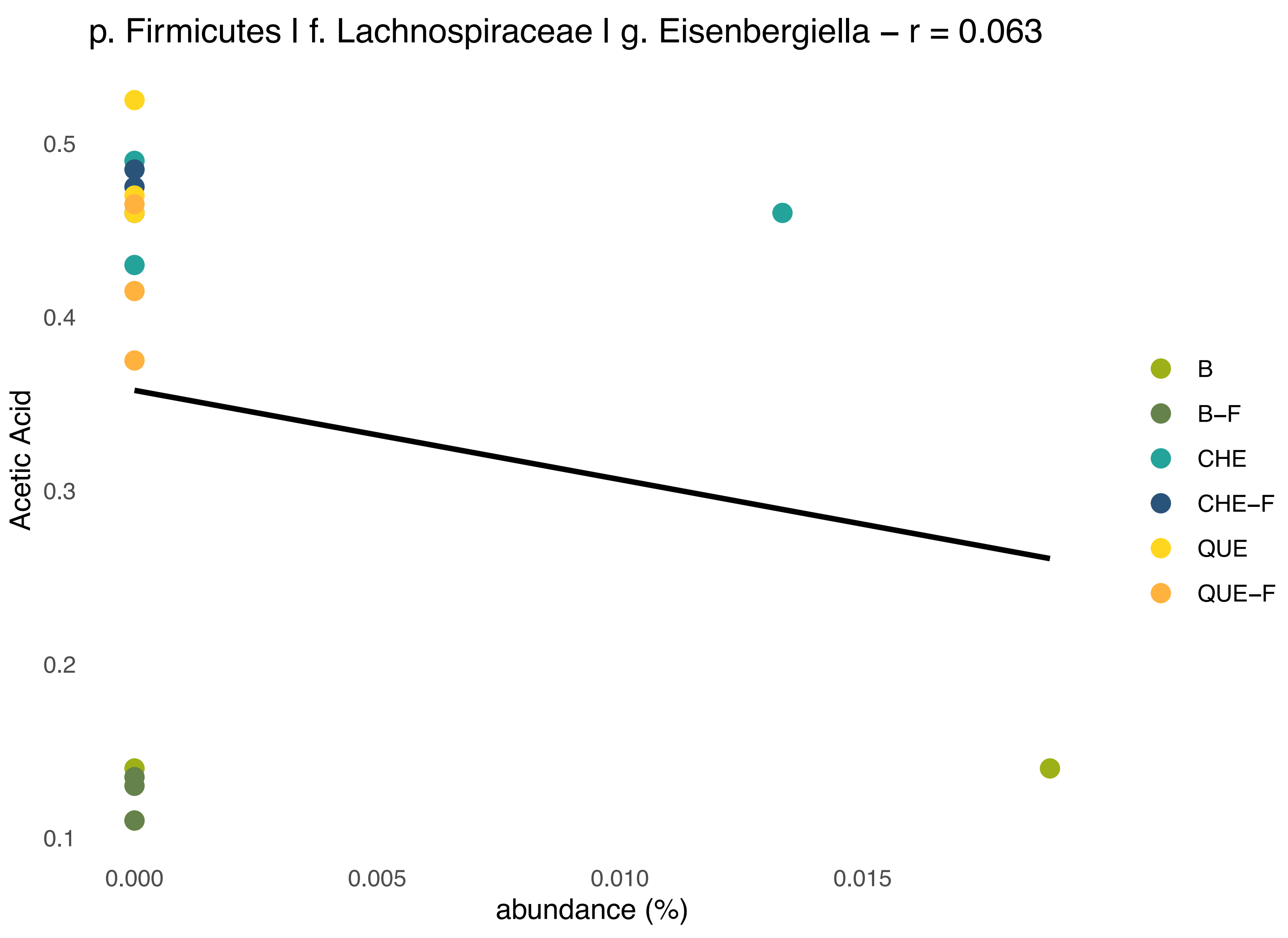

p. Firmicutes | f. Lachnospiraceae | g. Eisenbergiella – r = 0.063
Acetic Acid
0.5
0.4
0.3
0.2
0.1
0.000
0.005
0.010
0.015
abundance (%)
B
B–F
CHE
CHE–F
QUE
QUE–F

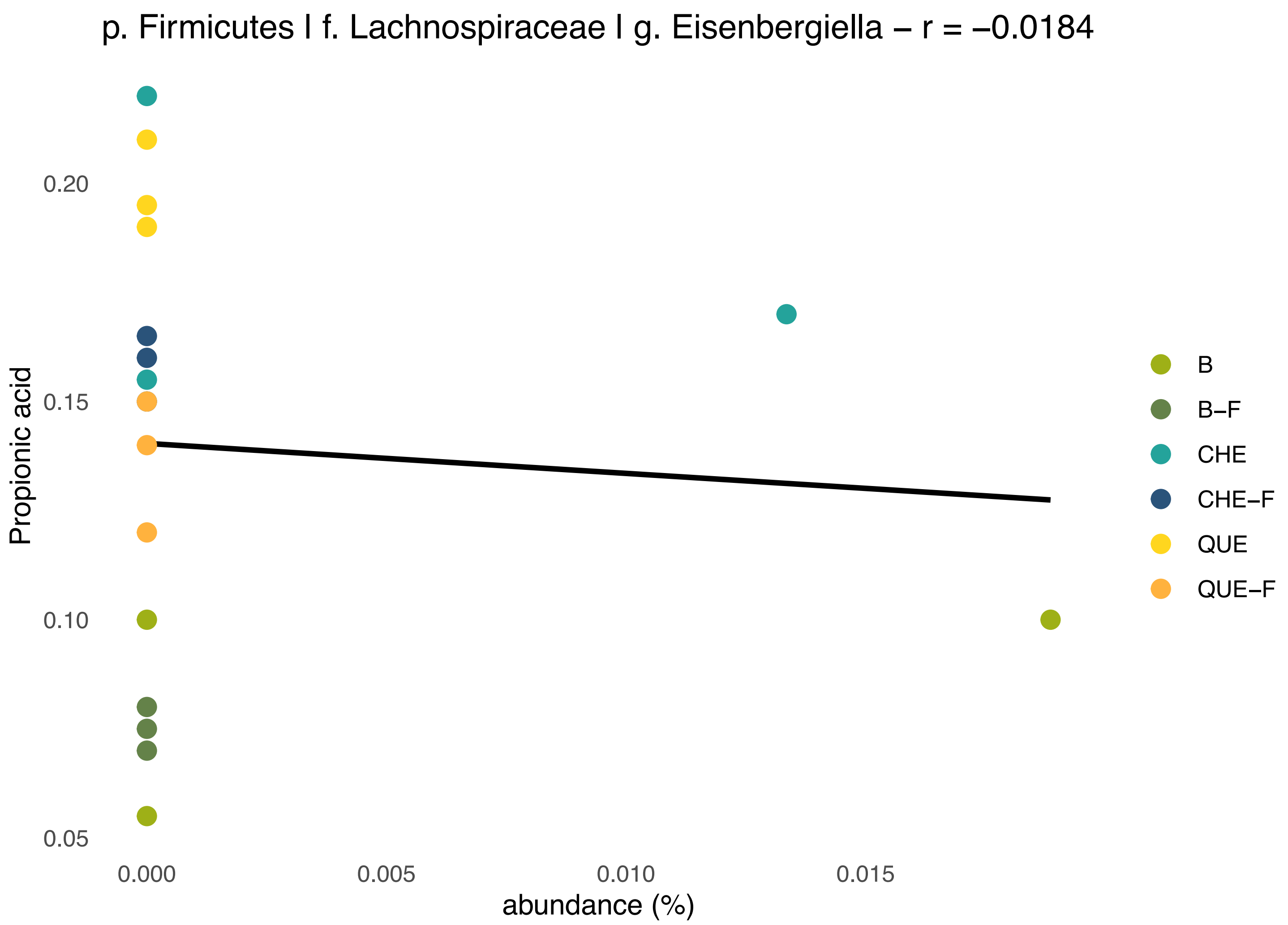
p. Firmicutes l f. Lachnospiraceae l g. Eisenbergiella – r = –0.0184
Propionic acid
0.20
0.15
0.10
0.05
0.000
0.005
0.010
0.015
abundance (%)
B
B–F
CHE
CHE–F
QUE
QUE–F

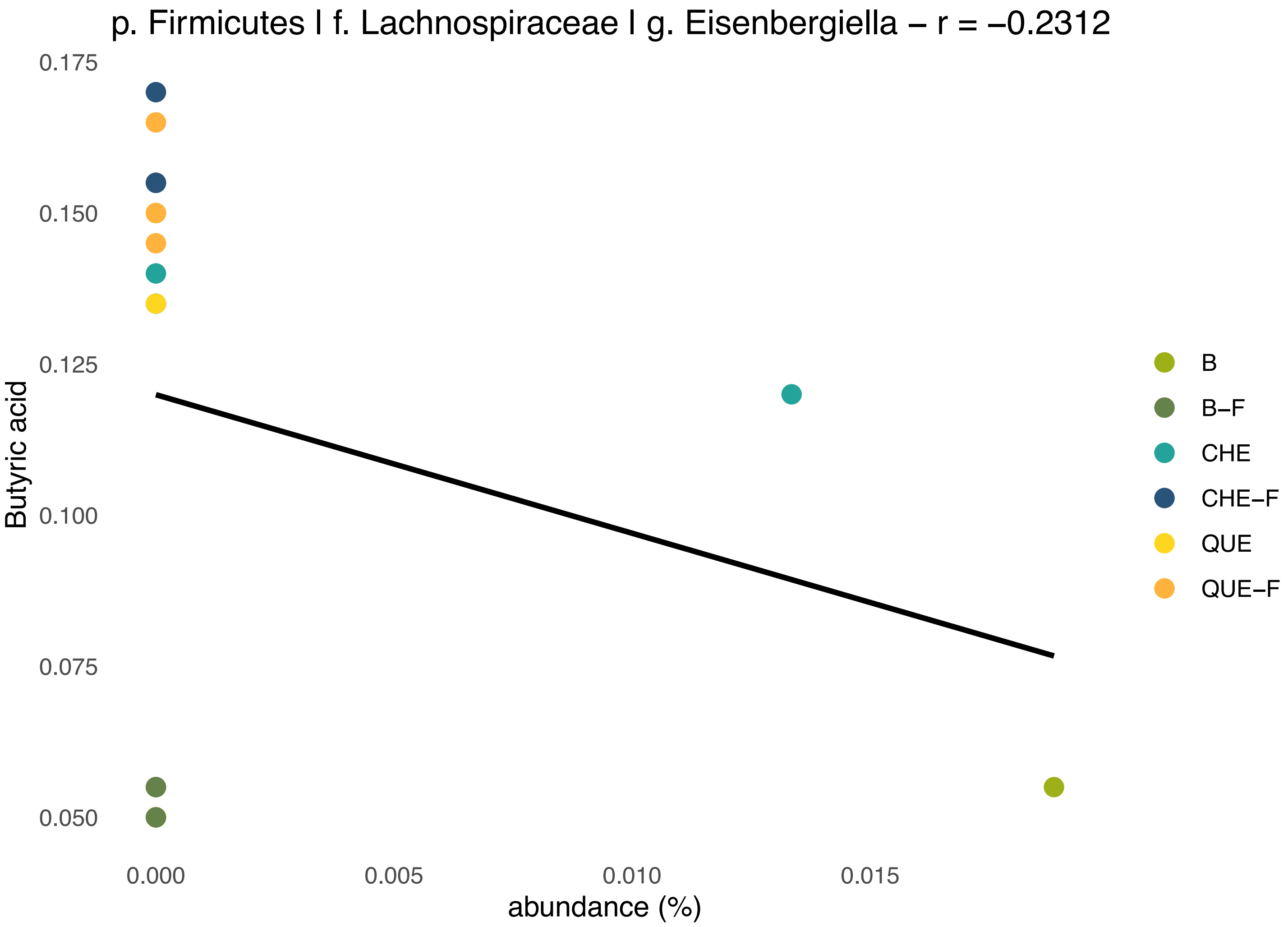

p. Firmicutes | f. Lachnospiraceae | g. Eisenbergiella – r = –0.2312
Butyric acid
0.175
0.150
0.125
0.100
0.075
0.050
0.000
0.005
0.010
0.015
abundance (%)
B
B–F
CHE
CHE–F
QUE
QUE–F

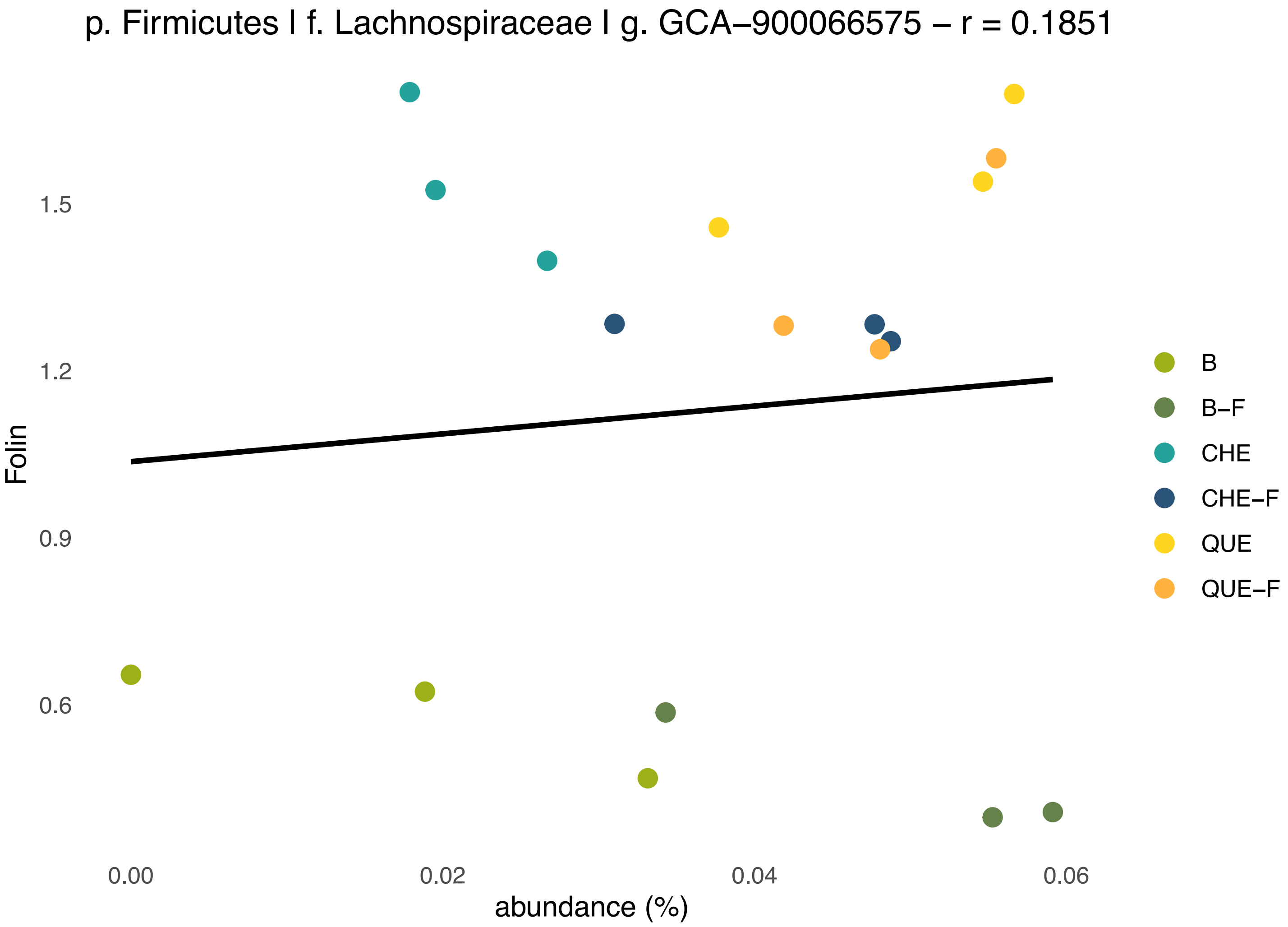
p. Firmicutes I f. Lachnospiraceae I g. GCA−900066575 − r = 0.1851
1.5
1.2
0.9
0.6
Folin
0.00
0.02
0.04
0.06
abundance (%)
B
B−F
CHE
CHE−F
QUE
QUE−F

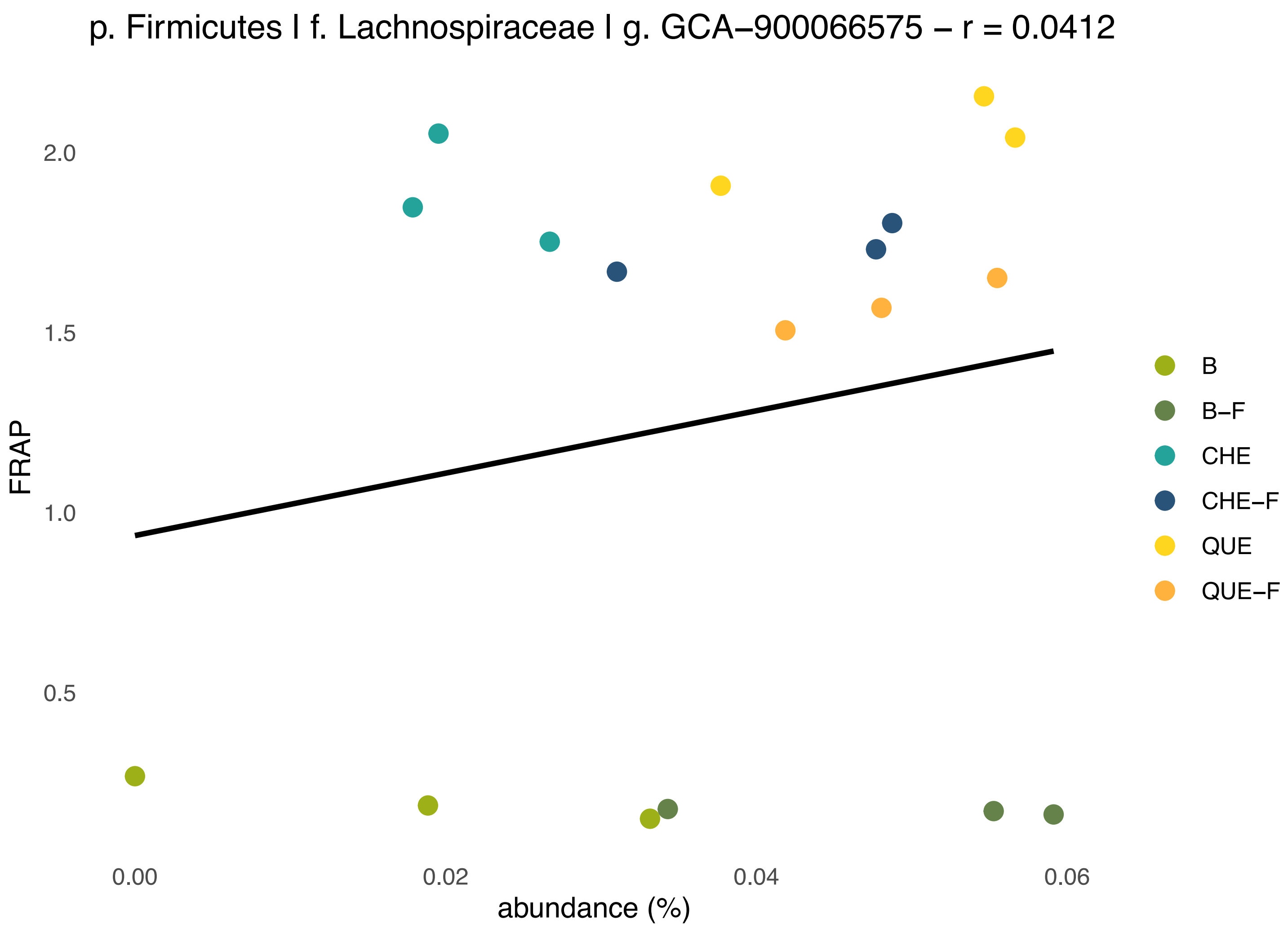
p. Firmicutes I f. Lachnospiraceae I g. GCA−900066575 − r = 0.0412
2.0
1.5
1.0
0.5
FRAP
0.00
0.02
0.04
0.06
abundance (%)
B
B−F
CHE
CHE−F
QUE
QUE−F

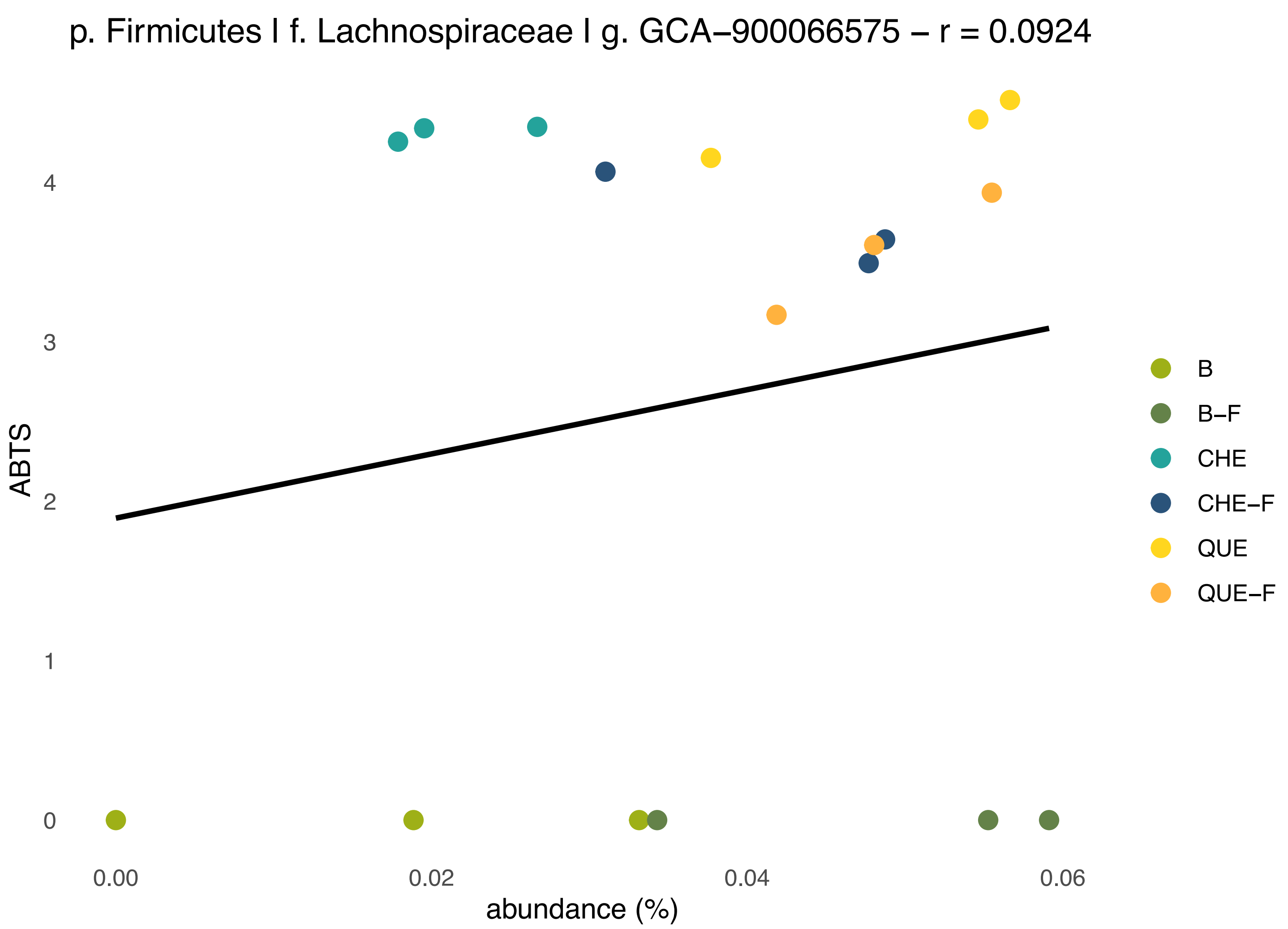
p. Firmicutes I f. Lachnospiraceae I g. GCA−900066575 − r = 0.0924
ABTS
abundance (%)
0
1
2
3
4
0.00
0.02
0.04
0.06
B
B−F
CHE
CHE−F
QUE
QUE−F

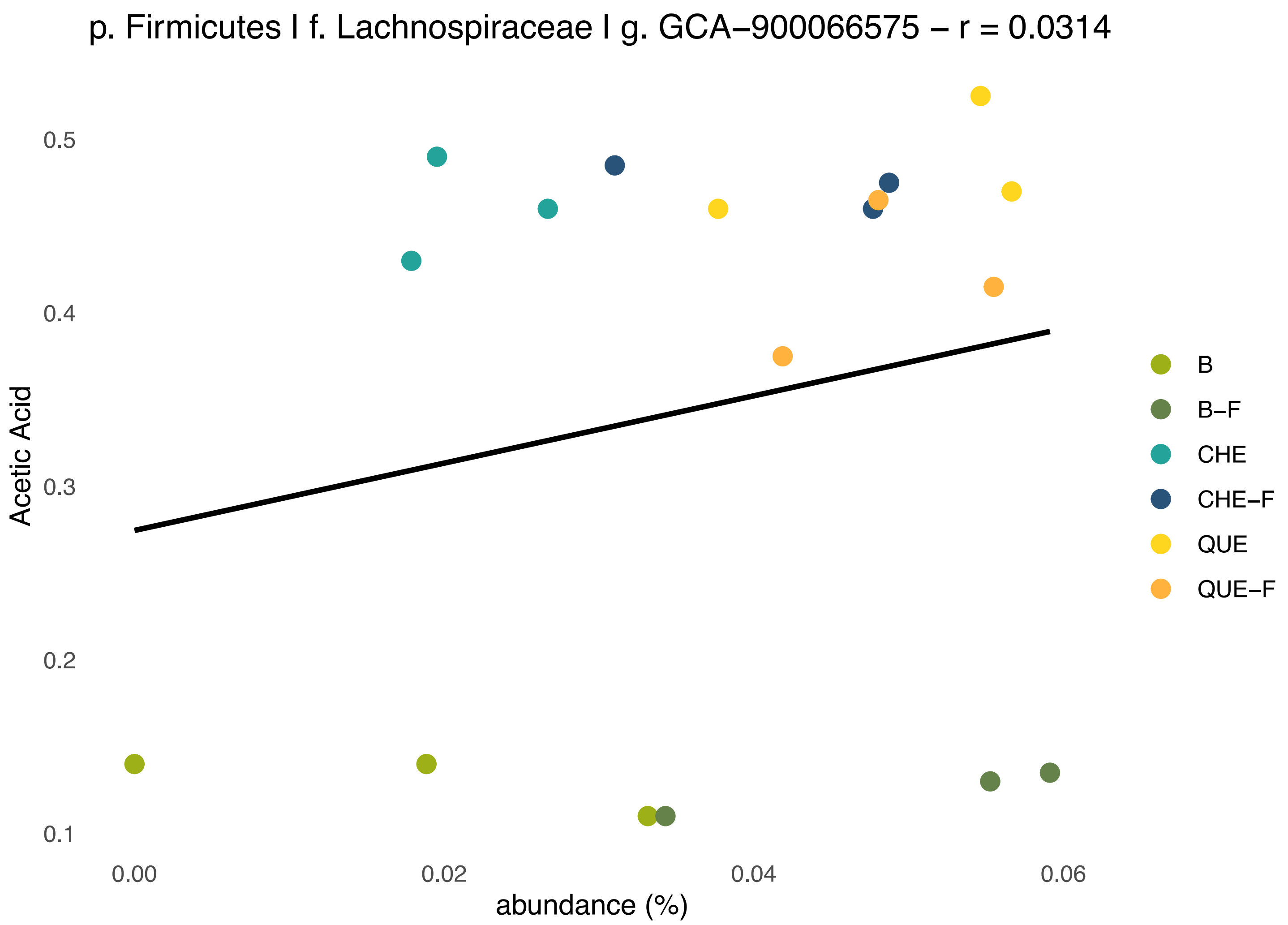

p. Firmicutes I f. Lachnospiraceae I g. GCA−900066575 − r = 0.0314
Acetic Acid
0.5
0.4
0.3
0.2
0.1
0.00
0.02
0.04
0.06
abundance (%)
B
B−F
CHE
CHE−F
QUE
QUE−F

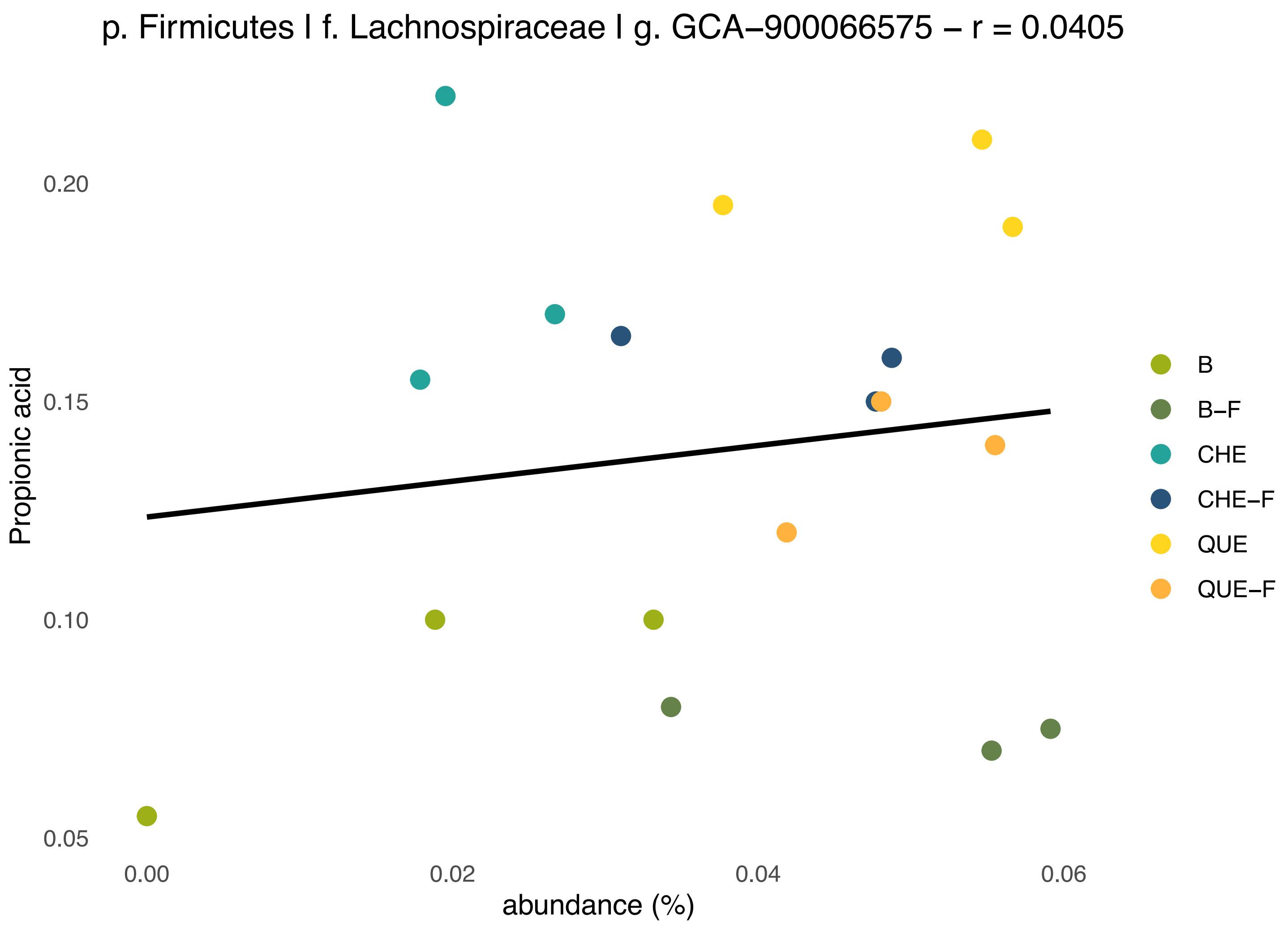

p. Firmicutes l f. Lachnospiraceae l g. GCA−900066575 − r = 0.0405
Propionic acid
0.20
0.15
0.10
0.05
0.00
0.02
0.04
0.06
abundance (%)
B
B−F
CHE
CHE−F
QUE
QUE−F

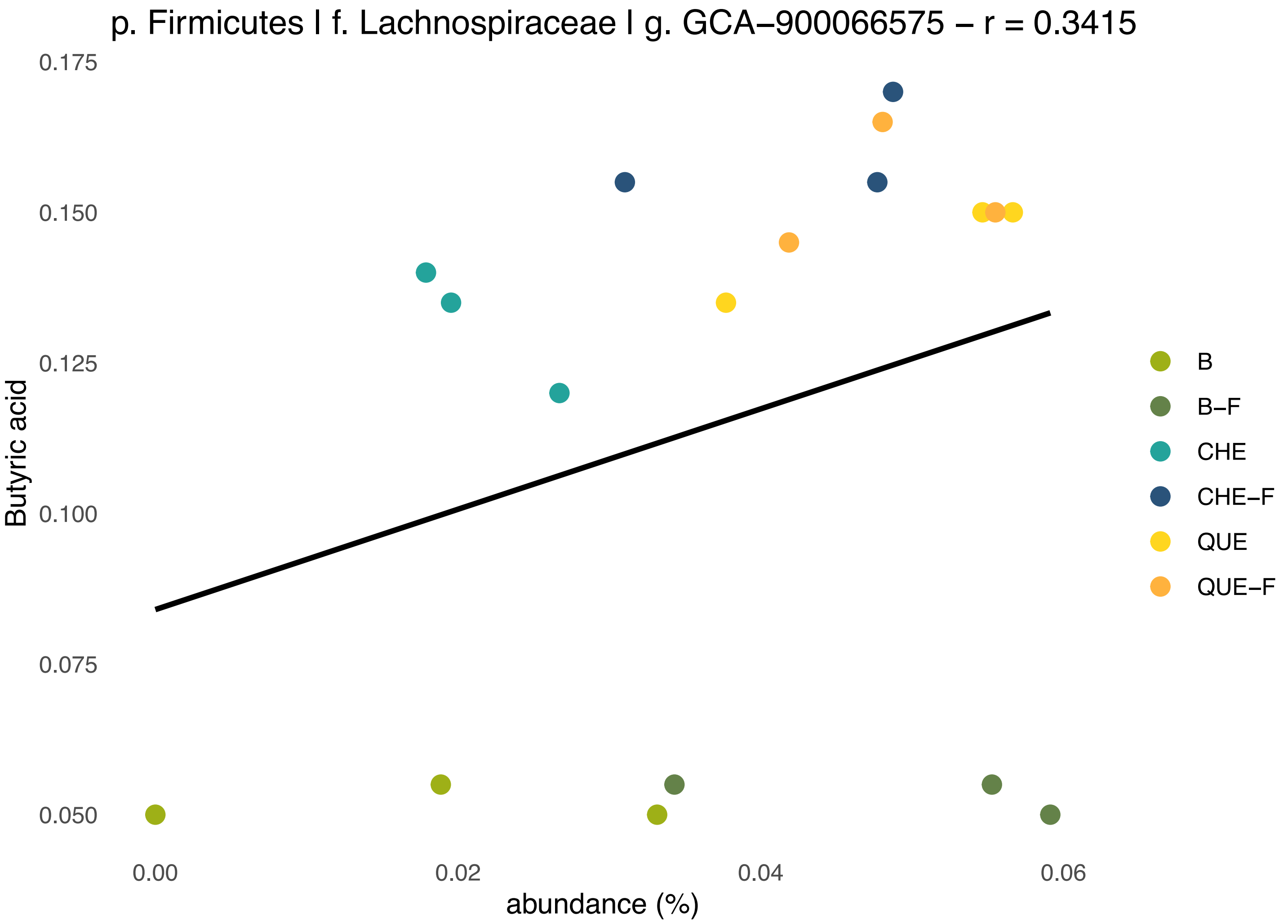
p. Firmicutes I f. Lachnospiraceae I g. GCA−900066575 − r = 0.3415
0.175
0.150
0.125
0.100
0.075
0.050
Butyric acid
0.00
0.02
0.04
0.06
abundance (%)
B
B−F
CHE
CHE−F
QUE
QUE−F

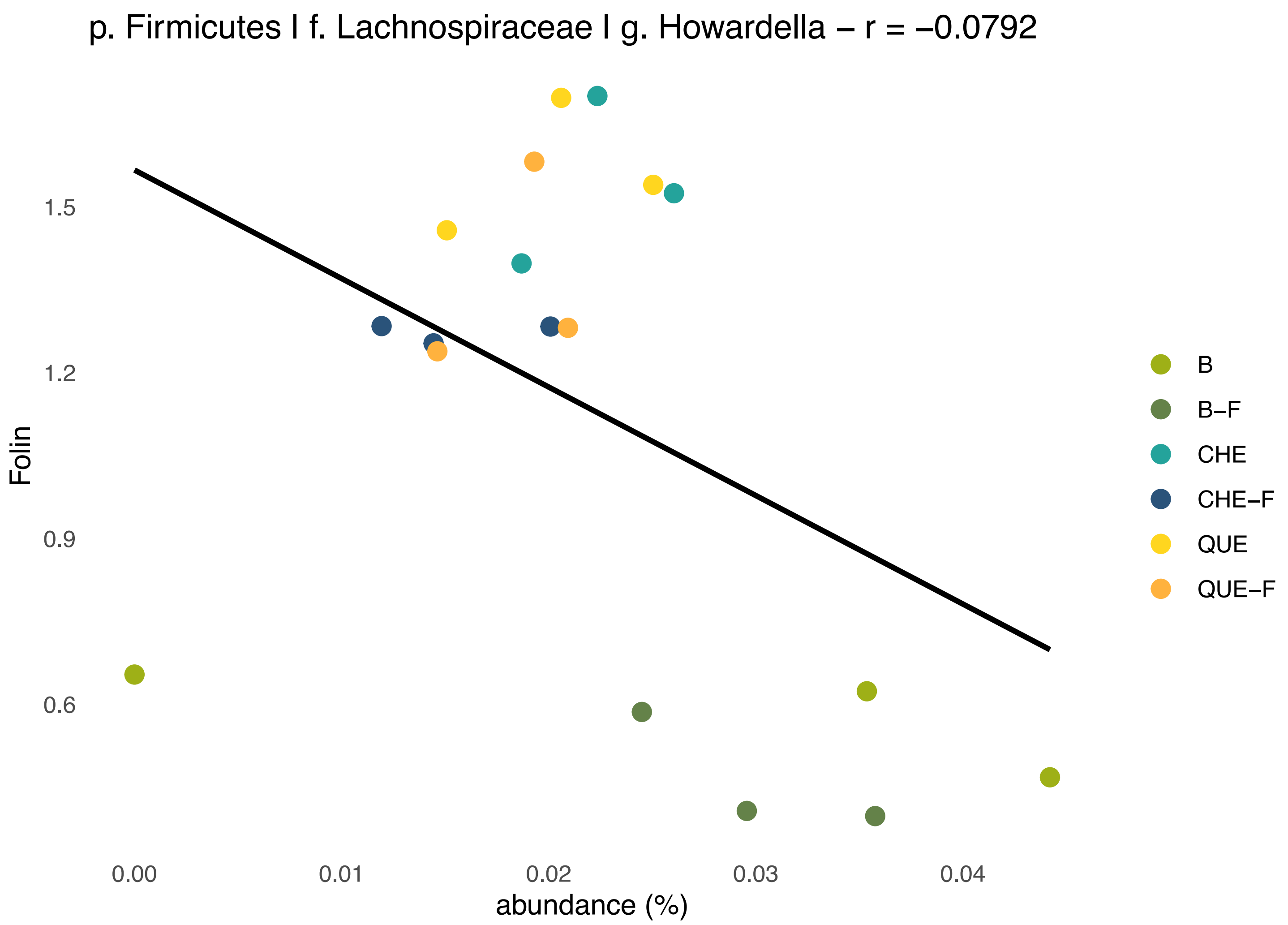

p. Firmicutes | f. Lachnospiraceae | g. Howardella – r = –0.0792
Folin
1.5
1.2
0.9
0.6
0.00
0.01
0.02
0.03
0.04
abundance (%)
B
B–F
CHE
CHE–F
QUE
QUE–F

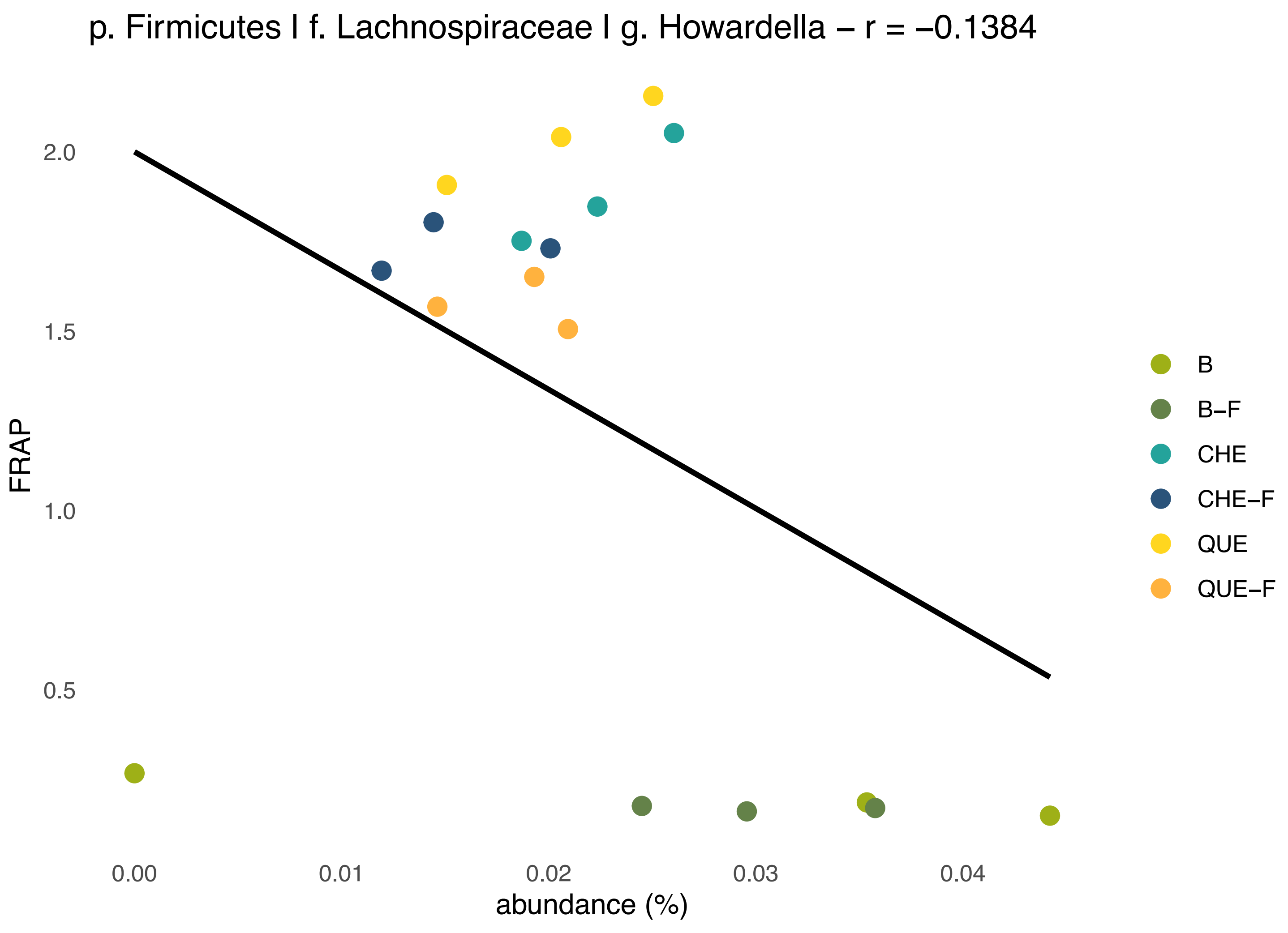

p. Firmicutes | f. Lachnospiraceae | g. Howardella – r = −0.1384
FRAP
2.0
1.5
1.0
0.5
0.00
0.01
0.02
0.03
0.04
abundance (%)
B
B−F
CHE
CHE−F
QUE
QUE−F

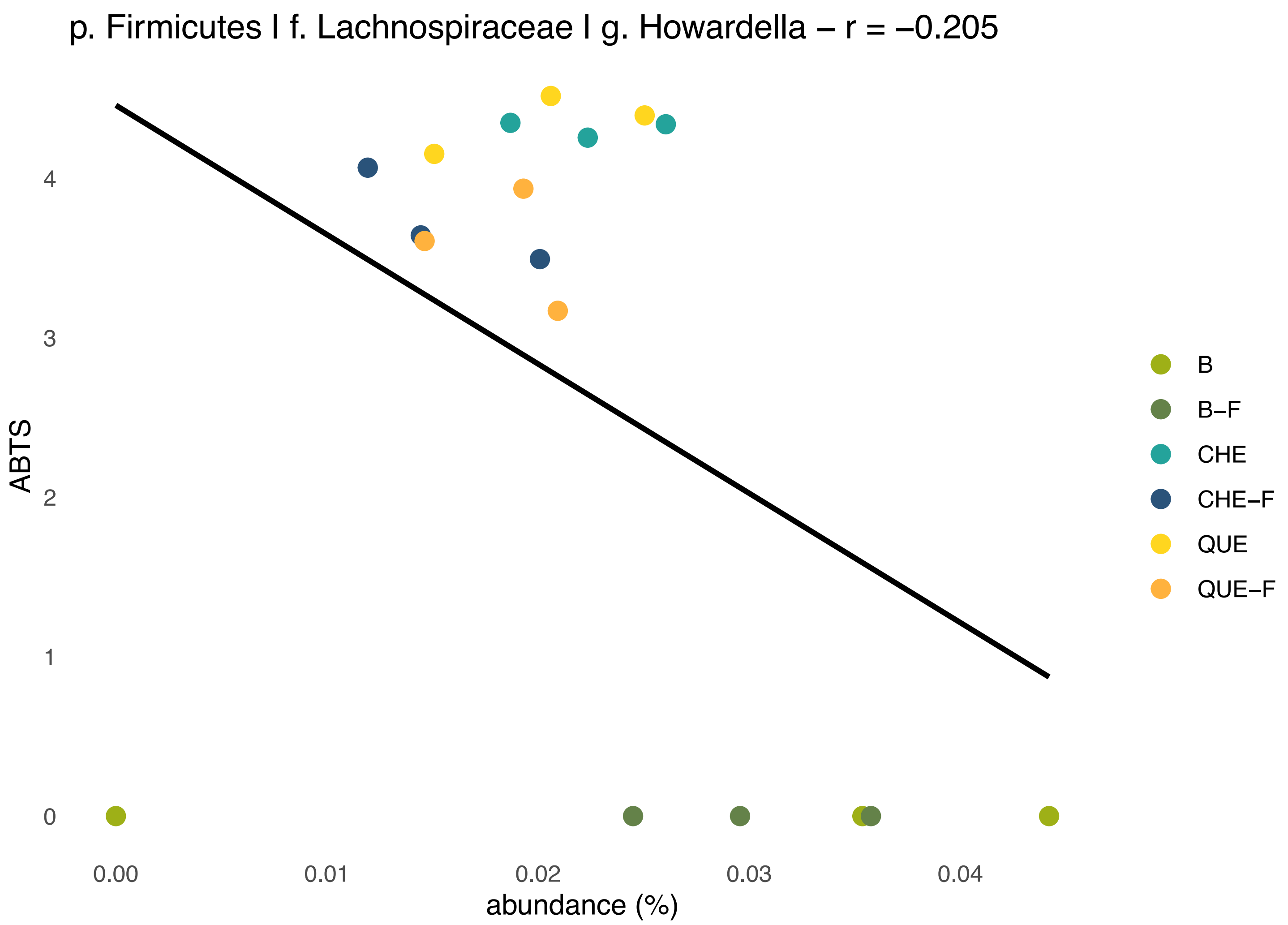

p. Firmicutes I f. Lachnospiraceae I g. Howardella – r = –0.205
ABTS
4
3
2
1
0
0.00
0.01
0.02
0.03
0.04
abundance (%)
B
B–F
CHE
CHE–F
QUE
QUE–F

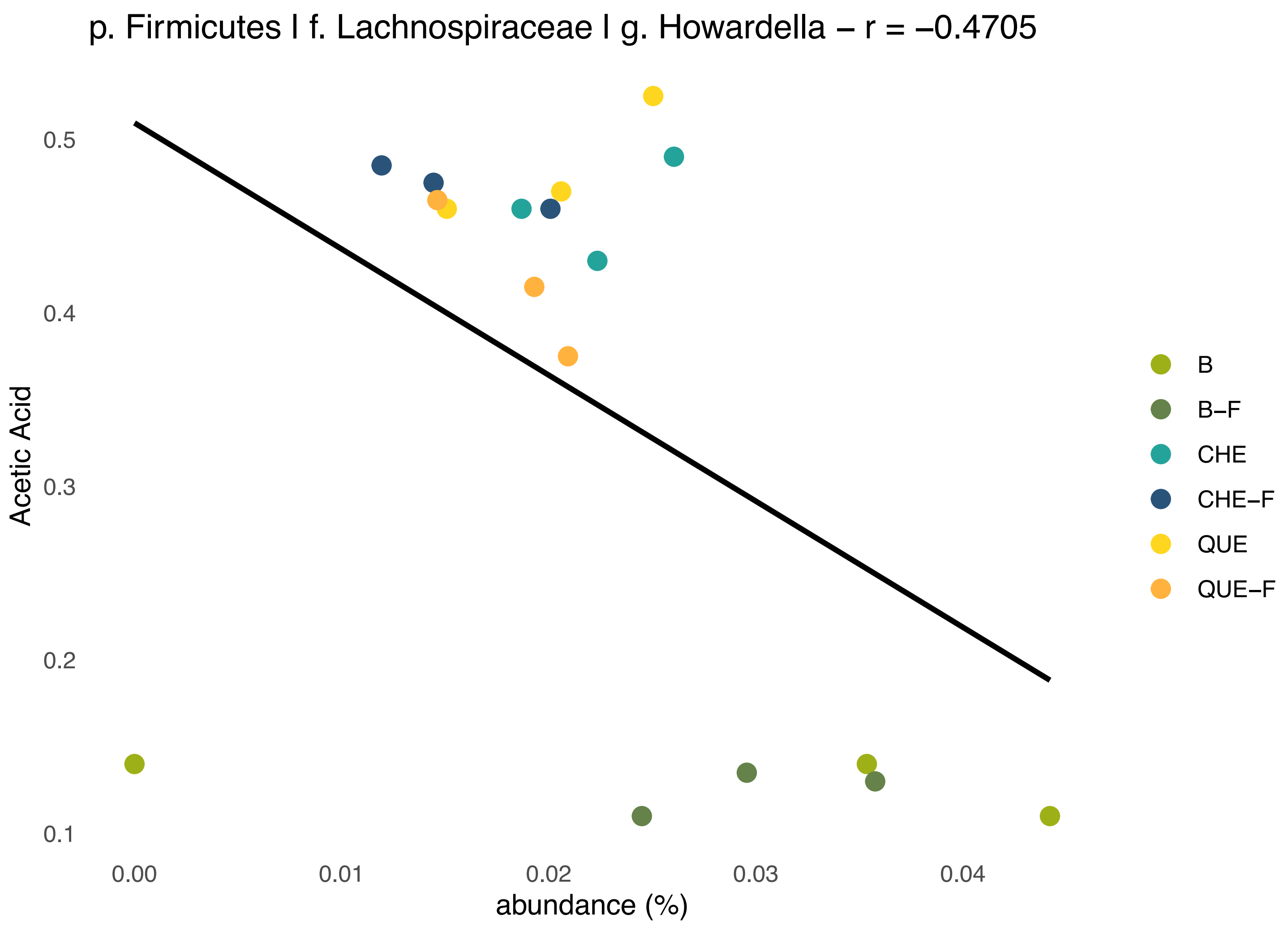
p. Firmicutes I f. Lachnospiraceae I g. Howardella – r = –0.4705
Acetic Acid
0.5
0.4
0.3
0.2
0.1
0.00
0.01
0.02
0.03
0.04
abundance (%)
B
B–F
CHE
CHE–F
QUE
QUE–F

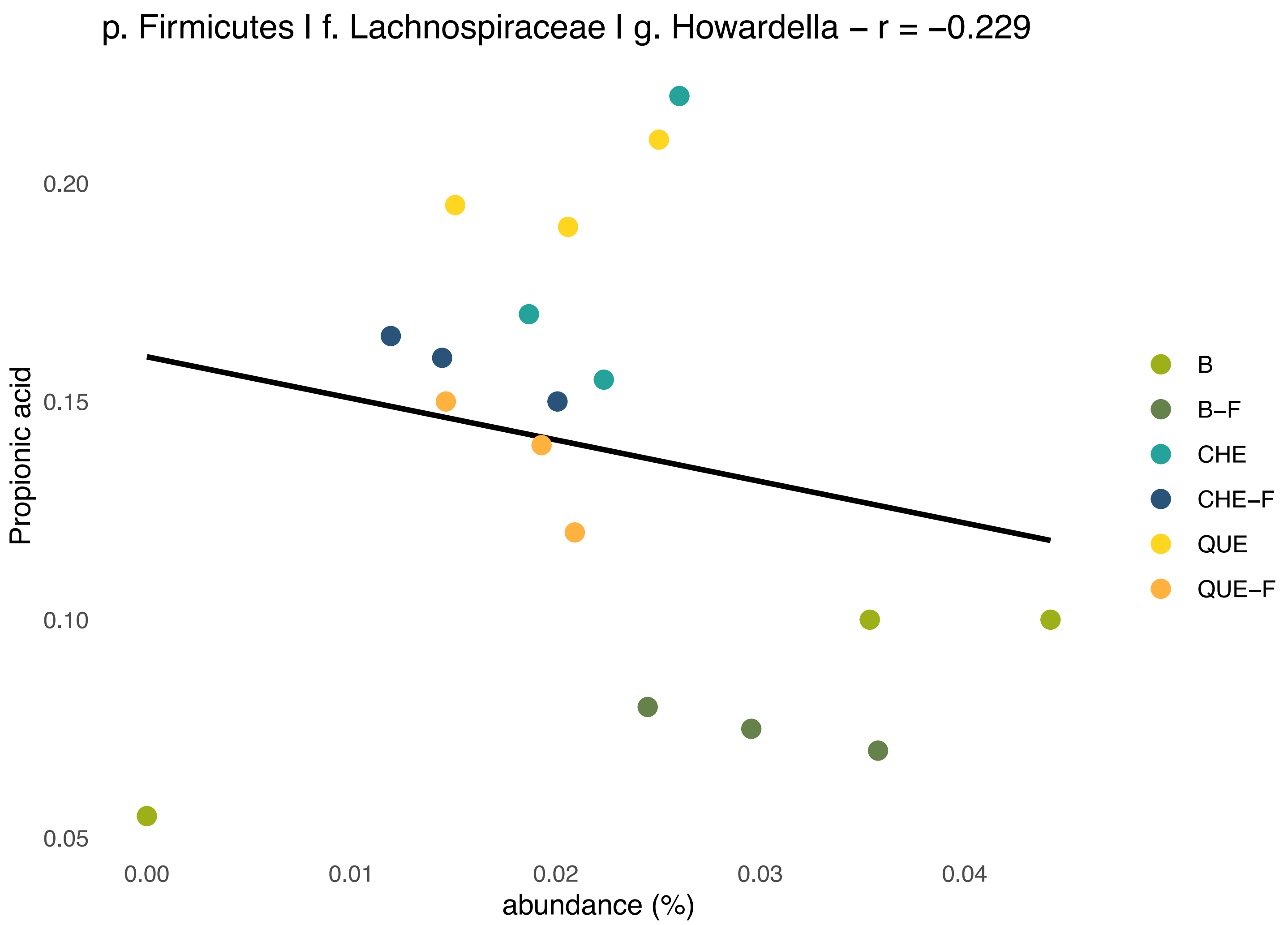
p. Firmicutes I f. Lachnospiraceae I g. Howardella – r = –0.229
Propionic acid
0.20
0.15
0.10
0.05
0.00
0.01
0.02
0.03
0.04
abundance (%)
B
B–F
CHE
CHE–F
QUE
QUE–F

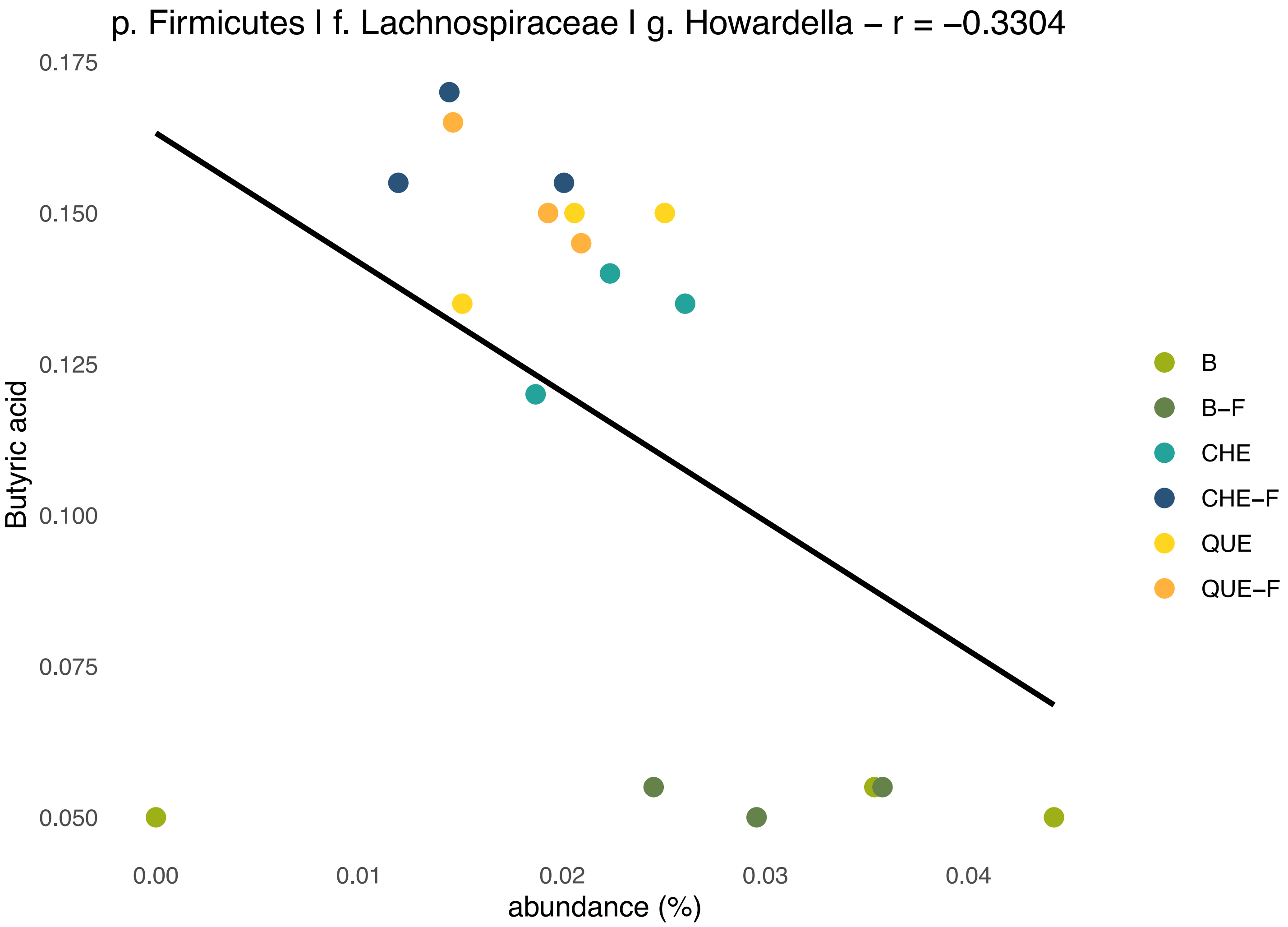

p. Firmicutes I f. Lachnospiraceae I g. Howardella – r = −0.3304
Butyric acid
0.175
0.150
0.125
0.100
0.075
0.050
0.00
0.01
0.02
0.03
0.04
abundance (%)
B
B−F
CHE
CHE−F
QUE
QUE−F

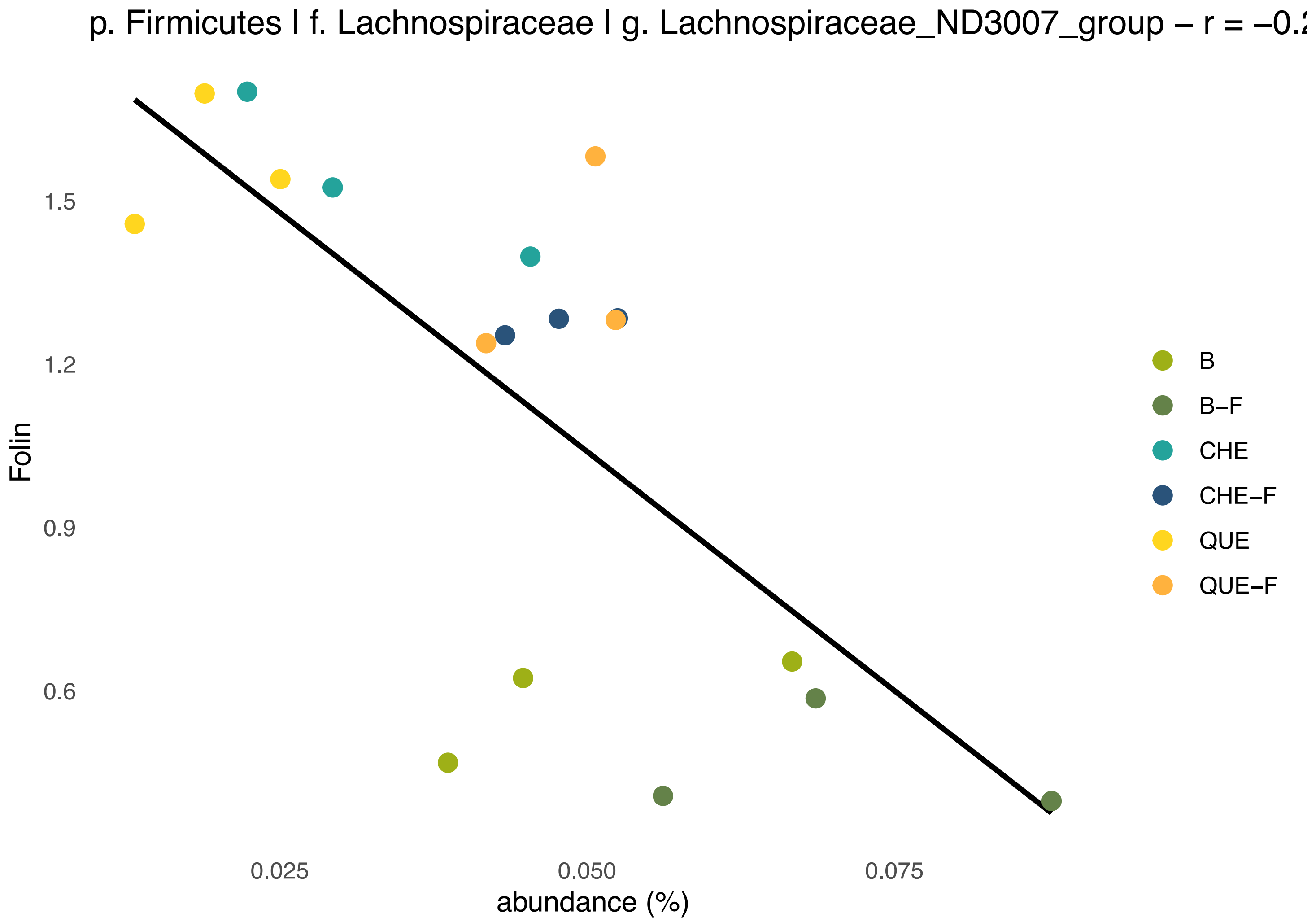

p. Firmicutes | f. Lachnospiraceae | g. Lachnospiraceae_ND3007_group – r = –0.
Folin
1.5
1.2
0.9
0.6
0.025
0.050
0.075
abundance (%)
B
B–F
CHE
CHE–F
QUE
QUE–F

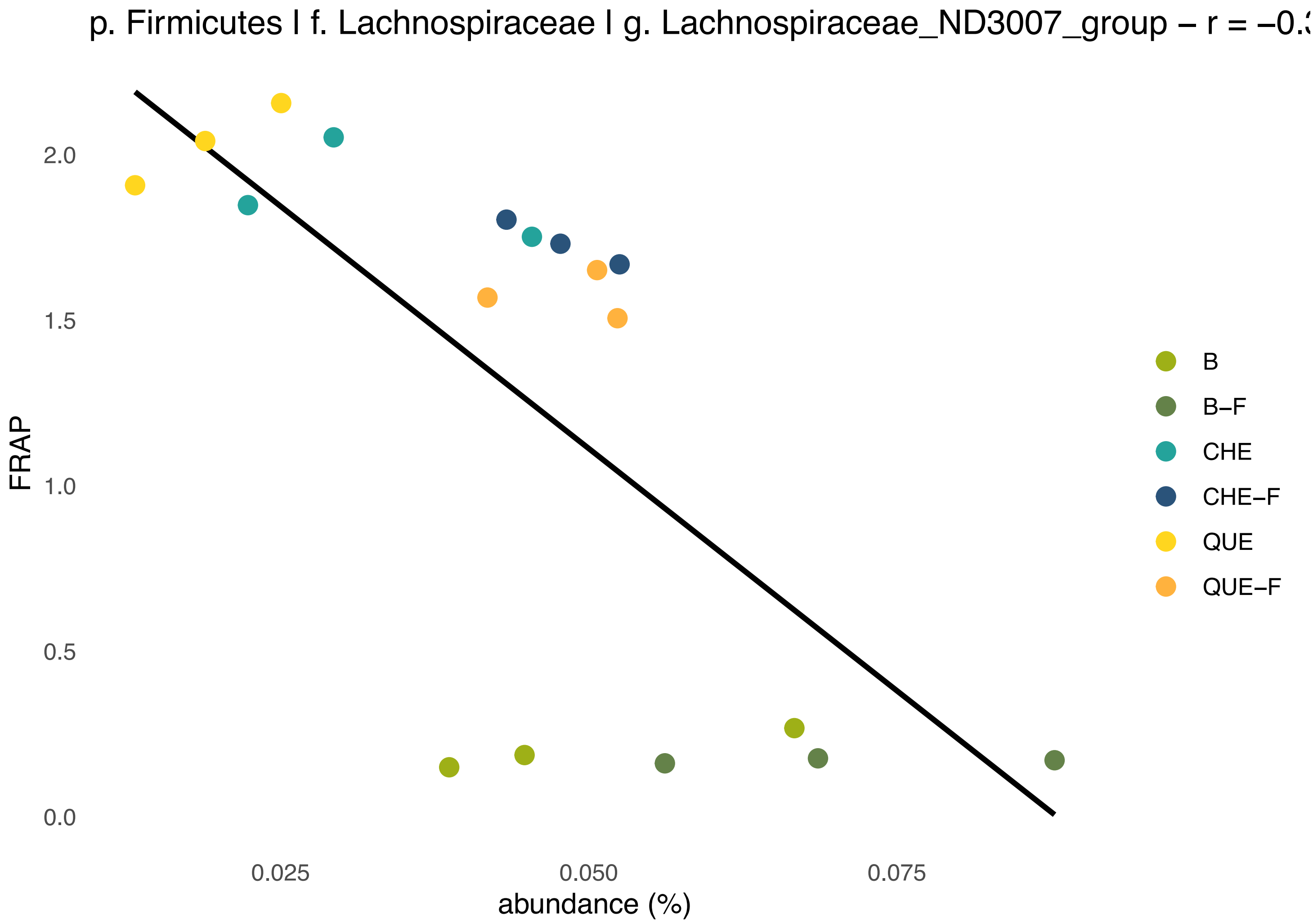
p. Firmicutes I f. Lachnospiraceae I g. Lachnospiraceae_ND3007_group – r = –0.
FRAP
2.0
1.5
1.0
0.5
0.0
0.025
0.050
0.075
abundance (%)
B
B–F
CHE
CHE–F
QUE
QUE–F

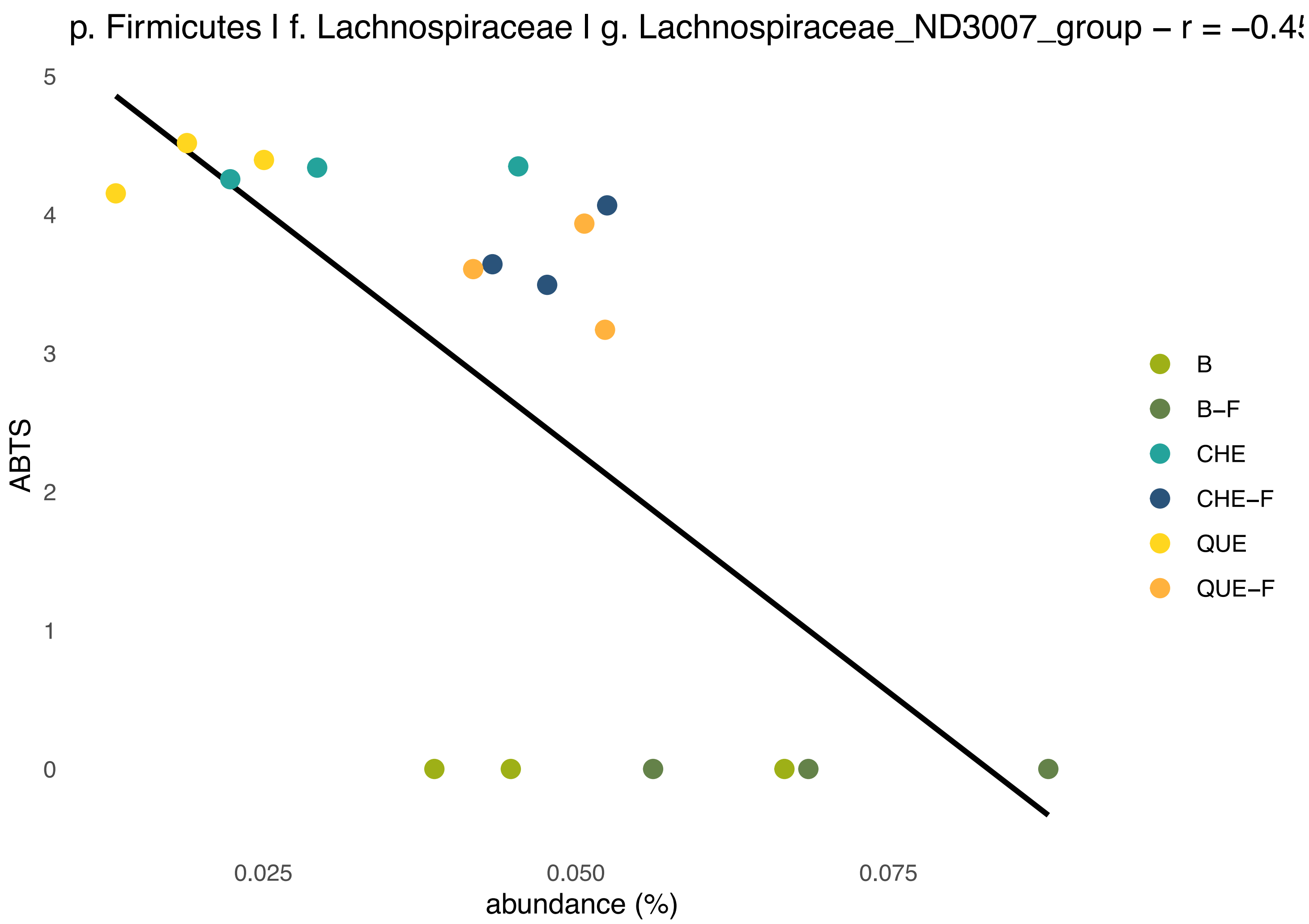
p. Firmicutes I f. Lachnospiraceae I g. Lachnospiraceae_ND3007_group – r = –0.4
ABTS
5
4
3
2
1
0
0.025
0.050
0.075
abundance (%)
B
B–F
CHE
CHE–F
QUE
QUE–F

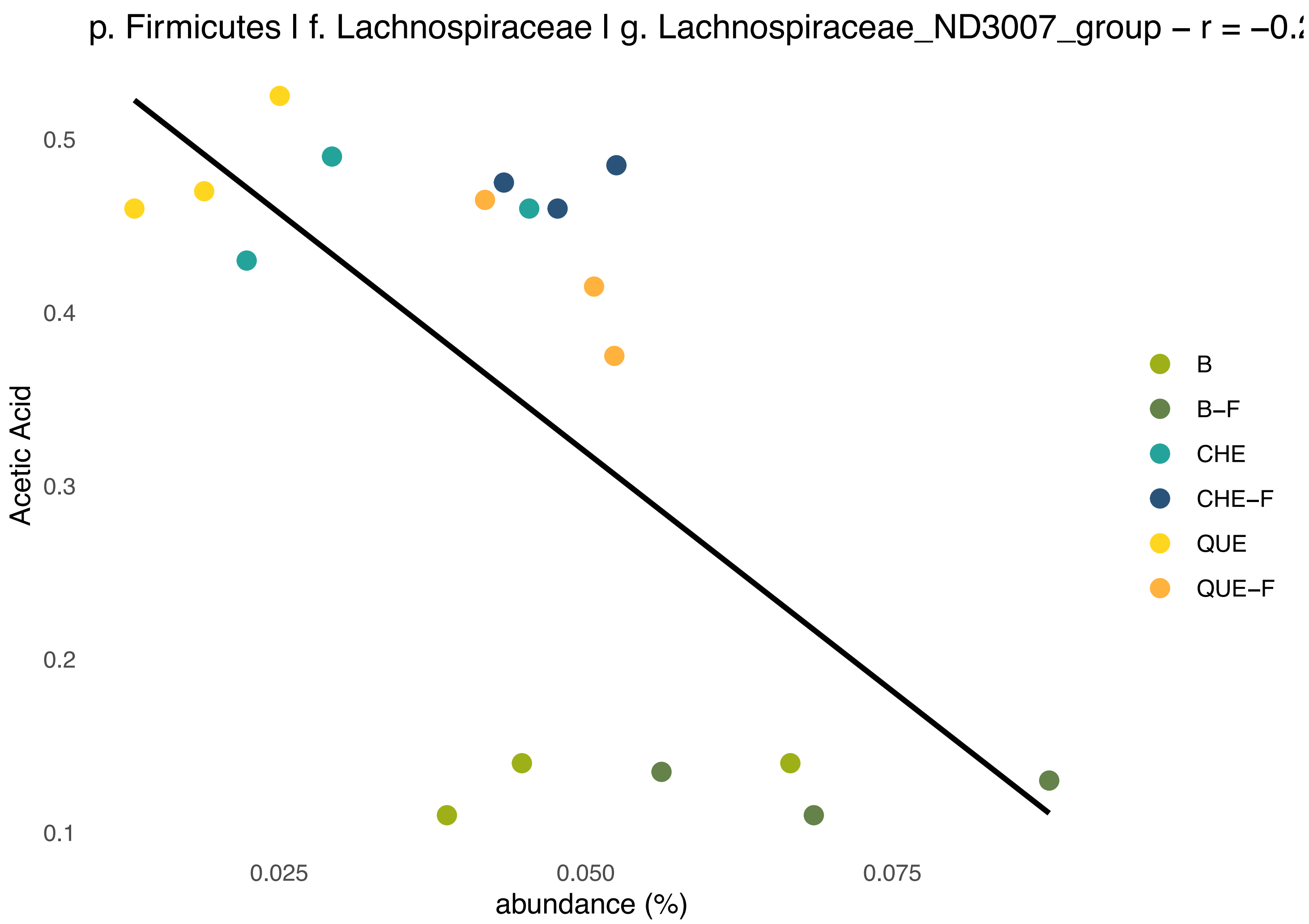
p. Firmicutes I f. Lachnospiraceae I g. Lachnospiraceae_ND3007_group – r = –0.
0.5
0.4
0.3
0.2
0.1
Acetic Acid
0.025
0.050
0.075
abundance (%)
B
B–F
CHE
CHE–F
QUE
QUE–F

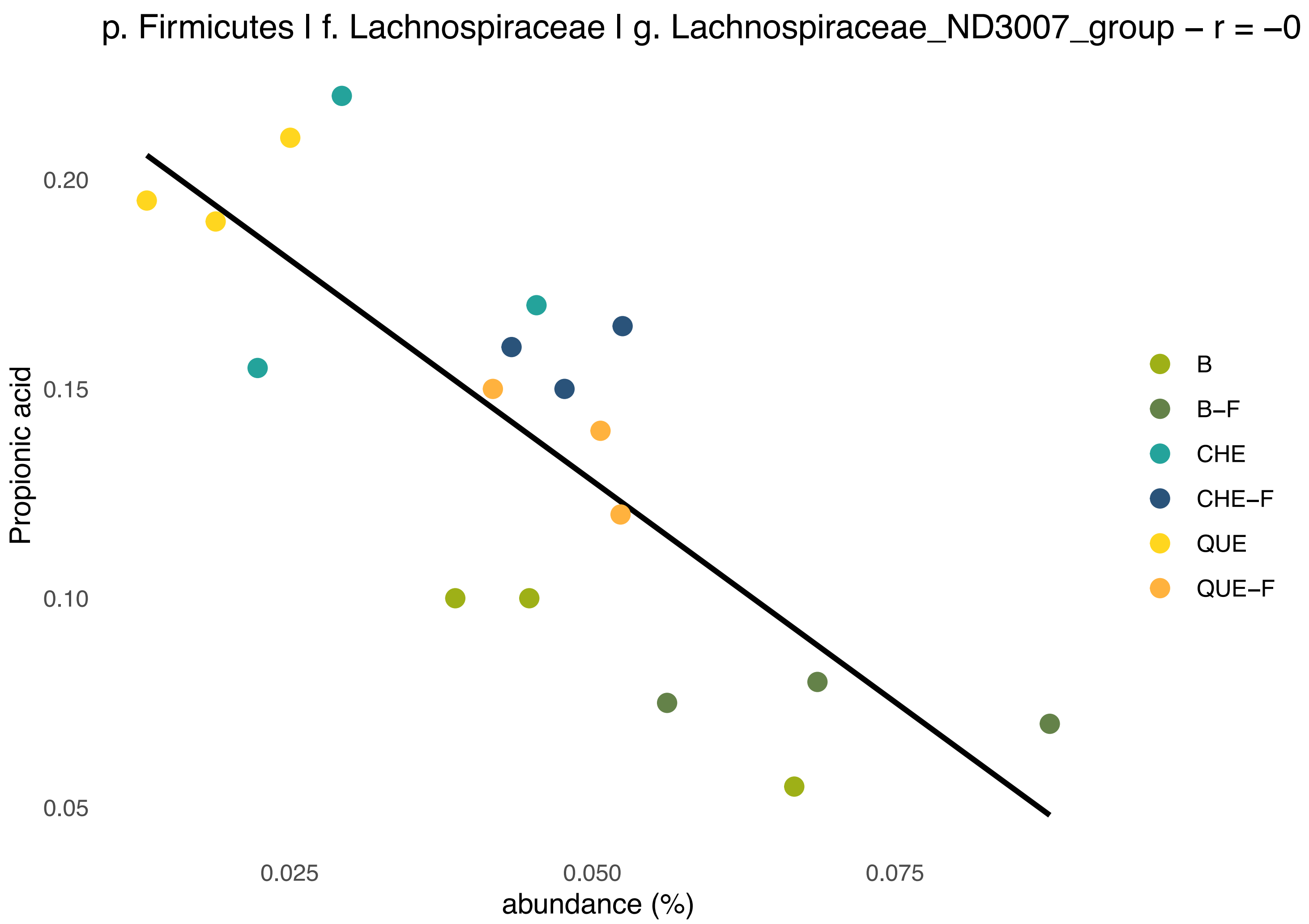

p. Firmicutes l f. Lachnospiraceae l g. Lachnospiraceae_ND3007_group – r = –0
Propionic acid
0.20
0.15
0.10
0.05
0.025
0.050
0.075
abundance (%)
B
B–F
CHE
CHE–F
QUE
QUE–F

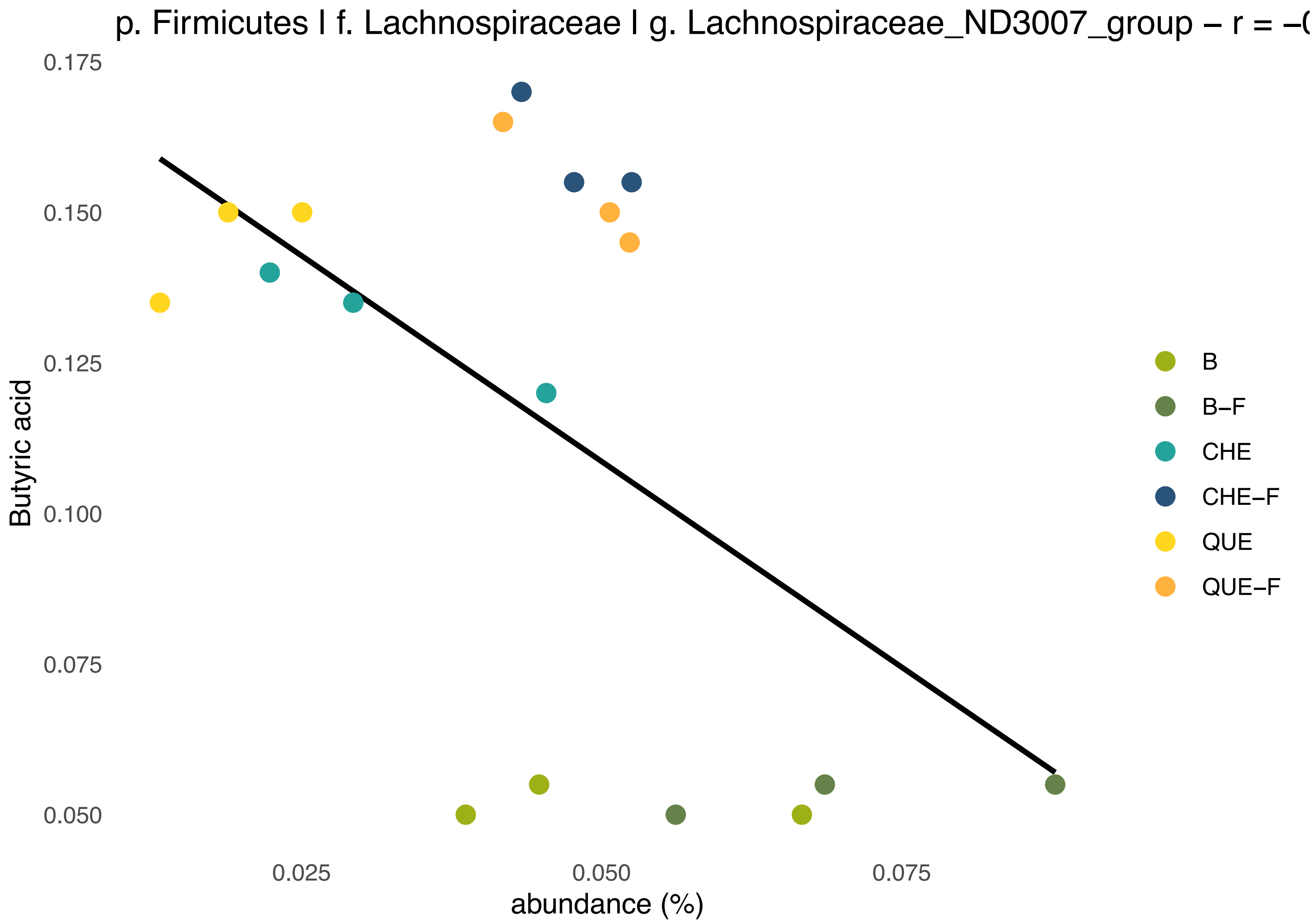

p. Firmicutes I f. Lachnospiraceae I g. Lachnospiraceae_ND3007_group – r = –(
Butyric acid
0.175
0.150
0.125
0.100
0.075
0.050
0.025
0.050
0.075
abundance (%)
B
B–F
CHE
CHE–F
QUE
QUE–F

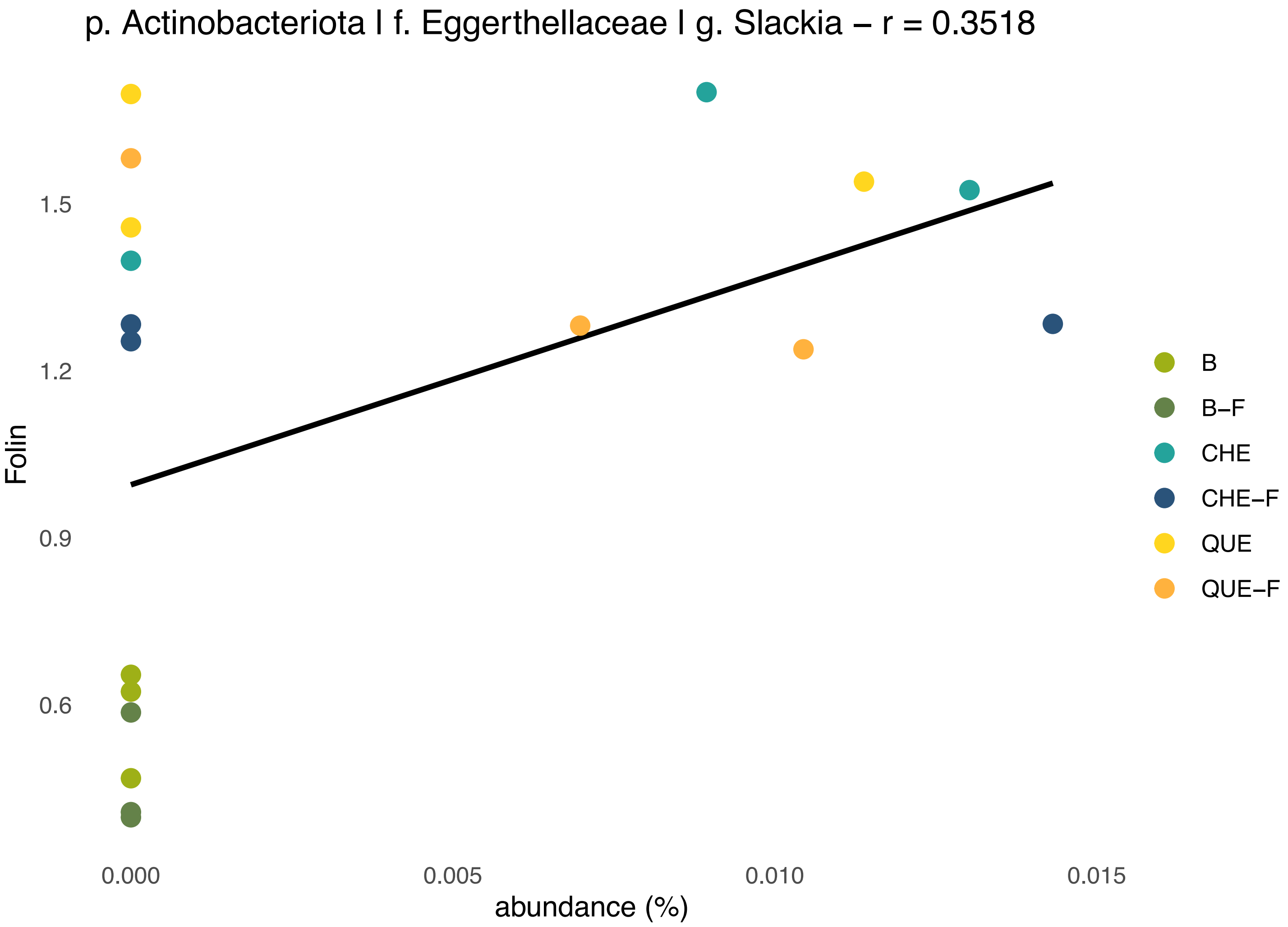

p. Actinobacteriota I f. Eggerthellaceae I g. Slackia – r = 0.3518
1.5
1.2
0.9
0.6
Folin
0.000
0.005
0.010
0.015
abundance (%)
B
B–F
CHE
CHE–F
QUE
QUE–F

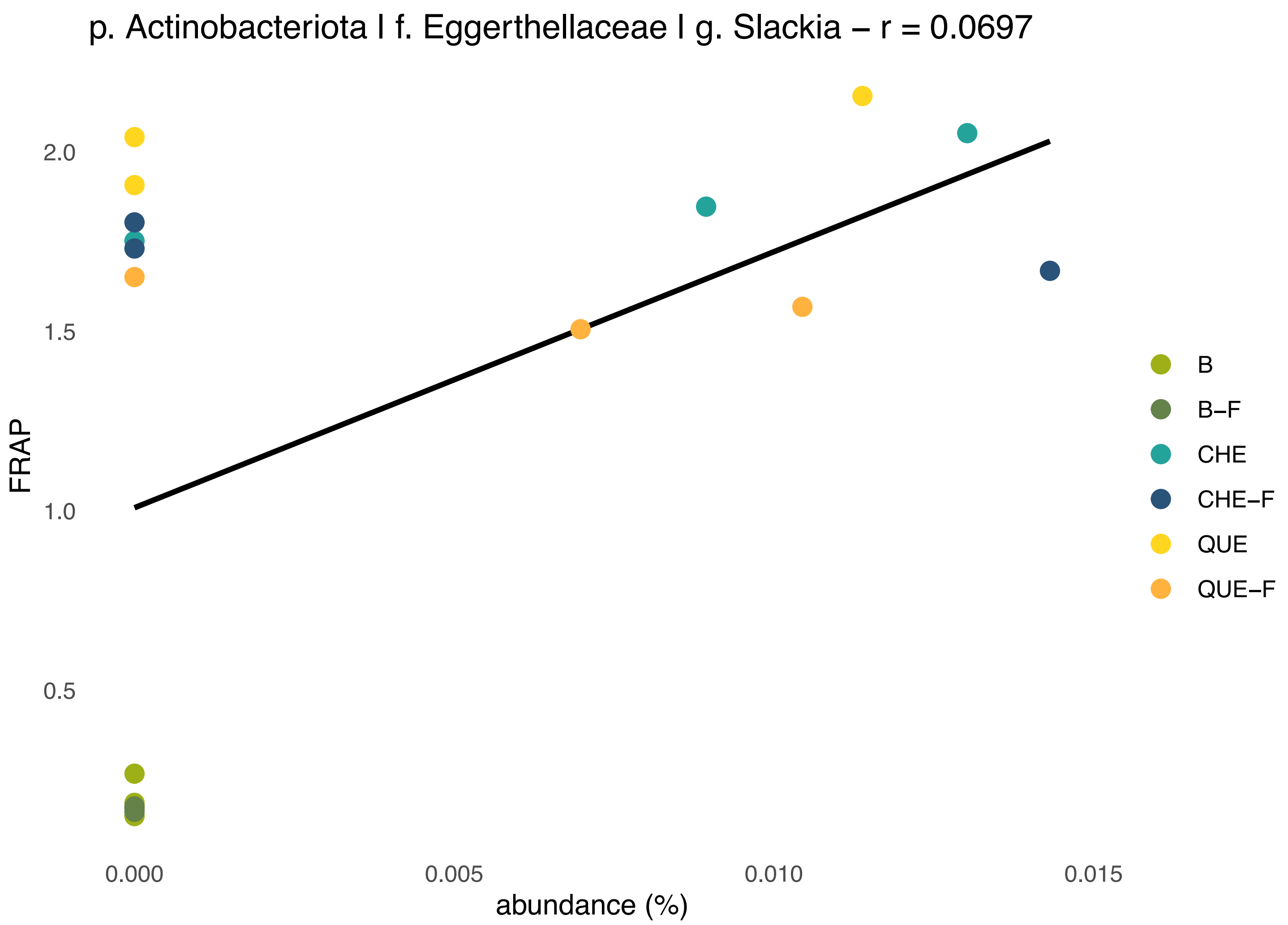
p. Actinobacteriota I f. Eggerthellaceae I g. Slackia – r = 0.0697
FRAP
2.0
1.5
1.0
0.5
0.000
0.005
0.010
0.015
abundance (%)
B
B–F
CHE
CHE–F
QUE
QUE–F

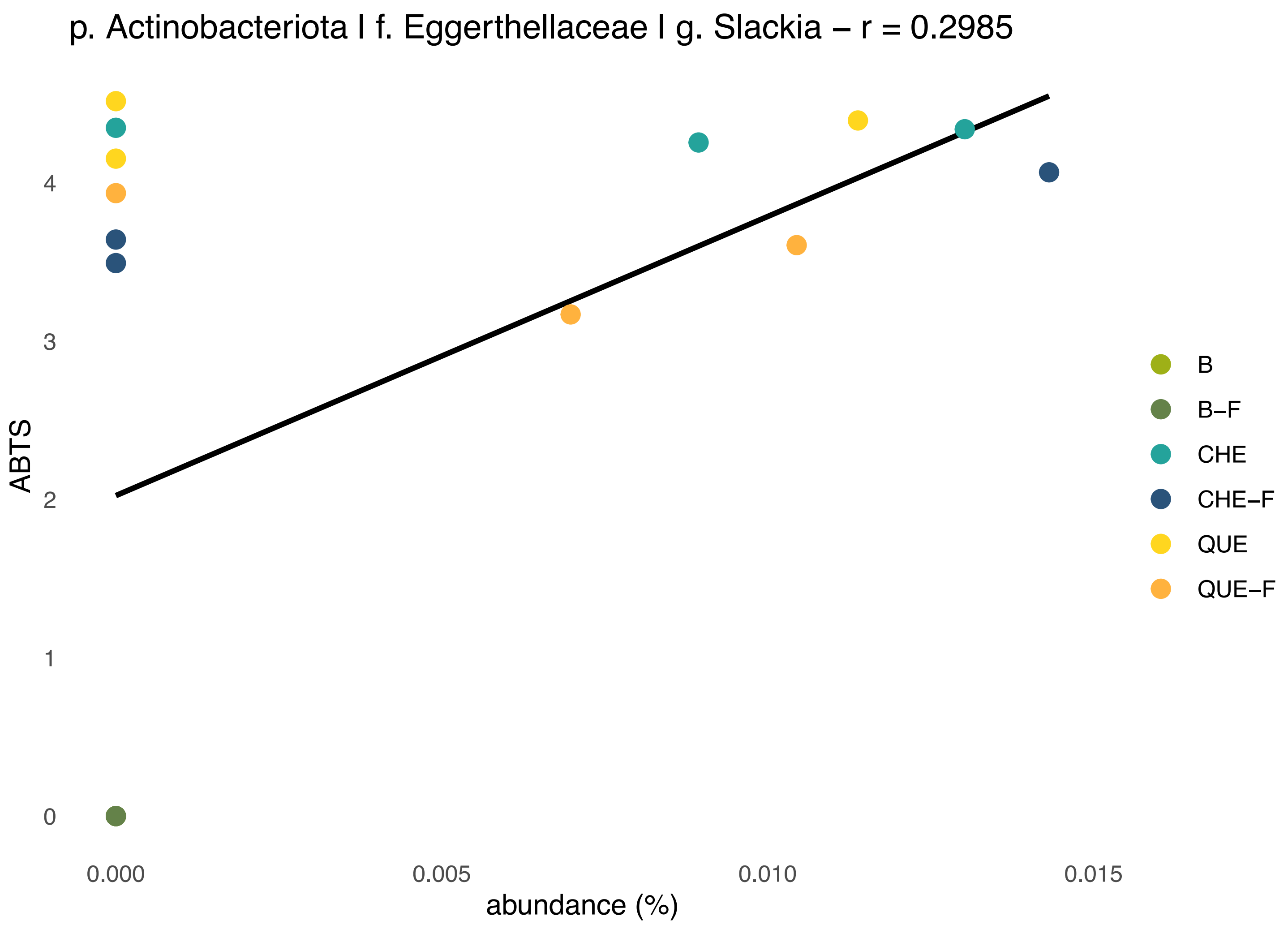

p. Actinobacteriota I f. Eggerthellaceae I g. Slackia – r = 0.2985
ABTS
0
1
2
3
4
0.000
0.005
0.010
0.015
abundance (%)
B
B–F
CHE
CHE–F
QUE
QUE–F

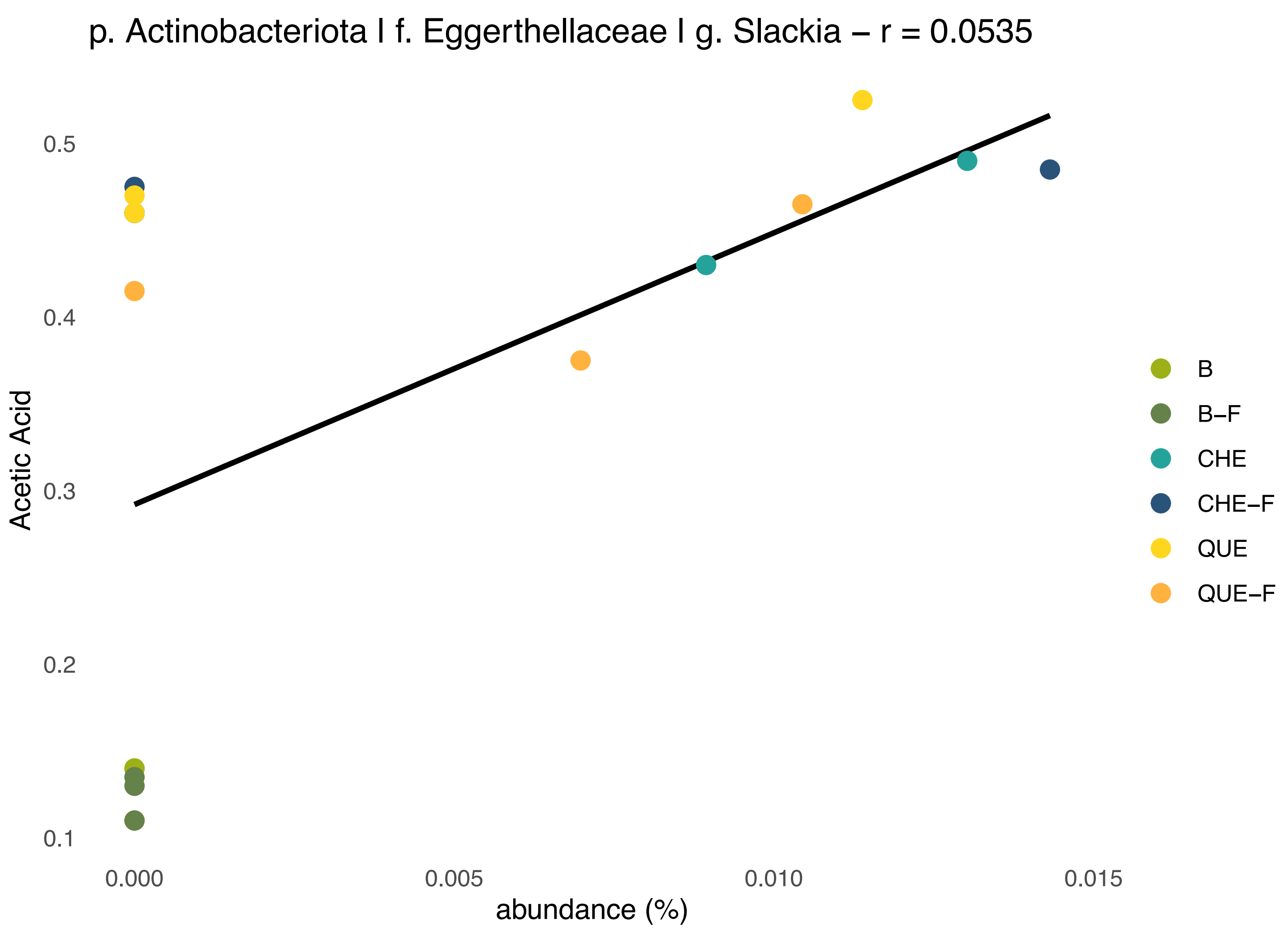
p. Actinobacteriota I f. Eggerthellaceae I g. Slackia – r = 0.0535
Acetic Acid
0.5
0.4
0.3
0.2
0.1
0.000
0.005
0.010
0.015
abundance (%)
B
B–F
CHE
CHE–F
QUE
QUE–F

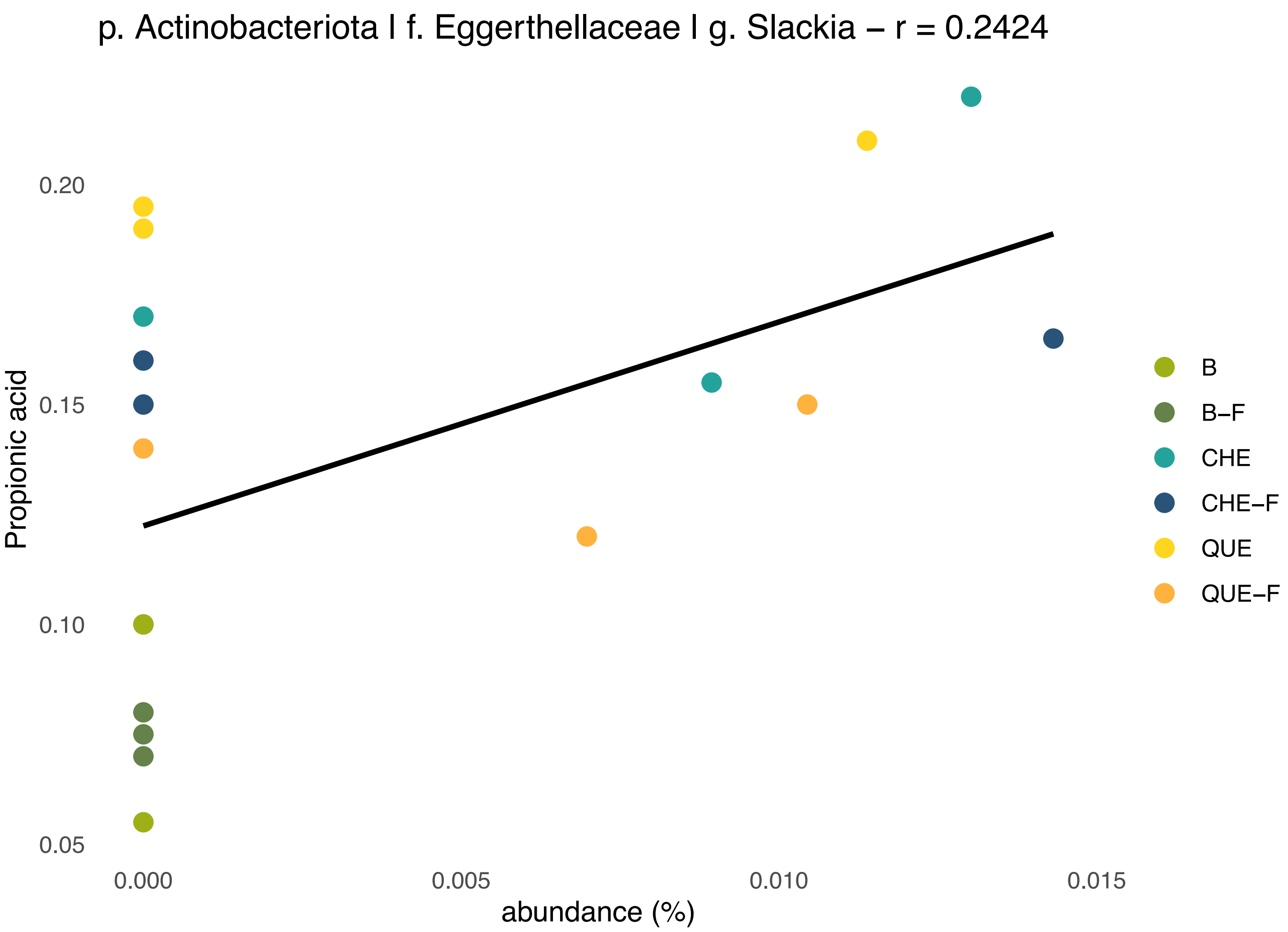
p. Actinobacteriota I f. Eggerthellaceae I g. Slackia – r = 0.2424
Propionic acid
0.20
0.15
0.10
0.05
0.000
0.005
0.010
0.015
abundance (%)
B
B–F
CHE
CHE–F
QUE
QUE–F

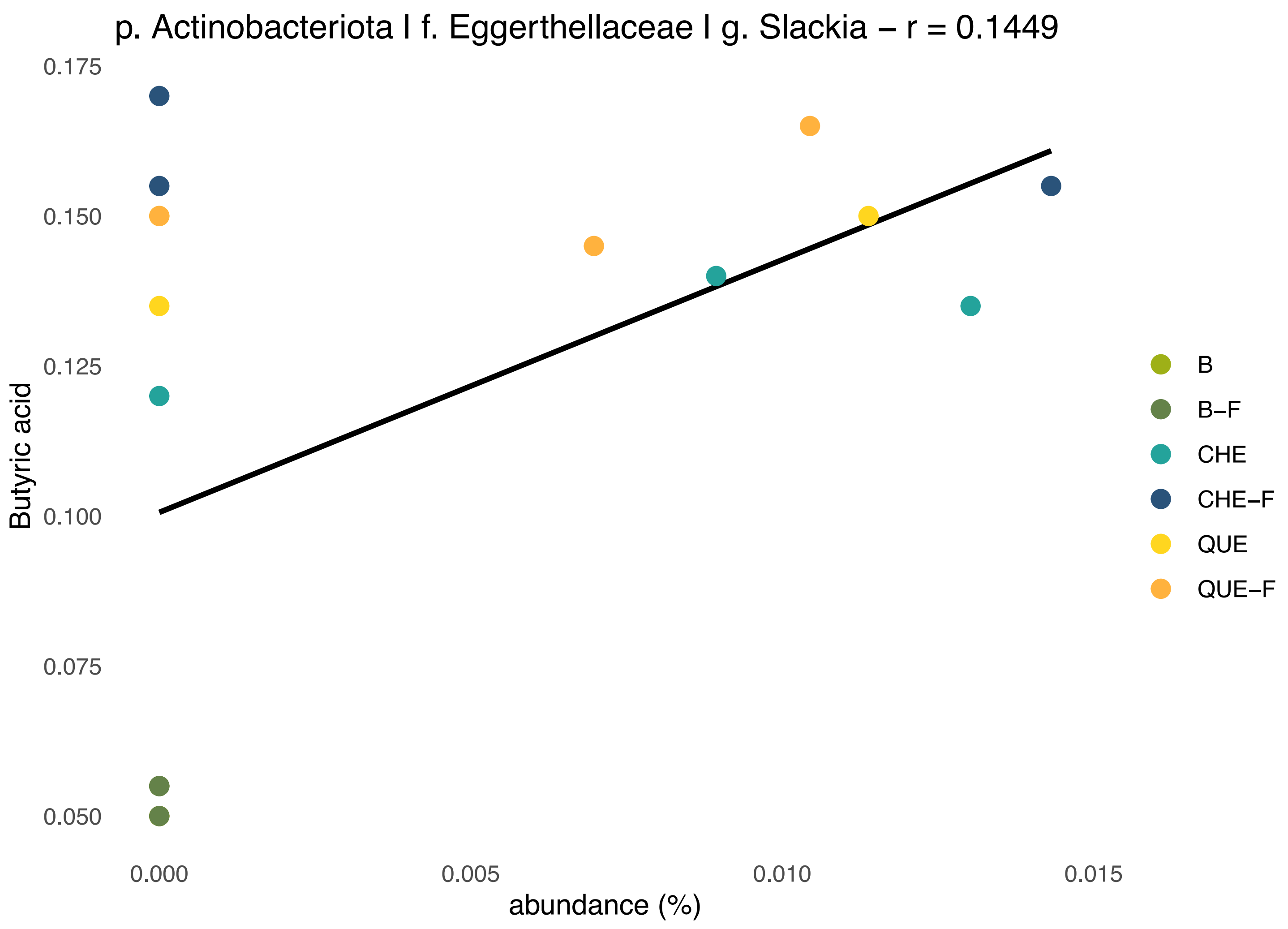
p. Actinobacteriota I f. Eggerthellaceae I g. Slackia – r = 0.1449
Butyric acid
0.175
0.150
0.125
0.100
0.075
0.050
0.000
0.005
0.010
0.015
abundance (%)
B
B–F
CHE
CHE–F
QUE
QUE–F

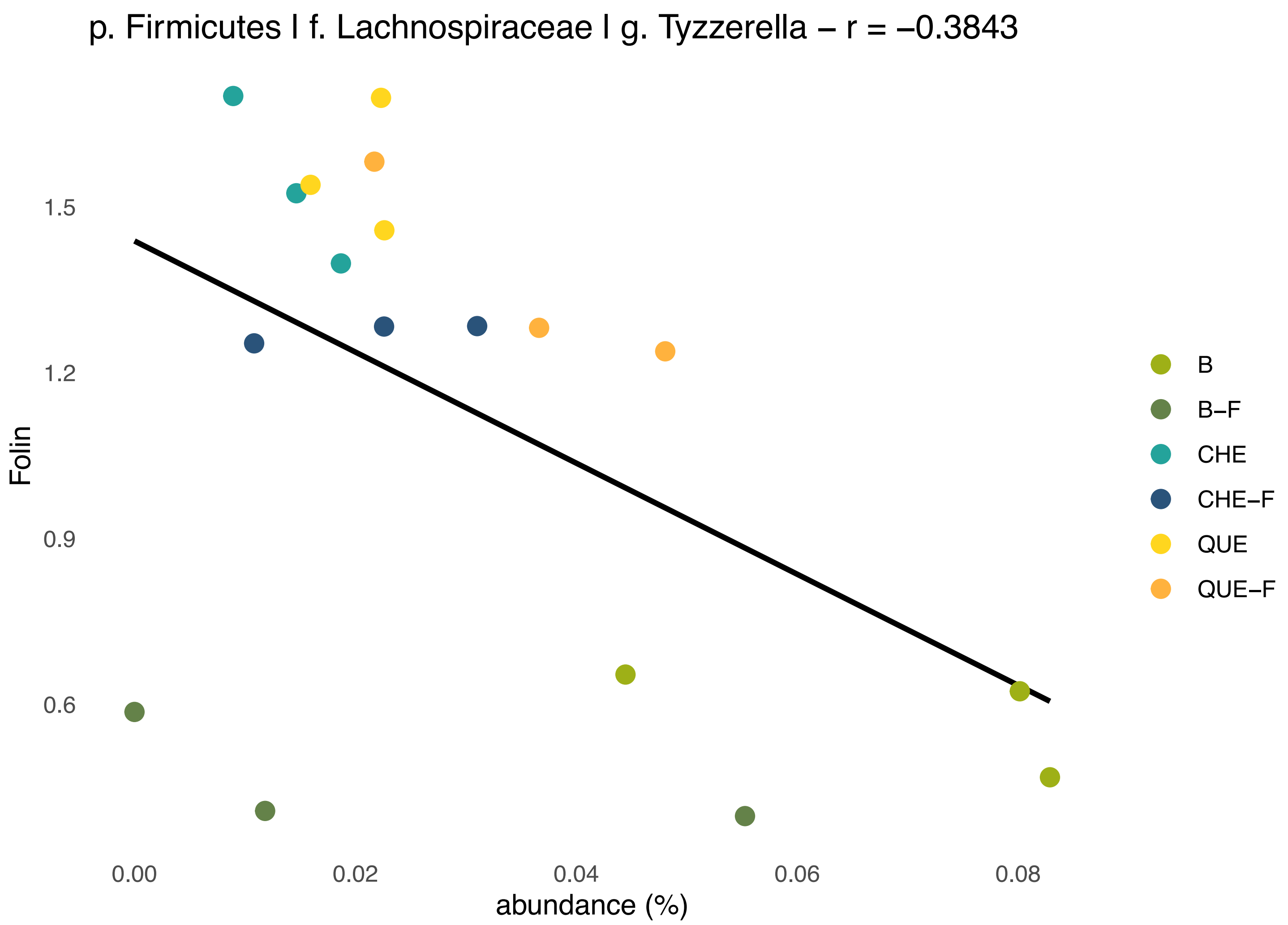
p. Firmicutes I f. Lachnospiraceae I g. Tyzzerella – r = –0.3843
Folin
1.5
1.2
0.9
0.6
0.00
0.02
0.04
0.06
0.08
abundance (%)
B
B–F
CHE
CHE–F
QUE
QUE–F

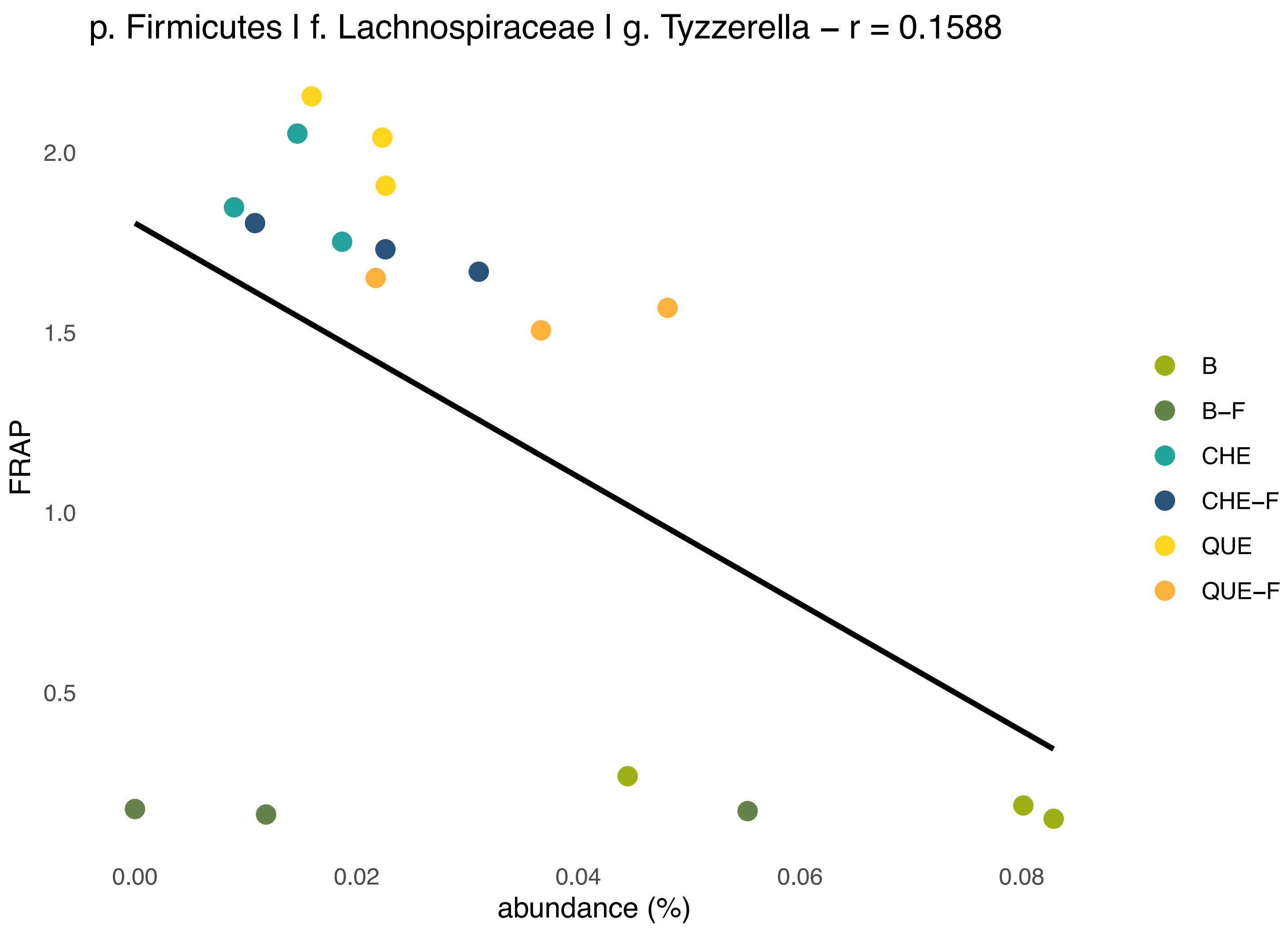
p. Firmicutes I f. Lachnospiraceae I g. Tyzzerella – r = 0.1588
FRAP
2.0
1.5
1.0
0.5
0.00
0.02
0.04
0.06
0.08
abundance (%)
B
B–F
CHE
CHE–F
QUE
QUE–F

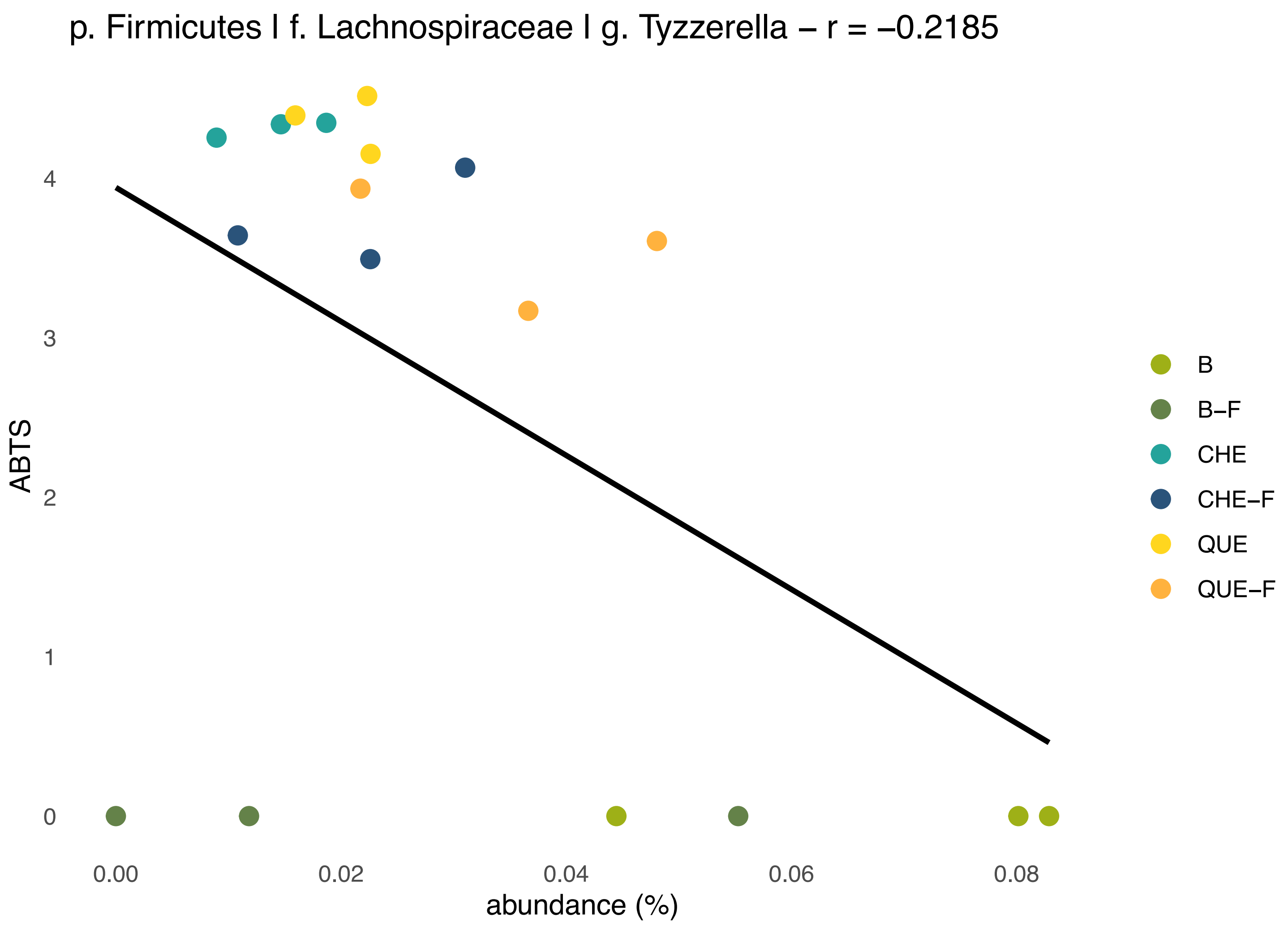
p. Firmicutes I f. Lachnospiraceae I g. Tyzzerella – r = –0.2185
ABTS
4
3
2
1
0
0.00
0.02
0.04
0.06
0.08
abundance (%)
B
B–F
CHE
CHE–F
QUE
QUE–F

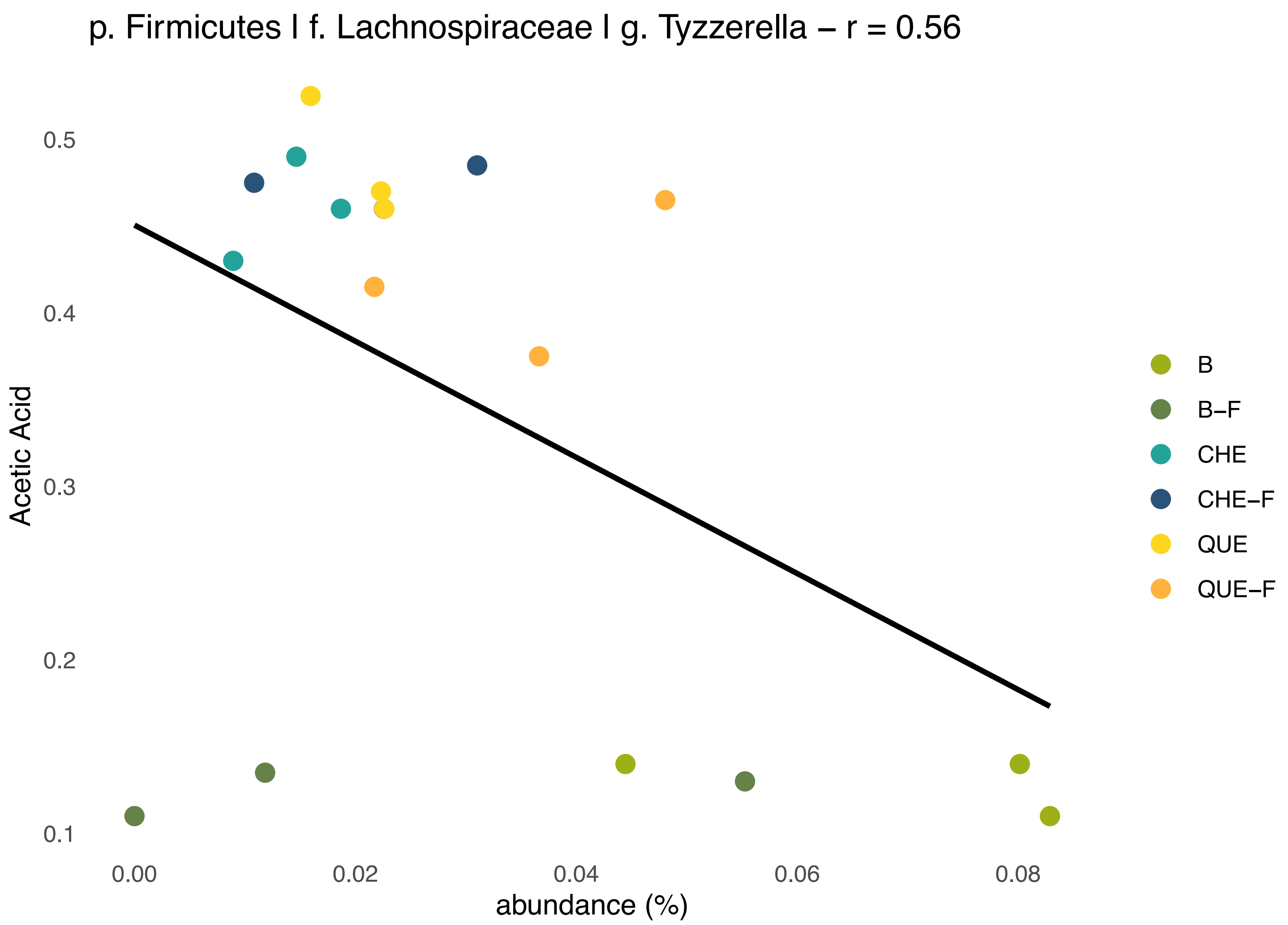

p. Firmicutes I f. Lachnospiraceae I g. Tyzzerella – r = 0.56
Acetic Acid
0.5
0.4
0.3
0.2
0.1
0.00
0.02
0.04
0.06
0.08
abundance (%)
B
B–F
CHE
CHE–F
QUE
QUE–F

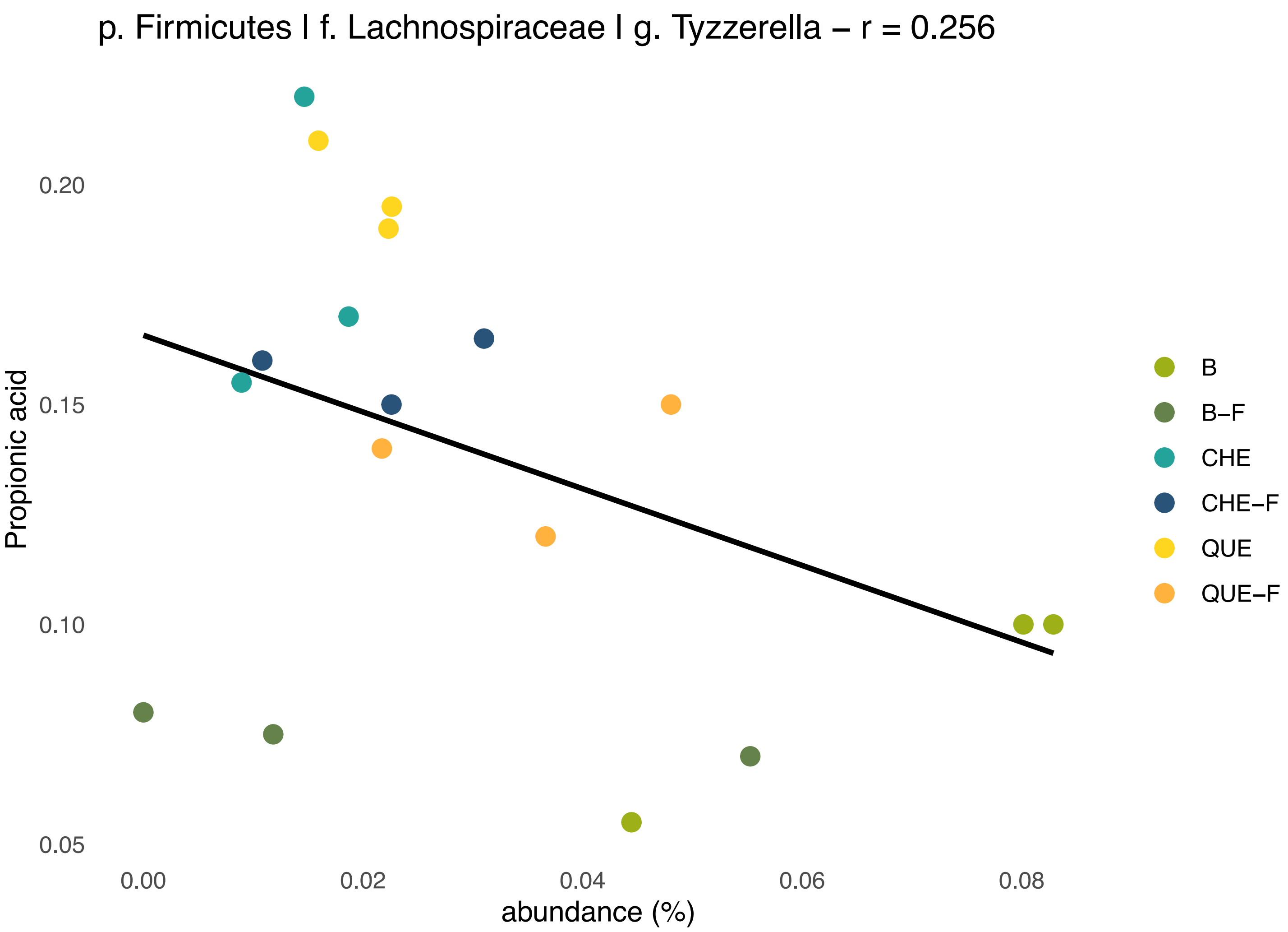
p. Firmicutes I f. Lachnospiraceae I g. Tyzzerella – r = 0.256
Propionic acid
0.20
0.15
0.10
0.05
0.00
0.02
0.04
0.06
0.08
abundance (%)
B
B–F
CHE
CHE–F
QUE
QUE–F

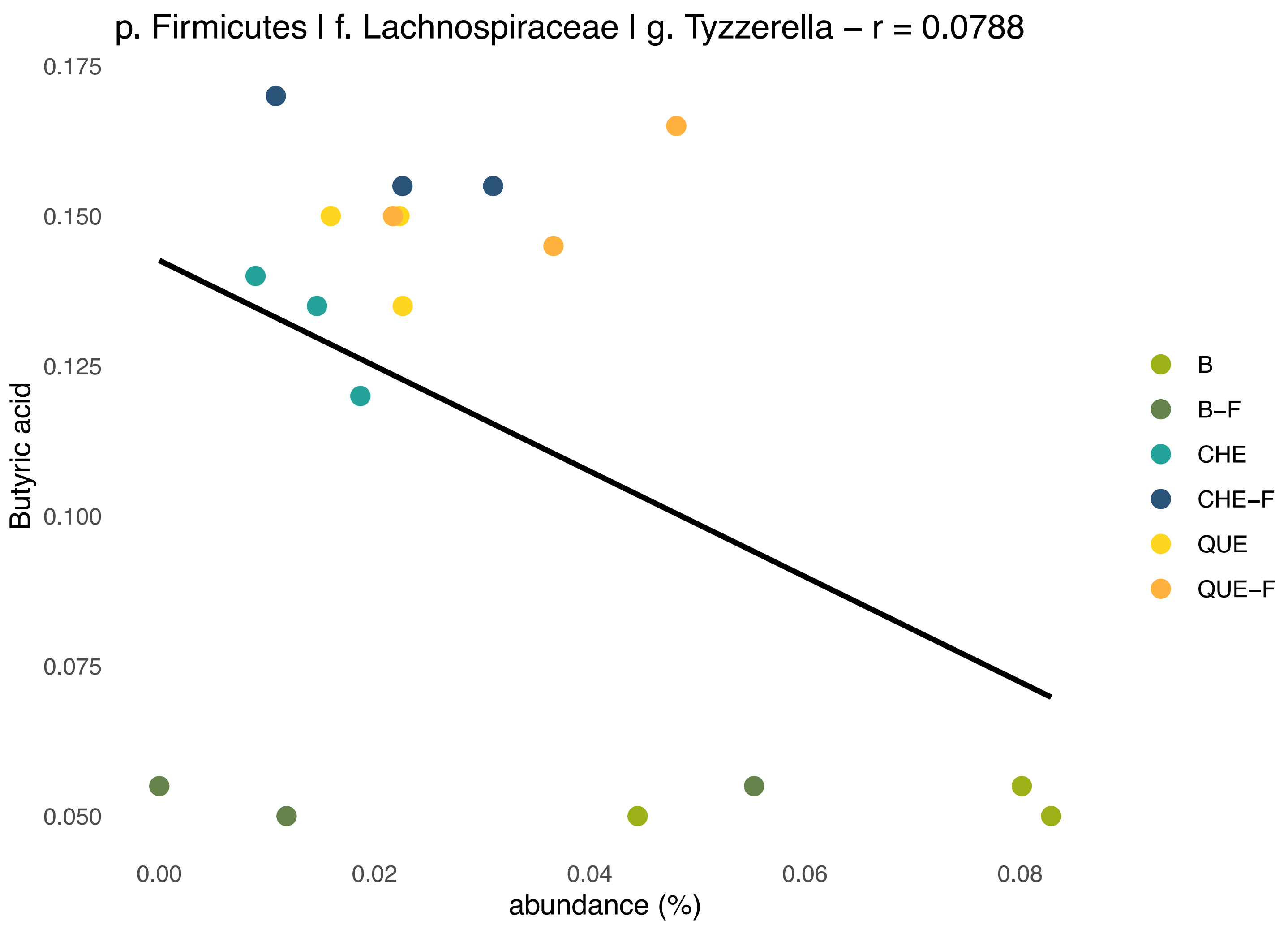

p. Firmicutes I f. Lachnospiraceae I g. Tyzzerella – r = 0.0788
0.175
0.150
0.125
0.100
0.075
0.050
Butyric acid
0.00
0.02
0.04
0.06
0.08
abundance (%)
B
B–F
CHE
CHE–F
QUE
QUE–F

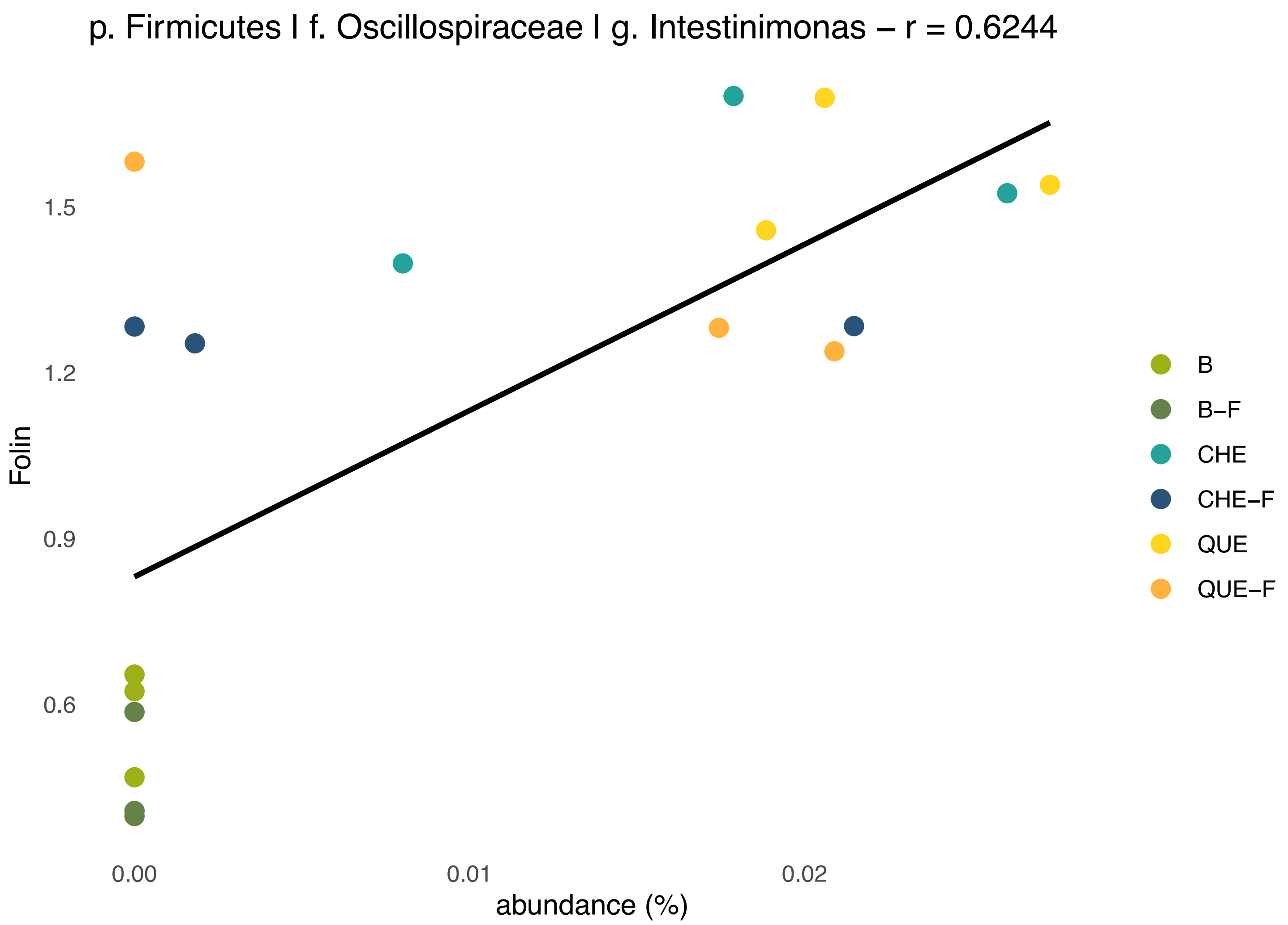
p. Firmicutes I f. Oscillospiraceae I g. Intestinimonas – r = 0.6244
Folin
1.5
1.2
0.9
0.6
0.00
0.01
0.02
abundance (%)
B
B–F
CHE
CHE–F
QUE
QUE–F

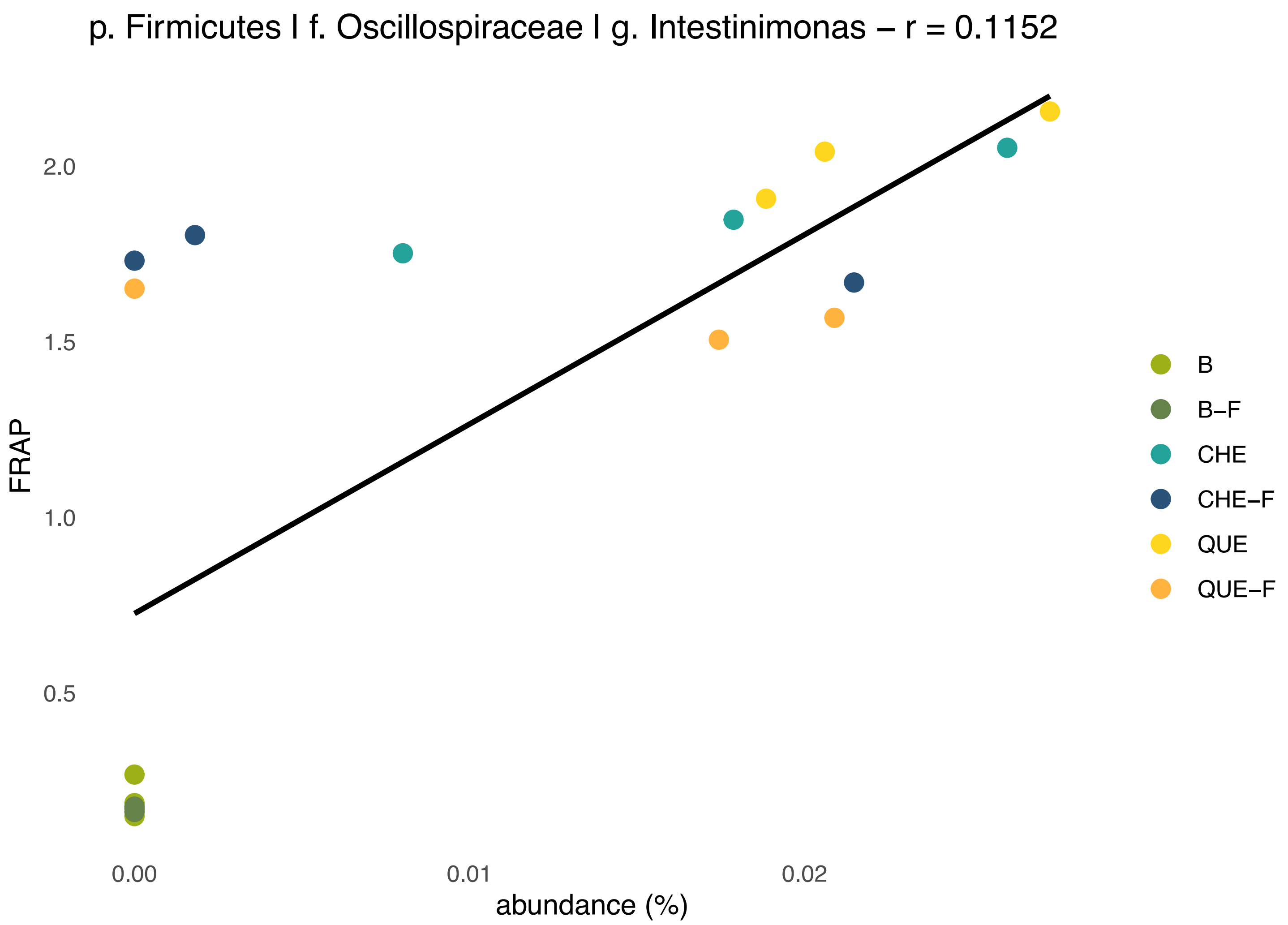

p. Firmicutes I f. Oscillospiraceae I g. Intestinimonas – r = 0.1152
FRAP
2.0
1.5
1.0
0.5
0.00
0.01
0.02
abundance (%)
B
B–F
CHE
CHE–F
QUE
QUE–F

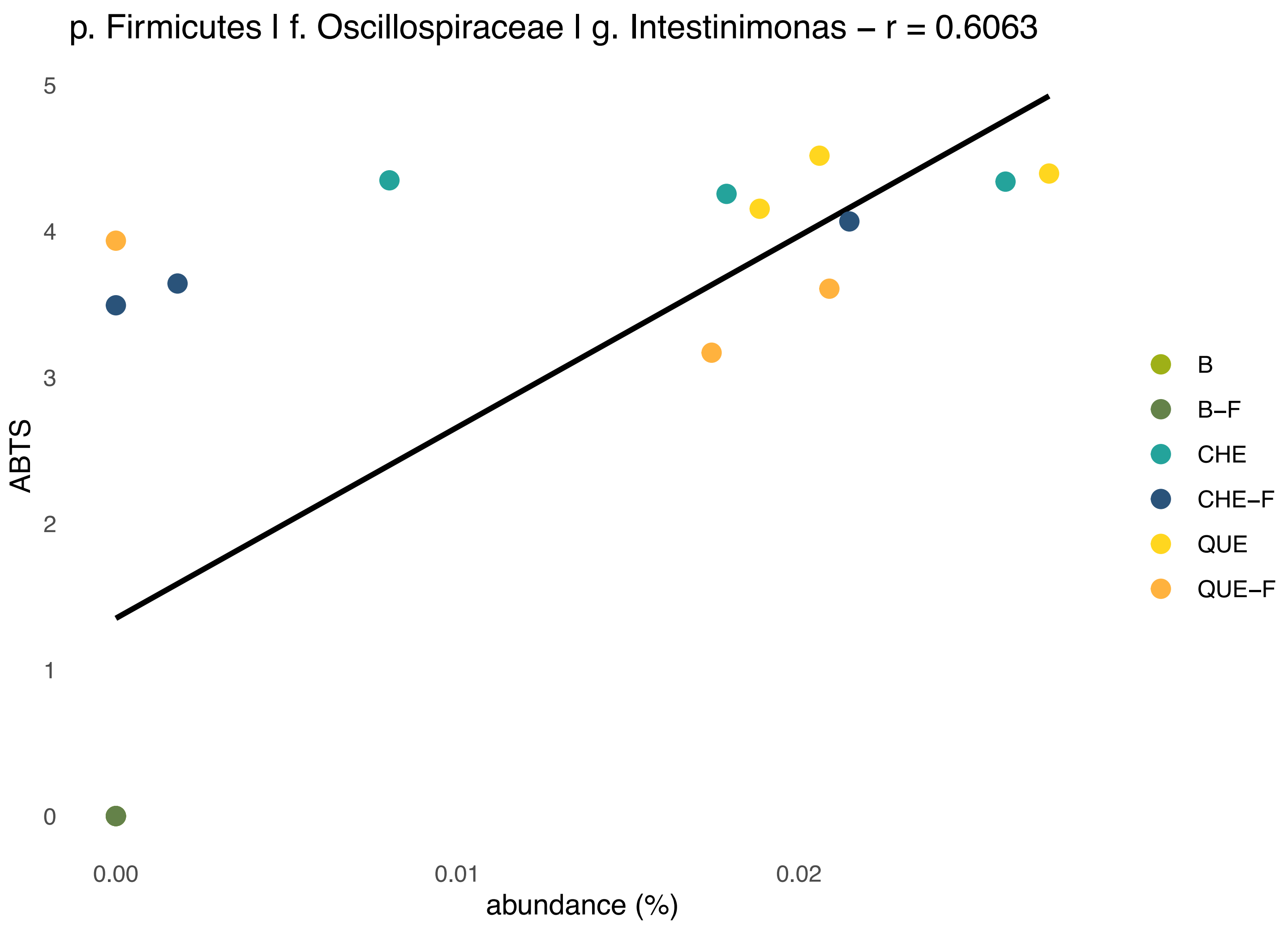

p. Firmicutes | f. Oscillospiraceae | g. Intestinimonas – r = 0.6063
ABTS
0
1
2
3
4
5
0.00
0.01
0.02
abundance (%)
B
B–F
CHE
CHE–F
QUE
QUE–F

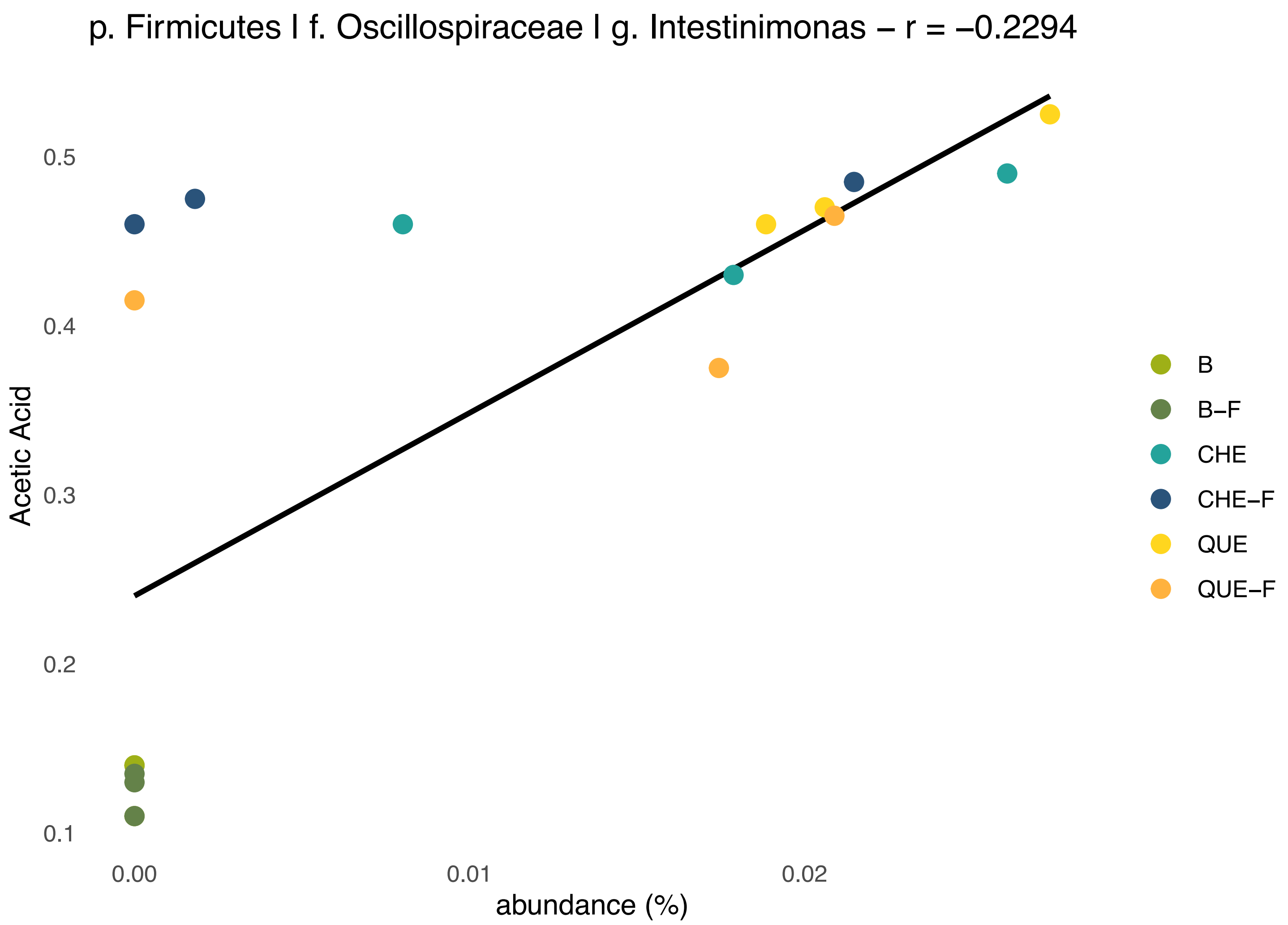
p. Firmicutes I f. Oscillospiraceae I g. Intestinimonas – r = −0.2294
0.5
0.4
0.3
0.2
0.1
Acetic Acid
0.00
0.01
0.02
abundance (%)
B
B−F
CHE
CHE−F
QUE
QUE−F

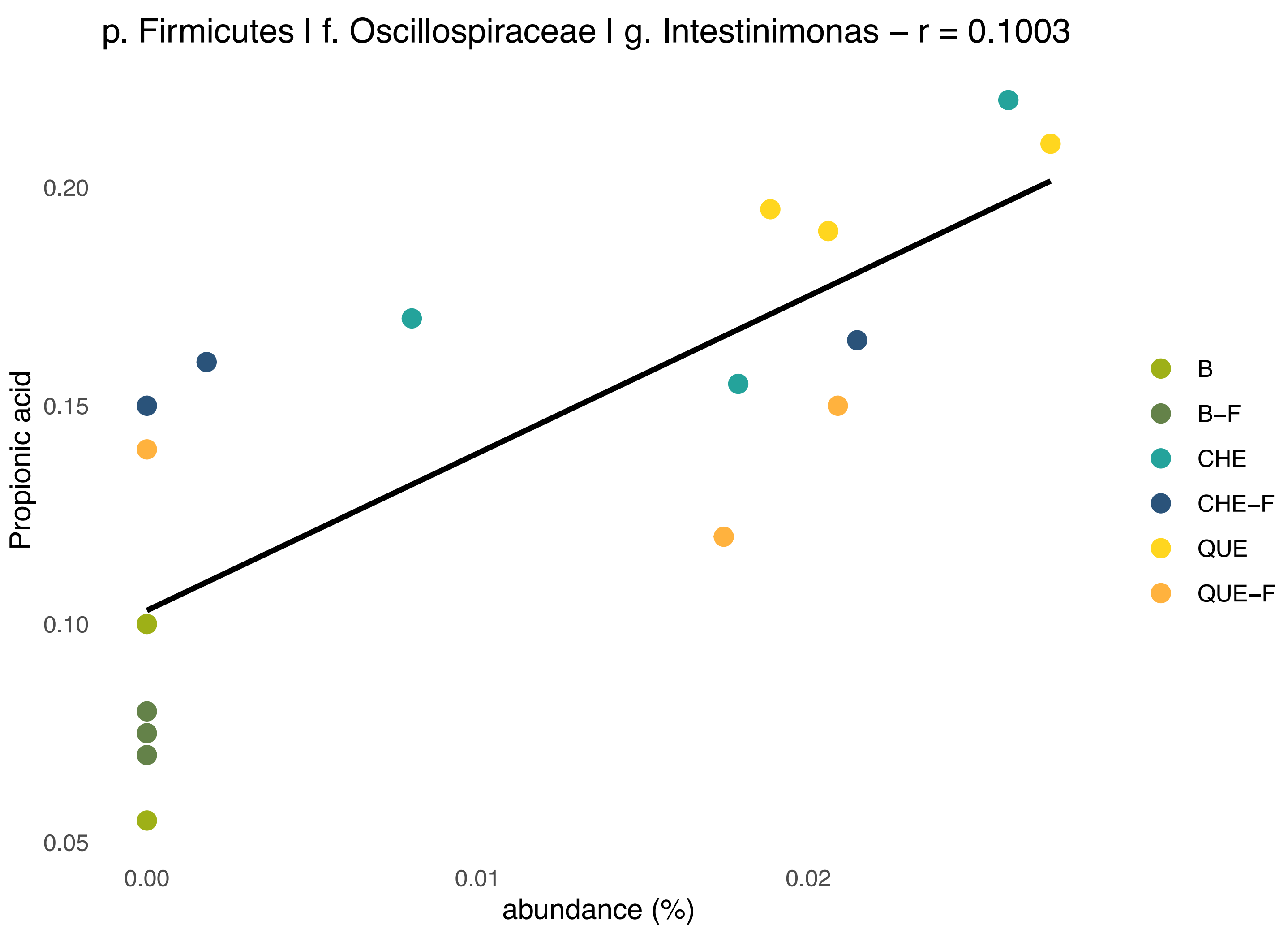

p. Firmicutes I f. Oscillospiraceae I g. Intestinimonas – r = 0.1003
Propionic acid
0.20
0.15
0.10
0.05
0.00
0.01
0.02
abundance (%)
B
B–F
CHE
CHE–F
QUE
QUE–F

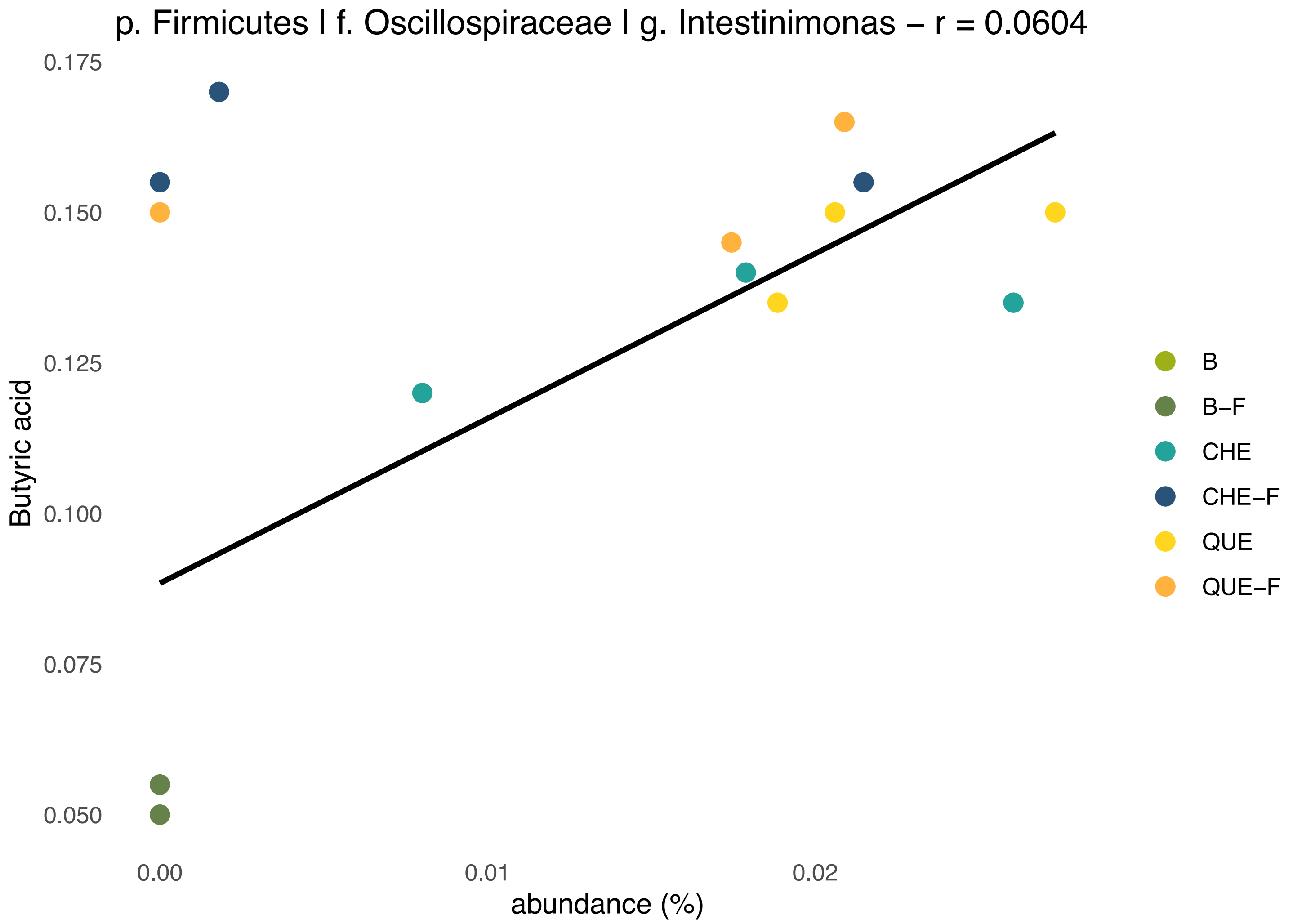

p. Firmicutes I f. Oscillospiraceae I g. Intestinimonas – r = 0.0604
Butyric acid
0.175
0.150
0.125
0.100
0.075
0.050
0.00
0.01
0.02
abundance (%)
B
B–F
CHE
CHE–F
QUE
QUE–F

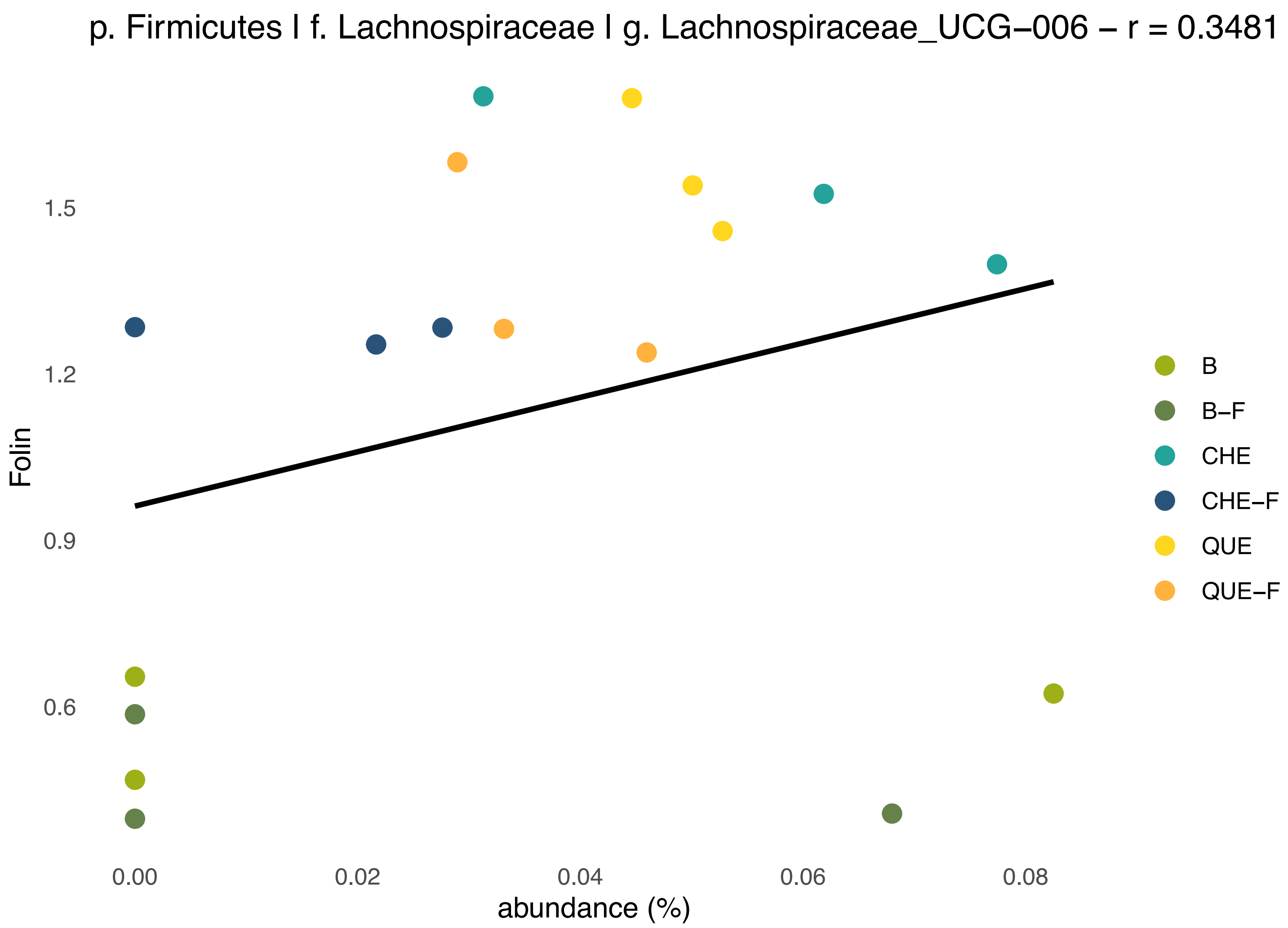

p. Firmicutes I f. Lachnospiraceae I g. Lachnospiraceae_UCG−006 − r = 0.3481
1.5
1.2
0.9
0.6
Folin
0.00
0.02
0.04
0.06
0.08
abundance (%)
B
B−F
CHE
CHE−F
QUE
QUE−F

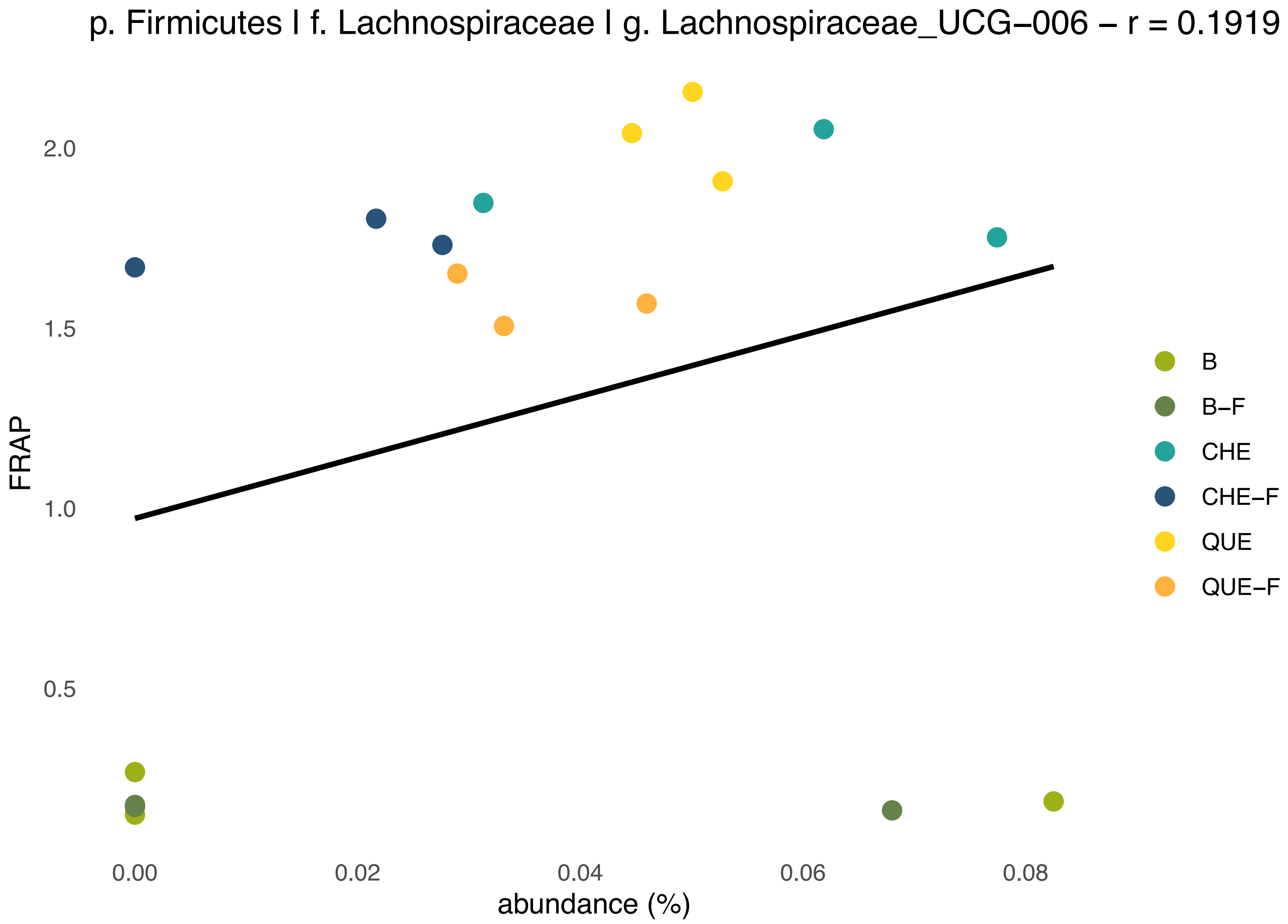
p. Firmicutes l f. Lachnospiraceae l g. Lachnospiraceae_UCG–006 – r = 0.1919
FRAP
2.0
1.5
1.0
0.5
0.00
0.02
0.04
0.06
0.08
abundance (%)
B
B–F
CHE
CHE–F
QUE
QUE–F

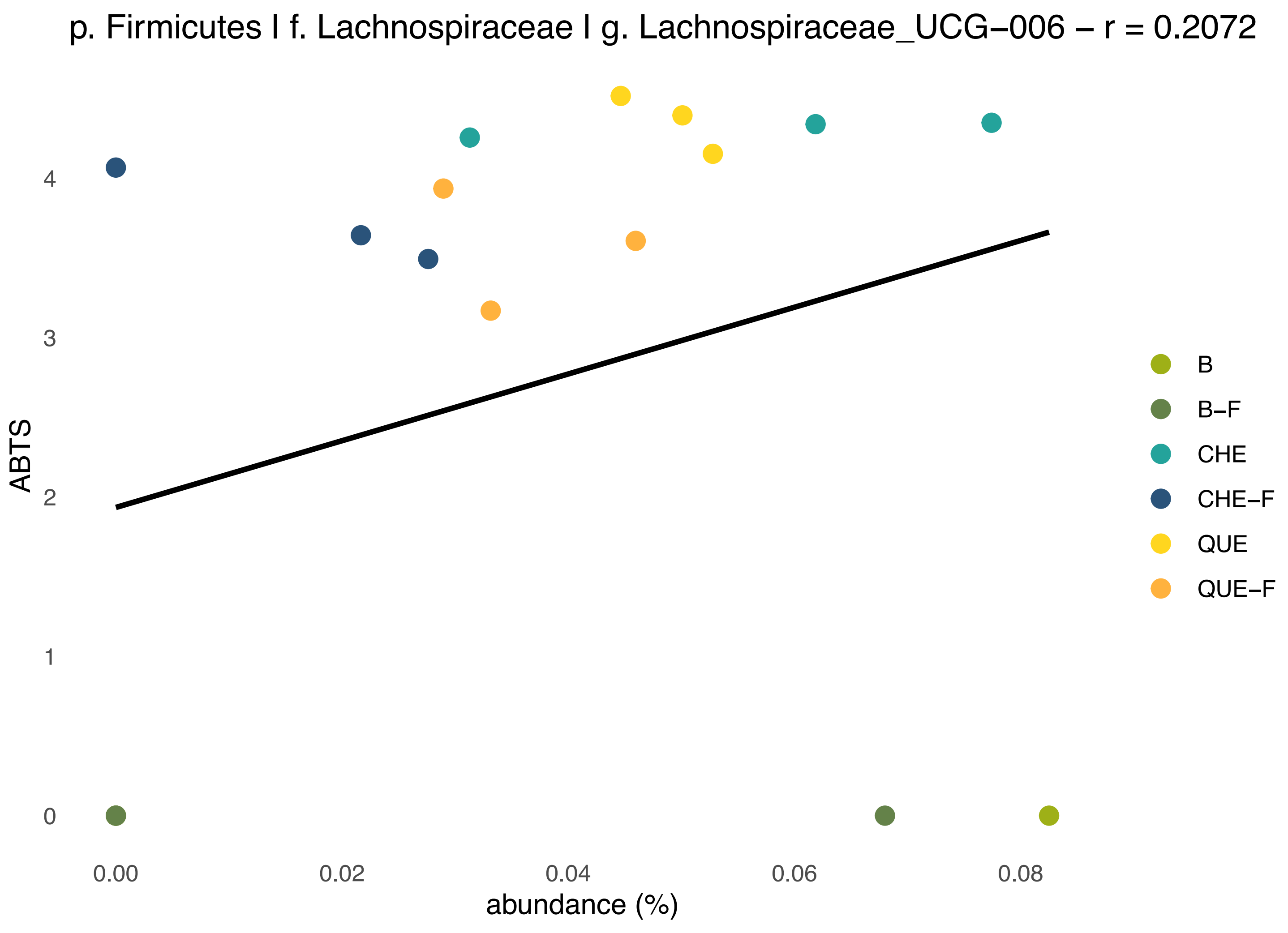

p. Firmicutes I f. Lachnospiraceae I g. Lachnospiraceae_UCG−006 − r = 0.2072
ABTS
0
1
2
3
4
0.00
0.02
0.04
0.06
0.08
abundance (%)
B
B−F
CHE
CHE−F
QUE
QUE−F

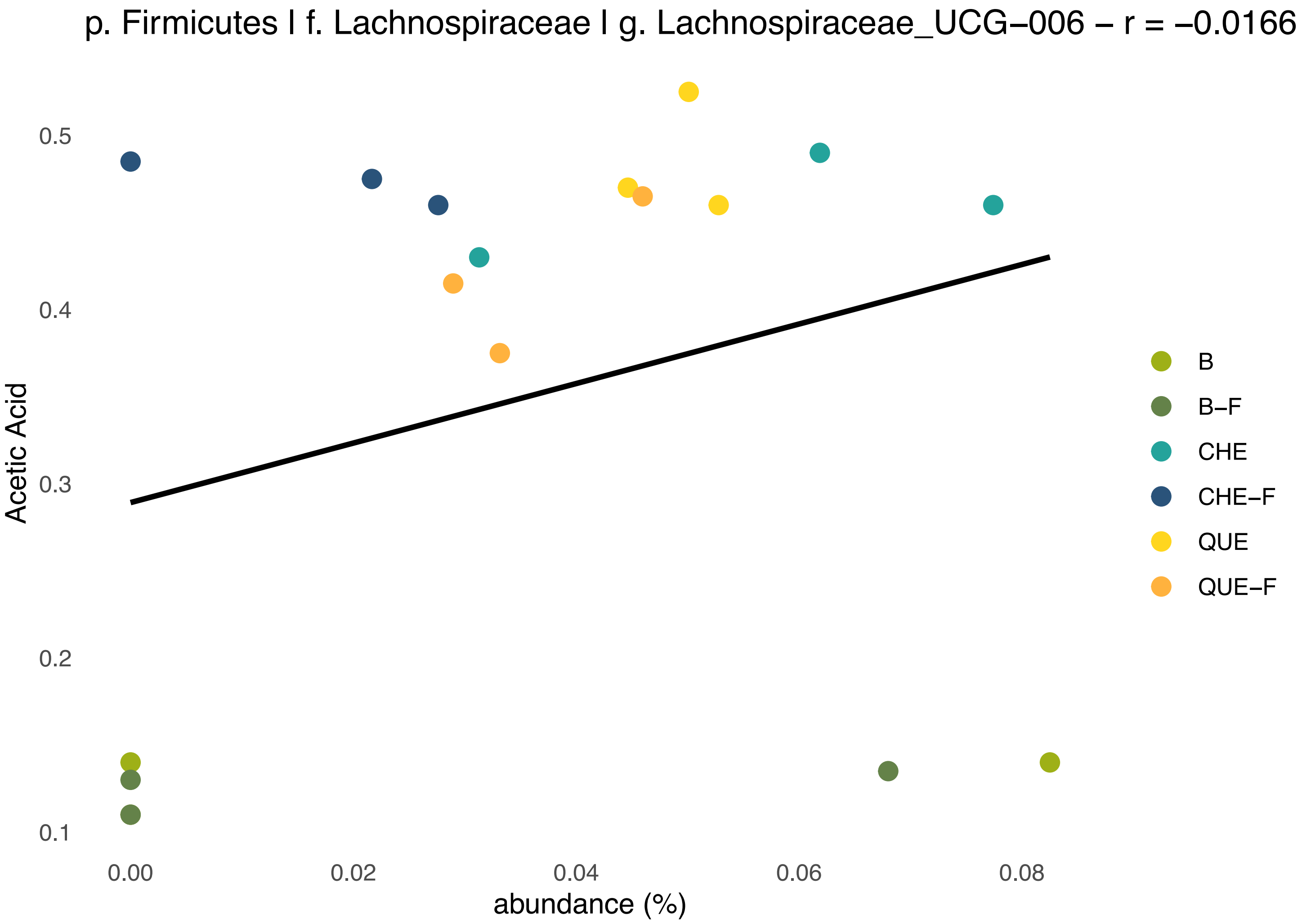

p. Firmicutes I f. Lachnospiraceae I g. Lachnospiraceae_UCG−006 − r = −0.0166
Acetic Acid
0.5
0.4
0.3
0.2
0.1
0.00
0.02
0.04
0.06
0.08
abundance (%)
B
B−F
CHE
CHE−F
QUE
QUE−F

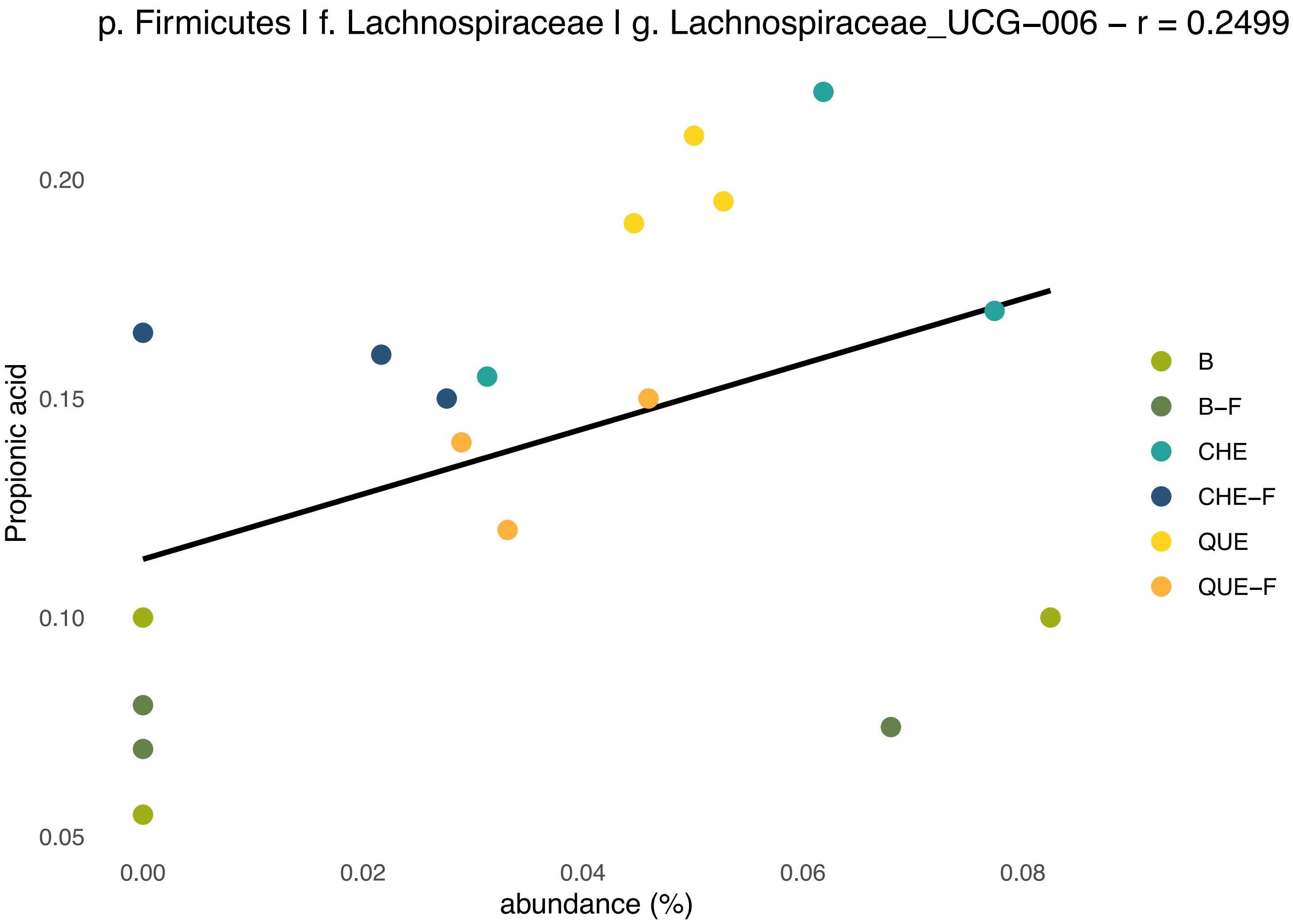

p. Firmicutes I f. Lachnospiraceae I g. Lachnospiraceae_UCG−006 − r = 0.2499
Propionic acid
0.20
0.15
0.10
0.05
0.00
0.02
0.04
0.06
0.08
abundance (%)
B
B−F
CHE
CHE−F
QUE
QUE−F

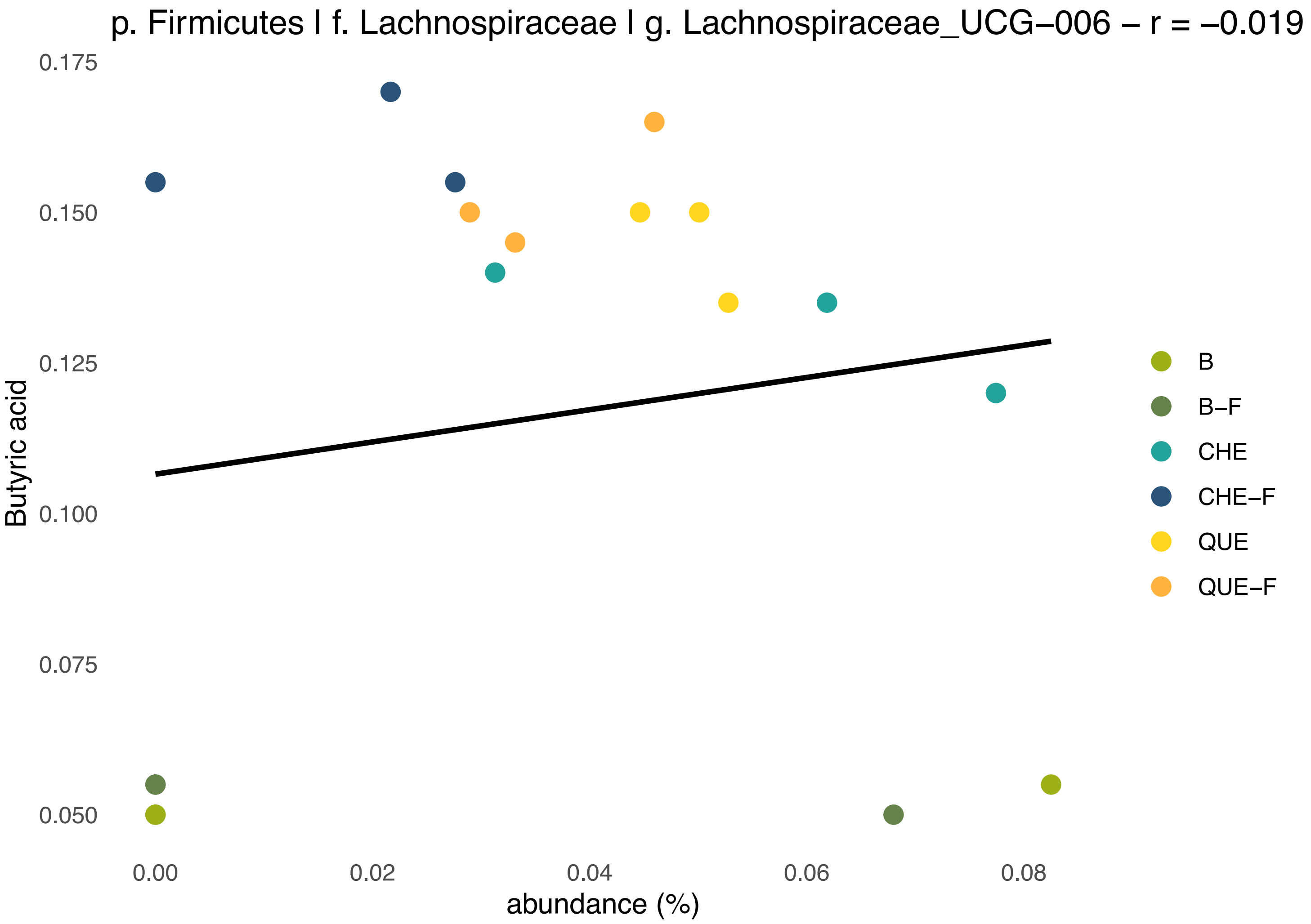
p. Firmicutes l f. Lachnospiraceae l g. Lachnospiraceae_UCG−006 − r = −0.019
Butyric acid
0.175
0.150
0.125
0.100
0.075
0.050
0.00
0.02
0.04
0.06
0.08
abundance (%)
B
B−F
CHE
CHE−F
QUE
QUE−F

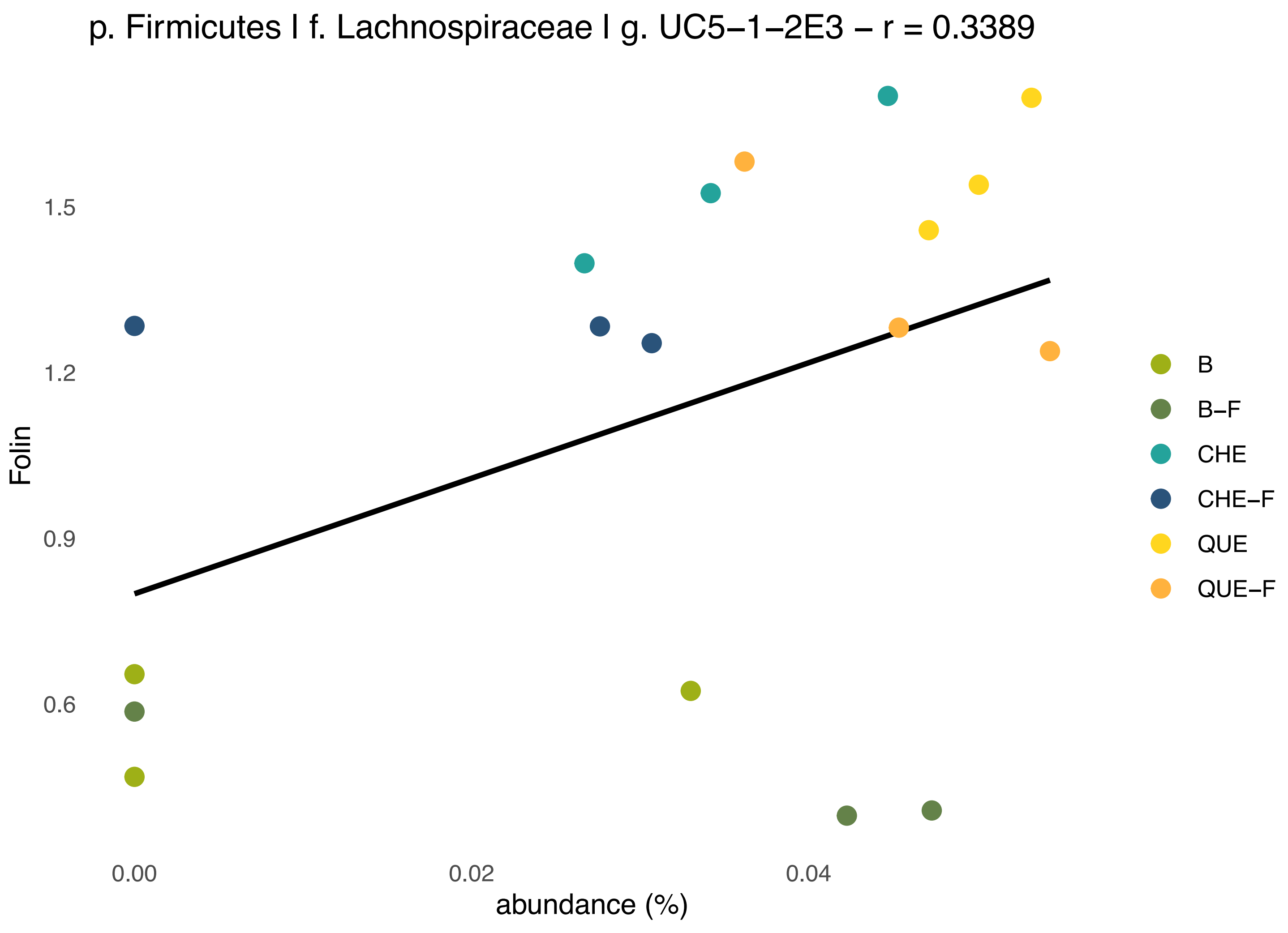
p. Firmicutes I f. Lachnospiraceae I g. UC5−1−2E3 − r = 0.3389
Folin
1.5
1.2
0.9
0.6
0.00
0.02
0.04
abundance (%)
B
B−F
CHE
CHE−F
QUE
QUE−F

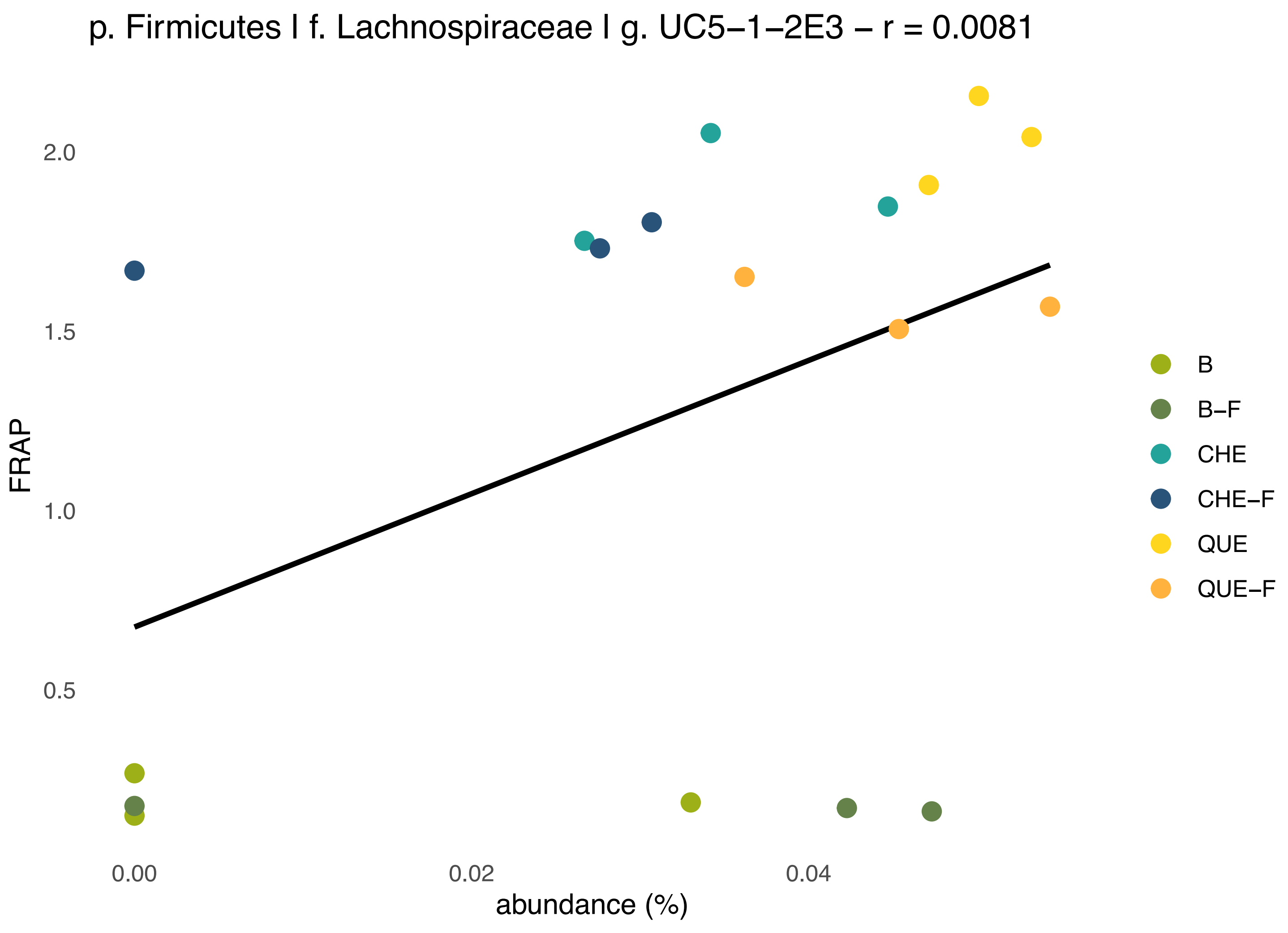

p. Firmicutes I f. Lachnospiraceae I g. UC5−1−2E3 − r = 0.0081
FRAP
2.0
1.5
1.0
0.5
0.00
0.02
0.04
abundance (%)
B
B−F
CHE
CHE−F
QUE
QUE−F

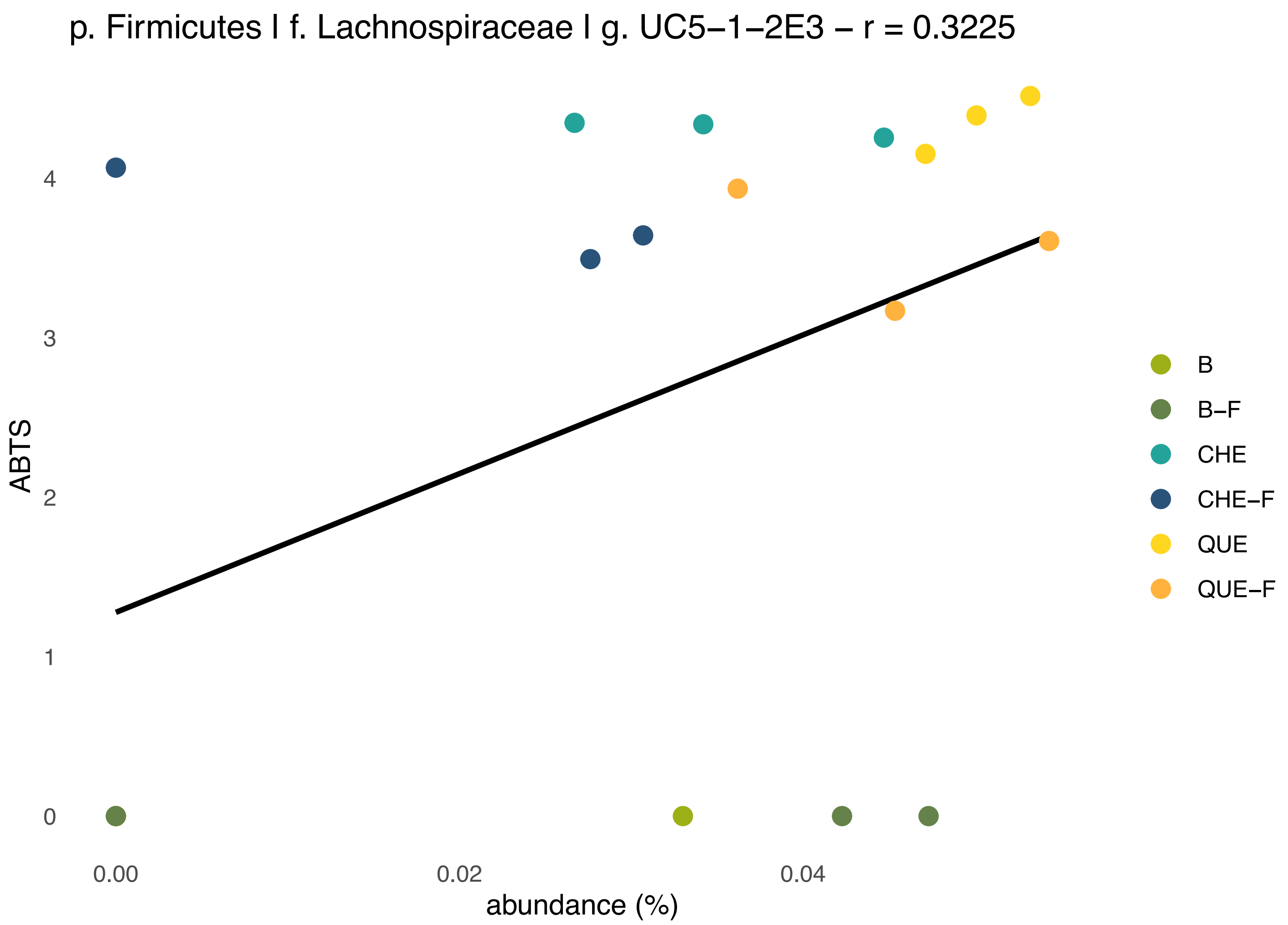

p. Firmicutes I f. Lachnospiraceae I g. UC5−1−2E3 − r = 0.3225
ABTS
4
3
2
1
0
0.00
0.02
0.04
abundance (%)
B
B−F
CHE
CHE−F
QUE
QUE−F

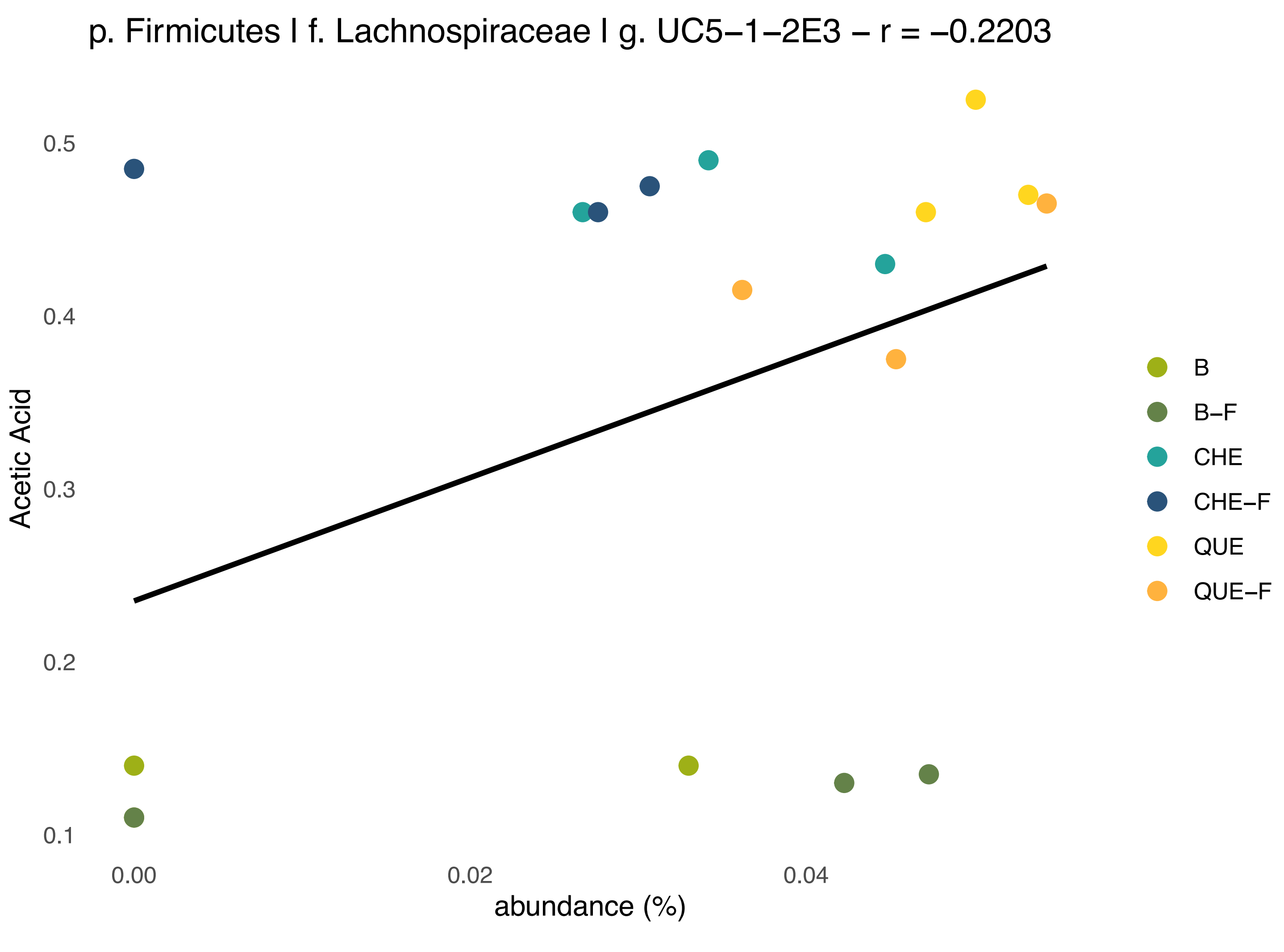

p. Firmicutes I f. Lachnospiraceae I g. UC5−1−2E3 − r = −0.2203
Acetic Acid
0.5
0.4
0.3
0.2
0.1
0.00
0.02
0.04
abundance (%)
B
B−F
CHE
CHE−F
QUE
QUE−F

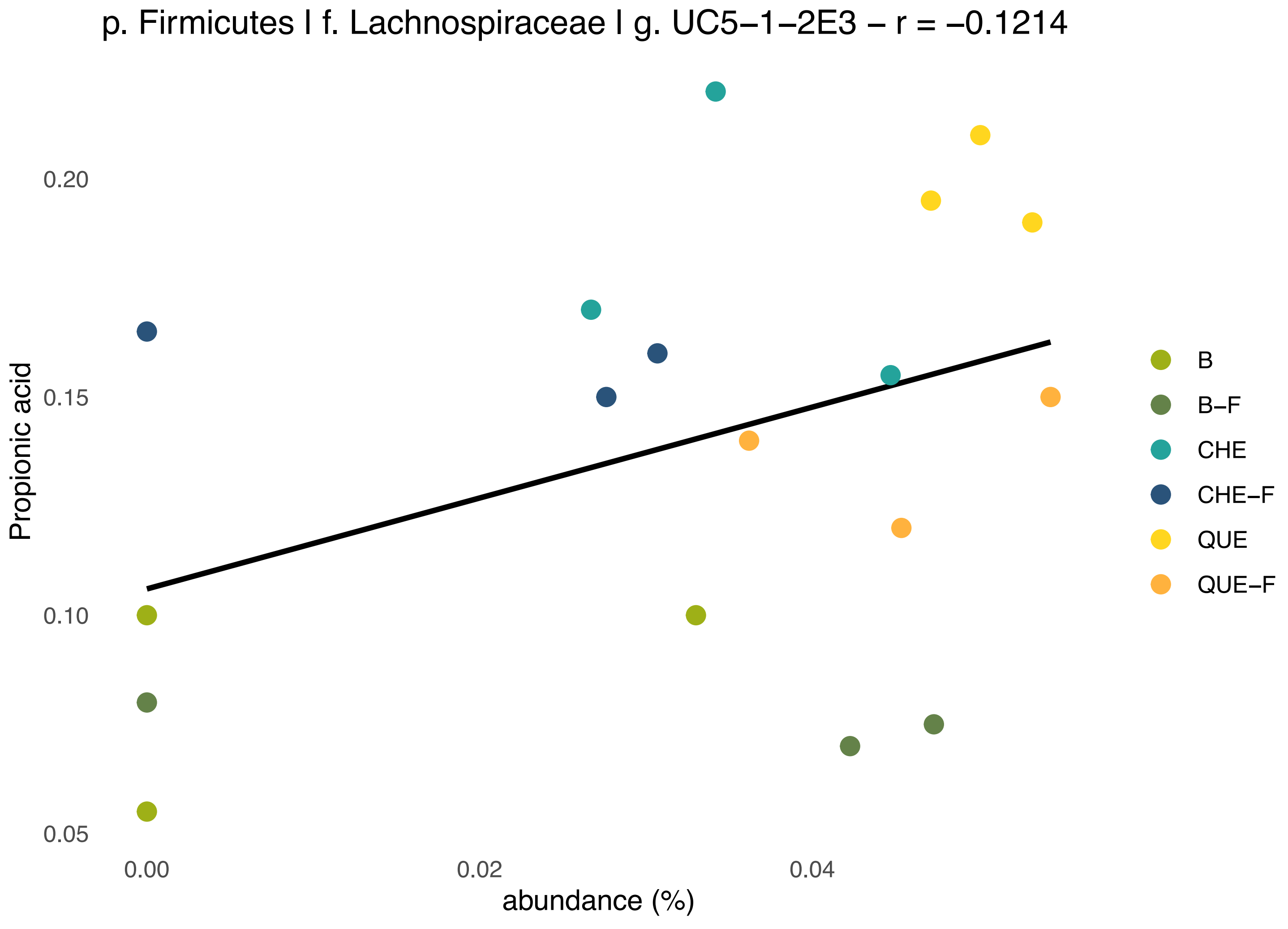

p. Firmicutes I f. Lachnospiraceae I g. UC5−1−2E3 − r = −0.1214
Propionic acid
0.20
0.15
0.10
0.05
0.00
0.02
0.04
abundance (%)
B
B−F
CHE
CHE−F
QUE
QUE−F

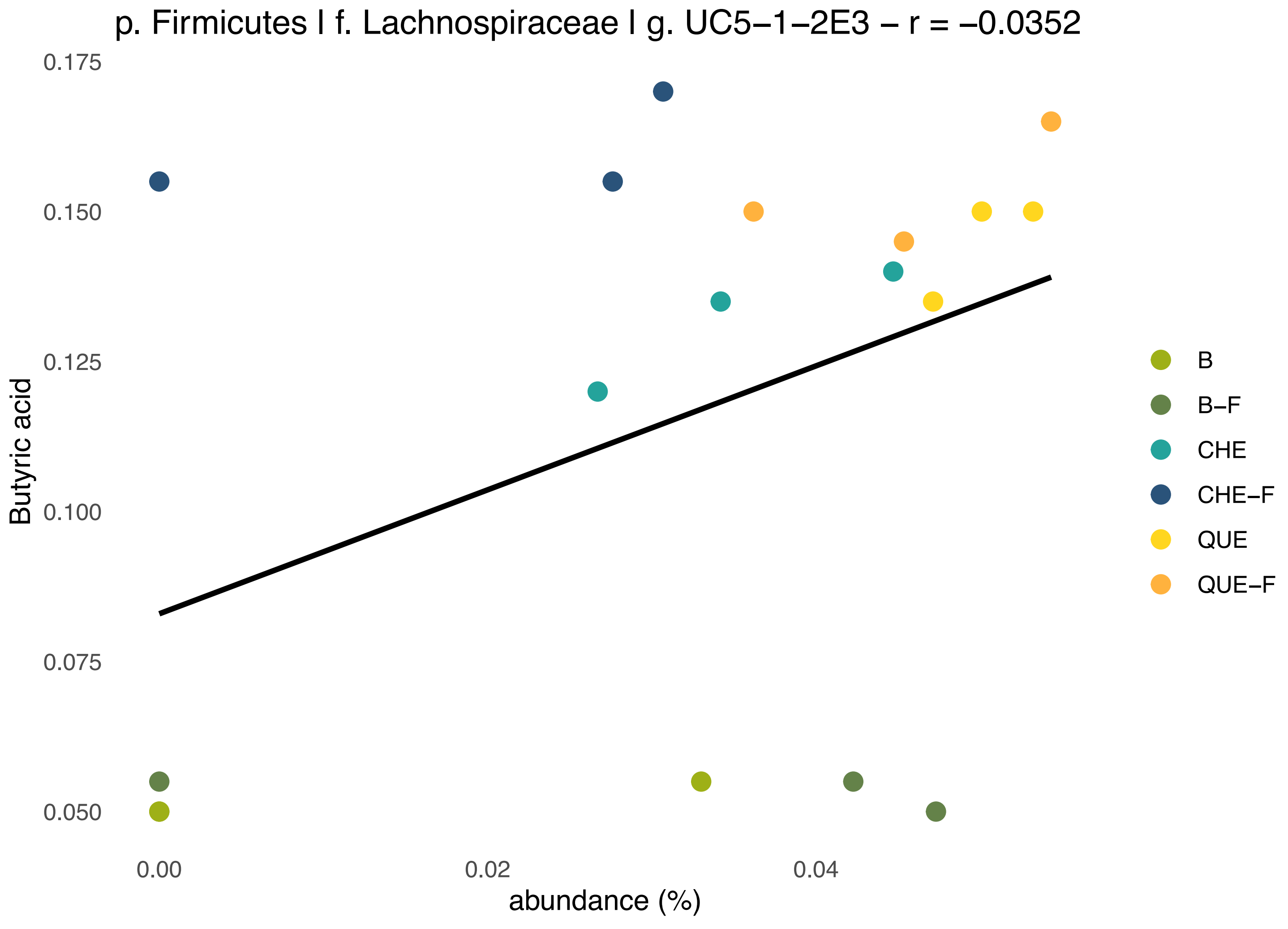
p. Firmicutes | f. Lachnospiraceae | g. UC5−1−2E3 − r = −0.0352
0.175
0.150
0.125
0.100
0.075
0.050
Butyric acid
0.00
0.02
0.04
abundance (%)
B
B−F
CHE
CHE−F
QUE
QUE−F

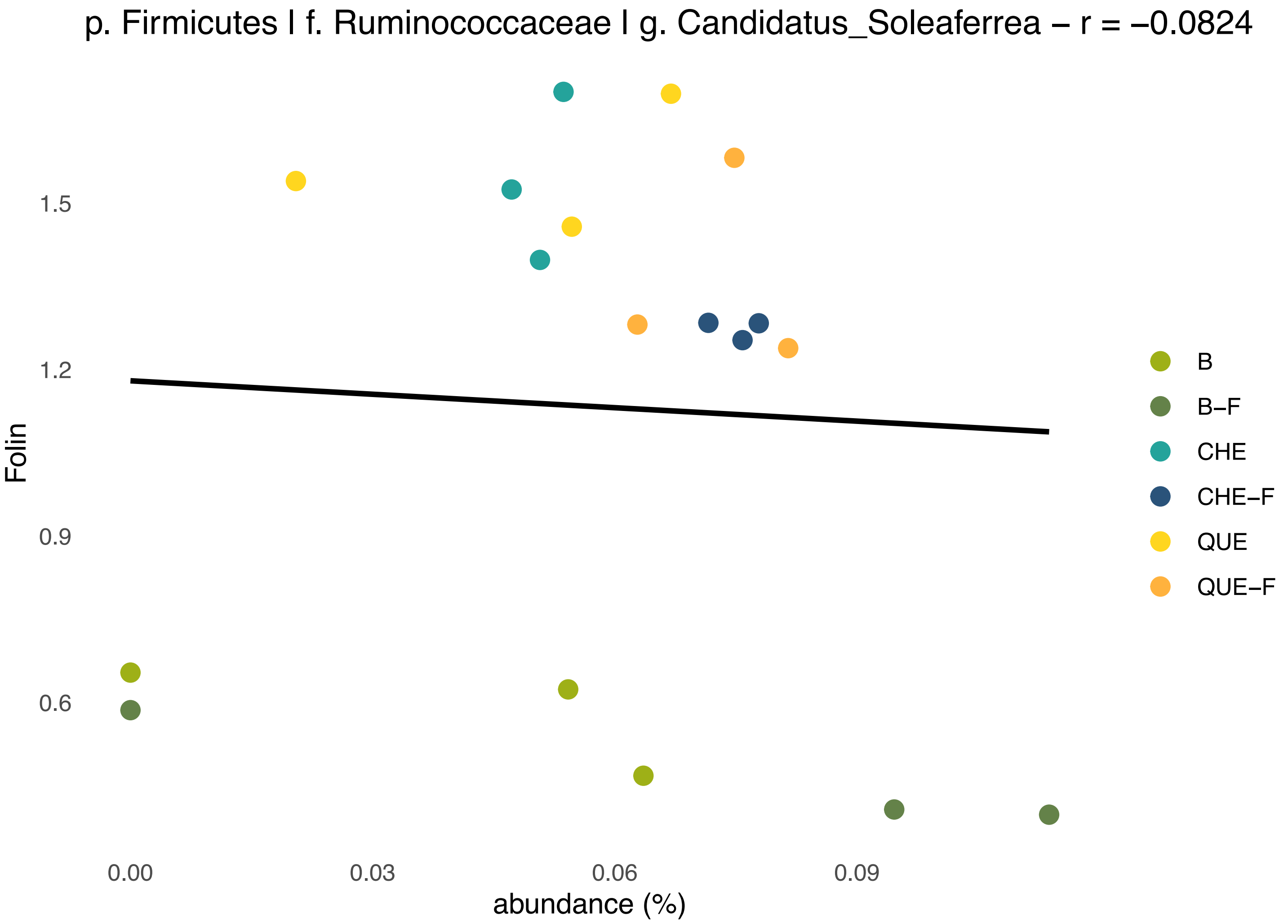

p. Firmicutes I f. Ruminococcaceae I g. Candidatus_Soleaferrea – r = –0.0824
1.5
1.2
0.9
0.6
Folin
0.00
0.03
0.06
0.09
abundance (%)
B
B–F
CHE
CHE–F
QUE
QUE–F

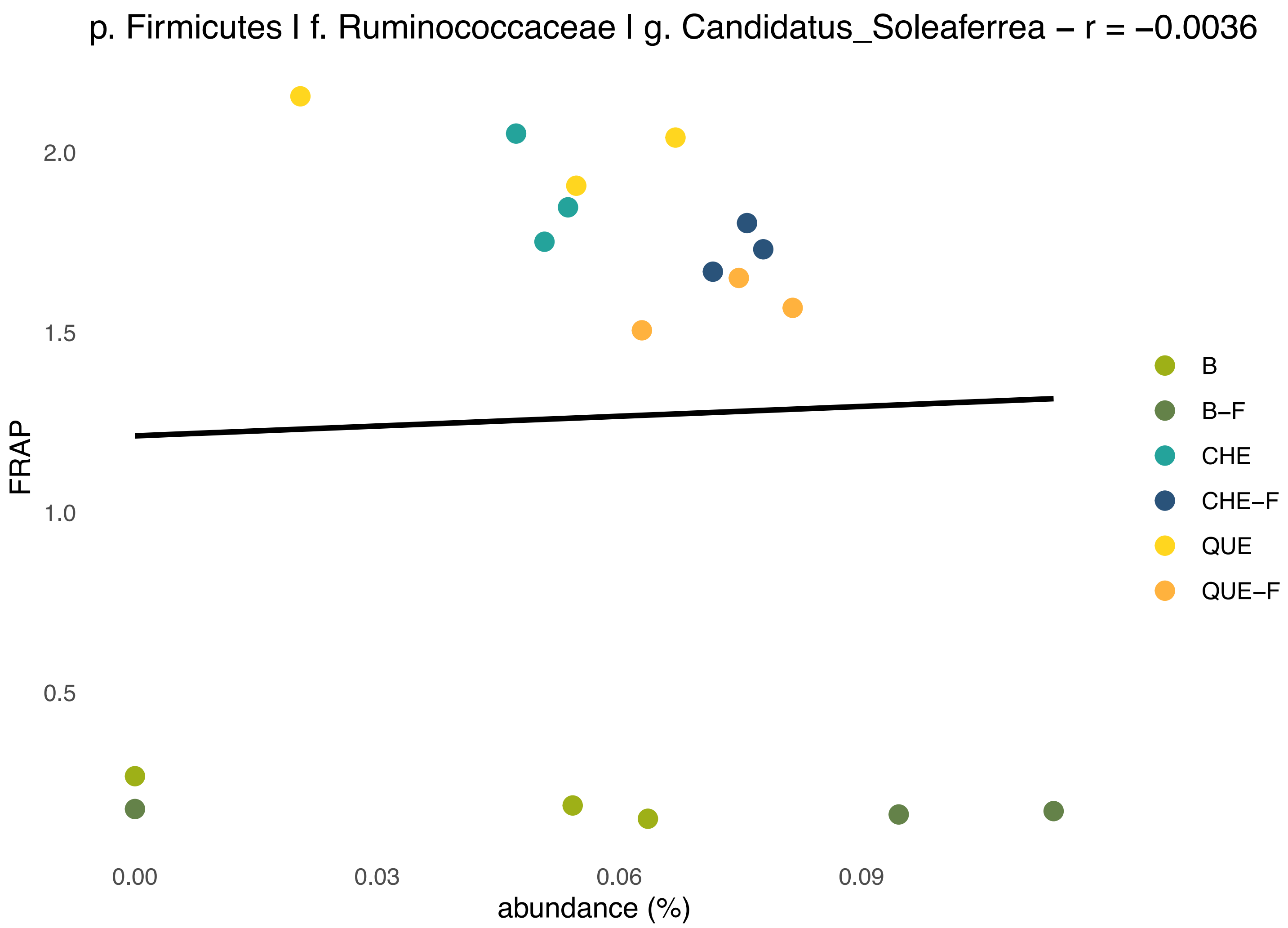

p. Firmicutes I f. Ruminococcaceae I g. Candidatus_Soleaferrea – r = –0.0036
FRAP
2.0
1.5
1.0
0.5
0.00
0.03
0.06
0.09
abundance (%)
B
B–F
CHE
CHE–F
QUE
QUE–F

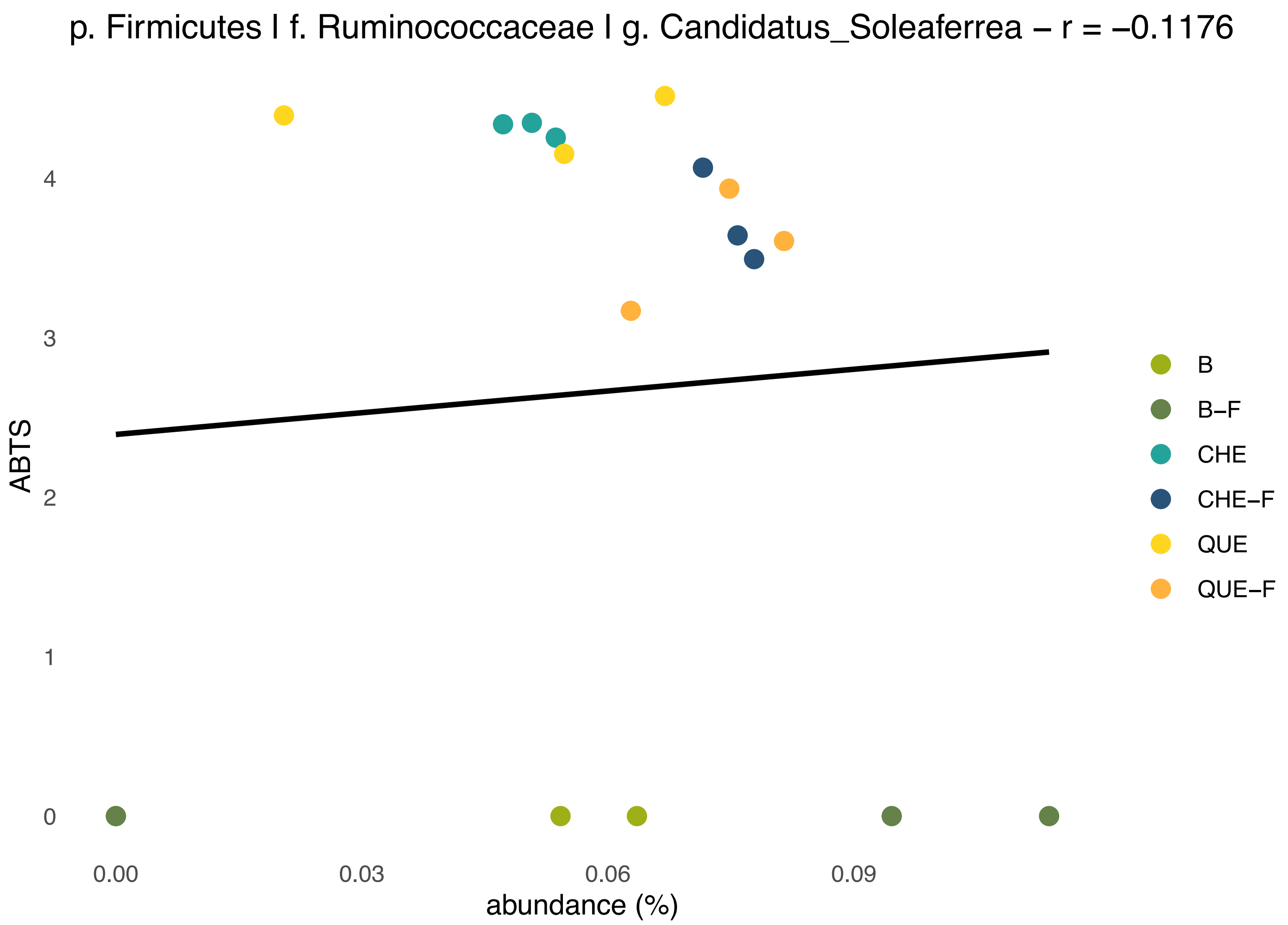
p. Firmicutes I f. Ruminococcaceae I g. Candidatus_Soleaferrea – r = –0.1176
ABTS
4
3
2
1
0
0.00
0.03
0.06
0.09
abundance (%)
B
B–F
CHE
CHE–F
QUE
QUE–F

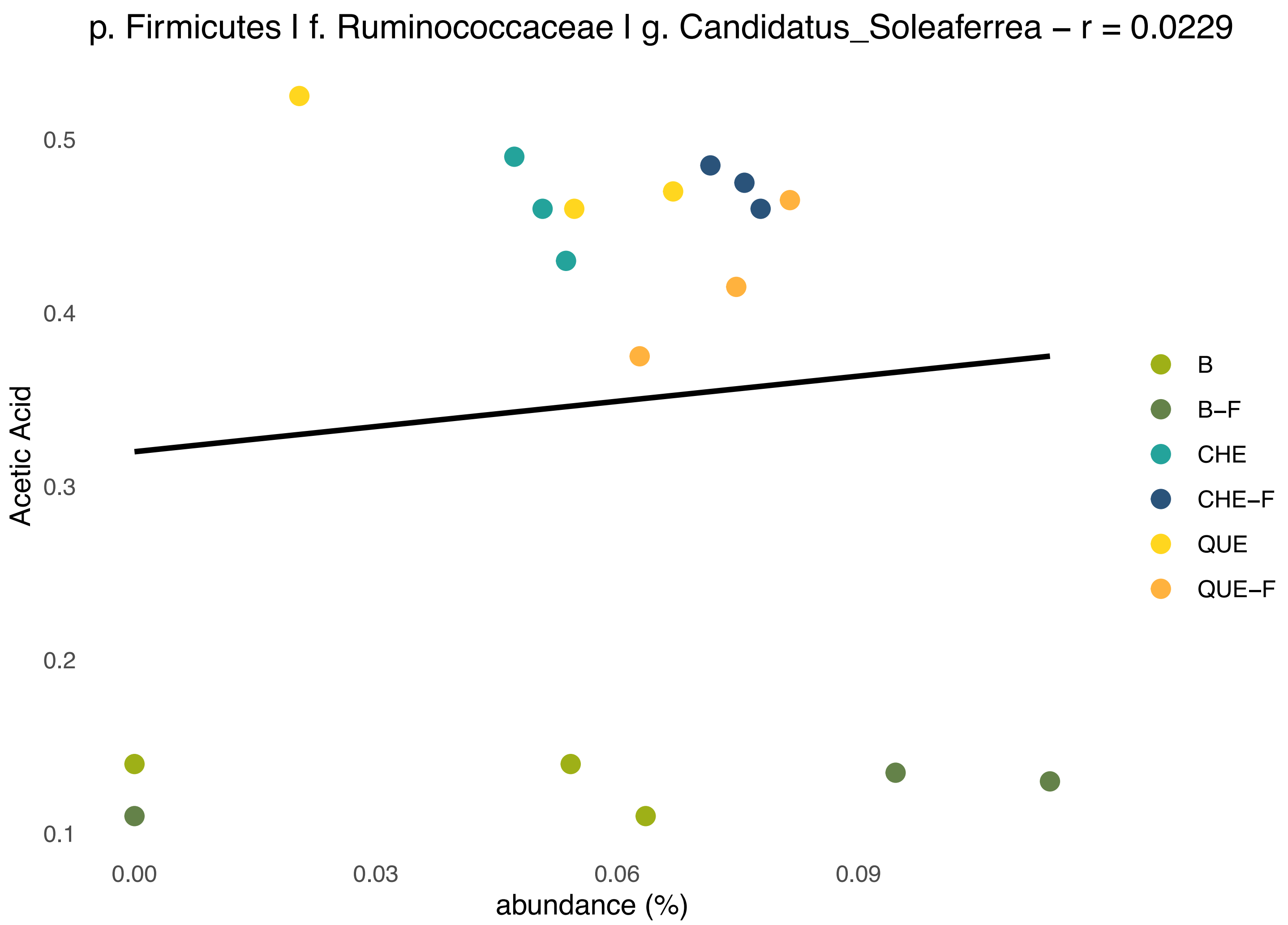

p. Firmicutes | f. Ruminococcaceae | g. Candidatus_Soleaferrea – r = 0.0229
Acetic Acid
0.5
0.4
0.3
0.2
0.1
0.00
0.03
0.06
0.09
abundance (%)
B
B–F
CHE
CHE–F
QUE
QUE–F

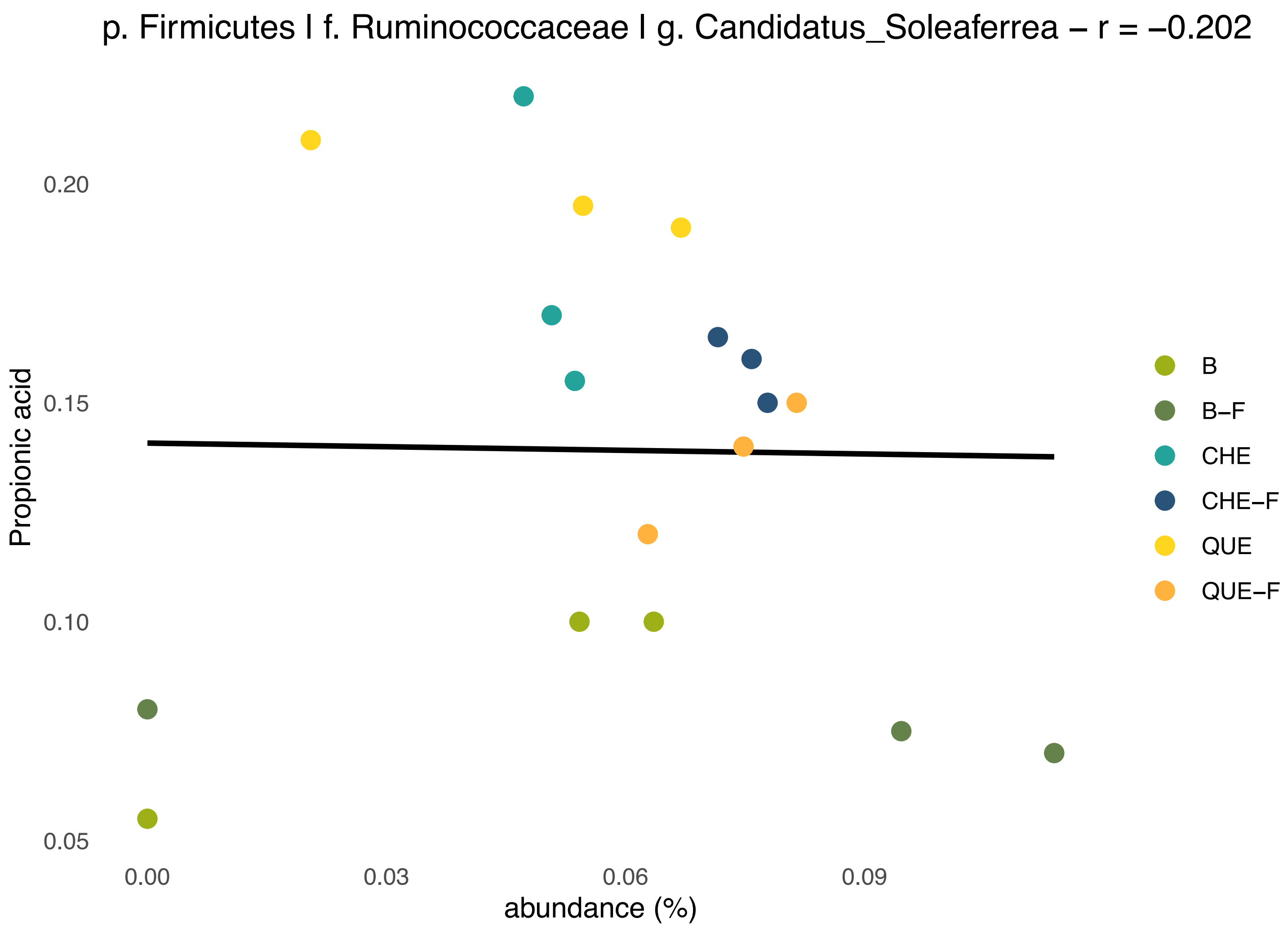

p. Firmicutes I f. Ruminococcaceae I g. Candidatus_Soleaferrea – r = −0.202
Propionic acid
0.20
0.15
0.10
0.05
0.00
0.03
0.06
0.09
abundance (%)
B
B−F
CHE
CHE−F
QUE
QUE−F

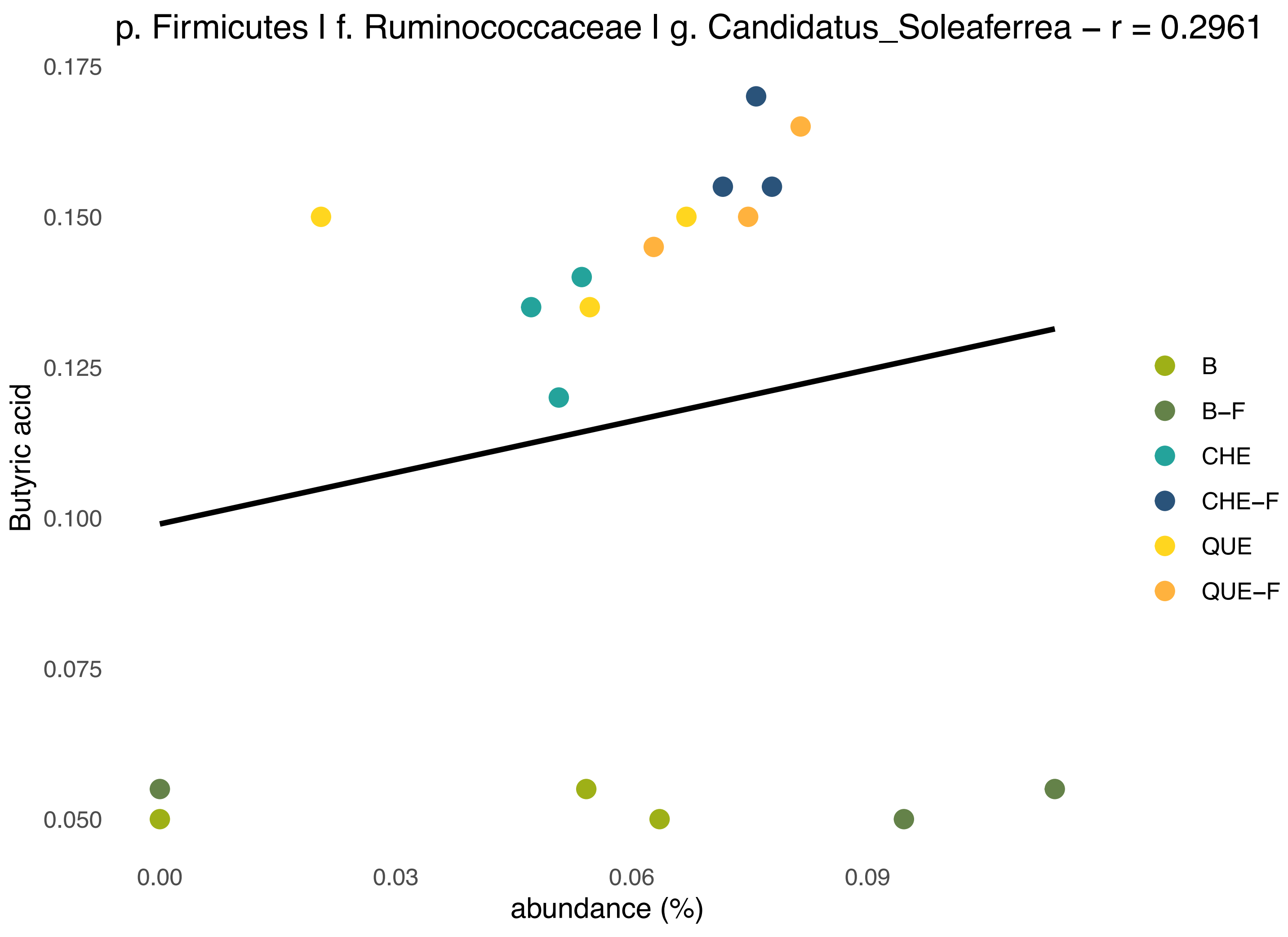
p. Firmicutes I f. Ruminococcaceae I g. Candidatus_Soleaferrea – r = 0.2961
Butyric acid
0.175
0.150
0.125
0.100
0.075
0.050
abundance (%)
0.00
0.03
0.06
0.09
B
B–F
CHE
CHE–F
QUE
QUE–F

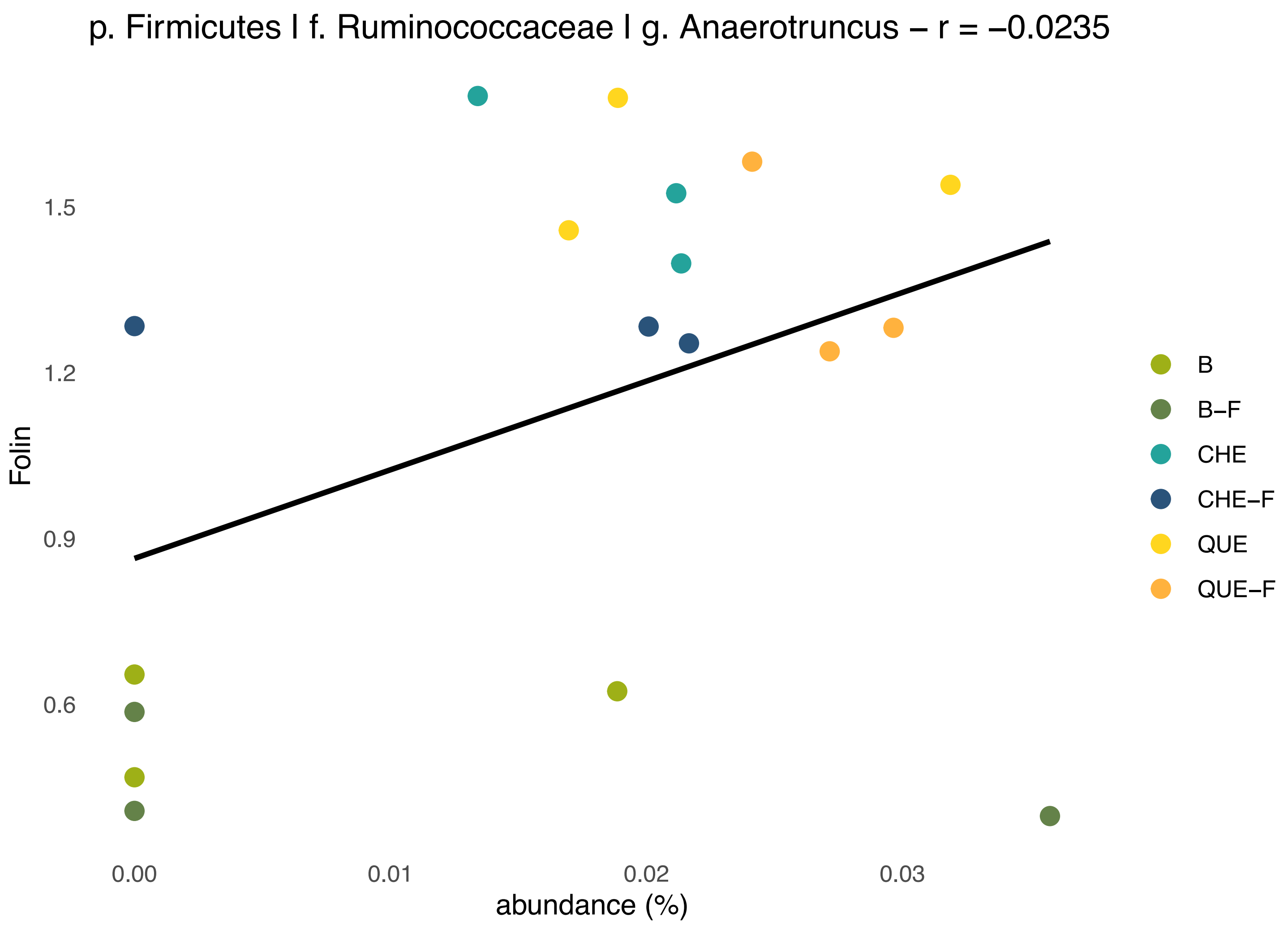

p. Firmicutes | f. Ruminococcaceae | g. Anaerotruncus – r = –0.0235
Folin
1.5
1.2
0.9
0.6
0.00
0.01
0.02
0.03
abundance (%)
B
B–F
CHE
CHE–F
QUE
QUE–F

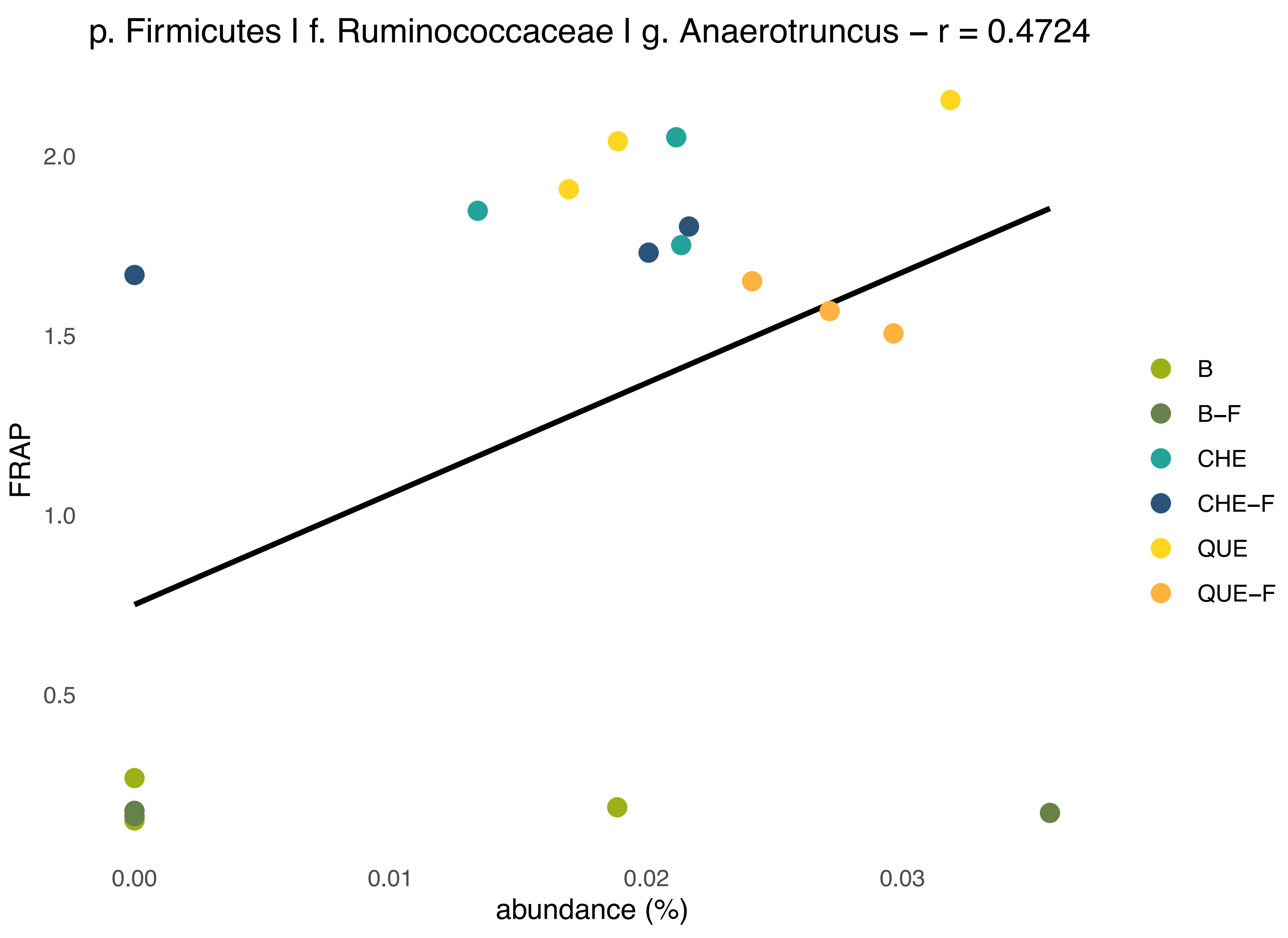
p. Firmicutes I f. Ruminococcaceae I g. Anaerotruncus – r = 0.4724
FRAP
2.0
1.5
1.0
0.5
0.00
0.01
0.02
0.03
abundance (%)
B
B–F
CHE
CHE–F
QUE
QUE–F

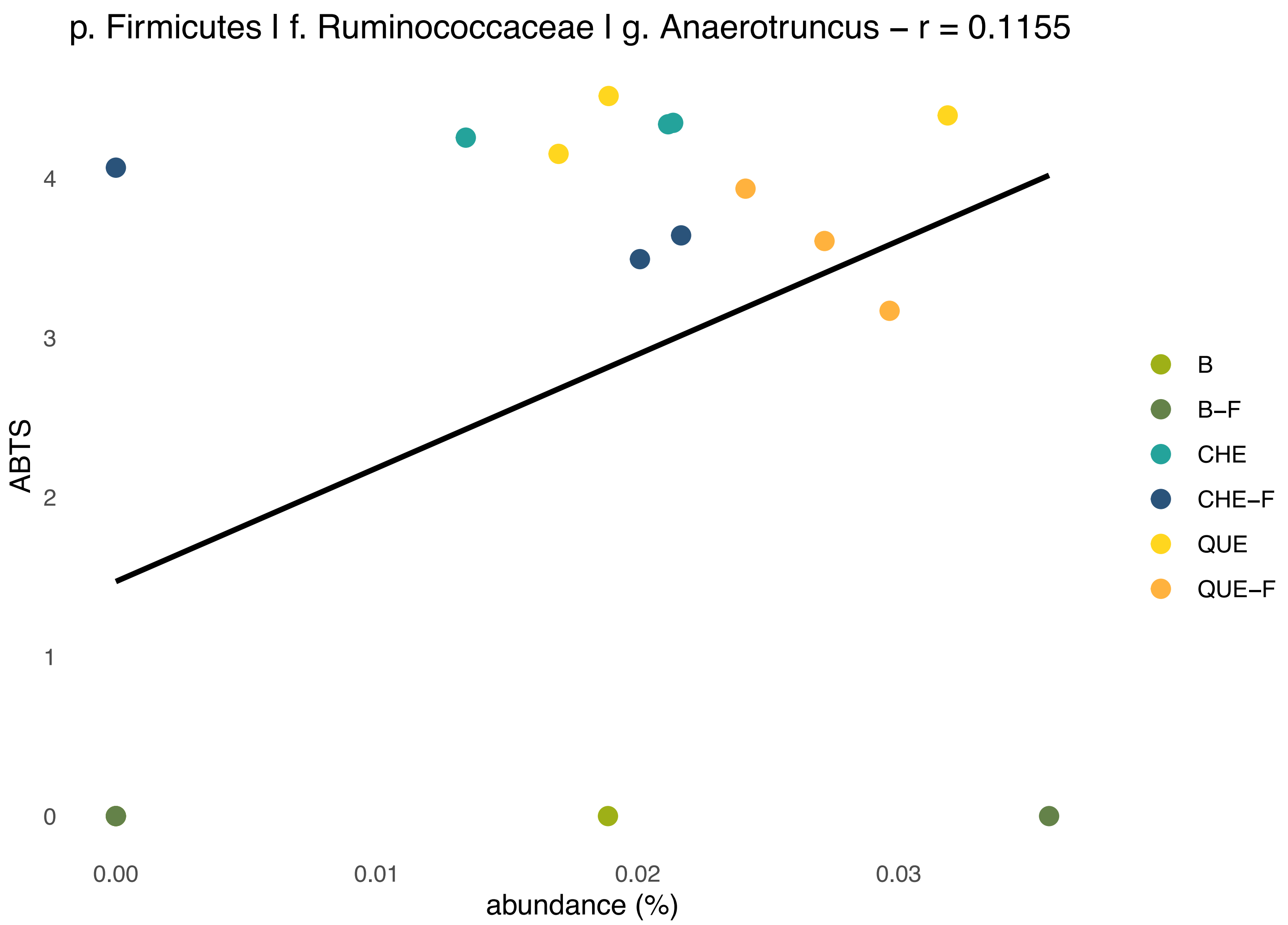

p. Firmicutes I f. Ruminococcaceae I g. Anaerotruncus – r = 0.1155
ABTS
4
3
2
1
0
0.00
0.01
0.02
0.03
abundance (%)
B
B–F
CHE
CHE–F
QUE
QUE–F

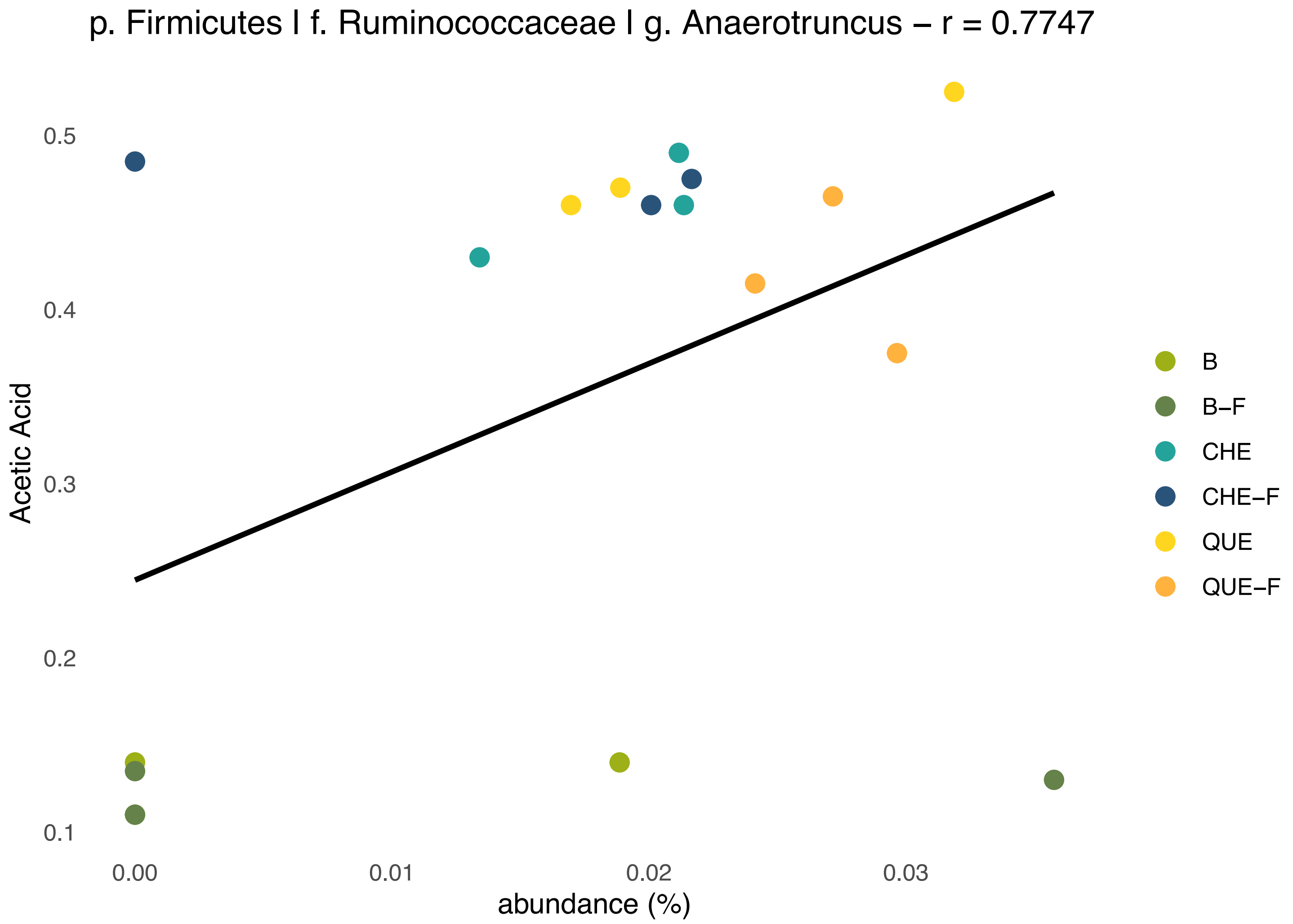

p. Firmicutes I f. Ruminococcaceae I g. Anaerotruncus – r = 0.7747
Acetic Acid
0.5
0.4
0.3
0.2
0.1
0.00
0.01
0.02
0.03
abundance (%)
B
B–F
CHE
CHE–F
QUE
QUE–F

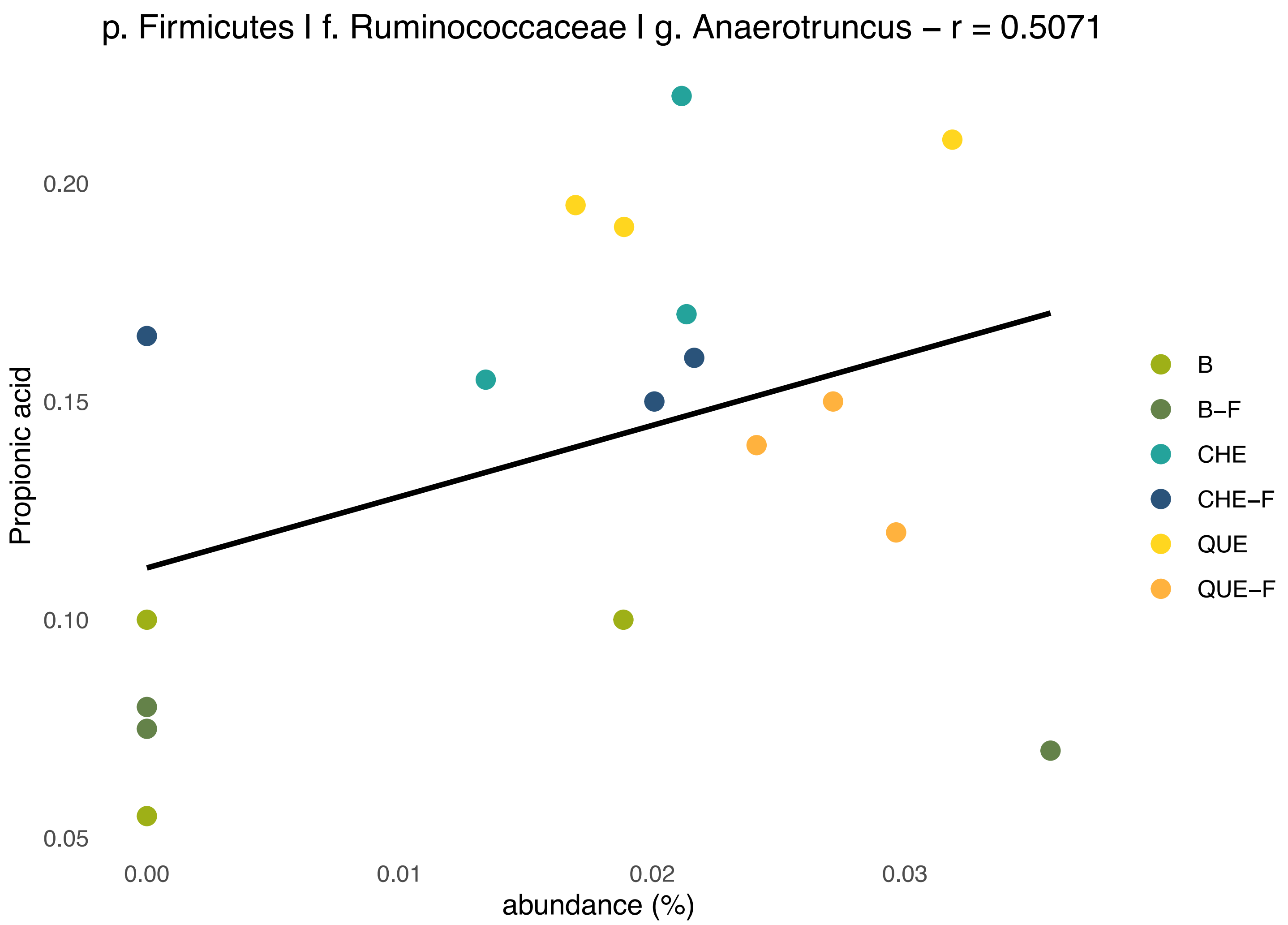

p. Firmicutes l f. Ruminococcaceae l g. Anaerotruncus – r = 0.5071
Propionic acid
0.20
0.15
0.10
0.05
0.00
0.01
0.02
0.03
abundance (%)
B
B–F
CHE
CHE–F
QUE
QUE–F

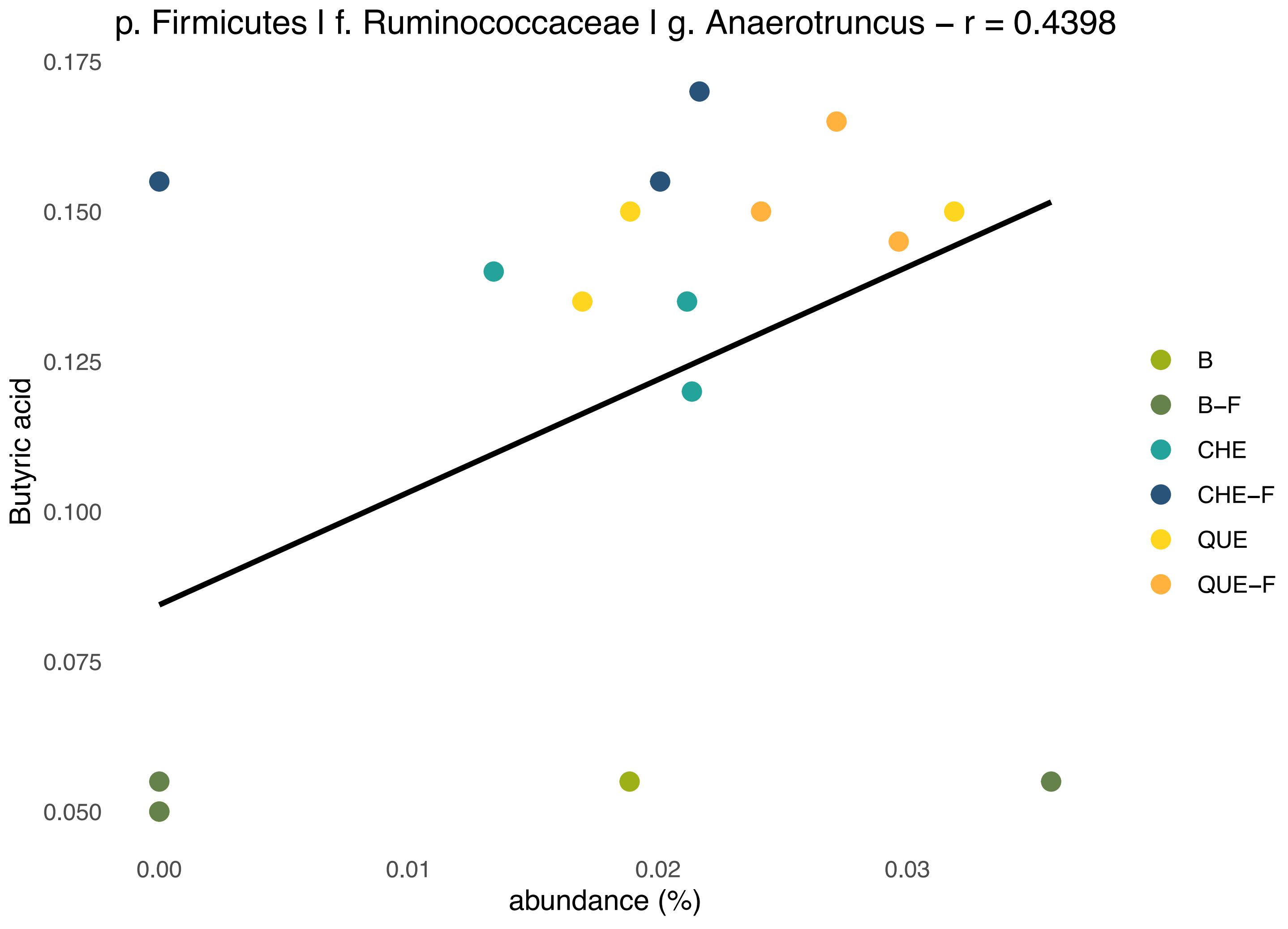
p. Firmicutes I f. Ruminococcaceae I g. Anaerotruncus – r = 0.4398
0.175
0.150
0.125
0.100
0.075
0.050
Butyric acid
0.00
0.01
0.02
0.03
abundance (%)
B
B–F
CHE
CHE–F
QUE
QUE–F

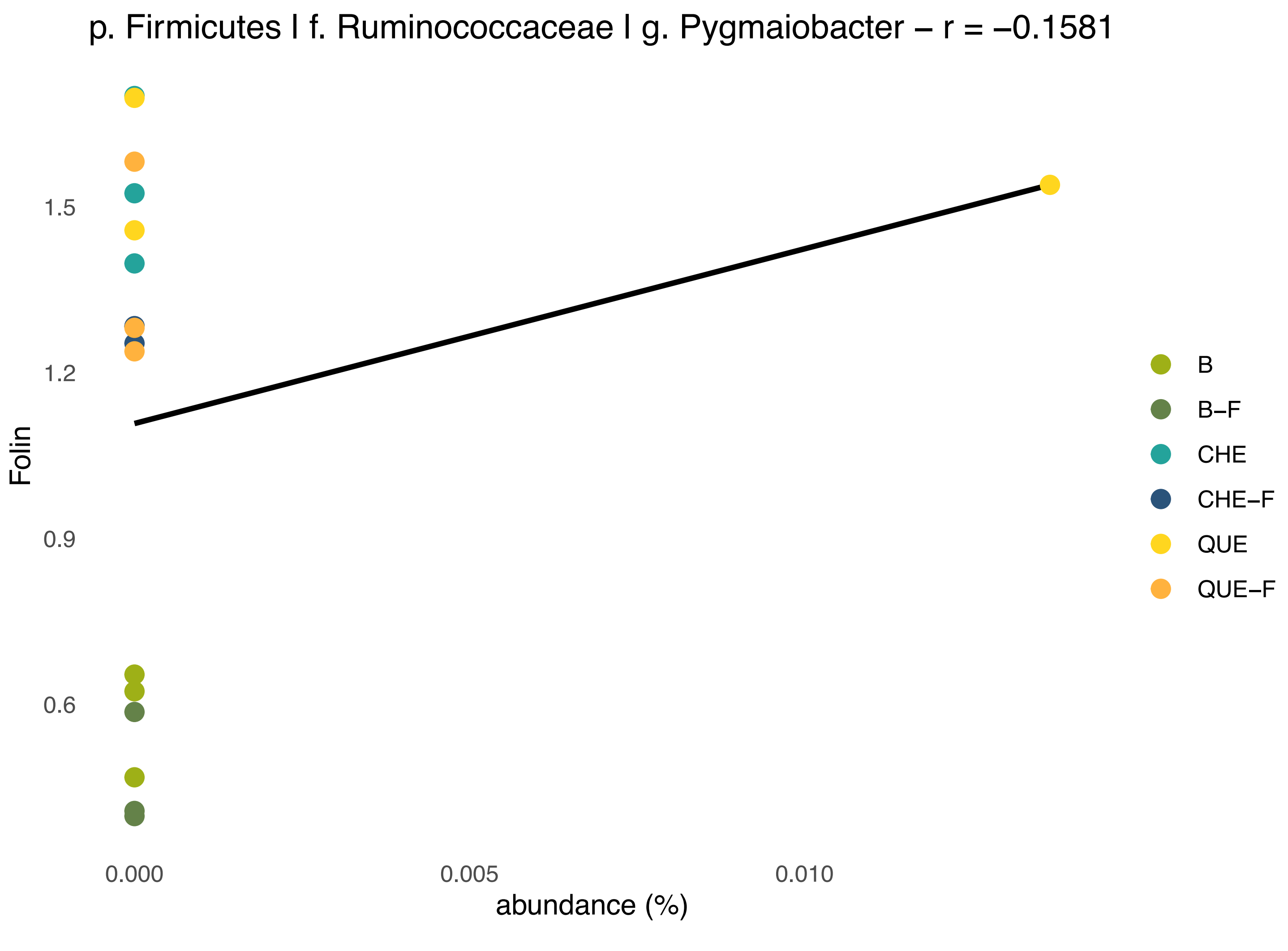

p. Firmicutes I f. Ruminococcaceae I g. Pygmaiobacter – r = −0.1581
Folin
1.5
1.2
0.9
0.6
0.000
0.005
0.010
abundance (%)
B
B−F
CHE
CHE−F
QUE
QUE−F

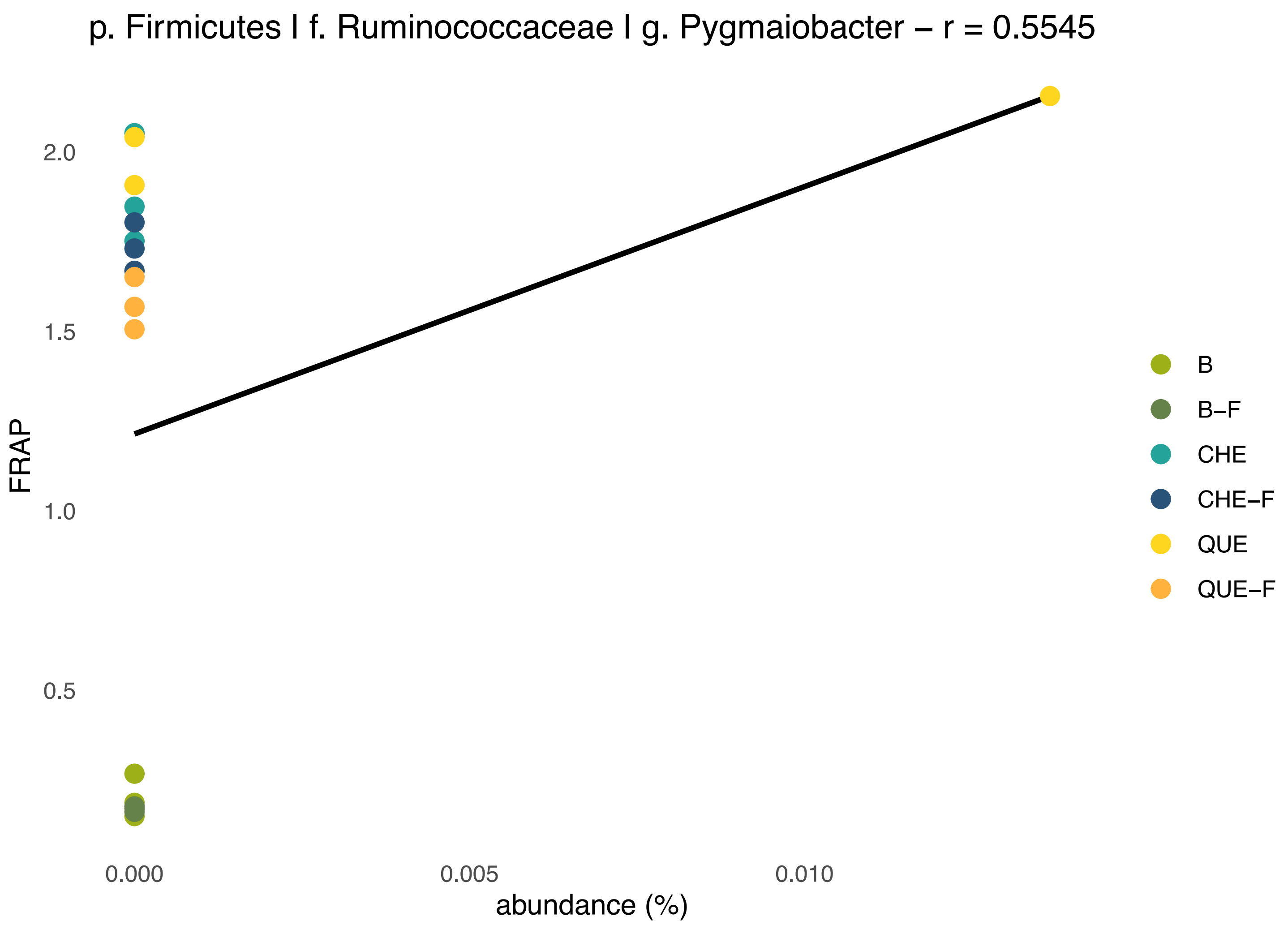

p. Firmicutes I f. Ruminococcaceae I g. Pygmaiobacter – r = 0.5545
FRAP
2.0
1.5
1.0
0.5
0.000
0.005
0.010
abundance (%)
B
B–F
CHE
CHE–F
QUE
QUE–F

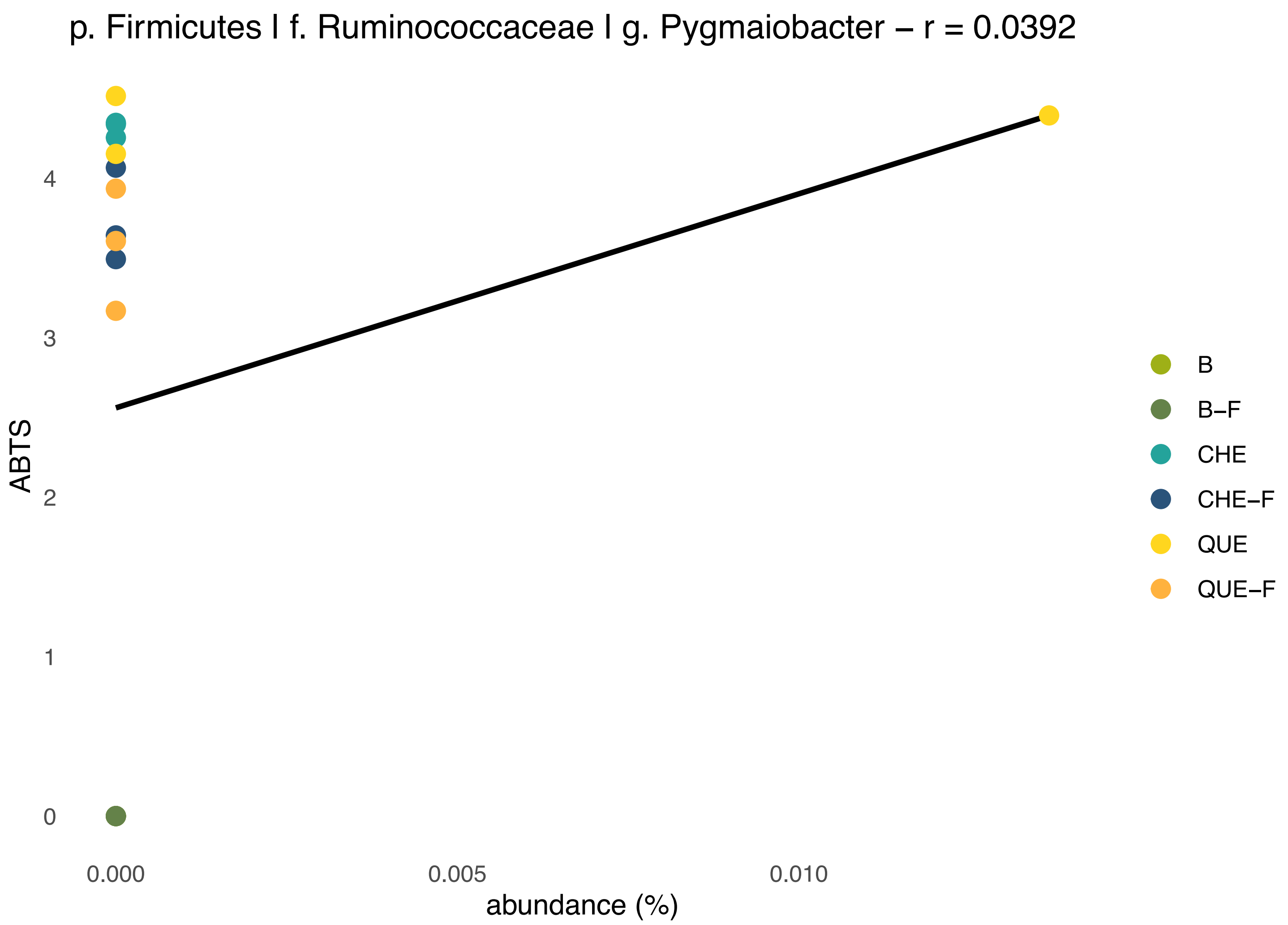

p. Firmicutes I f. Ruminococcaceae I g. Pygmaiobacter – r = 0.0392
ABTS
0
1
2
3
4
0.000
0.005
0.010
abundance (%)
B
B–F
CHE
CHE–F
QUE
QUE–F

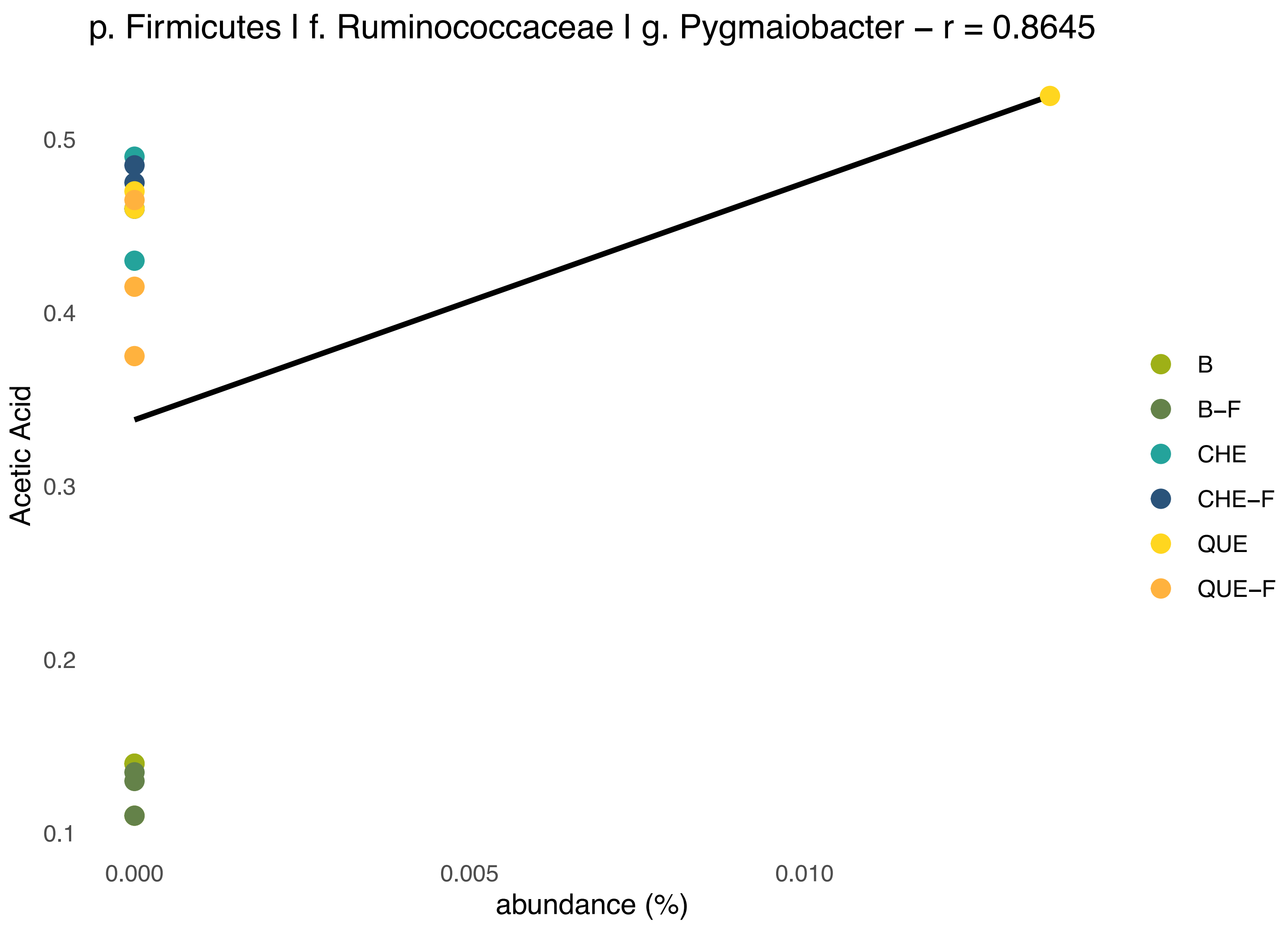

p. Firmicutes I f. Ruminococcaceae I g. Pygmaiobacter – r = 0.8645
Acetic Acid
0.5
0.4
0.3
0.2
0.1
0.000
0.005
0.010
abundance (%)
B
B–F
CHE
CHE–F
QUE
QUE–F

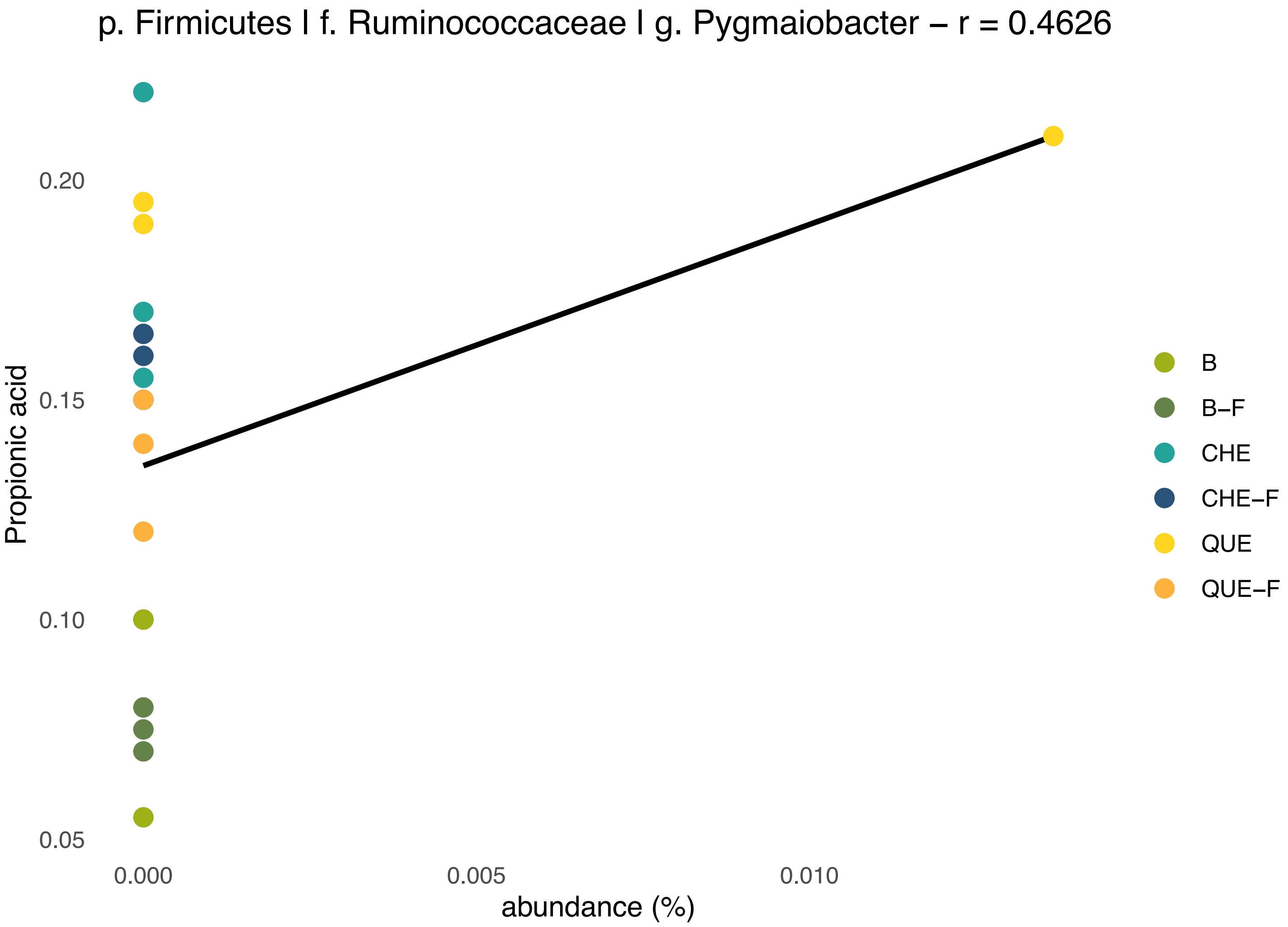
p. Firmicutes I f. Ruminococcaceae I g. Pygmaiobacter – r = 0.4626
Propionic acid
0.20
0.15
0.10
0.05
0.000
0.005
0.010
abundance (%)
B
B–F
CHE
CHE–F
QUE
QUE–F

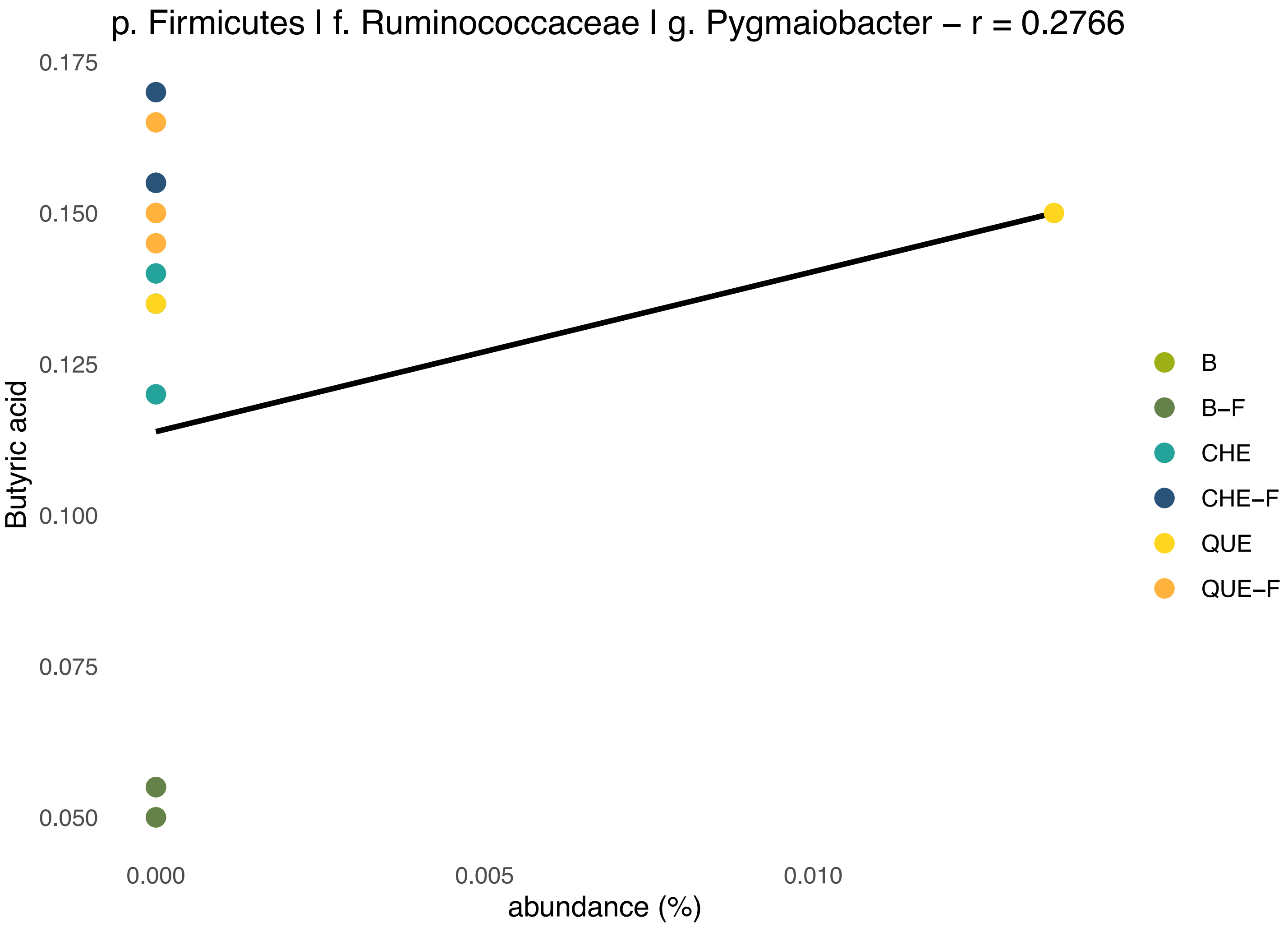

p. Firmicutes I f. Ruminococcaceae I g. Pygmaiobacter – r = 0.2766
0.175
0.150
0.125
0.100
0.075
0.050
Butyric acid
0.000
0.005
0.010
abundance (%)
B
B–F
CHE
CHE–F
QUE
QUE–F

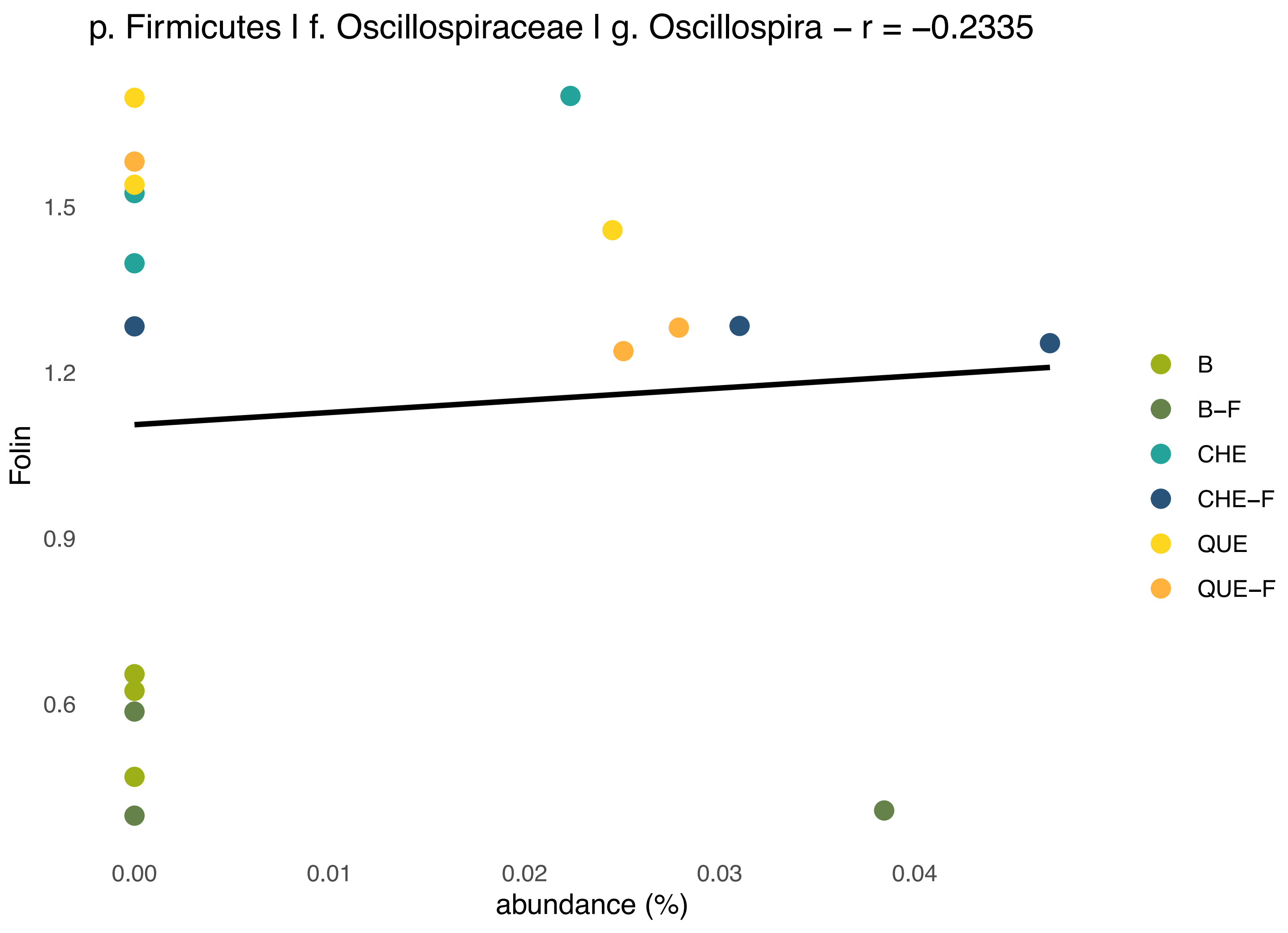
p. Firmicutes I f. Oscillospiraceae I g. Oscillospira – r = –0.2335
Folin
1.5
1.2
0.9
0.6
0.00
0.01
0.02
0.03
0.04
abundance (%)
B
B–F
CHE
CHE–F
QUE
QUE–F

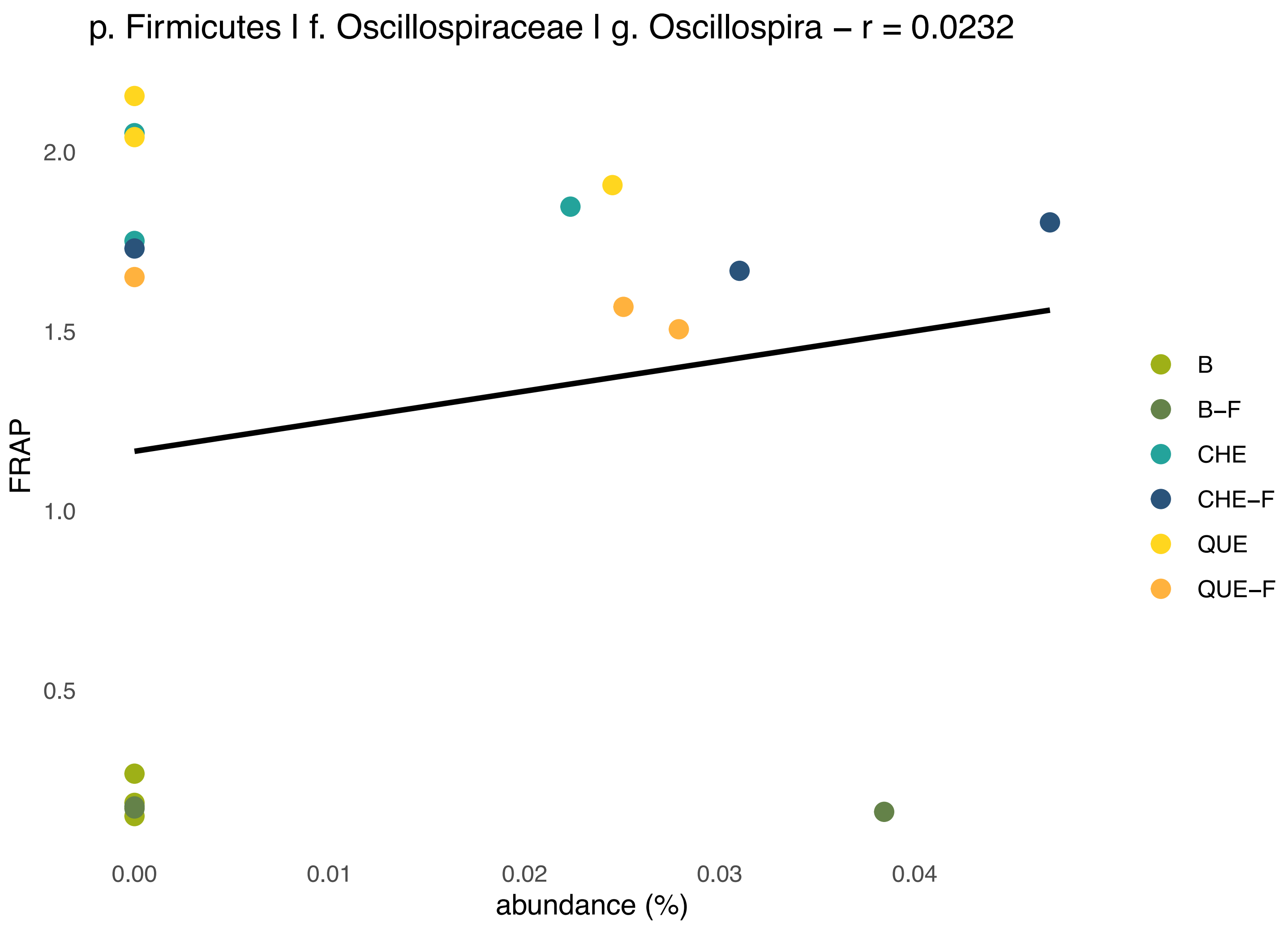

p. Firmicutes I f. Oscillospiraceae I g. Oscillospira – r = 0.0232
FRAP
2.0
1.5
1.0
0.5
0.00
0.01
0.02
0.03
0.04
abundance (%)
B
B–F
CHE
CHE–F
QUE
QUE–F

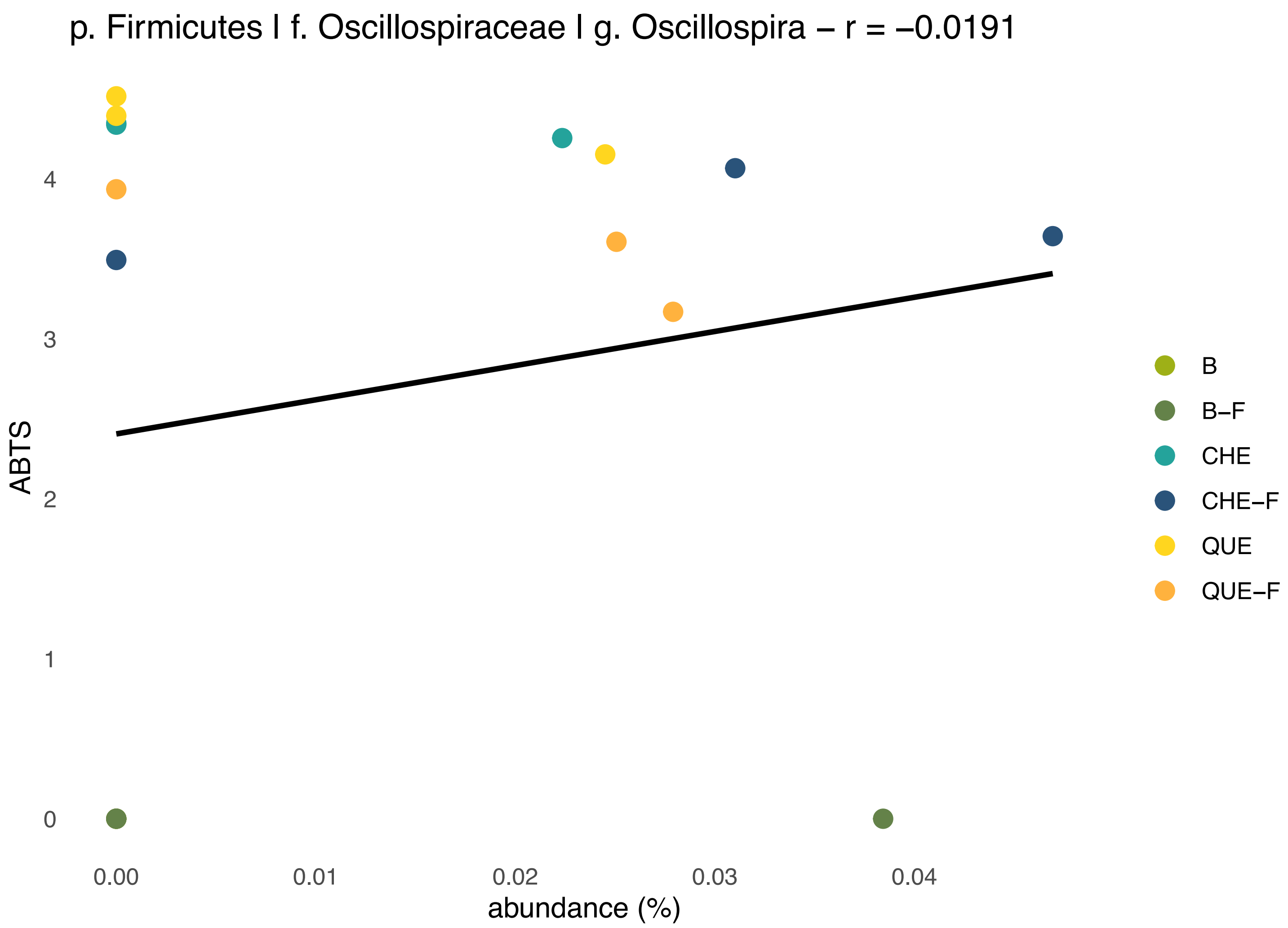
p. Firmicutes I f. Oscillospiraceae I g. Oscillospira – r = –0.0191
ABTS
4
3
2
1
0
0.00
0.01
0.02
0.03
0.04
abundance (%)
B
B–F
CHE
CHE–F
QUE
QUE–F

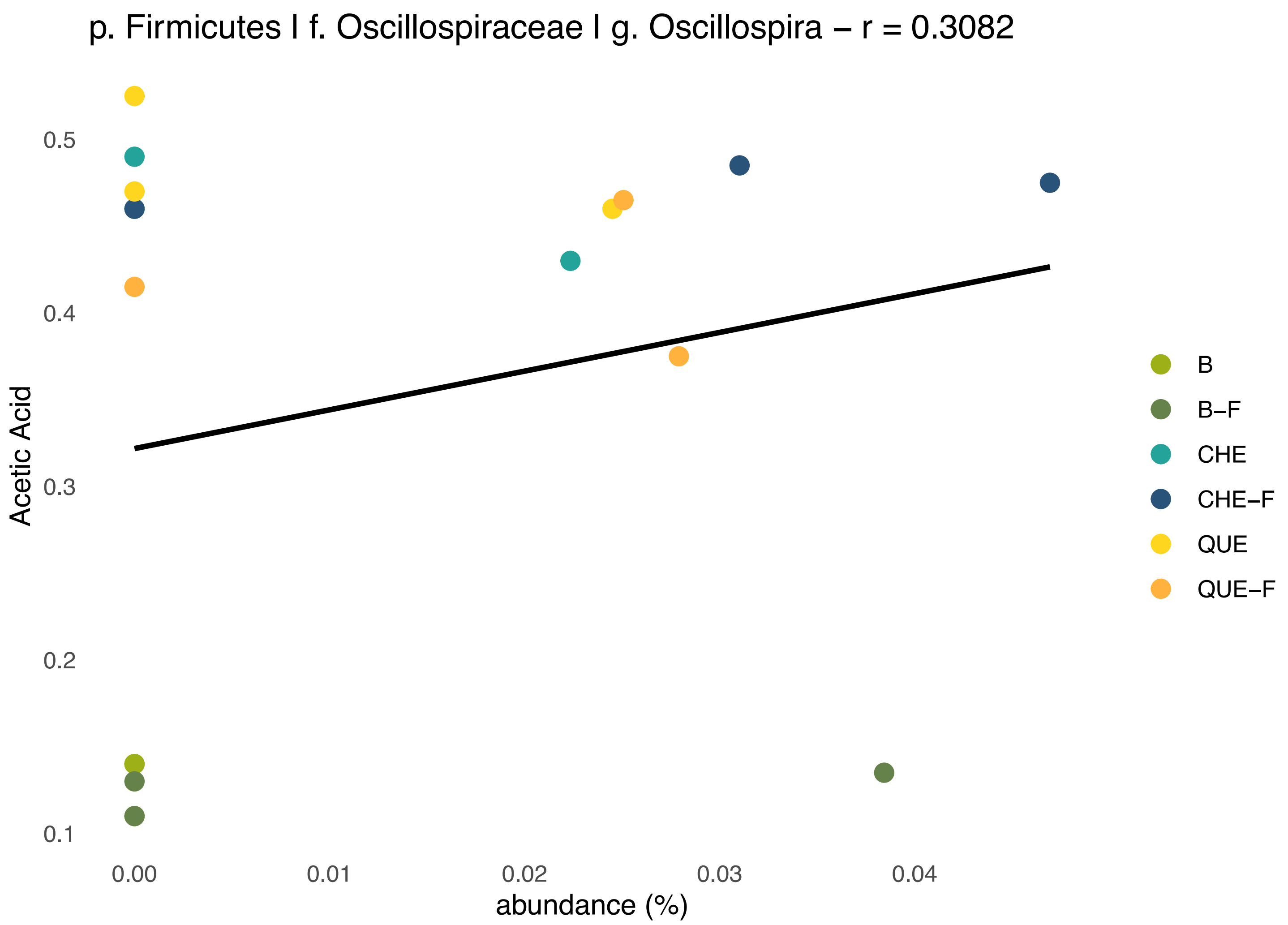

p. Firmicutes | f. Oscillospiraceae | g. Oscillospira – r = 0.3082
Acetic Acid
0.5
0.4
0.3
0.2
0.1
0.00
0.01
0.02
0.03
0.04
abundance (%)
B
B–F
CHE
CHE–F
QUE
QUE–F

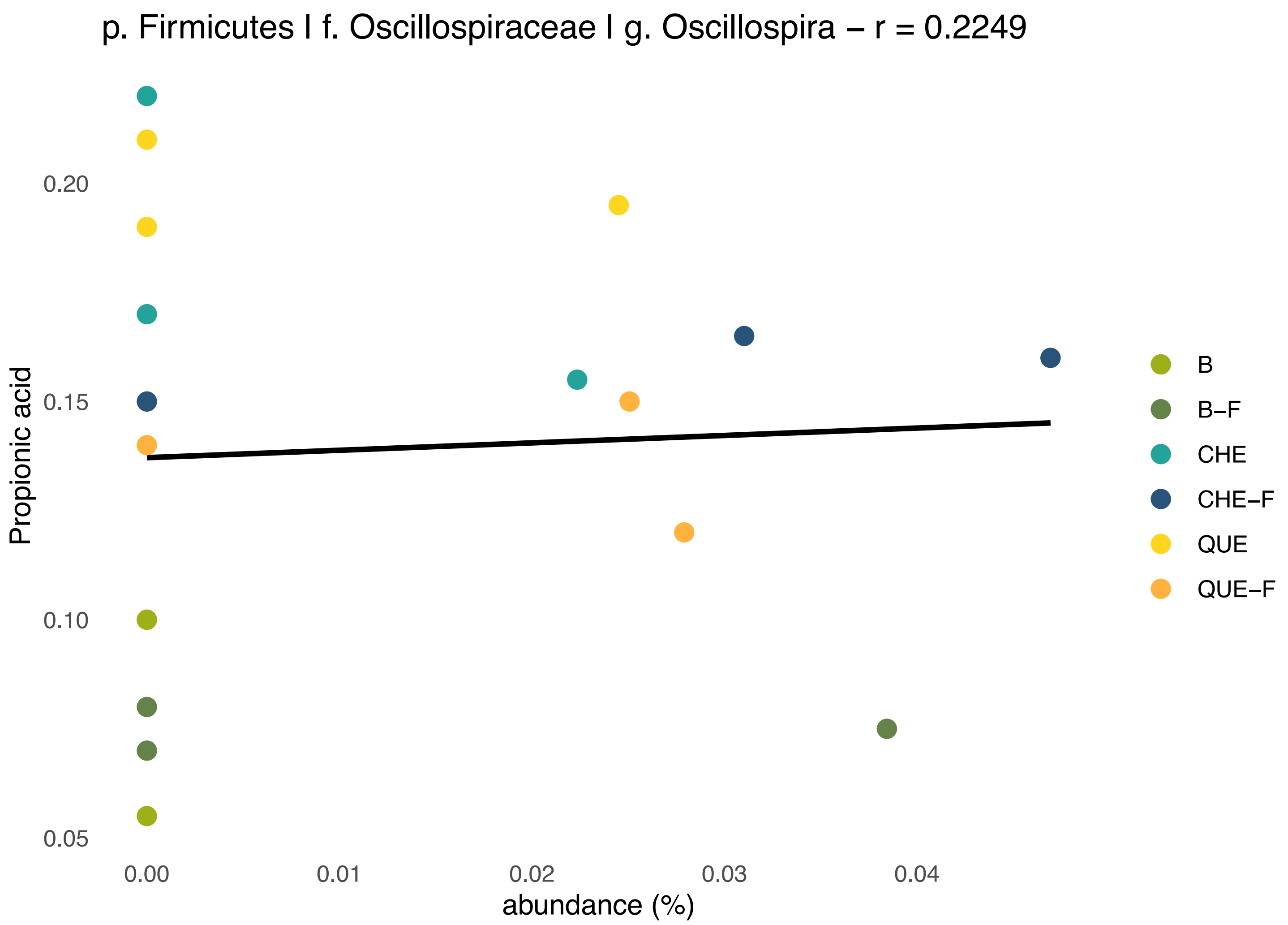
p. Firmicutes I f. Oscillospiraceae I g. Oscillospira – r = 0.2249
Propionic acid
0.20
0.15
0.10
0.05
0.00
0.01
0.02
0.03
0.04
abundance (%)
B
B–F
CHE
CHE–F
QUE
QUE–F

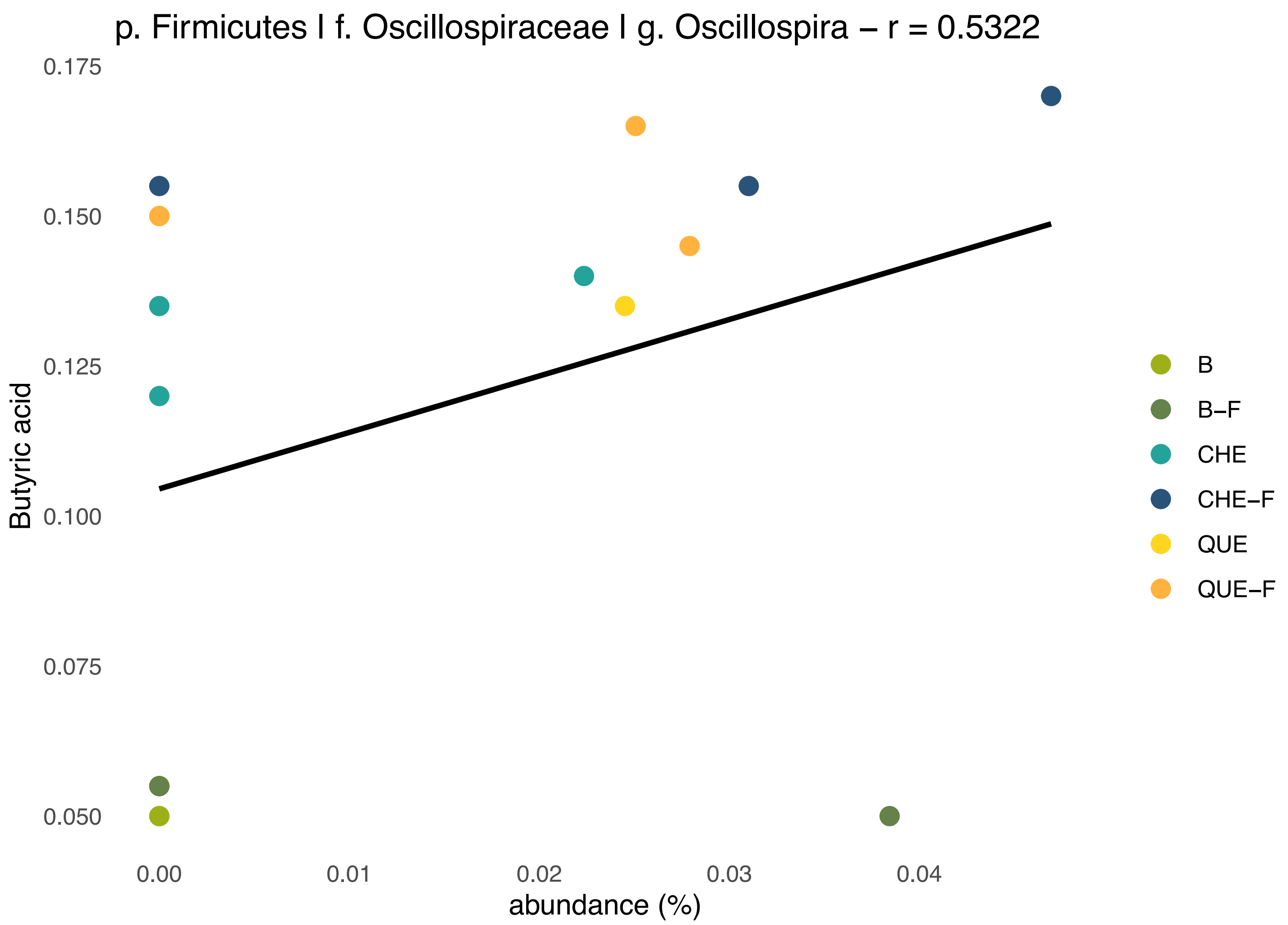

p. Firmicutes I f. Oscillospiraceae I g. Oscillospira – r = 0.5322
Butyric acid
0.175
0.150
0.125
0.100
0.075
0.050
0.00
0.01
0.02
0.03
0.04
abundance (%)
B
B–F
CHE
CHE–F
QUE
QUE–F

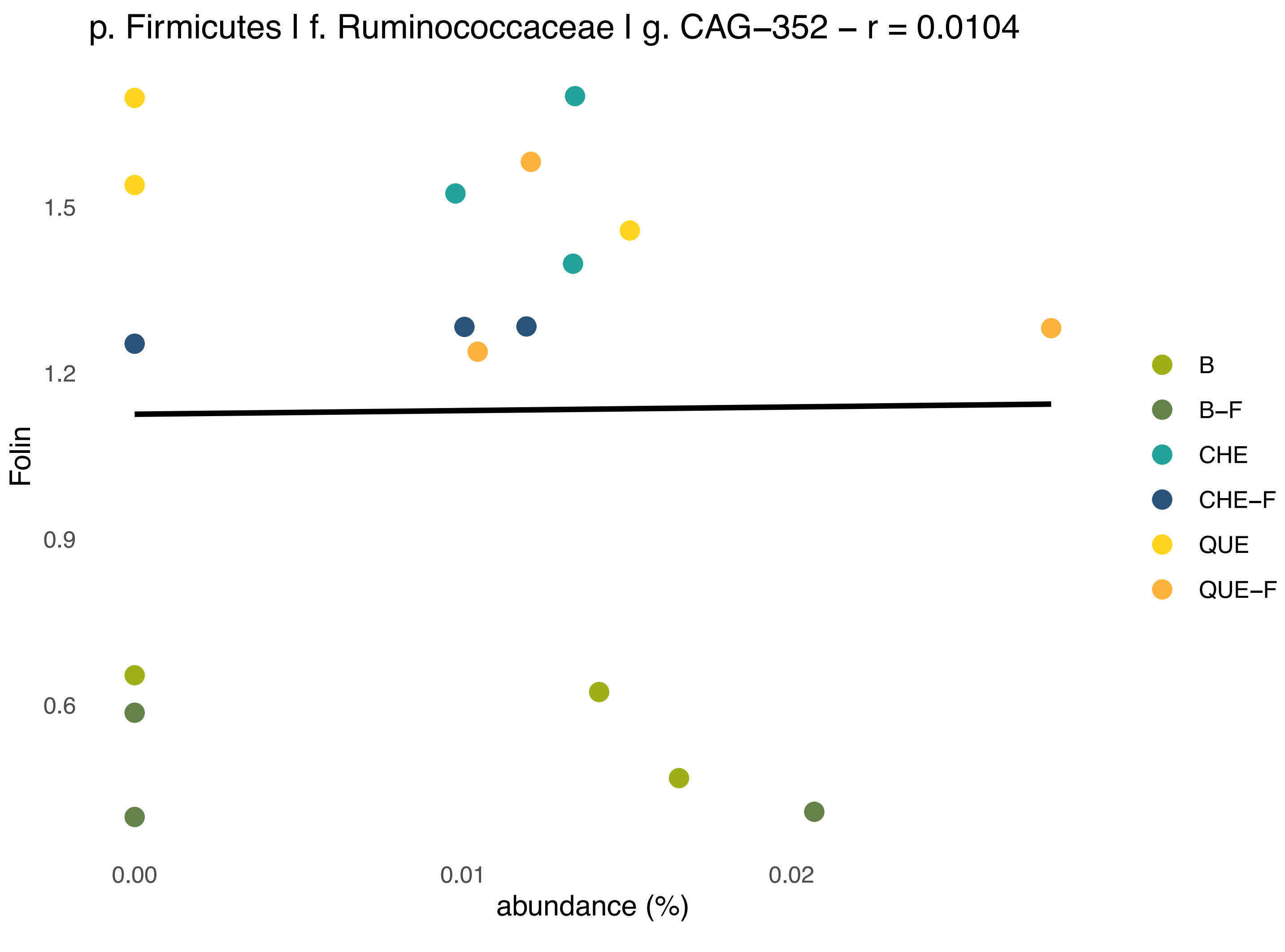
p. Firmicutes I f. Ruminococcaceae I g. CAG−352 − r = 0.0104
Folin
1.5
1.2
0.9
0.6
0.00
0.01
0.02
abundance (%)
B
B−F
CHE
CHE−F
QUE
QUE−F

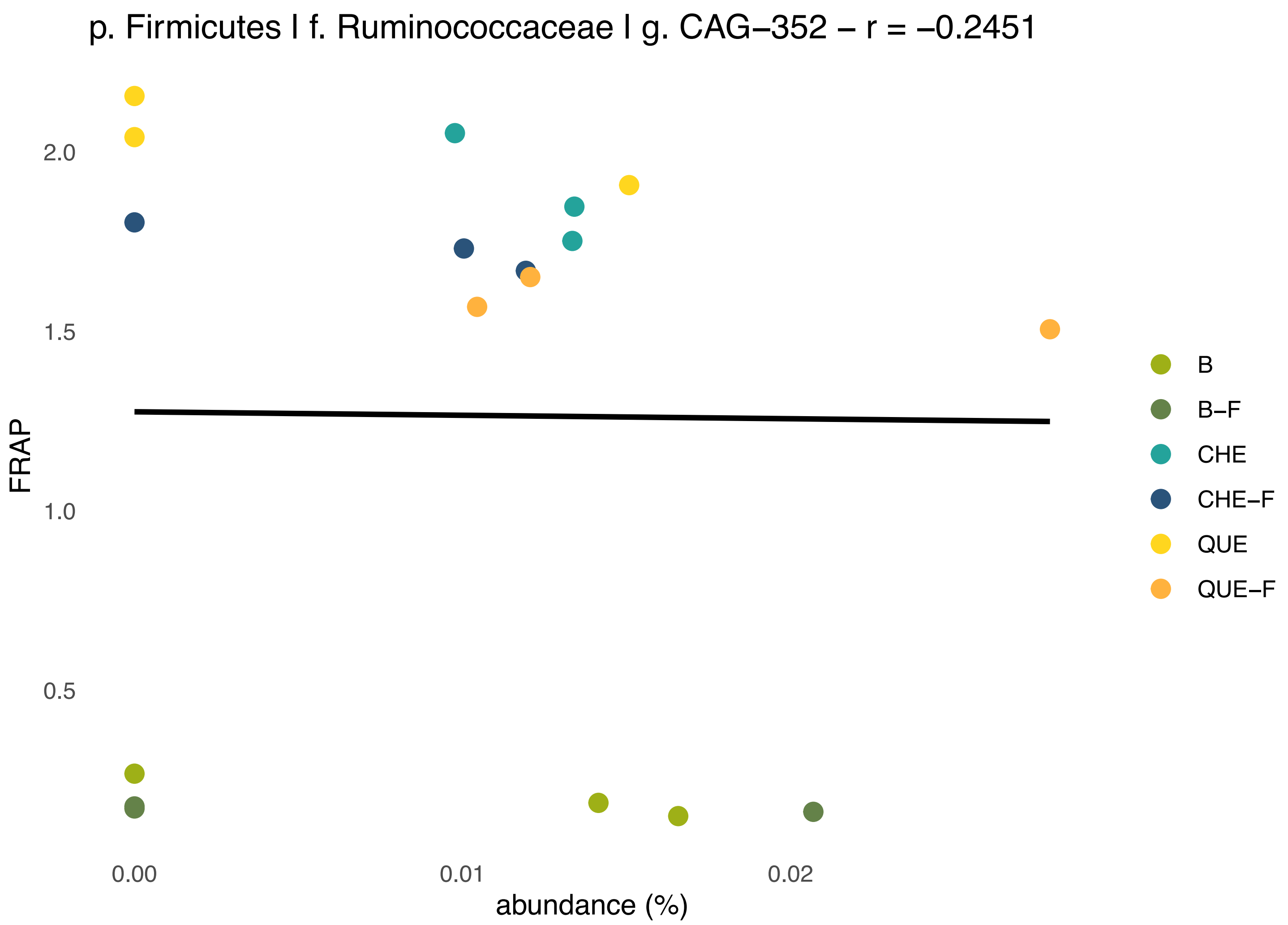

p. Firmicutes I f. Ruminococcaceae I g. CAG−352 − r = −0.2451
2.0
1.5
1.0
0.5
FRAP
0.00
0.01
0.02
abundance (%)
B
B−F
CHE
CHE−F
QUE
QUE−F

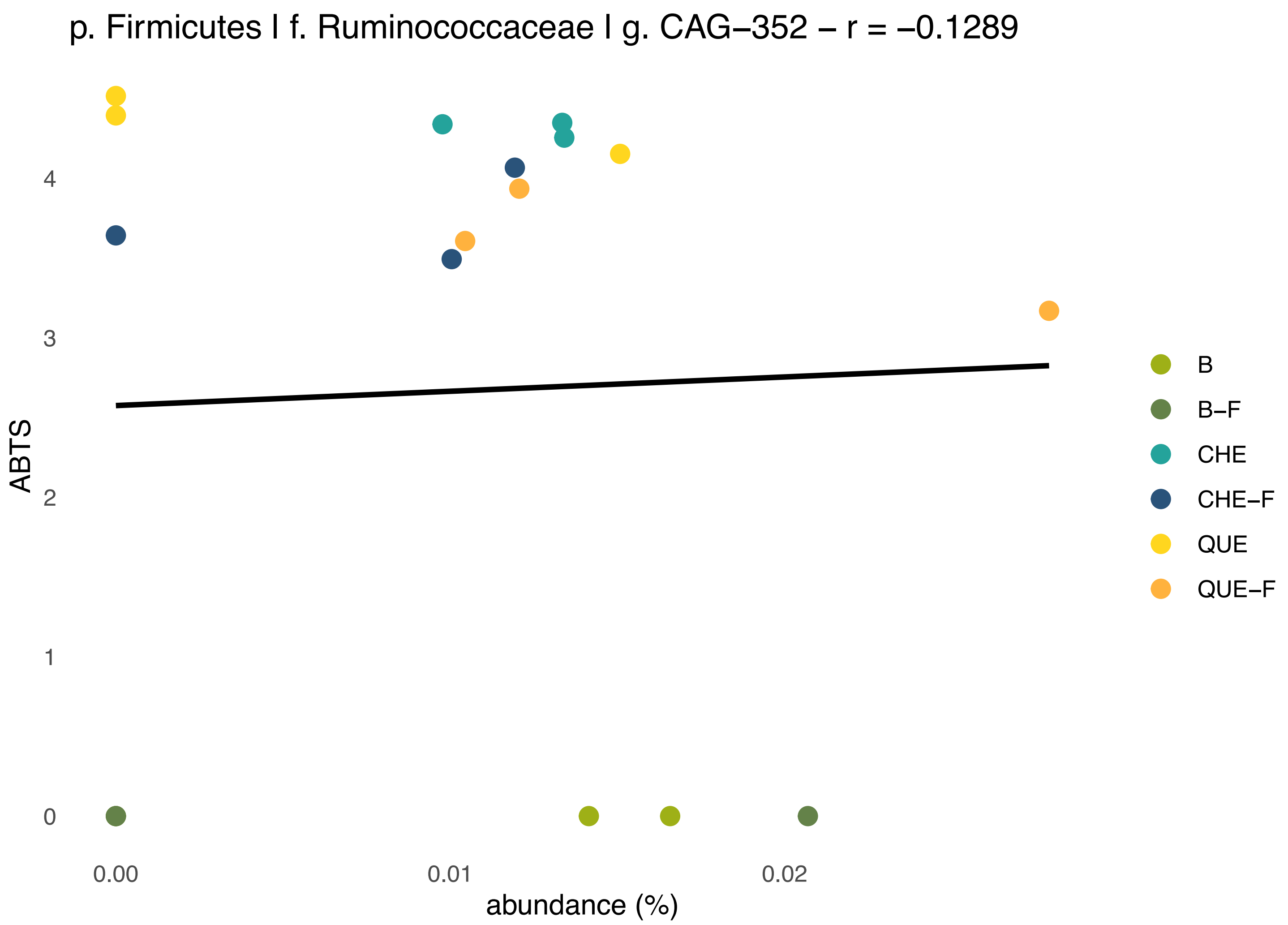

p. Firmicutes I f. Ruminococcaceae I g. CAG−352 − r = −0.1289
ABTS
4
3
2
1
0
0.00
0.01
0.02
abundance (%)
B
B−F
CHE
CHE−F
QUE
QUE−F

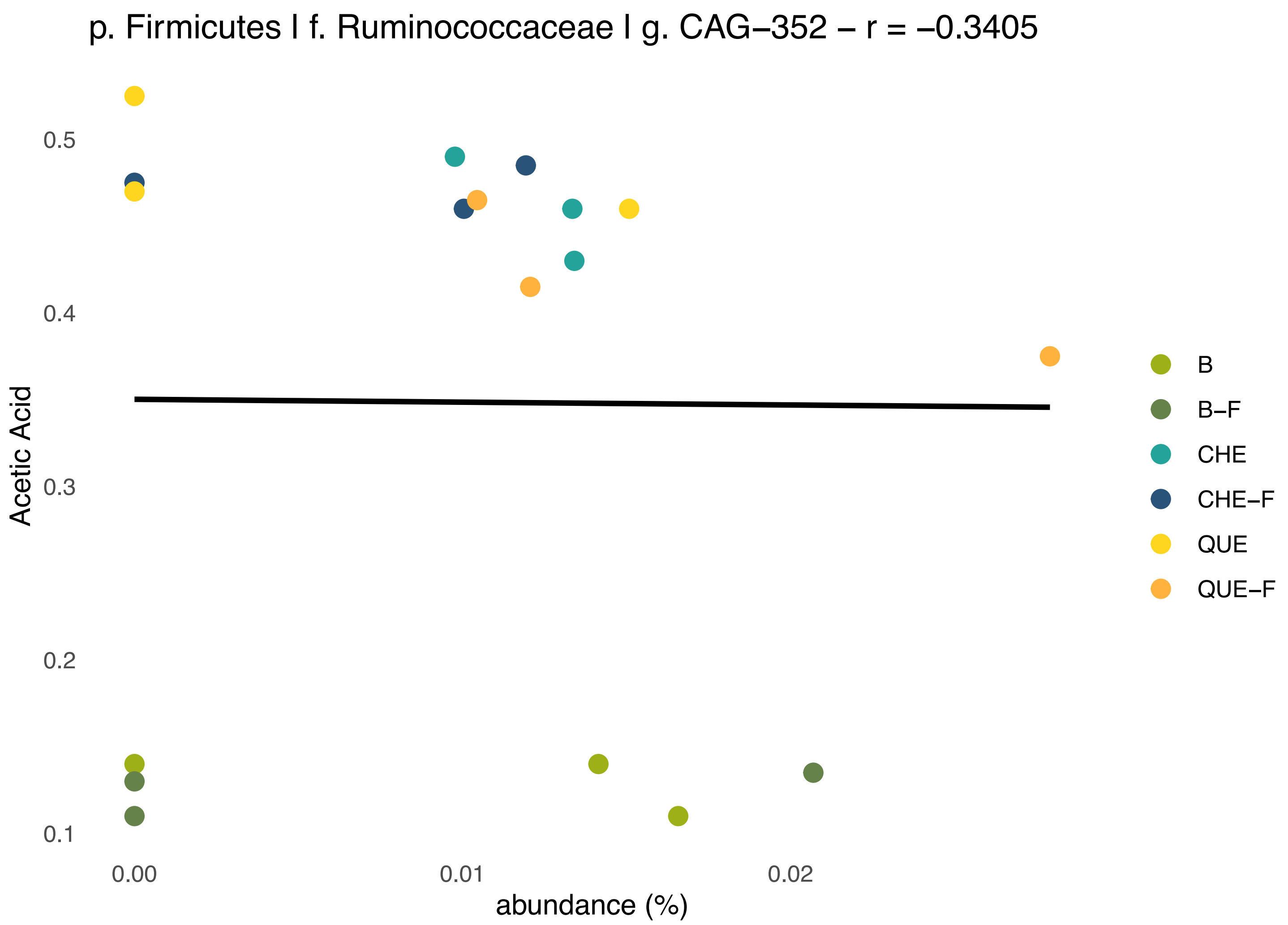
p. Firmicutes I f. Ruminococcaceae I g. CAG−352 − r = −0.3405
Acetic Acid
0.5
0.4
0.3
0.2
0.1
0.00
0.01
0.02
abundance (%)
B
B−F
CHE
CHE−F
QUE
QUE−F

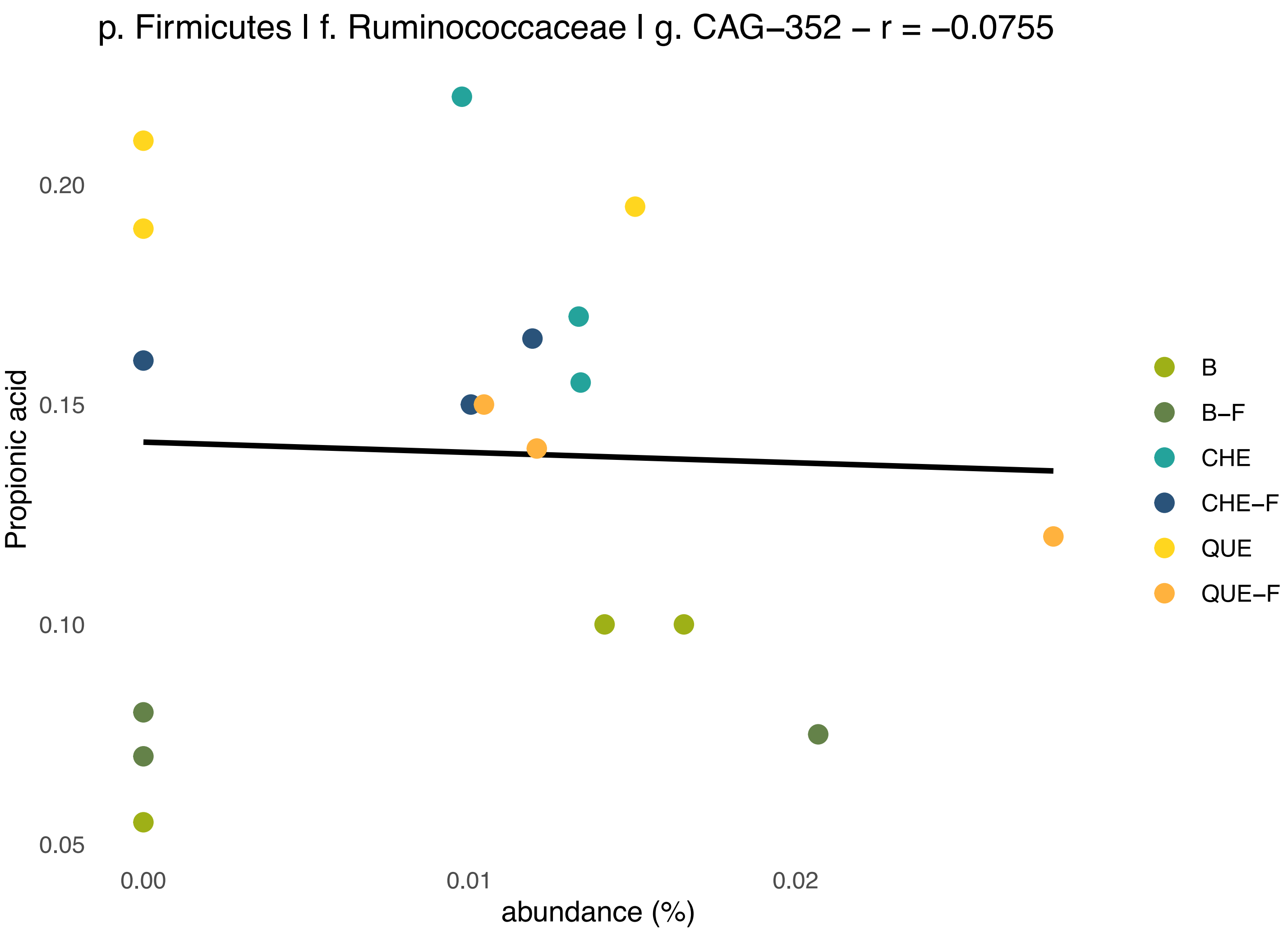

p. Firmicutes I f. Ruminococcaceae I g. CAG−352 − r = −0.0755
Propionic acid
0.05
0.10
0.15
0.20
0.00
0.01
0.02
abundance (%)
B
B−F
CHE
CHE−F
QUE
QUE−F

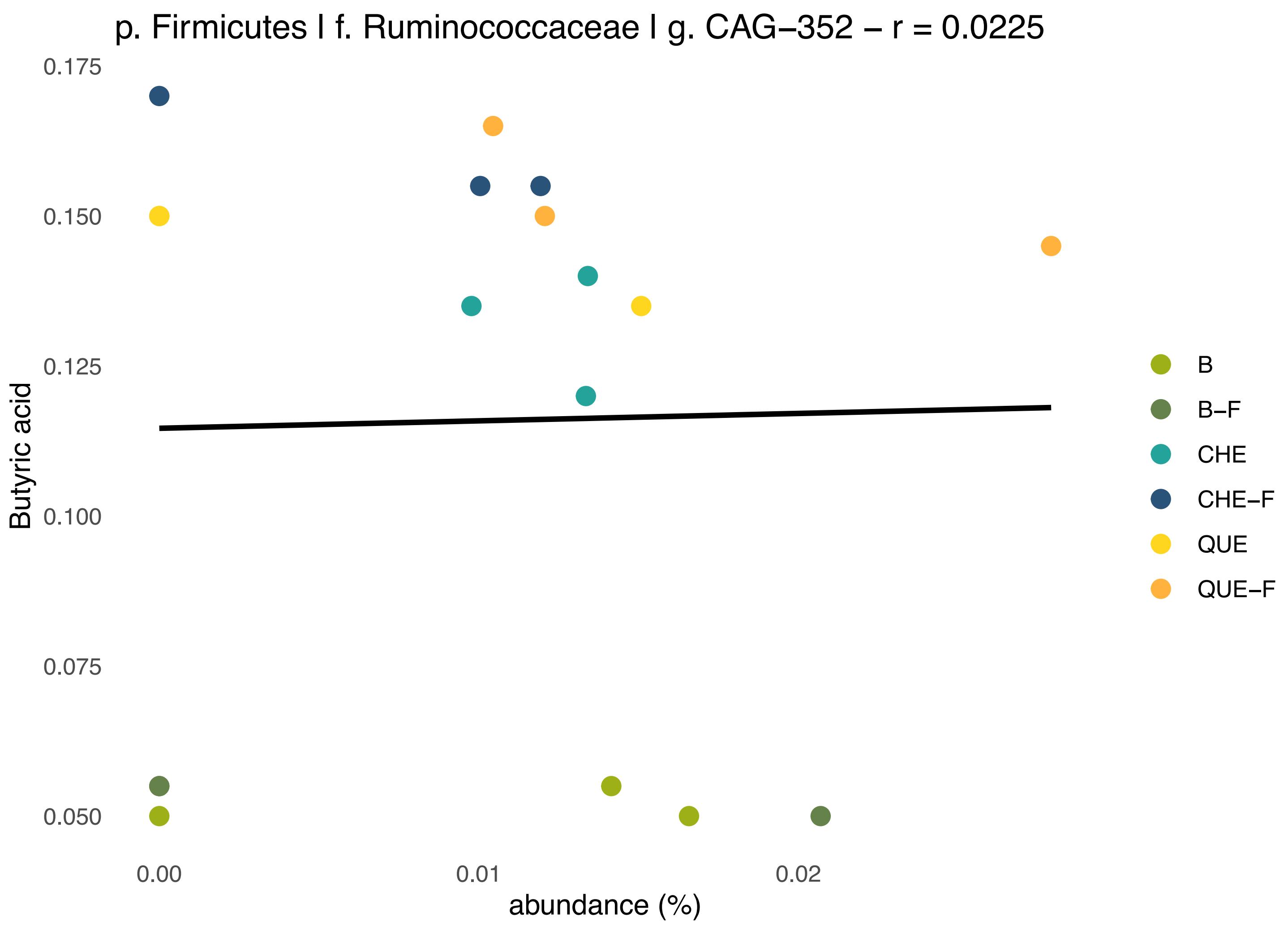

p. Firmicutes I f. Ruminococcaceae I g. CAG–352 – r = 0.0225
Butyric acid
0.175
0.150
0.125
0.100
0.075
0.050
0.00
0.01
0.02
abundance (%)
B
B–F
CHE
CHE–F
QUE
QUE–F

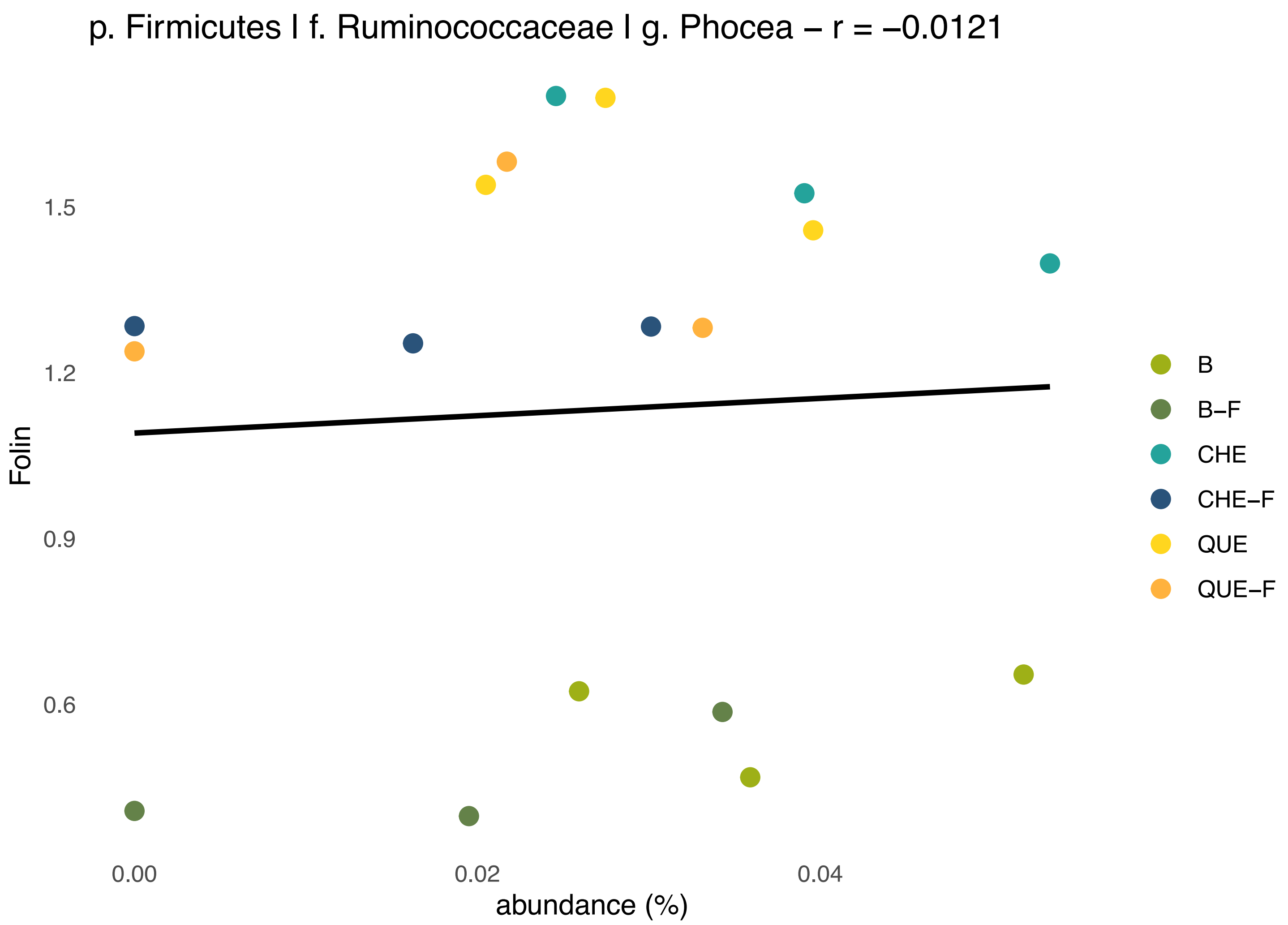
p. Firmicutes l f. Ruminococcaceae l g. Phocea – r = −0.0121
1.5
1.2
0.9
0.6
Folin
0.00
0.02
0.04
abundance (%)
B
B−F
CHE
CHE−F
QUE
QUE−F

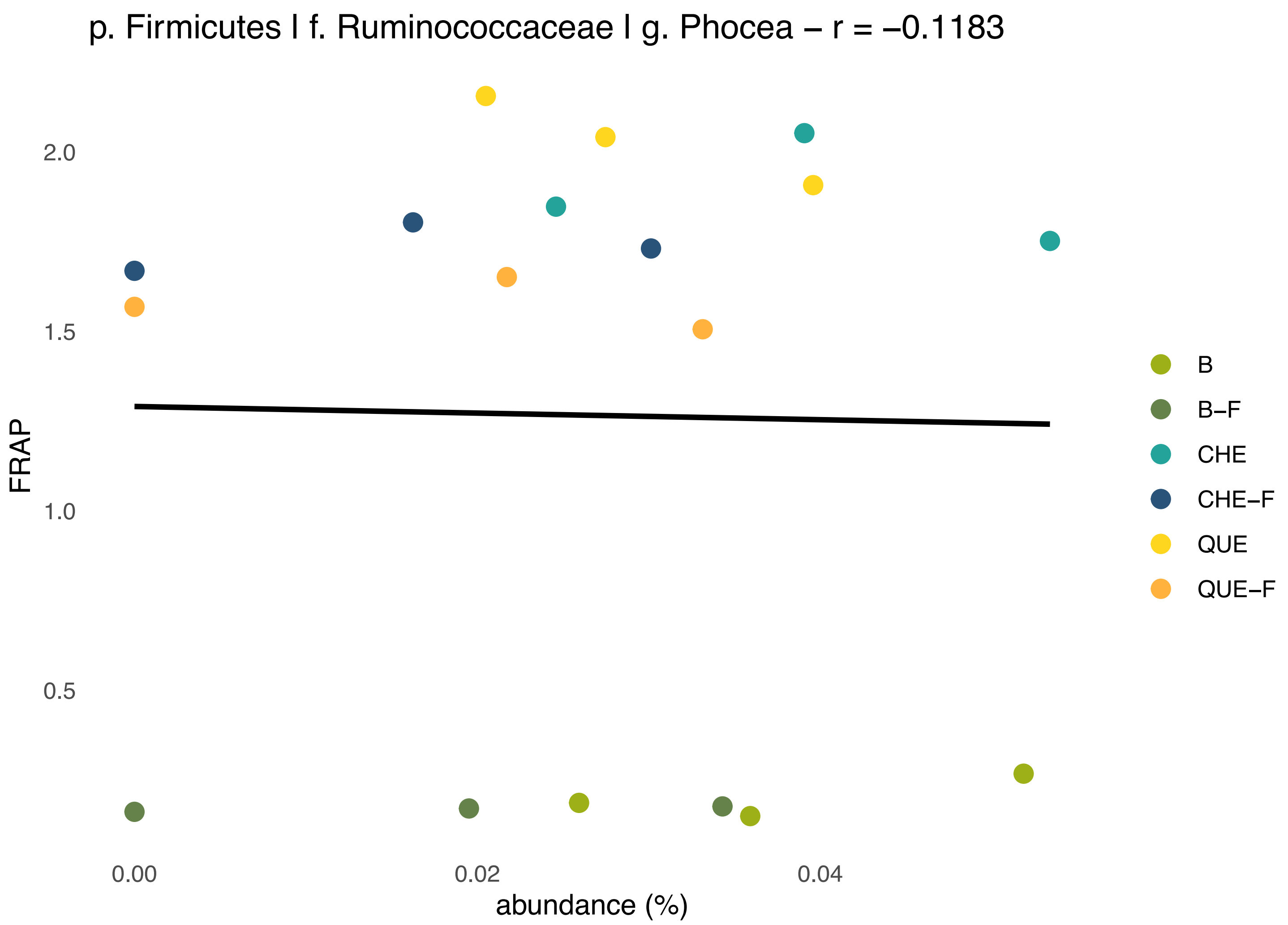

p. Firmicutes I f. Ruminococcaceae I g. Phocea – r = −0.1183
2.0
1.5
1.0
0.5
FRAP
0.00
0.02
0.04
abundance (%)
B
B−F
CHE
CHE−F
QUE
QUE−F

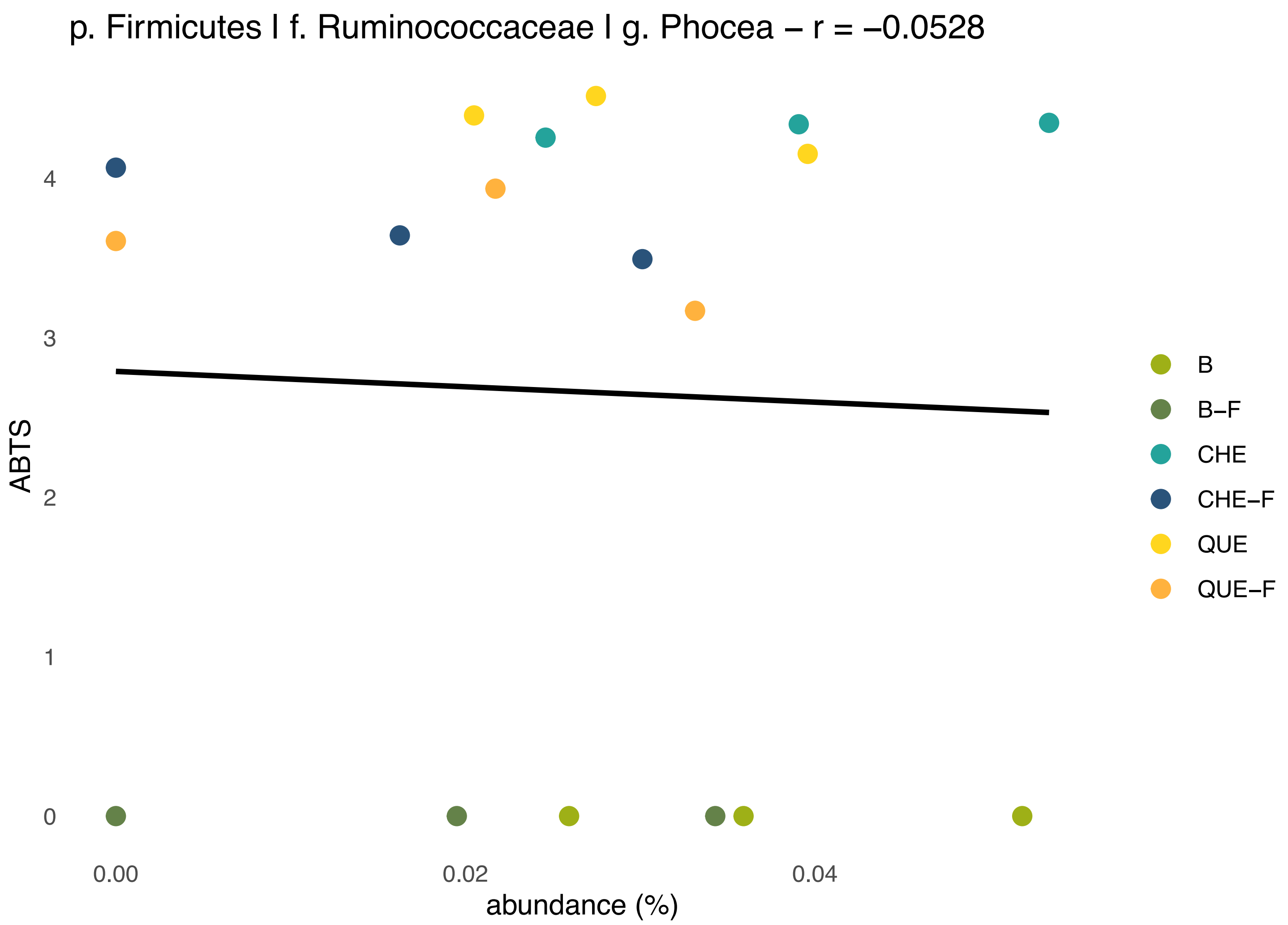
p. Firmicutes I f. Ruminococcaceae I g. Phocea – r = –0.0528
ABTS
4
3
2
1
0
0.00
0.02
0.04
abundance (%)
B
B–F
CHE
CHE–F
QUE
QUE–F

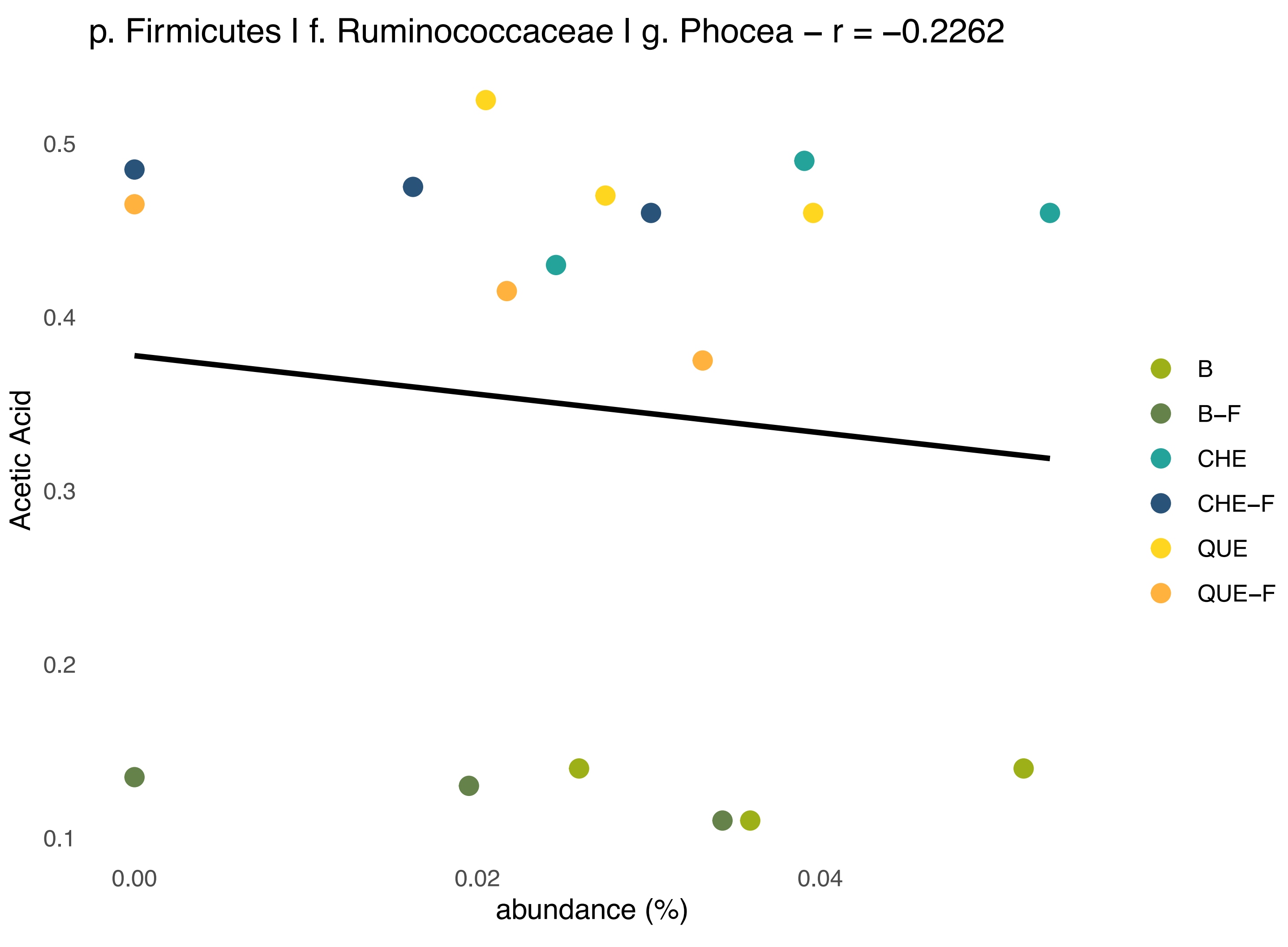
p. Firmicutes | f. Ruminococcaceae | g. Phocea – r = −0.2262
Acetic Acid
0.5
0.4
0.3
0.2
0.1
0.00
0.02
0.04
abundance (%)
B
B−F
CHE
CHE−F
QUE
QUE−F

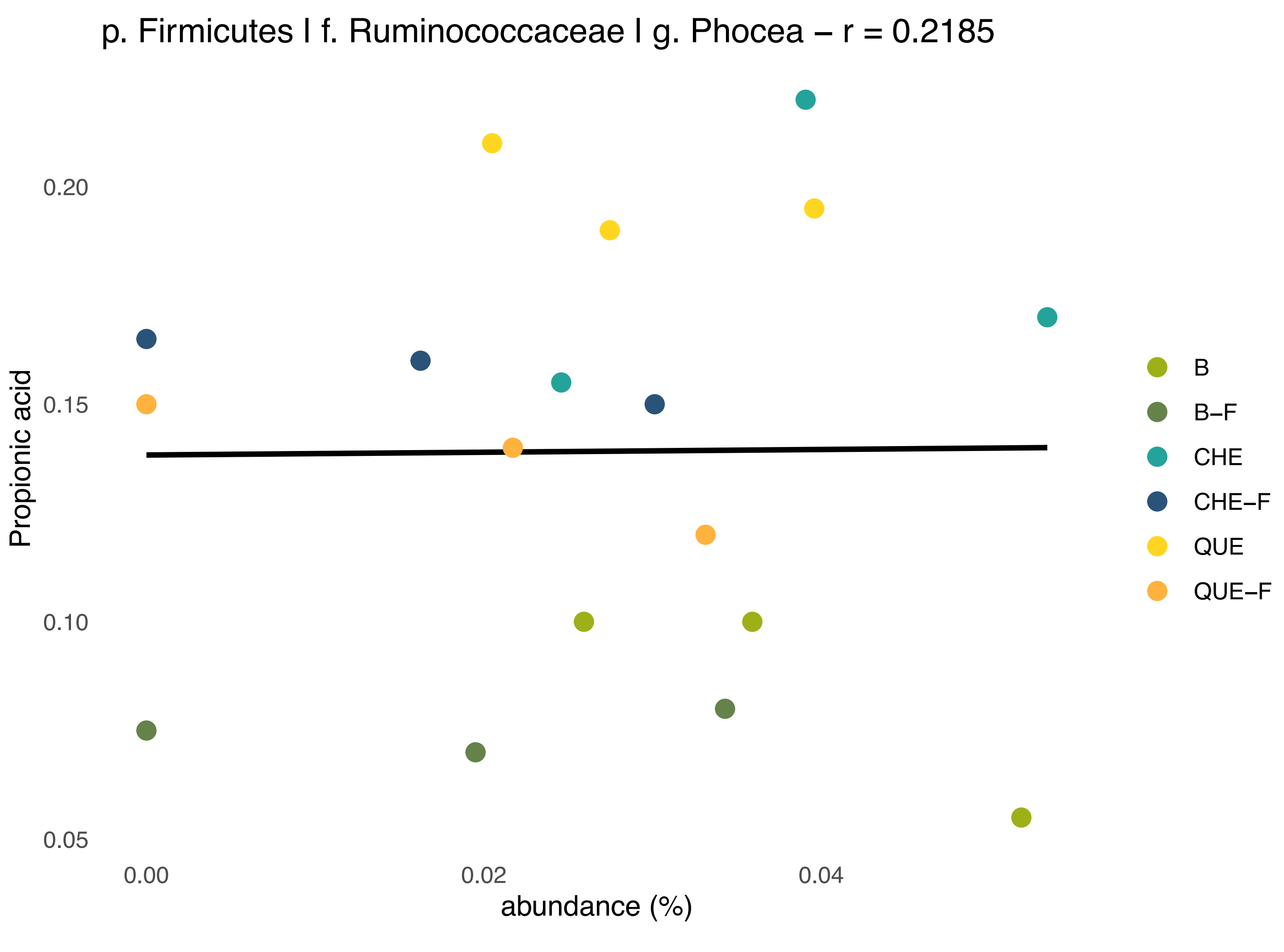
p. Firmicutes l f. Ruminococcaceae l g. Phocea – r = 0.2185
Propionic acid
0.20
0.15
0.10
0.05
0.00
0.02
0.04
abundance (%)
B
B–F
CHE
CHE–F
QUE
QUE–F

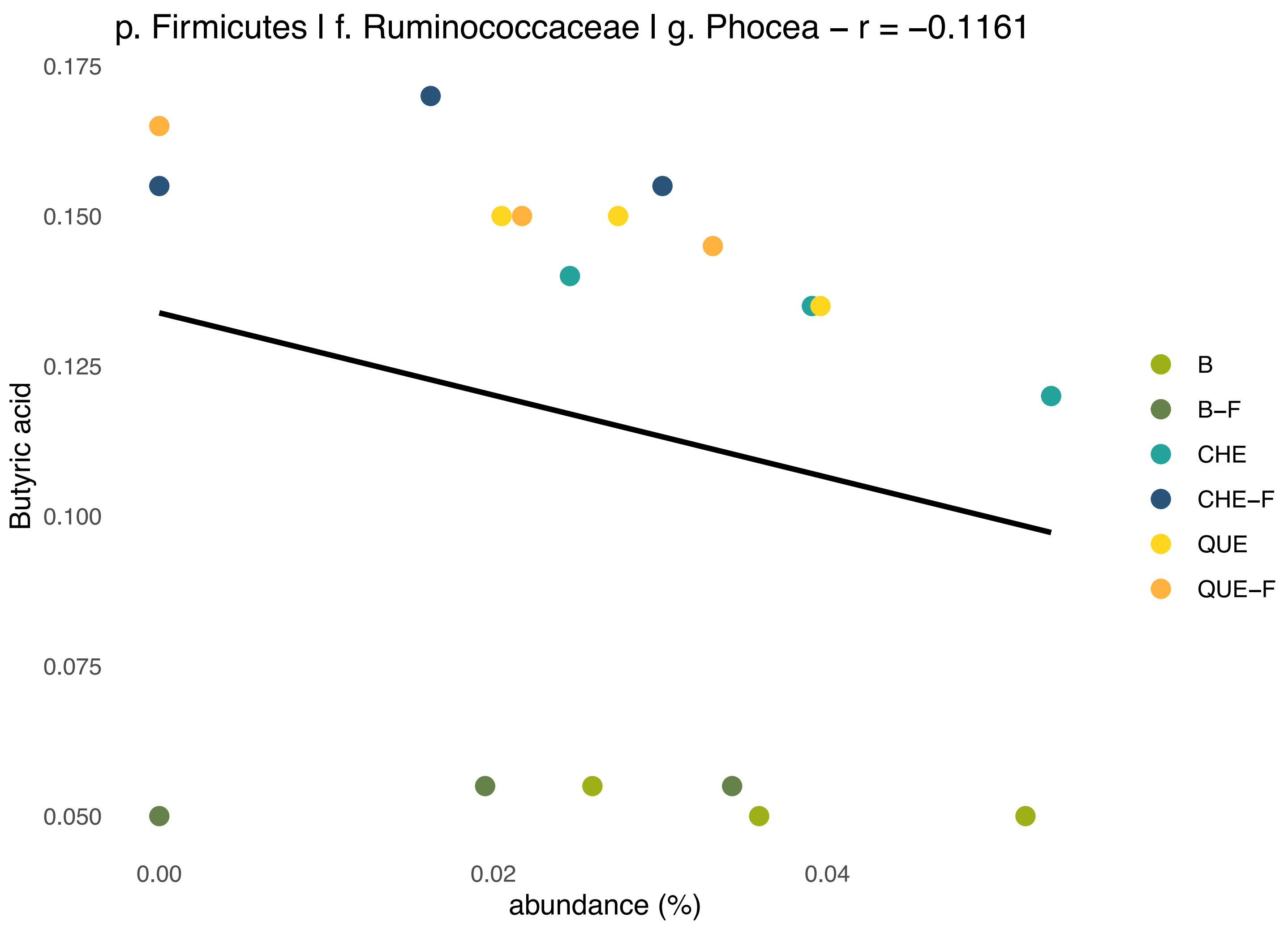

p. Firmicutes I f. Ruminococcaceae I g. Phocea – r = –0.1161
0.175
0.150
0.125
0.100
0.075
0.050
Butyric acid
0.00
0.02
0.04
abundance (%)
B
B–F
CHE
CHE–F
QUE
QUE–F

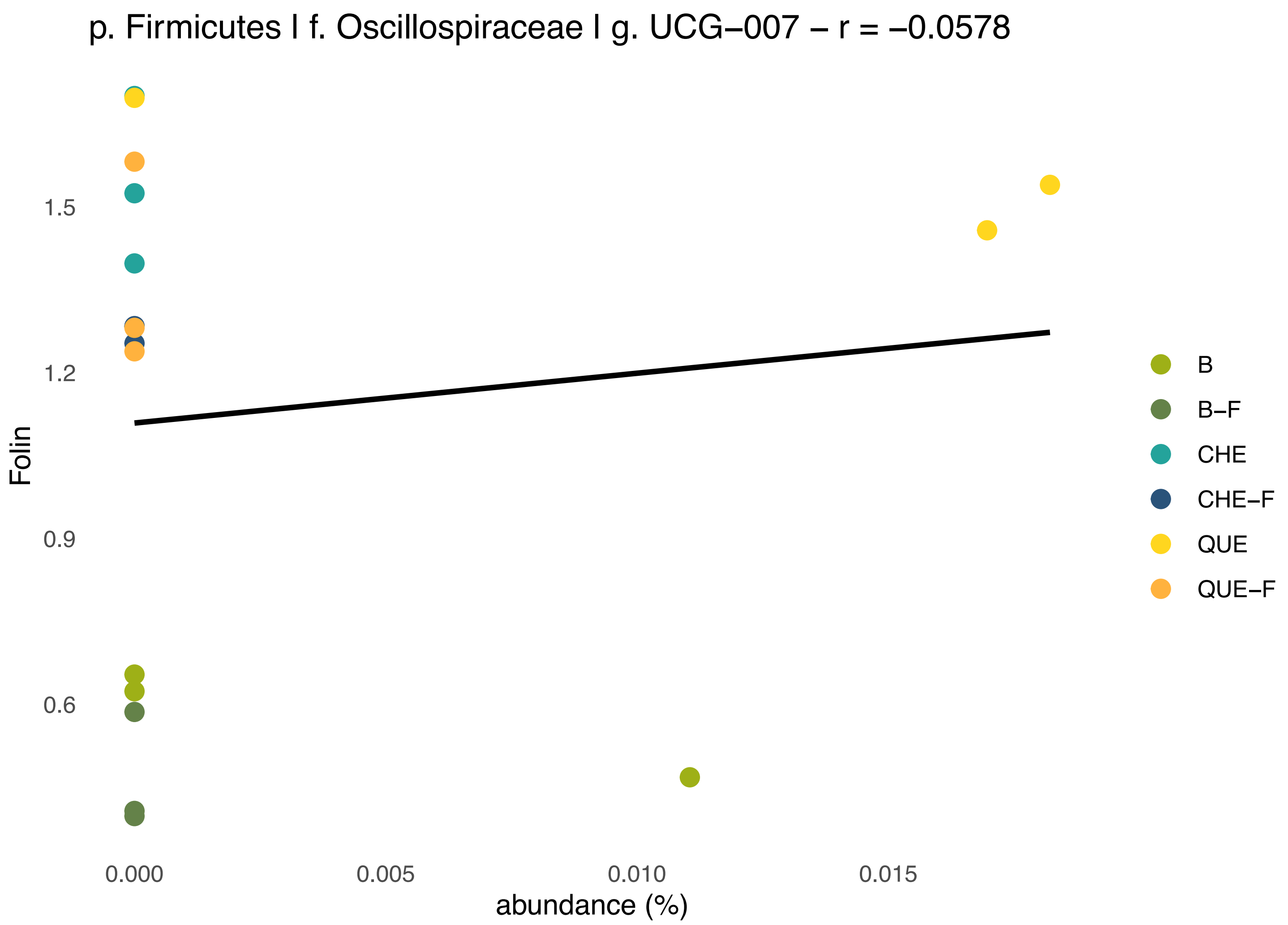

p. Firmicutes I f. Oscillospiraceae I g. UCG–007 – r = –0.0578
Folin
1.5
1.2
0.9
0.6
0.000
0.005
0.010
0.015
abundance (%)
B
B–F
CHE
CHE–F
QUE
QUE–F

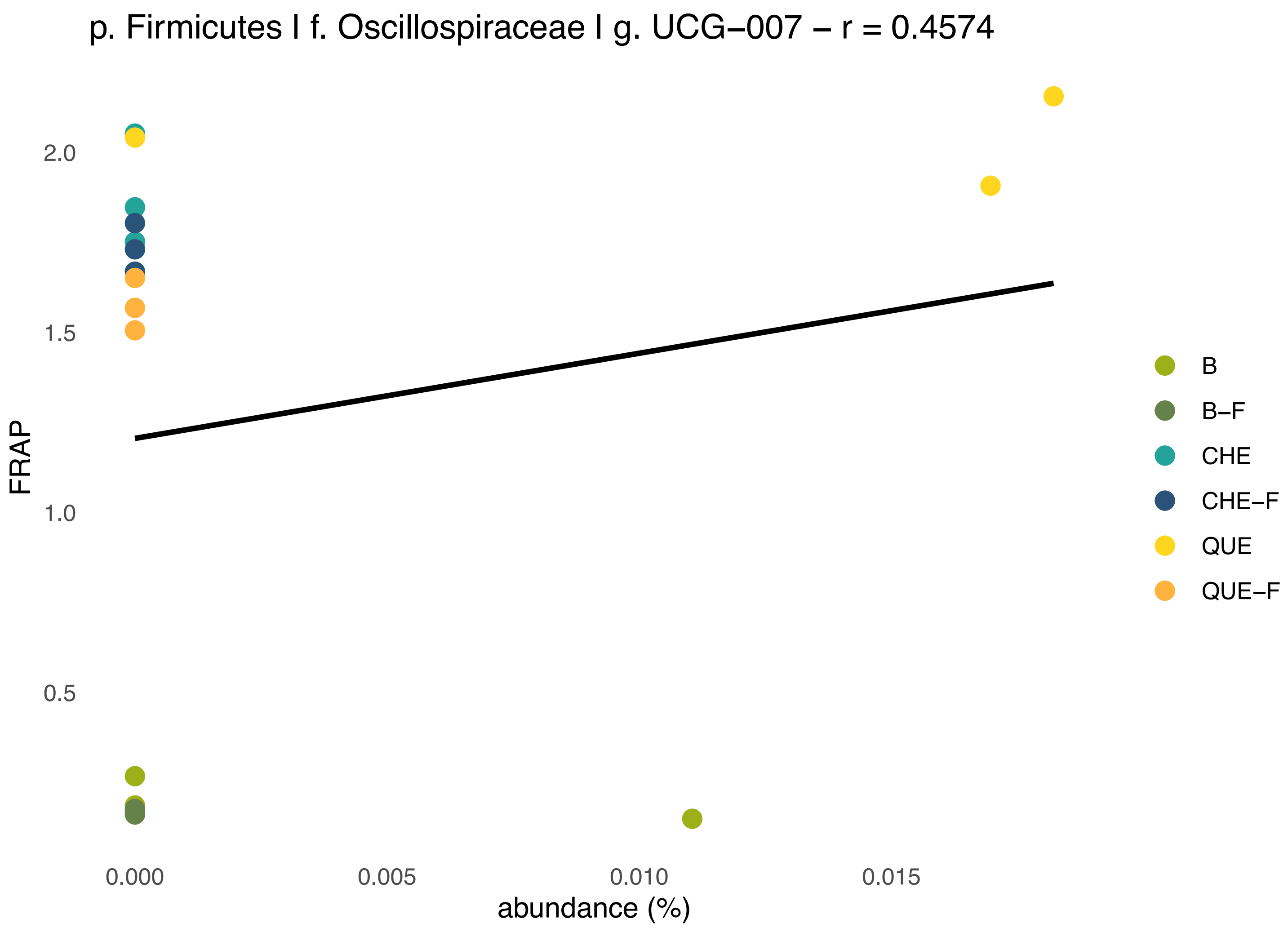

p. Firmicutes I f. Oscillospiraceae I g. UCG−007 − r = 0.4574
FRAP
2.0
1.5
1.0
0.5
0.000
0.005
0.010
0.015
abundance (%)
B
B−F
CHE
CHE−F
QUE
QUE−F

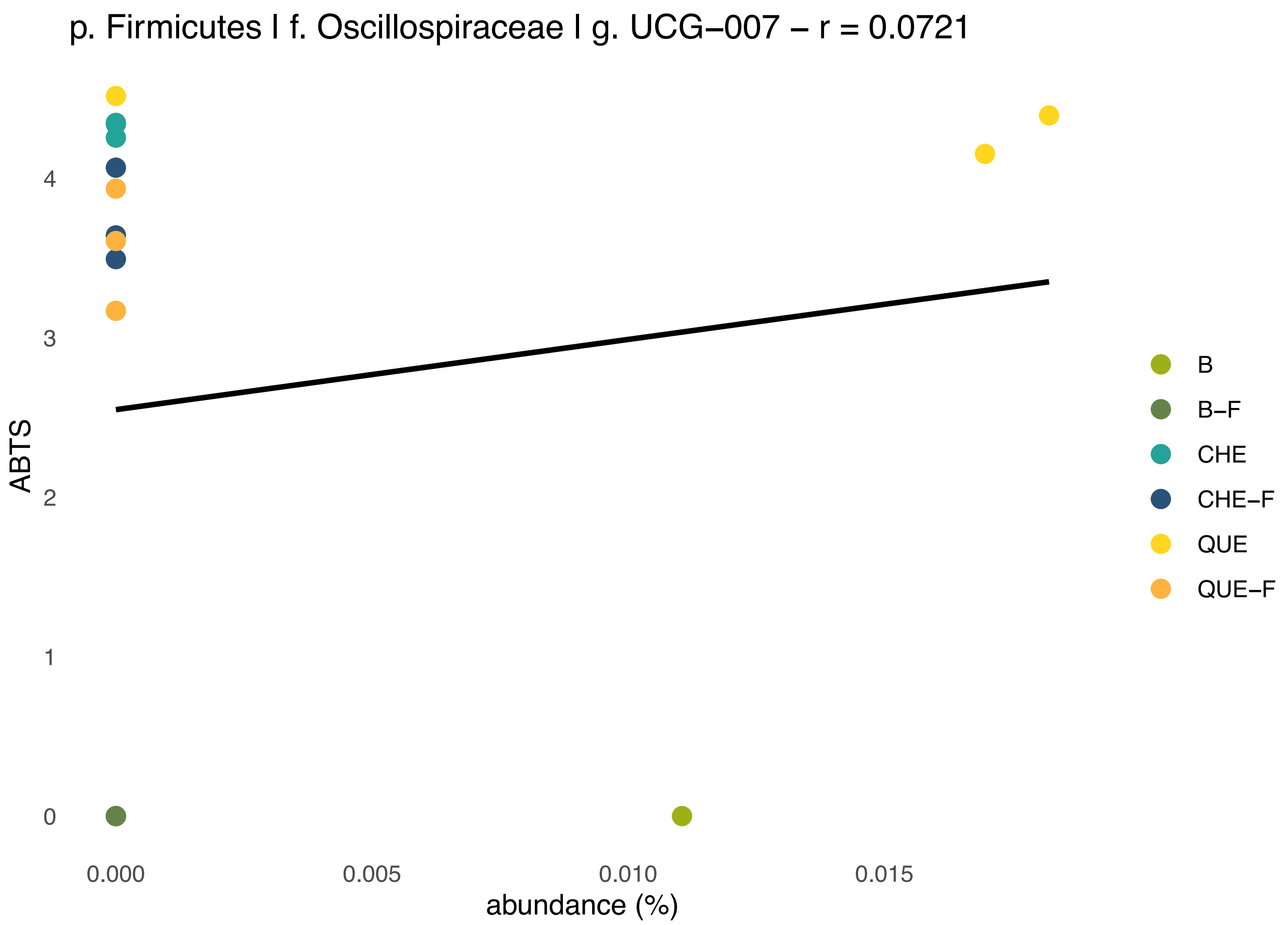

p. Firmicutes I f. Oscillospiraceae I g. UCG−007 − r = 0.0721
ABTS
0
1
2
3
4
0.000
0.005
0.010
0.015
abundance (%)
B
B−F
CHE
CHE−F
QUE
QUE−F

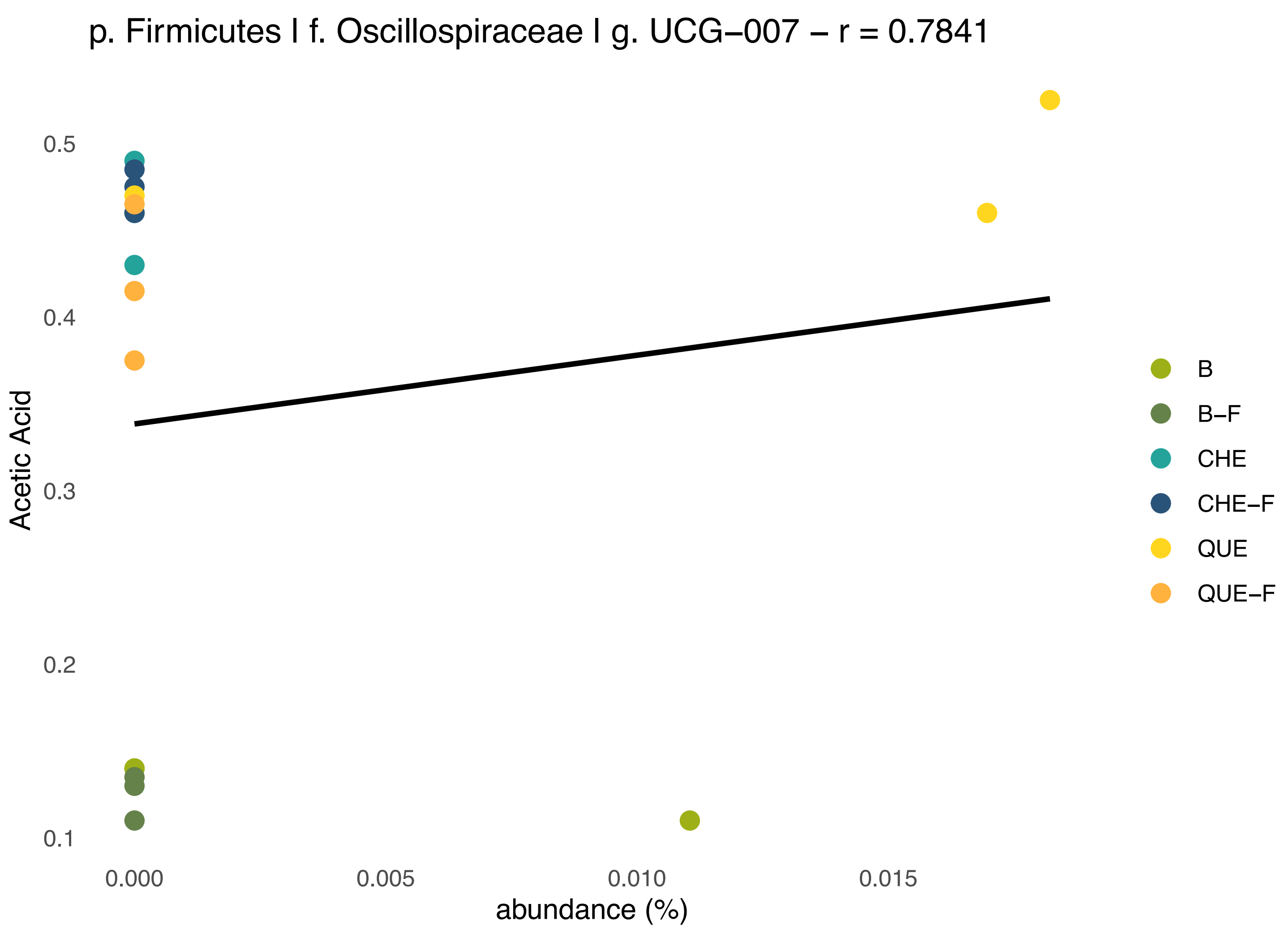
p. Firmicutes I f. Oscillospiraceae I g. UCG−007 − r = 0.7841
0.5
0.4
0.3
0.2
0.1
Acetic Acid
0.000
0.005
0.010
0.015
abundance (%)
B
B−F
CHE
CHE−F
QUE
QUE−F

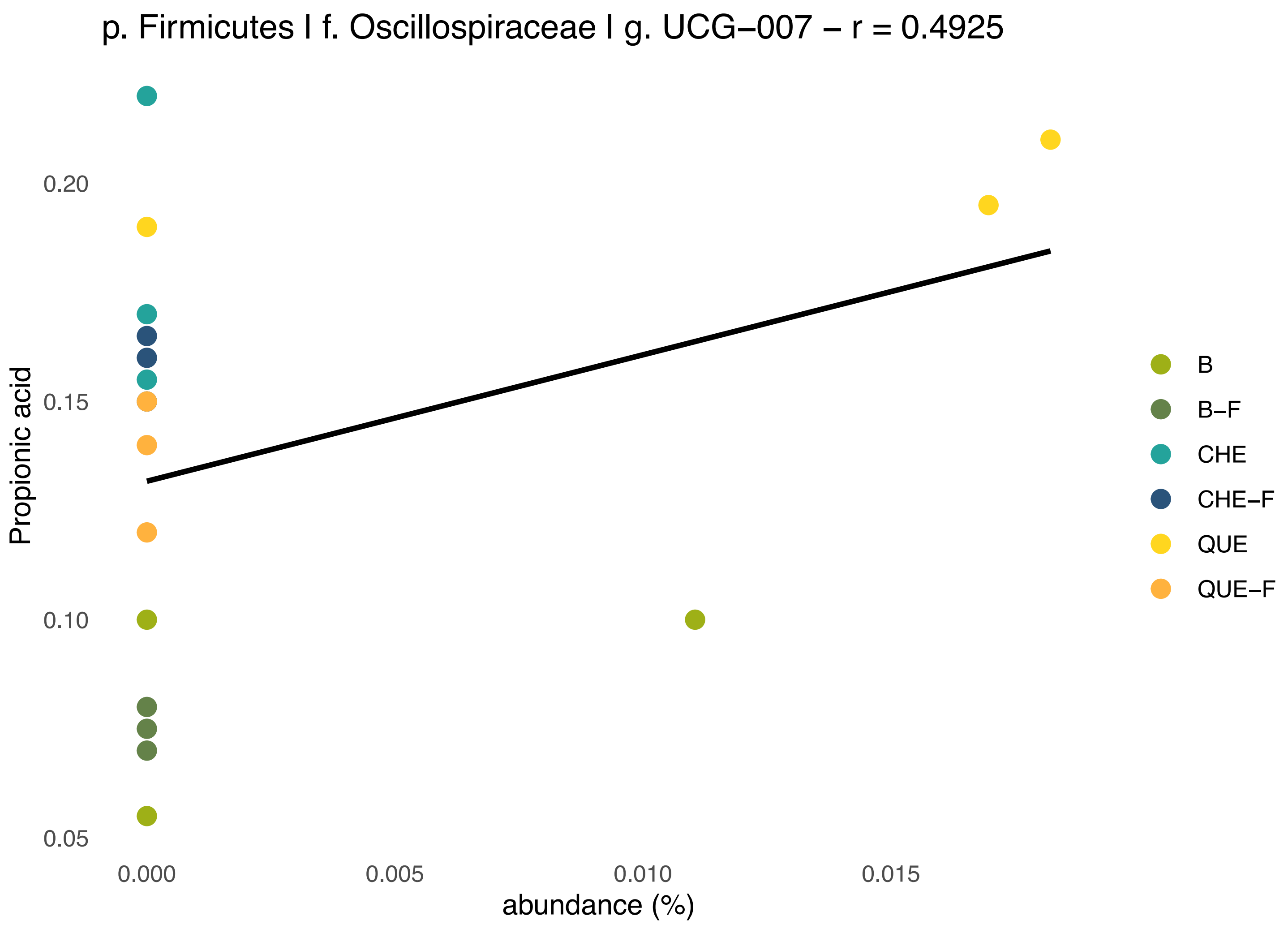

p. Firmicutes l f. Oscillospiraceae l g. UCG−007 − r = 0.4925
Propionic acid
0.20
0.15
0.10
0.05
0.000
0.005
0.010
0.015
abundance (%)
B
B−F
CHE
CHE−F
QUE
QUE−F

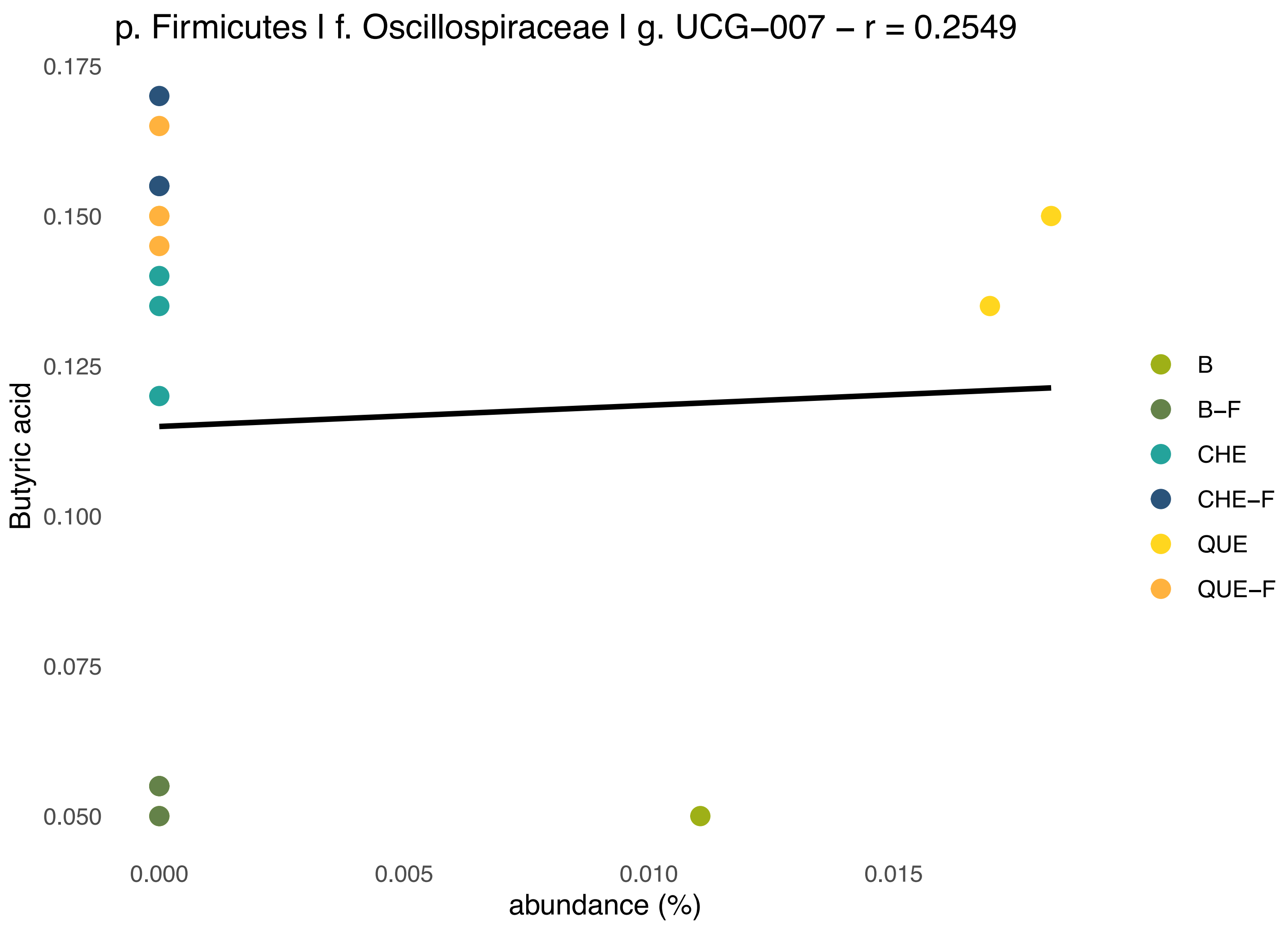
p. Firmicutes I f. Oscillospiraceae I g. UCG−007 − r = 0.2549
0.175
0.150
0.125
0.100
0.075
0.050
Butyric acid
0.000
0.005
0.010
0.015
abundance (%)
B
B−F
CHE
CHE−F
QUE
QUE−F

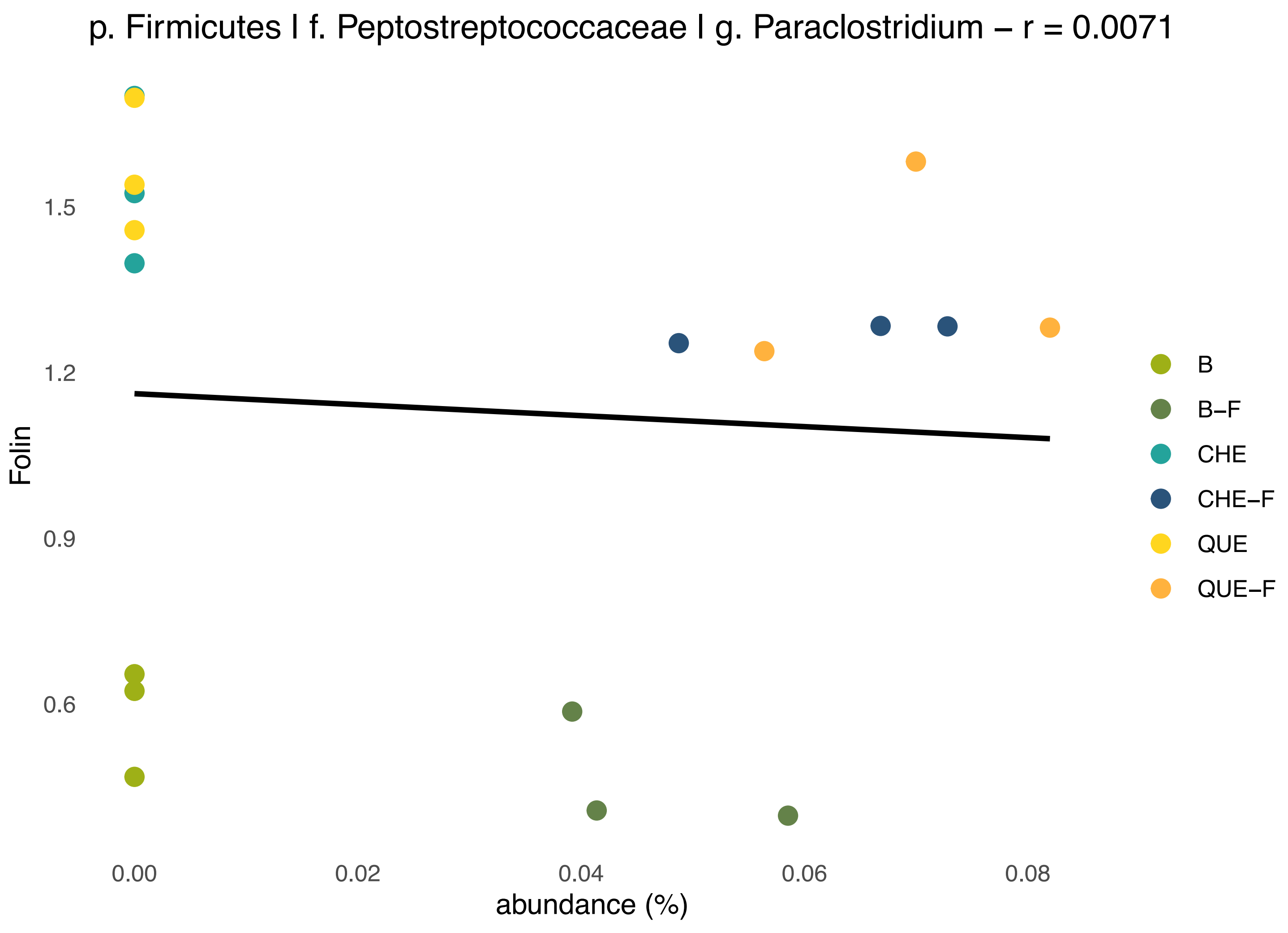

p. Firmicutes I f. Peptostreptococcaceae I g. Paraclostridium – r = 0.0071
Folin
1.5
1.2
0.9
0.6
0.00
0.02
0.04
0.06
0.08
abundance (%)
B
B–F
CHE
CHE–F
QUE
QUE–F

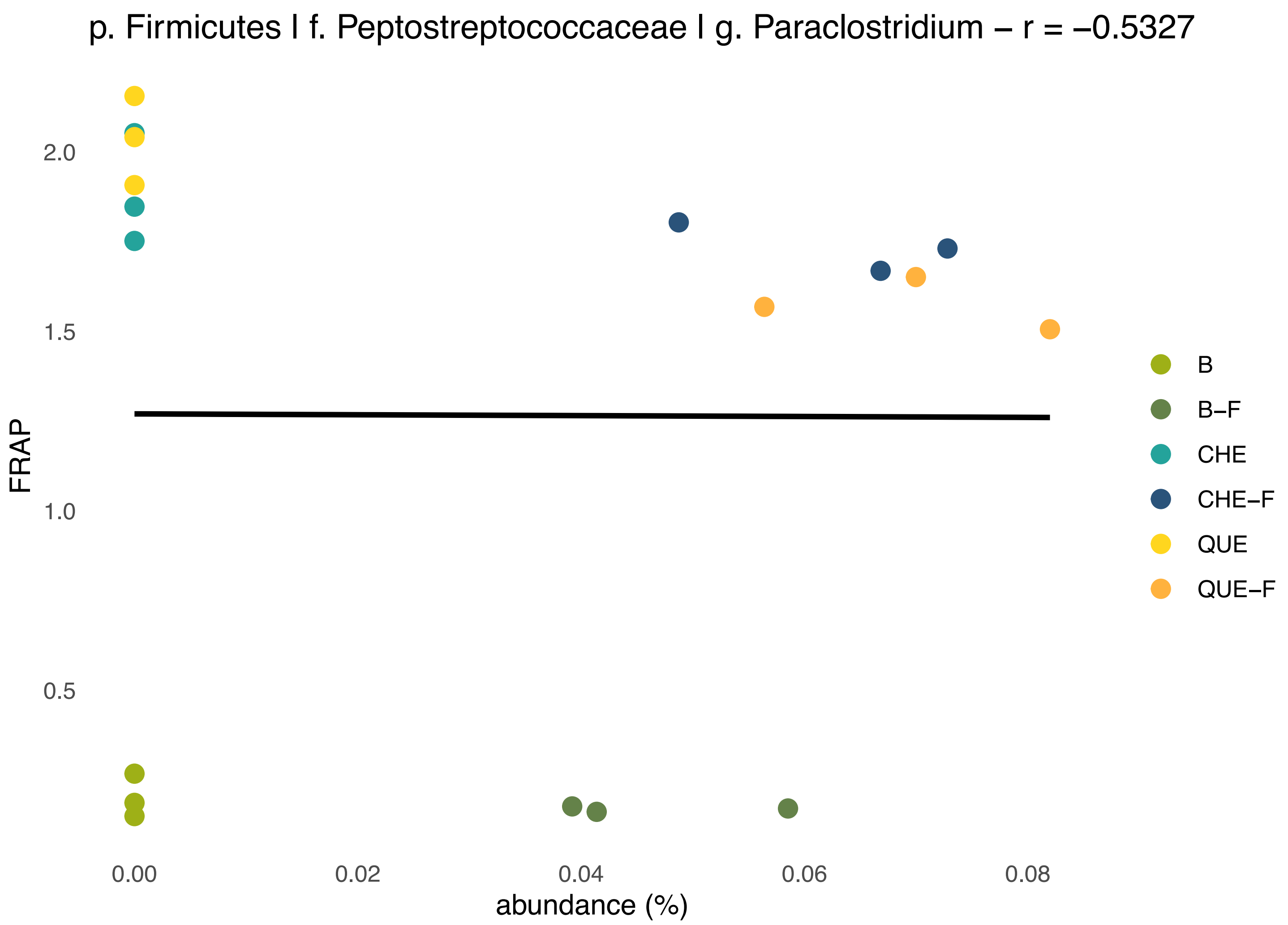
p. Firmicutes I f. Peptostreptococcaceae I g. Paraclostridium – r = –0.5327
FRAP
2.0
1.5
1.0
0.5
0.00
0.02
0.04
0.06
0.08
abundance (%)
B
B–F
CHE
CHE–F
QUE
QUE–F

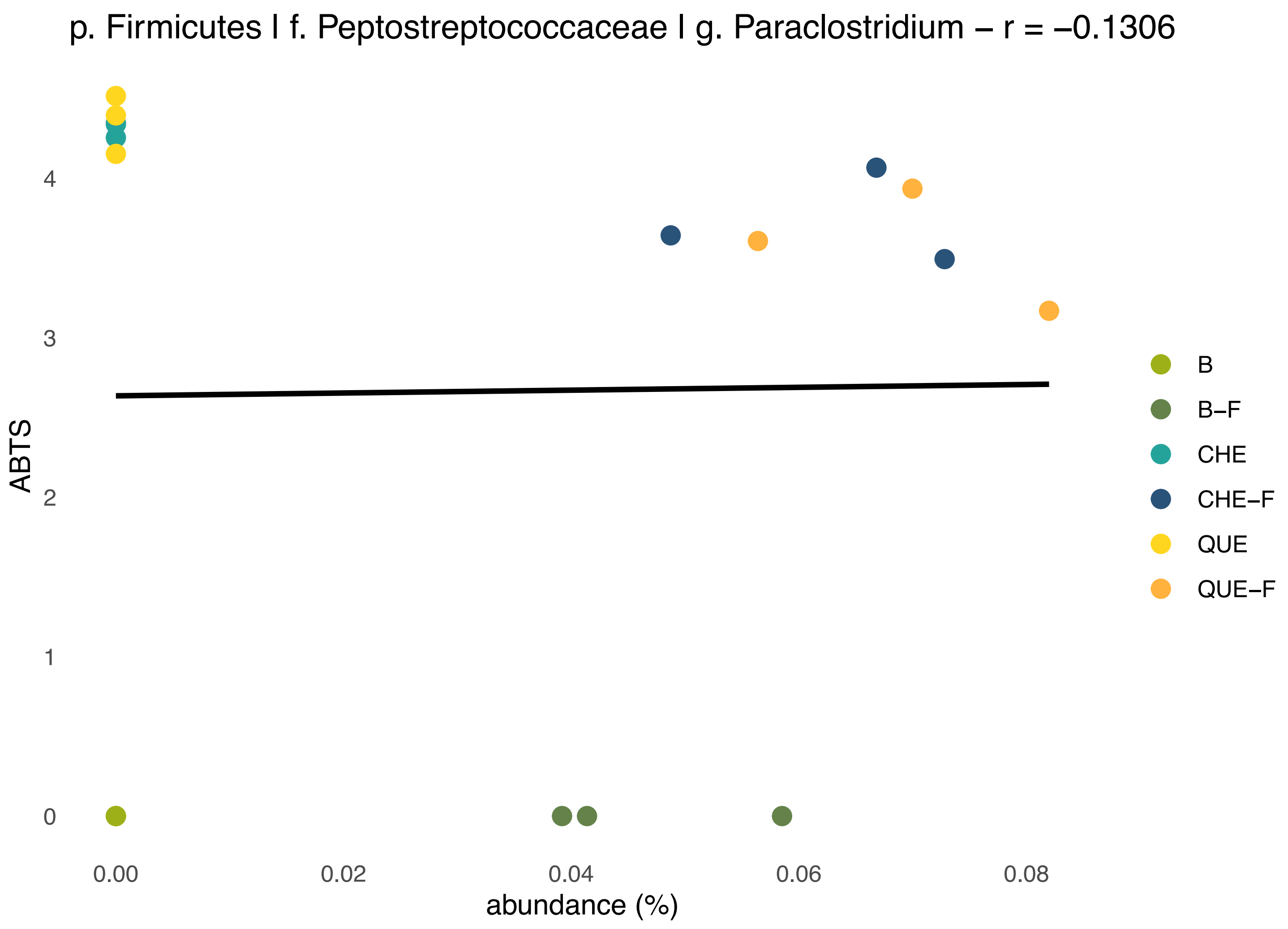

p. Firmicutes I f. Peptostreptococcaceae I g. Paraclostridium – r = –0.1306
ABTS
0
1
2
3
4
0.00
0.02
0.04
0.06
0.08
abundance (%)
B
B–F
CHE
CHE–F
QUE
QUE–F

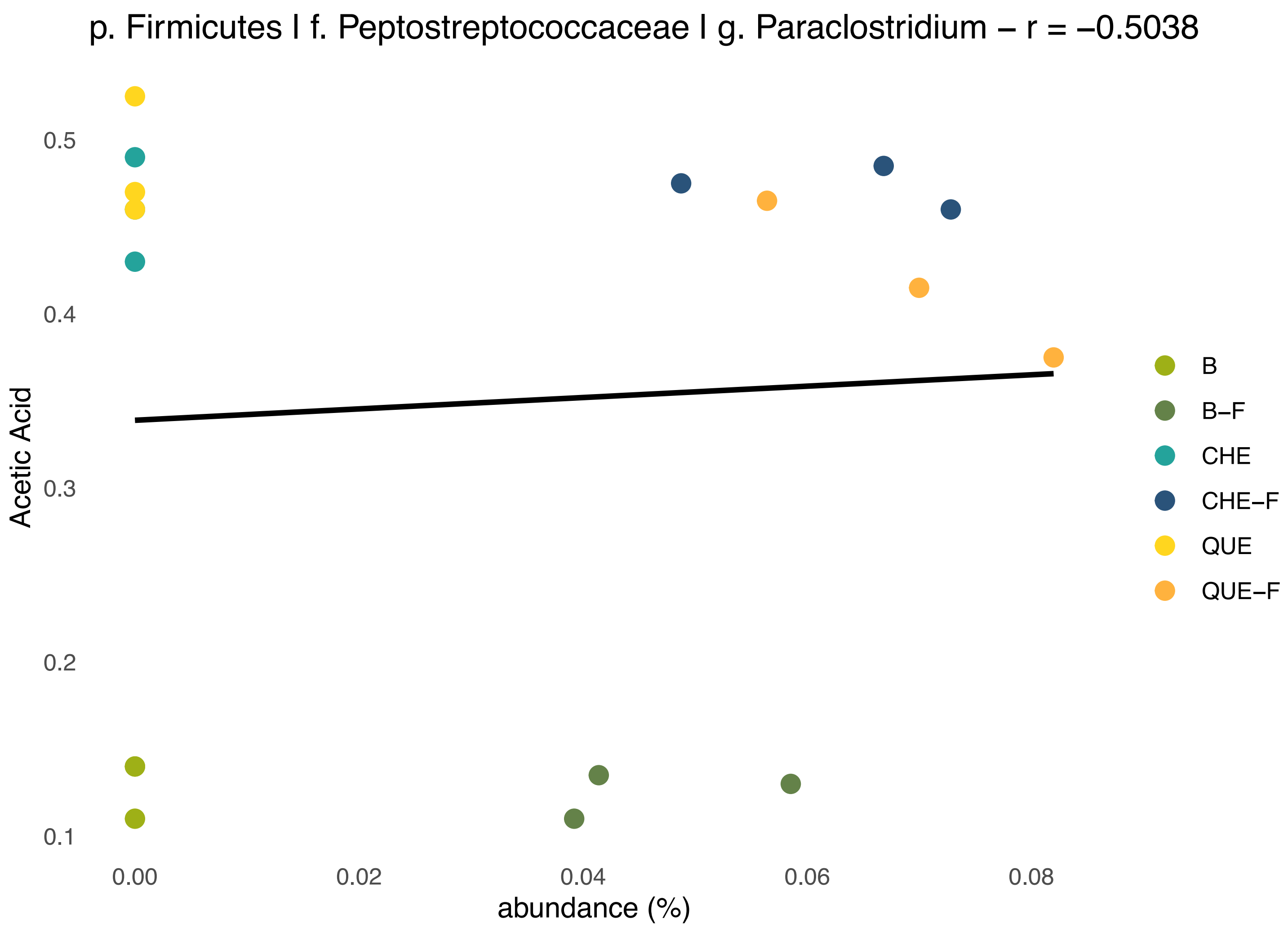
p. Firmicutes I f. Peptostreptococcaceae I g. Paraclostridium – r = –0.5038
Acetic Acid
0.5
0.4
0.3
0.2
0.1
0.00
0.02
0.04
0.06
0.08
abundance (%)
B
B–F
CHE
CHE–F
QUE
QUE–F

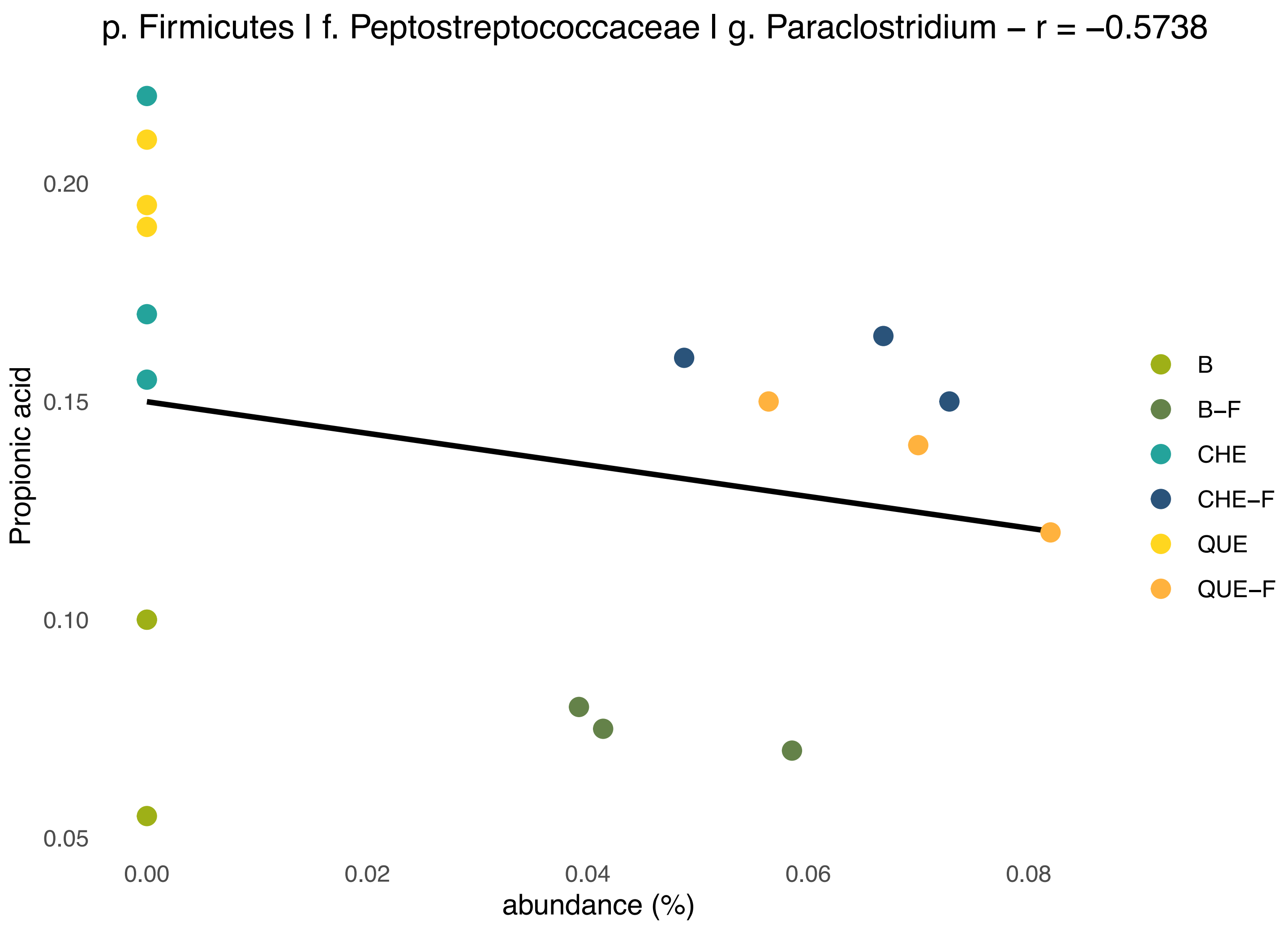
p. Firmicutes I f. Peptostreptococcaceae I g. Paraclostridium – r = –0.5738
Propionic acid
0.20
0.15
0.10
0.05
0.00
0.02
0.04
0.06
0.08
abundance (%)
B
B–F
CHE
CHE–F
QUE
QUE–F

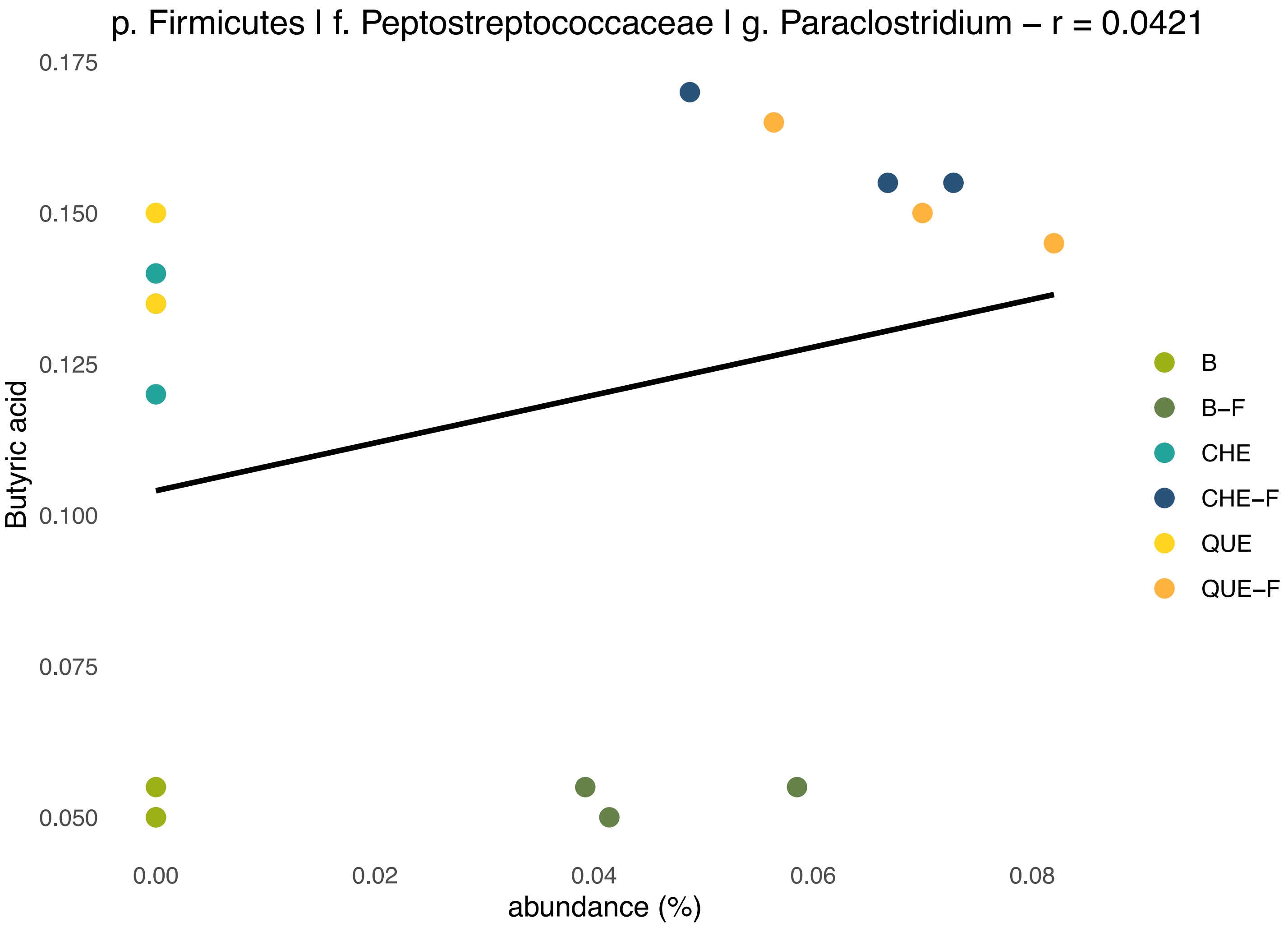
p. Firmicutes I f. Peptostreptococcaceae I g. Paraclostridium – r = 0.0421
0.175
0.150
0.125
0.100
0.075
0.050
Butyric acid
0.00
0.02
0.04
0.06
0.08
abundance (%)
B
B–F
CHE
CHE–F
QUE
QUE–F

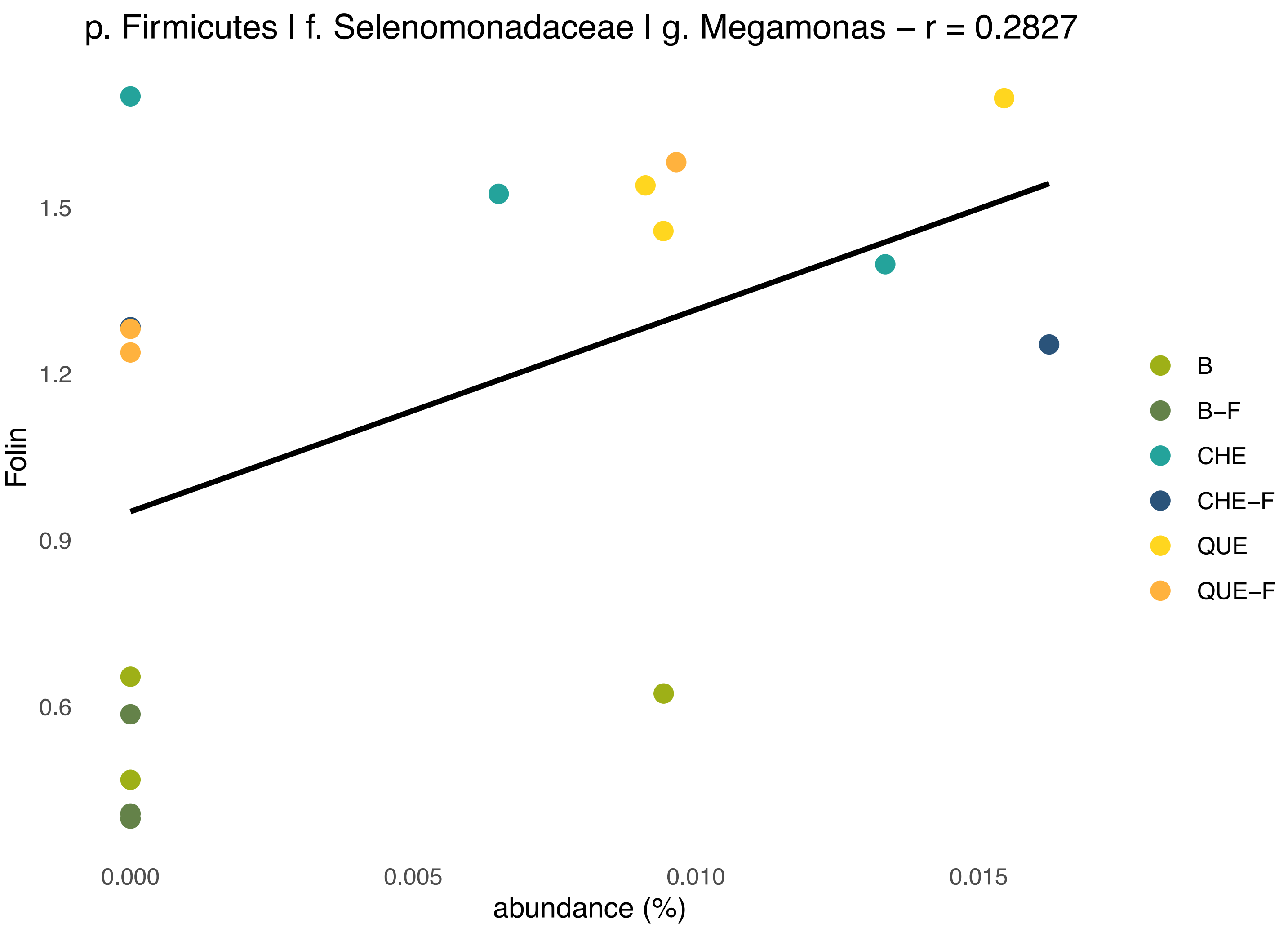

p. Firmicutes I f. Selenomonadaceae I g. Megamonas – r = 0.2827
Folin
1.5
1.2
0.9
0.6
0.000
0.005
0.010
0.015
abundance (%)
B
B–F
CHE
CHE–F
QUE
QUE–F

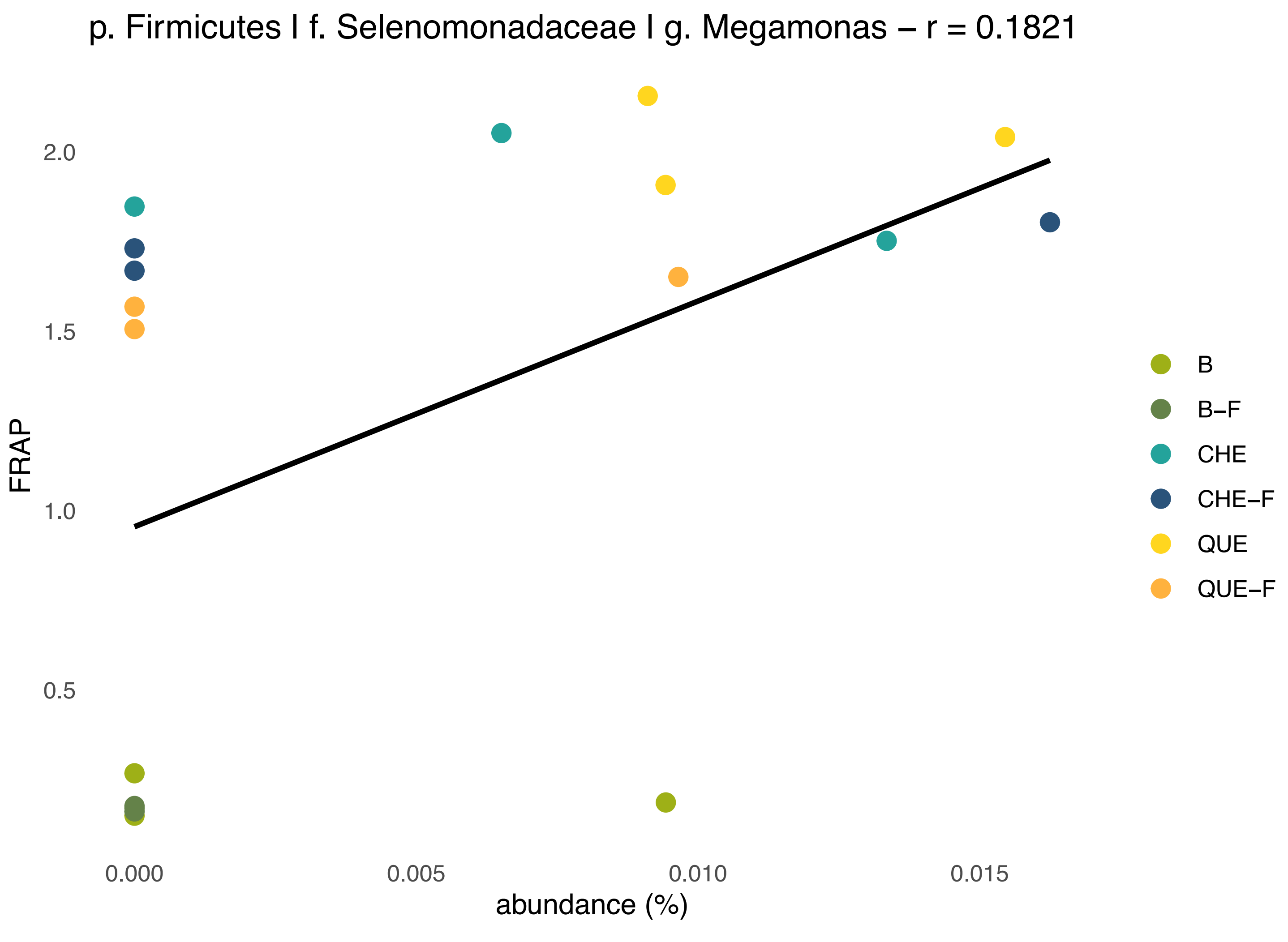
p. Firmicutes I f. Selenomonadaceae I g. Megamonas – r = 0.1821
FRAP
2.0
1.5
1.0
0.5
0.000
0.005
0.010
0.015
abundance (%)
B
B–F
CHE
CHE–F
QUE
QUE–F

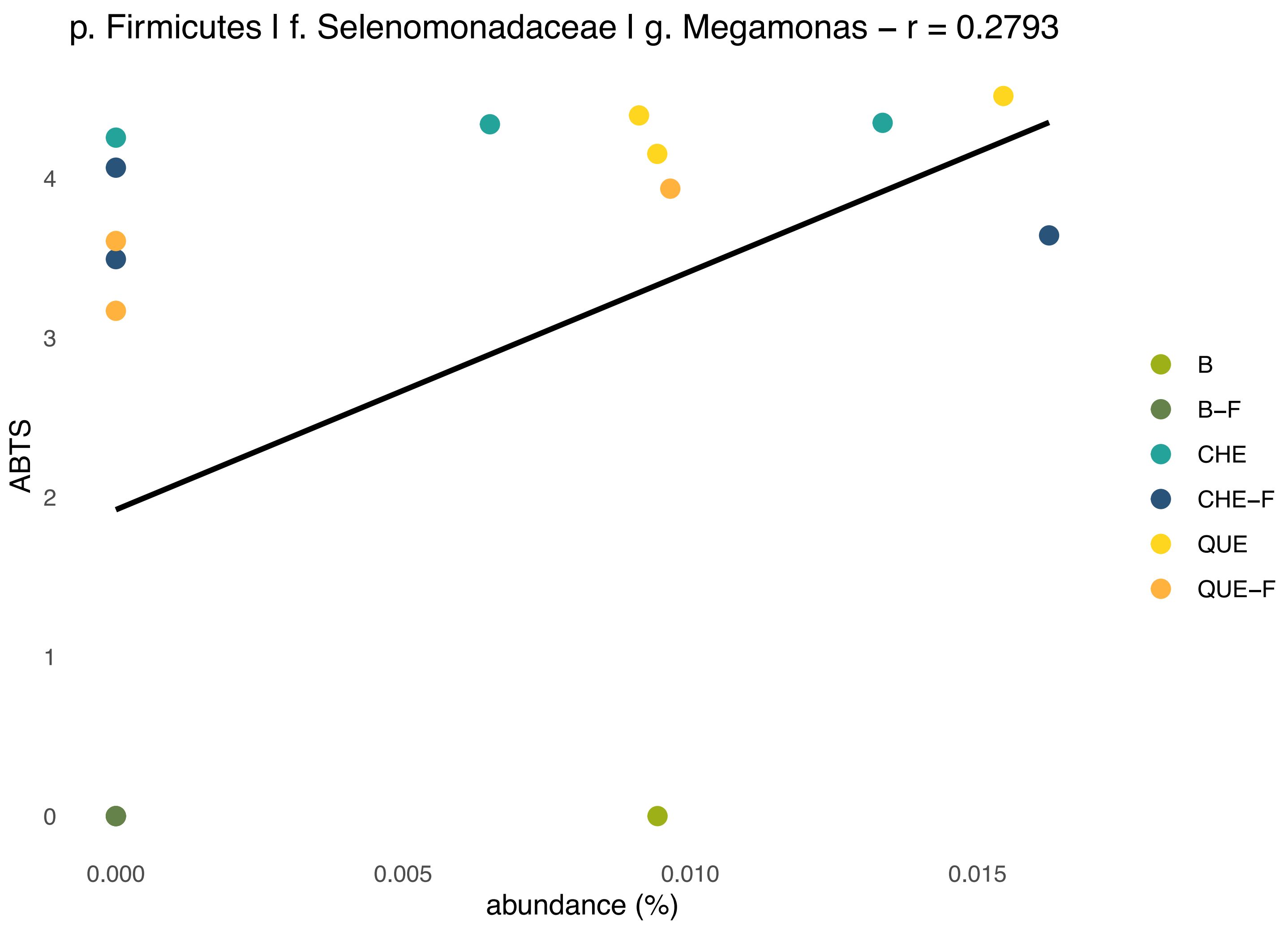

p. Firmicutes l f. Selenomonadaceae l g. Megamonas – r = 0.2793
ABTS
4
3
2
1
0
0.000
0.005
0.010
0.015
abundance (%)
B
B−F
CHE
CHE−F
QUE
QUE−F

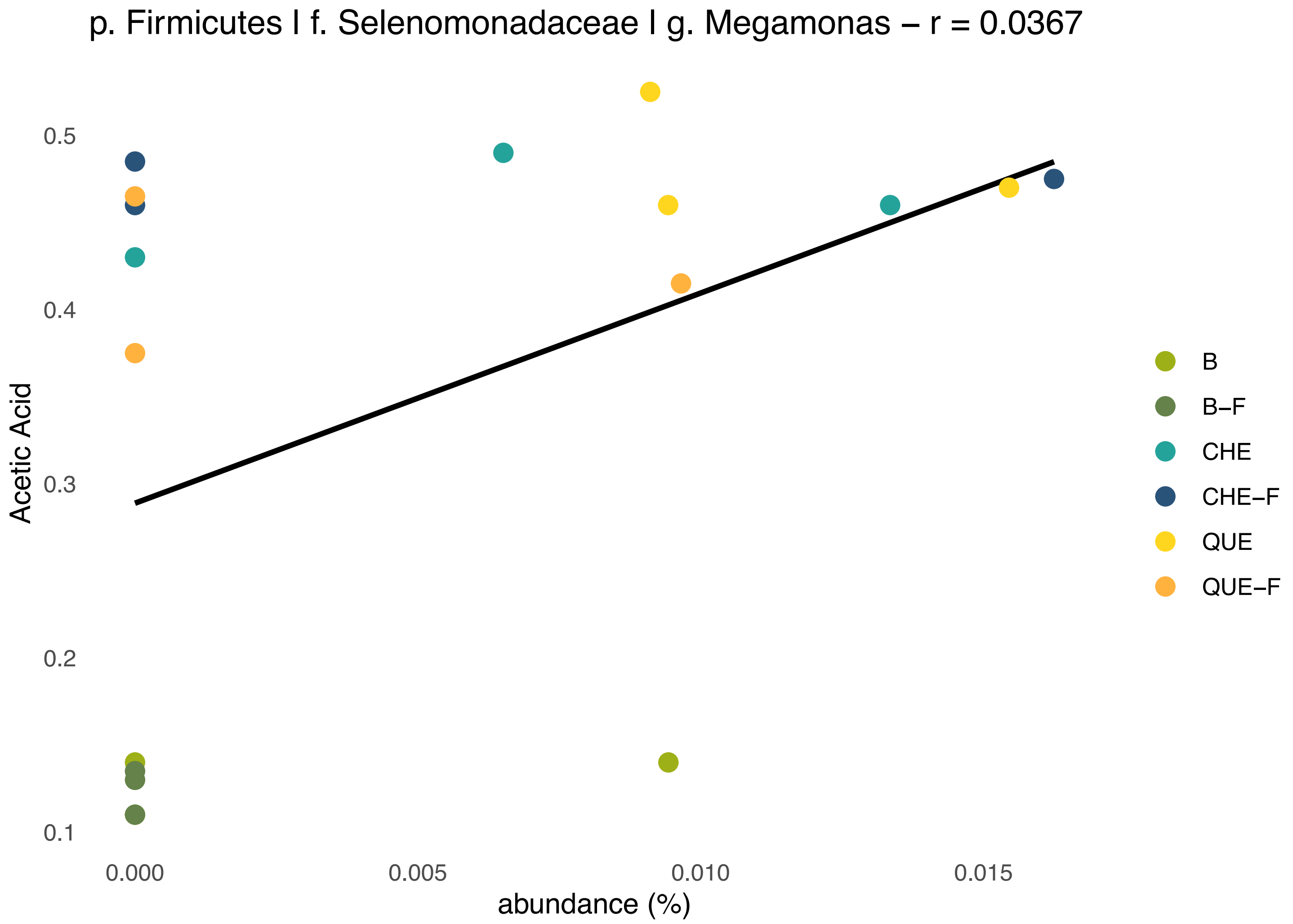
p. Firmicutes I f. Selenomonadaceae I g. Megamonas – r = 0.0367
Acetic Acid
0.5
0.4
0.3
0.2
0.1
0.000
0.005
0.010
0.015
abundance (%)
B
B–F
CHE
CHE–F
QUE
QUE–F

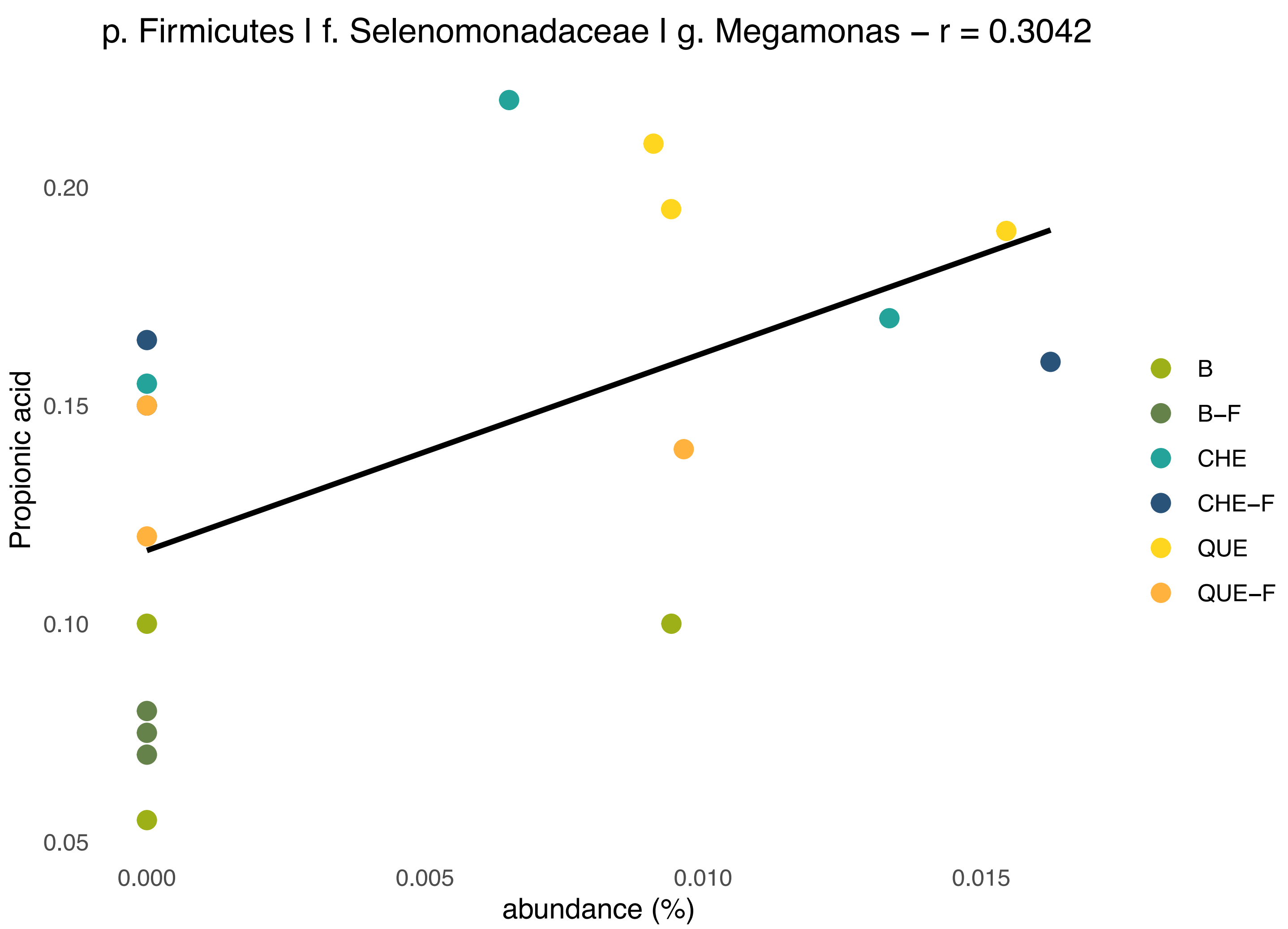

p. Firmicutes I f. Selenomonadaceae I g. Megamonas – r = 0.3042
Propionic acid
0.20
0.15
0.10
0.05
0.000
0.005
0.010
0.015
abundance (%)
B
B–F
CHE
CHE–F
QUE
QUE–F

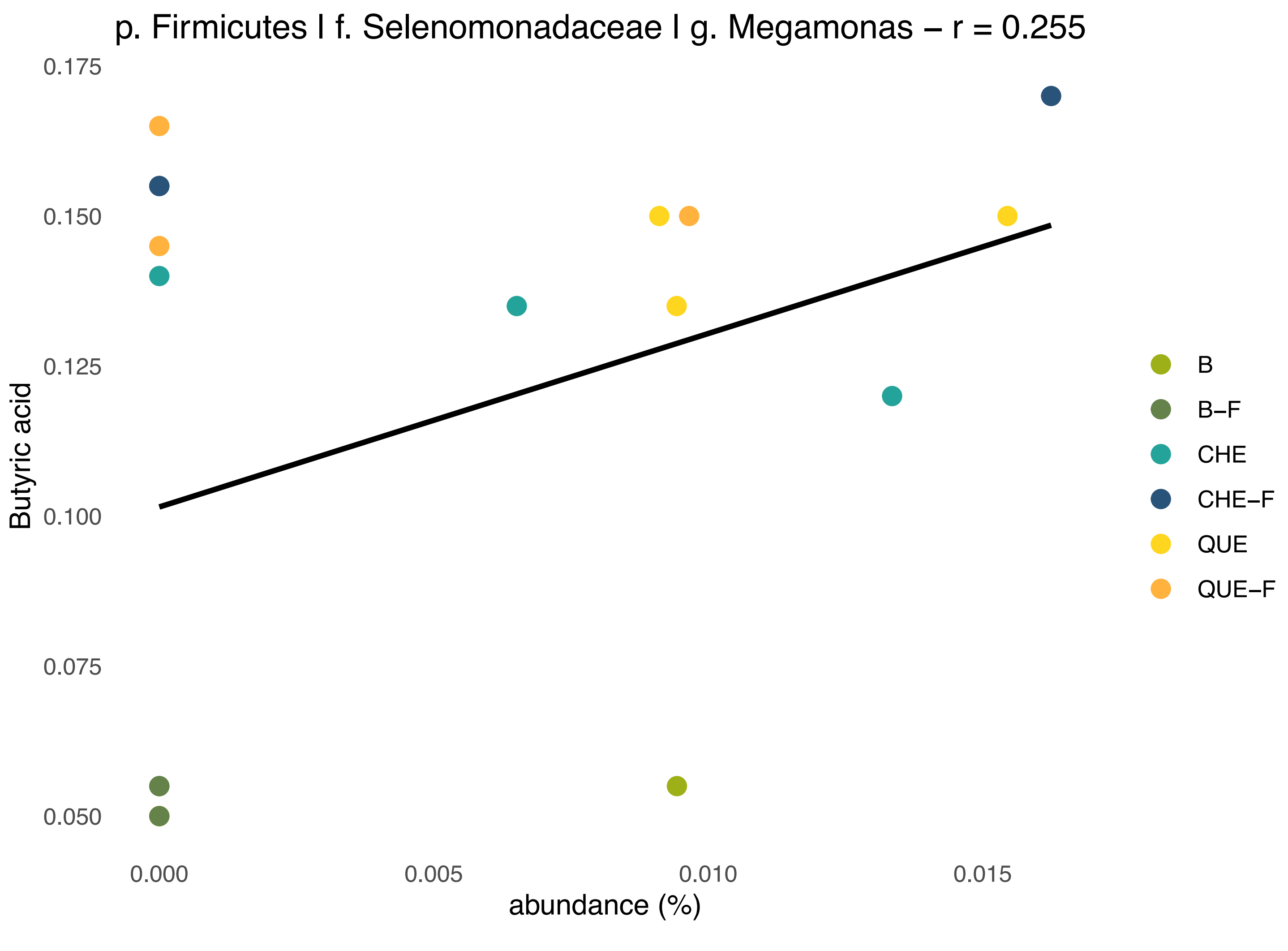
p. Firmicutes I f. Selenomonadaceae I g. Megamonas – r = 0.255
Butyric acid
0.175
0.150
0.125
0.100
0.075
0.050
0.000
0.005
0.010
0.015
abundance (%)
B
B–F
CHE
CHE–F
QUE
QUE–F

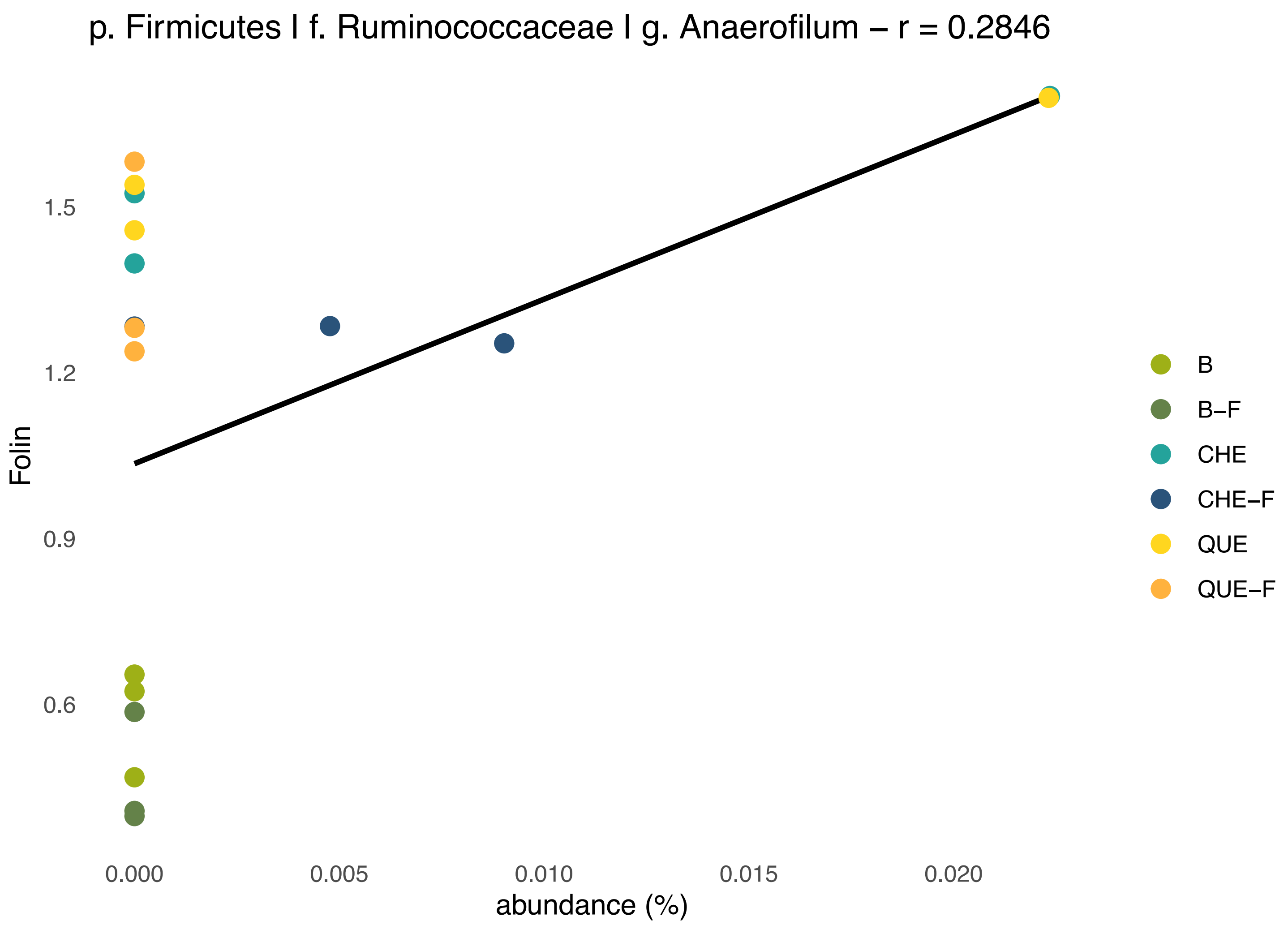

p. Firmicutes l f. Ruminococcaceae l g. Anaerofilum – r = 0.2846
Folin
1.5
1.2
0.9
0.6
0.000
0.005
0.010
0.015
0.020
abundance (%)
B
B–F
CHE
CHE–F
QUE
QUE–F

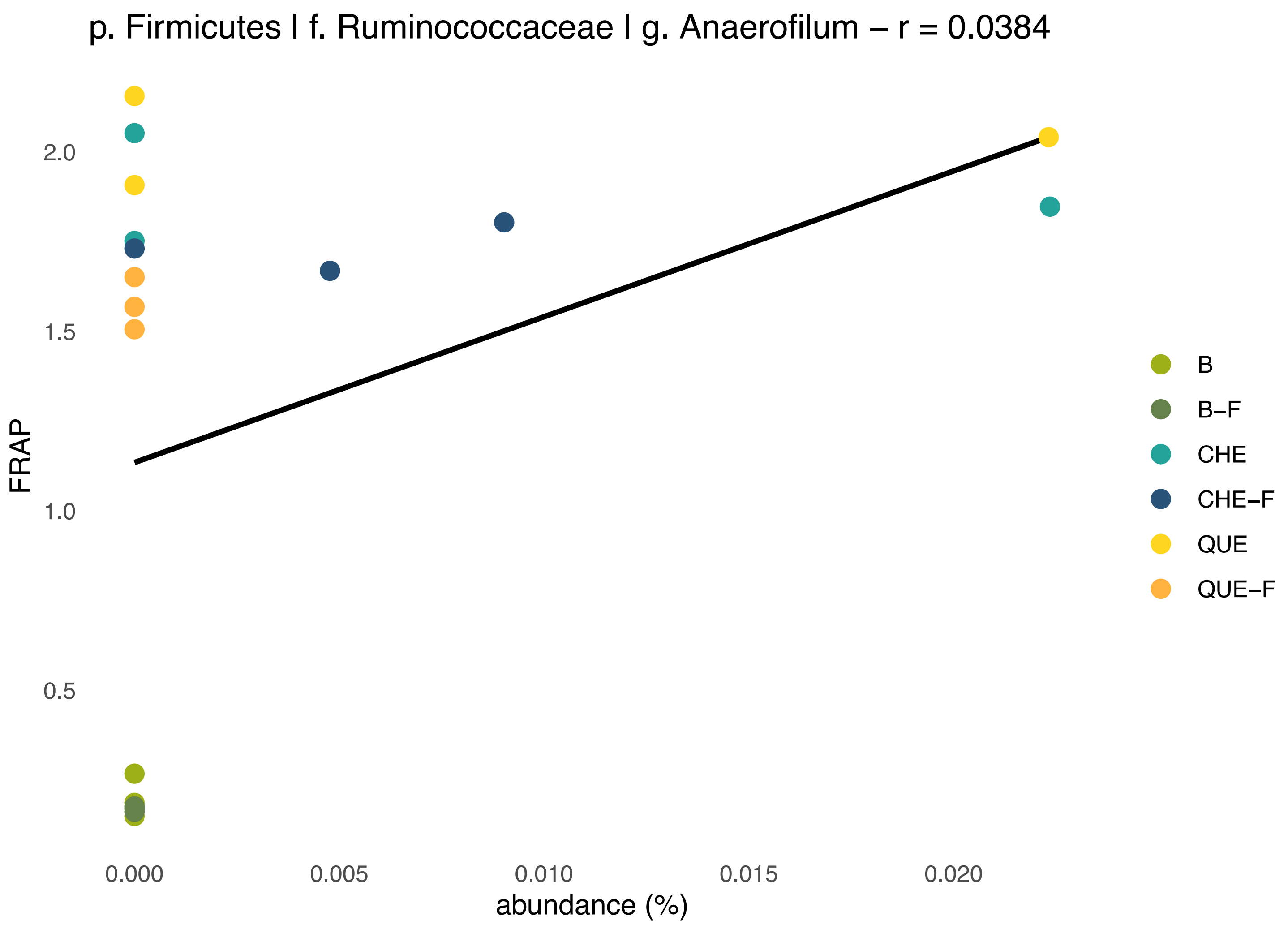

p. Firmicutes I f. Ruminococcaceae I g. Anaerofilum − r = 0.0384
FRAP
2.0
1.5
1.0
0.5
0.000
0.005
0.010
0.015
0.020
abundance (%)
B
B−F
CHE
CHE−F
QUE
QUE−F

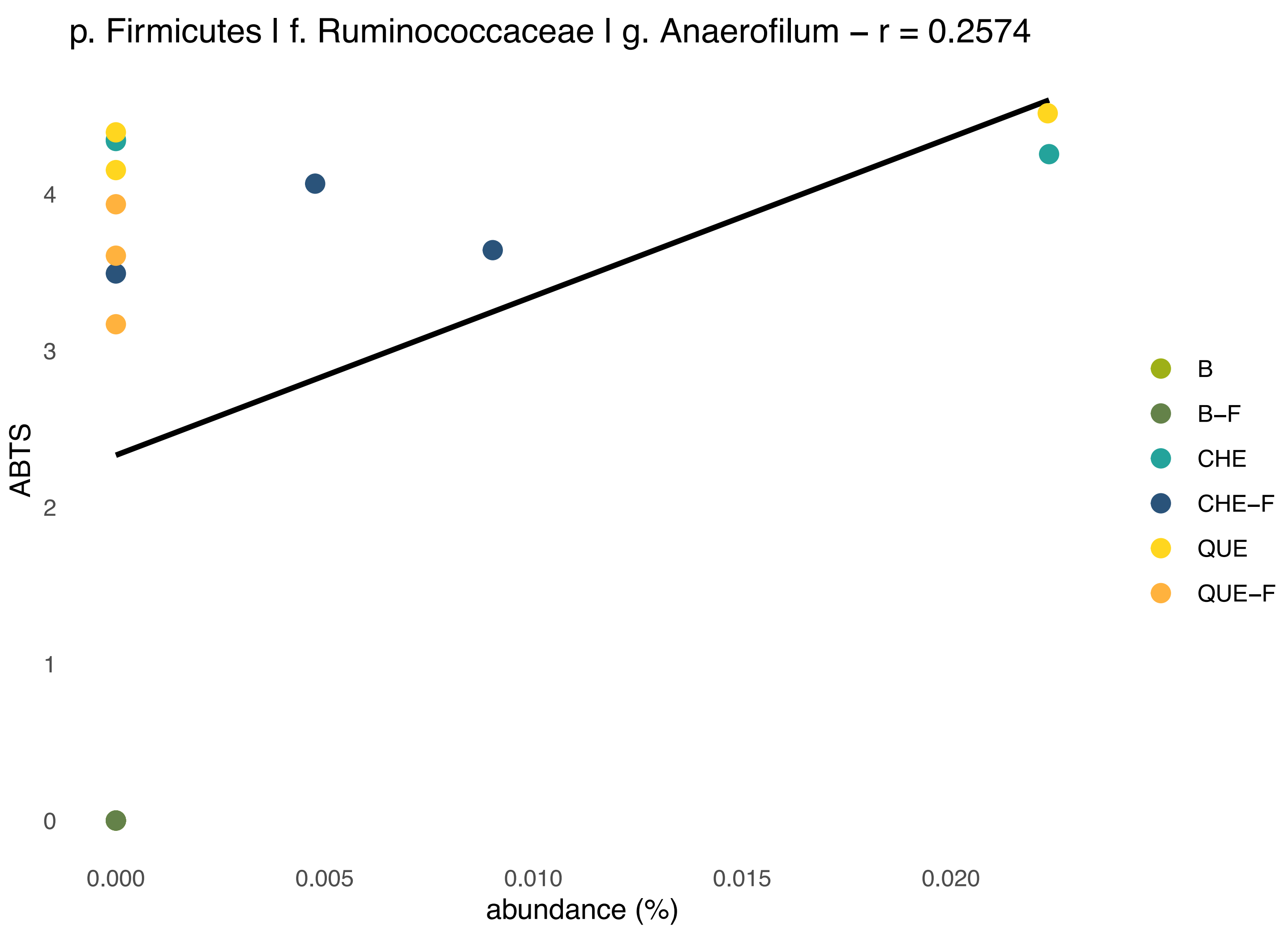
p. Firmicutes I f. Ruminococcaceae I g. Anaerofilum – r = 0.2574
ABTS
0
1
2
3
4
0.000
0.005
0.010
0.015
0.020
abundance (%)
B
B–F
CHE
CHE–F
QUE
QUE–F

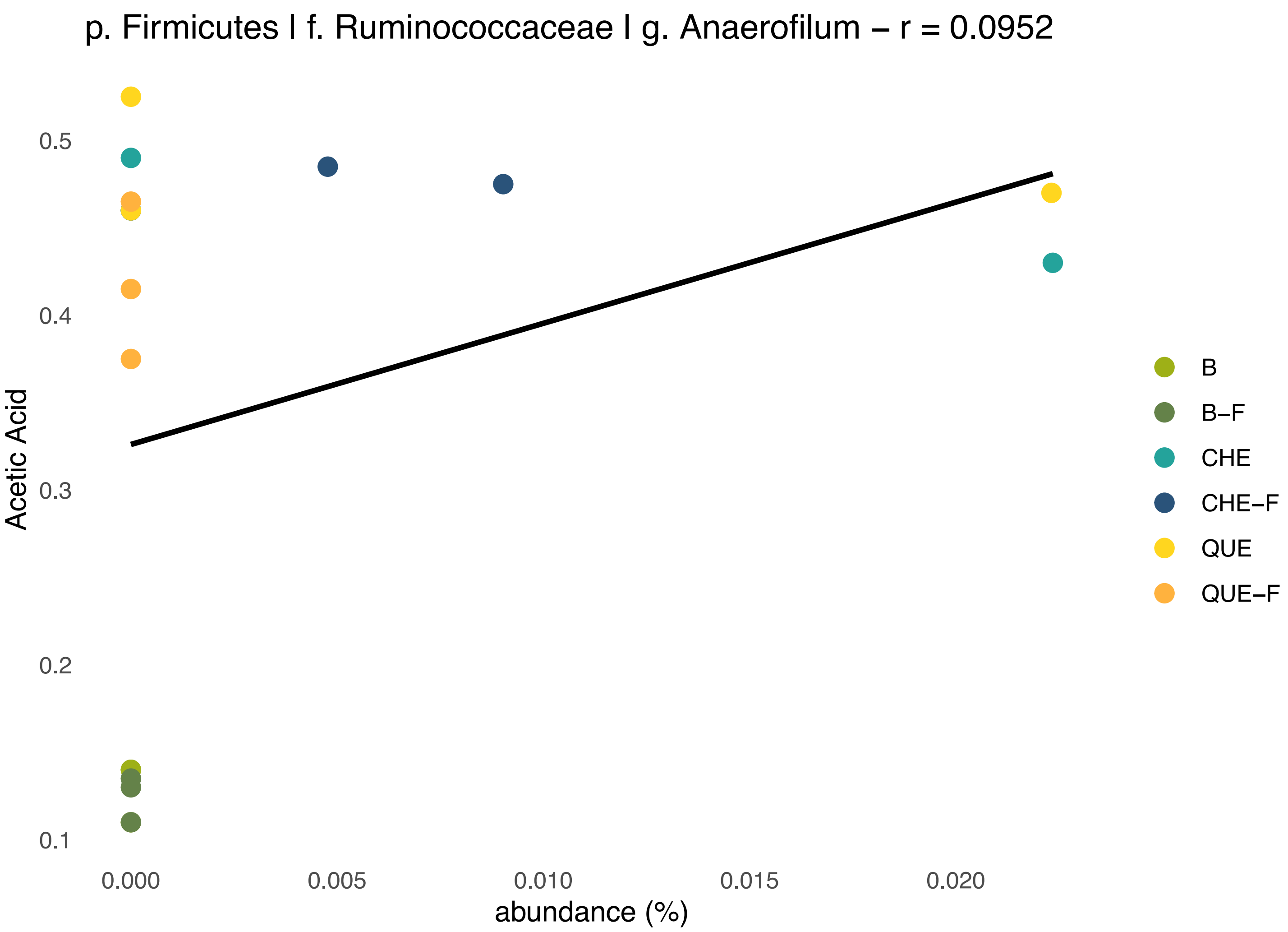

p. Firmicutes l f. Ruminococcaceae l g. Anaerofilum – r = 0.0952
Acetic Acid
0.5
0.4
0.3
0.2
0.1
0.000
0.005
0.010
0.015
0.020
abundance (%)
B
B−F
CHE
CHE−F
QUE
QUE−F

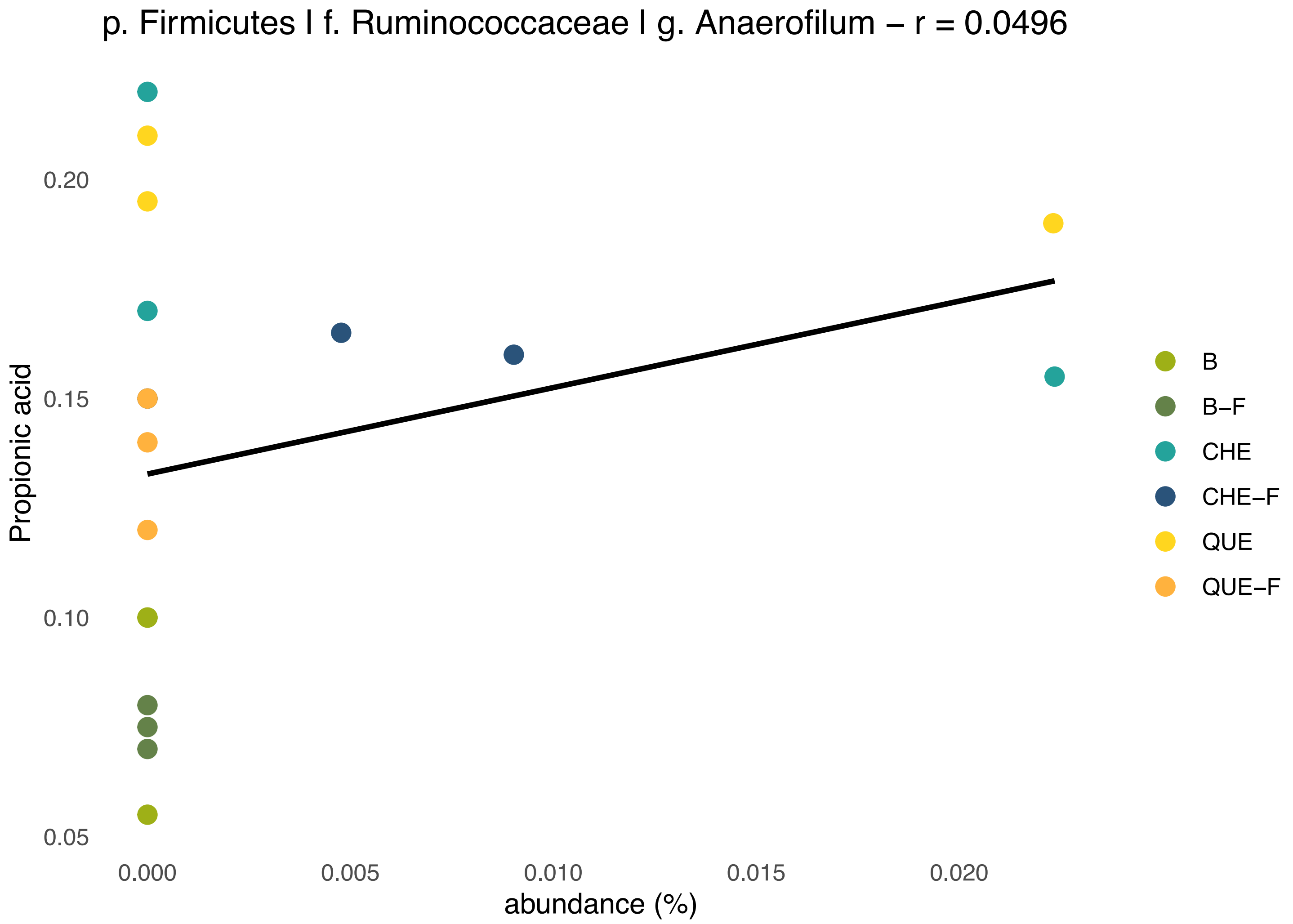

p. Firmicutes I f. Ruminococcaceae I g. Anaerofilum – r = 0.0496
Propionic acid
0.20
0.15
0.10
0.05
0.000
0.005
0.010
0.015
0.020
abundance (%)
B
B–F
CHE
CHE–F
QUE
QUE–F

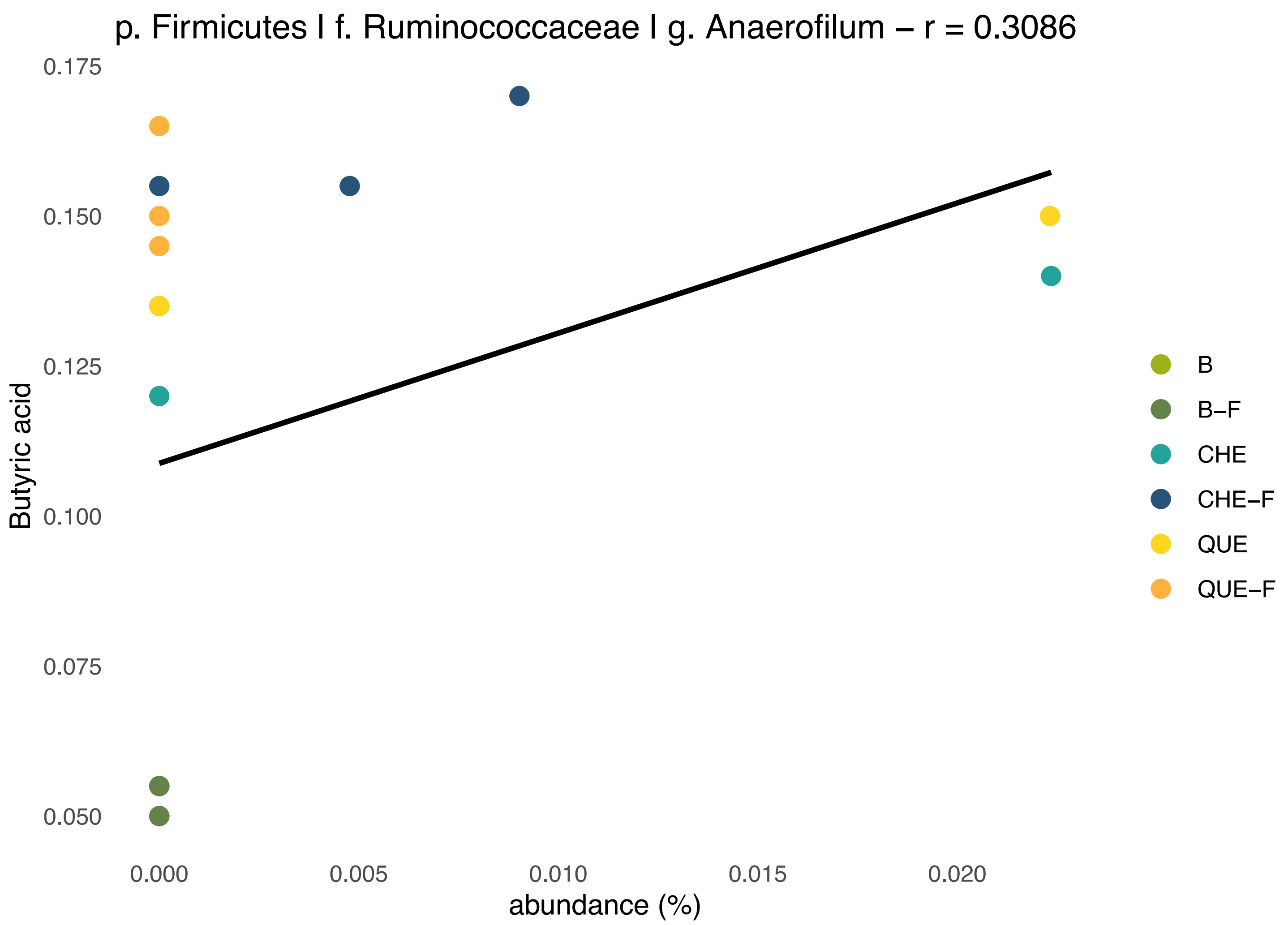

p. Firmicutes I f. Ruminococcaceae I g. Anaerofilum – r = 0.3086
Butyric acid
0.175
0.150
0.125
0.100
0.075
0.050
0.000
0.005
0.010
0.015
0.020
abundance (%)
B
B–F
CHE
CHE–F
QUE
QUE–F

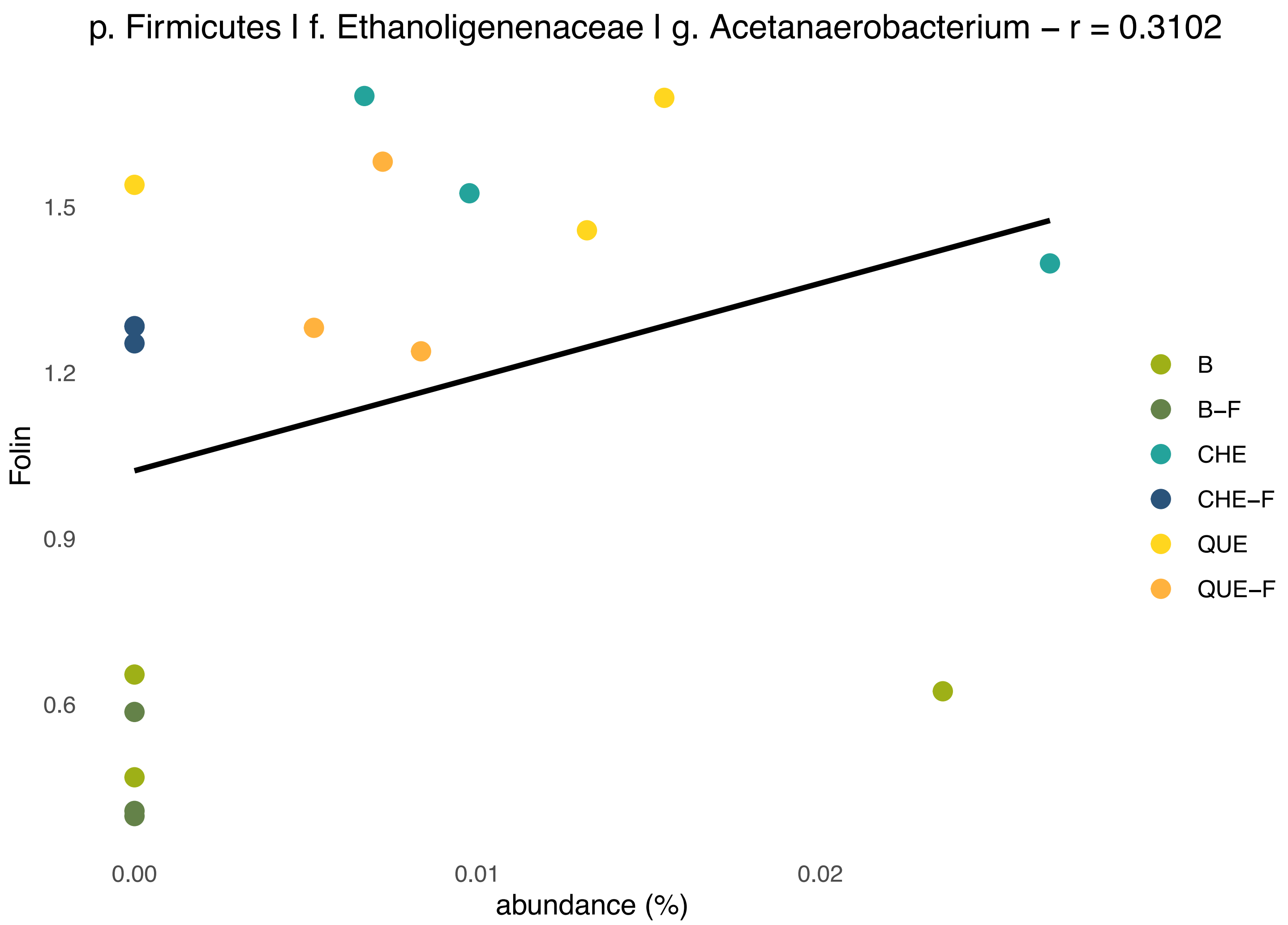

p. Firmicutes I f. Ethanoligenenaceae I g. Acetanaerobacterium – r = 0.3102
1.5
1.2
0.9
0.6
Folin
0.00
0.01
0.02
abundance (%)
B
B–F
CHE
CHE–F
QUE
QUE–F

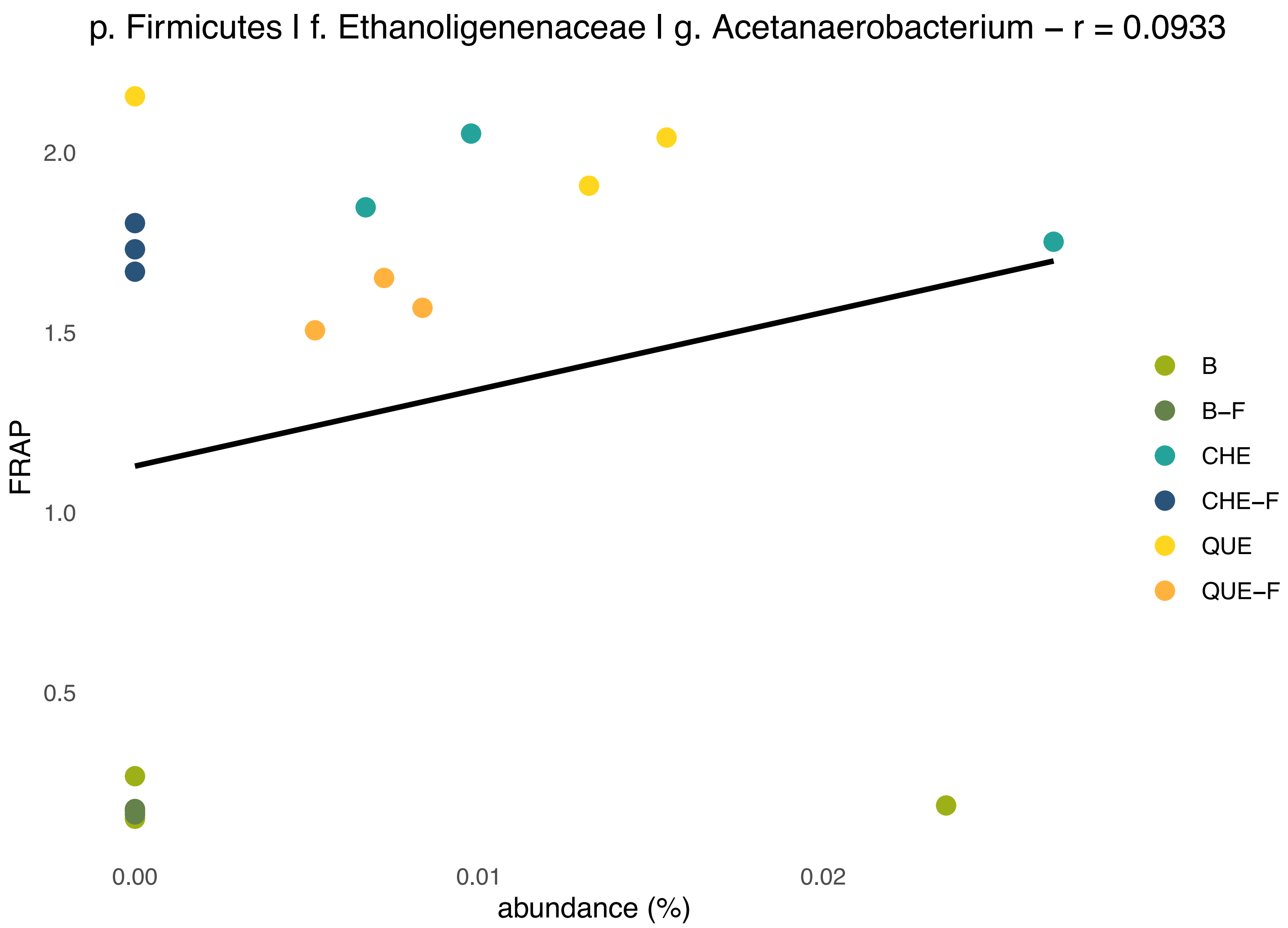

p. Firmicutes I f. Ethanoligenenaceae I g. Acetanaerobacterium – r = 0.0933
FRAP
2.0
1.5
1.0
0.5
0.00
0.01
0.02
abundance (%)
B
B–F
CHE
CHE–F
QUE
QUE–F

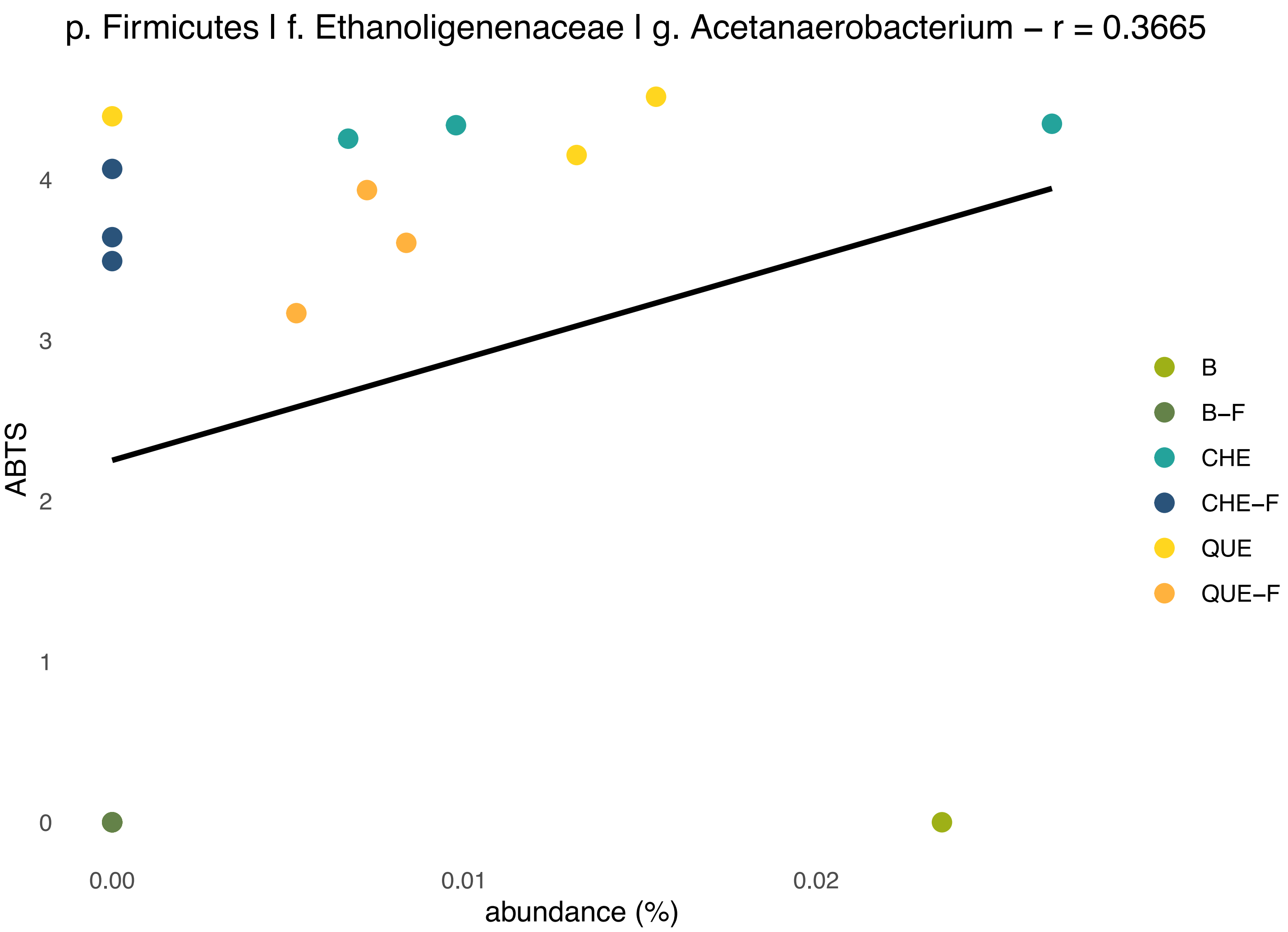
p. Firmicutes I f. Ethanoligenenaceae I g. Acetanaerobacterium – r = 0.3665
ABTS
4
3
2
1
0
0.00
0.01
0.02
abundance (%)
B
B–F
CHE
CHE–F
QUE
QUE–F

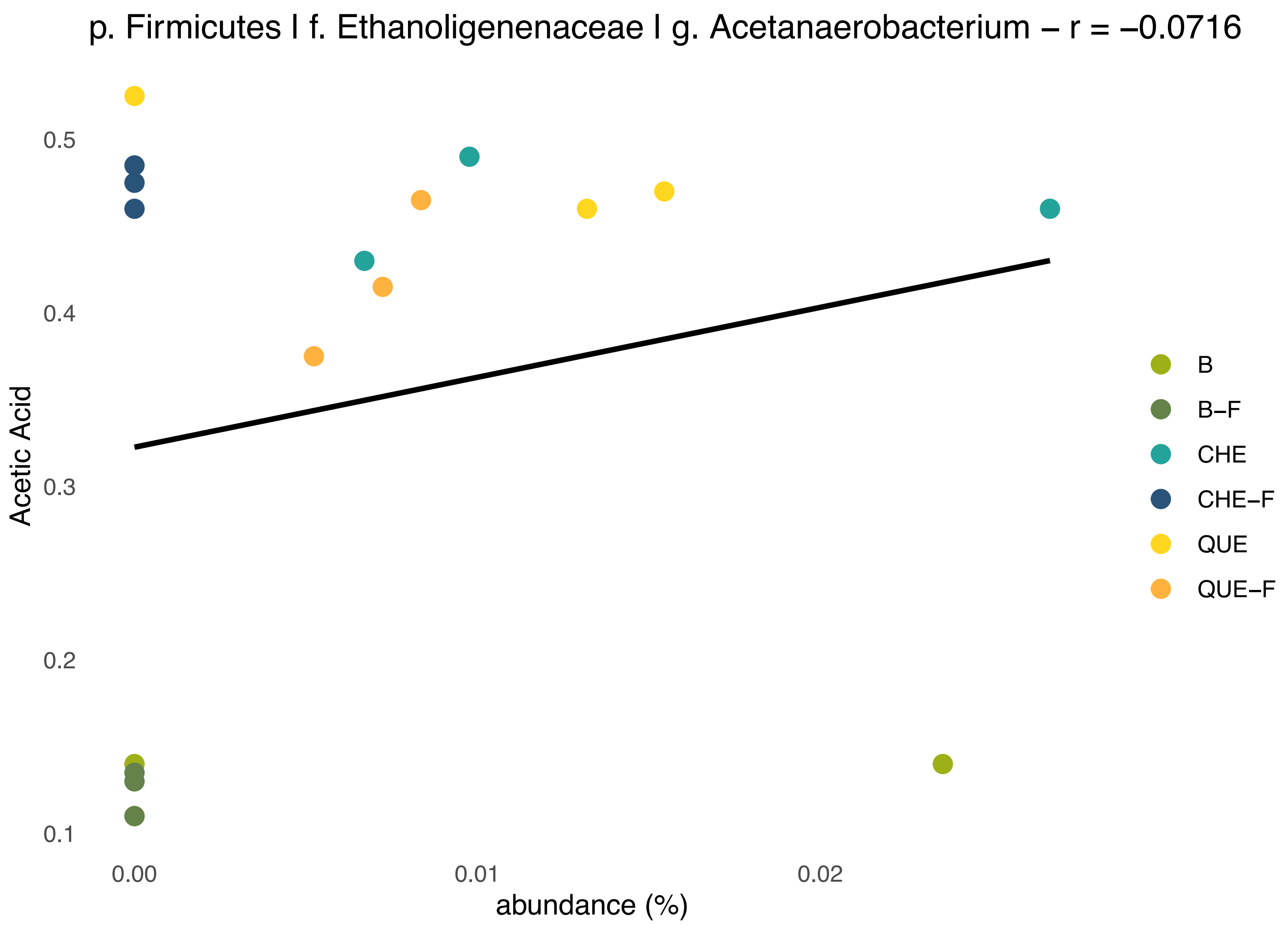
p. Firmicutes I f. Ethanoligenenaceae I g. Acetanaerobacterium – r = –0.0716
Acetic Acid
0.5
0.4
0.3
0.2
0.1
0.00
0.01
0.02
abundance (%)
B
B–F
CHE
CHE–F
QUE
QUE–F

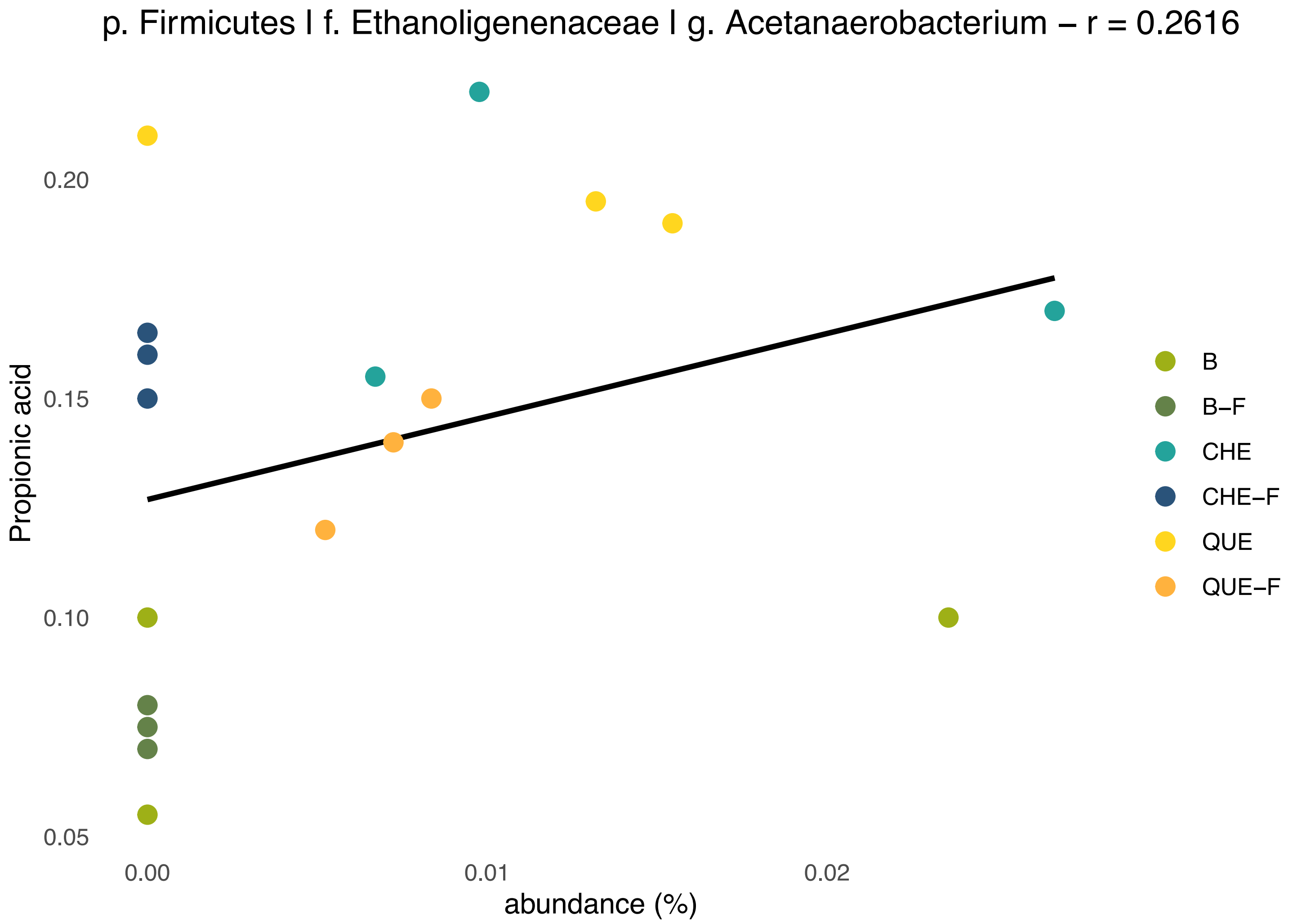

p. Firmicutes I f. Ethanoligenenaceae I g. Acetanaerobacterium − r = 0.2616
Propionic acid
0.20
0.15
0.10
0.05
0.00
0.01
0.02
abundance (%)
B
B−F
CHE
CHE−F
QUE
QUE−F

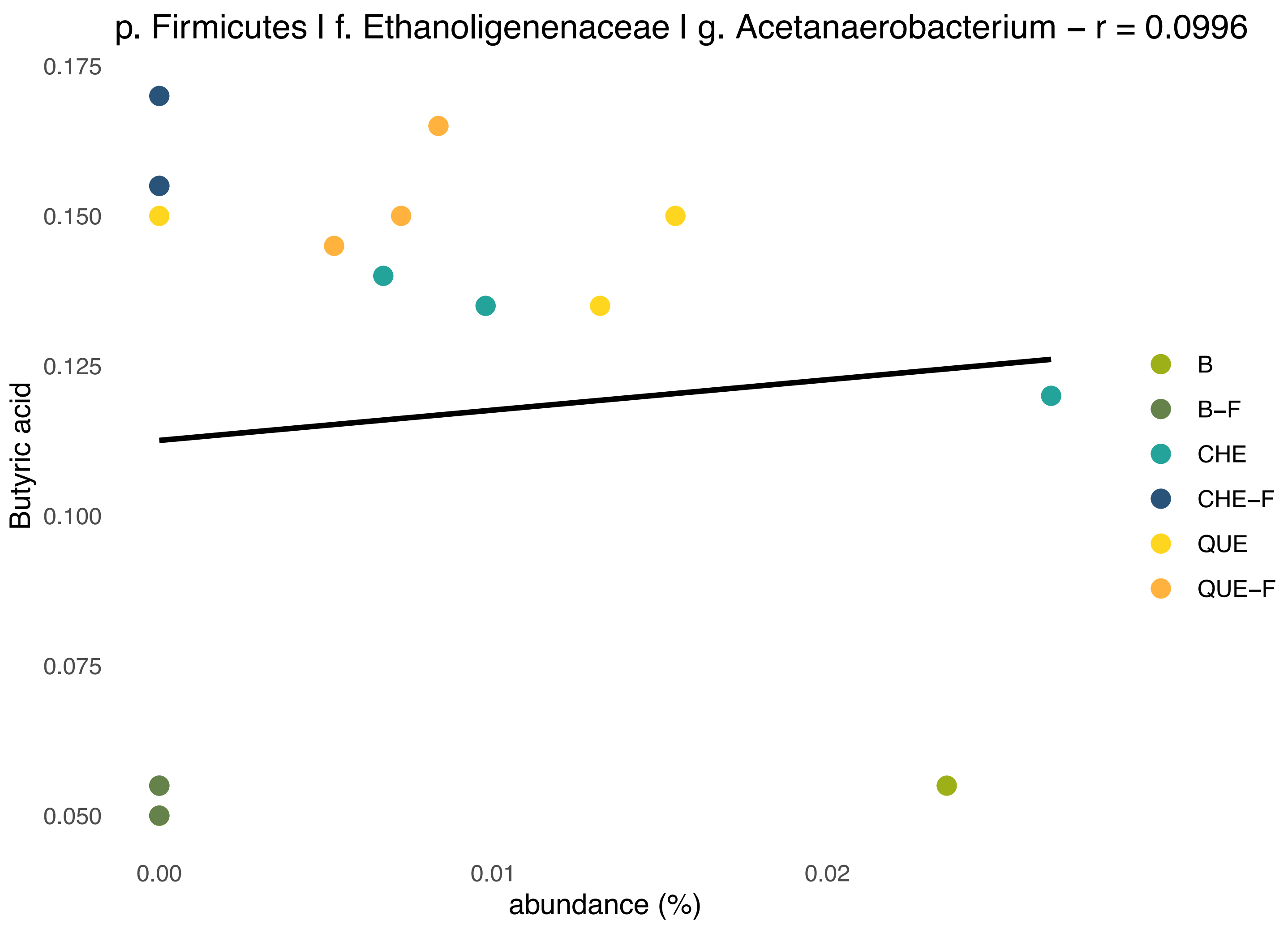
p. Firmicutes | f. Ethanoligenenaceae | g. Acetanaerobacterium – r = 0.0996
0.175
0.150
0.125
0.100
0.075
0.050
Butyric acid
0.00
0.01
0.02
abundance (%)
B
B–F
CHE
CHE–F
QUE
QUE–F

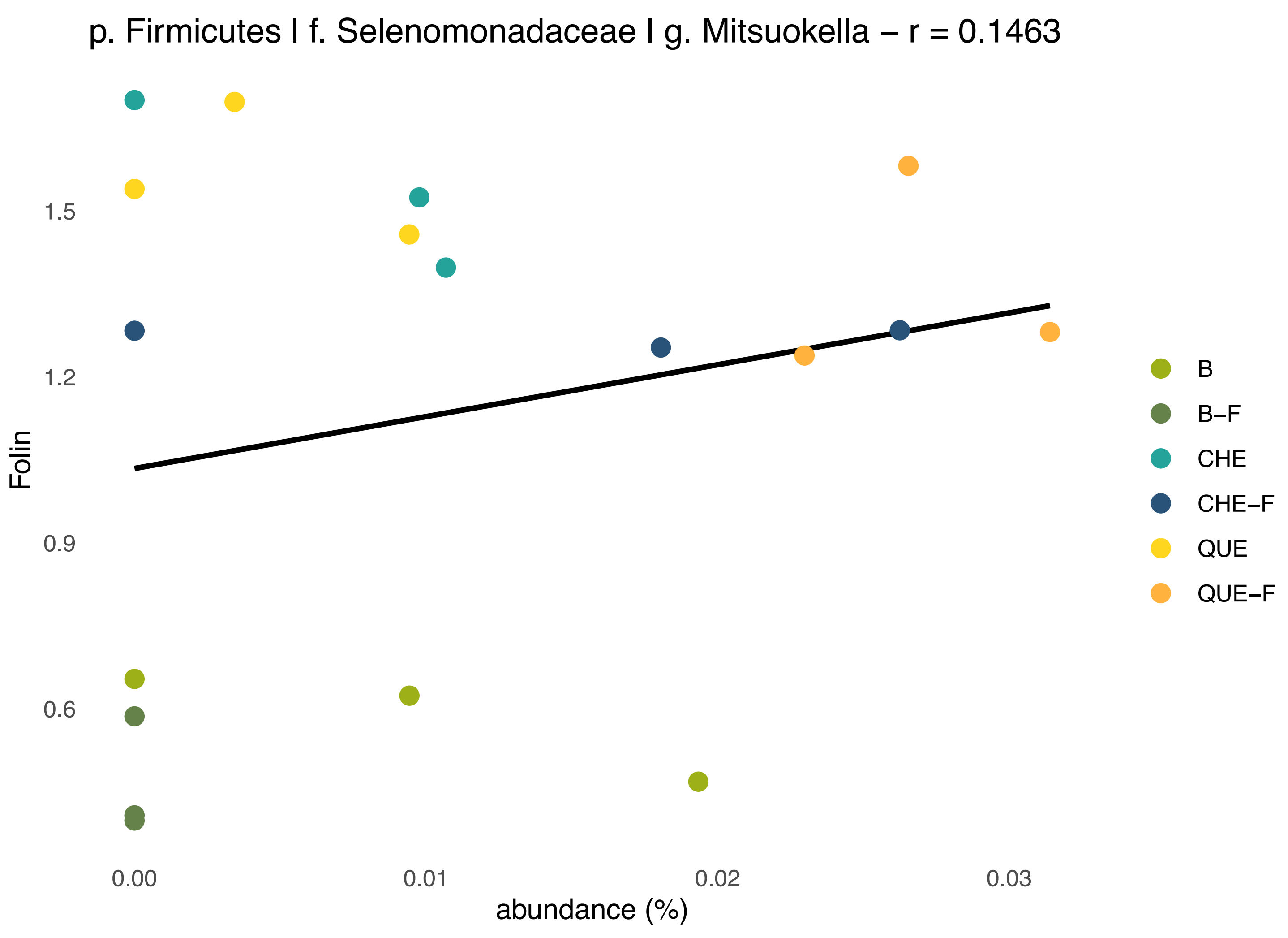
p. Firmicutes I f. Selenomonadaceae I g. Mitsuokella – r = 0.1463
1.5
1.2
0.9
0.6
Folin
0.00
0.01
0.02
0.03
abundance (%)
B
B–F
CHE
CHE–F
QUE
QUE–F

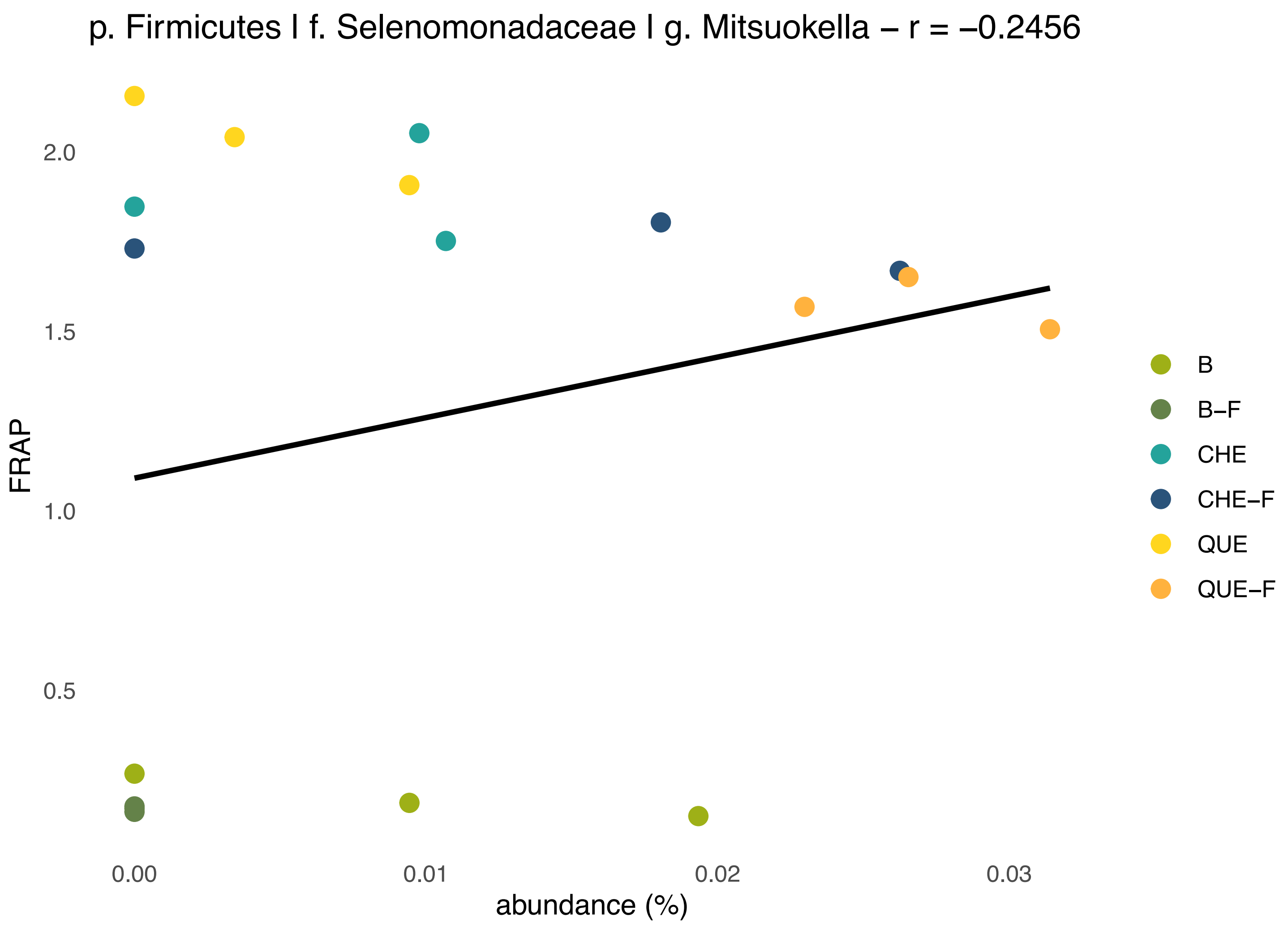
p. Firmicutes I f. Selenomonadaceae I g. Mitsuokella – r = –0.2456
FRAP
2.0
1.5
1.0
0.5
0.00
0.01
0.02
0.03
abundance (%)
B
B–F
CHE
CHE–F
QUE
QUE–F

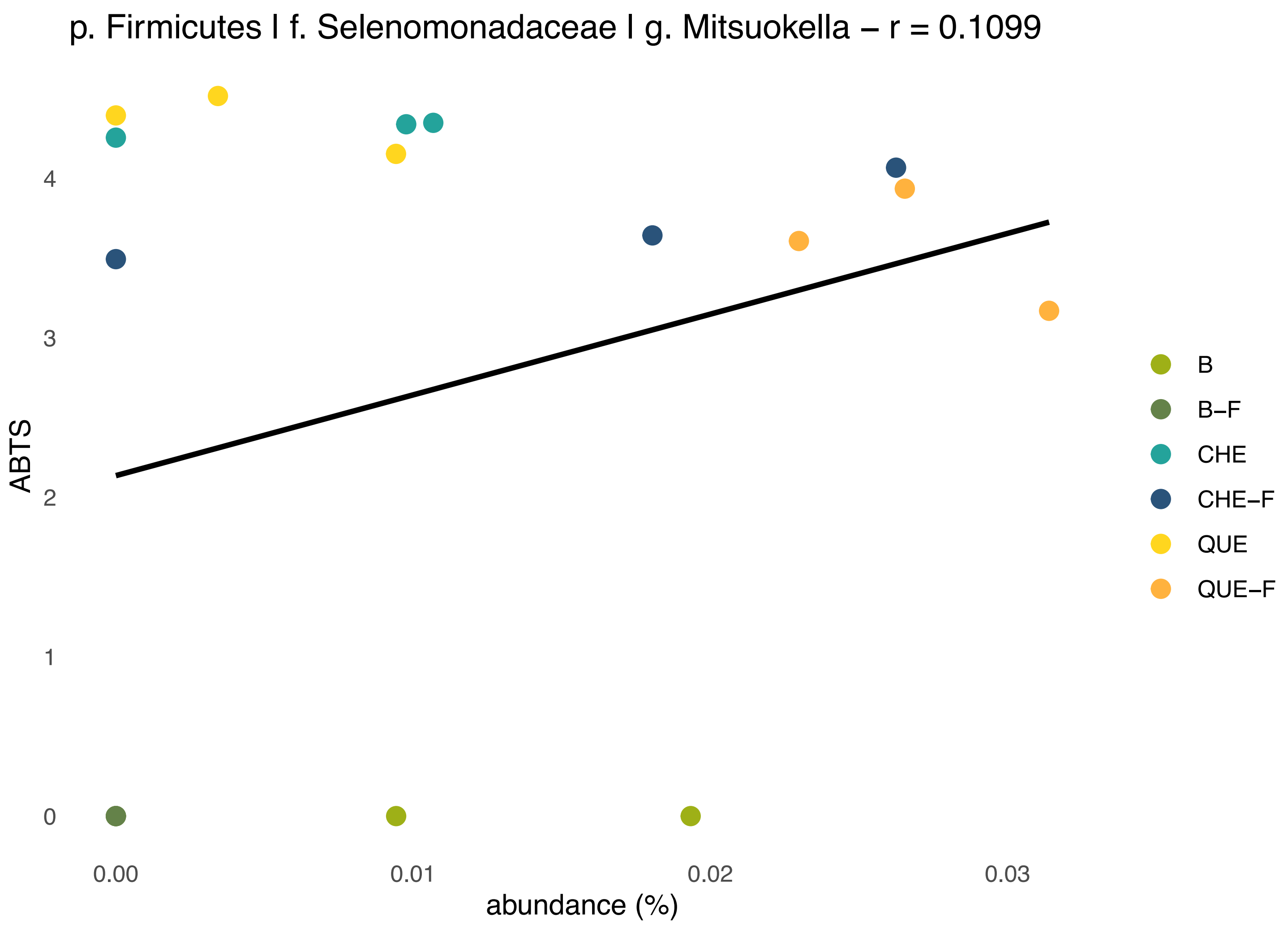
p. Firmicutes I f. Selenomonadaceae I g. Mitsuokella – r = 0.1099
ABTS
0
1
2
3
4
0.00
0.01
0.02
0.03
abundance (%)
B
B–F
CHE
CHE–F
QUE
QUE–F

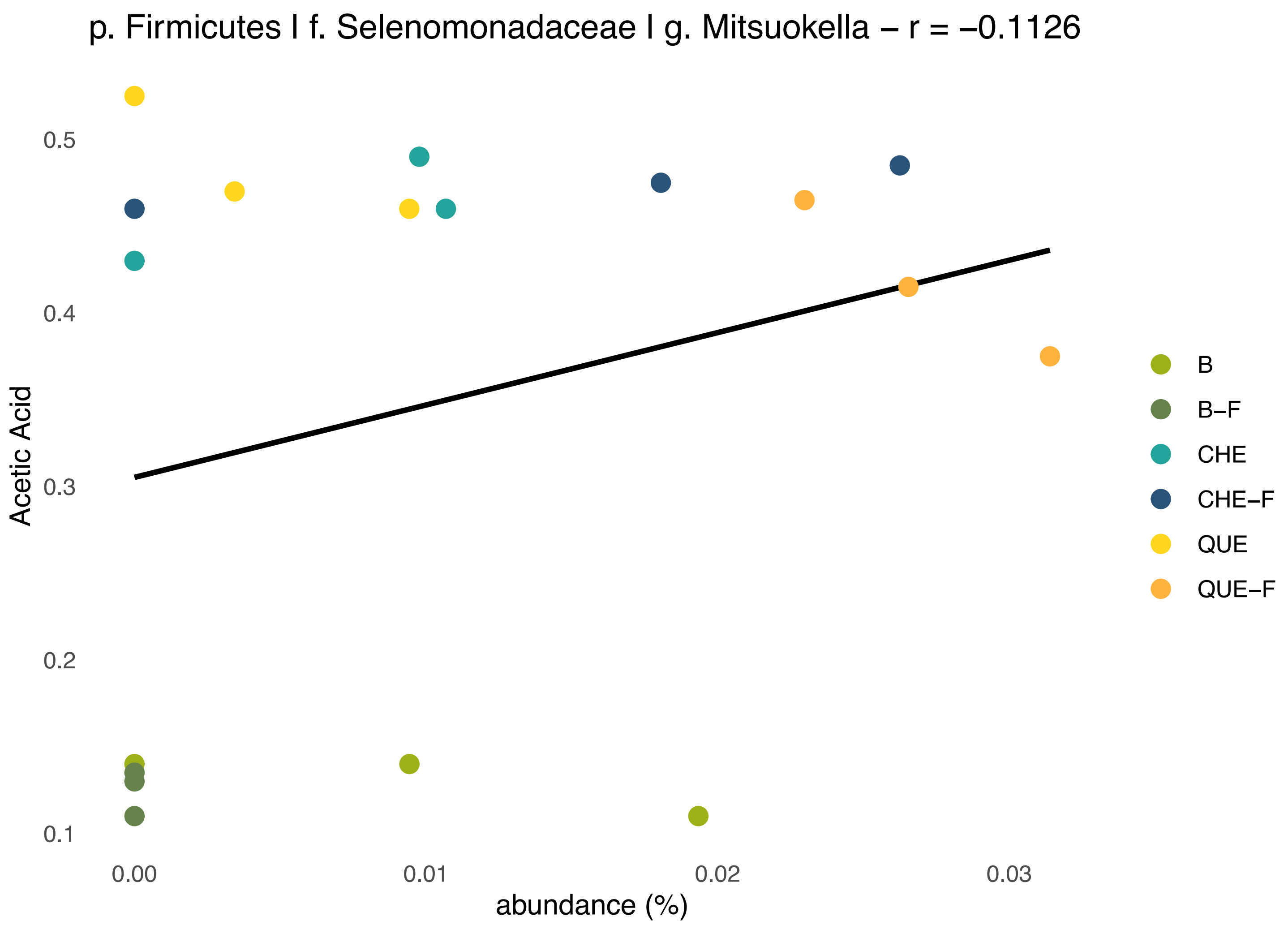

p. Firmicutes I f. Selenomonadaceae I g. Mitsuokella – r = –0.1126
Acetic Acid
0.5
0.4
0.3
0.2
0.1
0.00
0.01
0.02
0.03
abundance (%)
B
B–F
CHE
CHE–F
QUE
QUE–F

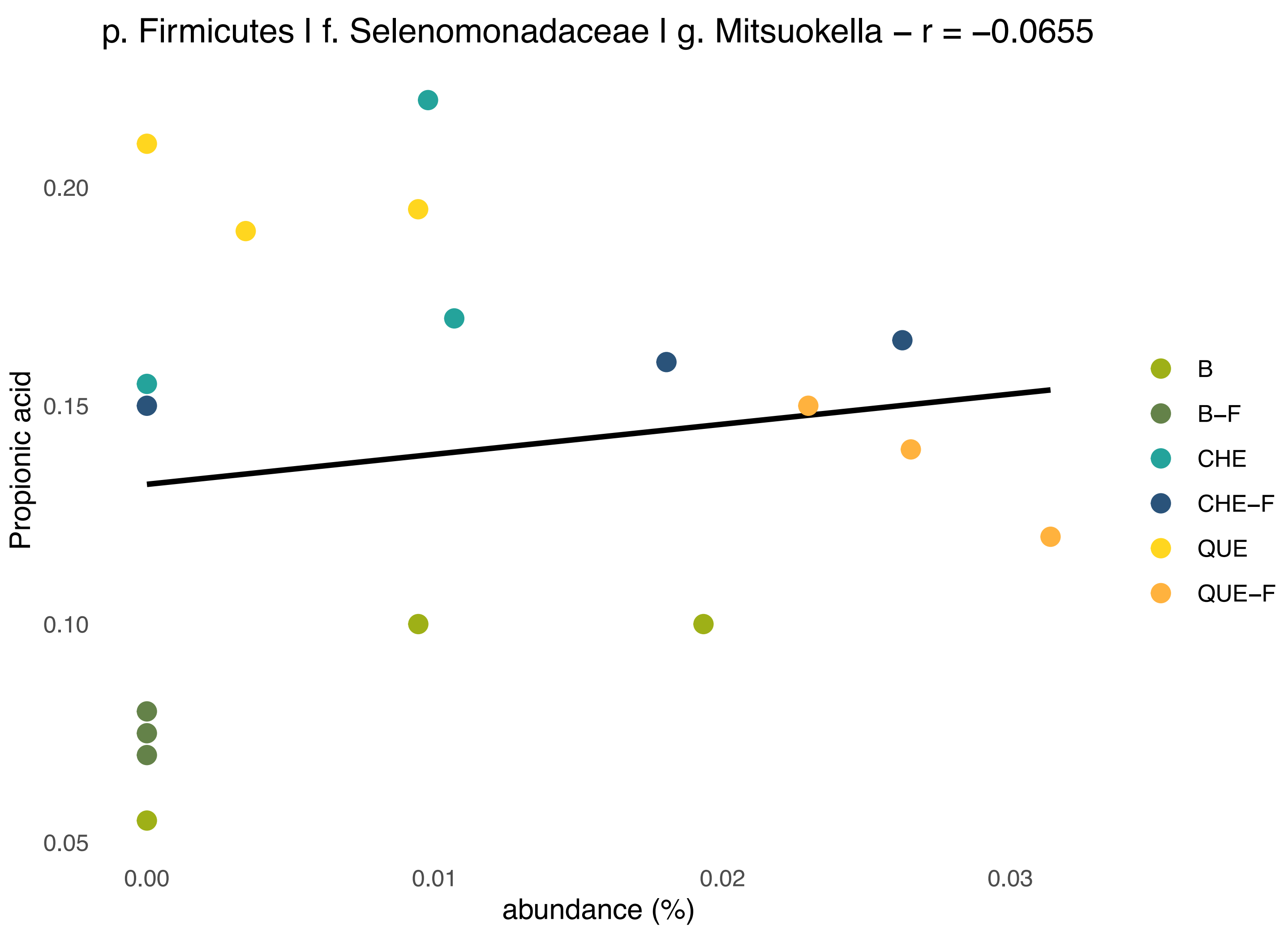
p. Firmicutes I f. Selenomonadaceae I g. Mitsuokella – r = –0.0655
Propionic acid
0.20
0.15
0.10
0.05
0.00
0.01
0.02
0.03
abundance (%)
B
B–F
CHE
CHE–F
QUE
QUE–F

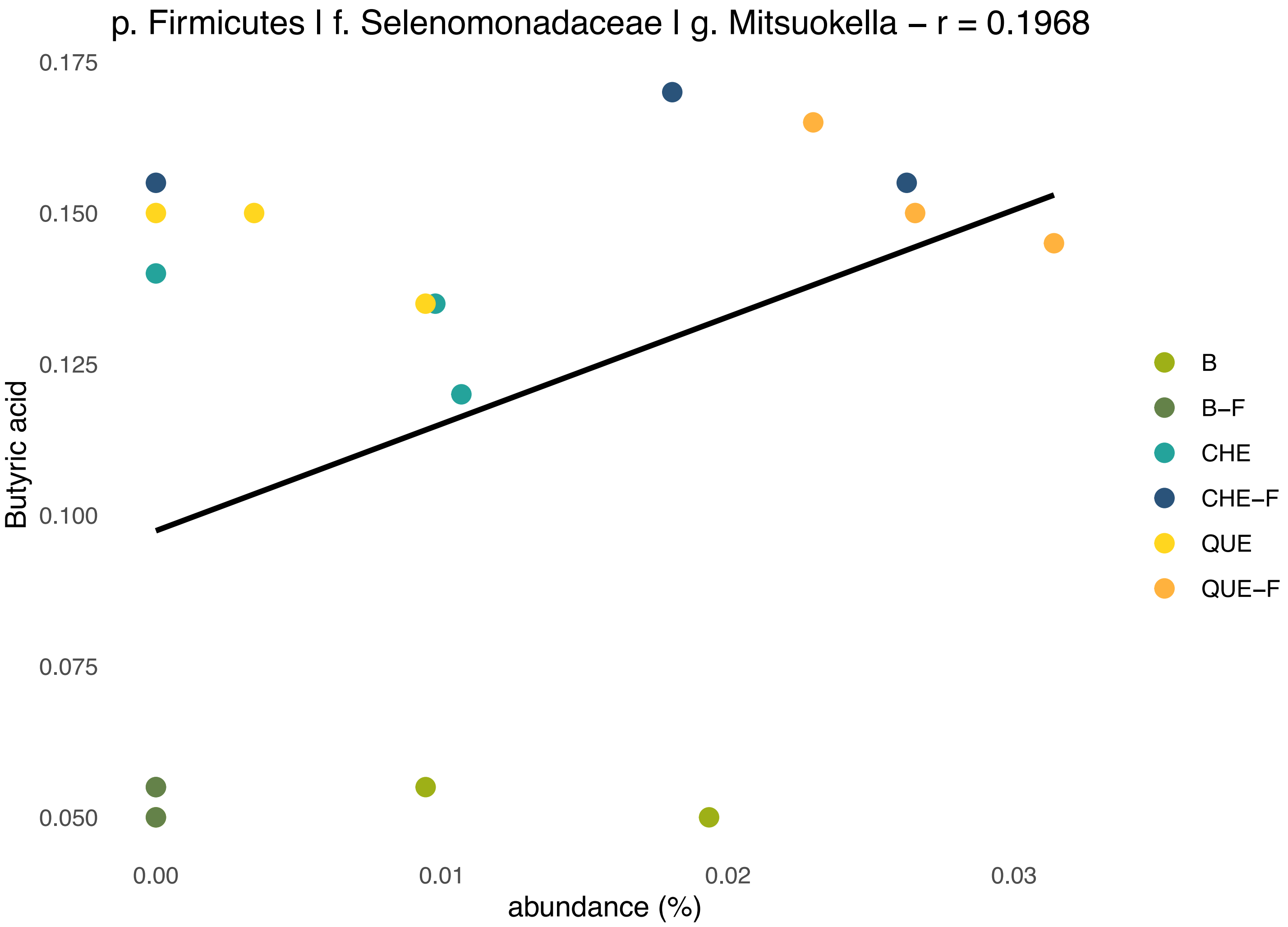
p. Firmicutes I f. Selenomonadaceae I g. Mitsuokella – r = 0.1968
Butyric acid
0.175
0.150
0.125
0.100
0.075
0.050
0.00
0.01
0.02
0.03
abundance (%)
B
B–F
CHE
CHE–F
QUE
QUE–F

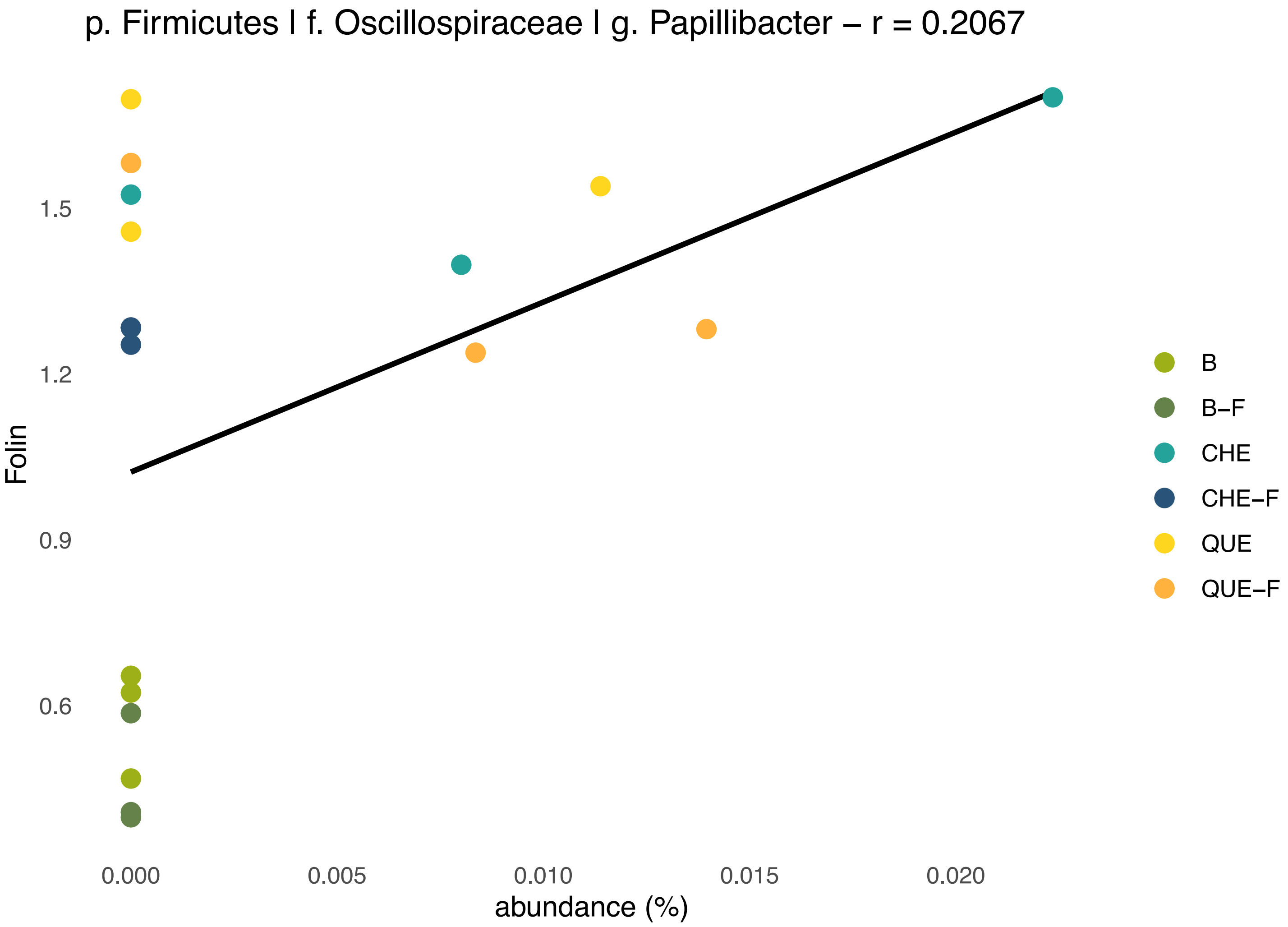
p. Firmicutes I f. Oscillospiraceae I g. Papillibacter – r = 0.2067
Folin
1.5
1.2
0.9
0.6
0.000
0.005
0.010
0.015
0.020
abundance (%)
B
B–F
CHE
CHE–F
QUE
QUE–F

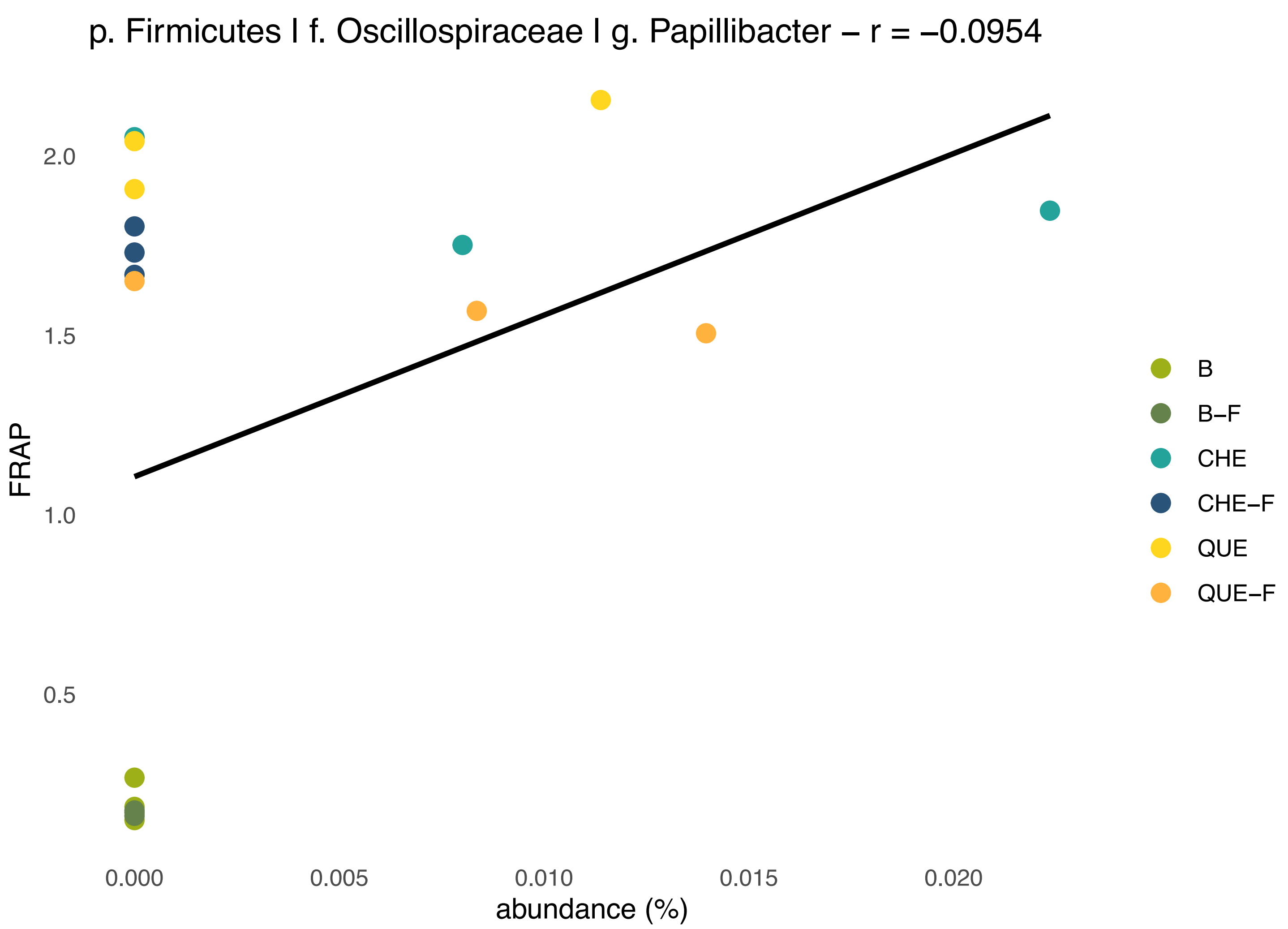
p. Firmicutes I f. Oscillospiraceae I g. Papillibacter – r = −0.0954
FRAP
2.0
1.5
1.0
0.5
0.000
0.005
0.010
0.015
0.020
abundance (%)
B
B−F
CHE
CHE−F
QUE
QUE−F

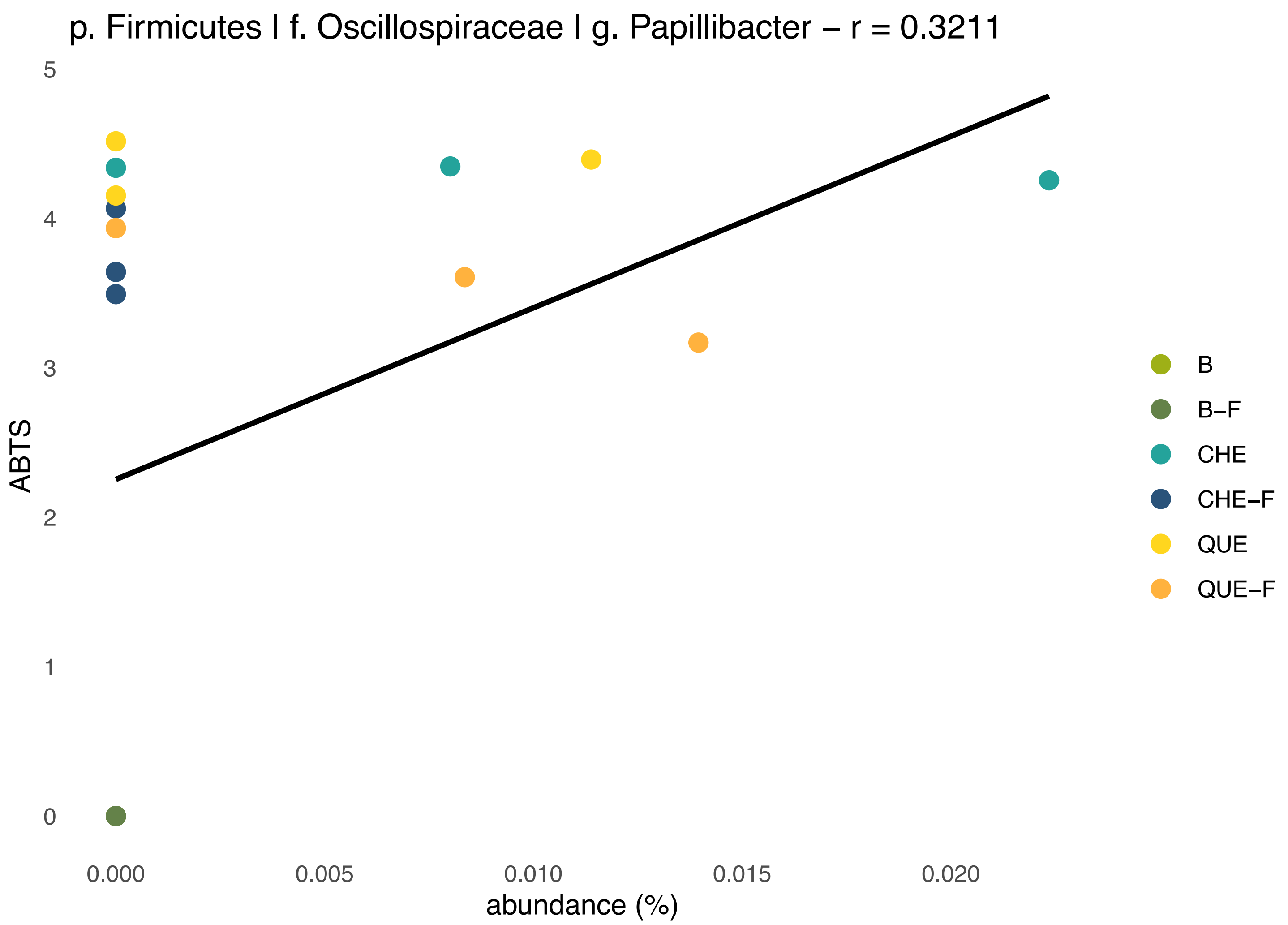
p. Firmicutes I f. Oscillospiraceae I g. Papillibacter – r = 0.3211
ABTS
0
1
2
3
4
5
0.000
0.005
0.010
0.015
0.020
abundance (%)
B
B–F
CHE
CHE–F
QUE
QUE–F

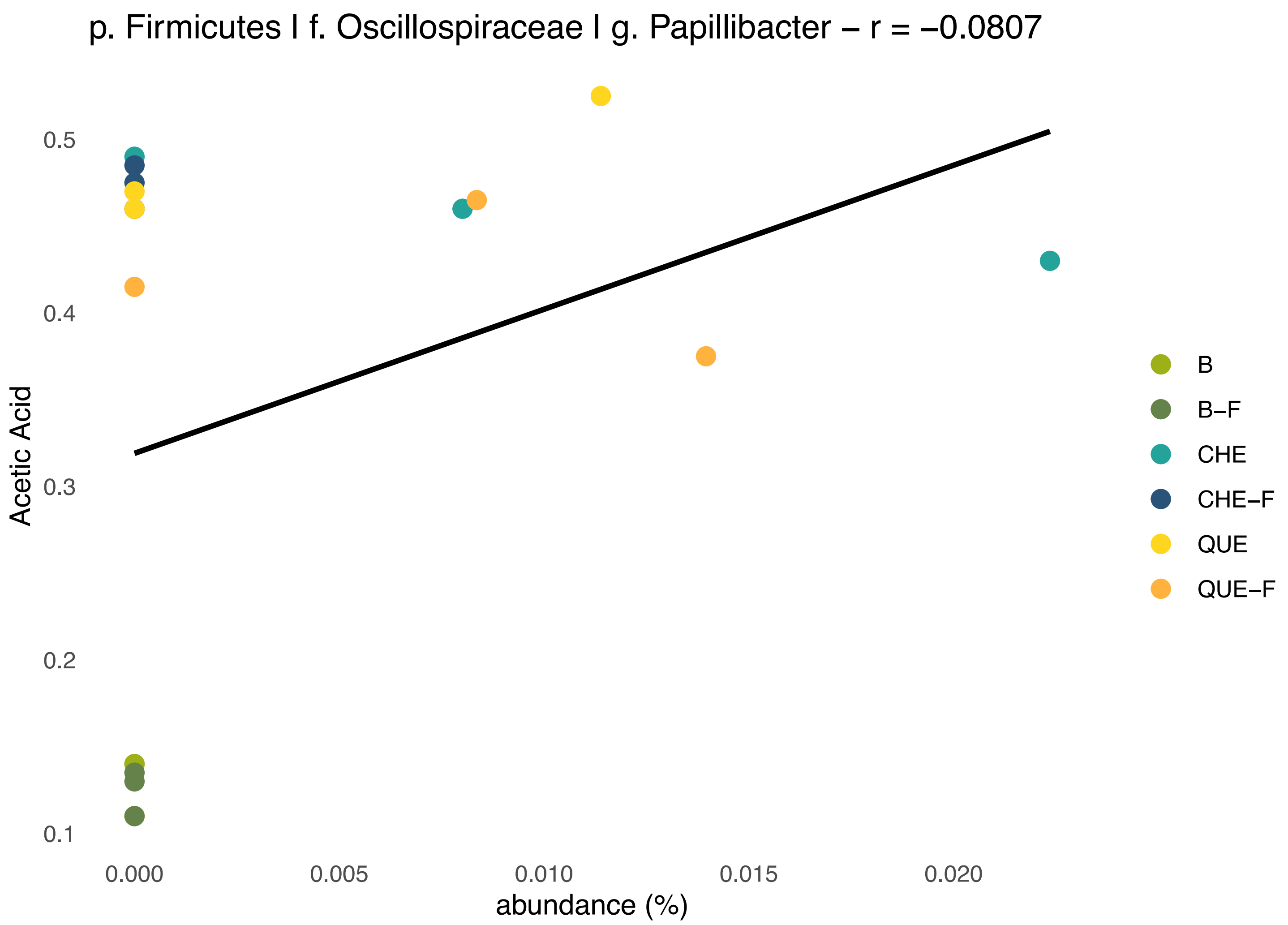

p. Firmicutes I f. Oscillospiraceae I g. Papillibacter – r = –0.0807
Acetic Acid
0.5
0.4
0.3
0.2
0.1
0.000
0.005
0.010
0.015
0.020
abundance (%)
B
B–F
CHE
CHE–F
QUE
QUE–F

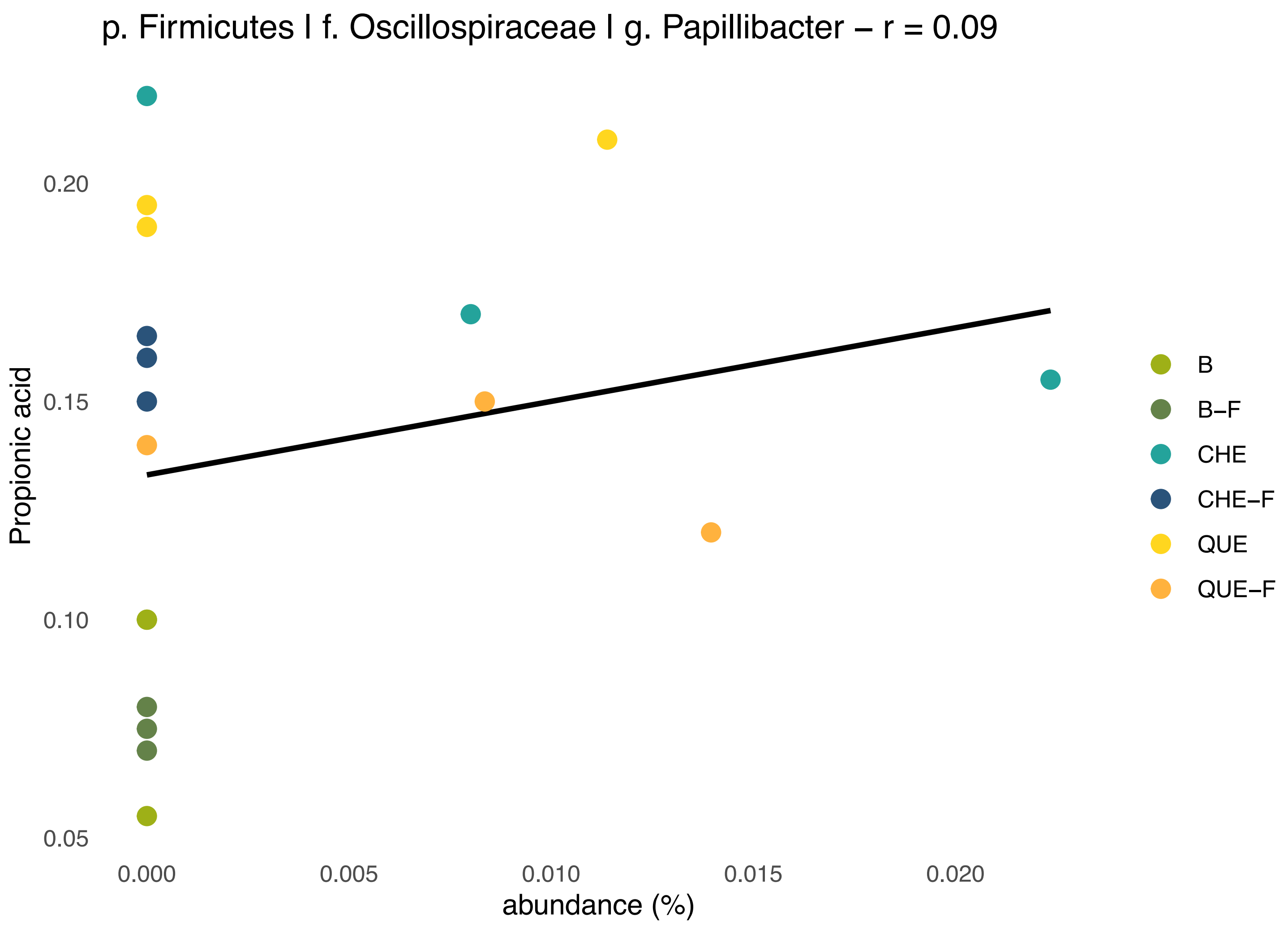

p. Firmicutes I f. Oscillospiraceae I g. Papillibacter – r = 0.09
Propionic acid
0.20
0.15
0.10
0.05
0.000
0.005
0.010
0.015
0.020
abundance (%)
B
B–F
CHE
CHE–F
QUE
QUE–F

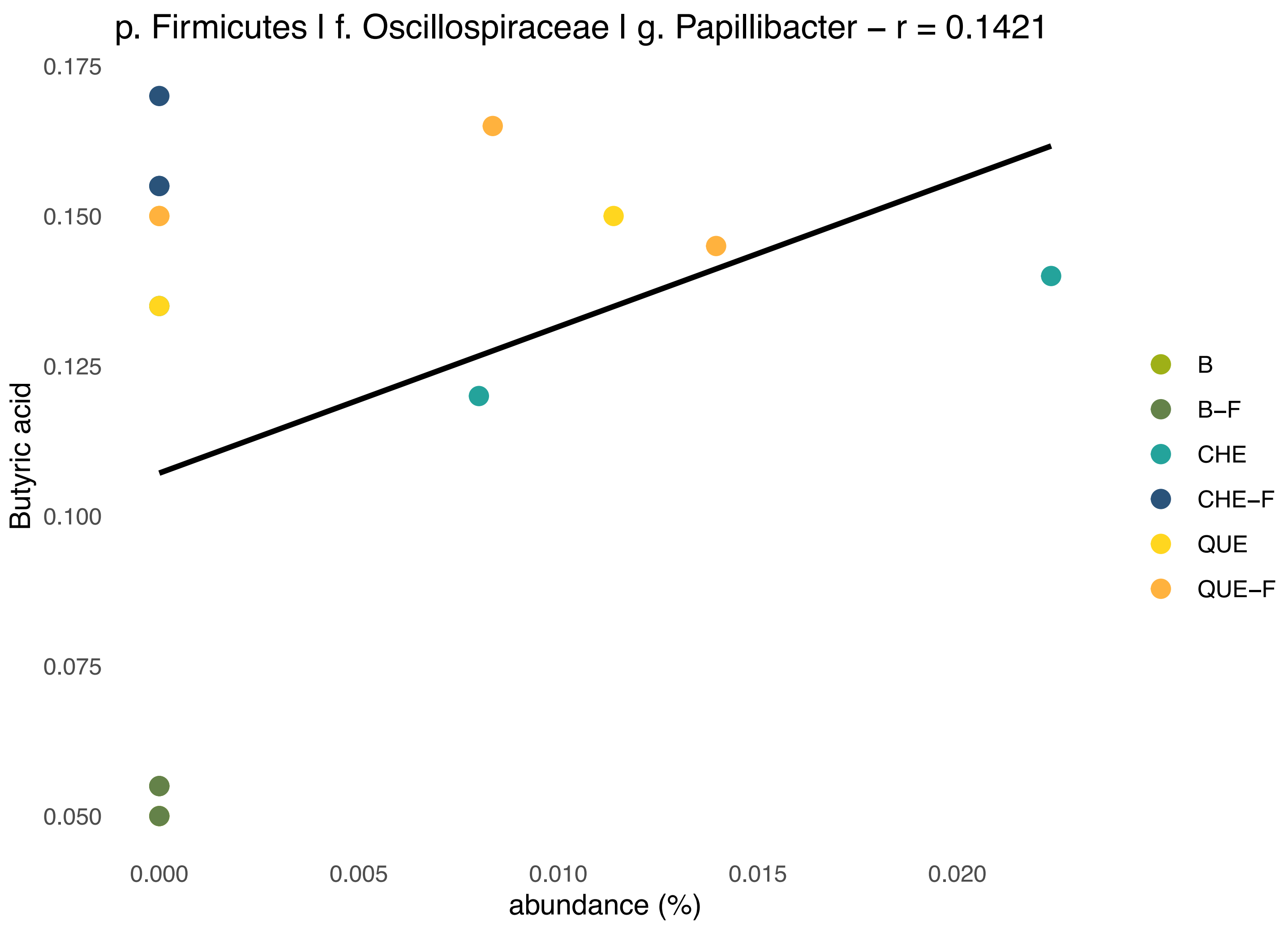
p. Firmicutes l f. Oscillospiraceae l g. Papillibacter – r = 0.1421
Butyric acid
0.175
0.150
0.125
0.100
0.075
0.050
0.000
0.005
0.010
0.015
0.020
abundance (%)
B
B–F
CHE
CHE–F
QUE
QUE–F

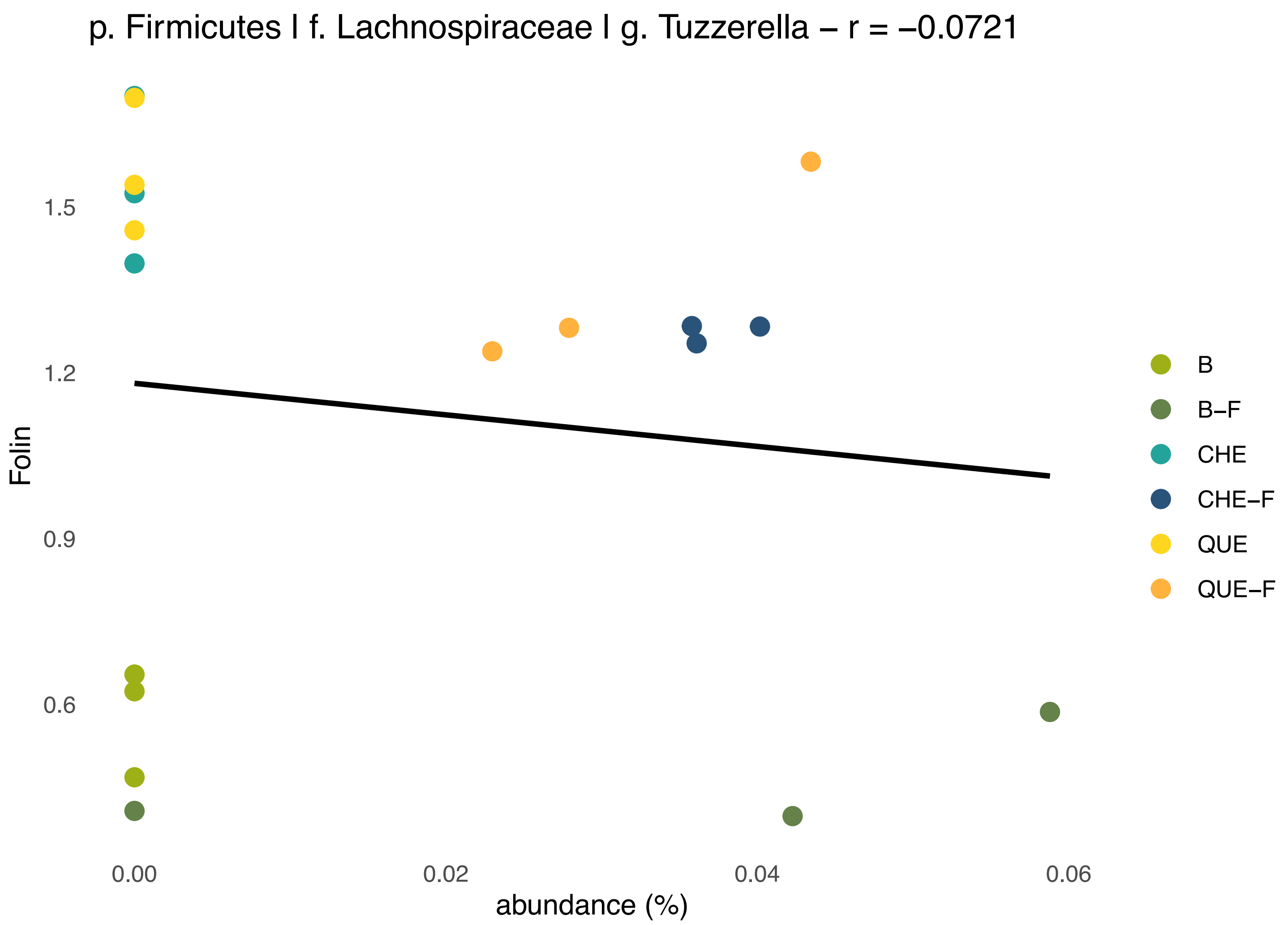
p. Firmicutes l f. Lachnospiraceae l g. Tuzzerella – r = –0.0721
1.5
1.2
0.9
0.6
Folin
0.00
0.02
0.04
0.06
abundance (%)
B
B–F
CHE
CHE–F
QUE
QUE–F

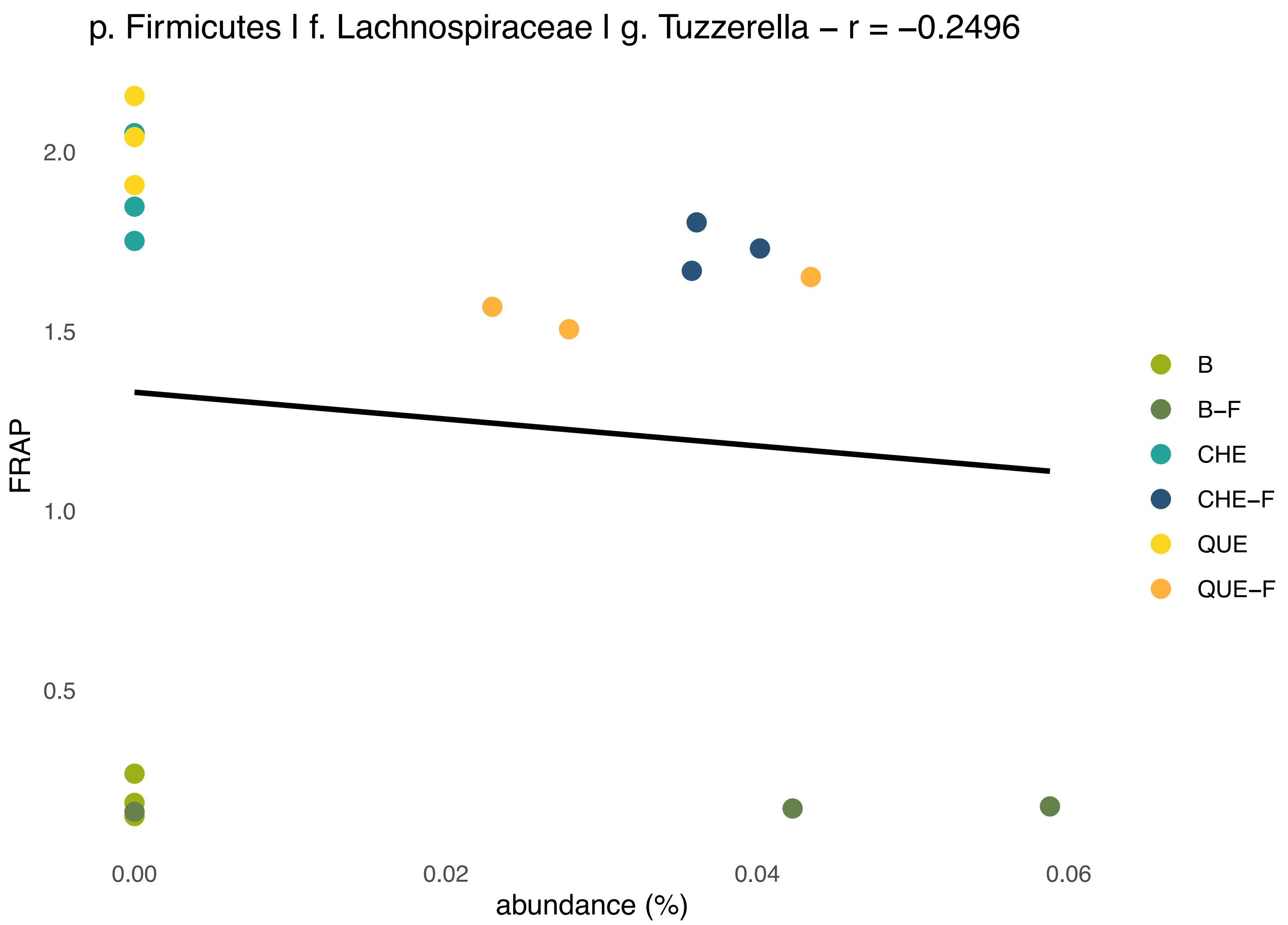

p. Firmicutes I f. Lachnospiraceae I g. Tuzzerella – r = –0.2496
FRAP
2.0
1.5
1.0
0.5
0.00
0.02
0.04
0.06
abundance (%)
B
B–F
CHE
CHE–F
QUE
QUE–F

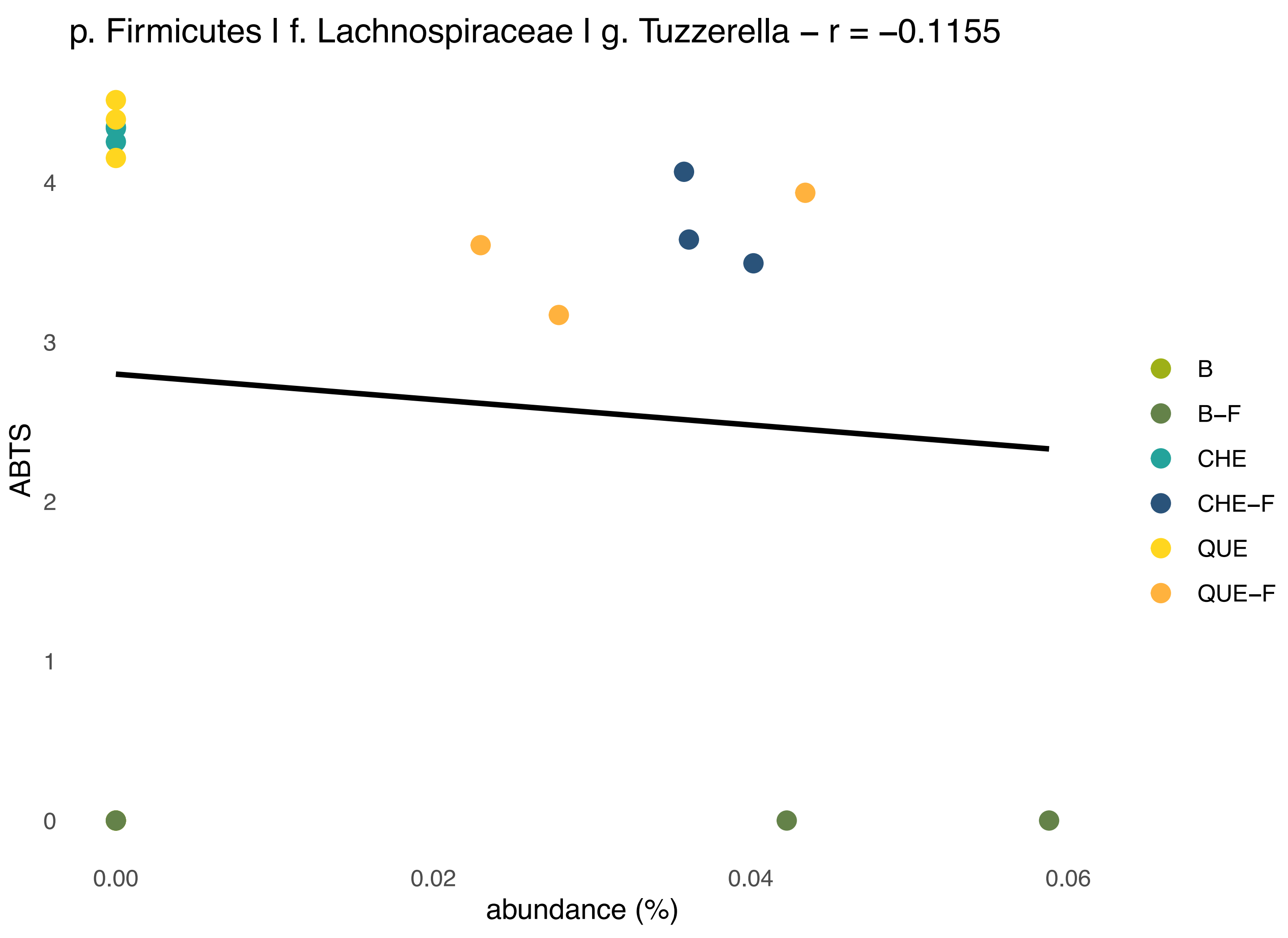

p. Firmicutes l f. Lachnospiraceae l g. Tuzzerella – r = −0.1155
ABTS
4
3
2
1
0
0.00
0.02
0.04
0.06
abundance (%)
B
B−F
CHE
CHE−F
QUE
QUE−F

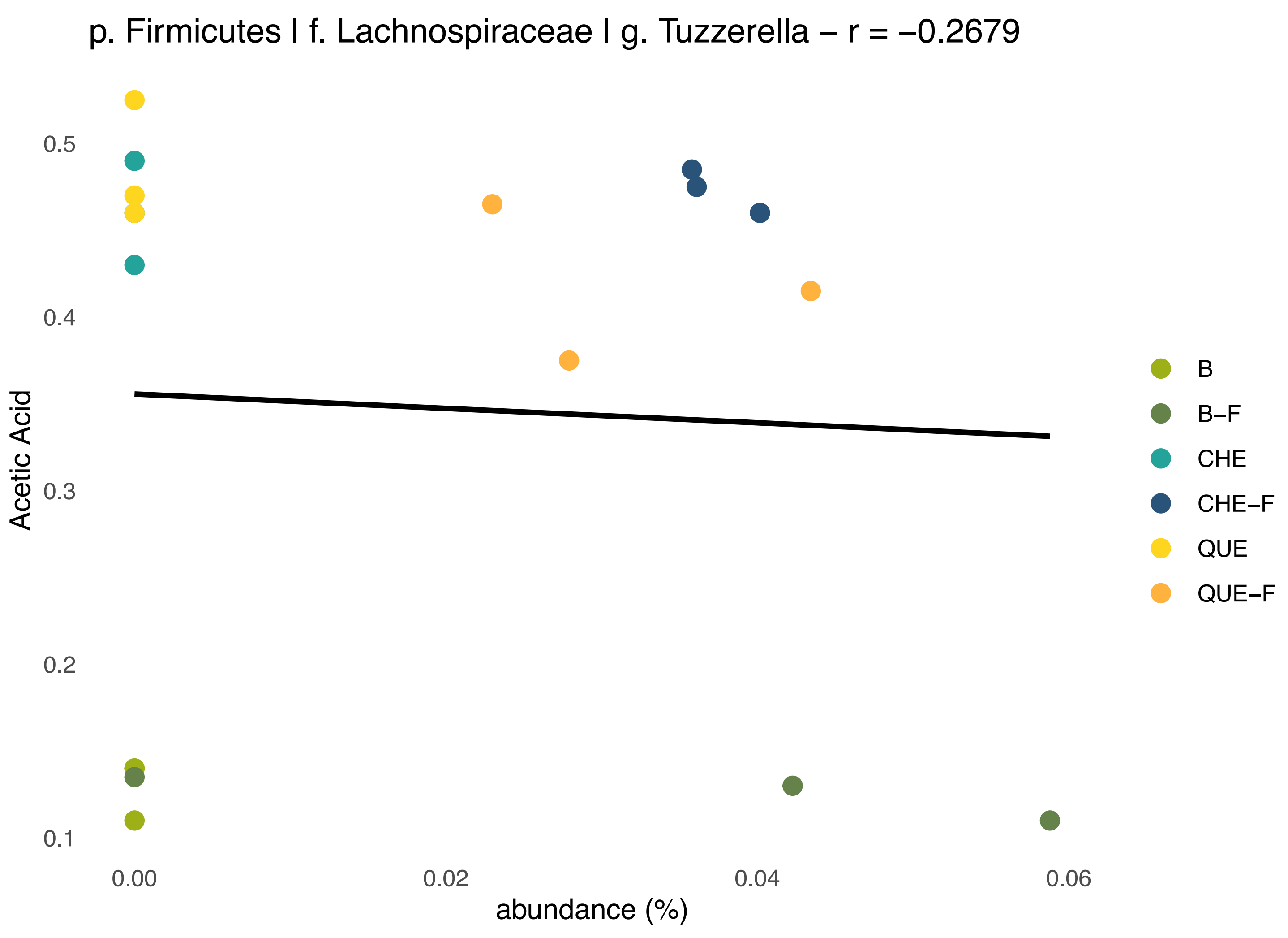
p. Firmicutes I f. Lachnospiraceae I g. Tuzzerella – r = –0.2679
Acetic Acid
0.5
0.4
0.3
0.2
0.1
0.00
0.02
0.04
0.06
abundance (%)
B
B–F
CHE
CHE–F
QUE
QUE–F

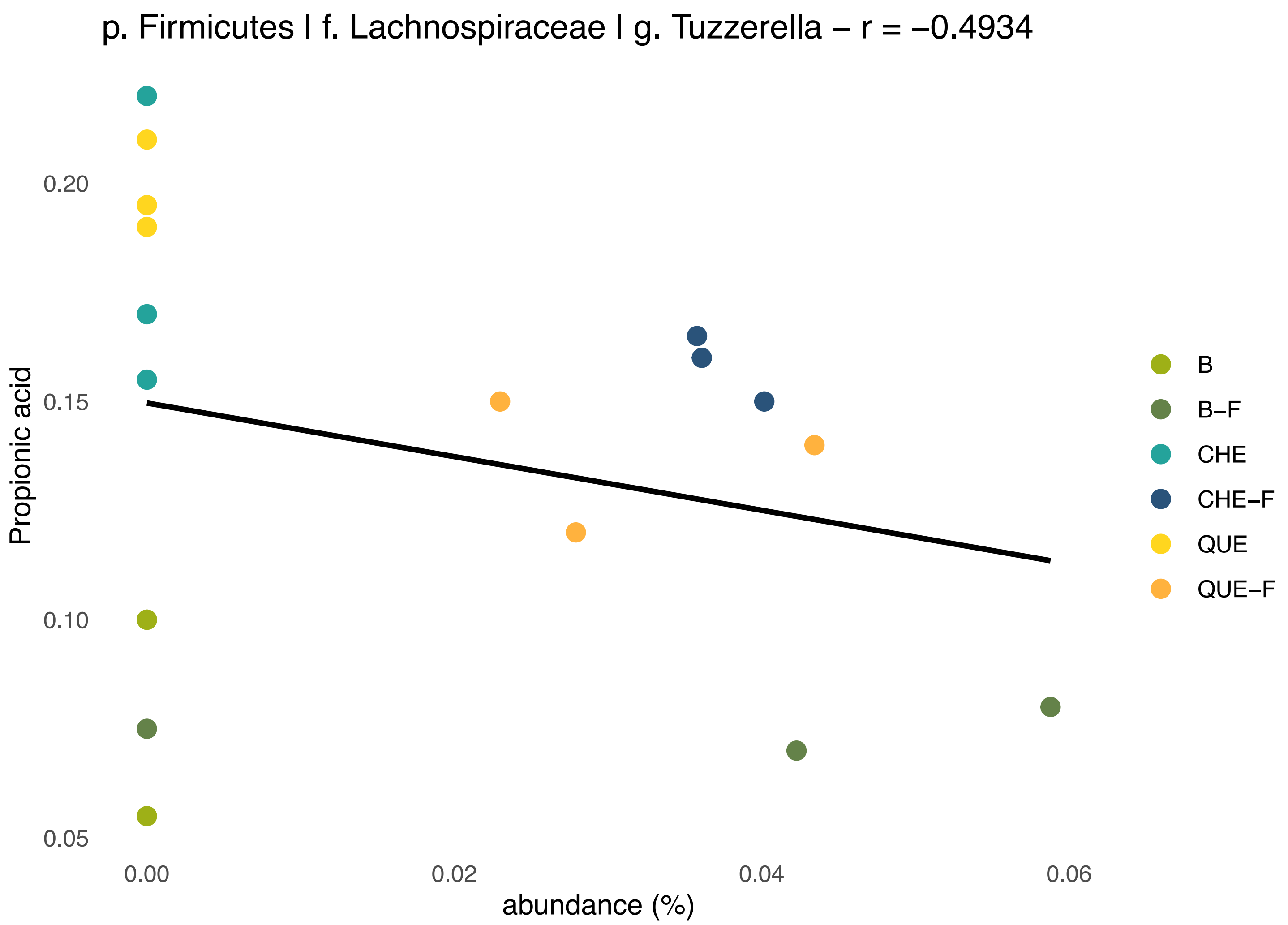

p. Firmicutes l f. Lachnospiraceae l g. Tuzzerella – r = –0.4934
Propionic acid
0.20
0.15
0.10
0.05
0.00
0.02
0.04
0.06
abundance (%)
B
B–F
CHE
CHE–F
QUE
QUE–F

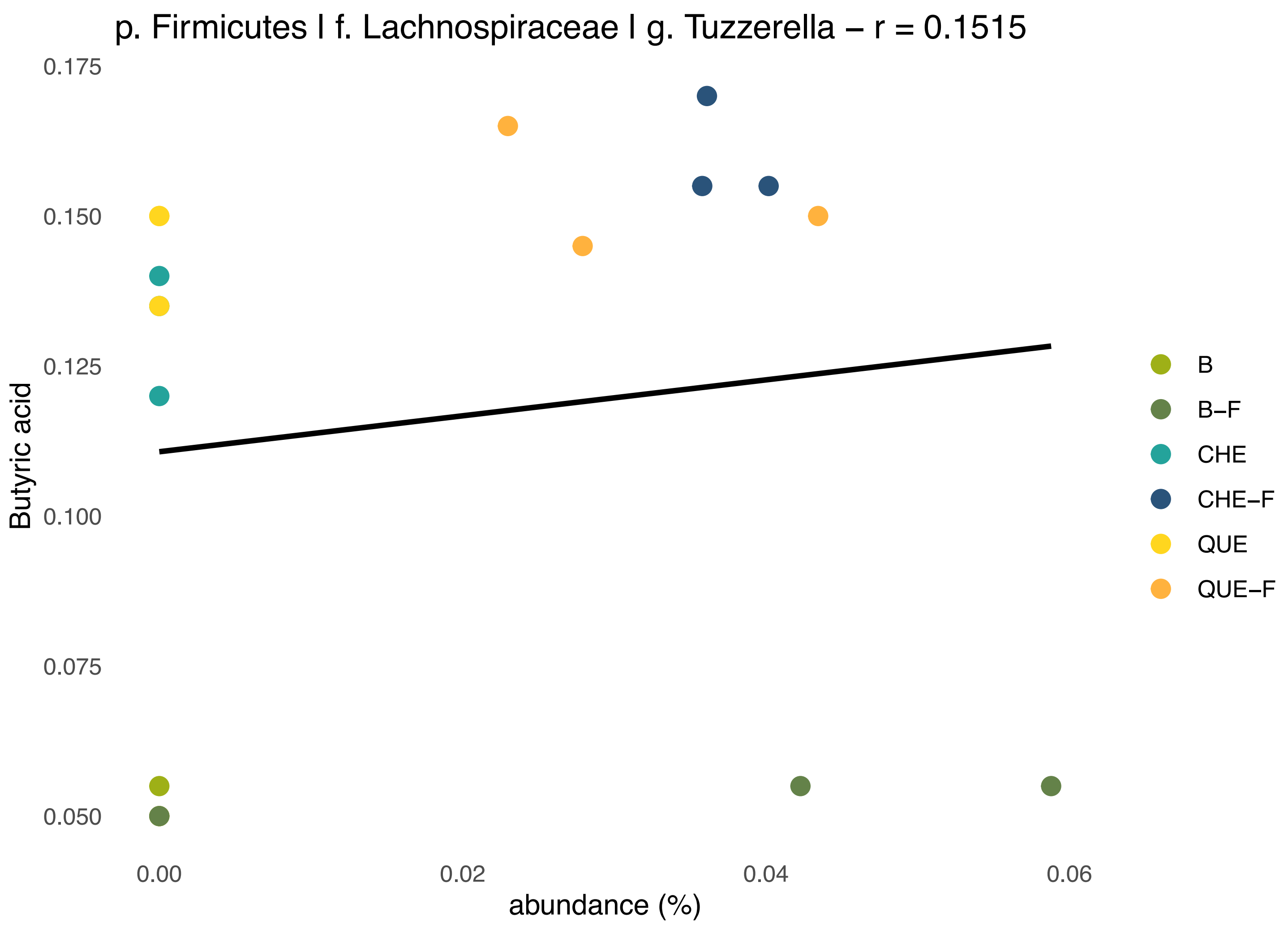
p. Firmicutes I f. Lachnospiraceae I g. Tuzzerella – r = 0.1515
Butyric acid
0.175
0.150
0.125
0.100
0.075
0.050
0.00
0.02
0.04
0.06
abundance (%)
B
B–F
CHE
CHE–F
QUE
QUE–F

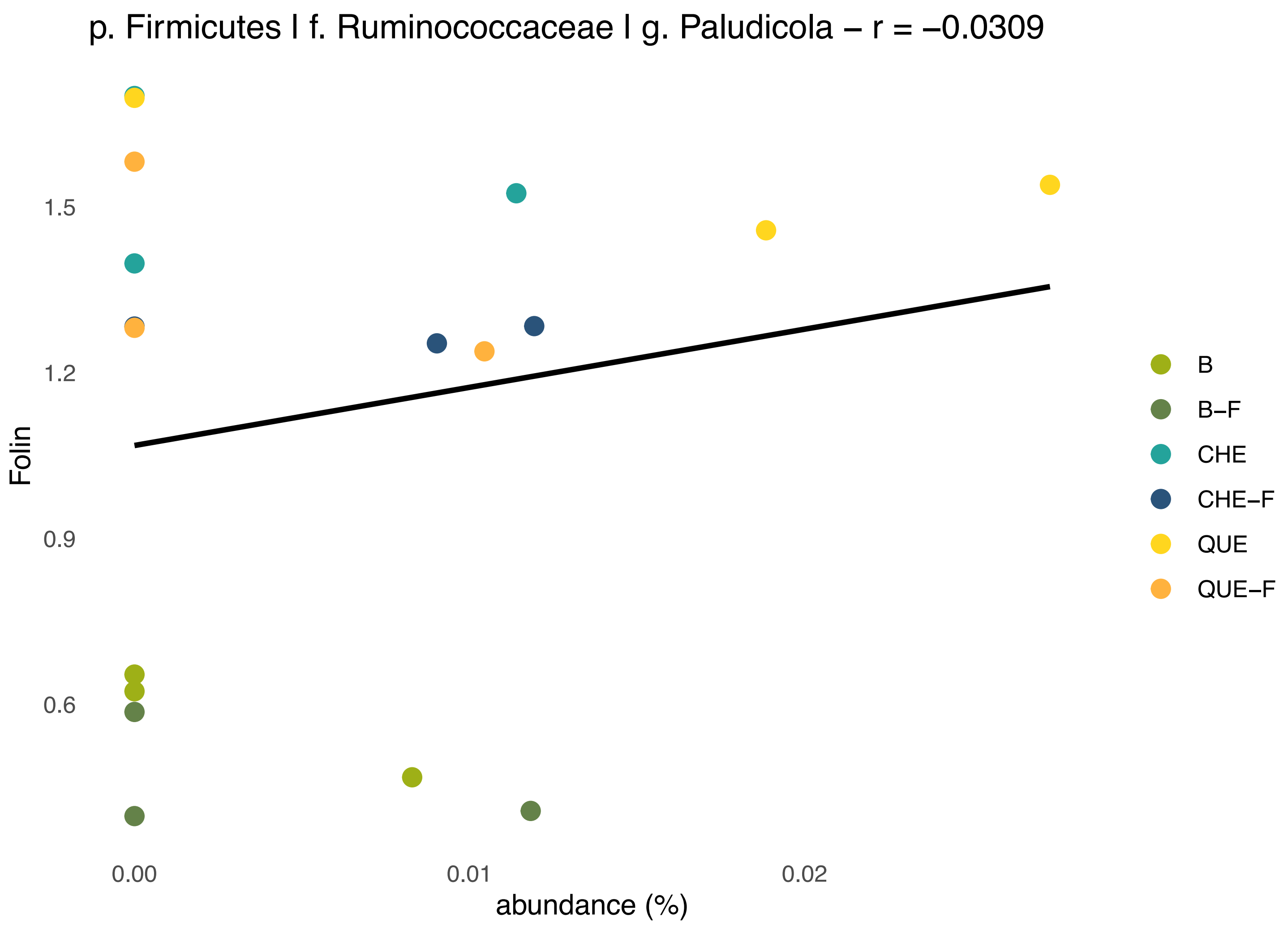

p. Firmicutes | f. Ruminococcaceae | g. Paludicola – r = –0.0309
Folin
1.5
1.2
0.9
0.6
0.00
0.01
0.02
abundance (%)
B
B–F
CHE
CHE–F
QUE
QUE–F

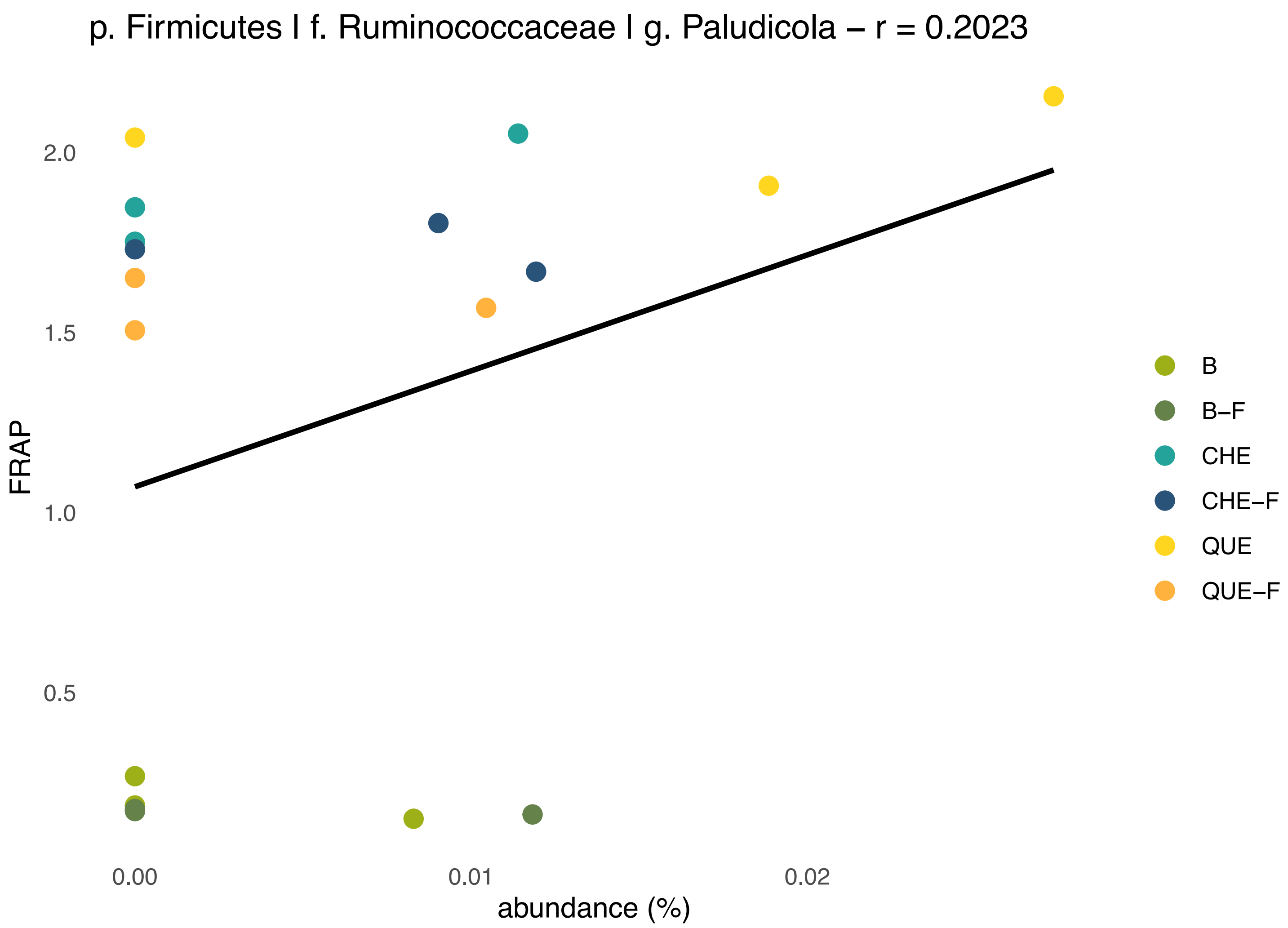
p. Firmicutes | f. Ruminococcaceae | g. Paludicola – r = 0.2023
FRAP
2.0
1.5
1.0
0.5
0.00
0.01
0.02
abundance (%)
B
B–F
CHE
CHE–F
QUE
QUE–F

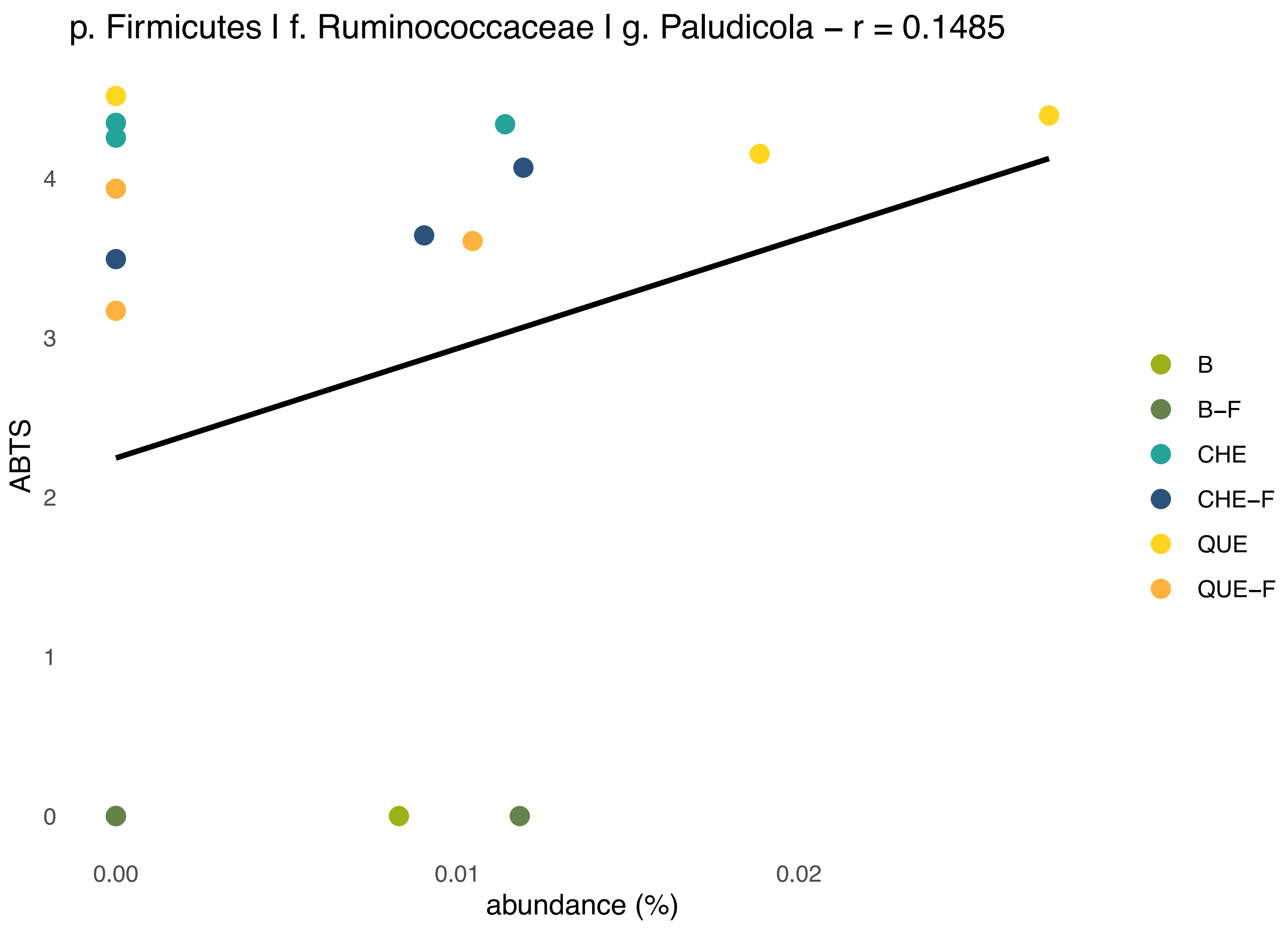

p. Firmicutes I f. Ruminococcaceae I g. Paludicola – r = 0.1485
ABTS
4
3
2
1
0
0.00
0.01
0.02
abundance (%)
B
B–F
CHE
CHE–F
QUE
QUE–F

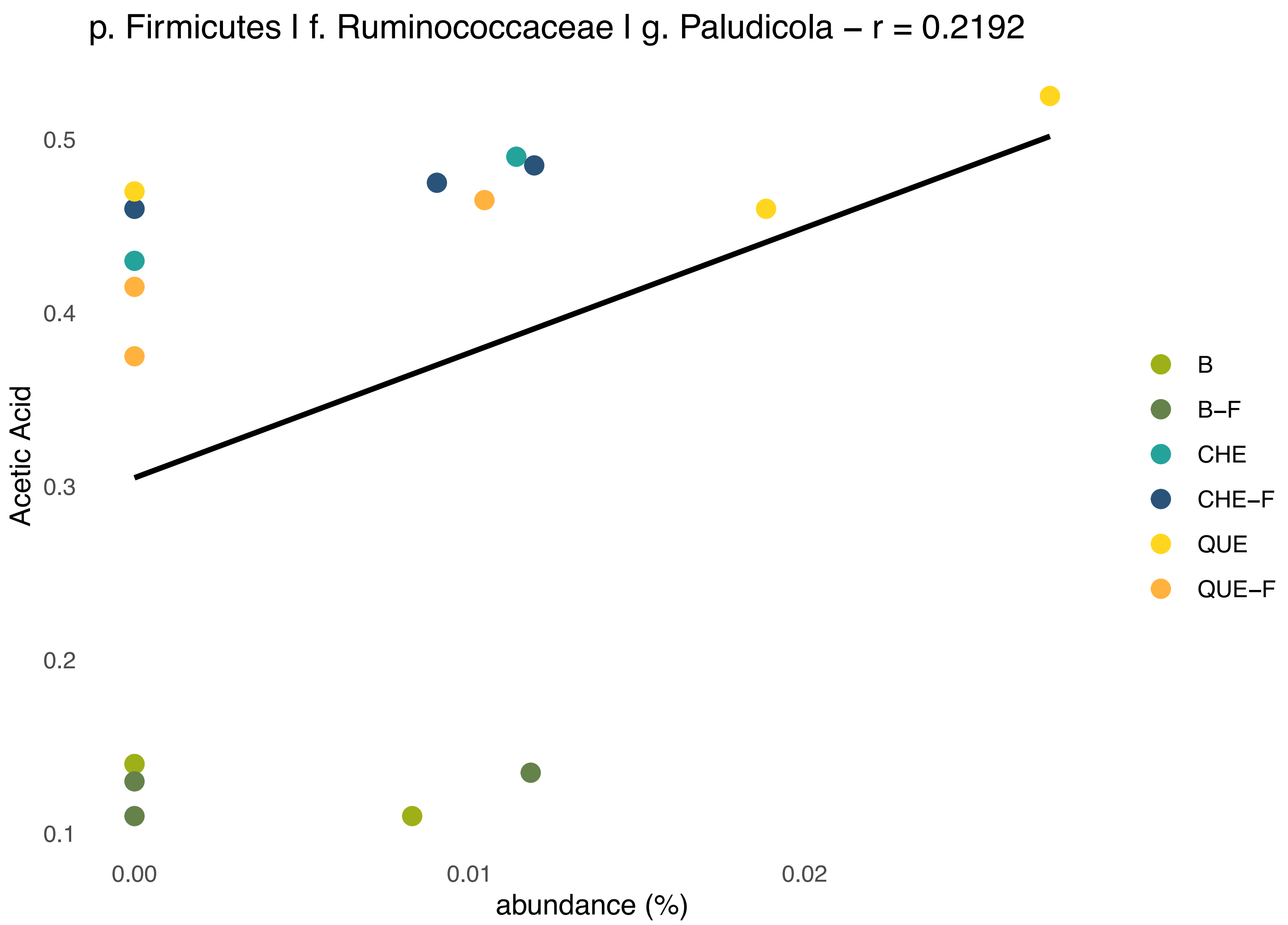
p. Firmicutes I f. Ruminococcaceae I g. Paludicola – r = 0.2192
Acetic Acid
0.5
0.4
0.3
0.2
0.1
0.00
0.01
0.02
abundance (%)
B
B−F
CHE
CHE−F
QUE
QUE−F

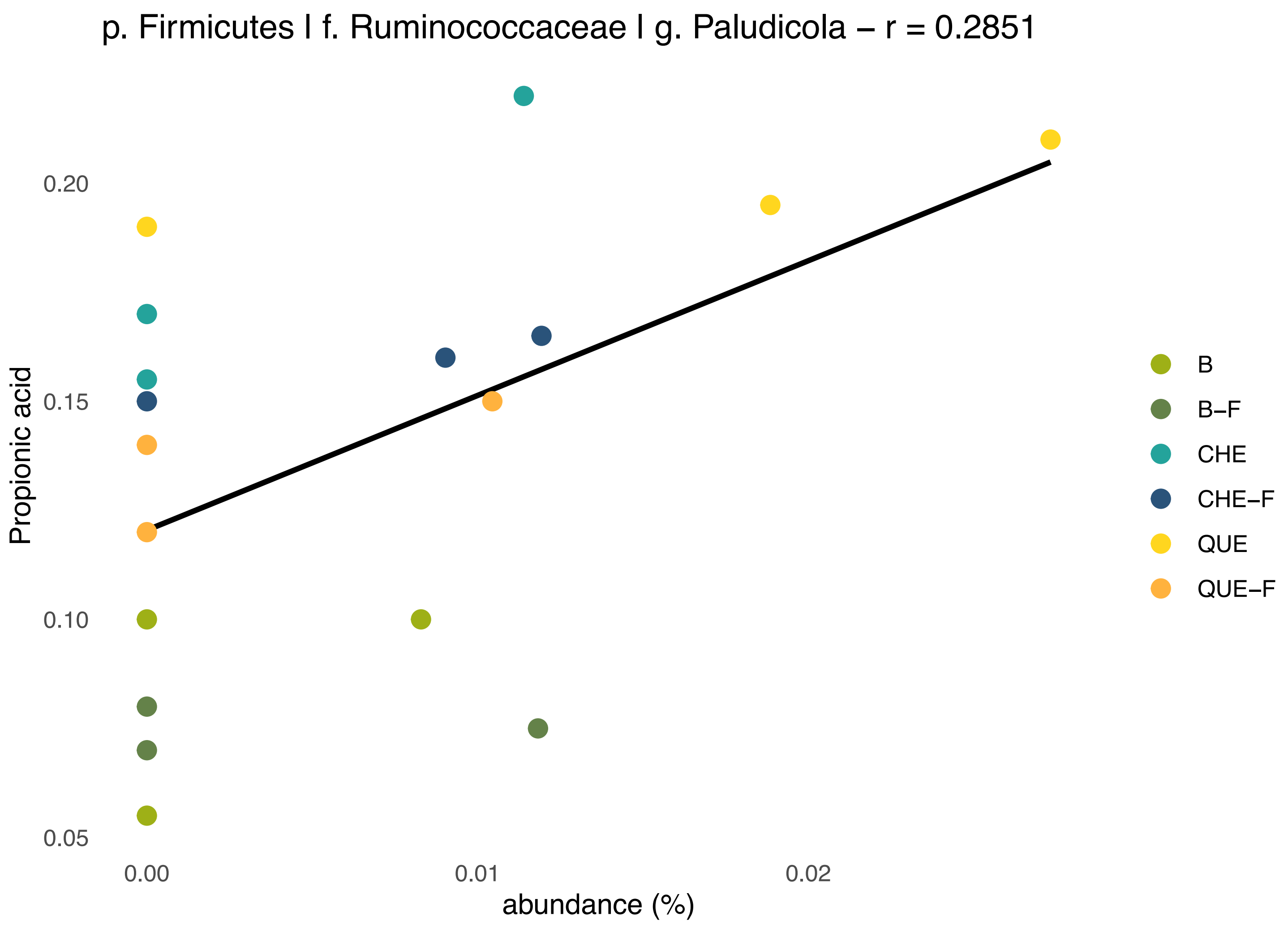

p. Firmicutes l f. Ruminococcaceae l g. Paludicola – r = 0.2851
Propionic acid
0.20
0.15
0.10
0.05
0.00
0.01
0.02
abundance (%)
B
B–F
CHE
CHE–F
QUE
QUE–F

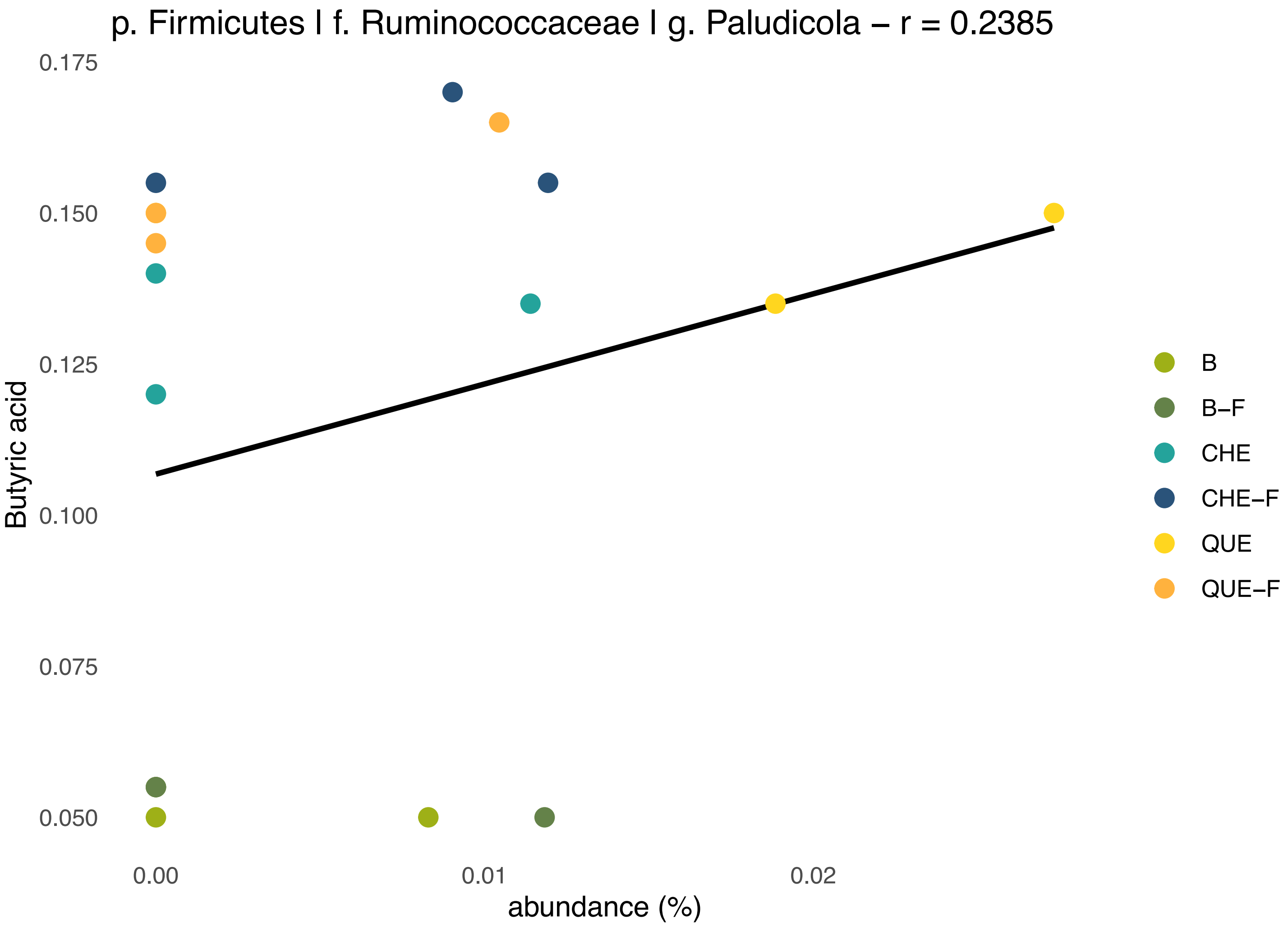

p. Firmicutes I f. Ruminococcaceae I g. Paludicola – r = 0.2385
0.175
0.150
0.125
0.100
0.075
0.050
Butyric acid
0.00
0.01
0.02
abundance (%)
B
B–F
CHE
CHE–F
QUE
QUE–F

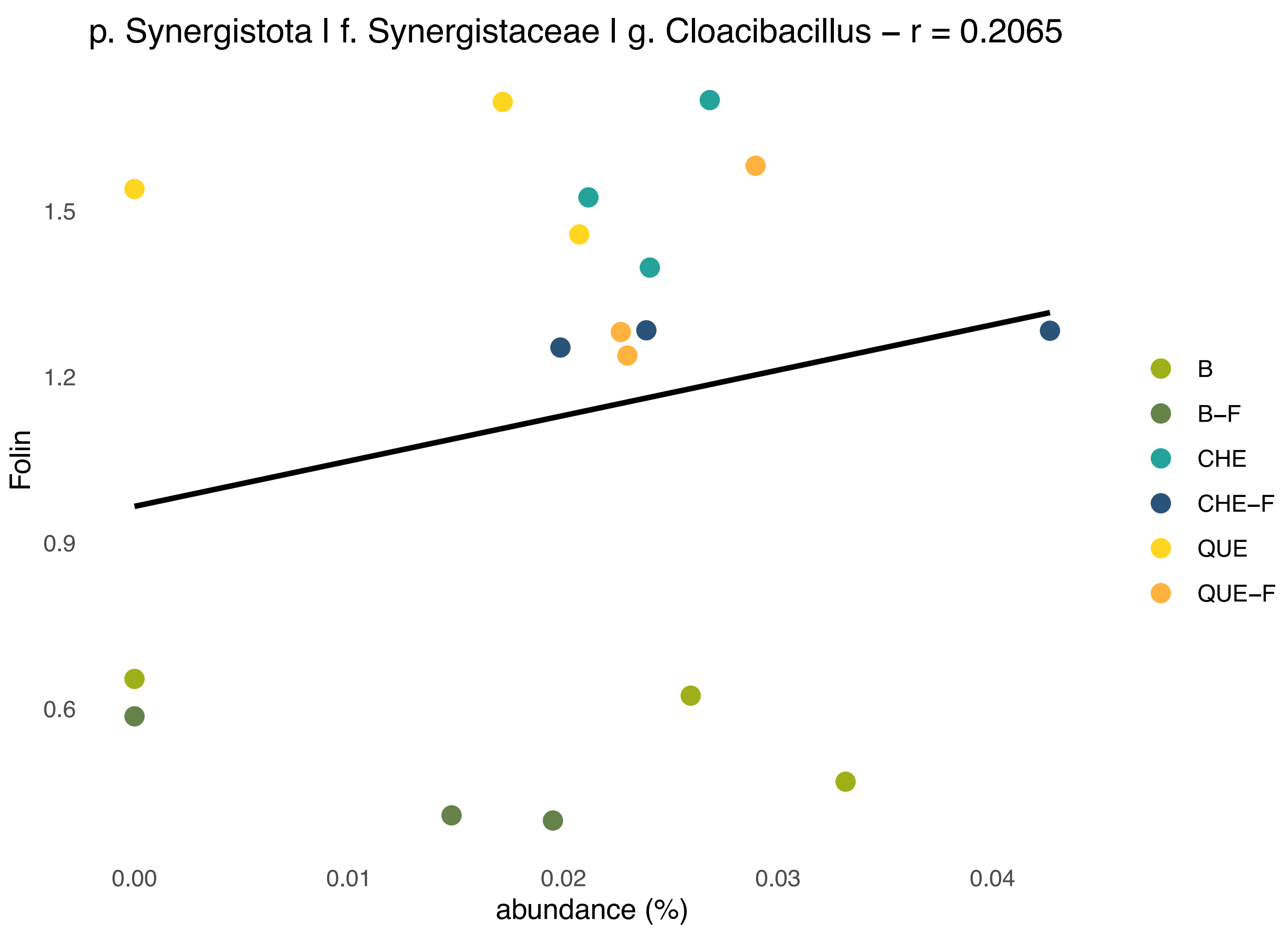
p. Synergistota I f. Synergistaceae I g. Cloacibacillus – r = 0.2065
Folin
1.5
1.2
0.9
0.6
0.00
0.01
0.02
0.03
0.04
abundance (%)
B
B–F
CHE
CHE–F
QUE
QUE–F

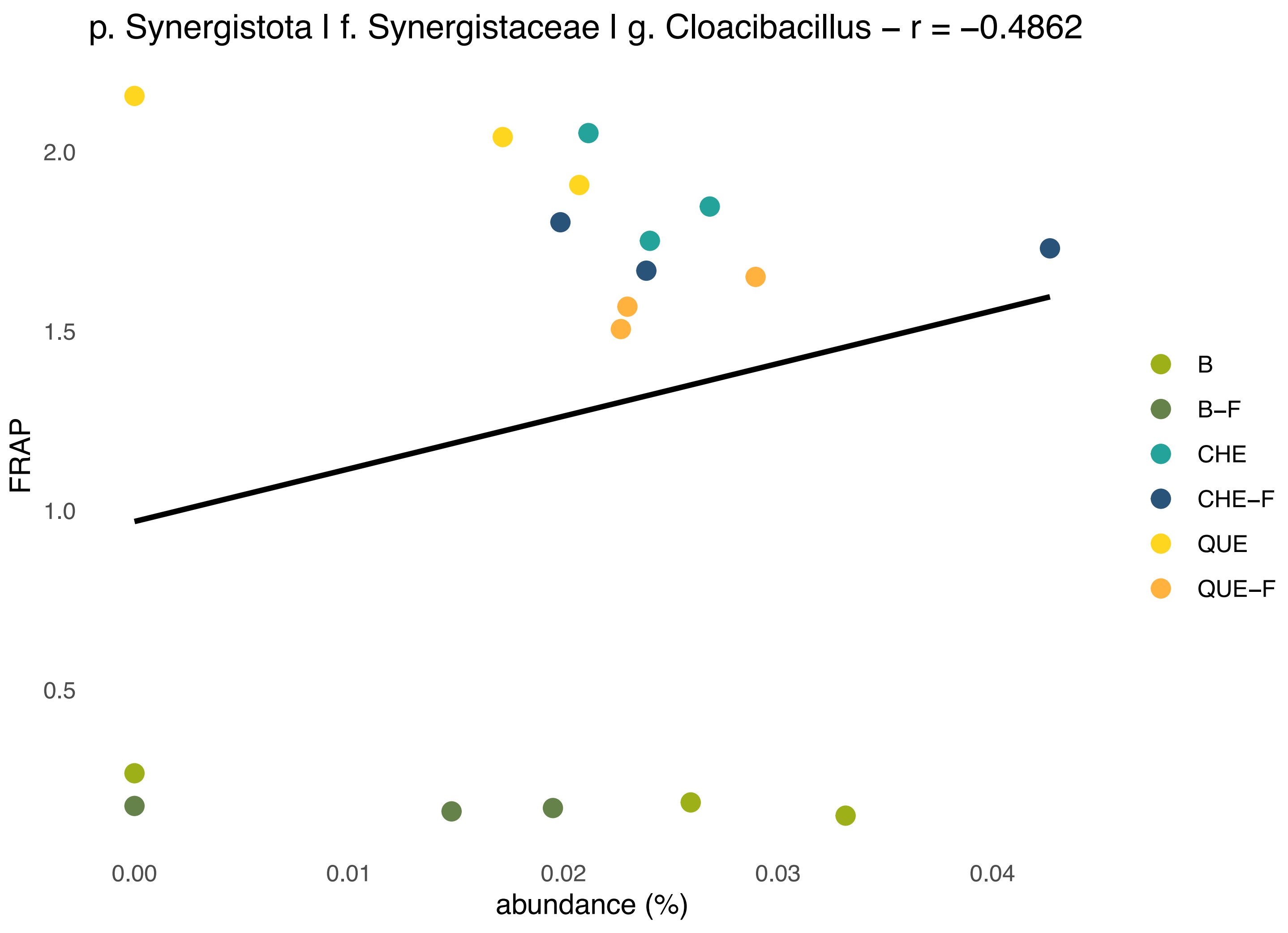

p. Synergistota | f. Synergistaceae | g. Cloacibacillus – r = –0.4862
FRAP
2.0
1.5
1.0
0.5
0.00
0.01
0.02
0.03
0.04
abundance (%)
B
B–F
CHE
CHE–F
QUE
QUE–F

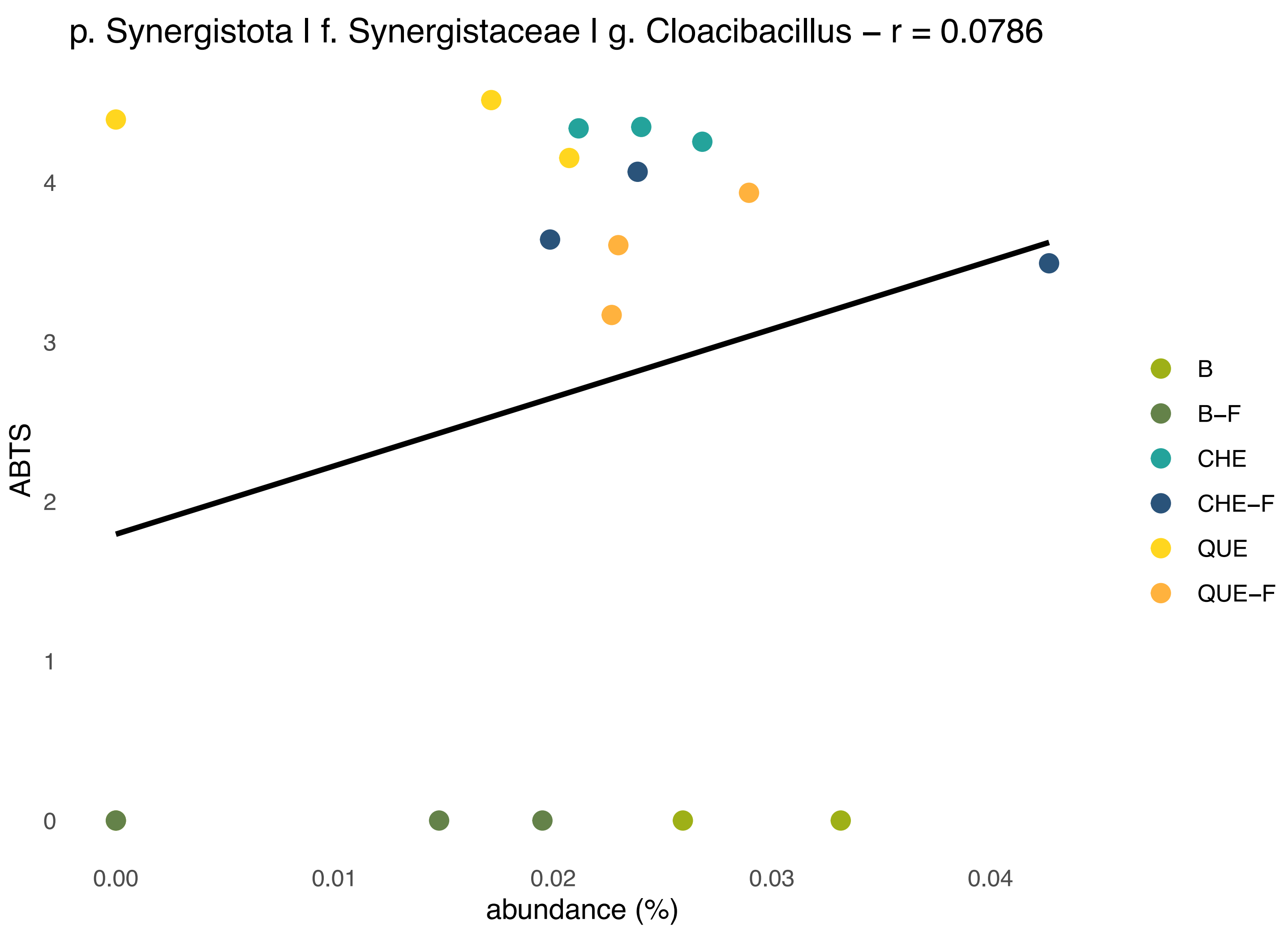

p. Synergistota I f. Synergistaceae I g. Cloacibacillus – r = 0.0786
ABTS
0
1
2
3
4
0.00
0.01
0.02
0.03
0.04
abundance (%)
B
B–F
CHE
CHE–F
QUE
QUE–F

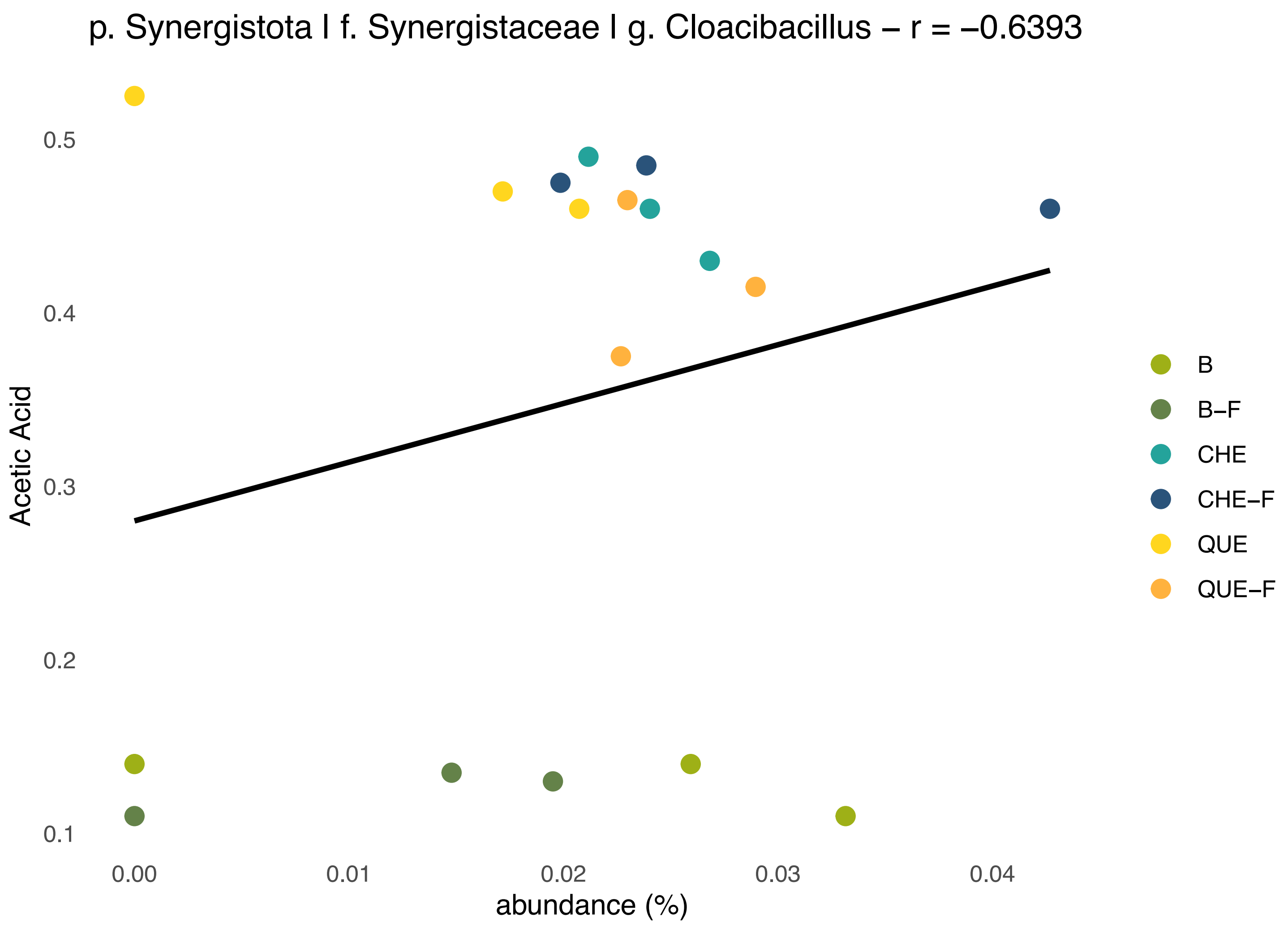
p. Synergistota I f. Synergistaceae I g. Cloacibacillus – r = –0.6393
Acetic Acid
0.5
0.4
0.3
0.2
0.1
0.00
0.01
0.02
0.03
0.04
abundance (%)
B
B–F
CHE
CHE–F
QUE
QUE–F

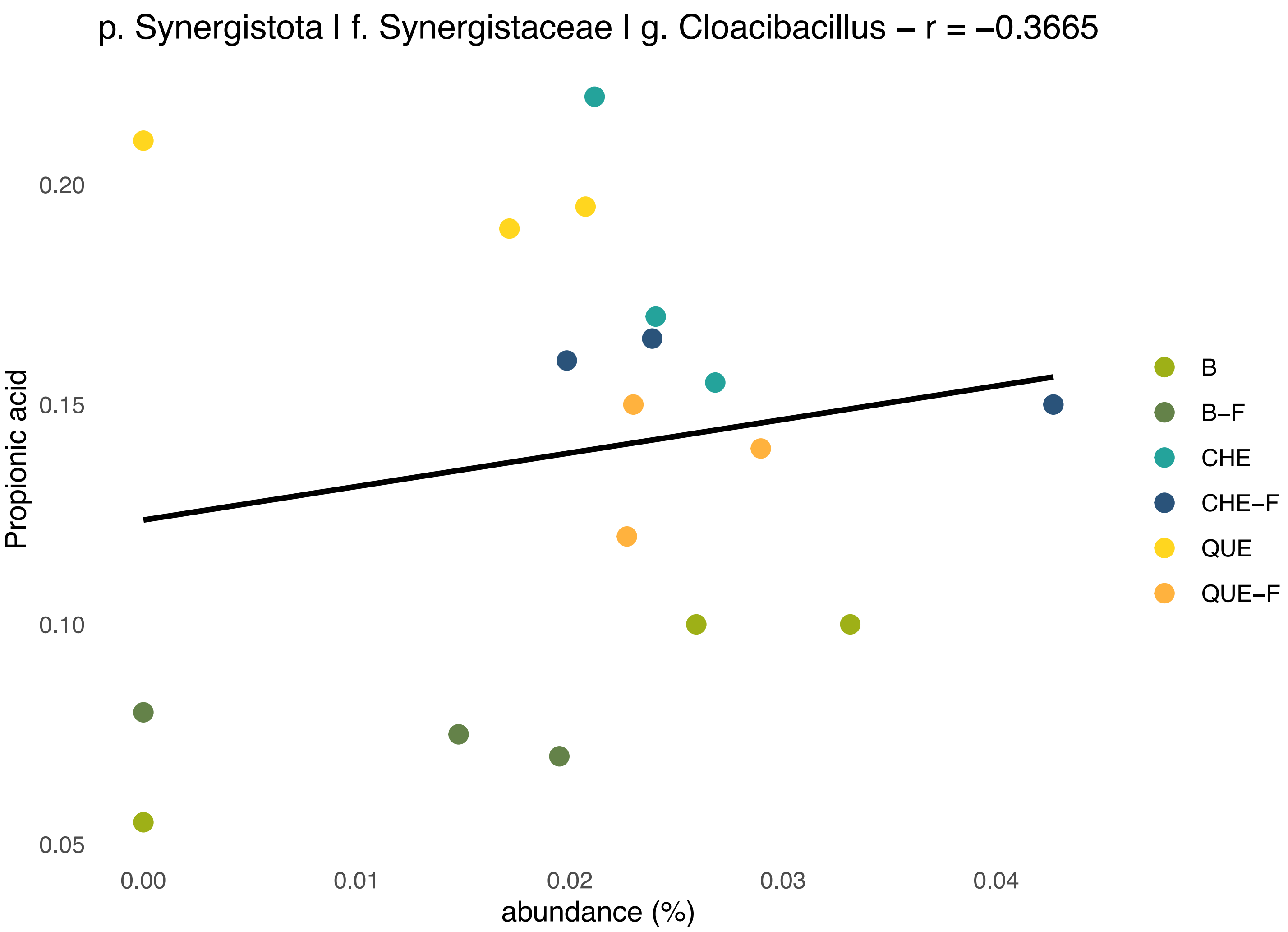
p. Synergistota I f. Synergistaceae I g. Cloacibacillus – r = –0.3665
Propionic acid
0.20
0.15
0.10
0.05
0.00
0.01
0.02
0.03
0.04
abundance (%)
B
B–F
CHE
CHE–F
QUE
QUE–F

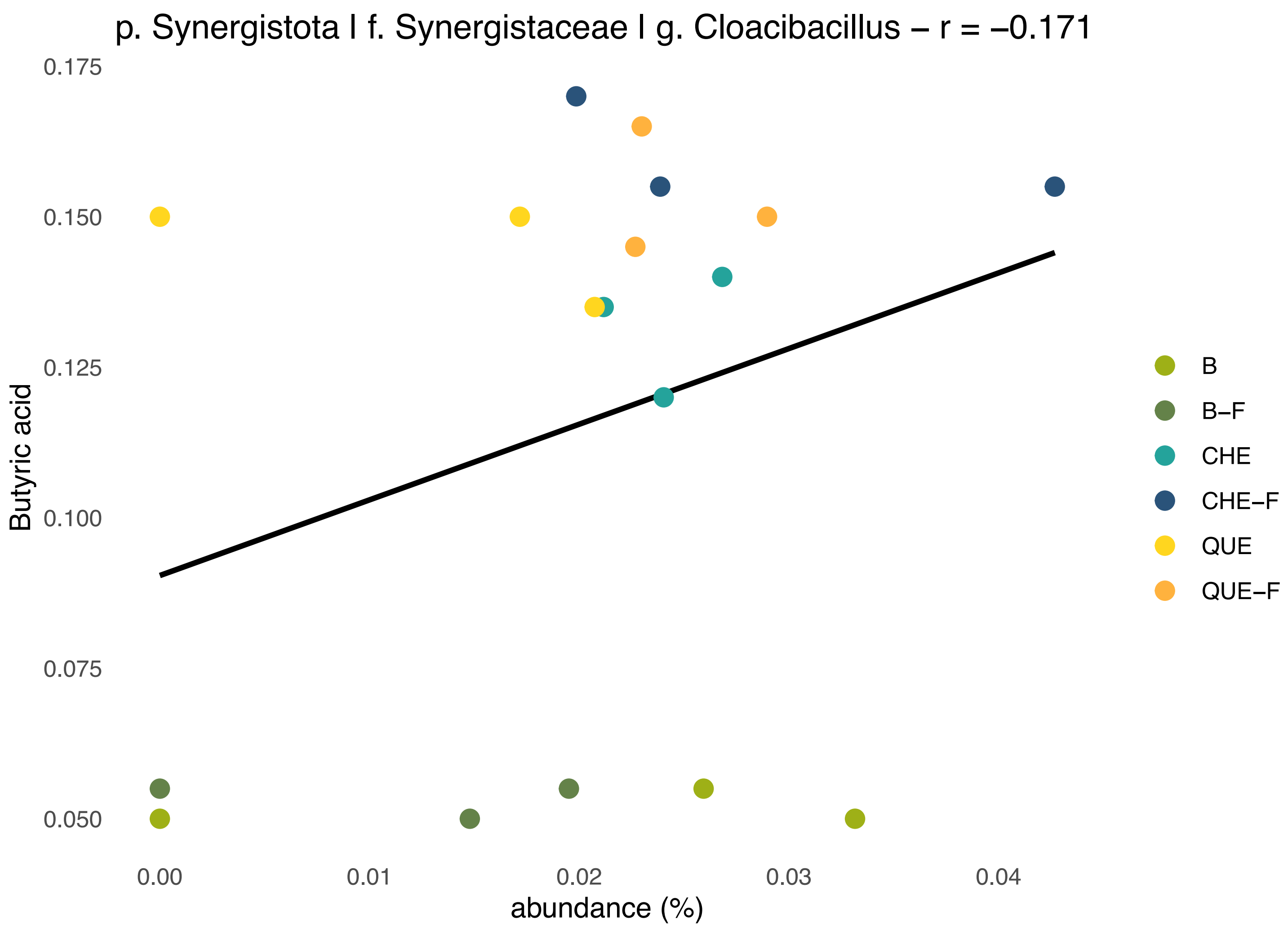

p. Synergistota I f. Synergistaceae I g. Cloacibacillus – r = –0.171
Butyric acid
0.175
0.150
0.125
0.100
0.075
0.050
0.00
0.01
0.02
0.03
0.04
abundance (%)
B
B–F
CHE
CHE–F
QUE
QUE–F

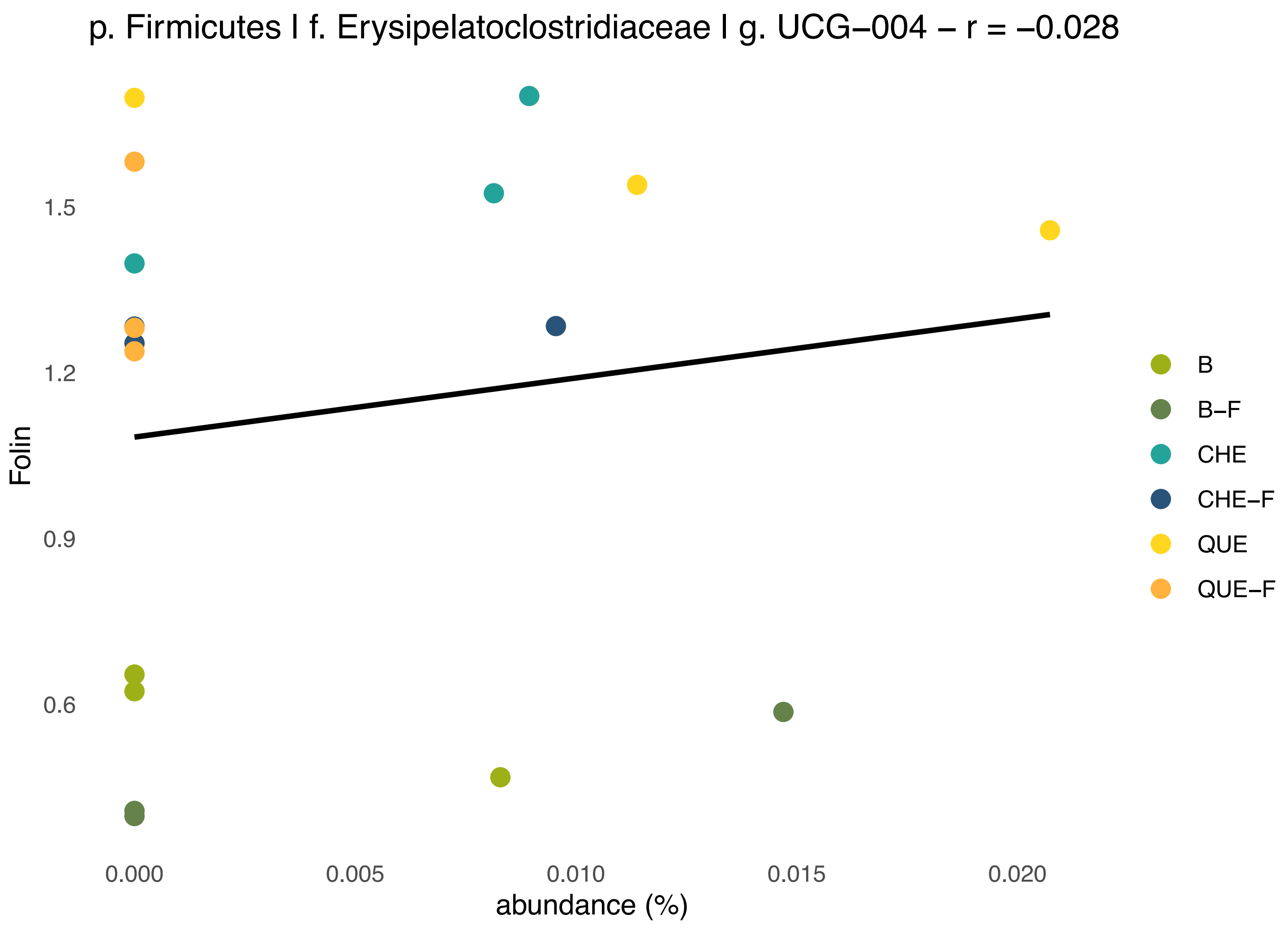
p. Firmicutes I f. Erysipelatoclostridiaceae I g. UCG−004 − r = −0.028
Folin
1.5
1.2
0.9
0.6
0.000
0.005
0.010
0.015
0.020
abundance (%)
B
B−F
CHE
CHE−F
QUE
QUE−F

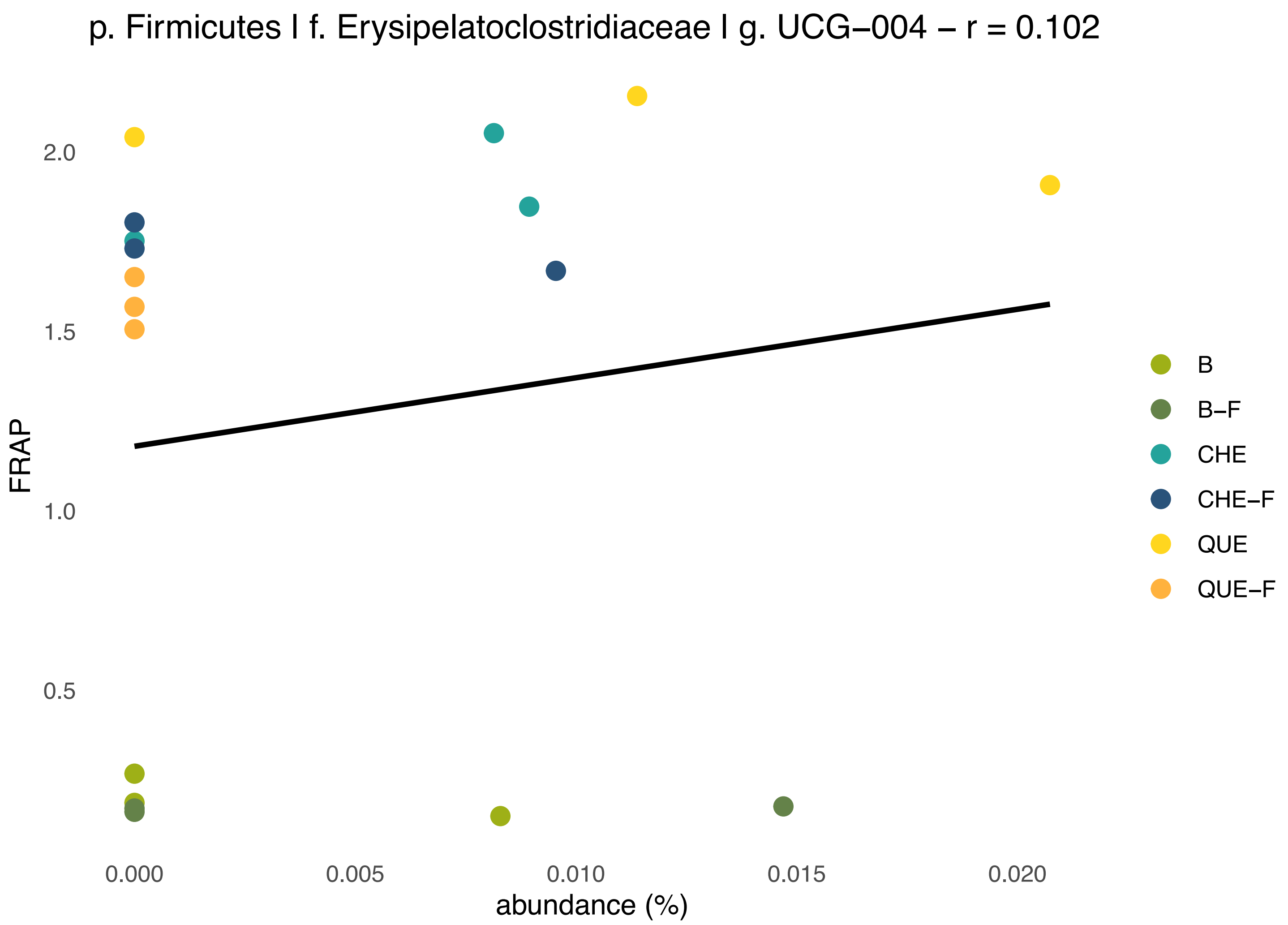
p. Firmicutes I f. Erysipelatoclostridiaceae I g. UCG−004 − r = 0.102
FRAP
2.0
1.5
1.0
0.5
0.000
0.005
0.010
0.015
0.020
abundance (%)
B
B−F
CHE
CHE−F
QUE
QUE−F

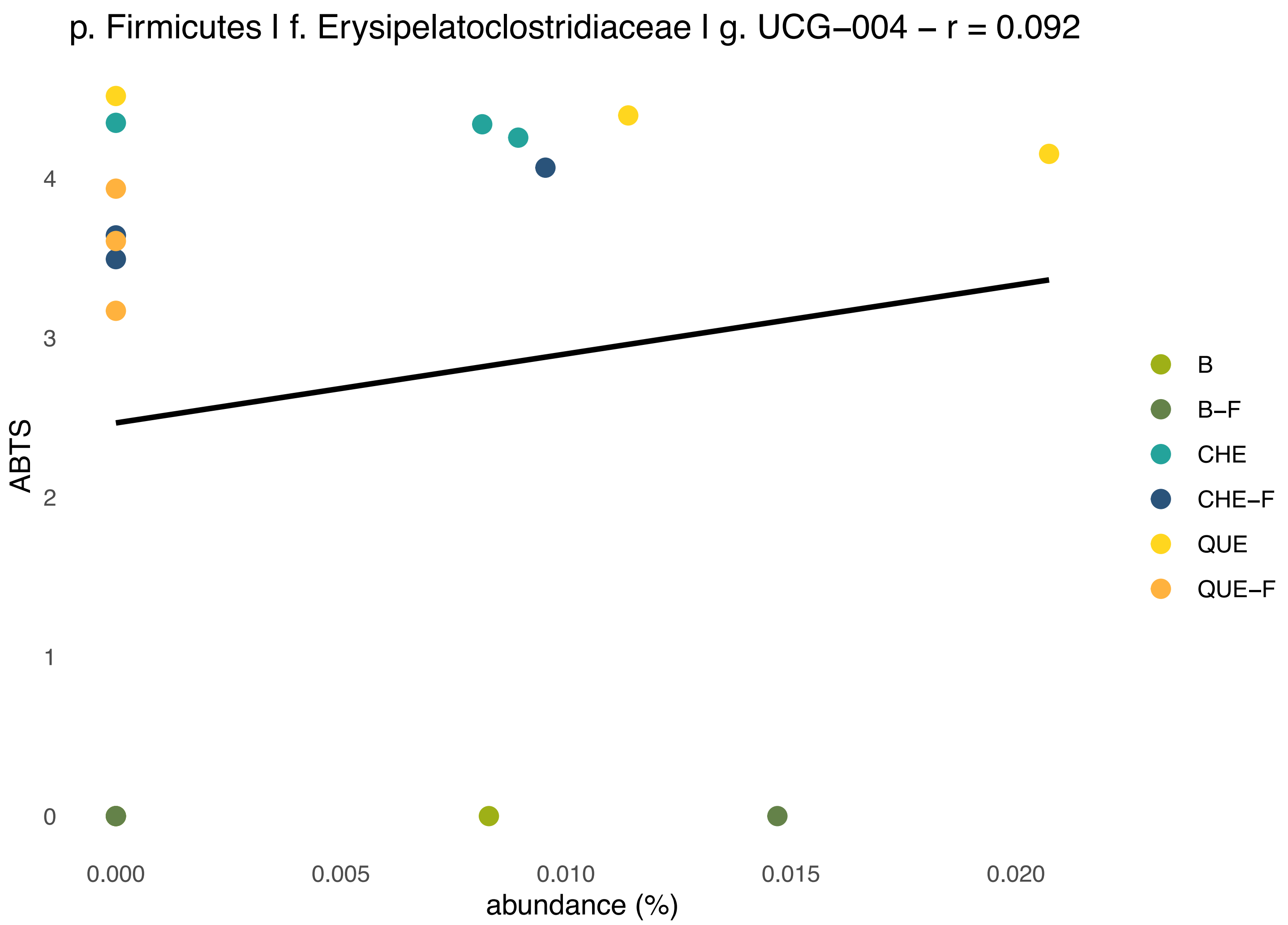

p. Firmicutes I f. Erysipelatoclostridiaceae I g. UCG−004 − r = 0.092
ABTS
0
1
2
3
4
0.000
0.005
0.010
0.015
0.020
abundance (%)
B
B−F
CHE
CHE−F
QUE
QUE−F

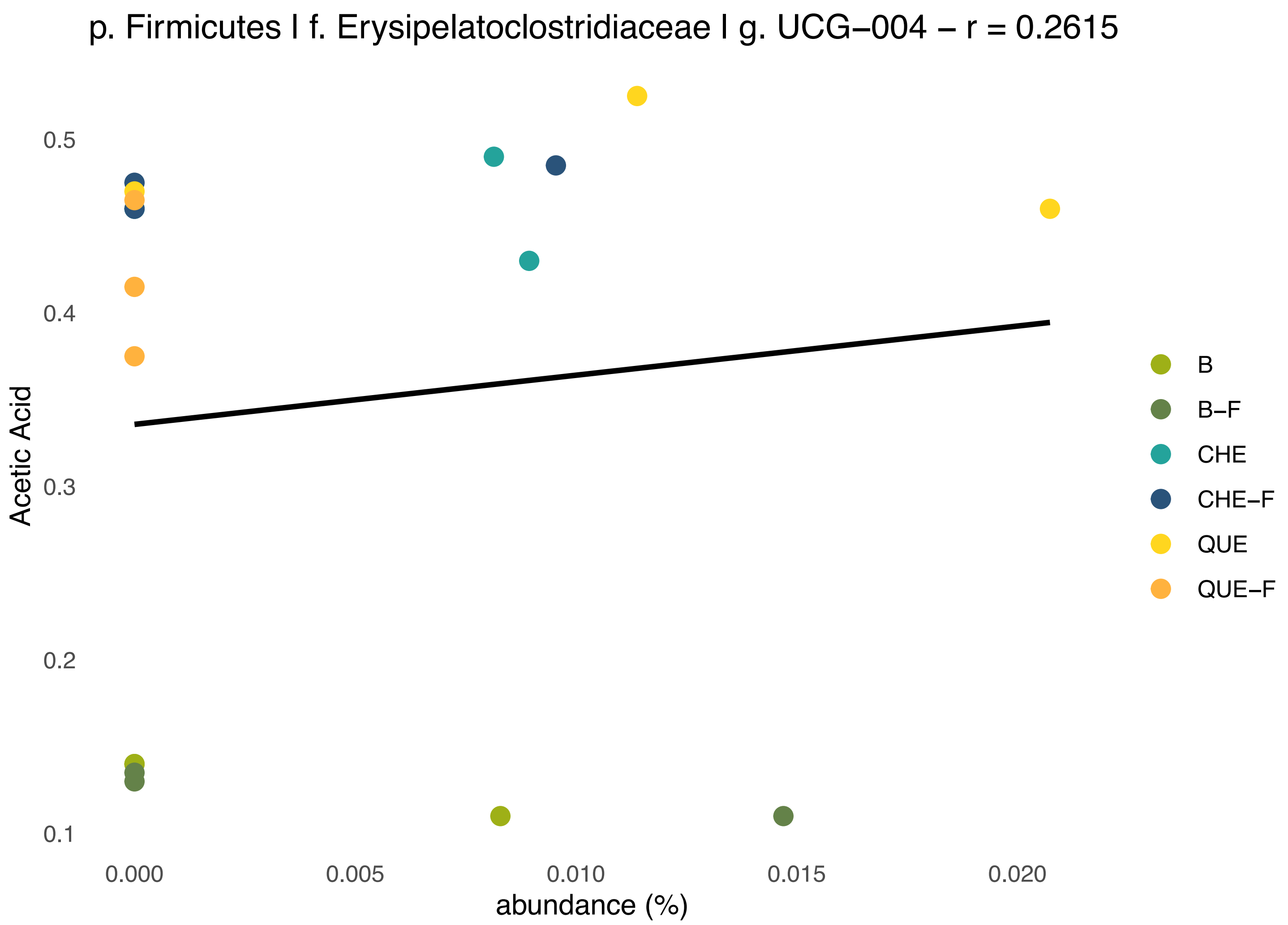

p. Firmicutes I f. Erysipelatoclostridiaceae I g. UCG−004 − r = 0.2615
0.5
0.4
0.3
0.2
0.1
Acetic Acid
0.000
0.005
0.010
0.015
0.020
abundance (%)
B
B−F
CHE
CHE−F
QUE
QUE−F

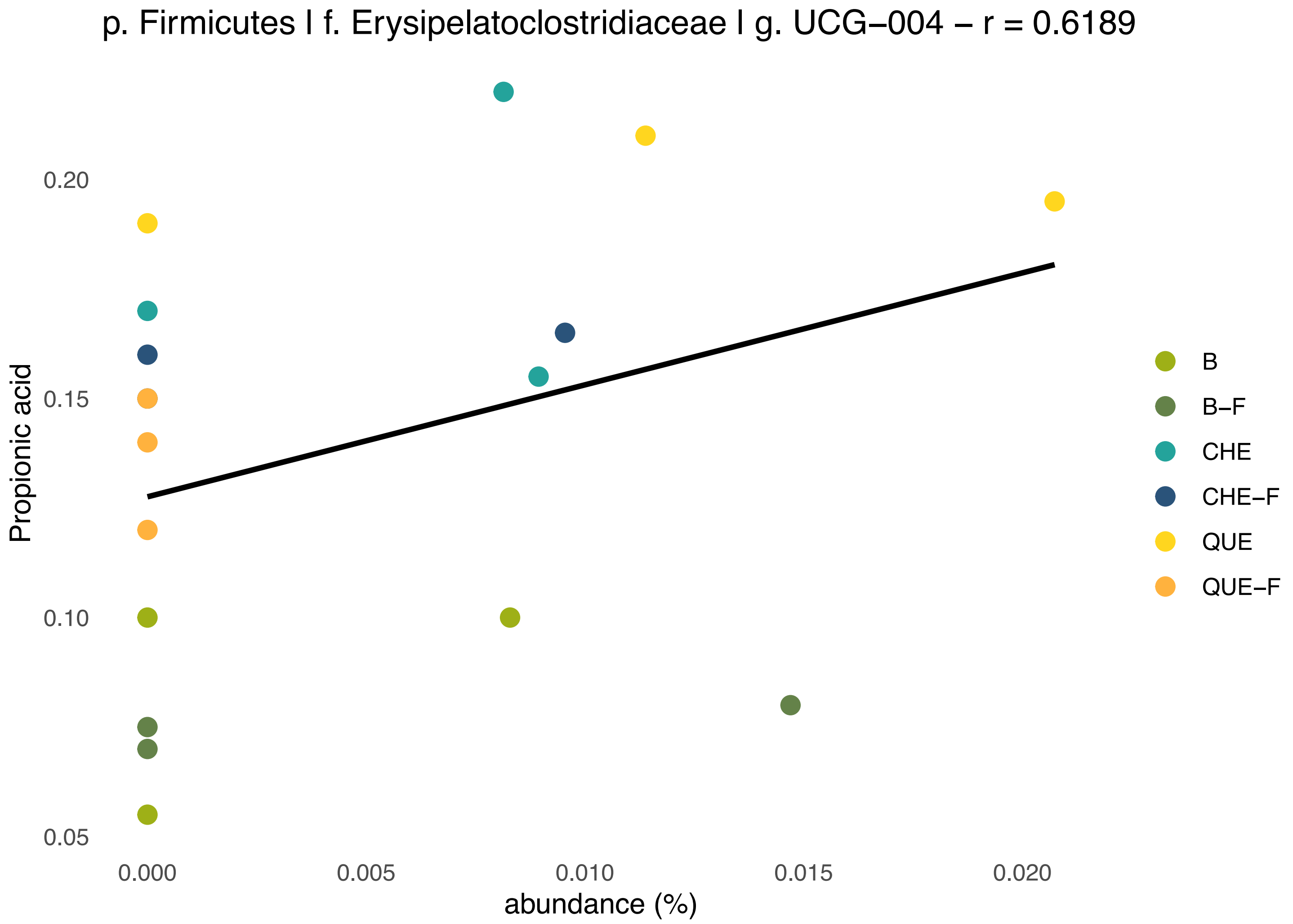

p. Firmicutes I f. Erysipelatoclostridiaceae I g. UCG−004 − r = 0.6189
Propionic acid
0.20
0.15
0.10
0.05
0.000
0.005
0.010
0.015
0.020
abundance (%)
B
B−F
CHE
CHE−F
QUE
QUE−F

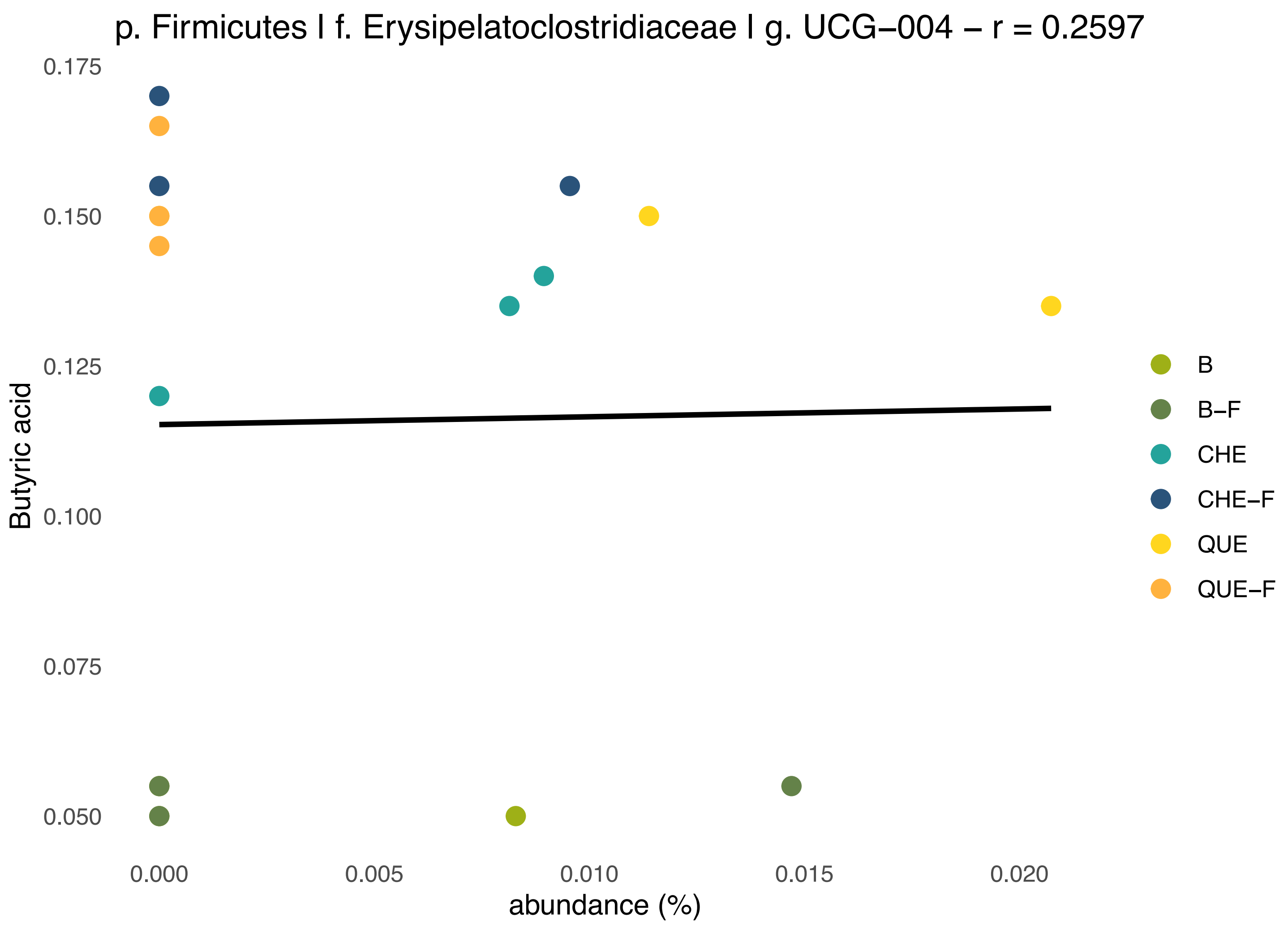

p. Firmicutes I f. Erysipelatoclostridiaceae I g. UCG−004 − r = 0.2597
0.175
0.150
0.125
0.100
0.075
0.050
Butyric acid
0.000
0.005
0.010
0.015
0.020
abundance (%)
B
B−F
CHE
CHE−F
QUE
QUE−F

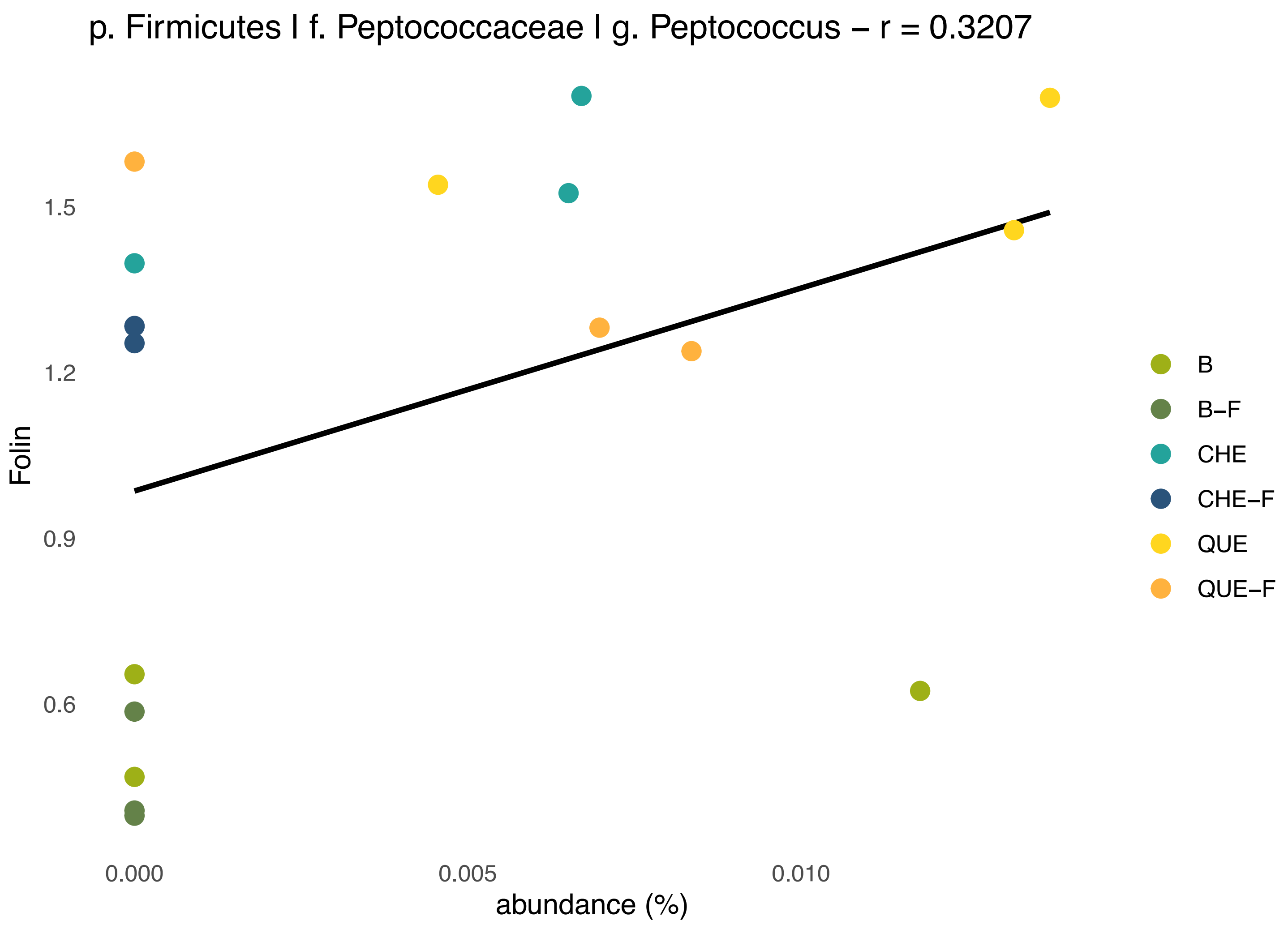
p. Firmicutes I f. Peptococcaceae I g. Peptococcus – r = 0.3207
Folin
1.5
1.2
0.9
0.6
0.000
0.005
0.010
abundance (%)
B
B–F
CHE
CHE–F
QUE
QUE–F

p. Firmicutes I f. Peptococcaceae I g. Peptococcus – r = 0.2235

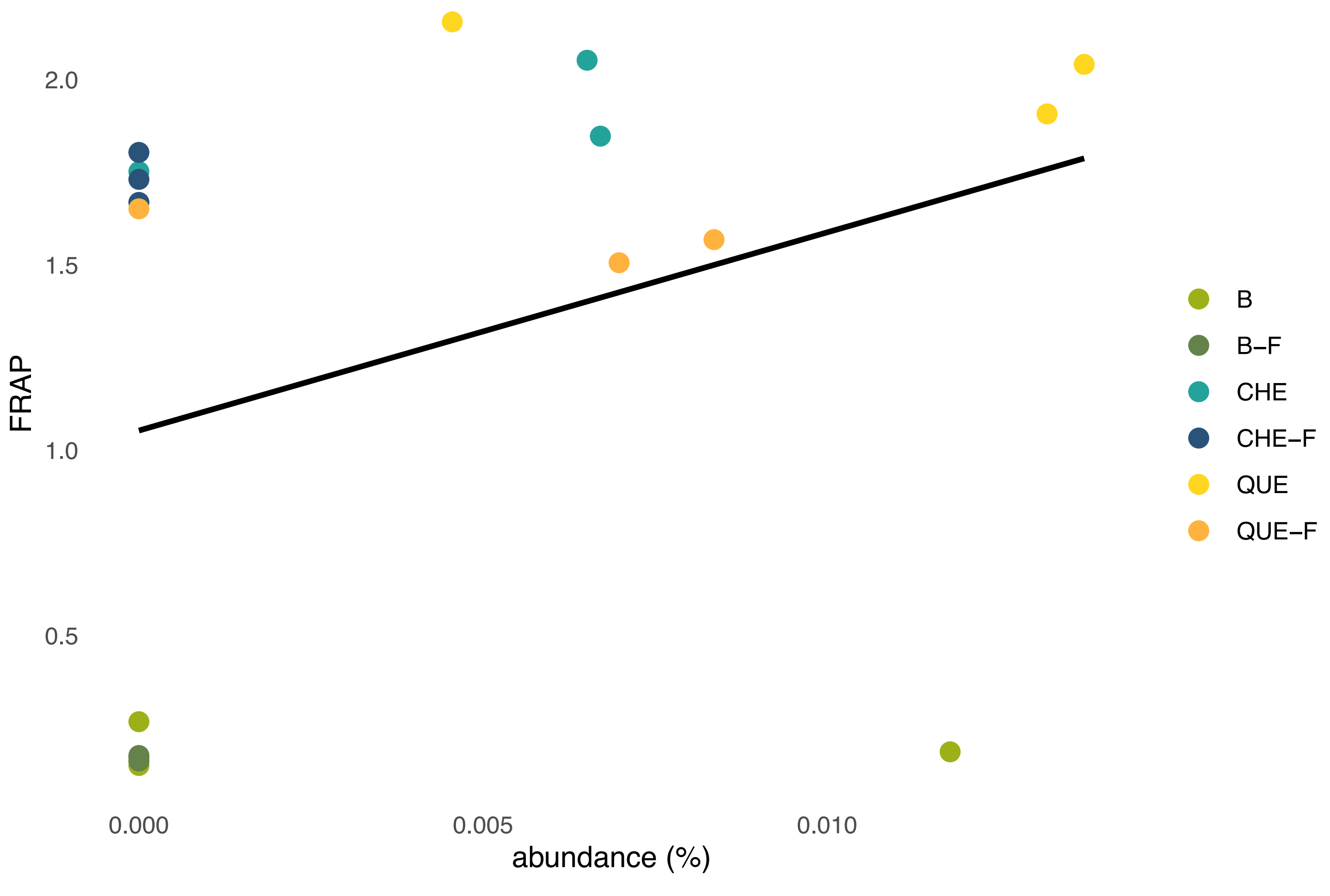

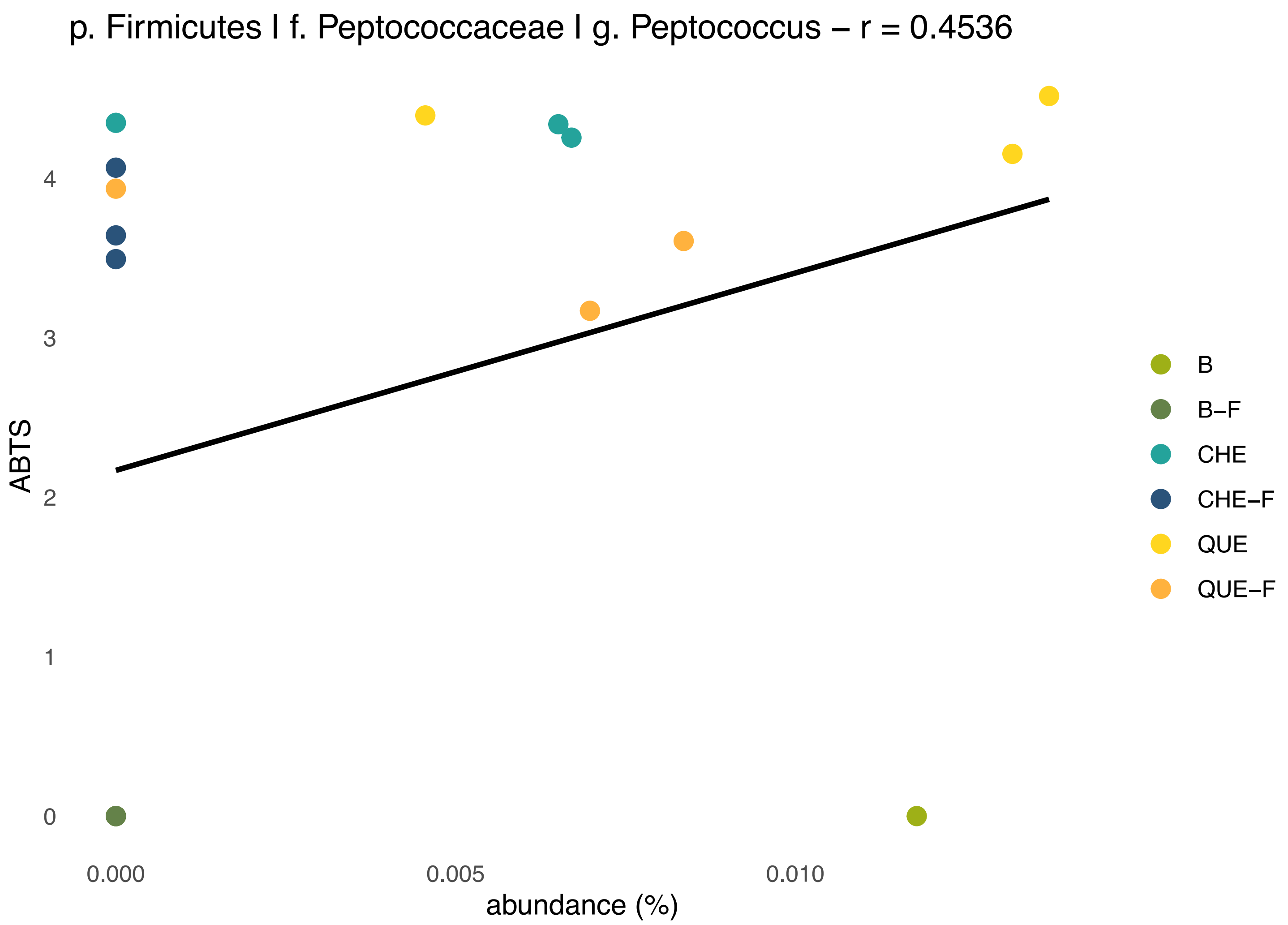

p. Firmicutes I f. Peptococcaceae I g. Peptococcus – r = 0.4536
ABTS
0
1
2
3
4
0.000
0.005
0.010
abundance (%)
B
B–F
CHE
CHE–F
QUE
QUE–F

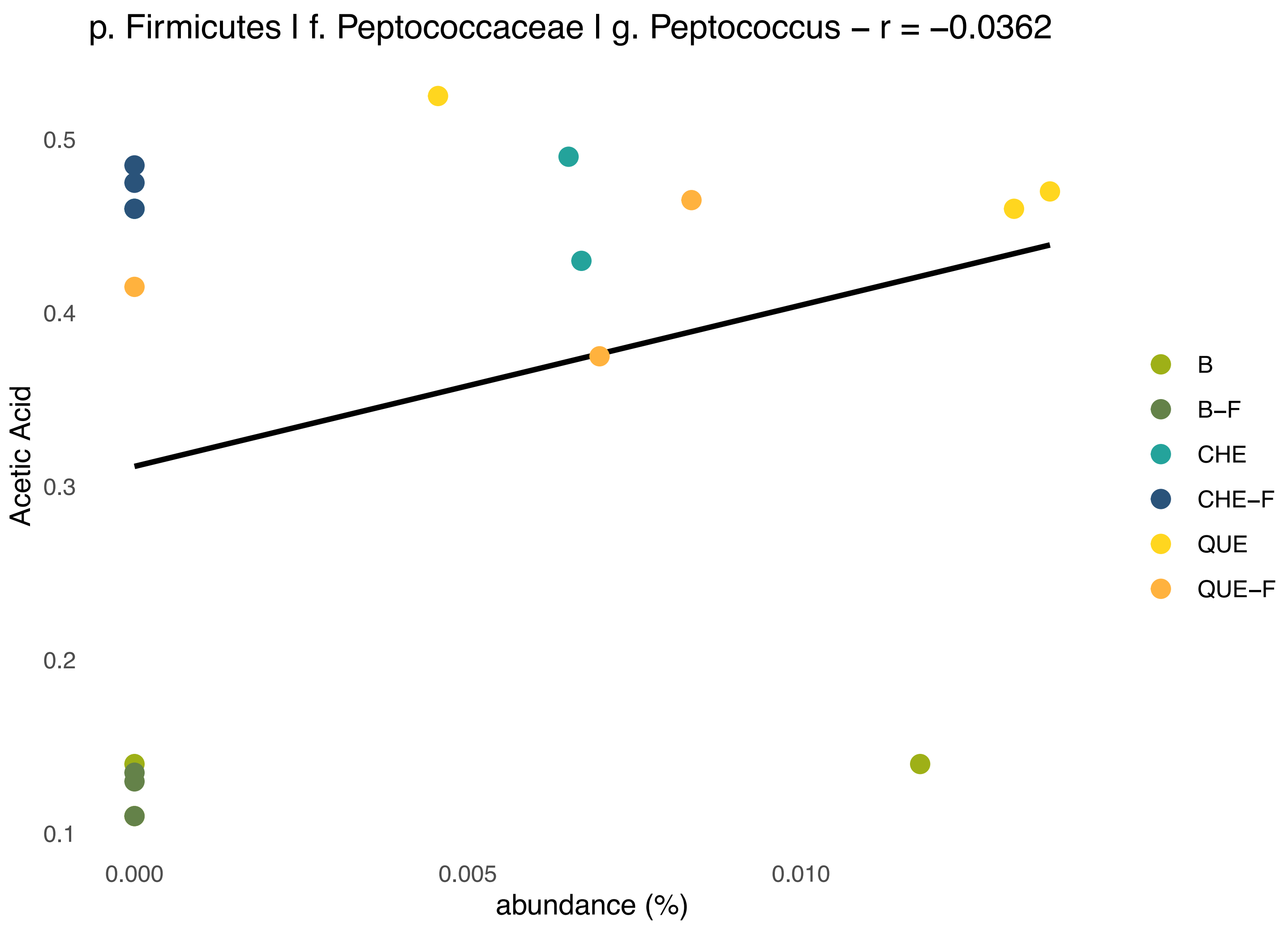

p. Firmicutes I f. Peptococcaceae I g. Peptococcus – r = −0.0362
Acetic Acid
0.1
0.2
0.3
0.4
0.5
0.000
0.005
0.010
abundance (%)
B
B−F
CHE
CHE−F
QUE
QUE−F

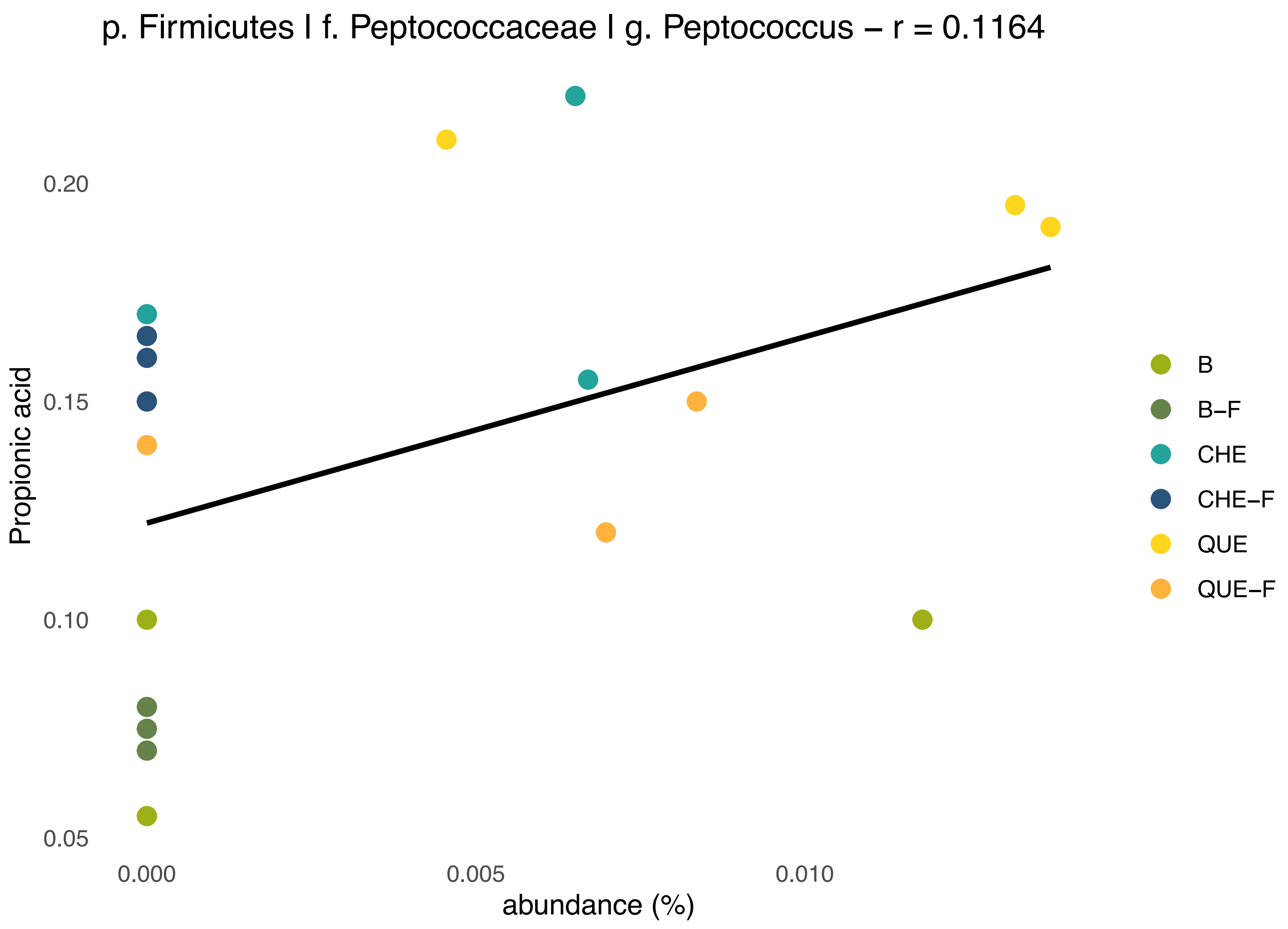

p. Firmicutes I f. Peptococcaceae I g. Peptococcus – r = 0.1164
Propionic acid
0.20
0.15
0.10
0.05
0.000
0.005
0.010
abundance (%)
B
B–F
CHE
CHE–F
QUE
QUE–F

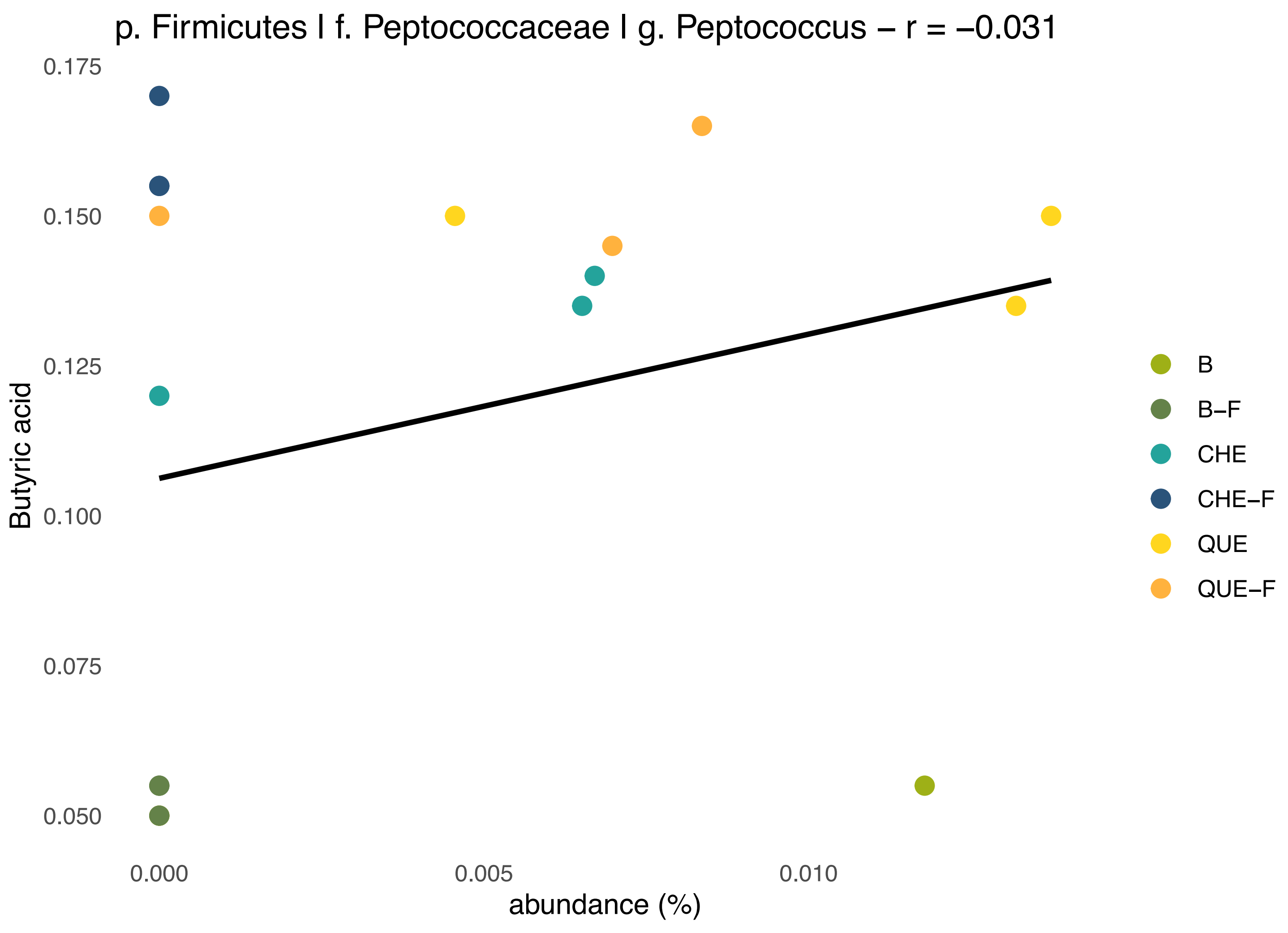

p. Firmicutes I f. Peptococcaceae I g. Peptococcus – r = –0.031
0.175
0.150
0.125
0.100
0.075
0.050
Butyric acid
0.000
0.005
0.010
abundance (%)
B
B–F
CHE
CHE–F
QUE
QUE–F

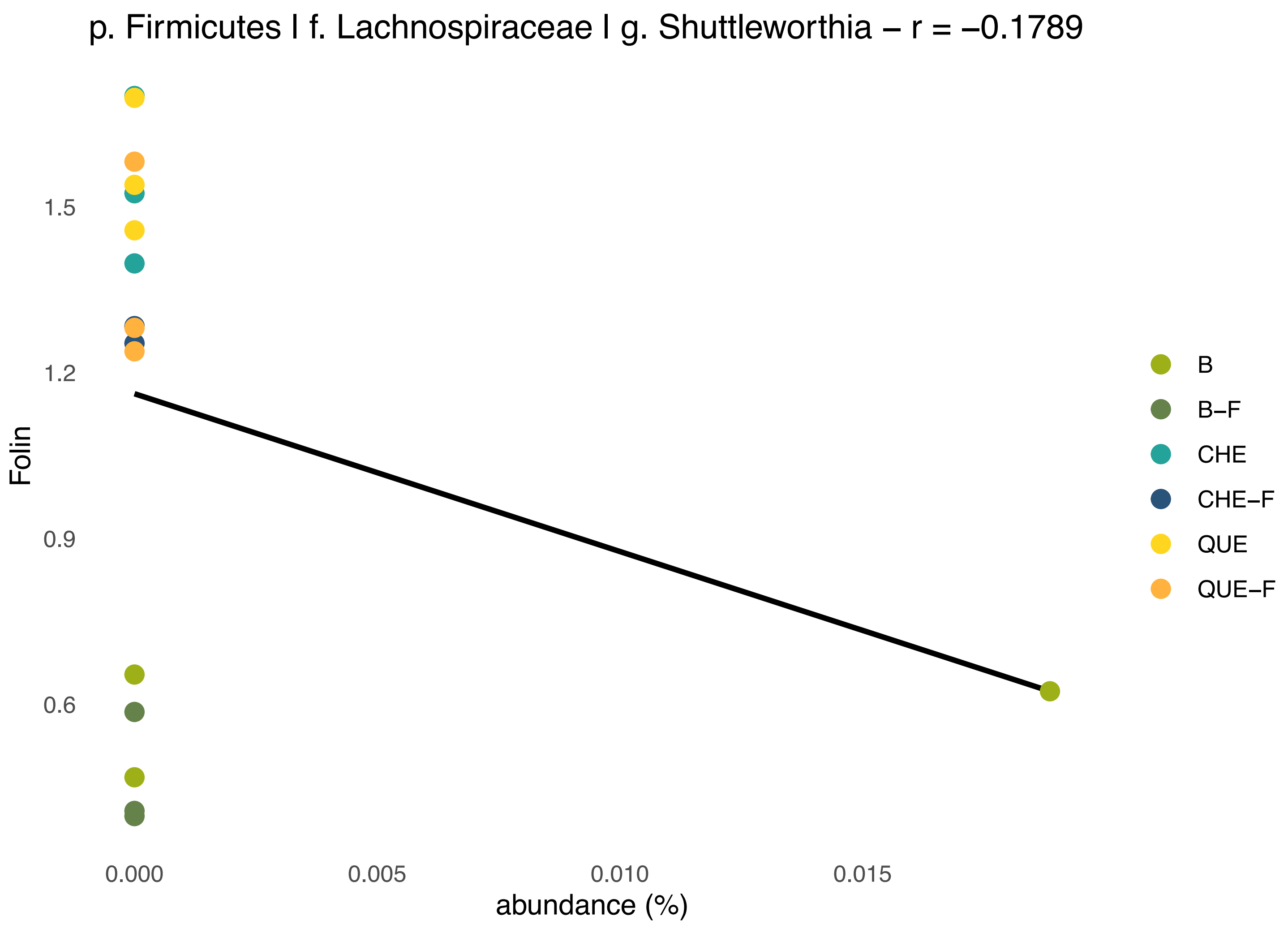
p. Firmicutes I f. Lachnospiraceae I g. Shuttleworthia – r = −0.1789
Folin
1.5
1.2
0.9
0.6
0.000
0.005
0.010
0.015
abundance (%)
B
B−F
CHE
CHE−F
QUE
QUE−F

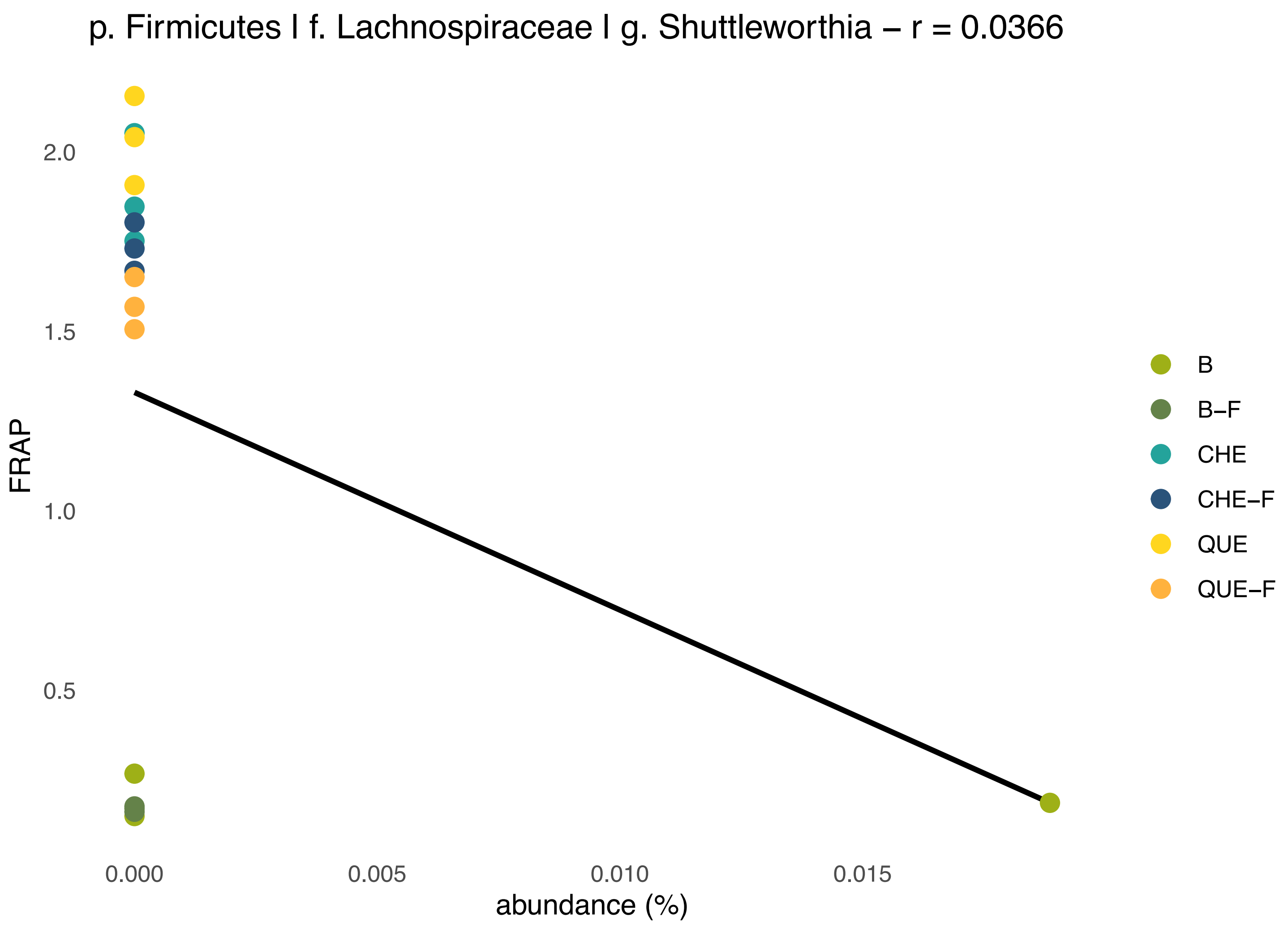
p. Firmicutes I f. Lachnospiraceae I g. Shuttleworthia – r = 0.0366
FRAP
2.0
1.5
1.0
0.5
0.000
0.005
0.010
0.015
abundance (%)
B
B–F
CHE
CHE–F
QUE
QUE–F

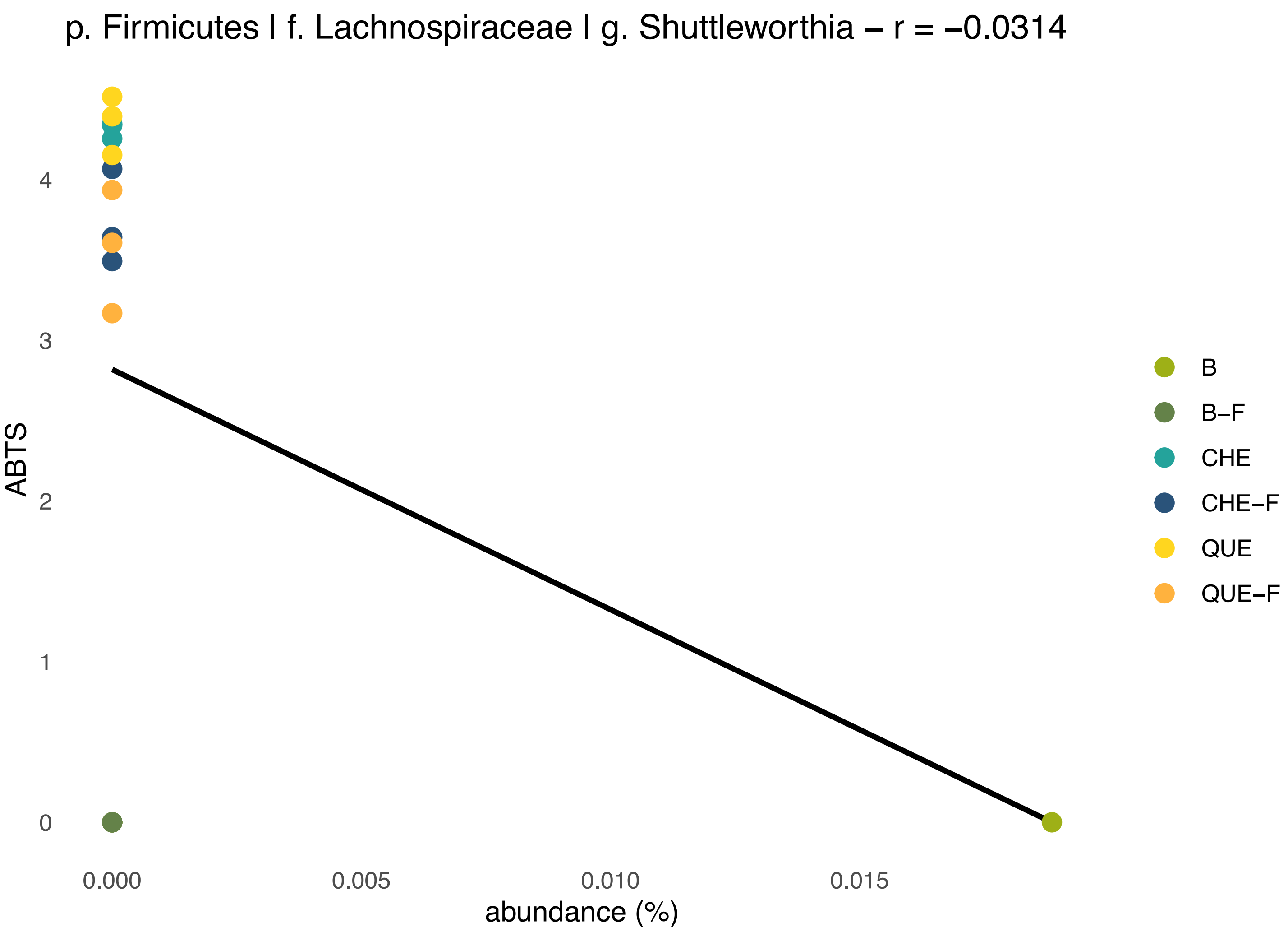
p. Firmicutes l f. Lachnospiraceae l g. Shuttleworthia – r = −0.0314
ABTS
0
1
2
3
4
0.000
0.005
0.010
0.015
abundance (%)
B
B−F
CHE
CHE−F
QUE
QUE−F

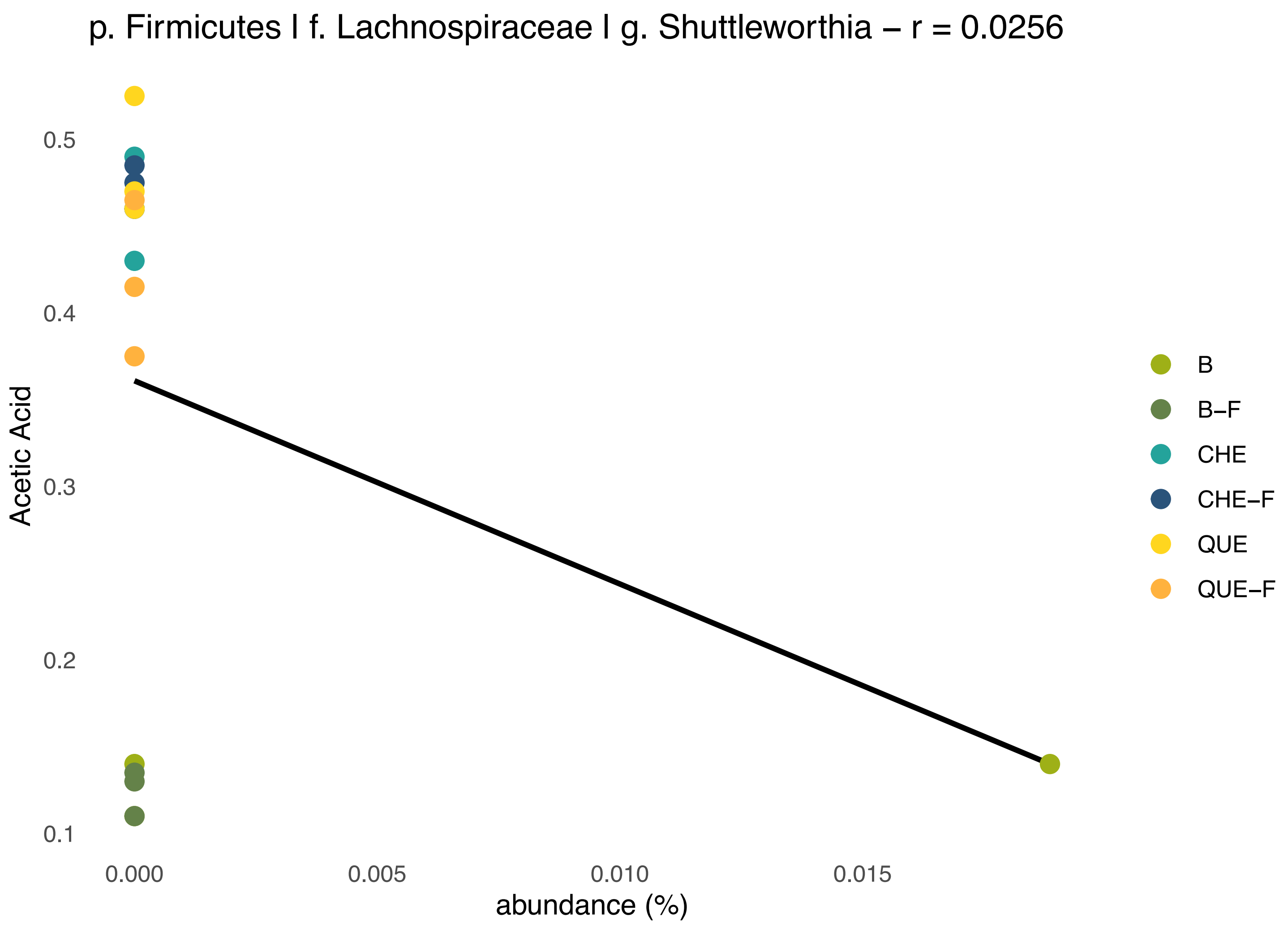

p. Firmicutes | f. Lachnospiraceae | g. Shuttleworthia – r = 0.0256
Acetic Acid
0.5
0.4
0.3
0.2
0.1
0.000
0.005
0.010
0.015
abundance (%)
B
B–F
CHE
CHE–F
QUE
QUE–F

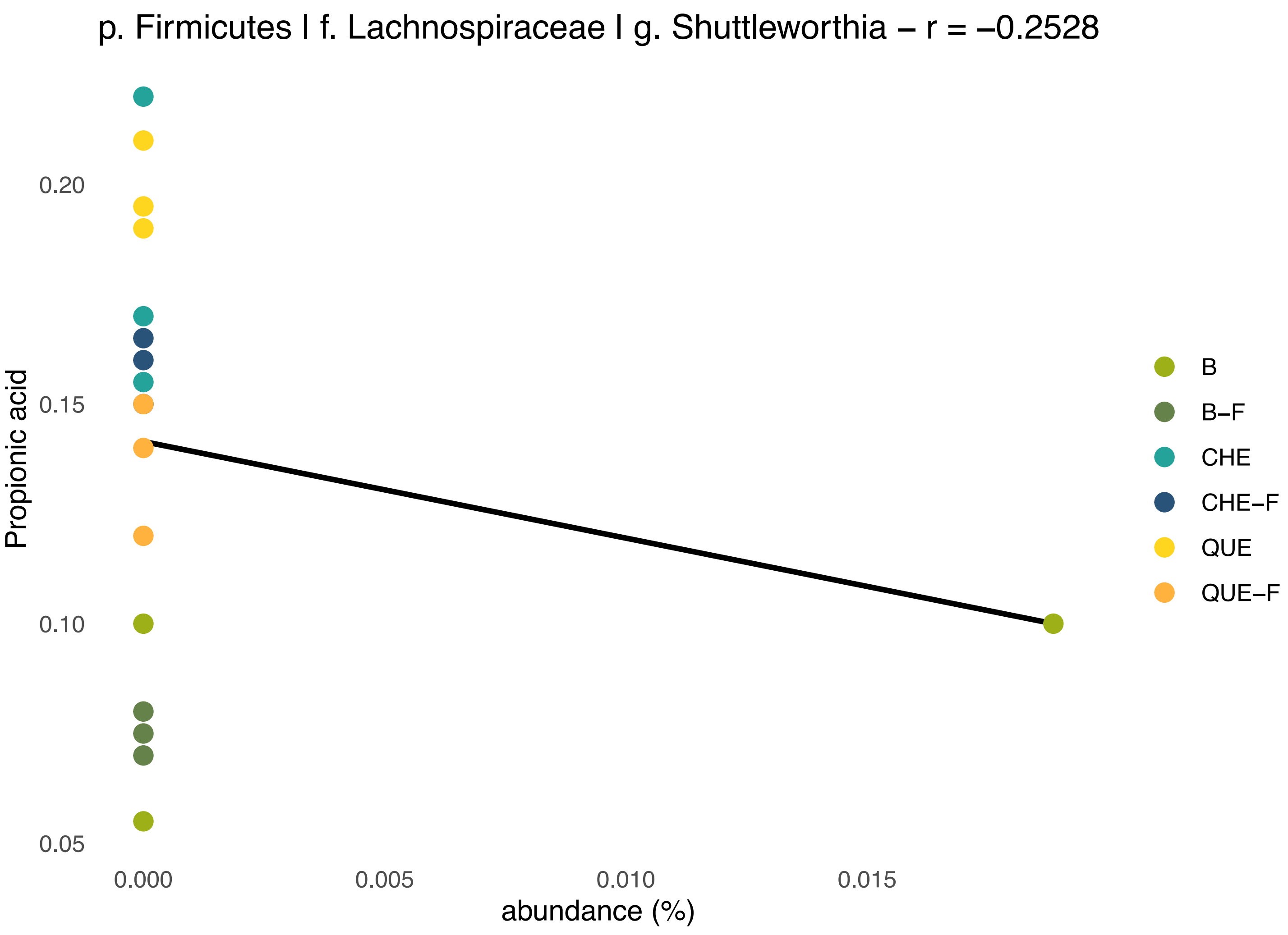

p. Firmicutes I f. Lachnospiraceae I g. Shuttleworthia – r = –0.2528
Propionic acid
0.20
0.15
0.10
0.05
0.000
0.005
0.010
0.015
abundance (%)
B
B–F
CHE
CHE–F
QUE
QUE–F

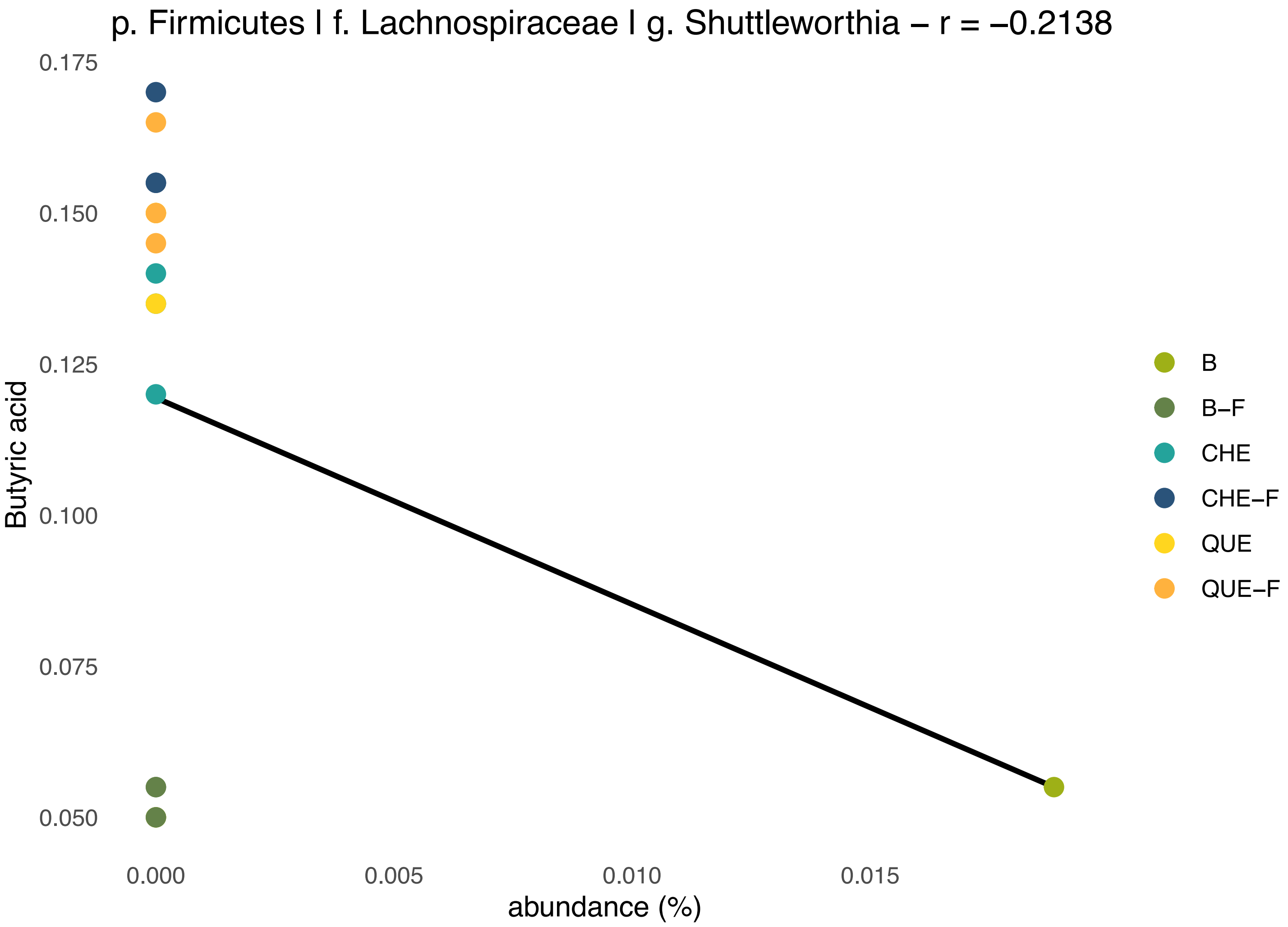
p. Firmicutes I f. Lachnospiraceae I g. Shuttleworthia – r = –0.2138
0.175
0.150
0.125
0.100
0.075
0.050
Butyric acid
0.000
0.005
0.010
0.015
abundance (%)
B
B–F
CHE
CHE–F
QUE
QUE–F

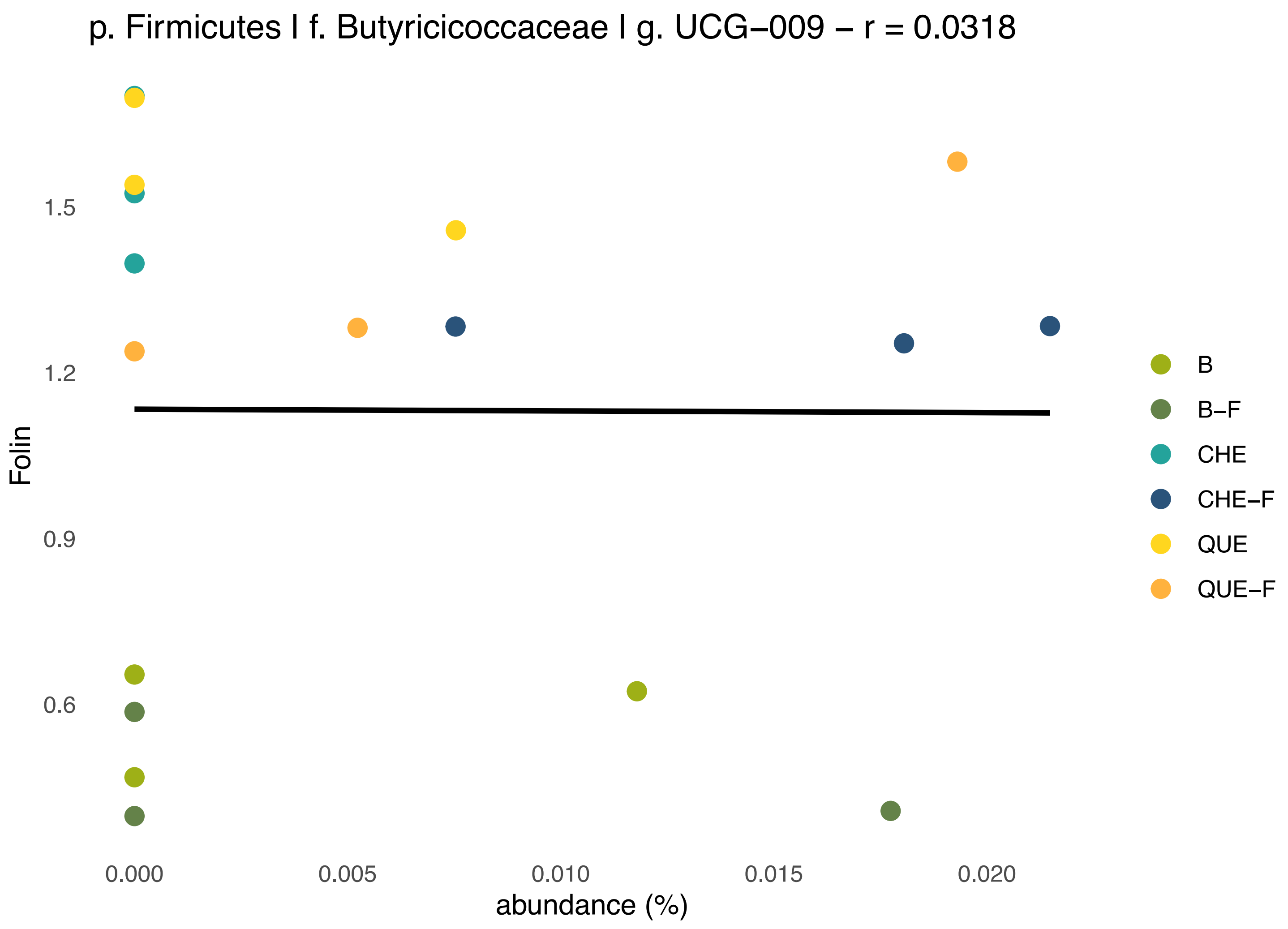

p. Firmicutes I f. Butyricicoccaceae I g. UCG−009 − r = 0.0318
1.5
1.2
0.9
0.6
Folin
0.000
0.005
0.010
0.015
0.020
abundance (%)
B
B−F
CHE
CHE−F
QUE
QUE−F

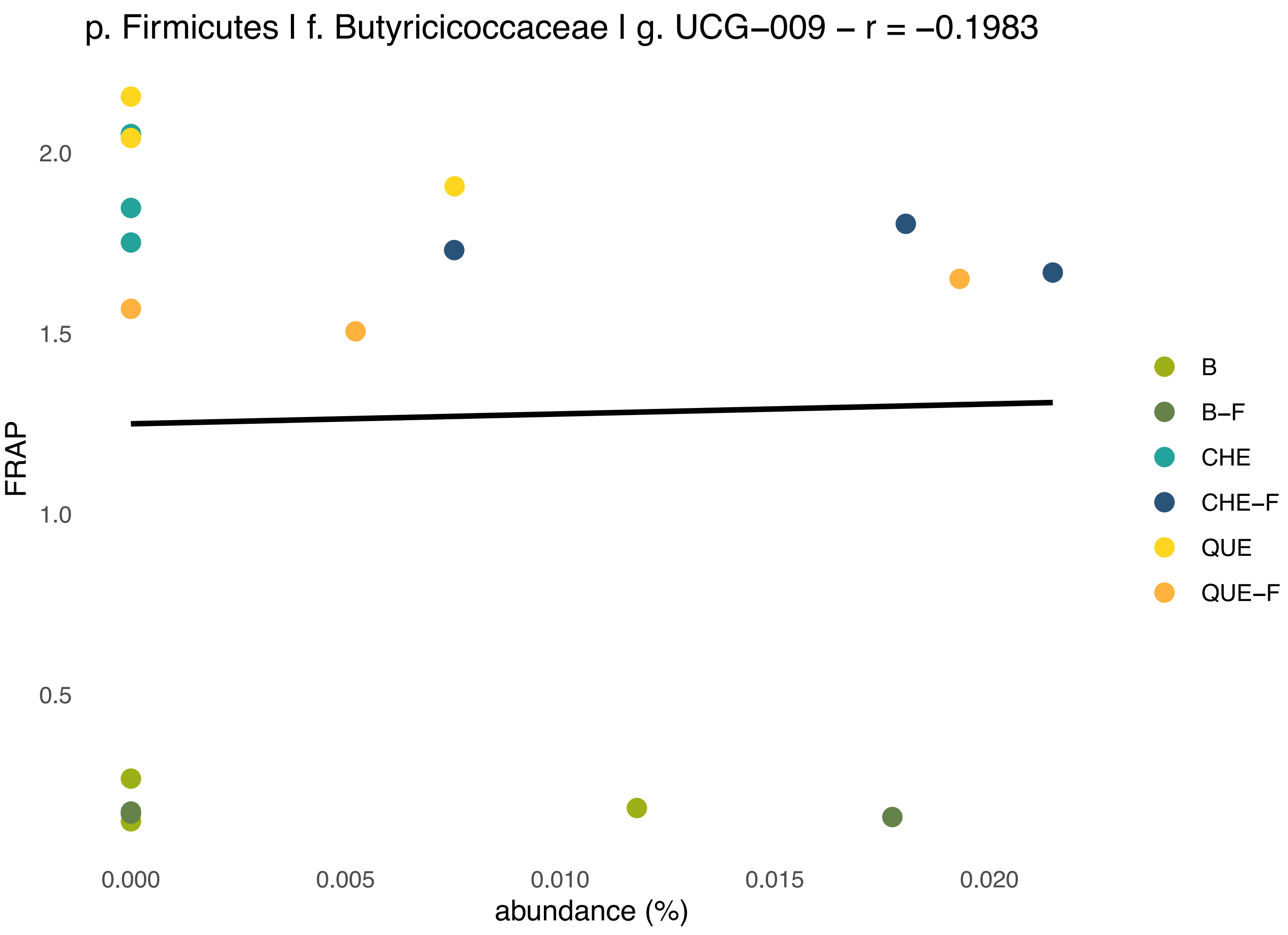

p. Firmicutes I f. Butyricicoccaceae I g. UCG−009 − r = −0.1983
FRAP
2.0
1.5
1.0
0.5
0.000
0.005
0.010
0.015
0.020
abundance (%)
B
B−F
CHE
CHE−F
QUE
QUE−F

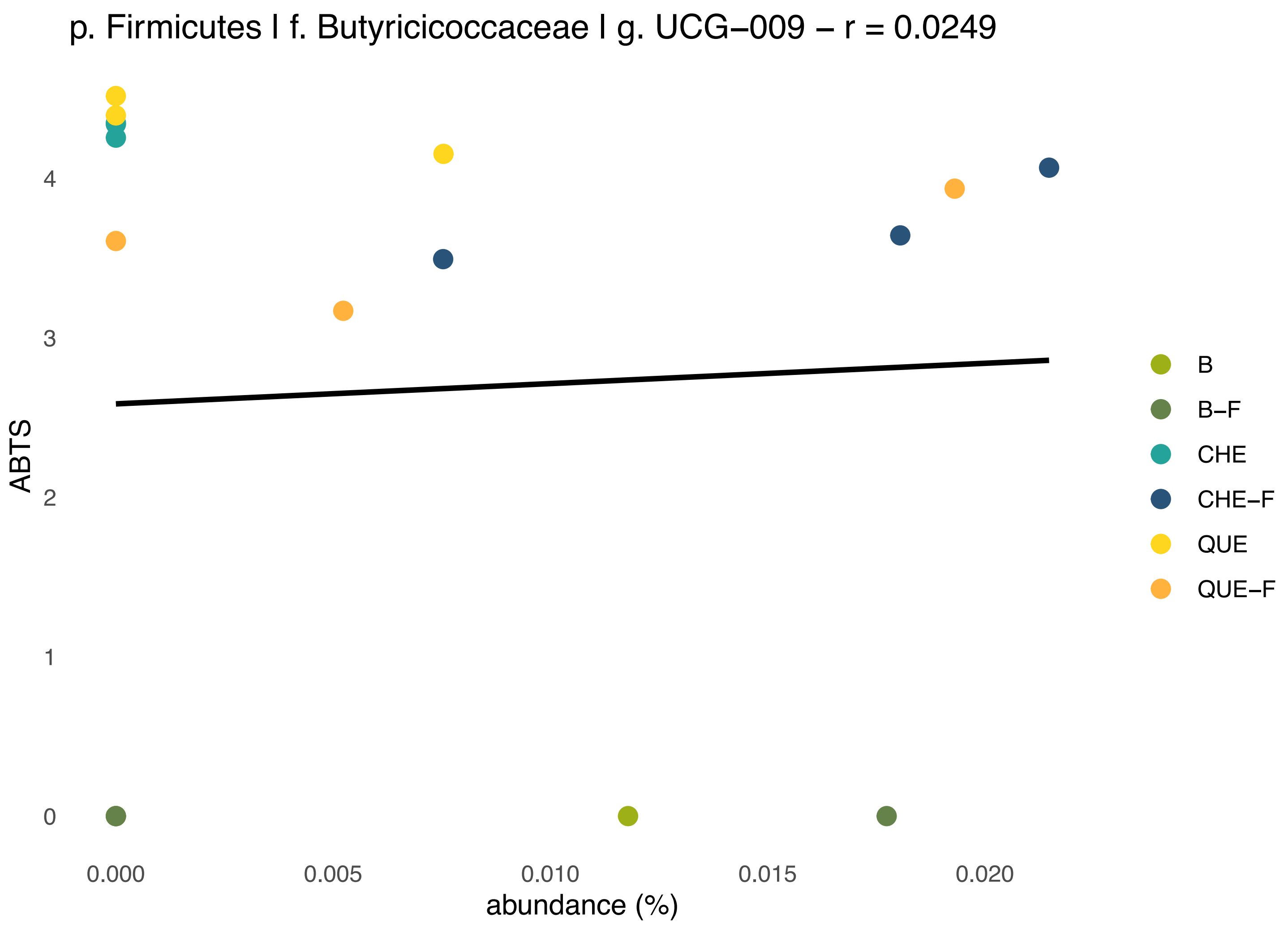

p. Firmicutes l f. Butyricicoccaceae l g. UCG−009 − r = 0.0249
ABTS
0
1
2
3
4
0.000
0.005
0.010
0.015
0.020
abundance (%)
B
B−F
CHE
CHE−F
QUE
QUE−F

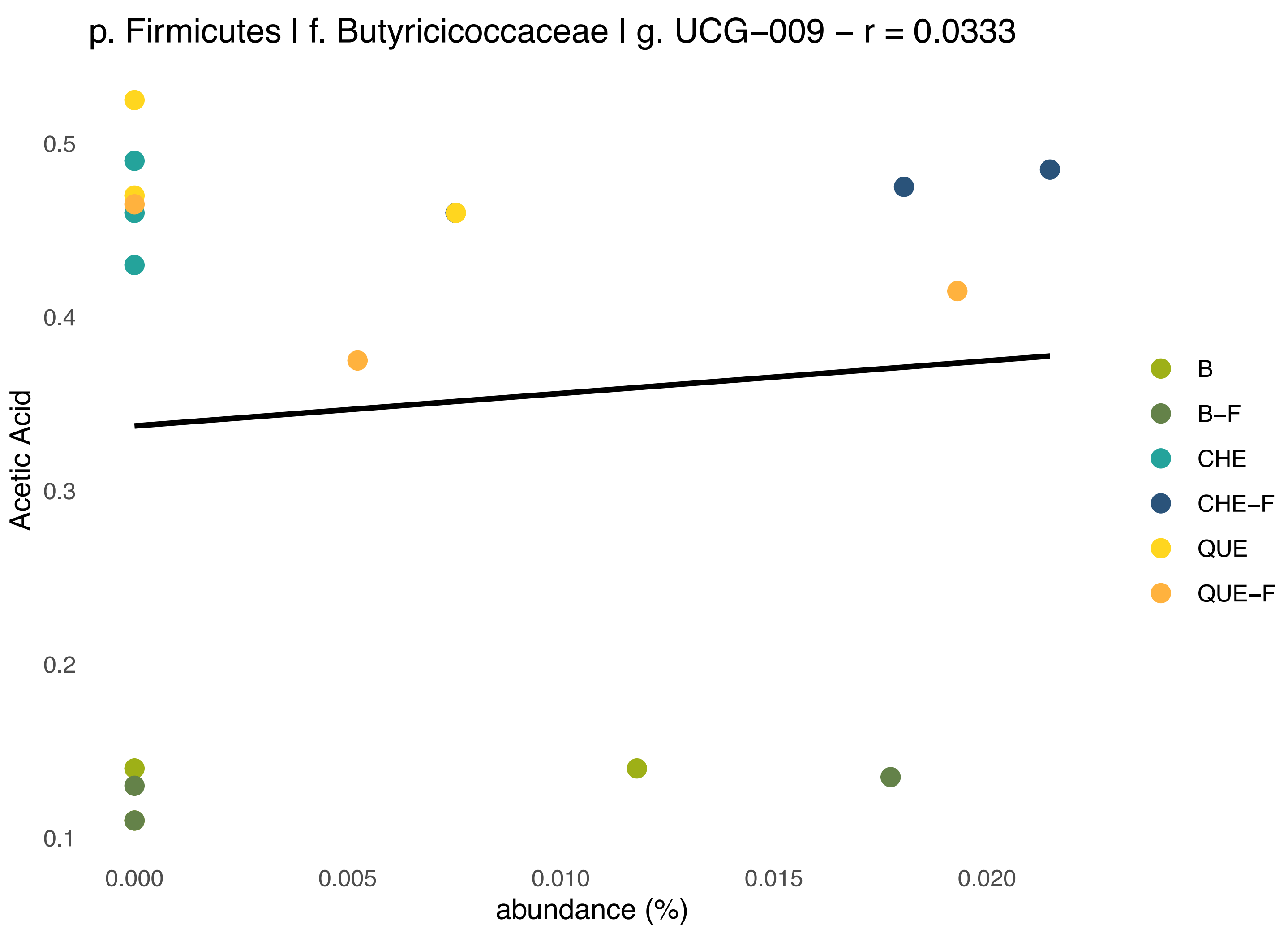
p. Firmicutes I f. Butyricicoccaceae I g. UCG−009 − r = 0.0333
Acetic Acid
0.5
0.4
0.3
0.2
0.1
0.000
0.005
0.010
0.015
0.020
abundance (%)
B
B−F
CHE
CHE−F
QUE
QUE−F

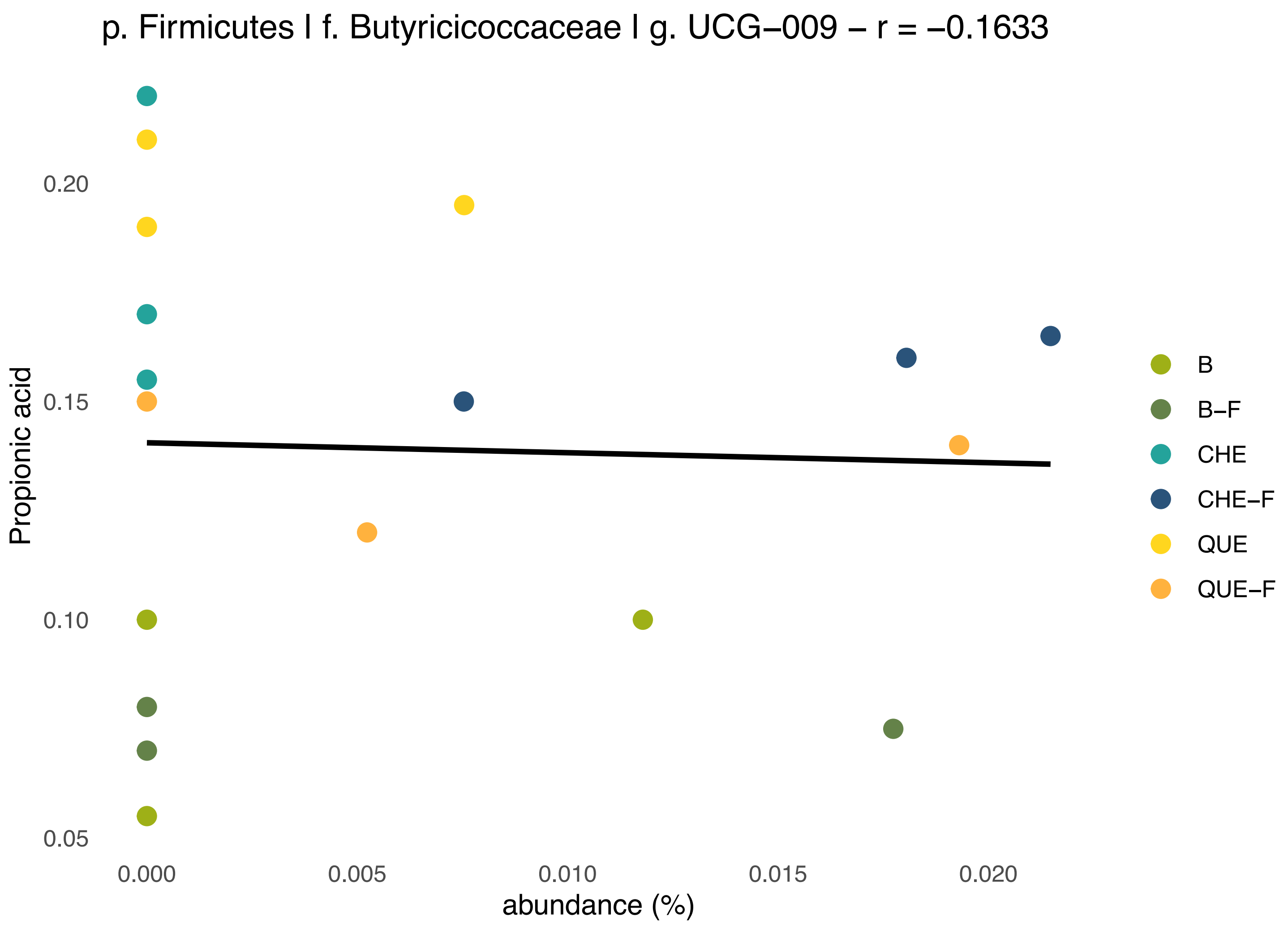
p. Firmicutes I f. Butyricicoccaceae I g. UCG−009 − r = −0.1633
Propionic acid
0.20
0.15
0.10
0.05
0.000
0.005
0.010
0.015
0.020
abundance (%)
B
B−F
CHE
CHE−F
QUE
QUE−F

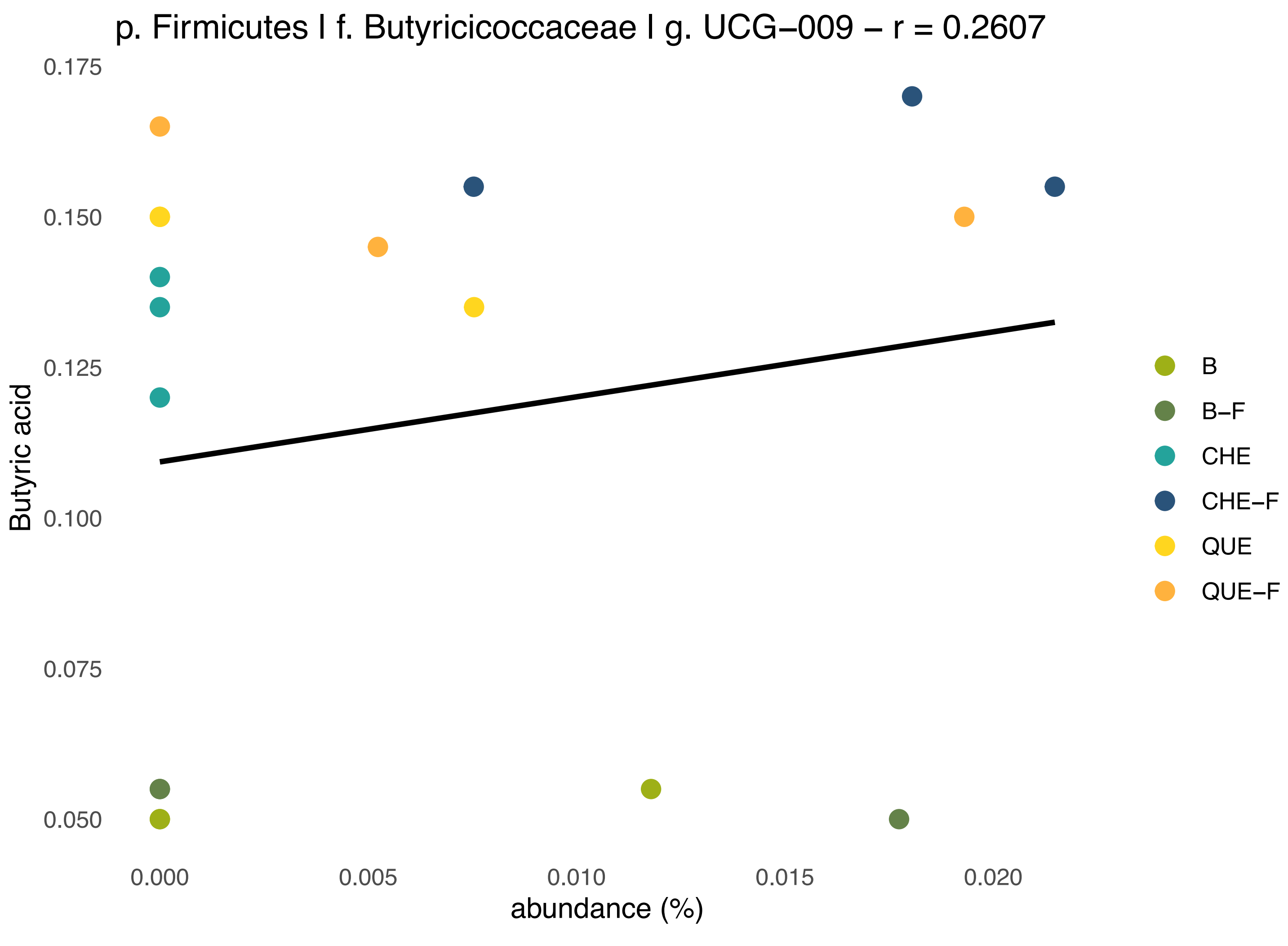

p. Firmicutes I f. Butyricicoccaceae I g. UCG−009 − r = 0.2607
0.175
0.150
0.125
0.100
0.075
0.050
Butyric acid
0.000
0.005
0.010
0.015
0.020
abundance (%)
B
B−F
CHE
CHE−F
QUE
QUE−F

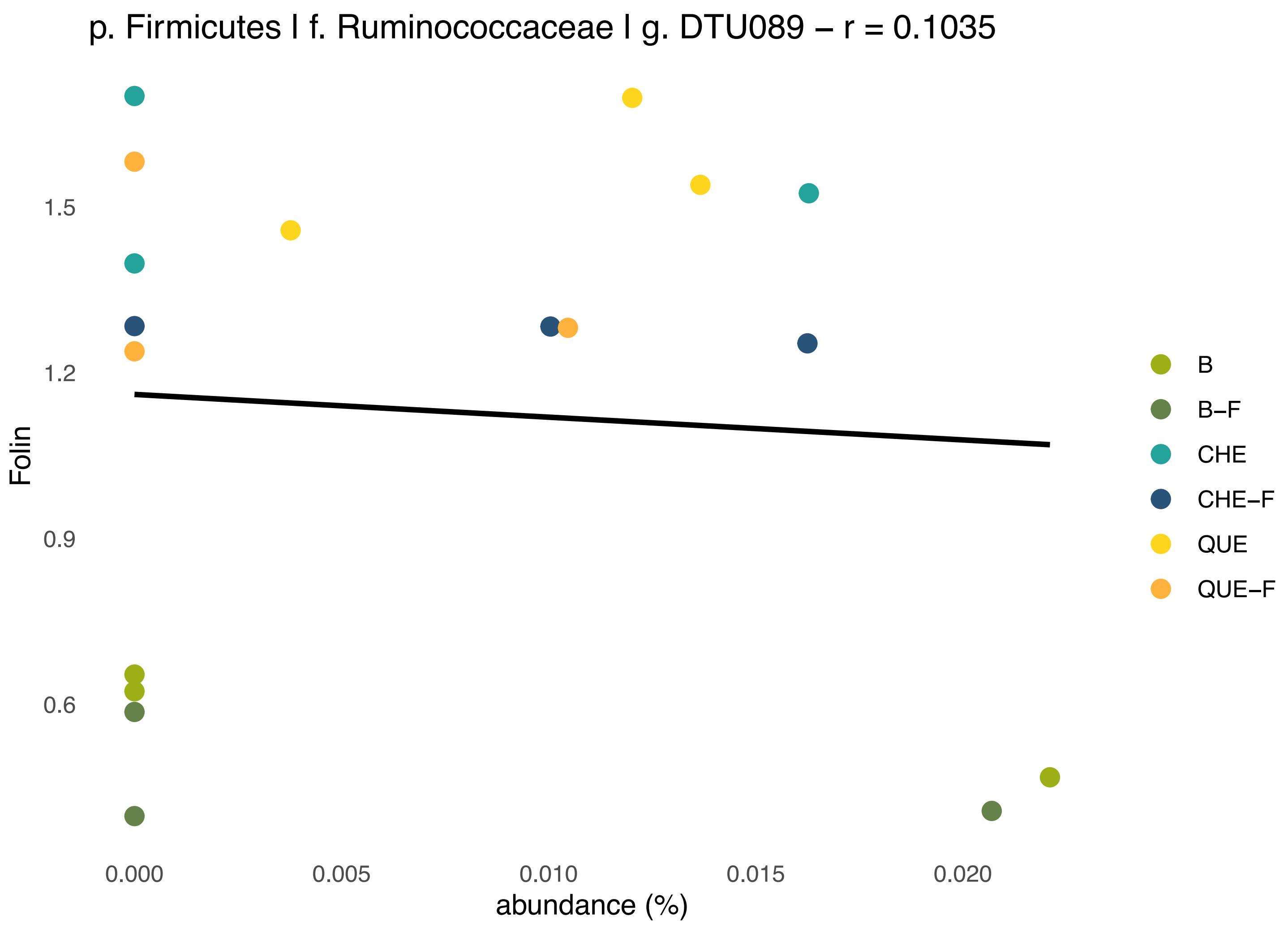
p. Firmicutes I f. Ruminococcaceae I g. DTU089 – r = 0.1035
1.5
1.2
0.9
0.6
Folin
0.000
0.005
0.010
0.015
0.020
abundance (%)
B
B–F
CHE
CHE–F
QUE
QUE–F

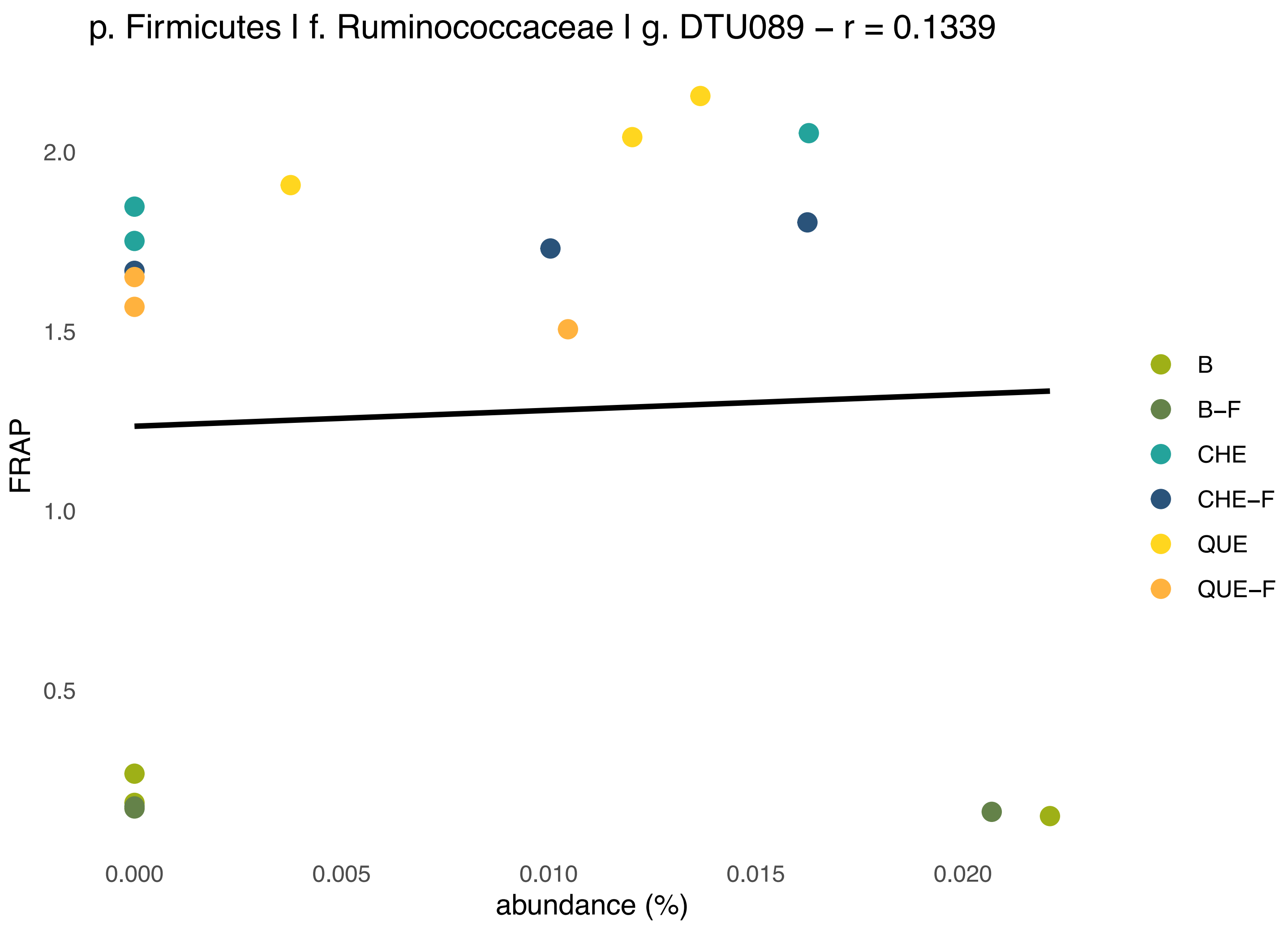

p. Firmicutes I f. Ruminococcaceae I g. DTU089 – r = 0.1339
FRAP
2.0
1.5
1.0
0.5
0.000
0.005
0.010
0.015
0.020
abundance (%)
B
B–F
CHE
CHE–F
QUE
QUE–F

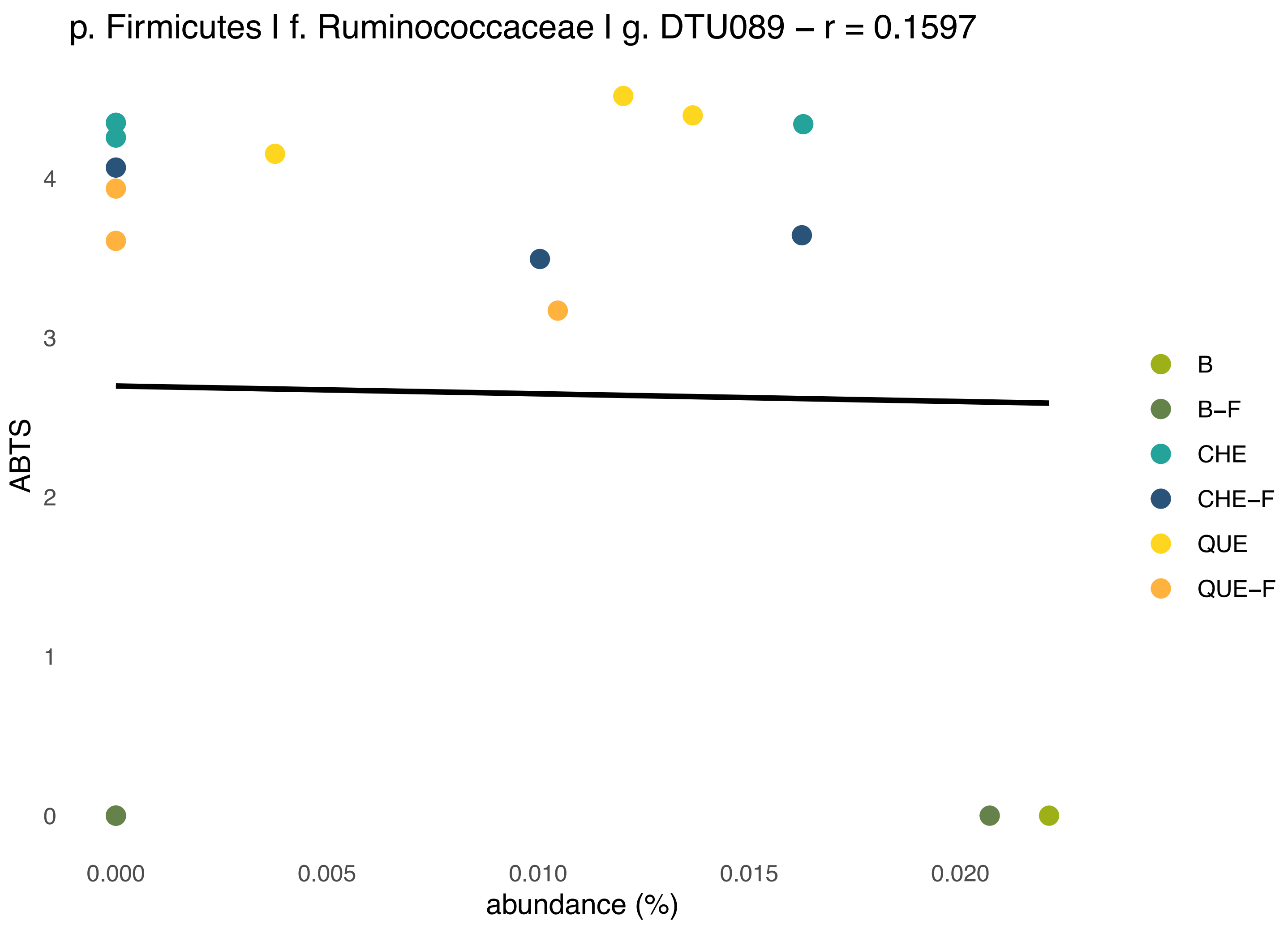
p. Firmicutes I f. Ruminococcaceae I g. DTU089 – r = 0.1597
ABTS
4
3
2
1
0
0.000
0.005
0.010
0.015
0.020
abundance (%)
B
B–F
CHE
CHE–F
QUE
QUE–F

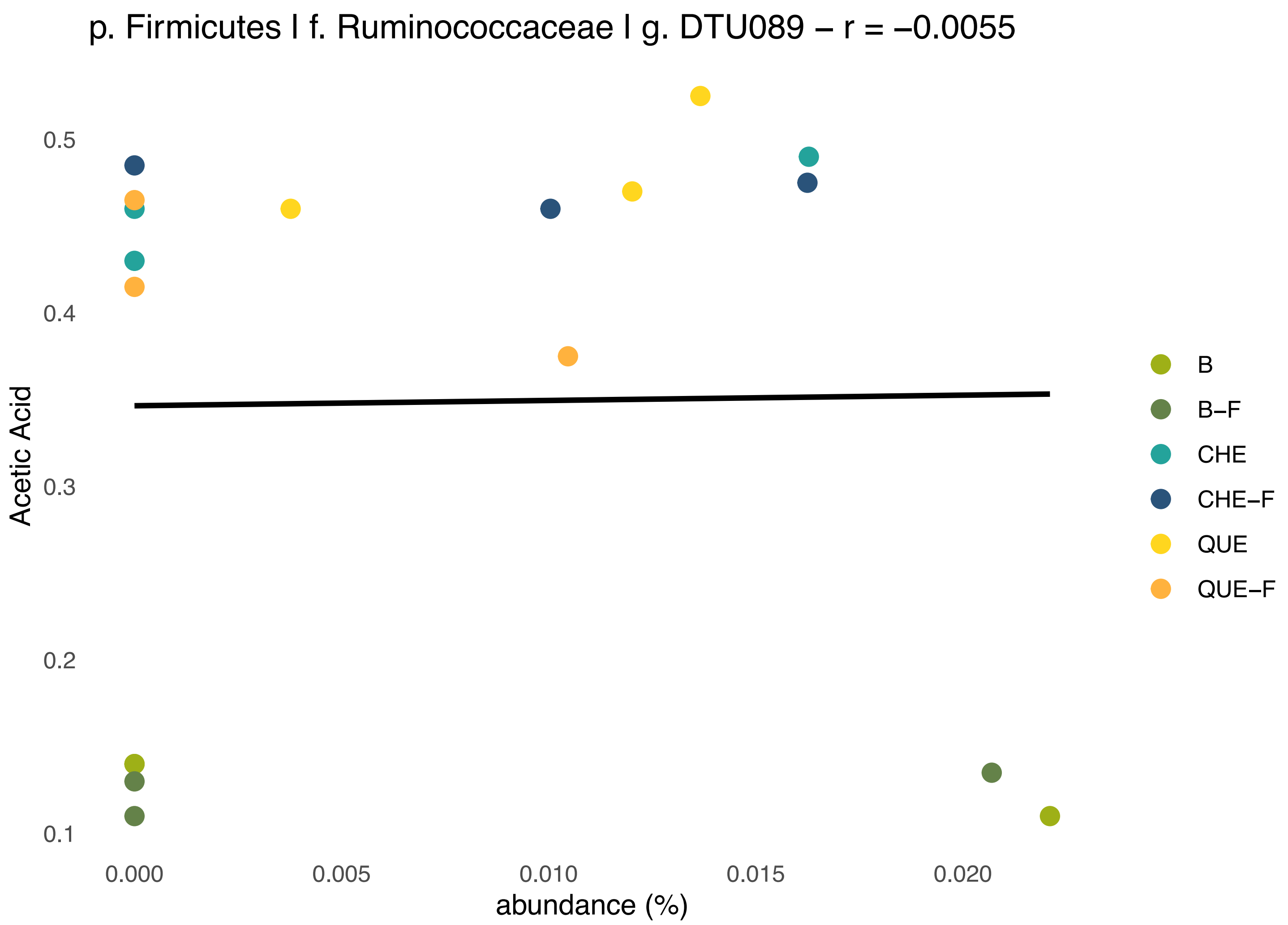

p. Firmicutes I f. Ruminococcaceae I g. DTU089 – r = –0.0055
Acetic Acid
0.5
0.4
0.3
0.2
0.1
0.000
0.005
0.010
0.015
0.020
abundance (%)
B
B–F
CHE
CHE–F
QUE
QUE–F

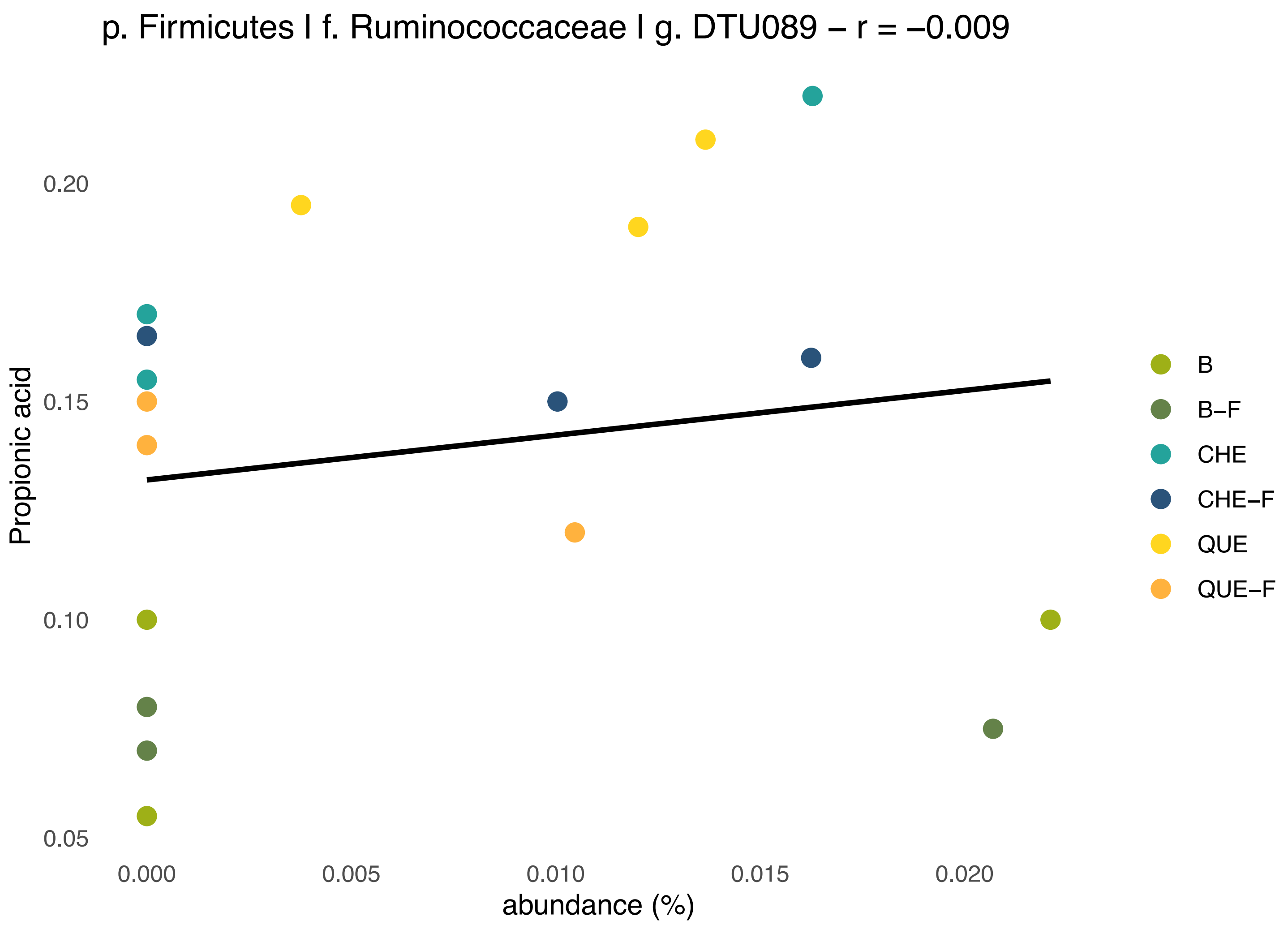
p. Firmicutes I f. Ruminococcaceae I g. DTU089 – r = −0.009
Propionic acid
0.20
0.15
0.10
0.05
0.000
0.005
0.010
0.015
0.020
abundance (%)
B
B−F
CHE
CHE−F
QUE
QUE−F

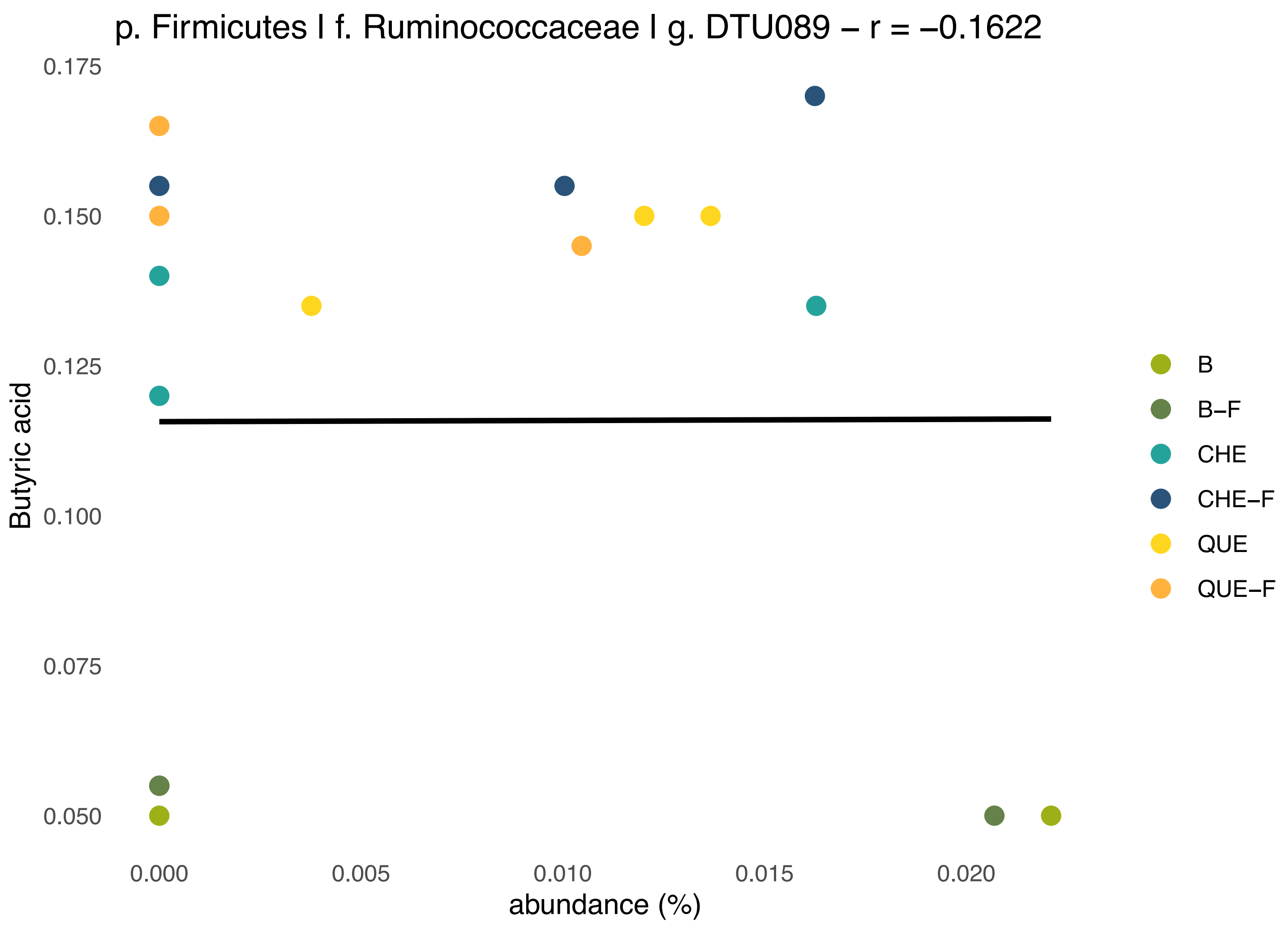

p. Firmicutes I f. Ruminococcaceae I g. DTU089 – r = −0.1622
Butyric acid
0.175
0.150
0.125
0.100
0.075
0.050
0.000
0.005
0.010
0.015
0.020
abundance (%)
B
B−F
CHE
CHE−F
QUE
QUE−F

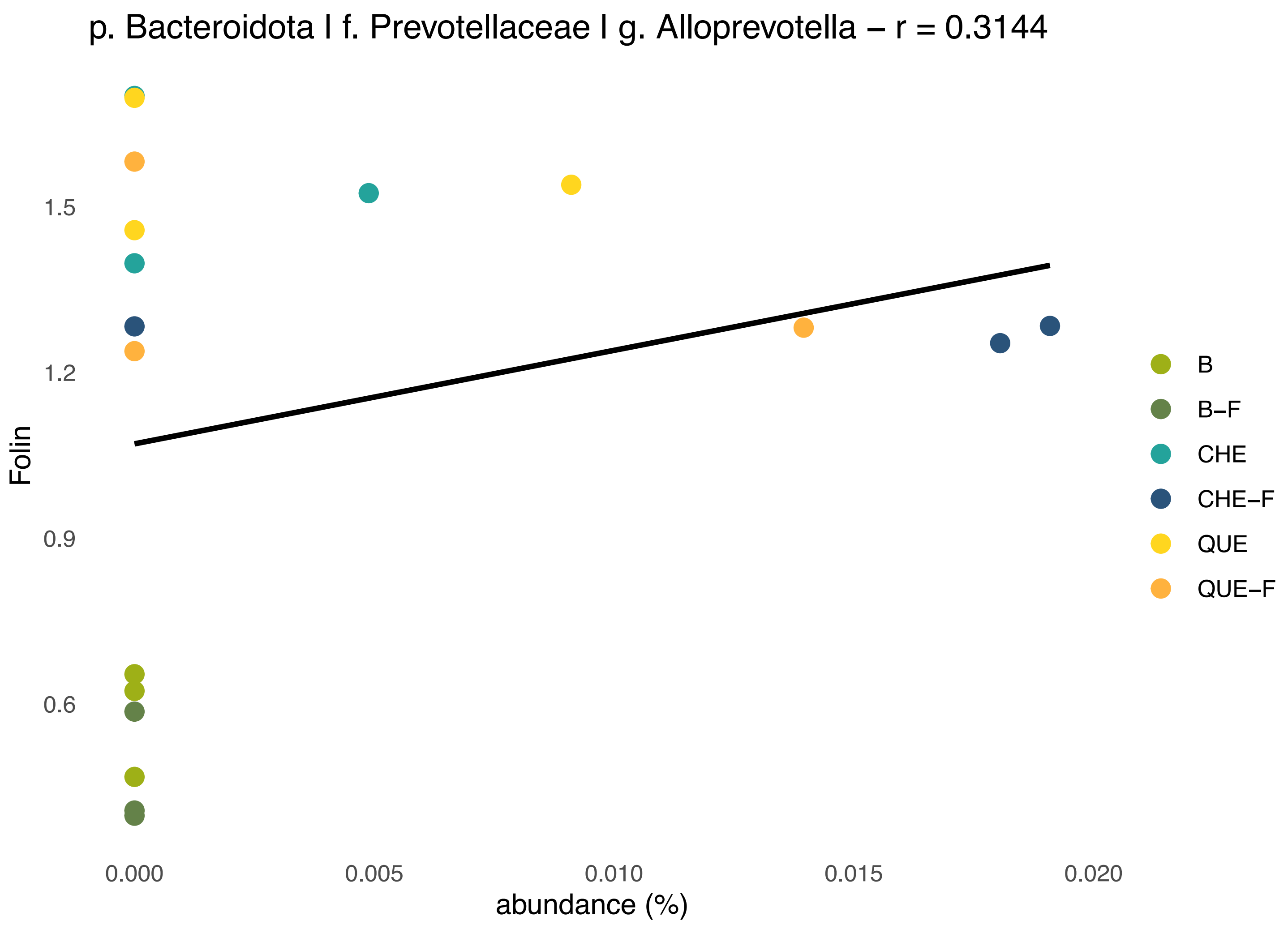
p. Bacteroidota | f. Prevotellaceae | g. Alloprevotella – r = 0.3144
1.5
1.2
0.9
0.6
Folin
0.000
0.005
0.010
0.015
0.020
abundance (%)
B
B–F
CHE
CHE–F
QUE
QUE–F

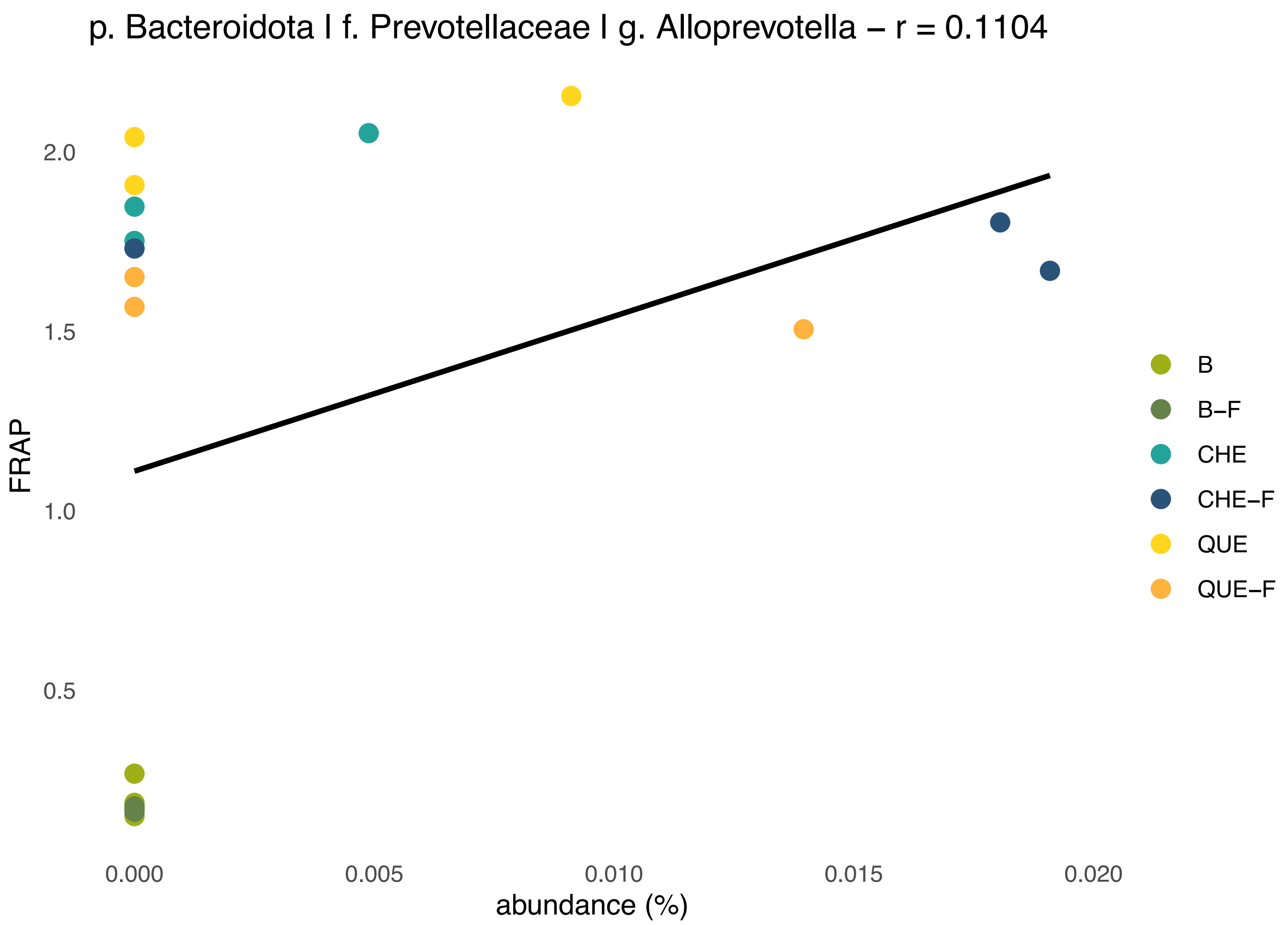

p. Bacteroidota I f. Prevotellaceae I g. Alloprevotella – r = 0.1104
FRAP
2.0
1.5
1.0
0.5
0.000
0.005
0.010
0.015
0.020
abundance (%)
B
B−F
CHE
CHE−F
QUE
QUE−F

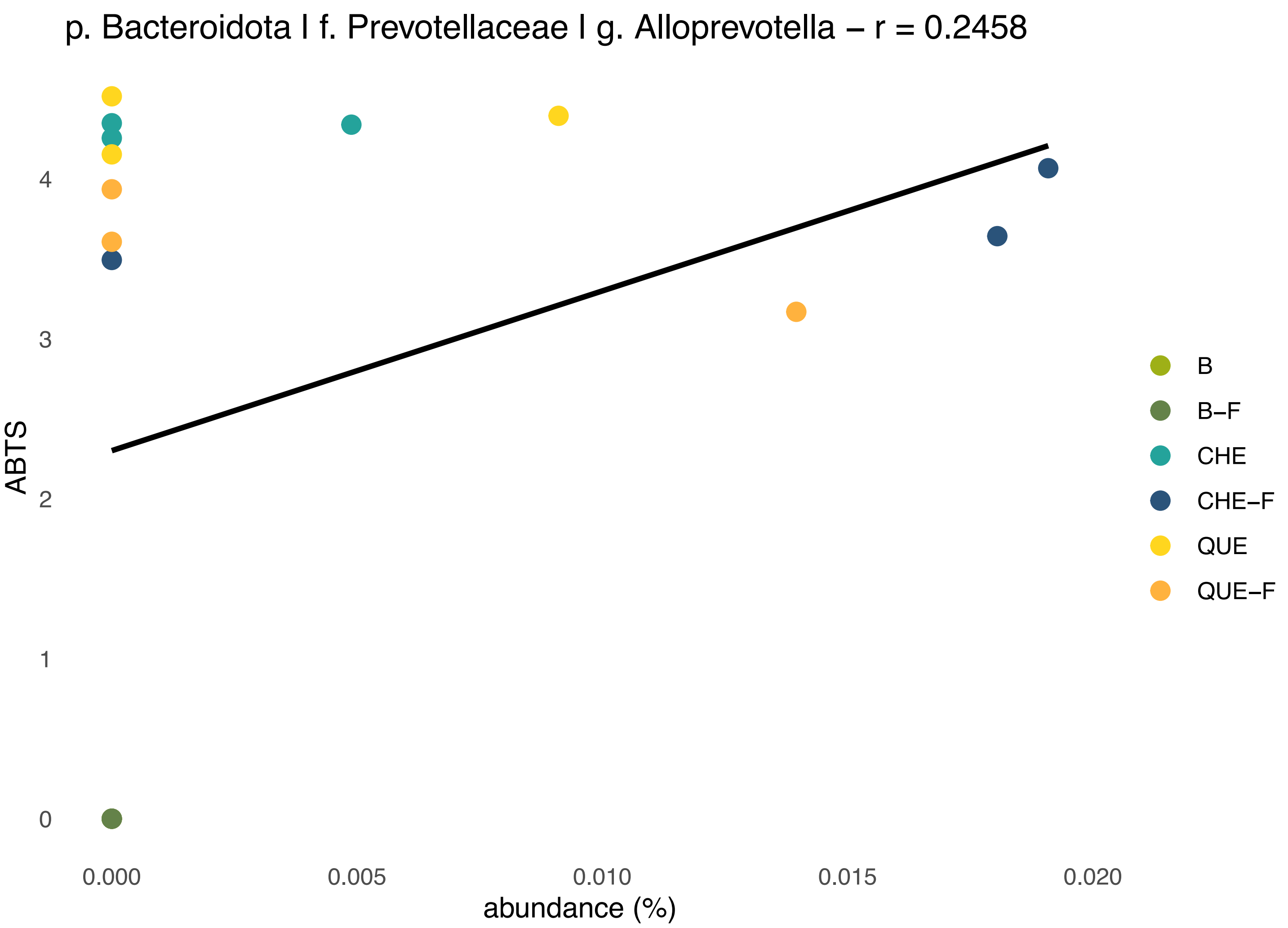
p. Bacteroidota I f. Prevotellaceae I g. Alloprevotella – r = 0.2458
ABTS
0
1
2
3
4
0.000
0.005
0.010
0.015
0.020
abundance (%)
B
B–F
CHE
CHE–F
QUE
QUE–F

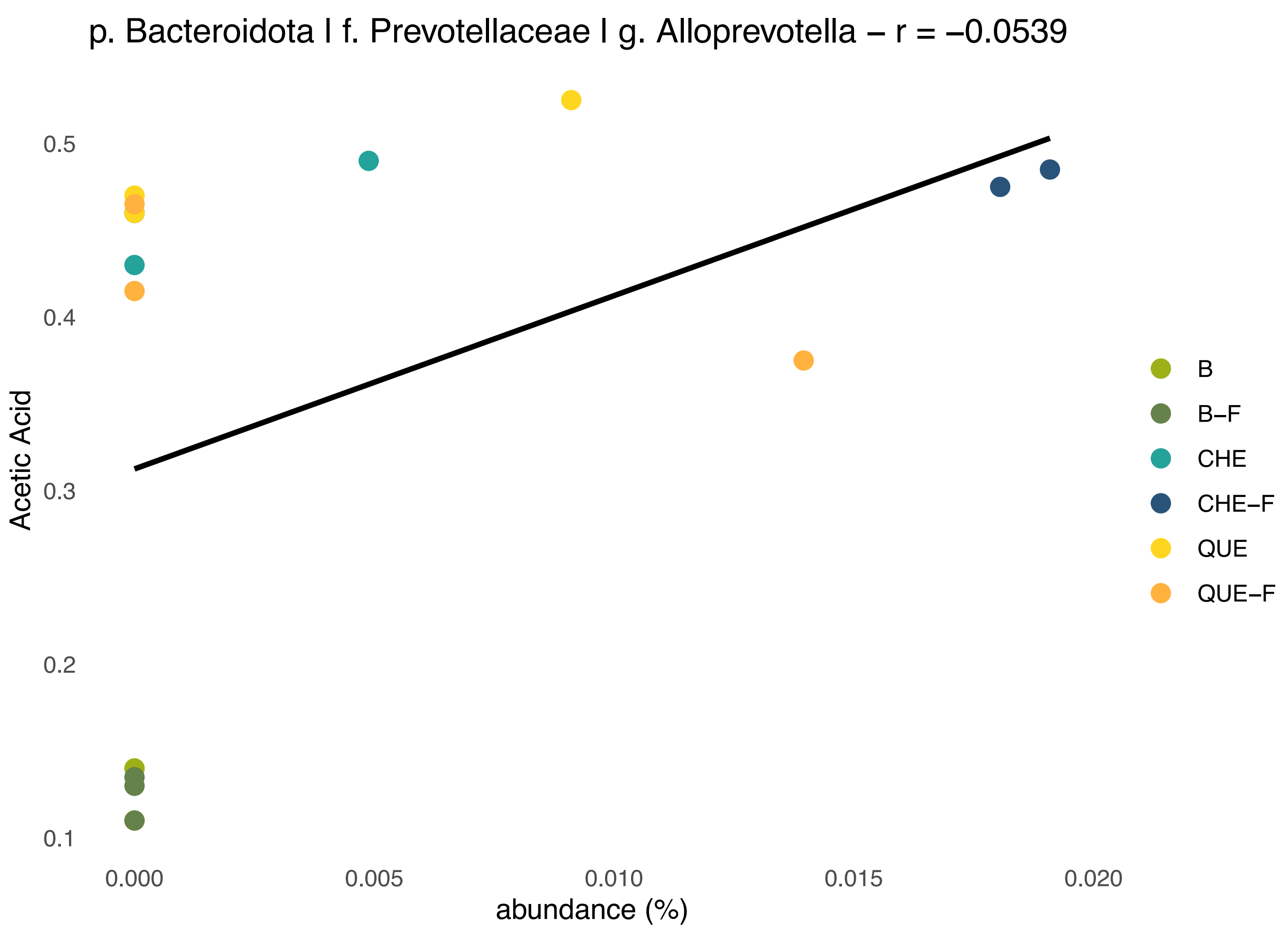
p. Bacteroidota I f. Prevotellaceae I g. Alloprevotella – r = –0.0539
Acetic Acid
0.5
0.4
0.3
0.2
0.1
0.000
0.005
0.010
0.015
0.020
abundance (%)
B
B–F
CHE
CHE–F
QUE
QUE–F

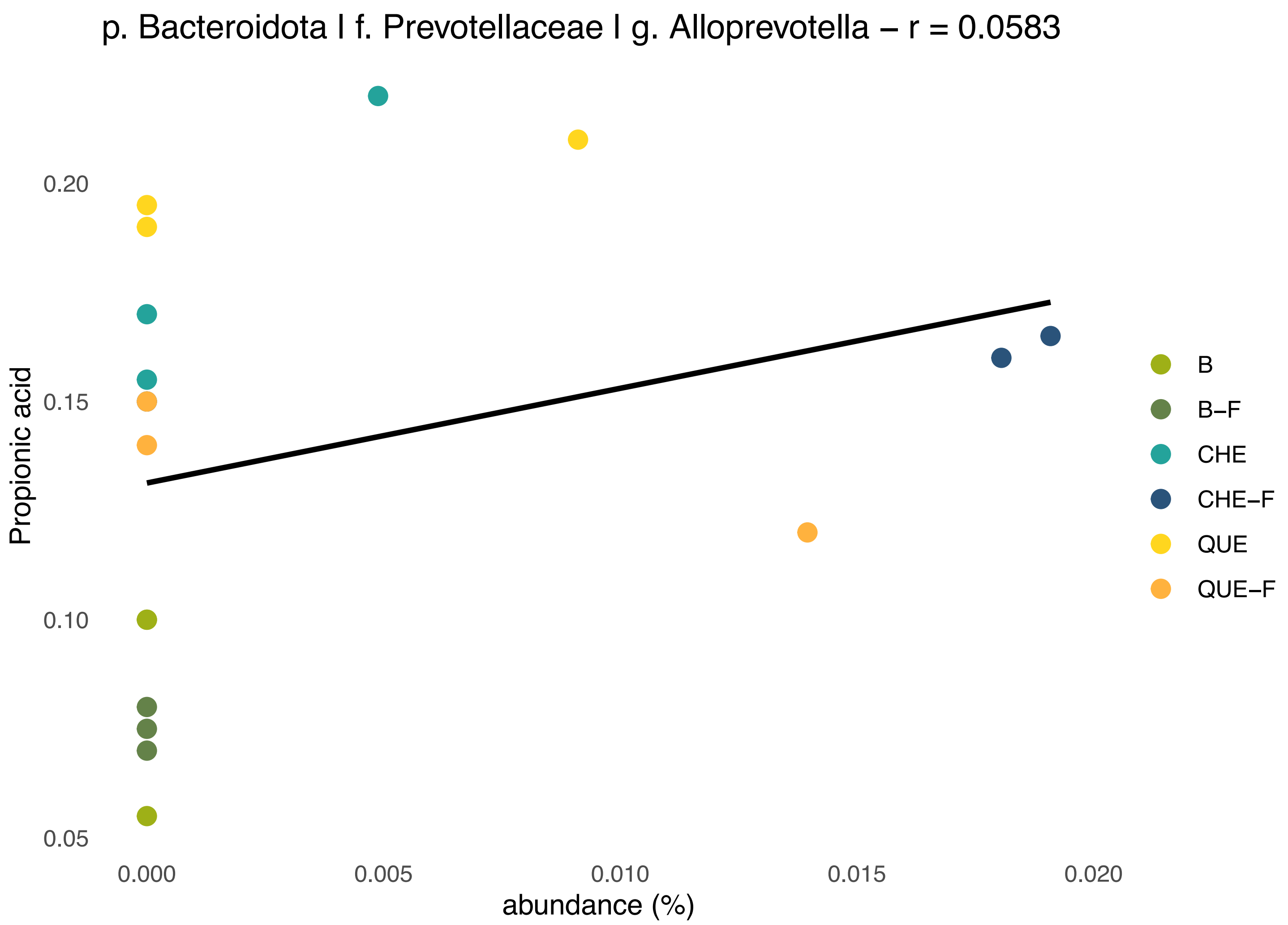

p. Bacteroidota l f. Prevotellaceae l g. Alloprevotella – r = 0.0583
Propionic acid
0.20
0.15
0.10
0.05
0.000
0.005
0.010
0.015
0.020
abundance (%)
B
B–F
CHE
CHE–F
QUE
QUE–F

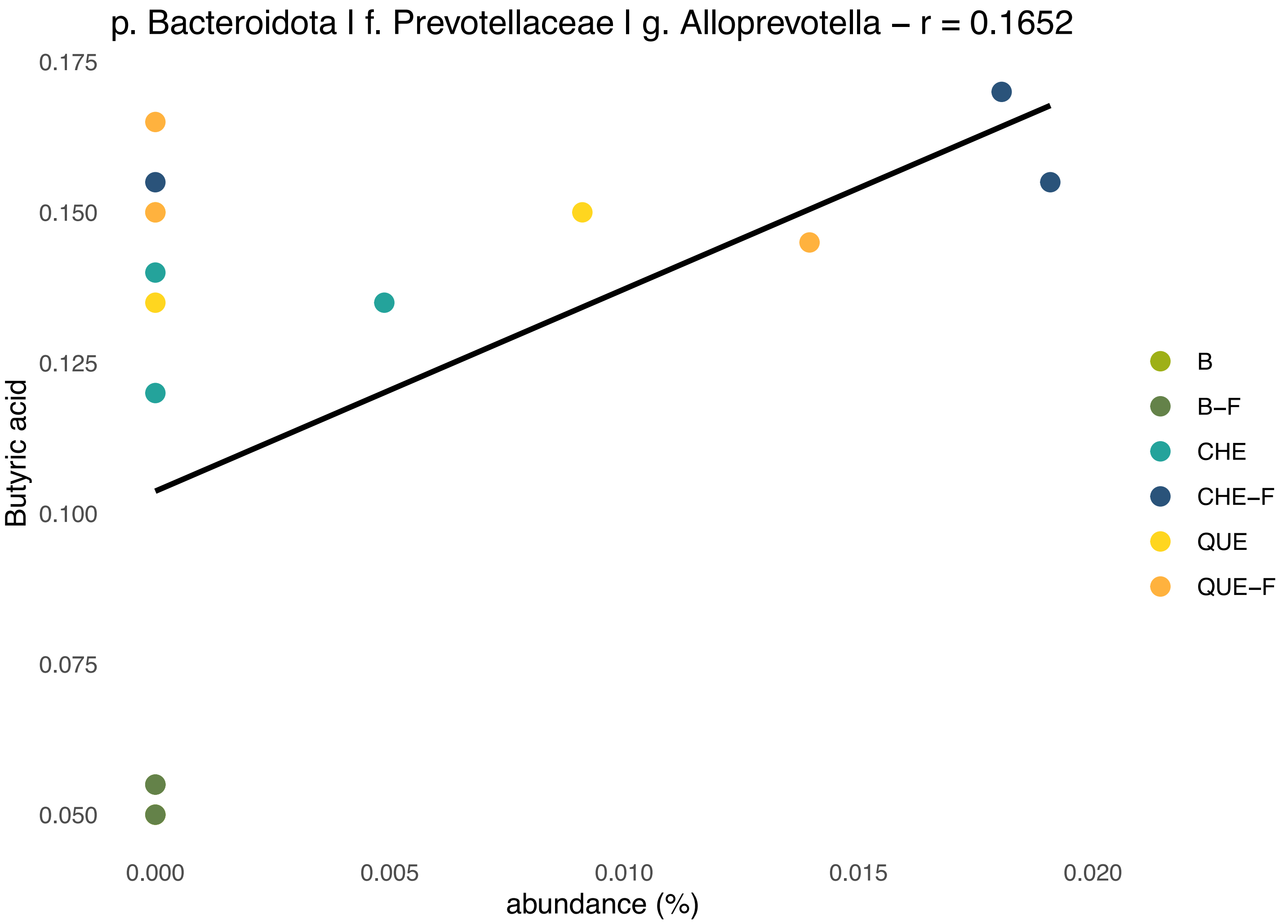

p. Bacteroidota I f. Prevotellaceae I g. Alloprevotella – r = 0.1652
Butyric acid
0.175
0.150
0.125
0.100
0.075
0.050
0.000
0.005
0.010
0.015
0.020
abundance (%)
B
B–F
CHE
CHE–F
QUE
QUE–F

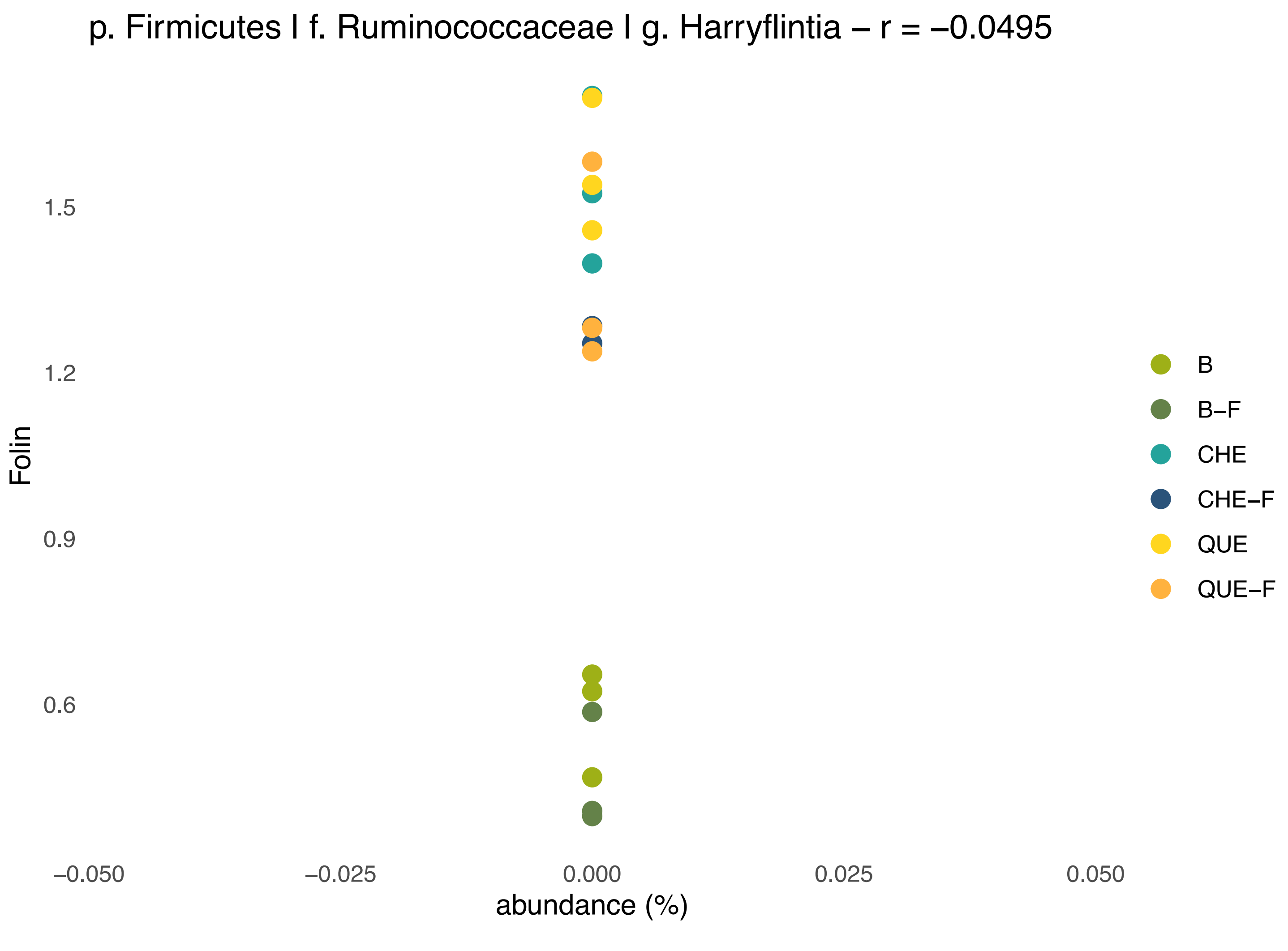

p. Firmicutes l f. Ruminococcaceae l g. Harryflintia – r = –0.0495
1.5
1.2
0.9
0.6
Folin
–0.050
–0.025
0.000
0.025
0.050
abundance (%)
B
B–F
CHE
CHE–F
QUE
QUE–F

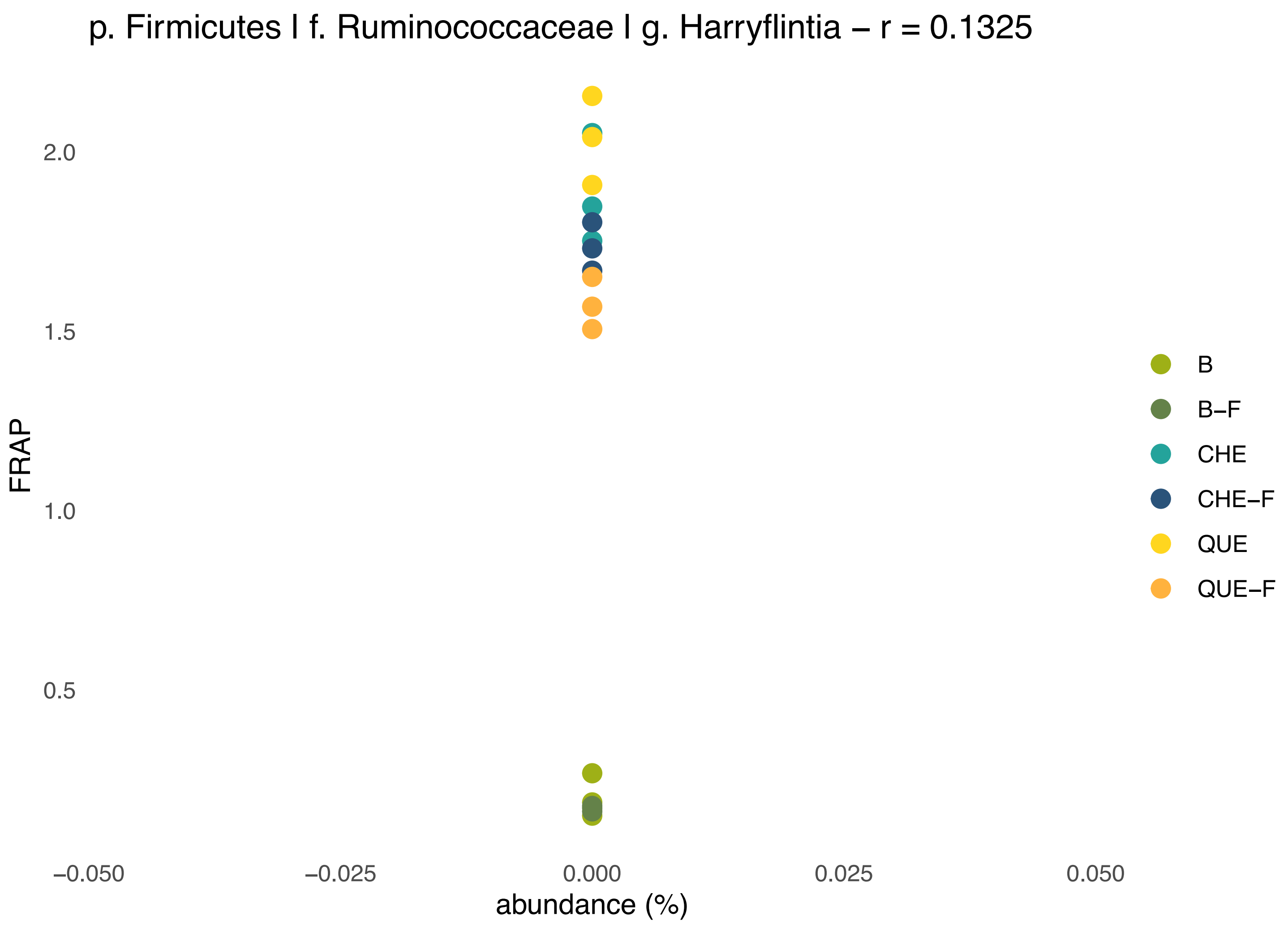

p. Firmicutes I f. Ruminococcaceae I g. Harryflintia – r = 0.1325
FRAP
2.0
1.5
1.0
0.5
−0.050
−0.025
0.000
0.025
0.050
abundance (%)
B
B−F
CHE
CHE−F
QUE
QUE−F

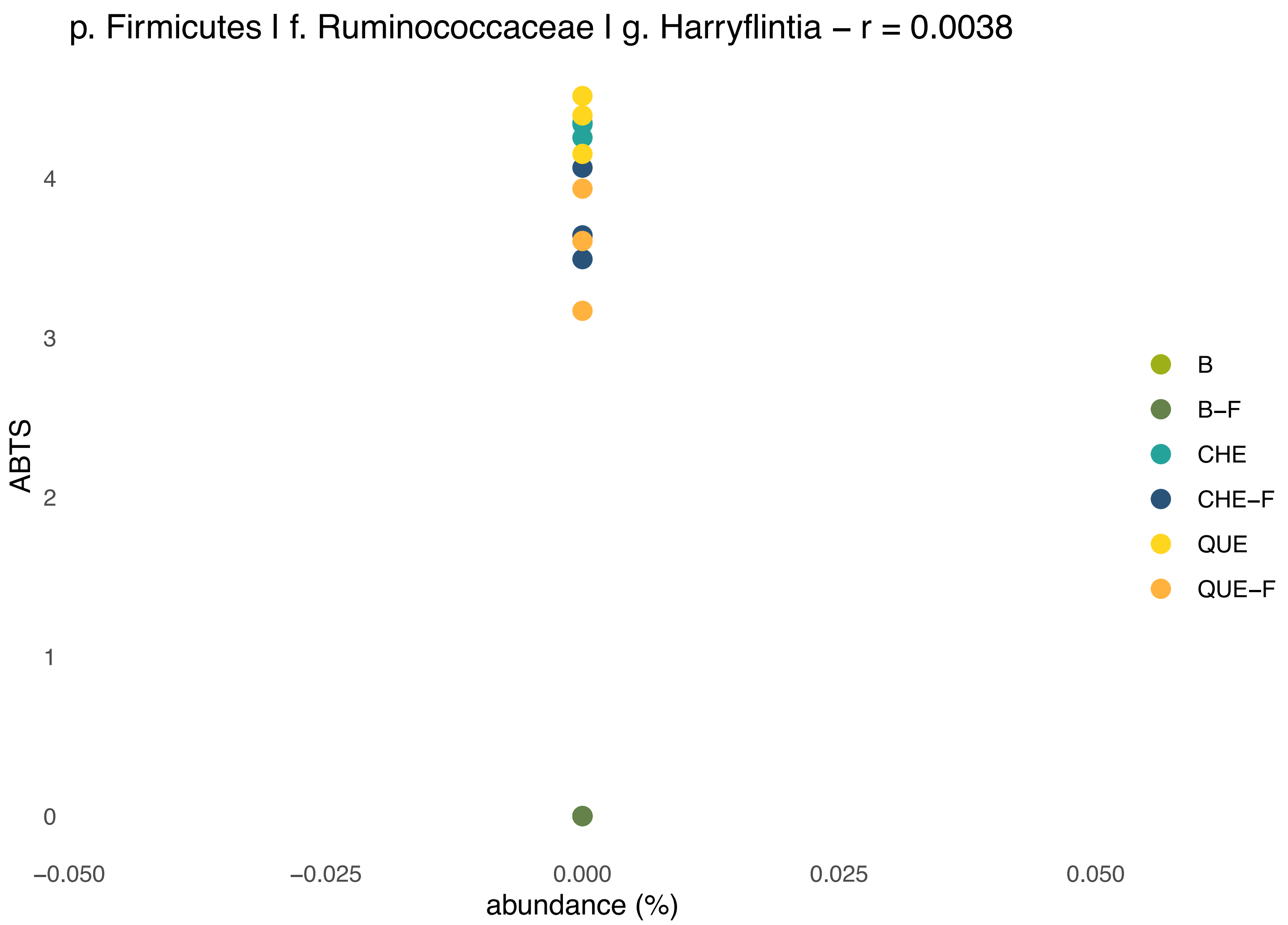
p. Firmicutes I f. Ruminococcaceae I g. Harryflintia – r = 0.0038
ABTS
4
3
2
1
0
–0.050
–0.025
0.000
0.025
0.050
abundance (%)
B
B–F
CHE
CHE–F
QUE
QUE–F

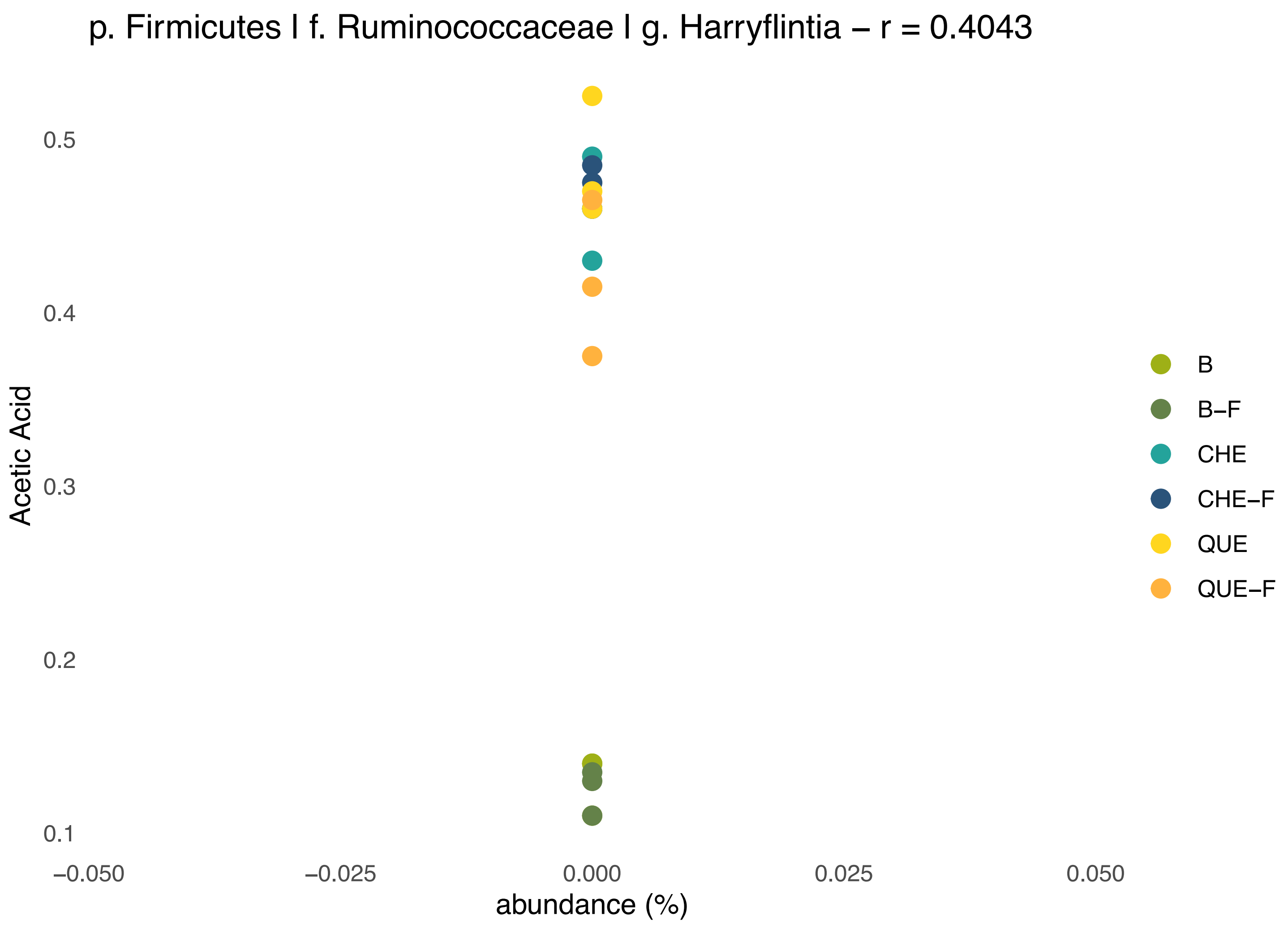

p. Firmicutes | f. Ruminococcaceae | g. Harryflintia – r = 0.4043
Acetic Acid
0.5
0.4
0.3
0.2
0.1
−0.050
−0.025
0.000
0.025
0.050
abundance (%)
B
B−F
CHE
CHE−F
QUE
QUE−F

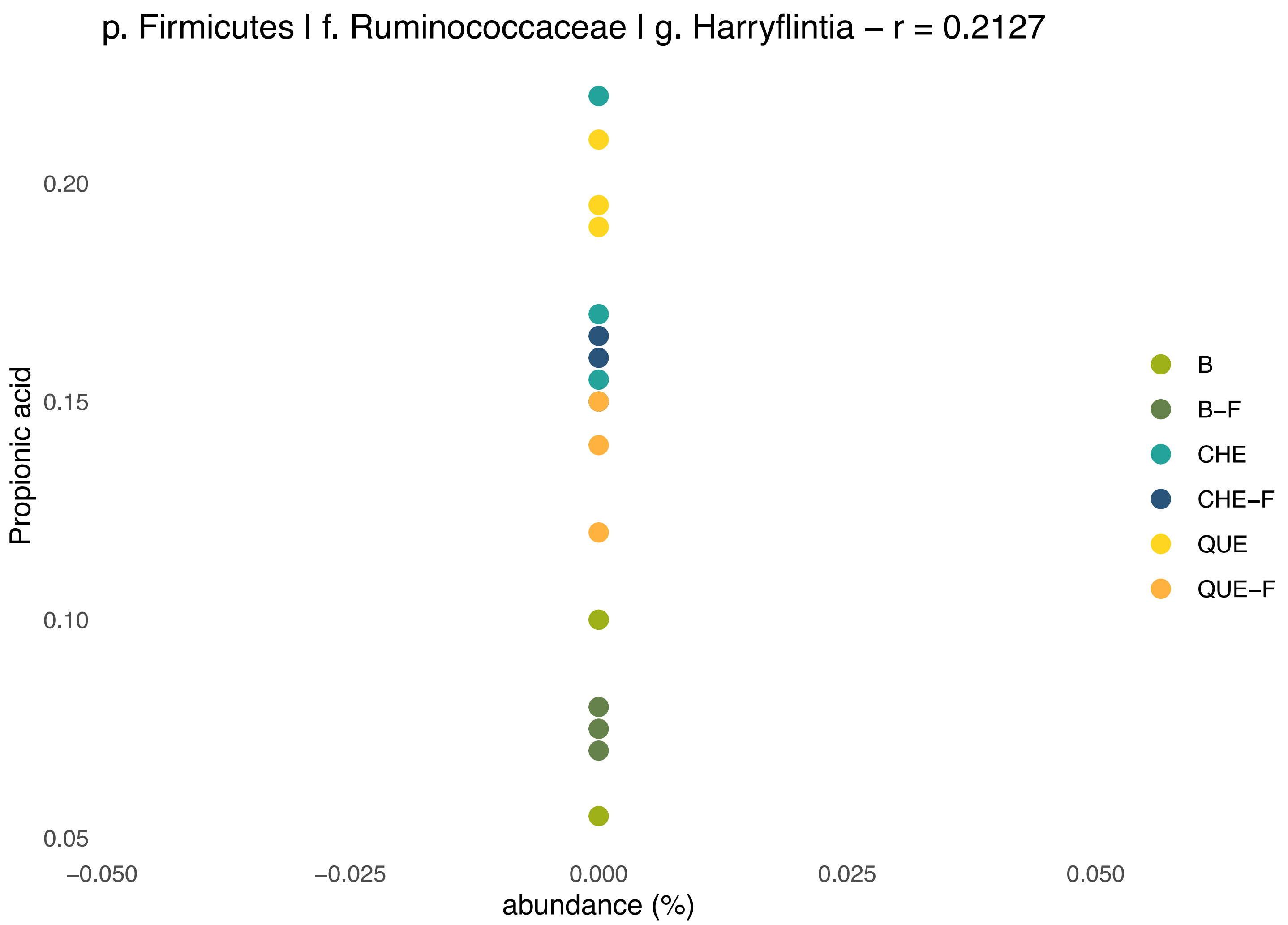
p. Firmicutes | f. Ruminococcaceae | g. Harryflintia – r = 0.2127
Propionic acid
0.20
0.15
0.10
0.05
–0.050
–0.025
0.000
0.025
0.050
abundance (%)
B
B–F
CHE
CHE–F
QUE
QUE–F

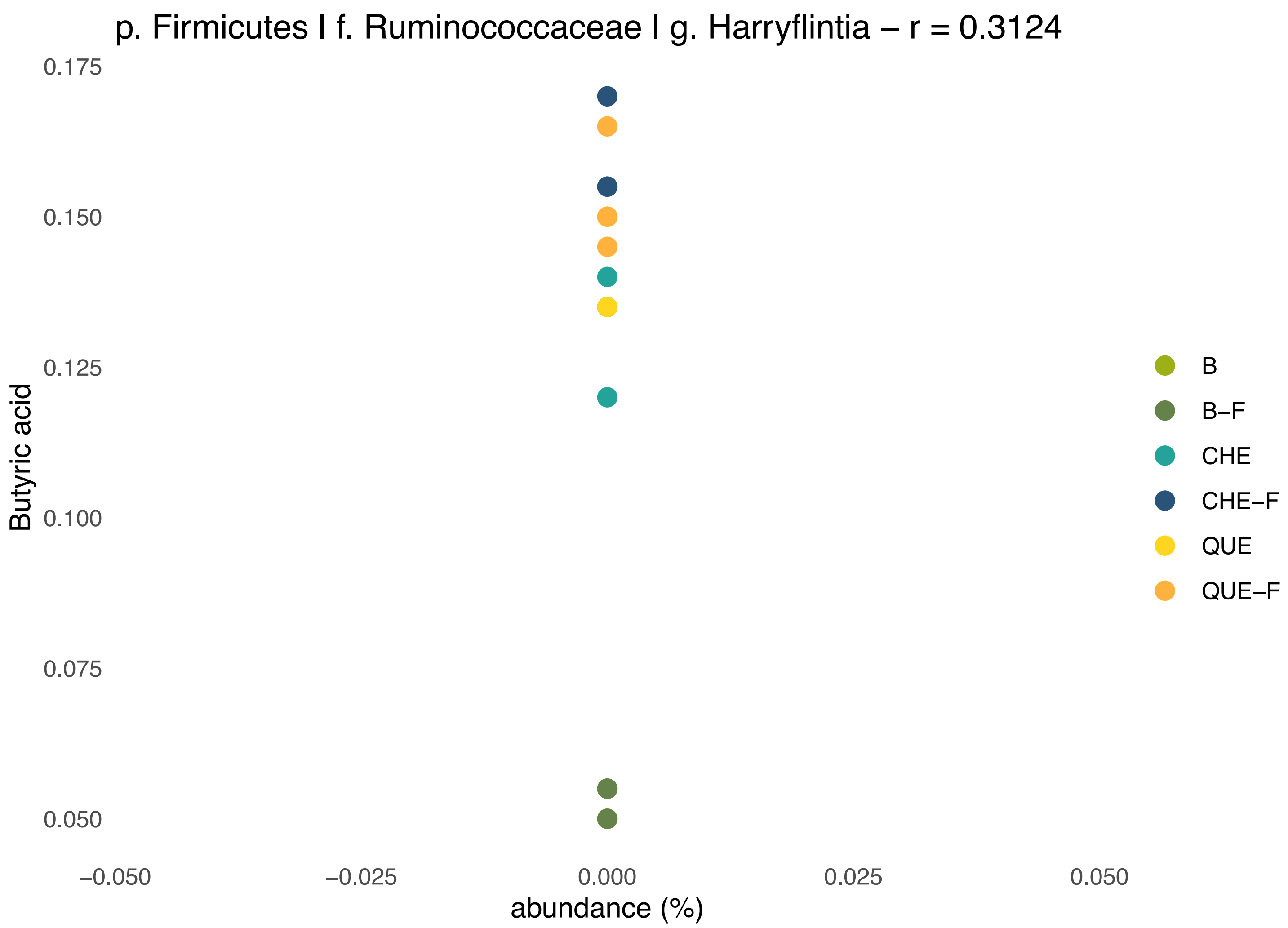
p. Firmicutes l f. Ruminococcaceae l g. Harryflintia – r = 0.3124
Butyric acid
0.175
0.150
0.125
0.100
0.075
0.050
–0.050
–0.025
0.000
0.025
0.050
abundance (%)
B
B–F
CHE
CHE–F
QUE
QUE–F

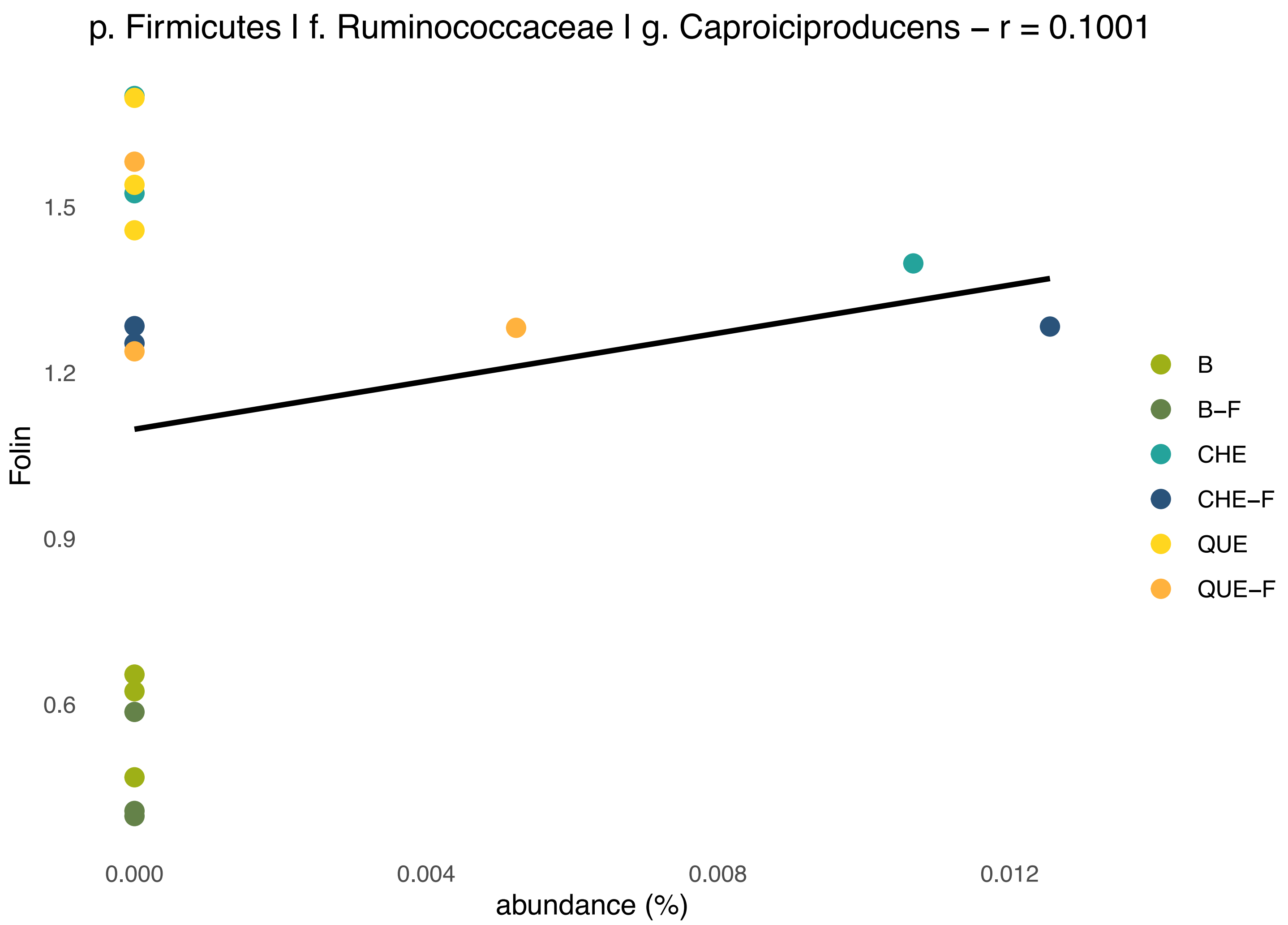
p. Firmicutes I f. Ruminococcaceae I g. Caproiciproducens − r = 0.1001
Folin
1.5
1.2
0.9
0.6
0.000
0.004
0.008
0.012
abundance (%)
B
B−F
CHE
CHE−F
QUE
QUE−F

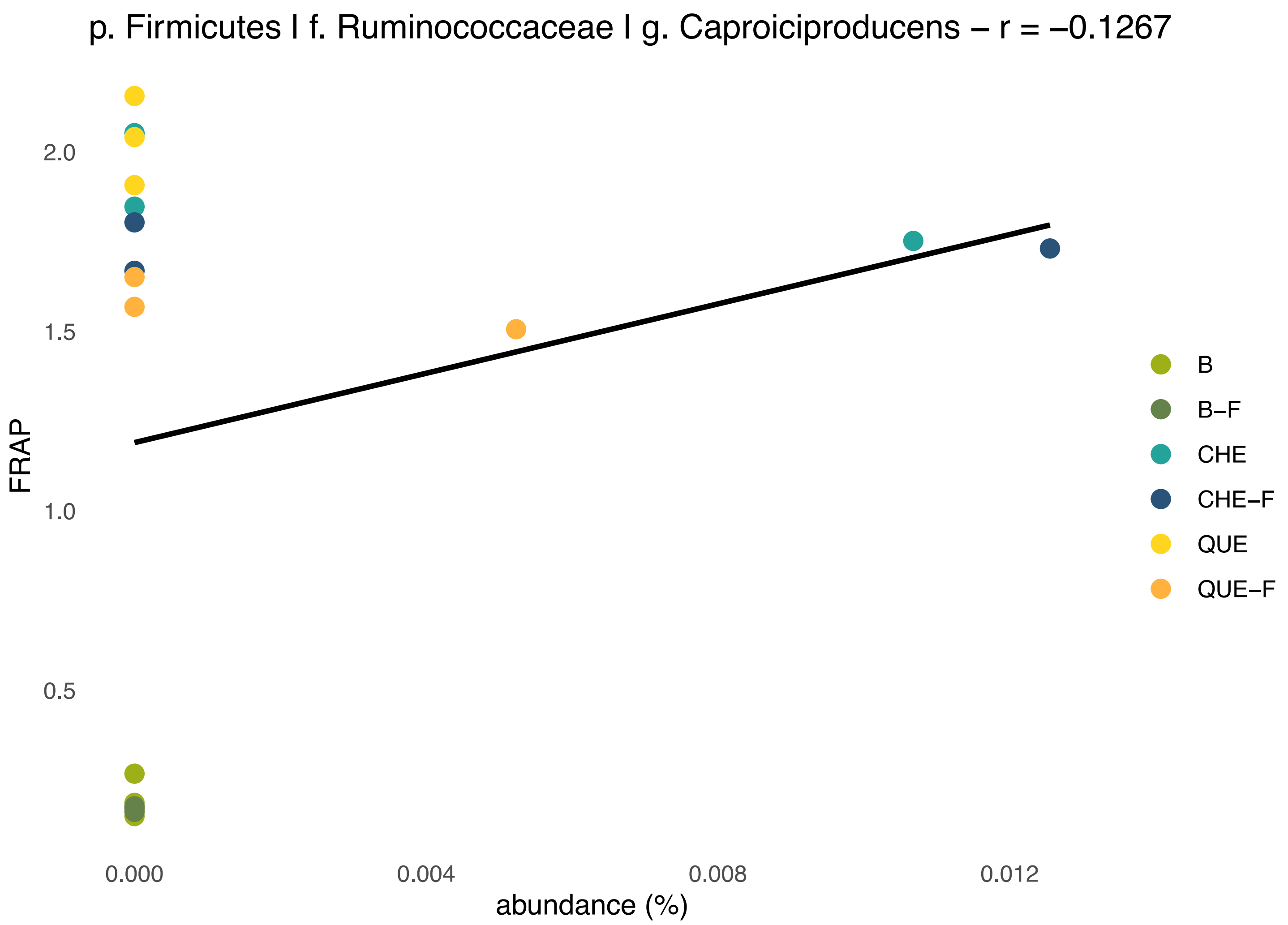
p. Firmicutes l f. Ruminococcaceae l g. Caproiciproducens – r = –0.1267
FRAP
2.0
1.5
1.0
0.5
0.000
0.004
0.008
0.012
abundance (%)
B
B–F
CHE
CHE–F
QUE
QUE–F

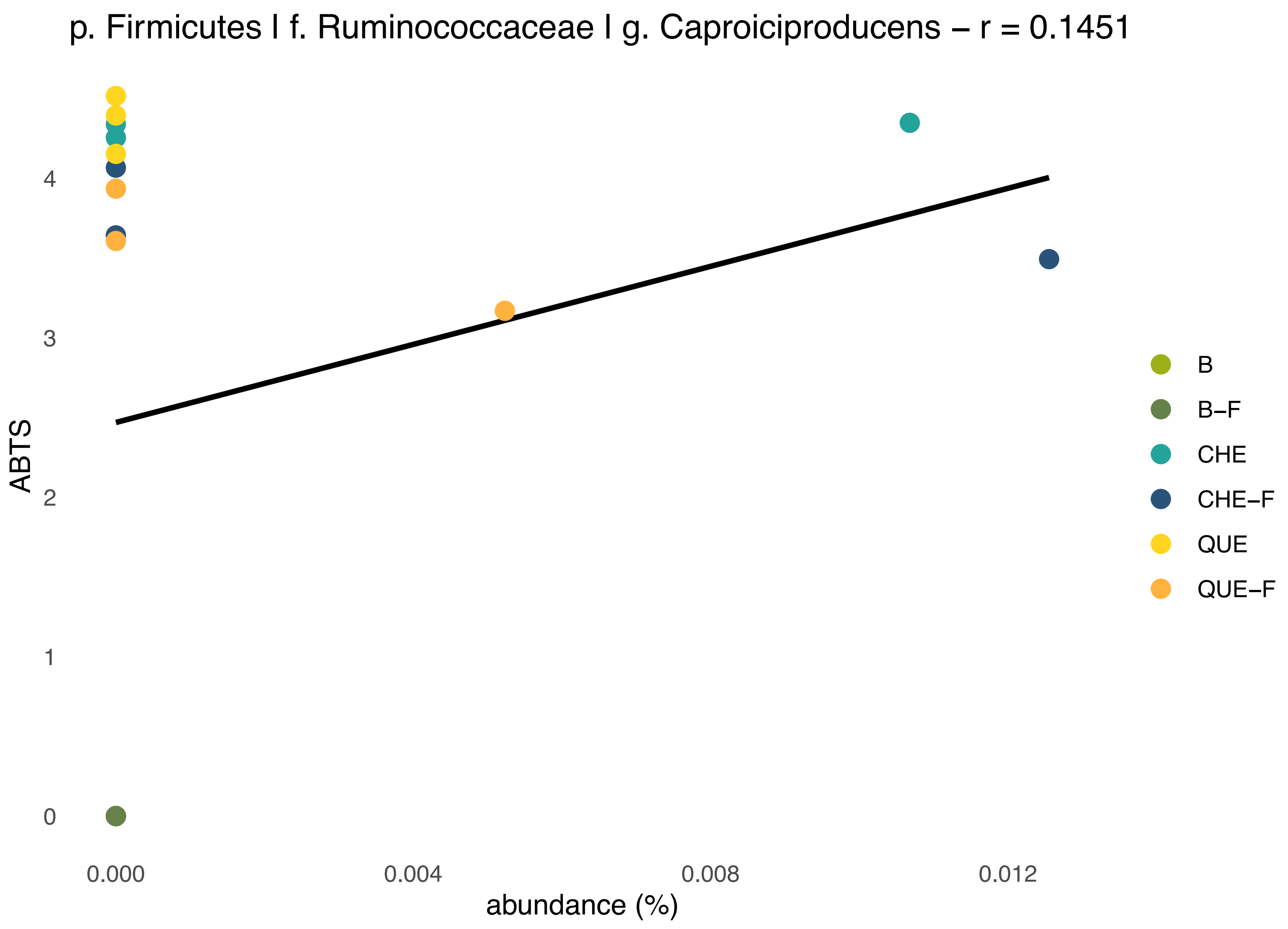

p. Firmicutes I f. Ruminococcaceae I g. Caproiciproducens – r = 0.1451
ABTS
0
1
2
3
4
0.000
0.004
0.008
0.012
abundance (%)
B
B–F
CHE
CHE–F
QUE
QUE–F

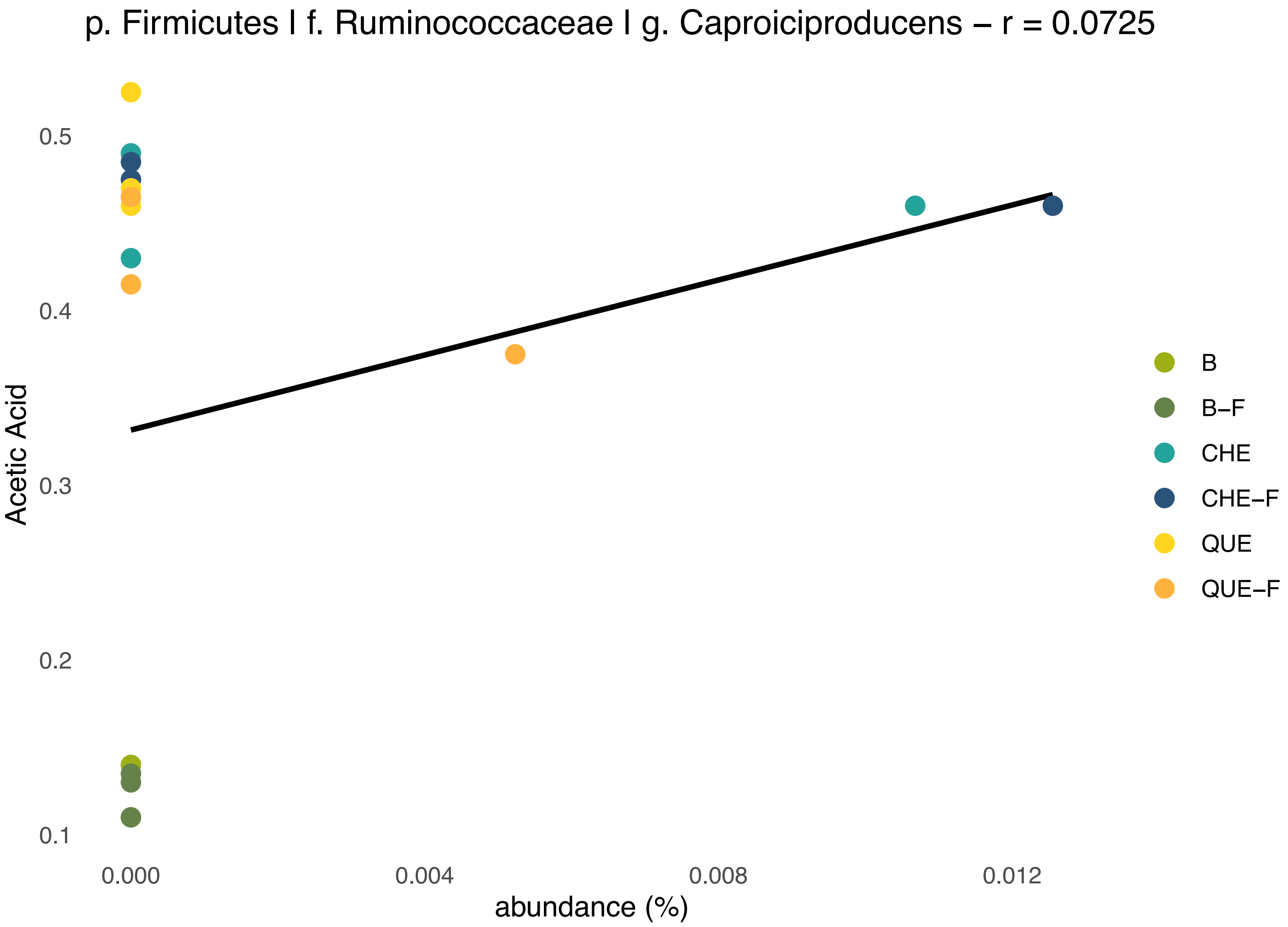

p. Firmicutes I f. Ruminococcaceae I g. Caproiciproducens – r = 0.0725
Acetic Acid
0.5
0.4
0.3
0.2
0.1
0.000
0.004
0.008
0.012
abundance (%)
B
B–F
CHE
CHE–F
QUE
QUE–F

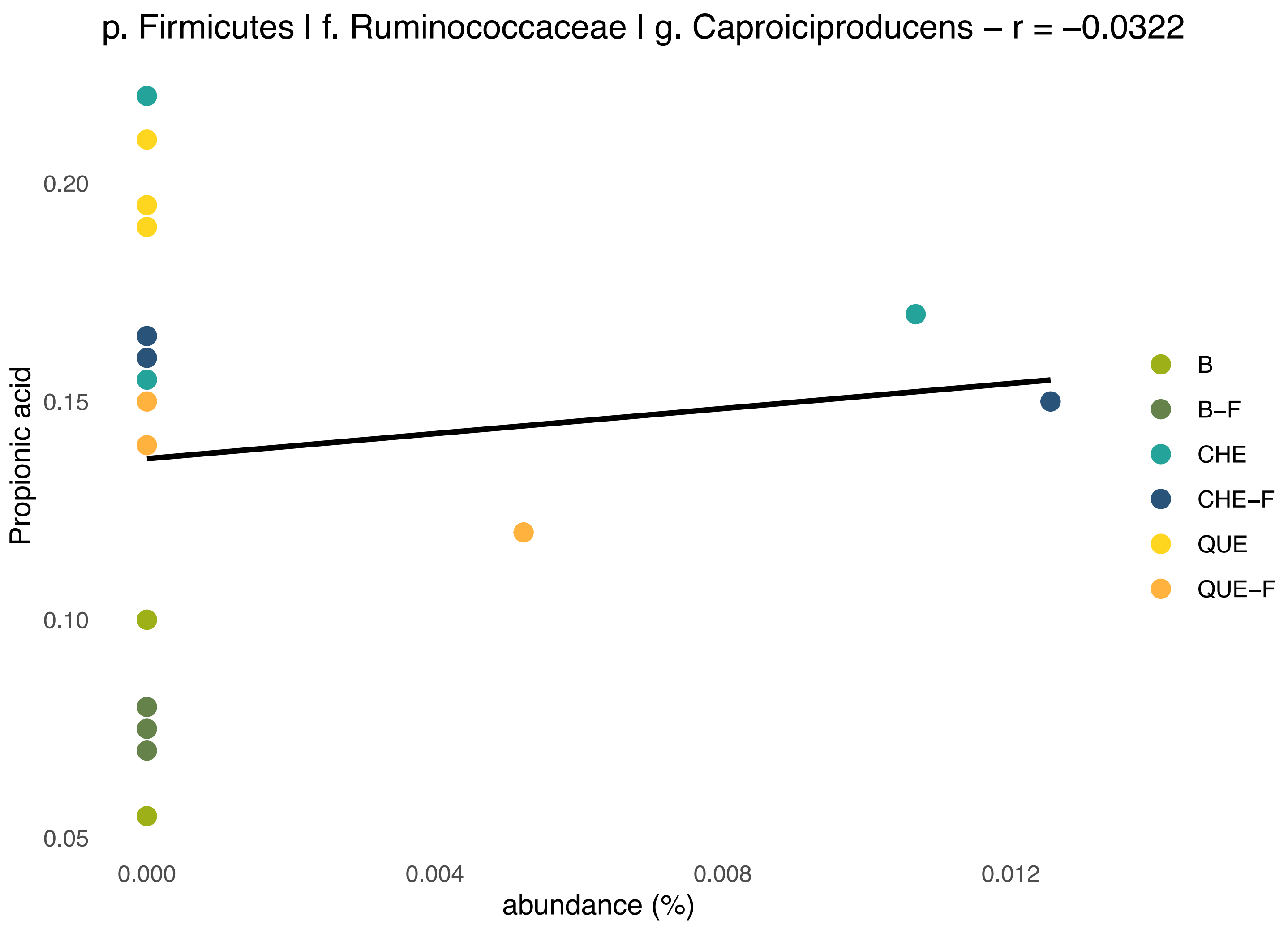
p. Firmicutes I f. Ruminococcaceae I g. Caproiciproducens – r = −0.0322
Propionic acid
0.20
0.15
0.10
0.05
0.000
0.004
0.008
0.012
abundance (%)
B
B−F
CHE
CHE−F
QUE
QUE−F

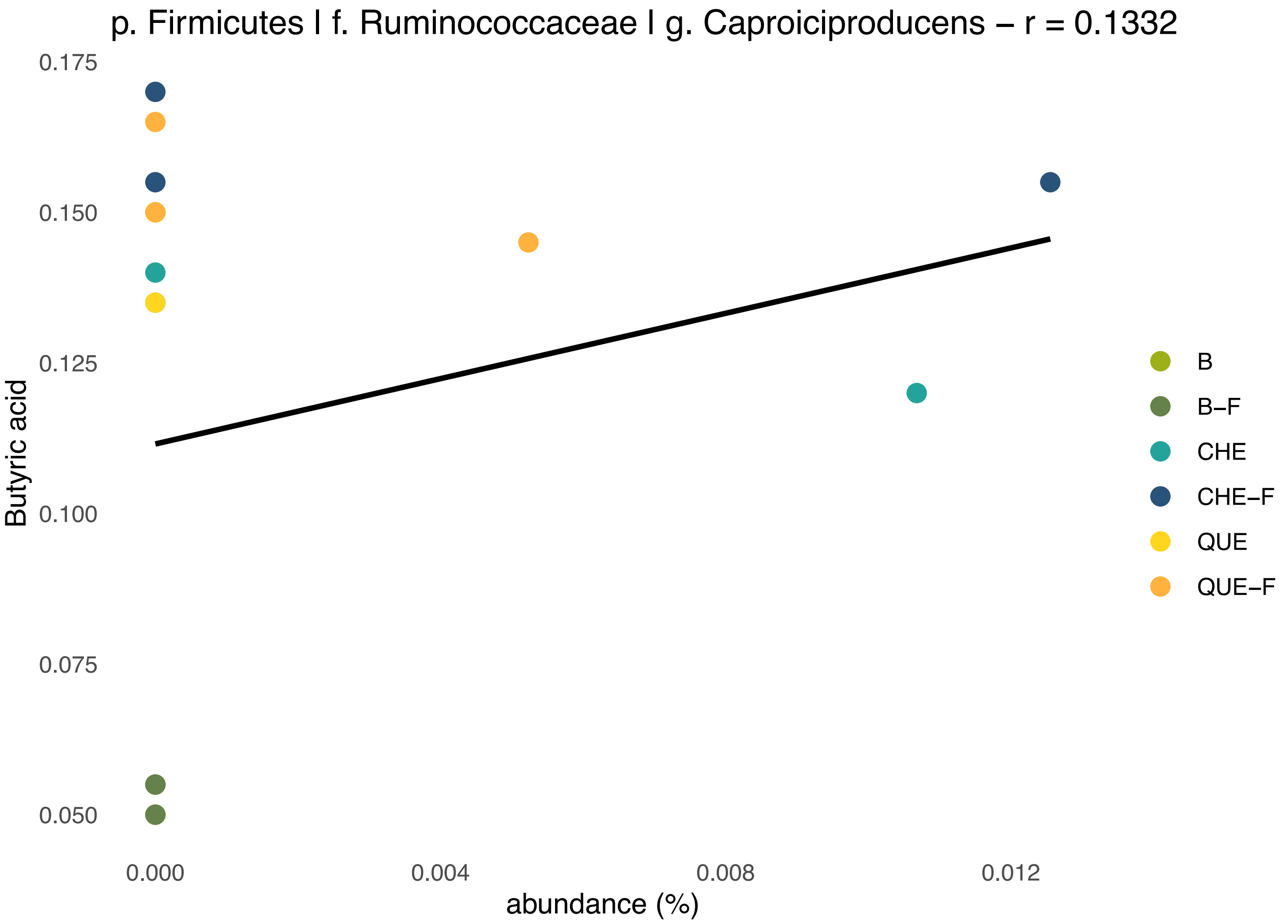

p. Firmicutes I f. Ruminococcaceae I g. Caproiciproducens − r = 0.1332
Butyric acid
0.175
0.150
0.125
0.100
0.075
0.050
0.000
0.004
0.008
0.012
abundance (%)
B
B−F
CHE
CHE−F
QUE
QUE−F

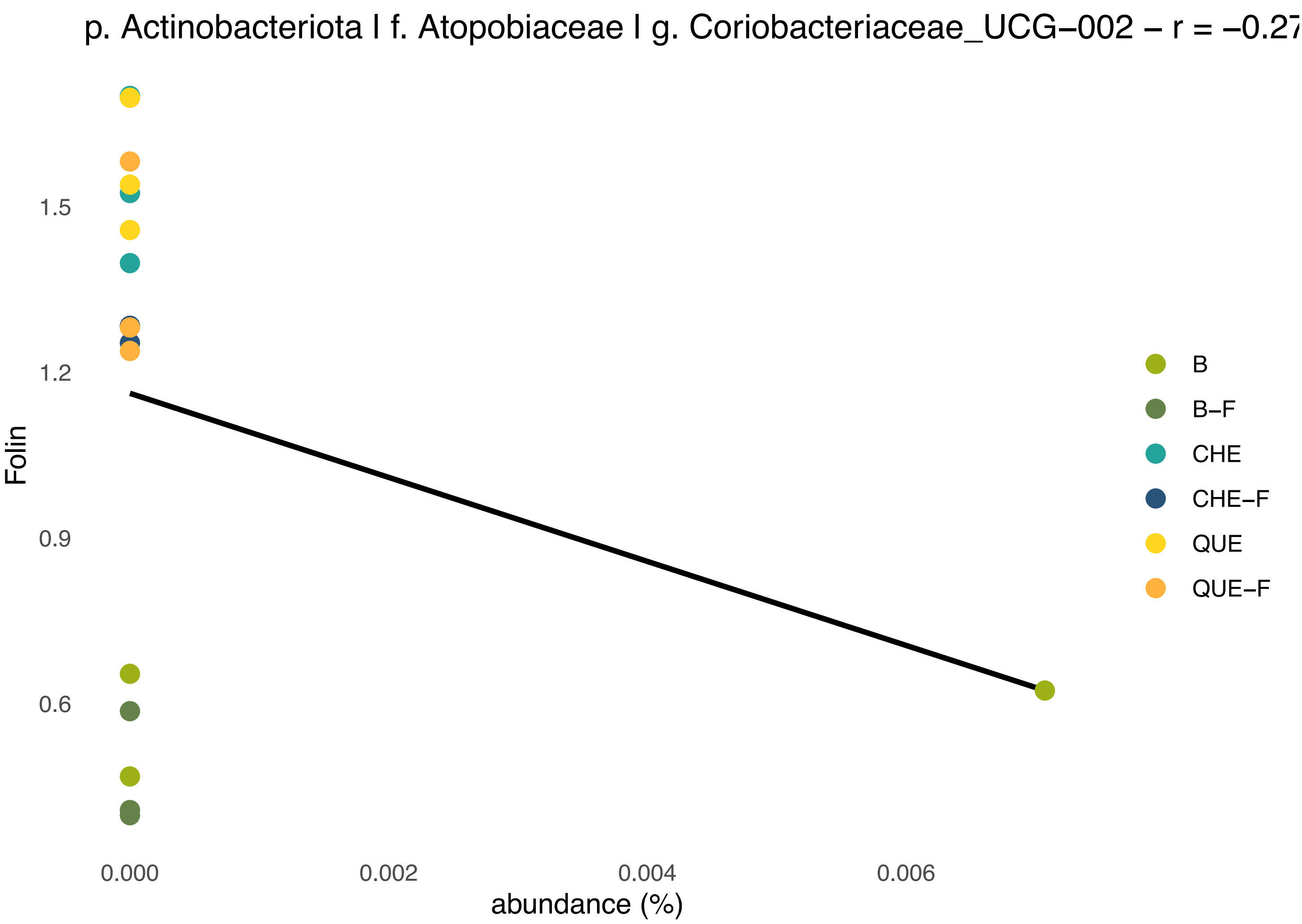
p. Actinobacteriota I f. Atopobiaceae I g. Coriobacteriaceae_UCG−002 − r = −0.27
Folin
1.5
1.2
0.9
0.6
0.000
0.002
0.004
0.006
abundance (%)
B
B−F
CHE
CHE−F
QUE
QUE−F

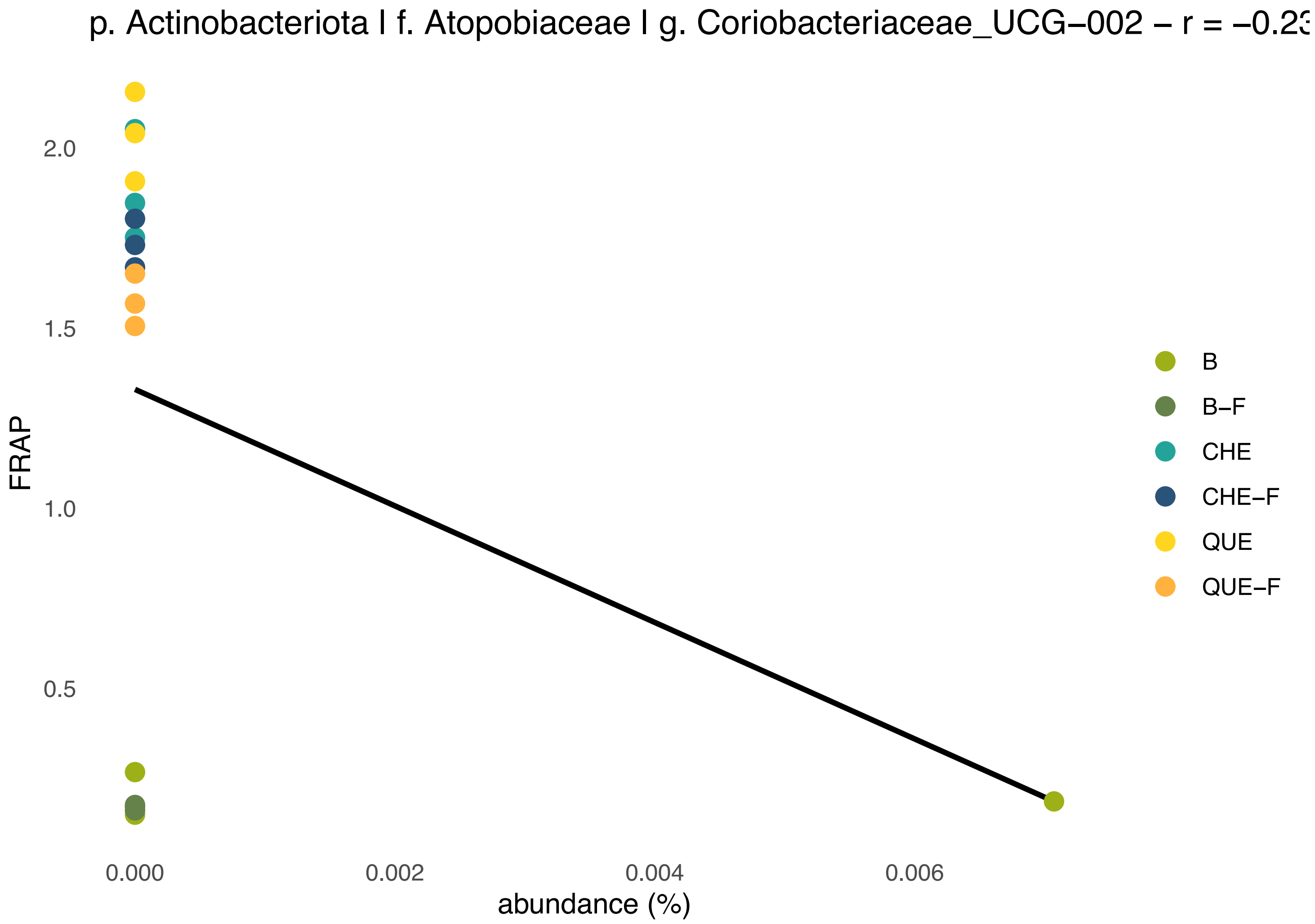
p. Actinobacteriota l f. Atopobiaceae l g. Coriobacteriaceae_UCG−002 − r = −0.23
FRAP
2.0
1.5
1.0
0.5
0.000
0.002
0.004
0.006
abundance (%)
B
B−F
CHE
CHE−F
QUE
QUE−F

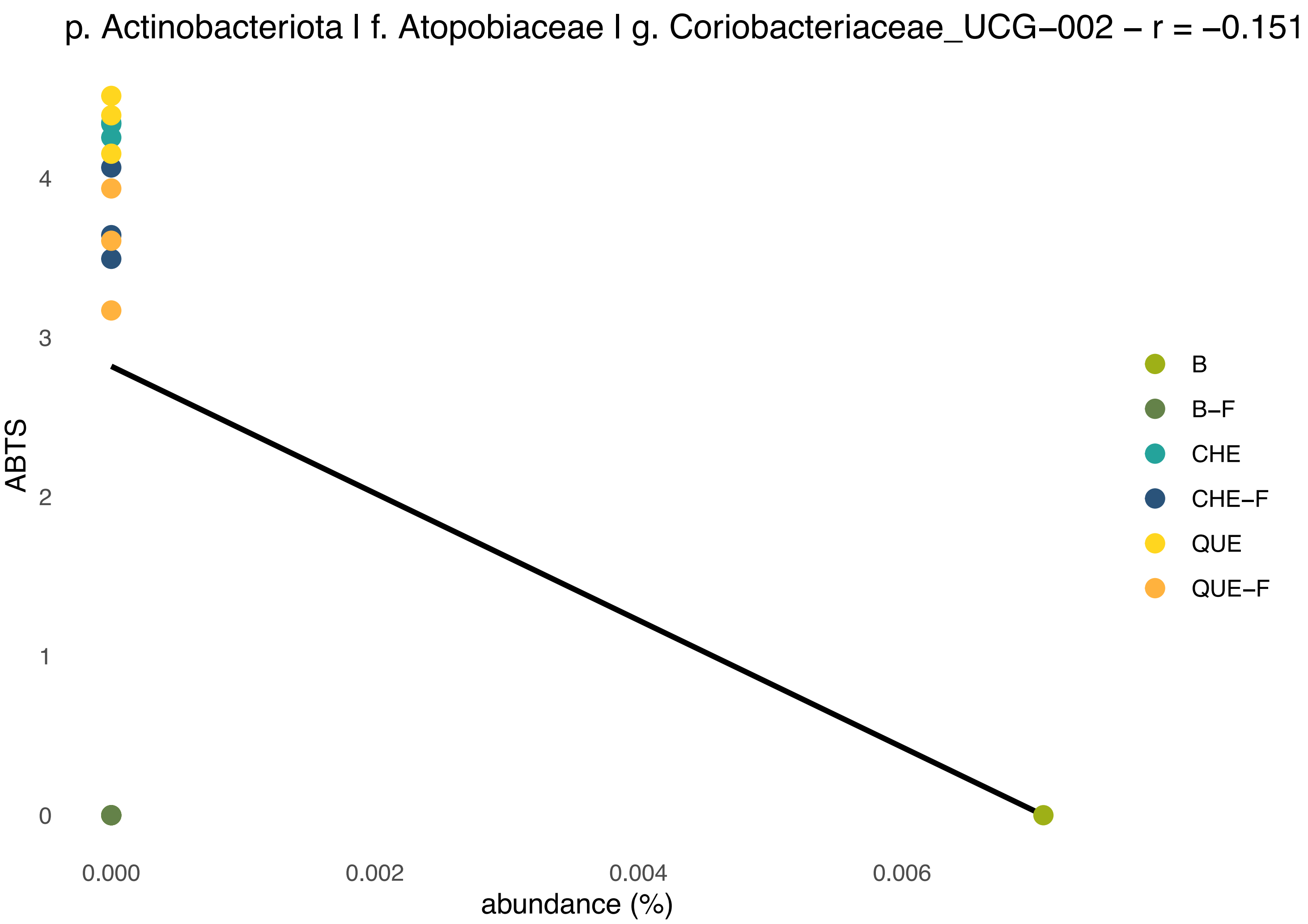
p. Actinobacteriota I f. Atopobiaceae I g. Coriobacteriaceae_UCG−002 – r = −0.151
ABTS
4
3
2
1
0
0.000
0.002
0.004
0.006
abundance (%)
B
B−F
CHE
CHE−F
QUE
QUE−F

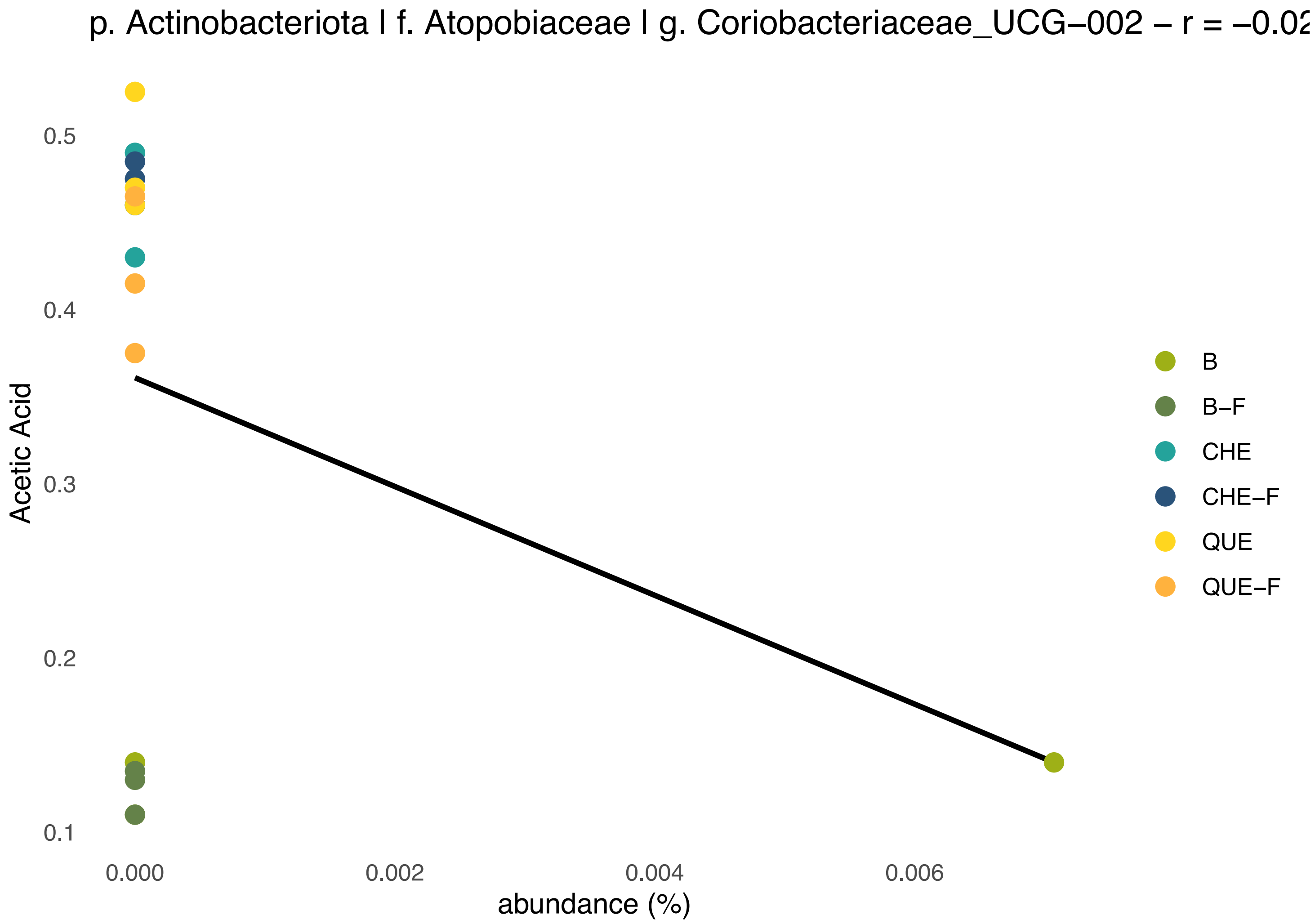

p. Actinobacteriota I f. Atopobiaceae I g. Coriobacteriaceae_UCG−002 − r = −0.02
Acetic Acid
0.5
0.4
0.3
0.2
0.1
0.000
0.002
0.004
0.006
abundance (%)
B
B−F
CHE
CHE−F
QUE
QUE−F

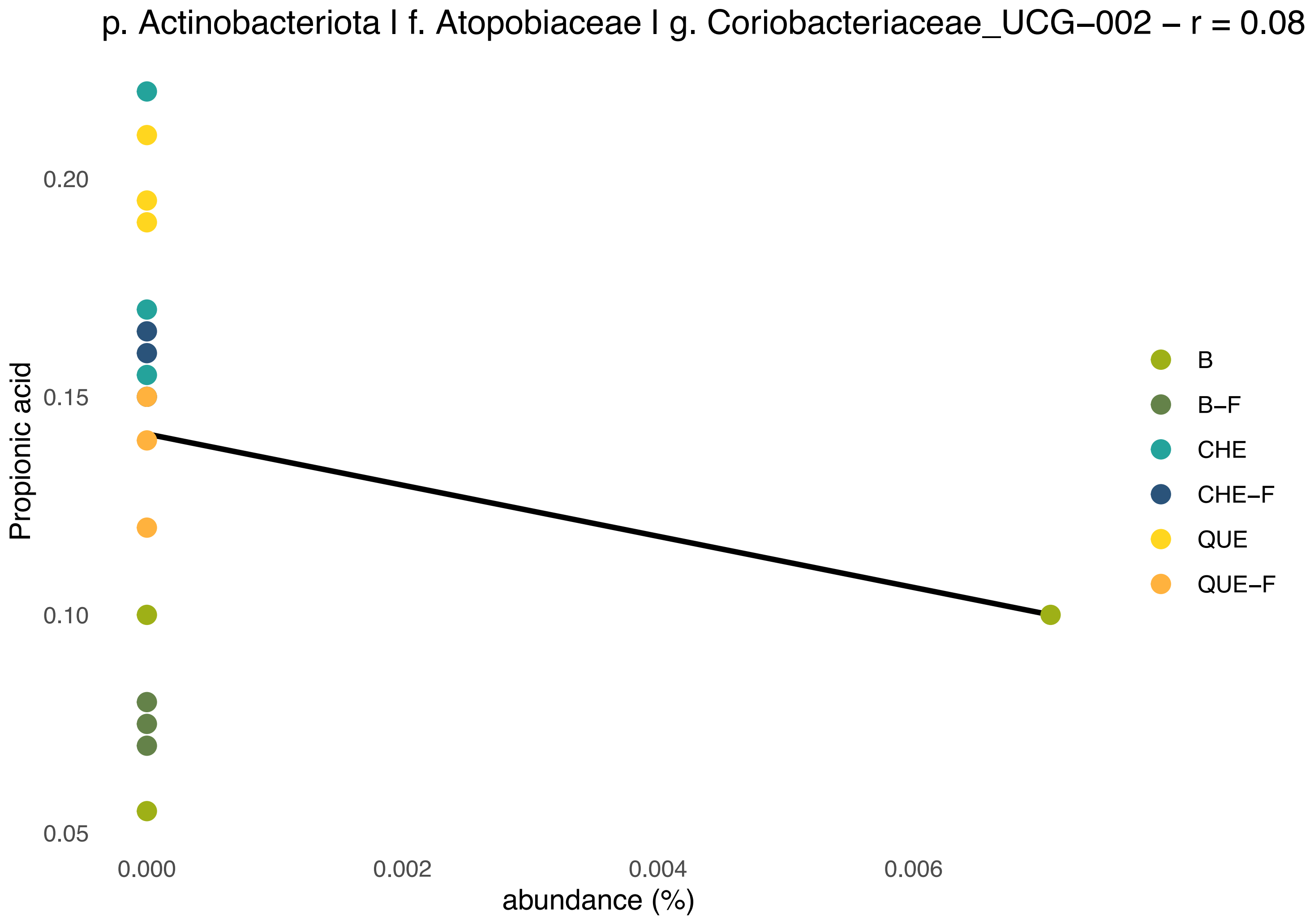

p. Actinobacteriota I f. Atopobiaceae I g. Coriobacteriaceae_UCG−002 − r = 0.08
Propionic acid
0.20
0.15
0.10
0.05
0.000
0.002
0.004
0.006
abundance (%)
B
B−F
CHE
CHE−F
QUE
QUE−F

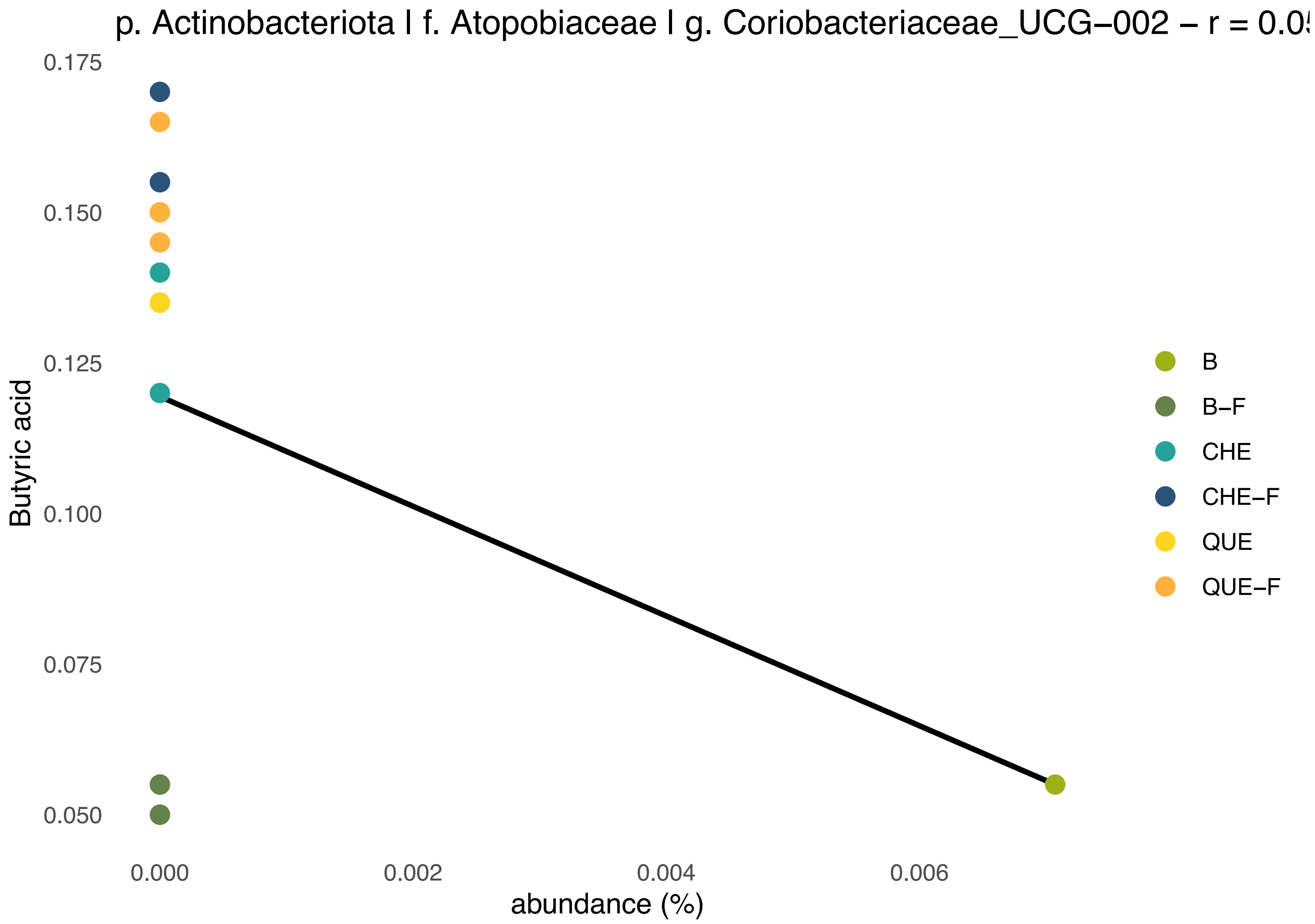
p. Actinobacteriota I f. Atopobiaceae I g. Coriobacteriaceae_UCG−002 − r = 0.0
0.175
0.150
0.125
0.100
0.075
0.050
Butyric acid
0.000
0.002
0.004
0.006
abundance (%)
B
B−F
CHE
CHE−F
QUE
QUE−F

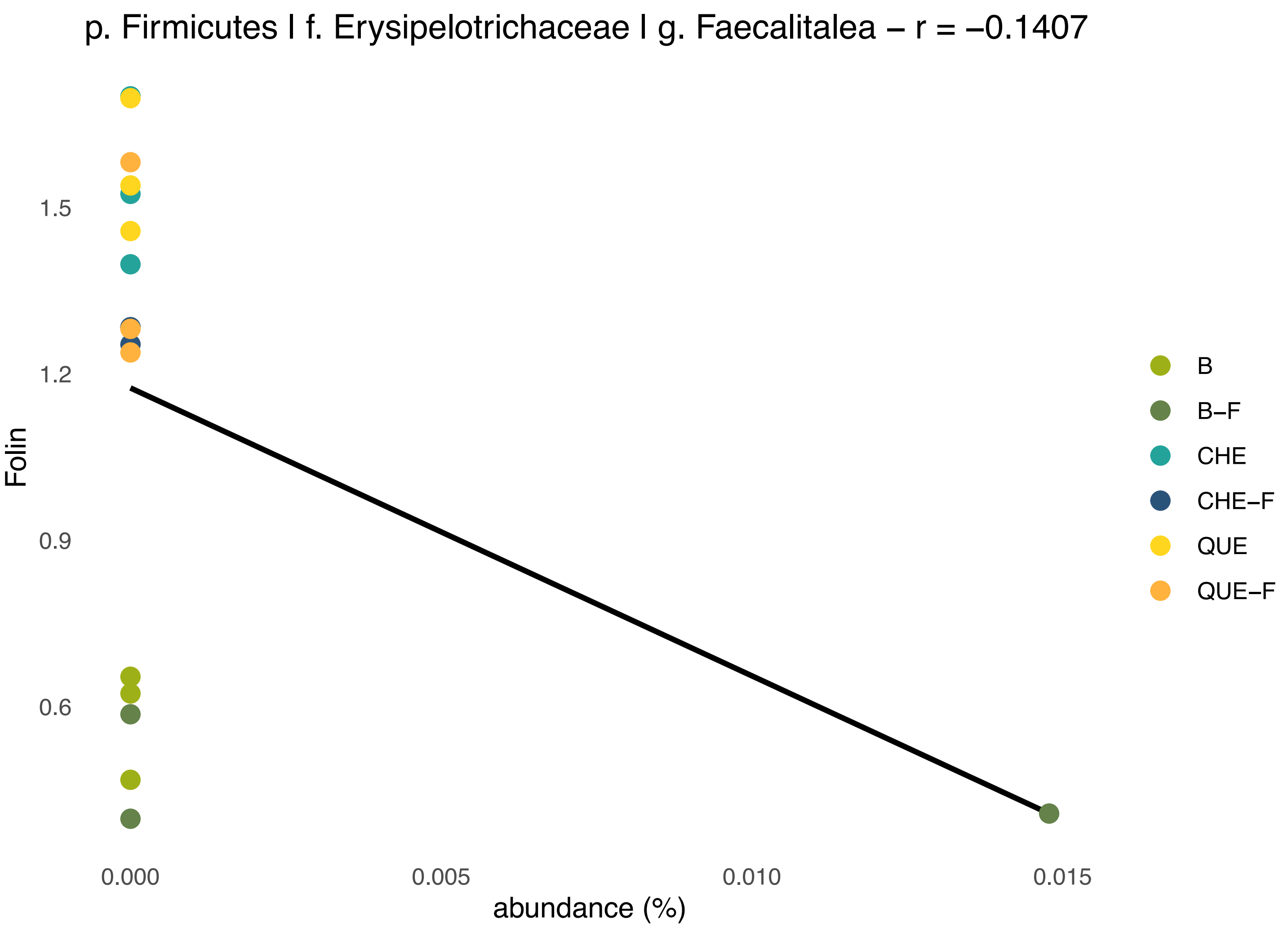

p. Firmicutes I f. Erysipelotrichaceae I g. Faecalitalea − r = −0.1407
1.5
1.2
0.9
0.6
Folin
0.000
0.005
0.010
0.015
abundance (%)
B
B−F
CHE
CHE−F
QUE
QUE−F

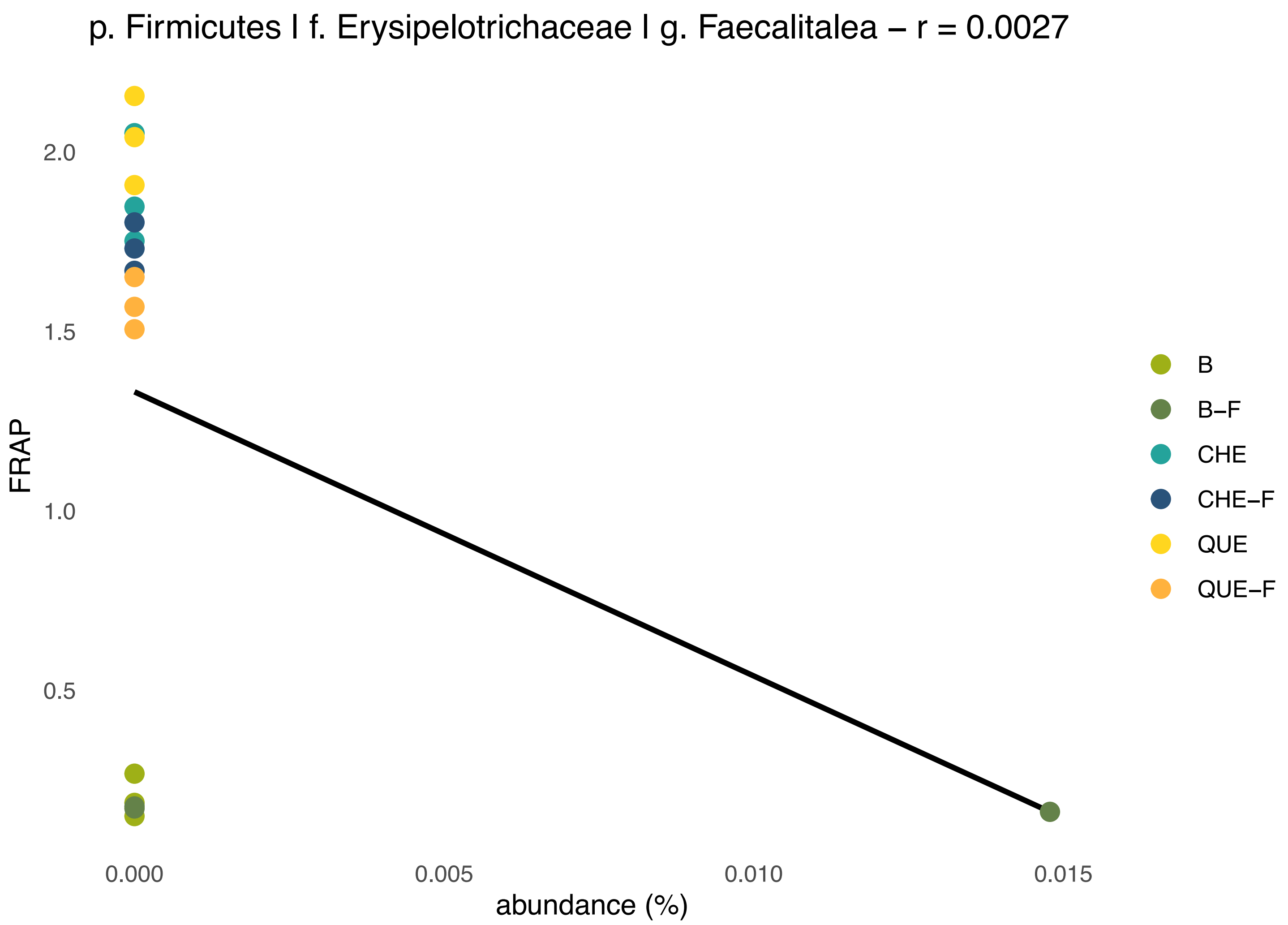

p. Firmicutes l f. Erysipelotrichaceae l g. Faecalitalea – r = 0.0027
FRAP
2.0
1.5
1.0
0.5
0.000
0.005
0.010
0.015
abundance (%)
B
B–F
CHE
CHE–F
QUE
QUE–F

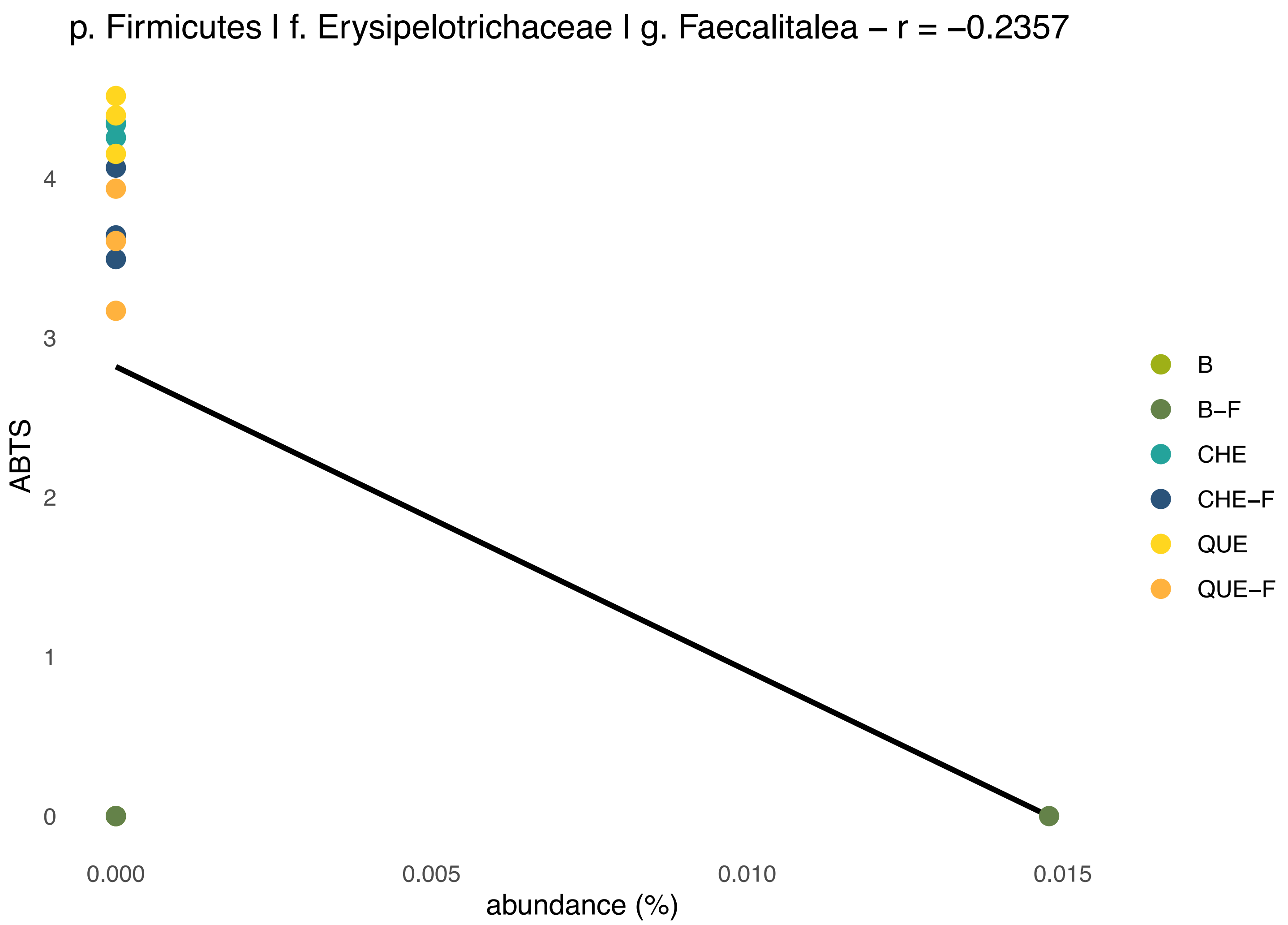

p. Firmicutes l f. Erysipelotrichaceae l g. Faecalitalea – r = –0.2357
ABTS
0
1
2
3
4
0.000
0.005
0.010
0.015
abundance (%)
B
B–F
CHE
CHE–F
QUE
QUE–F

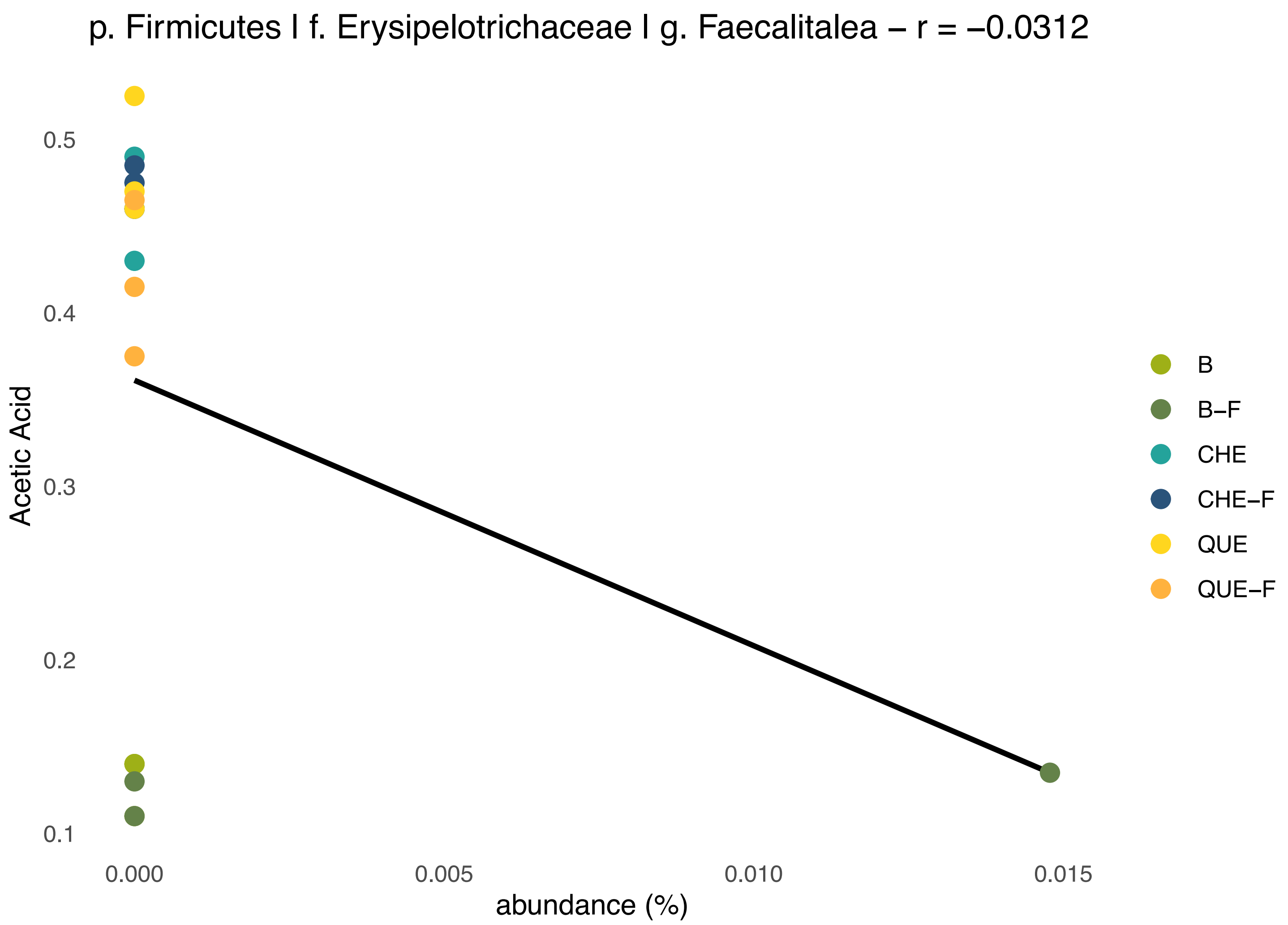

p. Firmicutes I f. Erysipelotrichaceae I g. Faecalitalea – r = –0.0312
Acetic Acid
0.5
0.4
0.3
0.2
0.1
0.000
0.005
0.010
0.015
abundance (%)
B
B–F
CHE
CHE–F
QUE
QUE–F

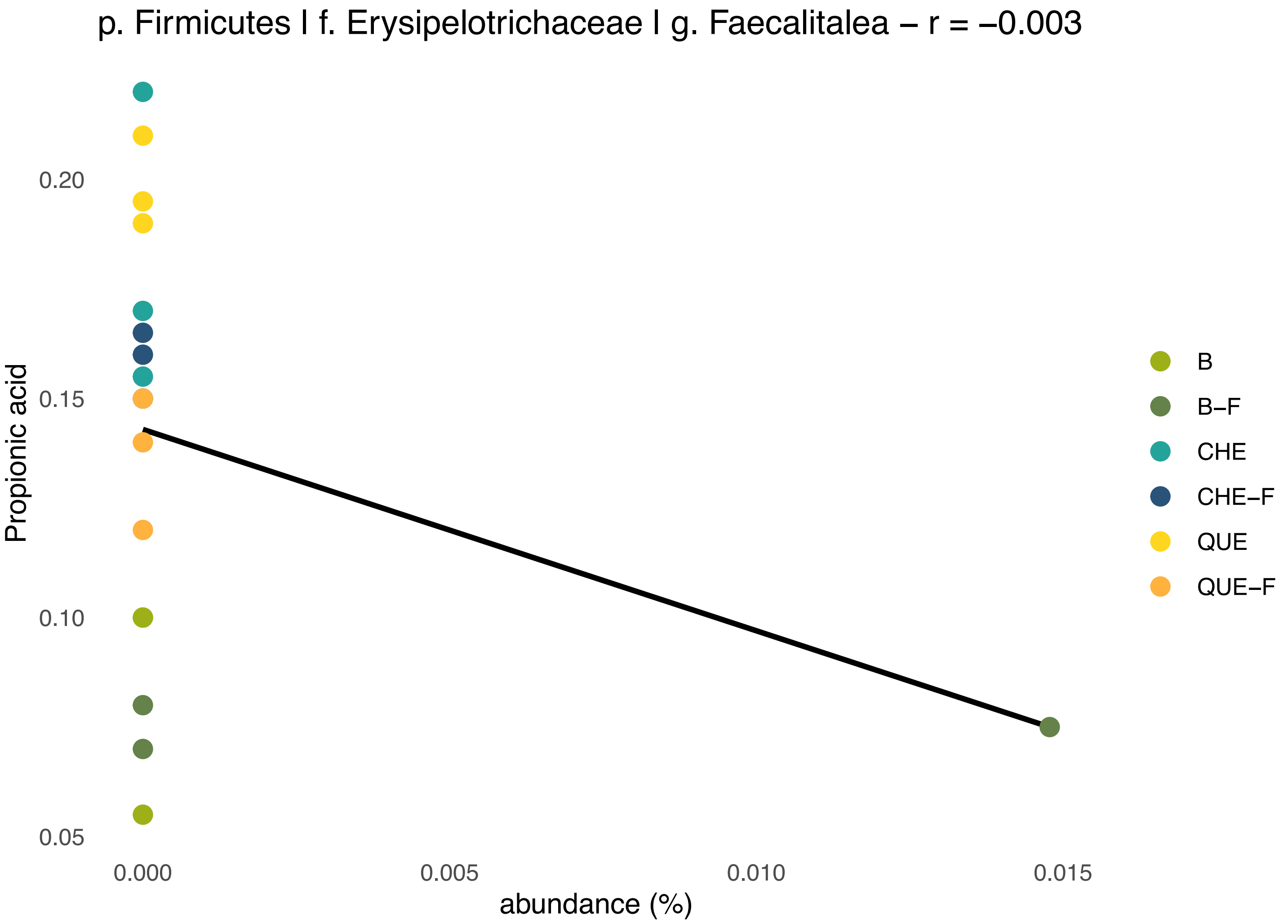

p. Firmicutes l f. Erysipelotrichaceae l g. Faecalitalea − r = −0.003
Propionic acid
0.20
0.15
0.10
0.05
0.000
0.005
0.010
0.015
abundance (%)
B
B−F
CHE
CHE−F
QUE
QUE−F

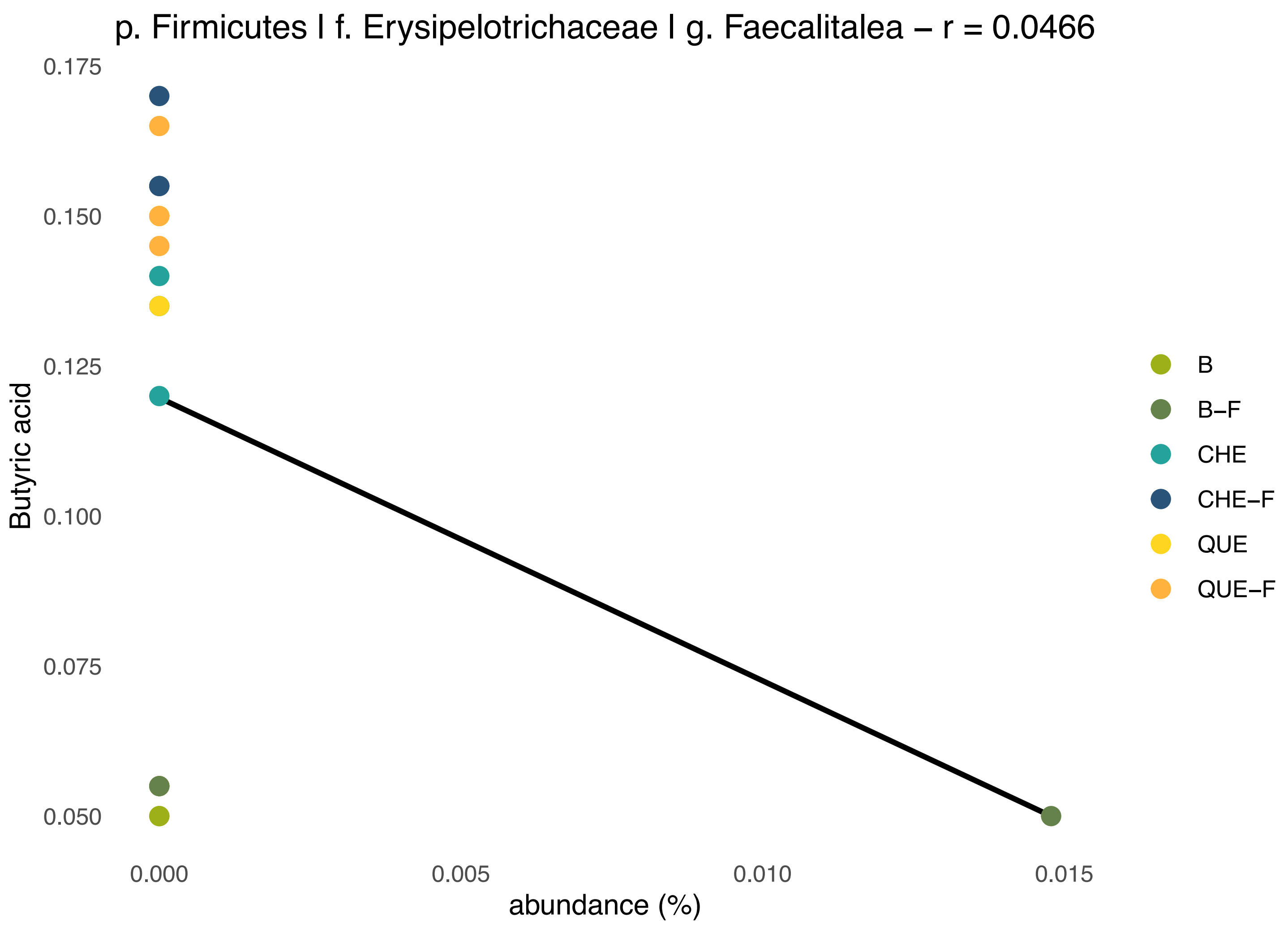

p. Firmicutes I f. Erysipelotrichaceae I g. Faecalitalea – r = 0.0466
Butyric acid
0.175
0.150
0.125
0.100
0.075
0.050
0.000
0.005
0.010
0.015
abundance (%)
B
B–F
CHE
CHE–F
QUE
QUE–F

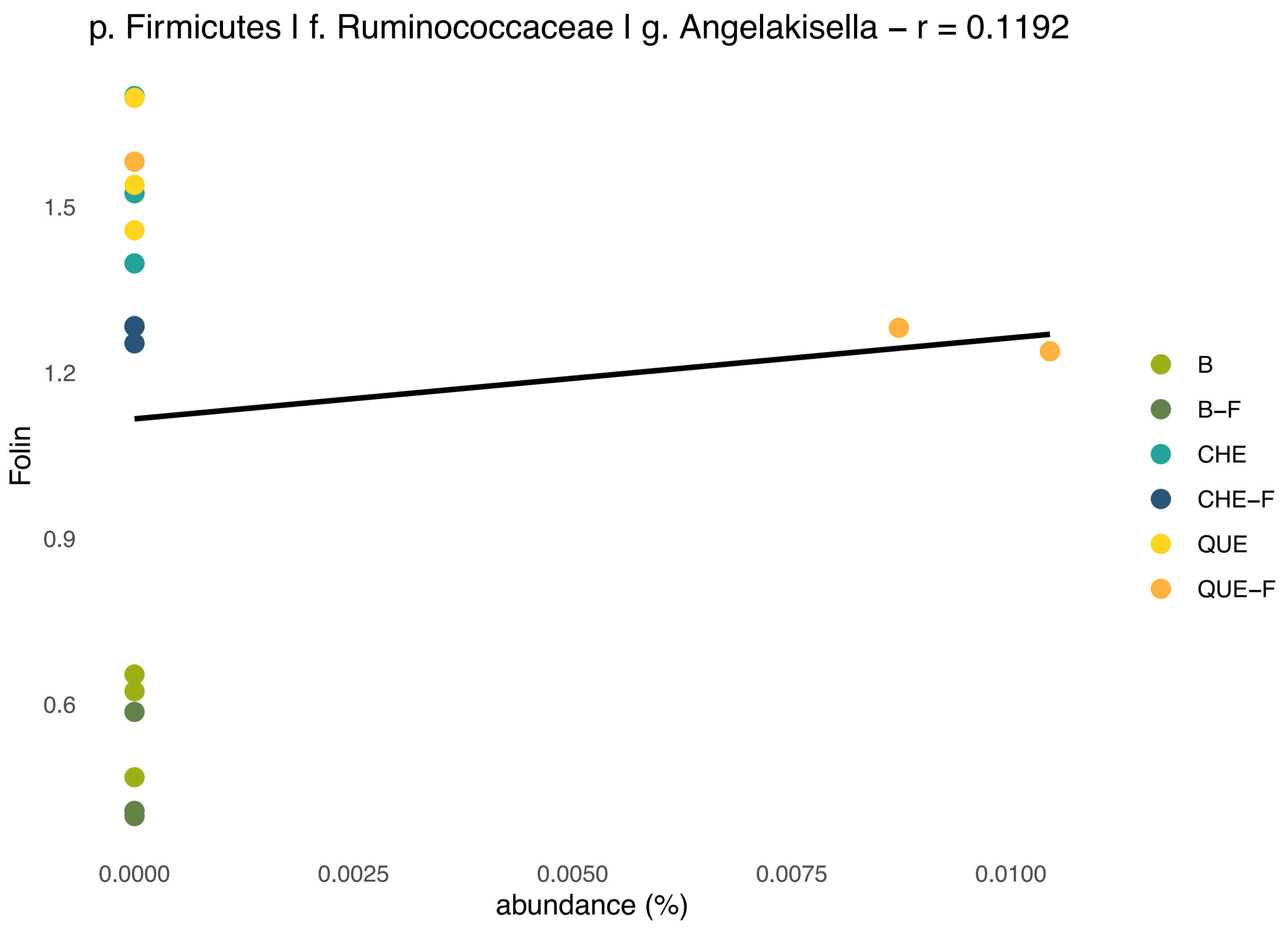
p. Firmicutes I f. Ruminococcaceae I g. Angelakisella – r = 0.1192
Folin
1.5
1.2
0.9
0.6
0.0000
0.0025
0.0050
0.0075
0.0100
abundance (%)
B
B–F
CHE
CHE–F
QUE
QUE–F

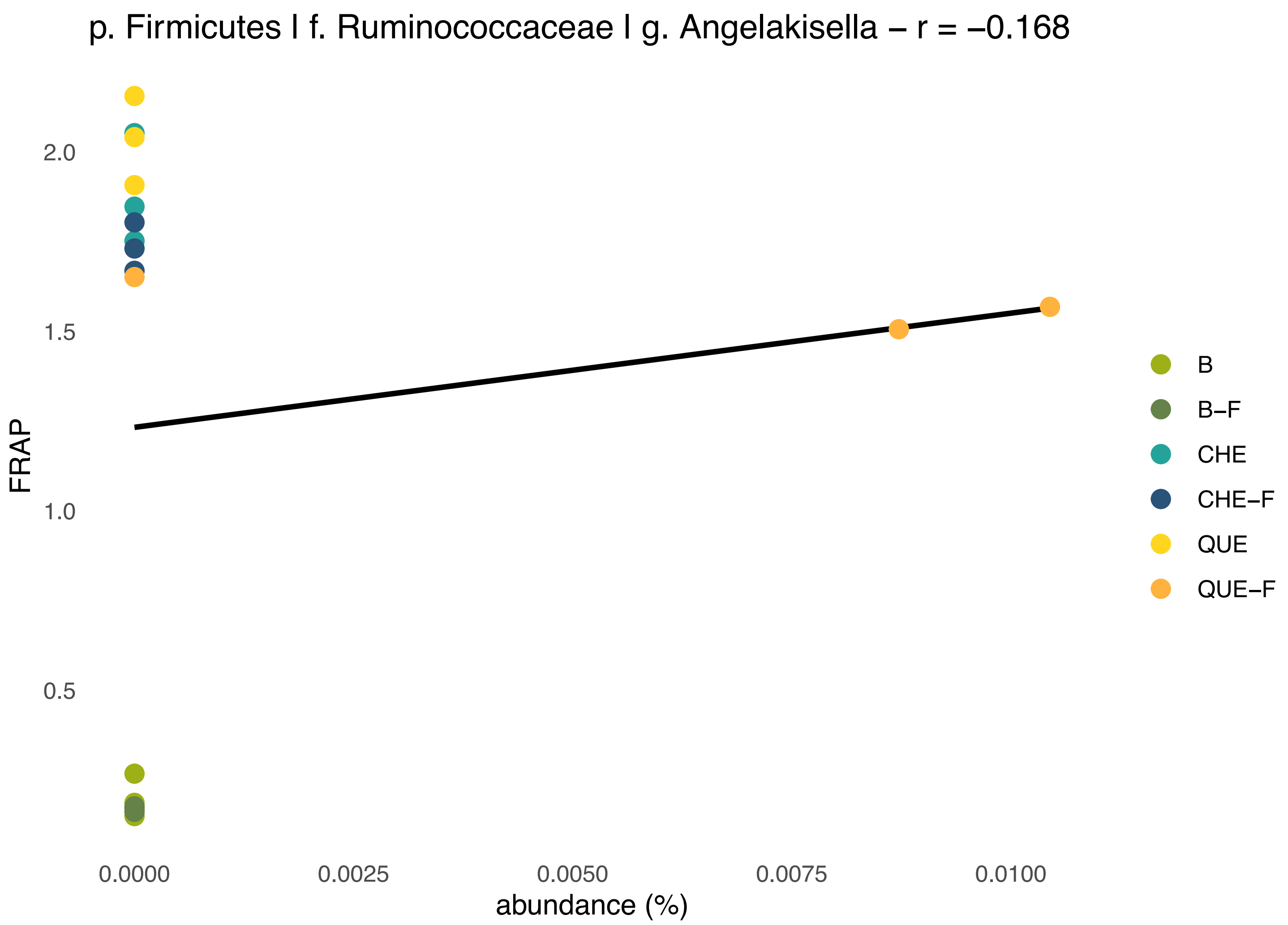

p. Firmicutes I f. Ruminococcaceae I g. Angelakisella – r = –0.168
FRAP
2.0
1.5
1.0
0.5
0.0000
0.0025
0.0050
0.0075
0.0100
abundance (%)
B
B–F
CHE
CHE–F
QUE
QUE–F

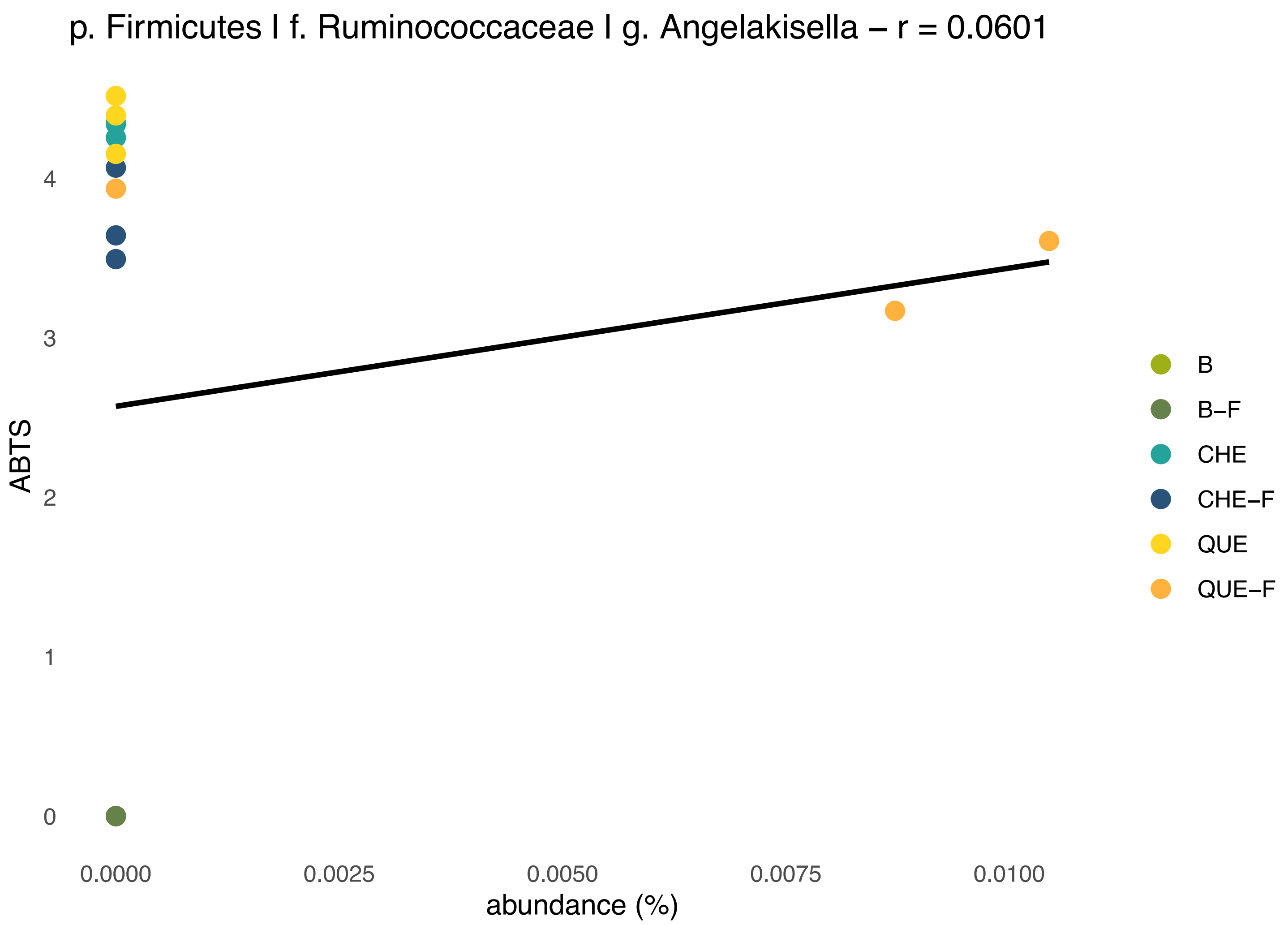

p. Firmicutes I f. Ruminococcaceae I g. Angelakisella – r = 0.0601
ABTS
0
1
2
3
4
0.0000
0.0025
0.0050
0.0075
0.0100
abundance (%)
B
B–F
CHE
CHE–F
QUE
QUE–F

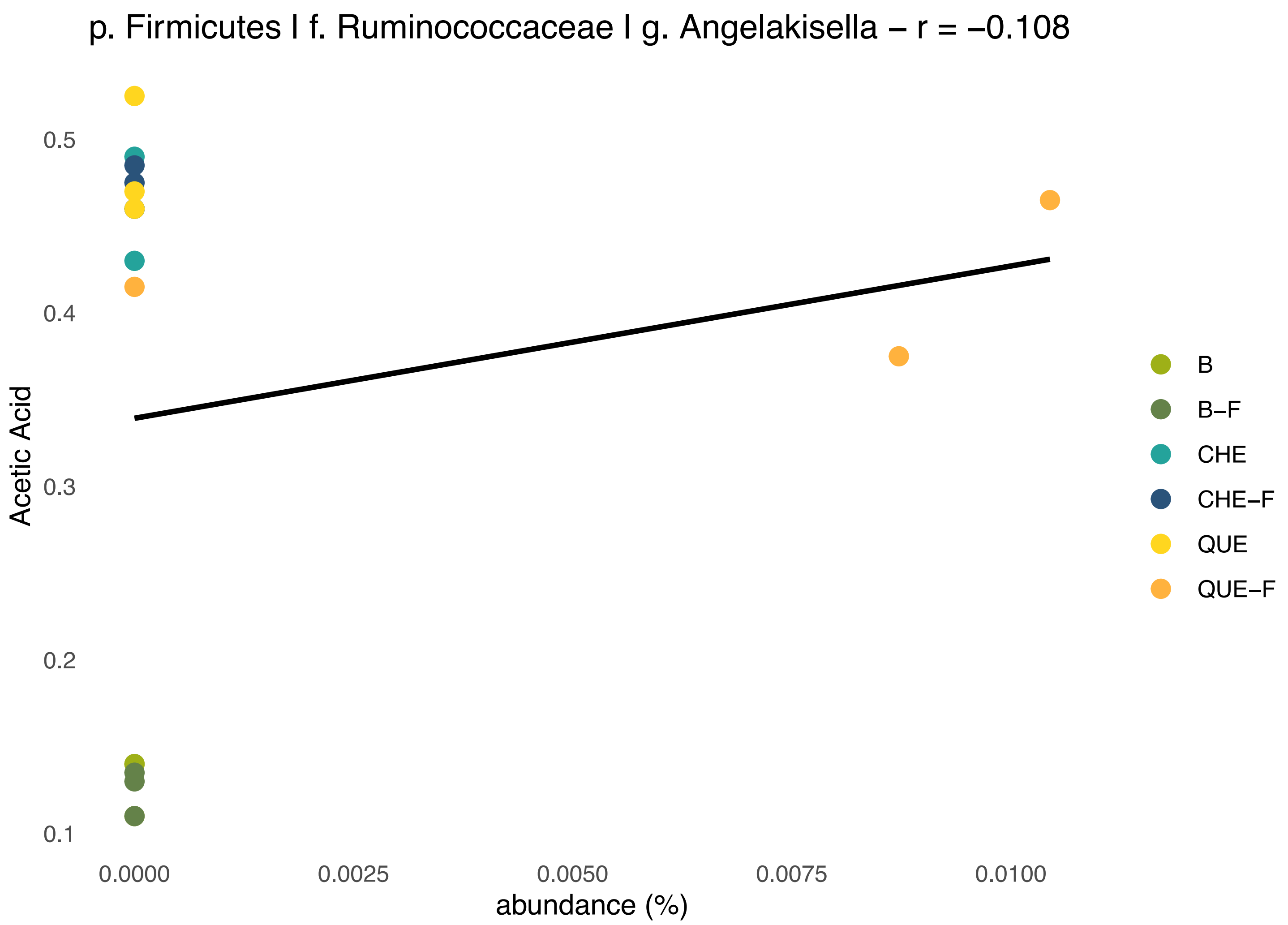
p. Firmicutes l f. Ruminococcaceae l g. Angelakisella – r = –0.108
Acetic Acid
0.5
0.4
0.3
0.2
0.1
0.0000
0.0025
0.0050
0.0075
0.0100
abundance (%)
B
B–F
CHE
CHE–F
QUE
QUE–F

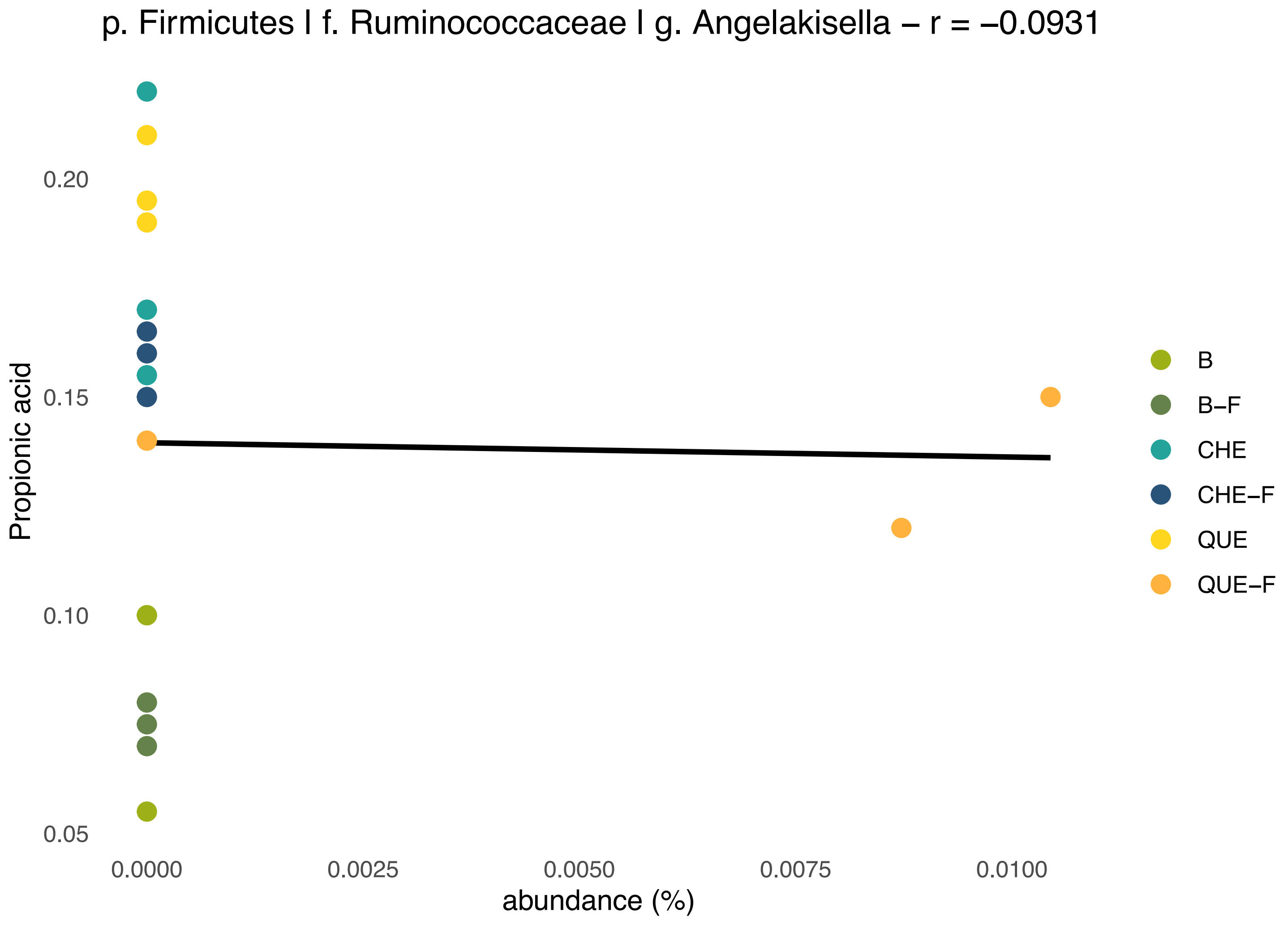

p. Firmicutes | f. Ruminococcaceae | g. Angelakisella – r = −0.0931
Propionic acid
0.20
0.15
0.10
0.05
0.0000
0.0025
0.0050
0.0075
0.0100
abundance (%)
B
B−F
CHE
CHE−F
QUE
QUE−F

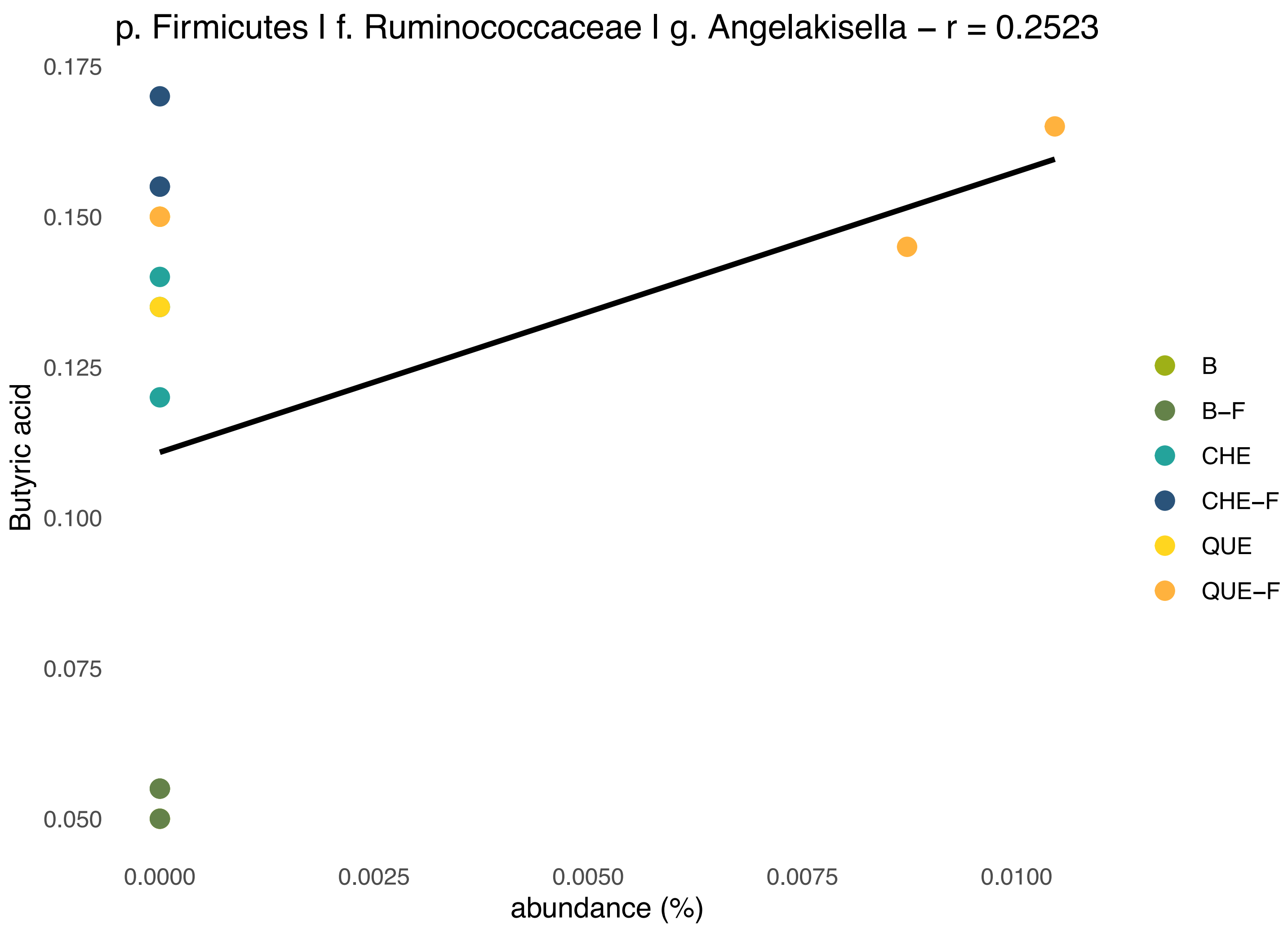
p. Firmicutes I f. Ruminococcaceae I g. Angelakisella – r = 0.2523
Butyric acid
0.175
0.150
0.125
0.100
0.075
0.050
0.0000
0.0025
0.0050
0.0075
0.0100
abundance (%)
B
B–F
CHE
CHE–F
QUE
QUE–F
